# Chemoselective umpolung of thiols to episulfoniums for cysteine bioconjugation

**DOI:** 10.1038/s41557-023-01388-7

**Published:** 2023-12-20

**Authors:** Philipp Hartmann, Kostiantyn Bohdan, Moritz Hommrich, Fabio Juliá, Lara Vogelsang, Jürgen Eirich, Rene Zangl, Christophe Farès, Julia Beatrice Jacobs, Dwaipayan Mukhopadhyay, Johanna Marie Mengeler, Alessandro Vetere, Marie Sophie Sterling, Heike Hinrichs, Stefan Becker, Nina Morgner, Wolfgang Schrader, Iris Finkemeier, Karl-Josef Dietz, Christian Griesinger, Tobias Ritter

**Affiliations:** 1 Max-Planck-Institut für Kohlenforschung, Mülheim an der Ruhr, Germany; 2 Institute of Organic Chemistry, RWTH Aachen University, Aachen, Germany; 3 Biochemistry and Physiology of Plants, Faculty of Biology, Bielefeld University, Bielefeld, Germany; 4 Institute of Plant Biology and Biotechnology, University of Münster, Münster, Germany; 5 Institute of Physical and Theoretical Chemistry, Goethe University Frankfurt/Main, Frankfurt/Main, Germany; 6 Max Planck Institute for Multidisciplinary Sciences, Göttingen, Germany

**Keywords:** Synthetic chemistry methodology, Reaction mechanisms

## Abstract

Cysteine conjugation is an important tool in protein research and relies on fast, mild and chemoselective reactions. Cysteinyl thiols can either be modified with prefunctionalized electrophiles, or converted into electrophiles themselves for functionalization with selected nucleophiles in an independent step. Here we report a bioconjugation strategy that uses a vinyl thianthrenium salt to transform cysteine into a highly reactive electrophilic episulfonium intermediate in situ, to enable conjugation with a diverse set of bioorthogonal nucleophiles in a single step. The reactivity profile can connect several nucleophiles to biomolecules through a short and stable ethylene linker, ideal for introduction of infrared labels, post-translational modifications or NMR probes. In the absence of reactive exogenous nucleophiles, nucleophilic amino acids can react with the episulfonium intermediate for native peptide stapling and protein–protein ligation. Ready synthetic access to isotopologues of vinyl thianthrenium salts enables applications in quantitative proteomics. Such diverse applications demonstrate the utility of vinyl-thianthrenium-based bioconjugation as a fast, selective and broadly applicable tool for chemical biology.

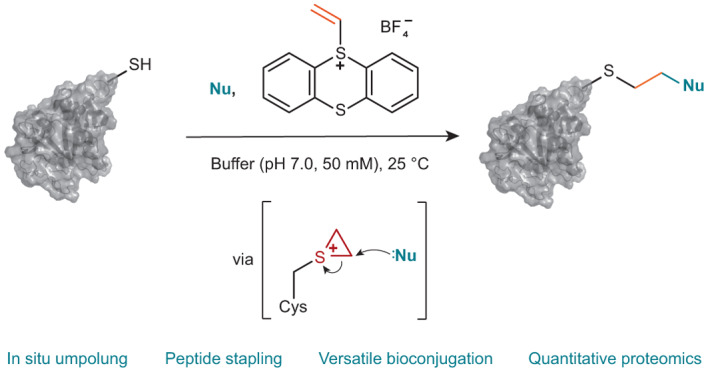

## Abstract (web summary)

Cysteine bioconjugation is an important method to modify biomolecules, but synthetic efforts to diversify reactive warheads and the low reactivity of introducible linchpins often impede application in biological laboratories. Now, a thianthrenium-based reagent permits site-selective installation of episulfonium on biomacromolecules, enabling one-step addition of bioorthogonal nucleophiles and further applications in quantitative proteomics and cross-linking.

## Main

Efficient functionalization of proteins is challenging because it requires fast and chemoselective reactions that produce stable conjugates under biocompatible conditions. Introduction of a functional group at a specific position in the protein is achieved with methods that modify amino acid residues with low natural abundance^1^ such as cysteine (Cys)^2^, methionine^3,4^, tryptophan^5,6^ and tyrosine^7^. Due to its high intrinsic reactivity and easy incorporation via site-directed mutagenesis, Cys is often the target of choice for site-selective bioconjugations under physiological conditions^8^. A common approach exploits the reactivity between Cys and electrophiles, such as Michael acceptors^9–11^, α-halocarbonyls^12^, organometallic reagents^13^, disulfides^14^, hypervalent iodine reagents^15,16^, phosphonamidates^17^ or perfluoroarenes^18^, that carry a payload of interest. Many methods allow for fast and efficient Cys functionalization. However, each functionalization to introduce a specific payload requires synthesis of a new derivative of the reagent if not commercially available.

An alternative strategy entails Cys transformation into an electrophilic linchpin for subsequent follow-up modifications. For example, reagents that contain multiple electrophilic positions can react with Cys and retain an electrophilic site^19–22^, Cys can be converted to form dehydroalanine by elimination^23–25^, or be transformed into electrophilic palladium complexes^26^. In an additional, subsequent step, nucleophiles are added to the initially formed electrophilic products to form the desired bioconjugates. The inherent advantage of this approach is that multiple bioconjugates can be formed based on a single introduced linchpin. However, the two-step sequence used in current methods is necessary to convert Cys into a linchpin before the nucleophile can be added to avoid cross-reactivity. Otherwise, the reagent required to furnish the linchpin would be quenched by the excess nucleophile. It is therefore necessary for the formed electrophilic intermediates to persist in solution, and that these intermediates can only be functionalized with strong nucleophiles in the next step because their stability results in attenuated reactivity.

We envisioned that a Cys-selective reagent that, upon conjugation, results in formation of a more reactive electrophile in situ would enable reactions with various nucleophiles that are not reactive enough to be used with current methods. Our group has previously reported the synthesis and reactivity of several thianthrenium-based compounds, for example, aryl-^27^ and trifluoromethyl^28^ thianthrenium salts, which can access reactivity that goes beyond what has been possible with conventional halides or pseudohalides^29,30^. Based on the promising and distinct reactivity that the thianthrenium substituent can impart, we hypothesized that vinyl thianthrenium salts can be used to achieve versatile functionalization of biomolecules with different complexity at biocompatible conditions, and provide the opportunity to install a stable linker that is shorter than other methods allow.

## Results and discussion

### Kinetic profile of the bioconjugation

**Fig. 1:**
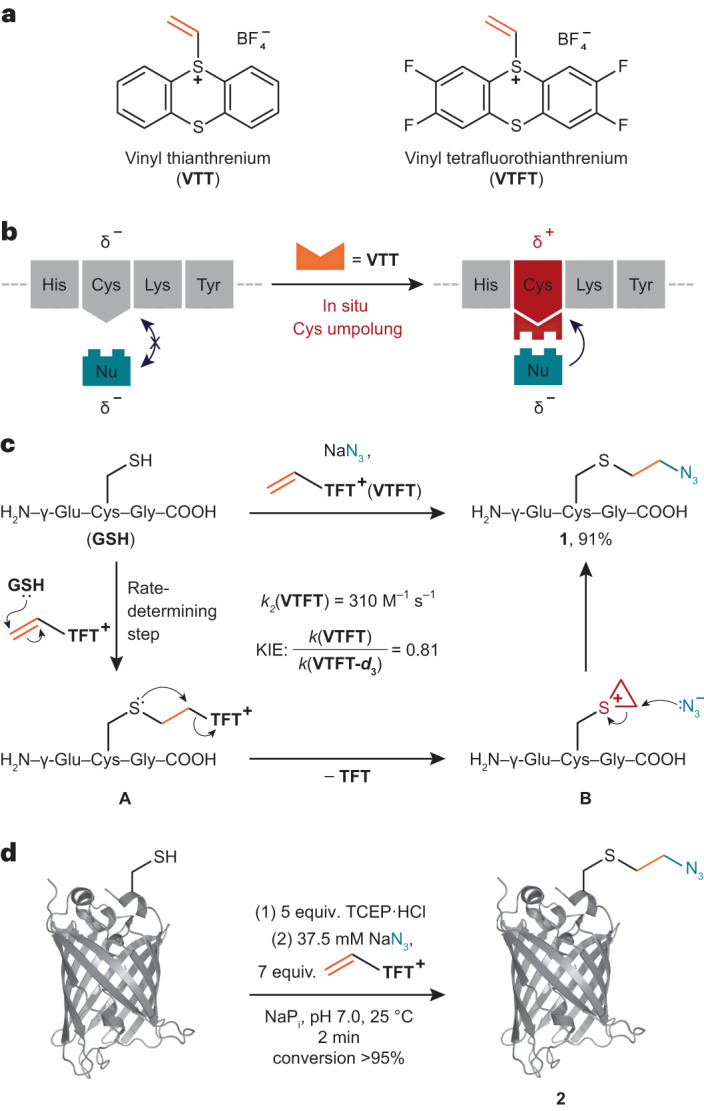
Biofunctionalization with vinyl thianthrenium reagents. **a**, The vinyl thianthrenium reagents **VTT** and **VTFT** utilized in this work. **b**, Strategy for the in situ activation of Cys based on site-selective linchpin formation in the presence of other amino acids and exogenous nucleophiles. **c**, Proposed mechanism for the umpolung of thiols based on the KIE determined via a competition experiment between **VTFT** and **VTFT-*d***_**3**_ and the second-order rate constant for the functionalization of **GSH** with **VTFT**. **d**, Bioconjugation of sfGFP S147C with **VTFT** and sodium azide. The following publicly available protein structure was used: sfGFP (PDB:2B3P). His, histidine; Lys, Lysine; Tyr, tyrosine; Nu, nucleophile.

Vinyl thianthrenium tetrafluoroborate (**VTT**) and vinyl tetrafluorothianthrenium tetrafluoroborate (**VTFT**) (Fig. 1a) are bench-stable, water-soluble vinylating reagents, which can be accessed in one step from ethylene gas^31^. The fundamental difference in reactivity of **VTT** and its tetrafluoro derivative **VTFT** when compared to conventional Michael acceptors such as maleimides lies in the identity and reactivity of the cationic intermediates formed after chemoselective addition to Cys to enable a one-step bioconjugation with otherwise unreactive nucleophiles present in the mixture (Fig. 1b). The cationic thianthrenium moiety in **VTT** and **VTFT** makes the reagents reactive Michael acceptors, while its ability as a leaving group enables its ensuing displacement from alkylsulfonium intermediate **A** to yield episulfonium intermediate **B** (Fig. 1c), a reactive species for reactions with various nucleophiles.

For example, in situ formation of **B** in the presence of sodium azide results in formation of the azidated tripeptide glutathione (**GSH**) **1** in 91% yield, without any observed side reactivity. Formation of **B** as the key intermediate is supported by NMR studies: when the reaction is performed in a deuterated buffer, the formed product has an equal distribution of deuterium over the ethylene bridge, consistent with the presence of a cyclic, symmetric intermediate prior to addition of azide (Supplementary Fig. 208). Kinetic studies revealed reaction orders of 0 for azide and 1.0 for **VTFT** and **GSH**, respectively, in line with episulfonium ring-opening after the rate-determining step. To distinguish between rate-determining Cys addition to **VTFT** and episulfonium formation, we performed a competition experiment between **VTFT** and **VTFT-*d***_**3**_ and observed an inverse secondary kinetic isotope effect (KIE) of 0.81 (Supplementary Figs. 201 and 202). The KIE value is consistent with a change of hybridization from *sp*^2^ to *sp*^3^ at the reacting carbon in the rate-limiting step and excludes rate-limiting episulfonium formation^32^. The data are in agreement with rate-limiting addition of Cys to **VTFT**, followed by fast episulfonium formation and ensuing rapid, irreversible nucleophilic opening with azide.

The reaction between **GSH**, **VTT** and NaN_3_ in sodium phosphate buffer (NaP_i_) at pH 7.4 has a second-order rate constant of 38.1 ± 0.1 M^−1^ s^−1^, and proceeds faster than the click reactions commonly used for chemical ligation^33^ or Cys alkylation with iodoacetamide^34^. **VTFT** functionalizes Cys even faster with a determined second-order rate constant of 310 ± 8 M^−1^ s^−1^, which is the same order of magnitude as the conjugation with alkylmaleimides^10^. The difference in rate between **VTT** and **VTFT** is due to the electronic effects arising from the four fluoride substituents on tetrafluorothianthrene (**TFT**)^35^. Due to the high reactivity and chemoselectivity of both **VTT** and **VTFT**, the conjugation reactions were readily extended to proteins. For example, full conversion of the superfolder green fluorescent protein (sfGFP) S147C mutant to the azidated product **2** was observed within 120 s under biocompatible reaction conditions at pH 7.0 (Fig. 1d and Supplementary Figs. 118 and 119). To ensure that the redox-active cysteinyl thiols for protein ligation are not present in higher oxidation states^36^, a small excess of reducing tris(2-carboxyethyl)phosphine (TCEP) was added prior to addition of vinyl thianthrenium. No reported method allows for introduction of alkyl azides into proteins from azide at such high rates and mild conditions in a single step.

### Site selectivity and site specificity of the reaction

**Fig. 2:**
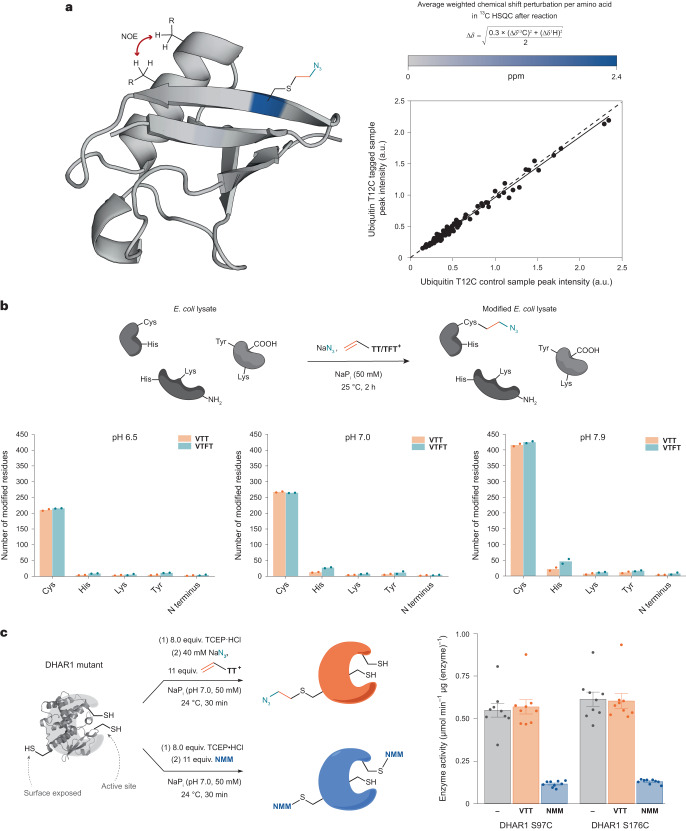
Selectivity of bioconjugation with vinyl thianthrenium salts. **a**, Three-dimensional representation of the average ^1^H and ^13^C NMR shift perturbations of each amino acid after functionalization of ^13^C,^15^N-labelled ubiquitin T12C and the NOESY correlation plot of scaled cross-peak heights before and after functionalization of ubiquitin T12C. The linear regression of the experimental data is indicated as a solid line. **b**, Mean number of modified residues in *E. coli* lysate resulting from reaction with **VTT/VTFT** and sodium azide at different pH values. Data are derived from two technical replicates. TCEP was used for 1 h pre-reduction at 37 °C. **c**, Reactivity of DHAR1 mutants with **VTT** and *N*-methylmaleimide (**NMM**) and enzyme assay subsequent to the modification. The data are expressed as mean ± s.e. of nine independent experiments. The statistical analysis was performed via a two-sided analysis of variance, followed by post hoc Tukey test with *P* < 0.05 (see Supplementary Tables 11 and 12 for exact *P* values). The following publicly available protein structures were used: ubiquitin (PDB:1D3Z) and DHAR1 (PDB:5EL8). Source data

Chemical modifications of biomolecules are often utilized to study native structures and enzymatic activity. Therefore, it is important that bioconjugation is site selective and the protein structure not significantly altered^37^. We used protein NMR as a non-invasive method to inquire about the chemoselectivity of the thianthrene-based transformation and the potential resulting structural perturbation in the tertiary structure of the protein^38^. Heteronuclear single quantum coherence (HSQC) NMR experiments of ^13^C,^15^N-labelled ubiquitin T12C prior to and after reaction with **VTT** and sodium azide showed an average weighted chemical shift perturbation of 2.40 ppm for Cys in the ^13^C NMR resonances. All other amino acids only displayed changes of up to a maximum of 0.11 ppm in the ^13^C NMR resonances, and amino acids at a distance larger than 16 Å relative to the Cys sulfur atom only showed perturbations up to 0.01 ppm. The data are consistent with high chemoselectivity for Cys modification (Fig. 2a). In addition, nuclear Overhauser effect spectroscopy (NOESY) experiments of unlabelled protein before and after conjugation were carried out. The obtained data indicate less than 3% change in interproton distances and therefore minimal structural changes and preservation of the tertiary protein structure (Fig. 2a).

When the reaction was performed on an entire *Escherichia coli* (*E. coli*) cell lysate containing various native proteins, we observed 90% chemoselectivity for Cys modification for **VTT** and 85% for **VTFT** at pH 7.0, as determined by liquid chromatography–tandem mass spectrometry (LC–MS/MS) analysis of the tryptic digest (Fig. 2b). Even when the pH was changed to 6.5 and 7.9, respectively, the high selectivity was retained in all cases, which is noteworthy given the presence of a variety of native proteins with different microenvironments and numerous competing amino acid residues, including hyper-reactive residues^12,39^. Previously reported Cys-selective reagents for proteomics such as iodoacetamide or tetrafluoroalkyl benziodoxoles reach Cys coverages of 0.5% and 1.6%, respectively^40,41^. At pH 7.9, **VTFT** labels 2.7% of all available cysteines even at concentrations of only 0.2 mM (Supplementary Table 2).

Site-specific Cys modifications are often challenging due to insufficient discrimination between different Cys residues. For example, maleimides are usually not able to distinguish between multiple Cys residues on the same protein and therefore cannot usually be utilized to modify proteins that contain catalytically active cysteinyl thiols^8^. To evaluate a potential site specificity of **VTT**, we introduced surface-exposed Cys residues into dehydroascorbate reductase 1 (DHAR1), which already features two catalytic Cys residues in the active site. Functionalization of both S97C and S176C mutants with an excess of **VTT** led to a single modification of the introduced surface-exposed Cys and preservation of the enzyme activity. In contrast, conjugation of both enzymes with the equimolar concentration of *N*-methylmaleimide led to functionalization of both surface-exposed and internal, catalytic Cys, with a concomitant substantial decrease in catalytic activity (Fig. 2c). When no surface-exposed Cys was introduced into the protein, **VTT** did not interfere with the catalytic Cys residues of DHAR1. However, maleimide reagents with different substituents readily react with the catalytic Cys residues of the native protein (Supplementary Figs. 141–143). Although steric repulsion might prevent **VTT** from reacting with the catalytic Cys residues, the unspecific reactivity of different arylmaleimide derivatives with DHAR1 suggests that the size of the reagent might not be the decisive factor for the site specificity. We speculate that the site specificity of vinyl thianthrenium reagents arises from the cationic charge of the thianthrene moiety which leads to repulsion in hydrophobic areas or environments with high concentrations of positively charged amino acids. These results bode well for potential applications of **VTT** for site-specific functionalization of proteins or enzymes that contain several and even catalytic Cys residues.

### Episulfonium enables versatile bioconjugation

**Fig. 3:**
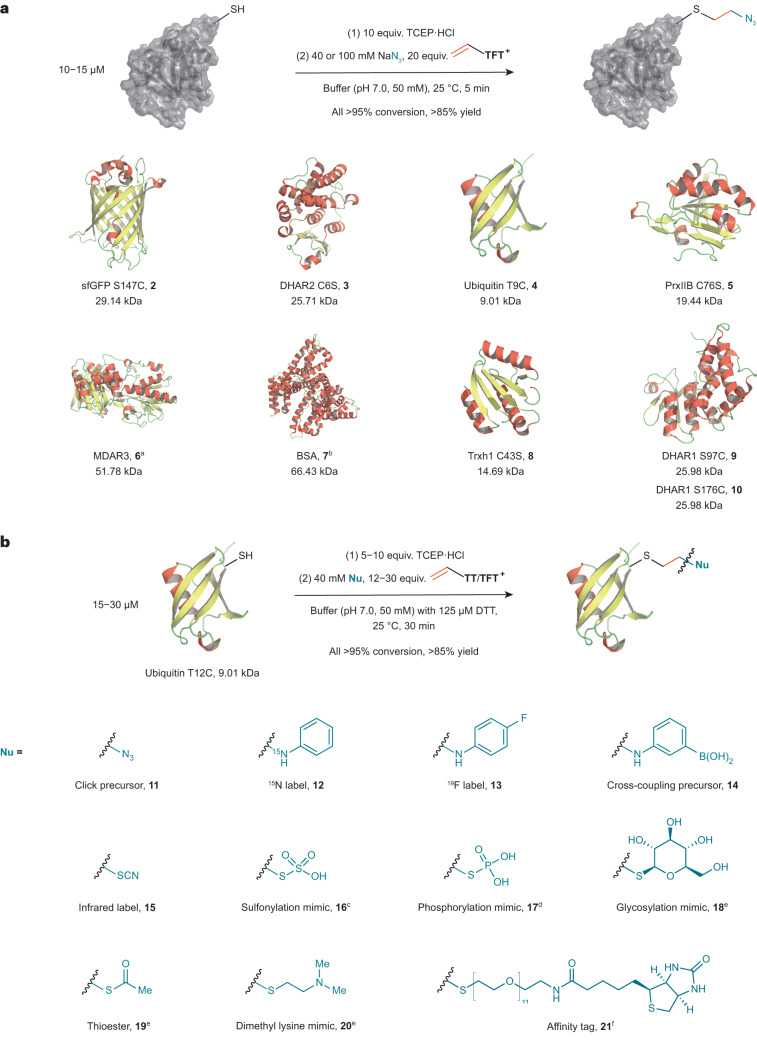
Scope of vinyl thianthrenium salts as bioconjugation reagents. **a**, Scope of proteins used for conjugation reactions with NaN_3_. **b**, Scope of functionalities introduced with **VTT**/**VTFT**. ^a^40 equiv. **VTFT**, yield not determined; ^b^yield not determined; ^c^10 mM Nu; ^d^3 mM Nu, 35 equiv. **VTT**; ^e^40 mM sodium iodide as nucleophile with subsequent addition of 40 mM S-centred nucleophiles after 3 min; ^f^ubiquitin T9C, HEPES (8.0, 50 mM), 40 mM sodium iodide as nucleophile with subsequent addition of 20 mM S-centred nucleophile after 2 min, yield, 74%. The following publicly available protein structures were used: sfGFP (PDB:2B3P), DHAR2 (PDB:5LOL), ubiquitin (PDB:1UBQ), Trxh1 (PDB:1XFL), DHAR1 (PDB:5EL8), BSA (PDB:3V03). MDAR, monodehydroascorbate reductase; PrxIIB, type 2 peroxiredoxin; Trxh1, thioredoxin-*h*1; DTT, dithiothreitol.

Based on their promising reactivity and selectivity, we evaluated a diverse set of recombinant and native Cys-containing proteins of various sizes and functions for azidoethylation (Fig. 3a). In each case, the LC–MS analysis of the product shows >95% conversion in all the cases and formation of the desired species in yields of at least 85% in all the quantifiable cases. Although close to the protein surface as a consequence of the short linker, the furnished azide can readily be utilized in copper-catalysed or strain-promoted click reactions (Supplementary Figs. 97–100).

In contrast to the previously reported methods for cysteine bis-alkylation^42^, the episulfonium intermediate, such as **B**, does not eliminate to form dehydroalanine. Instead, given the high reactivity of **B** for nucleophilic ring opening, bioconjugation through opening of **B** with other bioorthogonal nucleophiles, less reactive than azide, is possible and efficient (Fig. 3b). For example, anilines can be used to install NMR-active nuclei^43^ (**12**, **13**) or bioorthogonal functionalities^44^ (**14**). Introduction of sulfur-centred nucleophiles gives access to thiocyanate (**15**) as an infrared label^45^, thiosulfate (**16**) as a mimic for sulfation^46^ and thiophosphate as mimic for phosphorylation^47^ (**17**). Iodide can be utilized to form primary alkyl iodide intermediates (Supplementary Figs. 211, 213 and 214), which can then be functionalized with thiol-based nucleophiles to introduce mimics for glycosylation^48^ (**18**), reversible modifications in the form of thioesters^49^ (**19**), lysine methylation^50^ (**20**) or to attach an affinity tag for purification (**21**). The episulfonium approach enables bioconjugation with a broad set of different nucleophiles as weak as anilines in a convenient one-pot process, which no other method appears to allow. No hydrolysis, that is, episulfonium opening with the solvent water, was detected for the various nucleophiles shown in Fig. 3b, except for the anilines and **21**, when up to 15% of the water addition by-product was observed as determined by LC–MS (Supplementary Table 5).

To investigate potential side reactivity that might go undetected in the presence of excess exogenous nucleophiles, we performed control reactions without any additives except for reductant and buffer. When sfGFP S147C, DHAR1 S97C and DHAR1 S176C were modified with **VTT** in the absence of additional nucleophiles, LC–MS measurements revealed intramolecular ring opening of the episulfonium intermediates and phosphate adducts from the phosphate buffer in addition to the already observed hydrolysis side reaction. Because intramolecular cross-linking can be accompanied by structural alteration and loss of function^51^, the proteins were subjected to activity assays to probe such scenarios. All activity assays of the modified proteins, however, showed no statistically relevant alteration of protein function compared to the non-modified proteins (Supplementary Figs. 162 and 167). We speculate that the formation of the covalent linkage between two nucleophilic amino acids only takes place if the two residues are already located in close proximity to each other and therefore does not influence the tertiary protein structure and the function of the protein upon intramolecular reaction. Dose-dependence experiments with sodium azide revealed that the presence of 40 mM of exogenous nucleophile is sufficient to ensure the formation of the desired product (Supplementary Fig. 110). The fact that different nucleophiles can be installed only four atoms remote from the amide backbone allows for modulation of the protein in close proximity to its surface.

### Peptide stapling with vinyl thianthrenium

**Fig. 4:**
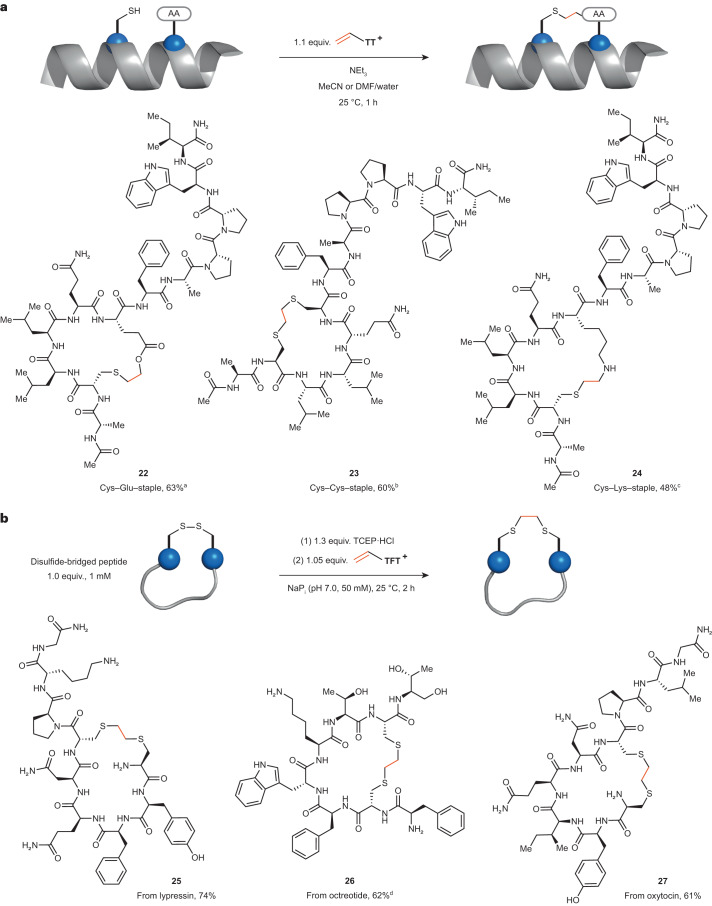
Utilization of vinyl thianthreniums for stapling and macrocyclization of peptides. **a**, Stapling of nucleophilic amino acids in native peptides with **VTT**. ^a^MeCN:H_2_O 9:1, 2.2 equiv. NEt_3_. ^b^DMF:H_2_O 1:1, 5.0 equiv. NEt_3_, ^c^MeCN:H_2_O 9:1, 3.0 equiv. NEt_3_. **b**, Macrocyclization of reduced disulfide bonds with **VTFT**. ^d^NaP_i_ (pH 6.5, 50 mM). AA, amino acid.

We hypothesized that the intramolecular reactivity of the episulfonium intermediate with nucleophilic amino acids can be utilized for the synthesis of macrocyclic, stapled peptides. Peptide stapling can induce and stabilize helicity in peptides, which increases metabolic stability^52^ – a major goal in the development of peptide-based therapeutics^53^. Stapling of natural amino acids is attractive because it allows the use of native peptides to avoid the synthesis of unnatural amino acids and peptides^54^. We used **VTT** to introduce the short, stable ethylene linker into peptides containing Cys and another nucleophilic amino acid in an *i*, *i* + 4 relationship^55^. Cys–Lys, Cys–Cys and Cys–Glu linkages were introduced to form stapled peptides in 48–63% isolated yield (Fig. 4a). This strategy provides an excellent method for stapling of three different pairs of natural amino acids, including non-activated carboxylic acids, via a very small hydrocarbon-based linker. Vinyl thianthrenium salts can also be used to irreversibly transform labile disulfide bonds in pharmaceutically relevant peptides into thioethers and provide immediate access to stable disulfide mimetics under physiological conditions. Macrocyclic analogues of oxytocin, octreotide and lypressin were obtained directly from the native peptides in 61–74% isolated yield (Fig. 4b).

### Quantitative proteomics and protein–protein cross-linking

**Fig. 5:**
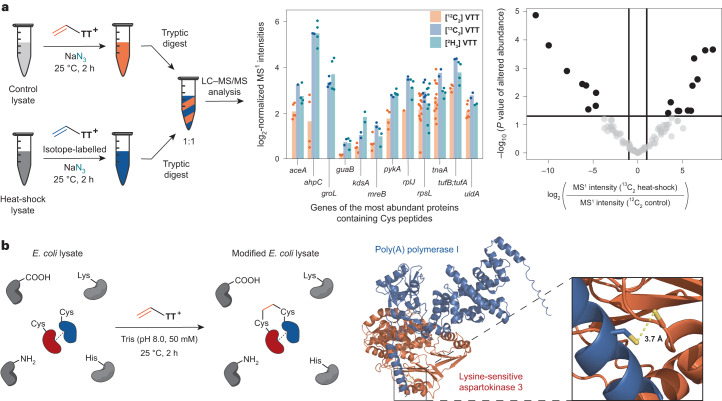
Applications of VTT in proteomics. **a**, Application of **VTT** isotopologues in quantitative proteomics. Reaction with control and heat-shock lysates from *E. coli* cultures incubated at 37 °C or 43 °C, respectively, prior to lysis; mean of MS^1^ intensities from Cys-containing peptides functionalized with isotopologues of **VTT** and their respective protein origins. Data are derived from two technical replicates; volcano-plot with auxiliary lines resulting from two-sided linear models for microarray data (LIMMA)-based differential expression analysis^60^ with *P* < 0.05 derived from MS^1^ intensities of identified peptides based on two technical replicates. All *P* values are associated with empirical Bayes moderated *t*-statistics without adjustments for multiple comparisons. The horizontal auxiliary line indicates the limit of significance based on *P* < 0.05. The vertical auxiliary lines indicate the values at which the protein populations have at least halved or doubled. **b**, Identified and simulated PPI between poly(A) polymerase I and lysine-sensitive aspartokinase 3 via **VTT** induced cross-linking in an *E. coli* lysate. MS^1^, ion spectra of intact peptides. Source data

The scalability of and easy synthetic access to **VTT** and **VTFT** from ethylene allows for a straightforward synthesis of isotopically enriched reagents for advanced applications in mass spectrometry (MS). For example, MS-based quantitative proteomics relies on isotope-enriched reagents and is used to quantify proteins^56^ or redox states^57^ in complex native systems. We accomplished the synthesis of **[**^**2**^**H**_**3**_**]VTT** and **[**^**13**^**C**_**2**_**]VTT** isotopologues on a decagram scale in a single step. To evaluate whether the reagents can serve to read out and quantify cellular stress responses, we performed a heat-shock experiment with *E. coli* as a model organism. We treated *E. coli* lysates derived from cell cultures that were incubated at 37 °C and 43 °C with unlabelled and labelled **VTT**, respectively, in the presence of azide. Equimolar mixtures of unlabelled and labelled tryptic digests of the lysates were then analysed via LC–MS/MS. The 1:1 mixture of ^12^C- and ^13^C-modified lysates revealed 244 Cys-containing peptides, of which 20 showed a statistically significant change in abundance caused by the elevated temperature (Fig. 5a). The same experiment with ^2^H_3_-labelled **VTT** resulted in a Pearson correlation coefficient of 0.76 when compared to the results with ^13^C-labelled **VTT**. The reliability of the experimental set-up was further supported by data obtained for the *groL* gene that codes for a heat-shock protein^58^ (Fig. 5a). Heat-shock proteins are expressed to ensure proper folding of other proteins under extreme conditions, and the corresponding peptides were only found in the lysates incubated at 43 °C. If desired, the introduced azide can be utilized to attach an additional affinity handle after the initial functionalization step to increase sensitivity of the whole set-up^15,16,40^. Hence, vinyl thianthrenium salts can be used for isotope-coded affinity tagging strategy with adjustable sensitivity induced by the choice of nucleophile.

A salient feature of **VTT** is its ability to introduce a short two-carbon linker, which may identify protein–protein interactions (PPIs) that are not detectable with other reagents^59^. To test this hypothesis, we treated *E. coli* lysates with **VTT** in the absence of additional exogenous nucleophiles. Tryptic digestion and LC–MS/MS analysis led to the identification of a previously unappreciated PPI, discovered through a Cys–Cys cross-link between lysine-sensitive aspartokinase 3 and poly(A) polymerase I. Based on the short length of the ethylene linkage and the identity and the position of the cross-linked amino acids, a protein-docking simulation with geometrical restraints from 2 to 6 Å was performed. The calculation indicates a PPI between the two proteins where both Cys residues are located 3.7 Å apart from each other (Fig. 5b). The same PPI was detected in similar abundance when the heat-shock lysate was modified with isotope-labelled **VTT** derivatives. Currently, no other Cys–Cys cross-linking reagents appear to be available that would furnish such a short linkage and, hence, be able to link two residues so proximal to one another. Moreover, there appears to be no comparable system that provides a quantifiable tool for applications in Cys tagging and cross-linking based on a single reagent.

## Conclusions

Cys conjugation is a state-of-the-art tactic to introduce modifications into biomolecules, but access to a variety of modifications currently requires substantial synthetic effort to prepare new reagents or is complicated by the low reactivity of introduced linchpins. Vinyl thianthrenium salts unite versatility and site selectivity, and lead to a common, reactive, cationic species that can be diversified to a large battery of conjugates with a short two-carbon linker. The single set of a few globally applicable and practical thianthrenium salts, including their readily accessible isotopologues, permits user-friendly applications in quantitative derivatization and analyses of single peptides, proteins and complex protein mixtures in different important disciplines in chemical biology.

## Methods

### General protein modification protocol

At 20–25 °C, the protein starting material in NaP_i_, HEPES or Bis-Tris buffer (pH 7.0, 50 mM) was added to a 1.5 ml Eppendorf tube. Next, the protein solution was diluted with buffer and TCEP (5–10 equiv.) in ultra-high-quality (UHQ)-H_2_O was added. The mixture was vortexed for 1 s, transferred into a Thermocycler preheated at 37 °C, and incubated at 37 °C for 1 h at 400 r.p.m. Next, a nucleophile stock solution in the respective buffer (pH 7.0, 50 mM) was added to the mixture at 20–25 °C to obtain a nucleophile concentration of 3–100 mM and a protein concentration of 15–30 µM. The nucleophile concentration varies depending on the chosen nucleophile (Supplementary Information). Next, a vinyl thianthrenium salt (12–30 equiv.) was added as a stock solution in dimethylformamide (DMF). The amount of DMF should not exceed 1% v/v of the whole mixture. The reaction mixture was vortexed for 1 s, transferred into a Thermocycler preheated at 25 °C, and incubated at 25 °C at 400 r.p.m. for either 5 min (**VTFT**) or 30 min (**VTT**).

### Protein modification protocol for thiol nucleophiles

At 20–25 °C, the protein starting material in NaP_i_, HEPES or Bis-Tris buffer (pH 7.0, 50 mM) was added to a 1.5 ml Eppendorf tube. Next, the protein solution was diluted with buffer and TCEP (5–10 equiv.) in UHQ-H_2_O was added. The mixture was vortexed for 1 s, transferred into a Thermocycler preheated at 37 °C and incubated at 37 °C for 1 h at 400 r.p.m. Next, a sodium iodide stock solution in the respective buffer (pH 7.0, 50 mM) was added to the mixture at 20–25 °C to obtain an iodide concentration of 40 mM and a protein concentration of 15–30 µM. Next, 15–30 equiv. of **VTFT** was added as a stock solution in DMF. The amount of DMF should not exceed 1% v/v of the whole mixture. The reaction mixture was vortexed for 1 s, transferred into a Thermocycler preheated at 25 °C, and incubated at 25 °C at 400 r.p.m. for exactly 3 min. Immediately afterwards, a thiol stock solution in the respective buffer (pH 7.0 or 8.0, 50 mM) was added to the mixture to obtain a nucleophile concentration of 20–50 mM. The concentration varies depending on the chosen thiol (Supplementary Information). The mixture was incubated at 25 °C at 400 r.p.m. for 30 min.

### Peptide stapling

At 20–25 °C, a screwcap vial (20 ml) was charged with peptide **S10** (10.0 mg, 7.00 µmol, 1.00 equiv.) and a Teflon-coated magnetic stirring bar. MeCN (4.5 ml) and UHQ-H_2_O (500 µl) were added and the resulting solution was stirred for 3 min. Then, NEt_3_ (2.15 µ, 1.56 mg, 15.4 µmol, 2.20 equiv.) was added and the mixture was stirred again for 3 min. Next, a 1 M solution of **VTT** in DMF (7.70 µl, 7.70 µmol, 1.10 equiv.) was added and the solution was stirred for 1 h. The reaction mixture was filtered and the filtrate was purified by HPLC. Fractions containing the product were collected and lyophilized to afford the stapled peptide **22** as a colourless powder (6.4 mg, 4.4 µmol, 63% yield).

### Disulfide rebridging

At 20–25 °C, a round-bottom flask (25 ml) was charged with Lypressin (10.5 mg, 9.94 µmol, 1.00 equiv.) and a Teflon-coated magnetic stirring bar. Then, 5.0 ml of sodium phosphate buffer (pH 7.0, 100 mM) and 4.1 ml of UHQ-H_2_O were added to the flask and the starting material was dissolved while stirring at 300 r.p.m. Next, a freshly prepared 20 mM solution of TCEP·HCl in UHQ-H_2_O (0.65 ml, 13 µmol, 1.3 equiv.) was added to the flask and the mixture was stirred at 300 r.p.m. for 1.5 h at 25 °C. A 50 mM solution of **VTFT** in DMF (0.21 ml, 10 µmol, 1.1 equiv.) was added dropwise over 1 min and the mixture was stirred for 15 min. The reaction mixture was filtered and the filtrate was purified by HPLC. Fractions containing the product were collected and lyophilized to afford the stapled peptide as a colourless powder (9.7 mg, 7.4 µmol, 74% yield).

### Lysate labelling for quantitative proteomics

At 20–25 °C, 6.0 µl of non-stressed control or heat-shock lysate (8.6–8.7 mg ml^−1^, 7.8 × 10^2^ µM Cys, 5.0 nmol Cys, 1.0 equiv.) in NaP_i_ buffer (pH 7.0, 50 mM) was added to a 1.5 ml Eppendorf tube and diluted with 0.13 ml of NaP_i_ buffer (pH 7.0, 50 mM). Subsequently, 5.0 µl of a TCEP stock solution (10 mM, 50 nmol, 13 µg, 10 equiv.) in UHQ-H_2_O was added to the mixture. The mixture was vortexed for 1 s, transferred into a Thermocycler preheated at 37 °C and incubated at 37 °C for 1 h at 400 r.p.m. Next, a 0.30 M sodium azide stock solution (30 µl, 9.0 µmol, 0.59 mg, 1.8 × 10^3^ equiv.) in NaP_i_ buffer (pH 7.0, 50 mM) was added to the mixture at 20–25 °C (nucleophile concentration = 38 mM), followed by addition of 0.5 µl of a **VTT** (**[**^**13**^**C**_**2**_**]VTT** or **[**^**2**^**H**_**3**_**]VTT** for the heat-shock lysate) stock solution (80 mM, 0.04 µmol, 0.01 mg, 8 equiv.) in DMF. The reaction mixture was vortexed for 1 s, transferred into a Thermocycler preheated at 25 °C and incubated at 25 °C at 400 r.p.m. for 120 min. Subsequently, 22 µl of a β-mercaptoethanol stock solution (0.15 M, 3.3 µmol, 6.6 × 10^2^ equiv.) in UHQ-H_2_O was added, and the mixture was incubated at 25 °C for 30 min.

### Reporting summary

Further information on research design is available in the Nature Portfolio Reporting Summary linked to this article.

## Online content

Any methods, additional references, Nature Portfolio reporting summaries, source data, extended data, supplementary information, acknowledgements, peer review information; details of author contributions and competing interests; and statements of data and code availability are available at 10.1038/s41557-023-01388-7.

### Supplementary information

Supplementary Information Supplementary Figs. 1–228, Tables 1–40, Discussion, kinetic data, MS of proteins, NMR data of proteins and small molecules, enzyme assays and proteomics experiments.

Reporting Summary

### Source data

Source Data Fig. 2 Numerical source data.

Source Data Fig. 5 Numerical source data.

## Data Availability

Raw SDS–PAGE, enzyme assay, protein LC–MS, LC–MS/MS and NMR data are deposited in the repositories: ownCloud (https://owncloud.gwdg.de/index.php/s/uf0bv5a6HLfIfcW), jPOSTrepo (https://repository.jpostdb.org/entry/JPST001937), BioMagRes-Bank (https://bmrb.io/; entry IDs 51721, 51725) and Zenodo (10.5281/zenodo.7472436). Source data are provided with this paper.
