# Author Correction: Chemoselective umpolung of thiols to episulfoniums for cysteine bioconjugation

**DOI:** 10.1038/s41557-024-01475-3

**Published:** 2024-02-15

**Authors:** Philipp Hartmann, Kostiantyn Bohdan, Moritz Hommrich, Fabio Juliá, Lara Vogelsang, Jürgen Eirich, Rene Zangl, Christophe Farès, Julia Beatrice Jacobs, Dwaipayan Mukhopadhyay, Johanna Marie Mengeler, Alessandro Vetere, Marie Sophie Sterling, Heike Hinrichs, Stefan Becker, Nina Morgner, Wolfgang Schrader, Iris Finkemeier, Karl-Josef Dietz, Christian Griesinger, Tobias Ritter

**Affiliations:** 1 Max-Planck-Institut für Kohlenforschung, Mülheim an der Ruhr, Germany; 2 Institute of Organic Chemistry, RWTH Aachen University, Aachen, Germany; 3 Biochemistry and Physiology of Plants, Faculty of Biology, Bielefeld University, Bielefeld, Germany; 4 Institute of Plant Biology and Biotechnology, University of Münster, Münster, Germany; 5 Institute of Physical and Theoretical Chemistry, Goethe University Frankfurt/Main, Frankfurt/Main, Germany; 6 Max Planck Institute for Multidisciplinary Sciences, Göttingen, Germany

**Keywords:** Synthetic chemistry methodology, Reaction mechanisms

Correction to: *Nature Chemistry* 10.1038/s41557-023-01388-7, published online 20 December 2023.

In the version of the article initially published, the Data availability section included an outdated link for jPOSTrepo. This has been amended to https://repository.jpostdb.org/entry/JPST001937 in the HTML and PDF versions of the article.

