# Supplementary material for: Chemoselective umpolung of thiols to episulfoniums for cysteine bioconjugation
Source: Nat Chem. 2023 Dec 20;16(3):380–8. doi: 10.1038/s41557-023-01388-7 (PMC10914617; doi:10.1038/s41557-023-01388-7)
Supplement: Supplementary file 1 — Supplementary Figs. 1–228, Tables 1–40, Discussion, kinetic data, MS of proteins, NMR data of proteins and small molecules, enzyme assays and proteomics experiments. [file 41557_2023_1388_MOESM1_ESM.pdf]

nature chemistry

Article https://doi.org/10.1038/s41557-023-01388-7

Chemoselective umpolung of thiols to episulfoniums for cysteine bioconjugation

In the format provided by the authors and unedited

TABLE OF CONTENTS

TABLE OF CONTENTS 1

MATERIALS AND METHODS 8

- Starting Materials 8
- Solvents 9
- Chromatography 9
- Mass spectrometry 9
- NMR Spectroscopy 9
- Liquid chromatography-mass spectrometry (LC-MS) 10
  - Analytical LC-MS measurements of small molecules and peptides 10
  - Preparative HPLC separations 10
  - Analytical LC-MS measurements of proteins 10
  - High resolution Mass Spectrometry (HRMS) measurements for proteins 12
  - LC-MS/MS analysis 12
  - SDS-PAGE 13
- Miscellaneous 13

EXPERIMENTAL DATA 14

Synthesis of vinyl-thianthrenium salts 14

- Preparation of **VTT** 14
- Preparation of **VTFT** 14
- Preparation of ^{2}**H$_3$-VTT** from ^{2}H-labeled ethylene 15
- Preparation of ^{13}**C$_2$-VTT** from ^{13}C-labeled ethylene 16
- Preparation of ^{2}**H$_3$-VTFT** from ^{2}H-labeled ethylene 17
- Preparation of ^{13}**C$_2$-VTFT** from ^{13}C-labeled ethylene 18
- Stability of vinylthianthrenium salts in DMF solutions 19
- Solubility of vinylthianthrenium salts in water 19
- Stability of tetrafluoroborate anion in a model reaction mixture 20

Synthesis of Glutathione derivatives with different nucleophiles 22

- Preparation of Glutathione–S–C_2H_4–N_3 trifluoroacetate (**1**) from **VTT** 22
- Preparation of Glutathione–S–C_2H_4–N_3 trifluoroacetate (**1**) from **VTFT** 23
- Preparation of Glutathione–S–C_2H_3D–N_3 trifluoroacetate (^{2}**H$_1$-1**) from **VTFT** 24
- Preparation of Glutathione–S–C_2H_4–SCN trifluoroacetate (**S1**) 26
- Preparation of Glutathione–S–C_2H_4–N-aniline trifluoroacetate (**S2**) 27
- Preparation of Glutathione–S–C_2H_4–N-(4-fluoroaniline) trifluoroacetate (**S3**) 29

Preparation of Glutathione–S–C_2H_4–N-(3-aminophenylboronic acid) trifluoroacetate (**S4**) 30

Synthesis of Cysteine derivatives with different nucleophiles 32

Preparation of Cysteine–S–C_2H_4–thioglucose (**S5**) 32

Preparation of Cysteine–(S–C_2H_4)$_2$–dimethylammonium trifluoroacetate (**S6**) 33

Preparation of Cysteine–S–C_2H_4–thiosulfate (**S7**) 34

Synthesis of *p*-nitrothiophenol derivatives 35

Thiophenol-substituted N-methylmaleimide (**S8**) 35

Thiophenol-derived azide (**S9**) 35

Peptide stapling and disulfide rebridging 37

General considerations for peptide synthesis 37

General set-up 37

Loading and deprotection of the resin 37

Coupling of amino acids to the resin 38

Fmoc-Deprotection of coupled amino acids 38

Cleavage of the peptide from the resin 38

Synthesis of Linear Peptides via SPPS 39

Ac-Ala-Cys-Leu-Leu-Gln-Glu-Phe-Ala-Pro-Pro-Trp-Ile-NH_2 (**S10**) 39

Ac-Ala-Cys-Leu-Leu-Gln-Cys-Phe-Ala-Pro-Pro-Trp-Ile-NH_2 (**S11**) 43

Ac-Ala-Cys-Leu-Leu-Gln-Lys-Phe-Ala-Pro-Pro-Trp-Ile-NH_2 (**S12**) 47

Synthesis of stapled peptides 51

Cys-Glu stapling with Ac-Ala-Cys-Leu-Leu-Gln-Glu-Phe-Ala-Pro-Pro-Trp-Ile-NH_2 (**22**) 51

Cys-Cys stapling with Ac-Ala-Cys-Leu-Leu-Gln-Cys-Phe-Ala-Pro-Pro-Trp-Ile-NH_2 (**23**) 52

Cys-Lys stapling with Ac-Ala-Cys-Leu-Leu-Gln-Lys-Phe-Ala-Pro-Pro-Trp-Ile-NH_2 (**24**) 54

Disulfide rebridging 55

Preparation of ethylene-bridged Lypressin (**25**) 55

Preparation of ethylene-bridged Octreotide (**26**) 57

Preparation of ethylene-bridged Oxytocin (**27**) 58

Functionalization of proteins 60

Quantification of conversion for protein modifications 60

Protein substrate scope 60

Functional group scope 60

Data on protein starting materials 60

DHAR1 61

DHAR1 S97C 61

DHAR1 S176C 62

sfGFP S147C 63

Ubiquitin T9C 63

Ubiquitin T12C 65

DHAR2 C6S 66

PrxIIB C76S 67

Trxh1 C43S 68

^{15}N,^{13}C-labeled ubiquitin T12C 69

Ubiquitin T12C (B) 69

Bovine Serum Albumin (BSA) 69

MDAR2 70

MDAR3 71

Data availability 72

Protein expression and purification 72

Site-directed-mutagenesis protocol 72

Generation of plasmids 72

Plasmid amplification 73

Protein expression and purification 74

His-tag cleavage of Ubiquitin proteins 74

Expression and purification of ^{15}N,^{13}C-lableled ubiquitin T12C and ubiquitin T12C (B) 74

Mass spectrometry of lysates 75

Heat treatment of *E. coli* and preparation of cell lysates 75

Selectivity studies with lysates 75

Cysteine coverage rate calculation 79

Quantitative Proteomics 80

Preparation of conjugated control cell lysates 80

Preparation of conjugated heat-shock cell lysates with $^{13}C_2$ labeling 81

Preparation of conjugated heat-shock cell lysates with $^{2}H_3$ labeling 81

Preparation of cross-linked cell lysates with **VTT** 82

LC-MS/MS analysis of heat-shock and control cell lysates 84

LC-MS/MS analysis for cross-link search 85

Protein-docking simulation 86

Data availability 86

Protein scope for azidation 86

Synthesis of (sfGFP S147C)–S–C_2H_4–N_3 (**2**) 86

Synthesis of (DHAR2 C6S)–S–C_2H_4–N_3 (**3**) 88

Synthesis of (Ubiquitin T9C)–S–C_2H_4–N_3 (**4**) 89

Synthesis of (PrxIIB C76S)–S–C_2H_4–N_3 (**5**) 90

Synthesis of (MDAR3)–S–C_2H_4–N_3 (**6**) 91

Synthesis of (BSA)–S–C_2H_4–N_3 (**7**) 92

Synthesis of (Trxh1 C43S)–S–C_2H_4–N_3 (**8**) 93

Synthesis of (DHAR1 S97C)–S–C_2H_4–N_3 (**9**) 94

Synthesis of (DHAR1 S176C)–S–C_2H_4–N_3 (**10**) 96

Synthesis of (MDAR2)–S–C_2H_4–N_3 (**S13**) 97

Assessment of side-reactivity and quantification for protein modifications 98

Functional group scope with Ubiquitin T12C 99

Synthesis of (Ubiquitin T12C)–S–C_2H_4–N_3 (**11**) 100

Synthesis of (Ubiquitin T12C)–S–C_2H_4–^{15}N-aniline (**12**) 101

Synthesis of (Ubiquitin T12C)–S–C_2H_4–N-(4-F-aniline) (**13**) 103

Synthesis of (Ubiquitin T12C)–S–C_2H_4–N-(3-aminophenylboronic acid) (**14**) 105

Synthesis of (Ubiquitin T12C)–S–C_2H_4–SCN (**15**) 107

Synthesis of (Ubiquitin T12C)–S–C_2H_4–S-thiosulfate (**16**) 108

Synthesis of (Ubiquitin T12C)–S–C_2H_4–S-thiophosphate (**17**) 110

Synthesis of (Ubiquitin T12C)–S–C_2H_4–S-thioglucose (**18**) 111

Synthesis of (Ubiquitin T12C)–S–C_2H_4–SAc (**19**) 113

Synthesis of (Ubiquitin T12C)–S–C_2H_4–dimethylamine (**20**) 114

Synthesis of (Ubiquitin T9C)–S–C_2H_4–S–PEG11–biotin (**21**) 116

Synthesis of (Ubiquitin T12C)–S–C_2H_4–S–PEG11–biotin (**S14**) 118

Assessment of side-reactivity and quantification of protein modifications 119

Synthesis of conjugates for protein NMR studies 122

Synthesis of (^{15}N,^{13}C-Ubiquitin T12C)–S–$^{13}C_2H_4$–N_3 (**S15**) 122

Synthesis of Ubiquitin T12C–S–C_2H_4–N_3 (**S16**) 123

Synthesis of (^{15}N,^{13}C-Ubiquitin T12C)–S–$^{13}C_2H_4$–(4-F-aniline) (**S17**) 124

Synthesis of azidated conjugates for follow-up transformations 124

Synthesis of Ubiquitin T9C–S–C_2H_4–N_3 (**4**) 124

Synthesis of Ubiquitin T12C–S–C_2H_4–N_3 (**11**) 125

Synthesis of (BSA)–S–C_2H_4–N_3 (**7**) 126

Synthesis of (MDAR2)–S–C_2H_4–N_3 (**S13**) 127

Synthesis of (MDAR3)–S–C_2H_4–N_3 (**6**) 128

Modification of the installed azide-group via Click-chemistry 128

CuAAC between (Ubiquitin T9C)–S–C_2H_4–N_3 and 6-FAM 128

CuAAC between (Ubiquitin T12C)–S–C_2H_4–N_3 and 6-FAM 129

CuAAC between (BSA)–S–C_2H_4–N_3 and 6-FAM 130

CuAAC between (MDAR2)–S–C_2H_4–N_3 and 6-FAM 131

CuAAC between (MDAR3)–S–C_2H_4–N_3 and 6-FAM 131

SPAAC between (Ubiquitin T9C)–S–C_2H_4–N_3 and BCN 132

SPAAC between (Ubiquitin T12C)–S–C_2H_4–N_3 and BCN 133

SDS PAGE of functionalized proteins 134

Ubiquitin T9C and Ubiquitin T12C functionalized with azide and 6-FAM 134

BSA, MDAR2, MDAR3 functionalized with azide and 6-FAM 134

Control Experiments 135

Detailed analysis of a model LC-MS chromatogram 135

Estimation of protein loss during the reaction 136

Comparison of LC-MS measurements with and without UV 138

Influence of c(Nu) on the formation of the hydrolysis product 139

Influence of pH on the formation of the hydrolysis product 140

Reaction with and without pre-reduction with TCEP 143

Experiment with IAA-precapping 145

Evaluation of the reaction time of **VTFT** with sfGFP 147

Evaluation of the reaction time of **VTT** with sfGFP 148

Evaluation of the stability of conjugated proteins 149

Influence of the counter-anion of **VTT** 151

Investigation of the tertiary structure preservation after the reaction with **VTT** 152

Reaction of DHAR1 S97C and **VTT** without exogenous nucleophile 152

Reaction of DHAR1 S176C and **VTT** without exogenous nucleophile 155

Reaction of sfGFP S147C and **VTT** without exogenous nucleophile 158

Investigation of site-specific functionalization of DHAR1 mutants 160

Synthesis of (DHAR1 S97C)–S–C_2H_4-N_3 with 11 equiv. **VTT** 160

Synthesis of (DHAR1 S97C)–S–C_2H_4-N_3 with 20 equiv. **VTT** 161

Synthesis of (DHAR1 S176C)–S–C_2H_4-N_3 with 11 equiv. **VTT** 162

Synthesis of (DHAR1 S176C)–S–C_2H_4-N_3 with 20 equiv. **VTT** 163

Synthesis of (DHAR1 S97C)–(S–NMM)$_2$ with 11 equiv. **NMM** 164

Synthesis of (DHAR1 S176C)–(S–NMM)$_2$ with 11 equiv. **NMM** 165

Synthesis of (DHAR1 S97C)–(S–NMM)$_2$ with 20 equiv. **NMM** 166

Synthesis of (DHAR1 S176C)–(S–NMM)$_2$ with 20 equiv. **NMM** 167

Reaction of DHAR1 with different maleimide reagents 168

Screening of different maleimide concentration in the conjugation with DHAR1 mutants 172

DHAR-activity assays 174

Screening of different NMM concentrations..........175

Activity comparison after functionalization with **NMM** or **VTT** + NaN_3 177

DHAR1 activity after modification with **VTT** without NaN_3 181

sfGFP S147C fluorescence assay 185

Mechanistic studies..........188

UV-vis Absorption Measurements..........188

Kinetic Reaction Profile for **VTT** 189

Reaction order in **GSH** 189

Reaction order in **VTT** 191

Reaction order in NaN_3..........193

Determination of the second-order rate constant for **VTT**..........195

Determination of the second-order rate constant for **$^2H_3$-VTT** 197

Kinetic isotope effect for functionalization of **GSH** with **VTT** 198

Competition experiment between **VTT** and **$^2H_3$-VTT**..........199

Kinetic Reaction Profile for **VTFT** 200

Reaction order in **GSH** 201

Reaction order in **VTFT**..........203

Reaction order in NaN_3..........205

Determination of the second-order rate constant for **VTFT**..........207

Competition experiment between **VTFT** and **$^2H_3$-VTFT** 209

Deuterium incorporation in deuterated buffer..........211

Reactivity of **VTT** and **VTFT** with NaN_3 213

Proposed mechanism for **GSH** functionalization..........214

Stability comparison with a maleimide conjugate..........215

Characterization of the in-situ generated iodide-intermediate..........216

HRMS-characterization 216

NMR-characterization..........218

NMR Experiments with Glutathione 220

NMR DATA 226

NMR-Characterization of vinyl-thianthrenium reagents 226

NMR-Characterization of glutathione derivatives 242

Glutathione–S–C_2H_4–N_3 trifluoroacetate (**1**)..........242

Glutathione–S–C_2H_3D–N_3 trifluoroacetate (**$^2H_1$-1**)..........253

Glutathione–S–C_2H_4–SCN trifluoroacetate (**S1**) 263

Glutathione–S–C_2H_4–N-aniline trifluoroacetate (**S2**): 273
Glutathione–S–C_2H_4–N-(4-fluoroaniline) trifluoroacetate (**S3**): 283
Glutathione–S–C_2H_4–N-(3-Aminophenylboronic acid) trifluoroacetate (**S4**): 293

NMR-Characterization of cysteine derivatives 304

Cysteine–S–C_2H_4–thioglucose (**S5**) 304
Cysteine–(S–C_2H_4)$_2$–dimethylammonium trifluoroacetate (**S6**) 312
Cysteine–S–C_2H_4–thiosulfate (**S7**): 322

NMR-Characterization of peptides 329

Ac-Ala-Cys-Leu-Leu-Gln-Glu-Phe-Ala-Pro-Pro-Trp-Ile-NH_2 (**S10**): 329
Ac-Ala-Cys-Leu-Leu-Gln-Cys-Phe-Ala-Pro-Pro-Trp-Ile-NH_2 (**S11**): 346
Ac-Ala-Cys-Leu-Leu-Gln-Lys-Phe-Ala-Pro-Pro-Trp-Ile-NH_2 (**S12**): 362
Stapled Ac-Ala-Cys-Leu-Leu-Gln-Glu-Phe-Ala-Pro-Pro-Trp-Ile-NH_2 (**22**): 379
Stapled Ac-Ala-Cys-Leu-Leu-Gln-Cys-Phe-Ala-Pro-Pro-Trp-Ile-NH_2 (**23**): 395
Stapled Ac-Ala-Cys-Leu-Leu-Gln-Lys-Phe-Ala-Pro-Pro-Trp-Ile-NH_2 (**24**): 411
Ethylene-bridged Lypressin (**25**) 430
Ethylene-bridged Octreotide (**26**) 443
Ethylene-bridged Oxytocin (**27**) 458

Protein NMR 472

General sample preparation and measurements 472
13C-HSQC of (^{15}N,^{13}C-Ubiquitin T12C)–S–$^{13}C_2H_4$–N_3 (**S15**) 473
^{15}N-HSQC of (^{15}N,^{13}C-Ubiquitin T12C)–S–$^{13}C_2H_4$–N_3 (**S15**) 476
NMR shifts for (^{15}N,^{13}C-Ubiquitin T12C)–S–$^{13}C_2H_4$–N_3 (**S15**) 477
NOESY-correlation between (Ubiquitin T12C)–S–C_2H_4–N_3 and Ubiquitin T12C 488
^{19}F NMR of (^{15}N,^{13}C-Ubiquitin T12C)–S–$^{13}C_2H_4$–(4-F-aniline) 497

Miscellaneous NMR Data 498

MS/MS DATA 502

MS/MS-Characterization of stapled peptides 502

Stapled Ac-Ala-Cys-Leu-Leu-Gln-Glu-Phe-Ala-Pro-Pro-Trp-Ile-NH_2 (**22**) 502
Stapled Ac-Ala-Cys-Leu-Leu-Gln-Cys-Phe-Ala-Pro-Pro-Trp-Ile-NH_2 (**23**) 505
Stapled Ac-Ala-Cys-Leu-Leu-Gln-Lys-Phe-Ala-Pro-Pro-Trp-Ile-NH_2 (**24**) 508
Ethylene-bridged Lypressin (**25**) 512
Ethylene-bridged Octreotide (**26**) 515
Ethylene-bridged Oxytocin (**27**) 518

REFERENCES 521

MATERIALS AND METHODS

Starting Materials

All substrates were used as received from the commercial suppliers:

Material	Vendor	Purity
^{13}C-ethylene	Sigma-Aldrich	99%
^{15}N-aniline	Sigma-Aldrich	98%
^{2}H-ethylene	Sigma-Aldrich	99%
3-(Trimethylsilyl)propionic-2,2,3,3 acid sodium salt d4	Sigma-Aldrich	98%
3-Aminobenzeneboronic acid	Chempur	98%
4-Fluoroaniline	Sigma-Aldrich	99%
6-FAM-Alkyne	Jena Bioscience	>95%
Ac-L-Ala-OH	Alfa Aesar	96%
Ammonium acetate	Chempur	>98%
Anisole	Acros	>99%
BCN	BLD Pharm	90%
Biotin-PEG11-SH	Polypure	99%
Bis-(2-hydroxy-ethyl)-amino-tris(hydroxymethyl)-methane	abcr	98%
Pierce™ Bovine Serum Albumin	Thermo Fisher	100%
$CuSO_4$	Sigma Aldrich	>99%
Diisopropylcarbodiimide	Iris Biotech	>99%
Dimethylaminoethanethiol hydrochloride	Fisher Scientific	95%
Dithiothreitol	Chempur	>99%
DMF (peptide grade)	Iris Biotech	>99%
Ethane-1,2-dithiol	Sigma-Aldrich	>98%
Fmoc-L-Ala-OH	abcr	95%
Fmoc-L-Cys(Trt)-OH	Iris Biotech	>99%
Fmoc-L-Gln(Trt)-OH	Iris Biotech	>99%
Fmoc-L-Glu(OtBu)-OH	Novabiochem	>99%
Fmoc-L-Ile-OH	Sigma-Aldrich	98%
Fmoc-L-Leu-OH	Sigma-Aldrich	>97%
Fmoc-L-Phe-OH	Sigma-Aldrich	98%
Fmoc-L-Pro-OH	Sigma-Aldrich	>99%
Fmoc-L-Trp(Boc)-OH	Iris Biotech	99%
Glycinamide hydrochloride	Chempur	98%
HEPES	TCI	>99%
L-Cysteine	Merck	>99%
L-Glutathione	TCI	>98%
Lypressin	Biosynth	>94%
Magnesium sulfate	Fisher Scientific	99%
Nitric acid	Sigma-Aldrich	>99%
N-methylmaleimide	TCI	>98%
N-phenylmaleimide	TCI	97%
N-(4-Carboxyphenyl)maleimide	Chempur	96%
Octreotide acetate	Chempur	>98%
Oxyma	Iris Biotech	>99%
Oxytocin	Fluorochem	>98%

Piperidine	IRIS Biotech	>99%
Potassium thioacetate	abcr	98%
Fmoc-Rink-Amide-2CT resin	Iris Biotech	n/a
Sodium ascorbate	Alfa Aesar	99%
Sodium azide	Fluka	>99%
Sodium bicarbonate	Acros	99%
Sodium formate	Sigma-Aldrich	>99%
Sodium hydroxide	VWR	97%
Sodium iodide	Sigma-Aldrich	>99%
Sodium phosphate monobasic monohydrate	Sigma-Aldrich	98%
Sodium tetrafluoroborate	Acros	98%
Sodium thiocyanate	Fluka	>98%
Sodium thiophosphate hydrate	Alfa Aesar	>95%
Sodium thiosulfate pentahydrate	Alfa Aesar	>99%
Thianthrene	Chempur	98%
Thioanisole	Sigma-Aldrich	99%
THPTA	TCI	>97%
Triethylamine	Sigma-Aldrich	>99%
Trifluoroacetic acid	abcr	99%
Trifluoroacetic anhydride	abcr	>99%
Tris(2-carboxyethyl)phosphine-hydrochloride	abcr	>98%
Tris-(hydroxymethyl)-methylamine	Alfa Aesar	>99%
β-D-Thioglucose sodium salt	Thermo Fisher	97%
β-Mercaptoethanol	TCI	>98%

Thianthrene-S-oxide, tetrafluorothianthrene, and tetrafluorothianthrene-S-oxide were synthesized according to literature reports[1,2].

Solvents

Water used to prepare buffers and as solvent was of ultra-high quality (UHQ) grade (18.2 $M\Omega \cdot cm^{-1}$). Methanol (>99%) was purchased from Sigma-Aldrich, dichloromethane (>99%) and acetonitrile (>99%) were purchased from Fisher Scientific. Dimethylformamide used to prepare vinyl thianthrenium stock solutions was purchased from Thermo Scientific (+99.8%).

Chromatography

Thin layer chromatography (TLC) was performed using EMD TLC plates pre-coated with 250 µm thickness silica gel 60 F254 plates and visualized by fluorescence quenching under UV light and $KMnO_4$ stain. Flash column chromatography was performed using silica gel (40–63 µm particle size) purchased from Geduran®.

Mass spectrometry

High resolution Mass Spectrometry (HRMS) experiments for small molecules and peptides were performed on a Thermo Scientific™ Q Exactive Plus or a Thermo Scientific™ Q Exactive GC Orbitrap device.

NMR Spectroscopy

Chemical shifts are reported in ppm (δ) relative to tetramethylsilane (TMS) with the solvent (residual) peak as the internal standard. For ^1H NMR: $CHCl_3$, δ 7.26; HDO, δ 4.79; $(CHD_2)(CD_3)NC(O)D$, δ 2.75; $(CHD_2)(CD_3)SO$,

δ 2.50; CHD_2CN, δ 1.94. For ^{13}C NMR: $CDCl_3$, δ 77.16; $(CD_3)_2NC(O)D$, δ 29.76; $(CD_3)_2SO$, δ 39.52; CD_3CN, δ 1.32[3]. ^{19}F and ^{15}N NMR spectra were referenced relative to ^{1}H using a unified chemical shift scale. Data are reported as follows: s = singlet, d = doublet, t = triplet, q = quartet, quint = quintet, m = multiplet, br = broad; coupling constants in Hz.

NMR spectra of small molecules and peptides were recorded on following instruments:

1. Bruker Avance III 500 spectrometer equipped with a BBFO probe head, operating at 500 MHz, 471 MHz, and 126 MHz, for ^{1}H, ^{19}F, and ^{13}C acquisitions, respectively.
2. Bruker Avance III 500 spectrometer equipped with a BBFO probe head, operating at 470 MHz for ^{19}F acquisitions.
3. AVANCE III 600 spectrometer equipped with a triple-channel “TCI” cryogenic probehead (Bruker GmbH, Rheinstetten) operating at 600 MHz, 61 MHz, and 151 MHz, for ^{1}H, ^{15}N, and ^{13}C acquisitions, respectively. All experiments used standard Bruker pulse sequence with standard parameter sets found in libraries of Topspin 3.6.

Protein NMR spectra were obtained with an AVANCE III 600 spectrometer equipped with a triple-channel “TCI” cryogenic probehead (Bruker GmbH, Rheinstetten). The 3D spectra employed a non-uniform sampling (25% or more) scheme in the indirect dimension and were reconstructed and processed by NMRPipe/NMRDraw. Backbone assignments were obtained using standard triple-resonance experiments (HNCO, HNCA, CBCAcoNH, HBHAcoNH). Aliphatic side-chain assignments were obtained from ^{13}C-edited 3D TOCSY (HcCH, hCCH, HcccoNH, and hCCcoNH) spectra. All spectra were analyzed with NMRFAM-SPARKY. Spectra were referenced indirectly to DSS.

Liquid chromatography-mass spectrometry (LC-MS)

Analytical LC-MS measurements of small molecules and peptides

Analytical LC-MS measurements of small molecules and peptides were performed on following instruments:

1. Agilent 1260 Infinity Automated LC System
2. Agilent 1290 infinity II
3. Shimadzu LCMS-2020

The utilized methods are described for each performed analysis.

Preparative HPLC separations

Preparative HPLC separations of small molecules and peptides were performed on a Shimadzu system using 2x LC-20AP, SIL-20A HT with 2 mL sample coil, CTO-20AC, SPD-20A variable cell 0.5 mm, CBM-20A, and FRC-10A modules.

Analytical LC-MS measurements of proteins

Method A:

Measurements were performed on Shimadzu LCMS-2020 coupled to a Shimadzu Nexera LC-20 system utilizing a Zorbax SB300-C8, 300 Å, 3.5 µm, 4.6x50 mm column. The following HPLC method was used for all

protein measurements with this setup:
Linear gradient from 20:80 v/v (0.1% v/v formic acid in MeCN : 0.1% v/v formic acid in H_2O) to 70:30 v/v (0.1% v/v formic acid in MeCN : 0.1% v/v formic acid in H_2O) over 15 minutes, followed by isocratic run for 5 minutes with 70:30 v/v (0.1% v/v formic acid in MeCN : 0.1% v/v formic acid in H_2O) followed by a gradient from 70:30 v/v (0.1% v/v formic acid in MeCN : 0.1% v/v formic acid in H_2O) to 20:80 v/v (0.1% v/v formic acid in MeCN : 0.1% v/v formic acid in H_2O) over 6 seconds at a flow rate of 0.5 mL / min.

LabSolutions 5.97 and UniDec 5.0.4 software was used for analysis and deconvolution.

Method B:

Measurements were performed on Shimadzu LCMS-9030 with SCL-40, 2x LC-40D XS, SIL-40C XS, CTO-40C, SPD-M40, equipped with an Agilent Zorbax 300SB-C8 column, 50 mm × 4.6 mm, 3.5 µm, flow rate 0.5 mL / min, 313 K.
Eluent A: 0.1% formic acid in UHQ-H2O; Eluent B: acetonitrile + 0.1% formic acid.
Using a linear gradient from 20% B to 70% B over 15 min, followed by an isocratic run over 5 min with 70% B, followed by a linear gradient to 20% B over 6 sec.
Absorption spectra were recorded at 214 nm (DAD), except for samples of MDAR2, MDAR3, sfGFP S147C, DHAR2 C6S and BSA, where no absorption spectra were recorded.
LabSolutions 5.114 and LabSolutions Insight Explore 3.8 SP4 software was used for analysis and deconvolution.

Method C:
Measurements were performed on a Shimadzu LCMS-9030 with SCL-40, 2x LC-40D XS, SIL-40C XS, CTO-40C, SPD-M40, equipped with an YMC Triart Bio C4, 50 mm × 3.0 mm, 1.9 µm, flow rate 0.2 mL / min, 313 K.
Eluent A: 0.1% formic acid in UHQ-H2O; Eluent B: acetonitrile + 0.1% formic acid.
Using a linear gradient from 20% B to 50% B over 15 min, followed by an isocratic run over 5 min with 50% B, followed by a linear gradient to 20% B in 6 sec.
LabSolutions 5.114 and LabSolutions Insight Explore 3.8 SP4 software was used for analysis and deconvolution.

Method D:
Measurements were performed on a Waters SYNAPT G2-S with a direct injection of the protein sample. The protein samples were first desalted *via* a Thermo Scientific™ Dionex Ultimate 3000 HPLC system with a Zorbax SB300-C3 column, 4.6x150mm. The following HPLC method was used for all protein measurements with this setup: linear gradient from 10:90 (0.1% formic acid in MeCN : 0.1% formic acid in H_2O, v/v) to 80:20 (0.1% formic acid in MeCN : 0.1% formic acid in H_2O, v/v) over 10 minutes, followed by gradient from 80:20 (0.1% formic acid in MeCN : 0.1% formic acid in H_2O, v/v) to 10:90 (0.1% formic acid in MeCN : 0.1% formic acid in H_2O, v/v) over 6 seconds and isocratic run with 10:90 (0.1% formic acid in MeCN : 0.1% formic acid in H_2O, v/v) over 10 minutes at a flow-rate of 0.5 mL / min.

UniDec 5.0.4 software was used for analysis and deconvolution.

Method E:

Measurements were performed on a Shimadzu LCMS-2020 with SCL-40, LC-40D XS, SIL-40C XS, CTO-40C, SPD-M40, equipped with an Agilent Zorbax 300SB-C3 column, 150 mm × 4.6 mm, 5 µm, flow rate 0.5 mL / min, 323 K.
Eluent A: 0.1% formic acid in UHQ-H2O; Eluent B: acetonitrile + 0.1% formic acid.
Using a linear gradient from 10% B to 80% B over 10 min, followed by a linear gradient to 10% B over 3 min.
Absorption spectra were recorded at 214 nm (DAD).
LabSolutions 5.114 and UniDec 5.0.4 software was used for analysis and deconvolution.

High resolution Mass Spectrometry (HRMS) measurements for proteins

High resolution Mass Spectrometry (HRMS) experiments for proteins were performed on following instruments: Thermo Scientific™ Orbitrap Elite™ coupled with a Thermo Scientific™ Dionex Ultimate 3000 LC system utilizing a Zorbax SB300-C3 column, 4.6 mm x 150 mm. The protein HRMS was performed with the most abundant ^{13}C-isotopologues within the molecular ions measured. The ionization was carried out *via* H-ESI at 275 °C. The following HPLC method was used for all protein measurements with this setup: linear gradient from 10:90 (0.1% formic acid in MeCN : 0.1% formic acid in H_2O, v/v) to 80:20 (0.1% formic acid in MeCN : 0.1% formic acid in H_2O, v/v) over 10 minutes, followed by gradient from 80:20 (0.1% formic acid in MeCN : 0.1% formic acid in H_2O, v/v) to 10:90 (0.1% formic acid in MeCN : 0.1% formic acid in H_2O, v/v) over 6 seconds and isocratic run with 10:90 (0.1% formic acid in MeCN : 0.1% formic acid in H_2O, v/v) over 10 minutes at a flow-rate of 0.5 mL / min.

LC-MS/MS analysis

LC-MS/MS analysis was performed using an EASY-nLC 1200 (Thermo Fisher) coupled to an Exploris 480 mass spectrometer (Thermo Fisher). Separation of peptides was performed on 20 cm frit-less silica emitters (CoAnn Technologies, 0.75 µm inner diameter), packed in-house with reversed-phase ReproSil-Pur C_{18} AQ 1.9 µm resin (Dr. Maisch). The column was constantly kept at 50 °C. Peptides were eluted in 115 minutes applying a segmented linear gradient from 0 : 100 (80 % MeCN, 0.1 % formic acid in H_2O : 0.1% formic acid in H_2O) to 98 : 2 (80 % MeCN, 0.1 % formic acid in H_2O : 0.1% formic acid in H_2O) at a flow-rate of 300 nL/min.

Mass spectra were acquired in data-dependent acquisition mode. MS^1 scans were acquired at an Orbitrap Resolution of 120 000 with a Scan Range (m/z) of 380-1500, a maximum injection time of 100 ms and a Normalized AGC Target of 300 %. For fragmentation, only precursors with charge states 2-6 were considered. Up to 20 Dependent Scans were taken. For dynamic exclusion the exclusion duration was set to 40 sec and a mass tolerance of ±10 ppm. The Isolation Window was set to 1.6 m/z with no offset. A normalized collision energy of 30 was used. MS^2 scans were taken at an Orbitrap Resolution of 15 000, with a fixed First Mass (m/z) = 120. Maximum injection time was 22 ms and the normalized AGC Target 50 %.

Deconvolution of protein mass spectra was performed with UniDec[4]. All signals in the TIC chromatogram that originate from proteins were considered for the subsequent deconvolution process. Impurities present prior to the reaction were not considered. Conversions were calculated from peak areas determined with Origin or UniDec after deconvolution by dividing the value for the product by the sum of the values for products and (residual) starting material.

SDS-PAGE

SDS-PAGE sample preparation and analysis was carried out as instructed in the manual of Bio-Rad[5]. Fluorescence measurements were carried out as instructed in the manual of Bio-Rad utilizing the "Fluorescein" analysis of the Bio-Rad Laboratories ChemiDocMP system. Coomassie staining of the gels was performed as instructed by the manufacturer. Fixation was performed for 15 min and Coomassie staining was performed for 20 h. All proteins smaller than 10 kDa were analyzed via Tris/Tricine/SDS gel electrophoresis. All other proteins were analyzed with Tris/Glycine/SDS gel electrophoresis.

Image Lab Version 6.1.0 build 7 software from Bio-Rad Laboratories Inc. was used for analysis and processing of SDS-PAGE data.

Reagents used in the SDS-PAGE workflow:

Bio-Rad Laboratories Any kD MP TGX Stain-Free 10W 30 µL

Bio-Rad Laboratories 10x Tris/Glycine/SDS buffer

Bio-Rad Laboratories 4x Laemmli sample buffer

Bio-Rad Laboratories QC Colloidal Coomassie stain

Bio-Rad Laboratories Precision Plus Protein™ Dual Xtra Prestained Protein Standards

Bio-Rad Laboratories 16.5% MP Tris-Tricine 10W 30 µL

Bio-Rad Laboratories 10x Tris/Tricine/SDS

Bio-Rad Laboratories Tricine Sample Buffer

Devices used in the SDS-PAGE workflow:

Bio-Rad Laboratories ChemiDocMP

Bio-Rad Laboratories Mini-PROTEAN Tetra Cell

Bio-Rad Laboratories PowerPac Basic

Miscellaneous

Lyophilization of purified products was performed using a BÜCHI Lyovapor™ L-200. Centrifugation was performed using a Thermo Scientific™ MegaFuge™ 8R. Pipetting of small volumes was performed with an Eppendorf Research® plus - Mechanical Pipette, 1-channel, 0,5–10 µL, an Eppendorf Eppendorf Research® plus - Mechanical Pipette, 1-channel, 10–100 µL, or an Eppendorf Eppendorf Research® plus - Mechanical Pipette, 1-channel, 100–1.000 µL. Protein reactions were conducted in an Eppendorf ThermoMixer® C equipped with an Eppendorf SmartBlock™ 1.5 mL. Protein desalting and rebuffering was performed with AMICON® filters units from Sigma Aldrich. Protein concentrations were determined by A_{280} absorption or BCA assay using a Thermo Scientific™ NanoDrop™ One^{C}. Extinction coefficients of the proteins used in this work were calculated based on the FASTA sequences with the assumption that all Cys residues are reduced. All protein structures without an entry in the RCSB PDB protein data bank were predicted by AlphaFold 2.0.

EXPERIMENTAL DATA

Synthesis of vinyl-thianthrenium salts

Preparation of VTT

VTT was synthesized according to the previously reported method[6].

Preparation of VTFT

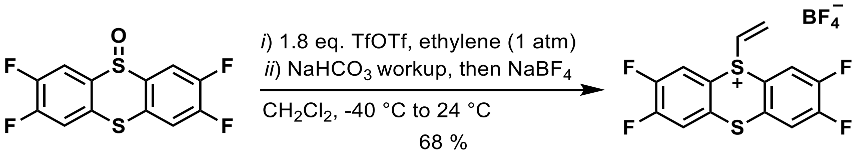

Under ambient atmosphere, a 100 mL round-bottom flask was equipped with a Teflon-coated magnetic stirring bar, tetrafluorothianthrene S-oxide (1.00 g, 3.29 mmol, 1.00 equiv.) and dichlormethane (52 mL, c = 63 mM). The flask was capped with a rubber septum and cooled to –40°C. Ethylene gas was then bubbled through the solution for 15 minutes, after which a balloon filled with ethylene was connected to the flask to maintain the ethylene atmosphere throughout the reaction. TfOTf (1.0 mL, 1.7 g, 5.9 mmol, 1.8 equiv.) was added dropwise to the reaction, and a dark purple suspension was progressively formed. After 16 hours the ethylene balloon and the rubber septum were removed, and saturated aqueous $NaHCO_3$ (50 mL) was added over 2 minutes. The mixture was vigorously shaken in a separation funnel, the organic phase was separated and the aqueous layer was extracted with dichlormethane (3 x 50 mL). All organic phases were combined, concentrated under reduced pressure to roughly 50 mL, washed with aqueous solution of 10% $NaBF_4$ (3 × 50 mL), dried over $MgSO_4$, filtered, and the solvent was evaporated under reduced pressure. Recrystallization from DCM/Et_2O afforded **VTFT** as an off-white solid, which was collected by filtration, washed with Et_2O (3 × 20 mL), and dried under vacuum to afford 1.04 g (2.24 mmol, 68%) of a colorless solid.

R_f = 0.50 (silica gel, MeOH / DCM, 1:9 (v/v)).

NMR Spectroscopy:

^{1}H NMR (600 MHz, CD_3CN, 298 K, δ): 8.22 (dd, *J* = 9.2, 7.2 Hz, 1H), 7.96 (dd, *J* = 10.0, 7.1 Hz, 1H), 6.57 (dd, *J* = 15.9, 8.9 Hz, 1H), 6.38 (dd, *J* = 9.0, 3.4 Hz, 1H), 6.02 (dd, *J* = 15.9, 3.4 Hz, 1H).

^{13}C NMR (151 MHz, CD_3CN, 298 K, δ): 154.7 (dd, *J* = 261.4, 13.1 Hz), 151.5 (dd, *J* = 255.2, 13.6 Hz), 135.2, 134.9 (dd, *J* = 8.5, 3.9 Hz), 124.8 (dd, *J* = 22.1, 2.5 Hz), 120.9 (d, *J* = 21.9 Hz), 119.7, 114.8 (dd, *J* = 7.3, 3.5 Hz).

^{19}F NMR (471 MHz, CD_3CN) δ –125.8 (m), –134.0 (m), –151.7 (s), –151.8 (s).

HRMS-ESI (m/z) calculated for $C_{14}H_7F_4S_2^+$ [M–BF_4]$^+$, 314.9920; found, 314.9920; deviation: 0.0 ppm.

Preparation of $^2H_3$-VTT from ^{2}H-labeled ethylene

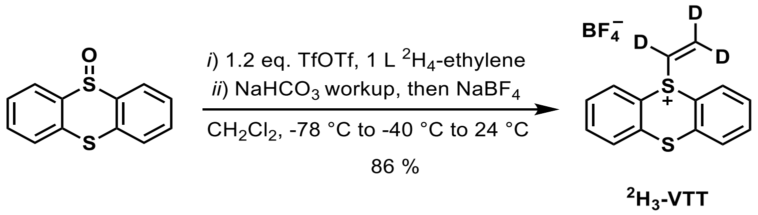

Under ambient atmosphere, a 500 mL three-necked round bottom flask equipped with a septum and a Teflon-coated magnetic stirring bar was charged with thianthrene S-oxide (9.60 g, 41.3 mmol, 1.00 equiv.) and connected to a vacuum pump. Next, the setup was connected to an ethylene bottle (1.0 L, 42 mmol), that was separated from the remaining setup by a cooling trap. The whole setup was left under vacuum for 16 hours. The cooling trap was cooled with liquid nitrogen. The three-necked round bottom flask was cooled to –78 °C via a dry ice acetone bath. The whole apparatus was evacuated and the cooling trap was separated from the remaining apparatus. The ethylene bottle was opened to the cooling trap for 5 min. This procedure was repeated one additional time. Next, the whole apparatus was opened to the ethylene bottle. After 5 minutes the ethylene bottle was separated from the system and dichloromethane (400 mL, c = 100 mM) was added to the three-necked round bottom flask via syringe. Then, a balloon was connected to the setup via the septum and the cooling trap was allowed to come to room temperature by removing the liquid nitrogen. After 10 minutes and equalization of the vacuum with nitrogen, the reaction mixture was warmed to –40 °C by exchanging the dry ice acetone bath by a dry ice acetonitrile bath. Next, TfOTf (8.3 mL, 50 mmol, 1.2 equiv.) was slowly added to the reaction mixture under stirring. The mixture was stirred for 90 minutes at – 40 °C. Next, the dry ice bath was removed, and the reaction mixture was stirred for another 90 minutes under ambient atmosphere. Afterwards, the reaction mixture was cooled to –40 °C via a dry ice acetonitrile bath, the cooling trap was purged with argon, and the gas was introduced into the reaction mixture via a needle. After 10 minutes, saturated sodium bicarbonate solution (100 mL) was added to the flask, and the mixture was stirred for another 10 min. Then, the mixture was transferred into a separation funnel, saturated sodium bicarbonate solution (300 mL) was added, and the phases were separated. The aqueous layer was extracted three times with dichloromethane (3 x 500 mL). The united organic layers were concentrated under reduced pressure (~ 200 mL), washed three times with 10 % (w/v) $NaBF_4$ solution (3 x 200 mL), and dried over $MgSO_4$. Next, the organic layer was filtered and concentrated under reduced pressure. Then, the concentrate was dissolved in dichloromethane (1.3 mL per 1 g crude). The solution was cooled with a water ice bath, and Et_2O (200 mL) was added in portions to cause the precipitation of a solid, which was collected via filtration. The obtained material was washed with Et_2O (50 mL) and dried under vacuum to afford **$^2H_3$-VTT** as an off-white solid (11.8 g, 35.4 mmol, 86 %).

R*f* = 0.15 (silica gel, EtOAc / hexanes, 1:4 (v/v)).

NMR Spectroscopy:

^{1}H NMR (500 MHz, CD_3CN, 298 K, δ): 8.13 (dd, *J* = 7.9, 1.4 Hz, 2H), 7.89 (dd, *J* = 8.0, 1.4 Hz, 2H), 7.78 (td, *J* = 7.7, 1.4 Hz, 2H), 7.67 (td, *J* = 7.7, 1.3 Hz, 2H).

^{13}C NMR (126 MHz, CD_3CN, 298 K, δ): 137.0, 135.8, 135.0, 133.6 (m), 131.3, 130.8, 120.3 (m), 119.0.

^{19}F NMR (471 MHz, CD_3CN) –151.16 (s), –151.20 (s).

HRMS-ESI (m/z) calculated for $C_{14}H_8{}^2H_3S_2{}^+$ $[M–BF_4]^+$, 246.0485; found, 246.0484; deviation: –0.4 ppm.

Preparation of $^{13}C_2$-VTT from ^{13}C-labeled ethylene

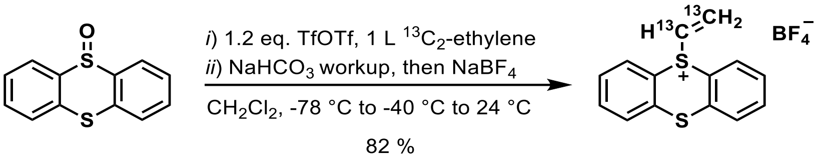

$^{13}C_2$-VTT

Under ambient atmosphere, a 500 mL three-necked round bottom flask equipped with a septum and a Teflon-coated magnetic stirring bar was charged with thianthrene S-oxide (9.60 g, 41.3 mmol, 1.00 equiv.) and connected to a vacuum pump. Next, the setup was connected to an ethylene bottle (1.0 L, 42 mmol), that was separated from the remaining setup by a cooling trap. The whole setup was left under vacuum for 16 hours. The cooling trap was cooled with liquid nitrogen. The three-necked round bottom flask was cooled to –78 °C via a dry ice acetone bath. The whole apparatus was evacuated and the cooling trap was separated from the remaining apparatus. The ethylene bottle was opened to the cooling trap for 5 min. This procedure was repeated one additional time. Next, the whole apparatus was opened to the ethylene bottle. After 5 minutes the ethylene bottle was separated from the system and dichloromethane (400 mL, 100 mM) was added to the three-necked round bottom flask via syringe. Then, a balloon was connected to the setup via the septum and the cooling trap was allowed to come to room temperature by removing the liquid nitrogen. After 10 minutes and equalization of the vacuum with nitrogen, the reaction mixture was warmed to –40 °C by exchanging the dry ice acetone bath by a dry ice acetonitrile bath. Next, TfOTf (8.3 mL, 50 mmol, 1.2 equiv.) was slowly added to the reaction mixture under stirring. The mixture was stirred for 90 minutes at –40 °C. Next, the dry ice bath was removed and the reaction mixture was stirred for another 90 minutes under ambient atmosphere. Afterwards, the reaction mixture was cooled to –40 °C via a dry ice acetonitrile bath, the cooling trap was purged with argon, and the gas was introduced into the reaction mixture via a needle. After 10 min, saturated sodium bicarbonate solution (100 mL) was added to the flask and the mixture was stirred for another 10 min. Then, the mixture was transferred into a separation funnel, saturated sodium bicarbonate solution (300 mL) was added, and the phases were separated. The aqueous layer was extracted three times with dichloromethane (3 x 500 mL). The united organic layers were concentrated under reduced pressure (~ 200 mL), washed three times with 10 % (w/v) $NaBF_4$ solution (3 x 200 mL), and dried over $MgSO_4$. Next, the organic layer was filtered and concentrated under reduced pressure. Then, the concentrate was dissolved in dichloromethane (1.3 mL per 1 g crude), cooled with a water ice bath and Et_2O (200 mL) was added in portions to cause the precipitation of a solid, which was collected via filtration. The obtained material was washed with Et_2O (50 mL) and dried under vacuum to afford **$^{13}C_2$-VTT** as an off-white solid (11.3 g, 34.0 mmol, 82 %).

R_f = 0.15 (silica gel, EtOAc / hexanes, 1:4 (v/v)).

NMR Spectroscopy:

^{1}H NMR (600 MHz, CD_3CN, 298 K, δ): 8.27 (ddt, *J* = 8.0, 1.4, 0.4 Hz, 2H), 7.95 (ddd, *J* = 8.0, 1.3, 0.4 Hz, 2H), 7.83 (dddd, *J* = 7.9, 7.4, 1.4, 0.4 Hz, 2H), 7.73 (ddd, *J* = 8.0, 7.5, 1.3 Hz, 2H), 6.72 (dddd, *J* = 198.0, 15.9, 8.9, 1.8 Hz, 1H), 6.28 (dddd, *J* = 168.1, 8.9, 5.4, 3.0 Hz, 1H), 5.94 (dddd, *J* = 164.8, 16.0, 6.6, 3.0 Hz, 1H).

^{13}C NMR (151 MHz, CD_3CN, 298 K, δ): 137.1, 135.8, 135.4, 133.8 (d, *J* = 72.5 Hz), 131.3, 130.8, 121.3 (d, *J* = 72.5 Hz), 119.3.

^{19}F NMR (470 MHz, CD_2Cl_2, 298 K, δ): –151.8 (s), –151.9 (s).

HRMS-ESI (m/z) calculated for $C_{12}H_{11}{}^{13}C_2S_2^+$ $[M–BF_4]^+$, 245.0364; found, 245.0362; deviation: –0.8 ppm.

Preparation of $^2H_3$-VTFT from ^{2}H-labeled ethylene

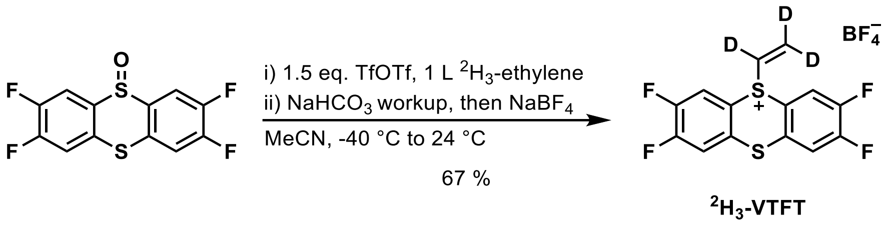

A 1000 mL three-necked round bottom flask equipped with a septum and a Teflon-coated magnetic stirring bar was charged with tetrafluorothianthrene S-oxide (12.6 g, 41.4 mmol) and connected to a vacuum pump. Next, the setup was connected to an ethylene bottle (1.0 L, 42 mmol), that was separated from the remaining setup by a cooling trap. The whole setup was left under vacuum for 16 hours. The cooling trap was cooled with liquid nitrogen. The three-necked round bottom flask was cooled to –40 °C via a dry ice acetonitrile bath. The whole apparatus was evacuated and the cooling trap was separated from the remaining apparatus. The ethylene bottle was opened to the cooling trap for 15 min. This procedure was repeated one additional time. Next, the whole apparatus was opened to the ethylene bottle. After 10 minutes the ethylene bottle was separated from the system and acetonitrile (660 mL, 0.06 M) was added to the three-necked round bottom flask via syringe. Then, a balloon was connected to the setup via the septum and the cooling trap was allowed to come to room temperature by removing the liquid nitrogen. After 10 minutes the vacuum was equalized with nitrogen. Next, TfOTf (10.45 mL, 62.11 mmol) was slowly added to the reaction mixture under stirring. The mixture was stirred for 180 minutes without addition of more dry ice. Afterwards, solid sodium bicarbonate (174 g, 2.07 mol) and 13 mL water (2%) were added to the flask and the mixture was stirred until the product formed completely. This process was monitored via ^{1}H NMR measurements. Then, the mixture was filtered and the flowthrough was concentrated under reduced pressure. Subsequently, dichloromethane (300 mL) and acetonitrile (200 mL) were added and the mixture was transferred into a separation funnel and washed three times with 10 % (w/v) $NaBF_4$ solution (3 x 400 mL). The aqueous layer was extracted once with 500 mL dichloromethane. The united organic layers were dried over $MgSO_4$, filtered, and concentrated under reduced pressure. Then, the concentrate was washed with Et_2O (150 mL) and dried under an argon stream to obtain **$^2H_3$-VTFT** as an off-white solid (11.3 g, 27.89 mmol, 67 %).

R_f = 0.50 (silica gel, MeOH / DCM, 1:9 (v/v)).

NMR Spectroscopy:

^{1}H NMR (500 MHz, CD_3CN, 298 K, δ): 8.26 (dd, *J* = 9.1, 7.3 Hz, 2H), 7.99 (dd, *J* = 10.0, 7.1 Hz, 2H).

^{2}H NMR (92 MHz, CD_3CN, 298 K, δ): 6.55 (s, 1H), 6.37 (s, 1H), 6.01 (s, 1H).

^{13}C NMR (126 MHz, CD_3CN, 298 K, δ): 154.5 (dd, *J* = 261.3, 13.2 Hz), 151.4 (dd, *J* = 255.1, 13.7 Hz), 134.8 (dd, *J* = 8.5, 4.0 Hz), 124.7 (d, *J* = 22.2 Hz), 120.8 (d, *J* = 21.8 Hz), 114.7 (m).

^{19}F NMR (471 MHz, CD_3CN, δ): –126.0 (m), –134.1 (m), –151.0 (s), –151.1 (s).

HRMS-ESI (m/z) calculated for $C_{14}H_4{}^2H_3F_4S_2^+$ $[M–BF_4]^+$, 318.0108; found, 318.0108; deviation: +0.0 ppm.

Preparation of $^{13}C_2$-VTFT from ^{13}C-labeled ethylene

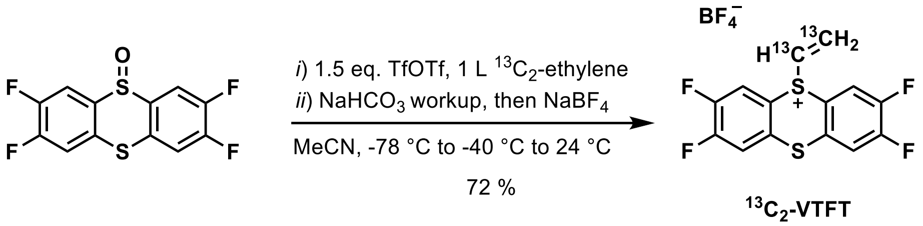

Under ambient atmosphere, a 1000 mL three-necked round bottom flask equipped with a septum and a magnetic stirring bar was charged with tetrafluorothianthrene S-oxide (12.6 g, 41.4 mmol, 1.00 equiv.) and connected to a vacuum pump. Next, the setup was connected to an ethylene bottle (1.0 L, 42 mmol), that was separated from the remaining setup by a cooling trap. The whole setup was left under vacuum for 16 hours. The cooling trap was cooled with liquid nitrogen. The three-necked round bottom flask was cooled to –40 °C via a dry ice acetonitrile bath. The whole apparatus was evacuated and the cooling trap was separated from the remaining apparatus. The ethylene bottle was opened to the cooling trap for 15 min. This procedure was repeated one additional time. Next, the whole apparatus was opened to the ethylene bottle. After 10 minutes the ethylene bottle was separated from the system and acetonitrile (660 mL, 600 mM) was added to the three-necked round bottom flask via syringe. Then, a balloon was connected to the setup via the septum and the cooling trap was allowed to come to room temperature by removing the liquid nitrogen. After 10 minutes the vacuum was equalized with nitrogen. Next, TfOTf (10.5 mL, 62.1 mmol, 1.50 equiv.) was slowly added to the reaction mixture under stirring. The mixture was stirred for 180 minutes without addition of more dry ice. Afterwards, solid sodium bicarbonate (174 g, 2.07 mol) and 13 mL water were added to the flask and the mixture was stirred until the product formed completely. This process was monitored via ^1H NMR measurements. Then, the mixture was filtered and the flowthrough was concentrated under reduced pressure. Subsequently, dichloromethane (300 mL) and acetonitrile (200 mL) were added and the mixture was transferred into a separation funnel and washed three times with 10 % (w/v) $NaBF_4$ solution (3 x 400 mL). The aqueous layer was extracted once with 500 mL dichloromethane. The united organic layers were dried over $MgSO_4$, filtered, and concentrated under reduced pressure. Then, the concentrate was washed with Et_2O (150 mL) and dried under an argon stream to obtain **$^{13}C_2$-VTFT** as an off-white solid (11.9 g, 29.6 mmol, 72 %).

R_f = 0.50 (silica gel, MeOH / DCM, 1:9 (v/v)).

NMR Spectroscopy:

^1H NMR (500 MHz, CD_3CN, 298 K, δ): 8.20 (dd, J = 9.2, 7.2 Hz, 2H), 7.92 (dd, J = 10.0, 7.1 Hz, 2H), 6.54 (dddd, J = 199.5, 15.9, 9.0, 1.6 Hz, 1H), 6.54 – 6.16 (m, 1H), 6.18 – 5.79 (m, 1H).

^{13}C NMR (126 MHz, CD_3CN, 298 K, δ): 154.5 (dd, J = 261.3, 13.2 Hz), 151.4 (dd, J = 255.1, 13.6 Hz), 135.1 (d, J = 72.6 Hz), 124.7 (d, J = 22.1 Hz), 120.8 (d, J = 21.8 Hz), 119.6 (d, J = 72.6 Hz), 114.8 – 114.7 (m).

^{19}F NMR (471 MHz, CD_3CN, 298 K, δ): –126.0 (m), –134.2 (m), –150.9 (s), –151.0 (s).

HRMS-ESI (m/z) calculated for $C_{12}{}^{13}C_2H_7F_4S_2^+$ $[M–BF_4]^+$, 316.9987; found, 316.9987; deviation: 0.0 ppm.

Stability of vinylthianthrenium salts in DMF solutions

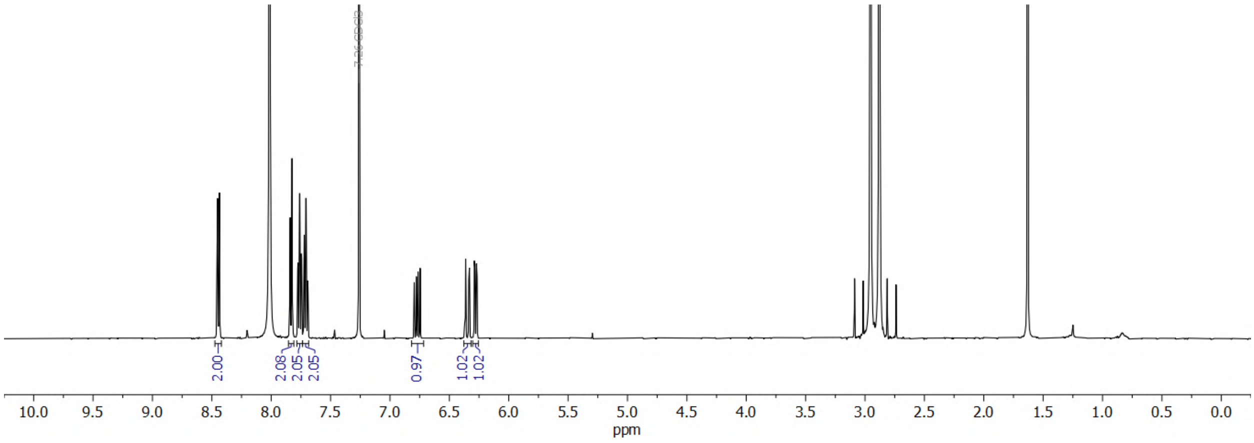

Figure S1. ^{1}H NMR spectrum of a sample of **VTT** stock solution in DMF (c = 0.8 M) stored for 8 months at –18 °C under air; $CDCl_3$, 500 MHz, 298 K.

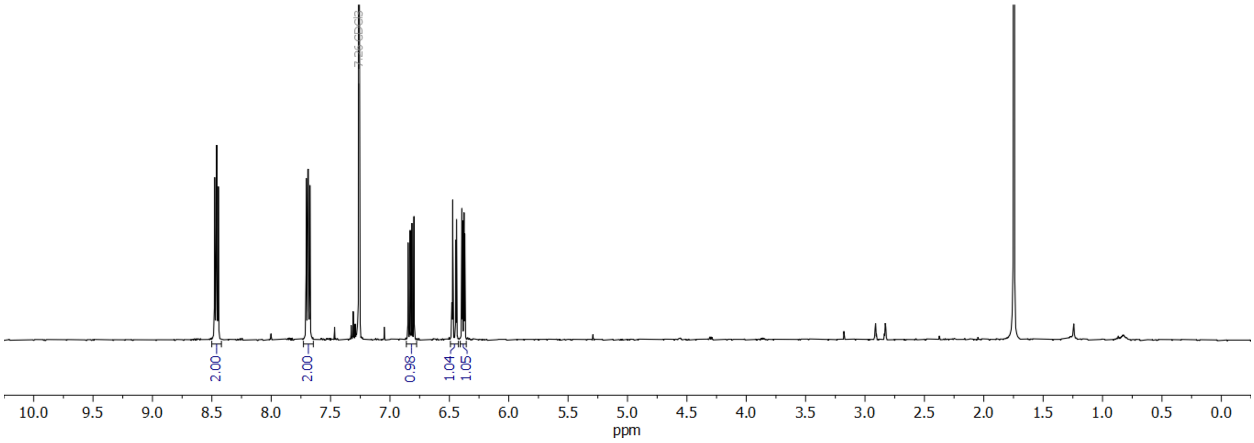

Figure S2. ^{1}H NMR spectrum of a sample of **VTFT** stock solution in DMF-d_7 (c = 0.5 M) stored for 6 months at –18 °C under air; $CDCl_3$, 500 MHz, 298 K.

Solubility of vinylthianthrenium salts in water

To compare the solubility of **VTT-OTf** and **VTT-BF$_4$** in water, 20 µmol of each salt was mixed with 450 µL of D_2O in an Eppendorf tube and dimethylsulfone in D_2O (50 µL, c = 50 mM) was added to each tube. The mixtures were transferred into a Thermocycler pre-heated at 25 °C, and incubated at 25 °C at 900 rpm for 120 minutes. Then, NMR spectra of each mixture were measured. Based on the integrated values:

- c(**VTT-BF$_4$**) = 38 mM
- c(**VTT-OTf**) = 34 mM

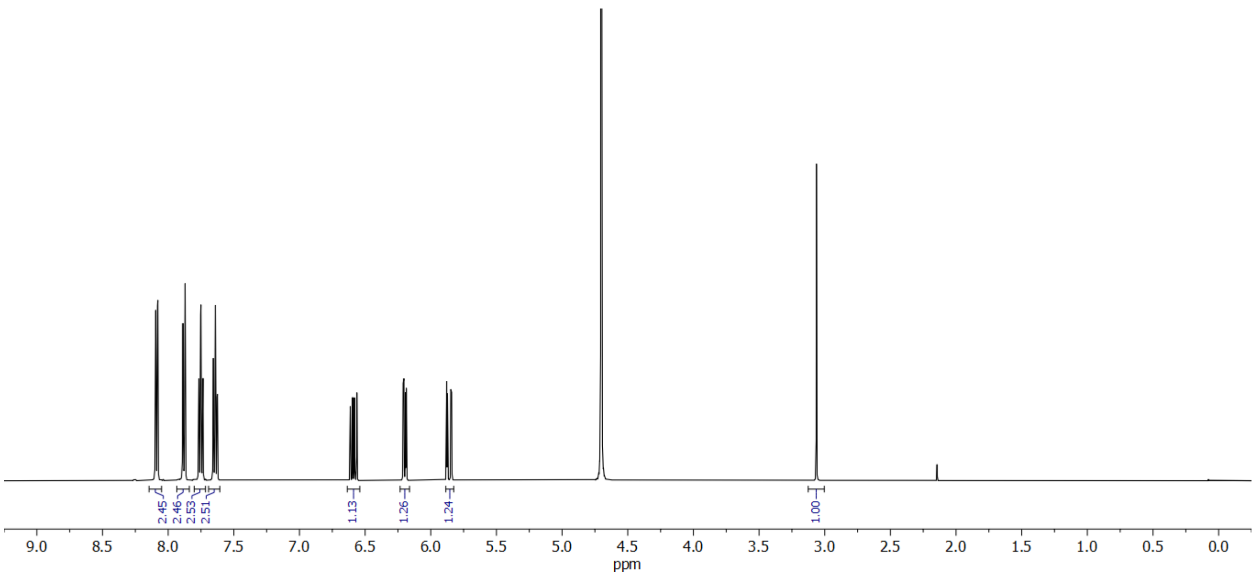

Figure S3. ^{1}H NMR spectrum of **VTT-BF$_4$** in D_2O with dimethylsulfone (5 mM) as internal standard.

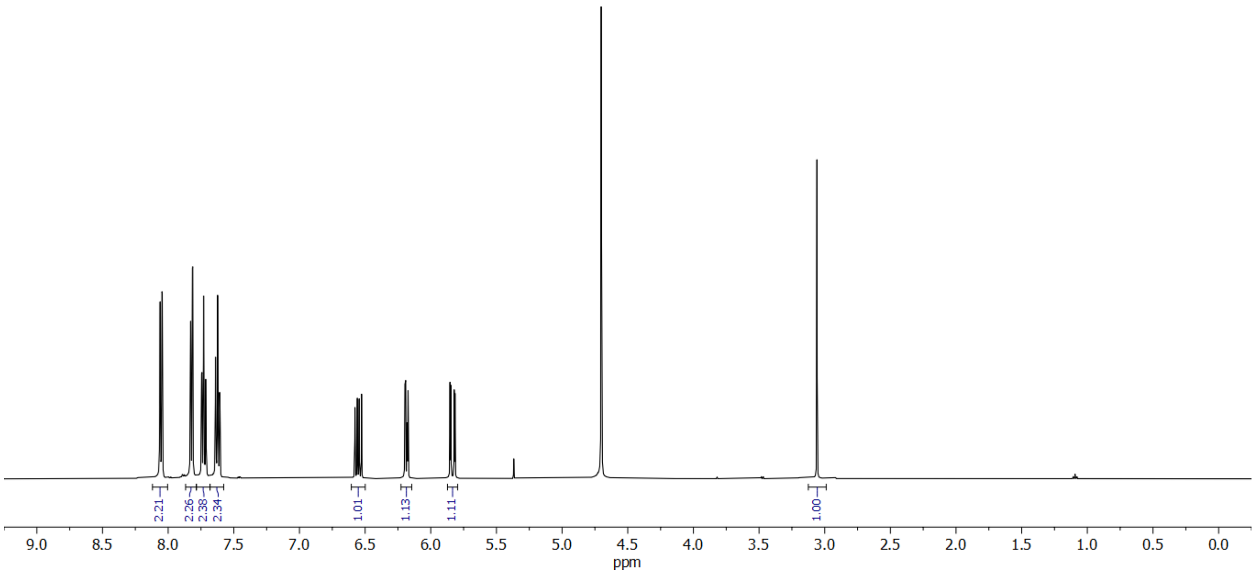

Figure S4. ^{1}H NMR spectrum of **VTT-OTf** in D_2O with dimethylsulfone (5 mM) as internal standard.

Stability of tetrafluoroborate anion in a model reaction mixture

In order to exclude potential side reactivity originating from the decomposition of the tetrafluoroborate anion in solution, we conducted the reaction in presence of an internal ^{19}F standard. The analysis indicates no detectable decomposition of the tetrafluoroborate within the given reaction conditions.

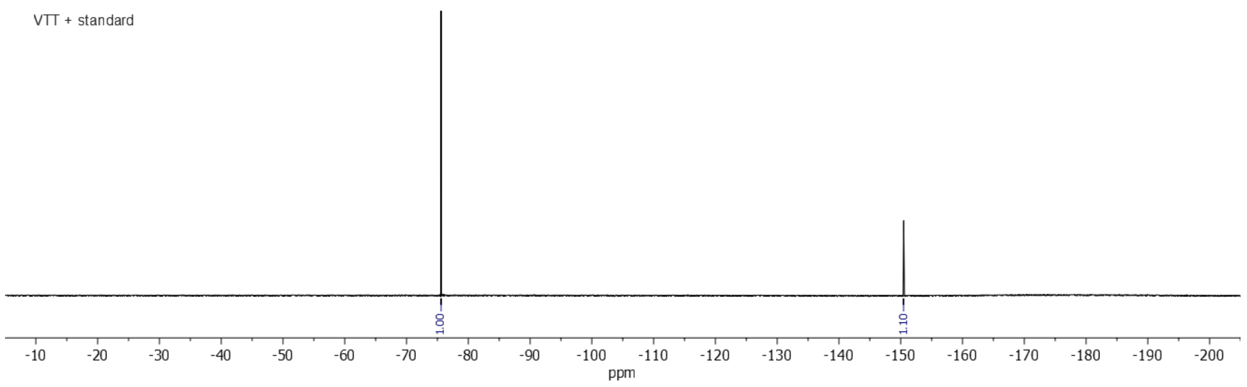

Figure S5. ^{19}F NMR spectrum of **VTT** (10 mM) and CF_3COOH (10 mM) as internal standard in D_2O.

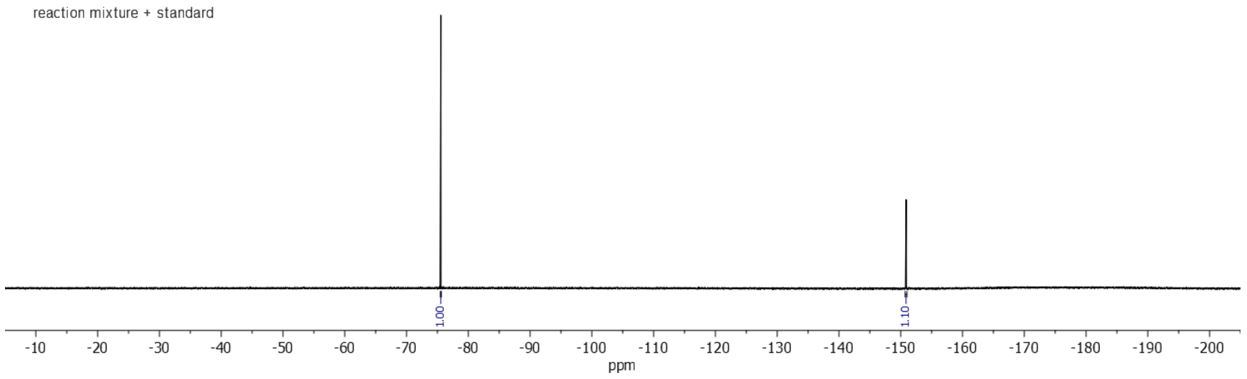

Figure S6. ^{19}F NMR spectrum measured 30 minutes after addition of **VTT** (10 mM) to the mixture containing glutathione (10 mM), NaN_3 (0.3 M), and CF_3COOH (10 mM) as internal standard in deuterated NaP_i buffer (pH 7.0, 50 mM).

Synthesis of Glutathione derivatives with different nucleophiles

Preparation of Glutathione–S–C_2H_4–N_3 trifluoroacetate (1) from VTT

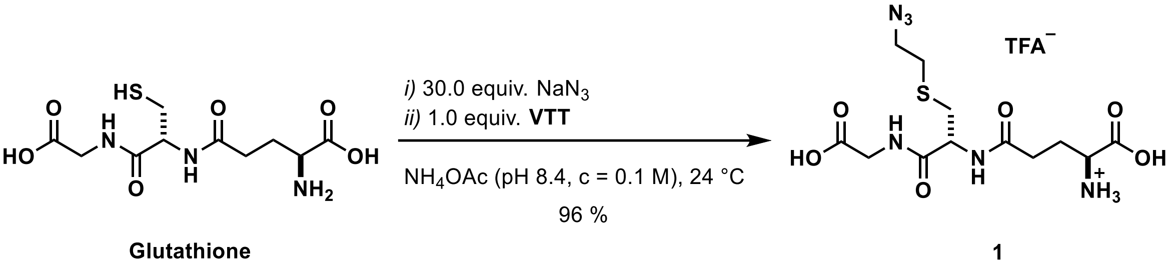

At 20–25 °C, a round-bottom flask (100 mL) was charged with glutathione (40.0 mg, 130 µmol, 1.00 equiv.) and equipped with a Teflon-coated magnetic stirring bar. Then, a 600 mM solution of sodium azide in UHQ-H_2O (6.51 mL, 3.90 mmol, 30.0 equiv.), 2.6 mL of aqueous NH_4OAc-buffer (pH 8.4, c = 0.5 M), and 3.9 mL of UHQ-H_2O were added to the flask. Subsequently, a 1.0 M solution of **VTT** in DMF (0.13 mL, 0.13 mmol, 1.0 equiv.) was added to the reaction mixture while stirring at 400 rpm. The reaction mixture was stirred at 400 rpm for 30 minutes at 24 °C. Then, the reaction mixture was filtered, the filtrate was collected, and the filter cake was washed with UHQ-H_2O (3 x 10 mL). The combined filtrate was lyophilized and the obtained residue was purified by HPLC on an YMC Triart C18 column ((150×30 mm, 5 µm), flow rate = 42.5 mL/min, 35 °C) with a linear gradient from 5:95 v/v (MeCN : 0.1% v/v TFA in H_2O) to 30:70 v/v (MeCN : 0.1% v/v TFA in H_2O) over 20 minutes. The fractions containing the product (t ≈ 8.1 min) were collected and lyophilized to afford the title compound as a colorless solid (61.4 mg, 125 µmol, 96%).

NMR Spectroscopy:

^{1}H NMR (600 MHz, D_2O, 298 K, δ): 8.57 (d, *J* = 6.1 Hz, 1H), 8.55 (d, *J* = 7.6 Hz, 1H), 8.03 (s, 3H), 4.63 (dd, *J* = 8.7, 5.1 Hz, 1H), 4.08 – 3.97 (m, 2H), 3.90 (t, *J* = 6.4 Hz, 1H), 3.58 (t, *J* = 6.4 Hz, 2H), 3.14 (dd, *J* = 14.1, 5.2 Hz, 1H), 2.96 (dd, *J* = 14.1, 8.7 Hz, 1H), 2.89 – 2.80 (m, 2H), 2.65 – 2.52 (m, 2H), 2.27 – 2.15 (m, 2H).

^{13}C NMR (151 MHz, D_2O, 298 K, δ): 174.8, 173.4, 173.3, 172.8, 163.1 (q, J = 35.5 Hz), 116.4 (q, J = 291.6 Hz), 53.6, 53.3, 50.5, 41.5, 33.0, 31.2, 31.2, 26.0.

^{19}F NMR (470 MHz, D_2O, 298 K, δ): –75.5.

HRMS-ESI (m/z) calculated for $C_{12}H_{21}N_6O_6S^+$ [M–TFA]$^+$, 377.1238; found, 377.1234; deviation: –1.1 ppm.

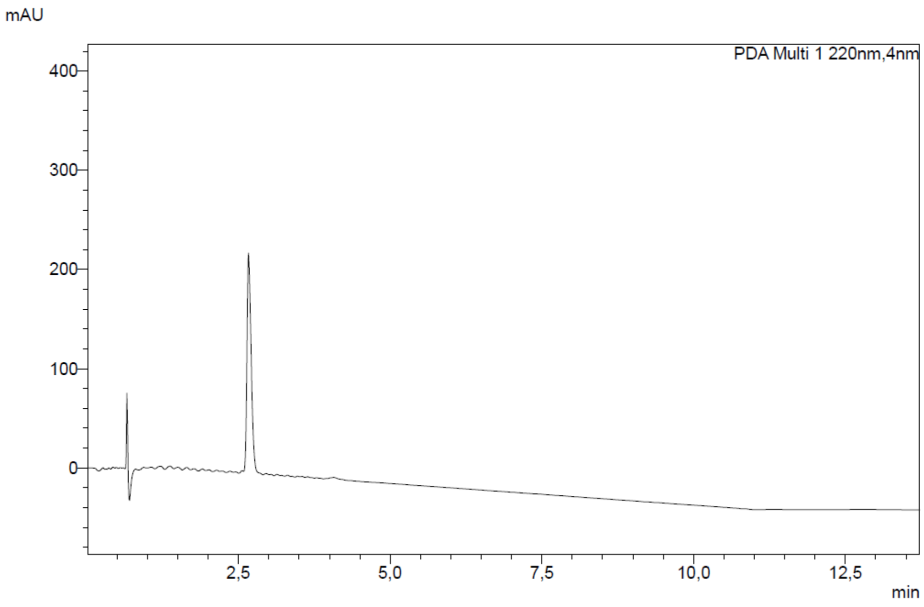

Figure S7. Analytical HPLC trace of **1** (Eclipse Plus C18 column, 50 x 4.6 mm, 1.8 µm, flow rate = 1.0 mL · min^{-1}) with a linear gradient from 5:95 v/v (MeCN : 0.1% v/v TFA in H_2O) to 30:70 v/v (MeCN : 0.1% v/v TFA in H_2O) over 10 min.

Preparation of Glutathione–S–C_2H_4–N_3 trifluoroacetate (1) from VTFT

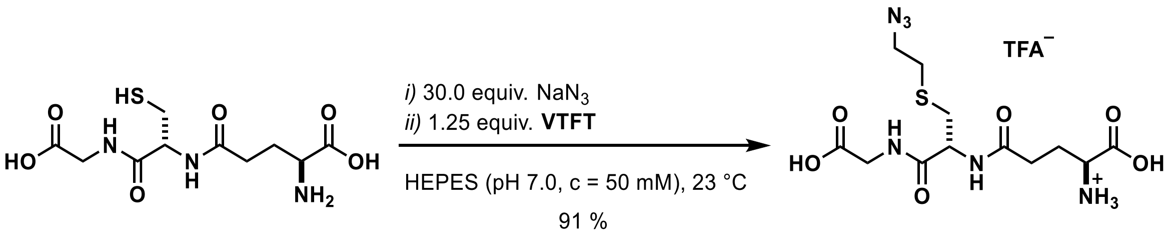

At 20–25 °C, a round-bottom flask (25 mL) was charged with glutathione (20.0 mg, 65.1 µmol, 1.00 equiv.) and equipped with a Teflon-coated magnetic stirring bar. Then, a 600 mM solution of sodium azide in UHQ-H_2O (3.25 mL, 1.95 mmol, 30.0 equiv.), 0.65 mL of aqueous HEPES-buffer (pH 7.0, c = 0.5 M), and 2.6 mL of UHQ-H_2O were added to the flask. Subsequently, a 1.0 M solution of **VTFT** in DMF (81 µL, 81 µmol, 1.2 equiv.) was added to the reaction mixture while stirring at 400 rpm. The reaction mixture was stirred at 400 rpm for 15 minutes at 23 °C. Then, the reaction mixture was filtered, the filtrate was collected, and the filter cake was washed with UHQ-H_2O (3 x 5 mL). The combined filtrate was lyophilized and the obtained residue was purified by HPLC on an YMC Triart C18 column ((150×30 mm, 5 µm) with a YMC Triart C18 precolumn (50×30 mm, 5 µm), flow rate = 42.5 mL/min, 35 °C) with a linear gradient from 5:95 v/v (MeCN : 0.1% v/v TFA in H_2O) to 20:80 v/v (MeCN : 0.1% v/v TFA in H_2O) over 20 minutes, followed by linear gradient from 20:80 v/v (MeCN : 0.1% v/v TFA in H_2O) to 50:50 v/v (MeCN : 0.1% v/v TFA in H_2O) over 10 minutes. The fractions containing the product (t ≈ 11 min) were collected and lyophilized to afford the title compound as a colorless solid (29.0 mg, 59.2 µmol, 91%).

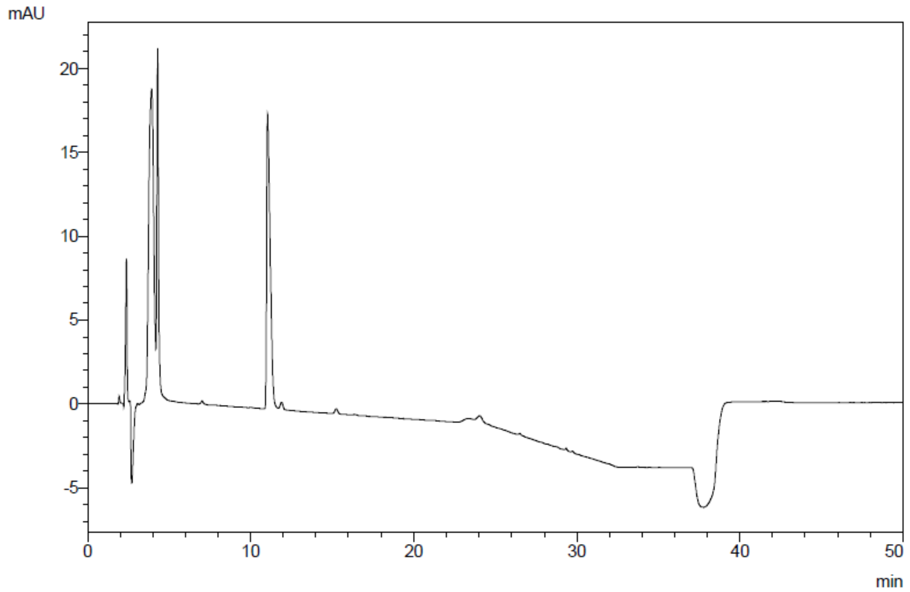

Figure S8. HPLC purification chromatogram of **1** (YMC Triart C18 column ((150×30 mm, 5 µm) with a YMC Triart C18 precolumn (50×30 mm, 5 µm), flow rate = 42.5 mL/min, 35 °C) with a linear gradient from 5:95 v/v (MeCN : 0.1% v/v TFA in H_2O) to 20:80 v/v (MeCN : 0.1% v/v TFA in H_2O) over 20 minutes, followed by linear gradient from 20:80 v/v (MeCN : 0.1% v/v TFA in H_2O) to 50:50 v/v (MeCN : 0.1% v/v TFA in H_2O) over 10 minutes). The peak at t ≈ 11 min was collected.

Preparation of Glutathione–S–C_2H_3D–N_3 trifluoroacetate ($^2H_1$-1) from VTFT

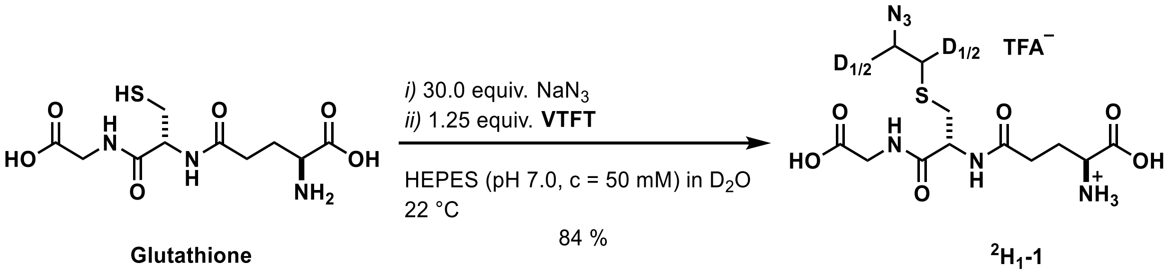

At 20–25 °C, a round-bottom flask (25 mL) was charged with glutathione (20.0 mg, 65.1 µmol, 1.00 equiv.) and equipped with a Teflon-coated magnetic stirring bar. Then, a 600 mM solution of sodium azide in D_2O (3.25 mL, 1.95 mmol, 30.0 equiv.), 0.65 mL of HEPES-buffer (pH 7.0, c = 0.5 M) in D_2O, and 2.6 mL of D_2O were added to the flask. Subsequently, a 1.0 M solution of **VTFT** in DMF (81 µL, 81 µmol, 1.2 equiv.) was added to the reaction mixture while stirring at 400 rpm. The reaction mixture was stirred at 400 rpm for 15 minutes at 23 °C. Then, the reaction mixture was filtered, the filtrate was collected, and the filter cake was washed with UHQ-H_2O (3 x 5 mL). The combined filtrate was lyophilized and the obtained residue was purified by HPLC on an YMC Triart C18 column ((150×30 mm, 5 µm) with a YMC Triart C18 precolumn (50×30 mm, 5 µm), flow rate = 42.5 mL/min, 35 °C) with a linear gradient from 5:95 v/v (MeCN : 0.1% v/v TFA in H_2O) to 15.5:84.5 v/v (MeCN : 0.1% v/v TFA in H_2O) over 14 minutes, followed by linear gradient from 15.5:84.5 v/v (MeCN : 0.1% v/v TFA in H_2O) to 50:50 v/v (MeCN : 0.1% v/v TFA in H_2O) over 2 minutes. The fractions containing the product (t ≈ 11 min) were collected and lyophilized to afford the title compound as a colorless solid (26.9 mg, 54.7 µmol, 84%).

NMR Spectroscopy:

^{1}**H NMR** (600 MHz, D_2O, 298 K, δ): 8.56 (t, *J* = 5.9 Hz, 1H), 8.52 (d, *J* = 7.5 Hz, 1H), 8.08 (s, 3H), 4.62 (dd, *J* = 8.6, 5.2 Hz, 1H), 4.06 – 3.97 (m, 2H), 3.91 (t, *J* = 6.4 Hz, 1H), 3.56 (d, *J* = 6.4 Hz, 1H), 3.55 (t, *J*

= 6.4 Hz, 1H), 3.13 (dd, J = 14.1, 5.2 Hz, 1H), 2.94 (dd, J = 14.1, 8.7 Hz, 1H), 2.85 (dd, J = 14.0, 6.4 Hz, 1H) 2.81 (dd, J = 14.0, 6.4 Hz, 1H), 2.63 – 2.52 (m, 2H), 2.23 – 2.18 (m, 2H).

^{13}C NMR (151 MHz, D_2O, 298 K, δ): 174.8, 173.2, 173.1, 172.8, 53.5, 53.2, 50.4, 50.2 (t, J = 22.2 Hz), 41.4, 32.9, 32.9, 31.2, 31.2, 31.1, 30.9 (t, J = 21.3 Hz), 25.9.

HRMS-ESI (m/z) calculated for $C_{12}H_{19}N_6O_6S_1D_1$ [M–TFA]$^+$, 378.1301; found, 378.1302; deviation: +0.3 ppm.

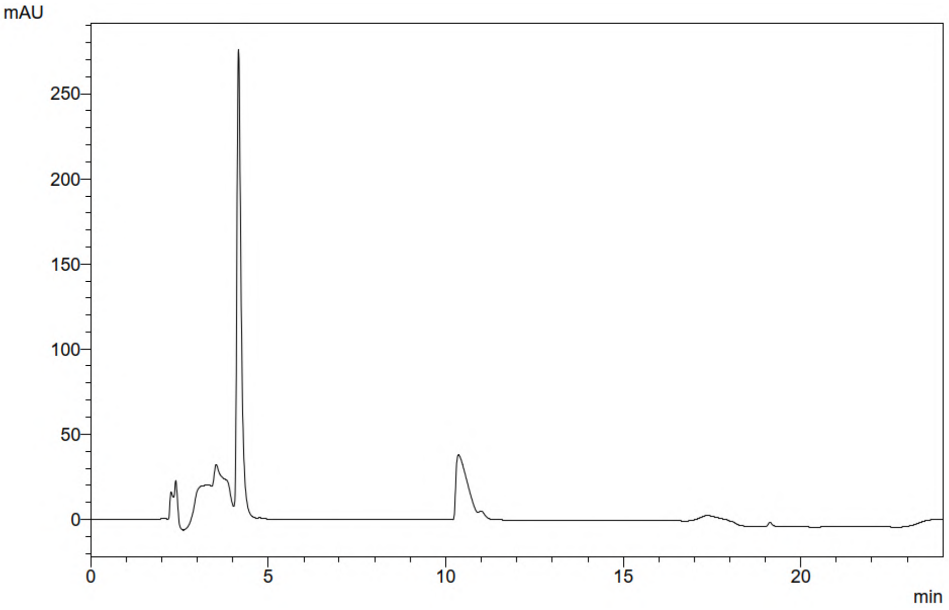

Figure S9. HPLC purification chromatogram of **$^2H_1$-1** (YMC Triart C18 column ((150×30 mm, 5 µm) with a YMC Triart C18 precolumn (50×30 mm, 5 µm), flow rate = 42.5 mL/min, 35 °C) with a linear gradient from 5:95 v/v (MeCN : 0.1% v/v TFA in H_2O) to 15.5:84.5 v/v (MeCN : 0.1% v/v TFA in H_2O) over 14 minutes, followed by linear gradient from 15.5:84.5 v/v (MeCN : 0.1% v/v TFA in H_2O) to 50:50 v/v (MeCN : 0.1% v/v TFA in H_2O) over 2 minutes.

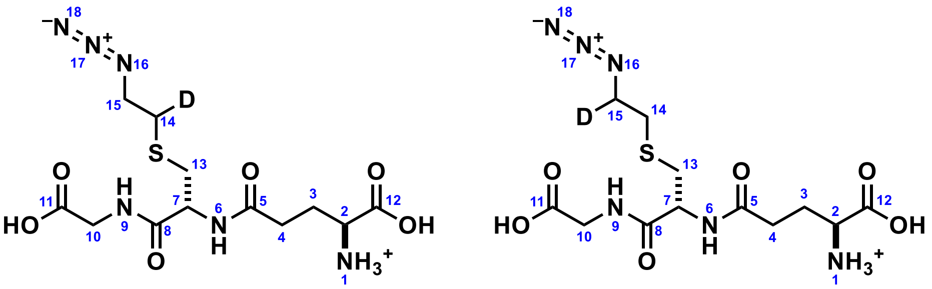

1:1

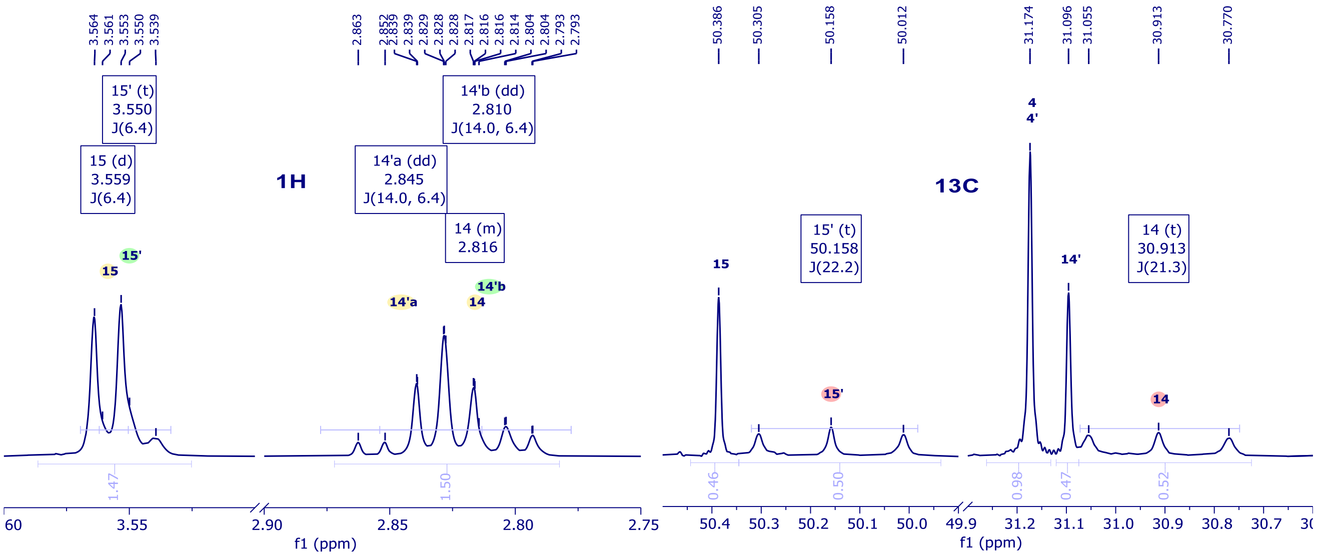

Figure S10. Annotation of the two constitutional isomers of ^{2}**H$_1$-1** formed within the reaction and zoom on the signals that originate from the ethylene linkage between GSH and the azide group.

Product formation in presence of D_2O leads to incorporation of deuterium, either in position 14 or 15. A ratio of 1:1 was determined from a ^{1}H and a quantitative ^{13}C experiment. The integrals of the respective ^{1}H signals of atom positions 14 and 15 are 1.5 instead of 2.0, which is consistent with a CH_2 group and a CHD group in 50 % of the cases each. The quantitative ^{13}C spectrum shows ^{2}H-coupled signals next to the equally intense corresponding singlets of C14 and C15. The 1H signals of the two regioisotopomers partially overlap for H15 and do fully overlap for H14. In the latter case, the splitting pattern of the CHD signal could not be reliably resolved due to the overlap, but there are probably two triplets of 6.4 Hz at 2.804 ppm and 2.828 ppm, which belong to the two diastereoisotopomers formed by monodeuteration at C14.

The experiment was designed to further support the formation of the cyclic episulfonium formation upon nucleophilic attack of cysteinyl thiols on vinyl thianthrenium derivatives. Hence, only measurements relevant for the indication of symmetrical incorporation of deuterium are depicted here and in the NMR data. All other experiments displayed similar results as the spectra for the isotopologues **1** and are therefore not shown here.

Preparation of Glutathione–S–C_2H_4–SCN trifluoroacetate (S1)

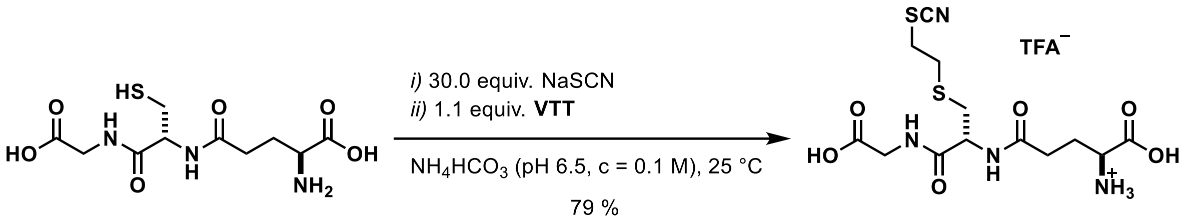

Glutathione **S1**

At 20–25 °C, a round-bottom flask (100 mL) was charged with glutathione (20.0 mg, 65.1 µmol, 1.00 equiv.) and equipped with a Teflon-coated magnetic stirring bar. Then, a 600 mM solution of sodium thiocyanate in UHQ-H_2O (3.25 mL, 1.95 mmol, 30.0 equiv.) and 3.25 mL of aqueous NH_4HCO_3-buffer (pH 6.5, c = 200 mM) were added to the flask. Subsequently, a 1.0 M solution of **VTT** in DMF (72 µL, 72 µmol, 1.1 equiv.) was added to the reaction mixture while stirring at 400 rpm. The reaction mixture was stirred at 400 rpm for 30 minutes at 25 °C. Then, the reaction mixture was filtered, the filtrate was collected, and the filter cake was washed with UHQ-H_2O (3 x 10 mL). The combined filtrate was lyophilized and the obtained residue was purified by HPLC on a YMC

Triart C18 column ((150×30 mm, 5µm) with a YMC Triart C18 precolumn (50×30 mm, 5 µm), flow rate = 42.5 mL/min, 35 °C) with an isocratic eluent 12.5:87.5 v/v (MeCN : 0.1% v/v TFA in H_2O) over 12 minutes. The fractions containing the product (t ≈ 6.4 min) were collected and lyophilized to afford the title compound as a colorless solid (26.1 mg, 51.5 µmol, 79%).

NMR Spectroscopy:

^{1}**H NMR** (600 MHz, D_2O, 298 K, δ): 8.57 (t, *J* = 5.7 Hz, 1H), 8.53 (d, *J* = 7.5 Hz, 1H), 8.14 (s, 3H), 4.64 (dd, *J* = 8.5, 5.2 Hz, 1H), 4.07 – 3.99 (m, 2H), 3.96 (t, *J* = 6.3 Hz, 1H), 3.37 – 3.27 (m, 2H), 3.14 (dd, *J* = 14.1, 5.2 Hz, 1H), 3.10 – 2.98 (m, 2H), 2.97 (dd, *J* = 14.1, 8.5 Hz, 1H), 2.65 – 2.52 (m, 2H), 2.28 – 2.17 (m, 2H).

^{13}**C NMR** (151 MHz, D_2O, 298 K, δ): 174.7, 173.1, 172.8, 172.7, 163.1, 116.4, 114.7, 53.2, 53.2, 41.3, 33.4, 32.8, 31.7, 31.1, 25.8.

^{19}**F NMR** (470 MHz, D_2O, 298 K, δ): –75.5.

HRMS-ESI (m/z) calculated for $C_{13}H_{21}N_4O_6S_2^+$ [M–TFA]$^+$, 393.0897; found, 393.0896; deviation: –0.3 ppm.

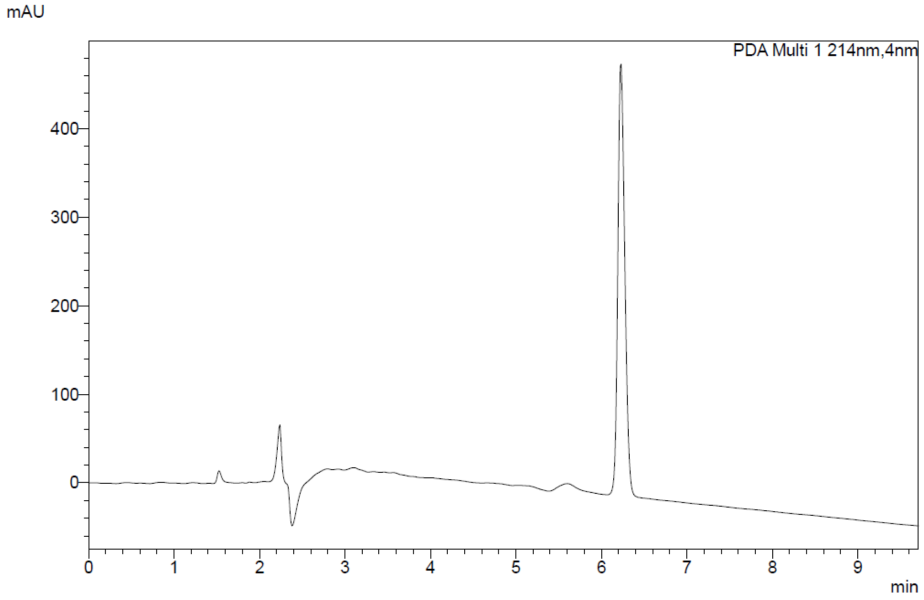

Figure S11. Analytical HPLC trace of **S1** (Eclipse Plus C18 column, 150 x 4.6 mm, 5.0 µm, flow rate = 1.0 mL · min^{-1}) with a linear gradient from 5:95 v/v (MeCN : 0.1% v/v TFA in H_2O) to 30:70 v/v (MeCN : 0.1% v/v TFA in H_2O) over 10 min.

Preparation of Glutathione–S–C_2H_4–N-aniline trifluoroacetate (S2)

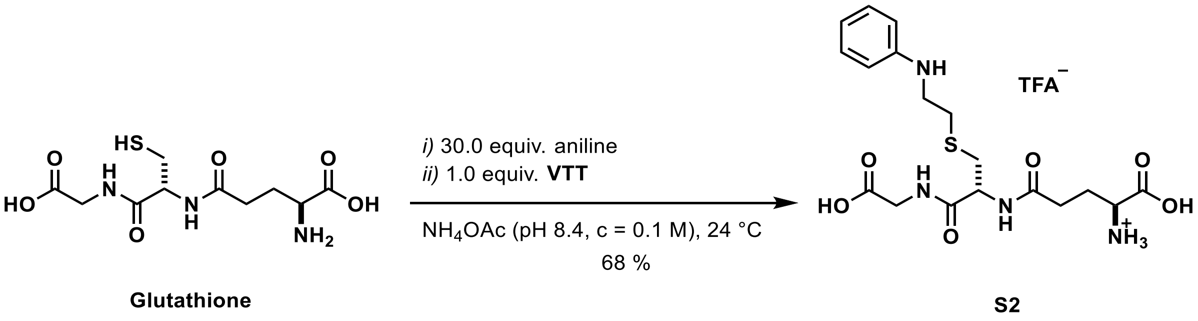

At 20–25 °C, a round-bottom flask (100 mL) was charged with glutathione (40.0 mg, 130 µmol, 1.0 equiv.) and

equipped with a Teflon-coated magnetic stirring bar. Then, a 100 mM solution of aniline in UHQ-H_2O (39.0 mL, 3.90 mmol, 30.0 equiv.), 10.4 mL of aqueous NH_4OAc-buffer (pH 8.4, c = 0.5 M), and 2.6 mL of UHQ-H_2O were added to the flask. Subsequently, a 1.0 M solution of **VTT** in DMF (0.13 mL, 0.13 mmol, 1.0 equiv.) was added to the reaction mixture while stirring at 400 rpm. The reaction mixture was stirred at 400 rpm for 30 minutes at 24 °C. Then, the reaction mixture was filtered, the filtrate was collected, and the filter cake was washed with UHQ-H_2O (3 x 10 mL). The combined filtrate was lyophilized and the obtained residue was purified by HPLC on a YMC Triart C18 column ((150×30 mm, 5µm), flow rate = 42.5 mL/min, 35 °C) with a linear gradient from 5:95 v/v (MeCN : 0.1% v/v TFA in H_2O) to 17.5:82.5 v/v (MeCN : 0.1% v/v TFA in H_2O) over 10 minutes. The fractions containing the product (t ≈ 7.9 min) were collected and lyophilized to afford the title compound as a colorless solid (47.6 mg, 88.1 µmol, 68%).

NMR Spectroscopy:

^{1}H NMR (600 MHz, D_2O, 298 K, δ): 8.54 (d, *J* = 7.7 Hz, 1H), 8.51 (t, *J* = 6.1 Hz, 1H), 7.96 (br, 3H), 7.61 – 7.56 (m, 2H), 7.54 – 7.50 (m, 1H), 7.47 – 7.43 (m, 2H), 4.60 (dd, *J* = 8.2, 5.3 Hz, 1H), 3.95 (s, 2H), 3.82 (t, *J* = 6.4 Hz, 1H), 3.67 (t, *J* = 6.9 Hz, 2H), 3.10 (dd, *J* = 14.2, 5.3 Hz, 1H), 2.94 (dd, *J* = 14.2, 8.2 Hz, 1H), 2.94 – 2.83 (m, 2H), 2.59 – 2.49 (m, 2H), 2.22 – 2.10 (m, 2H).

^{13}C NMR (151 MHz, D_2O, 298 K, δ): 174.9, 173.8, 173.6, 172.4, 163.1 (q, J = 35.3 Hz), 135.2, 130.5, 129.1, 122.0, 116.4 (q, J = 291.5 Hz), 53.9, 53.0, 49.5, 41.7, 32.6, 31.3, 27.2, 26.1.

^{19}F NMR (470 MHz, D_2O, 298 K, δ): –75.5.

HRMS-ESI (m/z) calculated for $C_{18}H_{27}N_4O_6S^+$ [M–TFA]$^+$, 427.1646; found, 427.1648; deviation: +0.5 ppm.

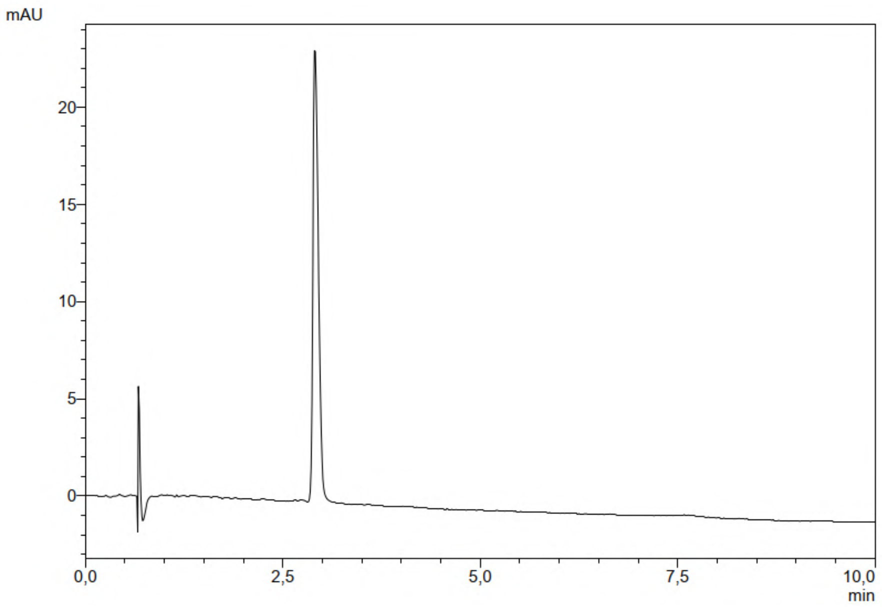

Figure S12. Analytical HPLC trace of **S2** (Eclipse Plus C18 column, 50 x 4.6 mm, 1.8 µm, flow rate = 1.0 mL · min^{-1}) with a linear gradient from 5:95 v/v (MeCN : 0.1% v/v TFA in H_2O) to 30:70 v/v (MeCN : 0.1% v/v TFA in H_2O) over 10 min).

Preparation of Glutathione–S–C_2H_4–N-(4-fluoroaniline) trifluoroacetate (S3)

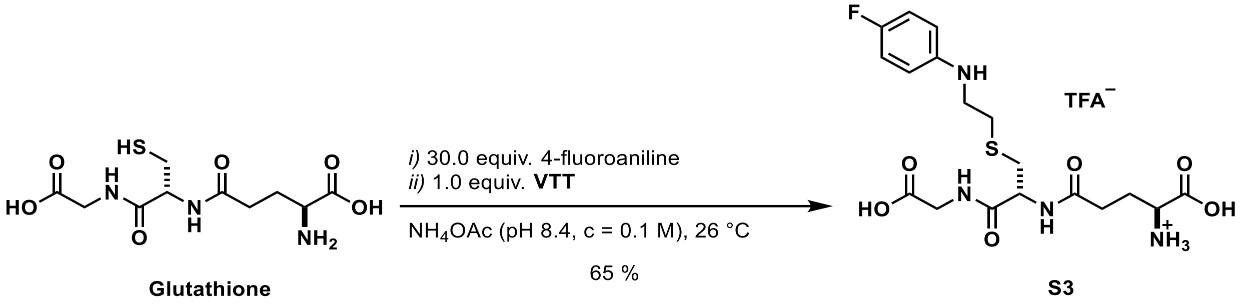

At 20–26 °C, a round-bottom flask (100 mL) was charged with glutathione (40.0 mg, 130 µmol, 1.00 equiv.) and equipped with a Teflon-coated magnetic stirring bar. Then, a 100 mM solution of 4-fluoroaniline in 5:95 v/v DMSO : UHQ-H_2O (39.0 mL, 3.90 mmol, 30.0 equiv.), 10.4 mL of aqueous NH_4OAc-buffer (pH 8.4, c = 0.5 M), and 2.6 mL of UHQ-H_2O were added to the flask. Subsequently, a 1.0 M solution of **VTT** in DMF (0.13 mL, 0.13 mmol, 1.0 equiv.) was added to the reaction mixture while stirring at 400 rpm. The reaction mixture was stirred at 400 rpm for 30 minutes at 26 °C. Then, the reaction mixture was filtered, the filtrate was collected, and the filter cake was washed with UHQ-H_2O (3 x 10 mL). The combined filtrate was lyophilized and the obtained residue was purified by HPLC on a YMC Triart C18 column ((150×30 mm, 5µm), flow rate = 42.5 mL/min, 35 °C) with a linear gradient from 5:95 v/v (MeCN : 0.1% v/v TFA in H_2O) to 17:83 v/v (MeCN : 0.1% v/v TFA in H_2O) over 12 minutes. The fractions containing the product (t ≈ 9.1 min) were collected and lyophilized to afford the title compound as a colorless solid (47.5 mg, 85.0 µmol, 65%).

NMR Spectroscopy:

^{1}H NMR (600 MHz, D_2O, 298 K, δ): 7.59 – 7.46 (m, 2H), 7.40 – 7.27 (m, 2H), 4.60 (dd, *J* = 8.2, 5.3 Hz, 1H), 3.97 (d, *J* = 1.8 Hz, 2H), 3.84 (t, *J* = 6.5 Hz, 1H), 3.67 (t, *J* = 6.8 Hz, 2H), 3.10 (dd, *J* = 14.1, 5.3 Hz, 1H), 2.94 (dd, *J* = 14.1, 8.2 Hz, 1H), 2.89 (td, *J* = 6.9, 3.6 Hz, 2H), 2.55 (td, *J* = 7.5, 4.5 Hz, 2H), 2.23 – 2.12 (m, 2H).

^{13}C NMR (151 MHz, D_2O, 298 K, δ): 174.8, 173.5, 173.5, 172.5, 163.0 (q, J = 35.0 Hz), 162.5 (d, J = 247.0 Hz), 130.7 (d, J = 2.9 Hz), 124.5 (d, J = 9.1 Hz), 117.3 (d, J = 23.6 Hz), 116.4 (q, J = 292.5 Hz), 53.7, 53.0, 50.0, 41.5, 32.6, 31.3, 27.0, 26.0.

^{19}F NMR (470 MHz, D_2O, 298 K, δ): –75.5, –112.0.

HRMS-ESI (m/z) calculated for $C_{18}H_{26}FN_4O_6S^+$ [M–TFA]$^+$, 445.1552; found, 445.1554; deviation: +0.4 ppm.

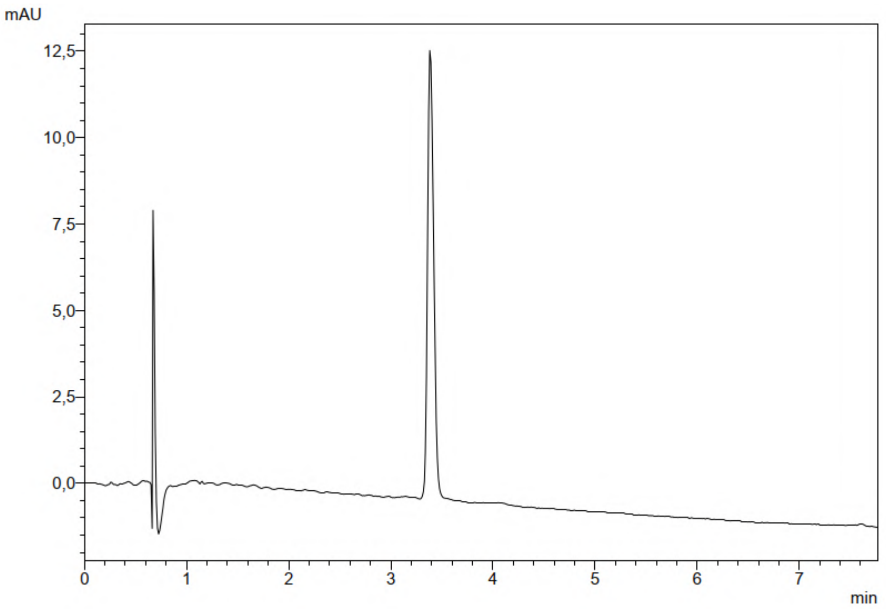

Figure S13. Analytical HPLC trace of **S3** (Eclipse Plus C18 column, 50 x 4.6 mm, 1.8 µm, flow rate = 1.0 mL · min^{-1}) with a linear gradient from 5:95 v/v (MeCN : 0.1% v/v TFA in H_2O) to 30:70 v/v (MeCN : 0.1% v/v TFA in H_2O) over 10 min.

Preparation of Glutathione–S–C_2H_4–N-(3-aminophenylboronic acid) trifluoroacetate (S4)

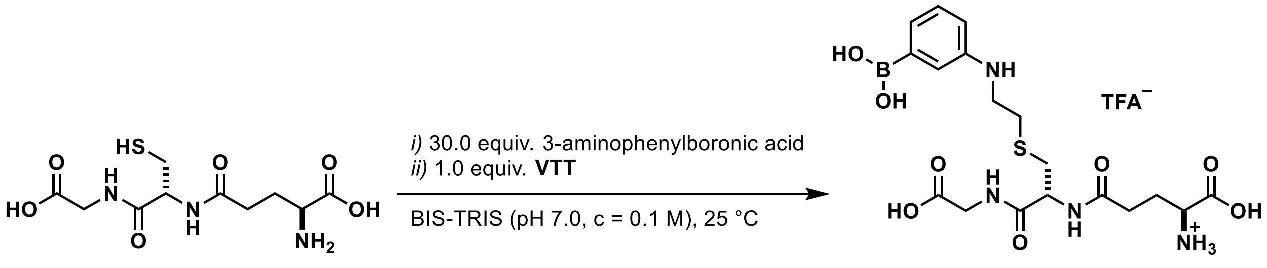

Glutathione **S4**

At 20–25 °C, a round-bottom flask (100 mL) was charged with glutathione (40.0 mg, 130 µmol, 1.00 equiv.) and equipped with a Teflon-coated magnetic stirring bar. Then, a 100 mM solution of 3-aminophenylboronic acid in 1:9 v/v DMSO : UHQ-H_2O (39.0 mL, 3.90 mmol, 30.0 equiv.), 10.4 mL of aqueous BIS-TRIS buffer (pH 7.0, c = 0.5 M), and 2.6 mL of UHQ-H_2O were added to the flask. Subsequently, a 1.0 M solution of **VTT** in DMF (0.13 mL, 0.13 mmol, 1.0 equiv.) was added to the reaction mixture while stirring at 400 rpm. The reaction mixture was stirred at 400 rpm for 30 minutes at 25 °C. Then, the reaction mixture was filtered, the filtrate was collected, and the filter cake was washed with UHQ-H_2O (3 x 10 mL). The combined filtrate was lyophilized and the obtained residue was purified by HPLC on a YMC Triart C18 column ((150×30 mm, 5µm) with a YMC Triart C18 precolumn (50×30mm, 5µm), flow rate = 42.5 mL/min, 35 °C) with a linear gradient from 5:95 v/v (MeCN : 0.1% v/v TFA in H_2O) to 30:70 v/v (MeCN : 0.1% v/v TFA in H_2O) over 20 minutes. The fractions containing the product (t ≈ 7.1 min) were collected and lyophilized to afford the title compound as a colorless solid (55.3 mg, 94.7 µmol, 73%).

NMR Spectroscopy:

^{1}**H NMR** (600 MHz, D_2O, 298 K, δ): 8.53 (d, *J* = 7.7 Hz, 1H), 8.52 (t, *J* = 5.9 Hz, 1H), 7.85 (d, *J* = 7.4 Hz, 1H), 7.77 (d, *J* = 2.3 Hz, 1H), 7.61 (dd, *J* = 8.0, 7.4 Hz, 1H), 7.54 (dd, *J* = 8.0, 2.3 Hz, 1H), 4.59 (dd, *J* = 8.1, 5.3 Hz, 1H), 3.99 – 3.92 (m, 2H), 3.83 (t, *J* = 6.4 Hz, 1H), 3.68 (t, *J* = 6.8 Hz, 2H), 3.09 (dd, *J* = 14.1,

5.3 Hz, 1H), 2.93 (dd, J = 14.1, 8.2 Hz, 1H), 2.94 – 2.83 (m, 2H), 2.59 – 2.48 (m, 2H), 2.21 – 2.11 (m, 2H).

^{13}C NMR (151 MHz, D_2O, 298 K, δ): 174.8, 173.5, 173.5, 172.5, 163.1, 135.4, 134.7, 134.3, 130.0, 126.9, 124.3, 116.4, 53.7, 53.0, 49.7, 41.6, 32.6, 31.3, 27.1, 26.0.

^{19}F NMR (470 MHz, D_2O, 298 K, δ): –75.5.

^{11}B NMR (160 MHz, D_2O, 298 K, δ): 28.8.

HRMS-ESI (m/z) calculated for $C_{18}H_{28}BN_4O_8S^+$ [M–TFA]$^+$, 471.1715; found, 471.1711; deviation: –0.8 ppm.

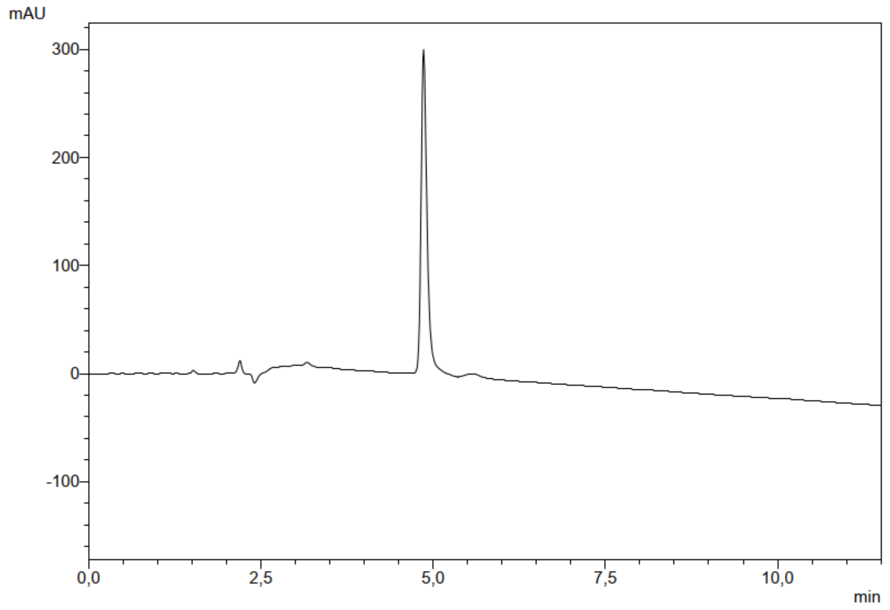

Figure S14. Analytical HPLC trace of **S4** (Eclipse Plus C18 column, 150 x 4.6 mm, 5.0 µm, flow rate = 1.0 mL · min^{-1}) with a linear gradient from 5:95 v/v (MeCN : 0.1% v/v TFA in H_2O) to 30:70 v/v (MeCN : 0.1% v/v TFA in H_2O) over 10 min).

Synthesis of Cysteine derivatives with different nucleophiles

Preparation of Cysteine–S–C_2H_4–thioglucose (S5)

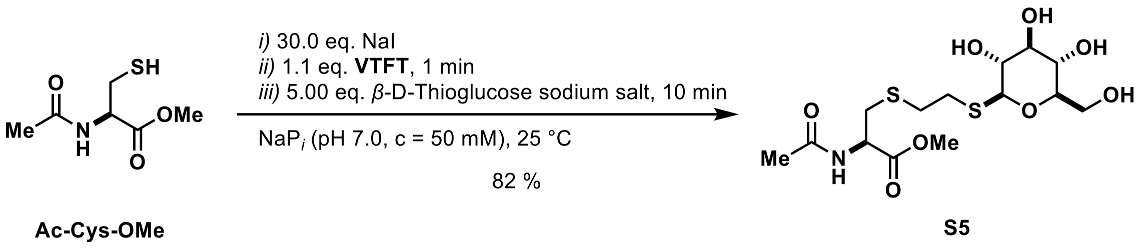

At 20–25 °C, a round-bottom flask (50 mL) was charged with N-acetyl-L-Cysteine methyl ester (35.0 mg, 197 µmol, 1.00 equiv.), sodium iodide (888 mg, 5.92 mmol, 30.0 equiv.), and a Teflon-coated magnetic stirring bar. Then, 17 mL of sodium phosphate buffer (pH 7.0, c = 50 mM) was added to the flask. Subsequently, a 1.0 M solution of **VTFT** in DMF (0.22 mL, 0.22 mmol, 1.1 equiv.) was added to the reaction mixture while stirring at 400 rpm. The reaction mixture was stirred at 400 rpm for 1 minute at 25 °C and a 0.33 M solution of β-D-thioglucose sodium salt in NaP$_i$ (3.00 mL, 0.99 mmol, 5.00 equiv.) was added to the mixture. The reaction mixture was stirred for 10 minutes at 25 °C, the mixture was filtered, and the filter cake was washed with UHQ-H_2O (6 x 5 mL). The obtained filtrate was lyophilized and the obtained residue was purified by HPLC on a YMC Triart C18 column ((150×30 mm, 5µm) with a YMC Triart C18 precolumn (50×30mm, 5µm), flow rate = 42.5 mL/min, 35 °C) with a linear gradient from 20:80 v/v (MeOH : 0.1% v/v TFA in H_2O) to 34:66 (MeOH : 0.1% v/v TFA in H_2O) over 7 minutes. The fractions containing the product (t ≈ 5.3 min) were collected and lyophilized to afford the title compound as a colorless solid (64.8 mg, 162 µmol, 82% yield).

NMR Spectroscopy:

^{1}H NMR (600 MHz, D_2O, 298 K, δ): 4.67 (dd, J = 8.3, 4.9, 1H), 4.59 (d, J = 9.9, 1H), 3.92 (dd, J = 12.5, 2.2, 1H), 3.80 (s, 3H), 3.72 (dd, J = 12.5, 5.8, 1H), 3.51 (t, J = 9.0, 1H), 3.48 (ddd, J = 9.8, 5.8, 2.2, 1H), 3.43 (dd, J = 9.8, 9.0, 1H), 3.33 (dd, J = 9.9, 9.0, 1H), 3.15 (dd, J = 14.0, 4.9, 1H), 3.04 (m, 1H), 2.98 (dd, J = 14.0, 8.3, 1H), 2.94 (m, 1H), 2.90 (m, 2H), 2.08 (s, 3H).

^{13}C NMR (151 MHz, D_2O, 298 K, δ): 174.3, 172.8, 85.5, 80.0, 77.3, 72.4, 69.6, 60.9, 53.2, 52.6, 32.4, 32.3, 30.0, 21.7.

HRMS-ESI (m/z) calculated for $C_{14}H_{25}NNaO_8S_2^+$ [M+Na]$^+$, 422.0914; found, 422.0911; deviation: –0.7 ppm.

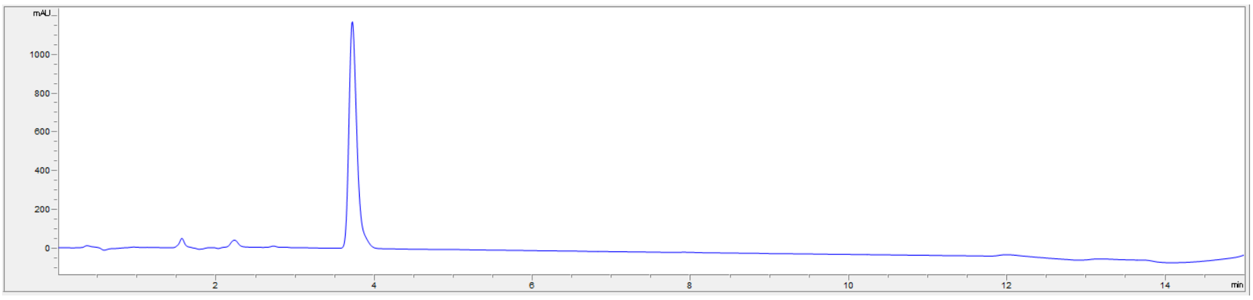

Figure S15. Analytical HPLC trace of **S5** (YMC-Triart C18 column, 150 x 4.6 mm, 5 µm, flow rate = 1.0 mL · min^{-1}) with a linear gradient from 20:80 v/v (MeOH : 0.1% v/v TFA in H_2O) to 50:50 v/v (MeOH : 0.1% v/v TFA in H_2O) over 10 min).

Preparation of Cysteine–(S–C_2H_4)$_2$–dimethylammonium trifluoroacetate (S6)

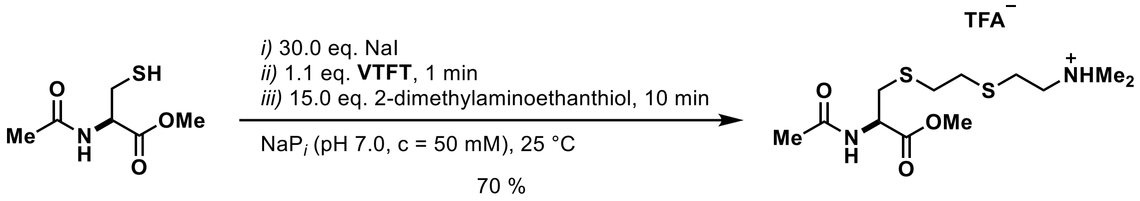

Ac-Cys-OMe **S6**

At 20–25 °C, a round-bottom flask (50 mL) was charged with N-acetyl-L-Cysteine methyl ester (35.0 mg, 197 µmol, 1.00 equiv.), sodium iodide (888 mg, 5.92 mmol, 30.0 equiv.), and a Teflon-coated magnetic stirring bar. Then, 17 mL of sodium phosphate buffer (pH 7.0, c = 50 mM) was added to the flask. Subsequently, a 1.0 M solution of **VTFT** in DMF (0.22 mL, 0.22 mmol, 1.1 equiv.) was added to the reaction mixture while stirring at 400 rpm. The reaction mixture was stirred at 400 rpm for 1 minute at 25 °C and a 0.99 M solution of 2-dimethylaminoethanthiol hydrochloride in NaP$_i$ (3.00 mL, 2.96 mmol, 15.0 equiv.) containing NaOH (2.96 mmol, 15.0 equiv., c = 0.99 M) was added to the mixture. The reaction mixture was stirred for 10 minutes at 25 °C, the mixture was filtered, and the filter cake was washed with UHQ-H_2O (6 x 5 mL). The obtained filtrate was lyophilized and the obtained residue was purified by HPLC on a YMC Triart C18 column ((150×30mm, 5µm) with a YMC Triart C18 precolumn (50×30mm, 5µm), flow rate = 42.5 mL/min, 35 °C) with a linear gradient from 20:80 v/v (MeOH : 0.1% v/v TFA in H_2O) to 50:50 v/v (MeOH : 0.1% v/v TFA in H_2O) over 10 minutes. The fractions containing the product (t ≈ 5.6 min) were collected and lyophilized to afford the title compound as a colorless oil (63.6 mg, 139 µmol, 70% yield).

NMR Spectroscopy:

^{1}H NMR (600 MHz, DMF-d_7, 298 K, δ): 8.46 (d, J = 8.0), 4.61 (td, J = 8.0, 5.7, 1H), 3.71 (s, 3H), 3.47 (m, 2H), 3.04 (m, 2H), 3.04 (dd, J = 13.7, 5.7, 1H), 3.00 (s, 6H), 2.90 (dd, J = 13.7, 7.9, 1H), 2.84 (m, 2H), 2.84 (m, 2H), 1.97 (s, 3H).

^{13}C NMR (151 MHz, DMF-d_7, 298 K, δ): 171.7, 170.1, 160.2 (q, J = 32), 117.7 (q, J = 297), 56.8, 52.9, 52.0, 42.4, 33.2, 32.2, 31.4, 25.4, 22.1.

^{19}F NMR (471 MHz, DMF-d_7, 298 K, δ): –74.9.

HRMS-ESI (m/z) calculated for $C_{12}H_{25}N_2O_3S_2^+$ [M–TFA]$^+$, 309.1301; found, 309.1302; deviation: +0.3 ppm.

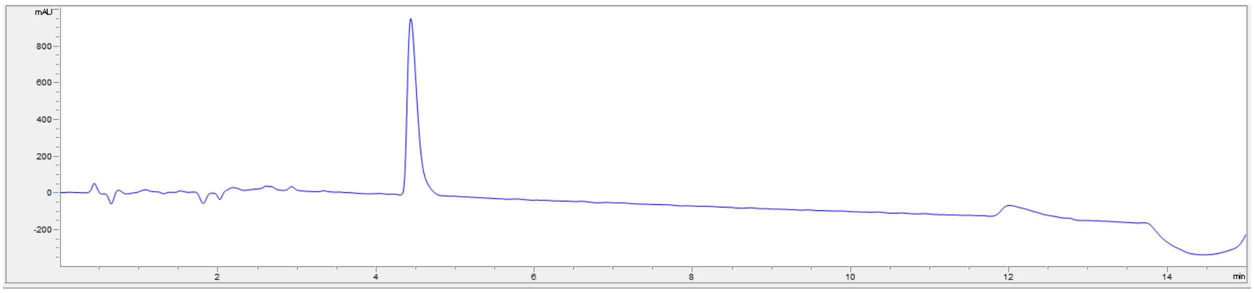

Figure S16. Analytical HPLC trace of **S6** (YMC-Triart C18 column, 150 x 4.6 mm, 5 µm, flow rate = 1.0 mL · min^{-1}) with a linear gradient from 20:80 v/v (MeOH : 0.1% v/v TFA in H_2O) to 50:50 v/v (MeOH : 0.1% v/v TFA in H_2O) over 10 min.

Preparation of Cysteine–S–C_2H_4–thiosulfate (S7)

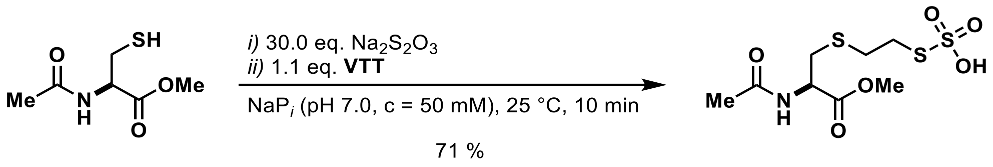

Ac-Cys-OMe **S7**

At 20–25 °C, a round-bottom flask (50 mL) was charged with N-acetyl-L-Cysteine methyl ester (35.0 mg, 197 µmol, 1.00 equiv.), sodium thiosulfate pentahydrate (1.47 g, 5.92 mmol, 30.0 equiv.), and a Teflon-coated magnetic stirring bar. Then, 20 mL of sodium phosphate buffer (pH 7.0, c = 50 mM) was added to the flask. Subsequently, a 1.0 M solution of **VTT** in DMF (0.22 mL, 0.22 mmol, 1.1 equiv.) was added to the reaction mixture while stirring at 400 rpm. The reaction mixture was stirred at 400 rpm for 10 minutes at 25 °C. Then, the reaction mixture was filtered, the filtrate was collected, and the filter cake was washed with UHQ-H_2O (6 x 5 mL). The combined filtrate was lyophilized and the obtained residue was purified by HPLC on a YMC Triart C18 column ((150×30 mm, 5µm) with a YMC Triart C18 precolumn (50×30mm, 5µm), flow rate = 42.5 mL/min, 35 °C) with a linear gradient from 10:90 v/v (MeCN : NH_4OAc buffer (pH 6.8, c = 50 mM)) to 31:69 v/v (MeCN : NH_4OAc buffer (pH 6.8, c = 50 mM)) over 7 minutes. The fractions containing the product (t ≈ 5.3 min) were collected and lyophilized to afford the title compound as a colorless oil (44.8 mg, 141 µmol, 71% yield).

NMR Spectroscopy:

^{1}H NMR (600 MHz, D_2O, 298 K, δ): 4.71 (dd, J = 8.0, 4.9, 1H), 3.83 (s, 3H), 3.34 (m, 2H), 3.19 (dd, J = 14.0, 4.9, 1H), 3.05 (dd, J = 14.0, 8.0, 1H), 3.04 (m, 2H), 2.106 (s, 3H).

^{13}C NMR (151 MHz, D_2O, 298 K, δ): 174.3, 172.7, 53.3, 52.9, 34.8, 32.5, 31.7, 21.8.

HRMS-ESI (m/z) calculated for $C_8H_{14}NO_6S_3^-$ [M-H]$^-$, 315.9989; found, 315.9988; deviation: –0.3 ppm.

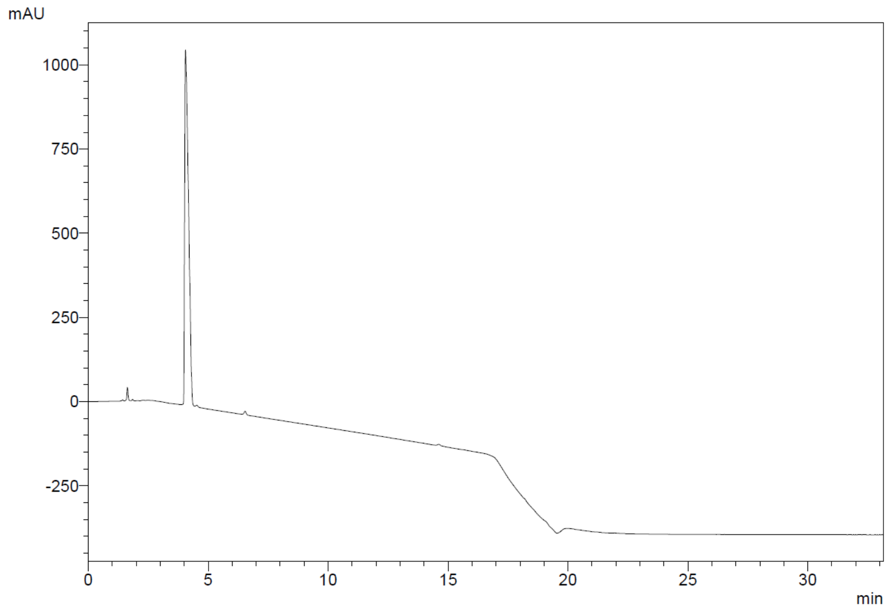

Figure S17. Analytical HPLC trace of **S7** (YMC-Triart C18 column, 150 x 4.6 mm, 5 µm, flow rate = 1.0 mL · min^{-1}) with a linear gradient from 10:90 v/v (MeCN : NH_4OAc buffer (pH 6.8, c = 50 mM)) to 40:60 v/v (MeCN : NH_4OAc buffer (pH 6.8, c = 50 mM)) over 15 minutes.

Synthesis of *p*-nitrothiophenol derivatives

Thiophenol-substituted N-methylmaleimide (S8)

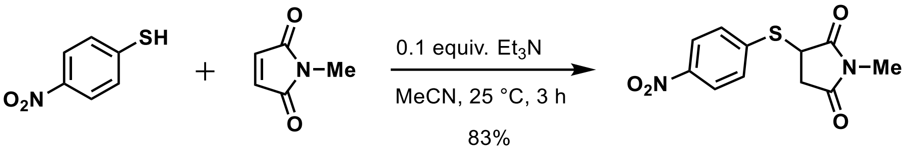

S8

At 20–25 °C, a 25 mL round-bottom flask equipped with a Teflon-coated magnetic stirring bar was charged with *p*-nitrothiophenol (140 mg, 0.90 mmol. 1.00 equiv.), N-methylmaleimide (100 mg, 0.90 mmol, 1.00 equiv.), and dry MeCN (10.0 mL, c = 0.09 M). Triethylamine (12.5 µL, 9.11 mg, 90 µmol, 0.10 equiv.) was added and the mixture was stirred at 300 rpm at 25 °C for 3 hours. The mixture was evaporated at reduced pressure and the obtained residue was purified by chromatography on silica gel eluting with hexanes / EtOAc (1:0 v/v to 3:2 v/v) to afford 199 mg (83%) of the title compound as a grey solid.

R$_f$ = 0.44 (silica gel, hexanes / EtOAc, 1:1 (v/v)).

NMR Spectroscopy:

^{1}H NMR (500 MHz, $CDCl_3$, 298 K, δ): 8.22 – 8.15 (m, 2H), 7.67 – 7.61 (m, 2H), 4.28 (dd, *J* = 9.3, 4.3 Hz, 1H), 3.30 (dd, *J* = 18.7, 9.3 Hz, 1H), 3.03 (s, 3H), 2.70 (dd, *J* = 18.7, 4.3 Hz, 1H).

^{13}C NMR (126 MHz, $CDCl_3$, 298 K, δ): 174.9, 173.5, 146.8, 142.1, 130.2, 124.1, 42.3, 35.8, 25.3.

HRMS-EI(m/z) calc'd for $C_{11}H_{10}N_2O_4S^+$ [M]$^+$, 266.0356; found, 266.0361; deviation: +1.9 ppm.

Thiophenol-derived azide (S9)

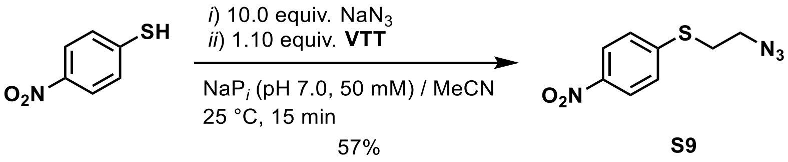

S9

At 20–25 °C, a 50 mL round-bottom flask equipped with a Teflon-coated magnetic stirring bar was charged with sodium azide (168 mg, 2.58 mmol, 10.0 equiv.). Then, 13 mL of sodium phosphate buffer (pH 7.0, c = 50 mM) and 13 mL of MeCN were added to the flask. 4-Nitrothiophenol (40.0 mg, 258 µmol, 1.00 equiv.) was added to the mixture while stirring at 300 rpm. Subsequently, **VTT** (93.6 mg, 284 µmol, 1.10 equiv.) was added to the mixture over 30 seconds. The mixture was stirred at 300 rpm for 15 minutes at 25 °C and concentrated under reduced pressure. Ethyl acetate (25 mL) was added to the mixture, the organic layer was separated and the aqueous layer was extracted with ethyl acetate (3 × 25 mL). The combined organic layer was dried over magnesium sulfate, filtered, and dried under reduced pressure. The obtained residue was purified by chromatography on silica gel eluting with pentane / DCM (7:3 (v/v)) to afford 32.7 mg (57%) of the title compound as a yellow oil.

R$_f$ = 0.36 (silica gel, pentane / DCM, 3:2 (v/v))

NMR Spectroscopy:

^{1}H NMR (500 MHz, DMSO-d_6, 298 K, δ): 8.17 – 8.10 (m, 2H), 7.60 – 7.53 (m, 2H), 3.63 (t, *J* = 6.5 Hz, 2H), 3.38 (t, *J* = 6.5 Hz, 2H).

^{13}C NMR (126 MHz, DMSO-d_6, 298 K, δ): 146.2, 144.7, 126.7, 123.9, 49.3, 30.6.

HRMS-EI (m/z) calc'd for $C_8H_8N_4O_2S^+$ $[M]^+$, 224.0362; found, 224.0362; deviation: 0.0 ppm.

Peptide stapling and disulfide rebridging

General considerations for peptide synthesis

General set-up

Peptides were synthesized manually *via* solid-phase peptide synthesis (SPPS) following the Fmoc/tBu-strategy. Fmoc-Rink-Amide-2CT resin (200-400 mesh, 0.67 mmol/g loading, 1% DVB) was used as solid support. All reaction steps were carried out in a fritted peptide synthesis vessel that was fitted to a Heidolph Vibramax 100 to allow for automatic stirring of the reaction mixtures. Depending on the reaction scale, either a 50 mL or a 100 mL peptide synthesis vessel was used. Peptide-grade DMF was used in all steps. The amount of reactants and solvents used in individual steps are calculated based on the amount of resin. All reaction steps were performed at 25 °C under ambient atmosphere, unless otherwise noted. General descriptions of the individual reaction steps are provided with references. Detailed procedures for the synthesis of the discussed peptide sequences are provided below.

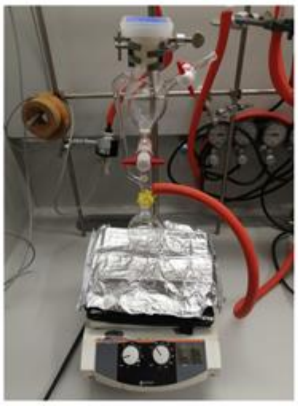

Figure S18. Standard set-up used for the synthesis of peptides.

Loading and deprotection of the resin

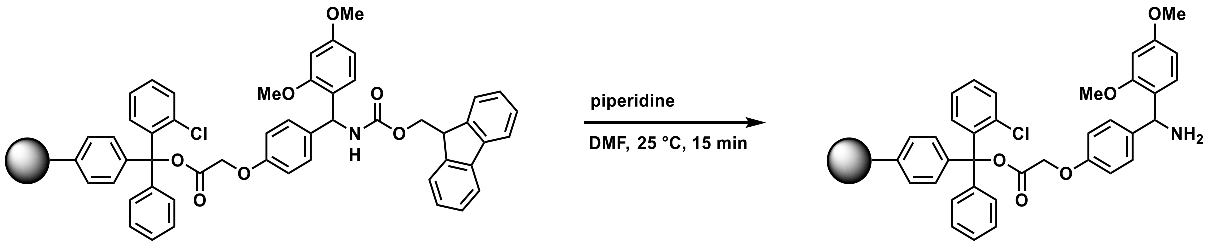

Upon charging the reaction vessel with Fmoc-Rink-Amide-2CT resin (500 mg for a typical synthesis), DCM was added (20 mL · g^{-1}) and the suspension was shaken for 30 minutes at 25 °C to swell the resin. The solvent was removed through vacuum filtration and the resin was washed once with DCM (20 mL · g^{-1}) and three times with DMF (20 mL · g^{-1}). The swollen resin was then incubated with a solution of 20% piperidine in DMF (v/v, 20 mL · g^{-1}) for 15 minutes for Fmoc-deprotection. The liquid was removed via vacuum filtration and the resin was washed once with DMF (20 mL · g^{-1}). After that, the resin was incubated with 20% piperidine in DMF (v/v, 20 mL · g^{-1}) for 5 min. After removing the liquid via vacuum filtration, deprotection was completed by washing the resin five times with DMF (20 mL · g^{-1})[7].

Coupling of amino acids to the resin

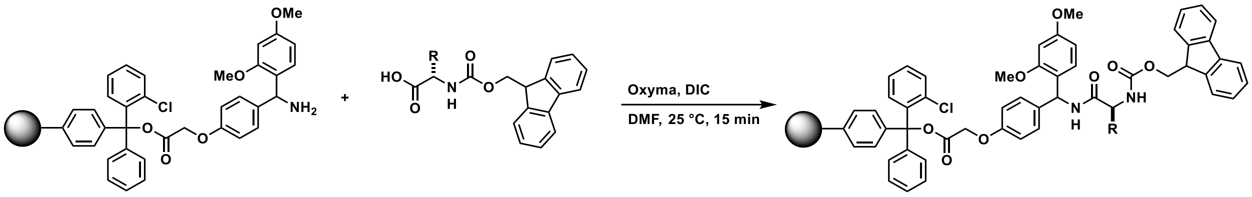

The deprotected and washed resin was incubated with a pre-mixed solution of the respective Fmoc-protected amino acid (Fmoc-AA-OH, 4.00 equiv.), Oxyma (4.00 equiv.) and diisopropylcarbodiimide (DIC, 4.00 equiv.) in DMF (20 mL · g^{-1}) for 1 h at 25 °C. The coupling step was completed by removing the solution via vacuum filtration and washing the resin five times with DMF (20 mL · g^{-1})[8].

Fmoc-Deprotection of coupled amino acids

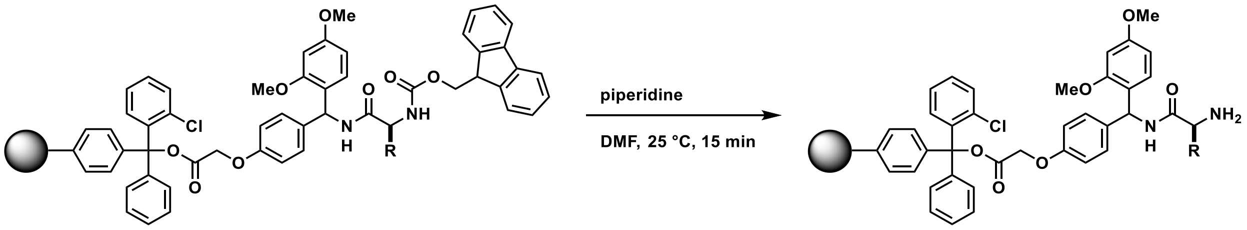

The loaded resin was incubated with a solution of 20% piperidine in DMF (v/v, 20 mL · g^{-1}) for 15 min. The liquid was removed via vacuum filtration and the resin was washed once with DMF (20 mL · g^{-1}). After that, the resin was incubated with 20% piperidine in DMF (v/v, 20 mL · g^{-1}) for 5 min. After removing the liquid via vacuum filtration, deprotection was completed by washing the resin five times with DMF (20 mL · g^{-1})[7].

Cleavage of the peptide from the resin

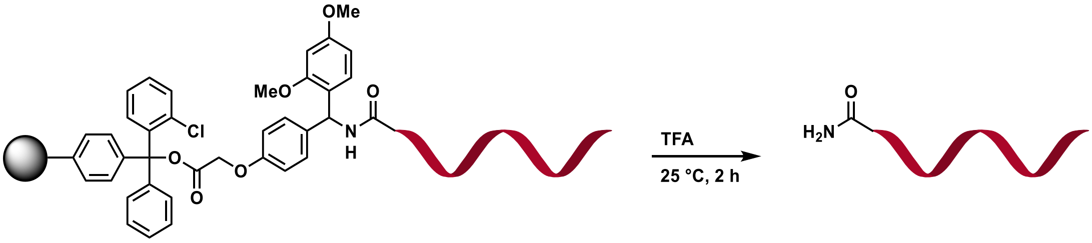

The completed peptide sequence was washed five times with DCM (20 mL · g^{-1}). Then, the resin was incubated with a mixture of 90% TFA + 5% thioanisole + 3% 1,2-ethanedithiol + 2% anisole, (v/v, 10 mL · g^{-1}) for 2 h at 25 °C. The mixture was then added dropwise over 10 minutes into 50 mL cold diethyl ether (–20 °C), where immediate precipitation of the peptide could be observed. Argon was bubbled through the suspension for 5 minutes and precipitation of the peptide was completed over night at –20 °C. The crude peptide mixture was then obtained by filtration and drying of the precipitate under an argon stream. Purification was performed by reversed phase HPLC. HPLC methods are provided for every individual example.

Synthesis of Linear Peptides via SPPS

Ac-Ala-Cys-Leu-Leu-Gln-Glu-Phe-Ala-Pro-Pro-Trp-Ile-NH_2 (S10)

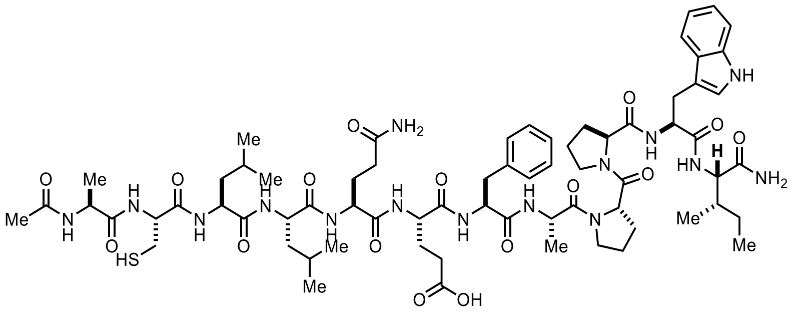

S10 (51%)

All steps were performed under ambient atmosphere at 25 °C. A 50 mL fritted peptide synthesis vessel was charged with Fmoc-Rink-Amide-2CT resin (200-400 mesh, 0.67 mmol · g^{-1} loading, 1% DVB, 0.335 mmol, 500 mg, 1.00 equiv.) and DCM (10 mL, 20 mL · g^{-1}) was added. The suspension was shaken on a Heidolph Vibramax 100 for 30 min, the liquid was removed via vacuum filtration and the resin was washed once with DCM (10 mL, 20 mL · g^{-1}), and three times with DMF (10 mL, 20 mL · g^{-1}). The resin was incubated with 20% piperidine in DMF (v/v, 10 mL, 20 mL · g^{-1}) for 15 min, the liquid was removed via vacuum filtration and the resin was washed with DMF (10 mL, 20 mL · g^{-1}). Deprotection was completed by incubation of the resin with 20% piperidine in DMF (v/v, 10 mL, 20 mL · g^{-1}) for 5 minutes, and after vacuum-filtration, washing five times with DMF (10 mL, 20 mL · g^{-1}).

In a 50 mL round bottom flask, equipped with a Teflon-coated magnetic stirring bar, a solution of Fmoc-Ile-OH (474 mg, 1.34 mmol, 4.00 equiv.), Oxyma (190 mg, 1.34 mmol, 4.00 equiv.) and DIC (208 µL, 169 mg, 1.34 mmol, 4.00 equiv.) in DMF (10 mL) was stirred for 5 minutes at 25 °C and then added to the resin. After 1 h, the coupling mixture was removed via vacuum filtration and the resin was washed five times with DMF (10 mL, 20 mL · g^{-1}). The resin was incubated with 20% piperidine in DMF (v/v, 10 mL, 20 mL · g^{-1}) for 15 min, then the liquid was removed via vacuum filtration and the resin was washed once with DMF (10 mL, 20 mL · g^{-1}). Deprotection was completed by incubation of the resin with 20% piperidine in DMF (v/v, 10 mL, 20 mL · g^{-1}) for 5 minutes and washing five times with DMF (10 mL, 20 mL · g^{-1}). In a 50 mL round bottom flask, equipped with a Teflon-coated magnetic stirring bar, a solution of Fmoc-Trp(Boc)-OH (706 mg, 1.34 mmol, 4.00 equiv.), Oxyma (190 mg, 1.34 mmol, 4.00 equiv.) and DIC (208 µL, 169 mg, 1.34 mmol, 4.00 equiv.) in DMF (10 mL) was stirred for 5 minutes at 25 °C and then added to the resin. After 1 h, the coupling mixture was removed via vacuum filtration and the resin was washed five times with DMF (10 mL, 20 mL · g^{-1}). The resin was incubated with 20% piperidine in DMF (v/v, 10 mL, 20 mL · g^{-1}) for 15 min, then the liquid was removed via vacuum filtration and the resin was washed once with DMF (10 mL, 20 mL · g^{-1}). Deprotection was completed by incubation of the resin with 20% piperidine in DMF (v/v, 10 mL, 20 mL · g^{-1}) for 5 minutes and washing five times with DMF (10 mL, 20 mL · g^{-1}). In a 50 mL round bottom flask, equipped with a Teflon-coated magnetic stirring bar, a solution of Fmoc-Pro-OH (452 mg, 1.34 mmol, 4.00 equiv.), Oxyma (190 mg, 1.34 mmol, 4.00 equiv.) and DIC (208 µL, 169 mg, 1.34 mmol, 4.00 equiv.) in DMF (10 mL) was stirred for 5 minutes at 25 °C and then added to the resin. After 1 h, the coupling mixture was removed via vacuum filtration and the resin was washed five times with DMF (10 mL, 20 mL · g^{-1}). The resin was incubated with 20% piperidine in DMF (v/v, 10 mL, 20 mL · g^{-1}) for 15 min, then the liquid was removed via vacuum filtration and the resin was

washed once with DMF (10 mL, 20 mL · g^{-1}). Deprotection was completed by incubation of the resin with 20% piperidine in DMF (v/v, 10 mL, 20 mL · g^{-1}) for 5 minutes and washing five times with DMF (10 mL, 20 mL · g^{-1}). In a 50 mL round bottom flask, equipped with a Teflon-coated magnetic stirring bar, a solution of Fmoc-Pro-OH (452 mg, 1.34 mmol, 4.00 equiv.), Oxyma (190 mg, 1.34 mmol, 4.00 equiv.) and DIC (208 µL, 169 mg, 1.34 mmol, 4.00 equiv.) in DMF (10 mL) was stirred for 5 minutes at 25 °C and then added to the resin. After 1 h, the coupling mixture was removed via vacuum filtration and the resin was washed five times with DMF (10 mL, 20 mL · g^{-1}). The resin was incubated with 20% piperidine in DMF (v/v, 10 mL, 20 mL · g^{-1}) for 15 min, then the liquid was removed via vacuum filtration and the resin was washed once with DMF (10 mL, 20 mL · g^{-1}). Deprotection was completed by incubation of the resin with 20% piperidine in DMF (v/v, 10 mL, 20 mL · g^{-1}) for 5 minutes and washing five times with DMF (10 mL, 20 mL · g^{-1}). In a 50 mL round bottom flask, equipped with a Teflon-coated magnetic stirring bar, a solution of Fmoc-Ala-OH (417 mg, 1.34 mmol, 4.00 equiv.), Oxyma (190 mg, 1.34 mmol, 4.00 equiv.) and DIC (208 µL, 169 mg, 1.34 mmol, 4.00 equiv.) in DMF (10 mL) was stirred for 5 minutes at 25 °C and then added to the resin. After 1 h, the coupling mixture was removed via vacuum filtration and the resin was washed five times with DMF (10 mL, 20 mL · g^{-1}). The resin was incubated with 20% piperidine in DMF (v/v, 10 mL, 20 mL · g^{-1}) for 15 min, then the liquid was removed via vacuum filtration and the resin was washed once with DMF (10 mL, 20 mL · g^{-1}). Deprotection was completed by incubation of the resin with 20% piperidine in DMF (v/v, 10 mL, 20 mL · g^{-1}) for 5 minutes and washing five times with DMF (10 mL, 20 mL · g^{-1}). In a 50 mL round bottom flask, equipped with a Teflon-coated magnetic stirring bar, a solution of Fmoc-Phe-OH (519 mg, 1.34 mmol, 4.00 equiv.), Oxyma (190 mg, 1.34 mmol, 4.00 equiv.) and DIC (208 µL, 169 mg, 1.34 mmol, 4.00 equiv.) in DMF (10 mL) was stirred for 5 minutes at 25 °C and then added to the resin. After 1 h, the coupling mixture was removed via vacuum filtration and the resin was washed five times with DMF (10 mL, 20 mL · g^{-1}). The resin was incubated with 20% piperidine in DMF (v/v, 10 mL, 20 mL · g^{-1}) for 15 min, then the liquid was removed via vacuum filtration and the resin was washed once with DMF (10 mL, 20 mL · g^{-1}). Deprotection was completed by incubation of the resin with 20% piperidine in DMF (v/v, 10 mL, 20 mL · g^{-1}) for 5 minutes and washing five times with DMF (10 mL, 20 mL · g^{-1}). In a 50 mL round bottom flask, equipped with a Teflon-coated magnetic stirring bar, a solution of Fmoc-Glu(O^{t}Bu)-OH (440 mg, 1.34 mmol, 4.00 equiv.), Oxyma (190 mg, 1.34 mmol, 4.00 equiv.) and DIC (208 µL, 169 mg, 1.34 mmol, 4.00 equiv.) in DMF (10 mL) was stirred for 5 minutes at 25 °C and then added to the resin. After 1 h, the coupling mixture was removed via vacuum filtration and the resin was washed five times with DMF (10 mL, 20 mL · g^{-1}). The resin was incubated with 20% piperidine in DMF (v/v, 10 mL, 20 mL · g^{-1}) for 15 min, then the liquid was removed via vacuum filtration and the resin was washed once with DMF (10 mL, 20 mL · g^{-1}). Deprotection was completed by incubation of the resin with 20% piperidine in DMF (v/v, 10 mL, 20 mL · g^{-1}) for 5 minutes and washing five times with DMF (10 mL, 20 mL · g^{-1}). In a 50 mL round bottom flask, equipped with a Teflon-coated magnetic stirring bar, a solution of Fmoc-Gln(Trt)-OH (818 mg, 1.34 mmol, 4.00 equiv.), Oxyma (190 mg, 1.34 mmol, 4.00 equiv.) and DIC (208 µL, 169 mg, 1.34 mmol, 4.00 equiv.) in DMF (10 mL) was stirred for 5 minutes at 25 °C and then added to the resin. After 1 h, the coupling mixture was removed via vacuum filtration and the resin was washed five times with DMF (10 mL, 20 mL · g^{-1}). The resin was incubated with 20% piperidine in DMF (v/v, 10 mL, 20 mL · g^{-1}) for 15 min, then the liquid was removed via vacuum filtration and the resin was washed once with DMF (10 mL, 20 mL · g^{-1}). Deprotection was completed by incubation of the resin with 20% piperidine in DMF (v/v, 10 mL, 20 mL · g^{-1}) for 5 minutes and washing five times with DMF (10 mL, 20 mL · g^{-1}). In a 50 mL round bottom flask, equipped with a Teflon-coated magnetic stirring bar, a solution of Fmoc-Leu-OH

(474 mg, 1.34 mmol, 4.00 equiv.), Oxyma (190 mg, 1.34 mmol, 4.00 equiv.) and DIC (208 µL, 169 mg, 1.34 mmol, 4.00 equiv.) in DMF (10 mL) was stirred for 5 minutes at 25 °C and then added to the resin. After 1 h, the coupling mixture was removed via vacuum filtration and the resin was washed five times with DMF (10 mL, 20 mL · g^{-1}). The resin was incubated with 20% piperidine in DMF (v/v, 10 mL, 20 mL · g^{-1}) for 15 min, then the liquid was removed via vacuum filtration and the resin was washed once with DMF (10 mL, 20 mL · g^{-1}). Deprotection was completed by incubation of the resin with 20% piperidine in DMF (v/v, 10 mL, 20 mL · g^{-1}) for 5 minutes and washing five times with DMF (10 mL, 20 mL · g^{-1}). In a 50 mL round bottom flask, equipped with a Teflon-coated magnetic stirring bar, a solution of Fmoc-Leu-OH (474 mg, 1.34 mmol, 4.00 equiv.), Oxyma (190 mg, 1.34 mmol, 4.00 equiv.) and DIC (208 µL, 169 mg, 1.34 mmol, 4.00 equiv.) in DMF (10 mL) was stirred for 5 minutes at 25 °C and then added to the resin. After 1 h, the coupling mixture was removed via vacuum filtration and the resin was washed five times with DMF (10 mL, 20 mL · g^{-1}). The resin was incubated with 20% piperidine in DMF (v/v, 10 mL, 20 mL · g^{-1}) for 15 min, then the liquid was removed via vacuum filtration and the resin was washed once with DMF (10 mL, 20 mL · g^{-1}). Deprotection was completed by incubation of the resin with 20% piperidine in DMF (v/v, 10 mL, 20 mL · g^{-1}) for 5 minutes and washing five times with DMF (10 mL, 20 mL · g^{-1}). In a 50 mL round bottom flask, equipped with a Teflon-coated magnetic stirring bar, a solution of Fmoc-Cys(Trt)-OH (785 mg, 1.34 mmol, 4.00 equiv.), Oxyma (190 mg, 1.34 mmol, 4.00 equiv.) and DIC (208 µL, 169 mg, 1.34 mmol, 4.00 equiv.) in DMF (10 mL) was stirred for 5 minutes at 25 °C and then added to the resin. After 1 h, the coupling mixture was removed via vacuum filtration and the resin was washed five times with DMF (10 mL, 20 mL · g^{-1}). The resin was incubated with 20% piperidine in DMF (v/v, 10 mL, 20 mL · g^{-1}) for 15 min, then the liquid was removed via vacuum filtration and the resin was washed once with DMF (10 mL, 20 mL · g^{-1}). Deprotection was completed by incubation of the resin with 20% piperidine in DMF (v/v, 10 mL, 20 mL · g^{-1}) for 5 minutes and washing five times with DMF (10 mL, 20 mL · g^{-1}). In a 50 mL round bottom flask, equipped with a Teflon-coated magnetic stirring bar, a solution of Ac-Ala-OH (176 mg, 1.34 mmol, 4.00 equiv.), Oxyma (190 mg, 1.34 mmol, 4.00 equiv.) and DIC (208 µL, 169 mg, 1.34 mmol, 4.00 equiv.) in DMF (10 mL) was stirred for 5 minutes at 25 °C and then added to the resin. After 1 h, the coupling mixture was removed via vacuum filtration and the resin was washed five times with DMF (10 mL, 20 mL · g^{-1}). The resin was incubated with 20% piperidine in DMF (v/v, 10 mL, 20 mL · g^{-1}) for 15 min, then the liquid was removed via vacuum filtration and the resin was washed once with DMF (10 mL, 20 mL · g^{-1}). Deprotection was completed by incubation of the resin with 20% piperidine in DMF (v/v, 10 mL, 20 mL · g^{-1}) for 5 minutes and washing five times with DMF (10 mL, 20 mL · g^{-1}).

The completed peptide sequence was washed five times with DCM (10 mL, 20 mL · g^{-1}). Then, the resin was incubated with 5 mL of a mixture of 90% TFA + 5% thioanisole + 3% 1,2-ethanedithiol + 2% anisole, (v/v) for 2 h at 25 °C. The mixture was then added dropwise over 10 minutes into 50 mL cold diethyl ether (–20 °C), where immediate precipitation of the peptide was observed. Argon was bubbled through the suspension for 5 minutes and precipitation of the peptide was completed over night at –20 °C. The crude peptide mixture was then obtained by filtration and drying of the precipitate under an argon stream. Purification was performed by reversed phase HPLC using an YMC Triart C18 column ((150×30mm, 5 µm) with an YMC Triart C18 precolumn (50×30 mm, 5 µm), flow rate = 42.5 mL/min, 35 °C) with a linear gradient from 35:65 v/v (MeCN : 0.1% v/v TFA in H_2O) to 66:34 v/v (MeCN : 0.1% v/v TFA in H_2O) over 9 min, followed by 70:30 v/v (MeCN : 0.1% v/v TFA in H_2O) for 8 min. The fractions containing the desired product (t ≈ 10 min) were collected and lyophilized to afford the title compound **S10** as a colorless powder (249 mg, 0.174 mmol, 51% yield).

NMR Spectroscopy:

^{1}H NMR (600 MHz, DMSO-d_6, 298 K, δ): 10.81 (d, *J* = 2.4 Hz, 1H), 8.14 (d, *J* = 7.2 Hz, 1H), 8.11 (d, *J* = 7.0 Hz, 1H), 8.05 (d, *J* = 7.9 Hz, 1H), 7.96 (d, *J* = 8.0 Hz, 1H), 7.95 (d, *J* = 7.9 Hz, 1H), 7.91 (d, *J* = 8.2 Hz, 1H), 7.89 (d, *J* = 7.8 Hz, 1H), 7.84 (d, *J* = 7.9 Hz, 1H), 7.76 (d, *J* = 7.6 Hz, 1H), 7.63 (d, *J* = 9.0 Hz, 1H), 7.52 (ddt, *J* = 7.9, 1.1, 0.8 Hz, 1H), 7.31 (dt, *J* = 8.1, 0.9 Hz, 1H), 7.27 (m, 1H), 7.22 (m, 4H), 7.20 (m, 1H), 7.16 (m, 1H), 7.11 (d, *J* = 2.4 Hz, 1H), 7.05 (ddd, *J* = 8.2, 7.0, 1.2 Hz, 1H), 7.04 (m, 1H), 6.96 (ddd, *J* = 8.0, 6.9, 1.0 Hz, 1H), 6.78 (m, 1H), 4.50 (m, 1H), 4.47 (m, 2H), 4.36 (ddd, *J* = 7.9, 7.4, 5.3 Hz, 1H), 4.30 (dd, *J* = 8.5, 2.7 Hz, 1H), 4.28 (m, 1H), 4.27 (m, 1H), 4.25 (quint, *J* = 7.1 Hz, 1H), 4.18 (m, 1H), 4.16 (m, 1H), 4.13 (dd, *J* = 9.0, 6.9 Hz, 1H), 3.60 (dt, *J* = 9.6, 7.4 Hz, 1H), 3.53 (dt, *J* = 9.6, 7.0 Hz, 1H), 3.45 (m, 1H), 3.40 (dt, *J* = 9.7, 7.5 Hz, 1H), 3.15 (dd, *J* = 14.9, 5.3 Hz, 1H), 3.01 (dd, *J* = 15.0, 7.8 Hz, 1H), 2.99 (dd, *J* = 13.7, 5.0 Hz, 1H), 2.78 (ddd, *J* = 13.5, 8.9, 5.3 Hz, 1H), 2.74 (dd, *J* = 13.7, 9.5 Hz, 1H), 2.71 (ddd, *J* = 13.5, 8.0, 7.4 Hz, 1H), 2.30 (dd, *J* = 8.9, 8.0 Hz, 1H), 2.12 (m, 2H), 2.05 (m, 2H), 1.99 (m, 1H), 1.95 (m, 1H), 1.84 (s, 3H), 1.83 (m, 1H), 1.82 (m, 2H), 1.81 (m, 2H), 1.79 (m, 1H), 1.75 (m, 1H), 1.70 (m, 1H), 1.68 (m, 1H), 1.66 (m, 1H), 1.59 (m, 2H), 1.57 (m, 1H), 1.45 (m, 2H), 1.44 (m, 2H), 1.38 (dqd, *J* = 13.7, 7.4, 3.7 Hz, 1H), 1.18 (d, *J* = 7.1 Hz, 3H), 1.15 (d, *J* = 6.9 Hz, 3H), 1.05 (ddq, *J* = 13.7, 9.4, 7.4 Hz, 1H), 0.87 (d, *J* = 6.6 Hz, 3H), 0.86 (d, *J* = 6.4 Hz, 3H), 0.82 (d, *J* = 6.6 Hz, 3H), 0.81 (d, *J* = 6.6 Hz, 3H), 0.80 (t, *J* = 7.4 Hz, 3H), 0.80 (d, *J* = 6.8 Hz, 3H).

^{13}C NMR (151 MHz, $(CD_3)_2SO$, 25°C, δ): 173.9, 173.8, 172.7, 172.7, 172.0, 171.8, 171.3, 171.0, 170.9, 170.7, 170.3, 170.2, 169.6, 169.5, 169.3, 137.5, 136.0, 129.1, 128.0, 127.4, 126.2, 123.5, 120.8, 118.3, 118.2, 111.2, 109.7, 59.4, 57.4, 56.7, 54.9, 53.5, 53.4, 52.2, 51.8, 51.2, 51.0, 48.4, 46.6, 46.5, 46.1, 40.6, 40.4, 37.4, 36.8, 31.4, 30.0, 28.6, 27.7, 27.6, 27.5, 26.9, 25.9, 24.5, 24.3, 24.1, 24.1, 24.0, 23.1, 23.0, 22.4, 21.5, 17.9, 16.7, 15.3, 11.1.

HRMS-ESI (m/z) calc'd for $C_{69}H_{101}N_{15}O_{16}SNa^+$ $[M+Na]^+$, 1450.716; found, 1450.715; deviation: –0.7 ppm.

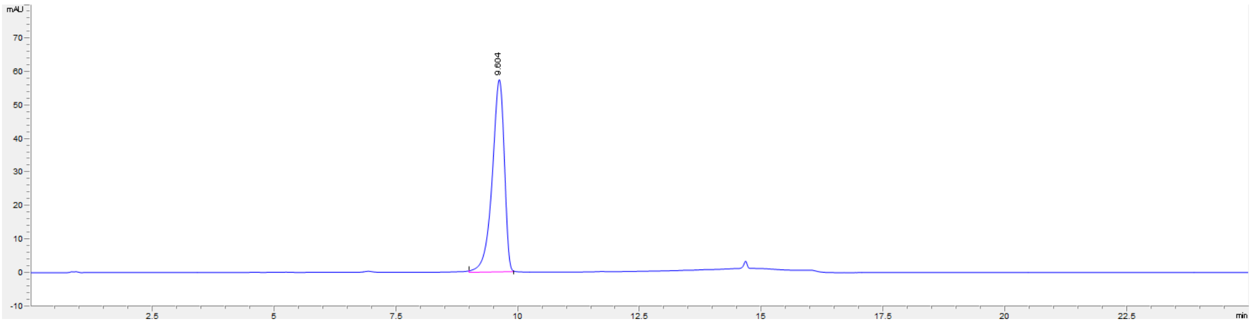

Figure S19. Analytical HPLC Trace of S10 (YMC Triart C18, 150 × 4.6 mm, 5 µm, flow rate = 1 mL· min^{-1}) with a linear gradient from 65:35 v/v (0.1% v/v TFA in H_2O : MeCN) to 60:40 v/v (0.1% v/v TFA in H_2O : MeCN) over 10 minutes to 40:60 v/v (0.1% v/v TFA in H_2O : MeCN) over 5 minutes.

Ac-Ala-Cys-Leu-Leu-Gln-Cys-Phe-Ala-Pro-Pro-Trp-Ile-NH_2 (S11)

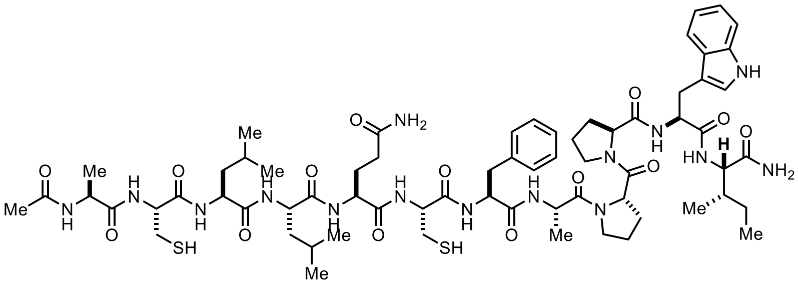

S11 (38%)

All steps were performed under ambient atmosphere at 25 °C. A 50 mL fritted peptide synthesis vessel was charged with Fmoc-Rink-Amide-2CT resin (200-400 mesh, 0.67 mmol · g^{-1} loading, 1% DVB, 0.335 mmol, 500 mg, 1.00 equiv.) and DCM (10 mL, 20 mL · g^{-1}) was added. The suspension was shaken on a Heidolph Vibramax 100 for 30 min, the liquid was removed via vacuum filtration and the resin was washed once with DCM (10 mL, 20 mL · g^{-1}), and three times with DMF (10 mL, 20 mL · g^{-1}). The resin was incubated with 20% piperidine in DMF (v/v, 10 mL, 20 mL · g^{-1}) for 15 min, the liquid was removed via vacuum filtration and the resin was washed with DMF (10 mL, 20 mL · g^{-1}). Deprotection was completed by incubation of the resin with 20% piperidine in DMF (v/v, 10 mL, 20 mL · g^{-1}) for 5 minutes, and after vacuum-filtration, washing five times with DMF (10 mL, 20 mL · g^{-1}).

In a 50 mL round bottom flask, equipped with a Teflon-coated magnetic stirring bar, a solution of Fmoc-Ile-OH (474 mg, 1.34 mmol, 4.00 equiv.), Oxyma (190 mg, 1.34 mmol, 4.00 equiv.) and DIC (208 µL, 169 mg, 1.34 mmol, 4.00 equiv.) in DMF (10 mL) was stirred for 5 minutes at 25 °C and then added to the resin. After 1 h, the coupling mixture was removed via vacuum filtration and the resin was washed five times with DMF (10 mL, 20 mL · g^{-1}). The resin was incubated with 20% piperidine in DMF (v/v, 10 mL, 20 mL · g^{-1}) for 15 min, then the liquid was removed via vacuum filtration and the resin was washed once with DMF (10 mL, 20 mL · g^{-1}). Deprotection was completed by incubation of the resin with 20% piperidine in DMF (v/v, 10 mL, 20 mL · g^{-1}) for 5 minutes and washing five times with DMF (10 mL, 20 mL · g^{-1}). In a 50 mL round bottom flask, equipped with a Teflon-coated magnetic stirring bar, a solution of Fmoc-Trp(Boc)-OH (706 mg, 1.34 mmol, 4.00 equiv.), Oxyma (190 mg, 1.34 mmol, 4.00 equiv.) and DIC (208 µL, 169 mg, 1.34 mmol, 4.00 equiv.) in DMF (10 mL) was stirred for 5 minutes at 25 °C and then added to the resin. After 1 h, the coupling mixture was removed via vacuum filtration and the resin was washed five times with DMF (10 mL, 20 mL · g^{-1}). The resin was incubated with 20% piperidine in DMF (v/v, 10 mL, 20 mL · g^{-1}) for 15 min, then the liquid was removed via vacuum filtration and the resin was washed once with DMF (10 mL, 20 mL · g^{-1}). Deprotection was completed by incubation of the resin with 20% piperidine in DMF (v/v, 10 mL, 20 mL · g^{-1}) for 5 minutes and washing five times with DMF (10 mL, 20 mL · g^{-1}). In a 50 mL round bottom flask, equipped with a Teflon-coated magnetic stirring bar, a solution of Fmoc-Pro-OH (452 mg, 1.34 mmol, 4.00 equiv.), Oxyma (190 mg, 1.34 mmol, 4.00 equiv.) and DIC (208 µL, 169 mg, 1.34 mmol, 4.00 equiv.) in DMF (10 mL) was stirred for 5 minutes at 25 °C and then added to the resin. After 1 h, the coupling mixture was removed via vacuum filtration and the resin was washed five times with DMF (10 mL, 20 mL · g^{-1}). The resin was incubated with 20% piperidine in DMF (v/v, 10 mL, 20 mL · g^{-1}) for 15 min, then the liquid was removed via vacuum filtration and the resin was washed once with DMF (10 mL, 20 mL · g^{-1}). Deprotection was completed by incubation of the resin with 20% piperidine in DMF (v/v, 10 mL, 20 mL · g^{-1}) for 5 minutes and washing five times with DMF (10 mL, 20 mL · g^{-1}).

In a 50 mL round bottom flask, equipped with a Teflon-coated magnetic stirring bar, a solution of Fmoc-Pro-OH (452 mg, 1.34 mmol, 4.00 equiv.), Oxyma (190 mg, 1.34 mmol, 4.00 equiv.) and DIC (208 µL, 169 mg, 1.34 mmol, 4.00 equiv.) in DMF (10 mL) was stirred for 5 minutes at 25 °C and then added to the resin. After 1 h, the coupling mixture was removed via vacuum filtration and the resin was washed five times with DMF (10 mL, 20 mL · g^{-1}). The resin was incubated with 20% piperidine in DMF (v/v, 10 mL, 20 mL · g^{-1}) for 15 min, then the liquid was removed via vacuum filtration and the resin was washed once with DMF (10 mL, 20 mL · g^{-1}). Deprotection was completed by incubation of the resin with 20% piperidine in DMF (v/v, 10 mL, 20 mL · g^{-1}) for 5 minutes and washing five times with DMF (10 mL, 20 mL · g^{-1}). In a 50 mL round bottom flask, equipped with a Teflon-coated magnetic stirring bar, a solution of Fmoc-Ala-OH (417 mg, 1.34 mmol, 4.00 equiv.), Oxyma (190 mg, 1.34 mmol, 4.00 equiv.) and DIC (208 µL, 169 mg, 1.34 mmol, 4.00 equiv.) in DMF (10 mL) was stirred for 5 minutes at 25 °C and then added to the resin. After 1 h, the coupling mixture was removed via vacuum filtration and the resin was washed five times with DMF (10 mL, 20 mL · g^{-1}). The resin was incubated with 20% piperidine in DMF (v/v, 10 mL, 20 mL · g^{-1}) for 15 min, then the liquid was removed via vacuum filtration and the resin was washed once with DMF (10 mL, 20 mL · g^{-1}). Deprotection was completed by incubation of the resin with 20% piperidine in DMF (v/v, 10 mL, 20 mL · g^{-1}) for 5 minutes and washing five times with DMF (10 mL, 20 mL · g^{-1}). In a 50 mL round bottom flask, equipped with a Teflon-coated magnetic stirring bar, a solution of Fmoc-Phe-OH (519 mg, 1.34 mmol, 4.00 equiv.), Oxyma (190 mg, 1.34 mmol, 4.00 equiv.) and DIC (208 µL, 169 mg, 1.34 mmol, 4.00 equiv.) in DMF (10 mL) was stirred for 5 minutes at 25 °C and then added to the resin. After 1 h, the coupling mixture was removed via vacuum filtration and the resin was washed five times with DMF (10 mL, 20 mL · g^{-1}). The resin was incubated with 20% piperidine in DMF (v/v, 10 mL, 20 mL · g^{-1}) for 15 min, then the liquid was removed via vacuum filtration and the resin was washed once with DMF (10 mL, 20 mL · g^{-1}). Deprotection was completed by incubation of the resin with 20% piperidine in DMF (v/v, 10 mL, 20 mL · g^{-1}) for 5 minutes and washing five times with DMF (10 mL, 20 mL · g^{-1}). In a 50 mL round bottom flask, equipped with a Teflon-coated magnetic stirring bar, a solution of Fmoc-Cys(Trt)-OH (785 mg, 1.34 mmol, 4.00 equiv.), Oxyma (190 mg, 1.34 mmol, 4.00 equiv.) and DIC (208 µL, 169 mg, 1.34 mmol, 4.00 equiv.) in DMF (10 mL) was stirred for 5 minutes at 25 °C and then added to the resin. After 1 h, the coupling mixture was removed via vacuum filtration and the resin was washed five times with DMF (10 mL, 20 mL · g^{-1}). The resin was incubated with 20% piperidine in DMF (v/v, 10 mL, 20 mL · g^{-1}) for 15 min, then the liquid was removed via vacuum filtration and the resin was washed once with DMF (10 mL, 20 mL · g^{-1}). Deprotection was completed by incubation of the resin with 20% piperidine in DMF (v/v, 10 mL, 20 mL · g^{-1}) for 5 minutes and washing five times with DMF (10 mL, 20 mL · g^{-1}). In a 50 mL round bottom flask, equipped with a Teflon-coated magnetic stirring bar, a solution of Fmoc-Gln(Trt)-OH (818 mg, 1.34 mmol, 4.00 equiv.), Oxyma (190 mg, 1.34 mmol, 4.00 equiv.) and DIC (208 µL, 169 mg, 1.34 mmol, 4.00 equiv.) in DMF (10 mL) was stirred for 5 minutes at 25 °C and then added to the resin. After 1 h, the coupling mixture was removed via vacuum filtration and the resin was washed five times with DMF (10 mL, 20 mL · g^{-1}). The resin was incubated with 20% piperidine in DMF (v/v, 10 mL, 20 mL · g^{-1}) for 15 min, then the liquid was removed via vacuum filtration and the resin was washed once with DMF (10 mL, 20 mL · g^{-1}). Deprotection was completed by incubation of the resin with 20% piperidine in DMF (v/v, 10 mL, 20 mL · g^{-1}) for 5 minutes and washing five times with DMF (10 mL, 20 mL · g^{-1}). In a 50 mL round bottom flask, equipped with a Teflon-coated magnetic stirring bar, a solution of Fmoc-Leu-OH (474 mg, 1.34 mmol, 4.00 equiv.), Oxyma (190 mg, 1.34 mmol, 4.00 equiv.) and DIC (208 µL, 169 mg, 1.34 mmol, 4.00 equiv.) in DMF (10 mL) was stirred for 5 minutes at

25 °C and then added to the resin. After 1 h, the coupling mixture was removed via vacuum filtration and the resin was washed five times with DMF (10 mL, 20 mL · g^{-1}). The resin was incubated with 20% piperidine in DMF (v/v, 10 mL, 20 mL · g^{-1}) for 15 min, then the liquid was removed via vacuum filtration and the resin was washed once with DMF (10 mL, 20 mL · g^{-1}). Deprotection was completed by incubation of the resin with 20% piperidine in DMF (v/v, 10 mL, 20 mL · g^{-1}) for 5 minutes and washing five times with DMF (10 mL, 20 mL · g^{-1}). In a 50 mL round bottom flask, equipped with a Teflon-coated magnetic stirring bar, a solution of Fmoc-Leu-OH (474 mg, 1.34 mmol, 4.00 equiv.), Oxyma (190 mg, 1.34 mmol, 4.00 equiv.) and DIC (208 µL, 169 mg, 1.34 mmol, 4.00 equiv.) in DMF (10 mL) was stirred for 5 minutes at 25 °C and then added to the resin. After 1 h, the coupling mixture was removed via vacuum filtration and the resin was washed five times with DMF (10 mL, 20 mL · g^{-1}). The resin was incubated with 20% piperidine in DMF (v/v, 10 mL, 20 mL · g^{-1}) for 15 min, then the liquid was removed via vacuum filtration and the resin was washed once with DMF (10 mL, 20 mL · g^{-1}). Deprotection was completed by incubation of the resin with 20% piperidine in DMF (v/v, 10 mL, 20 mL · g^{-1}) for 5 minutes and washing five times with DMF (10 mL, 20 mL · g^{-1}). In a 50 mL round bottom flask, equipped with a Teflon-coated magnetic stirring bar, a solution of Fmoc-Cys(Trt)-OH (785 mg, 1.34 mmol, 4.00 equiv.), Oxyma (190 mg, 1.34 mmol, 4.00 equiv.) and DIC (208 µL, 169 mg, 1.34 mmol, 4.00 equiv.) in DMF (10 mL) was stirred for 5 minutes at 25 °C and then added to the resin. After 1 h, the coupling mixture was removed via vacuum filtration and the resin was washed five times with DMF (10 mL, 20 mL · g^{-1}). The resin was incubated with 20% piperidine in DMF (v/v, 10 mL, 20 mL · g^{-1}) for 15 min, then the liquid was removed via vacuum filtration and the resin was washed once with DMF (10 mL, 20 mL · g^{-1}). Deprotection was completed by incubation of the resin with 20% piperidine in DMF (v/v, 10 mL, 20 mL · g^{-1}) for 5 minutes and washing five times with DMF (10 mL, 20 mL · g^{-1}). In a 50 mL round bottom flask, equipped with a Teflon-coated magnetic stirring bar, a solution of Ac-Ala-OH (176 mg, 1.34 mmol, 4.00 equiv.), Oxyma (190 mg, 1.34 mmol, 4.00 equiv.) and DIC (208 µL, 169 mg, 1.34 mmol, 4.00 equiv.) in DMF (10 mL) was stirred for 5 minutes at 25 °C and then added to the resin. After 1 h, the coupling mixture was removed via vacuum filtration and the resin was washed five times with DMF (10 mL, 20 mL · g^{-1}). The resin was incubated with 20% piperidine in DMF (v/v, 10 mL, 20 mL · g^{-1}) for 15 min, then the liquid was removed via vacuum filtration and the resin was washed once with DMF (10 mL, 20 mL · g^{-1}). Deprotection was completed by incubation of the resin with 20% piperidine in DMF (v/v, 10 mL, 20 mL · g^{-1}) for 5 minutes and washing five times with DMF (10 mL, 20 mL · g^{-1}).
The completed peptide sequence was washed five times with DCM (10 mL, 20 mL · g^{-1}). Then, the resin was incubated with 5 mL of a mixture of 90% TFA + 5% thioanisole + 3% 1,2-ethanedithiol + 2% anisole, (v/v) for 2 h at 25 °C. The mixture was then added dropwise over 10 minutes into 50 mL cold diethyl ether (–20 °C), where immediate precipitation of the peptide was observed. Argon was bubbled through the suspension for 5 minutes and precipitation of the peptide was completed over night at –20 °C. The crude peptide mixture was then obtained by filtration and drying of the precipitate under an argon stream.
Purification was performed by reversed phase HPLC using an YMC Triart C18 column ((150×30mm, 5 µm) with an YMC Triart C18 precolumn (50×30 mm, 5 µm), flow rate = 42,5 mL/min, 35 °C) with an isocratic eluent 40:60 v/v (0.1% v/v TFA in MeCN : 0.1% v/v TFA in H_2O) over 20 min, followed by 95:5 v/v (0.1% v/v TFA in MeCN : 0.1% v/v TFA in H_2O) over 10 minutes. The fractions containing the desired peptide (t ≈ 19.0 min) were collected and lyophilized to afford the title compound **S11** as a colorless powder (180 mg, 0.128 mmol, 38% yield).

NMR Spectroscopy:

^{1}H NMR (600 MHz, $(CD_3)_2SO$, 25°C, δ): 10.82 (d, *J* = 2.4 Hz, 1H), 8.13 (d, *J* = 7.4 Hz, 1H), 8.13 (d, *J* = 7.4 Hz, 1H), 8.06 (d, *J* = 7.9 Hz, 1H), 8.02 (d, *J* = 8.2 Hz, 1H), 7.97 (d, *J* = 7.7 Hz, 1H), 7.97 (d, *J* = 7.7 Hz, 1H) 7.89 (d, *J* = 7.9 Hz, 1H), 7.88 (d, *J* = 7.9 Hz, 1H), 7.76 (d, *J* = 7.6 Hz, 1H), 7.63 (d, *J* = 9.0 Hz, 1H), 7.52 (dq, *J* = 7.9, 1.0 Hz, 1H), 7.31 (dt, *J* = 8.1, 1.0 Hz, 1H), 7.27 (d, *J* = 2.2 Hz, 1H), 7.21 (m, 1H), 7.23 (m, 2H), 7.22 (m, 2H), 7.17 (m, 1H), 7.11 (d, *J* = 2.4 Hz, 1H), 7.05 (ddd, *J* = 8.1, 7.0, 1.2 Hz, 1H), 7.04 (s, 1H), 6.96 (ddd, *J* = 7.9, 7.0, 1.0 Hz, 1H), 6.78 (d, *J* = 1.9 Hz, 1H), 4.51 (m, 1H), 4.48 (quint, *J* = 7.1 Hz, 1H), 4.50 (m, 1H), 4.47 (m, 1H), 4.36 (td, *J* = 7.5, 5.3 Hz, 1H), 4.31 (ddd, *J* = 7.9, 7.5, 5.3 Hz, 1H), 4.29 (m, 1H), 4.27 (m, 1H), 4.24 (p, *J* = 7.1 Hz, 1H), 4.18 (ddd, *J* = 8.3, 7.7, 5.5 Hz, 1H), 4.13 (dd, *J* = 8.9, 6.9 Hz, 1H), 3.60 (dt, *J* = 10.0, 7.2 Hz, 1H), 3.55 (dt, *J* = 10.0, 6.8 Hz, 1H), 3.44 (m, 1H), 3.41 (dt, *J* = 10.0, 6.9 Hz, 1H), 3.14 (dd, *J* = 14.9, 5.2 Hz, 1H), 3.01 (dd, *J* = 14.9, 7.4 Hz, 1H), 3.00 (dd, *J* = 14.2, 4.1 Hz, 1H), 2.78 (m, 1H), 2.76 (dd, *J* = 14.2, 9.6 Hz, 1H), 2.72 (m, 1H), 2.66 (m, 2H), 2.31 (dd, *J* = 8.8, 8.2 Hz, 1H), 2.23 (dd, *J* = 9.3, 7.9 Hz, 1H), 2.07 (t, *J* = 7.8 Hz, 2H), 1.99 (m, 1H), 1.95 (m, 1H), 1.85 (m, 1H), 1.84 (s, 3H), 1.84 (m, 1H), 1.82 (m, 2H), 1.82(m, 1H), 1.76 (m, 1H), 1.74 (m, 1H), 1.68 (dquintd, *J* = 9.0, 6.8, 3.6 Hz, 1H), 1.60 (m, 1H), 1.59 (m, 1H), 1.57 (m, 1H), 1.46 (m, 2H), 1.45 (m, 2H), 1.38 (dqd, *J* = 14.0, 7.4, 3.6 Hz, 1H), 1.18 (d, *J* = 7.1 Hz, 3H), 1.16 (d, *J* = 6.9 Hz, 3H), 1.05 (dqd, *J* = 14.0, 9.0, 7.4 Hz, 1H), 0.87 (d, *J* = 6.6 Hz, 3H), 0.86 (d, *J* = 6.6 Hz, 3H), 0.83 (d, *J* = 6.6 Hz, 3H), 0.81 (d, *J* = 6.6 Hz, 3H), 0.80 (t, *J* = 7.4 Hz, 3H), 0.80 (d, *J* = 6.8 Hz, 3H).

^{13}C NMR (151 MHz, $(CD_3)_2SO$, 25°C, δ): 173.8, 172.7, 172.7, 172.0, 171.9, 171.3, 171.1, 170.9, 170.3, 170.2, 169.7, 169.6, 169.3, 169.3, 137.5, 136.0, 129.2, 128.0, 127.5, 126.3, 123.5, 120.8, 118.3, 118.2, 111.2, 109.7, 59.4, 57.5, 56.7, 54.9, 54.9, 53.7, 53.4, 52.3, 51.3, 51.0, 48.5, 46.6, 46.6, 46.1, 40.5, 40.4, 37.3, 36.8, 31.4, 28.6, 27.7, 27.6, 26.9, 26.2, 25.9, 24.5, 24.3, 24.1, 24.1, 24.0, 23.1, 23.1, 22.4, 21.5, 21.5, 17.9, 16.8, 15.3, 11.1.

HRMS-ESI (m/z) calc'd for $C_{67}H_{100}N_{15}O_{14}S_2^+$ $[M+H]^+$, 1402.701; found, 1402.701; deviation: 0.0 ppm.

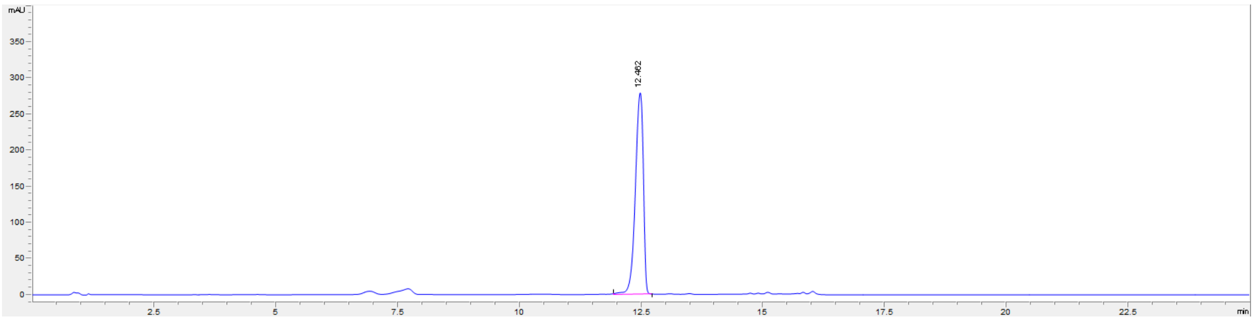

Figure S20. Analytical HPLC Trace of **S11** (YMC Triart C18, 150 × 4.6 mm, 5 µm, flow rate = 1 mL· min^{-1}) with a linear gradient from 65:35 v/v (0.1% v/v TFA in H_2O : MeCN) to 60:40 v/v (0.1% v/v TFA in H_2O : MeCN) over 10 minutes to 40:60 v/v (0.1% v/v TFA in H_2O : MeCN) over 5 minutes.

Ac-Ala-Cys-Leu-Leu-Gln-Lys-Phe-Ala-Pro-Pro-Trp-Ile-NH2 (S12)

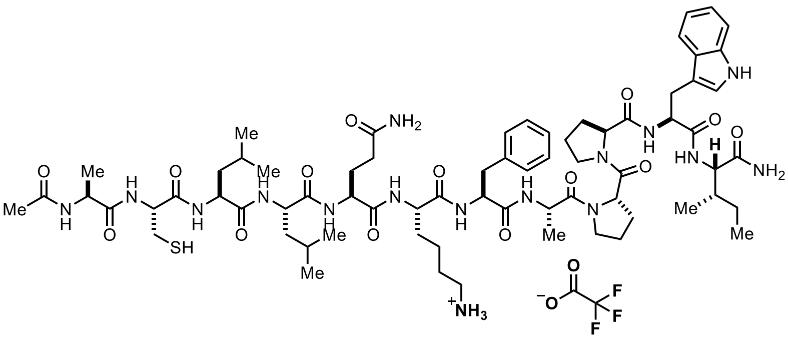

S12 (53%)

All steps were performed under ambient atmosphere at 25 °C. A 50 mL fritted peptide synthesis vessel was charged with Fmoc-Rink-Amide-2CT resin (200-400 mesh, 0.67 mmol · g^{-1} loading, 1% DVB, 0.335 mmol, 500 mg, 1.00 equiv.) and DCM (10 mL, 20 mL · g^{-1}) was added. The suspension was shaken on a Heidolph Vibramax 100 for 30 min, the liquid was removed via vacuum filtration and the resin was washed once with DCM (10 mL, 20 mL · g^{-1}), and three times with DMF (10 mL, 20 mL · g^{-1}). The resin was incubated with 20% piperidine in DMF (v/v, 10 mL, 20 mL · g^{-1}) for 15 min, the liquid was removed via vacuum filtration and the resin was washed with DMF (10 mL, 20 mL · g^{-1}). Deprotection was completed by incubation of the resin with 20% piperidine in DMF (v/v, 10 mL, 20 mL · g^{-1}) for 5 minutes, and after vacuum-filtration, washing five times with DMF (10 mL, 20 mL · g^{-1}).

In a 50 mL round bottom flask, equipped with a Teflon-coated magnetic stirring bar, a solution of Fmoc-Ile-OH (474 mg, 1.34 mmol, 4.00 equiv.), Oxyma (190 mg, 1.34 mmol, 4.00 equiv.) and DIC (208 µL, 169 mg, 1.34 mmol, 4.00 equiv.) in DMF (10 mL) was stirred for 5 minutes at 25 °C and then added to the resin. After 1 h, the coupling mixture was removed via vacuum filtration and the resin was washed five times with DMF (10 mL, 20 mL · g^{-1}). The resin was incubated with 20% piperidine in DMF (v/v, 10 mL, 20 mL · g^{-1}) for 15 min, then the liquid was removed via vacuum filtration and the resin was washed once with DMF (10 mL, 20 mL · g^{-1}). Deprotection was completed by incubation of the resin with 20% piperidine in DMF (v/v, 10 mL, 20 mL · g^{-1}) for 5 minutes and washing five times with DMF (10 mL, 20 mL · g^{-1}). In a 50 mL round bottom flask, equipped with a Teflon-coated magnetic stirring bar, a solution of Fmoc-Trp(Boc)-OH (706 mg, 1.34 mmol, 4.00 equiv.), Oxyma (190 mg, 1.34 mmol, 4.00 equiv.) and DIC (208 µL, 169 mg, 1.34 mmol, 4.00 equiv.) in DMF (10 mL) was stirred for 5 minutes at 25 °C and then added to the resin. After 1 h, the coupling mixture was removed via vacuum filtration and the resin was washed five times with DMF (10 mL, 20 mL · g^{-1}). The resin was incubated with 20% piperidine in DMF (v/v, 10 mL, 20 mL · g^{-1}) for 15 min, then the liquid was removed via vacuum filtration and the resin was washed once with DMF (10 mL, 20 mL · g^{-1}). Deprotection was completed by incubation of the resin with 20% piperidine in DMF (v/v, 10 mL, 20 mL · g^{-1}) for 5 minutes and washing five times with DMF (10 mL, 20 mL · g^{-1}). In a 50 mL round bottom flask, equipped with a Teflon-coated magnetic stirring bar, a solution of Fmoc-Pro-OH (452 mg, 1.34 mmol, 4.00 equiv.), Oxyma (190 mg, 1.34 mmol, 4.00 equiv.) and DIC (208 µL, 169 mg, 1.34 mmol, 4.00 equiv.) in DMF (10 mL) was stirred for 5 minutes at 25 °C and then added to the resin. After 1 h, the coupling mixture was removed via vacuum filtration and the resin was washed five times with DMF (10 mL, 20 mL · g^{-1}). The resin was incubated with 20% piperidine in DMF (v/v, 10 mL, 20 mL · g^{-1}) for 15 min, then the liquid was removed via vacuum filtration and the resin was washed once with DMF (10 mL, 20 mL · g^{-1}). Deprotection was completed by incubation of the resin with 20%

piperidine in DMF (v/v, 10 mL, 20 mL · g^{-1}) for 5 minutes and washing five times with DMF (10 mL, 20 mL · g^{-1}). In a 50 mL round bottom flask, equipped with a Teflon-coated magnetic stirring bar, a solution of Fmoc-Pro-OH (452 mg, 1.34 mmol, 4.00 equiv.), Oxyma (190 mg, 1.34 mmol, 4.00 equiv.) and DIC (208 µL, 169 mg, 1.34 mmol, 4.00 equiv.) in DMF (10 mL) was stirred for 5 minutes at 25 °C and then added to the resin. After 1 h, the coupling mixture was removed via vacuum filtration and the resin was washed five times with DMF (10 mL, 20 mL · g^{-1}). The resin was incubated with 20% piperidine in DMF (v/v, 10 mL, 20 mL · g^{-1}) for 15 min, then the liquid was removed via vacuum filtration and the resin was washed once with DMF (10 mL, 20 mL · g^{-1}). Deprotection was completed by incubation of the resin with 20% piperidine in DMF (v/v, 10 mL, 20 mL · g^{-1}) for 5 minutes and washing five times with DMF (10 mL, 20 mL · g^{-1}). In a 50 mL round bottom flask, equipped with a Teflon-coated magnetic stirring bar, a solution of Fmoc-Ala-OH (417 mg, 1.34 mmol, 4.00 equiv.), Oxyma (190 mg, 1.34 mmol, 4.00 equiv.) and DIC (208 µL, 169 mg, 1.34 mmol, 4.00 equiv.) in DMF (10 mL) was stirred for 5 minutes at 25 °C and then added to the resin. After 1 h, the coupling mixture was removed via vacuum filtration and the resin was washed five times with DMF (10 mL, 20 mL · g^{-1}). The resin was incubated with 20% piperidine in DMF (v/v, 10 mL, 20 mL · g^{-1}) for 15 min, then the liquid was removed via vacuum filtration and the resin was washed once with DMF (10 mL, 20 mL · g^{-1}). Deprotection was completed by incubation of the resin with 20% piperidine in DMF (v/v, 10 mL, 20 mL · g^{-1}) for 5 minutes and washing five times with DMF (10 mL, 20 mL · g^{-1}). In a 50 mL round bottom flask, equipped with a Teflon-coated magnetic stirring bar, a solution of Fmoc-Phe-OH (519 mg, 1.34 mmol, 4.00 equiv.), Oxyma (190 mg, 1.34 mmol, 4.00 equiv.) and DIC (208 µL, 169 mg, 1.34 mmol, 4.00 equiv.) in DMF (10 mL) was stirred for 5 minutes at 25 °C and then added to the resin. After 1 h, the coupling mixture was removed via vacuum filtration and the resin was washed five times with DMF (10 mL, 20 mL · g^{-1}). The resin was incubated with 20% piperidine in DMF (v/v, 10 mL, 20 mL · g^{-1}) for 15 min, then the liquid was removed via vacuum filtration and the resin was washed once with DMF (10 mL, 20 mL · g^{-1}). Deprotection was completed by incubation of the resin with 20% piperidine in DMF (v/v, 10 mL, 20 mL · g^{-1}) for 5 minutes and washing five times with DMF (10 mL, 20 mL · g^{-1}). In a 50 mL round bottom flask, equipped with a Teflon-coated magnetic stirring bar, a solution of Fmoc-Lys(Boc)-OH (628 mg, 1.34 mmol, 4.00 equiv.), Oxyma (190 mg, 1.34 mmol, 4.00 equiv.) and DIC (208 µL, 169 mg, 1.34 mmol, 4.00 equiv.) in DMF (10 mL) was stirred for 5 minutes at 25 °C and then added to the resin. After 1 h, the coupling mixture was removed via vacuum filtration and the resin was washed five times with DMF (10 mL, 20 mL · g^{-1}). The resin was incubated with 20% piperidine in DMF (v/v, 10 mL, 20 mL · g^{-1}) for 15 min, then the liquid was removed via vacuum filtration and the resin was washed once with DMF (10 mL, 20 mL · g^{-1}). Deprotection was completed by incubation of the resin with 20% piperidine in DMF (v/v, 10 mL, 20 mL · g^{-1}) for 5 minutes and washing five times with DMF (10 mL, 20 mL · g^{-1}). In a 50 mL round bottom flask, equipped with a Teflon-coated magnetic stirring bar, a solution of Fmoc-Gln(Trt)-OH (818 mg, 1.34 mmol, 4.00 equiv.), Oxyma (190 mg, 1.34 mmol, 4.00 equiv.) and DIC (208 µL, 169 mg, 1.34 mmol, 4.00 equiv.) in DMF (10 mL) was stirred for 5 minutes at 25 °C and then added to the resin. After 1 h, the coupling mixture was removed via vacuum filtration and the resin was washed five times with DMF (10 mL, 20 mL · g^{-1}). The resin was incubated with 20% piperidine in DMF (v/v, 10 mL, 20 mL · g^{-1}) for 15 min, then the liquid was removed via vacuum filtration and the resin was washed once with DMF (10 mL, 20 mL · g^{-1}). Deprotection was completed by incubation of the resin with 20% piperidine in DMF (v/v, 10 mL, 20 mL · g^{-1}) for 5 minutes and washing five times with DMF (10 mL, 20 mL · g^{-1}). In a 50 mL round bottom flask, equipped with a Teflon-coated magnetic stirring bar, a solution of Fmoc-Leu-OH (474 mg, 1.34 mmol, 4.00 equiv.), Oxyma (190 mg, 1.34 mmol,

4.00 equiv.) and DIC (208 µL, 169 mg, 1.34 mmol, 4.00 equiv.) in DMF (10 mL) was stirred for 5 minutes at 25 °C and then added to the resin. After 1 h, the coupling mixture was removed via vacuum filtration and the resin was washed five times with DMF (10 mL, 20 mL · g^{-1}). The resin was incubated with 20% piperidine in DMF (v/v, 10 mL, 20 mL · g^{-1}) for 15 min, then the liquid was removed via vacuum filtration and the resin was washed once with DMF (10 mL, 20 mL · g^{-1}). Deprotection was completed by incubation of the resin with 20% piperidine in DMF (v/v, 10 mL, 20 mL · g^{-1}) for 5 minutes and washing five times with DMF (10 mL, 20 mL · g^{-1}). In a 50 mL round bottom flask, equipped with a Teflon-coated magnetic stirring bar, a solution of Fmoc-Leu-OH (474 mg, 1.34 mmol, 4.00 equiv.), Oxyma (190 mg, 1.34 mmol, 4.00 equiv.) and DIC (208 µL, 169 mg, 1.34 mmol, 4.00 equiv.) in DMF (10 mL) was stirred for 5 minutes at 25 °C and then added to the resin. After 1 h, the coupling mixture was removed via vacuum filtration and the resin was washed five times with DMF (10 mL, 20 mL · g^{-1}). The resin was incubated with 20% piperidine in DMF (v/v, 10 mL, 20 mL · g^{-1}) for 15 min, then the liquid was removed via vacuum filtration and the resin was washed once with DMF (10 mL, 20 mL · g^{-1}). Deprotection was completed by incubation of the resin with 20% piperidine in DMF (v/v, 10 mL, 20 mL · g^{-1}) for 5 minutes and washing five times with DMF (10 mL, 20 mL · g^{-1}). In a 50 mL round bottom flask, equipped with a Teflon-coated magnetic stirring bar, a solution of Fmoc-Cys(Trt)-OH (785 mg, 1.34 mmol, 4.00 equiv.), Oxyma (190 mg, 1.34 mmol, 4.00 equiv.) and DIC (208 µL, 169 mg, 1.34 mmol, 4.00 equiv.) in DMF (10 mL) was stirred for 5 minutes at 25 °C and then added to the resin. After 1 h, the coupling mixture was removed via vacuum filtration and the resin was washed five times with DMF (10 mL, 20 mL · g^{-1}). The resin was incubated with 20% piperidine in DMF (v/v, 10 mL, 20 mL · g^{-1}) for 15 min, then the liquid was removed via vacuum filtration and the resin was washed once with DMF (10 mL, 20 mL · g^{-1}). Deprotection was completed by incubation of the resin with 20% piperidine in DMF (v/v, 10 mL, 20 mL · g^{-1}) for 5 minutes and washing five times with DMF (10 mL, 20 mL · g^{-1}). In a 50 mL round bottom flask, equipped with a Teflon-coated magnetic stirring bar, a solution of Ac-Ala-OH (176 mg, 1.34 mmol, 4.00 equiv.), Oxyma (190 mg, 1.34 mmol, 4.00 equiv.) and DIC (208 µL, 169 mg, 1.34 mmol, 4.00 equiv.) in DMF (10 mL) was stirred for 5 minutes at 25 °C and then added to the resin. After 1 h, the coupling mixture was removed via vacuum filtration and the resin was washed five times with DMF (10 mL, 20 mL · g^{-1}). The resin was incubated with 20% piperidine in DMF (v/v, 10 mL, 20 mL · g^{-1}) for 15 min, then the liquid was removed via vacuum filtration and the resin was washed once with DMF (10 mL, 20 mL · g^{-1}). Deprotection was completed by incubation of the resin with 20% piperidine in DMF (v/v, 10 mL, 20 mL · g^{-1}) for 5 minutes and washing five times with DMF (10 mL, 20 mL · g^{-1}).

The completed peptide sequence was washed five times with DCM (10 mL, 20 mL · g^{-1}). Then, the resin was incubated with 5 mL of a mixture of 90% TFA + 5% thioanisole + 3% 1,2-ethanedithiol + 2% anisole, (v/v) for 2 h at 25 °C. The mixture was then added dropwise over 10 minutes into 50 mL cold diethyl ether (–20 °C), where immediate precipitation of the peptide was observed. Argon was bubbled through the suspension for 5 minutes and precipitation of the peptide was completed over night at –20 °C. The crude peptide mixture was then obtained by filtration and drying of the precipitate under an argon stream.

Purification was performed by reversed phase HPLC using an YMC Triart C18 column ((150×30mm, 5 µm) with an YMC Triart C18 precolumn (50×30 mm, 5 µm), flow rate = 42 mL/min, 35 °C) with an isocratic eluent 37.5:62.5 v/v (MeCN : 0.1% v/v TFA in H_2O) over 26 min. The fractions containing the desired peptide (t ≈ 10.1 min) were collected and lyophilized to afford the title compound **S12** as a colorless powder (214 mg, 0.139 mmol, 53% yield).

NMR Spectroscopy:

^{1}**H NMR** (600 MHz, DMSO-*d*$_6$, 298 K, δ): 10.81 (d, *J* = 2.4 Hz, 1H), 8.16 (d, *J* = 7.3 Hz, 1H), 8.14 (d, *J* = 6.8 Hz, 1H), 8.07 (d, *J* = 7.8 Hz, 1H), 7.96 (d, *J* = 7.9 Hz, 1H), 7.89 (d, *J* = 7.5 Hz, 1H), 7.86 (d, *J* = 8.1 Hz, 1H), 7.84 (d, *J* = 7.8 Hz, 1H), 7.84 (d, *J* = 8.0 Hz, 1H), 7.76 (d, *J* = 7.6 Hz, 1H), 7.64 (d, *J* = 9.0 Hz, 1H), 7.52 (ddt, *J* = 7.9, 1.1, 0.8 Hz, 1H), 7.31 (dt, *J* = 8.1, 0.9 Hz, 1H), 7.27 (d, *J* = 2.0 Hz, 1H), 7.21 (m, 1H), 7.22 (m, 2H), 7.22 (m, 2H), 7.17 (m, 1H), 7.11 (d, *J* = 2.4 Hz, 1H), 7.05 (ddd, *J* = 8.1, 6.9, 1.2 Hz, 1H), 7.05 (d, *J* = 2.0 Hz, 1H), 6.96 (td, *J* = 7.9, 6.9, 1.0 Hz, 1H), 6.80 (d, *J* = 2.0 Hz, 1H), 4.54 (ddd, *J* = 9.2, 8.2, 4.5 Hz, 1H), 4.51 (td, *J* = 7.5, 5.4 Hz, 1H), 4.47 (dd, *J* = 8.5, 4.7 Hz, 1H), 4.46 (quint, *J* = 7.1 Hz, 1H), 4.36 (td, *J* = 7.5, 5.2 Hz, 1H), 4.29 (dd, *J* = 8.4, 3.0 Hz, 1H), 4.26 (m, 1H), 4.23 (quint, *J* = 7.1 Hz, 1H), 4.15 (m, 1H), 4.14 (m, 1H), 4.13 (dd, *J* = 9.0, 6.9 Hz, 1H), 3.59 (dt, *J* = 10.2, 7.3 Hz, 1H), 3.52 (dt, *J* = 9.6, 6.8 Hz, 1H), 3.45 (m, 1H), 3.41 (dt, *J* = 9.6, 6.7 Hz, 1H), 3.15 (dd, *J* = 15.0, 5.5 Hz, 1H), 3.00 (dd, *J* = 15.1, 7.5 Hz, 1H), 3.00 (dd, *J* = 14.0, 4.6 Hz, 1H), 2.79 (ddd, *J* = 13.7, 9.0, 5.3 Hz, 1H), 2.75 (dd, *J* = 14.0, 9.2 Hz, 1H), 2.72 (ddd, *J* = 13.7, 8.1, 7.5 Hz, 1H), 2.70 (m, 2H), 2.32 (dd, *J* = 8.9, 8.1 Hz, 1H), 2.07 (m, 2H), 2.00 (m, 1H), 1.95 (m, 1H), 1.84 (s, 3H), 1.84 (m, 1H), 1.83 (m, 1H), 1.82 (m, 2H), 1.82 (m, 1H), 1.75 (m, 1H), 1.73 (m, 1H), 1.68 (dquintd, *J* = 9.4, 6.8, 3.7 Hz, 1H), 1.60 (m, 1H), 1.60 (m, 1H), 1.58 (m, 1H), 1.53 (m, 1H), 1.47 (m, 2H), 1.45 (m, 2H), 1.45 (m, 2H), 1.42 (m, 1H), 1.38 (dqd, *J* = 13.7, 7.4, 3.7 Hz, 1H), 1.19 (d, *J* = 7.1 Hz, 3H), 1.19 (m, 2H), 1.16 (d, *J* = 6.9 Hz, 3H), 1.05 (ddq, *J* = 13.7, 9.4, 7.4 Hz, 1H), 0.88 (d, *J* = 6.6 Hz, 3H), 0.87 (d, *J* = 6.6 Hz, 3H), 0.83 (d, *J* = 6.5 Hz, 3H), 0.81 (d, *J* = 6.6 Hz, 3H), 0.80 (d, *J* = 6.8 Hz, 3H), 0.80 (t, *J* = 7.4 Hz, 3H).

^{13}**C NMR** (151 MHz, $(CD_3)_2SO$, 25°C, δ): 173.8, 172.8, 172.7, 172.0, 171.8, 171.3, 171.1, 171.0, 170.9, 170.3, 170.2, 169.7, 169.6, 169.5, 158.1, 157.8 (q, J = 32.0 Hz), 157.6, 157.4, 137.5, 136.0, 129.2, 128.0, 127.5, 126.2, 123.5, 120.8, 118.3, 118.2, 111.2, 109.7, 59.4, 57.4, 56.7, 54.9, 53.4, 53.2, 52.3, 52.3, 51.3, 51.0, 48.6, 46.6, 46.5, 46.1, 40.5, 40.3, 38.7, 37.4, 36.8, 31.4, 31.3, 28.6, 27.7, 27.6, 27.0, 26.5, 25.8, 24.5, 24.3, 24.1, 24.1, 24.0, 23.1, 23.0, 22.4, 22.0, 21.5, 21.5, 17.8, 16.8, 15.3, 11.1.

^{19}**F NMR** (471 MHz, DMF-d$_7$, 298 K, δ): –74.9.

HRMS-ESI (m/z) calc'd for $C_{70}H_{107}N_{16}O_{14}S^{+}$ [M–TFA]$^+$, 1427.787; found, 1427.785; deviation: –1.4 ppm.

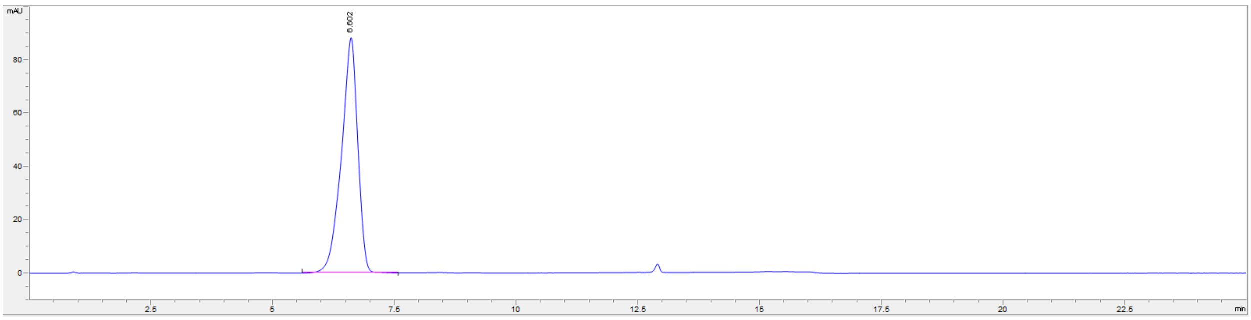

Figure S21. Analytical HPLC Trace of **S12** (YMC Triart C18, 150 × 4.6 mm, 5 µm, flow rate = 1 mL· min^{-1}) with a linear gradient from 65:35 v/v (0.1% v/v TFA in H_2O : MeCN) to 60:40 v/v (0.1% v/v TFA in H_2O : MeCN) over 10 minutes to 40:60 v/v (0.1% v/v TFA in H_2O : MeCN) over 5 minutes.

Synthesis of stapled peptides

Cys-Glu stapling with Ac-Ala-Cys-Leu-Leu-Gln-Glu-Phe-Ala-Pro-Pro-Trp-Ile-NH_2 (22)

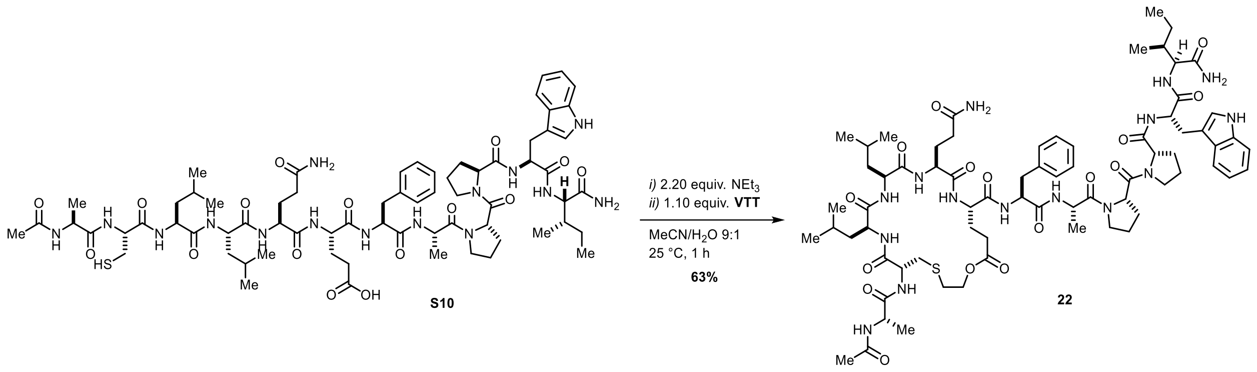

Under ambient atmosphere at 25 °C, a screwcap vial (20 mL) was charged with peptide **S10** (10.0 mg, 7.00 µmol, 1.00 equiv.) and a Teflon-coated magnetic stirring bar. MeCN (4.5 mL) and UHQ-water (500 µL) were added and the resulting solution was stirred for 3 min. Then, NEt_3 (2.15 µL, 1.56 mg, 15.4 µmol, 2.20 equiv.) was added and the mixture was stirred again for 3 min. Next, a 1 M solution of **VTT** in DMF (7.70 µL, 7.70 µmol, 1.10 equiv.) was added and the solution was stirred for 1 h. The reaction mixture was filtered and the filtrate was purified by HPLC using an YMC Triart C18 column ((150×30mm, 5 µm) with an YMC Triart C18 precolumn (50×30 mm, 5 µm), flow rate = 42.5 mL/min, 35 °C) with a linear gradient from 35:65 v/v (MeCN : 0.1% v/v TFA in H_2O) to 50:50 v/v (MeCN : 0.1% v/v TFA in H_2O) over 30 min. The collected fractions containing the product (t ≈ 25.5 min) were collected and lyophilized to afford the title compound **22** as a colorless powder (6.4 mg, 4.4 µmol, 63% yield).

NMR Spectroscopy:

^{1}H NMR (600 MHz, DMSO-d_6, 298 K, δ): 10.87 (d, J = 2.4 Hz, 1H), 8.40 (d, J = 7.9 Hz, 1H), 8.18 (d, J = 5.6 Hz, 1H), 8.17 (d, J = 7.8 Hz, 1H), 8.12 (d, J = 7.1 Hz, 1H), 8.11 (d, J = 7.4 Hz, 1H), 7.83 (d, J = 8.5 Hz, 1H), 7.80 (d, J = 7.6 Hz, 1H), 7.76 (d, J = 8.4 Hz, 1H), 7.68 (d, J = 9.0 Hz, 1H), 7.52 (dq, J = 7.9, 0.9 Hz, 1H), 7.36 (d, J = 8.3 Hz, 1H), 7.31 (dt, J = 8.2, 0.9 Hz, 1H), 7.30 (m, 2H), 7.25 (m, 2H), 7.21 (m, 2H), 7.16 (m, 1H), 7.12 (d, J = 2.4 Hz, 1H), 7.05 (m, 1H), 7.04 (ddd, J = 8.2, 7.0, 1.2 Hz, 1H), 6.95 (ddd, J = 7.9, 7.0, 1.0 Hz, 1H), 6.80 (m, 1H), 4.50 (m, 1H), 4.49 (m, 1H), 4.48 (m, 1H), 4.46 (quint, J = 7.1 Hz, 1H), 4.42 (td, J = 7.9, 6.2 Hz, 1H), 4.29 (dd, J = 8.3, 3.2 Hz, 1H), 4.27 (quint, J = 7.2 Hz, 1H), 4.24 (m, 1H), 4.20 (m, 1H), 4.19 (m, 2H), 4.18 (m, 1H), 4.12 (dd, J = 9.0, 6.9 Hz, 1H), 4.01 (dt, J = 8.0, 6.3 Hz, 1H), 3.60 (m, 1H), 3.56 (dt, J = 9.5, 7.0 Hz, 1H), 3.44 (m, 1H), 3.40 (m, 1H), 3.15 (dd, J = 14.9, 5.2 Hz, 1H), 3.02 (m, 1H), 3.01 (m, 1H), 3.00 (m, 1H), 2.88 (ddd, J = 14.3, 7.6, 4.4 Hz, 1H), 2.77 (m, 1H), 2.76 (m, 1H), 2.74 (m, 1H), 2.28 (ddd, J = 17.0, 10.0, 6.5 Hz, 1H), 2.23 (ddd, J = 17.0, 10.0, 5.8 Hz, 1H), 2.14 (ddd, J = 15.3, 9.2, 6.6 Hz, 1H), 2.08 (ddd, J = 15.3, 9.1, 6.1 Hz, 1H), 2.01 (m, 1H), 1.84 (m, 1H), 1.84 (s, 3H), 1.82 (m, 1H), 1.81 (m, 1H), 1.80 (m, 1H), 1.76 (m, 2H), 1.70 (m, 1H), 1.68 (m, 1H), 1.66 (m, 1H), 1.61 (m, 2H), 1.56 (m, 1H), 1.49 (m, 1H), 1.48 (m, 1H), 1.42 (m, 1H), 1.38 (m, 1H), 1.16 (d, J = 7.0 Hz, 3H), 1.16 (d, J = 7.2 Hz, 3H), 1.05 (m, 1H), 0.87 (d, J = 6.3 Hz, 3H), 0.87 (d, J = 6.7 Hz, 3H), 0.85 (d, J = 6.6 Hz, 3H), 0.80 (d, J = 7.1 Hz, 3H), 0.80 (d, J = 6.9 Hz, 3H), 0.80 (t, J = 7.4 Hz, 3H).

^{13}C NMR (151 MHz, $(CD_3)_2SO$, 25°C, δ): 173.8, 172.8, 172.7, 172.7, 172.4, 171.9, 171.4, 171.4, 170.9, 170.7, 170.5, 170.4, 170.3, 169.7, 169.2, 137.6, 136.0, 129.3, 128.0, 127.4, 126.2, 123.5, 120.8, 118.2, 118.2, 111.2, 109.7, 64.1, 59.4, 57.5, 56.8, 53.6, 53.5, 53.3, 53.0, 52.1, 51.6, 51.1, 48.3, 46.6, 46.6, 46.1, 40.9, 39.7, 37.4, 36.7, 34.2, 31.5, 31.1, 29.5, 28.6, 27.7, 26.9, 26.7, 26.3, 24.5, 24.3, 24.2, 24.1, 24.0, 23.2, 23.1, 22.5, 21.4, 21.0, 18.2, 16.7, 15.3, 11.1.

HRMS-ESI (m/z) calc'd for $C_{71}H_{104}N_{15}O_{16}S^+$ [M+H]$^+$, 1454.750; found, 1454.750; deviation: 0.0 ppm.

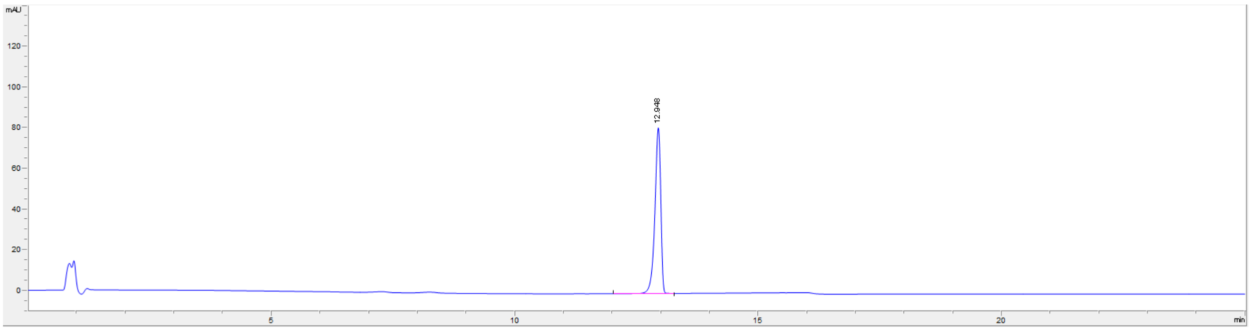

Figure S22. Analytical HPLC Trace of **22** (YMC Triart C18, 150 × 4.6 mm, 5 µm, flow rate = 1 mL· min^{-1}) with a linear gradient from 65:35 v/v (0.1% v/v TFA in H_2O : MeCN) to 60:40 v/v (0.1% v/v TFA in H_2O : MeCN) over 10 minutes to 40:60 v/v (0.1% v/v TFA in H_2O : MeCN) over 5 minutes.

Cys-Cys stapling with Ac-Ala-Cys-Leu-Leu-Gln-Cys-Phe-Ala-Pro-Pro-Trp-Ile-NH$_2$ (23)

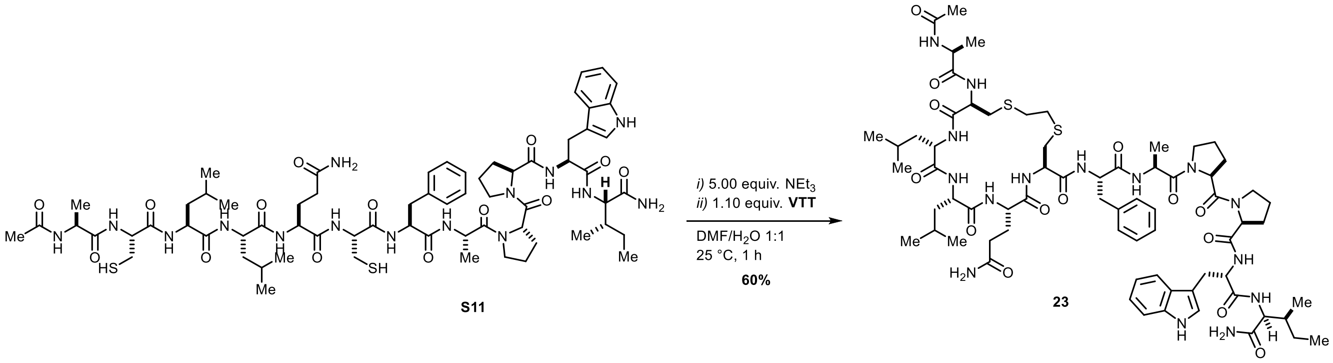

Under ambient atmosphere at 25 °C, a screwcap vial (20 mL) was charged with peptide **S11** (10.0 mg, 7.13 µmol, 1.00 equiv.) and a Teflon-coated magnetic stirring bar. DMF (2.5 mL) and UHQ-water (2.5 mL) were added and the resulting solution was stirred for 3 min. Then, NEt_3 (4.97 µL, 3.61 mg, 35.7 µmol, 5.00 equiv.) was added and the solution was stirred again for 3 min. Next, a 1 M solution of **VTT** in DMF (7.85 µL, 7.85 µmol, 1.10 equiv.) was added and the mixture was stirred for 1 h. A white precipitate formed. The reaction mixture was filtered and the filtrate was purified by HPLC using an YMC Triart C18 column ((150×30mm, 5 µm) with an YMC Triart C18 precolumn (50×30 mm, 5 µm), flow rate = 42.5 mL/min, 35 °C) with an isocratic eluent 60:40 v/v (MeCN : 0.1% v/v TFA in H_2O) over 26 min. The collected fractions containing the product (t ≈ 15.8 min) were collected and lyophilized to afford the title compound **23** as a colorless powder (6.0 mg, 4.3 µmol, 60% yield).

NMR Spectroscopy:

^{1}H NMR (600 MHz, $(CD_3)_2SO$, 25°C, δ): 10.81 (d, J = 2.4 Hz, 1H), 8.25 (d, J = 8.3 Hz, 1H), 8.23 (d, J = 7.8 Hz, 1H), 8.16 (d, J = 8.4 Hz, 1H), 8.11 (d, J = 7.5 Hz, 1H), 8.10 (d, J = 7.0 Hz, 1H), 8.01 (d, J = 8.1 Hz, 1H), 8.01 (d, J = 6.3 Hz, 1H), 7.76 (d, J = 7.5 Hz, 1H), 7.63 (d, J = 9.0 Hz, 1H), 7.52 (d, J = 8.0 Hz, 1H), 7.33 (d, J = 8.6 Hz, 1H), 7.31 (dt, J = 8.0, 0.9 Hz, 1H), 7.27 (m, 1H), 7.25 (m, 1H), 7.23

(m, 2H), 7.20 (m, 2H), 7.16 (m, 1H), 7.11 (d, *J* = 2.4 Hz, 1H), 7.05 (ddd, *J* = 8.0, 6.9, 1.1 Hz, 1H), 7.04 (m, 1H), 6.96 (ddd, *J* = 8.0, 6.9, 0.9 Hz, 1H), 6.81 (m, 1H), 4.51 (m, 1H), 4.50 (m, 1H), 4.47 (m, 1H), 4.47 (m, 1H), 4.46 (m, 1H), 4.37 (ddd, *J* = 8.4, 7.8, 5.7 Hz, 1H), 4.29 (m, 1H), 4.27 (m, 1H), 4.24 (quint, *J* = 7.0 Hz, 1H), 4.12 (dd, *J* = 9.0, 6.9 Hz, 1H), 4.10 (td, *J* = 8.3, 6.5 Hz, 1H), 4.05 (td, *J* = 7.6, 6.3 Hz, 1H), 3.59 (dt, *J* = 9.7, 7.1, 1H), 3.53 (dt, *J* = 9.7, 7.1 Hz, 1H), 3.45 (m, 1H), 3.40 (m, 1H), 3.14 (dd, *J* = 14.9, 5.2 Hz, 1H), 3.08 (td, *J* = 11.5, 5.4 Hz, 1H), 3.04 (dd, *J* = 14.2, 5.3 Hz, 1H), 3.01 (dd, *J* = 14.9, 7.5 Hz, 1H), 2.98 (dd, *J* = 13.7, 4.5 Hz, 1H), 2.85 (ddd, *J* = 13.5, 11.0, 5.3 Hz, 1H), 2.77 (m, 1H), 2.75 (m, 1H), 2.73 (m, 1H), 2.71 (m, 1H), 2.64 (dd, *J* = 13.8, 7.8 Hz, 1H), 2.62 (m, 1H), 2.14 (ddd, *J* = 15.5, 10.0, 6.0 Hz, 1H), 2.07 (ddd, *J* = 15.5, 10.0, 6.0 Hz, 1H), 1.99 (m, 1H), 1.94 (m, 1H), 1.84 (s, 3H), 1.84 (m, 1H), 1.82 (m, 2H), 1.81 (m, 1H), 1.81 (m, 1H), 1.75 (m, 1H), 1.72 (m, 1H), 1.68 (ddqd, *J* = 9.4, 6.9, 6.6, 3.7 Hz, 1H, 1.58 (m, 1H), 1.57 (m, 1H), 1.55 (m, 1H), 1.51 (m, 2H), 1.50 (m, 2H), 1.38 (dqd, *J* = 13.5, 7.5, 3.7 Hz, 1H), 1.17 (d, *J* = 7.0 Hz, 3H), 1.15 (d, *J* = 6.9 Hz, 3H), 1.05 (ddq, *J* = 13.5, 8.4, 7.5 Hz, 1H), 0.88 (d, *J* = 6.5 Hz, 6H), 0.87 (d, *J* = 6.4 Hz, 3H), 0.80 (t, *J* = 7.5 Hz, 3H), 0.80 (d, *J* = 6.6 Hz, 3H), 0.79 (d, *J* = 6.4 Hz, 3H).

^{13}C NMR (151 MHz, $(CD_3)_2SO$, 25°C, δ): 173.9, 173.2, 172.8, 171.8, 171.4, 171.4, 171.1, 170.9, 170.5, 170.3, 170.1, 169.8, 169.6, 169.4, 137.5, 136.0, 129.2, 128.0, 127.5, 126.3, 123.5, 120.9, 118.3, 118.2, 111.2, 109.7, 59.4, 57.5, 56.7, 54.7, 53.6, 53.4, 53.0, 52.9, 52.3, 50.4, 48.5, 46.6, 46.6, 46.1, 41.4, 40.0, 37.4, 36.8, 34.1, 33.1, 32.6, 31.3, 31.3, 28.6, 27.8, 27.0, 26.9, 24.5, 24.4, 24.3, 24.3, 24.1, 23.4, 22.9, 22.5, 21.4, 21.1, 18.2, 16.8, 15.3, 11.1.

HRMS-ESI (m/z) calc'd for $C_{69}H_{102}N_{15}O_{14}S_2^+$ [M+H]$^+$, 1428.717; found, 1428.716; deviation: –0.7 ppm.

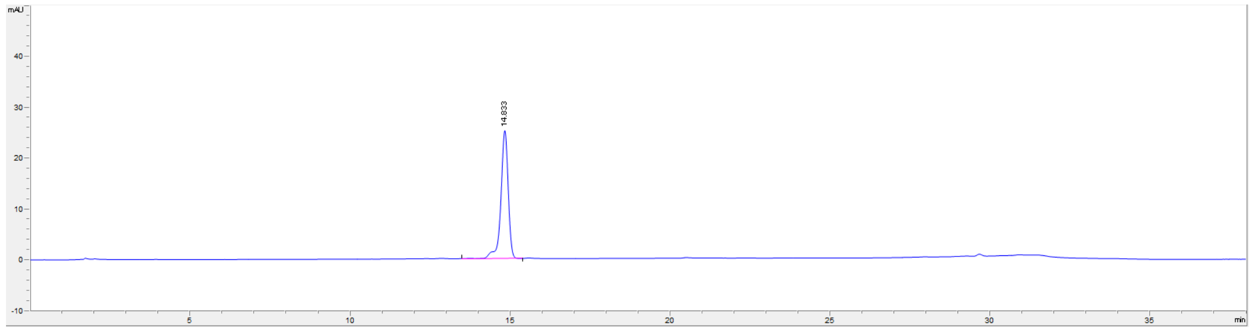

Figure S23. Analytical HPLC Trace of **23** (YMC Triart C18, 150 × 4.6 mm, 5 µm, flow rate = 1 mL· min^{-1}) with a linear gradient from 70:30 v/v (0.1% v/v TFA in H_2O : MeCN) to 40:60 v/v (0.1% v/v TFA in H_2O : MeCN) over 25 minutes to 20:80 v/v (0.1% v/v TFA in H_2O : MeCN) over 5 minutes.

Cys-Lys stapling with Ac-Ala-Cys-Leu-Leu-Gln-Lys-Phe-Ala-Pro-Pro-Trp-Ile-NH_2 (24)

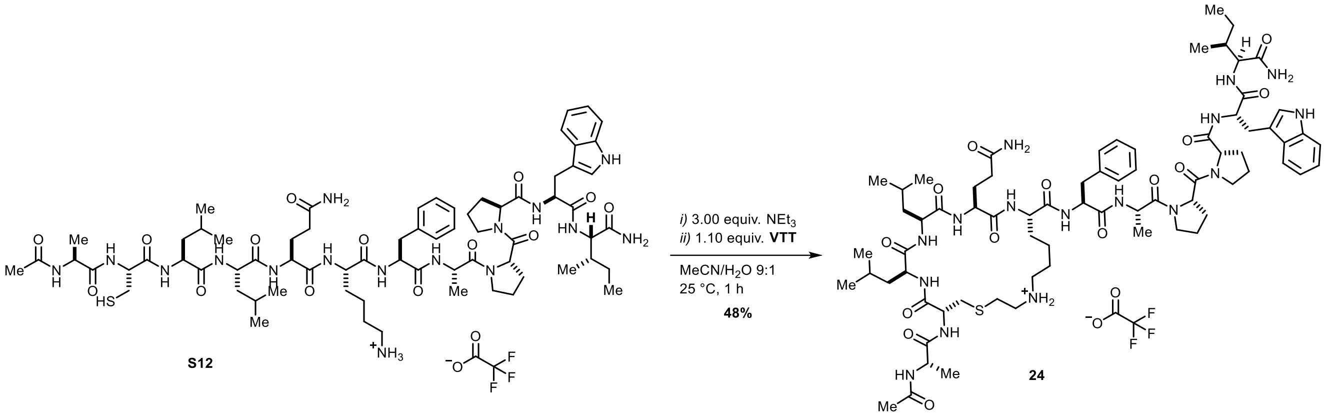

Under ambient atmosphere, a screwcap vial (20 mL) was charged with peptide **S12** (10.0 mg, 6.49 µmol, 1.00 equiv.) and a Teflon-coated magnetic stirring bar. MeCN (4.5 mL) and UHQ-water (500 µL) were added and the resulting solution was stirred for 3 min. Then, NEt_3 (2.71 µL, 1.97 mg, 19.5 µmol, 3.00 equiv.) was added and the mixture was stirred again for 3 min. A 0.5 M solution of **VTT** in DMF (14.3 µL, 7.14 µmol, 1.10 equiv.) was added and the mixture was stirred for 1 h. The reaction mixture was filtered and the filtrate was purified by HPLC using an YMC Triart C18 column ((150×30mm, 5 µm) with an YMC Triart C18 precolumn (50×30 mm, 5 µm), flow rate = 42.5 mL/min, 35 °C) with a linear gradient from 40:60 (MeCN : 0.1% TFA in H_2O, v/v) to 49.5:50.5 (MeCN : 0.1% TFA in H_2O, v/v) over 14 min. The collected fractions containing the product (t ≈ 11.0 min) were collected and lyophilized to afford the title compound **24** as a colorless powder (4.9 mg, 3.1 µmol, 48% yield).

NMR spectroscopy:

^{1}H NMR (600 MHz, $(CD_3)_2SO$, 25°C, δ): 10.82 (d, *J* = 2.4 Hz, 1H), 8.40 (d, *J* = 8.3 Hz, 1H), 8.40 (s, 2H), 8.25 (d, *J* = 7.8 Hz, 1H), 8.19 (d, *J* = 7.3 Hz, 1H), 8.10 (d, *J* = 6.9 Hz, 1H), 8.06 (d, *J* = 7.4 Hz, 1H), 7.94 (d, *J* = 8.5 Hz, 1H), 7.85 (d, *J* = 8.3 Hz, 1H), 7.76 (d, *J* = 7.6 Hz, 1H), 7.64 (d, *J* = 9.0 Hz, 1H), 7.52 (dq, *J* = 7.9, 0.9 Hz, 1H), 7.39 (d, *J* = 8.0 Hz, 1H), 7.31 (dt, *J* = 8.1, 0.9 Hz, 1H), 7.27 (d, *J* = 2.0 Hz, 1H), 7.26 (d, *J* = 2.3 Hz, 1H), 7.23 (m, 2H), 7.22 (m, 2H), 7.17 (m, 1H), 7.11 (d, *J* = 2.4, 1H), 7.05 (ddd, *J* = 8.1, 7.0, 1.0 Hz, 1H), 7.05 (m, 1H), 6.96 (ddd, *J* = 7.9, 7.0, 0.9 Hz, 1H), 6.81 (d, *J* = 2.3 Hz, 1H), 4.54 (ddd, *J* = 9.5, 8.3, 4.5 Hz, 1H), 4.51 (td, *J* = 7.7, 5.5 Hz, 1H), 4.47 (quint, *J* = 7.1 Hz, 1H), 4.47 (dd, *J* = 8.3, 4.8 Hz, 1H), 4.43 (dt, *J* = 7.8, 7.0 Hz, 1H), 4.31 (q, *J* = 7.5 Hz, 1H), 4.29 (dd, *J* = 8.4, 3.1 Hz, 1H), 4.27 (quint, *J* = 7.3 Hz, 1H), 4.23 (m, 1H), 4.22 (m, 1H), 4.13 (dd, *J* = 9.0, 6.9 Hz, 1H), 4.09 (q, *J* = 7.1 Hz, 1H), 3.59 (dt, *J* = 9.5, 7.1 Hz, 1H), 3.53 (dt, *J* = 9.7, 7.1 Hz, 1H), 3.45 (m, 1H), 3.41 (ddd, *J* = 9.9, 7.2, 6.8 Hz, 1H), 3.19 (m, 2H), 3.15 (dd, *J* = 15.0, 5.3 Hz, 1H), 3.01 (dd, *J* = 15.0, 7.7 Hz, 1H), 2.99 (dd, *J* = 13.8, 4.5 Hz, 1H), 2.93 (s, 1H), 2.88 (m, 1H), 2.84 (m, 1H), 2.82 (dd, *J* = 14.0, 7.0 Hz, 1H), 2.79 (m, 1H), 2.73 (dd, *J* = 13.8, 9.5 Hz, 1H), 2.71 (dd, *J* = 14.0, 7.0 Hz, 1H), 2.14 (ddd, *J* = 15.6, 9.6, 6.3 Hz, 1H), 2.07 (ddd, *J* = 15.6, 9.6, 6.0 Hz, 1H), 2.00 (dq, *J* = 12.7, 8.0 Hz, 1H), 1.95 (m, 1H), 1.84 (m, 1H), 1.82 (s, 3H), 1.82 (m, 2H), 1.82 (m, 1H), 1.79 (m, 1H), 1.75 (m, 1H), 1.73 (m, 1H), 1.68 (dquintd, *J* = 9.4, 6.8, 3.7 Hz, 1H), 1.63 (m, 1H), 1.61 (m, 1H), 1.61 (m, 1H), 1.58 (m, 1H), 1.57 (m, 1H), 1.51 (m, 2H), 1.48 (m, 1H), 1.43 (m, 1H), 1.42 (m, 2H), 1.38 (dqd, *J* = 13.5, 7.4, 3.7 Hz, 1H), 1.30 (m, 1H), 1.25 (m, 1H), 1.16 (d, *J* = 6.9 Hz, 3H), 1.14 (d, *J* = 7.1 Hz, 3H), 1.05 (ddq, *J* = 13.5, 9.4, 7.4 Hz, 1H), 0.88 (d, *J* = 6.6 Hz, 3H), 0.88 (d, *J* = 6.4 Hz, 3H), 0.85 (d, *J* = 6.6 Hz, 3H), 0.81 (d, *J* = 6.3 Hz, 3H), 0.80 (t, *J* = 7.4

Hz, 3H), 0.80 (d, *J* = 6.8 Hz, 3H).

^{13}C NMR (151 MHz, $(CD_3)_2SO$, 25°C, δ): 173.7, 172.8, 172.7, 172.2, 171.7, 171.3, 171.2, 171.1, 170.9, 170.3, 170.3, 170.3, 169.7, 169.1, 158.1 (q, J = 31.0 Hz), 137.6, 136.0, 129.2, 128.0, 127.5, 126.3, 123.5, 120.9, 118.3, 118.2, 111.2, 109.7, 59.4, 57.5, 56.7, 53.4, 53.3, 52.6, 52.4, 51.9, 51.5, 50.9, 48.0, 46.6, 46.6, 46.6, 46.1, 45.9, 41.3, 40.2, 37.5, 36.8, 32.6, 31.5, 30.6, 28.6, 27.8, 27.0, 27.0, 26.8, 24.5, 24.3, 24.3, 24.2, 24.2, 24.0, 23.1, 23.1, 22.5, 22.3, 21.6, 21.0, 18.2, 16.8, 15.4, 11.1.

^{19}F NMR (470 MHz, $(CD_3)_2SO$, 298 K, δ): –73.6.

HRMS-ESI (m/z) calc'd for $C_{72}H_{109}N_{16}O_{14}S^+$ [M–TFA]$^+$, 1453.802; found, 1453.801; deviation: –0.7 ppm.

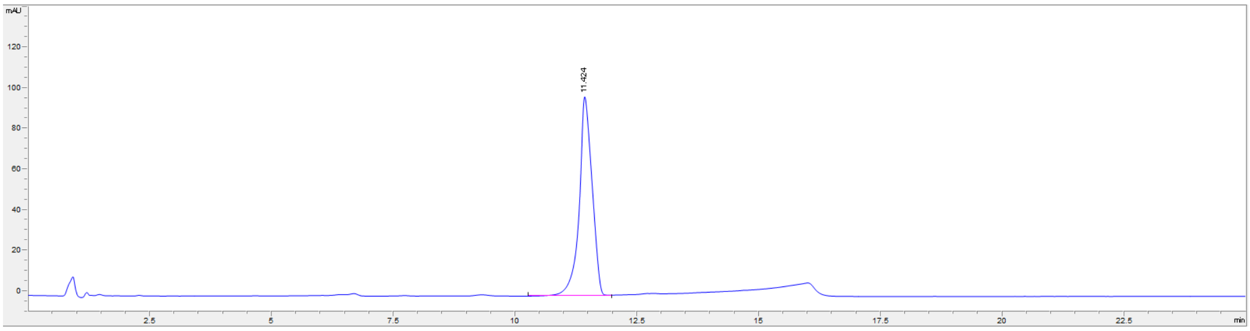

Figure S24. Analytical HPLC Trace of **24** (YMC Triart C18, 150 × 4.6 mm, 5 µm, flow rate = 1 mL· min^{-1}) with a linear gradient from 65:35 v/v (0.1% v/v TFA in H_2O : MeCN) to 60:40 v/v (0.1% v/v TFA in H_2O : MeCN) over 10 minutes to 40:60 v/v (0.1% v/v TFA in H_2O : MeCN) over 5 minutes.

Disulfide rebridging

Preparation of ethylene-bridged Lypressin (25)

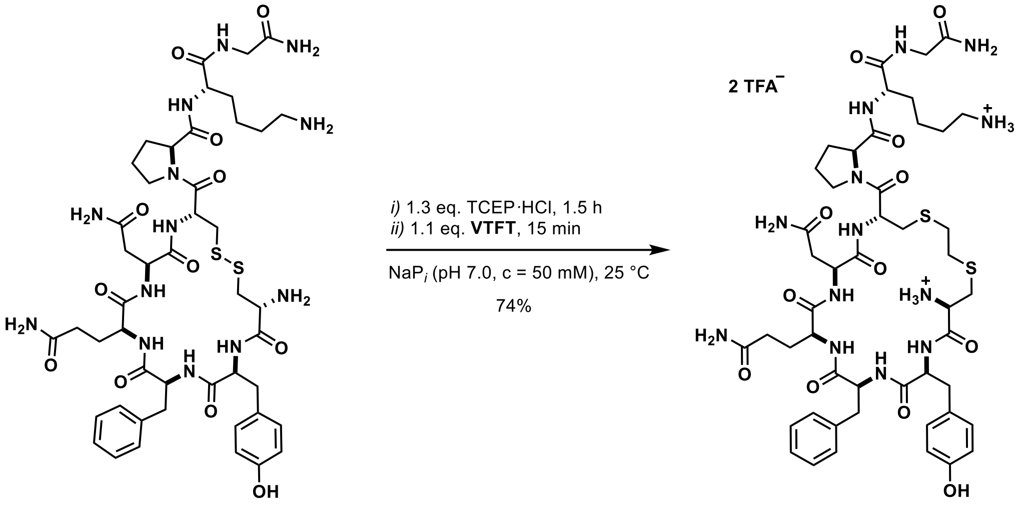

Lypressin **25**

At 20–25 °C, a round-bottom flask (25 mL) was charged with Lypressin (10.5 mg, 9.94 µmol, 1.00 equiv.) and a Teflon-coated magnetic stirring bar. Then, 5.0 mL of sodium phosphate buffer (pH 7.0, c = 100 mM) and 4.1 mL of UHQ-H_2O were added to the flask and the starting material was dissolved while stirring at 300 rpm. Next, a freshly prepared 20 mM solution of TCEP·HCl in UHQ-H_2O (0.65 mL, 13 µmol, 1.3 equiv.) was added to the flask and the mixture was stirred at 300 rpm for 1.5 hours at 25 °C. A 50 mM solution of **VTFT** in DMF (0.21 mL, 10 µmol, 1.1 equiv.) was added dropwise over 1 minute and the mixture was stirred for 15 minutes. The reaction

mixture was filtered, the filtrate was collected, the filter cake was washed with UHQ-H_2O (6 x 5 mL), and the combined filtrate was lyophilized. The obtained residue was purified by HPLC on an YMC Triart C18 column ((150×30mm, 5 µm) with an YMC Triart C18 precolumn (50×30mm, 5 µm), flow rate = 42.5 mL/min, 35°C) with a linear gradient from 10:90 v/v (MeCN : 0.1% v/v TFA in H_2O) to 22:78 v/v (MeCN : 0.1% v/v TFA in H_2O) over 18.0 minutes. The fractions containing the product (t ≈ 16.9 minutes) were collected and lyophilized to afford the title compound as a colorless powder (9.7 mg, 7.4 µmol, 74% yield).

NMR Spectroscopy:

^{1}H NMR (600 MHz, DMF-d_7, 298 K, δ): 9.56 (s, 1H), 9.22 (d, *J* = 8.4 Hz, 1H), 8.85 (d, *J* = 5.1 Hz, 1H), 8.85 (d, *J* = 5.0 Hz, 1H), 8.24 (d, *J* = 8.0 Hz, 1H), 8.03 (d, *J* = 8.5 Hz, 1H), 8.02 (t, *J* = 6.0 Hz, 1H), 7.77 (d, *J* = 8.0 Hz, 1H), 7.73 – 7.71 (m, 1H), 7.53 – 7.51 (m, 1H), 7.36 (s, 1H), 7.35 – 7.29 (m, 4H), 7.26 – 7.23 (m, 1H), 7.14 (d, *J* = 1.6 Hz, 1H), 7.11 (s, 1H), 7.08 – 7.04 (m, 2H), 6.92 (s, 1H), 6.74 – 6.69 (m, 2H), 4.85 (q, *J* = 5.6 Hz, 2H), 4.83 (td, *J* = 8.5, 5.3 Hz, 1H), 4.63 (ddd, *J* = 10.0, 8.3, 5.3 Hz, 1H), 4.51 (dt, *J* = 9.6, 5.3 Hz, 1H), 4.42 (t, *J* = 5.8 Hz, 1H), 4.39 (dd, *J* = 8.5, 4.3 Hz, 1H), 4.33 (td, *J* = 10.0, 4.5 Hz, 1H), 4.11 (dt, *J* = 8.5, 5.0 Hz, 1H), 3.84 (dd, *J* = 16.9, 6.1 Hz, 1H), 3.76 (dd, *J* = 16.9, 5.8 Hz, 1H), 3.75 (t, *J* = 6.8 Hz, 2H), 3.32 (dd, *J* = 14.6, 5.1 Hz, 1H), 3.28 (dd, *J* = 14.2, 5.3 Hz, 1H), 3.17 (dd, *J* = 14.6, 6.5 Hz, 1H), 3.12 (dd, *J* = 14.1, 9.6 Hz, 1H), 3.11 (dd, *J* = 13.9, 5.3 Hz, 1H), 3.06 (dd, *J* = 14.4, 5.6 Hz, 1H), 3.04 (t, *J* = 7.6 Hz, 2H), 2.94 (dd, *J* = 14.4, 7.6 Hz, 1H), 2.88 (dd, *J* = 15.8, 8.0 Hz, 1H), 2.86 – 2.84 (m, 1H), 2.81 (dd, *J* = 14.0, 10.0 Hz, 1H), 2.80 (dd, *J* = 15.8, 5.1 Hz, 2H), 2.79 – 2.78 (m, 2H), 2.40 – 2.34 (m, 2H), 2.17 – 2.11 (m, 1H), 2.09 (dtd, *J* = 14.1, 7.4, 5.0 Hz, 1H), 2.04 – 1.90 (m, 5H), 1.78 – 1.66 (m, 3H), 1.58 – 1.45 (m, 2H).

^{13}C NMR (151 MHz, DMF-d_7, 298 K, δ): 175.2, 173.3, 172.9, 172.6, 172.3, 171.9, 171.7, 169.9, 168.3, 159.6, 138.0, 130.4, 129.6, 128.6, 128.2, 126.8, 117.15 (q, *J* = 300.0 Hz), 115.3, 61.3, 57.1, 56.0, 55.3, 53.3, 52.6, 52.3, 50.4, 47.6, 42.5, 39.9, 37.3, 37.0, 36.6, 33.6, 33.2, 32.8, 32.7, 31.8, 30.8, 26.9, 25.1, 22.8.

^{19}F NMR (471 MHz, DMF-d_7, 298 K, δ): –74.9.

HRMS-ESI (m/z) calc'd for $C_{48}H_{71}N_{13}O_{12}S_2^{2+}$ $[M–2TFA]^{2+}$, 542.7388; found, 542.7391; deviation: +0.6 ppm.

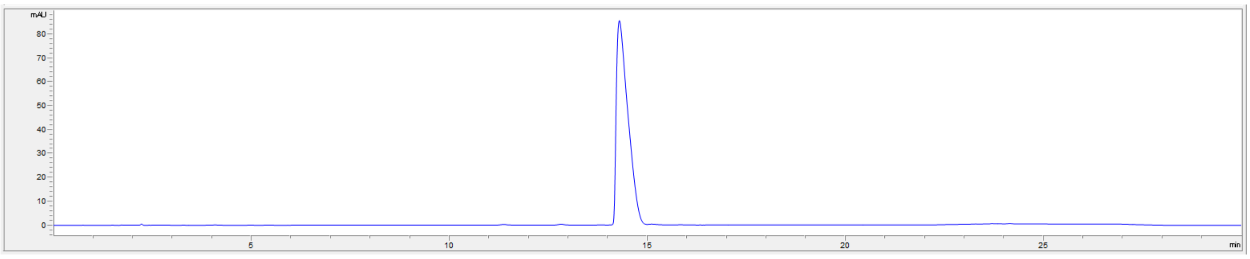

Figure S25. Analytical HPLC trace of **25** (YMC-Triart C18 column, 150 x 4.6 mm, 5 µm, flow rate = 1.0 mL · min^{-1}) with a linear gradient from 10:90 v/v (MeCN : 0.1% v/v TFA in H_2O) to 22:78 v/v (MeCN : 0.1% v/v TFA in H_2O) over 18.0 minutes.

Preparation of ethylene-bridged Octreotide (26)

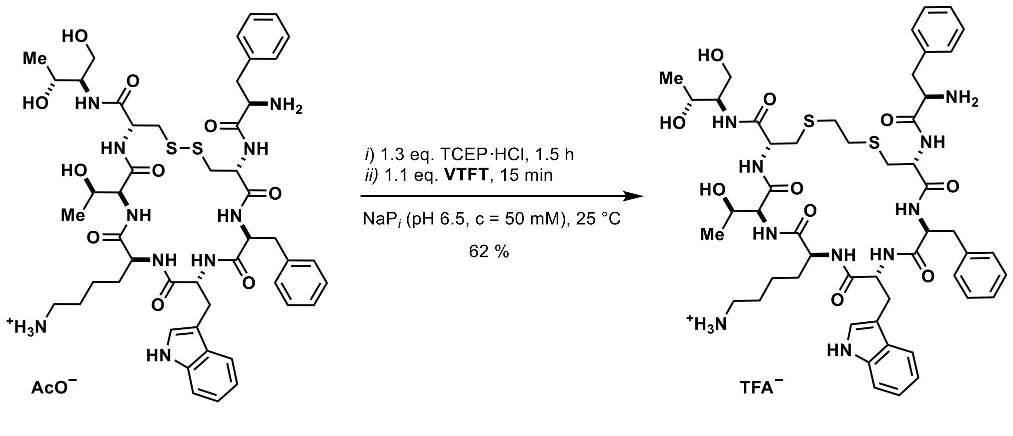

Octreotide 26

At 20–25 °C, a round-bottom flask (25 mL) was charged with Octreotide acetate (11.3 mg, 10.5 µmol, 1.00 equiv.) and a Teflon-coated magnetic stirring bar. Then, 5.2 mL of sodium phosphate buffer (pH 6.5, c = 100 mM) and 4.3 mL of UHQ-H_2O were added to the flask and the starting material was dissolved while stirring at 300 rpm. Next, a freshly prepared 20 mM solution of TCEP·HCl in UHQ-H_2O (0.68 mL, 14 µmol, 1.3 equiv.) was added to the flask and the mixture was stirred at 300 rpm for 1.5 hours at 25 °C. A 50 mM solution of **VTFT** in DMF (0.22 mL, 11 µmol, 1.1 equiv.) was added dropwise over 1 minute and the mixture was stirred for 15 minutes. The reaction mixture was filtered, the filtrate was collected, the filter cake was washed with UHQ-H_2O (6 x 5 mL), and the combined filtrate was lyophilized. The obtained residue was purified by HPLC on an YMC Triart C18 column ((150×30mm, 5 µm) with an YMC Triart C18 precolumn (50×30mm, 5 µm), flow rate = 42.5 mL/min, 35°C) with a linear gradient from 23:77 v/v (MeCN : 0.1% v/v TFA in H_2O) to 27:73 v/v (MeCN : 0.1% v/v TFA in H_2O) over 33.5 minutes. The fractions containing the product (t ≈ 24.8 min) were collected and lyophilized to afford the title compound as a colorless powder (7.6 mg, 6.5 µmol, 62% yield).

NMR Spectroscopy:

^{1}H NMR (600 MHz, DMF-d_7, 298 K, δ): 10.99 (s, 1H), 9.36 (d, *J* = 9.2 Hz, 1H), 9.06 (d, *J* = 4.9 Hz, 1H), 8.86 (d, *J* = 8.9 Hz, 1H), 8.56 (d, *J* = 7.6 Hz, 1H), 8.49 (d, *J* = 9.0 Hz, 1H), 8.11 (d, *J* = 9.3 Hz, 1H), 7.93 (d, *J* = 9.0 Hz, 1H), 7.50 (m, 2H), 7.49 (d, *J* = 8.0 Hz, 1H), 7.44 (d, *J* = 8.1 Hz, 1H), 7.37 (m, 2H), 7.33 (m, 1H), 7.30 (m, 2H), 7.25 (m, 1H), 7.19 (m, 2H), 7.11 (ddd, *J* = 8.2, 7.0, 1.2 Hz, 1H), 7.08 (s, 1H), 7.03 (ddd, *J* = 7.9, 6.9, 1.0 Hz, 1H), 5.19 (dt, *J* = 9.2, 6.5 Hz, 1H), 5.18 (dt, *J* = 8.9, 7.5 Hz, 1H), 5.00 (s, 1H), 4.75 (ddd, *J* = 8.3, 7.6, 6.4 Hz, 1H), 4.71 (dd, *J* = 9.1, 6.0 Hz, 1H), 4.51 (s, 1H), 4.38 (ddd, *J* = 9.2, 6.3, 5.2 Hz, 1H), 3.88 (dddd, *J* = 8.9, 7.8, 5.3, 2.3 Hz, 1H), 3.71 (dd, *J* = 10.7, 7.8 Hz, 1H), 3.62 (dd, *J* = 10.8, 5.3 Hz, 1H), 3.17 (dd, *J* = 14.3, 9.2 Hz, 1H), 1.90 (dddd, *J* = 13.8, 10.0, 6.7, 3.2 Hz, 1H), 1.59 (m, 1H), 1.52 (m, 1H), 1.37 (dtd, *J* = 13.8, 10.5, 4.5 Hz, 1H), 1.22 (d, *J* = 6.4 Hz, 3H), 1.15 (d, *J* = 6.4 Hz, 3H), 0.96 (m, 1H), 0.89 (m, 1H).

^{13}C NMR (151 MHz, DMF-d_7, 298 K, δ): 172.3, 171.9, 171.8, 171.7, 170.6, 170.2, 169.8, 159.5 (br m), 137.6, 137.0, 135.9, 130.1, 129.4, 128.9, 128.6, 127.7, 127.5, 126.9, 124.1, 121.4, 118.7, 118.5, 118.1 (q, J = 296.0 Hz), 111.8, 109.7, 68.3, 65.2, 61.5, 59.4, 56.5, 56.2, 55.3, 55.1, 53.0, 51.8, 51.4, 39.7, 38.8, 38.6, 34.1, 33.6, 30.5, 30.3, 30.0, 27.2, 26.4, 22.7, 19.9, 19.7.

^{19}F NMR (471 MHz, DMF-d_7, 298 K, δ): –74.8.

HRMS-ESI (m/z) calc'd for $C_{51}H_{71}N_{10}O_{10}S_2^+$ [M–TFA]$^+$, 1047.479; found, 1047.479; deviation: 0.0 ppm.

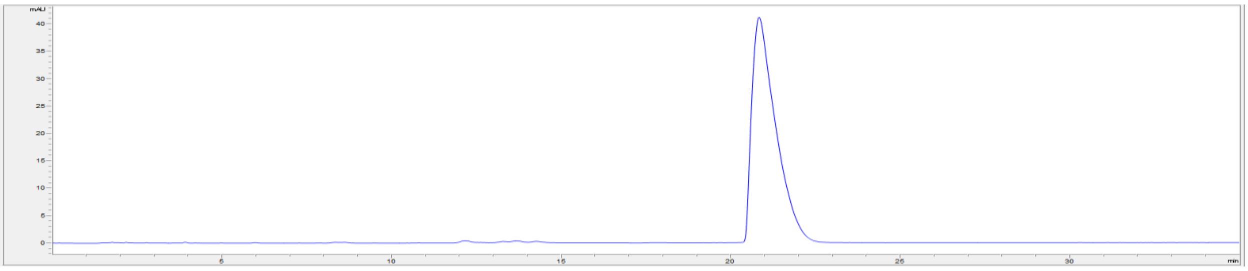

Figure S26. Analytical HPLC trace of **26** (YMC-Triart C18 column, 150 x 4.6 mm, 5 µm, flow rate = 1.0 mL · min^{-1}) with a linear gradient from 23:77 v/v (MeCN : 0.1% v/v TFA in H_2O) to 26:74 v/v (MeCN : 0.1% v/v TFA in H_2O) over 25 minutes.

Preparation of ethylene-bridged Oxytocin (27)

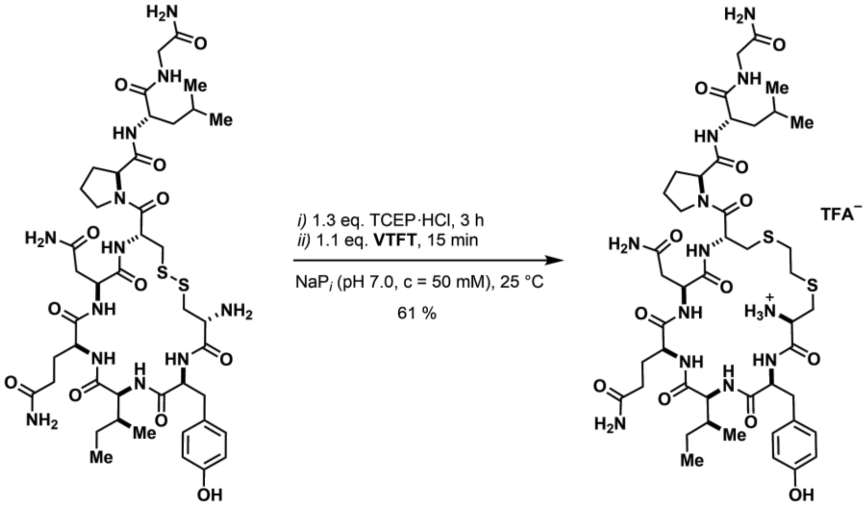

At 20–25 °C, a round-bottom flask (25 mL) was charged with Oxytocin (10.0 mg, 9.97 µmol, 1.00 equiv.) and a Teflon-coated magnetic stirring bar. Then, 5.0 mL of sodium phosphate buffer (pH 7.0, c = 100 mM) and 4.1 mL of UHQ-H_2O were added to the flask and the starting material was dissolved while stirring at 300 rpm. Next, a freshly prepared 20 mM solution of TCEP·HCl in UHQ-H_2O (0.65 mL, 13 µmol, 1.3 equiv.) was added to the flask and the mixture was stirred at 300 rpm for 3 hours at 25 °C. A 50 mM solution of **VTFT** in DMF (0.21 mL, 10 µmol, 1.1 equiv.) was added dropwise over 1 minute and the mixture was stirred for 15 minutes. The reaction mixture was filtered, the filtrate was collected, the filter cake was washed with UHQ-H_2O (6 x 5 mL), and the combined filtrate was lyophilized. The obtained residue was purified by HPLC on an YMC Triart C18 column ((150×30mm, 5 µm) with an YMC Triart C18 precolumn (50×30mm, 5 µm), flow rate = 42.5 mL/min, 35 °C) with a linear gradient from 20:80 v/v (MeCN : 0.1% v/v TFA in H_2O) to 27:73 v/v (MeCN : 0.1% v/v TFA in H_2O) over 10.5 minutes, followed by linear gradient to 60:40 v/v (MeCN : 0.1% v/v TFA in H_2O) over 1.5 minutes. The fractions containing the product (t ≈ 10.4 min) were collected and lyophilized to afford the title compound as a colorless powder (7.0 mg, 6.1 µmol, 61% yield).

NMR Spectroscopy:

^{1}H NMR (600 MHz, DMF-d$_7$, 298 K, δ): 9.43 (d, *J* = 8.1 Hz, 1H), 8.92 (s, 1H), 8.88 (d, *J* = 4.5 Hz, 1H), 8.70 (s, 3H), 8.20 (d, *J* = 8.0 Hz, 1H), 7.98 (t, *J* = 6.5 Hz, 1H), 7.96 (s, 1H), 7.86 (d, *J* = 8.7 Hz, 1H), 7.72 (d, *J* = 8.5 Hz, 1H), 7.62 (d, *J* = 1.7 Hz, 1H), 7.29 (d, *J* = 1.5 Hz, 1H), 7.28 – 7.23 (m, 2H), 7.09 (d, *J* = 1.5 Hz, 1H), 7.07 (d, *J* = 1.7 Hz, 1H), 6.94 (d, *J* = 1.7 Hz, 1H), 6.79 – 6.68 (m, 2H), 4.92 (dt, *J* = 8.5, 6.5 Hz, 1H), 4.91 (td, *J* = 9.5, 4.5 Hz, 1H), 4.79 (ddd, *J* = 11.3, 8.1, 3.4 Hz, 1H), 4.52 (t, *J* = 5.8 Hz, 1H), 4.42 – 4.37 (m, 1H), 4.35 (q, *J* = 7.8 Hz, 1H), 3.87 (dd, *J* = 16.8, 6.5 Hz, 1H), 3.76 – 3.69 (m, 2H), 3.67 (dd, *J* = 16.8, 5.7 Hz, 1H), 3.37 (dd, *J* = 14.2, 3.3 Hz, 1H), 3.32 (dd, *J* = 14.7, 5.1 Hz, 1H), 3.26 (dd, *J* = 14.6, 5.8 Hz, 1H), 3.04 – 2.99 (m, 1H), 3.00 (t, *J* = 14.2 Hz, 2H), 2.99 (dd, *J* = 15.6, 9.5 Hz, 1H), 2.87 (dd, *J* = 15.7, 4.4 Hz, 1H), 2.84 – 2.78 (m, 3H), 2.42 (dt, *J* = 16.0, 6.7 Hz, 1H), 2.37 (dt, *J* = 15.9, 6.7 Hz, 1H), 2.20 – 2.13 (m, 1H), 2.06 – 1.90 (m, 6H), 1.69 – 1.65 (m, 3H), 1.59 (ddd, *J* = 13.7, 7.5, 4.8 Hz, 1H), 1.29 (ddd, *J* = 13.6, 8.6, 7.3 Hz, 1H), 0.99 (d, *J* = 6.9 Hz, 3H), 0.91 (t, *J* = 7.4 Hz, 3H), 0.89 (d, *J* = 6.3 Hz, 3H), 0.86 (d, *J* = 6.2 Hz, 3H).

^{13}C NMR (151 MHz, DMF-d$_7$, 298 K, δ): 175.7, 174.4, 173.0, 172.8, 172.8, 172.3, 171.8, 171.8, 171.8, 170.2, 168.3, 159.3 (q, J = 35.0 Hz), 156.7, 130.6, 128.7, 117.0 (q, J = 293.0 Hz), 115.2, 61.8, 60.9, 55.9, 55.7, 52.4, 52.3, 52.1, 50.7, 47.6, 42.7, 39.9, 37.5, 36.3, 36.1, 33.9, 33.2, 33.0, 32.5, 31.8, 29.4, 26.3, 26.0, 25.1, 25.0, 23.1, 21.0, 15.6, 11.4.

^{19}F NMR (471 MHz, DMF-d$_7$, 298 K, δ): –74.7.

HRMS-ESI (m/z) calc'd for $C_{45}H_{71}N_{12}O_{12}S_2^+$ [M–TFA]$^+$, 1035.475; found, 1035.475; deviation: 0.0 ppm.

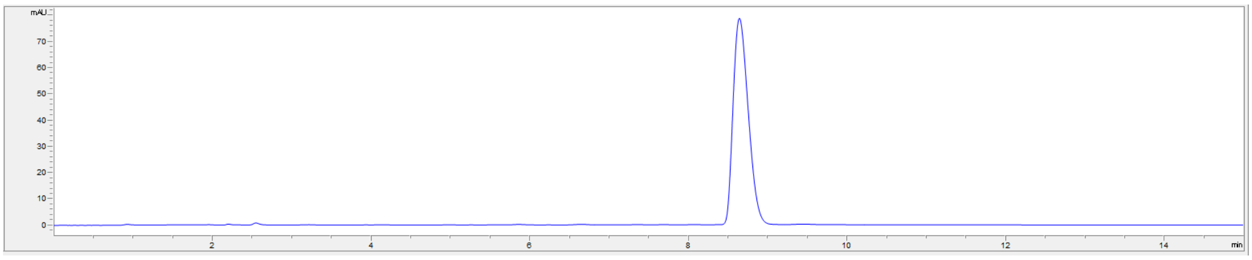

Figure S27. Analytical HPLC trace of **27** (YMC-Triart C18 column, 150 x 4.6 mm, 5 µm, flow rate = 1.0 mL · min^{-1}) with a linear gradient from 20:80 v/v (MeCN : 0.1% v/v TFA in H_2O) to 27:73 v/v (MeCN : 0.1% v/v TFA in H_2O) over 10.0 minutes.

Functionalization of proteins

Quantification of conversion for protein modifications

Protein substrate scope

Determination of conversion and yield for the protein substrate scope was either performed via peak integration after PDA analysis at 214 nm or based on peak areas of reconstructed zero-charge spectra after deconvolution. PDA analysis was used in all the cases where protein peak separation was achieved via HPLC. If no separation was achieved, the yield was determined via peak areas obtained from the zero-charge spectra after deconvolution. In case of zero-charge analysis, only product peaks resulting from the major peak present in the starting material were considered. Minor peak reactivity was reported but not considered for yield determination because side reactivities might go undetected due to the lower overall signal intensity. The determined yields should be considered semi-quantitative due to unknown extinction coefficients and altered ionization properties of newly formed species. Additionally, potential instability of introduced functional groups during the measurement needs to be considered. The conversion and yield are calculated as follows:

$$conversion = \frac{sum\ of the\ peak\ areas\ of\ all\ formed\ products}{sum\ of\ the\ peak\ areas\ of\ all\ formed\ products\ and\ the\ starting\ material}$$

$$yield = \frac{peak\ area\ of\ the\ desired\ product}{sum\ of\ the\ peak\ areas\ of\ all\ formed\ products\ and\ the\ starting\ material}$$

In case of BSA, MDAR2, and MDAR3 quantitation was not performed due to low resolution of the protein peaks in the respective ion series. However, LC-MS analysis provides the information that the desired modification is the major species in the mixture and additional SDS-PAGE analysis was performed to further verify the synthesis of the desired conjugate. Additional data on the quantification of the protein modifications reactions is provided in the corresponding sections. MDAR2 was included in the SI because it represents another example of a native protein which was modified even in the presence of other proteins that could not be removed during the purification process. The protein was not added to the substrate scope of the manuscript because the starting material was not considered pure enough.

Functional group scope

Determination of conversion and yield for the protein functional group scope was performed via integration of peak areas of reconstructed zero-charge spectra after deconvolution. The approach was verified via comparison of different methods (see Figure S91 to Figure S93). The determined yields should be considered semi-quantitative due to unknown extinction coefficients and altered ionization properties of newly formed species. Additionally, potential instability of introduced functional groups during the measurement needs to be considered. The conversion and yield are calculated as follows:

$$conversion = \frac{sum\ of the\ peak\ areas\ of\ all\ formed\ products}{sum\ of\ the\ peak\ areas\ of\ all\ formed\ products\ and\ the\ starting\ material}$$

$$yield = \frac{peak\ area\ of\ the\ desired\ product}{sum\ of\ the\ peak\ areas\ of\ all\ formed\ products\ and\ the\ starting\ material}$$

Data on protein starting materials

The LC-MS measurements were performed according to the methods described in the *materials and methods*

section. The average isotopic masses of the proteins were calculated based on their amino acid sequence. Because of the expression in *E.coli*, the cleavage of the N-terminal Methionine and its acetylation were considered in all the cases[9].

For some of the proteins (MDAR2, MDAR3, sfGFP S147C, DHAR2 C6S, BSA) no absorption spectrum was recorded, due to the observed formation of decomposition products in MS after UV-Vis measurements (See Figure S107 and Figure S108). For these proteins, only MS analysis is given.

Small peaks detected in LC-MS originating from impurities in the starting materials were not further evaluated.

* in the amino acid sequences indicates the end of the sequence.

DHAR1

Amino acid-sequence DHAR1:

MGSSHHHHHHSSGLVPRGSHMASMALEICVKAAVGAPDHLGDCPFSQRALLTLEEKSLTYKIHLINLSDKPQWF
LDISPQGKVPVLKIDDKWVTDSDVIVGILEEKYPDPPLKTPAEFASVGSNIFGTFGTFLKSKDSNDGSEHALLVEL
EALENHLKSHDGPFIAGERVSAVDLSLAPKLYHLQVALGHFKSWSVPESFPHVHNYMKTLFSLDSFEKTKTEEK
YVISGWAPKVNP*

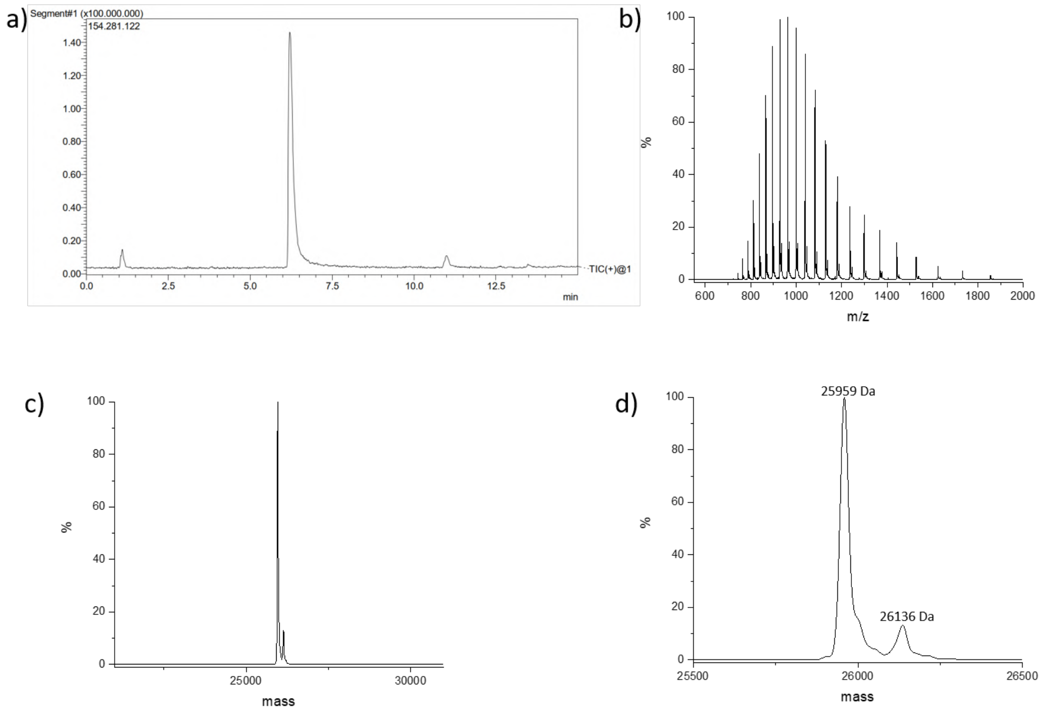

Figure S28. LC-MS analysis (Method A) of DHAR1 starting material. Sample measured after reduction with 10 equiv. TCEP for 1 h at 37 °C. Protein concentration for analytical sample: 0.14 mg/mL. a) total ion chromatogram b) ion series c) deconvoluted spectrum d) zoom on the major peaks; Calculated masses: DHAR1: 25963, DHAR1 with N-terminal Ac-Met: 26136 Da. Observed masses: 25959 Da, 26136 Da.

DHAR1 S97C

Amino acid-sequence DHAR1 S97C:

MGSSHHHHHHSSGLVPRGSHMASMALEICVKAAVGAPDHLGDCPFSQRALLTLEEKSLTYKIHLINLSDKPQWF

LDISPQGKVPVLKIDDKWVTDSDVIVGILEEKYPDPPLKTPAEFACVGSNIFGTFGTFLKSKDSNDGSEHALLVEL
EALENHLKSHDGPFIAGERVSAVDLSLAPKLYHLQVALGHFKSWSVPESFPHVHNYMKTLFSLDSFEKTKTEEK
YVISGWAPKVNP*

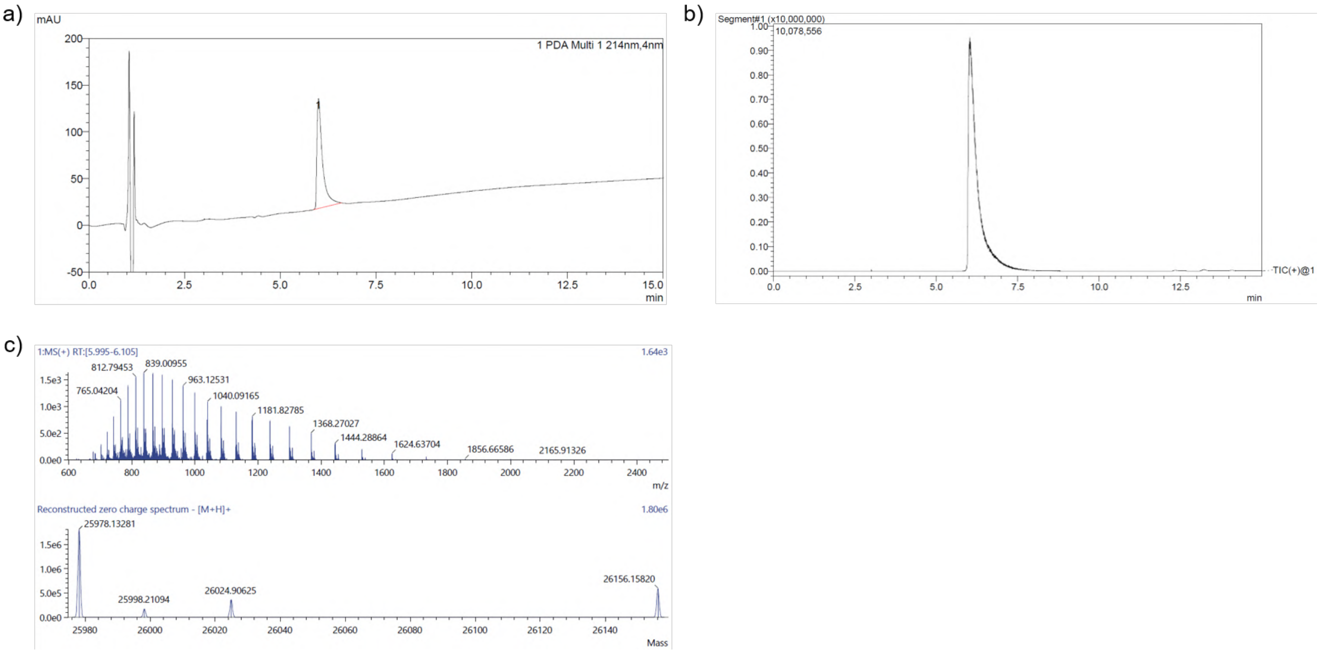

Figure S29. LC-MS analysis (Method B) of DHAR1 S97C starting material. Sample measured after reduction with 10 equiv. TCEP for 1 h at 37 °C. Protein concentration for analytical sample: 0.14 mg/mL. a) UV-trace b) total ion chromatogram c) Ion series and deconvoluted spectrum; Calculated mass: DHAR1 S97C: 25979 Da. Observed mass: 25978 Da.

DHAR1 S176C

Amino acid-sequence DHAR1 S176C:

MGSSHHHHHHSSGLVPRGSHMASMALEICVKAAVGAPDHLGDCPFSQRALLTLEEKSLTYKIHLINLSDKPQWF
LDISPQGKVPVLKIDDKWVTDSDVIVGILEEKYPDPPLKTPAEFASVGSNIFGTFGTFLKSKDSNDGSEHALLVEL
EALENHLKSHDGPFIAGERVSAVDLSLAPKLYHLQVALGHFKSWSVPECFPHVHNYMKTLFSLDSFEKTKTEEK
YVISGWAPKVNP*

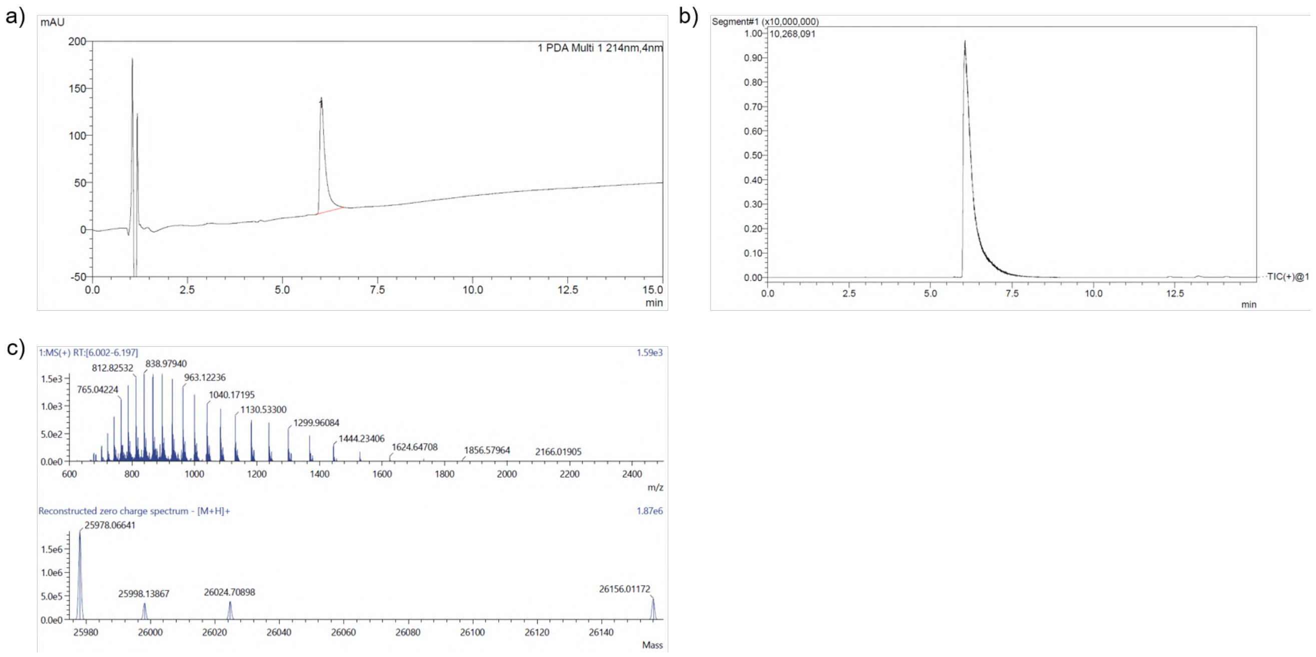

Figure S30. LC-MS analysis (Method B) of DHAR1 S176C starting material. Sample measured after reduction with 10 equiv. TCEP for 1 h at 37 °C. Protein concentration for analytical sample: 0.14 mg/mL. a) UV-trace b) total ion chromatogram c) Ion series and deconvoluted spectrum; Calculated mass: DHAR1 S176C: 25979 Da. Observed mass: 25978 Da.

sfGFP S147C

Amino acid-sequence sfGFP S147C:

MGSSHHHHHHSSGLVPRGSHMASMRKGEELFTGVVPILVELDGDVNGHKFSVRGEGEGDATNGKLTLKFICTT
GKLPVPWPTLVTTLTYGVQCFARYPDHMKQHDFFKSAMPEGYVQERTISFKDDGTYKTRAEVKFEGDTLVNRI
ELKGIDFKEDGNILGHKLEYNFNCHNVYITADKQKNGIKANFKIRHNVEDGSVQLADHYQQNTPIGDGPVLLPDN
HYLSTQSVLSKDPNEKRDHMVLLEFVTAAGITHGMDELYK*

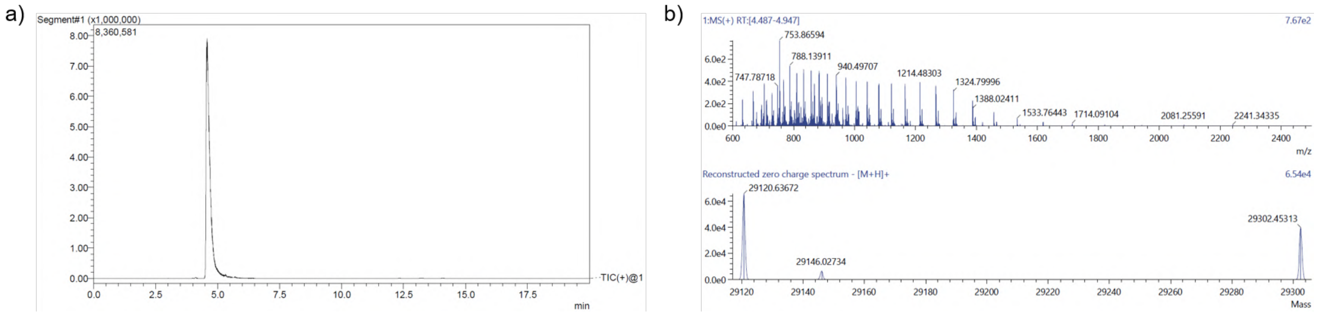

Figure S31. LC-MS analysis (Method B) of sfGFP S147C starting material. Sample measured after reduction with 10 equiv. TCEP for 1 h at 37 °C. Protein concentration for analytical sample: 0.14 mg/mL. a) total ion chromatogram b) Ion series and deconvoluted spectrum; Calculated masses: sfGFP S147C: 29144 Da, sfGFP S147C (matured, $-H_2O$, $-H_2$)[10]: 29124 Da, sfGFP S147C (matured, $-H_2O$, $-H_2$) with N-terminal Ac-Met: 29297 Da. Observed masses: 29121 Da, 29146 Da, 29302 Da.

Ubiquitin T9C

Amino acid-sequence Ubiquitin T9C:

GSHMASQIFVKTLCGKTITLEVEPSDTIENVKAKIQDKEGIPPDQQRLIFAGKQLEDGRTLSDYNIQKESTLHLVLR
LRGG*

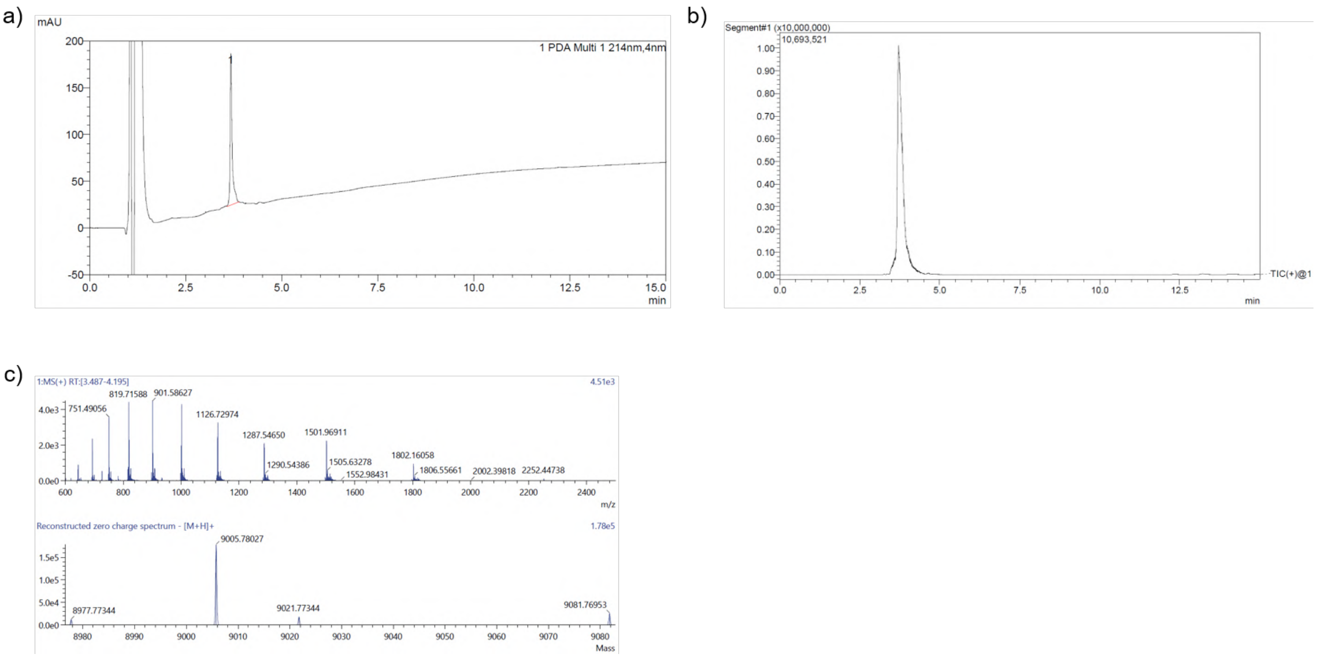

Figure S32. LC-MS analysis (Method B) of Ubiquitin T9C starting material. Sample measured after reduction with 10 equiv. TCEP for 1 h at 37 °C. Protein concentration for analytical sample: 0.14 mg/mL. a) UV-trace b) total ion chromatogram c) Ion series and deconvoluted spectrum; Calculated mass: Ubiquitin T9C: 9006 Da. Observed mass: 9006 Da.

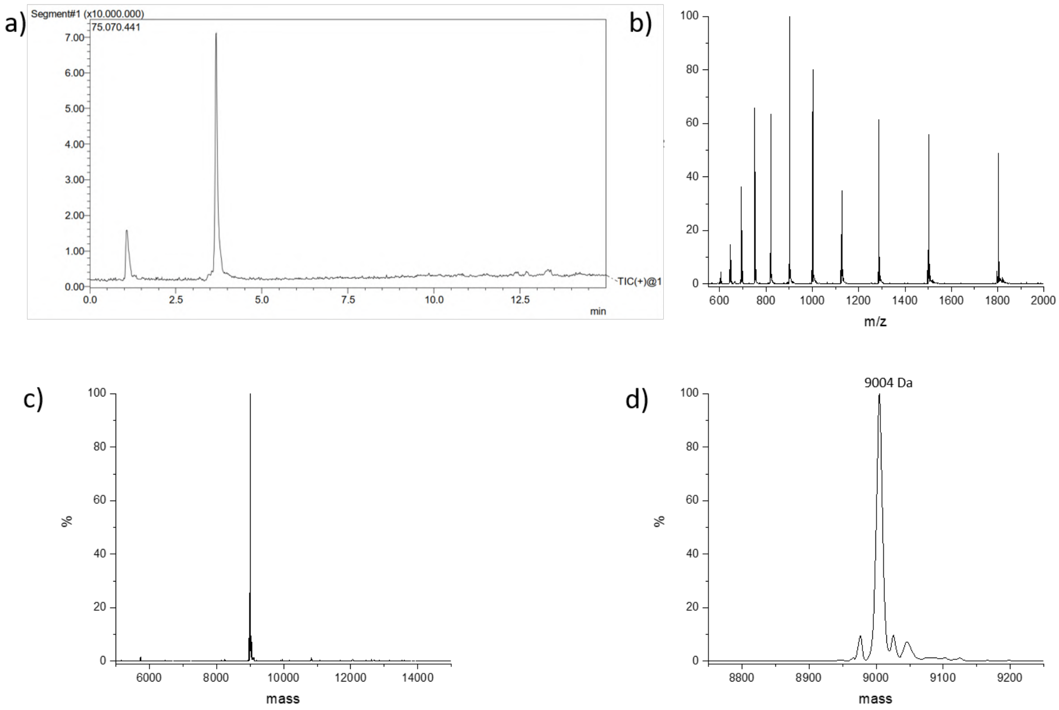

Figure S33. LC-MS analysis (Method A) of Ubiquitin T9C starting material. Sample measured after reduction with 10 equiv. TCEP for 1 h at 37 °C. Protein concentration for analytical sample: 0.14 mg/mL. a) total ion chromatogram b) Ion series c) deconvoluted spectrum d) zoom on the major peaks; Calculated mass: Ubiquitin T9C: 9006 Da. Observed mass: 9004 Da.

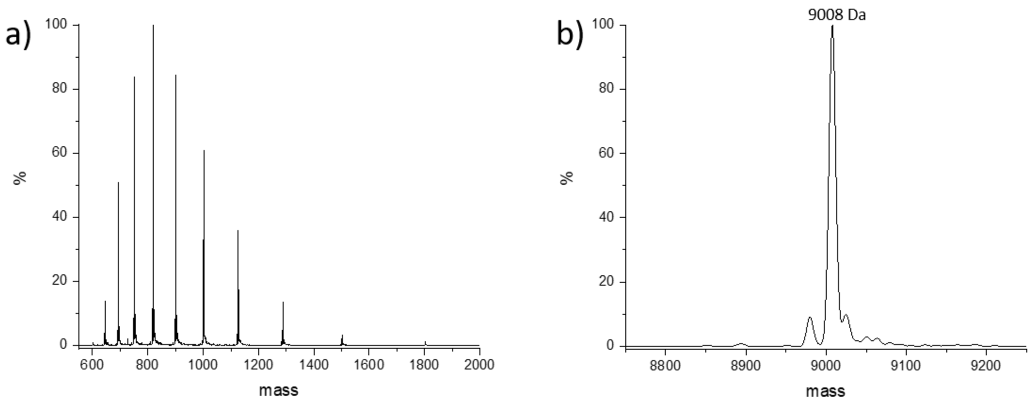

Figure S34. LC-MS analysis (Method E) of Ubiquitin T9C starting material. Sample measured after reduction with 10 equiv. TCEP for 1 h at 37 °C. Protein concentration for analytical sample: 0.14 mg/mL. a) deconvoluted spectrum b) zoom on the major peaks; Calculated mass: Ubiquitin T9C: 9006 Da. Observed mass: 9008 Da.

Ubiquitin T12C

Amino acid-sequence Ubiquitin T12C:

GSHMASQIFVKTLTGKCITLEVEPSDTIENVKAKIQDKEGIPPDQQRLIFAGKQLEDGRTLSDYNIQKESTLHLVLR LRGG*

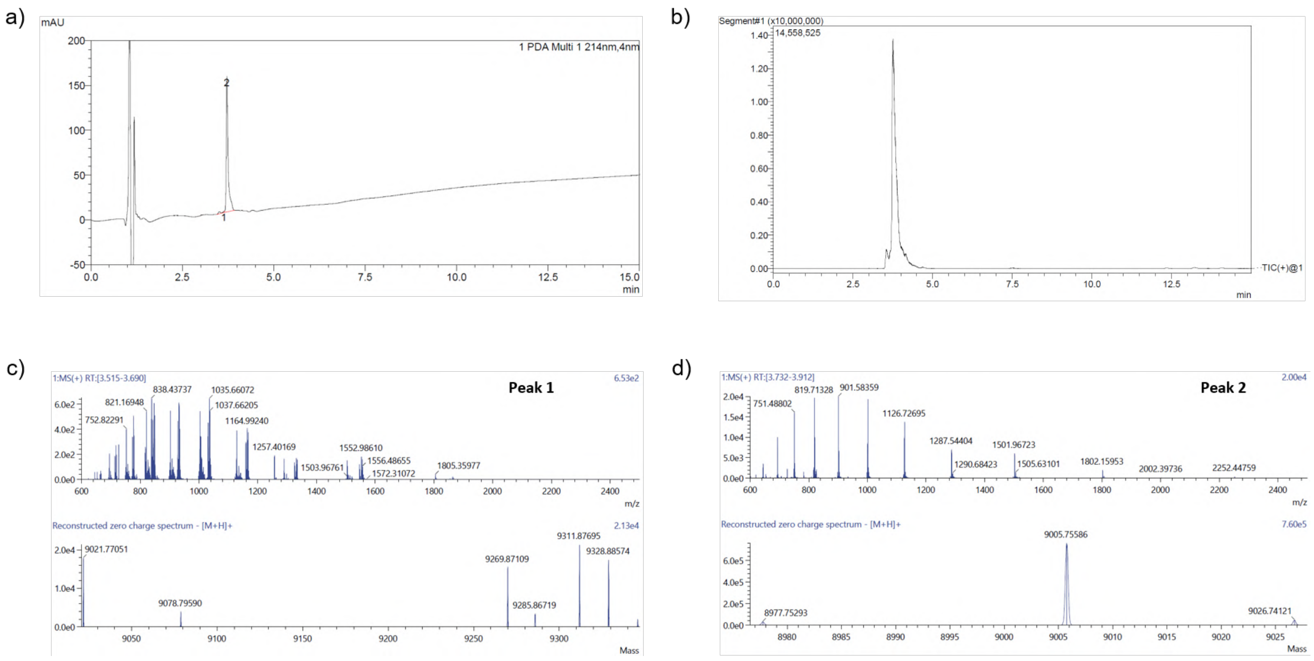

Figure S35. LC-MS analysis (Method B) of Ubiquitin T12C starting material. Sample measured after reduction with 10 equiv. TCEP for 1 h at 37 °C. Protein concentration for analytical sample: 0.14 mg/mL. a) UV-trace b) total ion chromatogram c) Peak 1 ion series and deconvoluted spectrum d) Peak 2 ion series and deconvoluted spectrum; Calculated mass: Ubiquitin T12C: 9006 Da. Observed mass: 9006 Da.

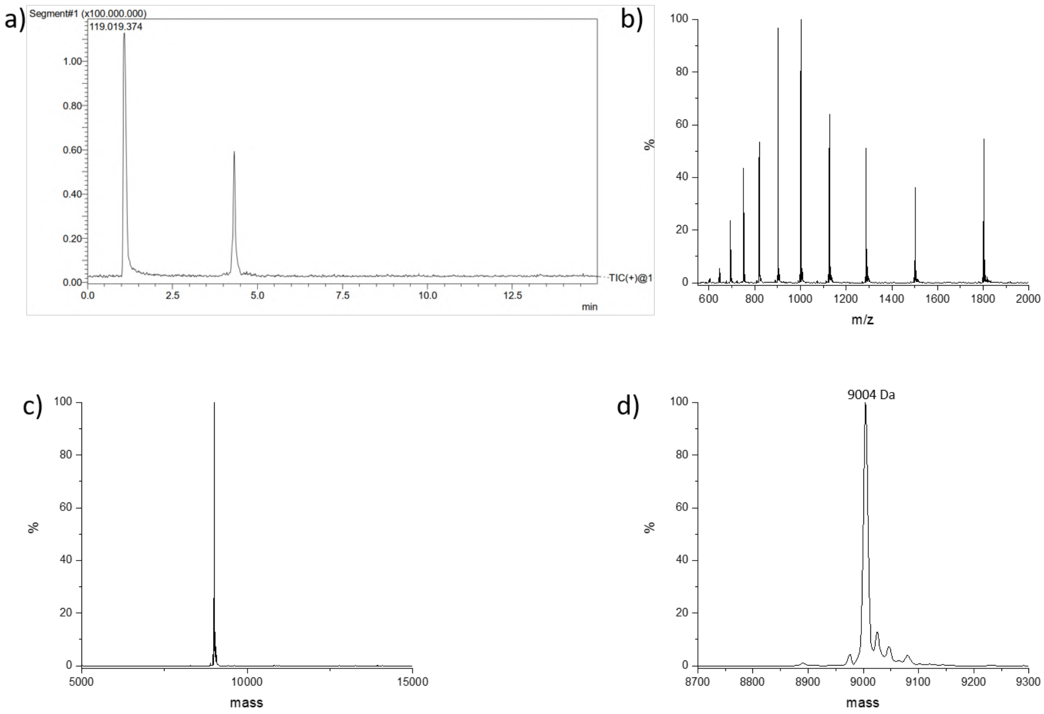

Figure S36. LC-MS analysis (Method A) of Ubiquitin T12C starting material. Sample measured after reduction with 10 equiv. TCEP for 1 h at 37 °C. Protein concentration for analytical sample: 0.14 mg/mL. a) total ion chromatogram b) Ion series c) deconvoluted spectrum d) zoom on the major peaks; Calculated mass: Ubiquitin T9C: 9006 Da. Observed mass: 9004 Da.

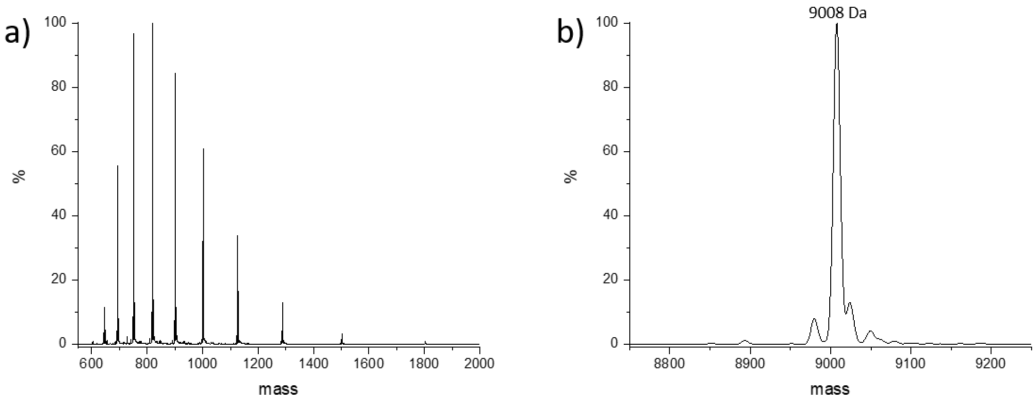

Figure S37. LC-MS analysis (Method E) of Ubiquitin T12C starting material. Sample measured after reduction with 10 equiv. TCEP for 1 h at 37 °C. Protein concentration for analytical sample: 0.14 mg/mL. a) deconvoluted spectrum b) zoom on the major peaks; Calculated mass: Ubiquitin T9C: 9006 Da. Observed mass: 9008 Da.

DHAR2 C6S

Amino acid-sequence DHAR2 C6S:

MGSSHHHHHHSSGLVPRGSHMASMALDISVKVAVGAPDVLGDCPFSQRVLLTLEEKKLPYKTHLINVSDKPQW

FLDISPEGKVPVVKLDGKWVADSDVIVGLLEEKYPEPSLKTPPEFASVGSKIFGAFVTFLKSKDANDGSEKALVD

ELEALENHLKTHSGPFVAGEKITAVDLSLAPKLYHLEVALGHYKNWSVPESLTSVRNYAKALFSRESFENTKAKK EIVVAGWESKVNA*

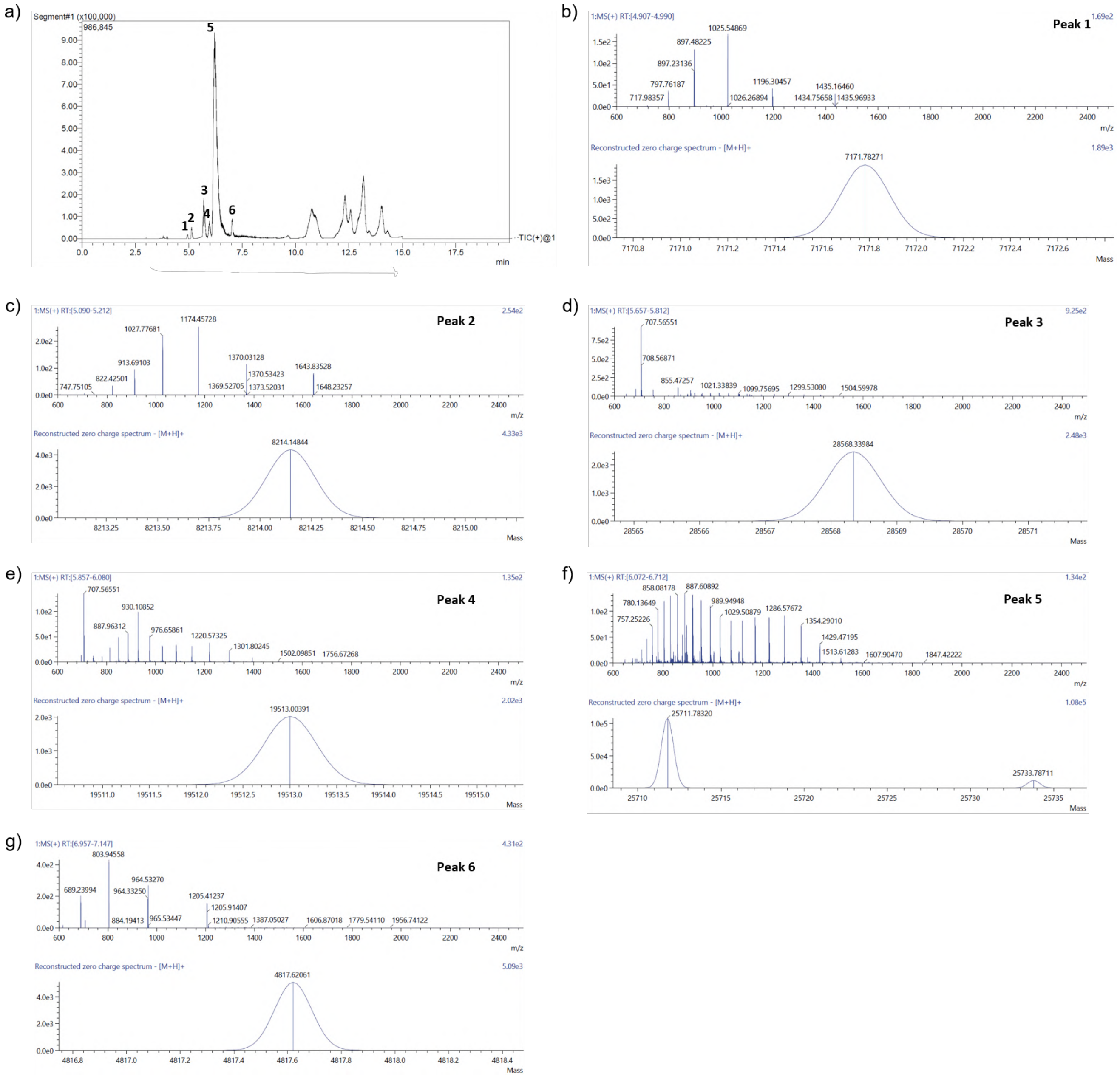

Figure S38. LC-MS analysis (Method B) of DHAR2 C6S starting material. Sample measured after reduction with 10 equiv. TCEP for 1 h at 37 °C. Protein concentration for analytical sample: 0.14 mg/mL. a) total ion chromatogram b) Peak 1 ion series and deconvoluted spectrum c) Peak 2 ion series and deconvoluted spectrum d) Peak 3 ion series and deconvoluted spectrum e) Peak 4 ion series and deconvoluted spectrum f) Peak 5 ion series and deconvoluted spectrum g) Peak 6 ion series and deconvoluted spectrum; Calculated mass: DHAR2 C6S: 25712 Da. Observed mass: 25712 Da.

PrxIIB C76S

FASTA-sequence PrxIIB C76S:

MGSSHHHHHHSSGLVPRGSHMAPIAVGDVVPDGTISFFDENDQLQTASVHSLAAGKKVILFGVPGAFTPTCSM KHVPGFIEKAEELKSKGVDEIISFSVNDPFVMKAWGKTYPENKHVKFVADGSGEYTHLLGLELDLKDKGLGVRS RRFALLLDDLKVTVANVESGGEFTVSSADDILKAL*

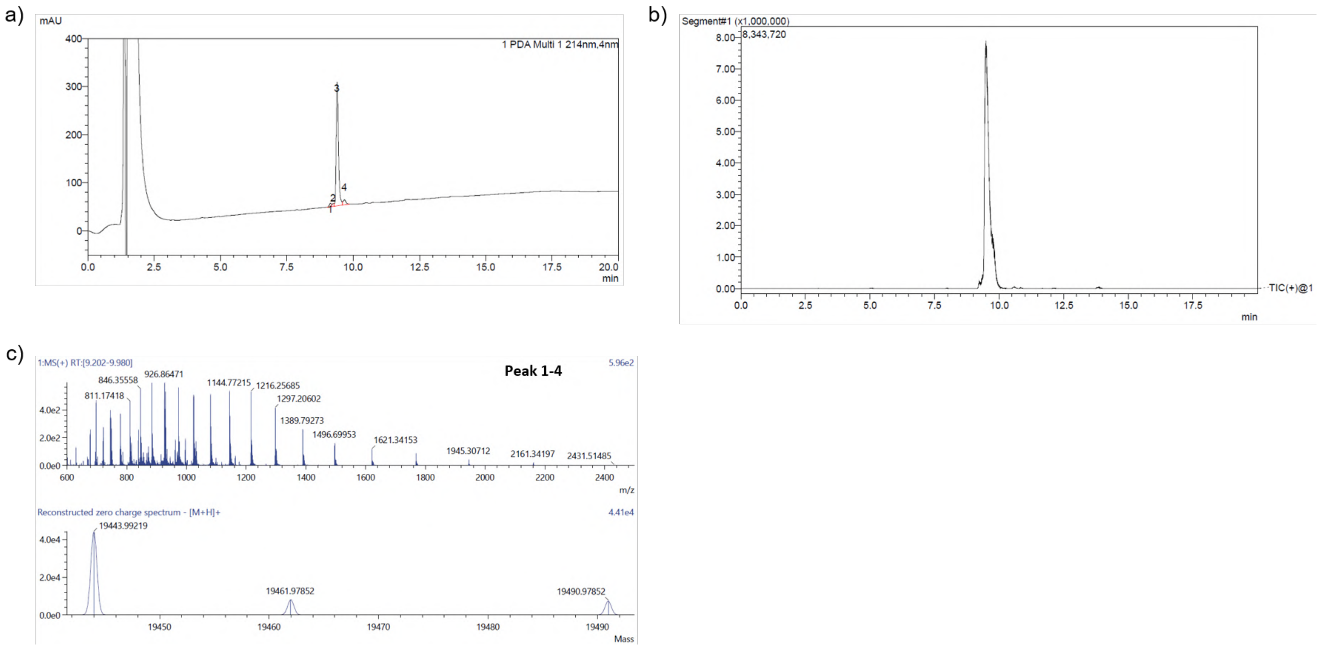

Figure S39. LC-MS analysis (Method C) of PrxIIB C76S starting material. Sample measured after reduction with 10 equiv. TCEP for 1 h at 37 °C. Protein concentration for analytical sample: 0.14 mg/mL. a) UV-trace b) total ion chromatogram c) Peak 1-4 ion series and deconvoluted spectrum; Calculated mass: PrxIIB C76S: 19444 Da. Observed mass: 19444 Da.

Trxh1 C43S

Amino acid-sequence Trxh1 C43S:

MGSSHHHHHHSSGLVPRGSHMASEEGQVIACHTVETWNEQLQKANESKTLVVVDFTASWCGPSRFIAPFFAD
LAKKLPNVLFLKVDTDELKSVASDWAIQAMPTFMFLKEGKILDKVVGAKKDELQSTIAKHLA *

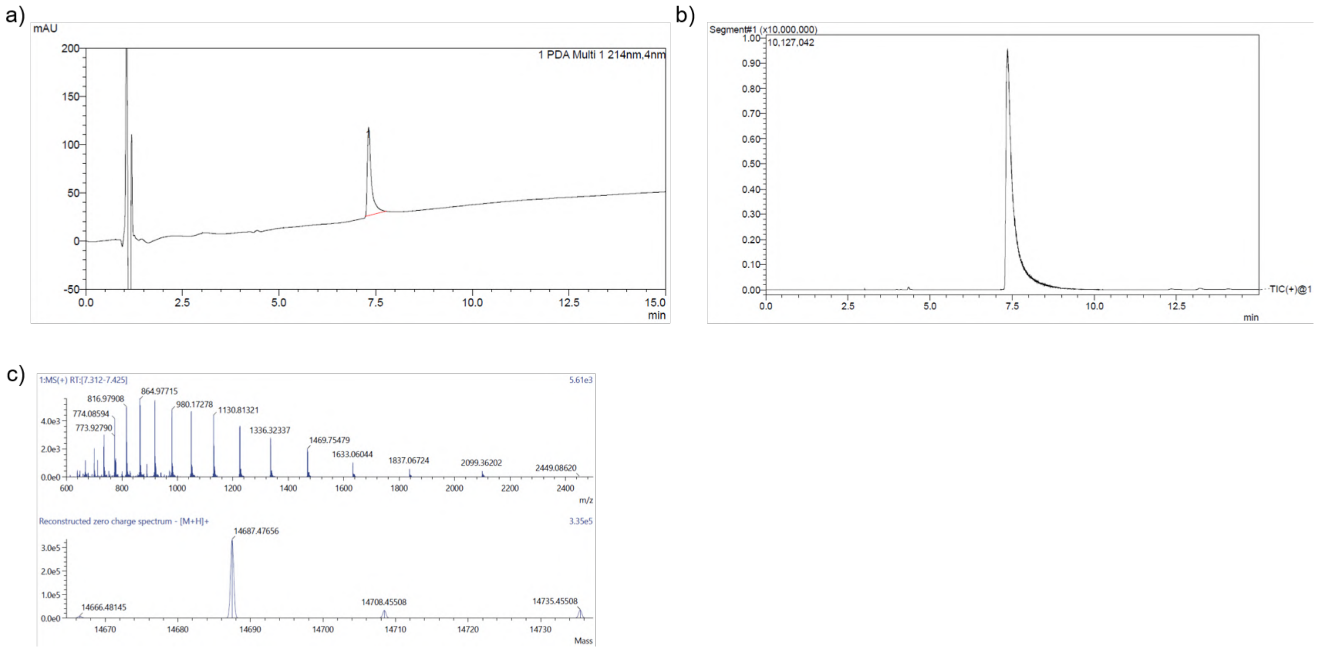

Figure S40. LC-MS analysis (Method B) of Trxh1 C43S starting material. Sample measured after reduction with 10 equiv. TCEP for 1 h at 37 °C. Protein concentration for analytical sample: 0.14 mg/mL: a) UV-trace b) total ion chromatogram c) Ion series and deconvoluted spectrum; Calculated mass: Trxh1 C43S: 14689 Da. Observed mass: 14687 Da.

^{15}N,^{13}C-labeled ubiquitin T12C

Amino acid-sequence ^{15}N,^{13}C-Ubiquitin T12C:

MQIFVKTLTGKCITLEVEPSDTIENVKAKIQDKEGIPPDQQRLIFAGKQLEDGRTLSDYNIQKESTLHLVLRLRGG*

Ubiquitin T12C (B)

Amino acid-sequence Ubiquitin T12C (B):

MQIFVKTLTGKCITLEVEPSDTIENVKAKIQDKEGIPPDQQRLIFAGKQLEDGRTLSDYNIQKESTLHLVLRLRGG*

a)

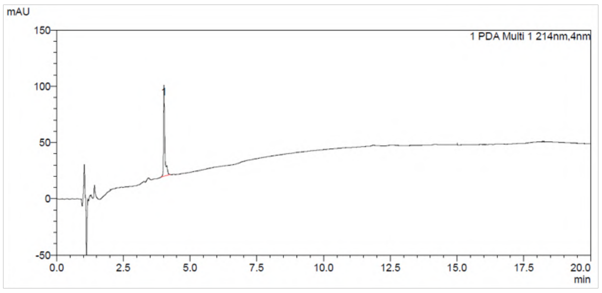

b)

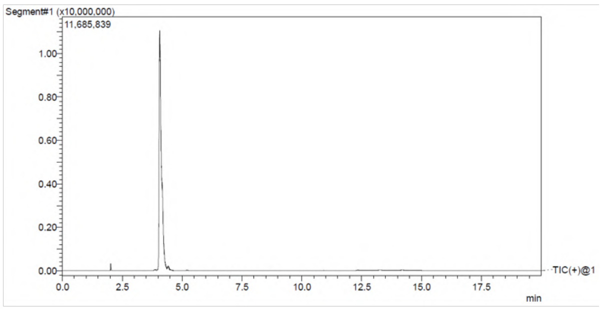

c)

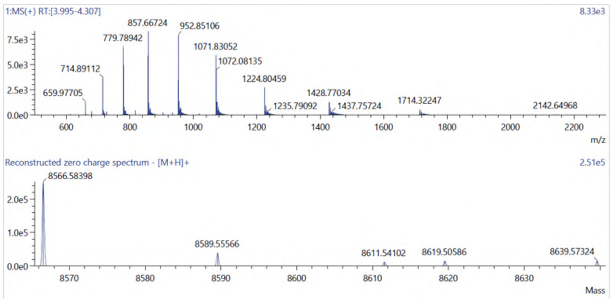

Figure S41. LC-MS analysis (Method B) of Ubiquitin T12C (B) starting material. Sample measured after reduction with 10 equiv. TCEP for 1 h at 25 °C. Protein concentration for analytical sample: 0.14 mg/mL: a) total ion chromatogram b) Ion series and deconvoluted spectrum; Calculated mass: Ubiquitin T12C (B): 8567 Da. Observed mass: 8567 Da.

Bovine Serum Albumin (BSA)

Amino acid-sequence BSA:

DTHKSEIAHRFKDLGEEHFKGLVLIAFSQYLQQCPFDEHVKLVNELTEFAKTCVADESHAGCEKSLHTLFGDELC
KVASLRETYGDMADCCEKQEPERNECFLSHKDDSPDLPKLKPDPNTLCDEFKADEKKFWGKYLYEIARRHPYF
YAPELLYYANKYNGVFQECCQAEDKGACLLPKIETMREKVLASSARQRLRCASIQKFGERALKAWSVARLSQKF
PKAEFVEVTKLVTDLTKVHKECCHGDLLECADDRADLAKYICDNQDTISSKLKECCDKPLLEKSHCIAEVEKDAIP
ENLPPLTADFAEDKDVCKNYQEAKDAFLGSFLYEYSRRHPEYAVSVLLRLAKEYEATLEECCAKDDPHACYSTV
FDKLKHLVDEPQNLIKQNCDQFEKLGEYGFQNALIVRYTRKVPQVSTPTLVEVSRSLGKVGTRCCTKPESERMP
CTEDYLSLILNRLCVLHEKTPVSEKVTKCCTESLVNRRPCFSALTPDETYVPKAFDEKLFTFHADICTLPDTEKQIK
KQTALVELLKHKPKATEEQLKTVMENFVAFVDKCCAADDKEACFAVEGPKLVVSTQTALA*

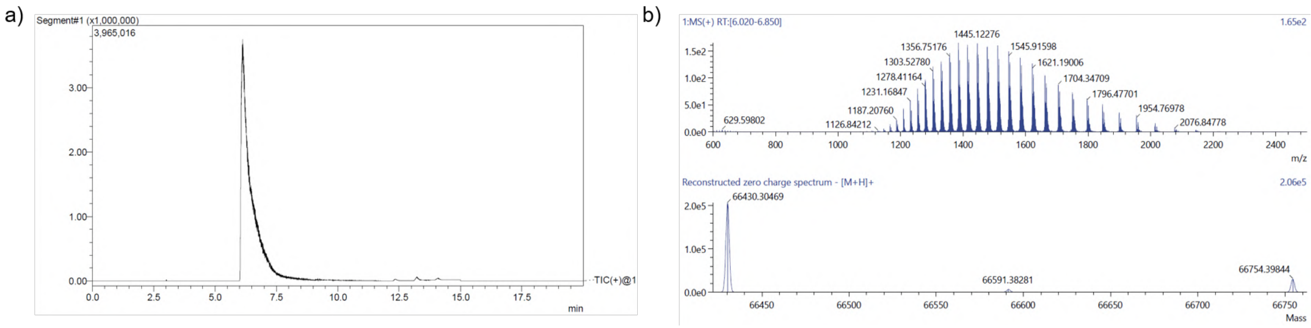

Figure S42. LC-MS analysis (Method B) of BSA starting material. Sample measured after reduction with 10 equiv. TCEP for 1 h at 25 °C. Protein concentration for analytical sample: 0.14 mg/mL: a) total ion chromatogram b) Ion series and deconvoluted spectrum; Calculated mass: BSA: 66433 Da. Observed mass: 66430 Da.

MDAR2

Amino acid-sequence MDAR2:

MGSSHHHHHHSSGLVPRGSHMASMTGGQQMGRGSMAEEKSFKYVIVGGGVAAGYAAREFFNQGVKPGELAI
ISREQVPPYERPALSKGYIHLENKATLPNFYVAAGIGGERQFPQWYKEKGIELILGTEIVKADLAAKTLVSGTGQV
FKYQTLLAATGSSVIRLSDFGVPGADAKNIFYLRELEDADYLAYAMETKEKGKAVVVGGGYIGLELGAALKANNL
DVTMVYPEPWCMPRLFTAGIASFYEGYYANKGINIVKGTVASGFTTNSNGEVTEVKLKDGRTLEADIVIVGVGGR
PIISLFKDQVEEEKGGLKTDGFFKTSLPDVYAIGDVATFPMKLYNEMRRVEHVDHARKSAEQAVKAIKAAEEGNS
IPEYDYLPYFYSRAFDLSWQFYGDNVGESVLFGDNDPESPKPKFGSYWIKERKVVGAFLEGGSPEENNAIAKLA
RAQPSVESLEVLSKEGLSFATNI*

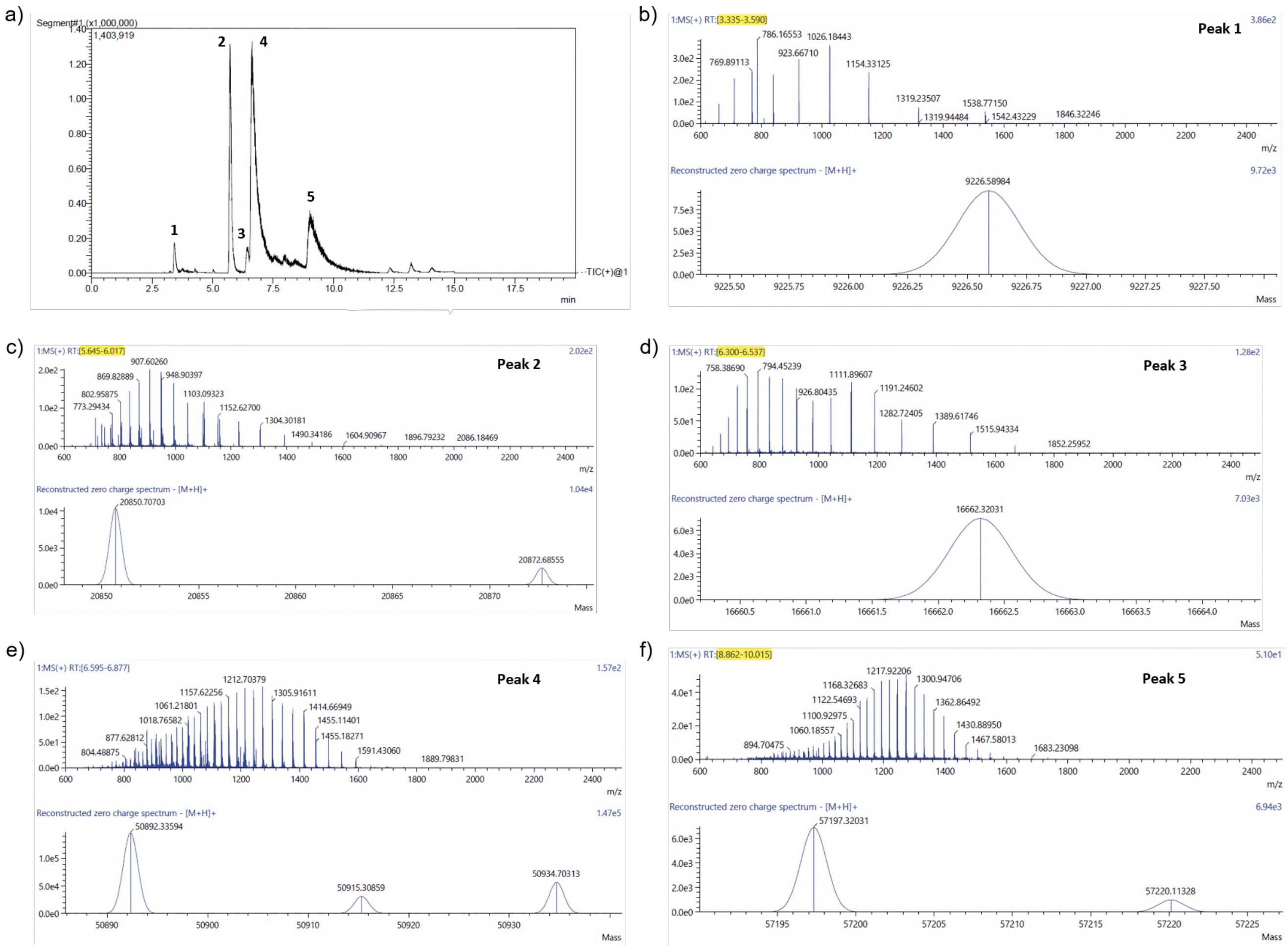

Figure S43. LC-MS analysis (Method B) of MDAR2 starting material. Sample measured after reduction with 10 equiv. TCEP for 1 h at 25 °C. Protein concentration for analytical sample: 0.14 mg/mL: a) total ion chromatogram b) Peak 1 ion series and deconvoluted spectrum c) Peak 2 ion series and deconvoluted spectrum d) Peak 3 ion series and deconvoluted spectrum e) Peak 4 ion series and deconvoluted spectrum f) Peak 5 ion series and deconvoluted spectrum; Calculated mass: MDAR2: 50893 Da. Observed mass: 50892 Da.

MDAR3

Amino acid-sequence MDAR3:

MGSSHHHHHHSSGLVPRGSHMASMTGGQQMGRGSMAEEKSYKYVIIGGGVAGGYAAREFSNQGLKPGELAII
SKEPVPPFERPELTKVYIDLEVNPTLANIYVCAGTGEAKQYPNWYKEKGIDLIVGTEIVKADLASKTLVSDDGKIYK
YQTLLIATGSTNIRLSEIGVQEADVKNIFYLREIEDSDELALAMELYVQRGKAVIIGGGFLGLEISSALRANNHEVTM
VFPEPWLVHRFFTAEIASFYESYYANKGIKIIKGTVATGFSTNSDGEVTEVKLEDGRTLEANIVVAGVGARPATSL
FKGQLEEEKGGIKTDGFFKTSVPDVYALGDVATFPMKMYGGTRRVEHADNARKSAAQAVKAIKAGEEGKTIPDY
DYLPYFYSRFFKLSWEFYGENVGESVLFGDNDPKSPKPKFGTYWVKDGKVVGVFLEGGTQEEHKAIAKVARAQ
PSVESLDVLSEEGLSFATKFYSTSL*

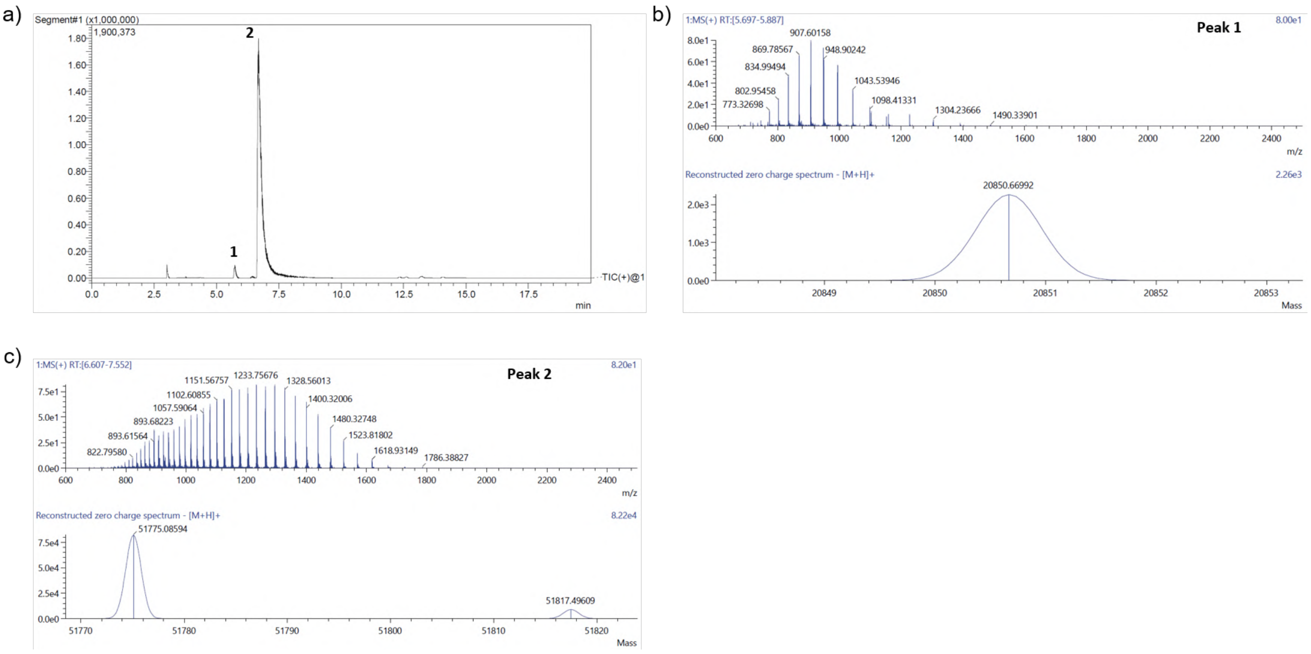

Figure S44. LC-MS analysis (Method B) of MDAR3 starting material. Sample measured after reduction with 20 equiv. TCEP for 1 h at 25 °C. Protein concentration for analytical sample: 0.14 mg/mL: a) total ion chromatogram b) Ion series and deconvoluted spectrum; Calculated mass: MDAR3: 51776 Da. Observed mass: 51775 Da.

Data availability

The LC-MS raw data is available via the following online repositories:

https://doi.org/10.5281/zenodo.7472436

https://owncloud.gwdg.de/index.php/s/uf0bv5a6HLflfcW

Protein expression and purification

Site-directed-mutagenesis protocol

Generation of plasmids

The coding sequences of *A. thaliana* DHAR1 (At1g19570), DHAR2 (At1g75270), PrxIIB (At1g65980), MDAR2 (At5g03630), and MDAR3 (At3g09940) were amplified from leaf cDNA using specific forward and reverse primers with restriction sites as indicated in Table S1. sfGFP plasmid pSB1C3 (BBa_K1758102) was kindly provided by the iGEM-team 2015 of Bielefeld University, Ubiquitin K63C (Plasmid #86589) was purchased from Addgene. The cysteine-mutants were generated by site-directed mutagenesis with specific primers (Table S1). All coding sequences were cloned into pET28a (Novagen, Darmstadt, Germany). The correctness of all constructs was confirmed by DNA sequencing.

Table S1. List of Primers utilized for cloning.

DHAR1_NheI_F	AAAAGCTAGCATGGCTCTGGAAATCTGTG
DHAR1_EcoRI_R	TTTTGAATTCTCAAGGGTTAACCTTGGGAG

DHAR1-S97C_F	CTGCTGAATTTGCCTGTGTTGGATCC
DHAR1-S97C_R	GGATCCAACACAGGCAAATTCAGCAG
DHAR1-S176C_F	CTGTCCCTGAGTGCTTTCCCCATG
DHAR1-S176C_R	CATGGGGAAAGCACTCAGGGACAG
DHAR2-C6S_NheI_F	AAAAGCTAGCATGGCTCTAGATATCTCCGTG
DHAR2_EcoRI_R	TTTTGAATTCTCACGCATTCACCTTCGATTC
PrxIIB_NdeI_F	AAAACATATGGCTCCAATTGCTGTCGGCGATG
PrxIIB_BamHI_R	TTTTGGATCCTTAGAGAGCCTTGAGGATATCATCGGCGCTG
PrxIIB_C76S_F	GTTGATGAGATCATTTCCTTTAGCGTGAACGATC
PrxIIB_C76S_R	GATCGTTCACGCTAAAGGAAATGATCTCATCAAC
Trxh1_NdeI_F	AAAACATATGGCTTCGGAAGAAGGACAAGTG
Trxh1_BamHI_R	TTTTGGATCCTTAAGCCAAGTGTTTGGCAATGGTAGAC
Trxh1-C43S_F	CTTCTTGGTGTGGACCAAGTCGTTTC
Trxh1-C43S_R	GAAACGACTTGGTCCACACCAAGAAG
MDAR2_BamHIF	AAAAGGATCCATGGCAGAAGAAAAGAGCTTCAAGTATG
MDAR2_NotIR	TTTTGCGGCCGCTTATATATTTGTGGCGAAGGAAAGGCCTTC
MDAR3_BamHIF	AAAAGGATCCATGGCGGAAGAGAAAAGCTACAAG
MDAR3_NotIR	TTTTGCGGCCGCTCAAAGAGAGGTGCTATAGAACTTGGTG
sfGFP-NheI_F	AAAAGCTAGCATGCGTAAAGGCGAAGAG
sfGFP-HindIII_R	TTTTAAGCTTTCATTTGTACAGTTCATCCATACCATG
sfGFP-S147C-F	CAATTTTAACTGCCACAATGTTTACATCACC
sfGFP-S147C-R	GGTGATGTAAACATTGTGGCAGTTAAAATTG
UBQ-C63K-F	GATTACAACATTCAGAAGGAGTCGACCTTACATC
UBQ-C63K-R	GATGTAAGGTCGACTCCTTCTGAATGTTGTAATC
UBQ-T9C-F	GTCAAGACGTTATGCGGTAAAACCATAACTCTGGAA
UBQ-T9C-R	TTCCAGAGTTATGGTTTTACCGCATAACGTCTTGAC
UBQ-T12C-F	GTCAAGACGTTAACCGGTAAATGCATAACTCTGGAA
UBQ-T12C-R	TTCCAGAGTTATGCATTTACCGGTTAACGTCTTGAC

Plasmid amplification

The plasmid was transformed into One Shot™ TOP10 Chemically Competent *E. coli* (Invitrogen) by a heat

shock protocol and grown over night on agar plates supplemented with kanamycin (50 µg/ml). A single colony was transferred to 20 mL of LB medium (50 µg/ml kanamycin) and incubated at 37 °C overnight. Plasmid purification was performed by NucleoSpin Plasmid kit (Macherey-Nagel) following the manufacturer's instructions.

Protein expression and purification

For recombinant protein expression, *E. coli* NEB NiCo21 (DE3) cells were transformed with plasmid DNA. After the induction of protein expression with 400 µM isopropyl-β-D-thiogalactopyranoside in the exponential phase (OD_{600}=0.6), expression was carried out for 4 h at 37 °C and 170 rpm, except of sfGFP. The sfGFP was expressed at 21°C and 170 rpm for 16 hours. Bacteria were harvested by centrifugation (11.000 × g, 10 min, 4 °C) and resuspended in lysis buffer (50 mM Tris-HCl pH 8.0, 300 mM NaCl, 5 mM imidazole, 10 mM β-mercaptoethanol, 1 mM PMSF). Cells were lysed by adding 200 µg/mL lysozyme for 30 minutes at RT, followed by sonication, and the soluble and insoluble fractions were separated by centrifugation at 20.000× g and 4 °C for 60 min. The lysate was incubated with Roti®Garose-His/Ni NTA-beads (Carl Roth) previously equilibrated with lysis buffer, under slow shaking at 4 °C for 60 min. After washing with 200 mL washing buffer I (50 mM Tris-HCl pH 8, 300 mM NaCl, 10 mM imidazole) and 50 mL washing buffer II (50 mM Tris-HCl pH 8, 300 mM NaCl, 20 mM imidazole), the His-tagged protein was eluted using the elution buffer (50 mM Tris-HCl pH 8, 300 mM NaCl, 250 mM imidazole, 10 mM β-mercaptoethanol, 1 mM PMSF). Eluted protein-containing fractions were pooled and concentrated. DHAR1, DHAR2, sfGFP, and ubiquitin proteins were dialyzed over night at 4 °C against 50 mM NaP_i, pH 7 (Dialysis tubing Membra-Cel™, cellulose, Carl Roth), with repeated changes of dialysis buffer. Ubiquitin proteins were also dialysed against 50 mM NaP_i, pH 6.5; PRXIIB, MDAR2, and MDAR3 proteins were dialyzed against 40 mM KP_i pH 7.2. Protein concentration was determined by absorption at 280 nm using the specific absorption co-efficient and a nanophotometer (Implen). Final purity was checked by separation in 12% (w/v) SDS-PAGE. Protein aliquots were stored at –80 °C.

His-tag cleavage of Ubiquitin proteins

Dialysis of Ubiquitin proteins was performed in presence of 10 units thrombin (bovine plasma, Sigma) per mg protein. Samples were cleared and supernatant was transferred to 1 µl benzamidine slurry (GE Healthcare Benzamidine Sepharose™ 4 fast flow (high sub)) / 2 u thrombin, slurry was washed for three times with dialysis buffer before. The samples were incubated for 1 h at 4 °C and centrifuged at 100× g for three minutes, the supernatant was transferred to Ni-NTA (Roti®Garose-His/Ni NTA-beads, Carl Roth), which was washed for three times with dialysis buffer before. The samples were incubated for 1 h at 4 °C again and centrifuged at 100× g for three minutes afterwards. Proteins in the supernatant were checked by SDS-PAGE, Thrombin-cleavage verified by Western blot with anti-His6 antibody (H-3, sc-8036, Santa Cruz Biotechnology) as the primary antibody and anti mouse IgG (peroxidase antibody produced in rabbit, A9044, Sigma Aldrich) as the secondary antibody. Proteins aliquots were stored at –80 °C.

Expression and purification of ^{15}N,^{13}C-lableled ubiquitin T12C and ubiquitin T12C (B)

The T12C mutant was generated by PCR-based site-directed mutagenesis using the QuikChange II™ Kit (Agilent) following the instructions of the supplier. The mutant ubiquitin was basically expressed and purified as originally published[11]. Briefly, the protein was expressed in *E. coli* strain BL21DE3/pLysS using minimal medium. For producing labeled protein the medium contained $^{13}C_6$-D-glucose and ^{15}N-ammonium chloride as sole carbon and

nitrogen sources. All buffers for protein purification were supplemented with 5 mM DTT. After lysis by ultrasound sonication, the protein was purified from the supernatant by cation exchange chromatography on a SP Sepharose column followed by gelfiltration on a Superdex 75 sizing column. After dialysis against 50 mM sodium phosphate pH 7.0 supplemented with 0.5 mM DTT, the protein was concentrated to final 1 mM concentration using a Vivascience concentrator with 5000 Da molecular weight cutoff.

Mass spectrometry of lysates

Heat treatment of *E. coli* and preparation of cell lysates

40 mL of an overnight culture of One Shot™ TOP10 Chemically Competent *E. coli* (Invitrogen) were inoculated into 800 mL LB and grown until the stationary phase was reached. Two times 200 mL were incubated at 37 °C for 1 hour, the remaining two times 200 mL at 43 °C for 150 minutes. The cells were harvested (11.000 × g, 10 min, 4 °C) and resuspended in the respective buffer system supplemented with 1 mM PMSF (Carl Roth). Cells were lyzed by 9-times sonication for 2 minutes at 70% power. The lysate was cleared at 20.000 × g for 1 hour at 4°C. Lysate aliquots were stored at –80 °C.

Protein concentrations of cell lysates were determined by following the standard protocol for the Micro BCA Protein Assay Kit (23235#, Pierce Company) as described by the manufacturer. The cysteine concentration was estimated by the following equation utilizing the determined protein concentration.

c(cysteine) = (β(lysate)/$M_{average}$(amino acid)) × abundance of Cys in *E. coli*[12].

The utilized average molecular weight of an amino acid is 110 g/mol. All calculations are based on the estimated concentration for cysteine in the sample.

Selectivity studies with lysates

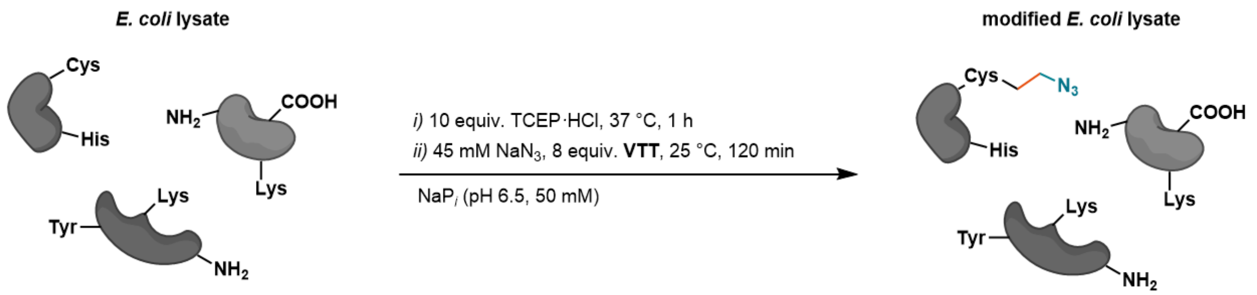

At 20–25 °C, 22 µL of lysate (2.50 mg/mL, 2.3 × 10^2 µM Cys, 5.0 nmol Cys, 1.0 equiv.) in NaP_i buffer (pH 6.5, 50 mM) was added to a 1.5 mL Eppendorf tube and diluted with 143 µL of NaP_i buffer (pH 6.5, 50 mM). Subsequently, 5.0 µL of a TCEP stock solution (10 mM, 50 nmol, 13 µg, 10 equiv.) in UHQ-H_2O was added to the mixture. The mixture was vortexed for 1 second, transferred into a Thermocycler pre-heated at 37 °C, and incubated at 37 °C for 1 hour at 400 rpm. Next, a 0.30 M sodium azide stock solution (30 µL, 9.0 µmol, 0.59 mg, 1.8 × 10^3 equiv.) in NaP_i buffer (pH 6.5, c = 50 mM) was added to the mixture at 20–25 °C (c_{Nu} = 45 mM), followed by addition of 0.5 µL of a **VTT** stock solution (80 mM, 0.04 µmol, 0.01 mg, 8 equiv.) in DMF. The reaction mixture was vortexed for 1 second, transferred into a Thermocycler pre-heated at 25 °C, and incubated at 25 °C at 400 rpm for 120 minutes. Subsequently, 22 µL of a β-mercaptoethanol stock solution (0.15 M, 3.3 µmol, 6.6 × 10^2 equiv.) in UHQ-H_2O was added, and the mixture was incubated at 25 °C for 30 minutes.

Next, the samples were prepared for LC-MS/MS analysis following the established standard SP3 protocol[13], using TCEP as reductant, chloroacetamide as alkylating reagent, and Trypsin + rLysC mix with a protein:trypsin ratio of 50:1 for digestion.

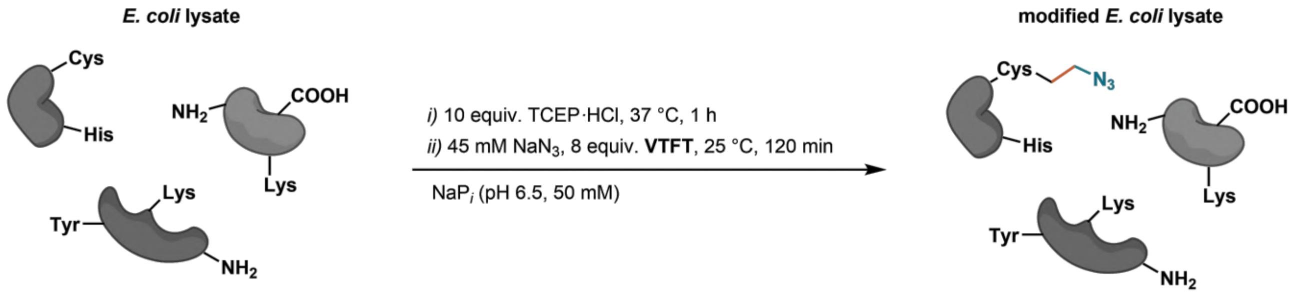

At 20–25 °C, 22 µL of lysate (2.50 mg/mL, 2.3 × 10^2 µM Cys, 5.0 nmol Cys, 1.0 equiv.) in NaP$_i$ buffer (pH 6.5, 50 mM) was added to a 1.5 mL Eppendorf tube and diluted with 143 µL of NaP$_i$ buffer (pH 6.5, 50 mM). Subsequently, 5.0 µL of a TCEP stock solution (10 mM, 50 nmol, 13 µg, 10 equiv.) in UHQ-H_2O was added to the mixture. The mixture was vortexed for 1 second, transferred into a Thermocycler pre-heated at 37 °C, and incubated at 37 °C for 1 hour at 400 rpm. Next, a 0.30 M sodium azide stock solution (30 µL, 9.0 µmol, 0.59 mg, 1.8 × 10^3 equiv.) in NaP$_i$ buffer (pH 6.5, c = 50 mM) was added to the mixture at 20–25 °C (c_{Nu} = 45 mM), followed by addition of 0.5 µL of a **VTFT** stock solution (80 mM, 0.04 µmol, 0.01 mg, 8 equiv.) in DMF. The reaction mixture was vortexed for 1 second, transferred into a Thermocycler pre-heated at 25 °C, and incubated at 25 °C at 400 rpm for 120 minutes. Subsequently, 22 µL of a β-mercaptoethanol stock solution (0.15 M, 3.3 µmol, 6.6 × 10^2 equiv.) in UHQ-H_2O was added, and the mixture was incubated at 25 °C for 30 minutes. Next, the samples were prepared for LC-MS/MS analysis following the established standard SP3 protocol[13], using TCEP as reductant, chloroacetamide as alkylating reagent, and Trypsin + rLysC mix with a protein:trypsin ratio of 50:1 for digestion.

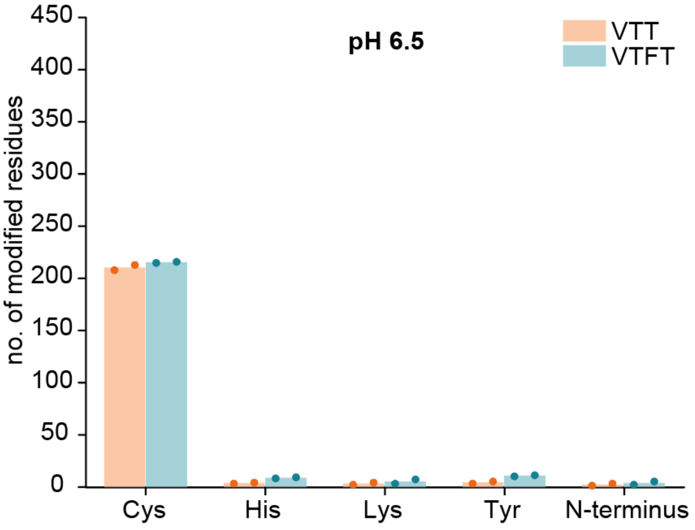

Figure S45. LC-MS/MS analysis of the selectivity studies with **VTT** and **VTFT** at pH 6.5. The mean number of unique modified residues was found according to the search described above. The samples were measured with two technical replicates.

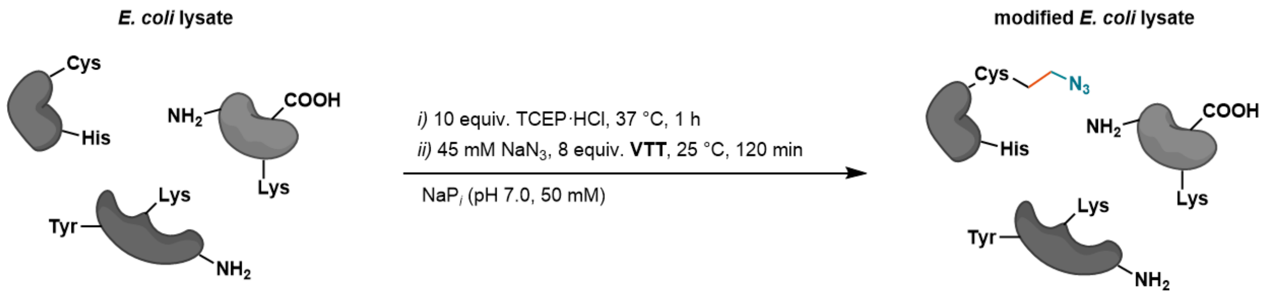

At 20–25 °C, 19 µL of lysate (2.88 mg/mL, 2.6×10^2 µM Cys, 5.0 nmol Cys, 1.0 equiv.) in NaP_i buffer (pH 7.0, 50 mM) was added to a 1.5 mL Eppendorf tube and diluted with 146 µL of NaP_i buffer (pH 7.0, 50 mM). Subsequently, 5.0 µL of a TCEP stock solution (10 mM, 50 nmol, 13 µg, 10 equiv.) in UHQ-H_2O was added to the mixture. The mixture was vortexed for 1 second, transferred into a Thermocycler pre-heated at 37 °C, and incubated at 37 °C for 1 hour at 400 rpm. Next, a 0.30 M sodium azide stock solution (30 µL, 9.0 µmol, 0.59 mg, 1.8×10^3 equiv.) in NaP_i buffer (pH 7.0, c = 50 mM) was added to the mixture at 20–25 °C (c_{Nu} = 45 mM), followed by addition of 0.5 µL of a **VTT** stock solution (80 mM, 0.04 µmol, 0.01 mg, 8 equiv.) in DMF. The reaction mixture was vortexed for 1 second, transferred into a Thermocycler pre-heated at 25 °C, and incubated at 25 °C at 400 rpm for 120 minutes. Subsequently, 22 µL of a β-mercaptoethanol stock solution (0.15 M, 3.3 µmol, 6.6×10^2 equiv.) in UHQ-H_2O was added, and the mixture was incubated at 25 °C for 30 minutes. Next, the samples were prepared for LC-MS/MS analysis following the established standard SP3 protocol[13], using TCEP as reductant, chloroacetamide as alkylating reagent, and Trypsin + rLysC mix with a protein:trypsin ratio of 50:1 for digestion.

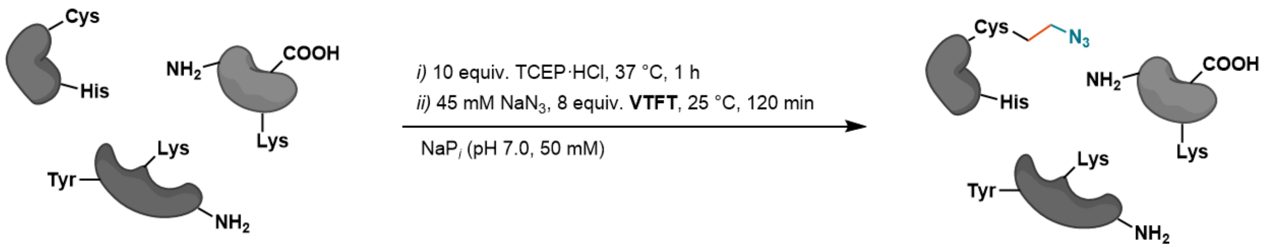

At 20–25 °C, 19 µL of lysate (2.88 mg/mL, 2.6×10^2 µM Cys, 5.0 nmol Cys, 1.0 equiv.) in NaP_i buffer (pH 7.0, 50 mM) was added to a 1.5 mL Eppendorf tube and diluted with 146 µL of NaP_i buffer (pH 7.0, 50 mM). Subsequently, 5.0 µL of a TCEP stock solution (10 mM, 50 nmol, 13 µg, 10 equiv.) in UHQ-H_2O was added to the mixture. The mixture was vortexed for 1 second, transferred into a Thermocycler pre-heated at 37 °C, and incubated at 37 °C for 1 hour at 400 rpm. Next, a 0.30 M sodium azide stock solution (30 µL, 9.0 µmol, 0.59 mg, 1.8×10^3 equiv.) in NaP_i buffer (pH 7.0, c = 50 mM) was added to the mixture at 20–25 °C (c_{Nu} = 45 mM), followed by addition of 0.5 µL of a **VTFT** stock solution (80 mM, 0.04 µmol, 0.01 mg, 8 equiv.) in DMF. The reaction mixture was vortexed for 1 second, transferred into a Thermocycler pre-heated at 25 °C, and incubated at 25 °C at 400 rpm for 120 minutes. Subsequently, 22 µL of a β-mercaptoethanol stock solution (0.15 M, 3.3 µmol, 6.6×10^2 equiv.) in UHQ-H_2O was added, and the mixture was incubated at 25 °C for 30 minutes. Next, the samples were prepared for LC-MS/MS analysis following the established standard SP3 protocol[13], using TCEP as reductant, chloroacetamide as alkylating reagent, and Trypsin + rLysC mix with a protein:trypsin ratio of 50:1 for digestion.

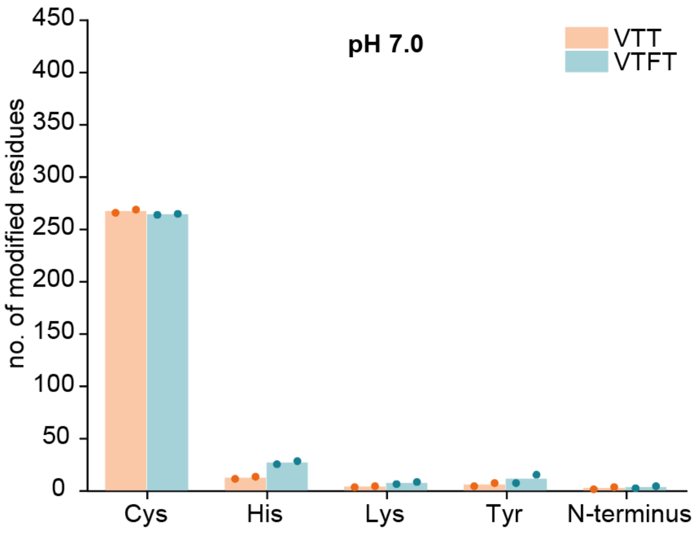

Figure S46. LC-MS/MS analysis of the selectivity studies with **VTT** and **VTFT** at pH 7.0. The mean number of unique modified residues was found according to the search described above. The samples were measured with two technical replicates.

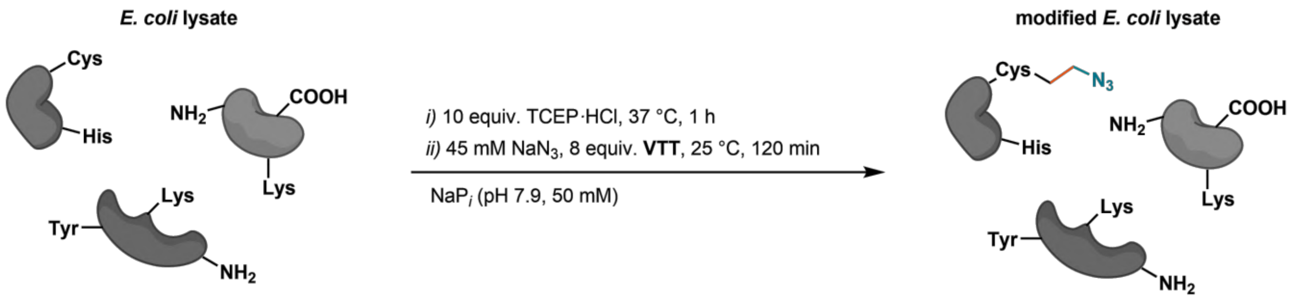

At 20–25 °C, 20 µL of lysate (2.73 mg/mL, 2.5 × 10^2 µM Cys, 5.0 nmol Cys, 1.0 equiv.) in NaP*i* buffer (pH 7.9, 50 mM) was added to a 1.5 mL Eppendorf tube and diluted with 145 µL of NaP*i* buffer (pH 7.9, 50 mM). Subsequently, 5.0 µL of a TCEP stock solution (10 mM, 50 nmol, 13 µg, 10 equiv.) in UHQ-H_2O was added to the mixture. The mixture was vortexed for 1 second, transferred into a Thermocycler pre-heated at 37 °C, and incubated at 37 °C for 1 hour at 400 rpm. Next, a 0.30 M sodium azide stock solution (30 µL, 9.0 µmol, 0.59 mg, 1.8 × 10^3 equiv.) in NaP*i* buffer (pH 7.9, c = 50 mM) was added to the mixture at 20–25 °C (c_{Nu} = 45 mM), followed by addition of 0.5 µL of a **VTT** stock solution (80 mM, 0.04 µmol, 0.01 mg, 8 equiv.) in DMF. The reaction mixture was vortexed for 1 second, transferred into a Thermocycler pre-heated at 25 °C, and incubated at 25 °C at 400 rpm for 120 minutes. Subsequently, 22 µL of a β-mercaptoethanol stock solution (0.15 M, 3.3 µmol, 6.6 × 10^2 equiv.) in UHQ-H_2O was added, and the mixture was incubated at 25 °C for 30 minutes. Next, the samples were prepared for LC-MS/MS analysis following the established standard SP3 protocol[13], using TCEP as reductant, chloroacetamide as alkylating reagent, and Trypsin + rLysC mix with a protein:trypsin ratio of 50:1 for digestion.

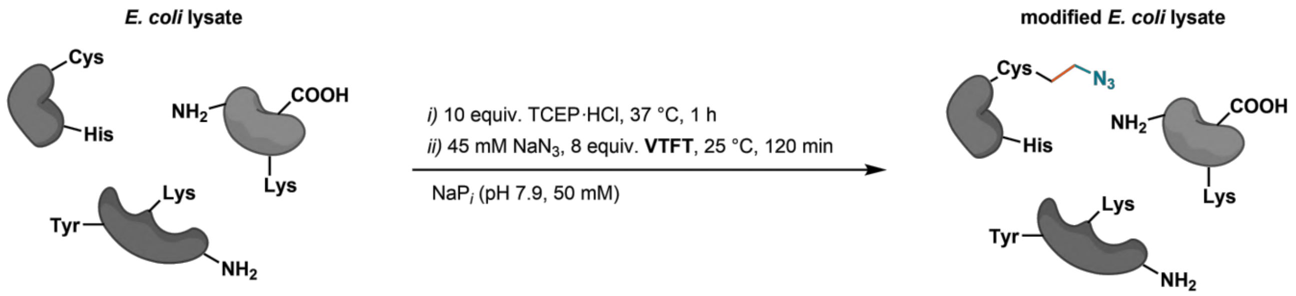

At 20–25 °C, 20 µL of lysate (2.73 mg/mL, 2.5 × 10^2 µM Cys, 5.0 nmol Cys, 1.0 equiv.) in NaP$_i$ buffer (pH 7.9, 50 mM) was added to a 1.5 mL Eppendorf tube and diluted with 145 µL of NaP$_i$ buffer (pH 7.9, 50 mM). Subsequently, 5.0 µL of a TCEP stock solution (10 mM, 50 nmol, 13 µg, 10 equiv.) in UHQ-H_2O was added to the mixture. The mixture was vortexed for 1 second, transferred into a Thermocycler pre-heated at 37 °C, and incubated at 37 °C for 1 hour at 400 rpm. Next, a 0.30 M sodium azide stock solution (30 µL, 9.0 µmol, 0.59 mg, 1.8 × 10^3 equiv.) in NaP$_i$ buffer (pH 7.9, c = 50 mM) was added to the mixture at 20–25 °C (c_{Nu} = 45 mM), followed by addition of 0.5 µL of a **VTFT** stock solution (80 mM, 0.04 µmol, 0.01 mg, 8 equiv.) in DMF. The reaction mixture was vortexed for 1 second, transferred into a Thermocycler pre-heated at 25 °C, and incubated at 25 °C at 400 rpm for 120 minutes. Subsequently, 22 µL of a β-mercaptoethanol stock solution (0.15 M, 3.3 µmol, 6.6 × 10^2 equiv.) in UHQ-H_2O was added, and the mixture was incubated at 25 °C for 30 minutes. Next, the samples were prepared for LC-MS/MS analysis following the established standard SP3 protocol[13], using TCEP as reductant, chloroacetamide as alkylating reagent, and Trypsin + rLysC mix with a protein:trypsin ratio of 50:1 for digestion.

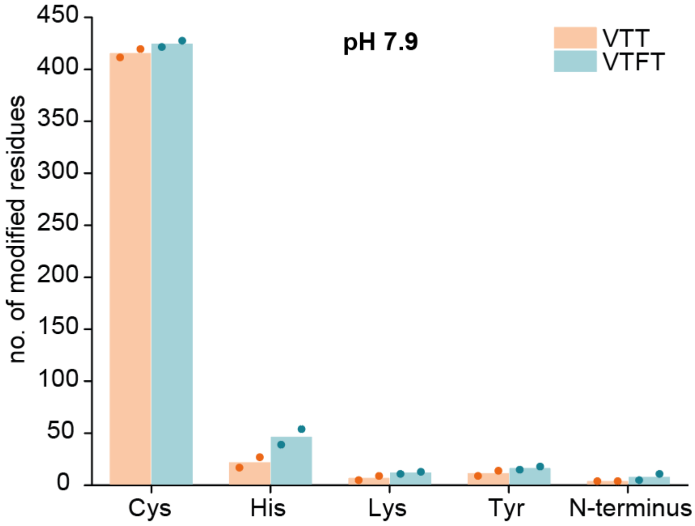

Figure S47. LC-MS/MS analysis of the selectivity studies with **VTT** and **VTFT** at pH 7.9. The mean number of unique modified residues was found according to the search described above. The samples were measured with two technical replicates.

Cysteine coverage rate calculation

In order to compare the labeling efficiency of different reagents that were used in lysates originating from different organisms, we calculated a ratio between alkylated Cys residues and globally available Cys residues. The number of available cysteine residues was calculated based on the proteomes of the organisms fetched from the uniprot website[14-16]. We compared the reagents to IAA[17] and TFBX[18]. In case of TFBX we calculated

the cysteine coverage with and without an additional streptavidin enrichment to demonstrate the potential of follow-up enrichment steps which would also be feasible for vinyl thianthrenium modification with sodium azide.

$$\text{labeling efficiency} = \frac{\text{number of alkylated cysteine residues}}{\text{number of globally available cysteine residues}} \cdot 100\ \%$$

Table S2. Comparison of Cys-labeling efficiency for different reagents.

reagent	c(reagent)<br>buffer & pH	Organism[a)]	Alkylated Cys	Number of available Cys	Labeling efficiency / %
**VTT**	0.2 mM<br>NaP_i, pH 7.9	*E.coli*	414	15785	2.6
**VTFT**	0.2 mM<br>NaP_i, pH 7.9	*E.coli*	423	15785	2.7
IAA[17]	1 mM<br>HEPES, pH 8.2	*S. cerevisiae*	217	41535	0.5
TFBX **1**[b),18]	2 mM<br>PBS with 0.5% SDS	*H. sapiens*	4169	262476	1.6
TFBX **2**[c),18]	0.1 mM<br>PBS	*H. sapiens*	10062	262476	3.8

a) Organism from which the cell lysate originates.
b) Pentafluoroethyl-3,3-dimethyl benziodoxoles, no peptide purification.
c) 1-(4-azido-1,1,2,2-tetrafluorobutyl)-3,3-dimethyl-1,3-dihydro-1λ 3-benzo[d][1,2]iodaoxole, peptide purification via streptavidin enrichment.

Quantitative Proteomics

Preparation of conjugated control cell lysates

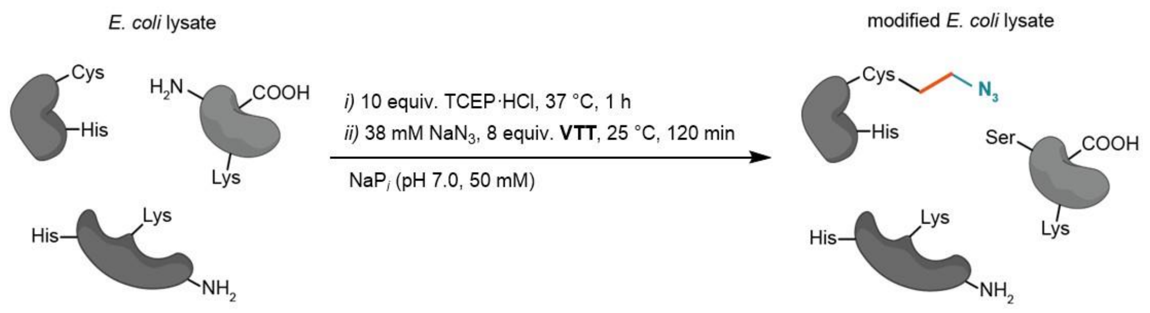

At 20–25 °C, 6.0 µL of non-stressed control lysate (8.6 mg/mL, 7.8 × 10^2 µM Cys, 5.0 nmol Cys, 1.0 equiv.) in NaP_i buffer (pH 7.0, 50 mM) was added to a 1.5 mL Eppendorf tube and diluted with 0.13 mL of NaP_i buffer (pH 7.0, 50 mM). Subsequently, 5.0 µL of a TCEP stock solution (10 mM, 50 nmol, 13 µg, 10 equiv.) in UHQ-H_2O was added to the mixture. The mixture was vortexed for 1 second, transferred into a Thermocycler pre-

heated at 37 °C, and incubated at 37 °C for 1 hour at 400 rpm. Next, a 0.30 M sodium azide stock solution (30 µL, 9.0 µmol, 0.59 mg, 1.8 × 10^3 equiv.) in NaP_i buffer (pH 7.0, c = 50 mM) was added to the mixture at 20–25 °C (c_{Nu} = 38 mM), followed by addition of 0.5 µL of a **VTT** stock solution (80 mM, 0.04 µmol, 0.01 mg, 8 equiv.) in DMF. The reaction mixture was vortexed for 1 second, transferred into a Thermocycler pre-heated at 25 °C, and incubated at 25 °C at 400 rpm for 120 minutes. Subsequently, 22 µL of a β-mercaptoethanol stock solution (0.15 M, 3.3 µmol, 6.6 × 10^2 equiv.) in UHQ-H_2O was added, and the mixture was incubated at 25 °C for 30 minutes. Next, the samples were prepared for LC-MS/MS analysis following the established standard SP3 protocol[13], using TCEP as reductant, chloroacetamide as alkylating reagent, and Trypsin + rLysC mix with a protein:trypsin ratio of 50:1 for digestion.

Preparation of conjugated heat-shock cell lysates with $^{13}C_2$ labeling

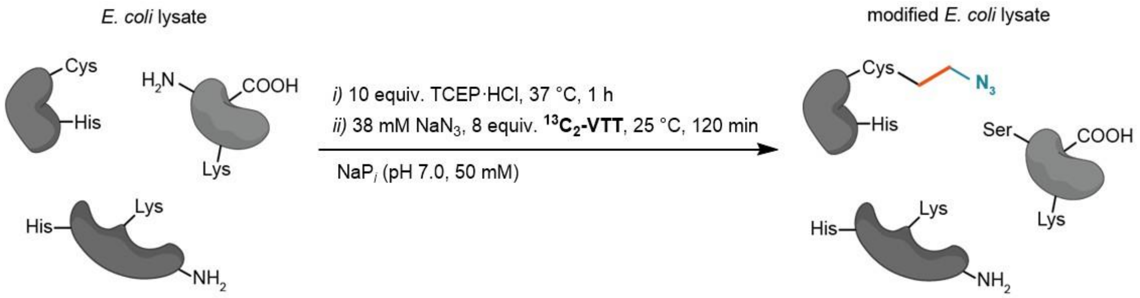

At 20–25 °C, 6.0 µL of heat-shock lysate (8.7 mg/mL, 7.8 × 10^2 µM Cys, 5.0 nmol Cys, 1.0 equiv.) in NaP_i buffer (pH 7.0, 50 mM) was added to a 1.5 mL Eppendorf tube and diluted with 0.13 mL of NaP_i buffer (pH 7.0, 50 mM). Subsequently, 5.0 µL of a TCEP stock solution (10 mM, 50 nmol, 13 µg, 10 equiv.) in UHQ-H_2O was added to the mixture. The mixture was vortexed for 1 second, transferred into a Thermocycler pre-heated at 37 °C, and incubated at 37 °C for 1 hour at 400 rpm. Next, a 0.30 M sodium azide stock solution (30 µL, 9.0 µmol, 0.59 mg, 1.8 × 10^3 equiv.) in NaP_i buffer (pH 7.0, c = 50 mM) was added to the mixture at 20–25 °C (c_{Nu} = 38 mM), followed by addition of 0.5 µL of a **$^{13}C_2$-VTT** stock solution (80 mM, 0.04 µmol, 0.01 mg, 8 equiv.) in DMF. The reaction mixture was vortexed for 1 second, transferred into a Thermocycler pre-heated at 25 °C, and incubated at 25 °C at 400 rpm for 120 minutes. Subsequently, 22 µL of a β-mercaptoethanol stock solution (0.15 M, 3.3 µmol, 6.6 × 10^2 equiv.) in UHQ-H_2O was added, and the mixture was incubated at 25 °C for 30 minutes. Next, the samples were prepared for LC-MS/MS analysis following the established standard SP3 protocol[13], using TCEP as reductant, chloroacetamide as alkylating reagent, and Trypsin + rLysC mix with a protein:trypsin ratio of 50:1 for digestion.

Preparation of conjugated heat-shock cell lysates with $^2H_3$ labeling

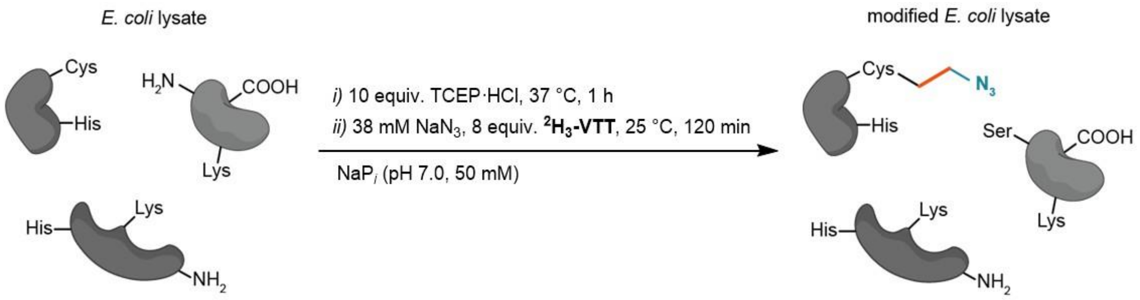

At 20–25 °C, 6.0 µL of heat-shock lysate (8.7 mg/mL, 7.8 × 10^2 µM Cys, 5.0 nmol Cys, 1.0 equiv.) in NaP_i buffer (pH 7.0, 50 mM) was added to a 1.5 mL Eppendorf tube and diluted with 0.13 mL of NaP_i buffer (pH 7.0, 50 mM). Subsequently, 5.0 µL of a TCEP stock solution (10 mM, 50 nmol, 13 µg, 10 equiv.) in UHQ-H_2O was added to the mixture. The mixture was vortexed for 1 second, transferred into a Thermocycler pre-heated at 37 °C, and incubated at 37 °C for 1 hour at 400 rpm. Next, a 0.30 M sodium azide stock solution (30 µL, 9.0 µmol, 0.59 mg, 1.8 × 10^3 equiv.) in NaP_i buffer (pH 7.0, c = 50 mM) was added to the mixture at 20–25 °C (c_{Nu} = 38 mM), followed by addition of 0.5 µL of a **$^2H_3$-VTT** stock solution (80 mM, 0.04 µmol, 0.01 mg, 8 equiv.) in DMF. The reaction mixture was vortexed for 1 second, transferred into a Thermocycler pre-heated at 25 °C, and incubated at 25 °C at 400 rpm for 120 minutes. Subsequently, 22 µL of a β-mercaptoethanol stock solution (0.15 M, 3.3 µmol, 6.6 × 10^2 equiv.) in UHQ-H_2O was added, and the mixture was incubated at 25 °C for 30 minutes. Next, the samples were prepared for LC-MS/MS analysis following the established standard SP3 protocol[13], using TCEP as reductant, chloroacetamide as alkylating reagent, and Trypsin + rLysC mix with a protein:trypsin ratio of 50:1 for digestion.

Preparation of cross-linked cell lysates with VTT

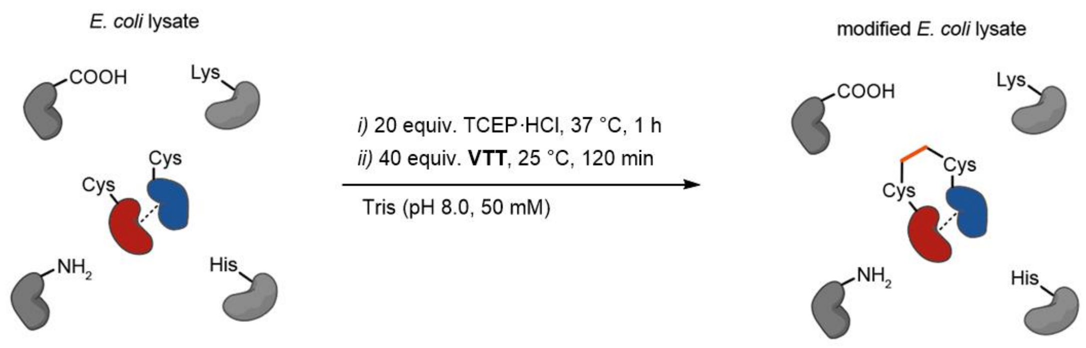

At 20–25 °C, 6.0 µL of a non-stressed control or heat-shock lysate (8.6 mg/mL, 7.8 × 10^2 µM Cys, 5.0 nmol Cys, 1.0 equiv.) in NaP_i buffer (pH 7.0, 50 mM) was added to a 1.5 mL Eppendorf tube and diluted with 0.18 mL of Tris buffer (pH 8.0, 50 mM). Subsequently, 10 µL of a TCEP stock solution (10 mM, 0.10 µmol, 25 µg, 20 equiv.) in Tris buffer (pH 8.0, 50 mM) was added to the mixture. The mixture was vortexed for 1 second, transferred into a Thermocycler pre-heated at 37 °C, and incubated at 37 °C for 1 hour at 400 rpm. Next, 2.5 µL of a **VTT** stock solution (80 mM, 0.20 µmol, 66 µg, 40 equiv.) in DMF was added to the mixture. The reaction mixture was vortexed for 1 second, transferred into a Thermocycler pre-heated at 25 °C, and incubated at 25 °C at 400 rpm for 120 minutes. Subsequently, 22 µL of a β-mercaptoethanol stock solution (0.15 M, 3.3 µmol, 6.6 × 10^2 equiv.) in UHQ-H_2O was added, and the mixture was incubated at 25 °C for 30 minutes. Next, the samples were prepared for LC-MS/MS analysis following the established standard SP3 protocol[13], using TCEP as reductant, chloroacetamide as alkylating reagent, and Trypsin + rLysC mix with a protein:trypsin ratio of 50:1 for digestion.

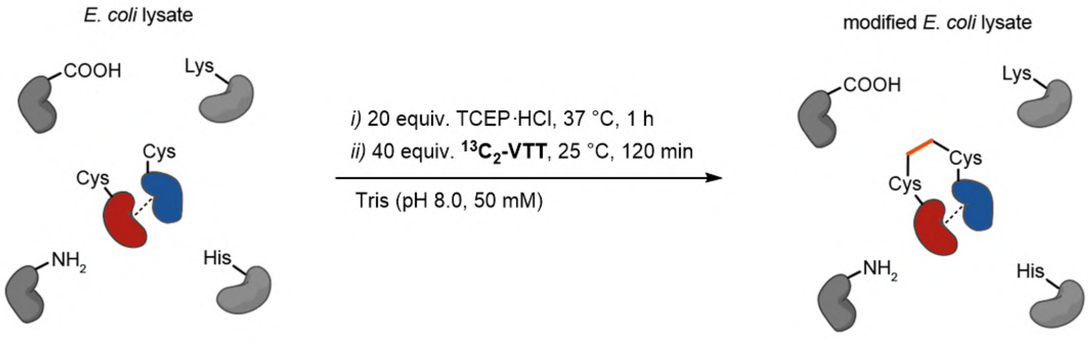

At 20–25 °C, 6.0 µL of a non-stressed or heat-shock lysate (8.6 mg/mL, 7.8 × 10^2 µM Cys, 5.0 nmol Cys, 1.0 equiv.) in NaP$_i$ buffer (pH 7.0, 50 mM) was added to a 1.5 mL Eppendorf tube and diluted with 0.18 mL of Tris buffer (pH 8.0, 50 mM). Subsequently, 10 µL of a TCEP stock solution (10 mM, 0.10 µmol, 25 µg, 20 equiv.) in Tris buffer (pH 8.0, 50 mM) was added to the mixture. The mixture was vortexed for 1 second, transferred into a Thermocycler pre-heated at 37 °C, and incubated at 37 °C for 1 hour at 400 rpm. Next, 2.5 µL of a **$^{13}C_2$-VTT** stock solution (80 mM, 0.20 µmol, 66 µg, 40 equiv.) in DMF was added to the mixture. The reaction mixture was vortexed for 1 second, transferred into a Thermocycler pre-heated at 25 °C, and incubated at 25 °C at 400 rpm for 120 minutes. Subsequently, 22 µL of a β-mercaptoethanol stock solution (0.15 M, 3.3 µmol, 6.6 × 10^2 equiv.) in UHQ-H_2O was added, and the mixture was incubated at 25 °C for 30 minutes. Next, the samples were prepared for LC-MS/MS analysis following the established standard SP3 protocol[13], using TCEP as reductant, chloroacetamide as alkylating reagent, and Trypsin + rLysC mix with a protein:trypsin ratio of 50:1 for digestion.

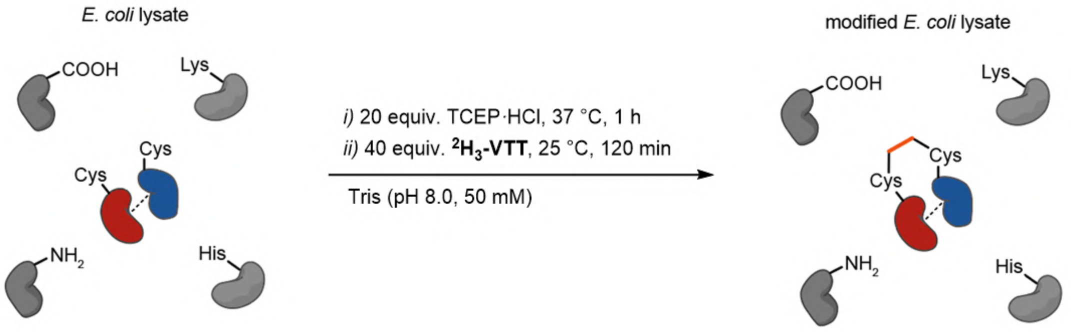

At 20–25 °C, 6.0 µL of a non-stressed or heat-shock lysate (8.6 mg/mL, 7.8 × 10^2 µM Cys, 5.0 nmol Cys, 1.0 equiv.) in NaP$_i$ buffer (pH 7.0, 50 mM) was added to a 1.5 mL Eppendorf tube and diluted with 0.18 mL of Tris buffer (pH 8.0, 50 mM). Subsequently, 10 µL of a TCEP stock solution (10 mM, 0.10 µmol, 25 µg, 20 equiv.) in Tris buffer (pH 8.0, 50 mM) was added to the mixture. The mixture was vortexed for 1 second, transferred into a Thermocycler pre-heated at 37 °C, and incubated at 37 °C for 1 hour at 400 rpm. Next, 2.5 µL of a **$^2H_3$-VTT** stock solution (80 mM, 0.20 µmol, 66 µg, 40 equiv.) in DMF was added to the mixture. The reaction mixture was vortexed for 1 second, transferred into a Thermocycler pre-heated at 25 °C, and incubated at 25 °C at 400 rpm for 120 minutes. Subsequently, 22 µL of a β-mercaptoethanol stock solution (0.15 M, 3.3 µmol, 6.6 × 10^2 equiv.) in UHQ-H_2O was added, and the mixture was incubated at 25 °C for 30 minutes. Next, the samples were prepared for LC-MS/MS analysis following the established standard SP3 protocol[13],

using TCEP as reductant, chloroacetamide as alkylating reagent, and Trypsin + rLysC mix with a protein:trypsin ratio of 50:1 for digestion.

LC-MS/MS analysis of heat-shock and control cell lysates

The samples for quantitative proteomics and cross-linking measurements were prepared by combination of labeled (heat-shock lysate) and unlabeled (non-stressed lysate) digested lysates. The concentration of each sample was assessed via A_{280} measurements. The samples were mixed in equimolar concentrations and analyzed via LC-MS/MS. Each labeled derivative, $^{13}C_2$ and $^{2}H_3$, respectively, was combined with the same non-labeled sample. The generated samples were measured in two technical replicates.

We have derived statistics from technical replicates because our goal was not to make new scientific claims based on the resulting statistics but to give possible applicants a mean to evaluate the robustness of the described method in the experimental sense (e.g. no decomposition of the analyzed species or strong deviations between different measurements caused by the formed species).

The Volcano-plot results from LIMMA-based[19] differential expression analysis with $p < 0.05$ derived from MS^1 intensities of identified peptides.

Processing of raw data was performed using the MaxQuant version 2.1.3.0. MS/MS spectra were assigned to the *E. coli* K12 proteome from Uniprot. During the search, sequences of 248 common contaminant proteins as well as decoy sequences were automatically added. Trypsin specificity was required and a maximum of two missed cleavages was allowed. Carbamidomethylation of cysteine residues was not included. Oxidation of methionine, and protein N-terminal acetylation were set as variable modifications. Modifications that resemble the respective modifications introduced by the vinyl-thianthrenium reagents or the respective isotopically labeled derivatives (D_3 or $^{13}C_2$) were defined and used as variable modifications in the respective searches. These were either $H_3C_2N_3$ on C, H, K, Y, or the N-terminus or $D_3C_2N_3$, $H_3{}^{13}C_2N_3$ on Cys only. A false discovery rate of 1% for peptide spectrum matches and proteins was applied.

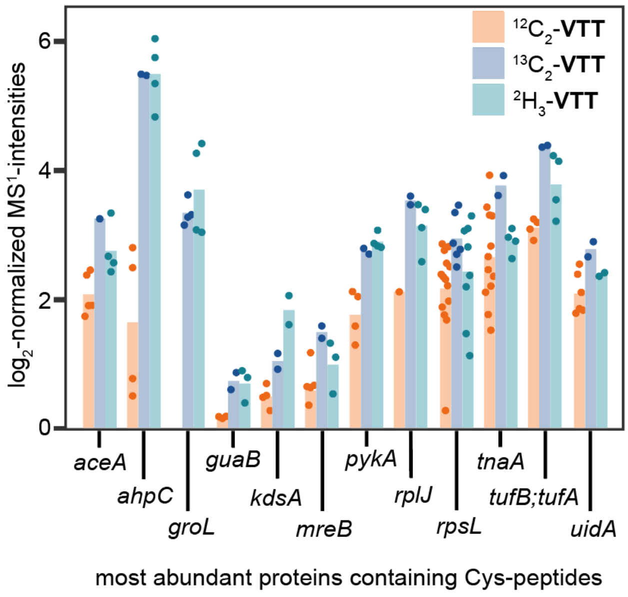

Figure S48. Mean of MS^1 intensities from Cys-containing peptides functionalized with isotopologues of **VTT** and their respective protein origins. The samples were measured with two technical replicates.

The chart above shows the quantitative results of LC-MS/MS analysis of the labeling experiments with *E. coli* cell lysates as described before. The control cell lysate was incubated at 37 °C and subsequently labeled with unlabeled **VTT**. The heat-shock cell lysates were incubated at 43 °C and were then labeled with the $^{2}H_{3}$- and $^{13}C_{2}$- isotopologues of **VTT**.

Error quantification by standard deviation was calculated based on the two technical replicates. The bars show the average of the two measurements. In cases where no error bar was plotted, the corresponding peptide was only found in one of the two measurements.

The volcano plot below represents the change of protein abundance between the heat-shock and control sample. Additionally, it provides the information whether the change in abundance can be considered mathematically significant. The border for significance is indicated as a solid horizontal line on the y-axis. The significance testing is based on a null-hypothesis testing with P equal to 0.05.

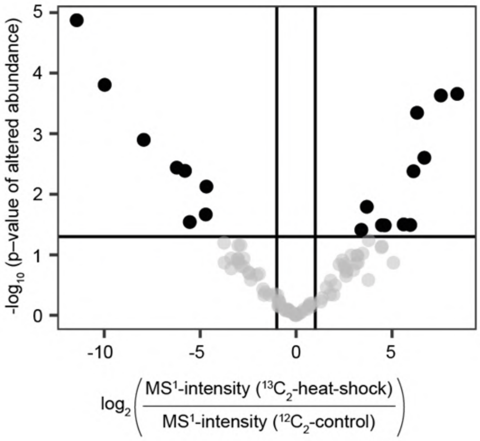

Figure S49. Volcano-plot resulting from two-sided LIMMA-based differential expression analysis[19] with $p < 0.05$ derived from MS^1 intensities of identified peptides based on two technical replicates. All p values are associated with empirical Bayes moderated *t*-statistics without adjustments for multiple comparisons.

LC-MS/MS analysis for cross-link search

For crosslink searches, apl files generated by MaxQuant and the *E. coli* K12 proteome from Uniprot were used as input for xiSEARCH 1.7.6.7. Asymmetric Single Amino Acid Restricted Cross Linkers bridging Cys to Cys, His, or Lys with the respective masses of vinyl-thianthrenium reagents were defined, taking into account the loss of 2 H and the respective D_3 and $^{13}C_2$ substitutions of the C_2H_4 unit.

The search lead to the candidate link of VANFCR to ACGVGK via the respective Cysteine residues and the corresponding stapled peptide was identified with each of the used vinylthianthrenium reagents. While the respective 2nd peptide contains a lysine, it is unlikely to be alkylated, as this would inhibit a tryptic cleavage at this particular position[20]. The search was repeated with Thermo Proteome Discoverer 3.0.1.27 and the xlinkx node, confirming the **VTT**-mediated VANFCR to ACGVGK Cys-Cys crosslink between the respective peptides / proteins:

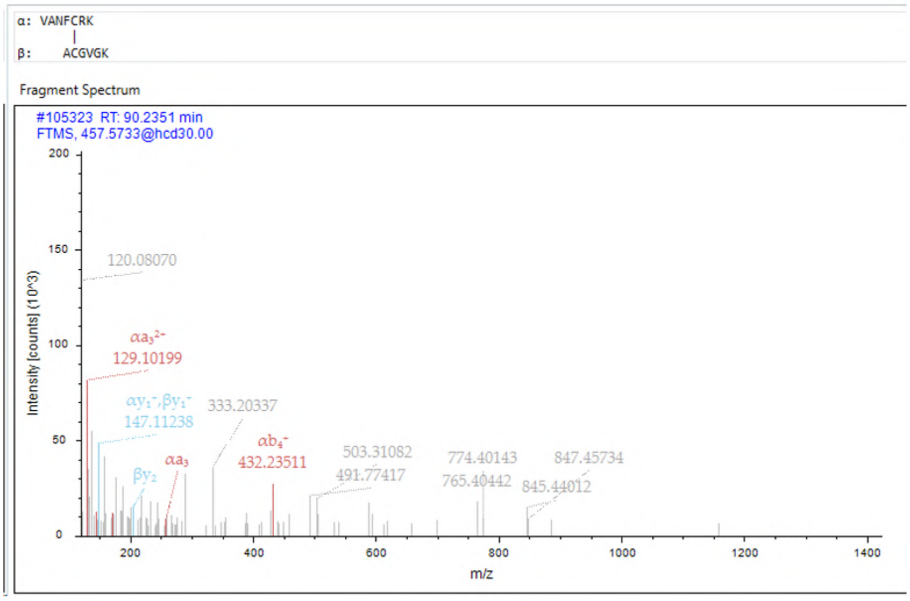

Figure S50. MS/MS spectrum of the identified cross-linked peptide.

MaxQuant output tables were imported to R 4.1.1 *via* Rstudio 2022.07.1 and visualized using ggplot2 version 3.3.6. For structural analysis, Alphafold[21] predictions were retrieved, for docking the respective Cys residues involved in a crosslink were set as active residues on the Haddock 2.4 webserver. Pymol 2.4 was used for visualization of the predicted protein and protein complex structures.

Protein-docking simulation

HADDOCK score	–100.1 ± 2.8
Van der Waals energy	–41.9 ± 5.6
Electrostatic energy	–336.4 ± 20.8

Data availability

MS raw data is available under the following link[22] for review and will be made publically available upon publication: https://repository.jpostdb.org/preview/169290337964832fb33c1bc

Protein scope for azidation

Synthesis of (sfGFP S147C)–S–C_2H_4–N_3 (2)

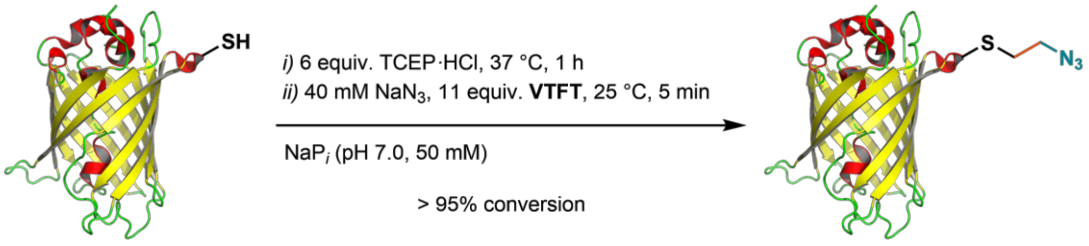

sfGFP S147C **2**

At 20–25 °C, 20 µL of sfGFP S147C (2.21 mg/mL, 75 µM, 1.5 nmol, 1.0 equiv.) in NaP_i buffer (pH 7.0,

c = 50 mM) was added to a 1.5 mL Eppendorf tube. Next, 66 µL of NaP$_i$ buffer (pH 7.0, c = 50 mM) was added. Then, 0.8 µL of a TCEP stock solution (10 mM, 9 nmol, 3 µg, 6 equiv.) in UHQ-H_2O was added. The mixture was vortexed for 1 second, transferred into a Thermocycler pre-heated at 37 °C, and incubated at 37 °C for 1 hour at 400 rpm. Next, a 0.30 M sodium azide stock solution (13 µL, 4.0 µmol, 0.26 mg, 2.7 × 10^3 equiv.) in NaP$_i$ buffer (pH 7.0, c = 50 mM) was added to the mixture at 20–25 °C (c_{Nu} = 40 mM), followed by addition of 0.7 µL of a **VTFT** stock solution (25 mM, 0.02 µmol, 7 µg, 1 × 10^1 equiv.) in DMF. The reaction mixture was vortexed for 1 second, transferred into a Thermocycler pre-heated at 25 °C, and incubated at 25 °C at 400 rpm for 5 minutes. Subsequently, 0.7 µL of a β-mercaptoethanol stock solution (25 mM, 0.02 µmol, 2 µg, 1 × 10^1 equiv.) in UHQ-H_2O was added, and the mixture was incubated at 25 °C for 15 minutes. The mixture was diluted with 0.23 mL of UHQ-H_2O to obtain a protein concentration of 0.14 mg/mL. The obtained mixture was analyzed by LC-MS.

HRMS-ESI (m/z) calc'd for $C_{1277}{}^{13}C_{17}H_{2025}N_{366}O_{389}S_9{}^{29+}$ [M–H_2O–H_2+29H$^+$]$^{29+}$, 1007.578; found, 1007.577; deviation: –1.0 ppm.

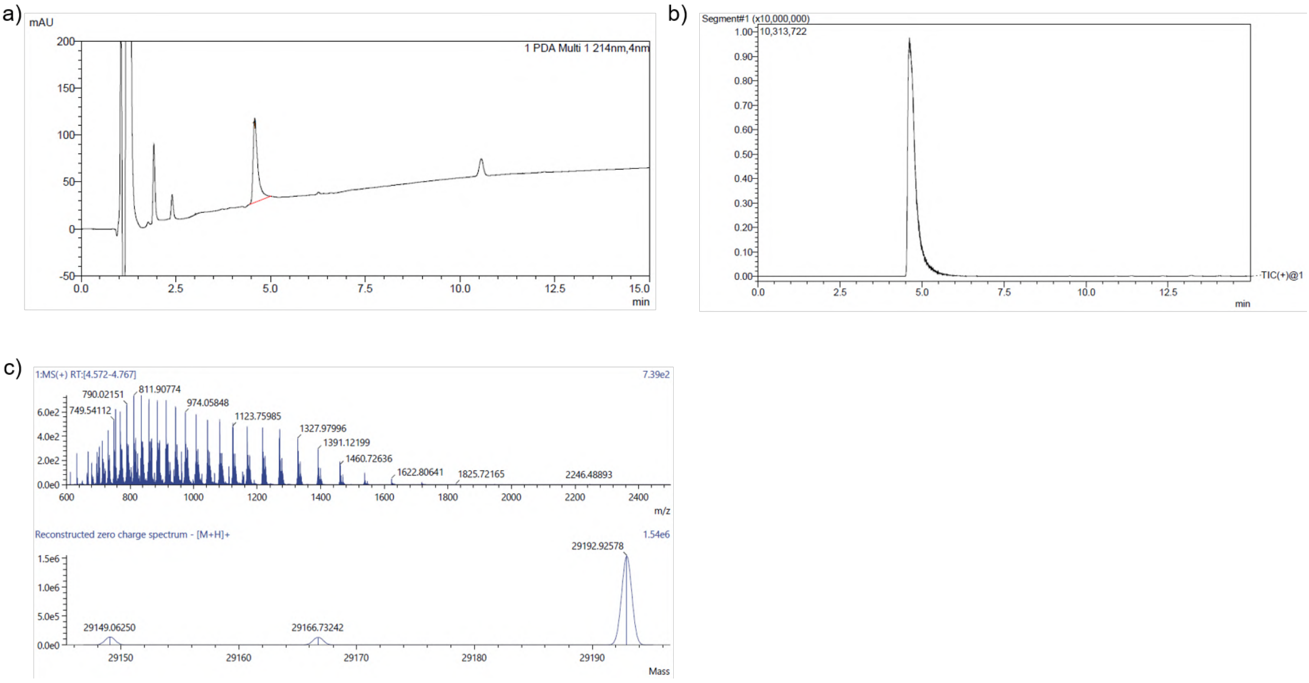

Figure S51. LC-MS analysis (Method B) of sfGFP S147C after reaction with **VTFT** and sodium azide. Protein concentration for analytical sample: 0.14 mg/mL. a) UV trace b) total ion chromatogram c) Ion series and deconvoluted spectrum; calculated masses: (sfGFP S147C)–S–C_2H_4–N_3: 29190 Da (peak detected in SM + 69 Da); (sfGFP S147C)–S–C_2H_4–OH: 29165 Da; stapled-(sfGFP S147C): 29146. Observed masses: 29149 Da, 29167 Da, 29193 Da.

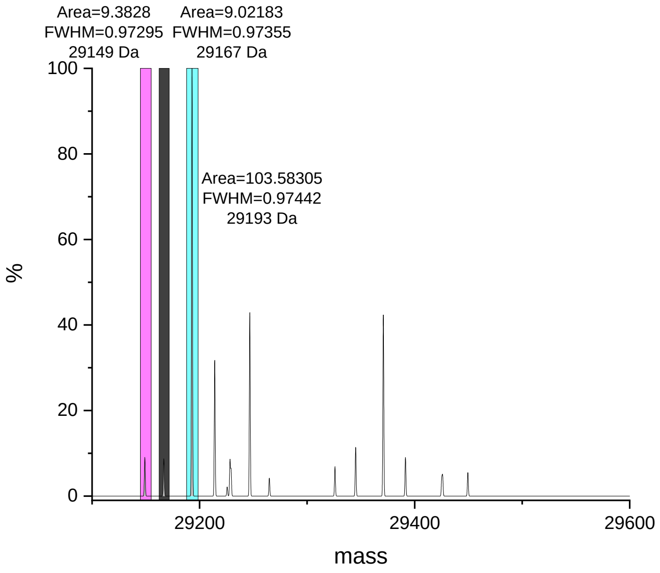

Figure S52. Zero-charge analysis of sfGFP S147C after reaction with **VTFT** and sodium azide for yield determination.

Synthesis of (DHAR2 C6S)–S–C_2H_4–N_3 (3)

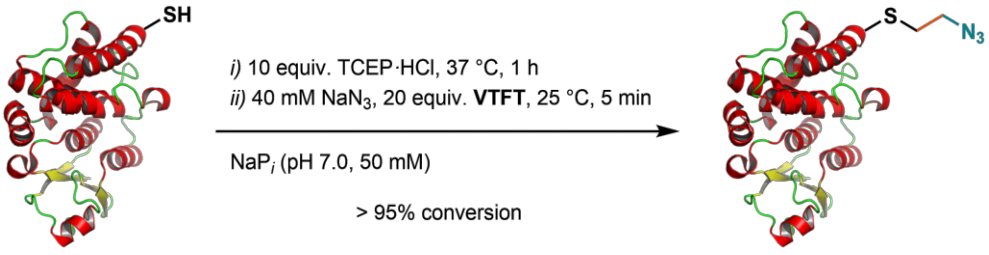

DHAR2 C6S **3**

At 20–25 °C, 17 µL of DHAR2 C6S (0.62 mg/mL, 24 µM, 0.40 nmol, 1.0 equiv.) in NaP*i* buffer (pH 7.0, c = 50 mM) was added to a 1.5 mL Eppendorf tube. Next, 26 µL of NaP*i* buffer (pH 7.0, c = 50 mM) was added. Then, 0.8 µL of a TCEP stock solution (5 mM, 4 nmol, 1 µg, 1 × 10^1 equiv.) in UHQ-H_2O was added. The mixture was vortexed for 1 second, transferred into a Thermocycler pre-heated at 37 °C, and incubated at 37 °C for 1 hour at 400 rpm. Next, a 0.30 M sodium azide stock solution (6.7 µL, 2.0 µmol, 0.30 mg, 5.× 10^3 equiv.) in NaP*i* buffer (pH 7.0, c = 50 mM) was added to the mixture at 20–25 °C (c_{Nu} = 40 mM), followed by addition of 0.5 µL of a **VTFT** stock solution (16 mM, 8 nmol, 3 µg, 2 × 10^1 equiv.) in DMF. The reaction mixture was vortexed for 1 second, transferred into a Thermocycler pre-heated at 25 °C, and incubated at 25 °C at 400 rpm for 5 minutes. The mixture was diluted with 24 µL of UHQ-H_2O to obtain a protein concentration of 0.14 mg/mL. The obtained mixture was analyzed by LC-MS.

HRMS-ESI (m/z) calc'd for $C_{1153}{}^{13}C_{15}H_{1849}N_{310}O_{342}S_3{}^{21+}$ $[M+21H^+]^{21+}$, 1228.649; found, 1228.649; deviation: 0.0 ppm.

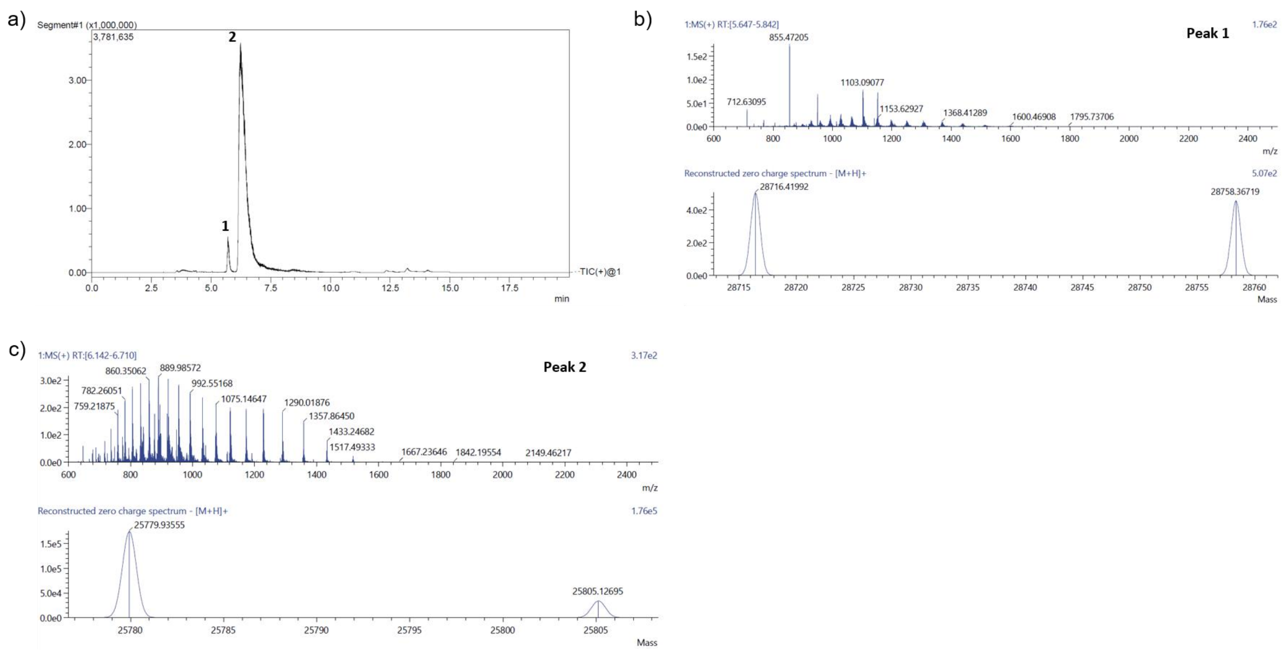

Figure S53. LC-MS analysis (Method B) of the collected product peak after reaction of DHAR2 C6S with **VTFT** and sodium azide. Protein concentration for analytical sample: 0.14 mg/mL. a) total ion chromatogram b) Peak 1 ion series and deconvoluted spectrum c) Peak 2 ion series and deconvoluted spectrum; calculated masses: (DHAR2 C6S)–S–C_2H_4–N_3: 25781 Da, 25803 Da (peaks detected in SM + 69 Da). Observed masses: 25780 Da, 25805 Da, 28716 Da, 28758 Da.

Synthesis of (Ubiquitin T9C)–S–C_2H_4–N_3 (4)

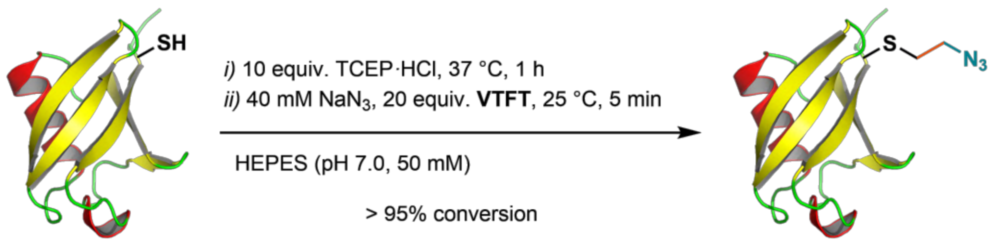

Ubiquitin T9C **4**

At 20–25 °C, 11 µL of Ubiquitin T9C (1.99 mg/mL, 221 µM, 2.5 nmol, 1.0 equiv.) in HEPES buffer (pH 7.0, c = 50 mM) was added to a 1.5 mL Eppendorf tube. Next, 73 µL of HEPES buffer (pH 7.0, c = 50 mM) was added. Then, 2.5 µL of a TCEP stock solution (10 mM, 25 nmol, 7.2 µg, 10 equiv.) in UHQ-H_2O was added. The mixture was vortexed for 1 second, transferred into a Thermocycler pre-heated at 37 °C, and incubated at 37 °C for 1 hour at 400 rpm. Next, a 0.30 M sodium azide stock solution (13 µL, 4.0 µmol, 0.60 mg, 1.6 × 10^3 equiv.) in HEPES buffer (pH 7.0, c = 50 mM) was added to the mixture at 20–25 °C (c_{Nu} = 40 mM), followed by addition of 1.0 µL of a **VTFT** stock solution (50 mM, 50 nmol, 20 µg, 20 equiv.) in DMF. The reaction mixture was vortexed for 1 second, transferred into a Thermocycler pre-heated at 25 °C, and incubated at 25 °C at 400 rpm for 5 minutes. Subsequently, 1.0 µL of a β-mercaptoethanol stock solution (50 mM, 50 nmol, 20 equiv.) in UHQ-H_2O was added, and the mixture was incubated at 25 °C for 15 minutes. The obtained mixture was analyzed by LC-MS.

HRMS-ESI (m/z) calc'd for $C_{391}{}^{13}C_5H_{667}N_{115}O_{124}S_2{}^{12+}$ $[M+12H^+]^{12+}$, 757.2414; found, 757.2415; deviation: +0.1 ppm.

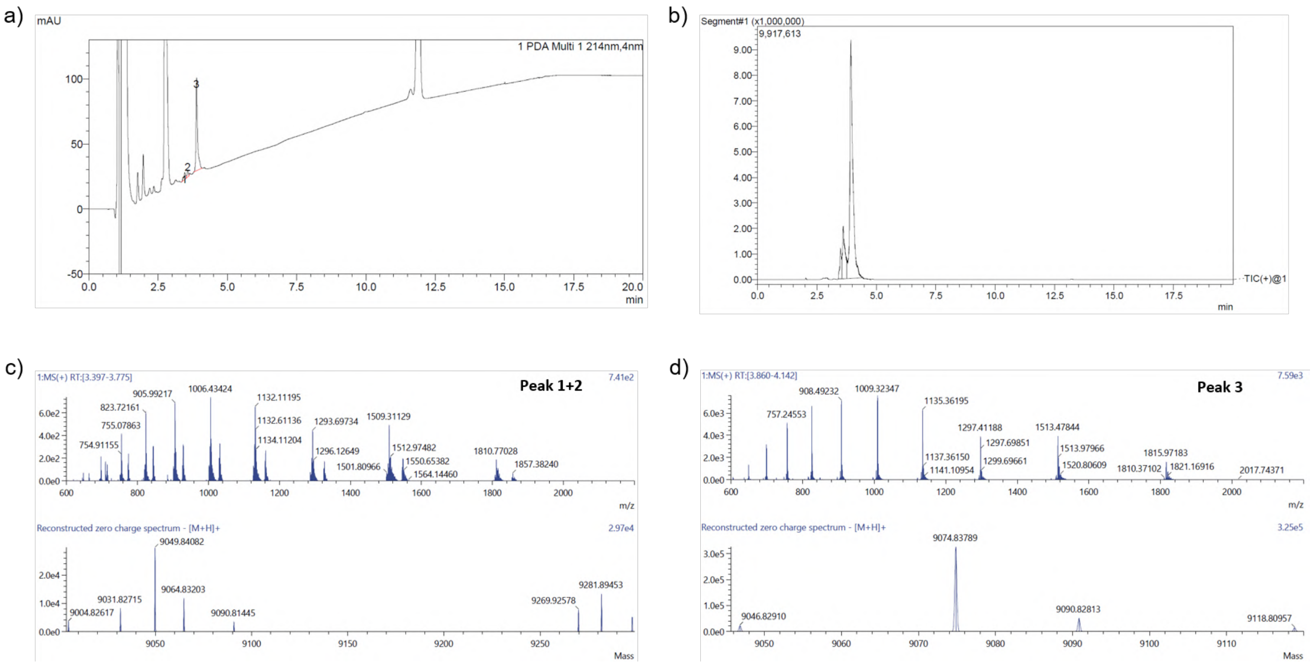

Figure S54. LC-MS analysis (Method B) of Ubiquitin T9C after reaction with **VTFT** and sodium azide: a) UV-trace b) total ion chromatogram c) Peak 1+2 ion series and deconvoluted spectrum d) Peak 3 ion series and deconvoluted spectrum; calculated masses: (Ubiquitin T9C)–S–C2H4–N3: 9075 Da, 9091 Da, 9047 Da (peaks detected in SM + 69 Da); (Ubiquitin T9C)–S–C2H4–OH: 9050 Da; (Ubiquitin T9C)–S–C2H4–HEPES: 9270 Da; stapled-(Ubiquitin T9C): 9032 Da. Observed masses: Peak 1+2: 9050 Da, 9032 Da, 9270 Da; Peak 3: 9047 Da, 9075 Da, 9091 Da.

Synthesis of (PrxIIB C76S)–S–C_2H_4–N_3 (5)

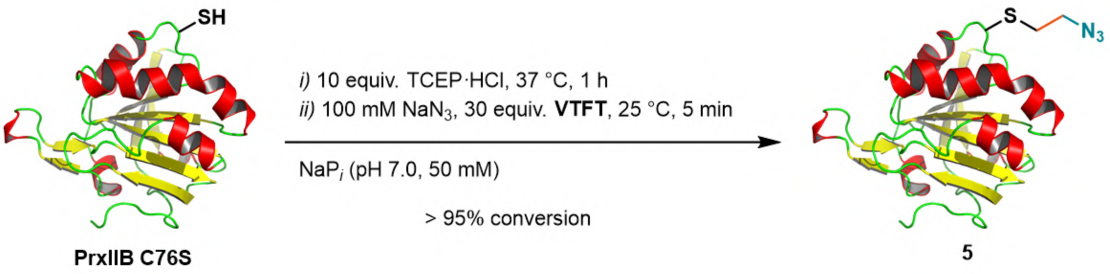

At 20–25 °C, 12 µL of PrxIIB C76S (1.63 mg/mL, 83 µM, 1.0 nmol, 1.0 equiv.) in NaP*i* buffer (pH 7.0, c = 50 mM) was added to a 1.5 mL Eppendorf tube. Next, 54 µL of NaP*i* buffer (pH 7.0, c = 50 mM) was added. Then, 0.5 µL of a TCEP stock solution (10 mM, 0.01 µmol, 3 µg, 1 × 10^1 equiv.) in UHQ-H_2O was added. The mixture was vortexed for 1 second, transferred into a Thermocycler pre-heated at 37 °C, and incubated at 37 °C for 1 hour at 400 rpm. Next, a 0.30 M sodium azide stock solution (33 µL, 10 µmol, 0.65 mg, 10 × 10^4 equiv.) in NaP*i* buffer (pH 7.0, c = 50 mM) was added to the mixture at 20–25 °C (c_{Nu} = 100 mM), followed by addition of 0.8 µL of a **VTFT** stock solution (40 mM, 3 × 10^1 nmol, 1 × 10^1 µg, 3 × 10^1 equiv.) in DMF. The reaction mixture was vortexed for 1 second, transferred into a Thermocycler pre-heated at 25 °C, and incubated at 25 °C at 400 rpm for 5 minutes. Subsequently, 0.8 µL of a β-mercaptoethanol stock solution (40 mM, 3 × 10^1 nmol, 3 × 10^1 equiv.) in UHQ-H_2O was added, and the mixture was incubated at 25 °C for 15 minutes. The mixture was diluted with 45 µL of UHQ-H_2O to obtain a protein concentration of 0.13 mg/mL. The obtained mixture was analyzed by LC-MS.

HRMS-ESI (m/z) calc'd for $C_{862}{}^{13}C_{11}H_{1381}N_{239}O_{261}S_4{}^{16+}$ $[M+16H^+]^{16+}$, 1220.508; found, 1220.508; deviation: 0.0 ppm.

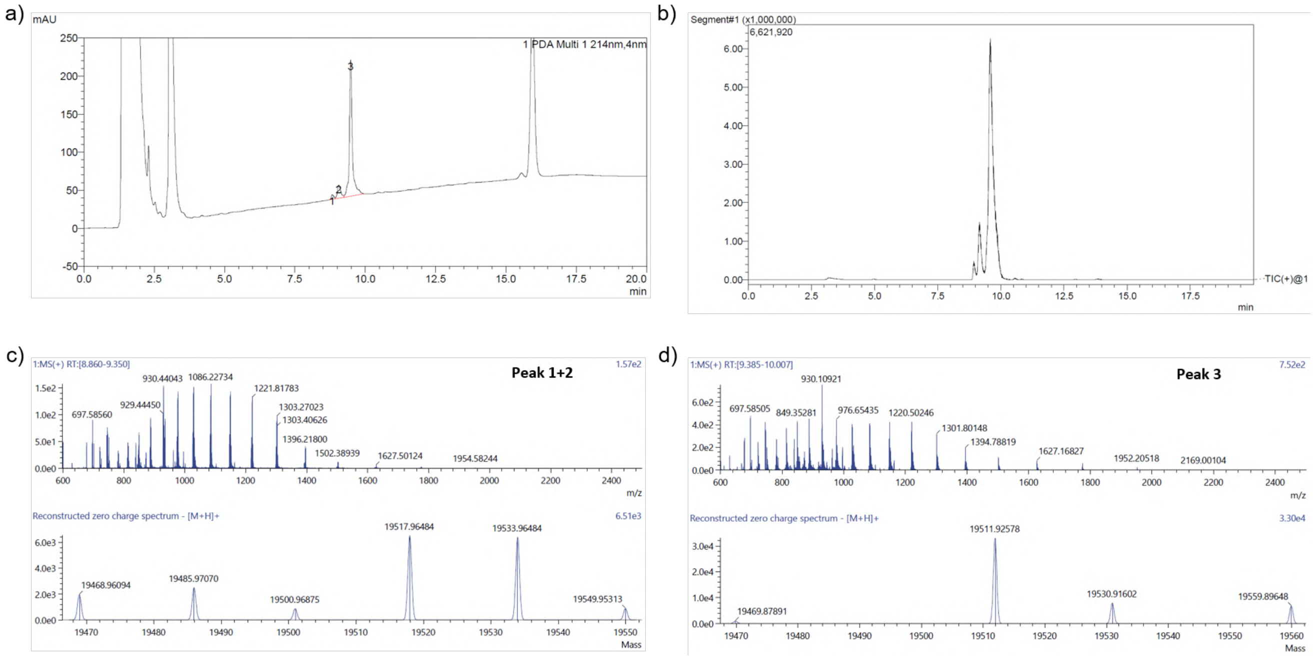

Figure S55. LC-MS analysis (Method C) of PrxIIB C76S after reaction with **VTFT** and sodium azide: a) UV-trace b) total ion chromatogram c) Peak 1+2 ion series and deconvoluted spectrum d) Peak 3 ion series and deconvoluted spectrum; calculated masses: (PrxIIB C76S)–S–C_2H_4–N_3: 19513 Da, 19531 Da, 19470 Da (peaks detected in SM + 69 Da); (PrxIIB C76S)–S–C_2H_4–OH: 19488 Da; stapled-(PrxIIB C76S): 19488 Da. Observed masses: Peak 1+2: 19486 Da; Peak 3: 19470 Da, 19512 Da, 19531 Da, 19560 Da.

Synthesis of (MDAR3)–S–C_2H_4–N_3 (6)

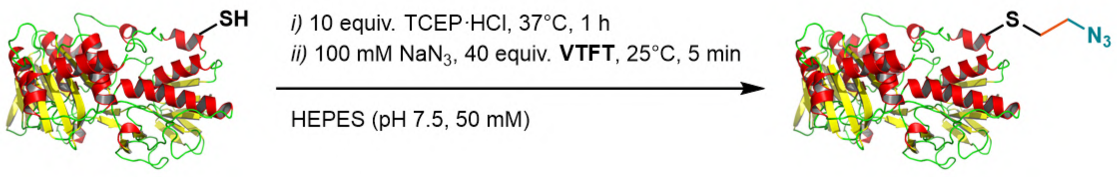

MDAR3 **6**

At 20–25 °C, 10 µL of MDAR3 (2.01 mg/mL, 39 µM, 0.40 nmol, 1.0 equiv.) in HEPES buffer (pH 7.5, c = 50 mM) was added to a 1.5 mL Eppendorf tube. Next, 22 µL HEPES buffer (pH 7.5, c = 50 mM) was added. Then, 0.8 µL of a TCEP stock solution (5 mM, 4 nmol, 1 µg, $1x10^1$ equiv.) in UHQ-H_2O was added. The mixture was vortexed for 1 second, transferred into a Thermocycler pre-heated at 25 °C, and incubated at 25 °C for 1 hour at 400 rpm. Next, a 0.3 M sodium azide solution (17 µL, 5.0 µmol, 0.33 mg, $13x10^4$ equiv.) in HEPES buffer (pH 7.5, c = 50 mM) was added to the mixture at 20–25 °C (c_{Nu} = 100 mM), followed by addition of 1.0 µL of a **VTFT** stock solution (16 mM, 16 nmol, 5.3 µg, 40 equiv.) in DMF. The reaction mixture was vortexed for 1 second, transferred into a Thermocycler pre-heated at 25 °C, and incubated at 25 °C at 400 rpm for 5 minutes. Subsequently, 1.0 µL of a β-mercaptoethanol stock solution (16 mM, 16 nmol, 1.5 µg, 40 equiv.) in UHQ-H_2O was added, and the mixture was incubated at 25 °C for 15 minutes. The obtained mixture was analyzed by LC-MS.

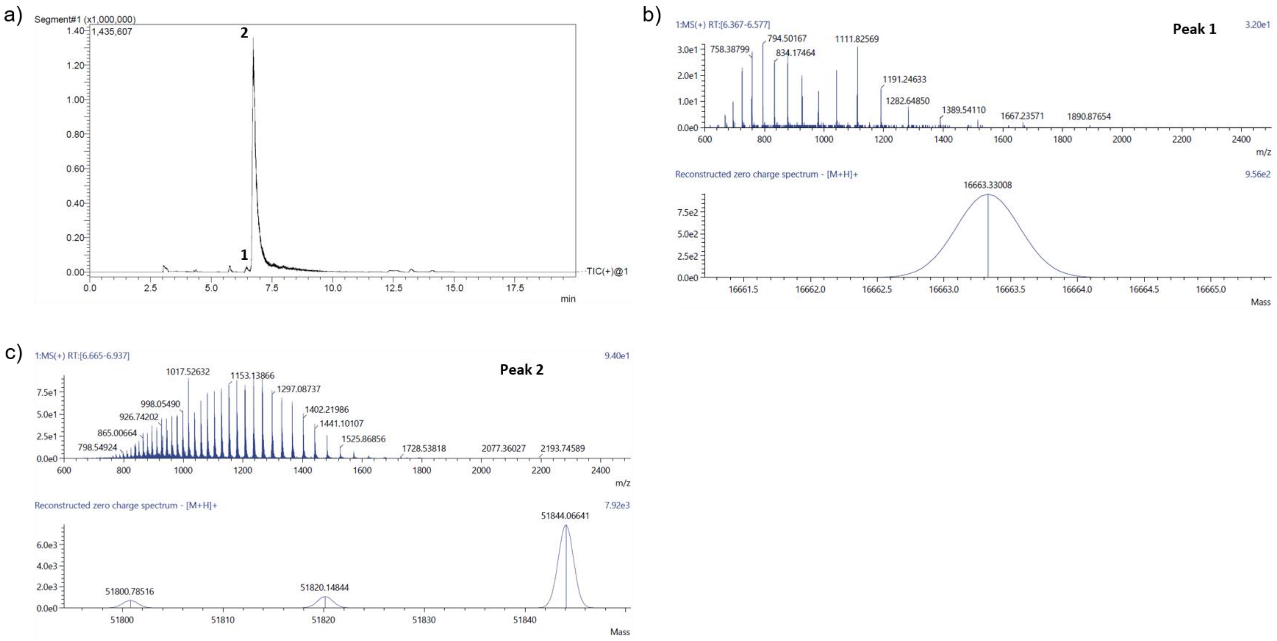

Figure S56. LC-MS analysis (Method B) of MDAR3 after reaction with **VTFT** and sodium azide: a) total ion chromatogram b) Peak 1 ion series and deconvoluted spectrum c) Peak 2 ion series and deconvoluted spectrum; calculated mass: (MDAR3)–S–C_2H_4–N_3: 51844 Da, (MDAR3)–S–C_2H_4–OH: 51819 Da, stapled-(MDAR3): 51801 Da; Observed masses: Peak 2: 51801 Da, 51820 Da, 51844 Da.

Synthesis of (BSA)–S–C_2H_4–N_3 (7)

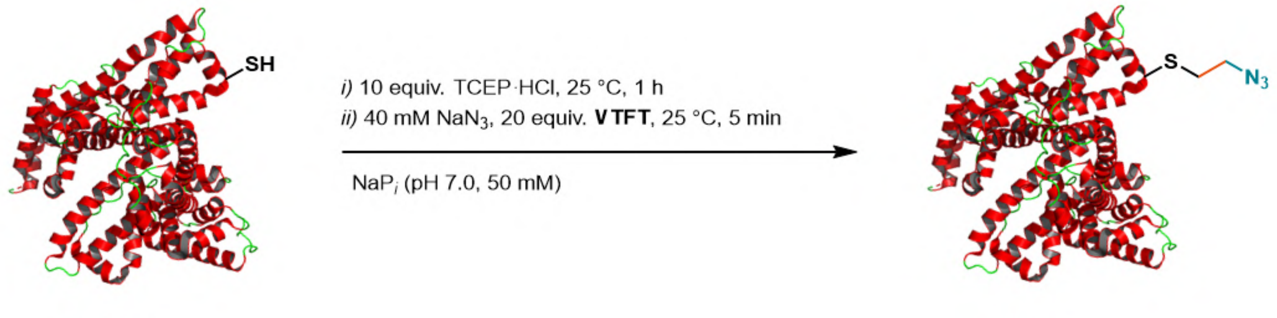

BSA **7**

At 20–25 °C, 13 µL of BSA (2.00 mg/mL, 30.0 µM, 0.40 nmol, 1.0 equiv.) in NaP*i* buffer (pH 7.0, c = 50 mM) was added to a 1.5 mL Eppendorf tube. Next, 29 µL NaP*i* buffer (pH 7.0, c = 50 mM) was added. Then, 0.8 µL of a TCEP stock solution (0.08 mM, 4 nmol, 1 µg, $1x10^1$ equiv.) in UHQ-H_2O was added. The mixture was vortexed for 1 second, transferred into a Thermocycler pre-heated at 37 °C, and incubated at 25 °C for 1 hour at 400 rpm. Next, a 0.3 M sodium azide solution (6.7 µL, 2.0 µmol, 0.13 mg, $50x10^2$ equiv.) in NaP*i* buffer (pH 7.0, c = 50 mM) was added to the mixture at 20–25 °C (c_{Nu} = 40 mM), followed by addition of 0.5 µL of a **VTFT** stock solution (16 mM, 8 nmol, 3 µg, $2x10^1$ equiv.) in DMF. The reaction mixture was vortexed for 1 second, transferred into a Thermocycler pre-heated at 25 °C, and incubated at 25 °C at 400 rpm for 5 minutes. Subsequently, 0.5 µL of a β-mercaptoethanol stock solution (16 mM, 8 nmol, 0.7 µg, $2x10^1$ equiv.) in UHQ-H_2O was added, and the mixture was incubated at 25 °C for 15 minutes. The obtained mixture was analyzed by LC-MS.

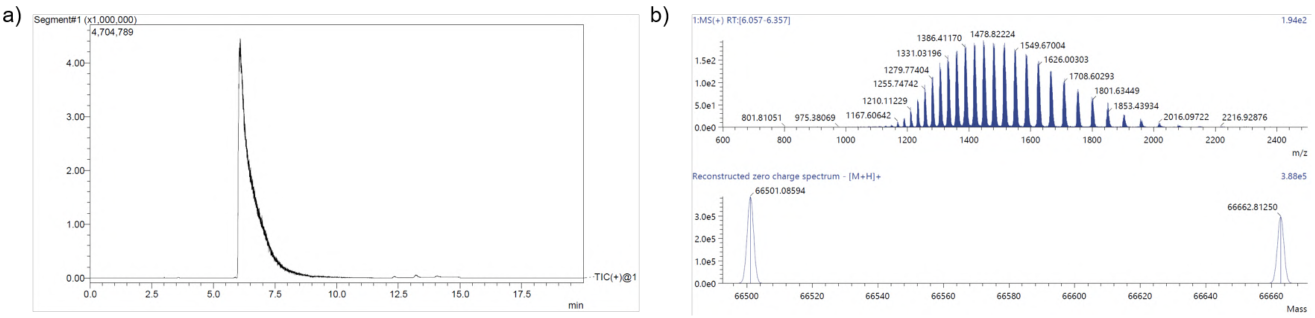

Figure S57. LC-MS analysis (Method B) of BSA after reaction with **VTFT** and sodium azide: a) total ion chromatogram b) ion series and deconvoluted spectrum; calculated mass: (BSA)–S–C_2H_4–N_3: 66499 Da, 66660 Da (peaks detected in SM + 69 Da); observed masses: 66501 Da, 66663 Da.

Synthesis of (Trxh1 C43S)–S–C_2H_4–N_3 (8)

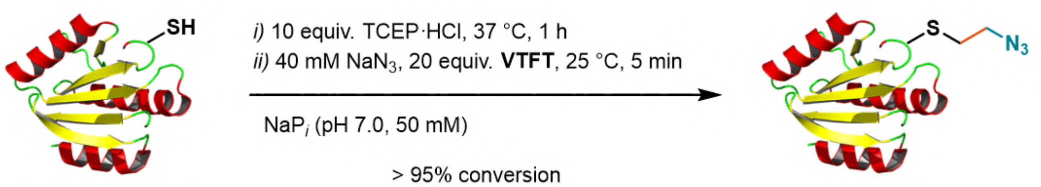

Trxh1 C43S **8**

At 20–25 °C, 28 µL of Trxh1 C43S (1.07 mg/mL, 72 µM, 2.0 nmol, 1.0 equiv.) in NaP$_i$ buffer (pH 7.0, c = 50 mM) was added to a 1.5 mL Eppendorf tube. Next, 57 µL NaP$_i$ buffer (pH 7.0, c = 50 mM) was added. Then, 2.0 µL of a TCEP stock solution (10 mM, 20 nmol, 5.7 µg, 10 equiv.) in UHQ-H_2O was added. The mixture was vortexed for 1 second, transferred into a Thermocycler pre-heated at 37 °C, and incubated at 37 °C for 1 hour at 400 rpm. Next, a 0.30 M sodium azide stock solution (13 µL, 4.0 µmol, 0.26 mg, 2.0 × 10^3 equiv.) in NaP$_i$ buffer (pH 7.0, c = 50 mM) was added to the mixture at 20–25 °C (c_{Nu} = 40 mM), followed by addition of 0.8 µL of a **VTFT** stock solution (50 mM, 0.04 µmol, 2 × 10^1 µg, 2 × 10^1 equiv.) in DMF. The reaction mixture was vortexed for 1 second, transferred into a Thermocycler pre-heated at 25 °C, and incubated at 25 °C at 400 rpm for 5 minutes. Subsequently, 0.8 µL of a β-mercaptoethanol stock solution (50 mM, 50 nmol, 4.8 µg, 80 equiv.) in UHQ-H_2O was added, and the mixture was incubated at 25 °C for 15 minutes The obtained mixture was analyzed by LC-MS.

HRMS-ESI (m/z) calc'd for $C_{652}{}^{13}C_9H_{1049}N_{183}O_{191}S_5{}^{18+}$ $[M+18H^+]^{16+}$, 820.8711; found, 820.8704; deviation: –0.9 ppm.

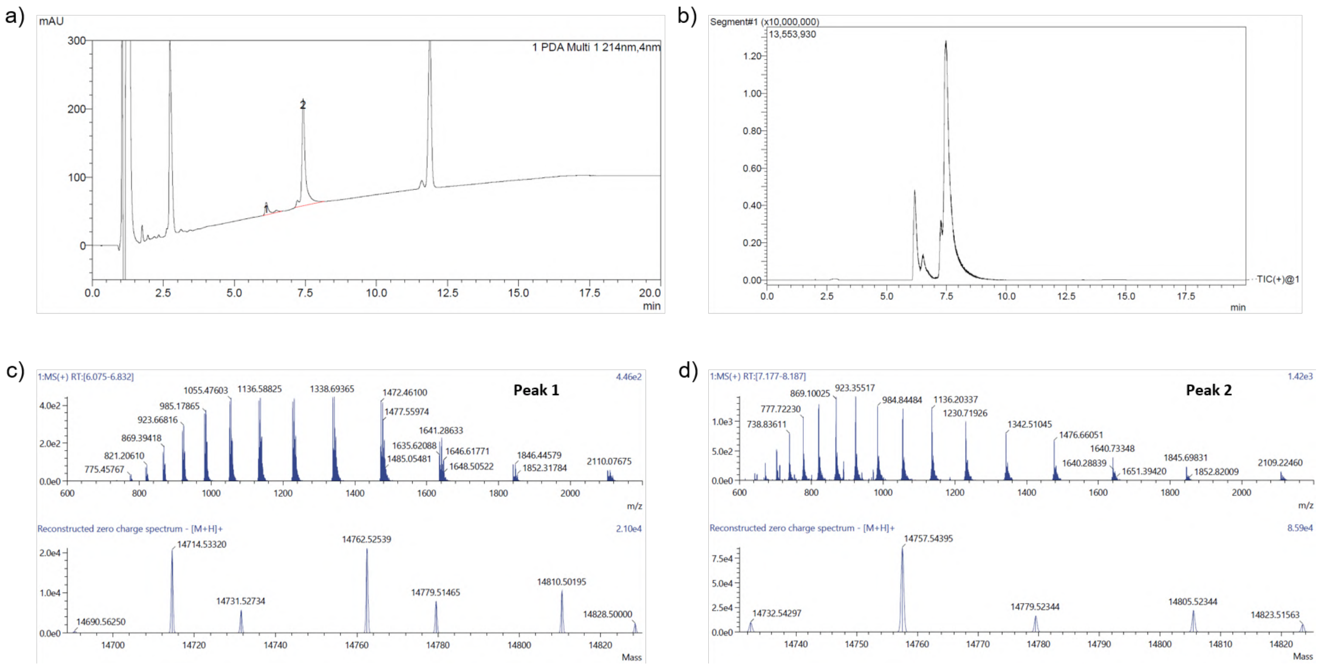

Figure S58. LC-MS analysis (Method B) of Trxh1 C43S after reaction with **VTFT** and sodium azide: a) UV-trace b) total ion chromatogram c) Peak 1 ion series and deconvoluted spectrum d) Peak 2 ion series and deconvoluted spectrum; calculated masses: (Trxh1 C43S)–S–C_2H_4–N_3: 14735 Da, 14756 Da, 14804 Da (peaks detected in SM + 69 Da); (Trxh1 C43S)–S–C_2H_4–OH: 14731 Da; stapled-(Trxh1 C43S): 14713 Da. Observed masses: Peak 1: 14715 Da, 14732 Da; Peak 2: 14733 Da, 14758 Da, 14806 Da.

Synthesis of (DHAR1 S97C)–S–C_2H_4–N_3 (9)

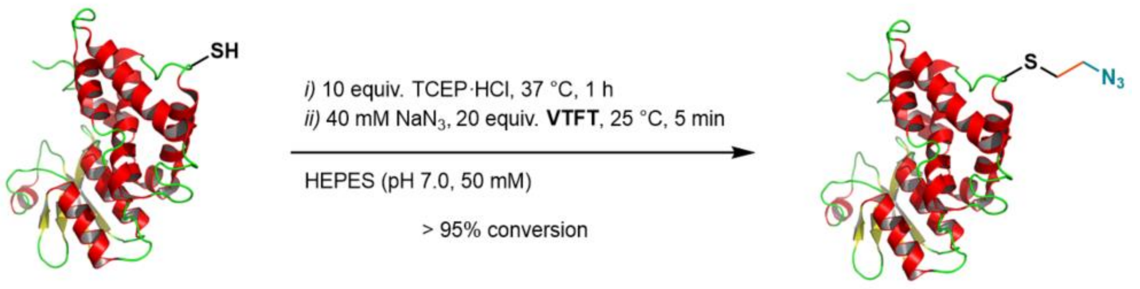

DHAR1 S97C **9**

At 20–25 °C, 23 µL of DHAR1 S97C (1.14 mg/mL, 44 µM, 1.0 nmol, 1.0 equiv.) in HEPES buffer (pH 7.0, c = 50 mM) was added to a 1.5 mL Eppendorf tube. Next, 63 µL HEPES buffer (pH 7.0, c = 50 mM) was added. Then, 1.0 µL of a TCEP stock solution (10 mM, 10 nmol, 2.9 µg, 10 equiv.) in UHQ-H_2O was added. The mixture was vortexed for 1 second, transferred into a Thermocycler pre-heated at 37 °C, and incubated at 37 °C for 1 hour at 400 rpm. Next, a 0.3 M sodium azide solution (13 µL, 4.0 µmol, 0.33 mg, 40x10^2 equiv.) in HEPES buffer (pH 7.0, c = 50 mM) was added to the mixture at 20–25 °C (c_{Nu} = 40 mM), followed by addition of 1.0 µL of a **VTFT** stock solution (20 mM, 20 nmol, 6.6 µg, 20 equiv.) in DMF. The reaction mixture was vortexed for 1 second, transferred into a Thermocycler pre-heated at 25 °C, and incubated at 25 °C at 400 rpm for 5 minutes. Subsequently, 1.0 µL of a β-mercaptoethanol stock solution (20 mM, 20 nmol, 1.9 µg, 20 equiv.) in UHQ-H_2O was added, and the mixture was incubated at 25 °C for 15 minutes. The obtained mixture was analyzed by LC-MS.

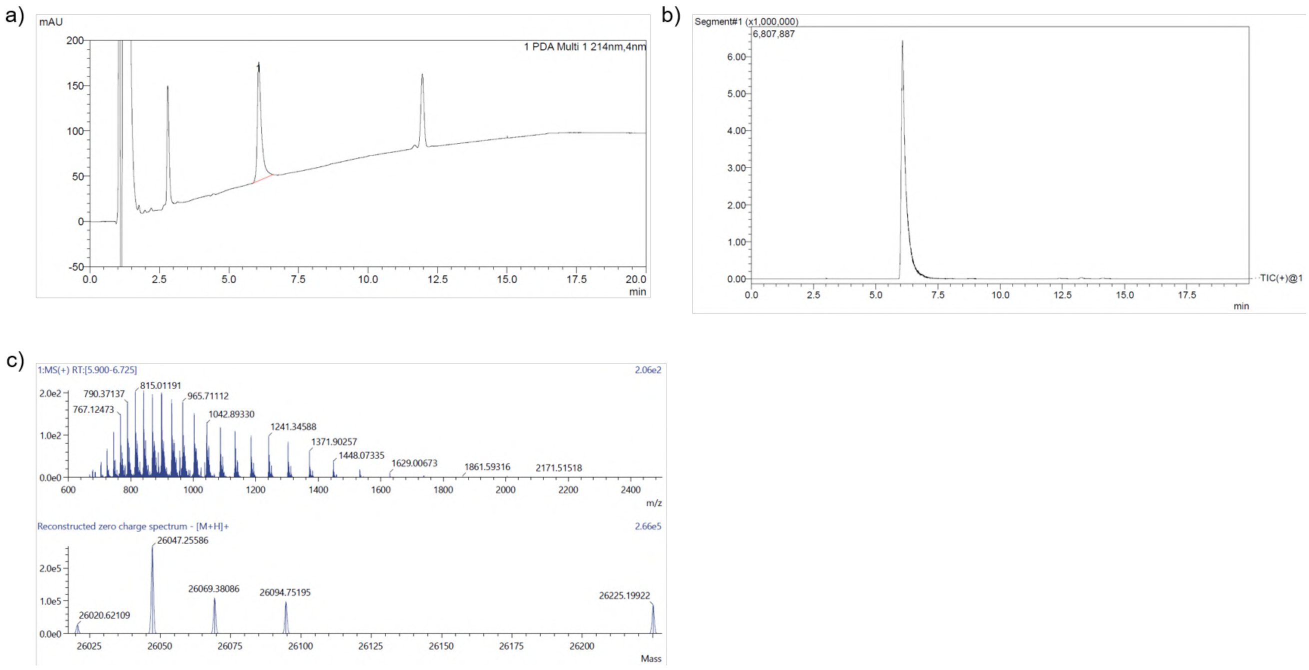

Figure S59. LC-MS analysis (Method B) of DHAR1 S97C after reaction with **VTFT** and sodium azide: a) UV trace b) total ion chromatogram c) Ion series and deconvoluted spectrum; calculated masses: (DHAR1 S97C)–S–C_2H_4–N_3: 26047 Da, 26069 Da, 26095 Da, 26225 Da, (DHAR1 S97C)–S–C_2H_4–OH: 26022 Da; Observed masses: 26021 Da, 26047 Da, 26069 Da, 26095 Da, 26225 Da.

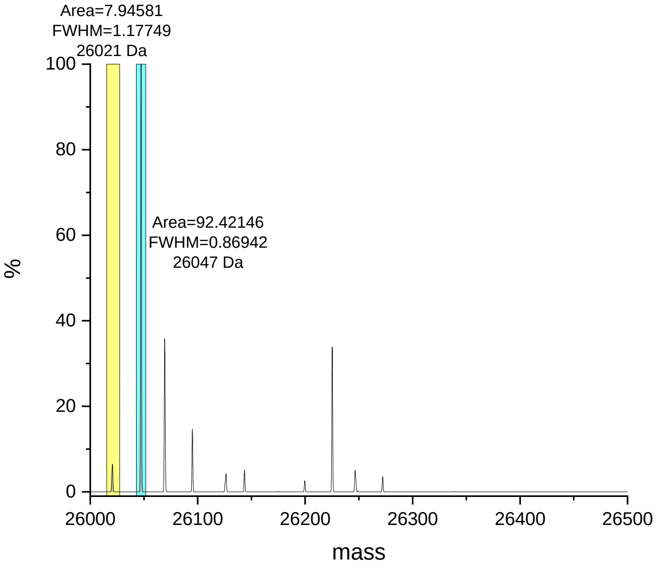

Figure S60. Zero-charge analysis of DHAR1 S97C after reaction with **VTFT** and sodium azide for yield determination.

Synthesis of (DHAR1 S176C)–S–C_2H_4–N_3 (10)

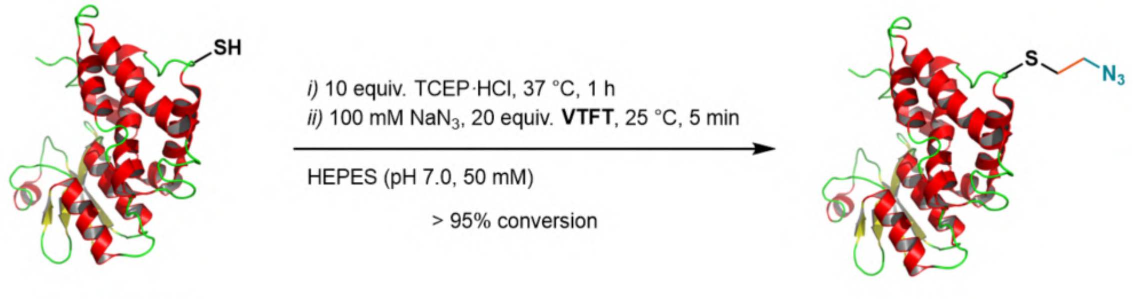

DHAR1 S176C **10**

At 20–25 °C, 23 µL of DHAR1 S176C (1.18 mg/mL, 45 µM, 1.0 nmol, 1.0 equiv.) in HEPES buffer (pH 7.0, c = 50 mM) was added to a 1.5 mL Eppendorf tube. Next, 64 µL HEPES buffer (pH 7.0, c = 50 mM) was added. Then, 1.0 µL of a TCEP stock solution (10 mM, 10 nmol, 2.9 µg, 10 equiv.) in UHQ-H_2O was added. The mixture was vortexed for 1 second, transferred into a Thermocycler pre-heated at 37 °C, and incubated at 37 °C for 1 hour at 400 rpm. Next, a 0.3 M sodium azide solution (33 µL, 10 µmol, 0.65 mg, 10x10^3 equiv.) in HEPES buffer (pH 7.0, c = 50 mM) was added to the mixture at 20–25 °C (c_{Nu} = 100 mM), followed by addition of 1.0 µL of a **VTFT** stock solution (20 mM, 20 nmol, 6.6 µg, 20 equiv.) in DMF. The reaction mixture was vortexed for 1 second, transferred into a Thermocycler pre-heated at 25 °C, and incubated at 25 °C at 400 rpm for 5 minutes. Subsequently, 1.0 µL of a β-mercaptoethanol stock solution (20 mM, 20 nmol, 1.9 µg, 20 equiv.) in UHQ-H_2O was added, and the mixture was incubated at 25 °C for 15 minutes. The obtained mixture was analyzed by LC-MS.

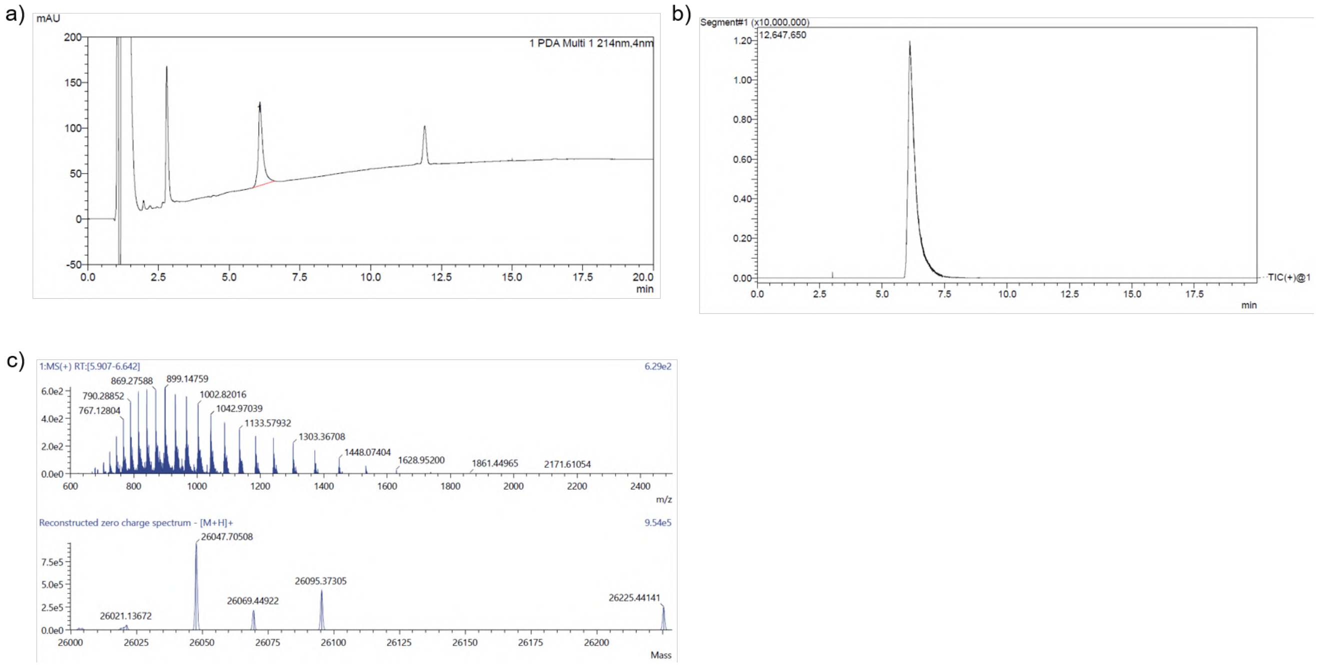

Figure S61. LC-MS analysis (Method B) of DHAR1 S176C after reaction with **VTFT** and sodium azide: a) UV trace b) total ion chromatogram c) Ion series and deconvoluted spectrum; calculated masses: (DHAR1 S176C)–S–C_2H_4–N_3: 26047 Da, 26067 Da, 26093 Da, 26225 Da (peaks observed in the starting material + 69 Da); (DHAR1 S176C)–S–C_2H_4–OH: 26022 Da, stapled-(DHAR1 S176C): 26004 Da; Observed masses: 26004 Da, 26021 Da, 26048 Da, 26069 Da, 26095 Da, 26225 Da.

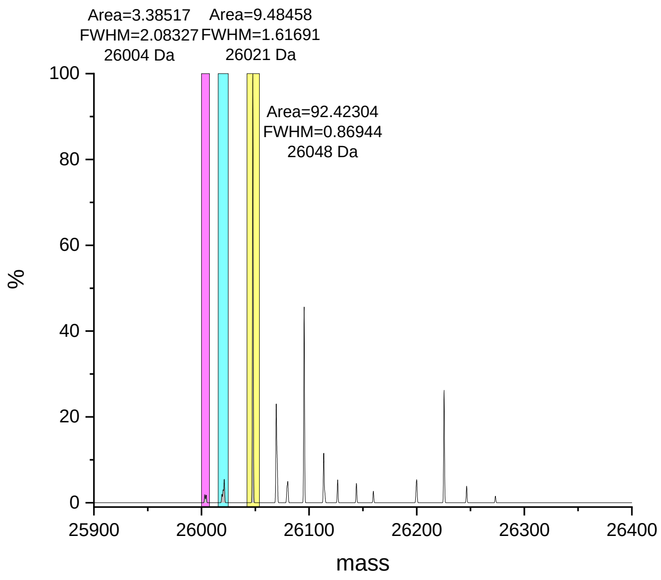

Figure S62. Zero-charge analysis of DHAR1 S176C after reaction with **VTFT** and sodium azide for yield determination.

Synthesis of (MDAR2)–S–C_2H_4–N_3 (S13)

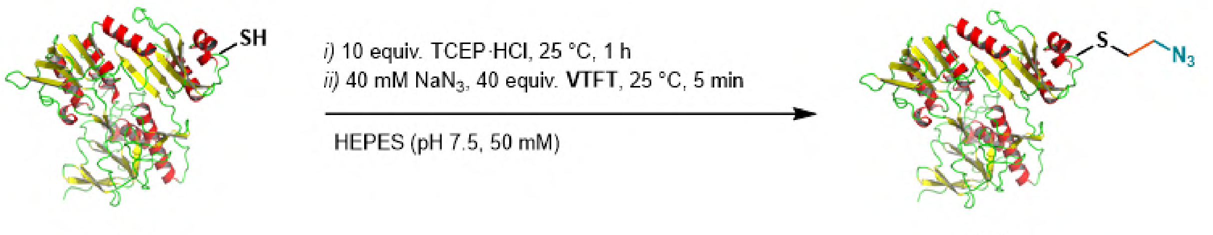

At 20–25 °C, 17 µL of MDAR2 (1.21 mg/mL, 24 µM, 0.40 nmol, 1.0 equiv.) in HEPES buffer (pH 7.5, c = 50 mM) was added to a 1.5 mL Eppendorf tube. Next, 26 µL HEPES buffer (pH 7.5, c = 50 mM) was added. Then, 0.8 µL of a TCEP stock solution (0.08 mM, 4 nmol, 1 µg, $1x10^1$ equiv.) in UHQ-H_2O was added. The mixture was vortexed for 1 second, transferred into a Thermocycler pre-heated at 25 °C, and incubated at 25 °C for 1 hour at 400 rpm. Next, a 0.3 M sodium azide solution (6.7 µL, 2.0 µmol, 0.13 mg, $50x10^2$ equiv.) in HEPES buffer (pH 7.5, c = 50 mM) was added to the mixture at 20–25 °C (c_{Nu} = 40 mM), followed by addition of 1.0 µL of a **VTFT** stock solution (16 mM, 16 nmol, 5.3 µg, 40 equiv.) in DMF. The reaction mixture was vortexed for 1 second, transferred into a Thermocycler pre-heated at 25 °C, and incubated at 25 °C at 400 rpm for 5 minutes. Subsequently, 1.0 µL of a β-mercaptoethanol stock solution (16 mM, 16 nmol, 1.5 µg, 40 equiv.) in UHQ-H_2O was added, and the mixture was incubated at 25 °C for 15 minutes. The obtained mixture was analyzed by LC-MS.

Due to the impurities present in the starting material, this transformation was not shown in the manuscript. However, as LC-MS data show, the reaction still works on the proteins present in the sample (possibly coming from expression in *E. coli* cells), which is an additional proof for the robustness of the reaction.

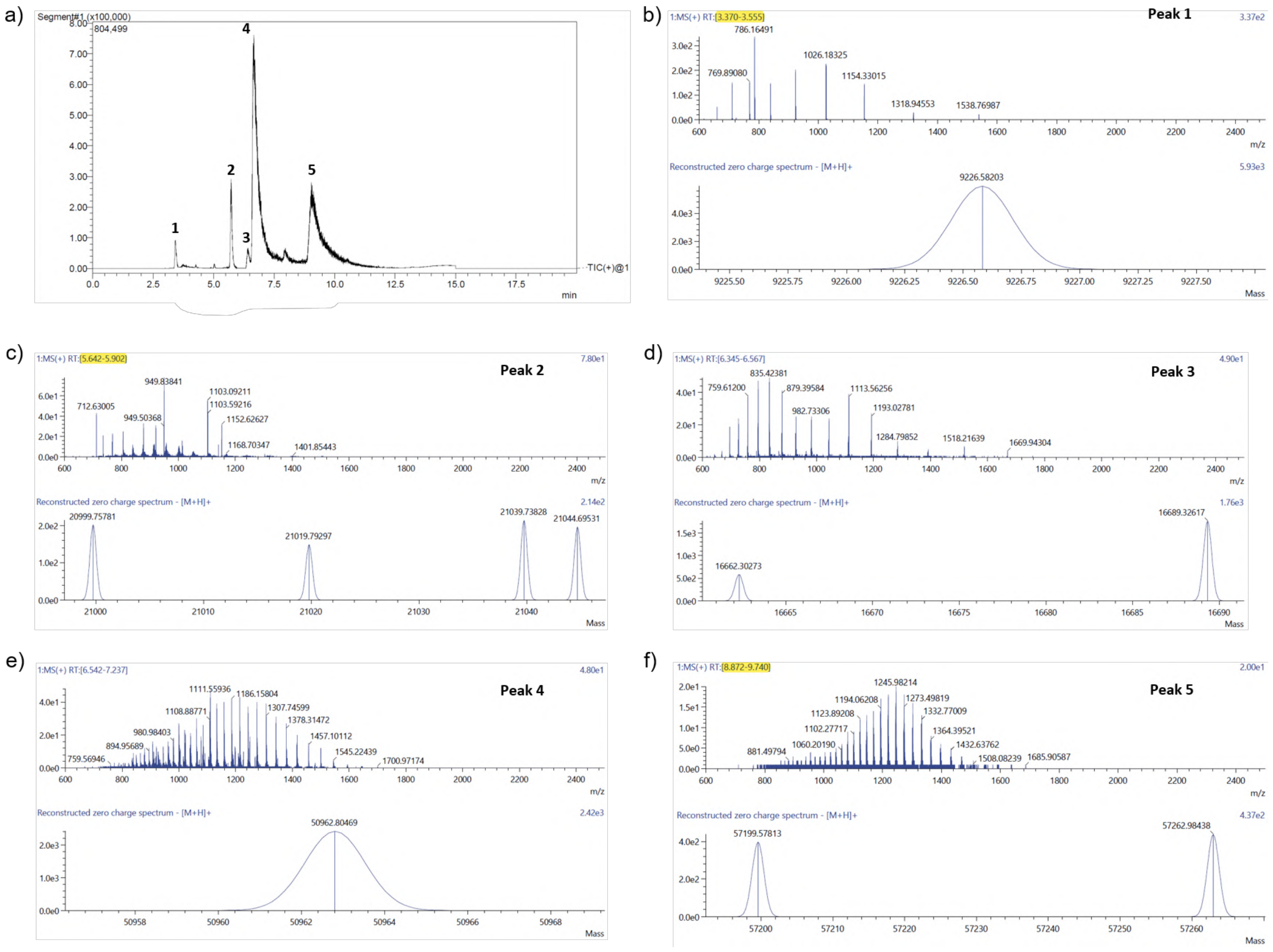

Figure S63. LC-MS analysis (Method B) of MDAR2 after reaction with **VTFT** and sodium azide: a) total ion chromatogram b) Peak 1 ion series and deconvoluted spectrum c) Peak 2 ion series and deconvoluted spectrum d) Peak 3 ion series and deconvoluted spectrum e) Peak 4 ion series and deconvoluted spectrum f) Peak 5 ion series and deconvoluted spectrum; calculated masses: (MDAR2)–S–C_2H_4–N_3: 50961 Da, 57266 Da (peaks detected in SM + 69 Da); observed masses: Peak 4: 50963 Da; Peak 5: 57263 Da.

Assessment of side-reactivity and quantification for protein modifications

In order to reveal side reactivities of the reaction, we looked for three reaction pathways in addition to the desired reaction with the exogenous nucleophile:

1. Reaction of the episulfonium intermediate with water: +44 Da
2. Reaction of the episulfonium intermediate with nucleophilic amino acids on the same protein (stapling): +26 Da
3. Reaction of the episulfonium intermediate with buffer molecules: +124 Da in case of NaP$_i$, +264 Da in case of HEPES, and +235 Da in case of BIS-TRIS

The following table provides an overview of the side-reactivity observed for the reactions between **VTFT**, NaN_3, and different proteins:

Table S3. Analysis of the substrate scope for the azidation reaction on different protein targets. The table summarizes the conversion of the utilized starting materials, gives yields for the desired products, and summarizes detected site reactivities for each target.

Protein	Conversion / %	Yield / %	Detected side reactivity
sfGFP S147C	>95	85[b)]	hydrolysis + stapling
PrxIIB C76S	>95	88[a)]	stapling
Trxh1 C43S	>95	87[a)]	hydrolysis + stapling
Ubiquitin T9C	>95	86[a)]	hydrolysis + stapling + HEPES adduct
DHAR1 S97C	>95	92[b)]	hydrolysis
DHAR1 S176C	>95	88[b)]	hydrolysis + stapling
MDAR2	>95	not determined	not detected
MDAR3	>95	not determined	hydrolysis + stapling
BSA	>95	not determined	not detected

a) Yield determination was performed via PDA analysis at 214 nm.

b) Yield determination was performed via reconstructed zero-charge spectra.

We speculate that the position of the cysteine has an important influence on the reactivity. For example, pre-coordinated water or enhanced steric hindrance might increase formation of the hydrolysis byproduct. Furthermore, nucleophilic residues in close proximity might cause an elevation in intramolecular reactivity. An additional hint for the influence of the local environment is the comparison of reactivity for proteins in which cysteine is located in different sites. For example, formation of a stapling product (+26 Da) in case of Ubiquitin T9C was detected, while for Ubiquitin T12C this reactivity was not observed.

When the reaction is carried out without nucleophile, one can also observe that in case of DHAR1 S97C the phosphate adduct is formed as major species. In case of DHAR1 S176C the main product results from hydrolysis (see experimental data in Figure S124 and Figure S128). This observation is in line with DHAR1 S176C requiring higher azide concentration than DHAR1 S97C to furnish the azidated product in quantities >85 %.

Functional group scope with Ubiquitin T12C

The equivalents of **VTT** were calculated based on the present Cys and the DTT buffer additive. The thiols detected in the analysis of **16** and **17** were probably formed in the acidic conditions during and after LC separation[23]. Side products were not estimated with the QTOF measurements (Method D) because the samples were exposed to acidic conditions before the execution of the measurements. The QTOF data are provided in order to verify the identity of the main product in each reaction with high resolution spectra.

Synthesis of (Ubiquitin T12C)–S–C_2H_4–N_3 (11)

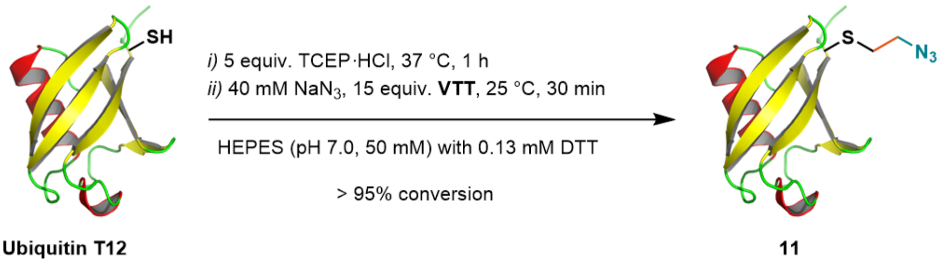

At 20–25 °C, 18 µL of Ubiquitin T12C (1.10 mg/mL, 122 µM, 2.3 nmol, 1.0 equiv.) in HEPES buffer (pH 7.0, c = 50 mM) with 0.13 mM DTT was added to a 1.5 mL Eppendorf tube. Next, 70 µL HEPES buffer (pH 7.0, c = 50 mM) with 0.13 mM DTT was added. Then, 1.1 µL of a TCEP stock solution (10 mM, 11 nmol, 2.8 µg, 5.0 equiv.) in UHQ-H_2O was added. The mixture was vortexed for 1 second, transferred into a Thermocycler pre-heated at 37 °C, and incubated at 37 °C for 1 hour at 400 rpm. Next, a 0.10 M sodium azide solution (60 µL , 6.0 µmol, 0.39 mg, 2.7 × 10^3 equiv.) in HEPES buffer (pH 7.0, c = 50 mM) was added to the mixture at 20–25 °C (c_{Nu} = 40 mM), followed by addition of 1.4 µL of a **VTT** stock solution (25 mM, 34 nmol, 11 µg, 15 equiv.) in DMF. The reaction mixture was vortexed for 1 second, transferred into a Thermocycler pre-heated at 25 °C, and incubated at 25 °C at 400 rpm for 30 minutes. Subsequently, 1.4 µL of a β-mercaptoethanol stock solution (25 mM, 34 nmol, 11 µg, 15 equiv.) in UHQ-H_2O was added, and the mixture was incubated at 25 °C for 15 minutes. The obtained mixture was analyzed by LC-MS.

HRMS-ESI (m/z) calc'd for $C_{391}{}^{13}C_5H_{667}N_{115}O_{124}S_2{}^{12+}$ $[M+12H^+]^{12+}$, 757.2414; found, 757.2400; deviation: –1.8 ppm.

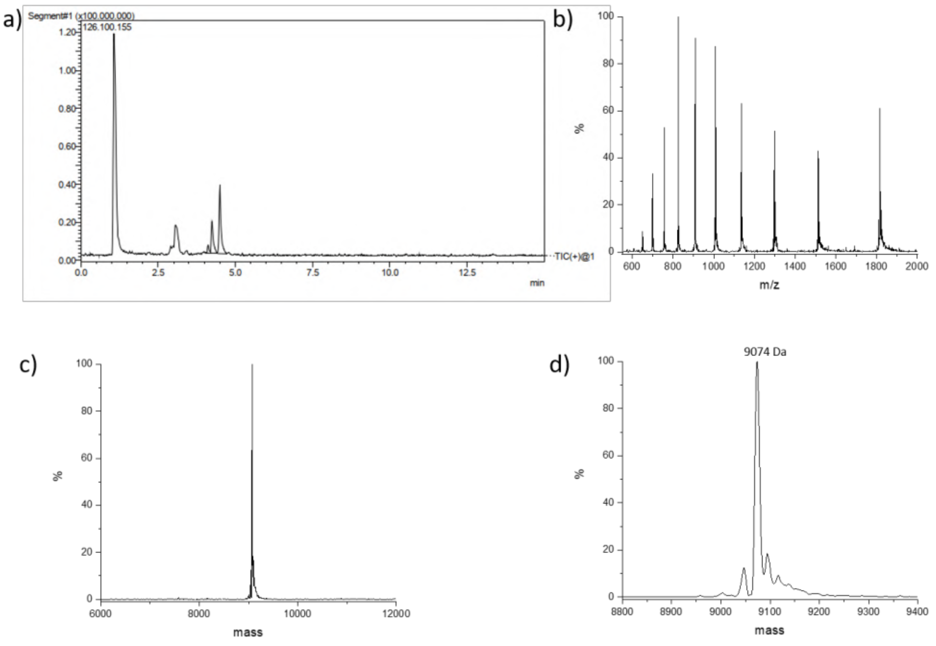

Figure S64 LC-MS analysis (Method A) of Ubiquitin T12C after reaction with **VTT** and sodium azide: a) total ion chromatogram b) ion series c) deconvoluted spectrum d) zoom on the major peaks; calculated mass: 9073 Da ((Ubiquitin T12C)–S–C_2H_4–N_3); observed mass: 9074 Da.

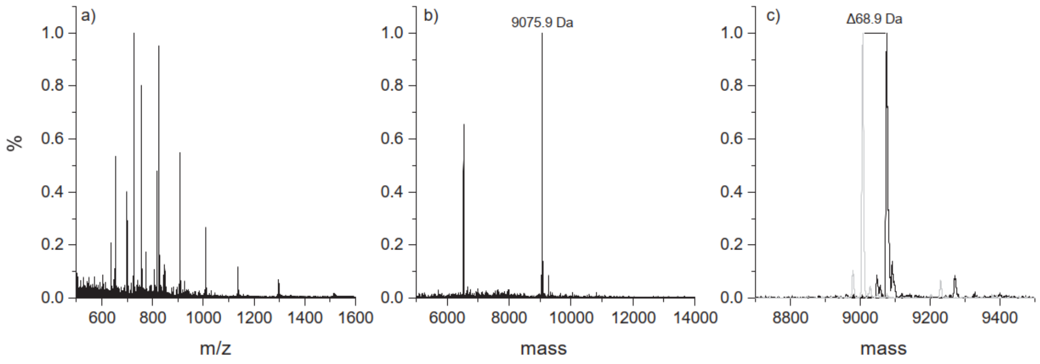

Figure S65. LC-MS analysis (Method D) of the collected product peak after reaction of Ubiquitin T12C with **VTT** and sodium azide: a) ion series b) deconvoluted spectrum c) zoom on the major peaks; calculated mass differences between Ubiquitin T12C and (Ubiquitin T12C)–S–C_2H_4–N_3: 69.1 Da; observed mass difference: 68.9 Da.

Synthesis of (Ubiquitin T12C)–S–C_2H_4–^{15}N-aniline (12)

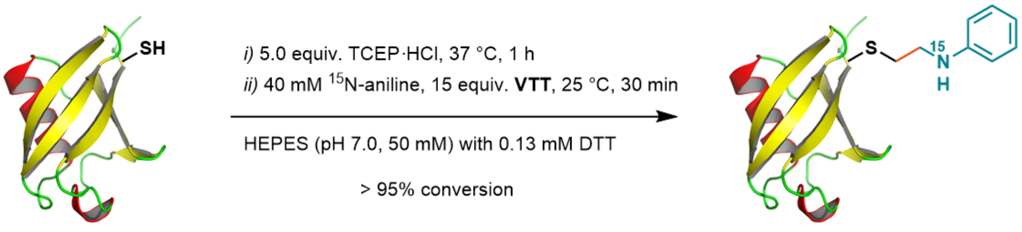

Ubiquitin T12C **12**

At 20–25 °C, 18 µL of Ubiquitin T12C (1.10 mg/mL, 122 µM, 2.3 nmol, 1.0 equiv.) in HEPES buffer (pH 7.0, c = 50 mM) with 0.13 mM DTT was added to a 1.5 mL Eppendorf tube. Next, 70 µL HEPES buffer (pH 7.0, c = 50 mM) with 0.13 mM DTT was added. Then, 1.1 µL of a TCEP stock solution (10 mM, 11 nmol, 2.8 µg, 5.0 equiv.) in UHQ-H_2O was added. The mixture was vortexed for 1 second, transferred into a Thermocycler pre-heated at 37 °C, and incubated at 37 °C for 1 hour at 400 rpm. Next, a 0.10 M ^{15}N–aniline solution (60 µL, 6.0 µmol, 0.56 mg, 2.7 × 10^3 equiv.) in HEPES buffer (pH 7.0, c = 50 mM) was added to the mixture at 20–25 °C (c_{Nu} = 40 mM), followed by addition of 1.4 µL of a **VTT** stock solution (25 mM, 34 nmol, 11 µg, 15 equiv.) in DMF. The reaction mixture was vortexed for 1 second, transferred into a Thermocycler pre-heated at 25 °C, and incubated at 25 °C at 400 rpm for 30 minutes. Subsequently, 1.4 µL of a β-mercaptoethanol stock solution (25 mM, 34 nmol, 11 µg, 15 equiv.) in UHQ-H_2O was added, and the mixture was incubated at 25 °C for 15 minutes. The obtained mixture was analyzed by LC-MS. The determined amount of the hydrolysis by-product (9048 Da) is 11%.

HRMS-ESI (m/z) calc'd for $C_{397}{}^{13}C_5H_{673}N_{112}{}^{15}NO_{124}S_2{}^{12+}$ $[M+12H^+]^{12+}$, 761.4945, found, 761.4934; deviation: –1.4 ppm.

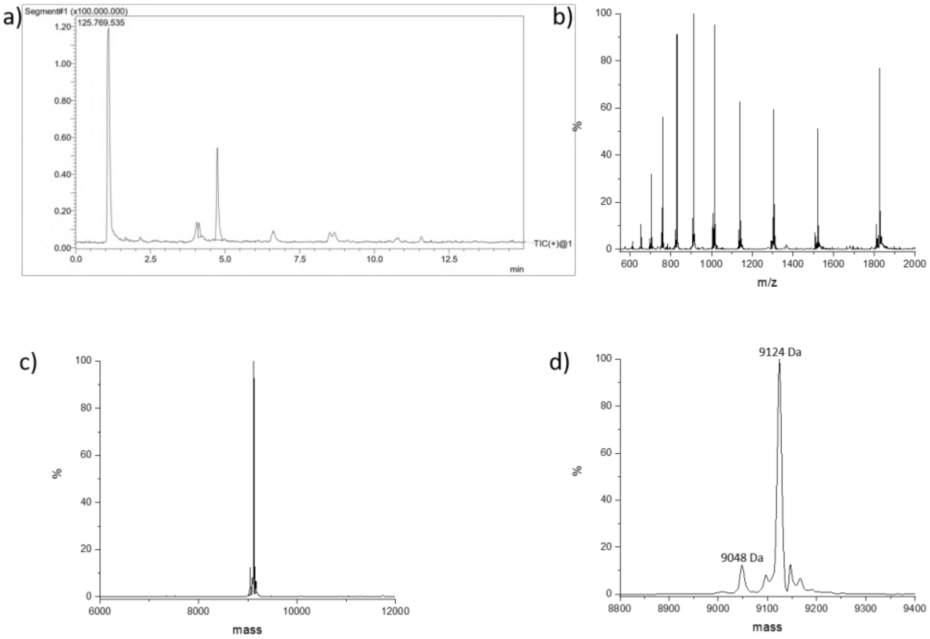

Figure S66 LC-MS analysis (Method A) of Ubiquitin T12C after reaction with **VTT** and ^{15}N-aniline: a) total ion chromatogram b) ion series c) deconvoluted spectrum d) zoom on the major peaks; calculated masses: 9048 Da ((Ubiquitin T12C)–S–C_2H_4–OH), 9124 Da ((Ubiquitin T12C)–S–C_2H_4–^{15}N-aniline); observed masses: 9048 Da, 9124 Da.

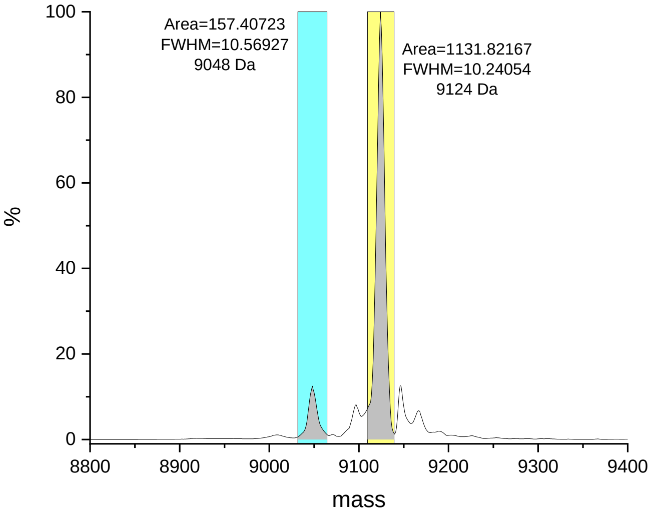

Figure S67. Zero-charge analysis of Ubiquitin T12C after reaction with **VTT** and ^{15}N-aniline for yield determination

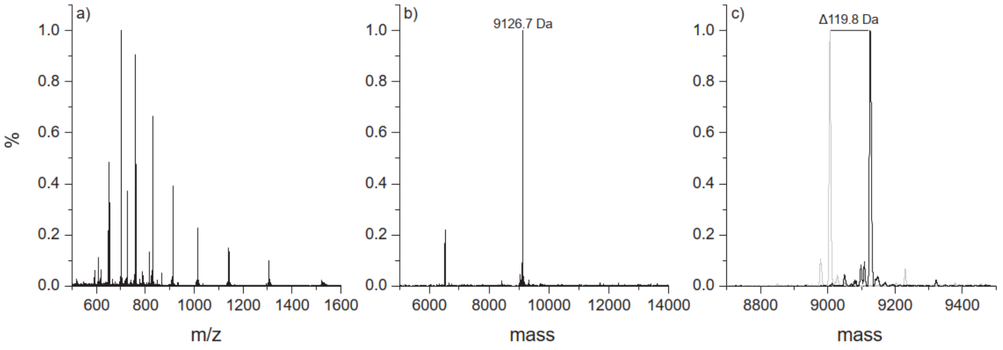

Figure S68. LC-MS analysis (Method D) of the collected product peak after reaction of Ubiquitin T12C with **VTT** and ^{15}N-aniline: a) ion series b) deconvoluted spectrum c) zoom on the major peaks; calculated mass difference between Ubiquitin T12C and (Ubiquitin T12C)–S–C_2H_4–^{15}N-aniline: 120.2 Da; observed mass difference: 119.8 Da.

Synthesis of (Ubiquitin T12C)–S–C_2H_4–N-(4-F-aniline) (13)

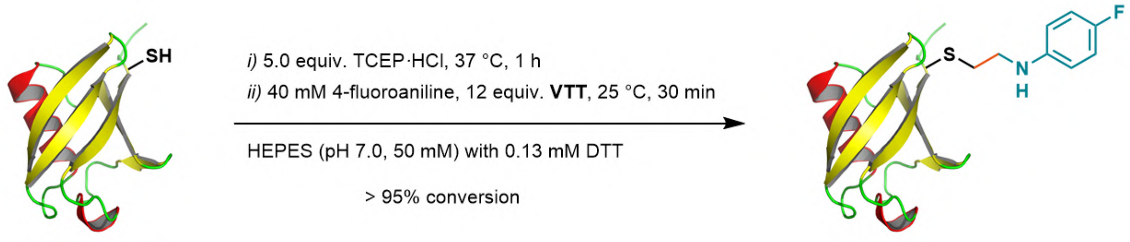

At 20–25 °C, 18 µL of Ubiquitin T12C (1.10 mg/mL, 122 µM, 2.3 nmol, 1.0 equiv.) in HEPES buffer (pH 7.0, c = 50 mM) with 0.13 mM DTT was added to a 1.5 mL Eppendorf tube. Next, 70 µL HEPES buffer (pH 7.0, c = 50 mM) with 0.13 mM DTT was added. Then, 1.1 µL of a TCEP stock solution (10 mM, 11 nmol, 2.8 µg, 5.0 equiv.) in UHQ-H_2O was added. The mixture was vortexed for 1 second, transferred into a Thermocycler pre-heated at 37 °C, and incubated at 37 °C for 1 hour at 400 rpm. Next, a 0.10 M 4–fluoroaniline solution (60 µL, 6.0 µmol, 0.67 mg, 2.7 × 10^3 equiv.) in HEPES buffer (pH 7.0, c = 50 mM) was added to the mixture at 20–25 °C (c_{Nu} = 40 mM), followed by addition of 1.1 µL of a **VTT** stock solution (25 mM, 27 nmol, 8.9 µg, 12 equiv.) in DMF. The reaction mixture was vortexed for 1 second, transferred into a Thermocycler pre-heated at 25 °C, and incubated at 25 °C at 400 rpm for 30 minutes. Subsequently, 1.1 µL of a β-mercaptoethanol stock solution (25 mM, 27 nmol, 8.9 µg, 12 equiv.) in UHQ-H_2O was added, and the mixture was incubated at 25 °C for 15 minutes. The obtained mixture was analyzed by LC-MS. The determined amount of the hydrolysis by-product (9048 Da) is 12%.

HRMS-ESI (m/z) calc'd for $C_{397}{}^{13}C_5H_{672}N_{113}O_{124}S_2F^{12+}$ $[M+12H^+]^{12+}$, 762.9107; found, 762.9099; deviation: –1.0 ppm.

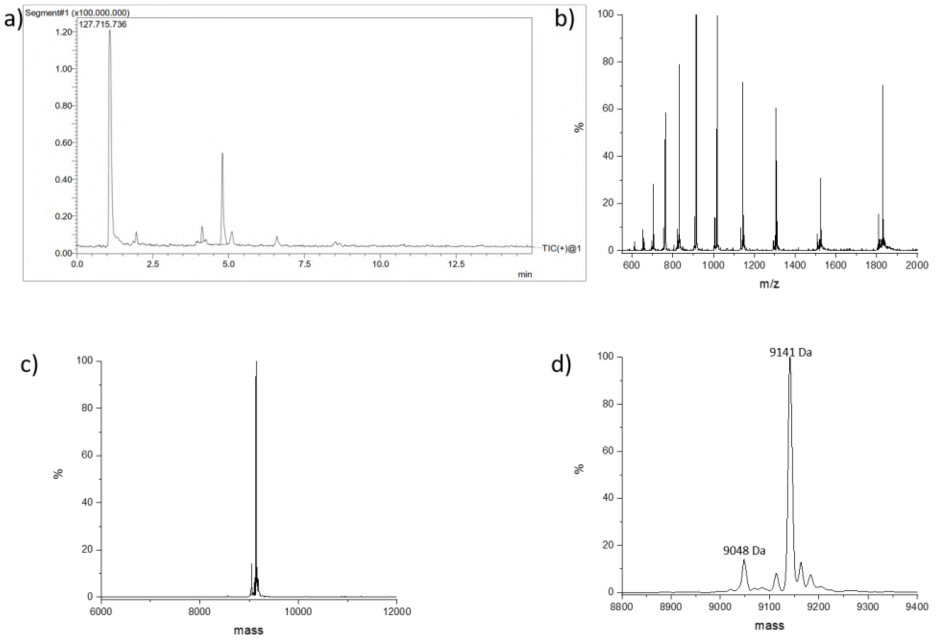

Figure S69 LC-MS analysis (Method A) of Ubiquitin T12C after reaction with **VTT** and 4-fluoroaniline: a) total ion chromatogram b) ion series c) deconvoluted spectrum d) zoom on the major peaks; calculated masses: 9048 Da ((Ubiquitin T12C)–S–C_2H_4–OH), 9141 Da ((Ubiquitin T12C)–S–C_2H_4–N-(4-F-aniline)); observed masses: 9048 Da, 9141 Da.

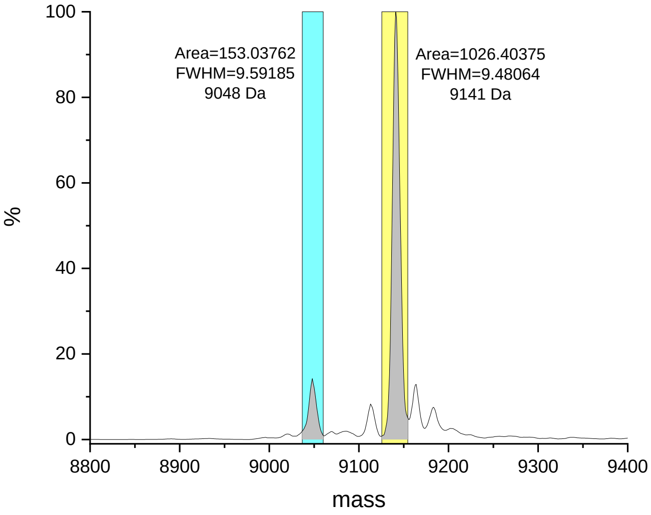

Figure S70. Zero-charge analysis of Ubiquitin T12C after reaction with **VTT** and 4-fluoroaniline for yield determination.

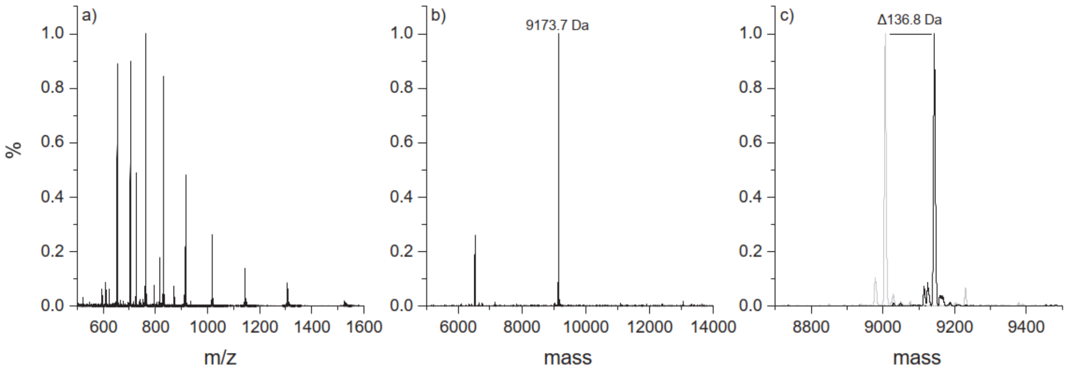

Figure S71. LC-MS analysis (Method D) of the product peak after reaction of Ubiquitin T12C with **VTT** and 4-fluoroaniline: a) ion series b) deconvoluted spectrum c) zoom on the major peaks; calculated mass difference between Ubiquitin T12C and ((Ubiquitin T12C)–S–C_2H_4–N-(4-F-aniline)): 137.2 Da; observed mass difference: 136.8 Da.

Synthesis of (Ubiquitin T12C)–S–C_2H_4–N-(3-aminophenylboronic acid) (14)

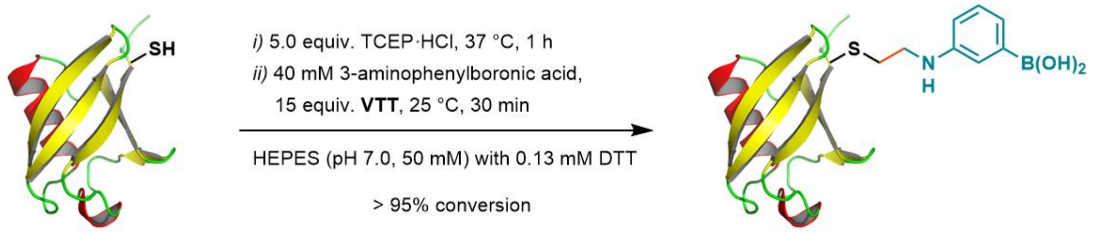

Ubiquitin T12C **14**

At 20–25 °C, 18 µL of Ubiquitin T12C (1.10 mg/mL, 122 µM, 2.3 nmol, 1.0 equiv.) in HEPES buffer (pH 7.0, c = 50 mM) with 0.13 mM DTT was added to a 1.5 mL Eppendorf tube. Next, 70 µL HEPES buffer (pH 7.0, c = 50 mM) with 0.13 mM DTT was added. Then, 1.1 µL of a TCEP stock solution (10 mM, 11 nmol, 2.8 µg, 5.0 equiv.) in UHQ-H_2O was added. The mixture was vortexed for 1 second, transferred into a Thermocycler pre-heated at 37 °C, and incubated at 37 °C for 1 hour at 400 rpm. Next, a 0.10 M 3-aminophenylboronic acid solution (60 µL, 6.0 µmol, 0.82 mg, 2.7 × 10^3 equiv.) in HEPES buffer (pH 7.0, c = 50 mM) was added to the mixture at 20–25 °C (c_{Nu} = 40 mM), followed by addition of 1.4 µL of a **VTT** stock solution (25 mM, 34 nmol, 11 µg, 15 equiv.) in DMF. The reaction mixture was vortexed for 1 second, transferred into a Thermocycler pre-heated at 25 °C, and incubated at 25 °C at 400 rpm for 30 minutes. Subsequently, 1.4 µL of a β-mercaptoethanol stock solution (25 mM, 34 nmol, 11 µg, 15 equiv.) in UHQ-H_2O was added, and the mixture was incubated at 25 °C for 15 minutes. The obtained mixture was analyzed by LC-MS. The determined amount of the hydrolysis by-product (9048 Da) is 13 %.

Detected product masses [M–H_2O] and [M–$2H_2O$] are in accordance to the data reported before[24,25].

HRMS-ESI (m/z) calc'd for $C_{397}{}^{13}C_5H_{670}N_{113}O_{124}S_2B^{12+}$ $[M+12H^+–2H_2O]^{12+}$, 762.0769; found, 762.0756; deviation: –1.7 ppm.

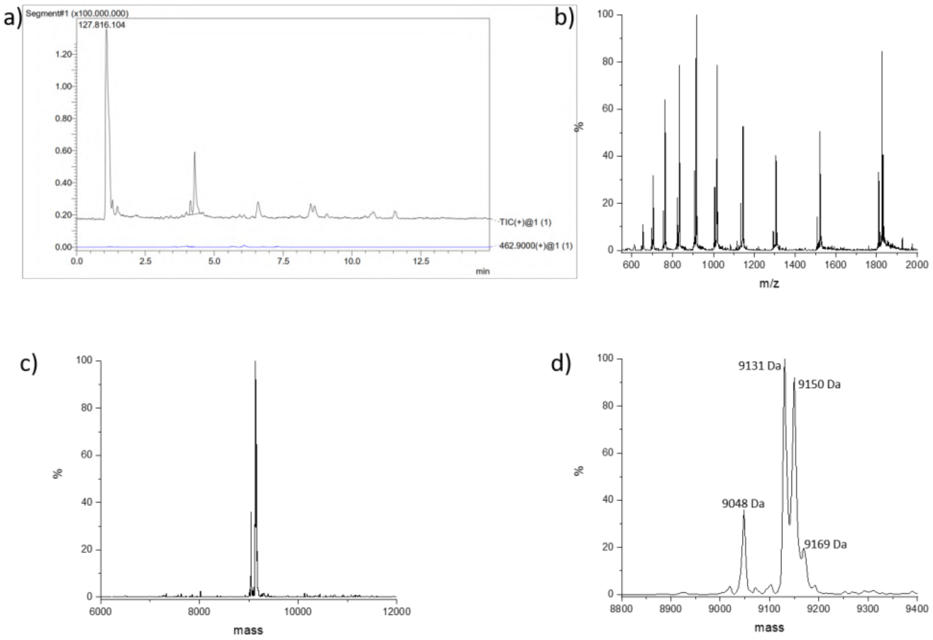

Figure S72 LC-MS analysis (Method A) of Ubiquitin T12C after reaction with **VTT** and 3-aminophenylboronic acid: a) total ion chromatogram b) ion series c) deconvoluted spectrum d) zoom on the major peaks; calculated masses: 9048 Da ((Ubiquitin T12C)–S–C_2H_4–OH), 9167 Da ((Ubiquitin T12C)–S–C_2H_4–N-(3-aminophenylboronic acid)), 9149 Da (product**–**H_2O), 9131 Da (product**–**2H_2O); observed masses: 9169 Da, 9150 Da, 9131 Da, 9048 Da.

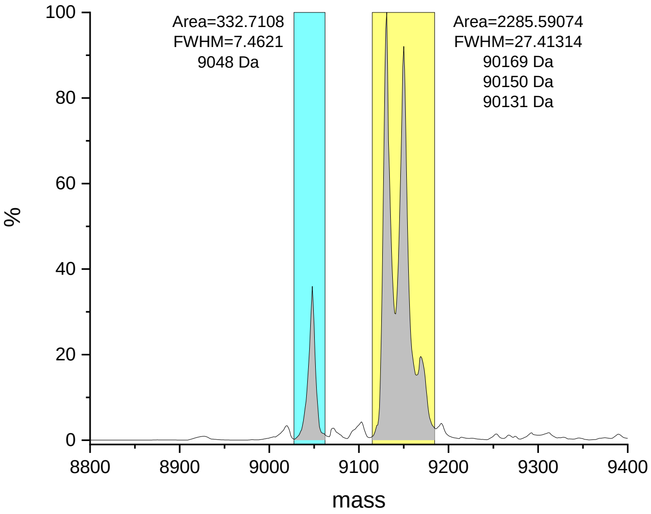

Figure S73. Zero-charge analysis of Ubiquitin T12C after reaction with **VTT** and 3-aminophenylboronic acid for yield determination.

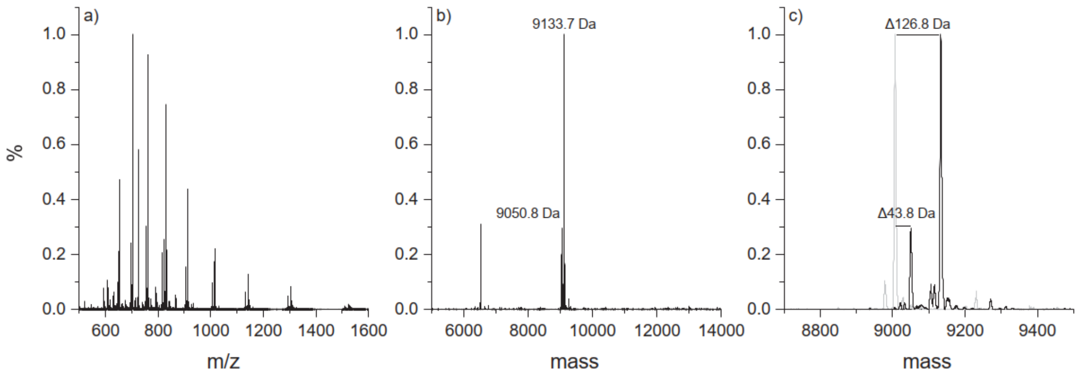

Figure S74. LC-MS analysis (Method D) of the product peak after reaction of Ubiquitin T12C with **VTT** and 3-aminophenylboronic acid: a) ion series b) deconvoluted spectrum c) zoom on the major peaks; calculated mass differences between Ubiquitin T12C and (Ubiquitin T12C)–S–C_2H_4–N-(3-aminophenylboronic acid): 163.0 Da, 145.0 (–H_2O), 127.0 (–$2H_2O$); observed mass differences: 126.8 Da, 43.8 Da (hydrolysis byproduct).

Synthesis of (Ubiquitin T12C)–S–C_2H_4–SCN (15)

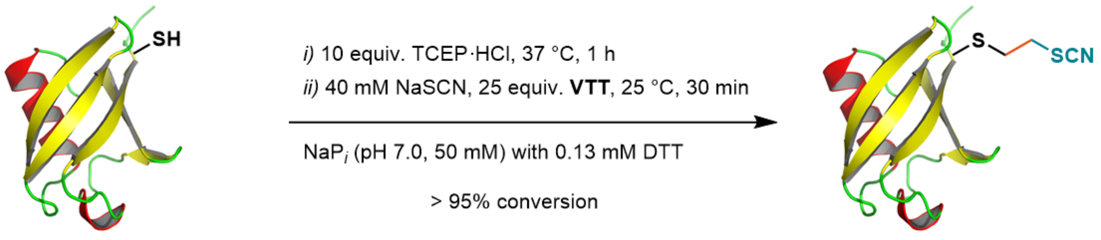

Ubiquitin T12C **15**

At 20–25 °C, 21 µL of Ubiquitin T12C (1.50 mg/mL, 166 µM, 3.5 nmol, 1.0 equiv.) in NaP$_i$ buffer (pH 7.0, c = 50 mM) with 0.13 mM DTT was added to a 1.5 mL Eppendorf tube. Next, 47 µL NaP$_i$ buffer (pH 7.0, c = 50 mM) with 0.13 mM DTT was added. Then, 1.0 µL of a TCEP stock solution (33 mM, 35 nmol, 8.8 µg, 10 equiv.) in UHQ-H_2O was added. The mixture was vortexed for 1 second, transferred into a Thermocycler pre-heated at 37 °C, and incubated at 37 °C for 1 hour at 400 rpm. Next, a 0.10 M sodium thiocyanate stock solution (46 µL, 4.6 µmol, 0.37 mg, 1.3 × 10^3 equiv.) in NaP$_i$ buffer (pH 7.0, c = 50 mM) was added to the mixture at 20–25 °C (c_{Nu} = 40 mM), followed by addition of 0.5 µL of a **VTT** stock solution (0.16 M, 0.09 µmol, 0.03 mg, 3 × 10^1 equiv.) in DMF. The reaction mixture was vortexed for 1 second, transferred into a Thermocycler pre-heated at 25 °C, and incubated at 25 °C at 400 rpm for 30 minutes. The mixture was diluted with 0.12 mL of UHQ-H_2O to obtain a protein concentration of 0.14 mg/mL. The obtained mixture was analyzed by LC-MS.

HRMS-ESI (m/z) calc'd for $C_{392}{}^{13}C_5H_{667}N_{113}O_{124}S_3{}^{12+}$ [M+12H]$^{12+}$, 758.5719; found, 758.5709; deviation: –1.3 ppm.

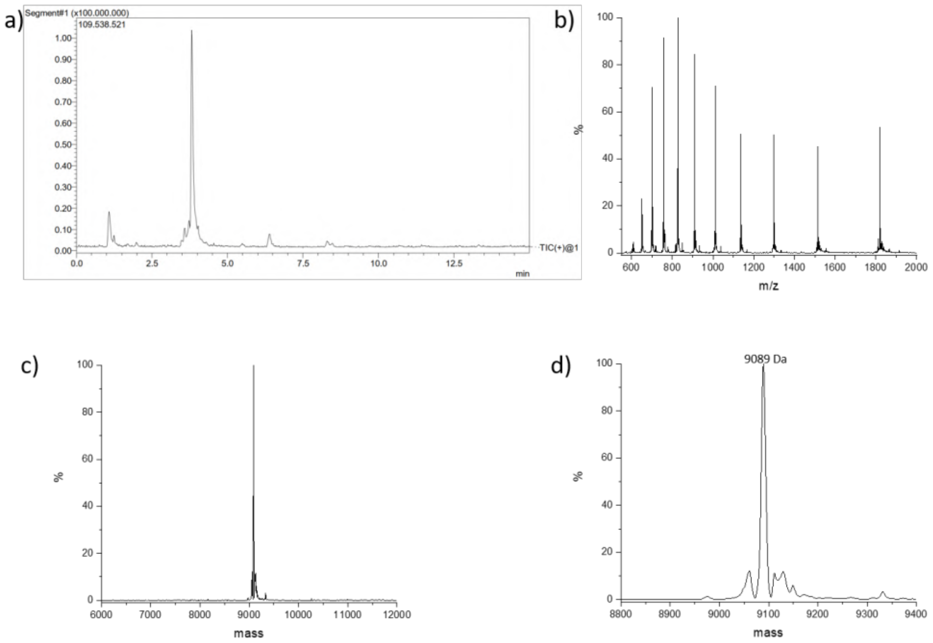

Figure S75 LC-MS analysis (Method A) of Ubiquitin T12C after reaction with **VTT** and thiocyanate: a) total ion chromatogram b) ion series c) deconvoluted spectrum d) zoom on the major peaks; calculated mass: 9089 Da ((Ubiquitin T12C)–S–C_2H_4–SCN); observed mass: 9089 Da.

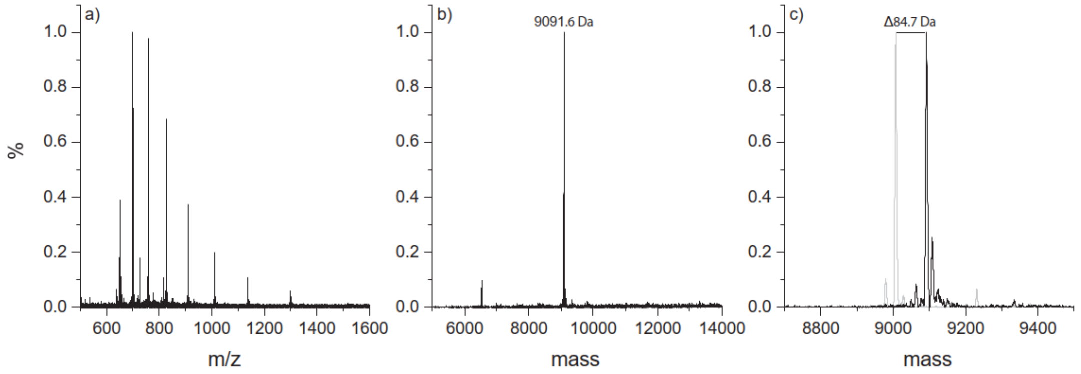

Figure S76. LC-MS analysis (Method D) of the product peak after reaction of Ubiquitin T12C with **VTT** and thiocyanate: a) ion series b) deconvoluted spectrum c) zoom on the major peaks; calculated mass difference between Ubiquitin T12C and (Ubiquitin T12C)–S–C_2H_4–SCN: 85.1 Da; observed mass difference: 84.7 Da.

Synthesis of (Ubiquitin T12C)–S–C_2H_4–S-thiosulfate (16)

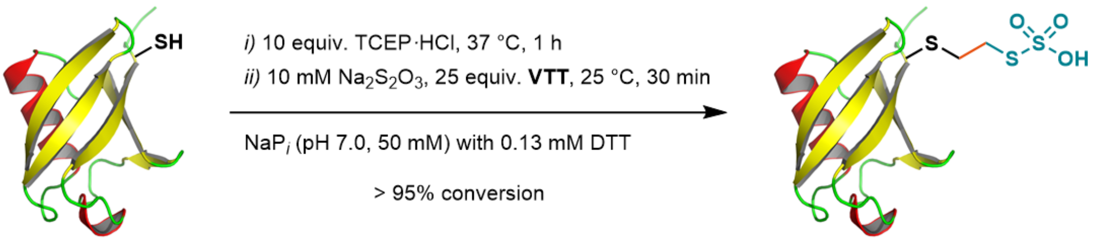

Ubiquitin T12C **16**

At 20–25 °C, 21 µL of Ubiquitin T12C (1.50 mg/mL, 166 µM, 3.5 nmol, 1.0 equiv.) in NaP$_i$ buffer (pH 7.0, c = 50 mM) with 0.13 mM DTT was added to a 1.5 mL Eppendorf tube. Next, 82 µL NaP$_i$ buffer (pH 7.0, c =

50 mM) was added. Then, 1.0 µL of a TCEP stock solution (33 mM, 35 nmol, 8.8 µg, 10 equiv.) in UHQ-H_2O was added. The mixture was vortexed for 1 second, transferred into a Thermocycler pre-heated at 37 °C, and incubated at 37 °C for 1 hour at 400 rpm. Next, a 0.10 M sodium thiosulfate stock solution (12 µL, 1.2 µmol, 0.13 mg, 3.3×10^2 equiv.) in NaP_i buffer (pH 7.0, c = 50 mM) was added to the mixture at 20–25 °C (c_{Nu} = 10 mM), followed by addition of 0.5 µL of a **VTT** stock solution (0.16 M, 0.09 µmol, 0.03 mg, 3×10^1 equiv.) in DMF. The reaction mixture was vortexed for 1 second, transferred into a Thermocycler pre-heated at 25 °C, and incubated at 25 °C at 400 rpm for 30 minutes. The mixture was diluted with 0.12 mL of UHQ-H_2O to obtain a protein concentration of 0.14 mg/mL. The obtained mixture was analyzed by LC-MS.

Because S-alkylthiosulfates undergo hydrolysis in acidic environment[23], the different abundance of the observed (Ubiquitin T12C)–S–C_2H_4–SH product in the spectra measured by different methods (A or D) might be attributed to the longer exposure of the sample to acidic conditions when method D was used.

HRMS-ESI (m/z) calc'd for $C_{391}{}^{13}C_5H_{668}N_{112}O_{127}S_4{}^{12+}$ $[M+12H]^{12+}$, 763.1520; found, 763.1513; deviation: –0.9 ppm.

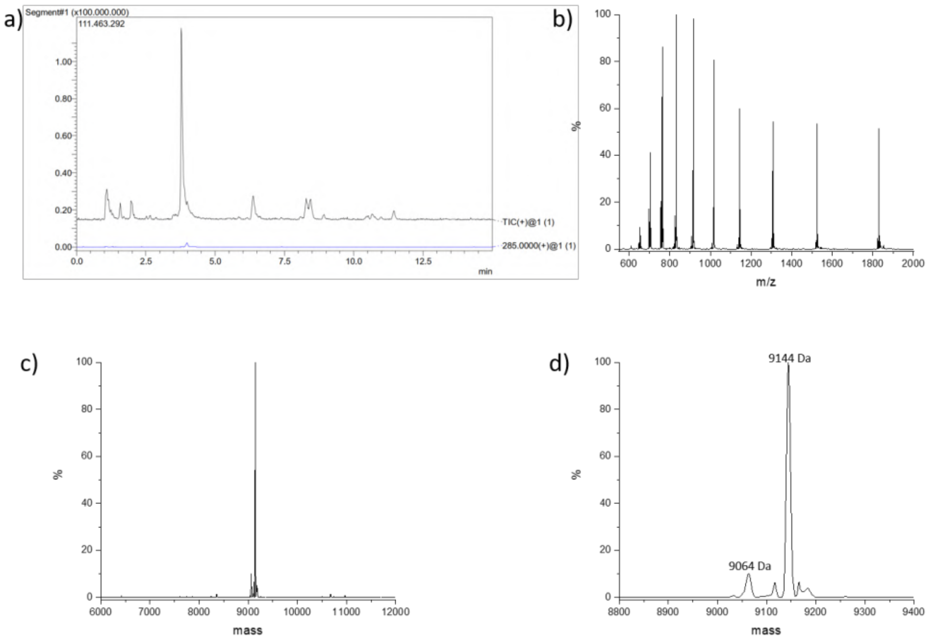

Figure S77 LC-MS analysis (Method A) of Ubiquitin T12C after reaction with **VTT** and thiosulfate: a) total ion chromatogram b) ion series c) deconvoluted spectrum d) zoom on the major peaks; calculated masses: 1944 Da ((Ubiquitin T12C)–S–C_2H_4–S-thiosulfate), 9064 Da ((Ubiquitin T12C)–S–C_2H_4–SH); observed masses: 9064 Da, 9144 Da.

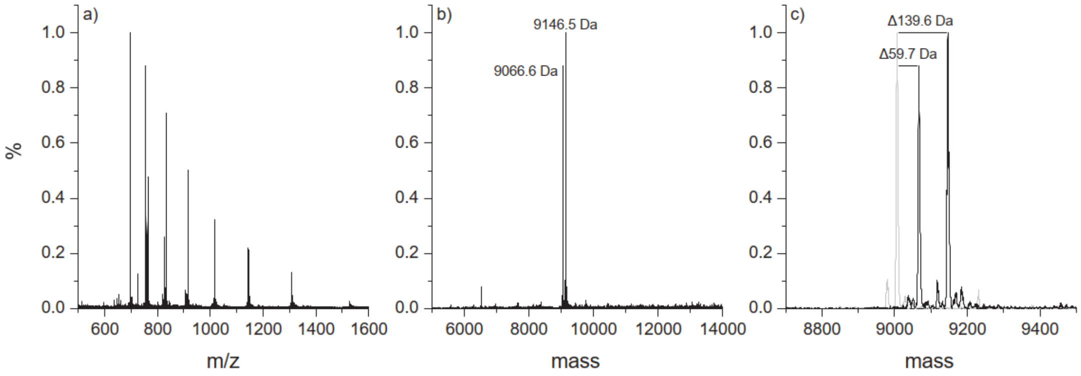

Figure S78. LC-MS analysis (Method D) of the product peak after reaction of Ubiquitin T12C with **VTT** and thiosulfate: a) ion series b) deconvoluted spectrum c) zoom on the major peaks; calculated mass differences between Ubiquitin T12C and (Ubiquitin T12C)–S–C_2H_4–S-thiosulfate: 140.2 Da, 60.1 Da ((Ubiquitin T12C)–S–C_2H_4–SH); observed mass differences: 139.6 Da, 59.7 Da.

Synthesis of (Ubiquitin T12C)–S–C_2H_4–S-thiophosphate (17)

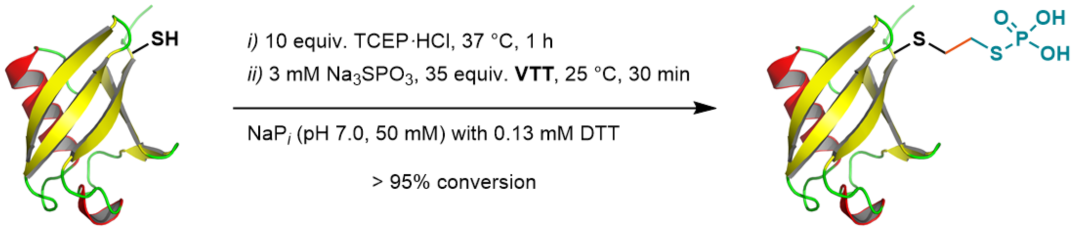

Ubiquitin T12C **17**

At 20–25 °C, 21 µL of Ubiquitin T12C (1.50 mg/mL, 166 µM, 3.5 nmol, 1.0 equiv.) in NaP*i* buffer (pH 7.0, c = 50 mM) with 0.13 mM DTT was added to a 1.5 mL Eppendorf tube. Next, 87 µL NaP*i* buffer (pH 7.0, c = 50 mM) was added. Then, 1.0 µL of a TCEP stock solution (33 mM, 35 nmol, 8.8 µg, 10 equiv.) in UHQ-H_2O was added. The mixture was vortexed for 1 second, transferred into a Thermocycler pre-heated at 37 °C, and incubated at 37 °C for 1 hour at 400 rpm. Next, a 58 mM sodium thiophosphate stock solution (5.8 µL, 0.33 µmol, 59 µg, 96 equiv.) in NaP*i* buffer (pH 7.0, c = 50 mM) was added to the mixture at 20–25 °C (c_{Nu} = 2.9 mM), followed by addition of 0.8 µL of a **VTT** stock solution (0.16 M, 0.1 µmol, 0.03 mg, 4 × 10^1 equiv.) in DMF. The reaction mixture was vortexed for 1 second, transferred into a Thermocycler pre-heated at 25 °C, and incubated at 25 °C at 400 rpm for 30 minutes. The mixture was diluted with 0.12 mL UHQ-H_2O to obtain a protein concentration of 0.14 mg/mL. The obtained mixture was analyzed by LC-MS.

We attribute the observed peak splitting on the LC-MS chromatogram (Figure S79 a) to formation of the hydrolyzed product ((Ubiquitin T12C)–S–C_2H_4–SH) during the LC-MS measurement. The hydrolyzed adduct was detected as indicated below.

HRMS-ESI (m/z) calc'd for $C_{391}{}^{13}C_5H_{668}N_{112}O_{127}S_3P^{11+}$ $[M+11H]^{11+}$, 832.4388; found, 832.4380; deviation: –1.0 ppm.

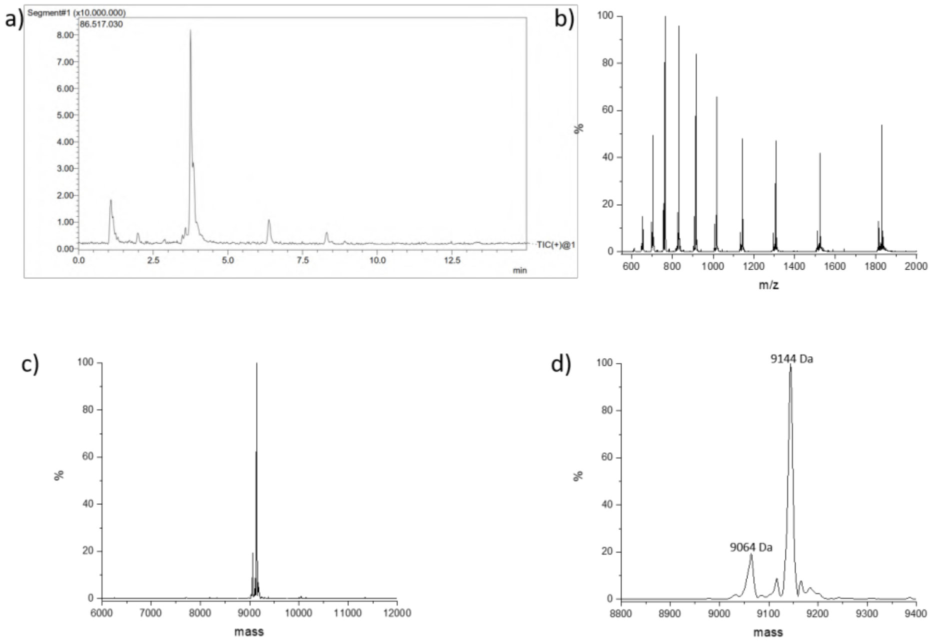

Figure S79. LC-MS analysis (Method A) of Ubiquitin T12C after reaction with **VTT** and thiophosphate: a) total ion chromatogram b) ion series c) deconvoluted spectrum d) zoom on the major peaks; calculated masses: 140 Da ((Ubiquitin T12C)–S–C_2H_4–S-thiophosphate), 9064 Da ((Ubiquitin T12C)–S–C_2H_4–SH); observed masses: 9064 Da, 9144 Da.

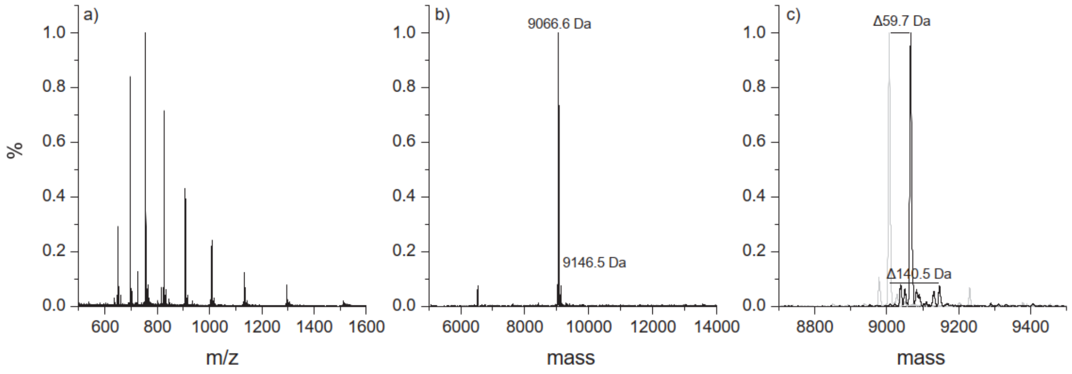

Figure S80. LC-MS analysis (Method D) of the product peak after reaction of Ubiquitin T12C with **VTT** and thiophosphate: a) ion series b) deconvoluted spectrum c) zoom on the major peaks; calculated mass differences between Ubiquitin T12C and (Ubiquitin T12C)–S–C_2H_4–S-thiophosphate: 140.1 Da, 60.1 Da ((Ubiquitin T12C)–S–C_2H_4–SH); observed mass differences: 140.5 Da, 59.7 Da.

Synthesis of (Ubiquitin T12C)–S–C_2H_4–S-thioglucose (18)

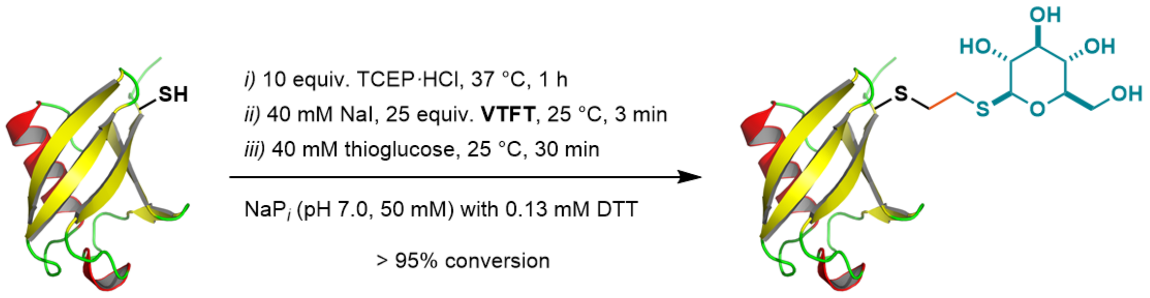

Ubiquitin T12C **18**

At 20–25 °C, 21 µL of Ubiquitin T12C (1.50 mg/mL, 166 µM, 3.5 nmol, 1.0 equiv.) in NaP_i buffer (pH 7.0, c = 50 mM) with 0.13 mM DTT was added to a 1.5 mL Eppendorf tube. Next, 47 µL NaP_i buffer (pH 7.0, c = 50 mM) was added. Then, 1.0 µL of a TCEP stock solution (33 mM, 35 nmol, 8.8 µg, 10 equiv.) in UHQ-H_2O was added. The mixture was vortexed for 1 second, transferred into a Thermocycler pre-heated at 37 °C, and incubated at 37 °C for 1 hour at 400 rpm. Next, a 0.10 M sodium iodide stock solution (46 µL, 4.6 µmol, 0.37 mg, 1.3 × 10^3 equiv.) in NaP_i buffer (pH 7.0, c = 50 mM) was added to the mixture at 20–25 °C (c_{Nu} = 40 mM), followed by addition of 0.5 µL of a **VTFT** stock solution (0.16 M, 0.09 µmol, 0.03 mg, 3 × 10^1 equiv.) in DMF. The reaction mixture was vortexed for 1 second, transferred into a Thermocycler pre-heated at 25 °C, and incubated at 25 °C at 400 rpm for 3 minutes. Immediately afterwards, a 80 mM sodium β-D-Thioglucose stock solution (0.12 mL , 9.2 µmol, 1.8 mg, 2.6 × 10^3 equiv.) in NaP_i buffer (pH 7.0, c = 50 mM) was added to the mixture. The mixture was incubated at 25 °C at 400 rpm for 30 minutes. The obtained mixture was analyzed by LC-MS.

HRMS-ESI (m/z) calc'd for $C_{397}{}^{13}C_5H_{678}N_{112}O_{129}S_3{}^{12+}$ $[M+12H]^{12+}$, 769.9933; found, 769.9929; deviation: –0.5 ppm.

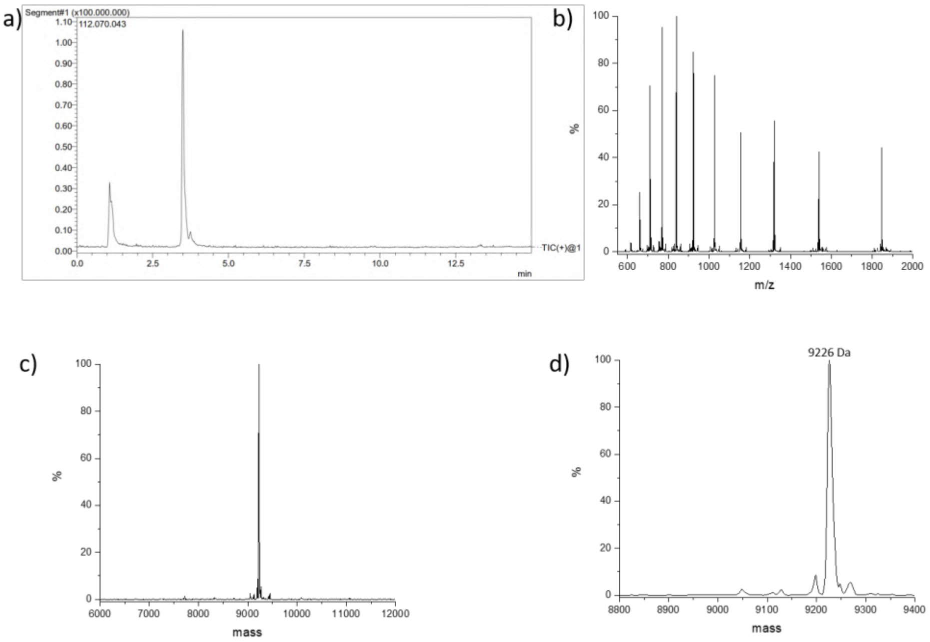

Figure S81. LC-MS analysis (Method A) of Ubiquitin T12C after reaction with **VTFT**, NaI, and thioglucose: a) total ion chromatogram b) ion series c) deconvoluted spectrum d) zoom on the major peaks; calculated mass: 9226 Da ((Ubiquitin T12C)–S–C_2H_4–S-thioglucose); observed mass: 9226 Da.

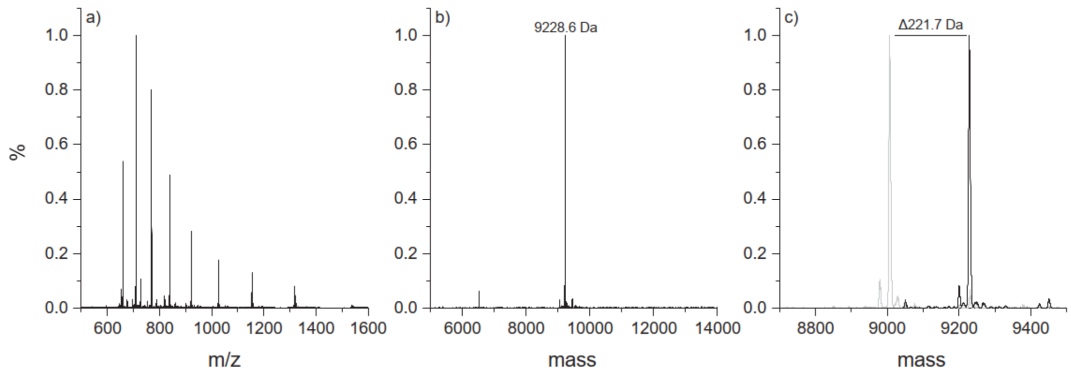

Figure S82. LC-MS analysis (Method D) of the product peak after reaction of Ubiquitin T12C with **VTFT**, NaI, and thioglucose: a) ion series b) deconvoluted spectrum c) zoom on the major peaks; calculated mass difference between Ubiquitin T12C and (Ubiquitin T12C)–S–C_2H_4–S-thioglucose: 222.3 Da; observed mass difference: 221.7 Da.

Synthesis of (Ubiquitin T12C)–S–C_2H_4–SAc (19)

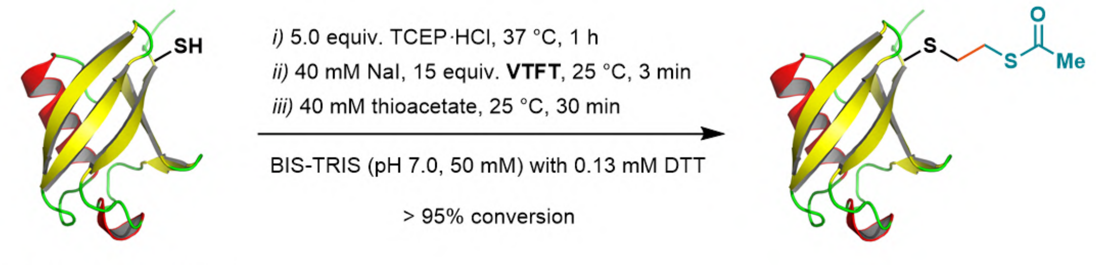

Ubiquitin T12C, 10 µM **19**

At 20–25 °C, 14 µL of Ubiquitin T12C (1.49 mg/mL, 165 µM, 2.3 nmol, 1.0 equiv.) in BIS-TRIS buffer (pH 7.0, c = 50 mM) with 0.13 mM DTT was added to a 1.5 mL Eppendorf tube. Next, 52 µL BIS-TRIS buffer (pH 7.0, c = 50 mM) with 0.13 mM DTT was added. Then, 1.1 µL of a TCEP stock solution (10 mM, 11 nmol, 2.8 µg, 5.0 equiv.) in UHQ-H_2O was added. The mixture was vortexed for 1 second, transferred into a Thermocycler pre-heated at 37 °C, and incubated at 37 °C for 1 hour at 400 rpm. Next, a 0.10 M sodium iodide stock solution (30 µL, 3.0 µmol, 0.45 mg, 1.3 × 10^3 equiv.) in BIS-TRIS buffer (pH 7.0, c = 50 mM) was added to the mixture at 20–25 °C (c_{Nu} = 40 mM), followed by addition of 0.6 µL of a **VTFT** stock solution (25 mM, 34 nmol, 11 µg, 15 equiv.) in DMF. The reaction mixture was vortexed for 1 second, transferred into a Thermocycler pre-heated at 25 °C, and incubated at 25 °C at 400 rpm for 3 minutes. Immediately afterwards, a 80 mM potassium thioacetate stock solution (75 µL, 6.0 µmol, 0.69 mg, 2.7 × 10^3 equiv.) in BIS-TRIS buffer (pH 7.0, c = 50 mM) was added to the mixture. The mixture was incubated at 25 °C at 400 rpm for 30 minutes. The obtained mixture was analyzed by LC-MS.

HRMS-ESI (m/z) calc'd for $C_{393}{}^{13}C_5H_{670}N_{112}O_{125}S_3{}^{12+}$ $[M+12H]^{12+}$, 759.9898; found, 759.9892; deviation: –0.8 ppm.

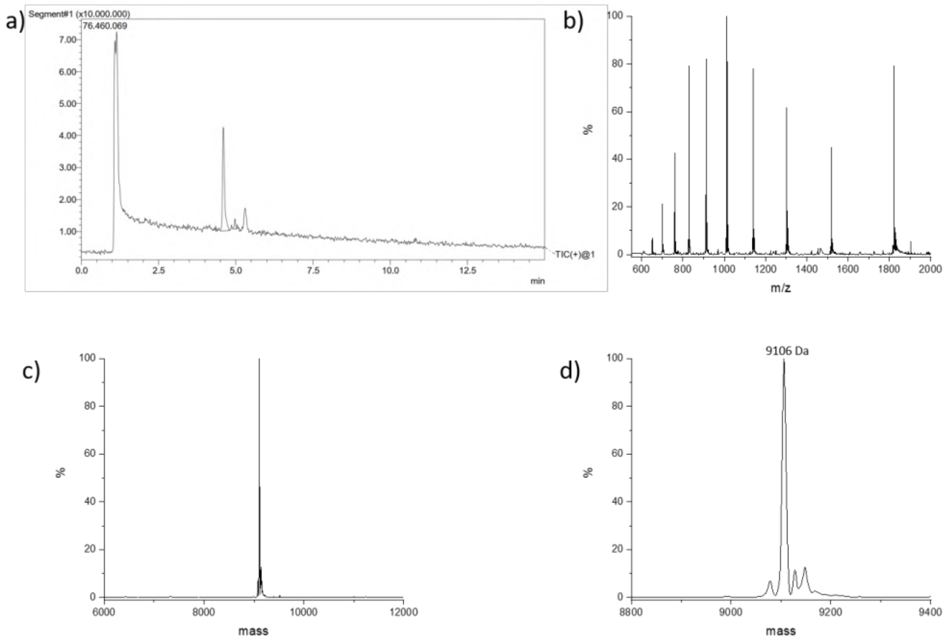

Figure S83. LC-MS analysis (Method A) of Ubiquitin T12C after reaction with **VTFT**, NaI, and thioacetate: a) total ion chromatogram b) ion series c) deconvoluted spectrum d) zoom on the major peaks; calculated mass: 9106 Da ((Ubiquitin T12C)–S–C_2H_4–SAc); observed mass: 9106 Da.

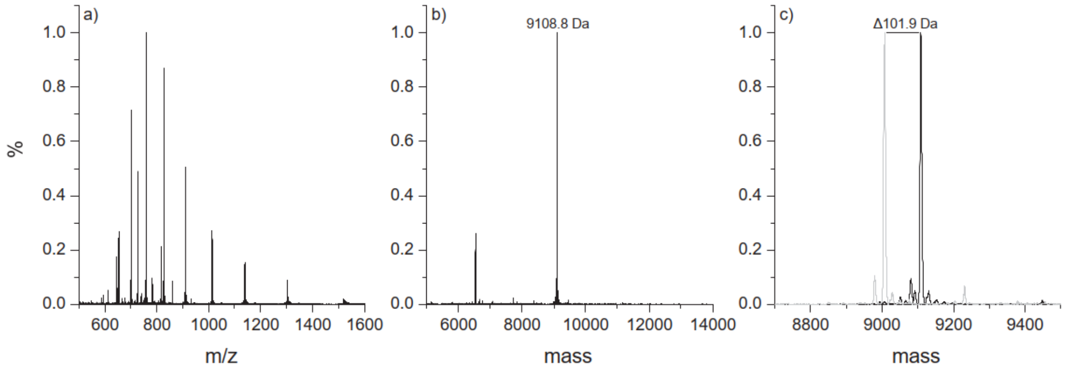

Figure S84. LC-MS analysis (Method D) of the product peak after reaction of Ubiquitin T12C with **VTFT**, NaI, and thioacetate: a) ion series b) deconvoluted spectrum c) zoom on the major peaks; calculated mass difference between Ubiquitin T12C and (Ubiquitin T12C)–S–C_2H_4–SAc: 102.2 Da; observed mass difference: 101.9 Da.

Synthesis of (Ubiquitin T12C)–S–C_2H_4–dimethylamine (20)

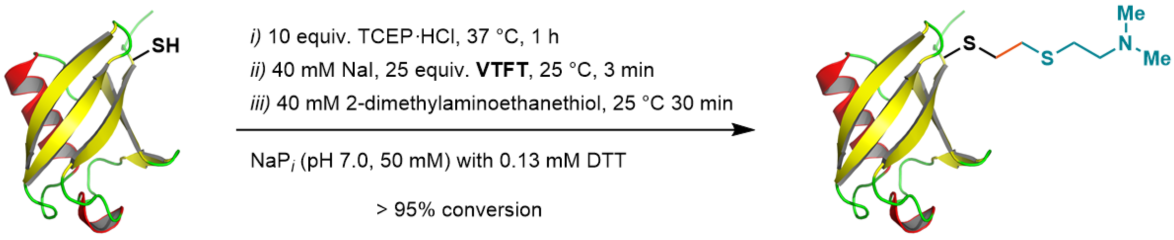

Ubiquitin T12C **20**

At 20–25 °C, 21 µL of Ubiquitin T12C (1.50 mg/mL, 166 µM, 3.5 nmol, 1.0 equiv.) in NaP*i* buffer (pH 7.0,

c = 50 mM) with 0.13 mM DTT was added to a 1.5 mL Eppendorf tube. Next, 47 µL NaP*i* buffer (pH 7.0, c = 50 mM) was added. Then, 1.0 µL of a TCEP stock solution (33 mM, 35 nmol, 8.8 µg, 10 equiv.) in UHQ-H_2O was added. The mixture was vortexed, transferred into a Thermocycler pre-heated at 37 °C, and incubated at 37 °C for 1 hour at 400 rpm. Next, a 0.10 M sodium iodide stock solution (46 µL, 4.6 µmol, 0.37 mg, 1.3 × 10^3 equiv.) in NaP*i* buffer (pH 7.0, c = 50 mM) was added to the mixture at 20–25 °C (c_{Nu} = 40 mM), followed by addition of 0.5 µL of a **VTFT** stock solution (0.16 M, 0.09 µmol, 0.03 mg, 3 × 10^1 equiv.) in DMF. The reaction mixture was vortexed, transferred into a Thermocycler pre-heated at 25 °C, and incubated at 25 °C at 400 rpm for 3 minutes. Immediately afterwards, a 80 mM 2-dimethylamino-ethanthiol stock solution (0.12 mL, 9.2 µmol, 0.97 mg, 2.6 × 10^3 equiv.) in NaP*i* buffer (pH 7.0, c = 50 mM) with 80 mM NaOH was added to the mixture. The mixture was incubated at 25 °C at 400 rpm for 30 minutes. The obtained mixture was analyzed by LC-MS.

HRMS-ESI (m/z) calc'd for $C_{395}{}^{13}C_5H_{677}N_{113}O_{124}S_3{}^{12+}$ $[M+12H]^{12+}$, 762.4117; found, 762.4111; deviation: –0.8 ppm.

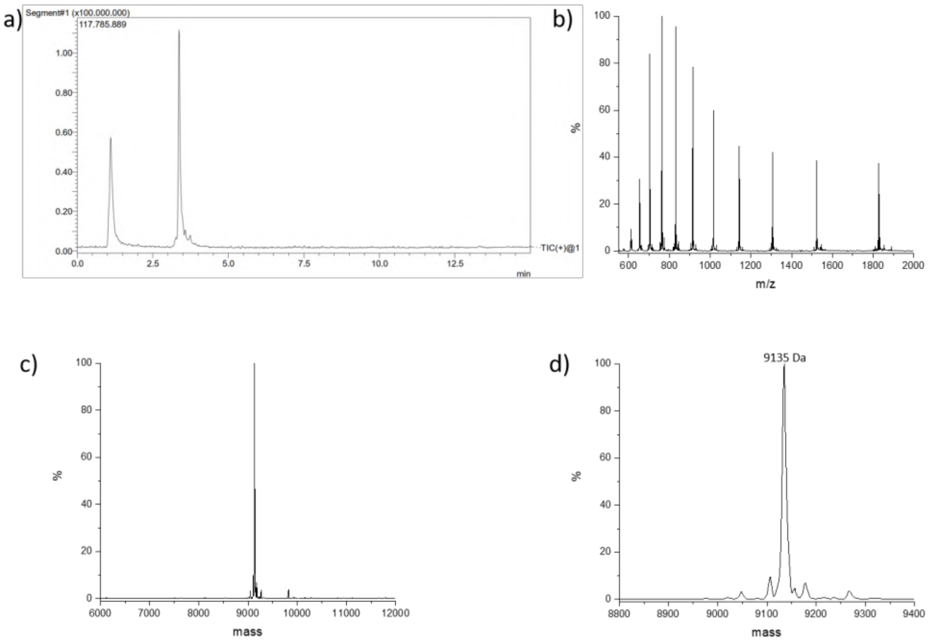

Figure S85. LC-MS analysis (Method A) of Ubiquitin T12C after reaction with **VTFT**, NaI, and 2-dimethylaminoethanedithiol: a) total ion chromatogram b) ion series c) deconvoluted spectrum d) zoom on the major peaks; calculated mass: 9135 Da ((Ubiquitin T12C)–$(S–C_2H_4)_2$-dimethylamine); observed mass: 9135 Da.

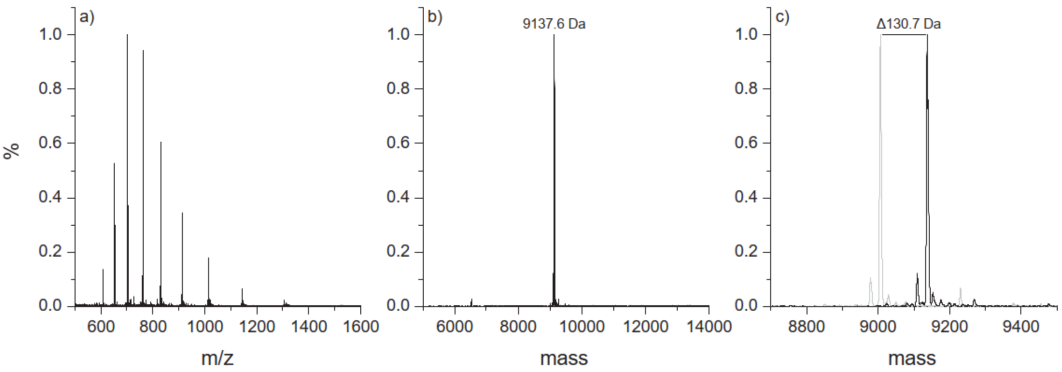

Figure S86. LC-MS analysis (Method D) of the product peak after reaction of Ubiquitin T12C with **VTFT**, NaI, and 2-dimethylaminoethanedithiol: a) ion series b) deconvoluted spectrum c) zoom on the major peaks; calculated mass difference between Ubiquitin T12C and (Ubiquitin T12C)–(S–C_2H_4)$_2$–dimethylamine: 131.2 Da; observed mass difference: 130.7 Da.

Synthesis of (Ubiquitin T9C)–S–C_2H_4–S–PEG11–biotin (21)

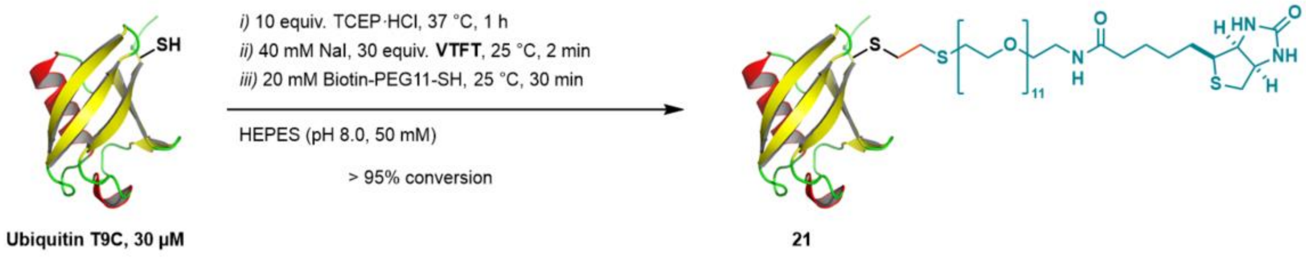

21

At 20–25 °C, 9.0 µL of Ubiquitin T9C (1.52 mg/mL, 168 µM, 1.5 nmol, 1.0 equiv.) in HEPES buffer (pH 8.0, c = 50 mM) was added to a 1.5 mL Eppendorf tube. Next, 34 µL HEPES buffer (pH 8.0, c = 50 mM) was added. Then, 0.5 µL of a TCEP stock solution (30 mM, 1.5 nmol, 4.3 µg, 10 equiv.) in UHQ-H_2O was added. The mixture was vortexed, transferred into a Thermocycler pre-heated at 37 °C, and incubated at 37 °C for 1 hour at 400 rpm. Next, a 300 mM sodium iodide stock solution (6.7 µL, 2.0 µmol, 0.30 mg, 1.3 × 10^3 equiv.) in HEPES buffer (pH 8.0, c = 50 mM) was added to the mixture at 20–25 °C (c_{Nu} = 40 mM), followed by addition of 0.8 µL of a **VTFT** stock solution (0.06 M, 0.09 µmol, 0.04 mg, 3 × 10^1 equiv.) in DMF. The reaction mixture was vortexed, transferred into a Thermocycler pre-heated at 25 °C, and incubated at 25 °C at 400 rpm for 2 minutes. Immediately afterwards, a 40 mM biotin-PEG11-SH stock solution (50 µL, 2.0 µmol, 1.6 mg, 1.3 × 10^3 equiv.) in HEPES buffer (pH 8.0, c = 50 mM) was added to the mixture. The mixture was incubated at 25 °C at 400 rpm for 30 minutes. The product mixture was rebuffered 4 times in HEPES buffer (pH 7.0, c = 50 mM) using an AMICON® filter with 3 kDa cutoff and analyzed by LC-MS. The obtained mixture was analyzed by LC-MS. The determined amounts of the hydrolysis by-product (9054 Da) and the HEPES buffer adduct (9273 Da) are 15 % and 11 %, respectively.

HRMS-ESI (m/z) calc'd for $C_{425}{}^{13}C_5H_{731}N_{115}O_{137}S_4{}^{12+}$ $[M+12H]^{12+}$, 819.2730; found, 819.2735; deviation: 0.6 ppm.

LC-MS analysis of the non protein peaks eluting after the protein adducts (>8 min) showed only thiol starting material and low-MW molecules.

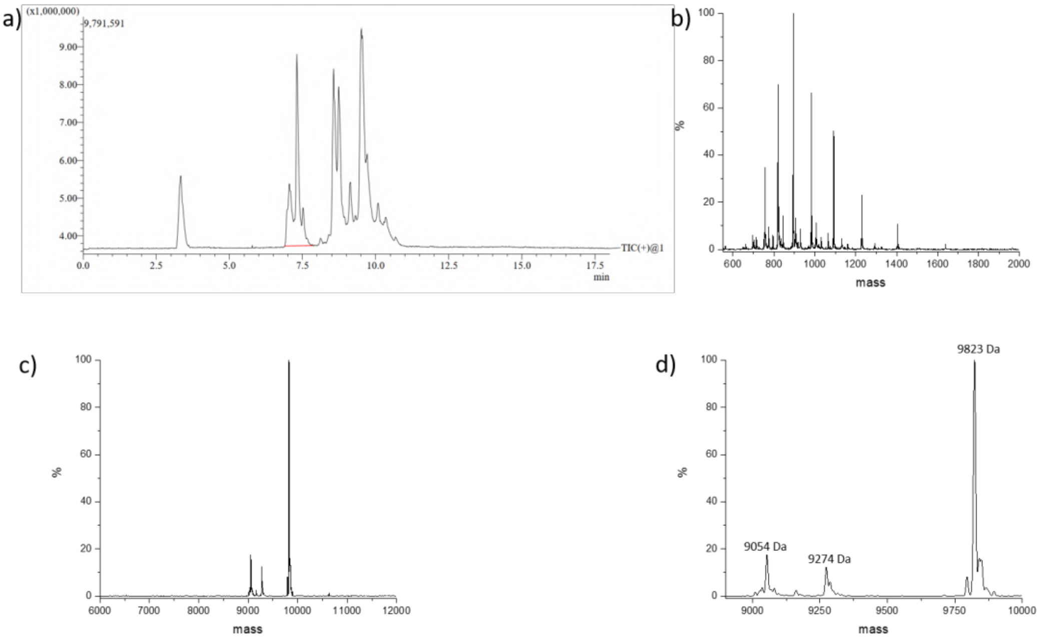

Figure S87. LC-MS analysis (Method E) of Ubiquitin T9C after reaction with **VTFT**, sodium iodide, and thiol-PEG11-biotin: a) total ion chromatogram b) ion series c) deconvoluted spectrum d) zoom on the major peaks; calculated masses: 9821 Da (Ubiquitin T9C)–S–C_2H_4–S–PEG11–biotin, 9052 Da (Ubiquitin T9C)–S–C_2H_4–OH, 9272 Da (Ubiquitin T9C)–S–C_2H_4–HEPES; Observed masses: 9054 Da, 9274 Da, 9823 Da.

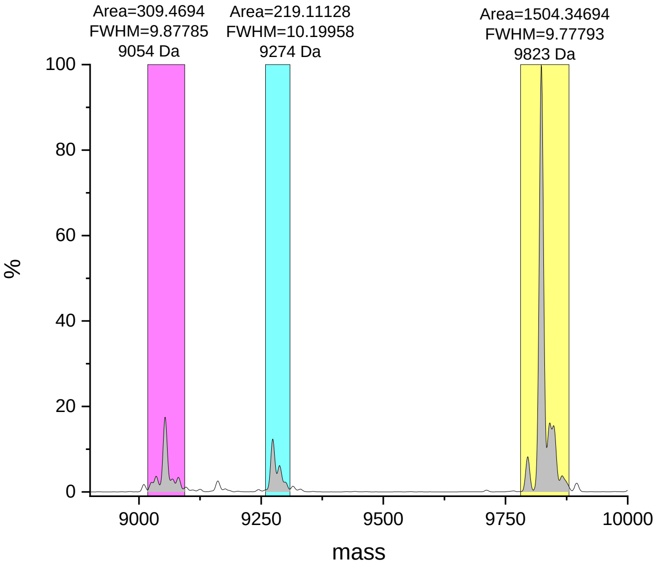

Figure S88. Zero-charge analysis of Ubiquitin T9C after reaction with **VTFT**, sodium iodide, and thiol-PEG11-biotin for yield determination.

Synthesis of (Ubiquitin T12C)–S–C_2H_4–S–PEG11–biotin (S14)

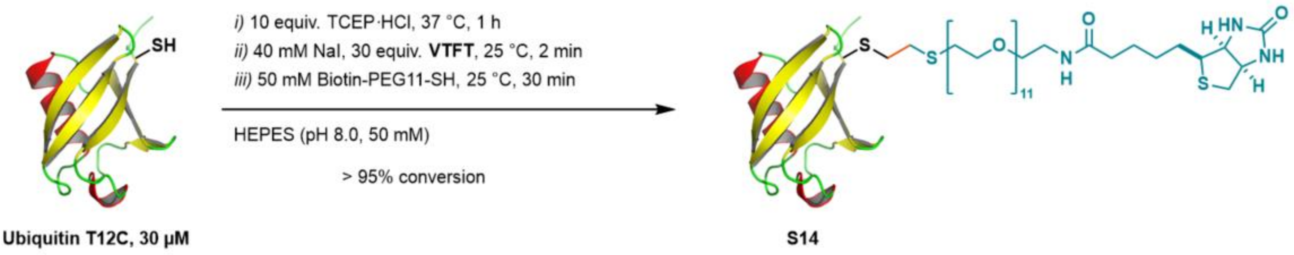

At 20–25 °C, 12.0 µL of Ubiquitin T12C (1.11 mg/mL, 123 µM, 1.50 nmol, 1.00 equiv.) in HEPES buffer (pH 8.0, c = 50 mM) was added to a 1.5 mL Eppendorf tube. Next, 31 µL HEPES buffer (pH 8.0, c = 50 mM) was added. Then, 0.5 µL of a TCEP stock solution (30 mM, 1.5 nmol, 4.3 µg, 10 equiv.) in UHQ-H_2O was added. The mixture was vortexed, transferred into a Thermocycler pre-heated at 37 °C, and incubated at 37 °C for 1 hour at 400 rpm. Next, a 300 mM sodium iodide stock solution (6.7 µL, 2.0 µmol, 0.30 mg, 1.3 × 10^3 equiv.) in HEPES buffer (pH 8.0, c = 50 mM) was added to the mixture at 20–25 °C (c_{Nu} = 40 mM), followed by addition of 0.8 µL of a **VTFT** stock solution (0.06 M, 0.05 µmol, 0.02 mg, 3 × 10^1 equiv.) in DMF. The reaction mixture was vortexed, transferred into a Thermocycler pre-heated at 25 °C, and incubated at 25 °C at 400 rpm for 2 minutes. Immediately afterwards, a 100 mM biotin-PEG11-SH stock solution (50 µL, 3.0 µmol, 2.4 mg, 2.0 × 10^3 equiv.) in HEPES buffer (pH 8.0, c = 50 mM) was added to the mixture. The mixture was incubated at 25 °C at 400 rpm for 30 minutes. The product mixture was rebuffered 4 times in HEPES buffer (pH 7.0, c = 50 mM) using an AMICON® filter with 3 kDa cutoff and analyzed by LC-MS. The determined amounts of the hydrolysis by-product (9054 Da) and the HEPES buffer adduct (9273 Da) are 35 % and 30 %, respectively.

HRMS-ESI (m/z) for (Ubiquitin T12C)–S–C_2H_4–S–PEG11–biotin not determined. Due to low conversion, the biotinylation was repeated with Ubiquitin T9C (for procedure and analysis see above). We assume that the difference in reactivity arises from a better access of the biotin-PEG11-thiol nucleophile to the C9 position compared to the C12 poistion in the two mutants.

LC-MS analysis of the non protein peaks eluting after the protein adducts (>8 min) showed only thiol starting material and low-MW molecules.

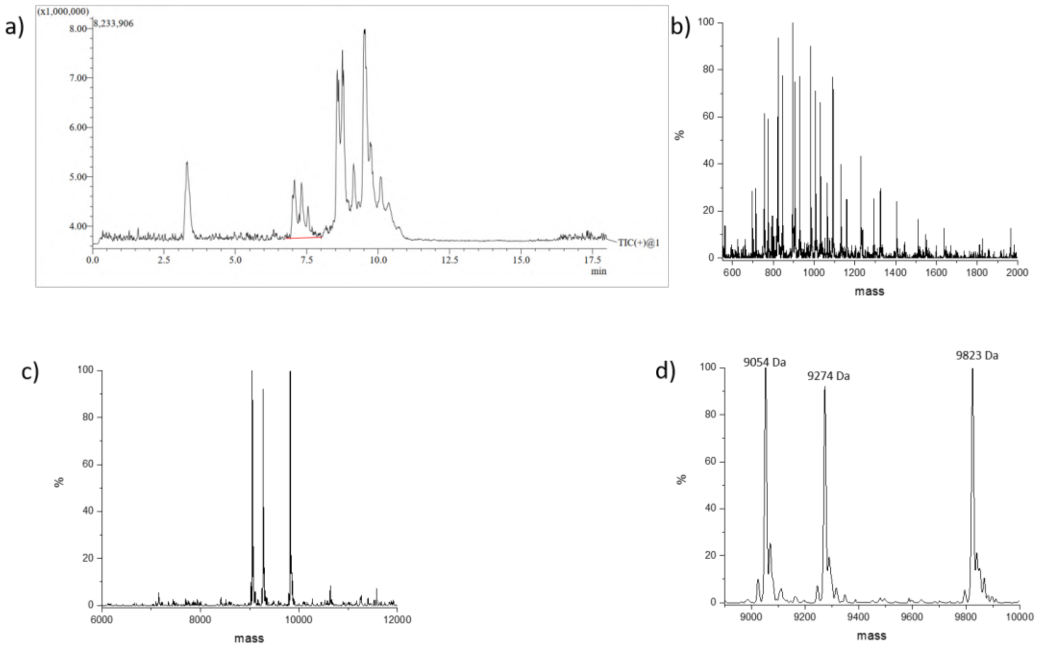

Figure S89. LC-MS analysis (Method E) of Ubiquitin T12C after reaction with **VTFT**, sodium iodide, and thiol-PEG11-biotin: a) total ion chromatogram b) ion series c) deconvoluted spectrum d) zoom on the major peaks; calculated masses: 9821 Da (Ubiquitin T12C)–S–C_2H_4–S–PEG11–biotin, 9052 Da (Ubiquitin T12C)–S–C_2H_4–OH, 9272 Da (Ubiquitin T12C)–S–C_2H_4–HEPES; Observed masses: 9054 Da, 9274 Da, 9823 Da

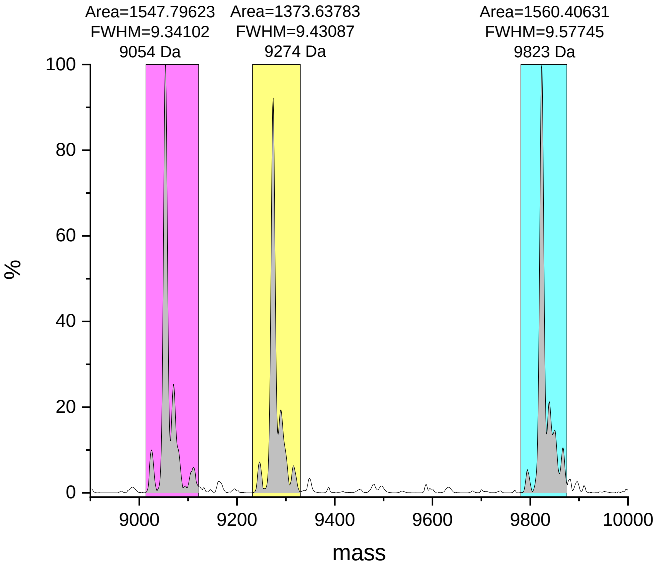

Figure S90. Zero-charge analysis of Ubiquitin T12C after reaction with **VTFT**, sodium iodide, and thiol-PEG11-biotin for yield determination.

Assessment of side-reactivity and quantification of protein modifications

The side reactivity for the functional group scope was assessed via three different approaches. Because of the high degree of comparability, also demonstrated in the example below, the side reactivity is estimated via peak areas of deconvoluted raw data in all the cases.

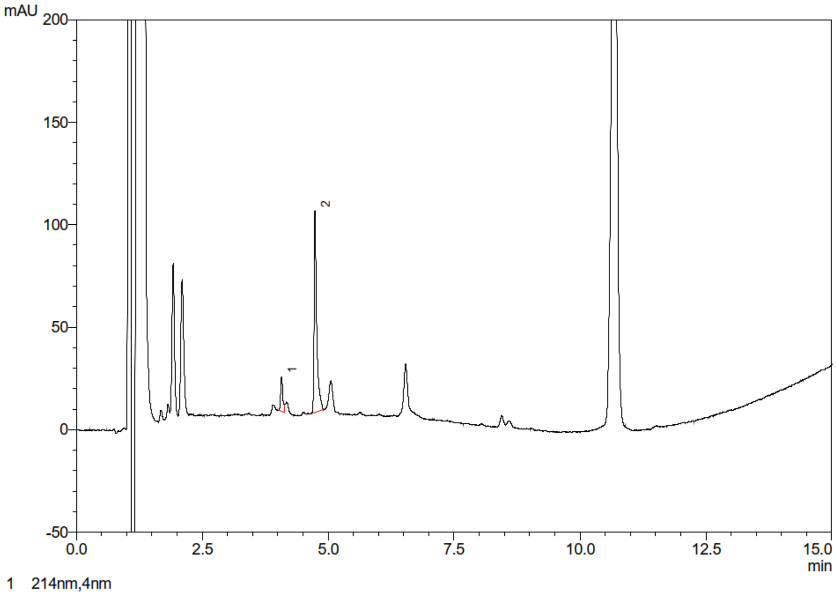

Figure S91. PDA analysis of Ubiquitin T12C after reaction with **VTT** and 4-fluoroaniline measured at 214 nm. Peaks utilized for integration of (Ubiquitin T12C)–S–C_2H_4–OH and (Ubiquitin T12C)–S–C_2H_4–NH–C_6H_5F labeled with **1** and **2**, respectively.

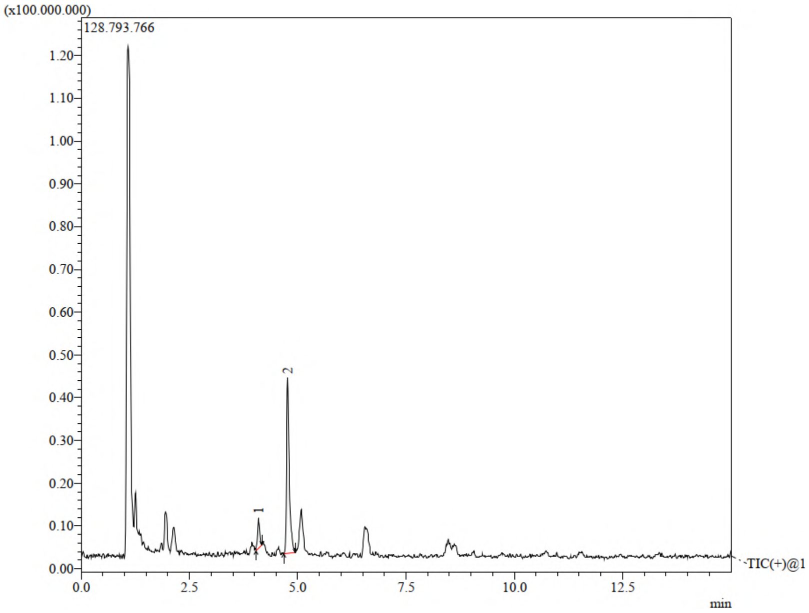

Figure S92. Total-Ion-Count chromatogram of Ubiquitin T12C after reaction with VTT and 4-fluoroaniline. Peaks utilized for integration of (Ubiquitin T12C)–S–C_2H_4–OH and (Ubiquitin T12C)–S–C_2H_4– NH–C_6H_5F labeled with **1** and **2**, respectively.

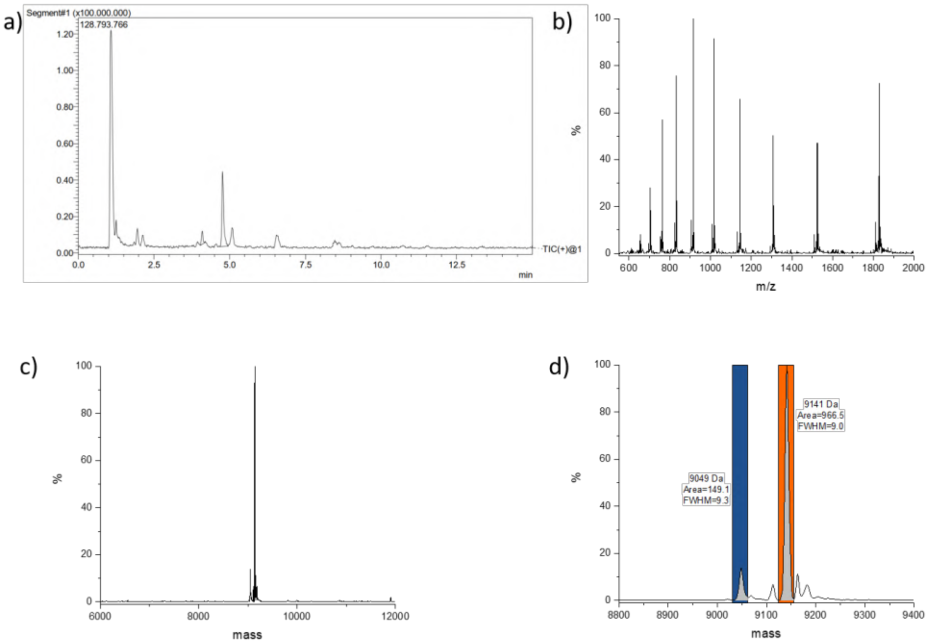

Figure S93. LC-MS analysis of Ubiquitin T12C after reaction with **VTT** and 4-fluoroaniline: a) ion series b) deconvoluted spectrum c) zoom on the major peaks and their respective peak areas determined by integration; calculated mass differences between Ubiquitin T12C and (Ubiquitin T12C)–S–C_2H_4–OH: 44 Da and between Ubiquitin T12C and (Ubiquitin T12C)–S–C_2H_4–NH–C_6H_5F: 137 Da, observed mass differences: 45 Da, 137 Da.

Table S4. Comparison of the degrees of hydrolysis based on PDA-analysis, TIC-integration, and peak areas of zero-charge spectra after deconvolution.

**Technique**	**Hydrolysis / %**
PDA	13
TIC	12
Deconvolution	13

Table S5. Amounts of hydrolysis detected for Ubiquitin T12C in the reaction with **VT**(**F**)**T** and different nucleophiles based on peak areas of zero-charge spectra after deconvolution.

**Compound number**	**Nucleophile**	**conversion / %**	**desired product / %**	**Hydrolysis byproduct / %**	**Buffer byproduct / %**
**11**	Azide	>95	100	not detected	not detected
**12**	^{15}N-aniline	>95	88	12	not detected
**13**	4-fluoroaniline	>95	87	13	not detected
**14**	3-aminophenylboronic acid	>95	87	13	not detected
**15**	Thiocyanate	>95	100	not detected	not detected
**16**	Thiosulfate	>95	100	not detected	not detected

**17**	Thiophosphate	>95	100	not detected	not detected
**18**	NaI → β-D-Thioglucose	>95	100	not detected	not detected
**19**	NaI → 2-dimethylamino-ethanthiol	>95	100	not detected	not detected
**20**	NaI → thioacetate	>95	100	not detected	not detected
**21**	NaI → Biotin-PEG11-SH	>95	74	15	11
**S14**	NaI → Biotin-PEG11-SH	>95	35	35	30

Synthesis of conjugates for protein NMR studies

Synthesis of (^{15}N,^{13}C-Ubiquitin T12C)–S–$^{13}C_2H_4$–N_3 (S15)

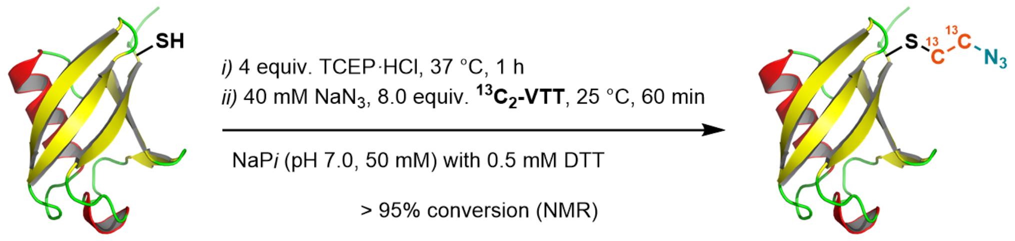

^{15}N,^{13}C-Ubiquitin T12C **S15**

At 20–25 °C, 150 µL of ^{15}N,^{13}C-Ubiquitin T12C (1.0 mM, 0.15 µmol, 1.0 equiv.) in NaP_i buffer (pH 7.0, c = 50 mM) with 0.5 mM DTT and 0.05% NaN_3 was added to a 1.5 mL Eppendorf tube. Next, 0.88 mL of NaP_i buffer (pH 7.0, c = 50 mM) with 0.5 mM DTT was added. Then, 6.0 µL of a TCEP stock solution (0.10 M, 0.60 µmol, 0.15 mg, 4.0 equiv.) in UHQ-H_2O was added. The mixture was vortexed for 1 second, transferred into a Thermocycler pre-heated at 37 °C, and incubated at 37 °C for 1 hour at 400 rpm. Next, a 0.30 M sodium azide solution (0.16 mL , 48 µmol, 3.1 mg, 3.2 × 10^2 equiv.) in NaP_i buffer (pH 7.0, c = 50 mM) was added to the mixture at 20–25 °C (c_{Nu} = 40 mM), followed by addition of 1.2 µL of a **$^{13}C_2$-VTT** stock solution (1.0 M, 1.2 µmol, 0.40 mg, 8.0 equiv.) in DMF. The reaction mixture was vortexed for 1 second, transferred into a Thermocycler pre-heated at 25 °C, and incubated at 25 °C at 400 rpm for 60 minutes. Subsequently, the reaction mixture was centrifuged with 12.000 × g for 10 min at 4 °C. The resulting supernatant was transferred into an AMICON® filter unit with a 3 kDa cutoff and rebuffered five times into NaP_i buffer (pH 7.0, c = 50 mM) with 0.5 mM DTT and 0.05% NaN_3. Next, the obtained protein solution was filled up to 250 µL with NaP_i buffer (pH 7.0, c = 50 mM) with 0.5 mM DTT and 0.05% NaN_3, transferred into a Shigemi NMR tube, and analyzed via NMR. NMR data can be found in the Protein-NMR section.

Synthesis of Ubiquitin T12C–S–C_2H_4–N_3 (S16)

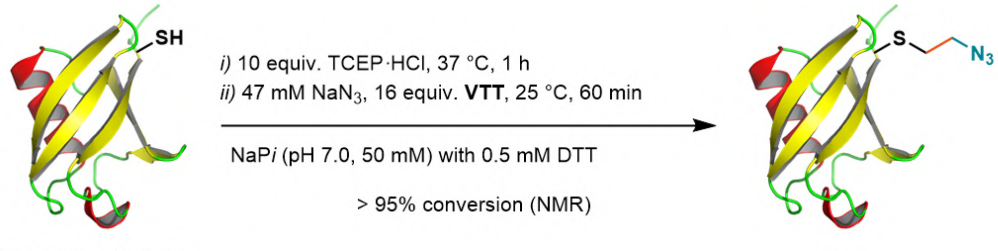

At 20–25 °C, 150 µL of Ubiquitin T12C (B) (1.0 mM, 0.15 µmol, 1.0 equiv.) in NaP_i buffer (pH 7.0, c = 50 mM) with 0.5 mM DTT and 0.05% NaN_3 was added to a 1.5 mL Eppendorf tube. Next, 0.24 mL of NaP_i buffer (pH 7.0, c = 50 mM) with 0.5 mM DTT was added. Then, 30 µL of a freshly prepared TCEP stock solution (0.050 M, 1.5 µmol, 0.38 mg, 10 equiv.) in NaP_i buffer (pH 7.0, c = 50 mM) with 0.5 mM DTT was added. The mixture was vortexed for 1 second, transferred into a Thermocycler pre-heated at 37 °C, and incubated at 37 °C for 1 hour at 400 rpm. Next, a 0.30 M sodium azide solution (78 µL, 23 µmol, 1.5 mg, 1.6 × 10^2 equiv.) in NaP_i buffer (pH 7.0, c = 50 mM) was added to the mixture at 20–25 °C (c_{Nu} = 47 mM), followed by addition of 2.4 µL of a **VTT** stock solution (1.0 M, 2.4 µmol, 0.79 mg, 16 equiv.) in DMF. The reaction mixture was vortexed for 1 second, transferred into a Thermocycler pre-heated at 25 °C, and incubated at 25 °C at 400 rpm for 60 minutes. Subsequently, the reaction mixture was centrifuged with 12.000 x g for 10 min at 4 °C. The resulting supernatant was transferred into an AMICON® filter unit with a 3 kDa cutoff and rebuffered three times into NaP_i buffer (pH 7.0, c = 50 mM) with 0.5 mM DTT. Next, the obtained protein solution was filled up to 160 µL with NaP_i buffer (pH 7.0, c = 50 mM) with 0.5 mM DTT and analyzed via LC/MS and protein NMR. NMR data can be found in the Protein-NMR section.

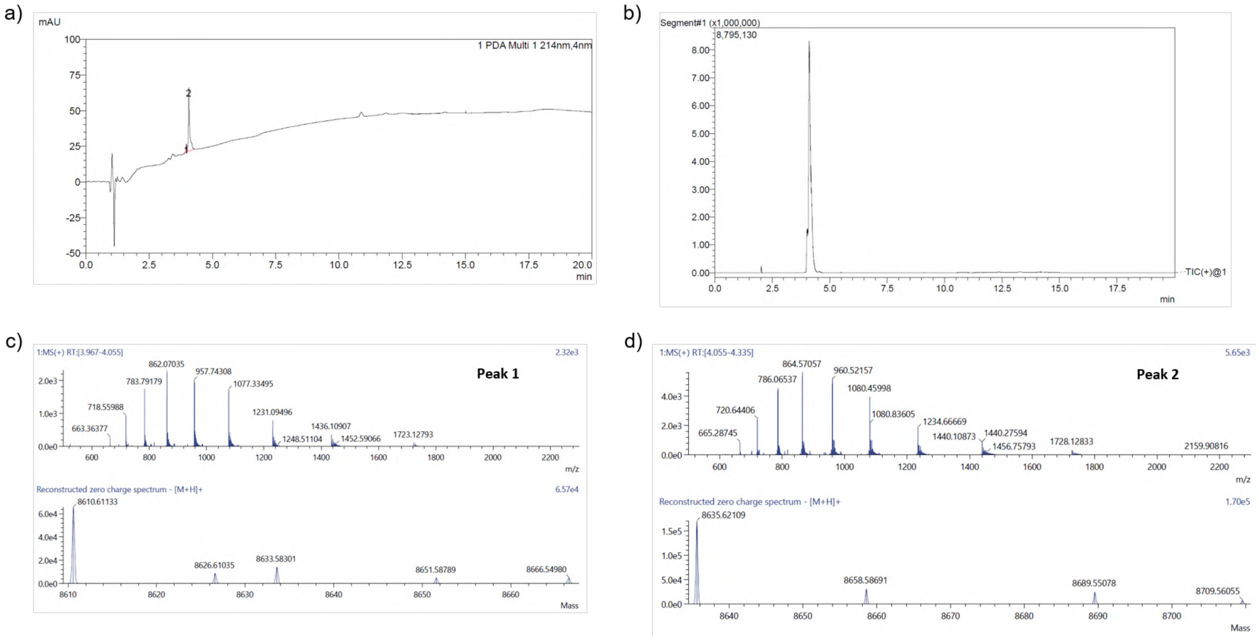

Figure S94. LC-MS analysis (Method B) of Ubiquitin T12C (B) after reaction with **VTT** and sodium azide: a) UV-trace b) total ion chromatogram c) Peak 1 ion series and deconvoluted spectrum d) Peak 2 ion series and deconvoluted spectrum; calculated masses: (Ubiquitin T12C (B))–S–C_2H_4–N_3: 8636 Da, (Ubiquitin T12C (B))–S–C_2H_4–OH: 8611 Da; Observed masses: Peak 1: 8611 Da; Peak 2: 8636 Da.

Synthesis of (^{15}N,^{13}C-Ubiquitin T12C)–S–$^{13}C_2H_4$–(4-F-aniline) (S17)

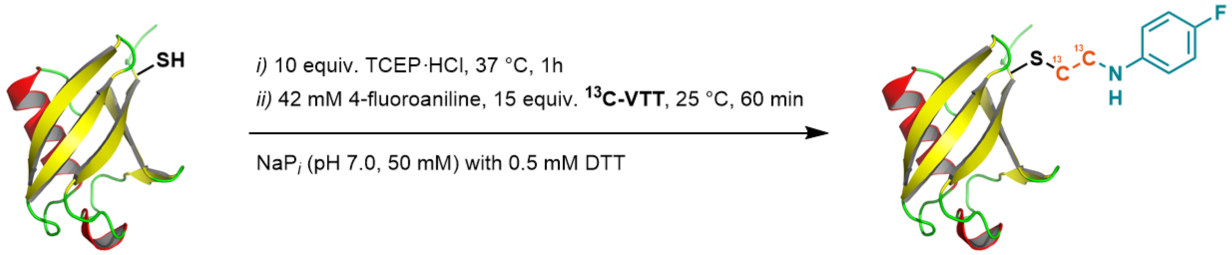

^{15}N,^{13}C-Ubiquitin T12C, 333 µM

S17

At 20–25 °C, 160 µL of ^{15}N,^{13}C-Ubiquitin T12C (1.0 mM, 0.16 µmol, 1.0 equiv.) in NaP_i buffer (pH 7.0, c = 50 mM) with 0.5 mM DTT was added to a 1.5 mL Eppendorf tube. Next, 0.11 mL of NaP_i buffer (pH 7.0, c = 50 mM) with 0.5 mM DTT was added. Then, 8.0 µL of a TCEP stock solution (0.20 M, 1.6 µmol, 0.4 mg, 10 equiv.) in UHQ-H_2O was added. The mixture was vortexed for 1 second, transferred into a Thermocycler pre-heated at 37 °C, and incubated at 37 °C for 1 hour at 400 rpm. Next, a 0.10 M 4-F-aniline solution (0.20 mL, 20 µmol, 2.2 mg, 1.2 × 10^2 equiv.) in NaP_i buffer (pH 7.0, c = 50 mM) was added to the mixture at 20–25 °C (c_{Nu} = 40 mM), followed by addition of 2.4 µL of a **$^{13}C_2$-VTT** stock solution (1.0 M, 2.4 µmol, 0.80 mg, 15 equiv.) in DMF. The reaction mixture was vortexed for 1 second, transferred into a Thermocycler pre-heated at 25 °C, and incubated at 25 °C at 400 rpm for 60 minutes. Subsequently, the reaction mixture was centrifuged with 12.000 x g for 10 min at 4 °C. The resulting supernatant was transferred into an AMICON® filter unit with a 3 kDa cutoff and rebuffered five times into NaP_i buffer (pH 7.0, c = 50 mM) with 0.5 mM DTT. Next, the obtained protein solution was filled up to 250 µL with NaP_i buffer (pH 7.0, c = 50 mM) with 0.5 mM DTT, transferred into a Shigemi NMR tube, and analyzed via NMR. NMR data can be found in the Protein-NMR section.

Synthesis of azidated conjugates for follow-up transformations

Synthesis of Ubiquitin T9C–S–C_2H_4–N_3 (4)

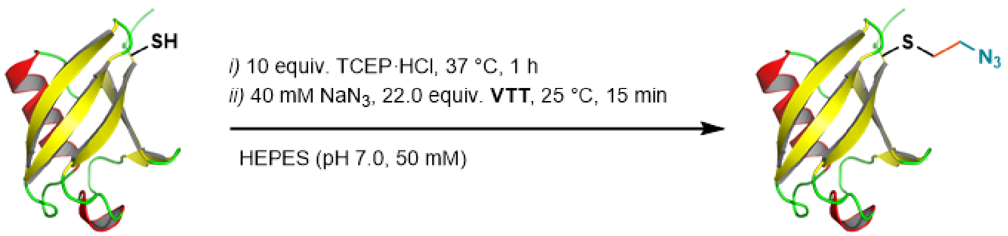

Ubiquitin T9C, 100 µM

4, 63%

At 20–25 °C, 382 µL of Ubiquitin T9C (1.89 mg/mL, 209 µM, 80.0 nmol, 1.0 equiv.) in HEPES buffer (pH 7.0, c = 50 mM) was added to a 1.5 mL Eppendorf tube. Next, 269 µL HEPES buffer (pH 7.0, c = 50 mM) was added. Then, 16.0 µL of a TCEP stock solution (50 mM, 800 nmol, 229 µg, 10 equiv.) in UHQ-H_2O was added. The mixture was vortexed for 1 second, transferred into a Thermocycler pre-heated at 37 °C, and incubated at 37 °C for 1 hour at 400 rpm. Next, a 300 mM sodium azide solution (133 µL, 40.0 µmol, 2.60 mg, 500 equiv.) in HEPES buffer (pH 7.0, c = 50 mM) was added to the mixture at 20–25 °C (c_{Nu} = 40 mM), followed by addition of 7.0 µL of a **VTT** stock solution (0.25 M, 1.8 µmol, 0.58 mg, 22 equiv.) in DMF. The reaction mixture was

vortexed for 1 second, transferred into a Thermocycler pre-heated at 25 °C, and incubated at 25 °C at 400 rpm for 15 minutes. Subsequently, 7.0 µL of a β-mercaptoethanol stock solution (0.25 M, 1.8 µmol, 0.16 mg, 22 equiv.) in UHQ-H_2O was added, and the mixture was incubated at 25 °C for 15 minutes. The product mixture was rebuffered 4 times in HEPES buffer (pH 7.0, c = 50 mM) using an AMICON® filter with 3 kDa cutoff. The obtained mixture was analyzed by LC-MS. The obtained chromatograms were in accordance with the results shown in the protein scope (Figure S54). The yield was determined by comparing means of PDA integrals of the product peak from four experiments with a calibration curve of the starting material (Figure S32). The obtained $A_{214\ nm}$ integral of the product peak was used to calculate the concentration of the product. Afterwards, the yield was determined via the following equation:

$$yield = \frac{\beta_{product} \cdot V_{product}}{\beta_{starting\ material} \cdot V_{starting\ material}} \cdot 100\%$$

The yield should be considered semi-quantitative because of altered potentially altered extinction coefficient of the starting material and the desired product.

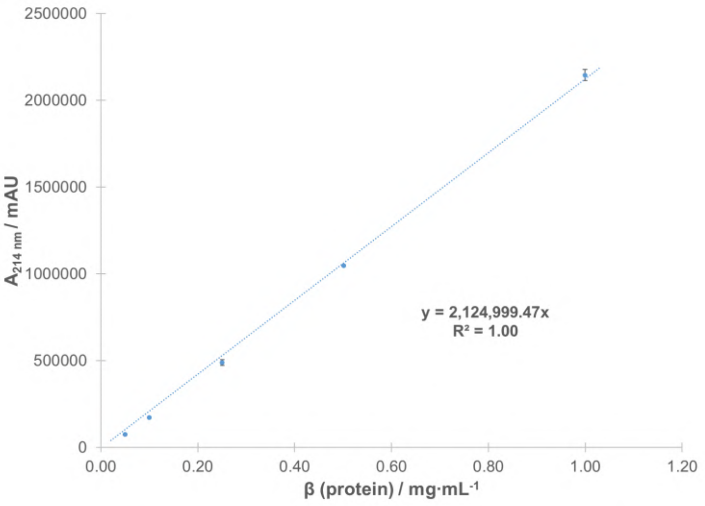

Figure S95. Calibration curve for Ubiquitin T9C. The absorption values of unmodified protein utilized to calculate the calibration curve are shown as means ± SD resulting from three technical replicates, respectively.

Synthesis of Ubiquitin T12C–S–C_2H_4–N_3 (11)

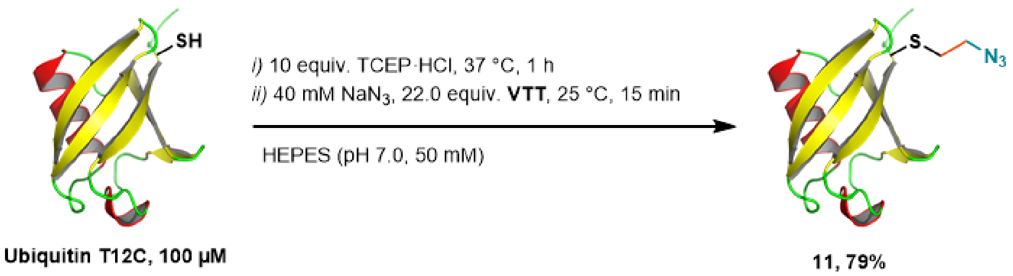

At 20–25 °C, 642 µL of Ubiquitin T12C (1.12 mg/mL, 209 µM, 80 nmol, 1.0 equiv.) in HEPES buffer (pH 7.0, c = 50 mM) was added to a 1.5 mL Eppendorf tube. Next, 9.0 µL HEPES buffer (pH 7.0, c = 50 mM) was added. Then, 16 µL of a TCEP stock solution (50 mM, 800 nmol, 229 µg, 10 equiv.) in UHQ-H_2O was added. The mixture was vortexed for 1 second, transferred into a Thermocycler pre-heated at 37 °C, and incubated at 37 °C

for 1 hour at 400 rpm. Next, a 300 mM sodium azide solution (133 µL, 40.0 µmol, 2.60 mg, 500 equiv.) in HEPES buffer (pH 7.0, c = 50 mM) was added to the mixture at 20–25 °C (c_{Nu} = 40 mM), followed by addition of 7.0 µL of a **VTT** stock solution (0.25 M, 1.8 µmol, 0.58 mg, 22 equiv.) in DMF. The reaction mixture was vortexed for 1 second, transferred into a Thermocycler pre-heated at 25 °C, and incubated at 25 °C at 400 rpm for 15 minutes. Subsequently, 7.0 µL of a β-mercaptoethanol stock solution (0.25 M, 1.8 µmol, 0.16 mg, 22 equiv.) in UHQ-H_2O was added, and the mixture was incubated at 25 °C for 15 minutes. The product mixture was rebuffered 4 times in HEPES buffer (pH 7.0, c = 50 mM) using an AMICON® filter with 3 kDa cutoff. The obtained mixture was analyzed by LC-MS. The obtained chromatograms were in accordance with the results shown in the functional group scope (Figure S64). The yield was determined by comparing means of PDA integrals of the product peak from four experiments with a calibration curve of the starting material (Figure S35). The obtained $A_{214\ nm}$ integral of the product peak was used to calculate the concentration of the product. Afterwards, the yield was determined via the following equation:

$$yield = \frac{\beta_{product} \cdot V_{product}}{\beta_{starting\ material} \cdot V_{starting\ material}} \cdot 100\%$$

The yield should be considered semi-quantitative because of altered potentially altered extinction coefficient of the starting material and the desired product.

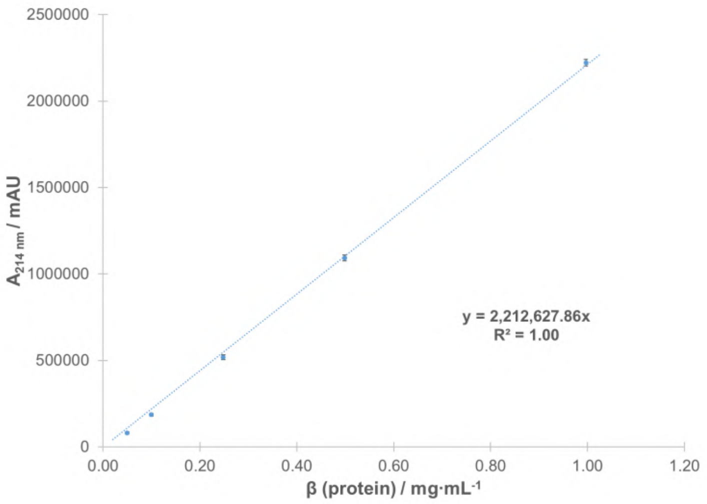

Figure S96. Calibration curve for Ubiquitin T12C. The absorption values of unmodified protein utilized to calculate the calibration curve are shown as means ± SD resulting from three technical replicates, respectively.

Synthesis of (BSA)–S–C_2H_4–N_3 (7)

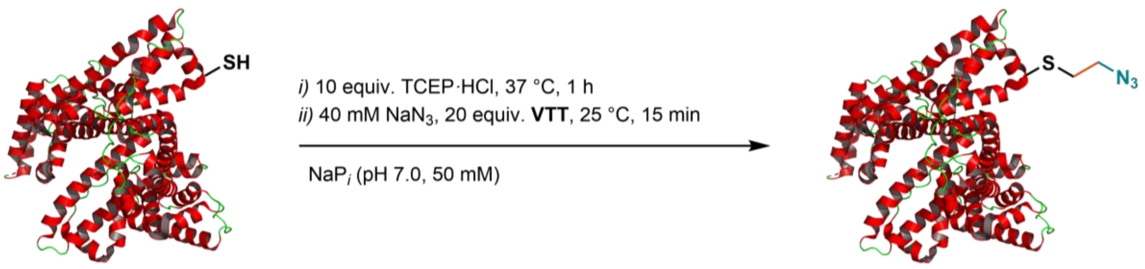

BSA, 8 µM **7**

At 20–25 °C, 13 µL of BSA (2.00 mg/mL, 30.0 µM, 0.40 nmol, 1.0 equiv.) in NaP$_i$ buffer (pH 7.0, c = 50 mM) was added to a 1.5 mL Eppendorf tube. Next, 29 µL NaP$_i$ buffer (pH 7.0, c = 50 mM) was added. Then, 0.8 µL of a TCEP stock solution (0.08 mM, 4 nmol, 1 µg, 1x10^1 equiv.) in UHQ-H_2O was added. The mixture was vortexed for 1 second, transferred into a Thermocycler pre-heated at 37 °C, and incubated at 37 °C for 1 hour at 400 rpm. Next, a 0.3 M sodium azide solution (6.7 µL, 2.0 µmol, 0.13 mg, 50x10^2 equiv.) in NaP$_i$ buffer (pH 7.0, c = 50 mM) was added to the mixture at 20–25 °C (c_{Nu} = 40 mM), followed by addition of 0.5 µL of a **VTT** stock solution (16 mM, 8 nmol, 3 µg, 2x10^1 equiv.) in DMF. The reaction mixture was vortexed for 1 second, transferred into a Thermocycler pre-heated at 25 °C, and incubated at 25 °C at 400 rpm for 15 minutes. Subsequently, 0.5 µL of a β-mercaptoethanol stock solution (16 mM, 8 nmol, 0.7 µg, 2x10^1 equiv.) in UHQ-H_2O was added, and the mixture was incubated at 25 °C for 15 minutes. The product mixture was rebuffered 4 times in HEPES buffer (pH 7.0, c = 50 mM) using an AMICON® filter with 50 kDa cutoff. The obtained mixture was analyzed by SDS-PAGE after conjugation via CuAAC with a fluorescent dye (See Figure S102).

Synthesis of (MDAR2)–S–C_2H_4–N_3 (S13)

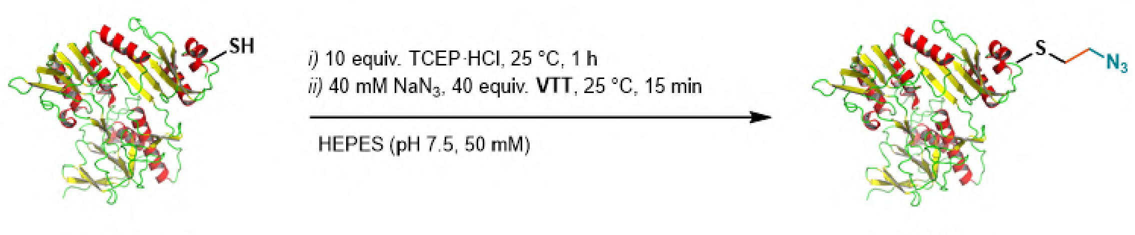

At 20–25 °C, 17 µL of MDAR2 (1.21 mg/mL, 24 µM, 0.40 nmol, 1.0 equiv.) in HEPES buffer (pH 7.5, c = 50 mM) was added to a 1.5 mL Eppendorf tube. Next, 26 µL HEPES buffer (pH 7.5, c = 50 mM) was added. Then, 0.8 µL of a TCEP stock solution (0.08 mM, 4 nmol, 1 µg, 1x10^1 equiv.) in UHQ-H_2O was added. The mixture was vortexed for 1 second, transferred into a Thermocycler pre-heated at 37 °C, and incubated at 37 °C for 1 hour at 400 rpm. Next, a 0.3 M sodium azide solution (6.7 µL, 2.0 µmol, 0.13 mg, 50x10^2 equiv.) in HEPES buffer (pH 7.5, c = 50 mM) was added to the mixture at 20–25 °C (c_{Nu} = 40 mM), followed by addition of 1.0 µL of a **VTT** stock solution (16 mM, 16 nmol, 5.3 µg, 40 equiv.) in DMF. The reaction mixture was vortexed for 1 second, transferred into a Thermocycler pre-heated at 25 °C, and incubated at 25 °C at 400 rpm for 15 minutes. Subsequently, 1.0 µL of a β-mercaptoethanol stock solution (16 mM, 16 nmol, 1.5 µg, 40 equiv.) in UHQ-H_2O was added, and the mixture was incubated at 25 °C for 15 minutes. The product mixture was rebuffered 4 times in HEPES buffer (pH 7.0, c = 50 mM) using an AMICON® filter with 10 kDa cutoff. The obtained mixture was analyzed by SDS-PAGE after conjugation via CuAAC with a fluorescent dye (See Figure S102).

Synthesis of (MDAR3)–S–C_2H_4–N_3 (6)

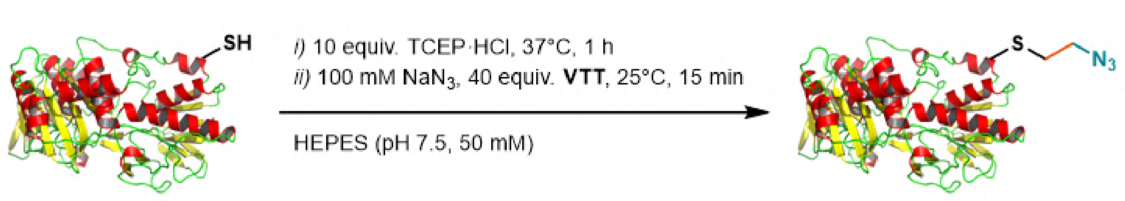

MDAR3 6

At 20–25 °C, 10 µL of MDAR3 (2.01 mg/mL, 39 µM, 0.40 nmol, 1.0 equiv.) in HEPES buffer (pH 7.5, c = 50 mM) was added to a 1.5 mL Eppendorf tube. Next, 22 µL HEPES buffer (pH 7.5, c = 50 mM) was added. Then, 0.8 µL of a TCEP stock solution (5 mM, 4 nmol, 1 µg, $1x10^1$ equiv.) in UHQ-H_2O was added. The mixture was vortexed for 1 second, transferred into a Thermocycler pre-heated at 37 °C, and incubated at 37 °C for 1 hour at 400 rpm. Next, a 0.3 M sodium azide solution (17 µL , 5.0 µmol, 0.33 mg, $13x10^4$ equiv.) in HEPES buffer (pH 7.5, c = 50 mM) was added to the mixture at 20–25 °C (c_{Nu} = 100 mM), followed by addition of 1.0 µL of a **VTT** stock solution (16 mM, 16 nmol, 5.3 µg, 40 equiv.) in DMF. The reaction mixture was vortexed for 1 second, transferred into a Thermocycler pre-heated at 25 °C, and incubated at 25 °C at 400 rpm for 15 minutes. Subsequently, 1.0 µL of a β-mercaptoethanol stock solution (16 mM, 16 nmol, 1.5 µg, 40 equiv.) in UHQ-H_2O was added, and the mixture was incubated at 25 °C for 15 minutes. The product mixture was rebuffered 4 times in HEPES buffer (pH 7.0, c = 50 mM) using an AMICON® filter with 10 kDa cutoff. The obtained mixture was analyzed by SDS-PAGE after conjugation via CuAAC with a fluorescent dye (See Figure S102).

Modification of the installed azide-group via Click-chemistry

CuAAC between (Ubiquitin T9C)–S–C_2H_4–N_3 and 6-FAM

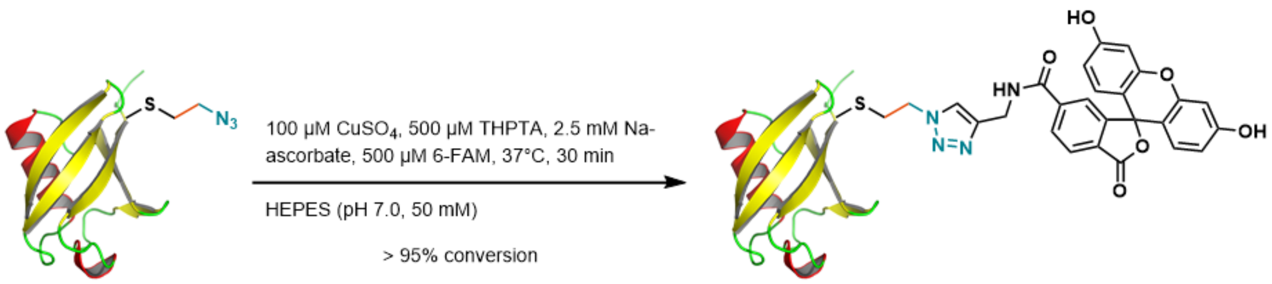

Ubiquitin T9C, 15 µM S18

At 20–25 °C, 10 µL of Ubiquitin T9C–S–C_2H_4–N_3 (1.39 mg/mL (concentration determined by BCA-assay), 118 µM, 1.5 nmol, 1.0 equiv.) in HEPES buffer (pH 7.0, c = 50 mM) was added to a 1.5 mL Eppendorf tube. Next, 83 µL of HEPES buffer (pH 7.0, c = 50 mM) was added. Then, 2.1 µL of 6-FAM alkyne solution (24 mM, 0.05 µmol, 2×10^1 µg, 3×10^1 equiv.) in DMSO was added. Next, 2.0 µL of Cu-THPTA mixture (10 mM $CuSO_4$ and 50 mM THPTA in UHQ-H_2O) premixed at 37 °C for 5 minutes was added. Then, 2.5 µL of a sodium ascorbate stock solution (0.10 M, 0.25 µmol, 50 µg, 1.7×10^2 equiv.) in UHQ-H_2O was added. The mixture was vortexed for 1 second, transferred into a Thermocycler pre-heated at 37 °C, and incubated at 37 °C for 30 minutes at 400 rpm. The obtained mixture was analyzed by LC-MS.

The product mixture was rebuffered 4 times in HEPES buffer (pH 7.0, c = 50 mM) using an AMICON® filter with 3 kDa cutoff before the mixture was analyzed by SDS-PAGE (See Figure S101).

HRMS-ESI (m/z) calc'd for $C_{415}{}^{13}C_5H_{682}N_{116}O_{130}S_2{}^{12+}$ $[M+12H]^{12+}$, 791.6655; found, 791.6660; deviation: 0.6 ppm.

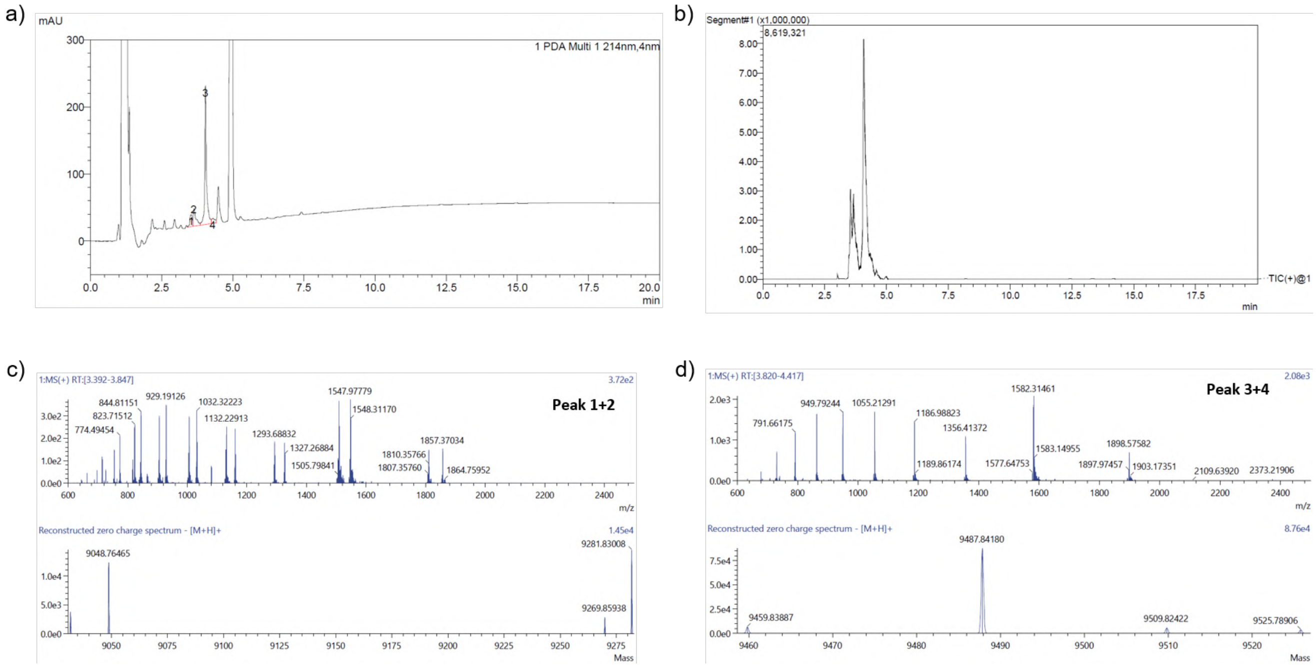

Figure S97. LC-MS analysis (Method B) of Ubiquitin T9C after CuAAC with 6-FAM: a) UV-trace b) total ion chromatogram c) Peak 1+2 ion series and deconvoluted spectrum d) Peak 3+4 ion series and deconvoluted spectrum; calculated masses: (Ubiquitin T9C)–S–C_2H_4–OH: 9050 Da, (Ubiquitin T9C)–6-FAM click-product: 9488 Da; Observed masses: Peak 1+2: 9049 Da; Peak 3+4: 9488 Da.

CuAAC between (Ubiquitin T12C)–S–C_2H_4–N_3 and 6-FAM

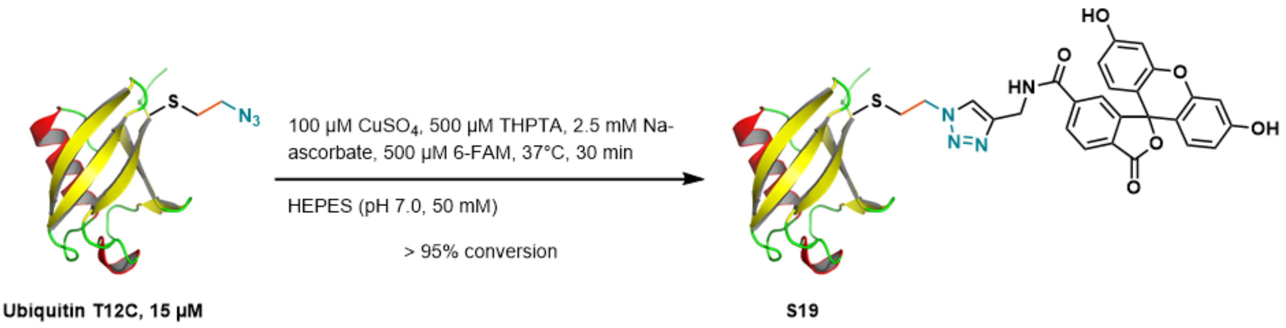

At 20–25 °C, 13 µL of Ubiquitin T12C–S–C_2H_4–N_3 (1.07 mg/mL (concentration determined by BCA-assay), 153 µM, 1.5 nmol, 1.0 equiv.) in HEPES buffer (pH 7.0, c = 50 mM) was added to a 1.5 mL Eppendorf tube. Next, 86 µL of HEPES buffer (pH 7.0, c = 50 mM) was added. Then, 2.1 µL of 6-FAM alkyne solution (24 mM, 0.05 µmol, 2 × 10^1 µg, 3 × 10^1 equiv.) in DMSO was added. Next, 2.0 µL of Cu-THPTA mixture (10 mM $CuSO_4$ and 50 mM THPTA in UHQ-H_2O) premixed at 37 °C for 5 minutes was added. Then, 2.5 µL of a sodium ascorbate stock solution (0.10 M, 0.25 µmol, 50 µg, 1.7 × 10^2 equiv.) in UHQ-H_2O was added. The mixture was vortexed for 1 second, transferred into a Thermocycler pre-heated at 37 °C, and incubated at 37 °C for

30 minutes at 400 rpm. The obtained mixture was analyzed by LC-MS.

The product mixture was rebuffered 4 times in HEPES buffer (pH 7.0, c = 50 mM) using an AMICON® filter with 3 kDa cutoff before the mixture was analyzed by SDS-PAGE (See Figure S101).

HRMS-ESI (m/z) calc'd for $C_{415}{}^{13}C_5H_{682}N_{116}O_{130}S_2{}^{12+}$ $[M+12H]^{12+}$, 791.6655; found, 791.6662; deviation: 0.9 ppm.

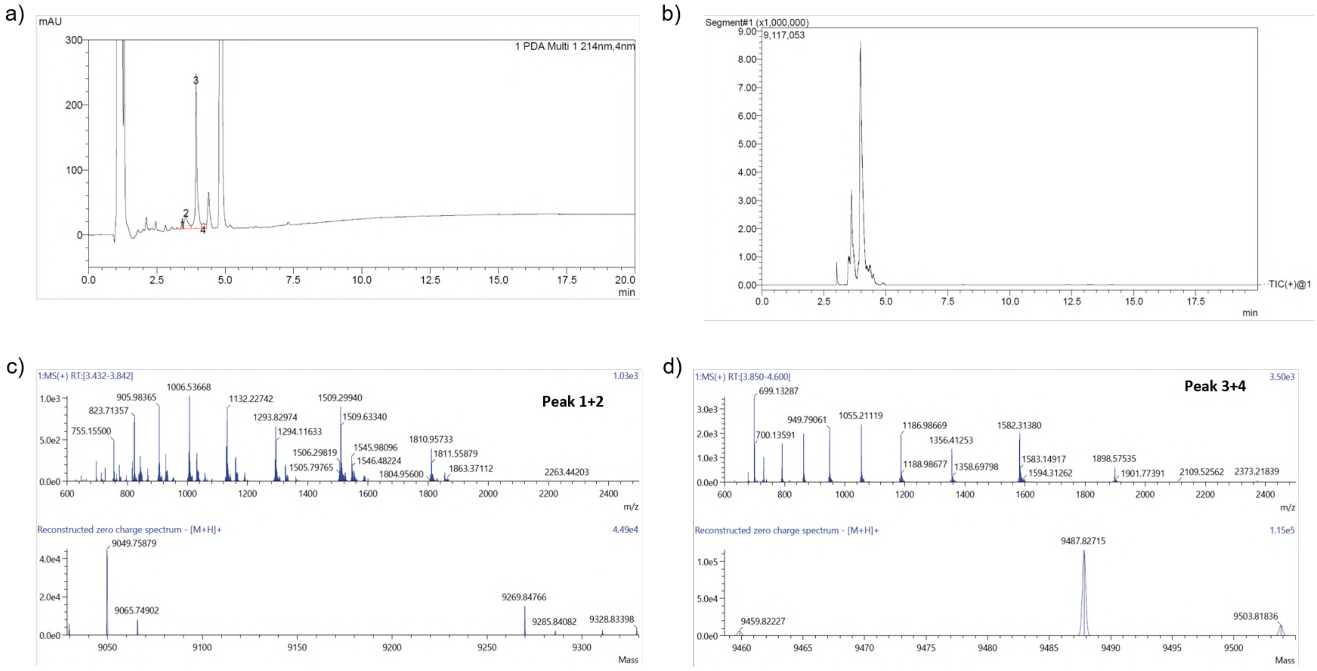

Figure S98. LC-MS analysis (Method B) of Ubiquitin T12C after CuAAC with 6-FAM: a) UV-trace b) total ion chromatogram c) Peak 1+2 ion series and deconvoluted spectrum d) Peak 3+4 ion series and deconvoluted spectrum; calculated masses: (Ubiquitin T12C)–S–C_2H_4–OH: 9050 Da, (Ubiquitin T12C)–6-FAM click-product: 9488 Da; Observed masses: Peak 1+2: 9050 Da; Peak 3+4: 9488 Da.

CuAAC between (BSA)–S–C_2H_4–N_3 and 6-FAM

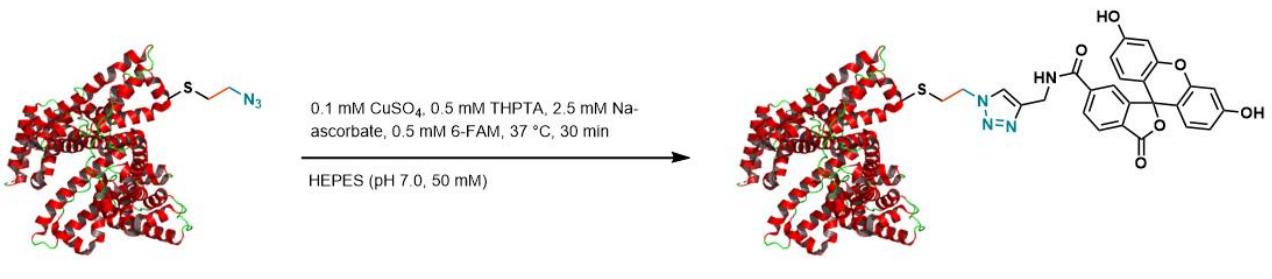

(BSA)–S–C_2H_4–N_3
15 µM

S20

At 20–25 °C, 166 µL of BSA–S–C_2H_4–N_3 (2.00 mg/mL (concentration determined by BCA-assay), 30 µM, 1.5 nmol, 1.0 equiv.) in HEPES buffer (pH 7.0, c = 50 mM) was added to a 1.5 mL Eppendorf tube. Next, 83 µL of HEPES buffer (pH 7.0, c = 50 mM) was added. Then, 2.1 µL of 6-FAM alkyne solution (24 mM, 0.05 µmol, 2 × 10^1 µg, 3 × 10^1 equiv.) in DMSO was added. Next, 2.0 µL of Cu-THPTA mixture (10 mM $CuSO_4$ and 50 mM THPTA in UHQ-H_2O) premixed at 37 °C for 5 minutes was added. Then, 2.5 µL of a sodium ascorbate stock solution (0.10 M, 0.25 µmol, 50 µg, 1.7 × 10^2 equiv.) in UHQ-H_2O was added. The mixture was vortexed for 1 second, transferred into a Thermocycler pre-heated at 37 °C, and incubated at 37 °C for 30 minutes at 400 rpm.

The product mixture was rebuffered 4 times in HEPES buffer (pH 7.0, c = 50 mM) using an AMICON® filter with 50 kDa cutoff before the mixture was analyzed by SDS-PAGE (See Figure S102).

CuAAC between (MDAR2)–S–C_2H_4–N_3 and 6-FAM

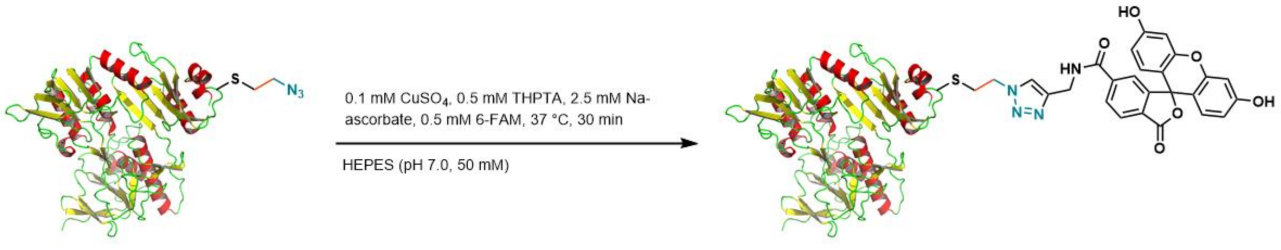

(MDAR2)–S–C_2H_4–N_3
15 µM

S21

At 20–25 °C, 10 µL of MDAR2–S–C_2H_4–N_3 (1.39 mg/mL (concentration determined by BCA-assay), 118 µM, 1.5 nmol, 1.0 equiv.) in HEPES buffer (pH 7.0, c = 50 mM) was added to a 1.5 mL Eppendorf tube. Next, 83 µL of HEPES buffer (pH 7.0, c = 50 mM) was added. Then, 2.1 µL of 6-FAM alkyne solution (24 mM, 0.05 µmol, 2 × 10^1 µg, 3 × 10^1 equiv.) in DMSO was added. Next, 2.0 µL of Cu-THPTA mixture (10 mM $CuSO_4$ and 50 mM THPTA in UHQ-H_2O) premixed at 37 °C for 5 minutes was added. Then, 2.5 µL of a sodium ascorbate stock solution (0.10 M, 0.25 µmol, 50 µg, 1.7 × 10^2 equiv.) in UHQ-H_2O was added. The mixture was vortexed for 1 second, transferred into a Thermocycler pre-heated at 37 °C, and incubated at 37 °C for 30 minutes at 400 rpm.

The product mixture was rebuffered 4 times in HEPES buffer (pH 7.0, c = 50 mM) using an AMICON® filter with 10 kDa cutoff before the mixture was analyzed by SDS-PAGE (See Figure S102).

CuAAC between (MDAR3)–S–C_2H_4–N_3 and 6-FAM

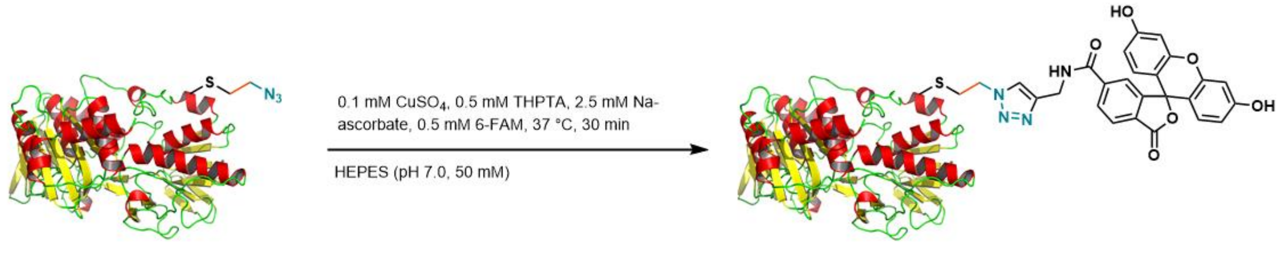

(MDAR3)–S–C_2H_4–N_3
15 µM

S22

At 20–25 °C, 10 µL of MDAR3–S–C_2H_4–N_3 (1.39 mg/mL (concentration determined by BCA-assay), 118 µM, 1.5 nmol, 1.0 equiv.) in HEPES buffer (pH 7.0, c = 50 mM) was added to a 1.5 mL Eppendorf tube. Next, 83 µL of HEPES buffer (pH 7.0, c = 50 mM) was added. Then, 2.1 µL of 6-FAM alkyne solution (24 mM, 0.05 µmol, 2 × 10^1 µg, 3 × 10^1 equiv.) in DMSO was added. Next, 2.0 µL of Cu-THPTA mixture (10 mM $CuSO_4$ and 50 mM THPTA in UHQ-H_2O) premixed at 37 °C for 5 minutes was added. Then, 2.5 µL of a sodium ascorbate stock solution (0.10 M, 0.25 µmol, 50 µg, 1.7 × 10^2 equiv.) in UHQ-H_2O was added. The mixture was vortexed for 1 second, transferred into a Thermocycler pre-heated at 37 °C, and incubated at 37 °C for 30 minutes at 400 rpm.

The product mixture was rebuffered 4 times in HEPES buffer (pH 7.0, c = 50 mM) using an AMICON® filter with

10 kDa cutoff before the mixture was analyzed by SDS-PAGE (See Figure S102).

SPAAC between (Ubiquitin T9C)–S–C_2H_4–N_3 and BCN

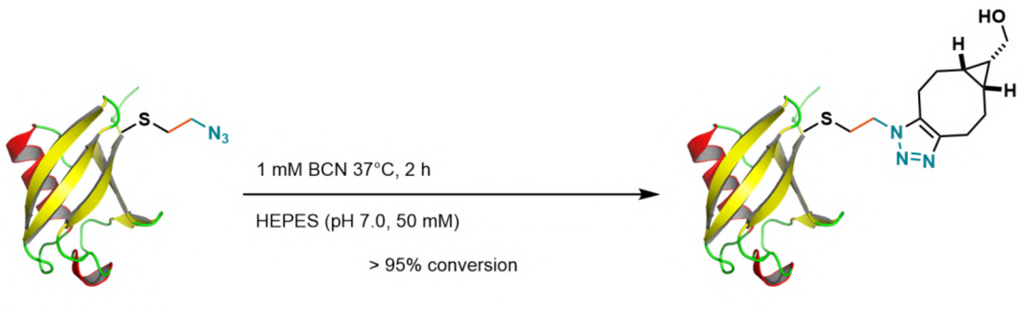

Ubiquitin T9C, 15 µM **S23**

At 20–25 °C, 10 µL of (Ubiquitin T9C)–S–C_2H_4–N_3 (1.39 mg/mL (concentration determined by BCA-assay), 153 µM, 1.5 nmol, 1.0 equiv.) in HEPES buffer (pH 7.0, c = 50 mM) was added to a 1.5 mL Eppendorf tube. Next, 90 µL of HEPES buffer (pH 7.0, c = 50 mM) was added. Then, 2.1 µL of BCN alkyne solution (48 mM, 0.10 µmol, 1 × 10^1 µg, 7 × 10^1 equiv.) in DMSO was added. The mixture was vortexed for 1 second, transferred into a Thermocycler pre-heated at 37 °C, and incubated at 37 °C for 120 minutes at 400 rpm. The obtained mixture was analyzed by LC-MS.

HRMS-ESI (m/z) calc'd for $C_{401}{}^{13}C_5H_{681}N_{115}O_{125}S_2{}^{12+}$ $[M+12H]^{12+}$, 769.7501; found, 769.7502; deviation: 0.1 ppm.

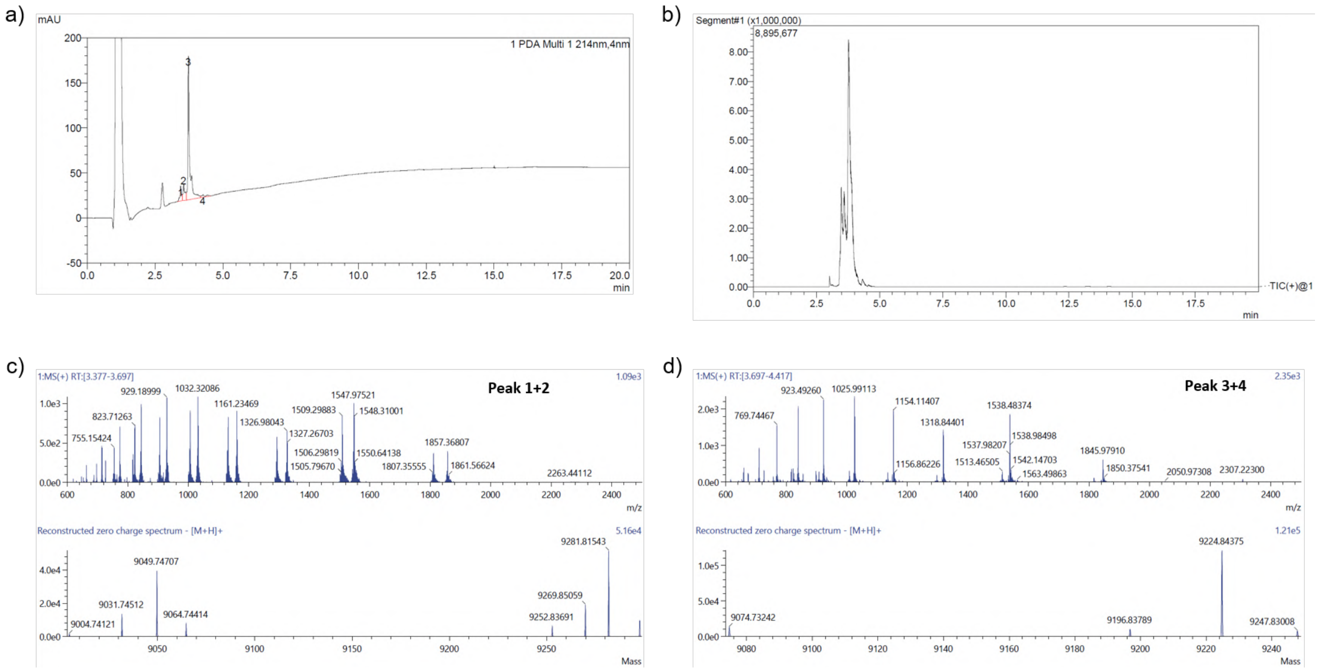

Figure S99. LC-MS analysis (Method B) of Ubiquitin T9C after SPAAC with BCN: a) UV-trace b) total ion chromatogram c) Peak 1+2 ion series and deconvoluted spectrum d) Peak 3+4 ion series and deconvoluted spectrum; calculated masses: (Ubiquitin T9C)–S–C_2H_4–N_3: 9075 Da, (Ubiquitin T9C)–S–C_2H_4–OH: 9050 Da, (Ubiquitin T9C)–BCN SPAAC-product: 9225 Da; Observed masses: Peak 1+2: 9050 Da; Peak 3+4: 9075 Da, 9225 Da.

SPAAC between (Ubiquitin T12C)–S–C_2H_4–N_3 and BCN

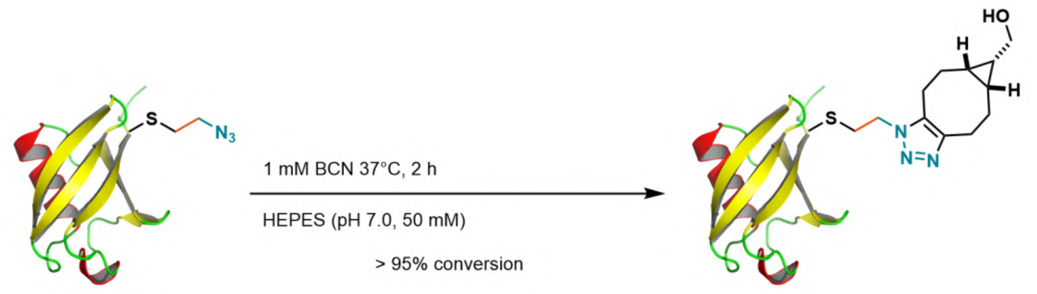

Ubiquitin T12C, 15 µM **S24**

At 20–25 °C, 13 µL of Ubiquitin T12C–S–C_2H_4–N_3 (1.07 mg/mL (concentration determined by BCA-assay), 118 µM, 1.5 nmol, 1.0 equiv.) in HEPES buffer (pH 7.0, c = 50 mM) was added to a 1.5 mL Eppendorf tube. Next, 87 µL of HEPES buffer (pH 7.0, c = 50 mM) was added. Then, 2.1 µL of BCN alkyne solution (48 mM, 0.10 µmol, 1 × 10^1 µg, 7 × 10^1 equiv.) in DMSO was added. The mixture was vortexed for 1 second, transferred into a Thermocycler pre-heated at 37 °C, and incubated at 37 °C for 120 minutes at 400 rpm. The obtained mixture was analyzed by LC-MS.

HRMS-ESI (m/z) calc'd for $C_{401}{}^{13}C_5H_{681}N_{115}O_{125}S_2{}^{12+}$ $[M+12H]^{12+}$, 769.7501; found, 769.7504; deviation: 0.4 ppm.

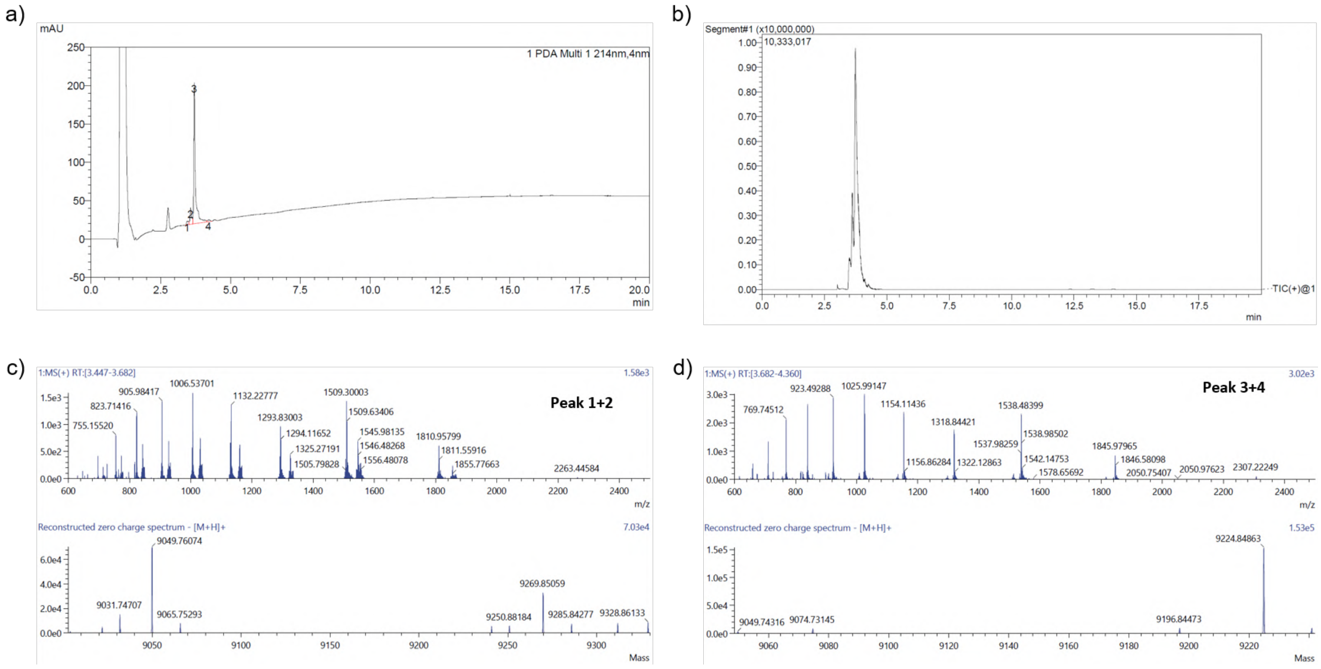

Figure S100. LC-MS analysis (Method B) of Ubiquitin T12C after SPAAC with BCN: a) UV-trace b) total ion chromatogram c) Peak 1+2 ion series and deconvoluted spectrum d) Peak 3+4 ion series and deconvoluted spectrum; calculated masses: (Ubiquitin T12C)–S–C_2H_4–N_3: 9075 Da, (Ubiquitin T12C)–S–C_2H_4–OH: 9050 Da, (Ubiquitin T12C)–BCN SPAAC-product: 9225 Da; Observed masses: Peak 1+2: 9050 Da; Peak 3+4: 9050 Da, 9075 Da, 9225 Da.

SDS PAGE of functionalized proteins

Ubiquitin T9C and Ubiquitin T12C functionalized with azide and 6-FAM

Lane	1	2	3	4	5	6	7	8	9	10	11	12	13	14
Assignment	Ladder mixture	Ubiquitin T9C	Ubiquitin T9C–S–C_2H_4–N_3	Ubiquitin T9C-6-FAM	Ubiquitin T12C	Ubiquitin T12C–S–C_2H_4–N_3	Ubiquitin T12C-6-FAM	Ladder mixture	Ubiquitin T9C, dilution 1:2	Ubiquitin T9C–S–C_2H_4–N_3, dilution 1:2	Ubiquitin T9C-6-FAM, dilution 1:2	Ubiquitin T12C, dilution 1:2	Ubiquitin T12C–S–C_2H_4–N_3, dilution 1:2	Ubiquitin T12C-6-FAM, dilution 1:2

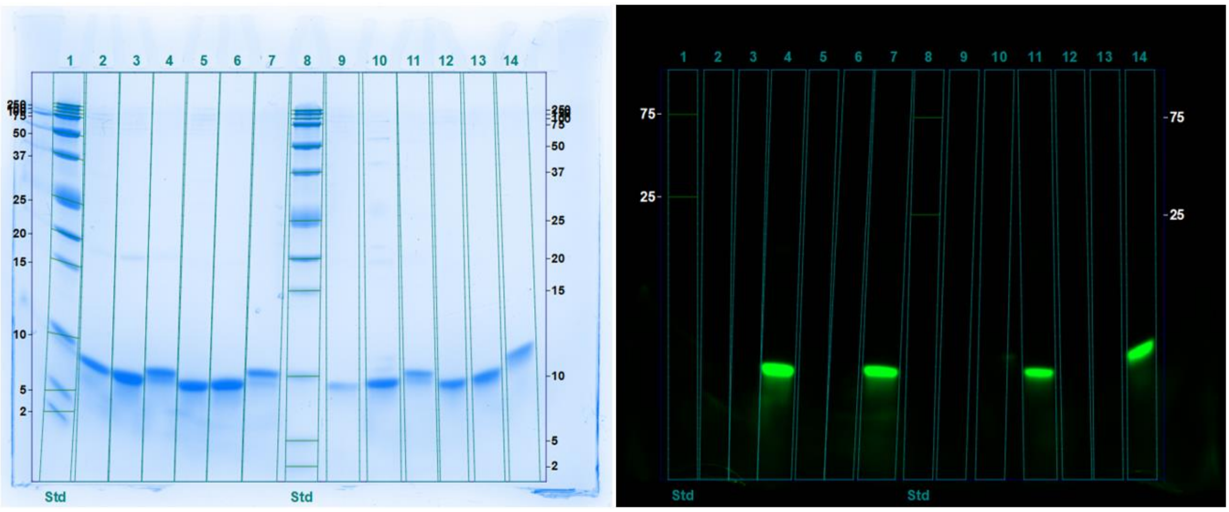

Figure S101. Images of the protein gel after Coomassie staining (left) and under irradiation utilizing the Fluorescein application (right). The experiment was conducted as a single experiment.

BSA, MDAR2, MDAR3 functionalized with azide and 6-FAM

Lane	1	2	3	4	5	6	7	8
Assignment	Ladder mixture	BSA–S–C_2H_4–N_3	BSA-6-FAM	MDAR2–S–C_2H_4–N_3	MDAR2-6-FAM	MDAR3–S–C_2H_4–N_3	MDAR3-6-FAM	Ladder mixture

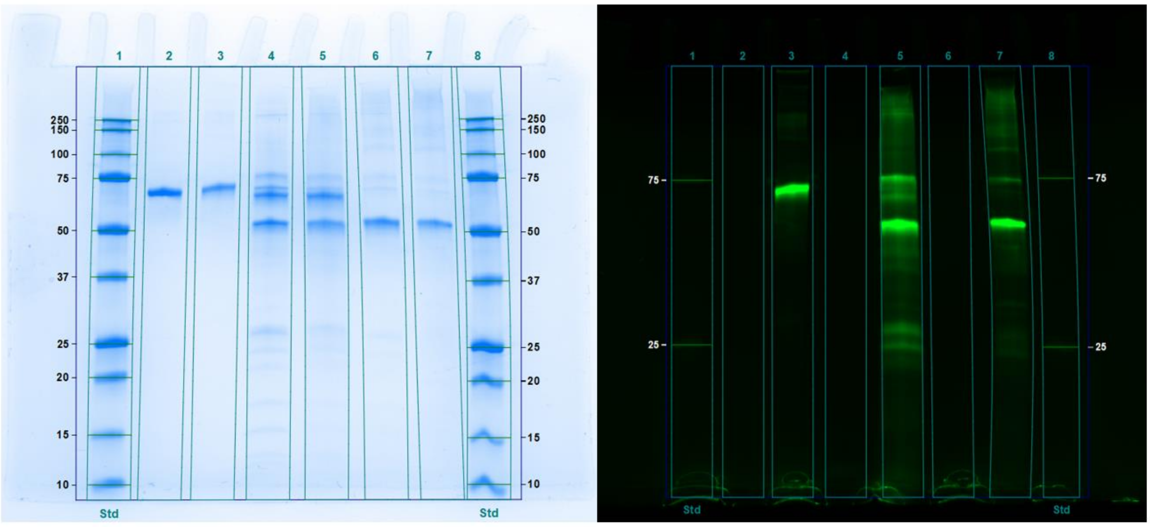

Figure S102. Images of the protein gel after Coomassie staining (left) and under irradiation utilizing the Fluorescein application (right). The experiment was conducted as a single experiment.

Control Experiments

Detailed analysis of a model LC-MS chromatogram

Following example showcases how LC-MS spectra for protein reactions were analyzed.

Only peaks with masses corresponding to protein products were used for the quantification of the reactions. Mass spectra of all peaks on a model chromatogram are shown below. In this case, only peak 7 was analyzed. The background peaks were present in blank measurements without any sample added. Hence, the background peaks were not analyzed further.

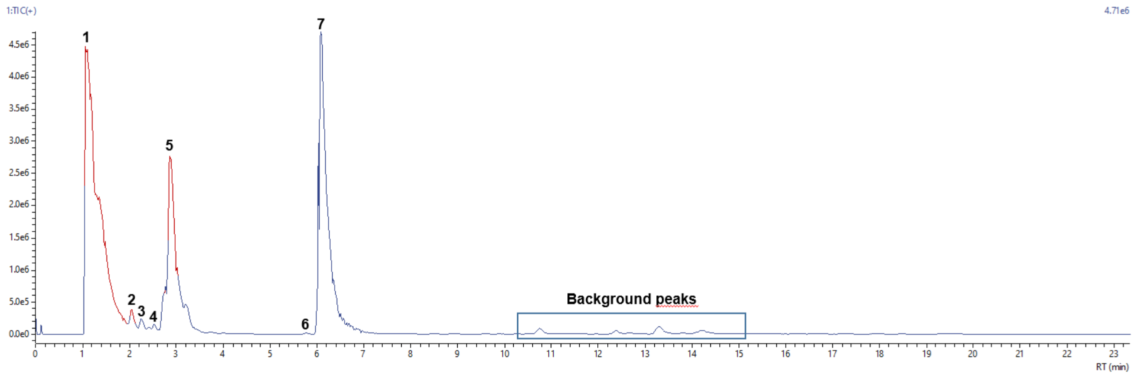

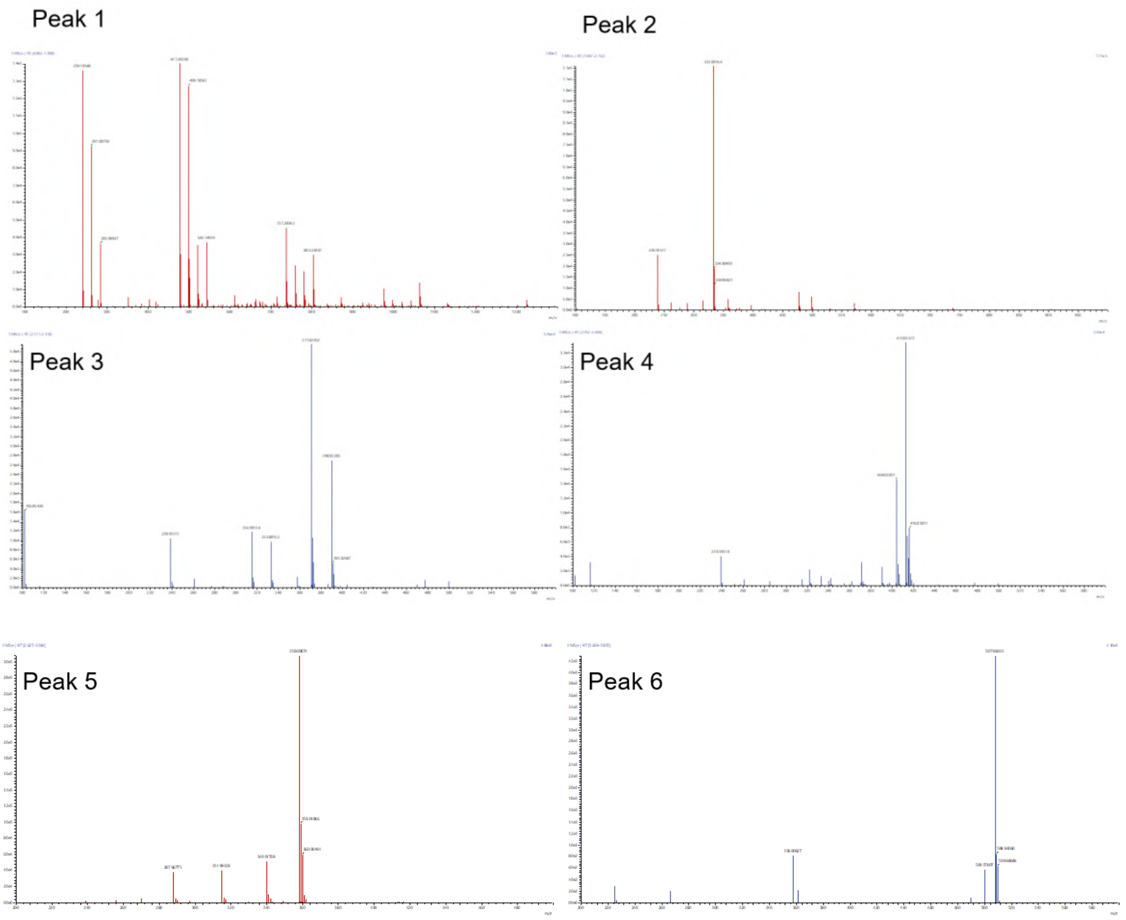

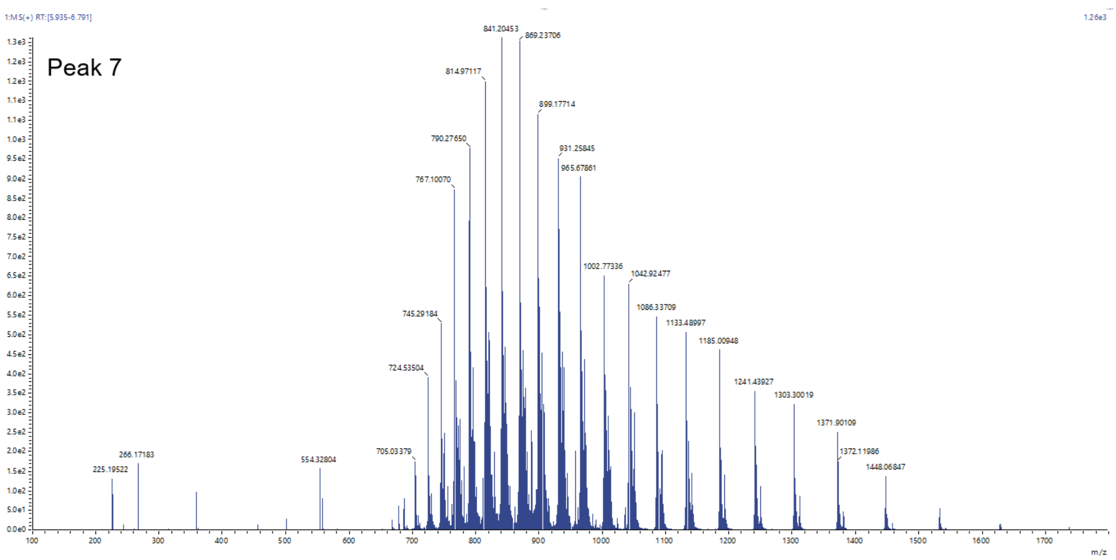

Figure S103. UV-vis chromatogram of the reaction mixture with pre-reduced DHAR1 S97C, **VTFT**, and sodium azide. For the general approach all peaks were assigned and the corresponding mass spectra are shown. All peaks that do not contain protein: e.g. small molecules or buffer were ignored in the analysis and not considered for yield and conversion determination.

Estimation of protein loss during the reaction

In order to exclude protein loss through precipitation, LC-MS analyses of pre-reduced sfGFP S147C starting material were performed at three different concentrations to obtain the UV-vis calibration curve at 214 nm as described before[26]. The measurements were performed in triplicates and the dilution series was prepared based on A_{280} measurements on a Nanodrop system. Afterwards, the peak areas of proteins were plotted in dependence of the concentration. The obtained data points were submitted to linear regression analysis. The errors of the A_{214nm} measurements were calculated based on standard deviation of the triplicates. Errors for the measured sample (indicated in red) are estimated via a Gaussian error propagation for the slope, the intercept, and the A_{214nm} measurement. The error for slope and interception arises out of the linear regression while the error of the A_{214nm} measurement is based on the standard deviation of 5 measurements that result out of 3 biological triplicates and 3 technical replicates performed for one of the biological triplicates.

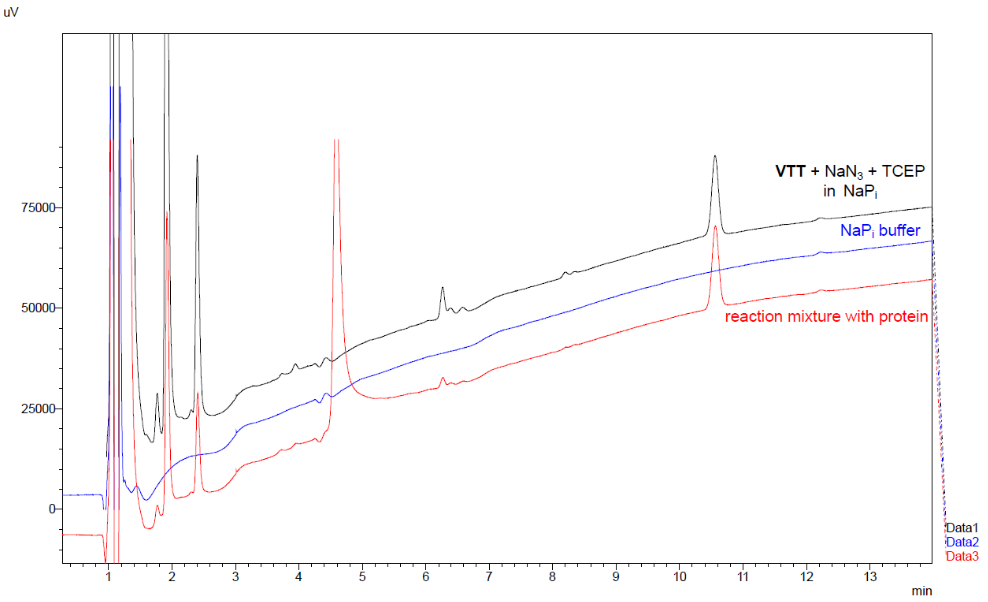

Figure S104. Chromatograms of a model reaction mixture with sfGFP S147C, NaP$_i$ buffer, and low-MW compounds present in the reaction mixture.

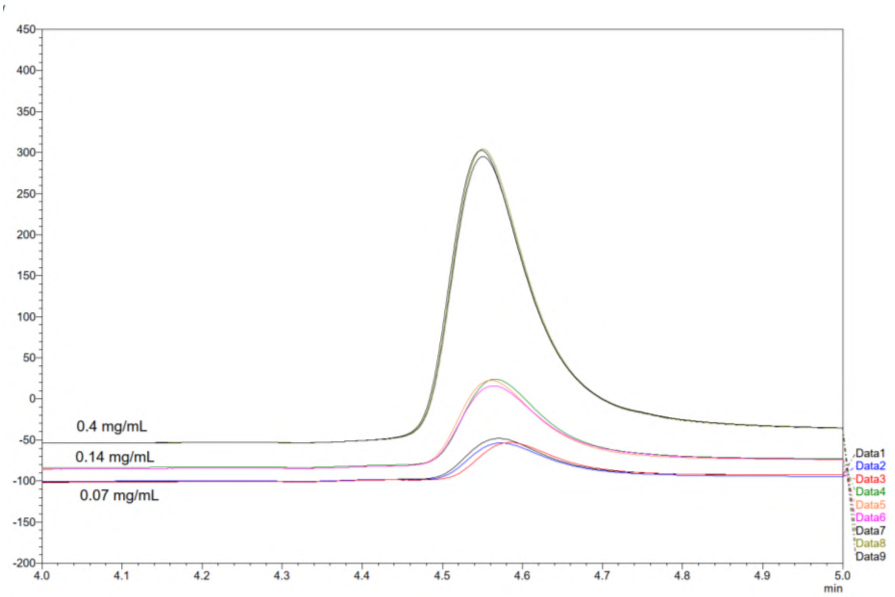

Figure S105. UV-vis chromatograms of solutions containing different concentrations of sfGFP measured as three technical replicates.

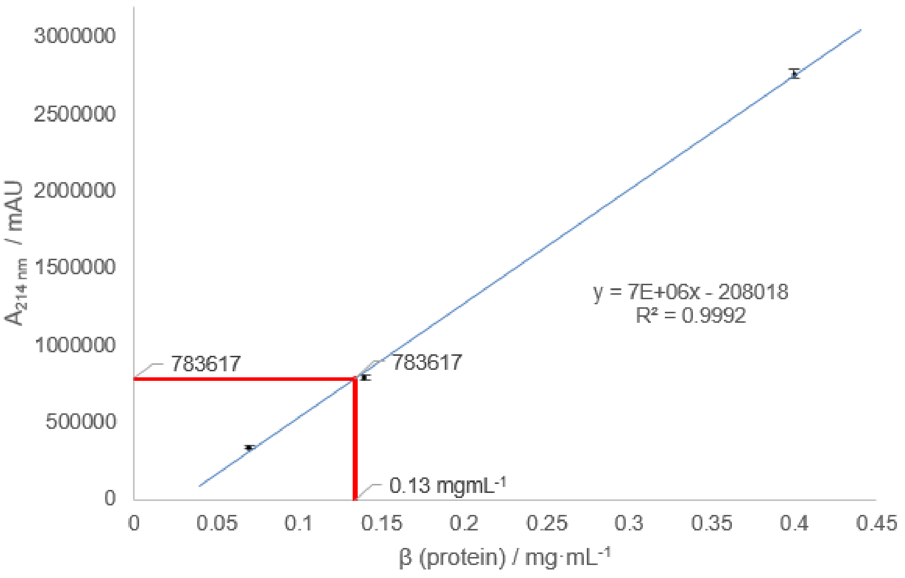

Figure S106. Calibration curve obtained for different concentrations of sfGFP S147C and calculated protein concentration in a model reaction mixture. The absorption values of unmodified protein utilized to calculate the calibration curve are shown as means ± SD resulting from three technical replicates, respectively. The absorption value of the modified protein is shown as a mean resulting from five experiments.

The concentration of the protein was determined after the reaction and indicates only minimal protein loss during the reaction:

β_{measured}(sfGFP S147C) = 0.13 $\pm$ 0.01 mg·mL^{-1}

$\beta_{\text{theoretical}}$ (sfGFP S147C) = 0.14 mg·mL^{-1}

$$\frac{\beta_{\text{measured}} - \beta_{\text{theoretical}}}{\beta_{\text{theoretical}}} = -7 \pm 7\,\%$$

The determined loss is expected to be similar for all other proteins utilized in this publication.

Comparison of LC-MS measurements with and without UV

To evaluate the influence of UV irradiation in LC-MS measurements, pre-reduced DHAR2 C6S starting material was measured in two different ways on the same LC-MS system. The first measurement was carried out with irradiation at 200-600 nm, the second measurement was performed without UV irradiation.

As shown in Figure S107 and Figure S108, irradiation during the LC-MS analysis has an influence on the total ion series of both peaks. Therefore, the influence of UV irradiation during LC-MS measurement was evaluated for each of the discussed proteins individually. If a negative influence was observed, all measurements were performed without UV irradiation and no UV trace is provided. This is the case for BSA, MDAR2, MDAR3, and DHAR2 C6S.

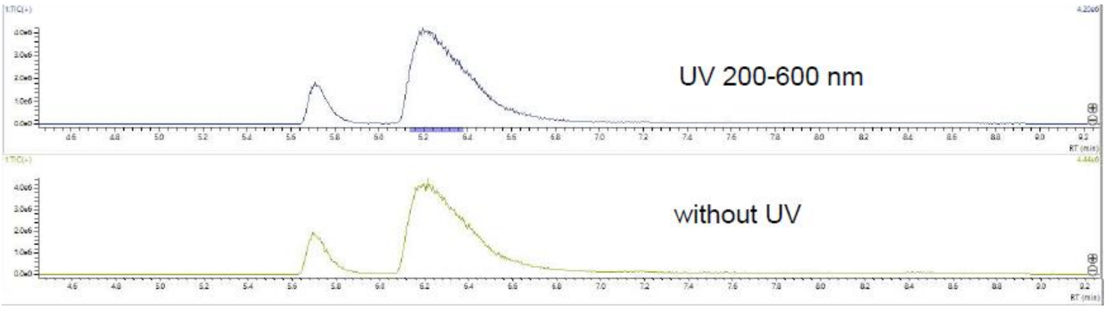

Figure S107. Total ion chromatograms of pre-reduced DHAR2 C6S starting material. The same sample was measured in both cases, the first TIC was measured after irradiation from 200-600 nm, the second TIC was obtained without irradiation.

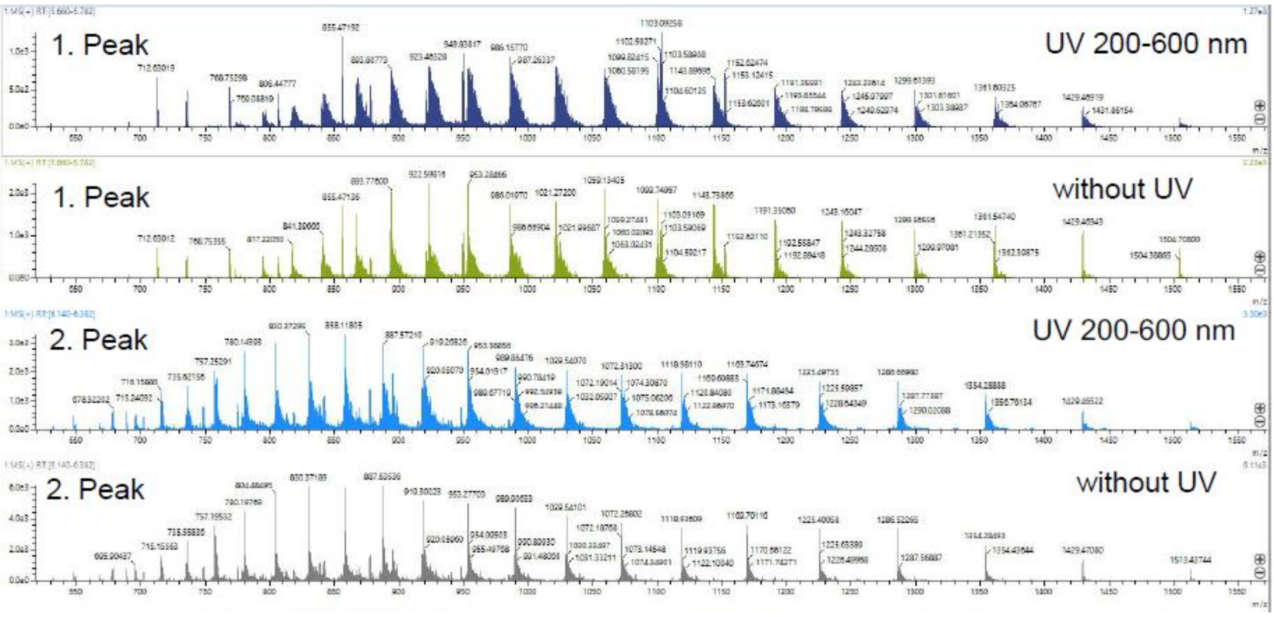

Figure S108. Total ion series of both peaks in the TIC chromatograms, each with and without irradiation.

Influence of c(Nu) on the formation of the hydrolysis product

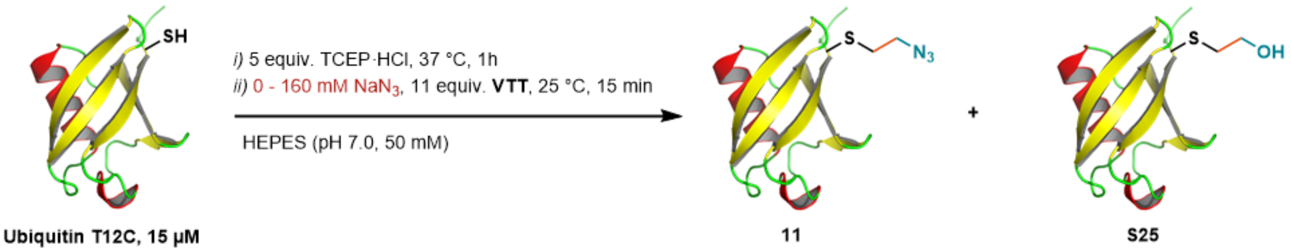

At 20–25 °C, 13 µL of Ubiquitin T12C (1.30 mg/mL, 144 µM, 1.5 nmol, 1.0 equiv.) in HEPES buffer (pH 7.0, c = 50 mM) was added to a 1.5 mL Eppendorf tube. Next, HEPES buffer (pH 7.0, c = 50 mM) was added (see table below). Then, 0.8 µL of a TCEP stock solution (10 mM, 0.01 µmol, 3 µg, 5 equiv.) in UHQ-H_2O was added. The mixture was vortexed for 1 second, transferred into a Thermocycler pre-heated at 37 °C, and incubated at 37 °C for 1 hour at 400 rpm. Next, a 0.30 M sodium azide stock solution in HEPES buffer (pH 7.0, c = 50 mM) was added to the mixture at 20–25 °C (c_{Nu} = 0 – 160 mM, see table below), followed by addition of 0.7 µL of a **VTT** stock solution (25 mM, 0.02 µmol, 5 µg, 1 × 10^1 equiv.) in DMF. The reaction mixture was vortexed for 1 second, transferred into a Thermocycler pre-heated at 25 °C, and incubated at 25 °C at 400 rpm for 15 minutes. Subsequently, 0.7 µL of a β-mercaptoethanol stock solution (25 mM, 0.02 µmol, 1 × 10^1 equiv.) in UHQ-H_2O was added, and the mixture was incubated at 25 °C for 15 minutes. The obtained mixture was analyzed by LC-MS.

Experiment	V(HEPES), µL	$c_{final}(NaN_3)$, mmol	n equiv. NaN_3	$V_{stock}(NaN_3)$, µL	Peak area of the product
1	89	0	0	0	0
2	87	5	0.33 × 10^3	1.7	330029
3	86	10	0.67 × 10^3	3.3	418356
4	82	20	1.3 × 10^3	6.7	463796
5	76	40	2.7 × 10^3	13.0	506946
6	62	80	5.3 × 10^3	26.7	515628
7	36	160	11 × 10^3	53.3	511704

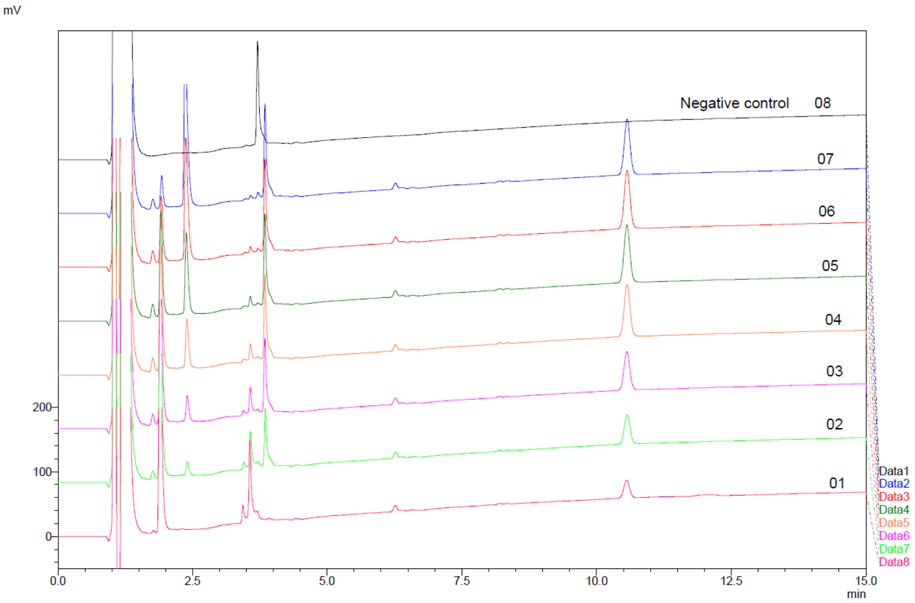

Figure S109. UV chromatograms of the reaction mixtures.

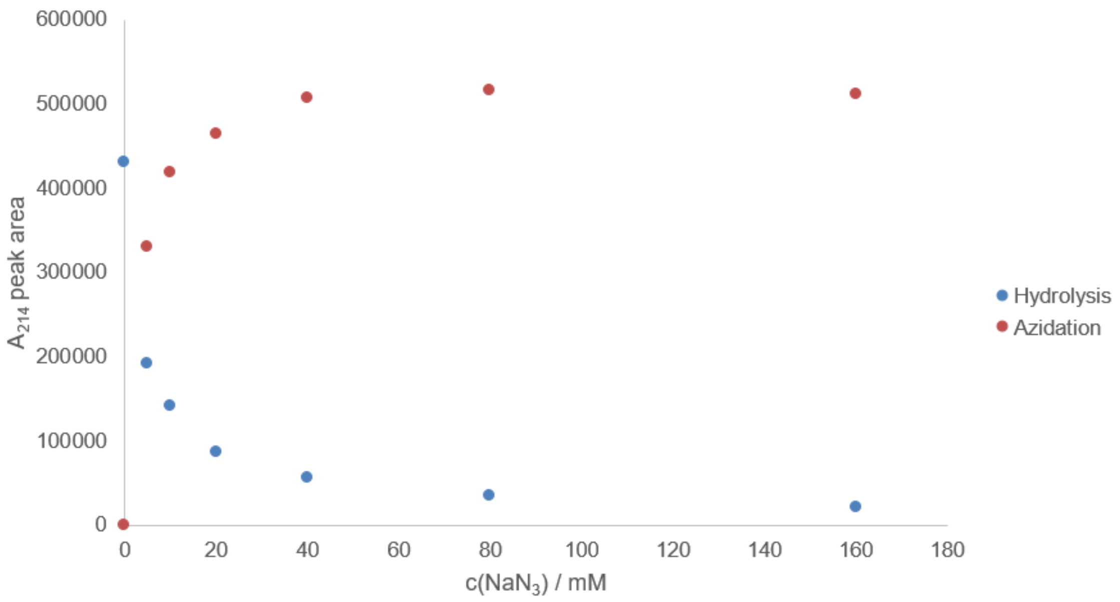

Figure S110. Dependence of the conversion to the azidation product (Ubiquitin T12C)–S–C_2H_4–N_3 and the hydrolysis product (Ubiquitin T12C)–S–C_2H_4–OH on the concentration of sodium azide present in the reaction mixture.

Influence of pH on the formation of the hydrolysis product

In order to assess the influence of pH on the amount of hydrolysis, we performed reactions with Ubiquitin T12C, **VTT**, and aniline. We intentionally chose a lower concentration of nucleophile than the one needed to achieve efficient labeling as described in the functional group scope, in order to better observe changes in the formation of the hydrolysis product.

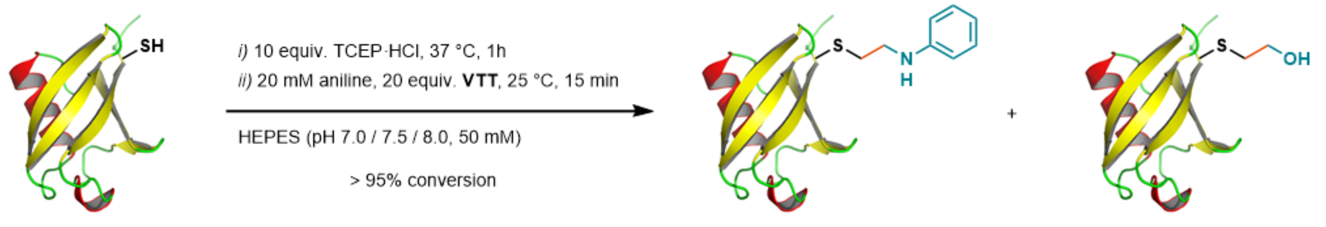

Ubiquitin T12C **S26** **S27**

At 20–25 °C, 13 µL of Ubiquitin T12C (1.06 mg/mL, 118 µM, 1.5 nmol, 1.0 equiv.) in HEPES buffer (pH 7.0 / 7.5 / 8.0, c = 50 mM) was added to a 1.5 mL Eppendorf tube. Next, 80 µL of HEPES buffer (pH 7.0 / 7.5 / 8.0, c = 50 mM) was added. Then, 0.5 µL of a TCEP stock solution (30 mM, 0.02 µmol, 4 µg, 1 × 10^1 equiv.) in UHQ-H_2O was added. The mixture was vortexed for 1 second, transferred into a Thermocycler pre-heated at 37 °C, and incubated at 37 °C for 1 hour at 400 rpm. Next, a 0.30 M aniline stock solution (7.0 µL, 2.0 µmol, 0.19 mg, 1.3 × 10^3 equiv.) in HEPES buffer (pH 7.0 / 7.5 / 8.0, c = 50 mM) was added to the mixture at 20–25 °C (c_{Nu} = 20 mM), followed by addition of 0.5 µL of a **VTT** stock solution (60 mM, 0.03 µmol, 1 × 10^1 µg, 2 × 10^1 equiv.) in DMF. The reaction mixture was vortexed for 1 second, transferred into a Thermocycler pre-heated at 25 °C, and incubated at 25 °C at 400 rpm for 15 minutes. Subsequently, 0.5 µL of a β-mercaptoethanol stock solution (60 mM, 0.03 µmol, 2 × 10^1 equiv.) in UHQ-H_2O was added, and the mixture was incubated at 25 °C for 15 minutes. The obtained mixture was analyzed by LC-MS.

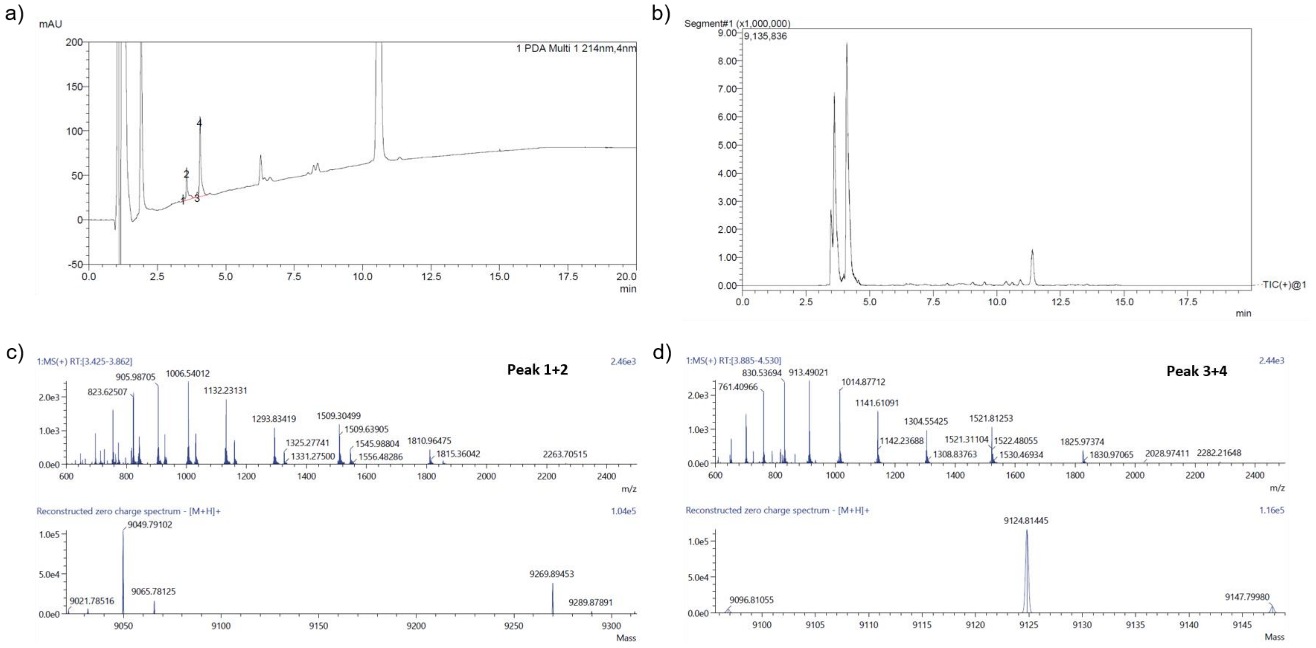

Figure S111. LC-MS analysis (Method B) of Ubiquitin T12C after reaction with **VTT**, and aniline at **pH 7.0**: a) UV-trace b) total ion chromatogram c) Peak 1+2 ion series and deconvoluted spectrum d) Peak 3+4 ion series and deconvoluted spectrum; calculated masses: (Ubiquitin T12C)–S–C_2H_4–N-aniline: 9125 Da, (Ubiquitin T12C)–S–C_2H_4–OH: 9050 Da, (Ubiquitin T12C)–S–C_2H_4–HEPES: 9270 Da; Observed masses: Peak 1+2: 9050 Da, 9270 Da; Peak 3+4: 9125 Da.

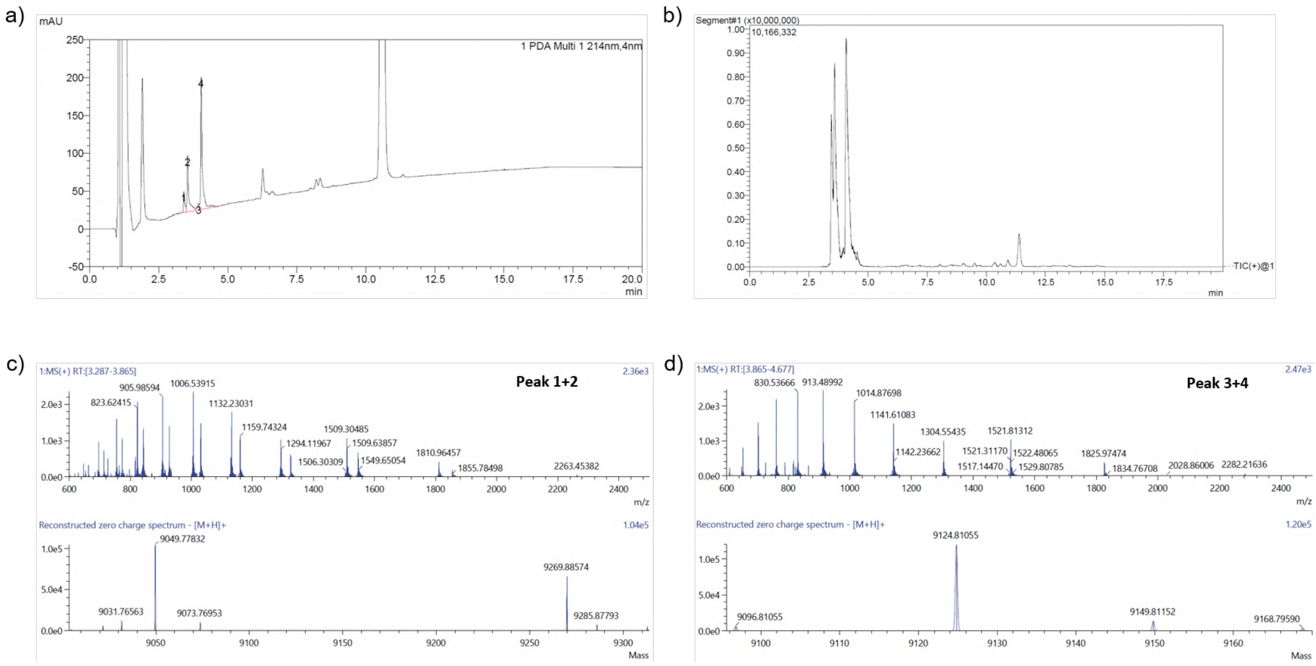

Figure S112. LC-MS analysis (Method B) of Ubiquitin T12C after reaction with **VTT**, and aniline at **pH 7.5**: a) UV-trace b) total ion chromatogram c) Peak 1+2 ion series and deconvoluted spectrum d) Peak 3+4 ion series and deconvoluted spectrum; calculated masses: (Ubiquitin T12C)–S–C_2H_4–N-aniline: 9125 Da, (Ubiquitin T12C)–S–C_2H_4–OH: 9050 Da, (Ubiquitin T12C)–S–C_2H_4–HEPES: 9270 Da; Observed masses: Peak 1+2: 9050 Da, 9270 Da; Peak 3+4: 9125 Da.

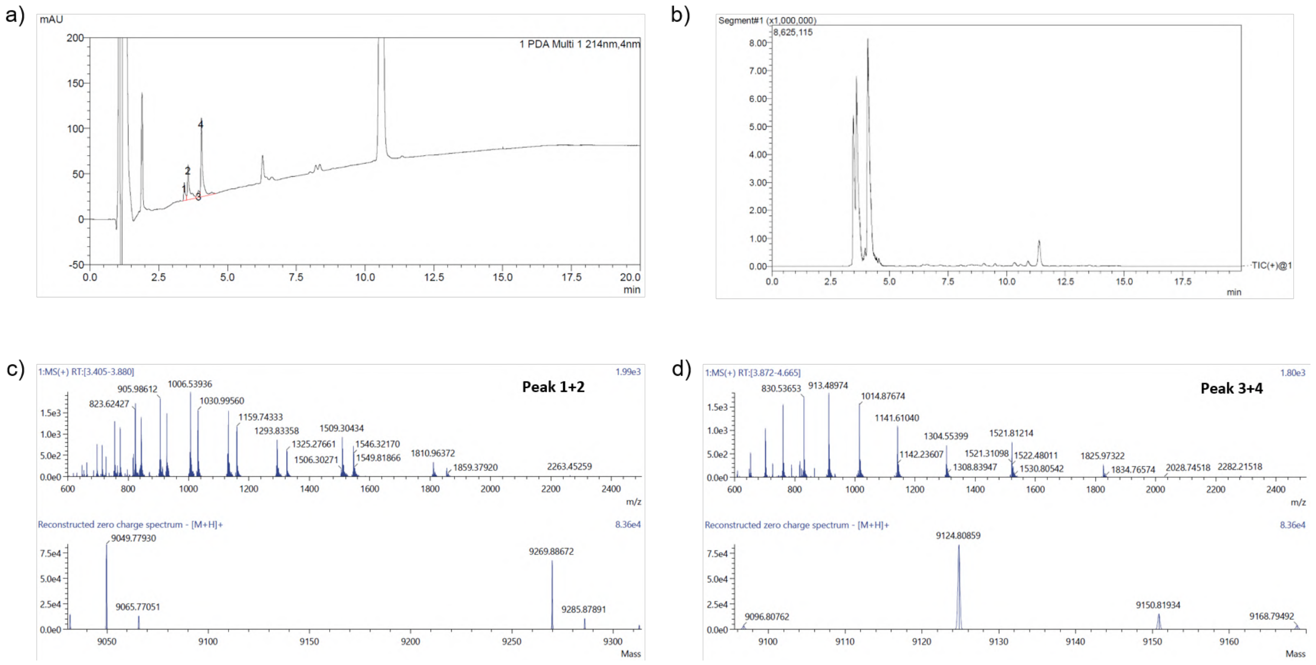

Figure S113. LC-MS analysis (Method B) of Ubiquitin T12C after reaction with **VTT**, and aniline at **pH 8.0** : a) UV-trace b) total ion chromatogram c) Peak 1+2 ion series and deconvoluted spectrum d) Peak 3+4 ion series and deconvoluted spectrum; calculated masses: (Ubiquitin T12C)–S–C_2H_4–N-aniline: 9125 Da, (Ubiquitin T12C)–S–C_2H_4–OH: 9050 Da, (Ubiquitin T12C)–S–C_2H_4–HEPES: 9270 Da; Observed masses: Peak 1+2: 9050 Da, 9270 Da; Peak 3+4: 9125 Da.

The following figure shows the PDA analysis at 214 nm of reactions carried out at pH 7.0, pH 7.5, and pH 8.0:

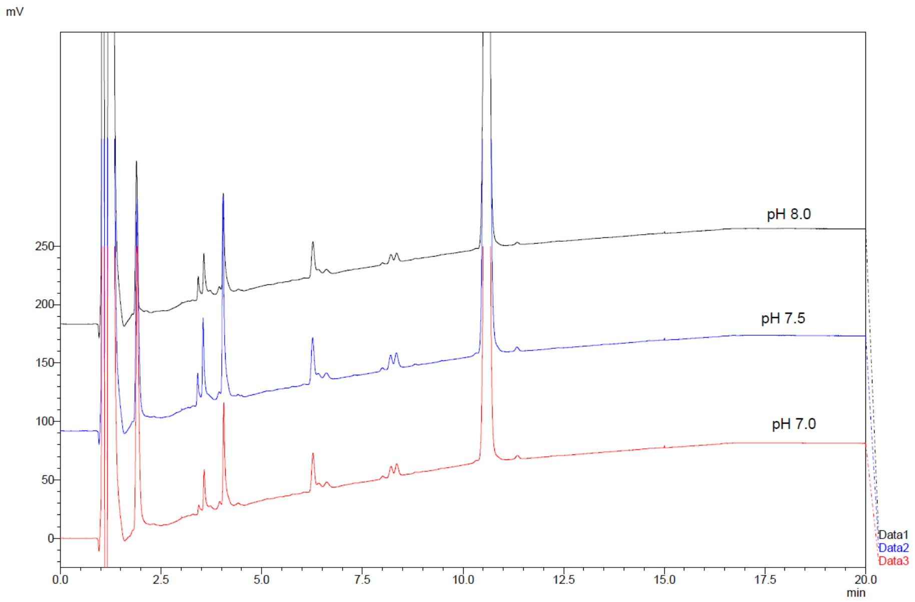

Figure S114. UV chromatograms of the reaction mixtures at pH 7.0, 7.5, and 8.0. The desired aniline product elutes at 4.0 min.

The amount of the hydrolysis product is similar in all the cases. The amount of HEPES adducts increase and lead to a decrease of product yield at higher pH values. The following table summarizes the outcome of the experiments:

pH value of the buffer	Hydrolysis / %	HEPES adduct / %	Product / %
7.0	29	5	66
7.5	28	8	64
8.0	30	10	60

Reaction with and without pre-reduction with TCEP

Following reactions were carried out to determine the influence of TCEP, used in all described reactions to ensure reduction of Cys residues in higher oxidation states. The reaction between Ubiquitin T9C, **VTT**, and sodium azide was carried out once with TCEP-reduction for 1 h at 37 °C prior to the azidation-reaction, and once without TCEP-reduction.

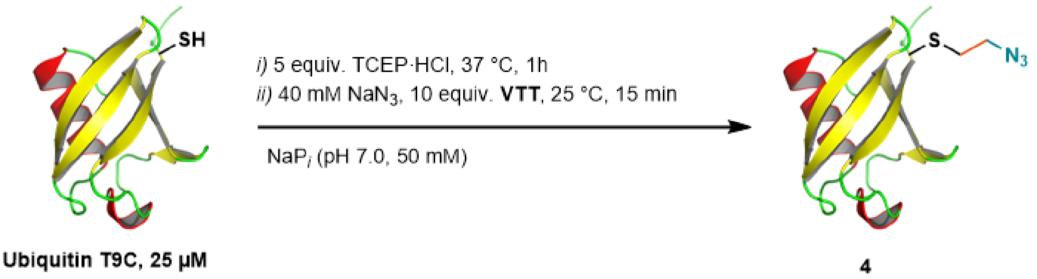

At 20–25 °C, 12 µL of Ubiquitin T9C (1.95 mg/mL, 217 µM, 2.5 nmol, 1.0 equiv.) in NaP_i buffer (pH 7.0,

c = 50 mM) was dded to a 1.5 mL Eppendorf tube. Next, 74 µL of NaP$_i$ buffer (pH 7.0, c = 50 mM) was added. Then, 1.3 µL of a TCEP stock solution (10 mM, 13 nmol, 3.1 mg, 5 equiv.) in UHQ-H_2O was added. The mixture was vortexed for 1 second, transferred into a Thermocycler pre-heated at 37 °C, and incubated at 37 °C for 1 hour at 400 rpm. Next, a 0.30 M sodium azide stock solution (13 µL, 4.0 µmol, 0.26 mg, 1.6 × 10^3 equiv.) in NaP$_i$ buffer (pH 7.0, c = 50 mM) was added to the mixture at 20–25 °C (c_{Nu} = 40 mM), followed by addition of 1.0 µL of a **VTT** stock solution (25 mM, 0.03 µmol, 1 × 10^1 µg, 1 × 10^1 equiv.) in DMF. The reaction mixture was vortexed for 1 second, transferred into a Thermocycler pre-heated at 25 °C, and incubated at 25 °C at 400 rpm for 15 minutes. Subsequently, 1.0 µL of a β-mercaptoethanol stock solution (25 mM, 0.03 µmol, 1 × 10^1 equiv.) in UHQ-H_2O was added, and the mixture was incubated at 25 °C for 15 minutes. The obtained mixture was analyzed by LC-MS.

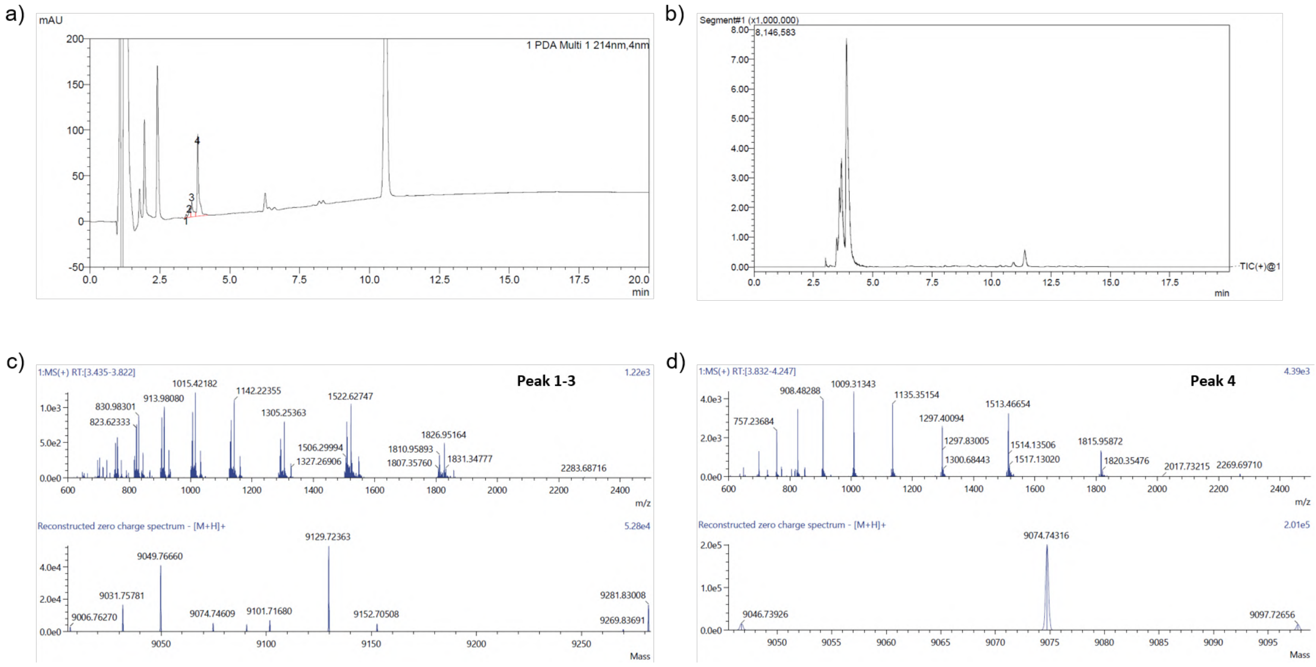

Figure S115. LC-MS analysis (Method B) of Ubiquitin T9C after reduction with TCEP and reaction with **VTT**, and sodium azide: a) UV-trace b) total ion chromatogram c) Peak 1-3 ion series and deconvoluted spectrum d) Peak 4 ion series and deconvoluted spectrum; calculated masses: (Ubiquitin T9C)–S–C_2H_4–N_3: 9075 Da, 9047 Da (peaks detected in the SM + 69 Da), (Ubiquitin T9C)–S–C_2H_4–OH: 9050 Da, (Ubiquitin T9C)–S–C_2H_4–OPO_3H_2: 9130 Da, (Ubiquitin T9C)–S–C_2H_4–TCEP: 9282 Da, stapled-(Ubiquitin T9C): 9032 Da; Observed masses: Peak 1-3: 9032 Da, 9050 Da, 9075 Da, 9130 Da, 9282 Da; Peak 4: 9047 Da, 9075 Da.

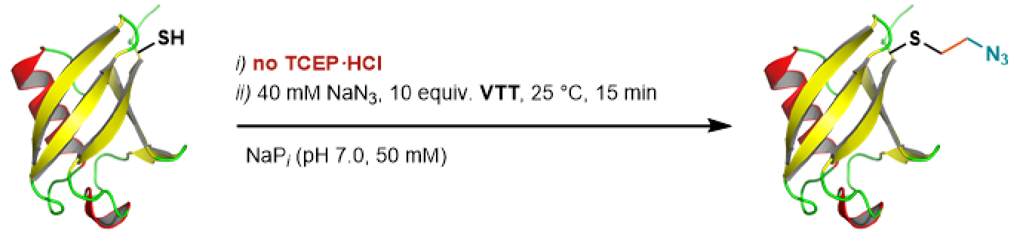

Ubiquitin T9C, 25 µM **4**

At 20–25 °C, 12 µL of Ubiquitin T9C (1.95 mg/mL, 217 µM, 2.5 nmol, 1.0 equiv.) in NaP$_i$ buffer (pH 7.0, c = 50 mM) was added to a 1.5 mL Eppendorf tube. Next, 74 µL of NaP$_i$ buffer (pH 7.0, c = 50 mM) was added. Then, 1.3 µL of UHQ-H_2O was added. The mixture was vortexed for 1 second, transferred into a Thermocycler pre-heated at 37 °C, and incubated at 37 °C for 1 hour at 400 rpm. Next, a 0.30 M sodium azide stock solution

(13 µL, 4.0 µmol, 0.26 mg, 1.6 × 10^3 equiv.) in NaP$_i$ buffer (pH 7.0, c = 50 mM) was added to the mixture at 20–25 °C (c_{Nu} = 40 mM), followed by addition of 1.0 µL of a **VTT** stock solution (25 mM, 0.03 µmol, 1 × 10^1 µg, 1 × 10^1 equiv.) in DMF. The reaction mixture was vortexed for 1 second, transferred into a Thermocycler pre-heated at 25 °C, and incubated at 25 °C at 400 rpm for 15 minutes. Subsequently, 1.0 µL of a β-mercaptoethanol stock solution (25 mM, 0.03 µmol, 1 × 10^1 equiv.) in UHQ-H_2O was added, and the mixture was incubated at 25 °C for 15 minutes. The obtained mixture was analyzed by LC-MS.

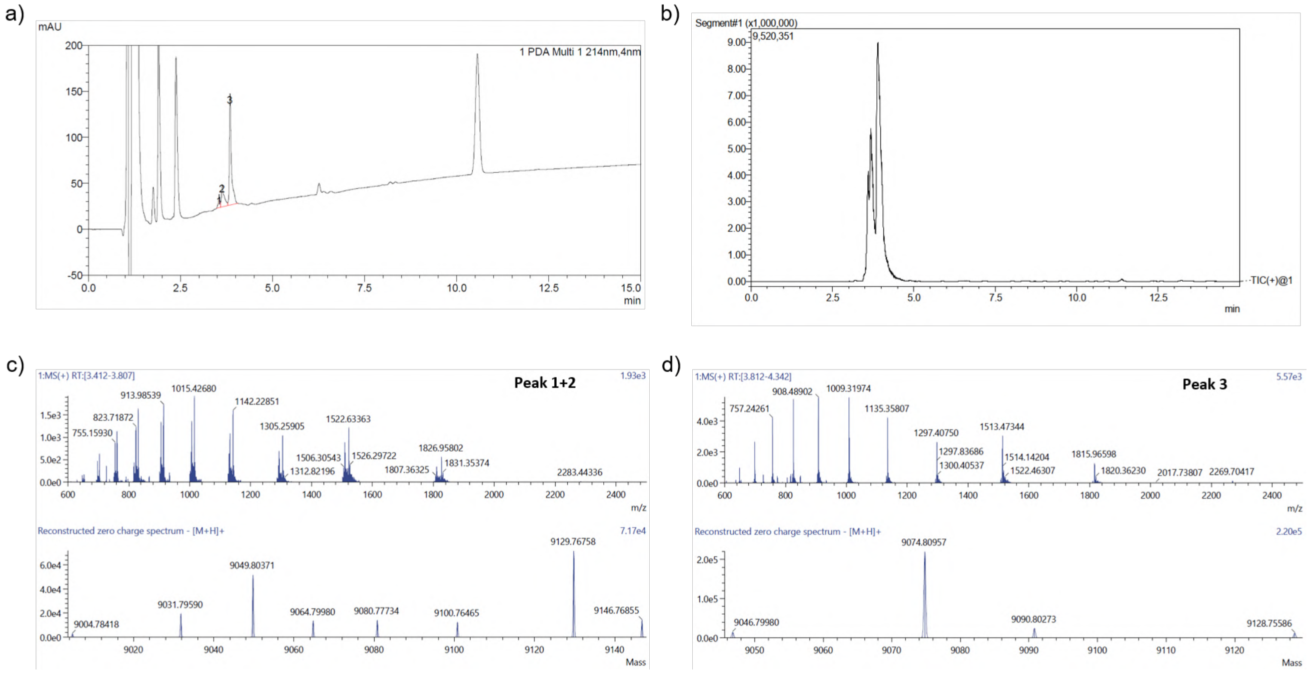

Figure S116. LC-MS analysis (Method B) of Ubiquitin T9C after reaction with **VTT**, and sodium azide **without** pre-reduction with TCEP: a) UV-trace b) total ion chromatogram c) Peak 1+2 ion series and deconvoluted spectrum d) Peak 3 ion series and deconvoluted spectrum; calculated masses: (Ubiquitin T9C)–S–C_2H_4–N_3: 9075 Da, 9047 Da (peaks detected in the SM + 69 Da), (Ubiquitin T9C)–S–C_2H_4–OH: 9050 Da, (Ubiquitin T9C)–S–C_2H_4–OPO_3H_2: 9130 Da, stapled-(Ubiquitin T9C): 9032 Da, (Ubiquitin T9C)–S–C_2H_4–TCEP: 9282 Da; Observed masses: Peak 1-3: 9032 Da, 9050 Da, 9075 Da, 9130 Da; Peak 4: 9047 Da, 9075 Da.

Experiments without a pre-reduction showed no negative or positive influence on the desired reactivity in the chosen example. However, we decided to use TCEP as a mild reducing agent for all bioconjugation reactions in order to ensure a reduced state of the thiols in all the cases.

Experiment with IAA-precapping

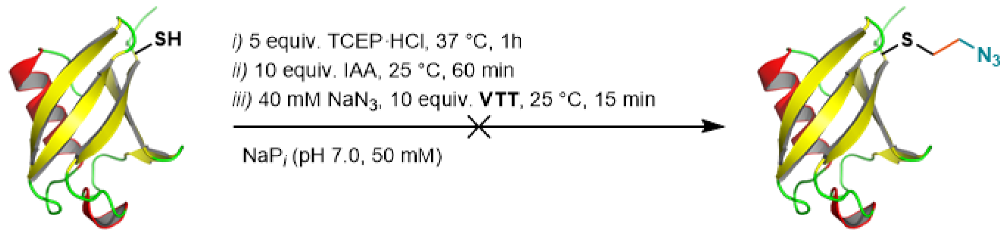

Ubiquitin T9C, 25 µM **4**

At 20–25 °C, 12 µL of Ubiquitin T9C (1.95 mg/mL, 217 µM, 2.5 nmol, 1.0 equiv.) in NaP$_i$ buffer (pH 7.0,

c = 50 mM) was added to a 1.5 mL Eppendorf tube. Next, 74 µL of NaP$_i$ buffer (pH 7.0, c = 50 mM) was added. Then, 1.3 µL of a TCEP stock solution (10 mM, 13 nmol, 3.1 mg, 5 equiv.) in UHQ-H_2O was added. The mixture was vortexed for 1 second, transferred into a Thermocycler pre-heated at 37 °C, and incubated at 37 °C for 1 hour at 400 rpm. Next, 1.0 µL of a **IAA** stock solution (25 mM, 0.03 µmol, 5 µg, 1 × 10^1 equiv.) in DMF. The reaction mixture was vortexed for 1 second, transferred into a Thermocycler pre-heated at 25 °C, and incubated at 25 °C at 400 rpm for 60 minutes. Next, a 0.30 M sodium azide stock solution (13 µL, 4.0 µmol, 0.26 mg, 1.6 × 10^3 equiv.) in NaP$_i$ buffer (pH 7.0, c = 50 mM) was added to the mixture at 20–25 °C (c_{Nu} = 40 mM), followed by addition of 1.0 µL of a **VTT** stock solution (25 mM, 0.03 µmol, 1 × 10^1 µg, 1 × 10^1 equiv.) in DMF. The reaction mixture was vortexed for 1 second, transferred into a Thermocycler pre-heated at 25 °C, and incubated at 25 °C at 400 rpm for 15 minutes. Subsequently, 1.0 µL of a β-mercaptoethanol stock solution (25 mM, 0.03 µmol, 1 × 10^1 equiv.) in UHQ-H_2O was added, and the mixture was incubated at 25 °C for 15 minutes. The obtained mixture was analyzed by LC-MS.

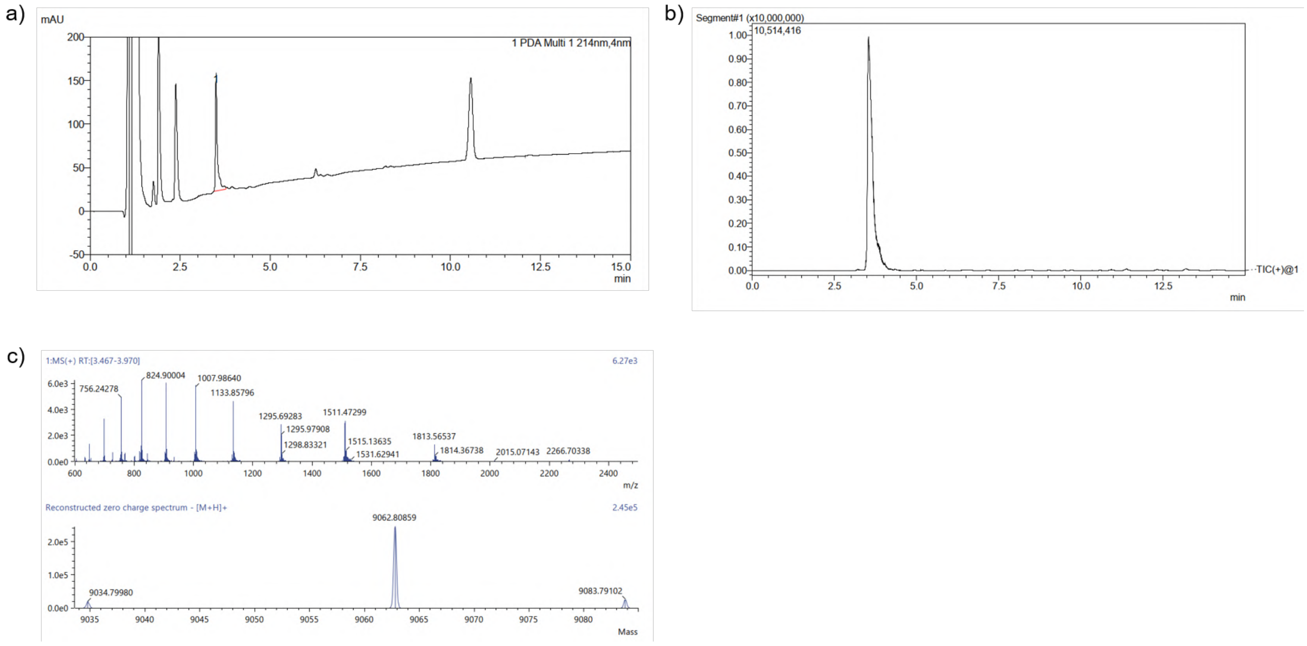

Figure S117. LC-MS analysis (Method B) of Ubiquitin T9C after reaction with **VTT**, and sodium azide with previously precapped Ubiquitin T9C using IAA: a) UV-trace b) total ion chromatogram c) Peak 1+2 ion series and deconvoluted spectrum d) Peak 3 ion series and deconvoluted spectrum; calculated mass: (Ubiquitin T9C)–S–CH_2–$CONH_2$: 9063 Da; Observed mass: 9063 Da.

In order to further evaluate the Cys-selectivity of vinyl-thianthrenium reagents, a control experiment was performed in which the Cys residue of Ubiquitin T9C was capped with IAA prior to the reaction with **VTT** and sodium azide. LC-MS analysis after the reaction sequence shows a single peak corresponding to the IAA-conjugation product. No reactivity with **VTT** was observed, further showcasing the Cys-selectivity of the reagent.

Evaluation of the reaction time of VTFT with sfGFP

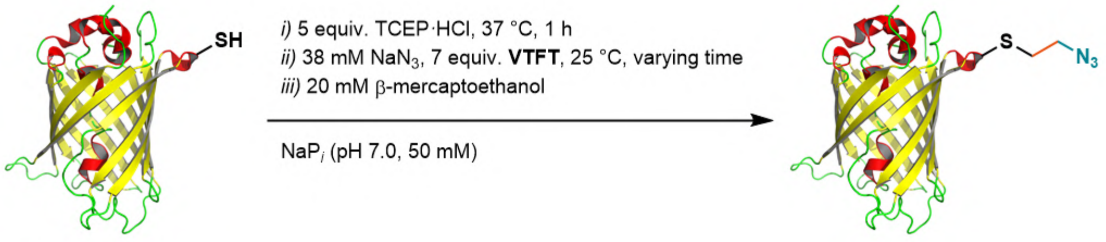

sfGFP S147C **2**

At 20–25 °C, 33 µL of sfGFP S147C (2.20 mg/mL, 75.0 µM, 2.5 nmol, 1.0 equiv.) in NaP_i buffer (pH 7.0, c = 50 mM) was added to a 1.5 mL Eppendorf tube. Next, 29 µL of NaP_i buffer (pH 7.0, c = 50 mM) was added. Then, 0.6 µL of a TCEP stock solution (20 mM, 0.01 µmol, 0.003 mg, 5 equiv.) in UHQ-H_2O was added. The mixture was vortexed for 1 s, transferred into a Thermocycler pre-heated at 37 °C, and incubated at 37 °C for 1 hour at 400 rpm. Next, a 0.10 M sodium azide solution (38 µL, 3.8 µmol, 0.25 mg, 1.5 × 10^3 equiv.) in NaP_i buffer (pH 7.0, c = 50 mM) was added to the mixture at 20–25 °C (c_{Nu} = 38 mM), followed by addition of 0.9 µL of a **VTFT** stock solution (20 mM, 0.02 µmol, 8 µg, 7 equiv.) in DMF. The reaction mixture was vortexed for 1 s, transferred into a Thermocycler pre-heated at 25 °C, and incubated at 25 °C at 400 rpm for 30 minutes. After different times (see Figure S118), 25 µL of a β-mercaptoethanol stock solution (0.10 M, 2.5 µmol, 0.20 mg, 1.0 × 10^3 equiv.) in UHQ-H_2O was added, and the mixture was incubated at 25 °C for 15 minutes. The obtained mixture was analyzed by LC-MS.

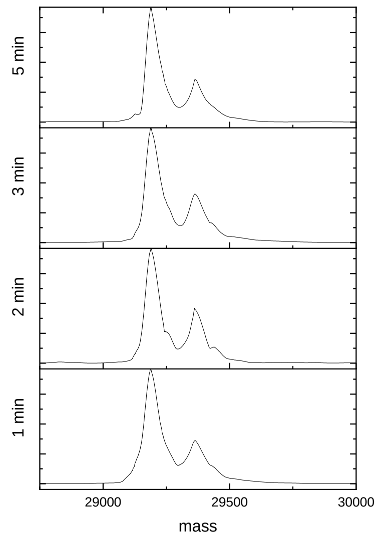

Figure S118. LC-MS analysis of sfGFP S147C after reaction with **VTFT** and NaN_3 quenched after different times with β-mercaptoethanol. Major peaks are plotted for varying reaction times.

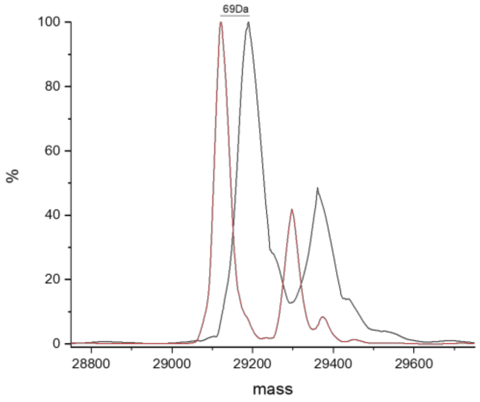

Figure S119. LC-MS analysis of sfGFP S147C after reaction with **VTFT** and NaN_3 quenched after 2 minutes with β-mercaptoethanol. The obtained mass spectrum after deconvolution (black) is plotted against the deconvoluted spectrum of the starting material (red).

The outcome of the experiment is in accordance with the rate constant of glutathione modification with **VTFT** and demonstrates a fast reactivity for macromolecular motifs. After 2 min reaction time, no change in product formation was observed. Because microenvironments of Cys depend on the protein structure, all protein conjugation reactions in the scope were quenched after 5 min to ensure complete labeling of even sterically hindered Cys residues.

Evaluation of the reaction time of VTT with sfGFP

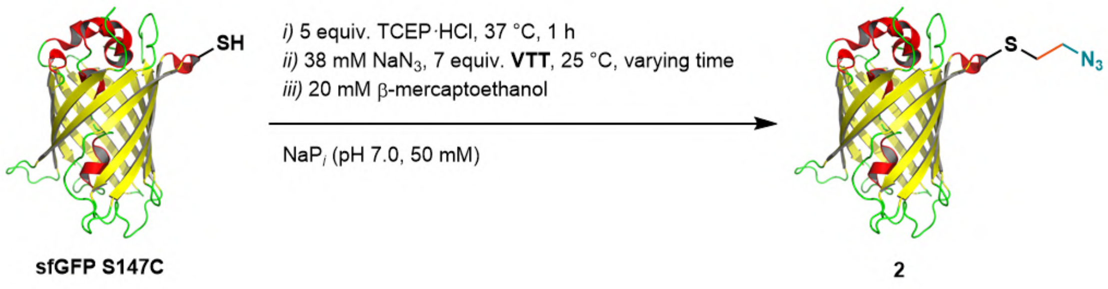

At 20–25 °C, 33 µL of sfGFP S147C (2.20 mg/mL, 75.0 µM, 2.5 nmol, 1.0 equiv.) in NaP_i buffer (pH 7.0, c = 50 mM) was added to a 1.5 mL Eppendorf tube. Next, 29 µL of NaP_i buffer (pH 7.0, c = 50 mM) was added. Then, 0.6 µL of a TCEP stock solution (20 mM, 0.01 µmol, 0.003 mg, 5 equiv.) in UHQ-H_2O was added. The mixture was vortexed for 1 s, transferred into a Thermocycler pre-heated at 37 °C, and incubated at 37 °C for 1 hour at 400 rpm. Next, a 0.10 M sodium azide solution (38 µL , 3.8 µmol, 0.25 mg, 1.5 × 10^3 equiv.) in NaP_i buffer (pH 7.0, c = 50 mM) was added to the mixture at 20–25 °C (c_{Nu} = 38 mM), followed by addition of 0.9 µL of a **VTT** stock solution (20 mM, 0.02 µmol, 7 µg, 7 equiv.) in DMF. The reaction mixture was vortexed for 1 s, transferred into a Thermocycler pre-heated at 25 °C, and incubated at 25 °C at 400 rpm for 30 minutes. After different times (see Figure S120), 25 µL of a β-mercaptoethanol stock solution (0.10 M, 2.5 µmol, 0.20 mg, 1.0 × 10^3 equiv.) in UHQ-H_2O was added, and the mixture was incubated at 25 °C for 15 minutes. The obtained mixture was analyzed by LC-MS.

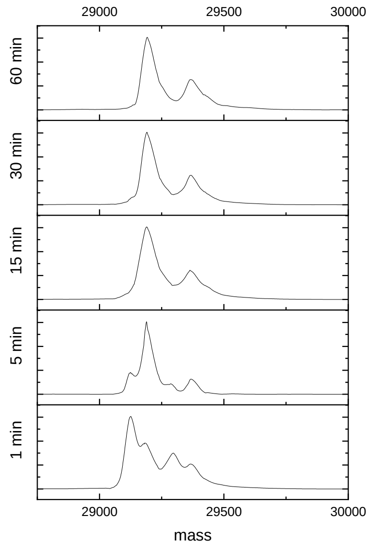

Figure S120. LC-MS analysis of sfGFP S147C after reaction with **VTT** and NaN_3 quenched after different times with β-mercaptoethanol. Major peaks are plotted for varying reaction times.

The outcome of the experiment is in accordance with the rate constant of glutathione modification with **VTT** and demonstrates a fast reactivity for macromolecular motifs. After 15 min reaction time, no change in product formation was observed.

Evaluation of the stability of conjugated proteins

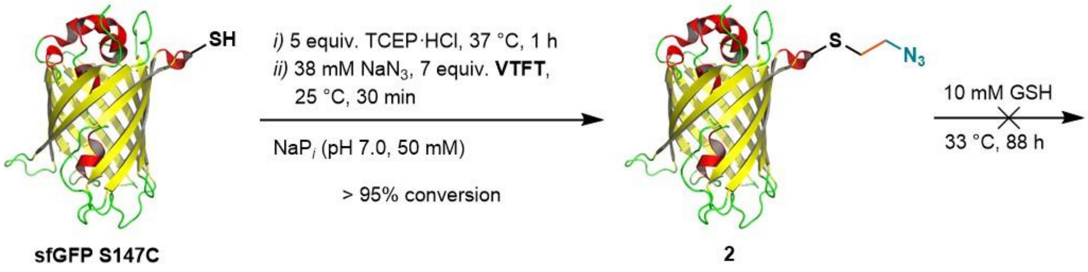

At 20–25 °C, 33 µL of sfGFP S147C (2.24 mg/mL, 76.5 µM, 2.5 nmol, 1.0 equiv.) in NaP_i buffer (pH 7.0, c = 50 mM) was added to a 1.5 mL Eppendorf tube. Next, 29 µL of NaP_i buffer (pH 7.0, c = 50 mM) was added. Then, 0.6 µL of a TCEP stock solution (20 mM, 0.01 µmol, 0.003 mg, 5 equiv.) in UHQ-H_2O was added. The mixture was vortexed for 1 s, transferred into a Thermocycler pre-heated at 37 °C, and incubated at 37 °C for 1 hour at 400 rpm. Next, a 0.10 M sodium azide solution (38 µL , 3.8 µmol, 0.25 mg, 1.5 × 10^3 equiv.) in NaP_i buffer (pH 7.0, c = 50 mM) was added to the mixture at 20–25 °C (c_{Nu} = 38 mM), followed by addition of 0.9 µL of a **VTFT** stock solution (20 mM, 0.02 µmol, 8 µg, 7 equiv.) in DMF. The reaction mixture was vortexed for 1 s, transferred into a Thermocycler pre-heated at 25 °C, and incubated at 25 °C at 400 rpm for 30 minutes. Afterwards, 40 µL of the reaction mixture was diluted into a 13 mM GSH solution in NaP_i buffer (pH 7.0, c = 50 mM). The obtained 10 mM protein-GSH mixture was transferred into an LC device incubated at 33 °C. After different reaction times (see Figure S120) samples were analyzed via LC-MS.

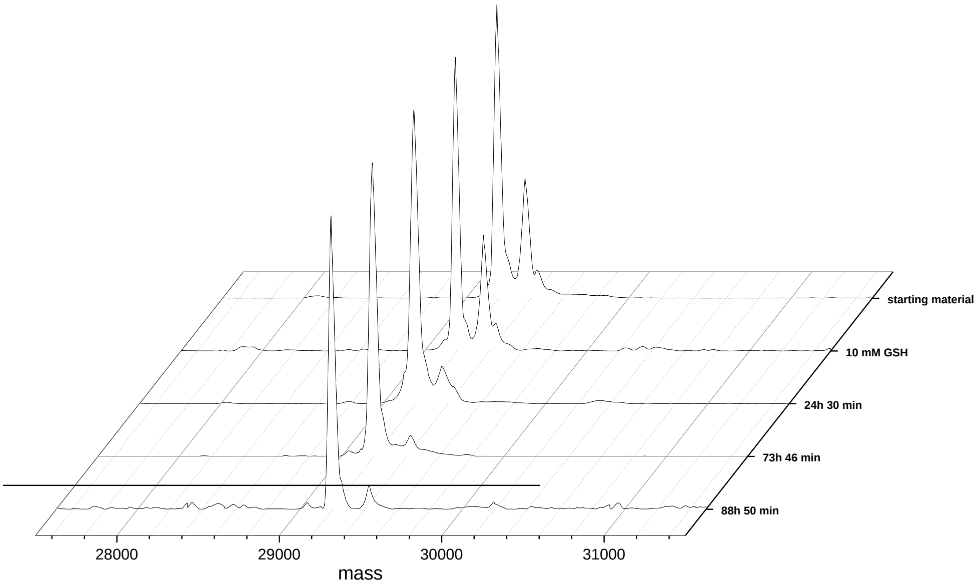

Figure S121. LC-MS analysis of sfGFP S147C after reaction with **VTFT** and NaN_3 with 10 mM GSH at 33 °C. The sample was injected after varying times.

Influence of the counter-anion of VTT

Synthesis of Ubiquitin T9C–S–C_2H_4–N_3 (4) with VTT OTf

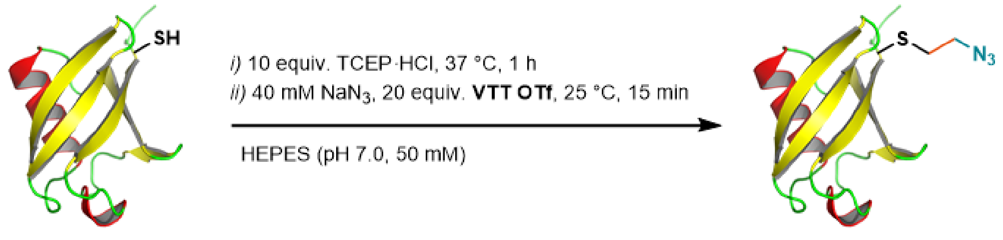

Ubiquitin T9C, 15 µM

4

At 20–25 °C, 11 µL of Ubiquitin T9C (1.18 mg/mL, 131 µM, 1.5 nmol, 1.0 equiv.) in HEPES buffer (pH 7.0, c = 50 mM) was added to a 1.5 mL Eppendorf tube. Next, 75 µL of HEPES buffer (pH 7.0, c = 50 mM) was added. Then, 0.5 µL of a TCEP stock solution (30 mM, 0.02 µmol, 4 µg, 1 × 10^1 equiv.) in UHQ-H_2O was added. The mixture was vortexed for 1 second, transferred into a Thermocycler pre-heated at 37 °C, and incubated at 37 °C for 1 hour at 400 rpm. Next, a 0.30 M sodium azide stock solution (13 µL, 4.0 µmol, 0.26 mg, 2.7 × 10^3 equiv.) in HEPES buffer (pH 7.0, c = 50 mM) was added to the mixture at 20–25 °C (c_{Nu} = 40 mM), followed by addition of 0.5 µL of a **VTT-OTf** stock solution (60 mM, 0.03 µmol, 1 × 10^1 µg, 2 × 10^1 equiv.) in DMF. The reaction mixture was vortexed for 1 second, transferred into a Thermocycler pre-heated at 25 °C, and incubated at 25 °C at 400 rpm for 15 minutes. Subsequently, 0.5 µL of a β-mercaptoethanol stock solution (60 mM, 0.03 µmol, 2 × 10^1 equiv.) in UHQ-H_2O was added, and the mixture was incubated at 25 °C for 15 minutes. The obtained mixture was analyzed by LC-MS.

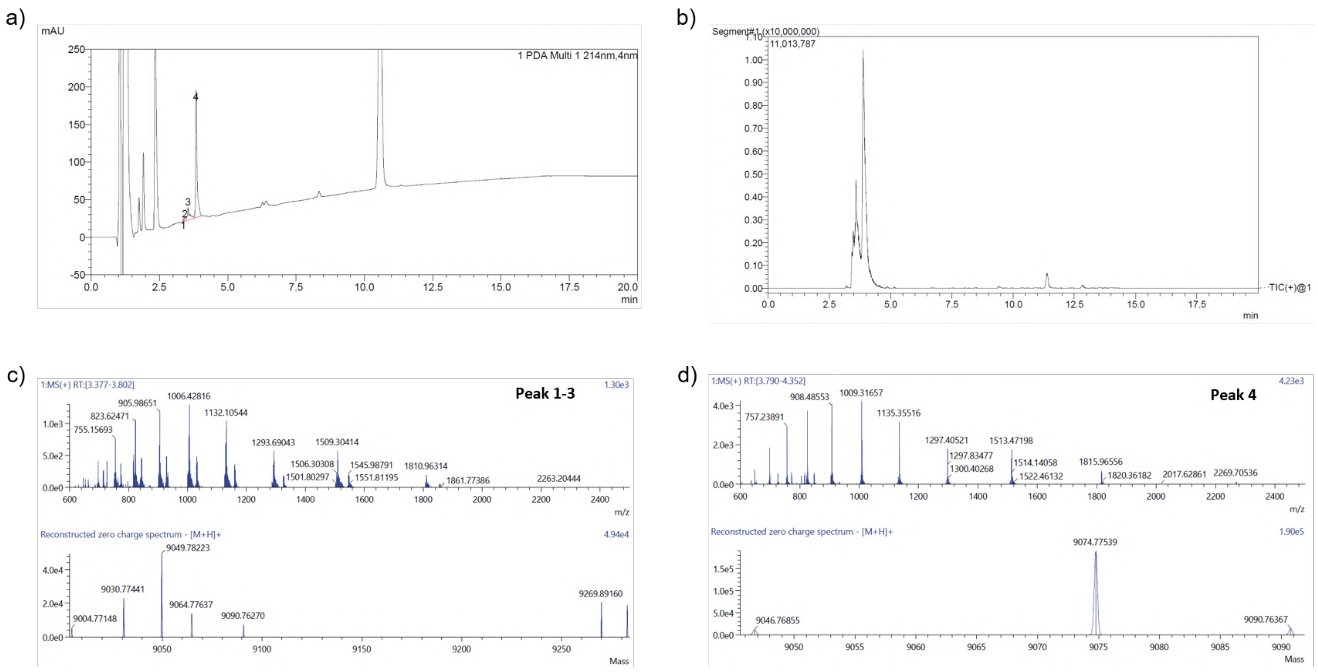

Figure S122. LC-MS analysis (Method B) of Ubiquitin T9C after reaction with **VTT OTf**, and sodium azide: a) UV-trace b) total ion chromatogram c) Peak 1-3 ion series and deconvoluted spectrum d) Peak 4 ion series and deconvoluted spectrum; Calculated masses: (Ubiquitin T9C)–S–C_2H_4–N_3: 9047 Da, 9075 Da, 9091 Da (detected peaks in SM + 69 Da), (Ubiquitin T9C)–S–C_2H_4–OH: 9050 Da, (Ubiquitin T9C)–S–C_2H_4–HEPES: 9270 Da, stapled-(Ubiquitin T9C): 9032 Da; Observed masses: Peak 1-3: 9050 Da, 9031 Da, 9270 Da; Peak 4: 9047 Da, 9075 Da, 9091 Da.

The experiment shows similar yields as observed for vinyl thianthrenium with BF_4 as the counterion (see Table S3). The 5% difference in yield is not considered a significant alteration due to the semi-quantitative nature of the analysis.

Investigation of the tertiary structure preservation after the reaction with VTT

To evaluate a possible negative influence of intramolecular cross linking of the episulfonium intermediates, we performed reactions in the absence of an exogenous nucleophile. We decided to use three different proteins for the evaluation which would allow for a facile activity assay afterwards. The three proteins (sfGFP S147C, DHAR1 S97C, and DHAR1 S176C) were therefore all treated with TCEP for 1 h at 37 °C and afterwards with **VTT** at pH 7.0. These conditions were chosen in order to mimic the standard reaction conditions but favor intramolecular reactivity by the absence of additional nucleophiles. In case of sfGFP S147C and DHAR1 S176C we were able to determine significant formation of the intramolecular episulfonium opening. Next, the modified proteins were submitted to activity assays.

Reaction of DHAR1 S97C and VTT without exogenous nucleophile

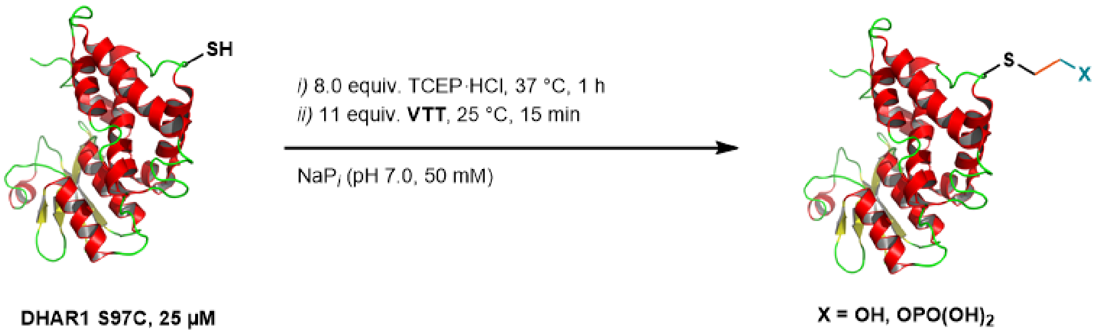

At 20–25 °C, 57 µL of DHAR1 S97C (1.14 mg/mL, 44 µM, 2.5 nmol, 1.0 equiv.) in NaP$_i$ buffer (pH 7.0, c = 50 mM) was added to a 1.5 mL Eppendorf tube. Next, 42 µL of NaP$_i$ buffer (pH 7.0, c = 50 mM) was added. Then, 1.0 µL of a TCEP stock solution (20 mM, 0.02 µmol, 6 µg, 8 equiv.) in UHQ-H_2O was added. The mixture was vortexed for 1 second, transferred into a Thermocycler pre-heated at 37 °C, and incubated at 37 °C for 1 hour at 400 rpm. Next, 0.7 µL of a **VTT** stock solution (25 mM, 0.03 µmol, 1×10^1 µg, 1×10^1 equiv.) in DMF. The reaction mixture was vortexed for 1 second, transferred into a Thermocycler pre-heated at 25 °C, and incubated at 25 °C at 400 rpm for 15 minutes. Subsequently, 0.7 µL of a β-mercaptoethanol stock solution (25 mM, 0.03 µmol, 1×10^1 µg, 1×10^1 equiv.) in UHQ-H_2O was added, and the mixture was incubated at 25 °C for 15 minutes. The obtained mixture was analyzed by LC-MS.

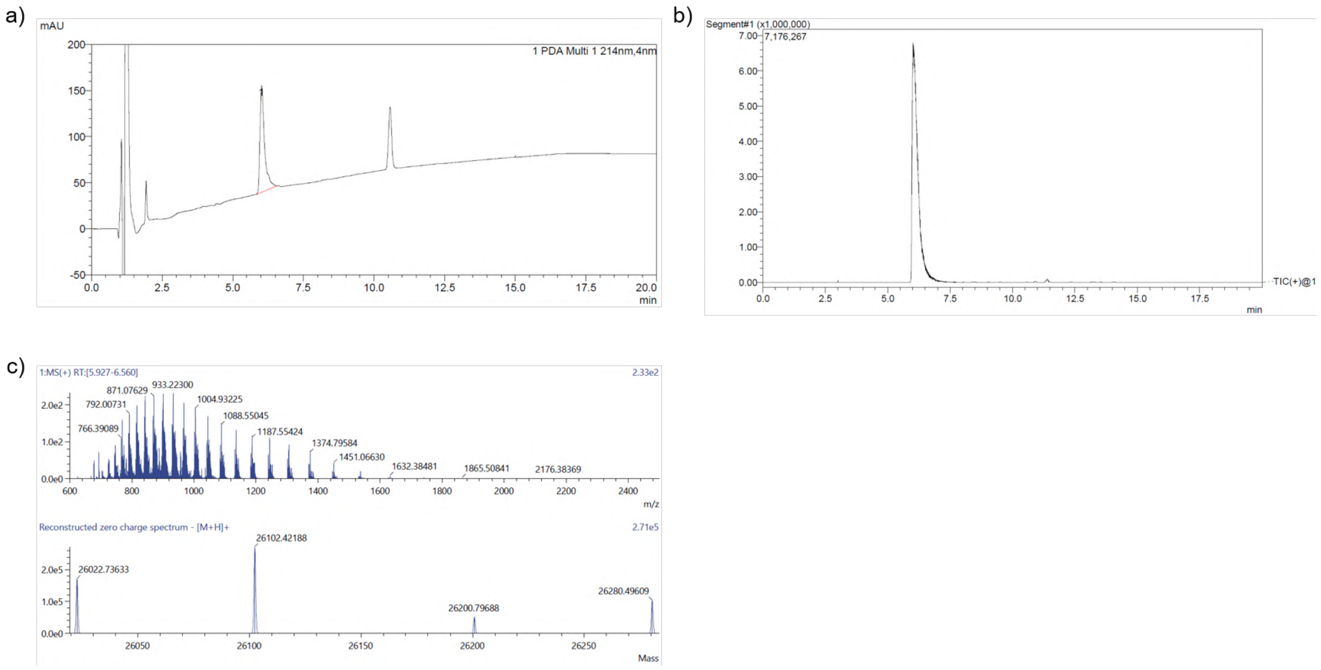

Figure S123. LC-MS analysis (Method B) of DHAR1 S97C after reaction with 11 equiv. **VTT**, without an exogenous nucleophile: a) UV-trace b) total ion chromatogram c) Ion series and deconvoluted spectrum; calculated masses: (DHAR1 S97C)–S–C_2H_4–OH: 26022 Da, 26200 Da (peaks detected in SM + 44 Da), (DHAR1 S97C)–S–C_2H_4–OPO_3H_2: 26102 Da, 26280 Da (peaks detected in SM + 124 Da); Observed masses: 26023 Da, 26102 Da, 26201 Da, 26280 Da.

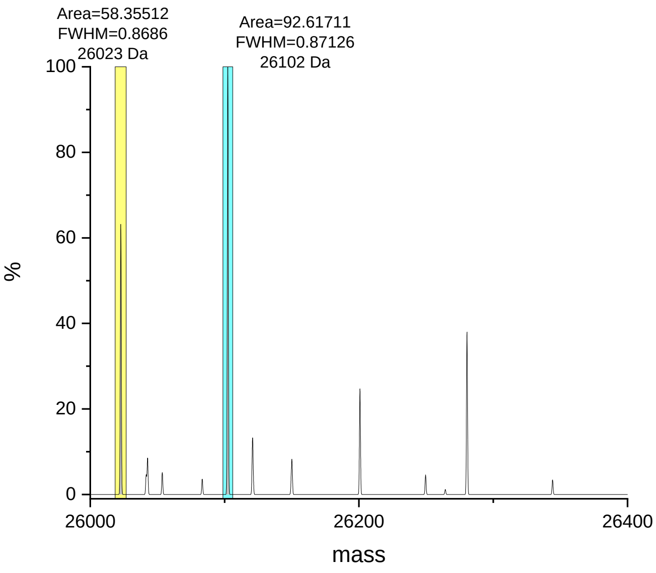

Figure S124. Peak areas of products after the reaction of DHAR1 S97C with 11 eq. **VTT** and in the absence of exogenous nucleophile. All observed side reactions were identified and integrated. To compare the results only side reactivity of the main peak in the starting material (25978 Da) was considered.

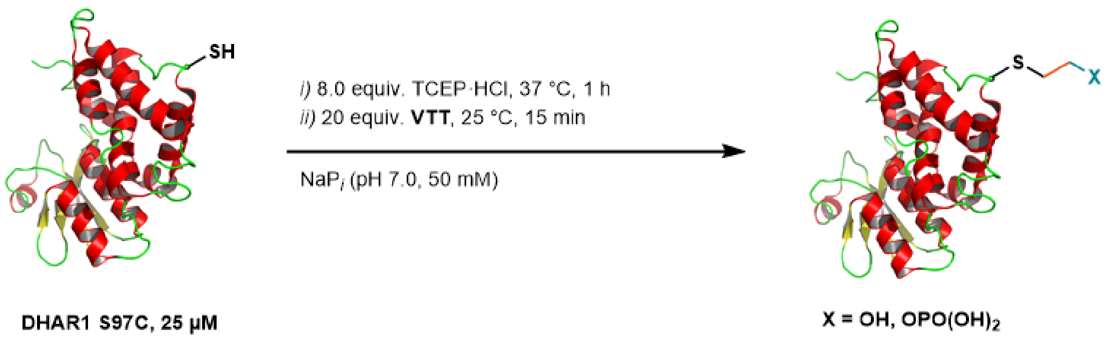

At 20–25 °C, 57 µL of DHAR1 S97C (1.14 mg/mL, 44 µM, 2.5 nmol, 1.0 equiv.) in NaP$_i$ buffer (pH 7.0, c = 50 mM) was added to a 1.5 mL Eppendorf tube. Next, 42 µL of NaP$_i$ buffer (pH 7.0, c = 50 mM) was added. Then, 1.0 µL of a TCEP stock solution (20 mM, 0.02 µmol, 6 µg, 8 equiv.) in UHQ-H_2O was added. The mixture was vortexed for 1 second, transferred into a Thermocycler pre-heated at 37 °C, and incubated at 37 °C for 1 hour at 400 rpm. Next, 1.3 µL of a **VTT** stock solution (25 mM, 50 nmol, 17 µg, 20 equiv.) in DMF. The reaction mixture was vortexed for 1 second, transferred into a Thermocycler pre-heated at 25 °C, and incubated at 25 °C at 400 rpm for 15 minutes. Subsequently, 1.3 µL of a β-mercaptoethanol stock solution (25 mM, 50 nmol, 4.7 µg, 20 equiv.) in UHQ-H_2O was added, and the mixture was incubated at 25 °C for 15 minutes. The obtained mixture was analyzed by LC-MS.

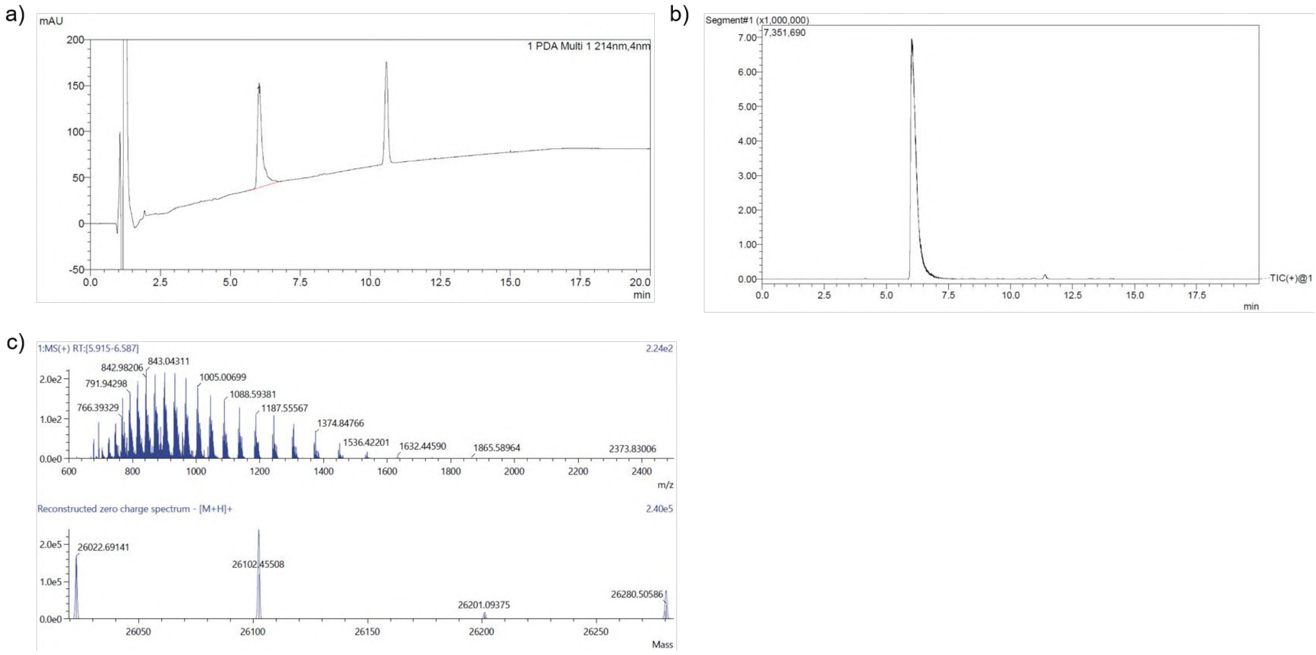

Figure S125. LC-MS analysis (Method B) of DHAR1 S97C after reaction with 20 equiv. **VTT**, without an exogenous nucleophile: a) UV-trace b) total ion chromatogram c) Ion series and deconvoluted spectrum; calculated masses: (DHAR1 S97C)–S–C_2H_4–OH: 26022 Da, 26200 Da (peaks detected in SM + 44 Da), (DHAR1 S97C)–S–C_2H_4–OPO_3H_2: 26102 Da, 26280 Da (peaks detected in SM + 124 Da); Observed masses: 26023 Da, 26102 Da, 26201 Da, 26281 Da.

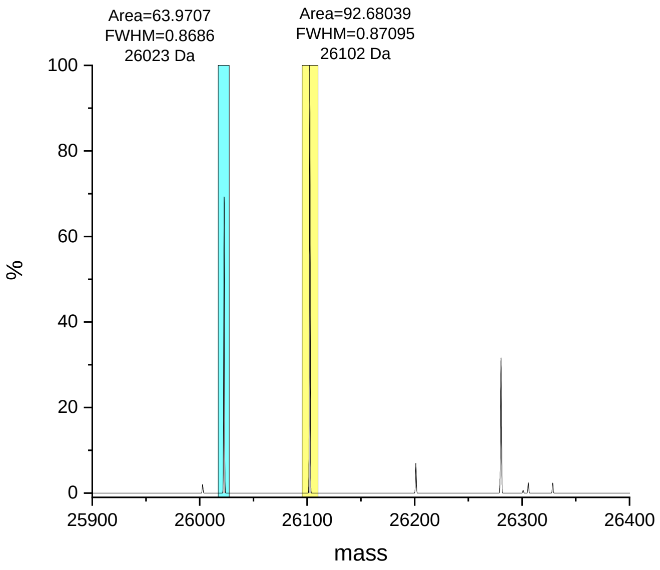

Figure S126. Peak areas of products after the reaction of DHAR1 S97C with 20 eq. **VTT** and in the absence of exogenous nucleophile. All observed side reactions were identified and integrated. To compare the results only side reactivity of the main peak in the starting material (25978 Da) was considered.

Reaction of DHAR1 S176C and VTT without exogenous nucleophile

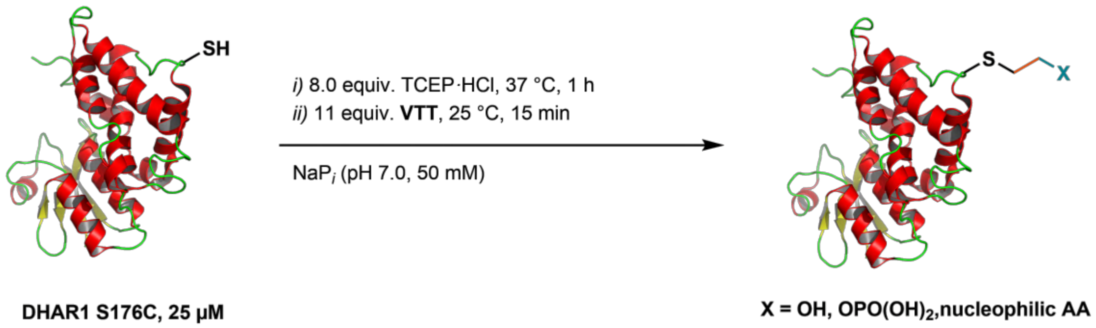

At 20–25 °C, 55 µL of DHAR1 S176C (1.18 mg/mL, 46 µM, 2.5 nmol, 1.0 equiv.) in NaP$_i$ buffer (pH 7.0, c = 50 mM) was added to a 1.5 mL Eppendorf tube. Next, 44 µL of NaP$_i$ buffer (pH 7.0, c = 50 mM) was added. Then, 1.0 µL of a TCEP stock solution (20 mM, 0.02 µmol, 6 µg, 8 equiv.) in UHQ-H_2O was added. The mixture was vortexed for 1 second, transferred into a Thermocycler pre-heated at 37 °C, and incubated at 37 °C for 1 hour at 400 rpm. Next, 0.7 µL of a **VTT** stock solution (25 mM, 0.03 µmol, 1 × 10^1 µg, 1 × 10^1 equiv.) in DMF. The reaction mixture was vortexed for 1 second, transferred into a Thermocycler pre-heated at 25 °C, and incubated at 25 °C at 400 rpm for 15 minutes. Subsequently, 0.7 µL of a β-mercaptoethanol stock solution (25 mM, 0.03 µmol, 1 × 10^1 µg, 1 × 10^1 equiv.) in UHQ-H_2O was added, and the mixture was incubated at 25 °C for 15 minutes. The obtained mixture was analyzed by LC-MS.

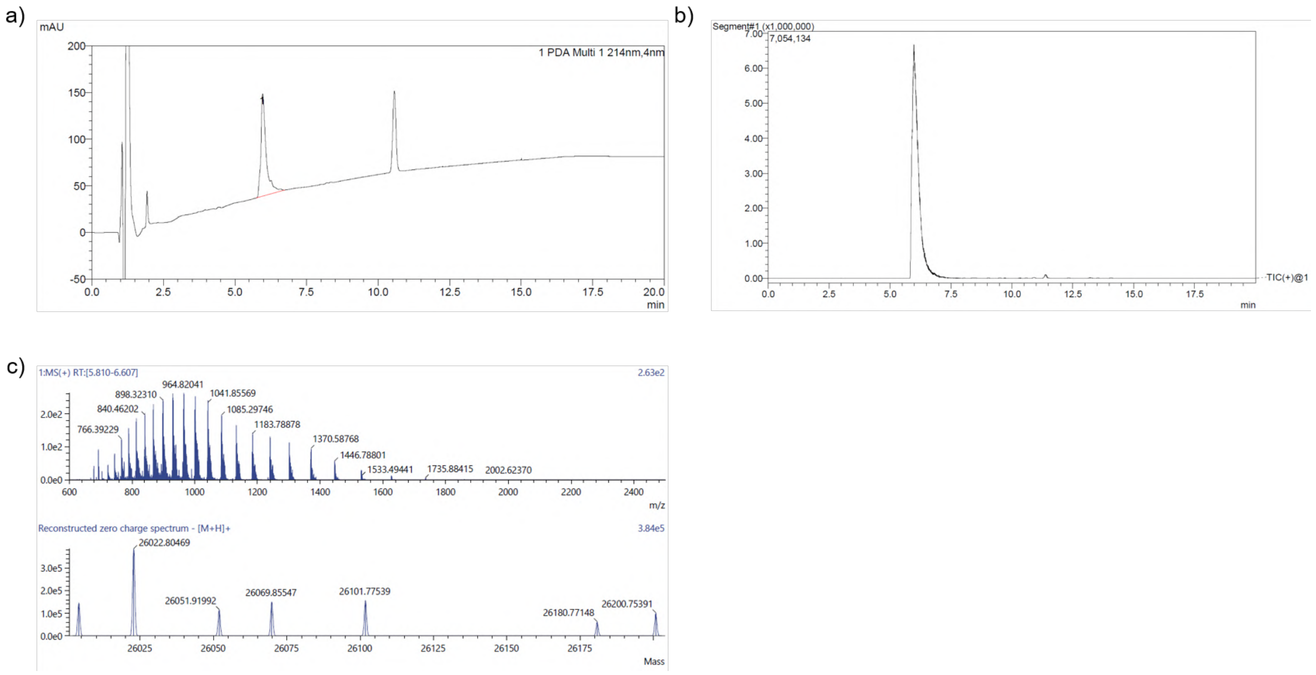

Figure S127. LC-MS analysis (Method B) of DHAR1 S176C after reaction with 11 equiv. **VTT**, without an exogenous nucleophile: a) UV-trace b) total ion chromatogram c) Ion series and deconvoluted spectrum; calculated masses: (DHAR1 S176C)–S–C_2H_4–OH: 26022 Da, 26200 Da, 26069 Da (peaks detected in SM + 44 Da), (DHAR1 S176C)–S–C_2H_4–OPO_3H_2: 26102 Da, stapled-(DHAR1 S176C): 26005 Da, 26051 Da, 26182 Da (peaks detected in SM + 26 Da); Observed masses: 26004 Da, 26023 Da, 26052 Da, 26070 Da, 26102 Da, 26181 Da, 26201 Da.

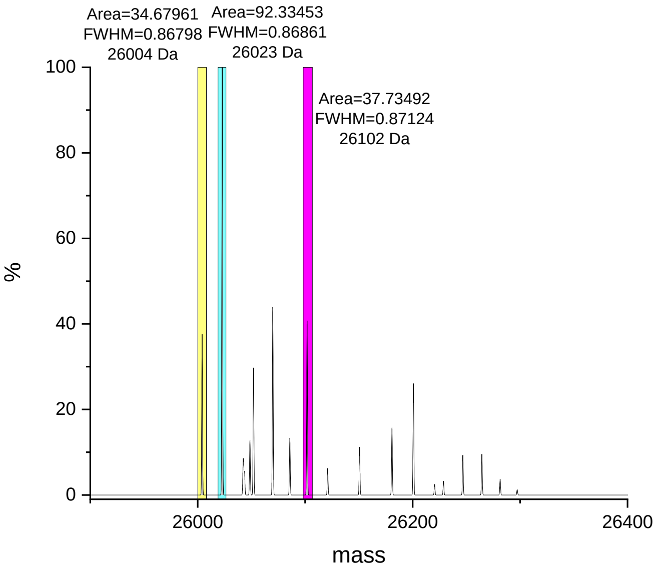

Figure S128. Peak areas of products after the reaction of DHAR1 S176C with 11 eq. **VTT** and in the absence of exogenous nucleophile. All observed side reactions were identified and integrated. To compare the results only side reactivity of the main peak in the starting material (25978 Da) was considered.

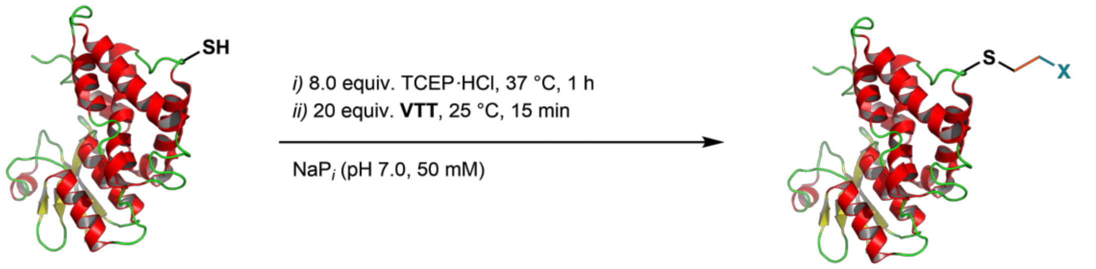

DHAR1 S176C, 25 µM **X = OH, OPO(OH)$_2$,nucleophilic AA**

At 20–25 °C, 55 µL of DHAR1 S176C (1.18 mg/mL, 46 µM, 2.5 nmol, 1.0 equiv.) in NaP$_i$ buffer (pH 7.0, c = 50 mM) was added to a 1.5 mL Eppendorf tube. Next, 44 µL of NaP$_i$ buffer (pH 7.0, c = 50 mM) was added. Then, 1.0 µL of a TCEP stock solution (20 mM, 0.02 µmol, 6 µg, 8 equiv.) in UHQ-H_2O was added. The mixture was vortexed for 1 second, transferred into a Thermocycler pre-heated at 37 °C, and incubated at 37 °C for 1 hour at 400 rpm. Next, 1.3 µL of a **VTT** stock solution (25 mM, 50 nmol, 17 µg, 20 equiv.) in DMF. The reaction mixture was vortexed for 1 second, transferred into a Thermocycler pre-heated at 25 °C, and incubated at 25 °C at 400 rpm for 15 minutes. Subsequently, 1.3 µL of a β-mercaptoethanol stock solution (25 mM, 50 nmol, 4.7 µg, 20 equiv.) in UHQ-H_2O was added, and the mixture was incubated at 25 °C for 15 minutes. The obtained mixture was analyzed by LC-MS.

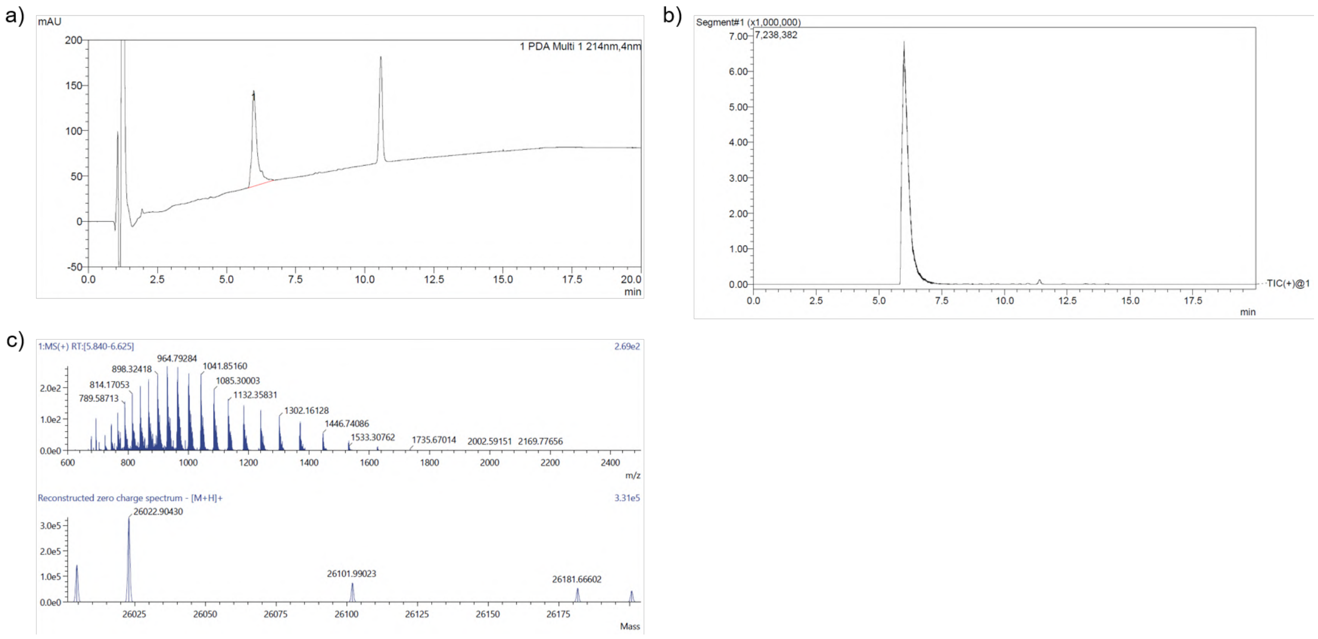

Figure S129. LC-MS analysis (Method B) of DHAR1 S176C after reaction with 20 equiv. **VTT**, without an exogenous nucleophile: a) UV-trace b) total ion chromatogram c) Ion series and deconvoluted spectrum; calculated masses: (DHAR1 S176C)–S–C_2H_4–OH: 26022 Da, 26200 Da (peaks detected in SM + 44 Da), (DHAR1 S176C)–S–C_2H_4–OPO_3H_2: 26102 Da, stapled-(DHAR1 S176C): 26005 Da, 26182 Da (peaks detected in SM + 26 Da); Observed masses: 26005 Da, 26023 Da, 26102 Da, 26182 Da, 26201 Da.

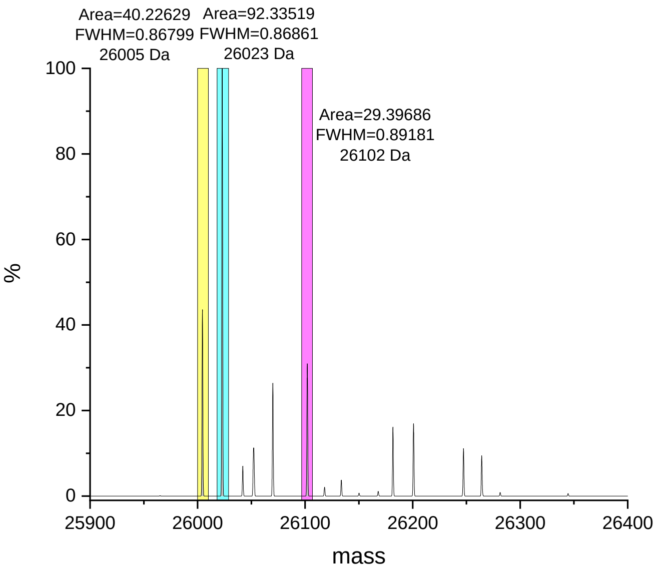

Figure S130. Peak areas of products after the reaction of DHAR1 S176C with 20 eq. **VTT** and in the absence of exogenous nucleophile. All observed side reactions were identified and integrated. To compare the results only side reactivity of the main peak in the starting material (25978 Da) was considered.

Reaction of sfGFP S147C and VTT without exogenous nucleophile

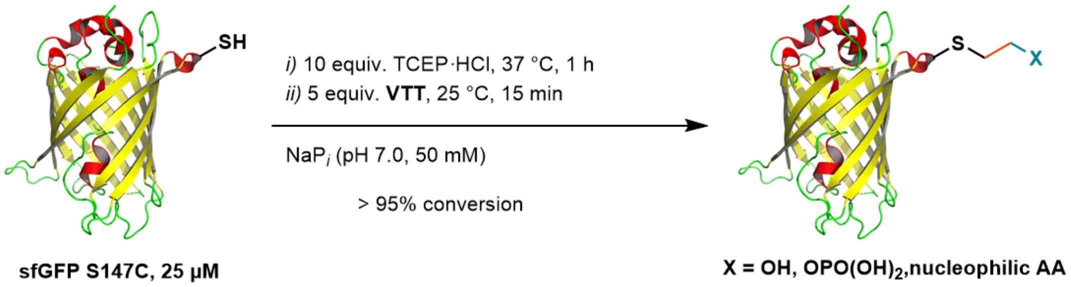

At 20–25 °C, 38 µL of sfGFP S147C (1.95 mg/mL, 67 µM, 2.5 nmol, 1.0 equiv.) in NaP*i* buffer (pH 7.0, c = 50 mM) was added to a 1.5 mL Eppendorf tube. Next, 62 µL of HEPES buffer (pH 7.0, c = 50 mM) was added. Then, 0.6 µL of a TCEP stock solution (20 mM, 0.01 µmol, 3 µg, 1 × 10^1 equiv.) in UHQ-H_2O was added. The mixture was vortexed for 1 second, transferred into a Thermocycler pre-heated at 37 °C, and incubated at 37 °C for 1 hour at 400 rpm. Next, 1.0 µL of a **VTT** stock solution (20 mM, 20 nmol, 6.6 µg, 5 equiv.) in DMF. The reaction mixture was vortexed for 1 second, transferred into a Thermocycler pre-heated at 25 °C, and incubated at 25 °C at 400 rpm for 15 minutes. Subsequently, 1.0 µL of a β-mercaptoethanol stock solution (20 mM, 20 nmol, 5 equiv.) in UHQ-H_2O was added, and the mixture was incubated at 25 °C for 15 minutes. The obtained mixture was analyzed by LC-MS.

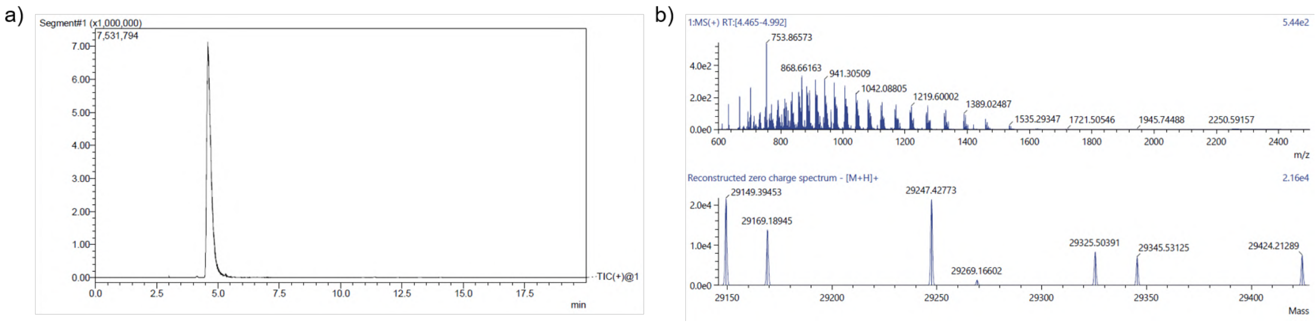

Figure S131. LC-MS analysis (Method B) of sfGFP S147C after reaction with 5 equiv. **VTT**, and without an exogeneous nucleophile: a) total ion chromatogram b) Ion series and deconvoluted spectrum; Calculated masses: (sfGFP S147C)–S–C_2H_4–OH: 29346 Da, stapled-(sfGFP S147C): 29147 Da, 29172 Da, 29328 Da, (sfGFP S147C)–S–C_2H_4–OPO_3H_2: 29245 Da, 29270 Da, 29426 Da; Observed masses: 29149 Da, 29169 Da, 29247 Da, 29269 Da, 29326 Da, 29346 Da, 29424 Da.

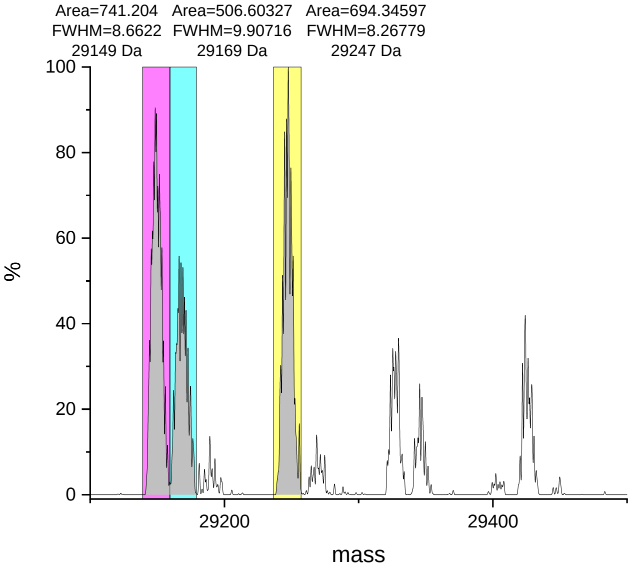

Figure S132. Peak areas of products after the reaction of sfGFP S147C with **VTT** and in the absence of exogenous nucleophile. All observed side reactions were identified and integrated. To compare the results only side reactivity of the main peak in the starting material (29121 Da) was considered.

The following table summarizes the observed side reactivities and quantifies them according to the calculated peak areas observed in the zero-charge spectra. The quantification was performed via the following equation:

$$side\ reactivcity = \frac{peak\ area\ of\ side\ product}{sum\ of\ peak\ areas\ of\ all\ side\ products}$$

Table S6. Observed side reactivities and their quantification based on calculated peak areas from zero-charge spectra.

protein	stapling (+26 Da) / %	hydrolysis (+44 Da) / %	phosphate adduct / %
DHAR1 S97C 11 eq. VTT	-	39	61
DHAR1 S97C 20 eq. VTT	-	41	59
DHAR1 S176C 11 eq. VTT	21	56	23
DHAR1 S176C 20 eq. VTT	25	57	18
sfGFP S147C	38	26	36

The results indicate no influence of the amount of vinyl thianthrenium reagent on the product distribution and side reactivity.

Investigation of site-specific functionalization of DHAR1 mutants

Synthesis of (DHAR1 S97C)–S–C_2H_4-N_3 with 11 equiv. VTT

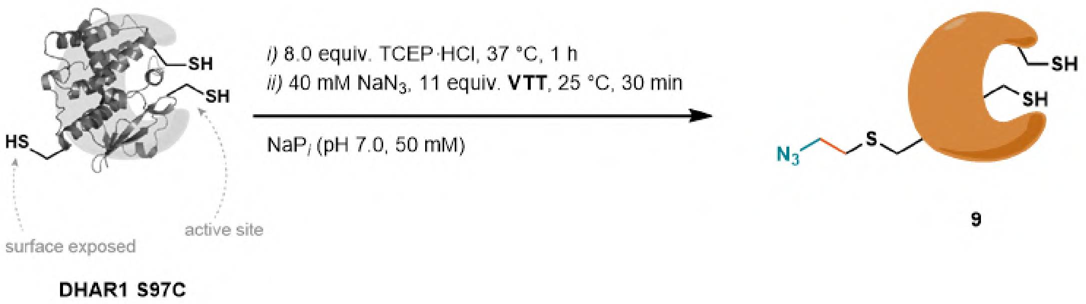

At 20–25 °C, 55 µL of DHAR1 S97C (1.18 mg/mL, 46.0 µM, 2.5 nmol, 1.0 equiv.) in NaP_i buffer (pH 7.0, c = 50 mM) was added to a 1.5 mL Eppendorf tube. Next, 31 µL NaP_i buffer (pH 7.0, c = 50 mM) was added. Then, 1.0 µL of a TCEP stock solution (20 mM, 20 nmol, 5.0 µg, 8.0 equiv.) in UHQ-H_2O was added. The mixture was vortexed, transferred into a Thermocycler pre-heated at 37 °C, and incubated at 37 °C for 1 hour at 400 rpm. Next, a 0.30 M sodium azide stock solution (13 µL, 4.0 µmol, 0.26 mg, 1.6 × 10^3 equiv.) in NaP_i buffer (pH 7.0, c = 50 mM) was added to the mixture at 20–25 °C (c_{Nu} = 40 mM), followed by addition of 0.7 µL of a **VTT** stock solution (40 mM, 0.03 µmol, 0.01 mg, 1 × 10^1 equiv.) in DMF. The reaction mixture was vortexed, transferred into a Thermocycler pre-heated at 25 °C, and incubated at 25 °C at 400 rpm for 30 minutes. After 30 minutes the reaction mixture was utilized for the DHAR1 enzyme assay.

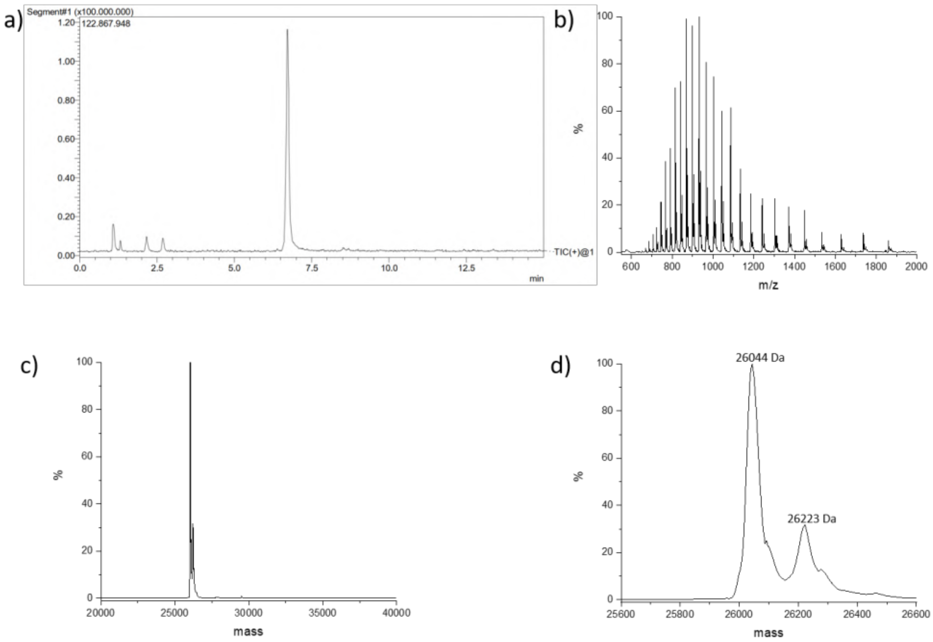

Figure S133. LC-MS analysis (Method A) of DHAR1 S97C after reaction with **VTT** and NaN_3: a) total ion chromatogram b) ion series c) deconvoluted spectrum d) zoom on the major peaks; calculated masses: ((DHAR1 S97C)–S–C_2H_4-N_3): 26045 Da, ((DHAR1 S97C)–S–C_2H_4-N_3 with N-terminal Ac-Met): 26220 Da observed masses: 26044 Da, 26223 Da. The spectra were recorded with a sample quenched with equimolar β-mercaptoethanol 30 minutes after addition of **VTT** to simulate the dilution into the DHAR enzyme assay.

Synthesis of (DHAR1 S97C)–S–C_2H_4-N_3 with 20 equiv. VTT

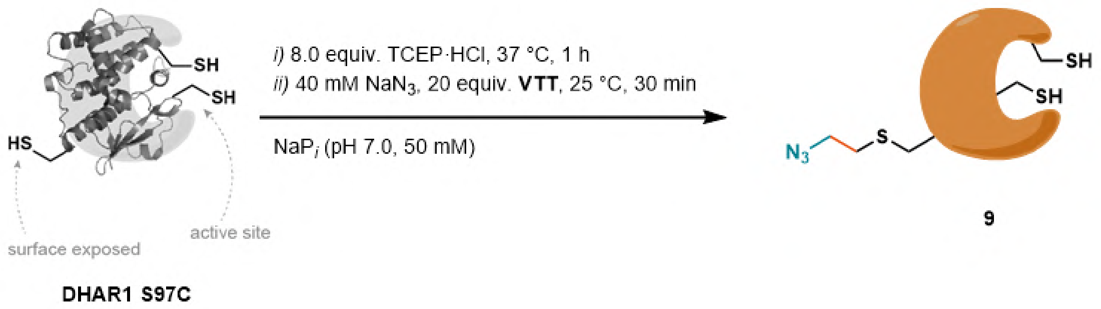

At 20–25 °C, 55 µL of DHAR1 S97C (1.18 mg/mL, 46.0 µM, 2.5 nmol, 1.0 equiv.) in NaP$_i$ buffer (pH 7.0, c = 50 mM) was added to a 1.5 mL Eppendorf tube. Next, 31 µL NaP$_i$ buffer (pH 7.0, c = 50 mM) was added. Then, 1.0 µL of a TCEP stock solution (20 mM, 20 nmol, 5.0 µg, 8.0 equiv.) in UHQ-H_2O was added. The mixture was vortexed, transferred into a Thermocycler pre-heated at 37 °C, and incubated at 37 °C for 1 hour at 400 rpm. Next, a 0.30 M sodium azide stock solution (13 µL, 4.0 µmol, 0.26 mg, 1.6 × 10^3 equiv.) in NaP$_i$ buffer (pH 7.0, c = 50 mM) was added to the mixture at 20–25 °C (c_{Nu} = 40 mM), followed by addition of 1.3 µL of a **VTT** stock solution (40 mM, 0.05 µmol, 0.02 mg, 2 × 10^1 equiv.) in DMF. The reaction mixture was vortexed, transferred into a Thermocycler pre-heated at 25 °C, and incubated at 25 °C at 400 rpm for 30 minutes. After 30 minutes the reaction mixture was utilized for the DHAR1 enzyme assay.

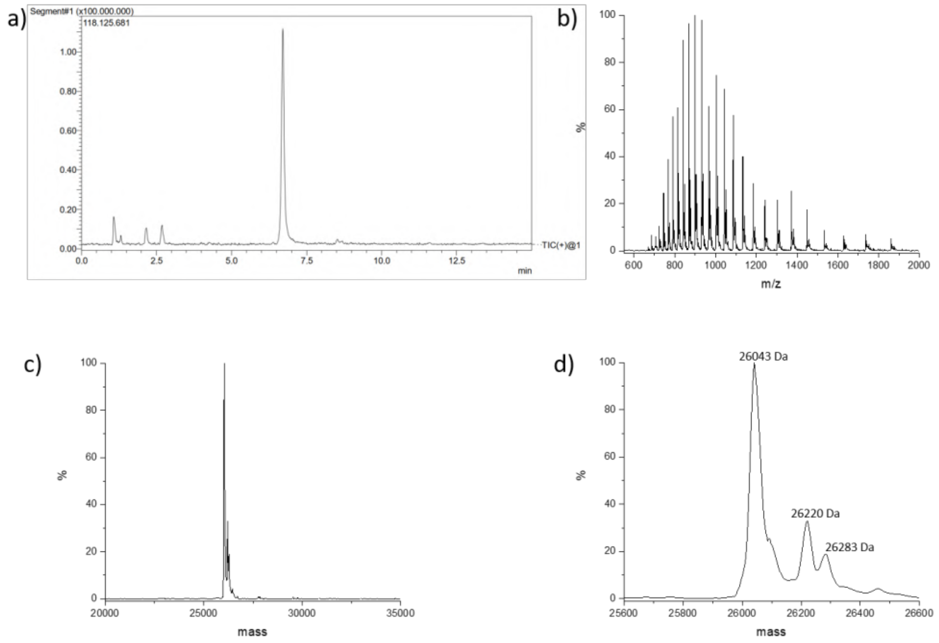

Figure S134. LC-MS analysis (Method A) of DHAR1 S97C after reaction with **VTT** and NaN_3: a) total ion chromatogram b) ion series c) deconvoluted spectrum d) zoom on the major peaks; calculated masses: ((DHAR1 S97C)–S–C_2H_4-N_3): 26045 Da, ((DHAR1 S97C)–S–C_2H_4-N_3 with N-terminal Ac-Met): 26220 Da observed masses: 26043 Da, 26220 Da, 26283 Da. The spectra were recorded with a sample quenched with equimolar β-mercaptoethanol 30 minutes after addition of **VTT** to simulate the dilution into the DHAR enzyme assay.

Synthesis of (DHAR1 S176C)–S–C_2H_4-N_3 with 11 equiv. VTT

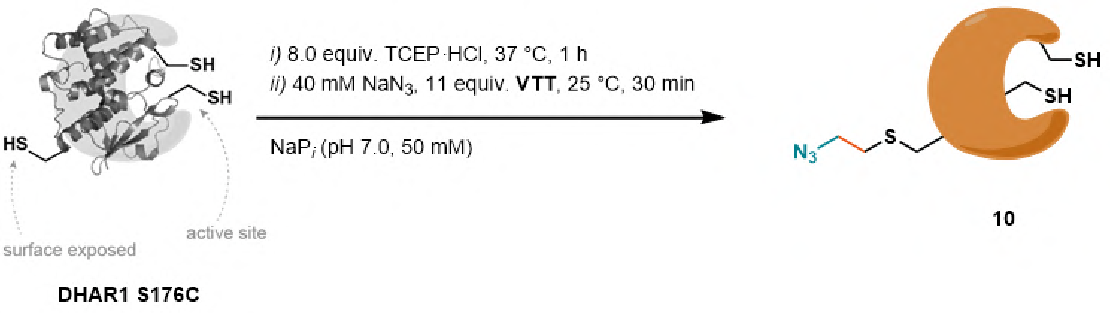

At 20–25 °C, 54 µL of DHAR1 S176C (1.20 mg/mL, 47.0 µM, 2.5 nmol, 1.0 equiv.) in NaP*i* buffer (pH 7.0, c = 50 mM) was added to a 1.5 mL Eppendorf tube. Next, 31 µL NaPi buffer (pH 7.0, c = 50 mM) was added. Then, 1.0 µL of a TCEP stock solution (20 mM, 20 nmol, 5.0 µg, 8.0 equiv.) in UHQ-H_2O was added. The mixture was vortexed, transferred into a Thermocycler pre-heated at 37 °C, and incubated at 37 °C for 1 hour at 400 rpm. Next, a 0.30 M sodium azide stock solution (13 µL, 4.0 µmol, 0.26 mg, 1.6×10^3 equiv.) in NaP*i* buffer (pH 7.0, c = 50 mM) was added to the mixture at 20–25 °C (c_{Nu} = 40 mM), followed by addition of 0.7 µL of a **VTT** stock solution (40 mM, 0.03 µmol, 0.01 mg, 1×10^1 equiv.) in DMF. The reaction mixture was vortexed, transferred into a Thermocycler pre-heated at 25 °C, and incubated at 25 °C at 400 rpm for 30 minutes. After 30 minutes the reaction mixture was utilized for the DHAR1 enzyme assay.

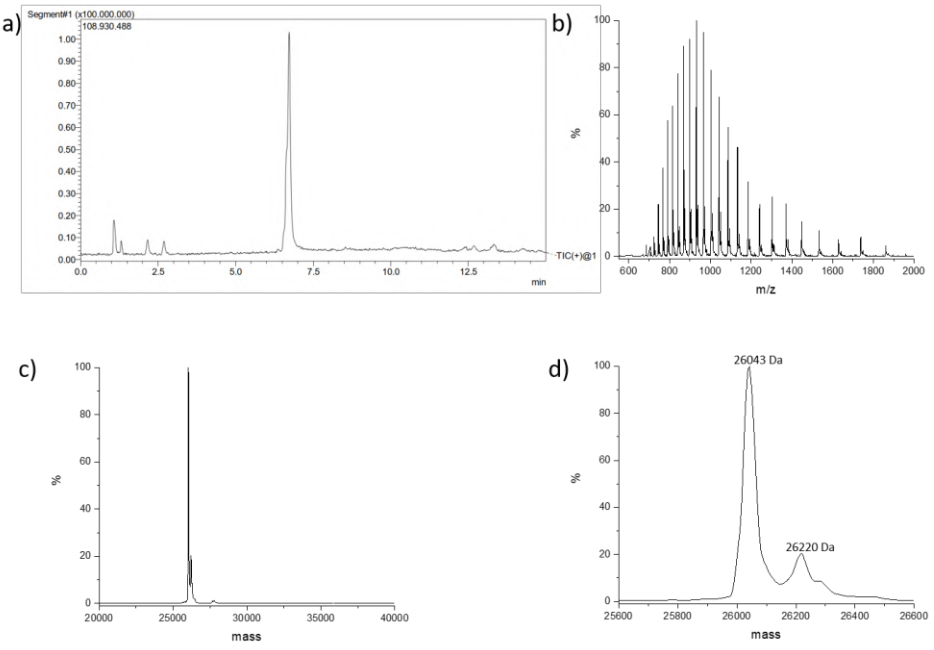

Figure S135. LC-MS analysis (Method A) of DHAR1 S176C after reaction with **VTT** and NaN_3: a) total ion chromatogram b) ion series c) deconvoluted spectrum d) zoom on the major peaks; calculated masses: ((DHAR1 S176C)–S–C_2H_4-N_3): 26043 Da, ((DHAR1 S176C)–S–C_2H_4-N_3 with N-terminal Ac-Met): 26221 Da observed masses: 26043 Da, 26220 Da. The spectra were recorded with a sample quenched with equimolar β-mercaptoethanol 30 minutes after addition of **VTT** to simulate the dilution into the DHAR enzyme assay.

Synthesis of (DHAR1 S176C)–S–C_2H_4-N_3 with 20 equiv. VTT

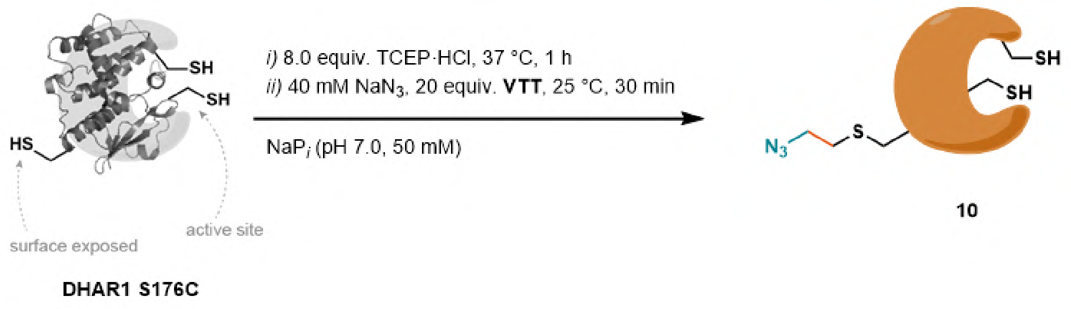

At 20–25 °C, 54 µL of DHAR1 S176C (1.20 mg/mL, 47.0 µM, 2.5 nmol, 1.0 equiv.) in NaP$_i$ buffer (pH 7.0, c = 50 mM) was added to a 1.5 mL Eppendorf tube. Next, 31 µL NaP$_i$ buffer (pH 7.0, c = 50 mM) was added. Then, 1.0 µL of a TCEP stock solution (20 mM, 20 nmol, 5.0 µg, 8.0 equiv.) in UHQ-H_2O was added. The mixture was vortexed, transferred into a Thermocycler pre-heated at 37 °C, and incubated at 37 °C for 1 hour at 400 rpm. Next, a 0.30 M sodium azide stock solution (13 µL, 4.0 µmol, 0.26 mg, 1.6×10^3 equiv.) in NaP$_i$ buffer (pH 7.0, c = 50 mM) was added to the mixture at 20–25 °C (c_{Nu} = 40 mM), followed by addition of 1.3 µL of a **VTT** stock solution (40 mM, 0.05 µmol, 0.02 mg, 2×10^1 equiv.) in DMF. The reaction mixture was vortexed, transferred into a Thermocycler pre-heated at 25 °C, and incubated at 25 °C at 400 rpm for 30 minutes. After 30 minutes the reaction mixture was utilized for the DHAR1 enzyme assay.

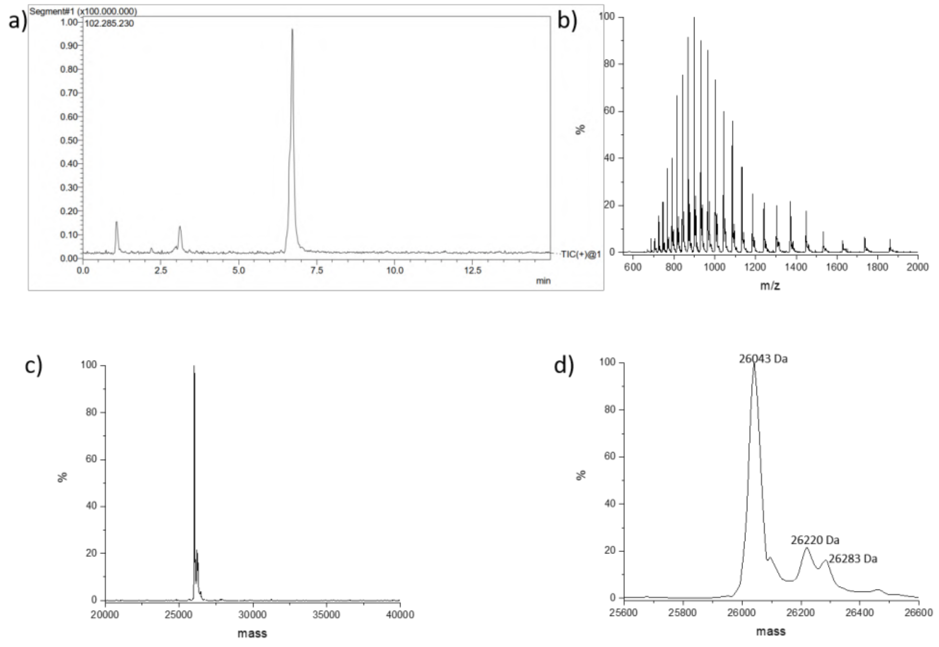

Figure S136. LC-MS analysis (Method A) of DHAR1 S176C after reaction with **VTT** and NaN_3: a) total ion chromatogram b) ion series c) deconvoluted spectrum d) zoom on the major peaks; calculated masses: ((DHAR1 S176C)–S–C_2H_4-N_3): 26043 Da, ((DHAR1 S176C)–S–C_2H_4-N_3 with N-terminal Ac-Met): 26221 Da observed masses: 26043 Da, 26220 Da, 26283 Da. The spectra were recorded with a sample quenched with equimolar β-mercaptoethanol 30 minutes after addition of **VTT** to simulate the dilution into the DHAR enzyme assay.

Synthesis of (DHAR1 S97C)–(S–NMM)$_2$ with 11 equiv. NMM

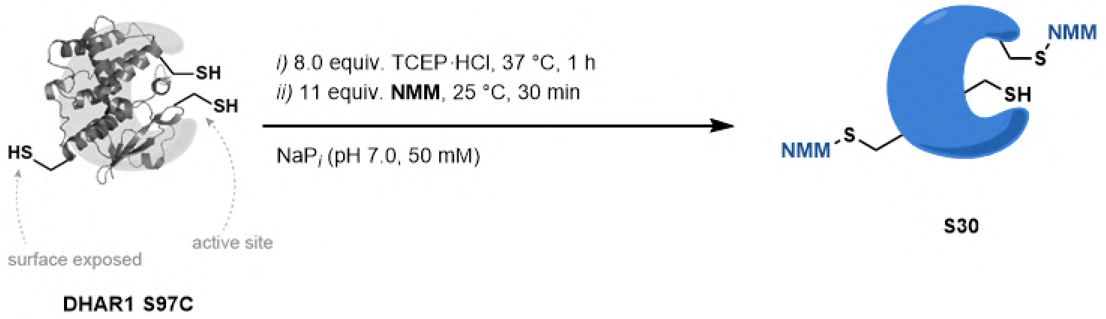

At 20–25 °C, 55 µL of DHAR1 S97C (1.18 mg/mL, 46.0 µM, 2.5 nmol, 1.0 equiv.) in NaP$_i$ buffer (pH 7.0, c = 50 mM) was added to a 1.5 mL Eppendorf tube. Next, 44 µL NaP$_i$ buffer (pH 7.0, c = 50 mM) was added. Then, 1.0 µL of a TCEP stock solution (20 mM, 20 nmol, 5.0 µg, 8.0 equiv.) in UHQ-H_2O was added. The mixture was vortexed, transferred into a Thermocycler pre-heated at 37 °C, and incubated at 37 °C for 1 hour at 400 rpm. Next, 0.7 µL of a **NMM** stock solution (40 mM, 0.03 µmol, 3 µg, 1 × 10^1 equiv.) in DMF were added to the mixture. The reaction mixture was vortexed, transferred into a Thermocycler pre-heated at 25 °C, and incubated at 25 °C at 400 rpm for 30 minutes. After 30 minutes the reaction mixture was utilized for the DHAR1 enzyme assay.

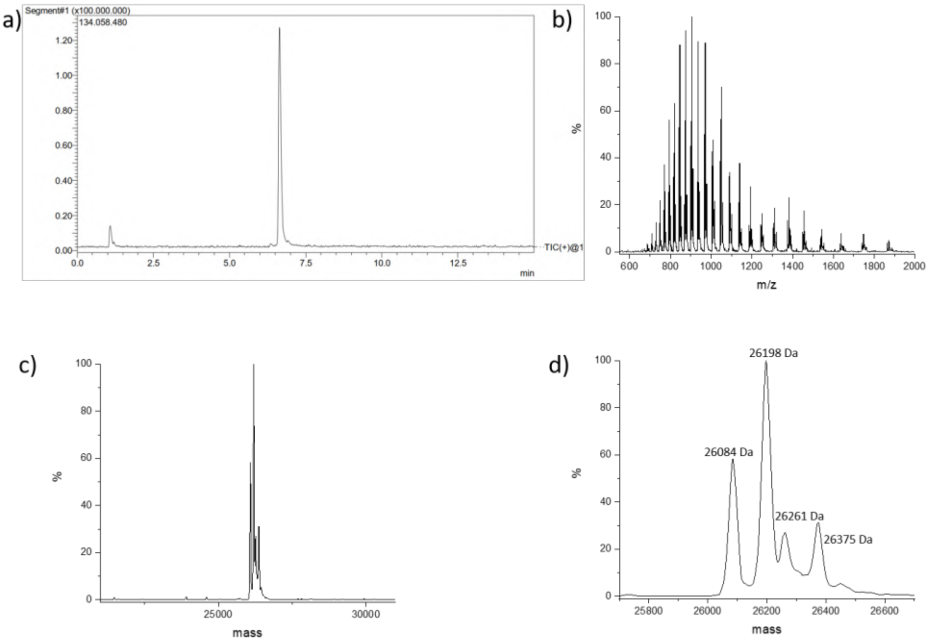

Figure S137. LC-MS analysis (Method A) of DHAR1 S97C after reaction with 11 eq. **NMM**: a) total ion chromatogram b) ion series c) deconvoluted spectrum d) zoom on the major peaks; calculated masses: ((DHAR1 S97C)–S–NMM): 26087 Da ((DHAR1 S97C)–(S–NMM)$_2$): 26198 Da, ((DHAR1 S97C)–S–NMM with N-terminal Ac-Met): 26262, ((DHAR1 S97C)–(S–NMM)$_2$ with N-terminal Ac-Met): 26373 Da observed masses: 26084 Da, 26198 Da, 26261 Da, 26375 Da. The spectra were recorded with a sample quenched with equimolar β-mercaptoethanol 30 minutes after addition of **NMM** to simulate the dilution into the DHAR enzyme assay.

Synthesis of (DHAR1 S176C)–(S–NMM)$_2$ with 11 equiv. NMM

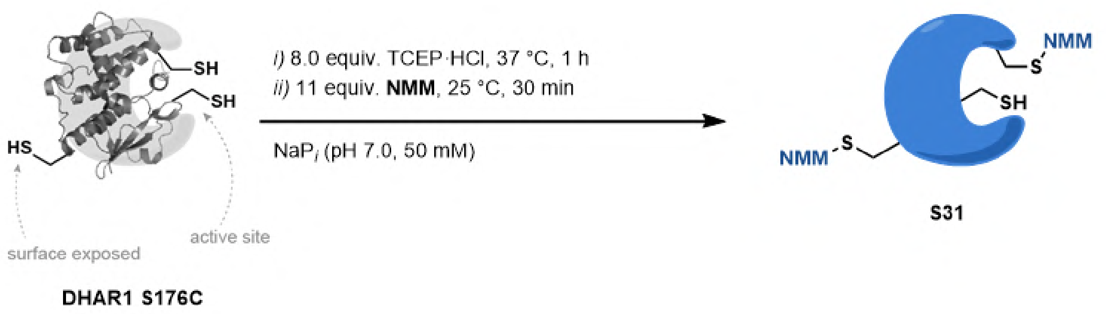

At 20–25 °C, 54 µL of DHAR1 S176C (1.20 mg/mL, 47.0 µM, 2.5 nmol, 1.0 equiv.) in NaP$_i$ buffer (pH 7.0, c = 50 mM) was added to a 1.5 mL Eppendorf tube. Next, 45 µL NaP$_i$ buffer (pH 7.0, c = 50 mM) was added. Then, 1.0 µL of a TCEP stock solution (20 mM, 20 nmol, 5.0 µg, 8.0 equiv.) in UHQ-H_2O was added. The mixture was vortexed, transferred into a Thermocycler pre-heated at 37 °C, and incubated at 37 °C for 1 hour at 400 rpm. Next, 0.7 µL of a **NMM** stock solution (40 mM, 0.03 µmol, 3 µg, 1×10^1 equiv.) in DMF were added to the mixture. The reaction mixture was vortexed, transferred into a Thermocycler pre-heated at 25 °C, and incubated at 25 °C at 400 rpm for 30 minutes. After 30 minutes the reaction mixture was utilized for the DHAR1 enzyme assay.

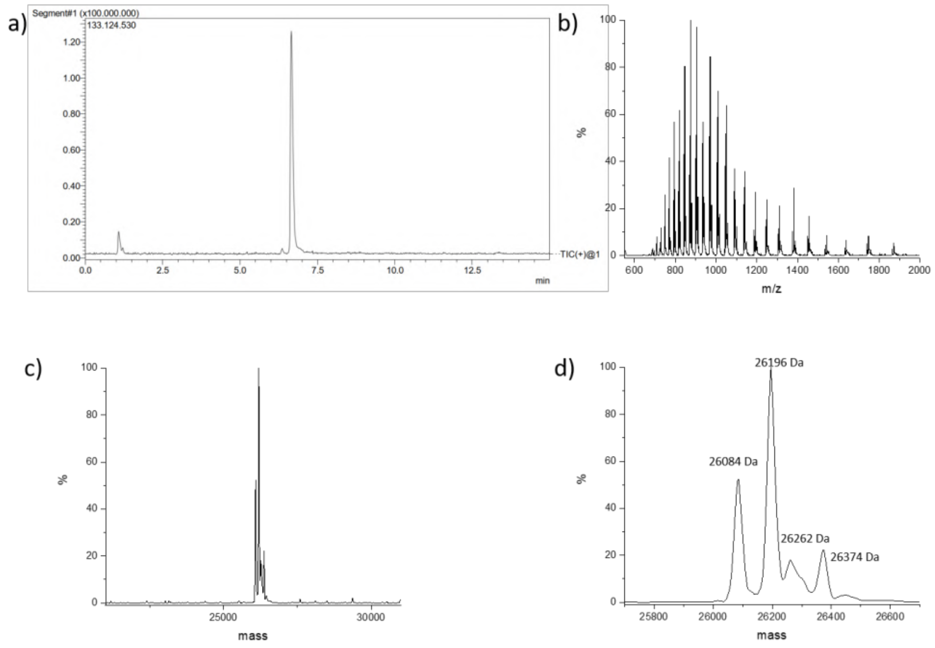

Figure S138. LC-MS analysis (Method A) of DHAR1 S176C after reaction with 11 eq. **NMM**: a) total ion chromatogram b) ion series c) deconvoluted spectrum d) zoom on the major peaks; calculated masses: ((DHAR1 S176C)–S–NMM): 26085 Da ((DHAR1 S176C)–(S–NMM)$_2$): 26196 Da, ((DHAR1 S176C)–S–NMM with N-terminal Ac-Met): 26263, ((DHAR1 S176C)–(S–NMM)$_2$ with N-terminal Ac-Met): 26374 Da observed masses: 26084 Da, 26196 Da, 26262 Da, 26374 Da. The spectra were recorded with a sample quenched with equimolar β-mercaptoethanol 30 minutes after addition of **NMM** to simulate the dilution into the DHAR enzyme assay.

Synthesis of (DHAR1 S97C)–(S–NMM)$_2$ with 20 equiv. NMM

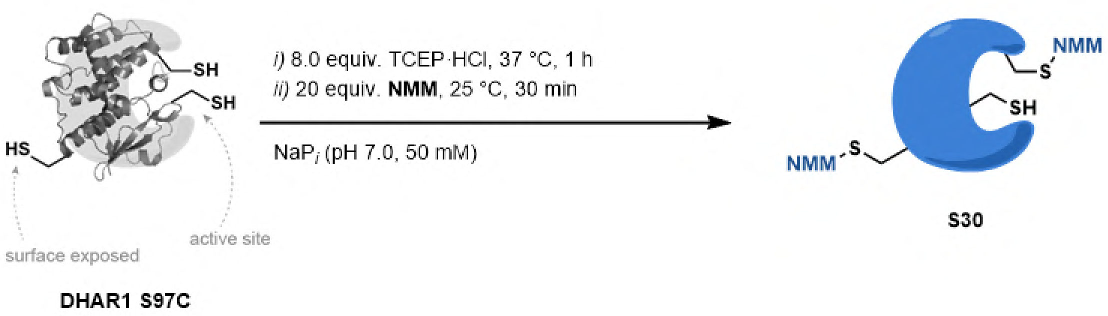

At 20–25 °C, 55 µL of DHAR1 S97C (1.18 mg/mL, 46.0 µM, 2.5 nmol, 1.0 equiv.) in NaP$_i$ buffer (pH 7.0, c = 50 mM) was added to a 1.5 mL Eppendorf tube. Next, 44 µL NaP$_i$ buffer (pH 7.0, c = 50 mM) was added. Then, 1.0 µL of a TCEP stock solution (20 mM, 20 nmol, 5.0 µg, 8.0 equiv.) in UHQ-H_2O was added. The mixture was vortexed, transferred into a Thermocycler pre-heated at 37 °C, and incubated at 37 °C for 1 hour at 400 rpm. Next, 0.7 µL of a **NMM** stock solution (40 mM, 50 nmol, 5.6 µg, 20 equiv.) in DMF were added to the mixture. The reaction mixture was vortexed, transferred into a Thermocycler pre-heated at 25 °C, and incubated at 25 °C at 400 rpm for 30 minutes. After 30 minutes the reaction mixture was utilized for the DHAR1 enzyme assay.

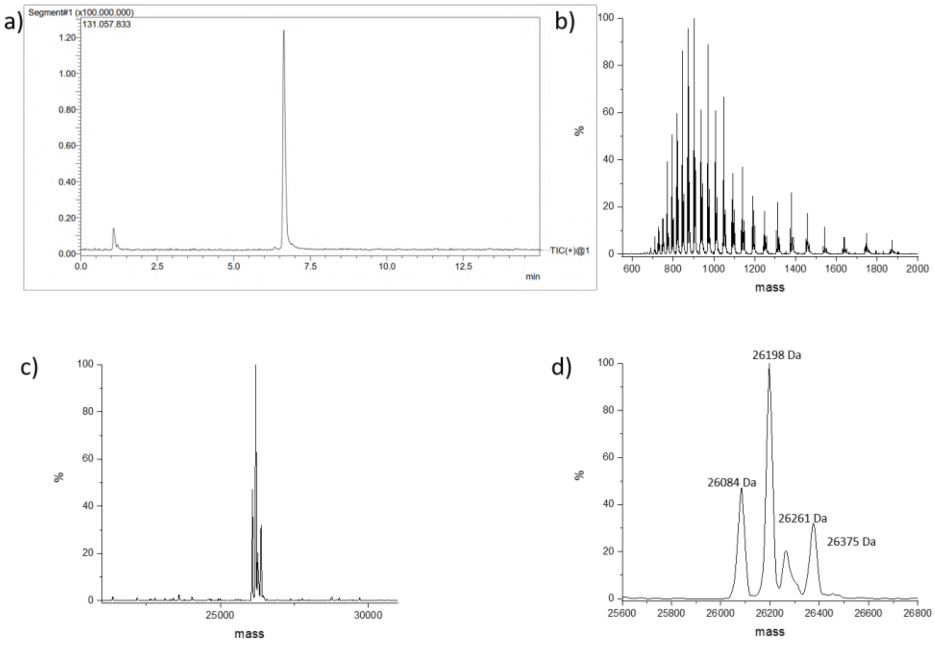

Figure S139. LC-MS analysis (Method A) of DHAR1 S97C after reaction with 20 eq. **NMM**: a) total ion chromatogram b) ion series c) deconvoluted spectrum d) zoom on the major peaks; calculated masses: ((DHAR1 S97C)–S–NMM): 26085 Da ((DHAR1 S97C)–(S–NMM)$_2$): 26196 Da, ((DHAR1 S97C)–S–NMM with N-terminal Ac-Met): 26263, ((DHAR1 S97C)–(S–NMM)$_2$ with N-terminal Ac-Met): 26374 Da observed masses: 26084 Da, 26198 Da, 26261 Da, 26375 Da. The spectra were recorded with a sample quenched with equimolar β-mercaptoethanol 30 minutes after addition of **NMM** to simulate the dilution into the DHAR enzyme assay.

Synthesis of (DHAR1 S176C)–(S–NMM)$_2$ with 20 equiv. NMM

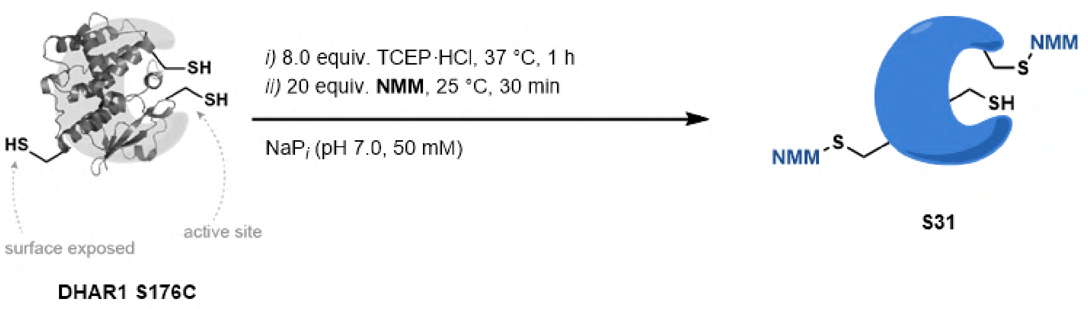

At 20–25 °C, 54 µL of DHAR1 S176C (1.20 mg/mL, 47.0 µM, 2.5 nmol, 1.0 equiv.) in NaP$_i$ buffer (pH 7.0, c = 50 mM) was added to a 1.5 mL Eppendorf tube. Next, 45 µL NaP$_i$ buffer (pH 7.0, c = 50 mM) was added. Then, 1.0 µL of a TCEP stock solution (20 mM, 20 nmol, 5.0 µg, 8.0 equiv.) in UHQ-H_2O was added. The mixture was vortexed, transferred into a Thermocycler pre-heated at 37 °C, and incubated at 37 °C for 1 hour at 400 rpm. Next, 1.3 µL of a **NMM** stock solution (40 mM, 50 nmol, 5.6 µg, 20 equiv.) in DMF were added to the mixture. The reaction mixture was vortexed, transferred into a Thermocycler pre-heated at 25 °C, and incubated at 25 °C at 400 rpm for 30 minutes. After 30 minutes the reaction mixture was utilized for the DHAR1 enzyme assay.

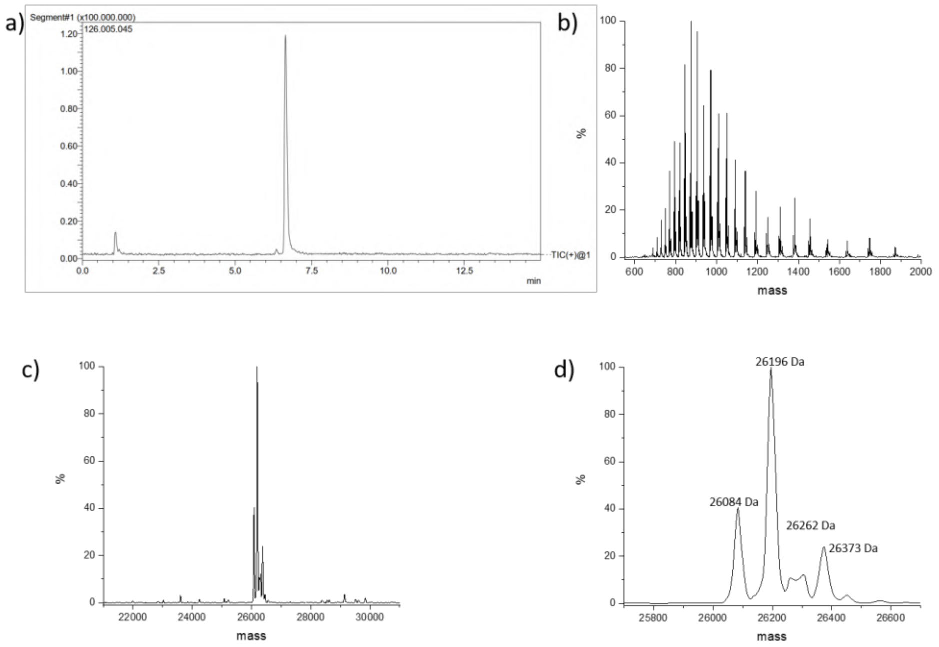

Figure S140. LC-MS analysis (Method A) of DHAR1 S176C after reaction with 20 eq. **NMM**: a) total ion chromatogram b) ion series c) deconvoluted spectrum d) zoom on the major peaks; calculated masses: ((DHAR1 S176C)–S–NMM): 26085 Da ((DHAR1 S176C)–(S–NMM)$_2$): 26196 Da, ((DHAR1 S176C)–S–NMM with N-terminal Ac-Met): 26263, ((DHAR1 S176C)–(S–NMM)$_2$ with N-terminal Ac-Met): 26374 Da observed masses: 26084 Da, 26196 Da, 26262 Da, 26373 Da. The spectra were recorded with a sample quenched with equimolar β-mercaptoethanol 30 minutes after addition of **NMM** to simulate the dilution into the DHAR enzyme assay.

Reaction of DHAR1 with different maleimide reagents

In order to exclude that only **NMM** displays the observed unspecific reactivity towards the catalytic Cys residues because of the small size of the reagent, we evaluated additional maleimide derivatives with larger substituents. Additionally, we conducted experiments with **VTT** and azide as nucleophile.

Synthesis of (DHAR1)–S–NMM with NMM

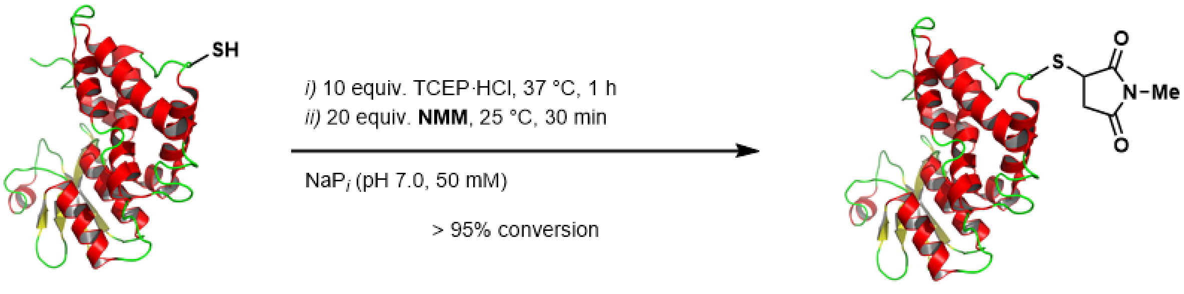

DHAR1 **S28**

At 20–25 °C, 26 µL of DHAR1 (2.51 mg/mL, 96.0 µM, 2.5 nmol, 1.0 equiv.) in NaP$_i$ buffer (pH 7.0, c = 50 mM) was added to a 1.5 mL Eppendorf tube. Next, 72 µL NaP$_i$ buffer (pH 7.0, c = 50 mM) was added. Then, 1.3 µL of a TCEP stock solution (20 mM, 25 nmol, 6.3 µg, 10 equiv.) in UHQ-H_2O was added. The mixture was vortexed, transferred into a Thermocycler pre-heated at 37 °C, and incubated at 37 °C for 1 hour at 400 rpm. Next, 1.0 µL of a **NMM** stock solution (50 mM, 50 nmol, 5.6 µg, 20 equiv.) in DMF were added to the mixture. The reaction mixture was vortexed, transferred into a Thermocycler pre-heated at 25 °C, and incubated at 25 °C at 400 rpm for 30 minutes. The mixture was diluted with 0.37 mL UHQ-H_2O to obtain a protein concentration of 0.14 mg/mL. The obtained mixture was analyzed by LC-MS.

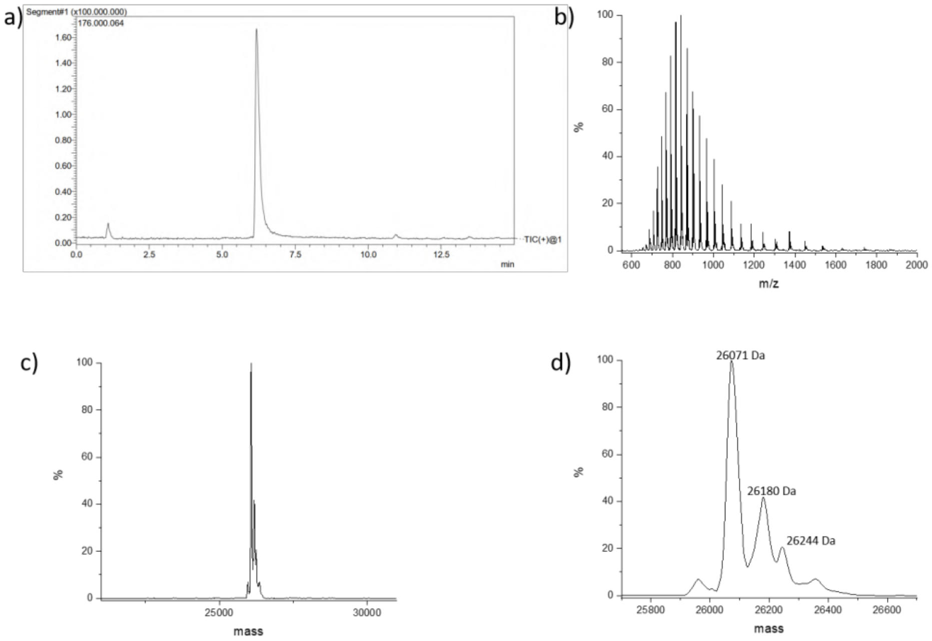

Figure S141. LC-MS analysis (Method A) of DHAR1 after reaction with **NMM**: a) total ion chromatogram b) ion series c) deconvoluted spectrum d) zoom on the major peaks; calculated masses: ((DHAR1)–S–NMM): 26070 Da, ((DHAR1)–(S–NMM)$_2$): 26181 Da, ((DHAR1)–S–NMM with N-terminal Ac-Met): 26247 Da, observed

masses: 26071 Da, 26180 Da, 26244 Da.

Synthesis of (DHAR1)–S–NPM with NPM

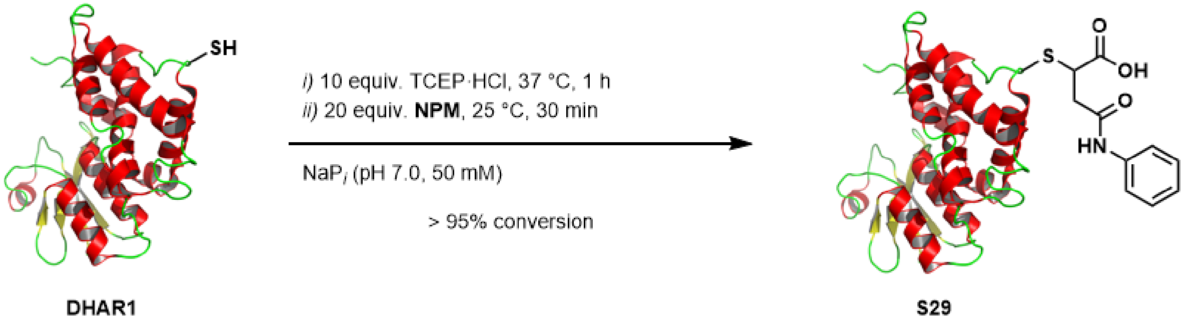

At 20–25 °C, 26 µL of DHAR1 (2.51 mg/mL, 96.0 µM, 2.5 nmol, 1.0 equiv.) in NaP$_i$ buffer (pH 7.0, c = 50 mM) was added to a 1.5 mL Eppendorf tube. Next, 72 µL NaP$_i$ buffer (pH 7.0, c = 50 mM) was added. Then, 1.3 µL of a TCEP stock solution (20 mM, 25 nmol, 6.3 µg, 10 equiv.) in UHQ-H_2O was added. The mixture was vortexed, transferred into a Thermocycler pre-heated at 37 °C, and incubated at 37 °C for 1 hour at 400 rpm. Next, 1.0 µL of a **NPM** stock solution (50 mM, 50 nmol, 8.7 µg, 20 equiv.) in DMF were added to the mixture. The reaction mixture was vortexed, transferred into a Thermocycler pre-heated at 25 °C, and incubated at 25 °C at 400 rpm for 30 minutes. The mixture was diluted with 0.37 mL UHQ-H_2O to obtain a protein concentration of 0.14 mg/mL. The obtained mixture was analyzed by LC-MS.

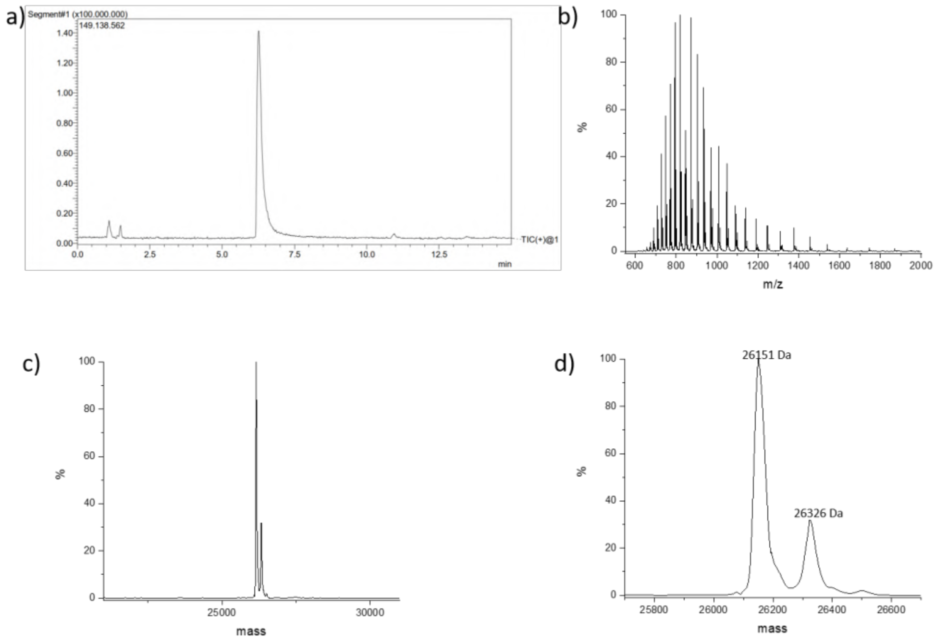

Figure S142. LC-MS analysis (Method A) of DHAR1 after reaction with **NPM**: a) total ion chromatogram b) ion series c) deconvoluted spectrum d) zoom on the major peaks; calculated masses: ((DHAR1)–S–(**NPM**+H_2O)): 26150 Da, (DHAR1)–S–(**NPM**+H_2O) with N-terminal Ac-Met: 26327 Da; observed masses: 26151 Da, 26326 Da.

Synthesis of (DHAR1)–S–NpCPM with NpCPM

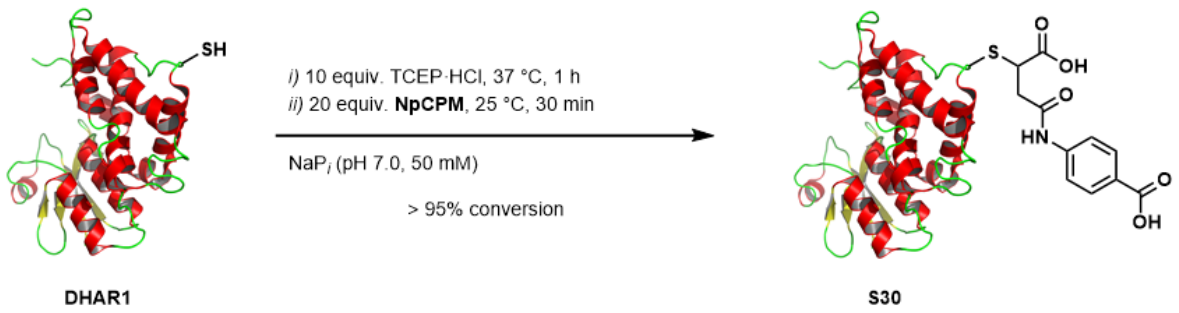

At 20–25 °C, 26 µL of DHAR1 (2.51 mg/mL, 96.0 µM, 2.5 nmol, 1.0 equiv.) in NaP_i buffer (pH 7.0, c = 50 mM) was added to a 1.5 mL Eppendorf tube. Next, 72 µL NaP_i buffer (pH 7.0, c = 50 mM) was added. Then, 1.3 µL of a TCEP stock solution (20 mM, 25 nmol, 6.3 µg, 10 equiv.) in UHQ-H_2O was added. The mixture was vortexed, transferred into a Thermocycler pre-heated at 37 °C, and incubated at 37 °C for 1 hour at 400 rpm. Next, 1.0 µL of a **NpCPM** stock solution (50 mM, 50 nmol, 11 µg, 20 equiv.) in DMF were added to the mixture. The reaction mixture was vortexed, transferred into a Thermocycler pre-heated at 25 °C, and incubated at 25 °C at 400 rpm for 30 minutes. The mixture was diluted with 0.37 mL UHQ-H_2O to obtain a protein concentration of 0.14 mg/mL. The obtained mixture was analyzed by LC-MS.

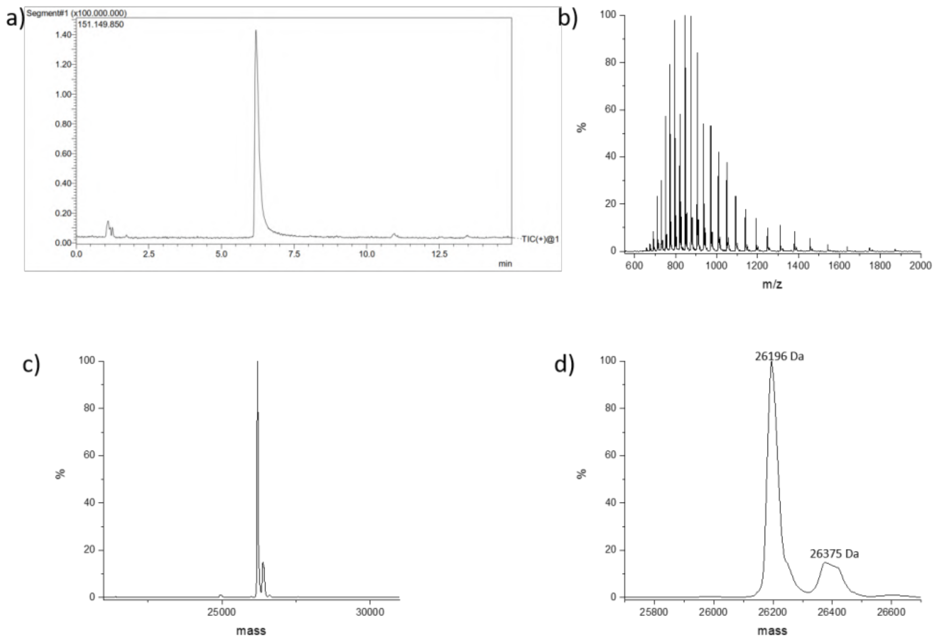

Figure S143. LC-MS analysis (Method A) of DHAR1 after reaction with **NpCPM**: a) total ion chromatogram b) ion series c) deconvoluted spectrum d) zoom on the major peaks; calculated masses: ((DHAR1)–S–(**NpCM**+H_2O)): 26194 Da, (DHAR1)–S–(**NpCPM**+H_2O) with N-terminal Ac-Met: 26371 Da; observed masses: 26196 Da, 26375 Da.

Reaction of DHAR1 with VTT and NaN_3

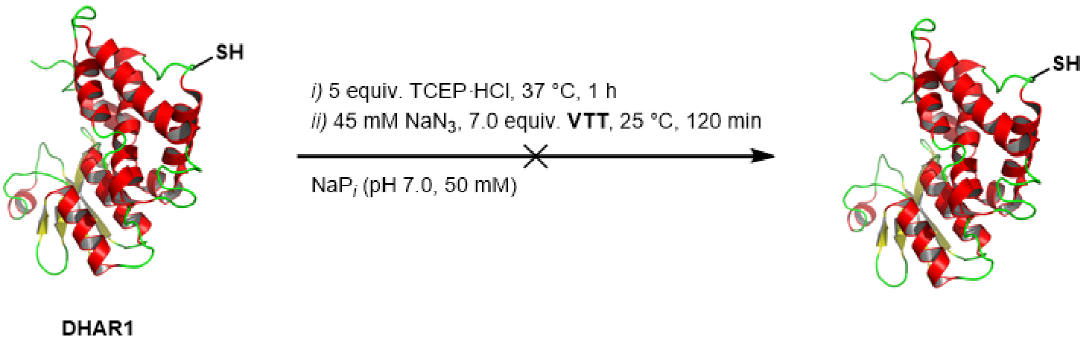

At 20–25 °C, 26 µL of DHAR1 (2.47 mg/mL, 94.7 µM, 2.5 nmol, 1.0 equiv.) in NaP_i buffer (pH 7.0, c = 50 mM) was added to a 1.5 mL Eppendorf tube. Next, 20 µL NaP_i buffer (pH 7.0, c = 50 mM) was added. Then, 1.1 µL of a TCEP stock solution (11 mM, 13 nmol, 3.3 µg, 5.0 equiv.) in UHQ-H_2O was added. The mixture was vortexed, transferred into a Thermocycler pre-heated at 37 °C, and incubated at 37 °C for 1 hour at 400 rpm. Next, a 0.30 M sodium azide stock solution (7.5 µL , 2.3 µmol, 0.15 mg, 9.0 × 10^2 equiv.) in NaP_i buffer (pH 7.0, c = 50 mM) was added to the mixture at 20–25 °C (c_{Nu} = 45 mM), followed by addition of 0.7 µL of a **VTT** stock solution (25 mM, 0.02 µmol, 8 µg, 7 equiv.) in DMF. The reaction mixture was vortexed, transferred into a Thermocycler pre-heated at 25 °C, and incubated at 25 °C at 400 rpm for 120 minutes. The mixture was diluted with 0.45 mL UHQ-H_2O to obtain a protein concentration of 0.13 mg/mL. The obtained mixture was analyzed by LC-MS.

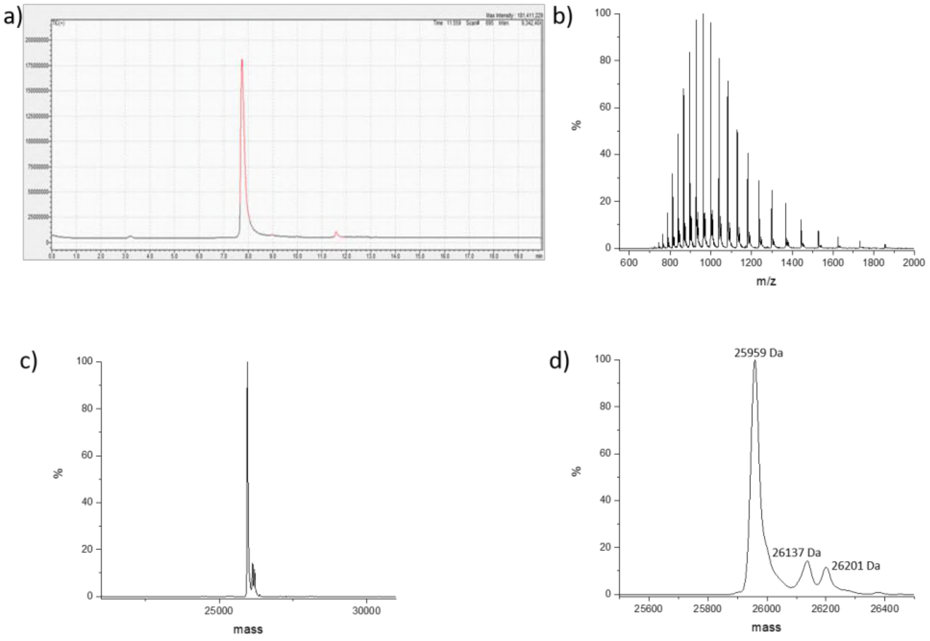

Figure S144. LC-MS analysis (Method A) of DHAR1 after reaction with **VTT** and sodium azide: a) total ion chromatogram b) ion series c) deconvoluted spectrum d) zoom on the major peaks; calculated masses: ((DHAR1)–S–C_2H_4–N_3): 26028 Da, ((DHAR1)–S– S–C_2H_4–N_3) with N-terminal Ac-Met): 26205 Da, observed masses: 25959 Da (starting material), 26137 Da (starting material), 26201 Da.

Screening of different maleimide concentration in the conjugation with DHAR1 mutants

In order to exclude that site-specific functionalization with NMM can be achieved if lower concentrations of NMM are used, we performed a dose dependence experiment with NMM and DHAR1 mutants. LC-MS analysis revealed that the lowest concentration chosen (equal to 3.1 eq NMM) is enough for monofunctionalization (<5% of starting material was left at these reaction conditions). We afterwards submitted all samples to an activity assay in order to check for preserved enzymatic activity and observed decrease in enzymatic activity in all cases (see Figure S149), in line with low site-specificity of NMM even at low concentrations.

Screening of different maleimide concentration in the conjugation with DHAR1 S97C

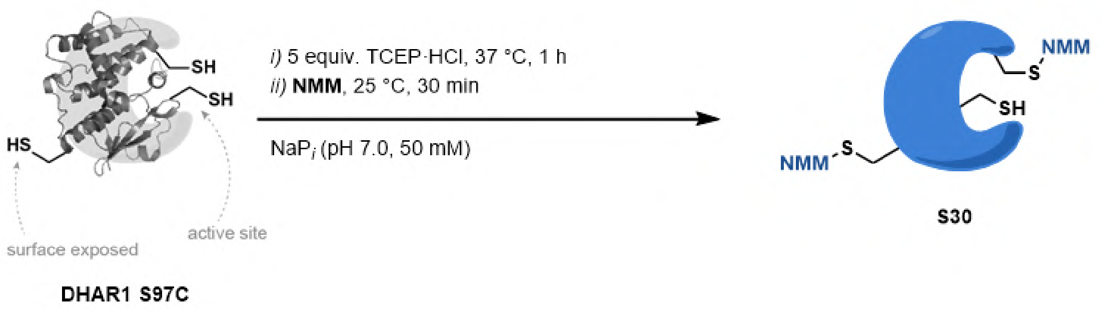

At 20–25 °C, 68 µL of DHAR1 S97C (1.22 mg/mL, 47.0 µM, 3.2 nmol, 1.0 equiv.) in NaP$_i$ buffer (pH 7.0, c = 50 mM) was added to a 1.5 mL Eppendorf tube. Next, 131 µL NaP$_i$ buffer (pH 7.0, c = 50 mM) was added. Then, 1.5 µL of a TCEP stock solution (10 mM, 15 nmol, 3.8 µg, 4.7 equiv.) in UHQ-H_2O was added. The mixture was vortexed, transferred into a Thermocycler pre-heated at 37 °C, and incubated at 37 °C for 1 hour at 400 rpm. Next, varying volumes (see Table S7) of a 10 mM **NMM** stock solution in DMF were added to the mixture. The reaction mixture was vortexed, transferred into a Thermocycler pre-heated at 25 °C, and incubated at 25 °C at 400 rpm for 30 minutes. After 30 minutes the reaction mixture was utilized for the DHAR1 enzyme assay.

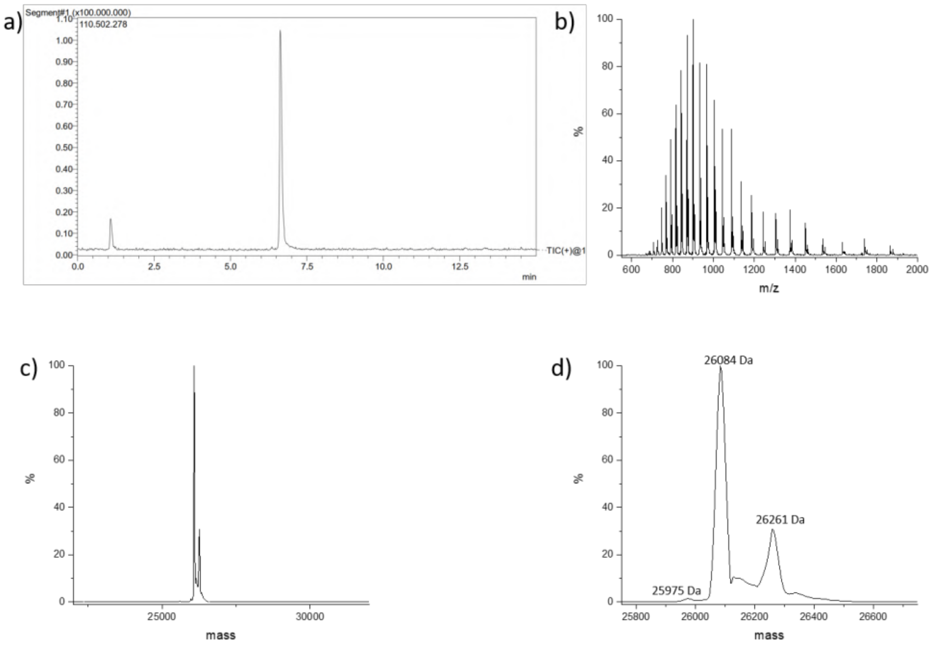

Figure S145. LC-MS analysis (Method A) of DHAR1 S97C after reaction with 3.1 equiv. of **NMM**: a) total ion chromatogram b) ion series c) deconvoluted spectrum d) zoom on the major peaks; calculated masses: ((DHAR1 S97C)–S–NMM): 26087 Da ((DHAR1 S97C)–(S–NMM)$_2$): 26198 Da, ((DHAR1 S97C)–S–NMM with N-terminal Ac-Met): 26262, ((DHAR1 S97C)–(S–NMM)$_2$ with N-terminal Ac-Met): 26373 Da; observed masses: 26375 Da (starting material), 26084 Da, 26261 Da. The spectra were recorded with a sample quenched with equimolar β-mercaptoethanol 30 minutes after addition of **NMM** to simulate the dilution into the DHAR enzyme assay.

Table S7. Different amounts of **NMM** solutions added to the reaction mixture

reaction	V(NMM stock) / µL	n(NMM) / nmol	m(NMM) / µg	equivalents
1	1.0	10	1.1	3.1
2	1.5	15	1.7	4.7
3	2	20	2.2	6.3
4	2.5	25	2.8	7.8

Screening of different maleimide concentration in the conjugation with DHAR1 S176C

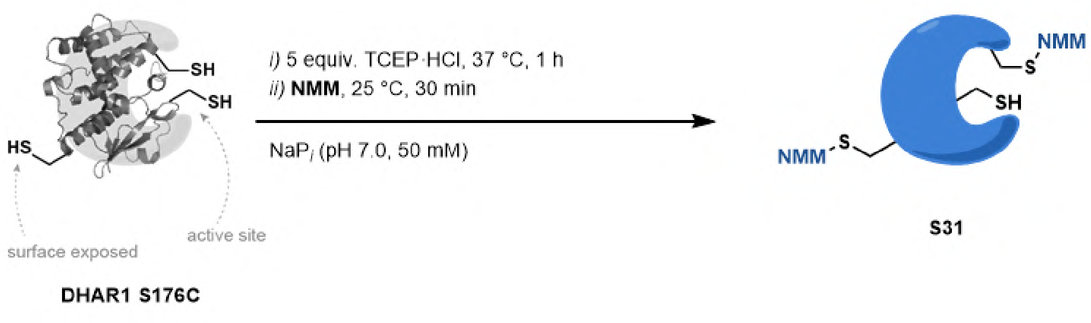

At 20–25 °C, 68 µL of DHAR1 S176C (1.22 mg/mL, 47.0 µM, 3.2 nmol, 1.0 equiv.) in NaP$_i$ buffer (pH 7.0, c = 50 mM) was added to a 1.5 mL Eppendorf tube. Next, 131 µL NaP$_i$ buffer (pH 7.0, c = 50 mM) was added. Then, 1.5 µL of a TCEP stock solution (10 mM, 15 nmol, 3.8 µg, 4.7 equiv.) in UHQ-H_2O was added. The mixture was vortexed, transferred into a Thermocycler pre-heated at 37 °C, and incubated at 37 °C for 1 hour at 400 rpm. Next, varying volumes (see Table S8) of a 10 mM **NMM** stock solution in DMF were added to the mixture. The reaction mixture was vortexed, transferred into a Thermocycler pre-heated at 25 °C, and incubated at 25 °C at 400 rpm for 30 minutes. After 30 minutes the reaction mixture was utilized for the DHAR1 enzyme assay.

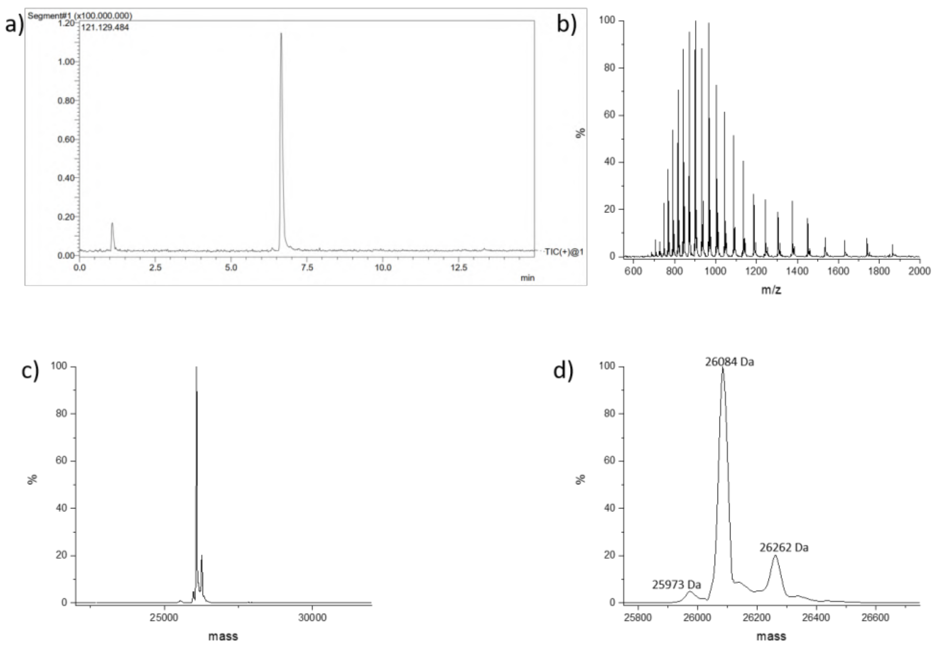

Figure S146. LC-MS analysis (Method A) of DHAR1 S176C after reaction with 3.1 equiv. **NMM**: a) total ion chromatogram b) ion series c) deconvoluted spectrum d) zoom on the major peaks; calculated masses: ((DHAR1 S176C)–S–NMM): 26085 Da ((DHAR1 S176C)–(S–NMM)$_2$): 26196 Da, ((DHAR1 S176C)–S–NMM with N-terminal Ac-Met): 26263, ((DHAR1 S176C)–(S–NMM)$_2$ with N-terminal Ac-Met): 26374 Da; observed masses: 25973 Da (starting material), 26084 Da, 26262 Da. The spectra were recorded with a sample quenched with equimolar β-mercaptoethanol 30 minutes after addition of **NMM** to simulate the dilution into the DHAR enzyme assay.

Table S8. Different amounts of NMM solutions added to the reaction mixture

reaction	V(NMM stock) / µL	n(NMM) / nmol	m(NMM) / µg	equivalents
1	1.0	10	1.1	3.1
2	1.5	15	1.7	4.7
3	2	20	2.2	6.3
4	2.5	25	2.8	7.8

DHAR-activity assays

DHAR enzyme activity was measured by monitoring ascorbate absorbance by spectrophotometry at 265 nm (Cary 3500 UV-Vis, Agilent, Santa Clara, CA, USA, software: Cary UV Workstation). The assay was performed in quartz cuvettes with a 100 µl reaction mixture containing 2 mM GSH and 0.5 mM DHA in 50 mM Na-Pi, pH 7 at 25 °C. The reaction mixture was equilibrated for 3 min, the baseline recorded, the reaction initiated by the addition of 5 nM DHAR and the absorption change monitored at 265 nm. Subsequent to the addition of DHAR, the slope of the initial linear decrease in Absorption was obtained by linear Regression analysis. Based on the slope, enzyme activities were calculated using an ε-value for ascorbate of 14,000 $M^{-1}cm^{-1}$. Values were corrected for spontaneous DHA reduction in absence of DHAR.

Screening of different NMM concentrations

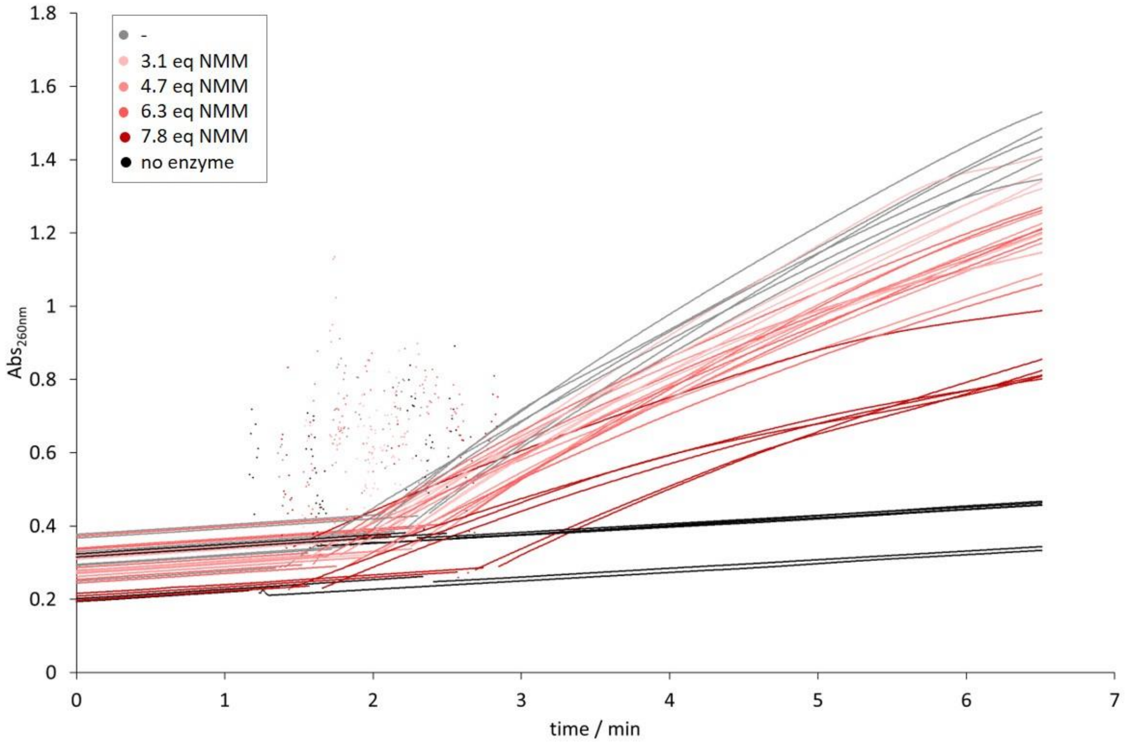

Figure S147. A260 measurements for DHAR1 S97C with varying equivalents of **NMM**.

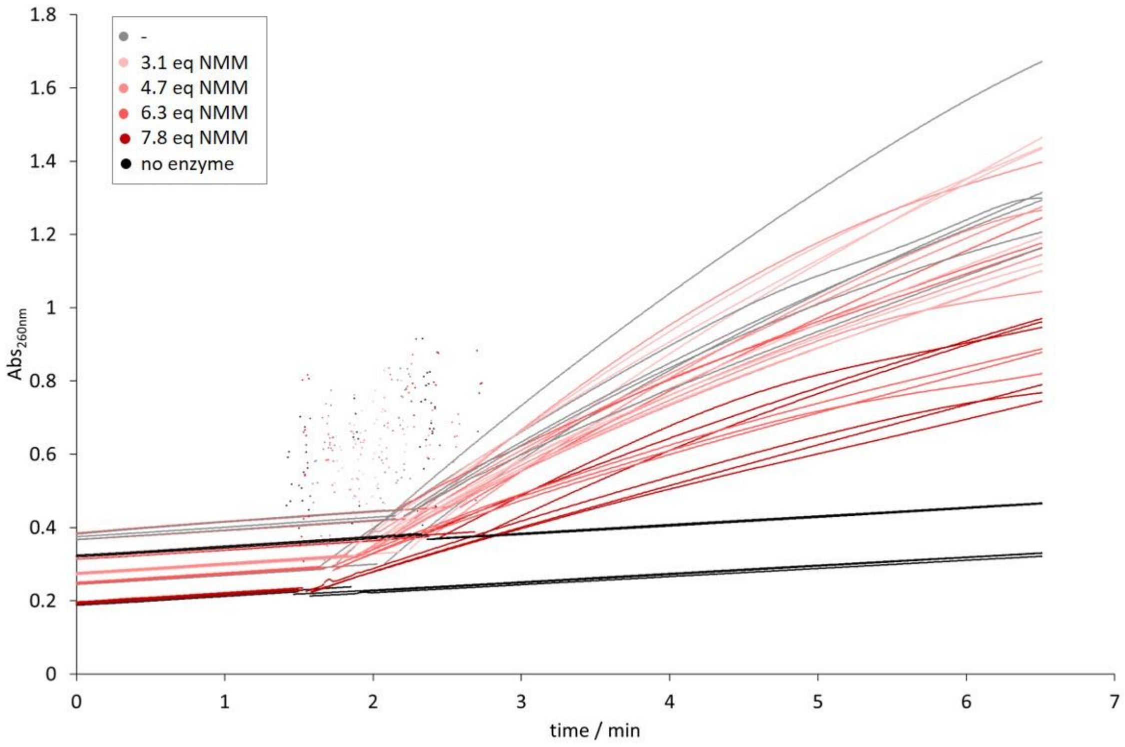

Figure S148. A260 measurements for DHAR1 S176C with varying equivalents of **NMM**.

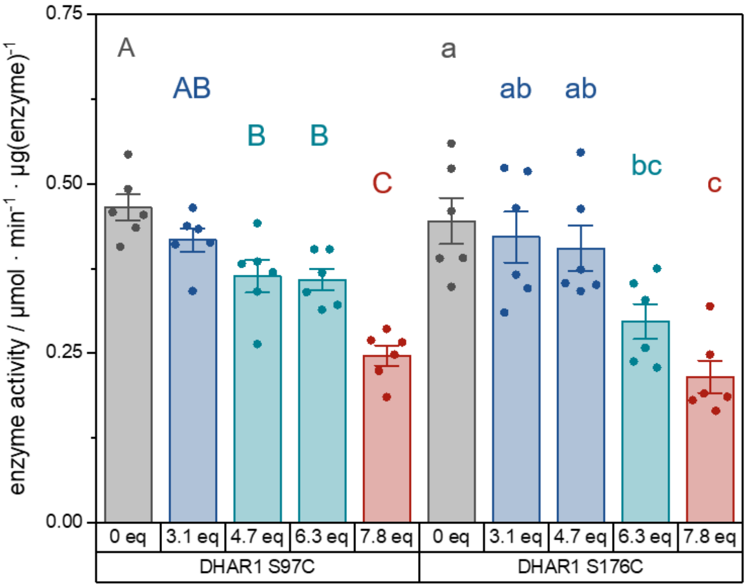

Figure S149. Enzyme activities measured for the DHAR1 mutants S97C and S176C after treatment with varying equivalents of **NMM**, respectively. Data are means ± SE of n = 6 independent experiments. The significance of difference was determined using a two-sided ANOVA, followed by post hoc Tukey test with $p < 0.05$ and is marked with letters a, b, and c for DHAR1 S97C and A, B, and C for DHAR1 S176C.

Table S9. Multiple comparisons for the two-sided ANOVA testing of Enzyme activities measured for the DHAR1 mutant S97C after treatment with varying equivalents of NMM. Data are means ± (standard error) SE of n = 6 independent experiments. The significance of difference was determined using ANOVA, followed by post hoc Tukey test with $p < 0.05$.

Conditions		Significance
0 eq	3.1 eq	0.3789679
	4.7 eq	0.0060021
	6.3 eq	0.0036469
	7.8 eq	0.0000001
3.1 eq	0 eq	0.3789679
	4.7 eq	0.2874535
	6.3 eq	0.2057083
	7.8 eq	0.0000082
4.7 eq	0 eq	0.0060021
	3.1 eq	0.2874535
	6.3 eq	0.9996007
	7.8 eq	0.0013094
6.3 eq	0 eq	0.0036469
	3.1 eq	0.2057083
	4.7 eq	0.9996007
	7.8 eq	0.0021770
7.8 eq	0 eq	0.0000001
	3.1 eq	0.0000082
	4.7 eq	0.0013094
	6.3 eq	0.0021770

Table S10. Multiple comparisons for the two-sided ANOVA testing of Enzyme activities measured for the DHAR1 mutant S176C after treatment with varying equivalents of NMM. Data are means ± SE of n = 6 independent experiments. The significance of difference was determined using ANOVA, followed by post hoc Tukey test with $p < 0.05$.

Conditions		Significance
0 eq	3.1 eq	0.9829806
	4.7 eq	0.8919793
	6.3 eq	0.0206692
	7.8 eq	0.0002142
3.1 eq	0 eq	0.9829806
	4.7 eq	0.9956293
	6.3 eq	0.0674269
	7.8 eq	0.0008298
4.7 eq	0 eq	0.8919793
	3.1 eq	0.9956293
	6.3 eq	0.1417699
	7.8 eq	0.0021230
6.3 eq	0 eq	0.0206692
	3.1 eq	0.0674269
	4.7 eq	0.1417699
	7.8 eq	0.3730070
7.8 eq	0 eq	0.0002142
	3.1 eq	0.0008298
	4.7 eq	0.0021230
	6.3 eq	0.3730070

Activity comparison after functionalization with NMM or VTT + NaN_3

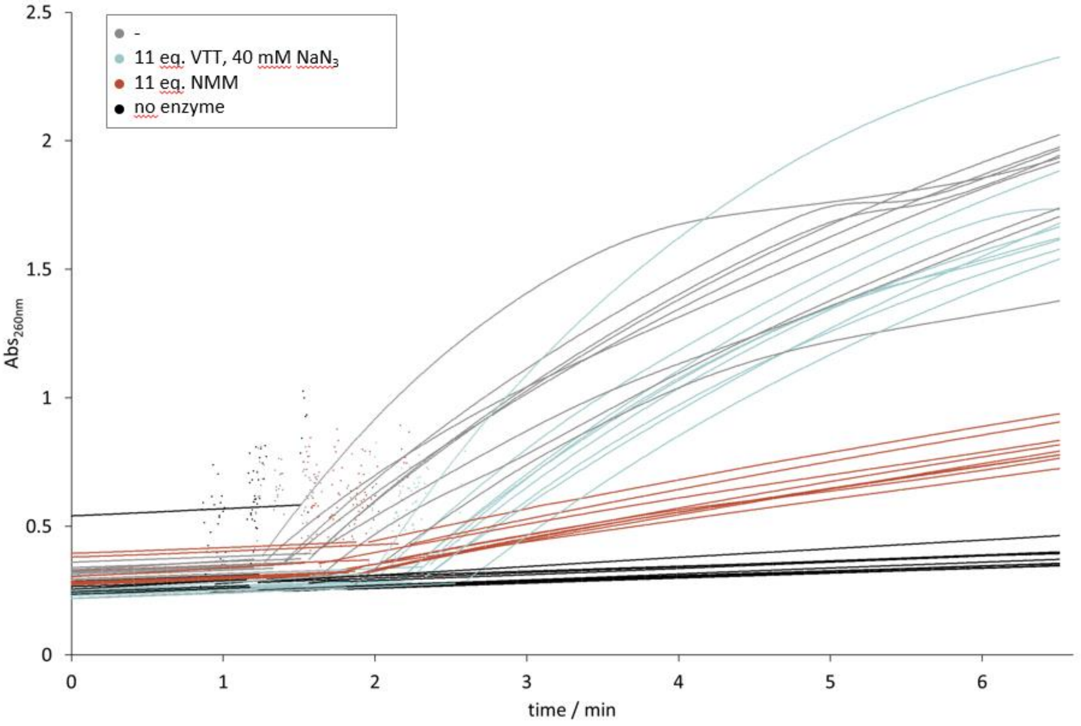

Figure S150. A_{260} measurements for DHAR1 S97C with 11 equiv. **VTT** and 40 mM NaN_3, 11 equiv. **NMM**, unmodified DHAR1 S97C, and a negative control.

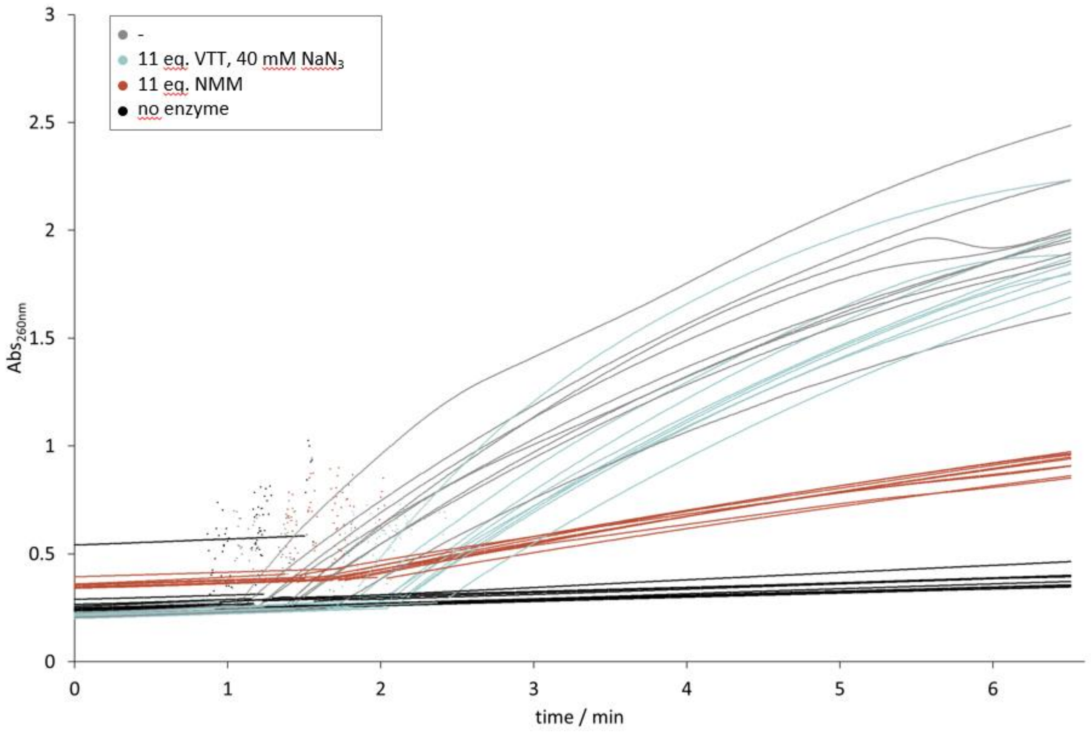

Figure S151. A_{260} measurements for DHAR1 S176C with 11 equiv. **VTT** and 40 mM NaN_3, 11 equiv. **NMM**, unmodified DHAR1 S176C, and a negative control.

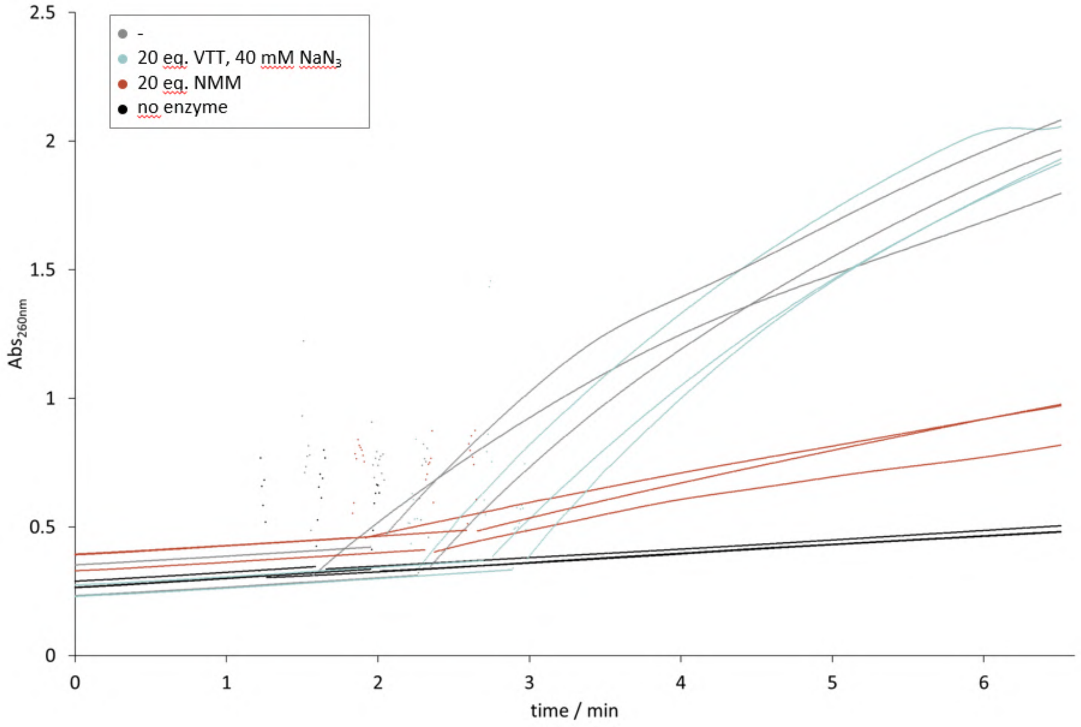

Figure S152. A_{260} measurements for DHAR1 S97C with 20 equiv. **VTT** and 40 mM NaN_3, 20 equiv. **NMM**, unmodified DHAR1 S97C, and a negative control.

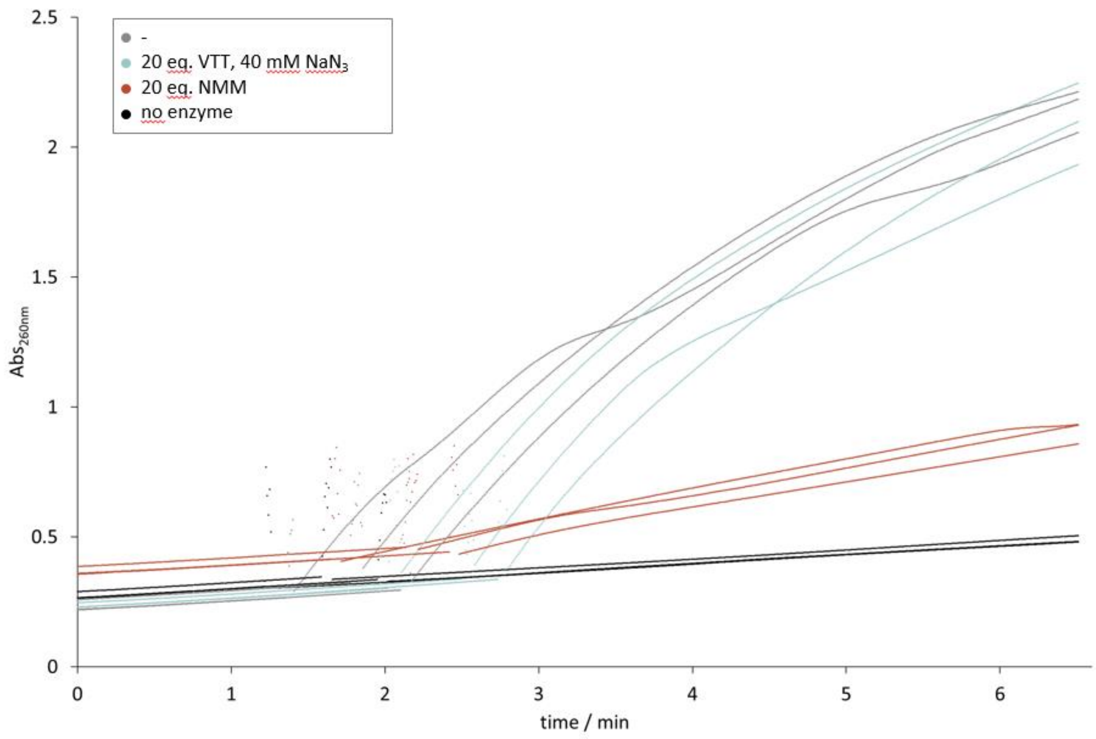

Figure S153. A260 measurements for DHAR1 S176C with 20 equiv. **VTT** and 40 mM NaN_3, 20 equiv. **NMM**, unmodified DHAR1 S97C, and a negative control.

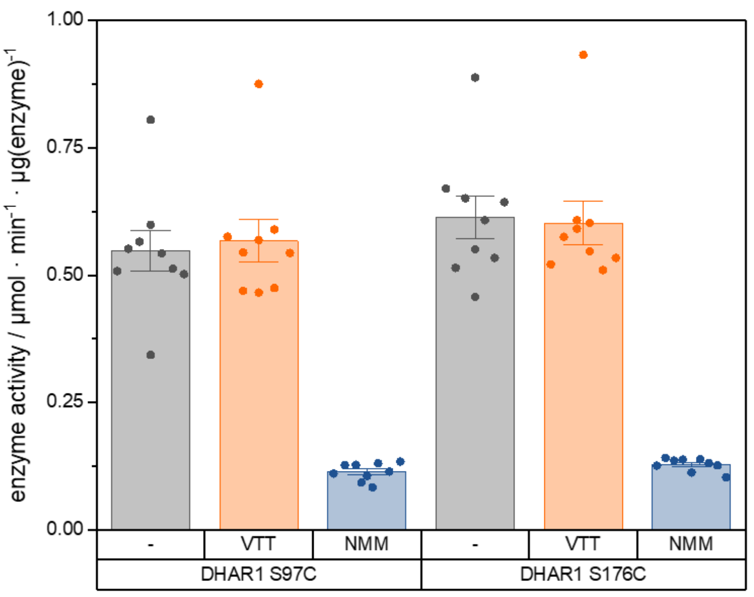

Figure S154. Enzyme activity of DHAR1 mutants after reaction with either 11 equiv. of **VTT** and 40 mM NaN_3 or 11 equiv. **NMM**, respectively. Data are means ± SE of n = 9 independent experiments. The significance of difference was determined using a two-sided ANOVA, followed by post hoc Tukey test with $p < 0.05$ and is marked with letters a and b for DHAR1 S176C and A and B for DHAR1 S97C.

Table S11. Multiple comparisons for the ANOVA testing of Enzyme activities measured for the DHAR1 mutant S97C after reaction with either 11 equiv. of **VTT** and 40 mM NaN_3 or 11 equiv. **NMM**, respectively. Data are means ± (standard error) SE of n = 9 independent experiments. The significance of difference was determined using ANOVA, followed by post hoc Tukey test with $p < 0.05$.

Conditions		Significance
-	VTT	0.91009
	NMM	0.00000
VTT	-	0.91009
	NMM	0.00000
NMM	-	0.00000
	VTT	0.00000

Table S12. Multiple comparisons for the two-sided ANOVA testing of Enzyme activities measured for the DHAR1 mutant S176C after reaction with either 11 equiv. of **VTT** and 40 mM NaN_3 or 11 equiv. **NMM**, respectively. Data are means ± (standard error) SE of n = 9 independent experiments. The significance of difference was determined using ANOVA, followed by post hoc Tukey test with $p < 0.05$.

Conditions		Significance
-	VTT	0.97434
	NMM	0.00000
VTT	-	0.97434
	NMM	0.00000
NMM	-	0.00000
	VTT	0.00000

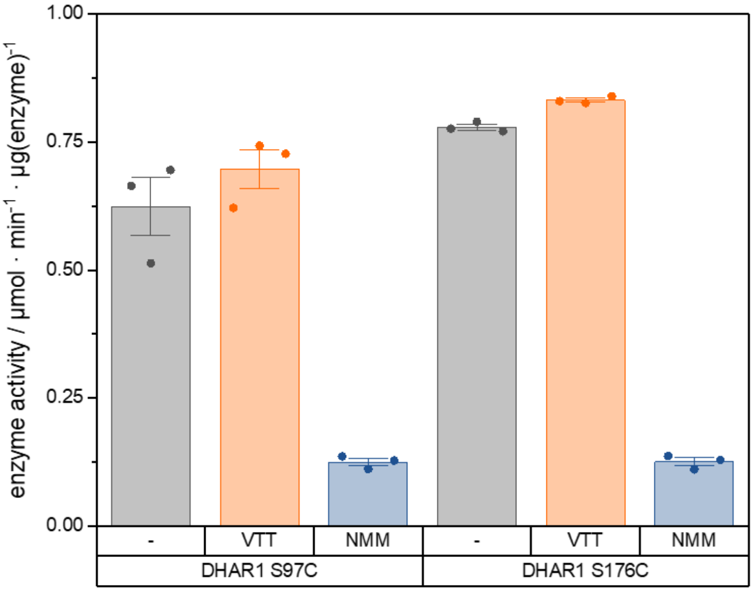

Figure S155. Enzyme activity of DHAR1 mutants after reaction with either 20 equiv. of **VTT** and 40 mM NaN_3 or 20 equiv. **NMM**, respectively. Data are means ± SE of n = 3 independent experiments. The significance of difference was determined using a two-sided ANOVA, followed by post hoc Tukey test with $p < 0.05$ and is marked with letters a, b, and c for DHAR1 S176C and A and B for DHAR1 S97C.

Table S13. Multiple comparisons for the two-sided ANOVA testing of Enzyme activities measured for the DHAR1 mutant S97C after reaction with either 20 equiv. of **VTT** and 40 mM NaN_3 or 20 equiv. **NMM**, respectively. Data are means ± (standard error) SE of n = 3 independent experiments. The significance of difference was determined using ANOVA, followed by post hoc Tukey test with $p < 0.05$.

Conditions		Significance
-	VTT	0.44391
	NMM	0.00027
VTT	-	0.44391
	NMM	0.00012
NMM	-	0.00027
	VTT	0.00012

Table S14. Multiple comparisons for the two-sided ANOVA testing of Enzyme activities measured for the DHAR1 mutant S176C after reaction with either 20 equiv. of **VTT** and 40 mM NaN_3 or 20 equiv. **NMM**, respectively. Data are means ± SE of n = 3 independent experiments. The significance of difference was determined using ANOVA, followed by post hoc Tukey test with $p < 0.05$.

Conditions		Significance
-	VTT	0.00190
	NMM	0.00000
VTT	-	0.00190
	NMM	0.00000
NMM	-	0.00000
	VTT	0.00000

DHAR1 activity after modification with VTT without NaN_3

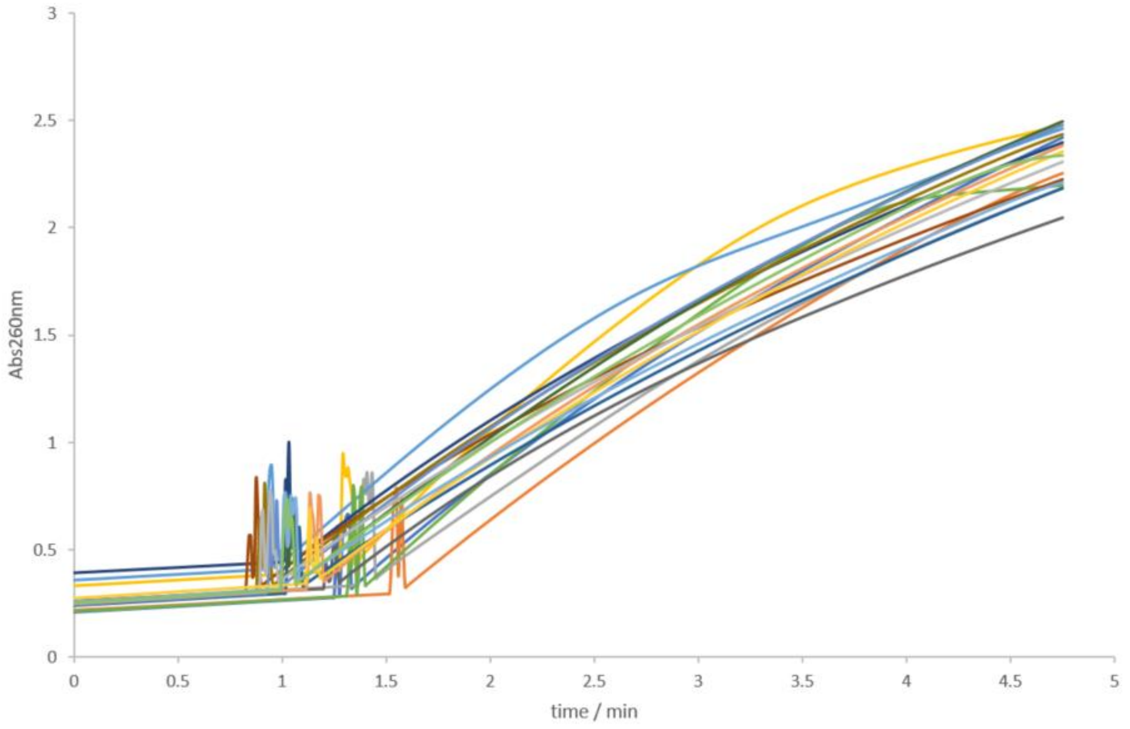

Figure S156. A_{260} measurements for DHAR1 S97C functionalized with 11 equiv. **VTT** in absence of exogenous nucleophile.

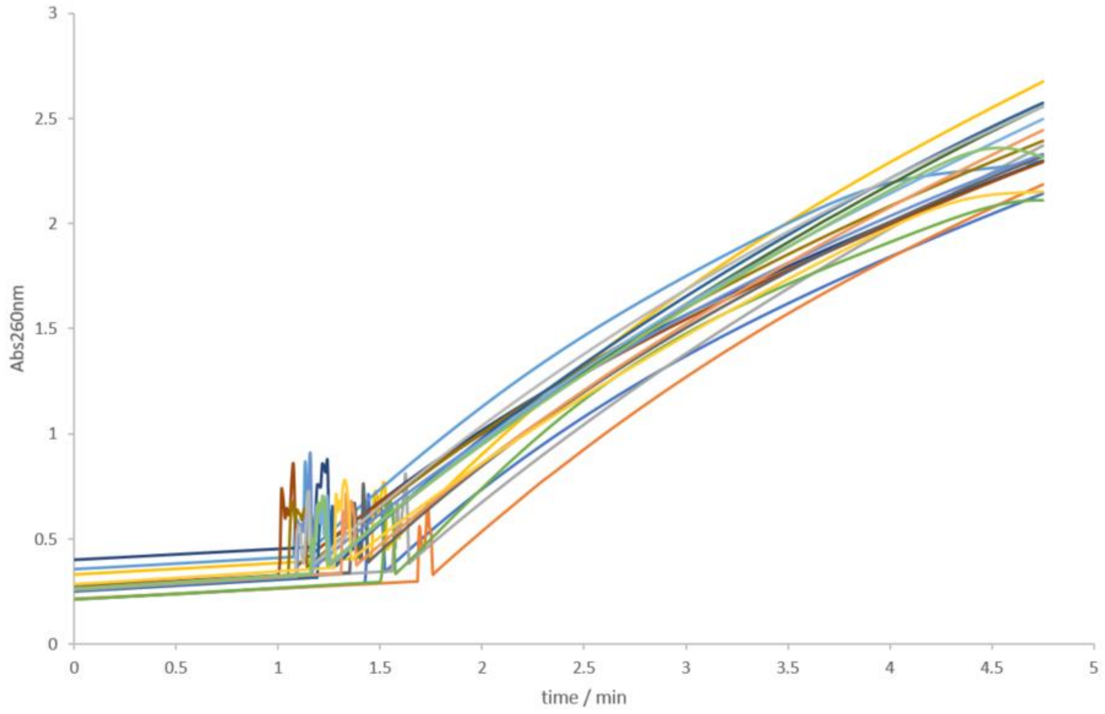

Figure S157. A_{260} measurements for DHAR1 S176C functionalized with 11 equiv. **VTT** in absence of exogenous nucleophile.

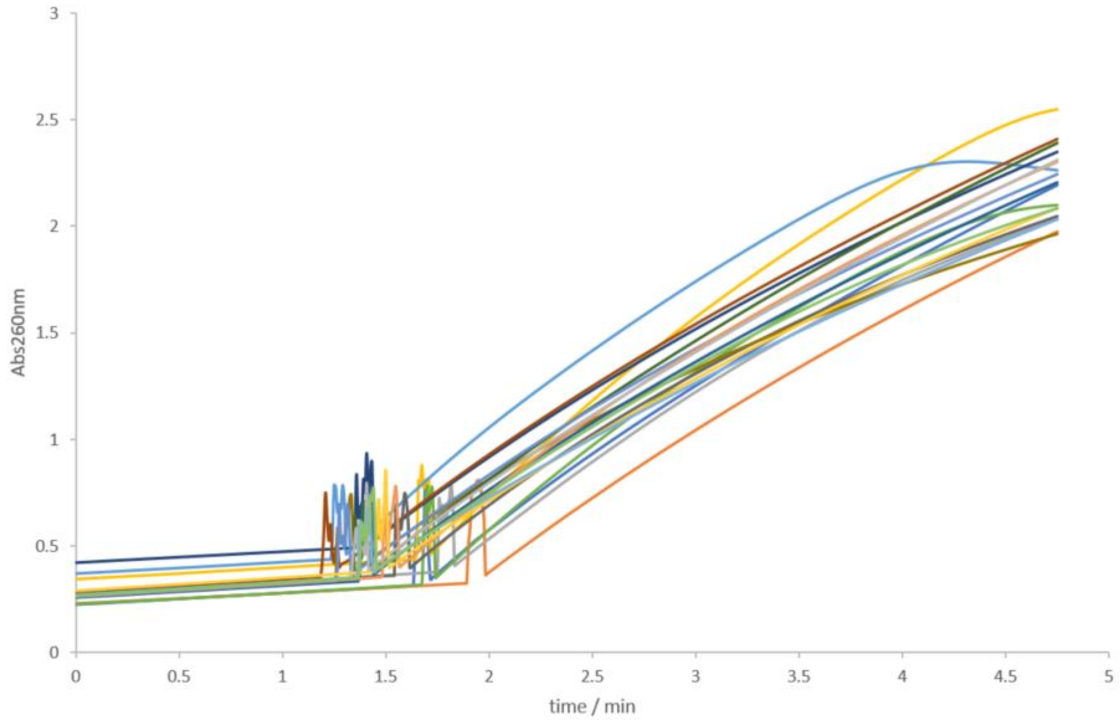

Figure S158. A_{260} measurements for DHAR1 S97C functionalized with 20 equiv. **VTT** in absence of exogenous nucleophile.

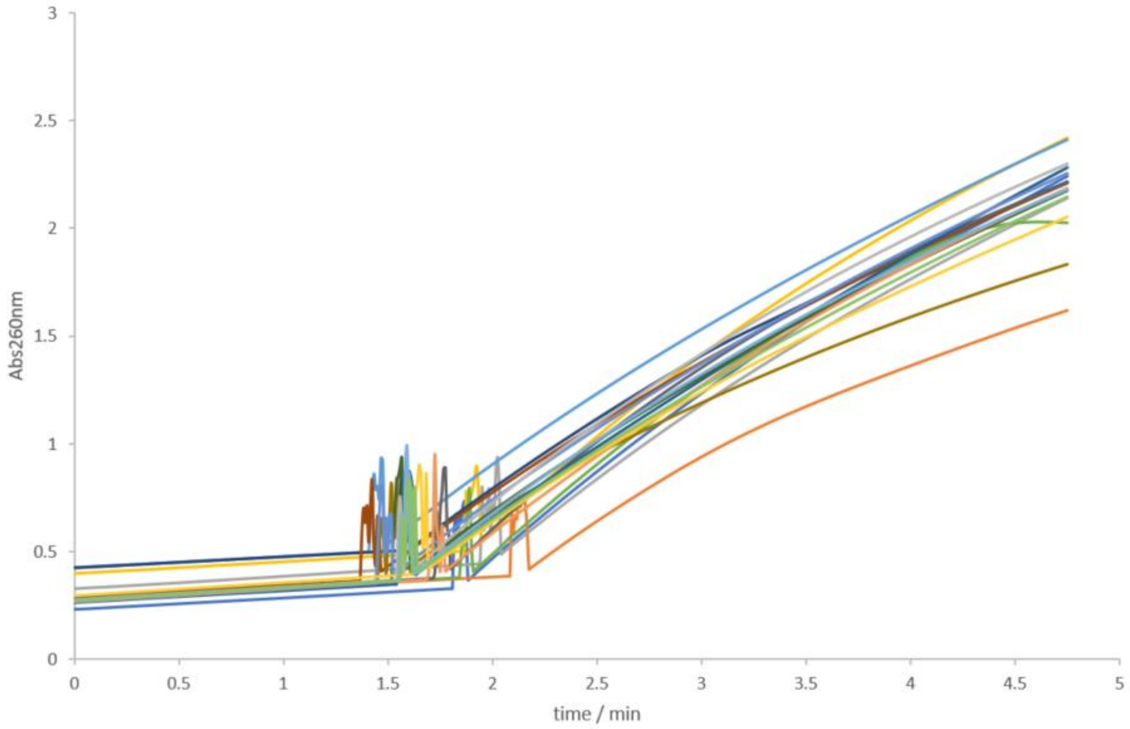

Figure S159. A_{260} measurements for DHAR1 S176C functionalized with 20 equiv. **VTT** in absence of exogenous nucleophile.

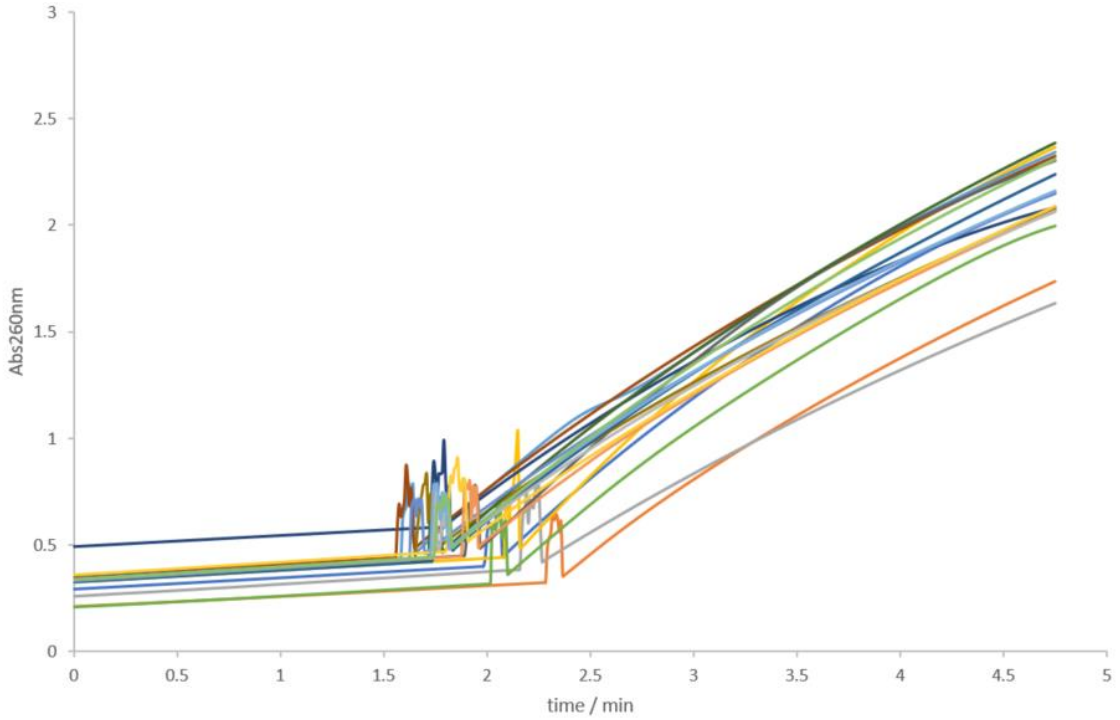

Figure S160. A_{260} measurements for unmodified DHAR1 S97C.

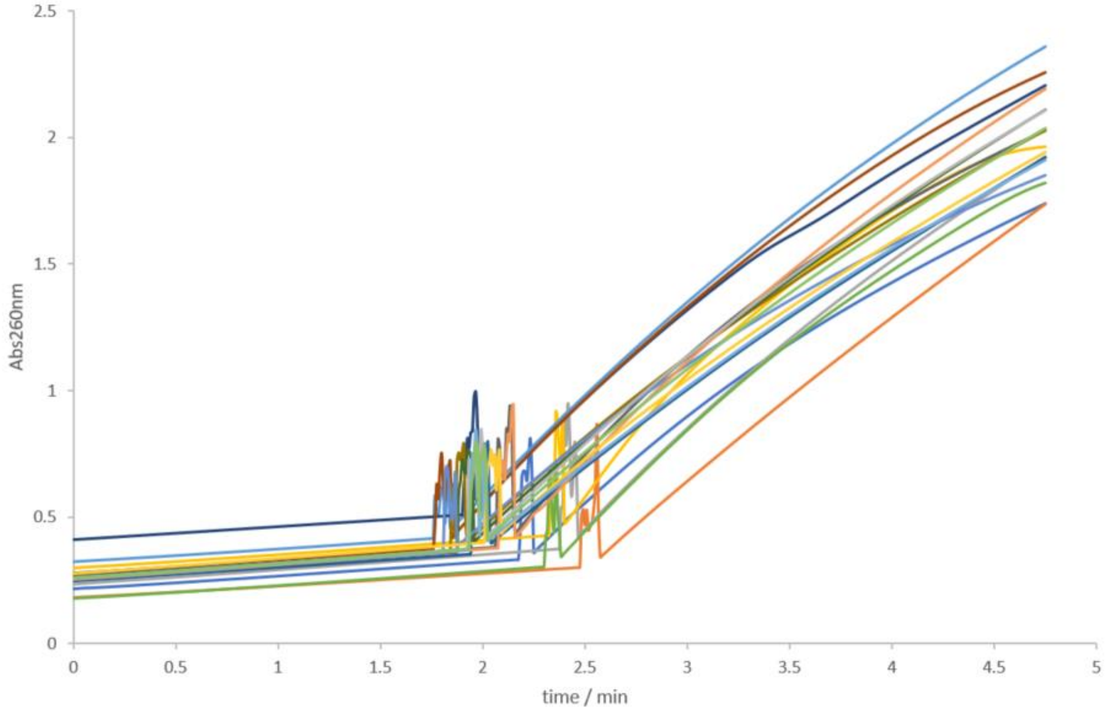

Figure S161. A_{260} measurements for unmodified DHAR1 S176C.

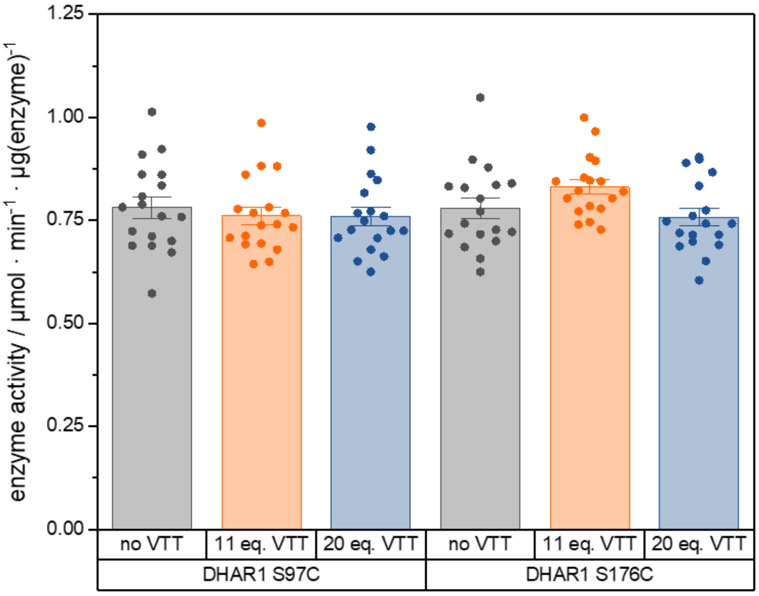

Figure S162. Enzyme activity of DHAR1 mutants before and after reaction with 11 or 20 eq. **VTT** in the absence of exogenous nucleophile. Data are means ± SE of n = 18 independent experiments. The significance of difference was determined using two-sided ANOVA.

Table S15. Multiple comparisons for the two-sided ANOVA testing of Enzyme activities measured for the DHAR1 mutant S176C after reaction with either no, 11 equiv., or 20 equiv. of **VTT**, respectively. Data are means ± (standard error) SE of n = 18 independent experiments. The significance of difference was determined using ANOVA, followed by post hoc Tukey test with $p < 0.05$.

Conditions		Significance
	11 eq. VTT DHAR1 S176C	0.228

11 eq. VTT DHAR1 S97C	20 eq. VTT DHAR1 S97C	1.000
	20 eq. VTT DHAR1 S176C	1.000
	no VTT DHAR1 S97C	0.987
	no VTT DHAR1 S176C	0.991
11 eq. VTT DHAR1 S176C	11 eq. VTT DHAR1 S97C	0.228
	20 eq. VTT DHAR1 S97C	0.216
	20 eq. VTT DHAR1 S176C	0.187
	no VTT DHAR1 S97C	0.601
	no VTT DHAR1 S176C	0.569
20 eq. VTT DHAR1 S97C	11 eq. VTT DHAR1 S97C	1.000
	11 eq. VTT DHAR1 S176C	0.216
	20 eq. VTT DHAR1 S176C	1.000
	no VTT DHAR1 S97C	0.985
	no VTT DHAR1 S176C	0.989
20 eq. VTT DHAR1 S176C	11 eq. VTT DHAR1 S97C	1.000
	11 eq. VTT DHAR1 S176C	0.187
	20 eq. VTT DHAR1 S97C	1.000
	no VTT DHAR1 S97C	0.976
	no VTT DHAR1 S176C	0.982
no VTT DHAR1 S97C	11 eq. VTT DHAR1 S97C	0.987
	11 eq. VTT DHAR1 S176C	0.601
	20 eq. VTT DHAR1 S97C	0.985
	20 eq. VTT DHAR1 S176C	0.976
	no VTT DHAR1 S176C	1.000
no VTT DHAR1 S176C	11 eq. VTT DHAR1 S97C	0.991
	11 eq. VTT DHAR1 S176C	0.569
	20 eq. VTT DHAR1 S97C	0.989
	20 eq. VTT DHAR1 S176C	0.982
	no VTT DHAR1 S97C	1.000

sfGFP S147C fluorescence assay

Four samples with different concentrations of either modified or unmodified sfGFP S147C were prepared in NaP$_i$ buffer (pH 7.0, 50 mM,). The absorbance of each sample at 475 nm was determined via NanoDrop. Then, the fluorescence intensity of each sample was measured on a Kontron SMF 25 at λ_{ex} = 475 nm. The maximum fluorescence intensity was plotted against absorbance of the samples at 475 nm and the data was fitted with linear regression. The gradients of the obtained plots were used to calculate relative quantum yield using 0.65 as a reference value for the quantum yield of sfGFP[27].

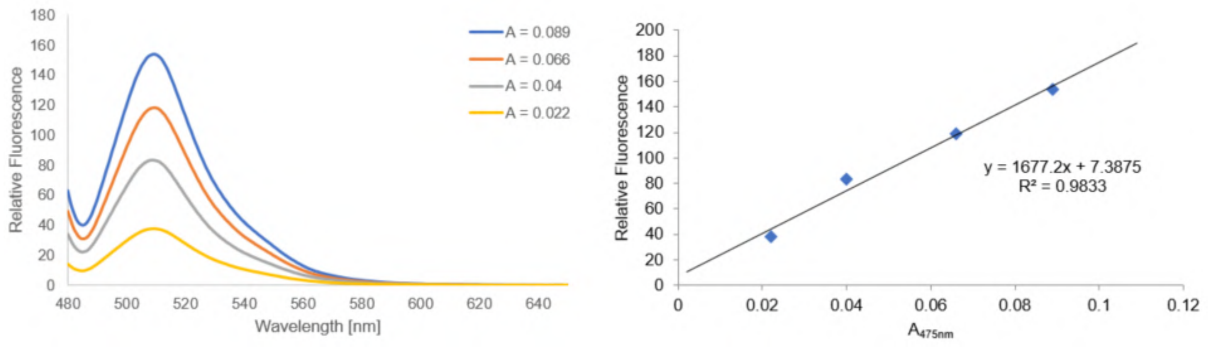

Figure S163. Dependence of fluorescence intensity on the absorbance (A) of samples containing different concentrations of sfGFP S147C, which was functionalized with **VTT** in NaP*i*

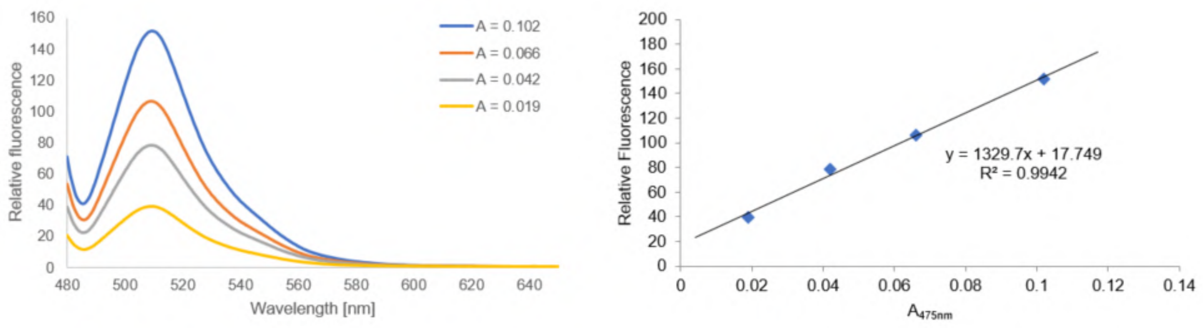

Figure S164. Dependence of fluorescence intensity on the absorbance (A) of samples containing different concentrations of sfGFP S147C, which was functionalized with **VTT** in NaP*i*

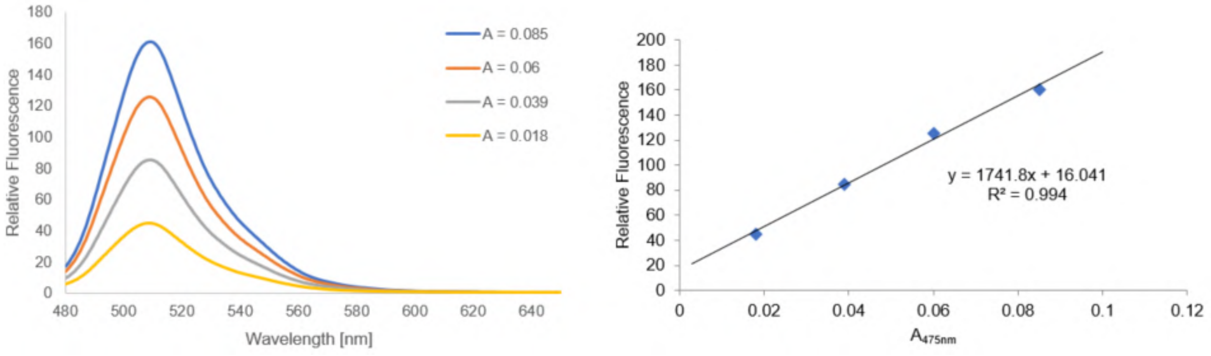

Figure S165. Dependence of fluorescence intensity on the absorbance (A) of samples containing different concentrations of unmodified sfGFP S147C

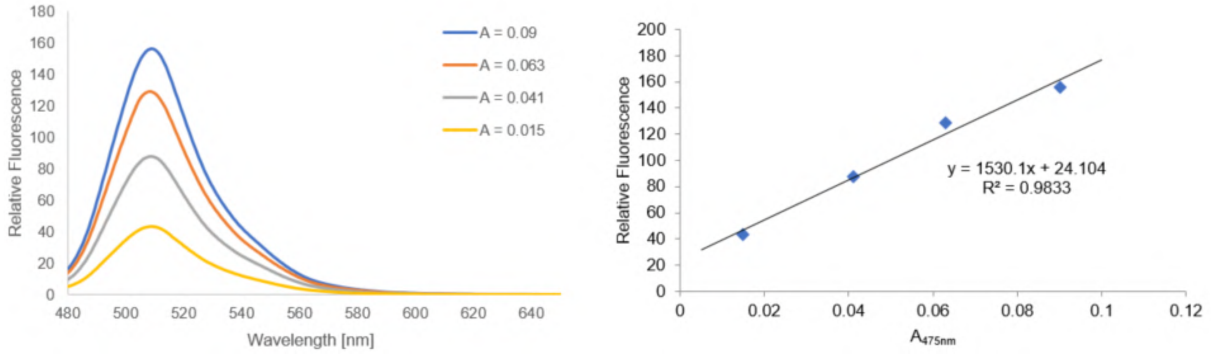

Figure S166. Dependance of fluorescence intensity on the absorbance (A) of samples containing different concentrations of unmodified sfGFP S147C

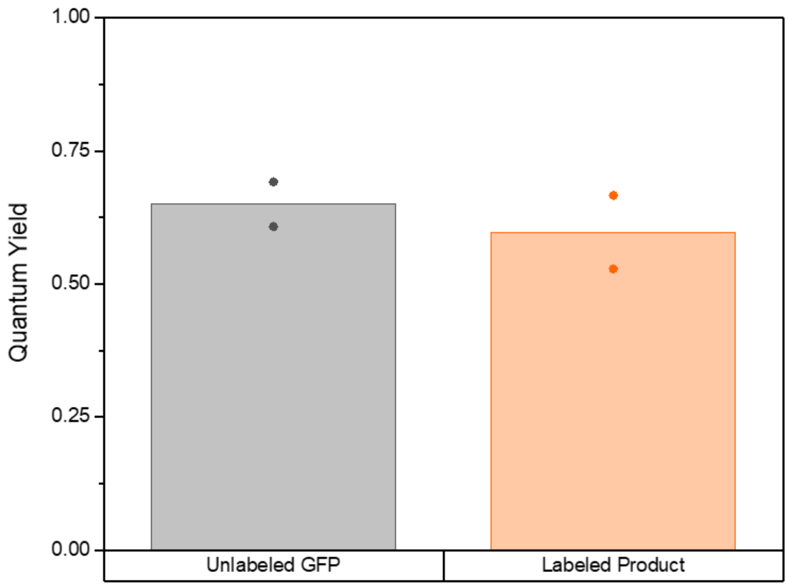

Figure S167. Calculated quantum yield for sfGFP S147C, which was functionalized with **VTT** in NaP*i* (labeled product), and unmodified sfGFP S147C (unlabeled GFP). Data are means of n = 2 independent experiments. A two-sided T-test was conducted to evaluate whether the two species are significantly different to each other. The exact p-value was determined as p = 0.93 which indicates no significant statistical difference between the two populations.

Mechanistic studies

UV-vis Absorption Measurements

All UV-vis measurements were recorded on a Shimadzu UV-vis Spectrophotometer UV-2600 with temperature controller Shimadzu S-1700 at (25 ± 0.1) °C unless stated otherwise. To monitor the reaction progress of glutathione (**GSH**) functionalization with **VTT** and sodium azide, the absorbance of thianthrene (**TT**) at 255 nm and the absorbance of **VTT** at 307 nm were measured over time.

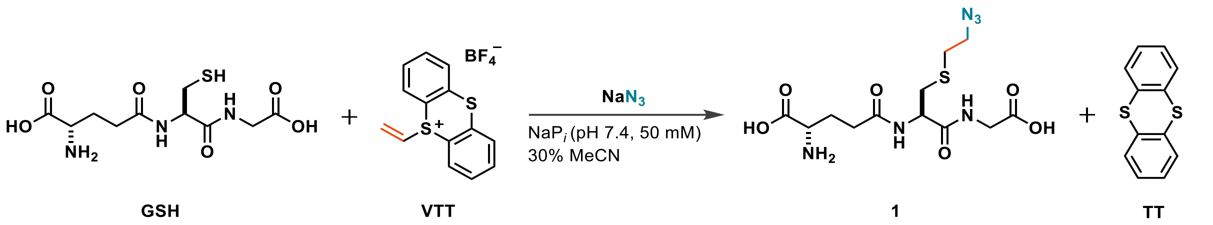

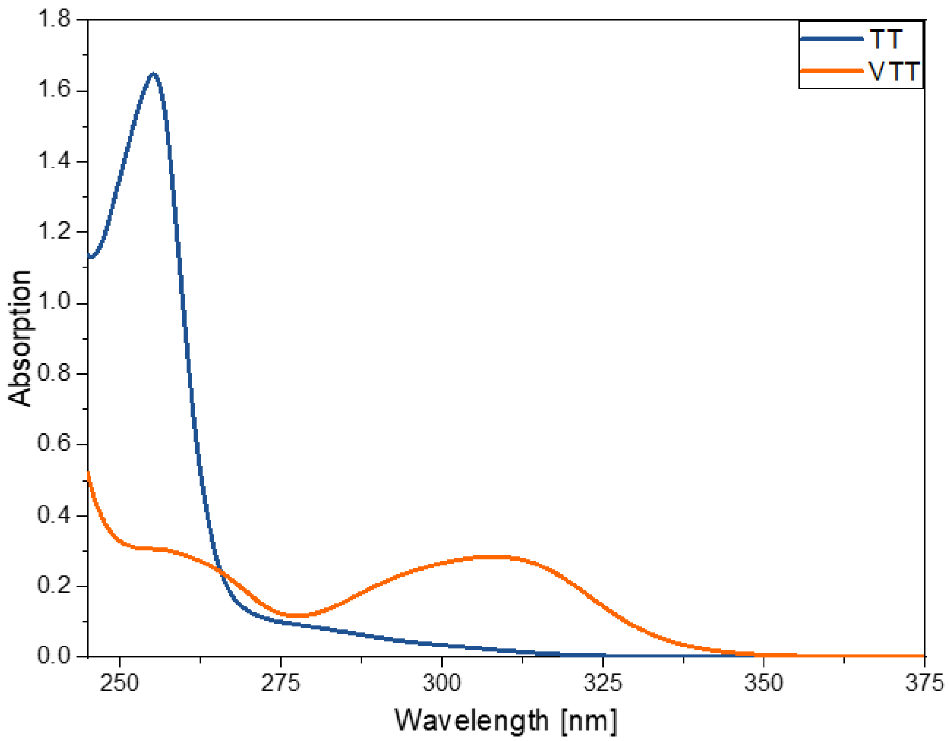

Figure S168. Absorbance spectra of 50 µM **TT** and **VTT** aqueous solutions containing 30% MeCN.

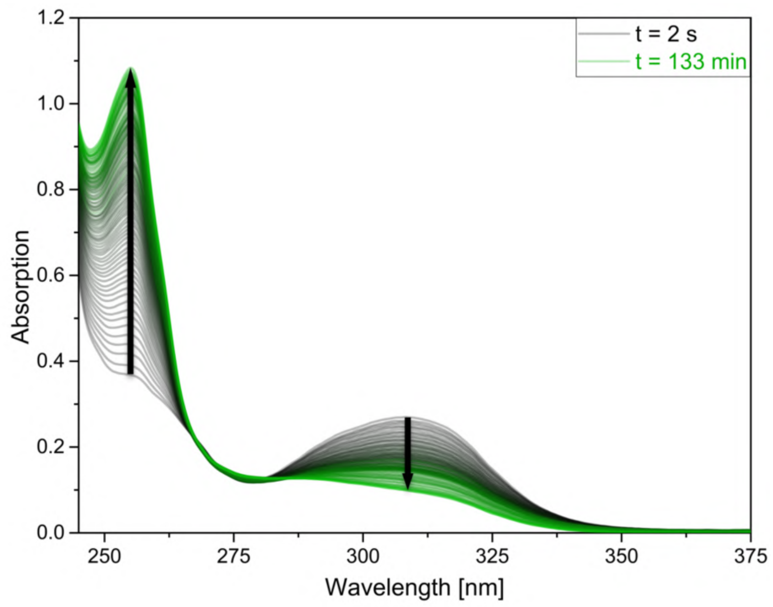

Figure S169. Absorption change for the reaction of 40 µM **GSH** with 50 µM **VTT** and 1.5 mM NaN_3 in sodium phosphate buffer (pH 7.4, 50 mM) containing 30% MeCN. The spectra were obtained with a NanoDrop One C at (37 ± 0.1) °C.

Kinetic Reaction Profile for VTT

The kinetic profile of glutathione functionalization with **VTT** and sodium azide was investigated using Variable Time Normalization Analysis (VTNA)[28]. The experiments were performed with an excess of **VTT** and NaN_3. Because **GSH** and **VTT** react in the 1:1 stoichiometric ratio to produce 1 equiv. of **TT** under these conditions, the concentration of **TT** can be used to determine the concentrations of **GSH** and **VTT** at any point in time. The reaction mixtures contained 30% MeCN to ensure the solubility of **TT** in the reaction mixture.

General procedure: Stock solutions of **GSH** (1.85 mM) and NaN_3 (17.8 mM) were prepared in sodium phosphate buffer (pH 7.4, c = 71 mM). NaP_i (pH 7.4, c = 71 mM), MeCN, NaN_3, and **GSH** stock solutions were pipetted into a quartz cuvette charged with a Teflon-coated magnetic stirring bar. The cuvette was thermostated for 5 minutes at (25 ± 0.1) °C in the cell holder. After performing the background measurement, 10 µL of a **VTT** stock solution in DMF was added to the cuvette. The obtained buffered mixture (V = 2.0 mL; 50 mM NaP_i; 30% v/v MeCN) contained 10 – 40 µM of **GSH**, 12.5 – 50 µM of **VTT**, and 0.5 – 4.5 mM of NaN_3. The reaction mixture was homogenized by pipetting the mixture up and down. Then, the cuvette was capped and the release of **TT** was monitored by measuring absorbance at 255 nm while stirring.

Reaction order in GSH

To determine the reaction order in **GSH**, three experiments were carried out with c(**GSH**) set to 10 µM, 20 µM, and 40 µM, keeping the concentrations of the other reagents constant: c(**VTT**) = 50 µM and c(NaN_3) = 1.5 mM. The concentration of **TT** was plotted against $\Sigma[\mathbf{GSH}]^{\beta}\bullet\Delta t$, generating graphs for different values of β (0, 0.5, 1.0, and 2.0) according to the Burés' method[28]. The graphs overlay for β = 1.0, suggesting that the reaction is first order in **GSH**.

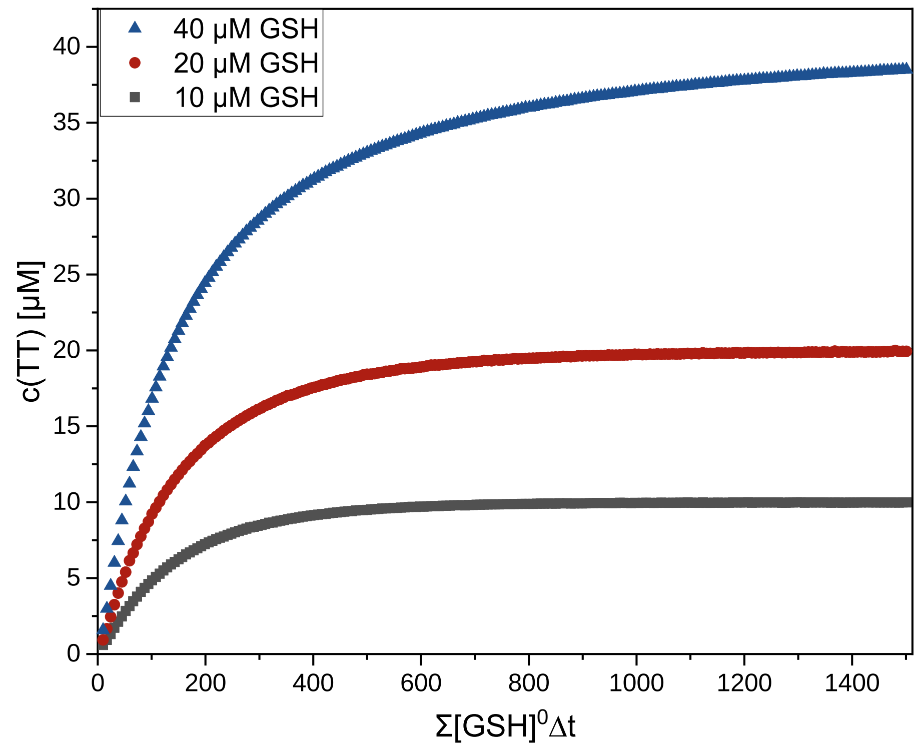

Figure S170. VTNA analysis for β = 0.

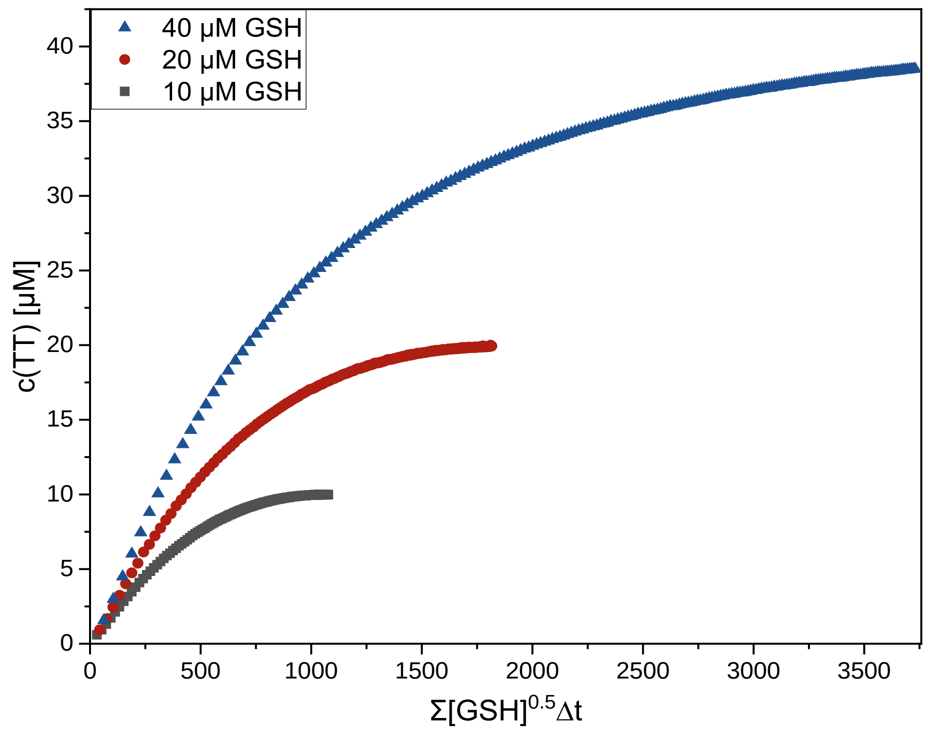

Figure S171. VTNA analysis for β = 0.5.

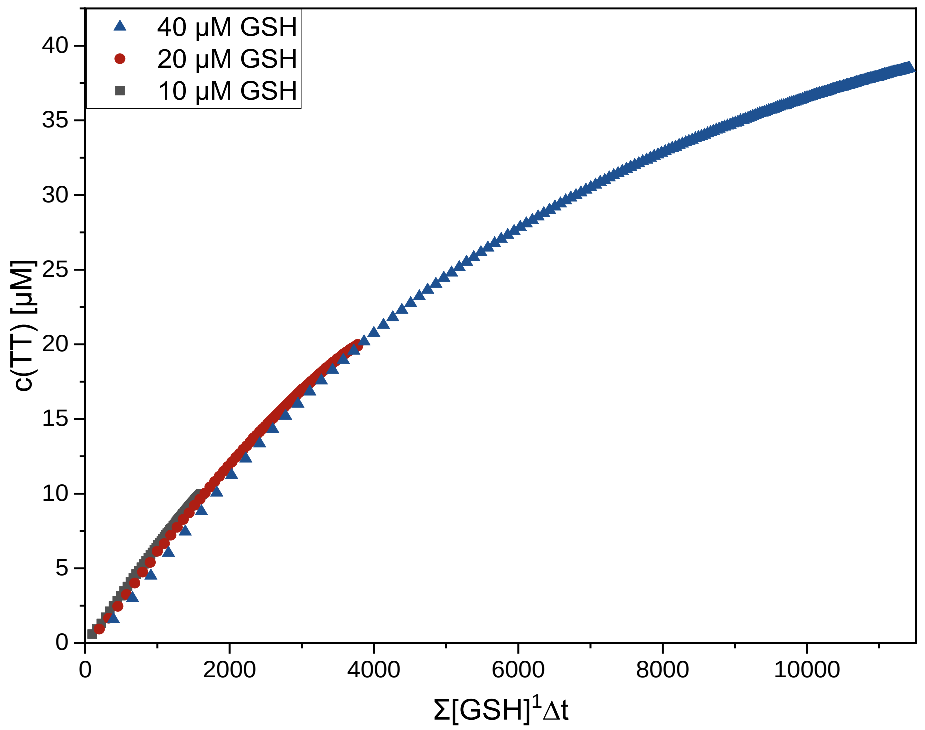

Figure S172. VTNA analysis for β = 1.0.

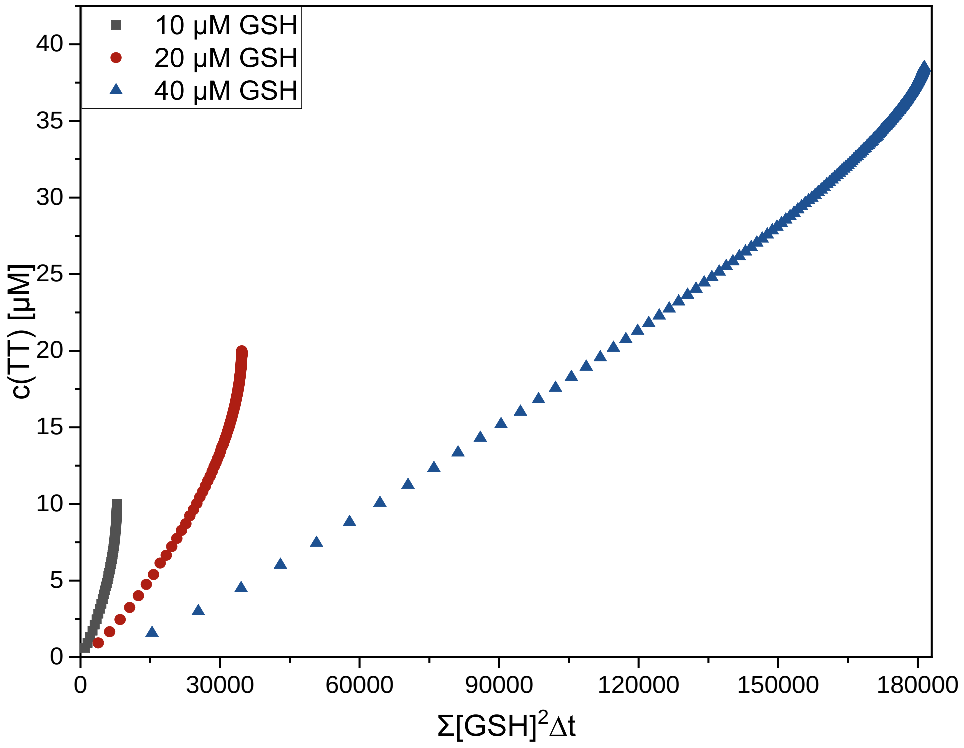

Figure S173. VTNA analysis for β = 2.0.

Reaction order in VTT

To determine the reaction order in **VTT**, c(**VTT**) was set to 12.5 µM, 25 µM, and 50 µM, keeping the concentrations of the other reagents constant: c(**GSH**) = 10 µM and c(NaN_3) = 1.5 mM. The concentration of **TT** was plotted against Σ[**VTT**]$^{\beta}$•Δt, generating graphs for different values of β (0, 0.5, 1.0, and 2.0). The graphs

overlay for β = 1.0, suggesting that the reaction is first order in **VTT**.

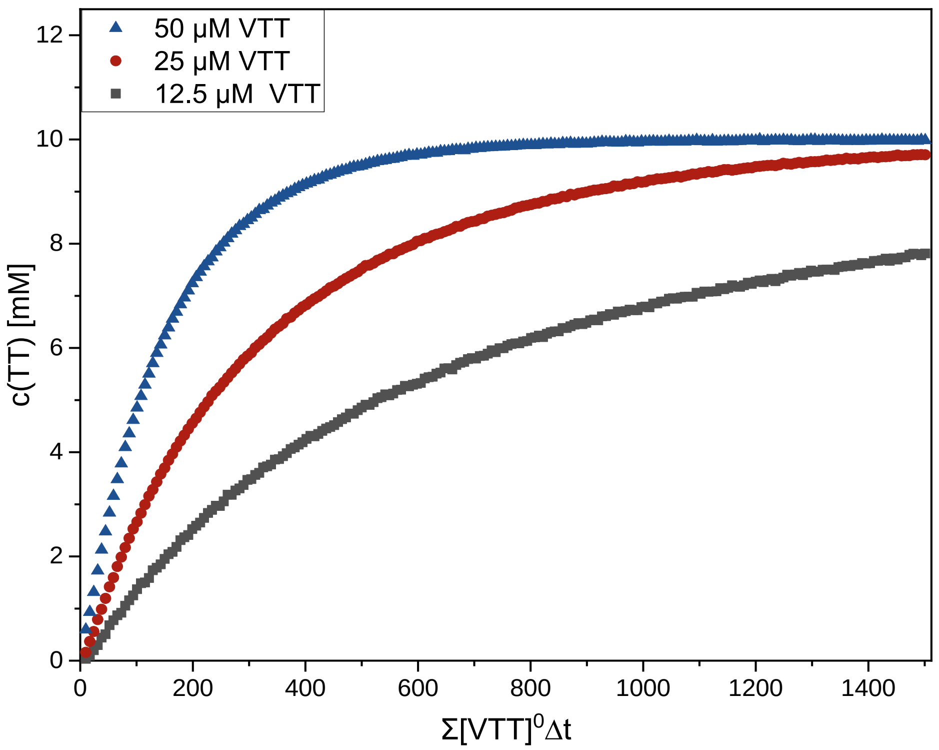

Figure S174. VTNA analysis for β = 0.

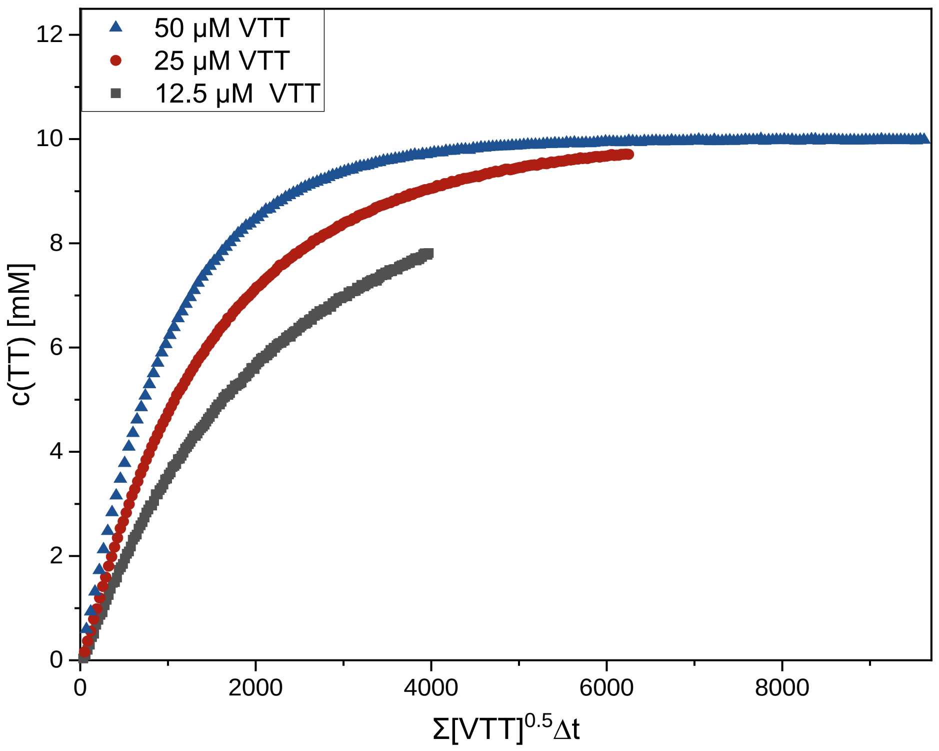

Figure S175. VTNA analysis for β = 0.5.

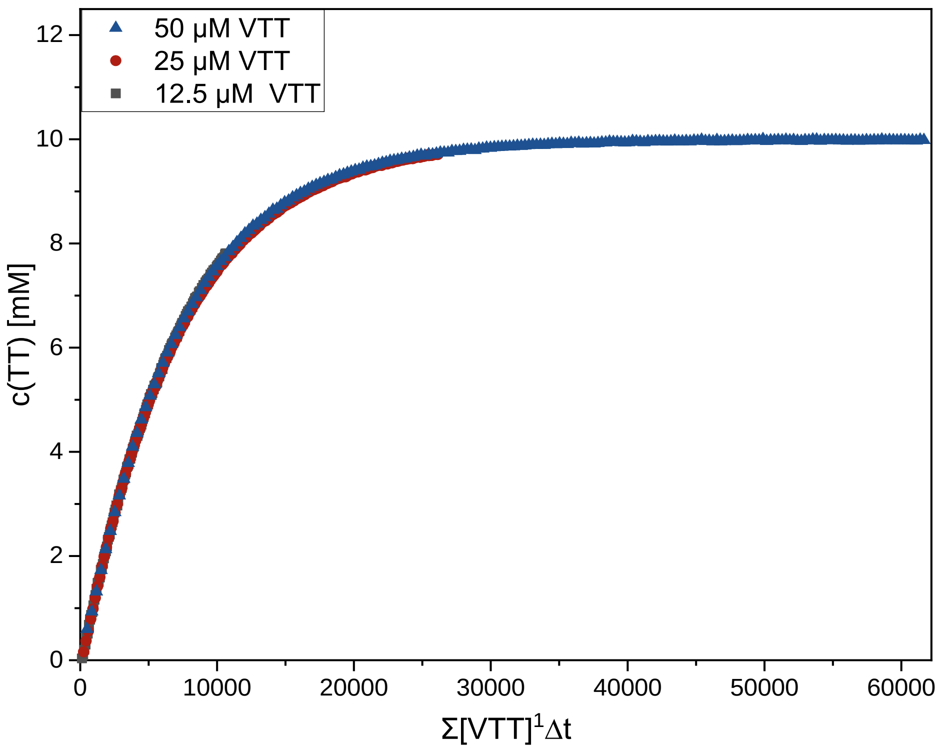

Figure S176. VTNA analysis for β = 1.0.

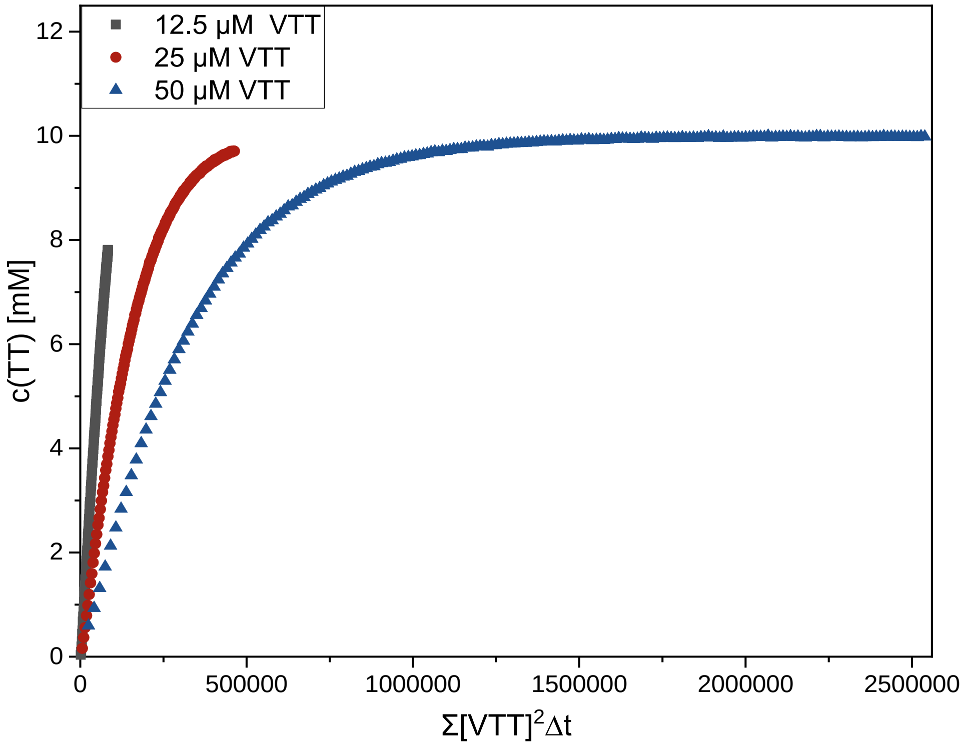

Figure S177. VTNA analysis for β = 2.0.

Reaction order in NaN_3

To determine the reaction order in NaN_3, c(NaN_3) was set to 0.5 mM, 1.5 mM, and 4.5 mM, keeping the concentrations of other reagents constant: c(**GSH**) = 10 μM and c(**VTT**) = 50 μM. Because NaN_3 was used in a large excess, a different normalized time scale analysis was applied[29] and the concentration of **TT** was plotted

against t•[NaN_3]$^{\beta}$, generating graphs for different values of β (0, 0.5, 1.0, and 2.0). The graphs overlay if β = 0, suggesting that the reaction is zero order in NaN_3.

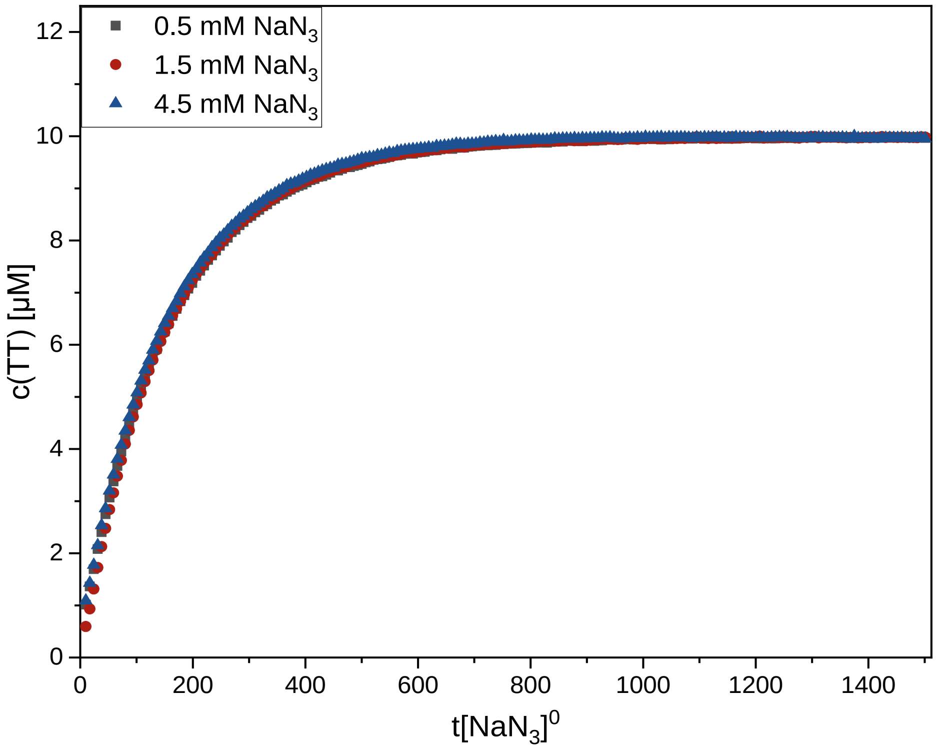

Figure S178. VTNA analysis for β = 0.

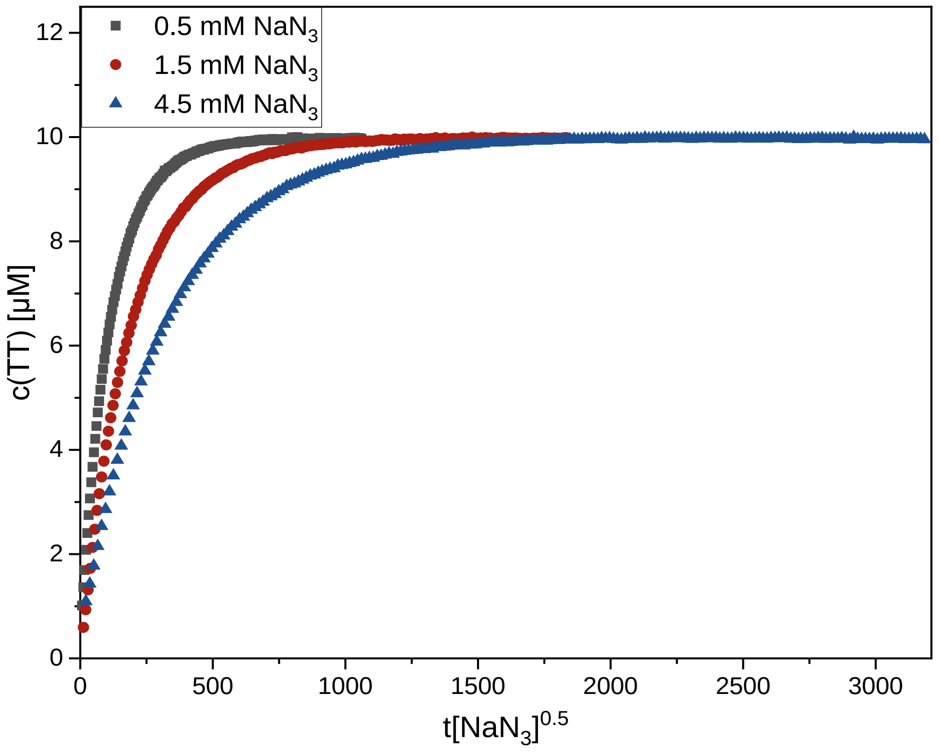

Figure S179. VTNA analysis for β = 0.5.

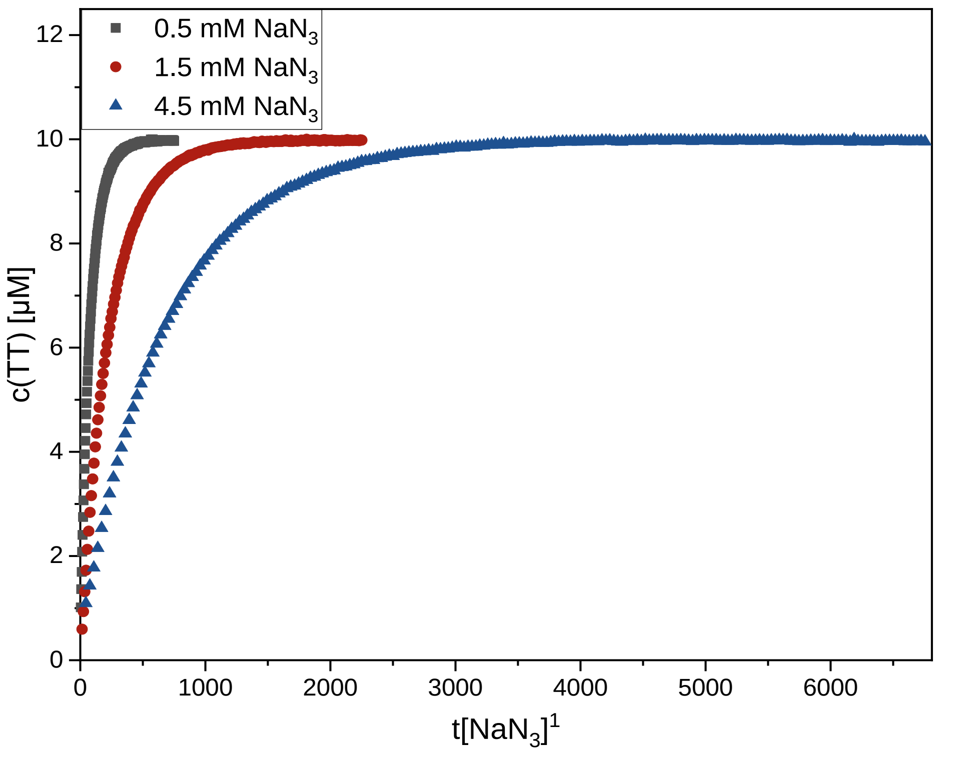

Figure S180. VTNA analysis for β = 1.0.

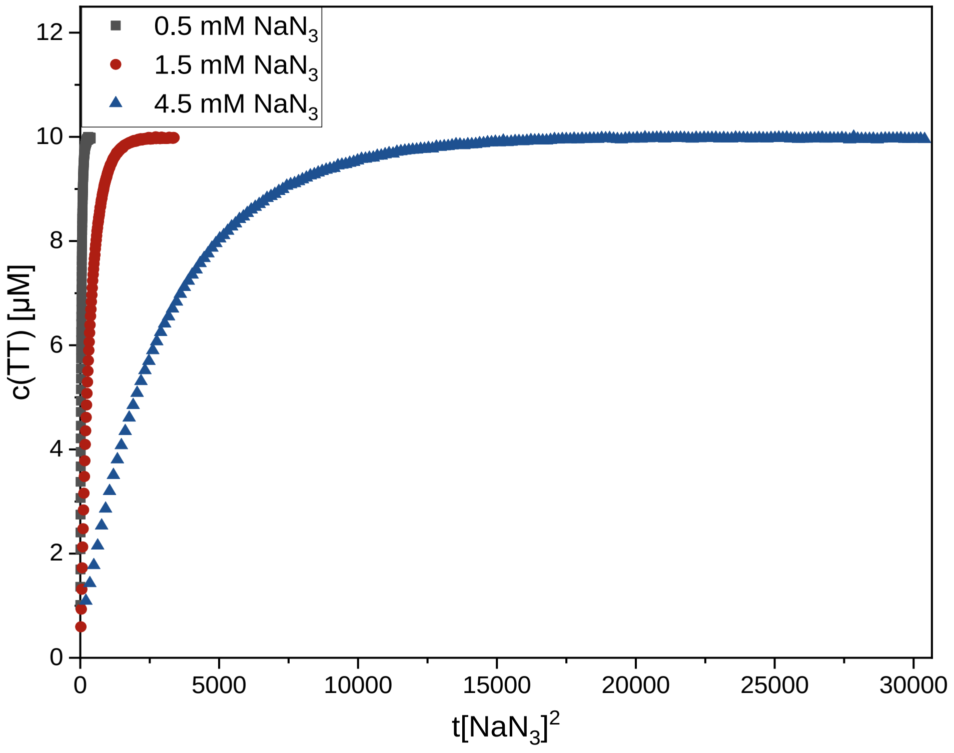

Figure S181. VTNA analysis for β = 2.0.

Determination of the second-order rate constant for VTT

Because the reaction of glutathione with **VTT** and NaN_3 follows second-order kinetics, the rate constant can be determined by carrying out reactions with equal concentrations of **GSH** and **VTT**, and plotting 1/c(**VTT**) against time. The slope of the plot provides the second-order rate constant.

The consumption of **VTT** over time was determined by measuring the absorbance of **VTT** at 307 nm. The reaction mixtures contained equal concentrations of **VTT** and **GSH** (30 µM) and an excess of NaN_3 (0.9 mM). Sodium phosphate buffer (pH 7.4, c = 50 mM) was used as solvent.

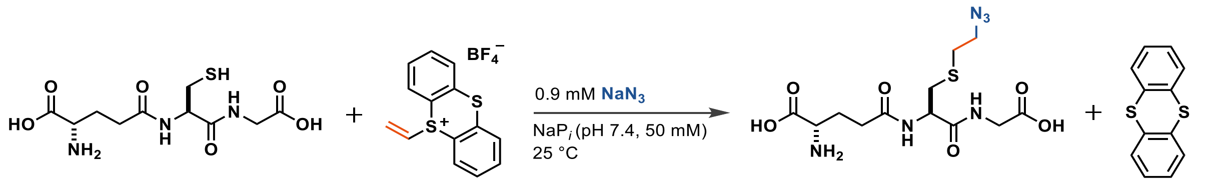

General procedure: Stock solutions of **GSH** (1.68 mM) and NaN_3 (13.8 mM) were prepared in sodium phosphate buffer (pH 7.4, c = 50 mM). NaP_i (0.182 mL, pH 7.4, c = 50 mM), stock solutions of NaN_3 (0.13 mL), and **GSH** (36 µL) were pipetted into a quartz cuvette charged with a Teflon-coated magnetic stirring bar. The cuvette was thermostated for 5 minutes at (25 ± 0.1) °C in the cell holder. After performing the background measurement, a stock solution of **VTT** in DMF (10 µL, c = 6.0 mM) was added to the cuvette. The reaction mixture was homogenized by pipetting the mixture up and down. Then, the cuvette was capped and the absorbance of **VTT** at 307 nm was measured while stirring.

Because formation of **TT** precipitate was observed after 5 minutes at these reaction conditions, each measurement used for the determination of the rate constant was stopped after 300 seconds. The reaction mixture was then transferred from the cuvette into a vial charged with a stirring bar and stirred at 25 °C for 24 hours. The suspension was filtered through a syringe filter and the absorbance of the clear solution at 307 nm was determined. This value was used to define the total range in which the absorbance of the reaction mixture at 307 nm changed during the reaction.

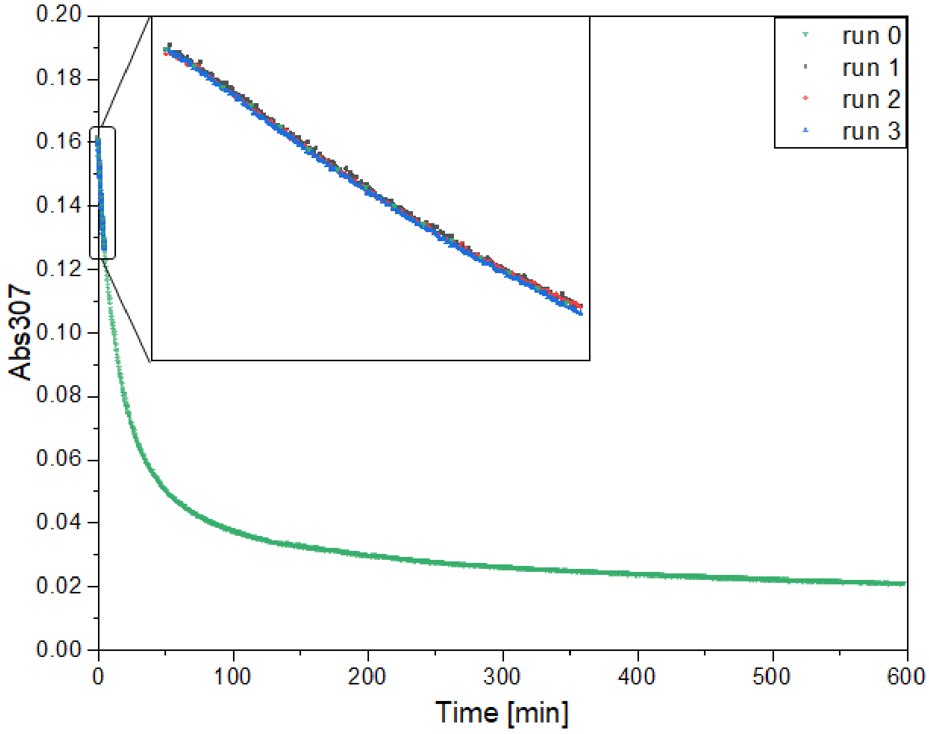

Figure S182. Decay of **VTT** absorption at 307 nm in reaction with **GSH** and NaN_3. Runs 1-3 were used to determine the rate constant.

The second-order initial rate constant was determined by plotting 1/c(**VTT**) against time and determining the slope of the linear fit for each graph.

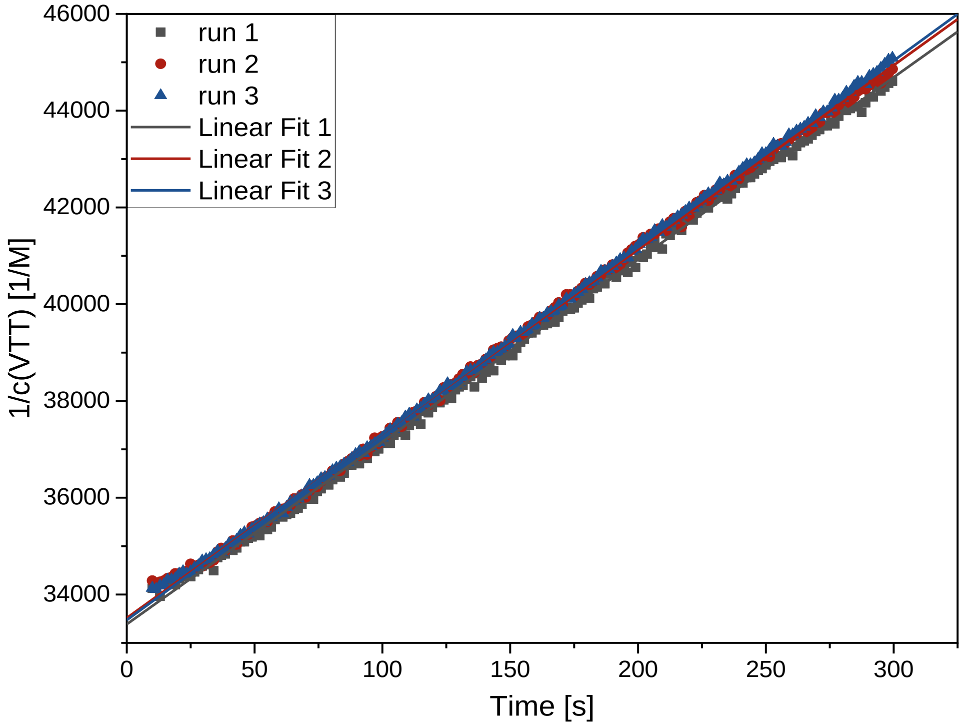

Figure S183. Estimation of the second-order rate constant for 25% conversion.

Table S16. Rate constant values determined from the slopes of the corresponding linear fit data.

Run	k_2, $M^{-1}s^{-1}$	R^2
1	37.7 ± 0.1	0.9991
2	38.1 ± 0.1	0.9993
3	38.5 ± 0.1	0.9996

Based on the data from three runs: **k_2(VTT) = 38.1 ± 0.1 $M^{-1}s^{-1}$**.

Determination of the second-order rate constant for $^2H_3$-VTT

The consumption of **$^2H_3$-VTT** over time was determined by measuring the absorbance of **$^2H_3$-VTT** at 307 nm. The reaction mixtures contained equal concentrations of **$^2H_3$-VTT** and **GSH** (30 µM) and an excess of NaN_3 (0.9 mM). Sodium phosphate buffer (pH 7.4, c = 50 mM) was used as solvent.

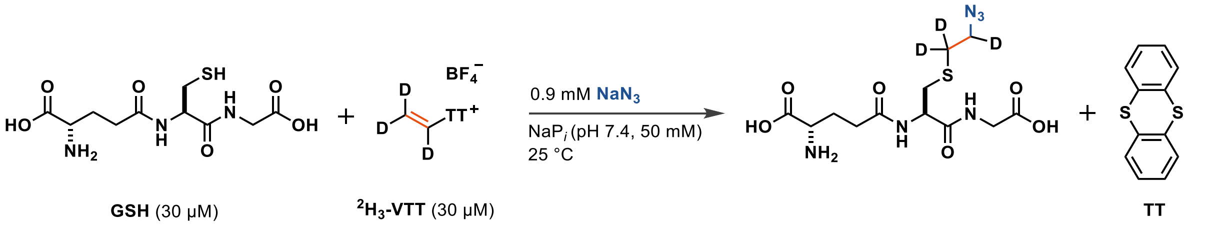

General procedure: Stock solutions of **GSH** (1.68 mM) and NaN_3 (13.8 mM) were prepared in sodium phosphate buffer (pH 7.4, c = 50 mM). NaP_i (0.182 mL, pH 7.4, c = 50 mM), stock solutions of NaN_3 (0.13 mL),

and **GSH** (36 µL) were pipetted into a quartz cuvette charged with a Teflon-coated magnetic stirring bar. The cuvette was thermostated for 5 minutes at (25 ± 0.1) °C in the cell holder. After performing the background measurement, a stock solution of ^{2}**H$_3$-VTT** in DMF (10 µL, c = 6.0 mM) was added to the cuvette. The reaction mixture was homogenized by pipetting the mixture up and down. Then, the cuvette was capped and the absorbance of ^{2}**H$_3$-VTT** at 307 nm was measured while stirring for 5 minutes. The reaction mixture was then transferred from the cuvette into a vial charged with a stirring bar and stirred at 25 °C for 24 hours. The suspension was filtered through a syringe filter and the absorbance of the clear solution at 307 nm was determined. This value was used to define the total range in which the absorbance of the reaction mixture at 307 nm changed during the reaction.

The second-order initial rate constant was determined by plotting 1/c(^{2}**H$_3$-VTT**) against time and determining the slope of the linear fit for each graph.

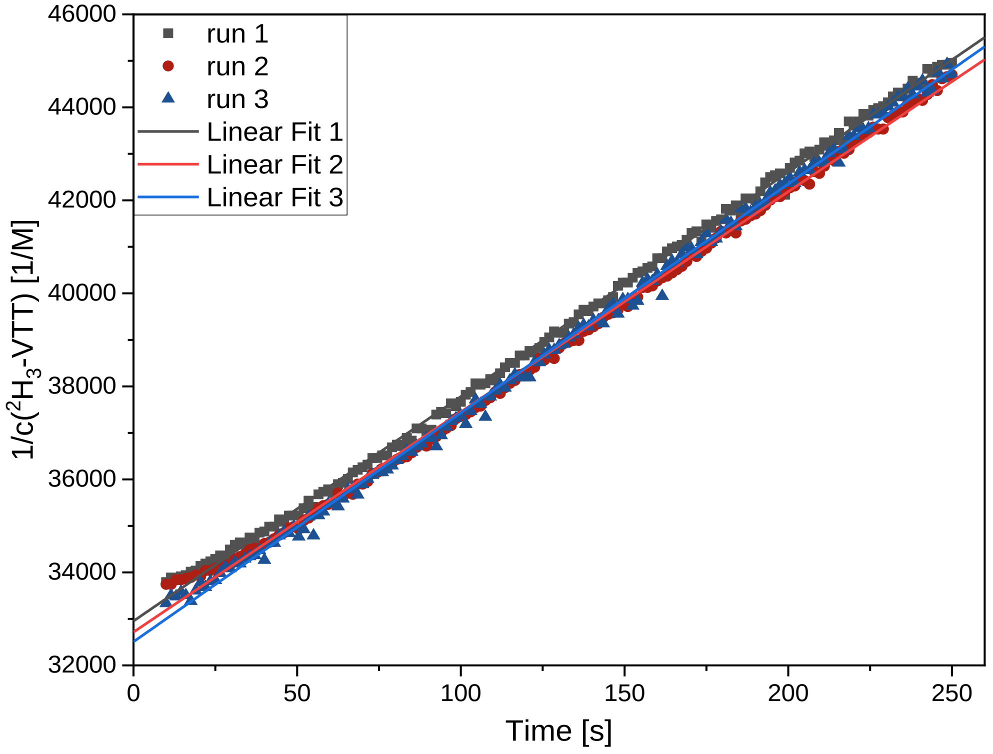

Figure S184. Estimation of the second-order rate constant at 25% conversion.

Table S17. Rate constant values determined from the slopes of the corresponding linear fit data.

**Run**	***k*$_2$, M^{-1}s^{-1}**	**R^2**
1	48.3 ± 0.1	0.9988
2	47.4 ± 0.2	0.9982
3	49.2 ± 0.2	0.9982

Based on the data from three runs: ***k*$_2$(^{2}H$_3$-VTT) = 48.3 ± 0.1 M^{-1}s^{-1}**.

Kinetic isotope effect for functionalization of GSH with VTT

Based on the second-order rate constants for **VTT** and ^{2}**H$_3$-VTT** (see Table S16 and Table S17):

$$\text{KIE} = \frac{k_2(\mathbf{VTT})}{k_2(^2\mathbf{H_3{-}VTT})} = \frac{38.1}{48.3} = 0.79.$$

Competition experiment between VTT and $^2H_3$-VTT

The reaction between **GSH**, NaN_3 and an excess of **VTT** was performed in deuterated buffer (D-NaP*i*, pH 7.0, c = 100 mM) and the reaction mixture was analyzed with ^1H NMR using 3-(trimethylsilyl)propionic-2,2,3,3-d_4 acid sodium salt (**TSP**) as an internal standard:

Stock solutions of **GSH** (50 mM), **TSP** (50 mM), and NaN_3 (1.0 M) were prepared in D_2O. D-NaP*i* (100 µL, pH 7.0, c = 0.5 M), D_2O (200 µL), stock solutions of NaN_3 (75 µL), **TSP** (50 µL), and **GSH** (50 µL) were pipetted into an Eppendorf tube and mixed by pipetting the mixture up and down. Subsequently, a stock solution of **VTT** in DMF-d_7 (25 µL, c = 1.0 M) was added to the tube to obtain a mixture containing **GSH** (5.0 mM, 1.0 equiv.), **TSP** (5.0 mM, 1.0 equiv.), NaN_3 (150 mM, 30.0 equiv.), and **VTT** (50 mM, 10 equiv.). The ^1H NMR spectrum of the mixture was measured 5 minutes after addition of **VTT**:

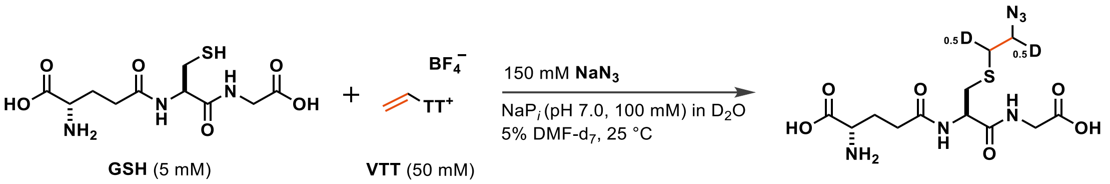

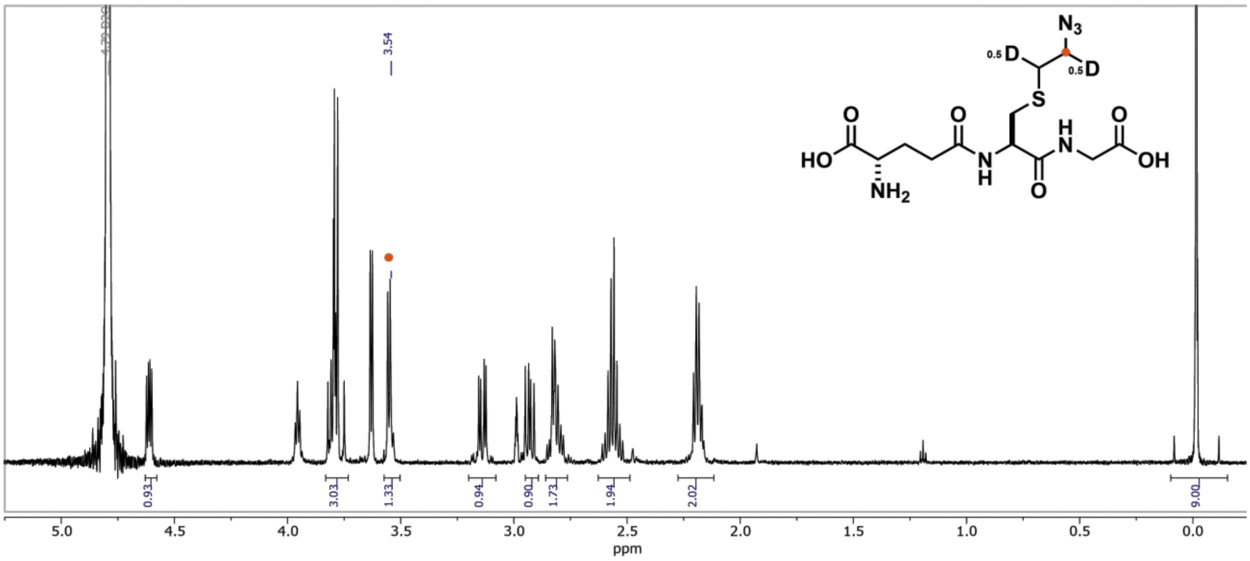

Figure S185 ^1H NMR spectrum of **GSH** (5.0 mM, 1.0 equiv.) in D-NaP*i* (7.0, 100 mM) after addition of 10 equiv. **VTT** in presence of 30 equiv. NaN_3 with **TSP** as internal standard

The reaction between **GSH**, NaN_3 and an excess of **VTT** and ^{2}**H$_3$-VTT** was performed in deuterated buffer (D-NaP*i*, pH 7.0, c = 100 mM) and the reaction mixture was analyzed with ^1H NMR using **TSP** as an internal standard:

Stock solutions of **GSH** (50 mM), **TSP** (50 mM), and NaN_3 (1.0 M) were prepared in D_2O. D-NaP*i* (100 µL, 500 mM), D_2O (200 µL), stock solutions of NaN_3 (75 µL), **TSP** (50 µL), and **GSH** (50 µL) were pipetted into an Eppendorf tube and mixed by pipetting the mixture up and down. Subsequently, 25 µL of a solution containing **VTT** (0.5 M) and ^{2}**H$_3$-VTT** (0.5 M) in DMF-d_7 was added to the tube to obtain a mixture containing **GSH** (5.0 mM, 1.0 equiv.), **TSP** (5.0 mM, 1.0 equiv.), NaN_3 (150 mM, 30.0 equiv.), **VTT** (25 mM, 5.0 equiv.), and ^{2}**H$_3$-VTT** (25

mM, 5.0 equiv.). The ^{1}H NMR spectrum of the mixture was measured 5 minutes after addition of the mixture containing **VTT** and **^{2}H$_3$-VTT**:

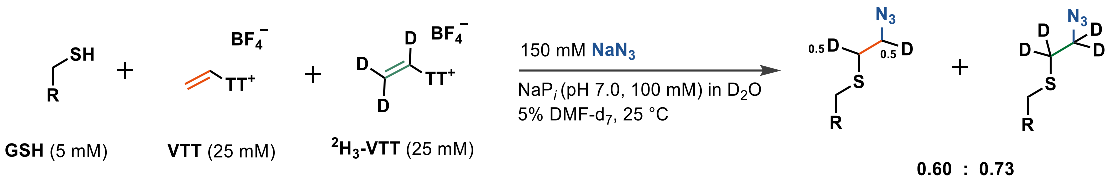

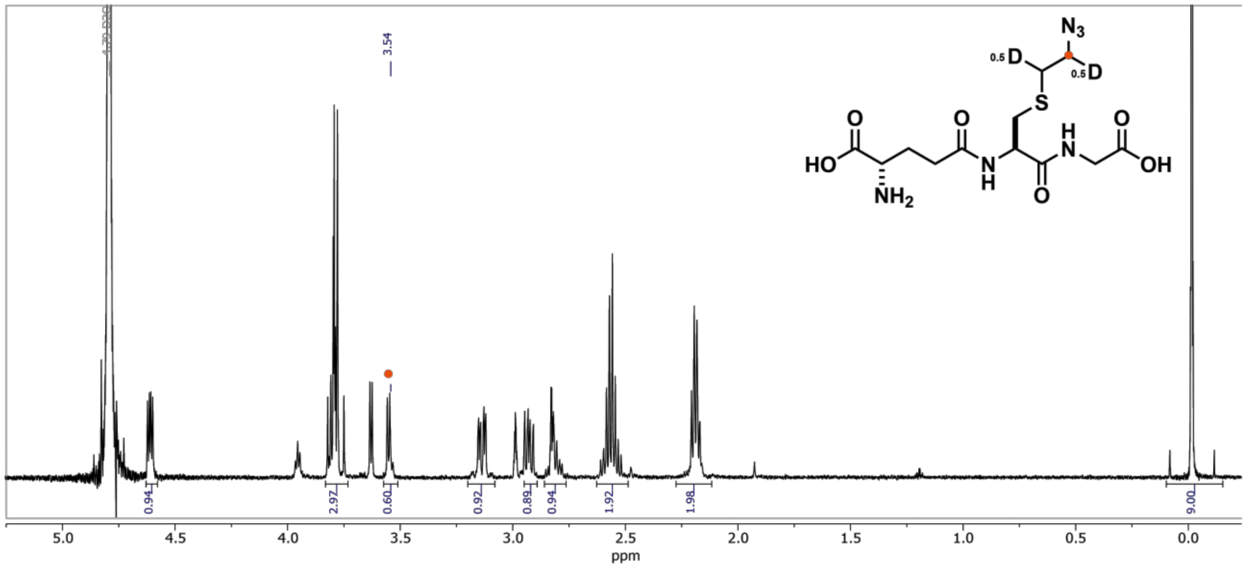

Figure S186 ^{1}H NMR spectrum of **GSH** (5.0 mM, 1.0 equiv.) in D-NaP$_i$ (7.0, 50 mM) after addition of 5.0 equiv. **VTT** and 5.0 equiv. **^{2}H$_3$-VTT** in presence of 30 equiv. NaN$_3$ with **TSP** as internal standard

The ratio of the obtained products formed in reaction with either **VTT** or **^{2}H$_3$-VTT** was determined by calculating the deuterium incorporation in the ethylene bridge for both reactions. The peak at 3.54 ppm corresponds to the methylene group alpha to the azide in the product obtained with **VTT** (see Table S19) and was integrated for both spectra to calculate the ratio of the products formed with either **VTT** or **^{2}H$_3$-VTT**. Based on the integral values:

$$\frac{k_2(\mathrm{VTT})}{k_2(^2\mathrm{H}_3\mathrm{-VTT})} = \frac{0.60}{1.33 - 0.60} = \frac{0.60}{0.73} = 0.82.$$

Kinetic Reaction Profile for VTFT

The kinetic profile of glutathione functionalization with **VTFT** and sodium azide was investigated in the same way as it was done for **VTT**. The experiments were performed with an excess of **VTFT** and NaN$_3$. Because **GSH** and **VTFT** react in the 1:1 stoichiometric ratio to produce 1 equivalent of **TFT** under these conditions, the concentration of **TFT** can be used to determine the concentrations of **GSH** and **VTFT** at any point in time. The reaction mixtures contained 30% MeCN to ensure the solubility of **TFT** in the reaction mixture.

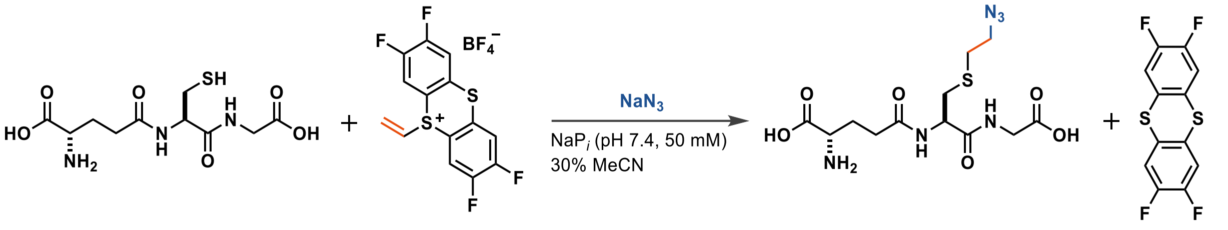

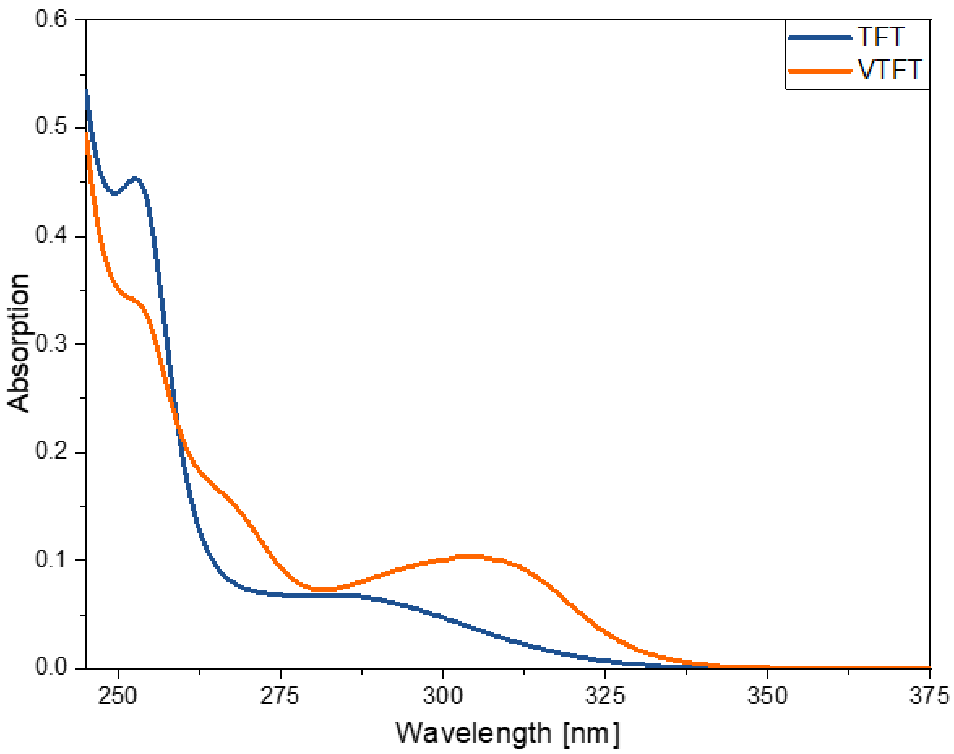

Figure S187. Absorbance spectra of 30 µM TFT and **VTFT** aqueous solutions containing 30% MeCN.

General procedure: Stock solutions of **GSH** (1.87 mM) and NaN_3 (17.6 mM) were prepared in sodium phosphate buffer (pH 7.4, 71 mM). NaP_i (pH 7.4, 71 mM), MeCN, NaN_3, and **GSH** stock solutions were pipetted into a quartz cuvette charged with a Teflon-coated magnetic stirring bar. The cuvette was thermostated for 5 minutes at (25 ± 0.1) °C in the cell holder. After performing the background measurement, 10 µL of a **VTFT** stock solution in DMF was added to the cuvette. The obtained buffered mixture (V = 2.0 mL; 50 mM NaP_i; 30% v/v MeCN) contained 10 – 30 µM of **GSH**, 12.5 – 50 µM of **VTFT**, and 0.5 – 4.5 mM of NaN_3. The reaction mixture was homogenized by pipetting the mixture up and down. Then, the cuvette was capped and the release of **TFT** was monitored by measuring absorbance at 253 nm while stirring.

Reaction order in GSH

To determine the reaction order in **GSH**, three experiments were carried out with c(**GSH**) set to 10 µM, 20 µM, and 30 µM, keeping the concentrations of the other reagents constant: c(**VTFT**) = 50 µM and c(NaN_3) = 1.5 mM. The concentration of **TFT** was plotted against Σ[**GSH**]$^{\beta}$ •Δt, generating graphs for different values of β (0, 0.5, 1.0, and 2.0) according to the Burés' method[28]. The graphs overlay for β = 1.0, suggesting that the reaction is first order in **GSH**.

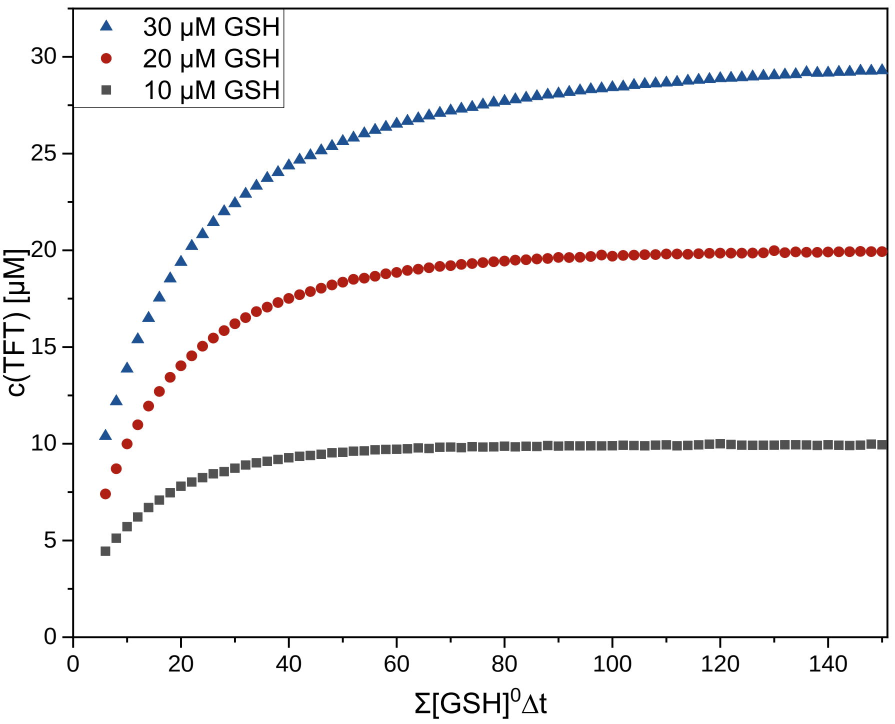

Figure S188. VTNA analysis for β = 0.

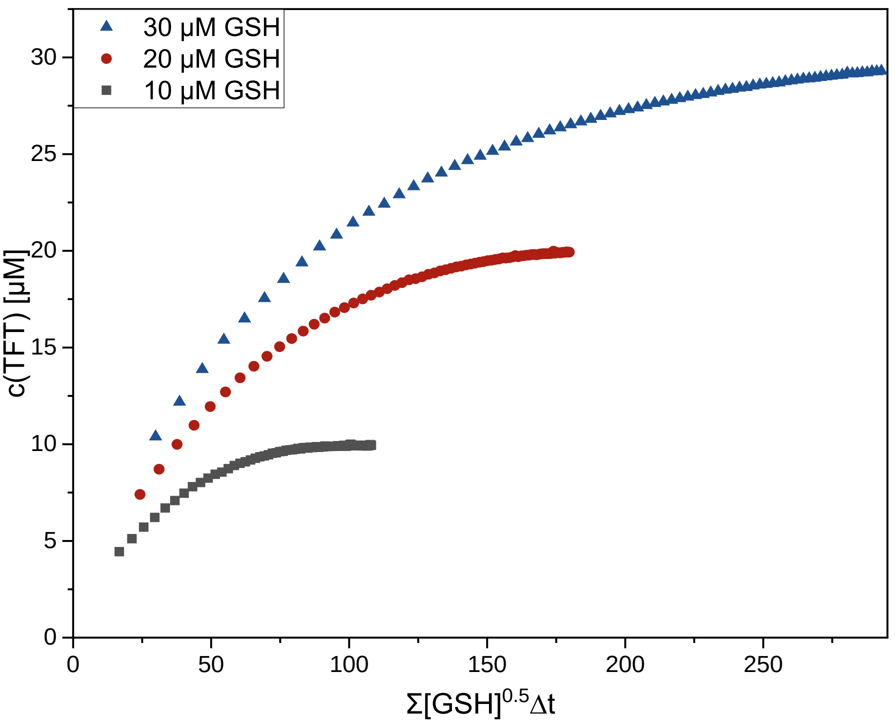

Figure S189. VTNA analysis for β = 0.5.

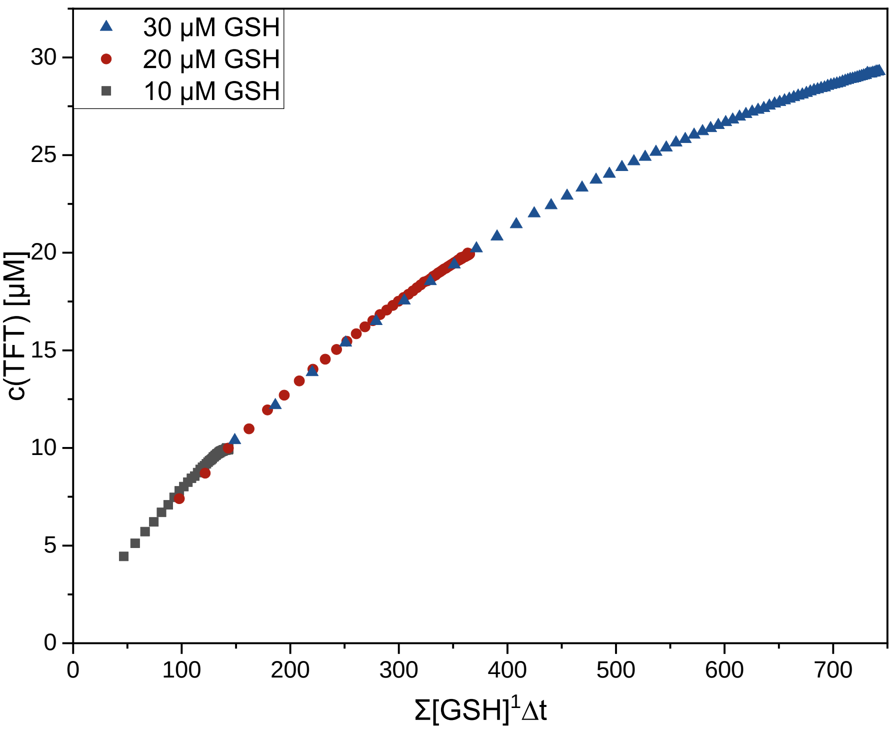

Figure S190. VTNA analysis for β = 1.0.

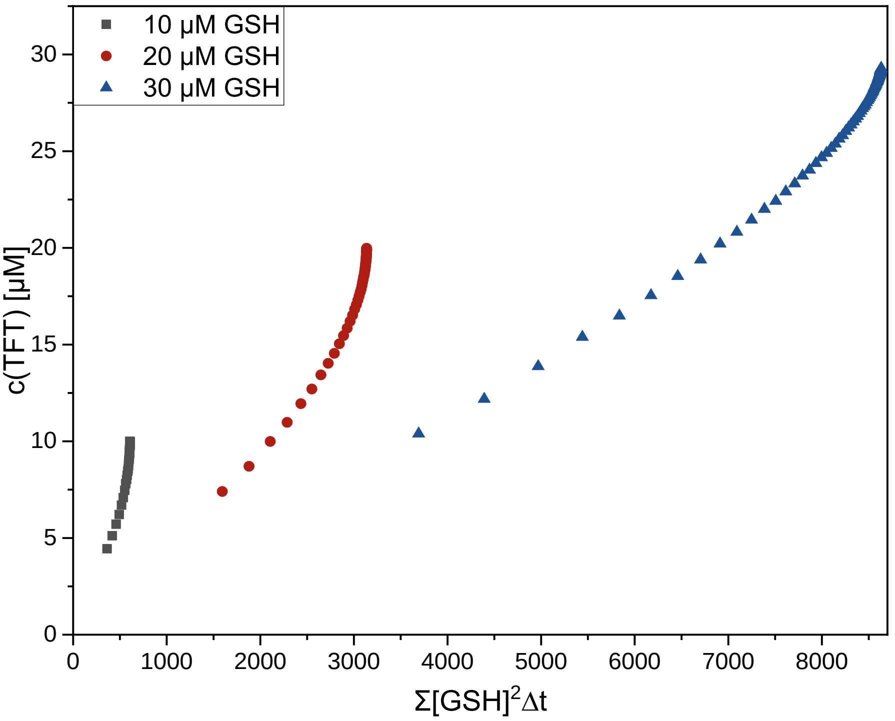

Figure S191. VTNA analysis for β = 2.0.

Reaction order in VTFT

To determine the reaction order in **VTFT**, c(**VTFT**) was set to 12.5 µM, 25 µM, and 50 µM, keeping the concentrations of the other reagents constant: c(**GSH**) = 10 µM and c(NaN_3) = 1.5 mM. The concentration of **TFT** was plotted against Σ[**VTFT**]^{β}•Δt, generating graphs for different values of β (0, 0.5, 1.0, and 2.0). The graphs overlay for β = 1.0, suggesting that the reaction is first order in **VTFT**.

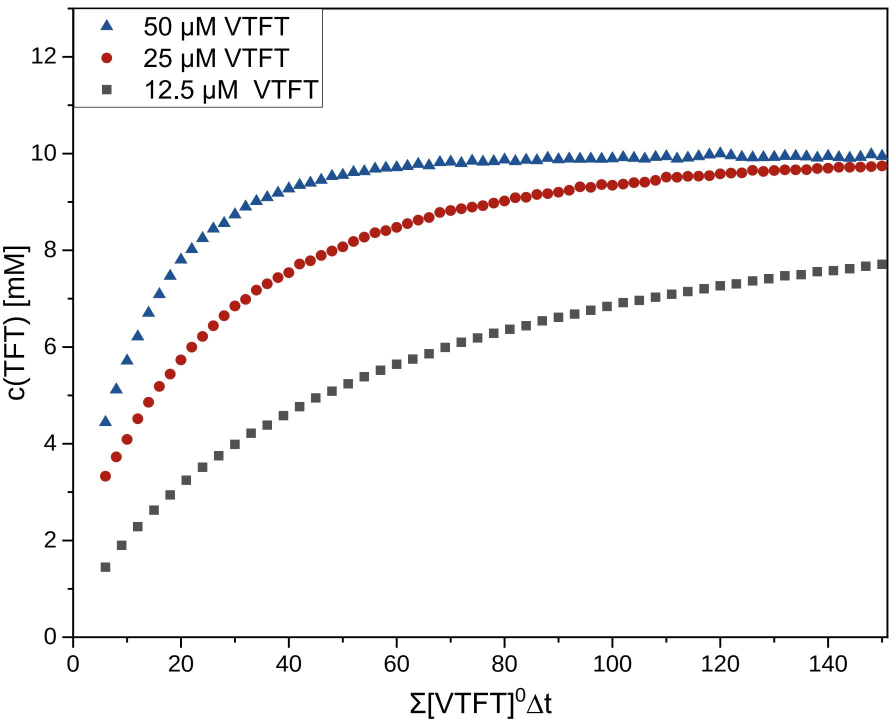

Figure S192. VTNA analysis for β = 0.

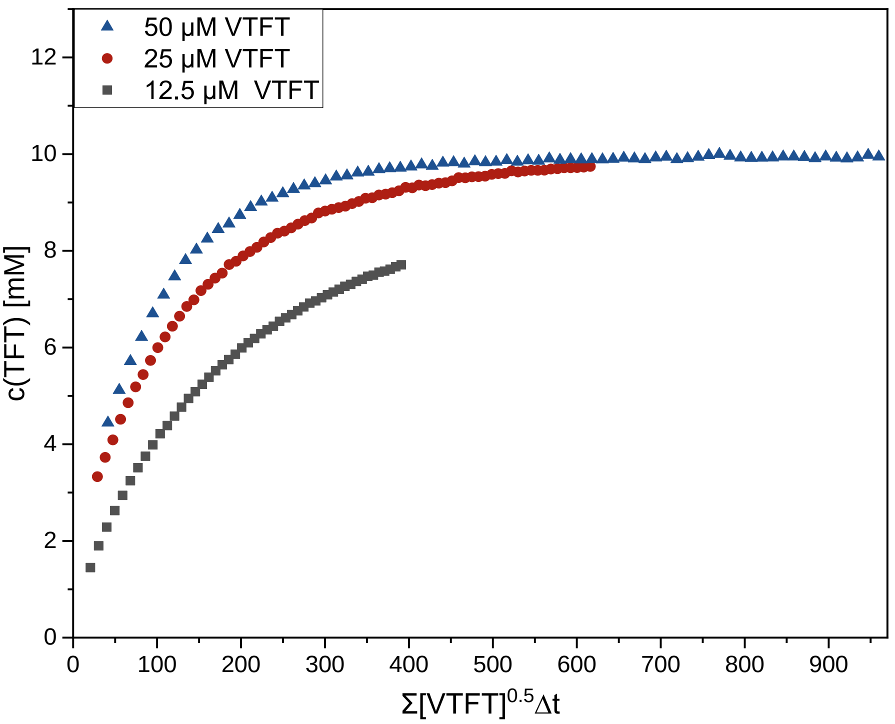

Figure S193. VTNA analysis for β = 0.5.

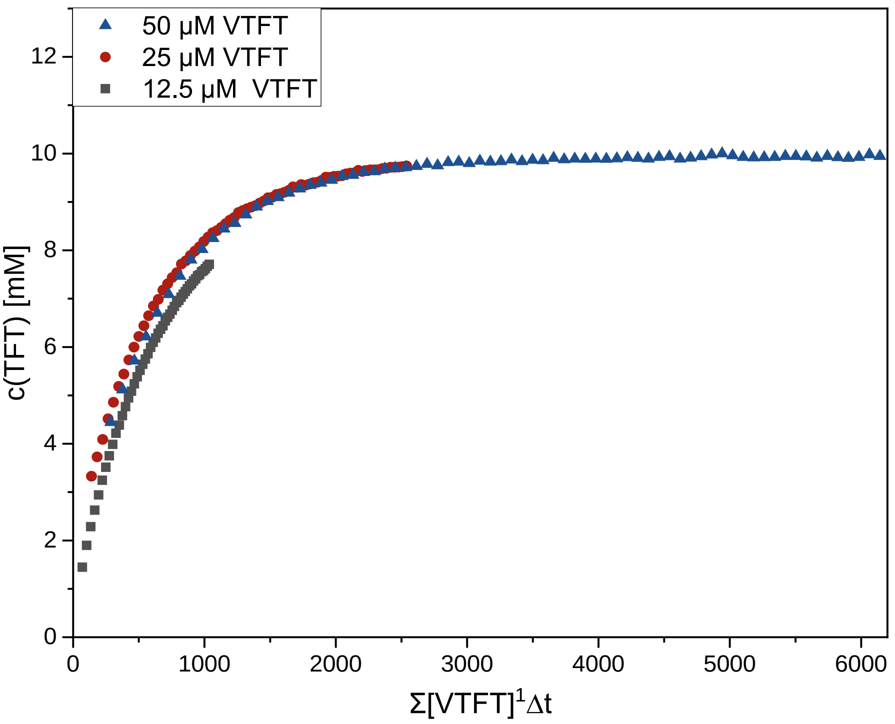

Figure S194. VTNA analysis for β = 1.0.

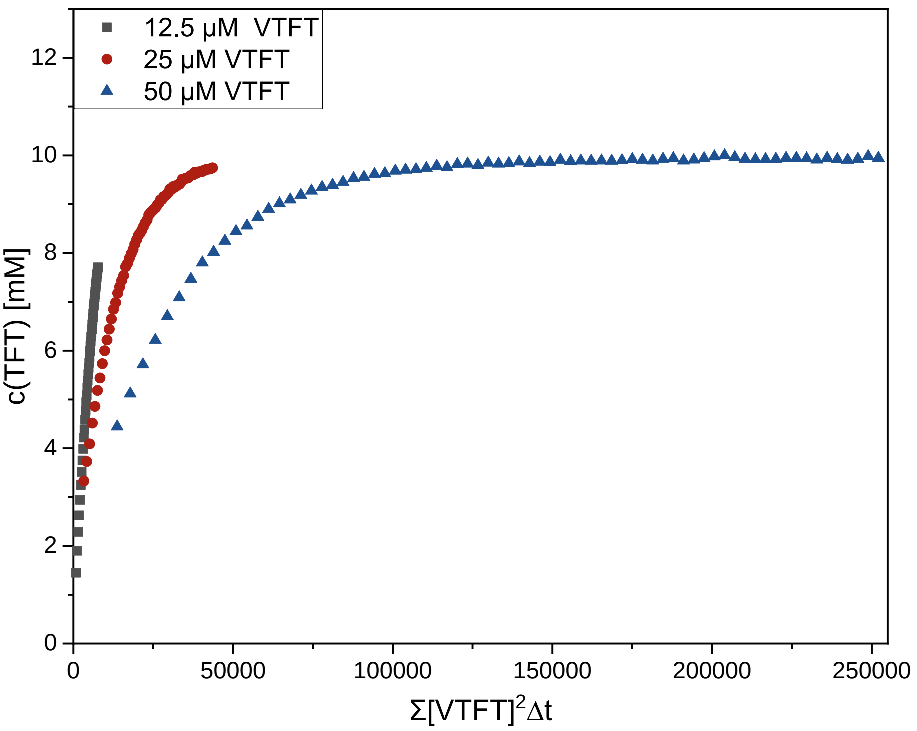

Figure S195. VTNA analysis for β = 2.0.

Reaction order in NaN_3

To determine the reaction order in NaN_3, c(NaN_3) was set to 0.5 mM, 1.5 mM, and 4.5 mM, keeping the concentrations of other reagents constant: c(**GSH**) = 10 µM and c(**VTFT**) = 50 µM. Because NaN_3 was used in a large excess, a different normalized time scale analysis was applied[29] and the concentration of **TFT** was plotted against t•[NaN_3]$^{\beta}$, generating graphs for different values of β (0, 0.5, 1.0, and 2.0). The graphs overlay for β = 0,

suggesting that the reaction is zero order in NaN_3.

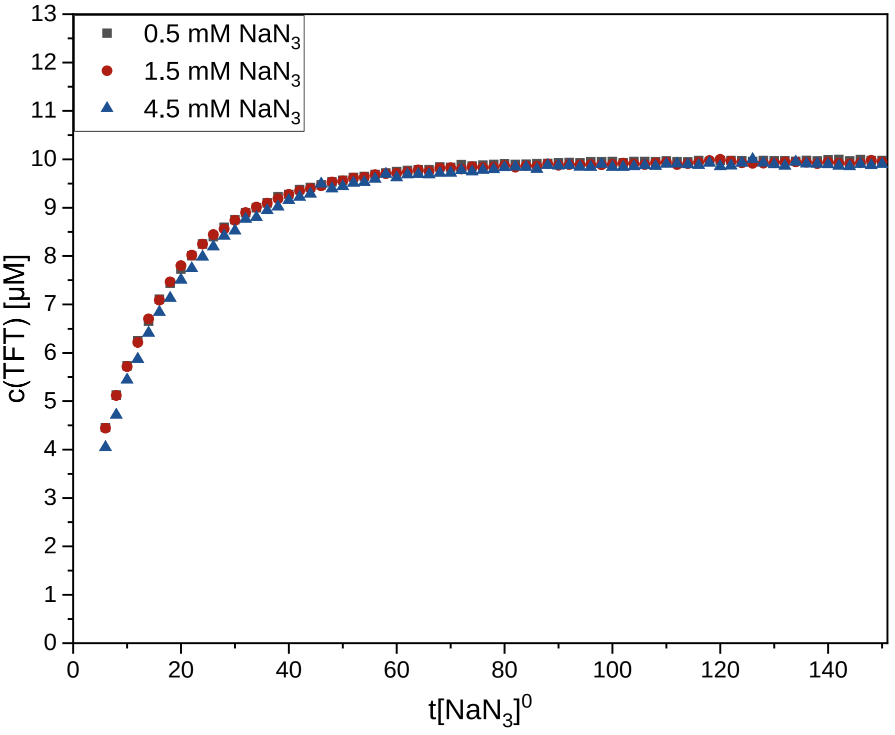

Figure S196. VTNA analysis for β = 0.

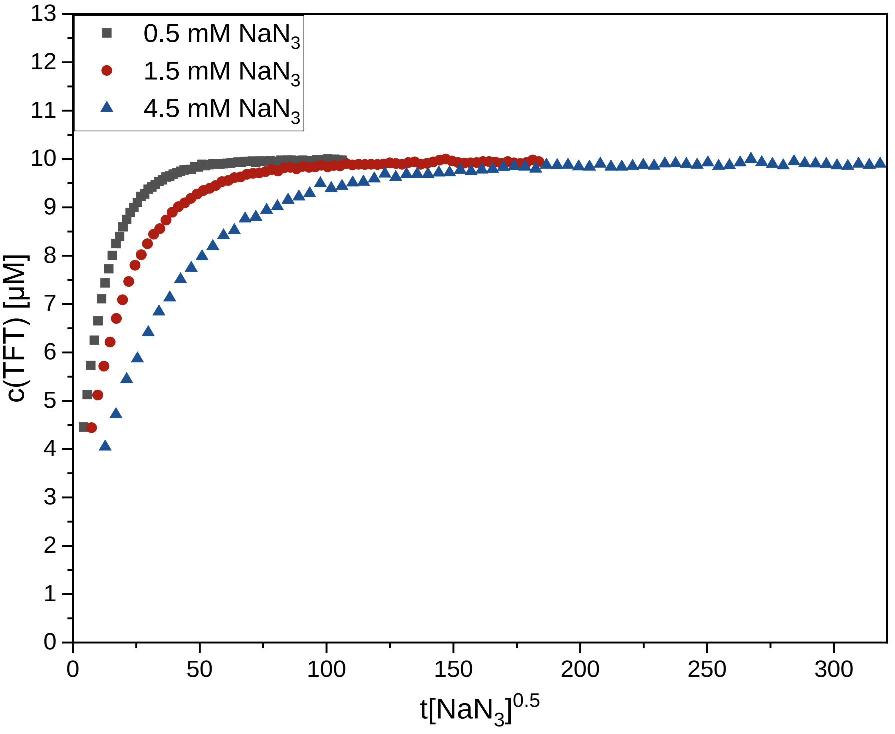

Figure S197. VTNA analysis for β = 0.5.

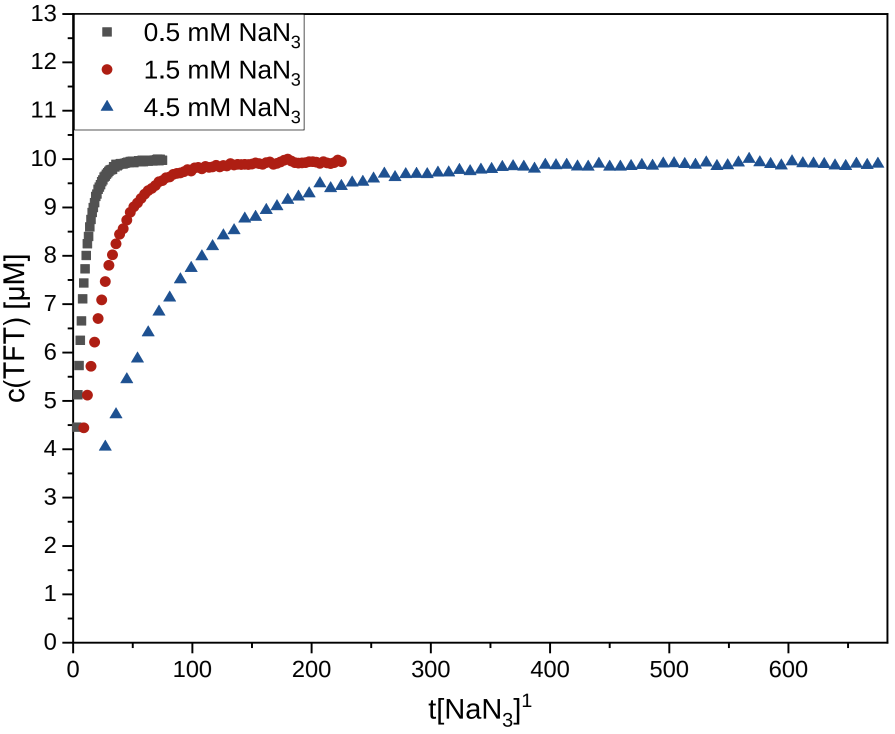

Figure S198. VTNA analysis for β = 1.0.

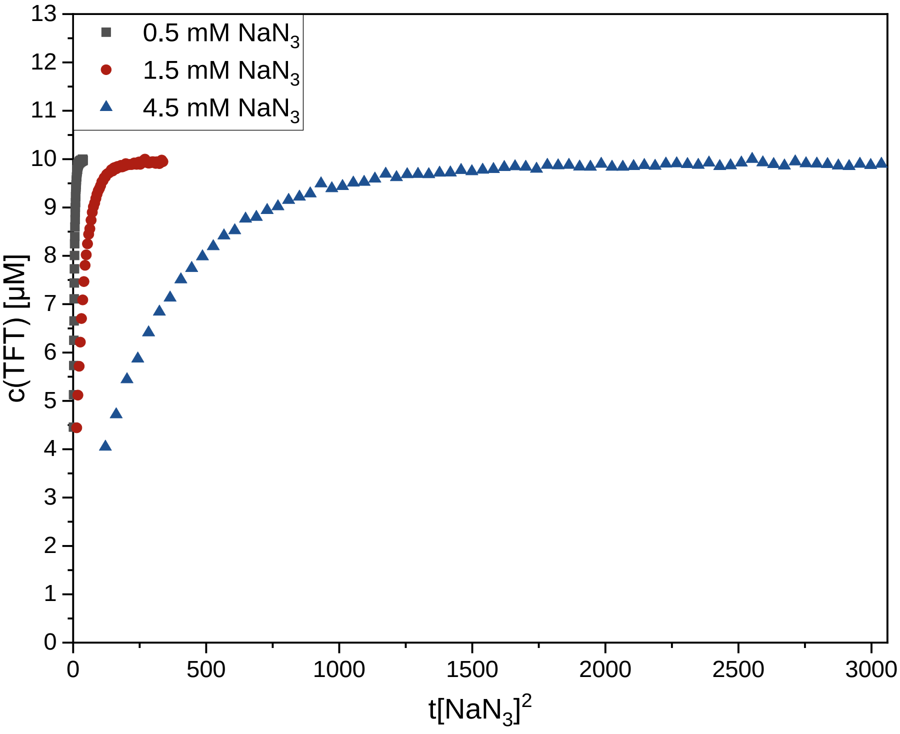

Figure S199. VTNA analysis for β = 2.0.

Determination of the second-order rate constant for VTFT

The rate constant for functionalization of **GSH** with **VTFT** and NaN_3 was determined by carrying out reactions with equal concentrations of **GSH** and **VTFT**, and plotting 1/c(**VTFT**) against time. The slope of the plot provides the second-order rate constant.

The consumption of **VTFT** over time was determined by measuring the absorbance of **VTFT** at 306 nm. The reaction mixtures contained equal concentrations of **VTFT** and **GSH** (30 µM) and an excess of NaN_3 (0.9 mM). Sodium phosphate buffer (pH 7.4, 50 mM) was used as solvent.

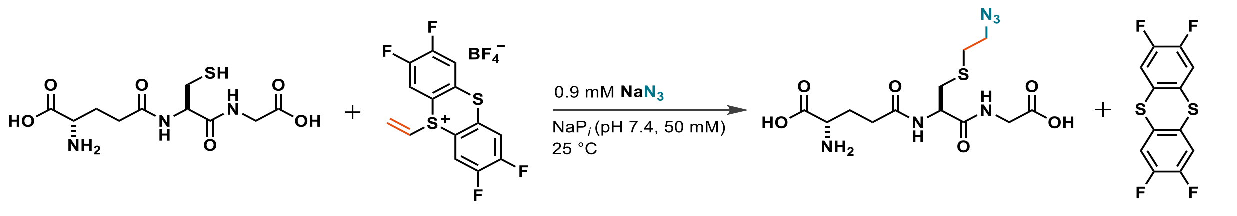

General procedure: Stock solutions of **GSH** (1.74 mM) and NaN_3 (14.2 mM) were prepared in sodium phosphate buffer (pH 7.4, 50 mM). NaP_i (0.18 mL), stock solutions of NaN_3 (127 µL), and **GSH** (35 µL) were pipetted into a quartz cuvette and charged with a Teflon-coated magnetic stirring bar. The cuvette was thermostated for 5 minutes at 25 °C in the cell holder. After performing the background measurement, a **VTFT** stock solution in DMF (10 µL, c = 6.0 mM) was added to the cuvette and the absorbance of **VTFT** at 306 nm was measured while stirring.

Because of the fast formation of **TFT** precipitate in the mixture, the absorbance change was measured for the first 11 seconds after addition of **VTFT** and before formation of **TFT** was observed. After that, the measurement was stopped, the reaction mixture was transferred from the cuvette into a vial charged with a stirring bar and stirred at 25 °C for 24 hours. The suspension was then filtered through a syringe filter, and the absorbance of the clear solution at 306 nm was determined. This value was used to define the total range in which the absorbance of the reaction mixture at 306 nm changed during the reaction.

The second-order initial rate constant was determined by plotting 1/c(**VTFT**) against time and determining the slope of the linear fit of each graph.

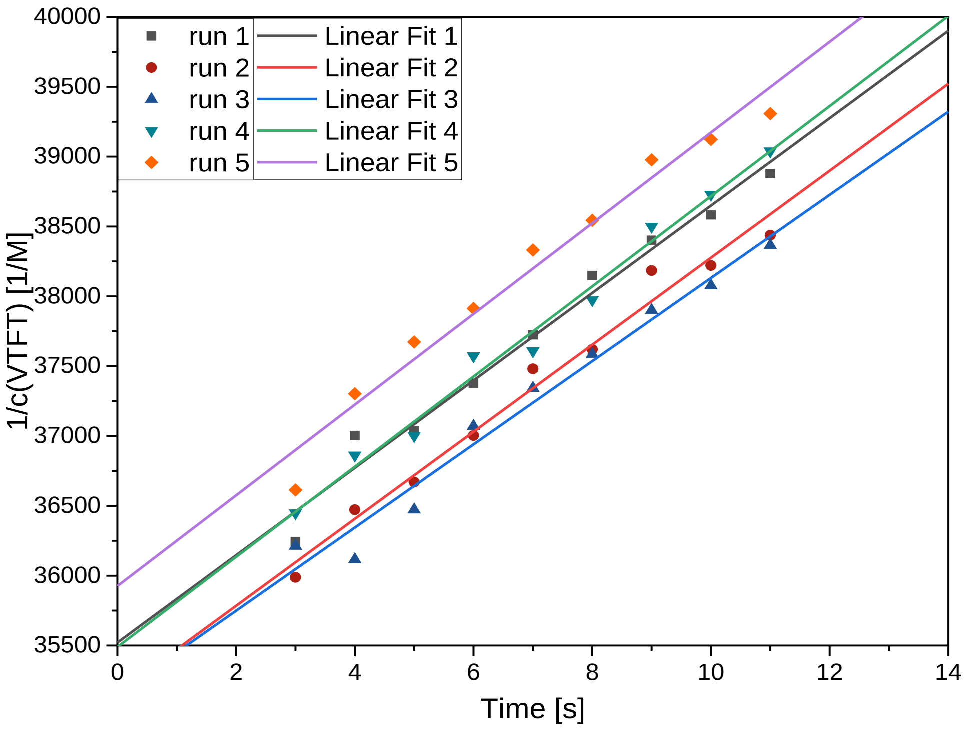

Figure S200. Estimation of the second-order rate constant at 15% conversion.

Table S18. Rate constant values determined from the slopes of the corresponding linear fit data.

Run	k_2, $M^{-1}s^{-1}$	R^2
1	313 ± 18	0.9778
2	311 ± 16	0.9809
3	297 ± 19	0.9725
4	322 ± 14	0.9876
5	325 ± 21	0.9727

Based on the data from five runs: $\mathbf{k_2}$**(VTFT) = 310 ± 8 $M^{-1}s^{-1}$**.

Competition experiment between VTFT and $^2H_3$-VTFT

The reaction between **GSH**, NaN_3 and an excess of **VTFT** was performed in deuterated buffer (D-NaP$_i$, pH 7.0, c = 100 mM) and the reaction mixture was analyzed with ^1H NMR using 3-(trimethylsilyl)propionic-2,2,3,3-d$_4$ acid sodium salt (**TSP**) as an internal standard:

Stock solutions of **GSH** (50 mM), **TSP** (50 mM), and NaN_3 (1.0 M) were prepared in D_2O. D-NaP$_i$ (100 µL, pH 7.0, c = 0.5 M), D_2O (175 µL), stock solutions of NaN_3 (75 µL), **TSP** (50 µL), and **GSH** (50 µL) were pipetted into an Eppendorf tube and mixed by pipetting the mixture up and down. Subsequently, a stock solution of **VTFT** in DMF-d$_7$ (50 µL, c = 0.5 M) was added to the tube to obtain a mixture containing **GSH** (5.0 mM, 1.0 equiv.), **TSP** (5.0 mM, 1.0 equiv.), NaN_3 (150 mM, 30.0 equiv.), and **VTFT** (50 mM, 10 equiv.). The ^1H NMR spectrum of the mixture was measured 5 minutes after addition of **VTFT**:

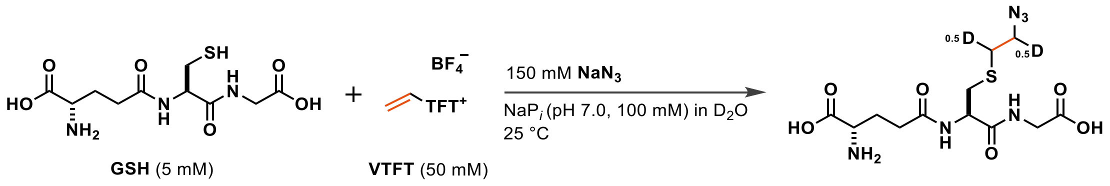

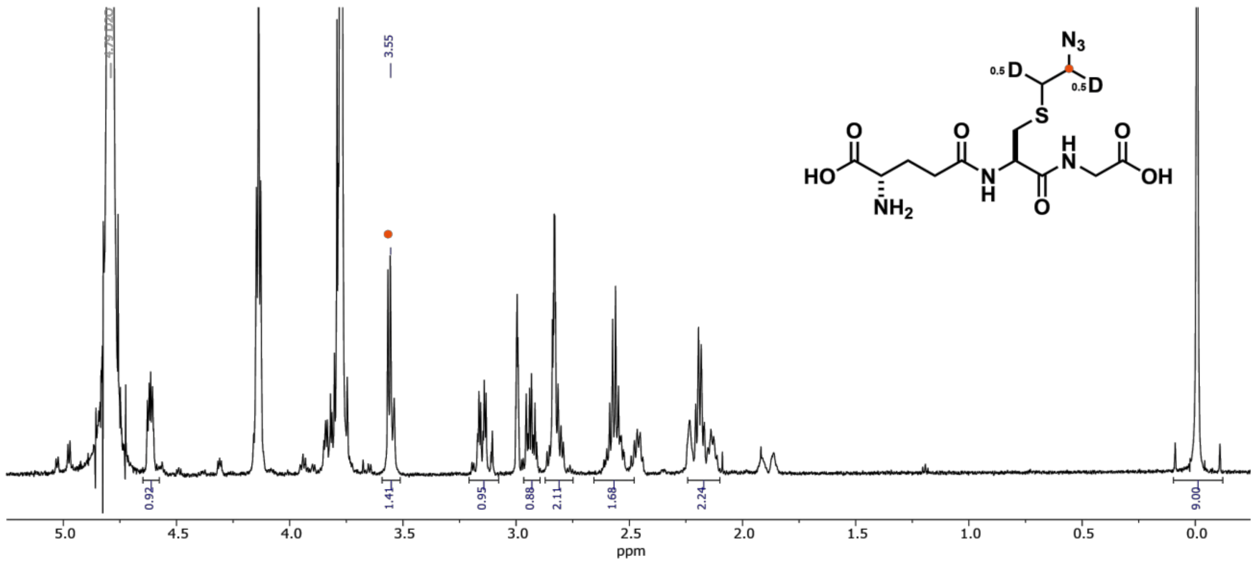

Figure S201. ^{1}H NMR spectrum of **GSH** (5.0 mM, 1.00 equiv.) in D-NaP$_i$ (pH 7.0, 50 mM) after addition of 10 equiv. **VTFT** in presence of 30.0 equiv. NaN_3 with **TSP** as internal standard.

The reaction between **GSH**, NaN_3 and an excess of **VTFT** and **$^{2}H_3$-VTFT** was performed in deuterated buffer (D-NaP$_i$, pH 7.0, 100 mM) and the reaction mixture was analyzed with ^{1}H NMR using **TSP** as an internal standard:

Stock solutions of **GSH** (50 mM), **TSP** (50 mM), and NaN_3 (1.0 M) were prepared in D_2O. D-NaP$_i$ (100 µL, pH 7.0, c = 0.5 M), D_2O (175 µL), stock solutions of NaN_3 (75 µL), **TSP** (50 µL), and **GSH** (50 µL) were pipetted into an Eppendorf tube and mixed by pipetting the mixture up and down. Subsequently, 50 µL of a solution containing **VTFT** (0.25 M) and **$^{2}H_3$-VTFT** (0.25 M) in DMF-d_7 was added to the tube to obtain a mixture containing **GSH** (5.0 mM, 1.0 equiv.), **TSP** (5.0 mM, 1.0 equiv.), NaN_3 (150 mM, 30.0 equiv.), **VTFT** (25 mM, 5.0 equiv.), and **$^{2}H_3$-VTFT** (25 mM, 5.0 equiv.). The ^{1}H NMR spectrum of the mixture was measured 5 minutes after addition of the mixture containing **VTFT** and **$^{2}H_3$-VTFT**:

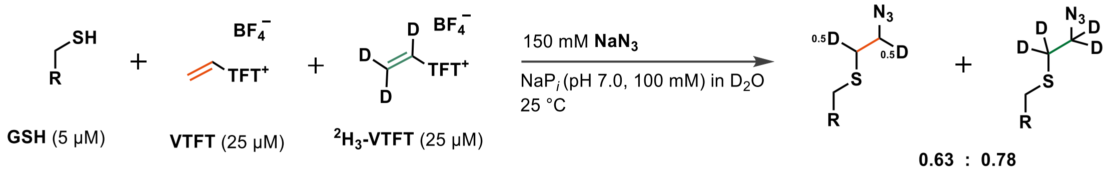

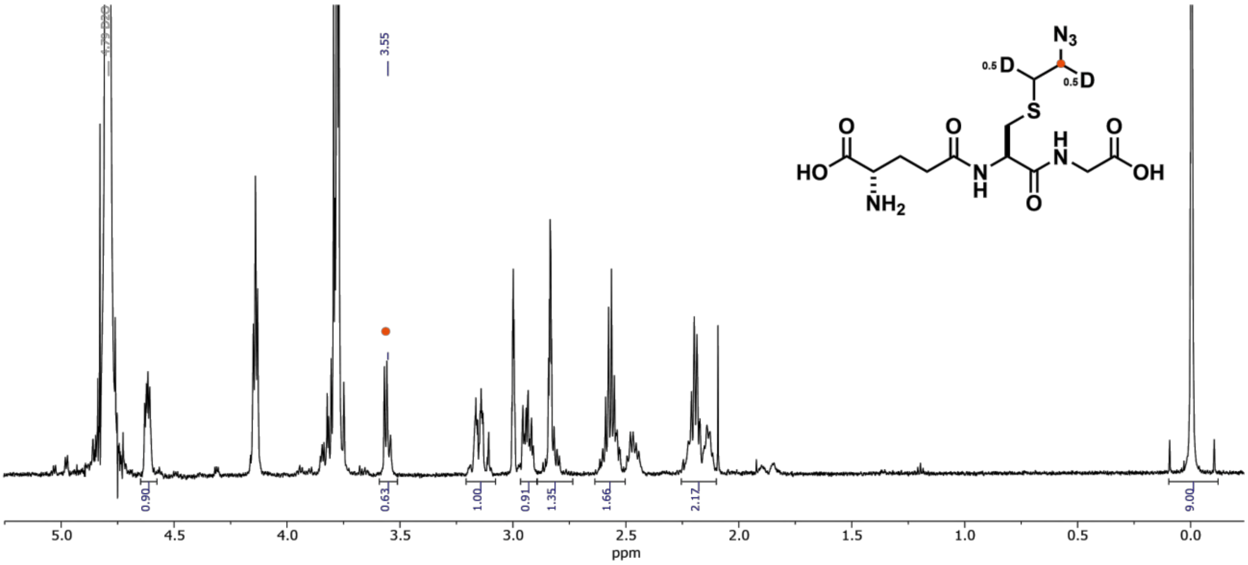

Figure S202. ^{1}H NMR spectrum of **GSH** (5.0 mM, 1.00 equiv.) in D-NaP$_i$ (pH 7.0, 50 mM) after addition of 5.0 equiv. **VTFT** and 5.0 equiv. **$^{2}H_3$-VTFT** in presence of 30.0 equiv. NaN_3 with TSP as internal standard.

The ratio of the obtained products formed in reaction with either **VTFT** or **$^{2}H_3$-VTFT** was determined by calculating the deuterium incorporation in the ethylene bridge for both reactions. The peak at 3.55 ppm corresponds to the methylene group alpha to the azide of the product obtained with **VTFT** (see Table S19) and was integrated for both spectra to calculate the ratio of the products formed with either **VTFT** or **$^{2}H_3$-VTFT**. Based on the integral values:

$$\frac{k_2(\mathbf{VTFT})}{k_2(^{2}\mathbf{H_3{-}VTFT})} = \frac{0.63}{1.41 - 0.63} = \frac{0.63}{0.78} = 0.81.$$

Deuterium incorporation in deuterated buffer

Reaction between **GSH**, NaN_3, and **VTT** or **VTFT** was performed in deuterated sodium phosphate buffer (D-NaP_i, pH 7.0, c = 100 mM). 3-(Trimethylsilyl)propionic-2,2,3,3-d_4 acid sodium salt (**TSP**) was used as an internal standard. The peaks at 3.58 ppm and 2.85 ppm corresponding to the introduced ethylene bridge were integrated to determine the deuterium incorporation in the product.

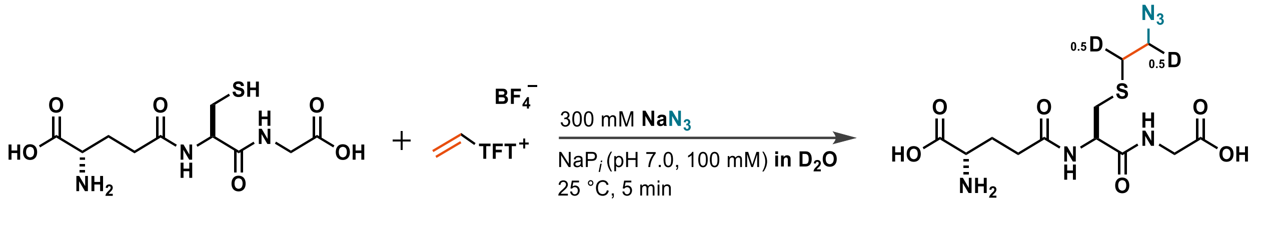

Stock solutions of **GSH** (50 mM), **TSP** (50 mM), and NaN_3 (1.5 M) were prepared in D_2O. D-NaP_i (100 µL, pH 7.0, c = 0.5 M), D_2O (95 µL), stock solutions of NaN_3 (100 µL), **TSP** (100 µL), and **GSH** (100 µL) were pipetted into an Eppendorf tube and mixed by pipetting the mixture up and down. Subsequently, a stock solution of **VTFT** in DMF-d_7 (5.0 µL, c = 1.0 M) was added to the tube to obtain a mixture containing **GSH** (10 mM, 1.0 equiv.), **TSP** (10 mM, 1.0 equiv.), NaN_3 (300 mM, 30.0 equiv.), and **VTFT** (10 mM, 1.0 equiv.). The ^1H NMR spectrum of the mixture was measured 5 minutes after addition of **VTFT**.

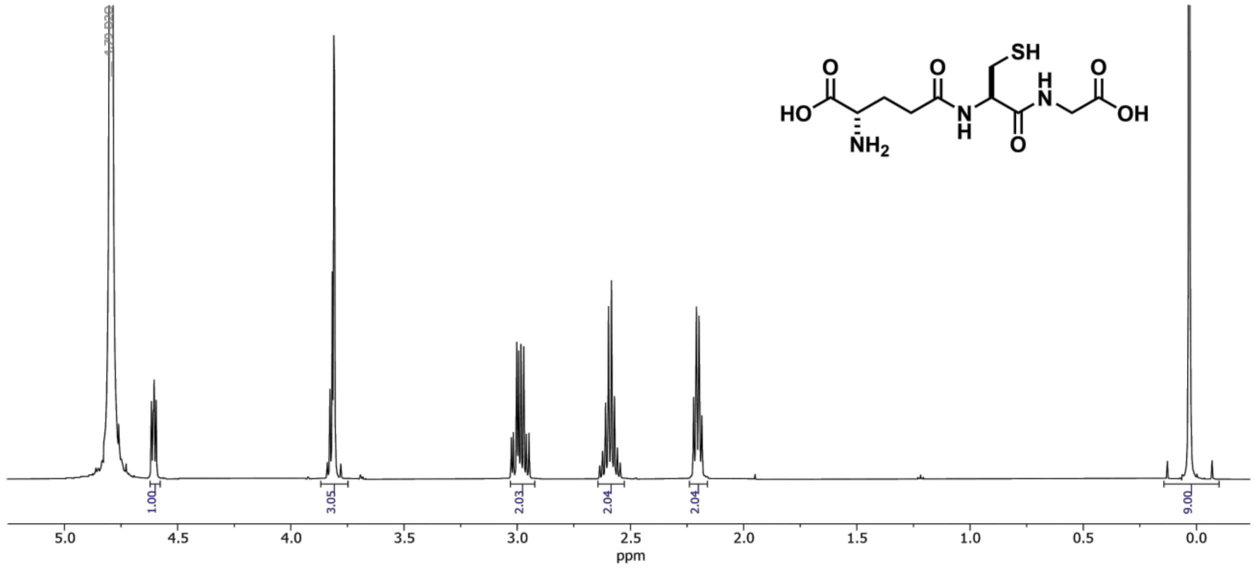

Figure S203. ^1H NMR spectrum of **GSH** (10 mM, 1.0 equiv.) in D-NaP_i (pH 7.0, 100 mM) in presence of sodium azide (300 mM, 30.0 equiv.) and **TSP** as internal standard.

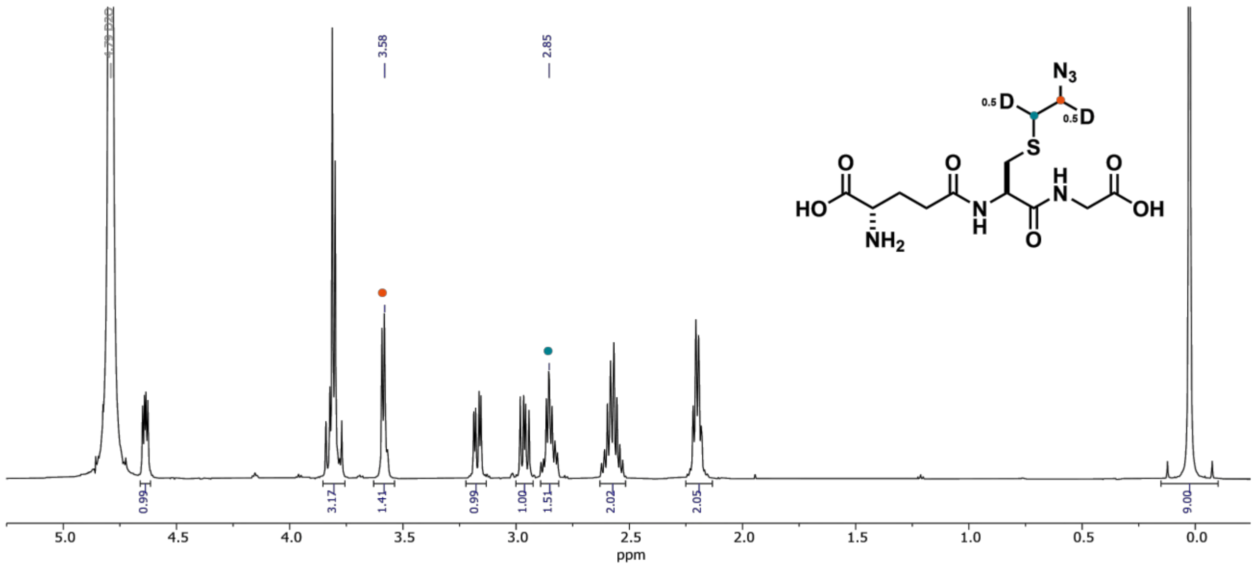

Figure S204. ^{1}H NMR spectrum of **GSH** (10 mM, 1.0 equiv.) in D-NaP$_i$ (pH 7.0, 100 mM) in presence of sodium azide (300 mM, 30.0 equiv.) and **TSP** as internal standard after addition of **VTFT** (10 mM, 1.0 equiv.).

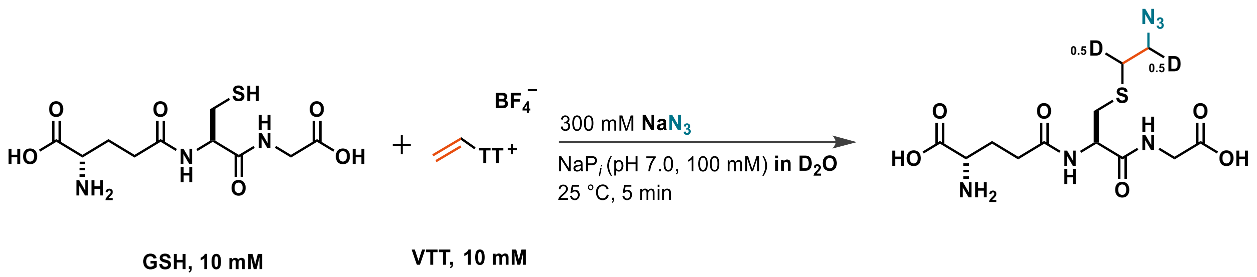

Stock solutions of **GSH** (50 mM), **TSP** (50 mM), and NaN_3 (1.5 M) were prepared in D_2O. D-NaP$_i$ (100 µL, pH 7.0, c = 0.5 M), D_2O (95 µL), stock solutions of NaN_3 (100 µL), **TSP** (100 µL), and **GSH** (100 µL) were pipetted into an Eppendorf tube and mixed by pipetting the mixture up and down. Subsequently, **VTT** stock solution in DMF-d_7 (5.0 µL, 1.0 M) was added to the tube to obtain a mixture containing **GSH** (10 mM, 1.0 equiv.), **TSP** (10 mM, 1.0 equiv.), NaN_3 (300 mM, 30.0 equiv.), and **VTT** (10 mM, 1.0 equiv.). The ^{1}H NMR spectrum of the mixture was measured 5 minutes after addition of **VTT**.

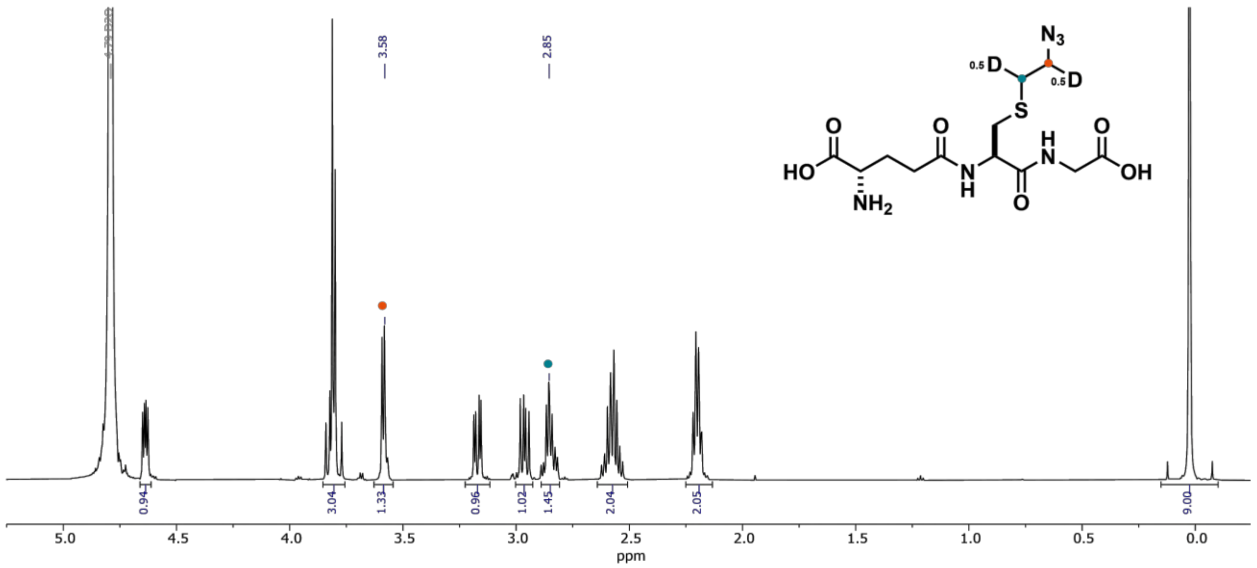

Figure S205. ^{1}H NMR spectrum of **GSH** (10 mM, 1.0 equiv.) in D-NaP$_i$ (pH 7.0, 100 mM) in presence of sodium azide (300 mM, 30.0 equiv.) and **TSP** (10 mM, 1.0 equiv.) as internal standard after addition of **VTT** (10 mM, 1.0 equiv.).

Reactivity of VTT and VTFT with NaN_3

The stability of vinylthianthrenium salts in the presence of excess NaN_3 was studied using NMR with 3-(trimethylsilyl)propionic-2,2,3,3-d$_4$ acid sodium salt (**TSP**) as an internal standard:

Stock solutions of **TSP** (50 mM) and NaN_3 (1.5 M) were prepared in D_2O. D-NaP$_i$ (100 µL, pH 7.0, c = 0.5 M), D_2O (195 µL), stock solutions of NaN_3 (100 µL) and **TSP** (100 µL) were pipetted into an Eppendorf tube and mixed by pipetting the mixture up and down. Subsequently, a stock solution of **VTT** or **VTFT** in DMF-d$_7$ (5.0 µL, c = 1.0 M) was added to the tube to obtain a mixture containing **VT(F)T** (10 mM, 1.0 equiv.), **TSP** (10 mM, 1.0 equiv.), and NaN_3 (0.3 M, 30 equiv.). The ^{1}H NMR spectrum of the mixture was measured 5 minutes after addition of **VT(F)T**.

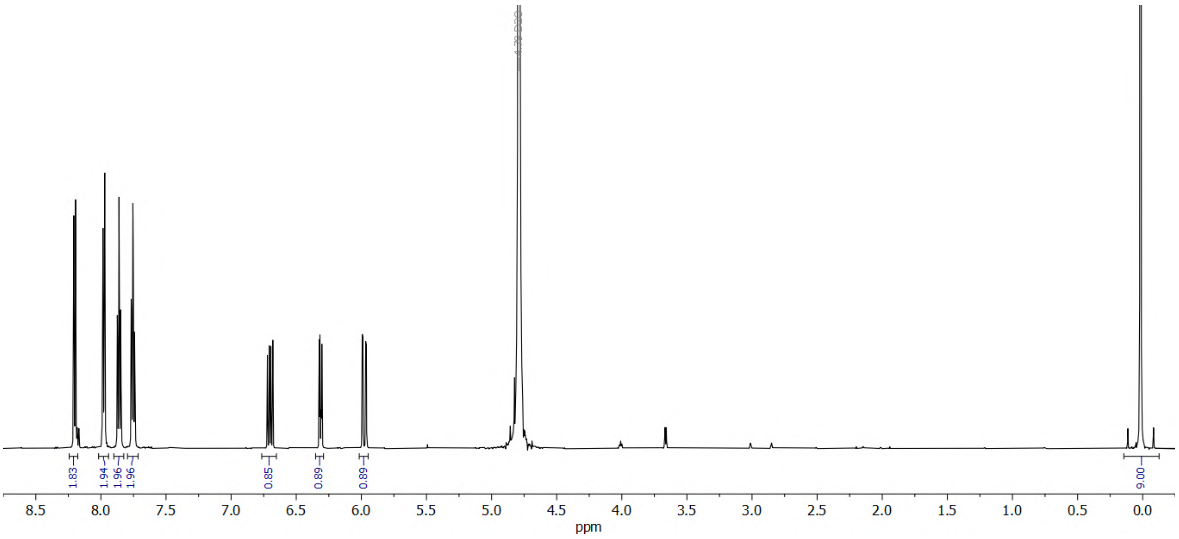

Figure S206. ^{1}H NMR spectrum of **VTT** (10 mM, 1.0 equiv.) in D-NaP$_i$ (pH 7.0, 100 mM) in presence of sodium azide (0.3 M, 30 equiv.) and **TSP** (10 mM, 1.0 equiv.) as internal standard.

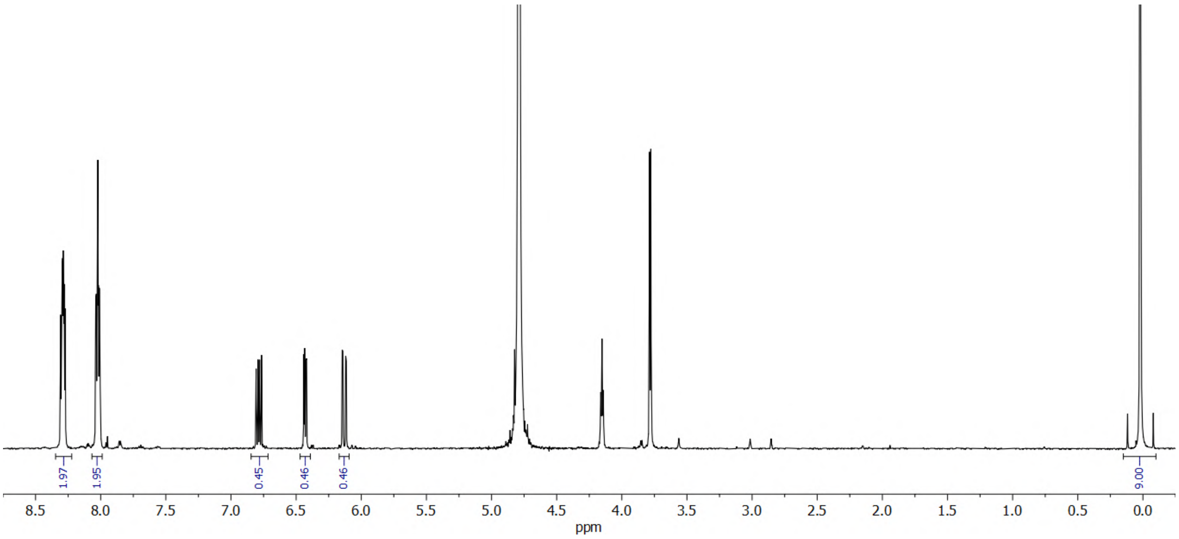

Figure S207. ^{1}H NMR spectrum of **VTFT** (10 mM, 1.0 equiv.) in D-NaP$_i$ (pH 7.0, 100 mM) in presence of sodium azide (0.3 M, 30 equiv.) and **TSP** (10 mM, 1.0 equiv.) as internal standard.

Proposed mechanism for GSH functionalization

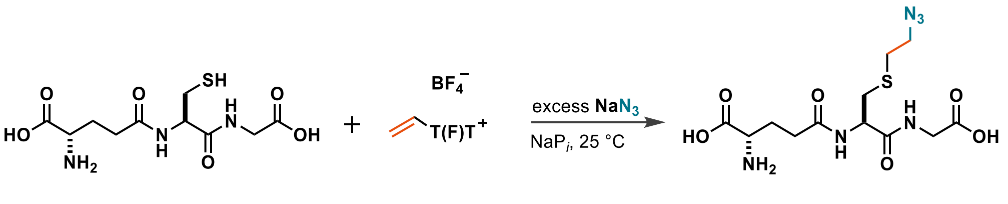

Incorporation of deuterium from deuterated phosphate buffer into both positions of the introduced ethylene bridge (Figure S203-Figure S205) is consistent with formation of a cyclic intermediate prior to addition of NaN_3, whereas direct substitution of the thianthrenium moiety by azide would lead to deuterium incorporation only in the position alpha to the azide (not observed):

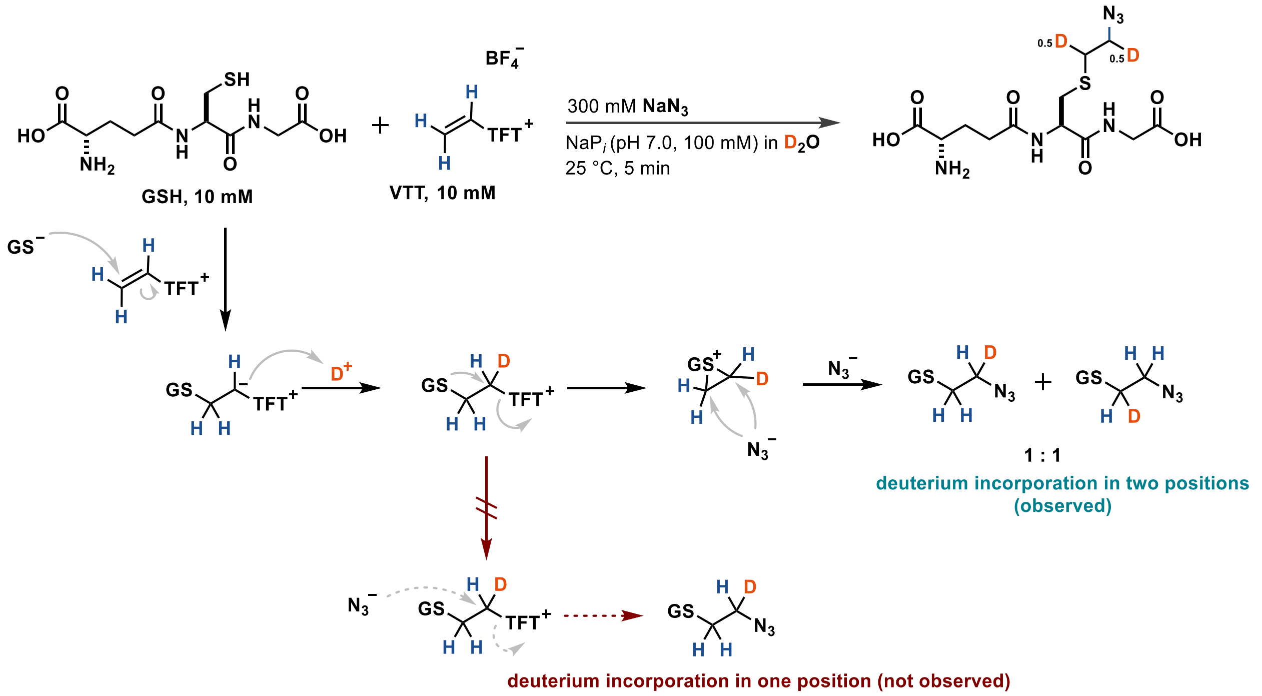

Figure S208. Proposed mechanism for functionalization of **GSH** with NaN_3 and **VTFT** in deuterated buffer.

Having obtained the data which is consistent with a formation of cyclic episulfonium intermediate prior to fast addition of NaN_3 (the reaction is 0th order in NaN_3), we performed the KIE measurements (see above) in order to distinguish between rate-determining **GSH**-addition and rate-determining episulfonium formation. The addition of **GSH** to the reactive sp^2-hybridized carbon atom of **VT(F)T** leads to the intermediate with sp^3-hybridization, whereas formation of the episulfonium leads to the small cyclic intermediate with a higher s-character at the C–H bonds (see Figure S209). The observed secondary inverse KIE is consistent with hybridization change from sp^2 to sp^3, in agreement with the rate-determining addition of the **GSH** to **VT(F)T**[30,31], and excludes rate-limiting episulfonium formation:

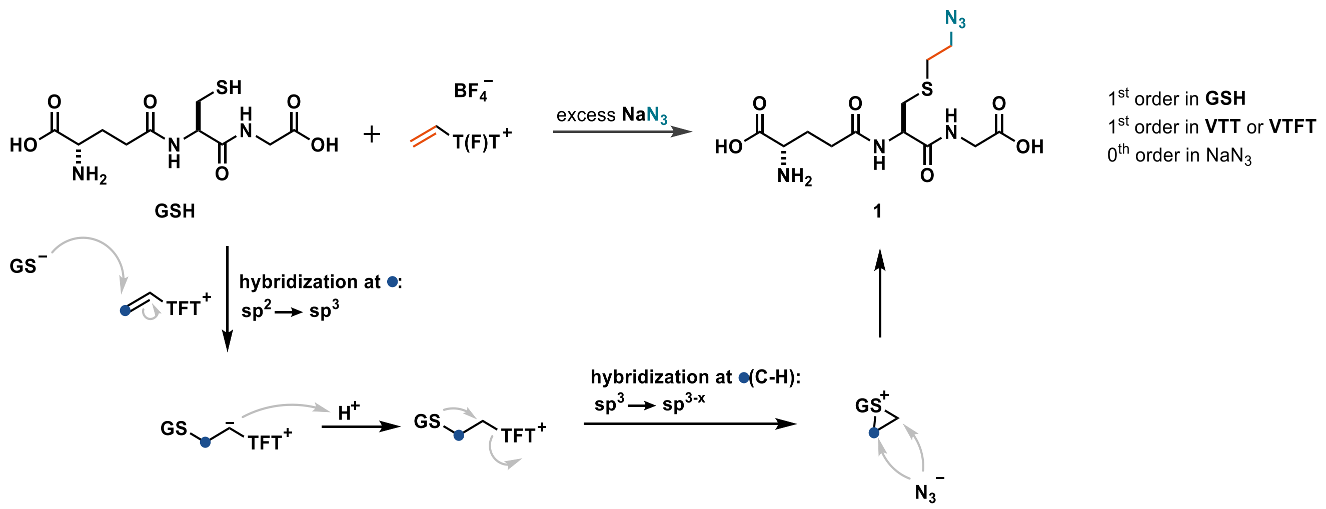

Figure S209. Proposed mechanism for functionalization of **GSH** with NaN₃ and **VTFT**.

Stability comparison with a maleimide conjugate

A quartz cuvette was charged with a Teflon-coated magnetic stirring bar. 1.4 mL of sodium phosphate buffer (pH 7.0, c = 50 mM) and 519 µL of MeCN were added to the cuvette, and the mixture was thermostated for 5 minutes at 37 °C in the cell holder of NanoDrop One C spectrophotometer. Then, a 3.7 mM stock solution of maleimide conjugate **S8** in MeCN (81 µL) was added to the mixture to obtain a buffered solution containing 0.15 mM of the maleimide conjugate. The absorbance at 410 nm was measured for 5 hours while stirring at 37 °C to detect the release of 4-nitrothiophenolate. The same measurement was performed with a solution containing 0.15 mM of thiophenol-derived azide **S9**.

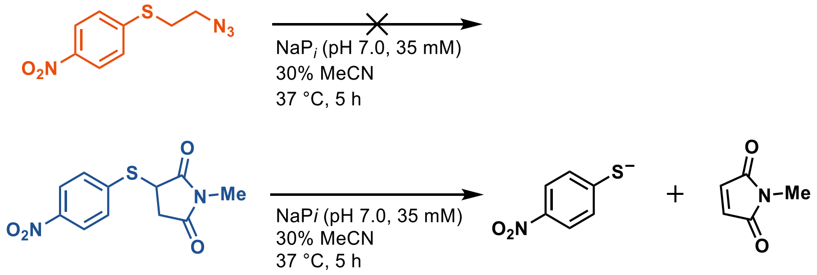

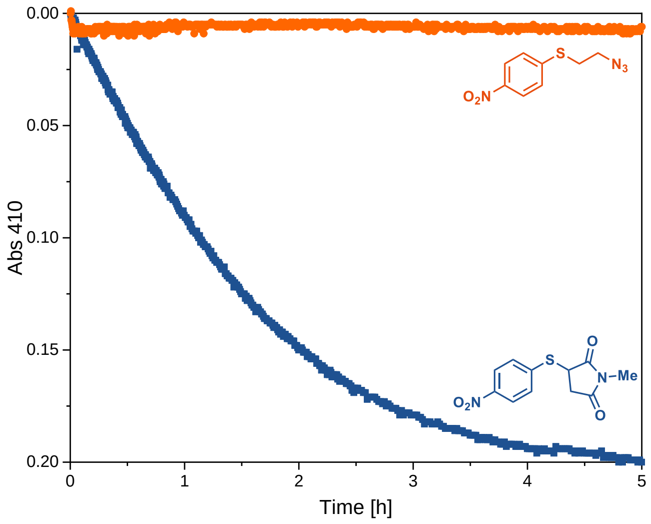

Figure S210. Change of absorbance at 410 nm corresponding to the release of p-nitrothiophenolate.

Characterization of the in-situ generated iodide-intermediate

HRMS-characterization

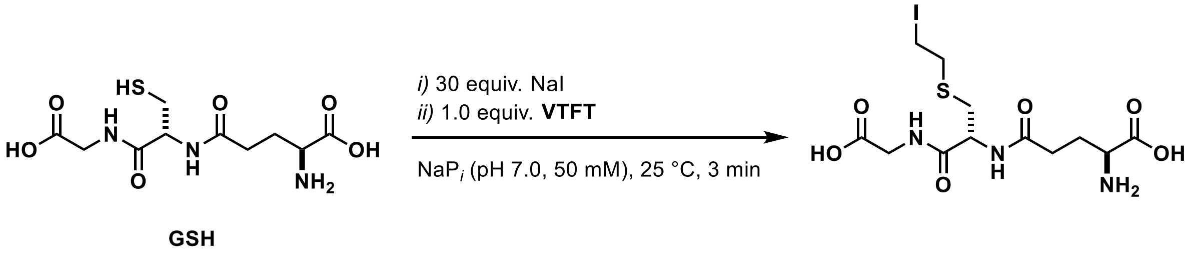

At 20–25 °C, NaP$_i$ (322 µL, pH 7.0, c = 50 mM) was mixed with freshly prepared stock solutions of glutathione (100 µL, 2.5 µmol, 1.0 equiv., c = 25 mM) and sodium iodide (75 µL, 75 µmol, 30 equiv., c = 1.0 M) in NaP$_i$ in a 1.5 mL Eppendorf tube. The mixture was mixed with a pipette and a 1.0 M solution of **VTFT** in DMF (2.5 µL, 2.5 µmol, 1.0 equiv.) was added to the mixture. The reaction mixture was vortexed for 1 minute and filtered through a syringe filter. The filtrate was diluted with UHQ-H_2O and used for the HRMS-measurement.

23.09.2022 12:16 p.1/2 *** Angegebene Mol.-Gewichte u. Massenzahlen basieren auf dem häufigsten Isotop der Elemente *** **MassLib**

```
ESI pos
Characteristic ions (singly charged):
315 = F4-thianthryl cation
352 = [351 + H]+ (C12H21O7N3S, hydrolyzed product)
374 = [351 + Na]+
366 = [365 + H]+ (C13H23O7N3S)
388 = [365 + Na]+
462 = [461 + H]+ (C12H20O6N3SI)
488 = [461 + Na]+

et al.

HRMS for 462 see below
```

```
23.09.2022
File: E39433b-00.RAW
Analyse: BOO-BA-403-01
RIV: Kostiantyn Bohdan

Messung:          API-MS
Ionisierung:      ESIpos+neg
Lösungsmittel:    ACN+H2O
Spektrometer:     Q Exactive Plus Orbitrap

Auswerter:        Vetere (2234)
```

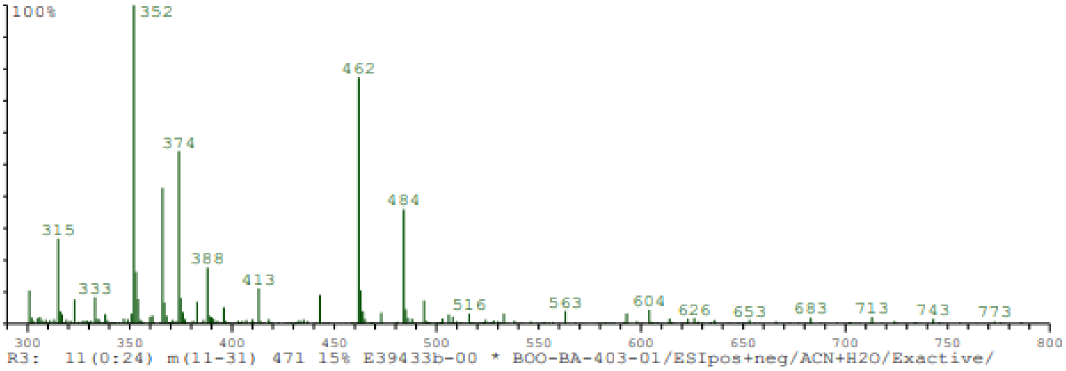

R3: 11(0:24) m(11-31) 471 15% E39433b-00 * BOO-BA-403-01/ESIpos+neg/ACN+H2O/Exactive/

Mass to be matched (m/z): 462.019010 Charge: 1

Mass Tolerance: ±0.001000
Restriction of atom numbers:

C	H	N	O	I	S
		max 5	max 10	max 1	

Number of calculated Formulas: 13

Formula	Diff.(ppm)	theor. m/z
C12 H21 N3 O6 I1 S1	0.05	462.019031
C12 H22 N4 O5 S5	-0.38	462.018833
C26 H12 N3 S3	-0.48	462.018789
C27 H10 O6 S1	0.55	462.019263
C19 H14 N2 O8 S2	-0.86	462.018613
C33 H4 N1 O3	-0.95	462.018569
C20 H18 N2 O3 S4	1.02	462.019483
C12 H30 O4 S7	-1.39	462.018368
C20 H17 N1 O4 I1	1.45	462.019681
C18 H15 N4 O3 I1	-1.46	462.018337
C13 H26 N4 S7	1.50	462.019703
C18 H16 N5 O2 S4	-1.89	462.018139
C13 H25 N3 O1 I1 S3	1.93	462.019901

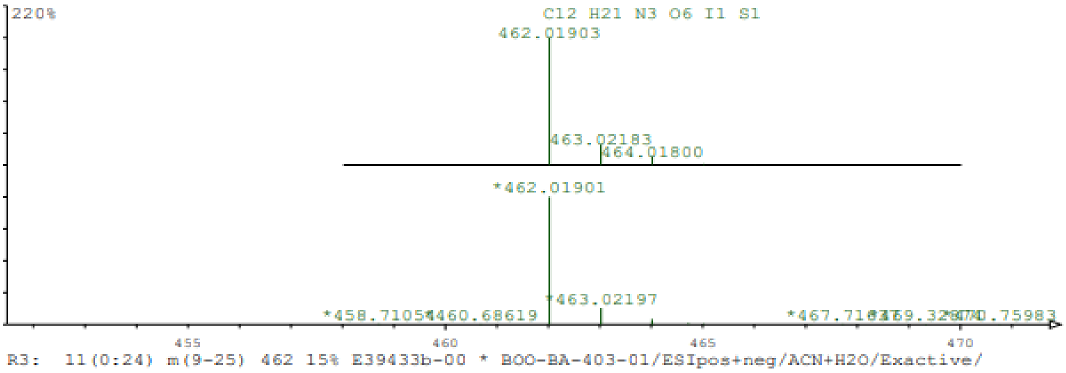

R3: 11(0:24) m(9-25) 462 15% E39433b-00 * BOO-BA-403-01/ESIpos+neg/ACN+H2O/Exactive/

MassLib V9.4 *** If not stated otherwise, molecular weights refer to the most abundant isotopes of the elements. *** MPI für Kohlenforschung

Figure S211. HMRS analysis of the glutathione-derived iodide intermediate.

NMR-characterization

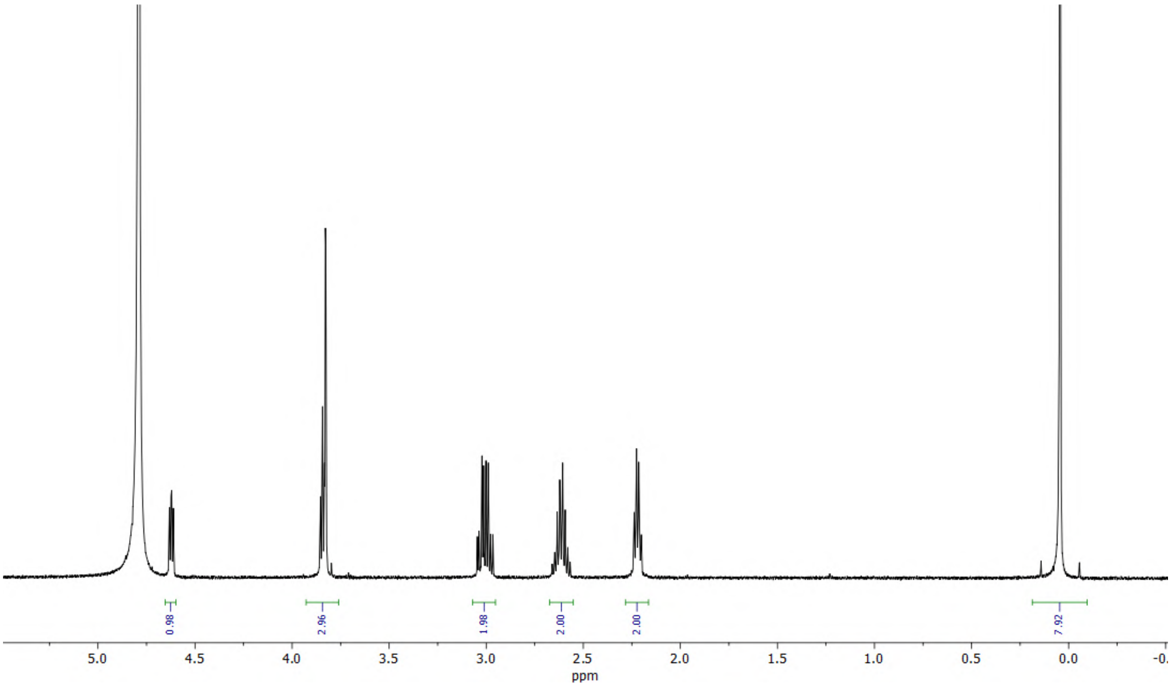

Figure S212. ^{1}H NMR spectrum of **GSH** (10 mM, 1.0 equiv) in NaP$_i$ (pH 8.0, 100 mM) in presence of 30.0 equiv. NaI and 1.0 equiv. of **TSP** as internal standard.

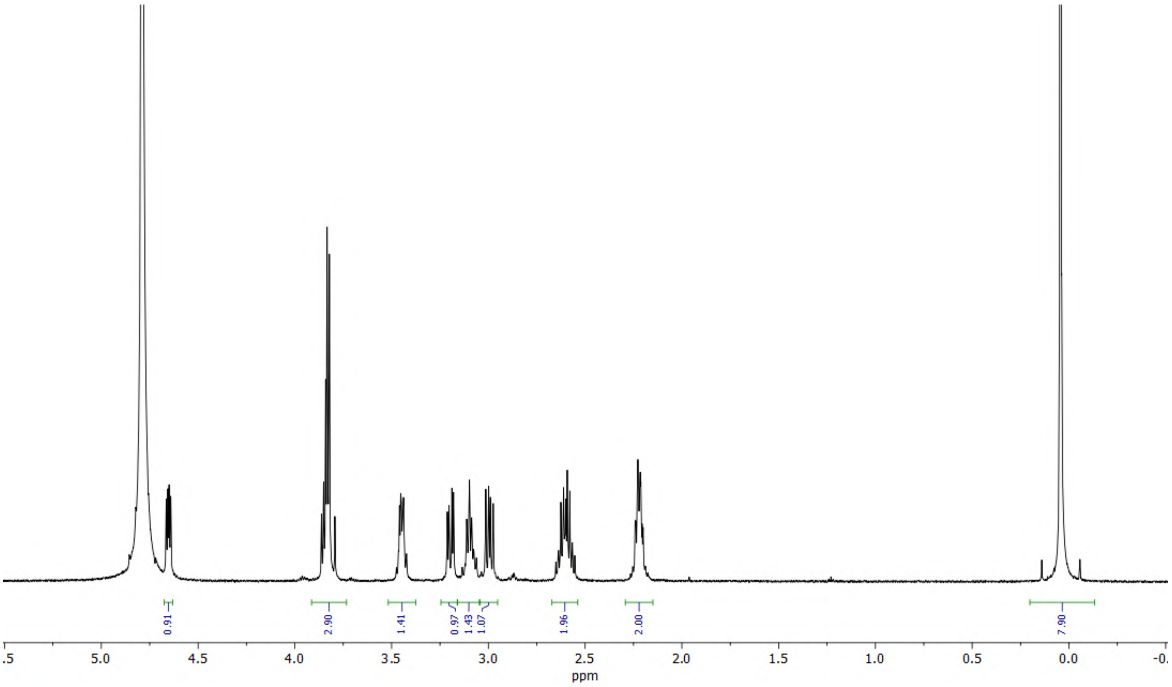

Figure S213. ^{1}H NMR spectrum of **GSH** (10 mM, 1.0 equiv.) in NaP$_i$ (pH 8.0, 100 mM) in presence of 30 equiv. NaI and 1.0 equiv. of **TSP** as internal standard after addition of **VTT** (10 mM; 1.0 equiv.).

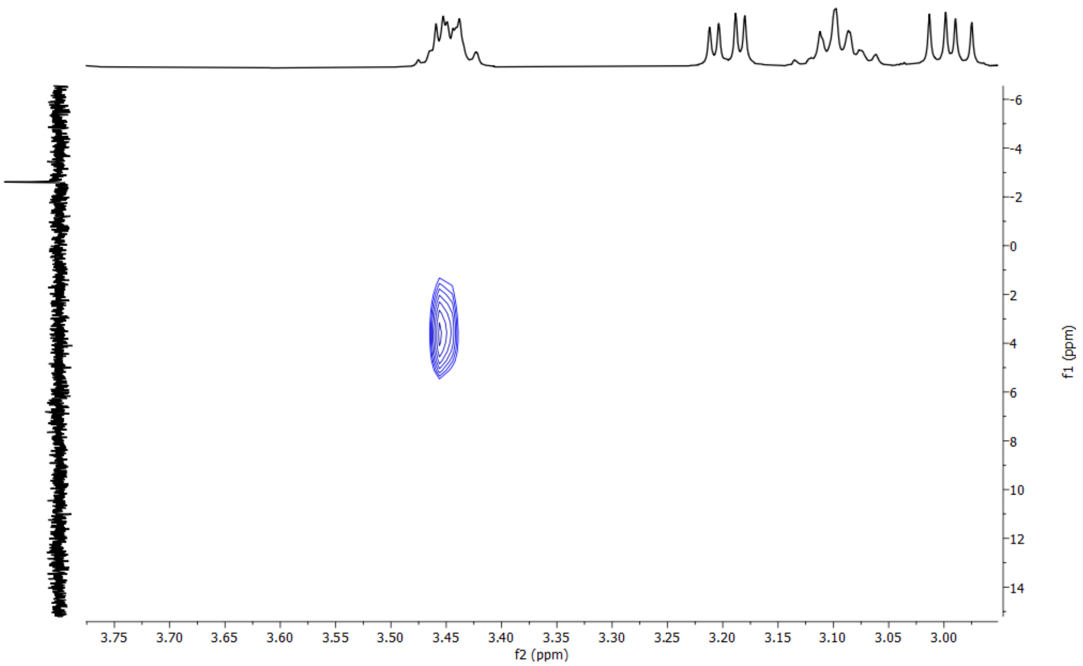

Figure S214. HSQC NMR spectrum of **GSH** (10 mM, 1.0 equiv.) in NaP$_i$ (pH 8.0, 100 mM) after addition of **VTT** (10 mM; 1.0 equiv.) in presence of 30 equiv. NaI and 1.0 equiv. of **TSP** as internal standard. Zoom onto the putative alpha carbon cross peak carrying the iodide.

NMR Experiments with Glutathione

Reactions between **GSH**, **VTT**, and external nucleophiles was performed in deuterated glycine amide buffer (H-Gly-NH_2, pH 7.4, c = 0.1 M) with sodium formate as an internal standard to verify the full conversion to the desired product prior to purification (for full characterization of the conjugates with glutathione or cysteine, see data on the isolated products **1**, **S1** – **S7**).

Stock solutions of **GSH** (0.10 M), internal standard (0.10 M), and nucleophile (0.5 – 1.0 M) were prepared in D_2O. H-Gly-NH_2 in D_2O (pH 7.4, c = 0.5 M), D_2O, stock solutions of nucleophile, sodium formate (30 µL), and **GSH** (30 µL) were pipetted into an Eppendorf tube and mixed by pipetting the mixture up and down. Subsequently, the mixture was transferred into a NMR tube and ^1H spectrum was measured. Next, a stock solution of **VTT** in DMF-d_7 (3.0 µL, c = 1.0 M) was added to the tube, and the mixture was mixed prior to the measurement. The ^1H NMR spectrum of the mixture was measured 5 minutes after addition of **VTT**.

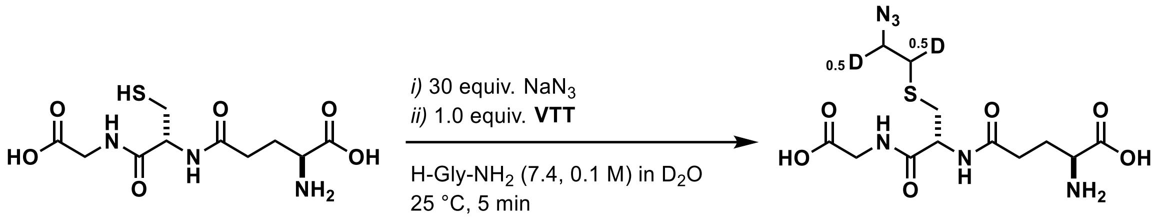

GSH

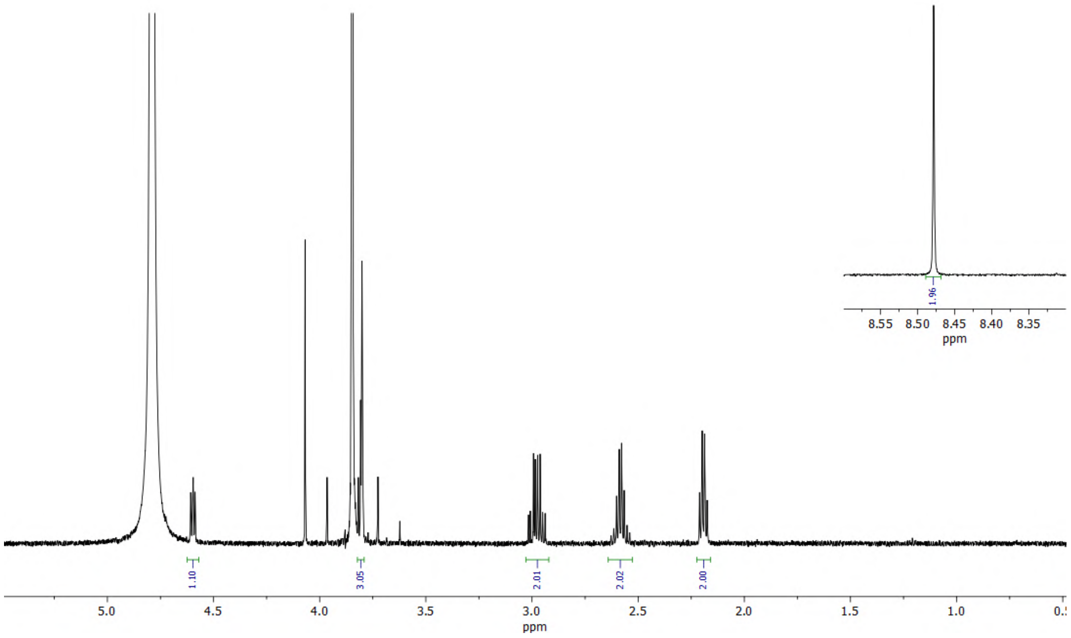

Figure S215. ^1H NMR spectrum of GSH in H-Gly-NH_2 buffer (pH 7.4, 100 mM) prior to addition of **VTT** in presence of 30 equiv. NaN_3. 2 equiv. sodium formate are used as an internal standard.

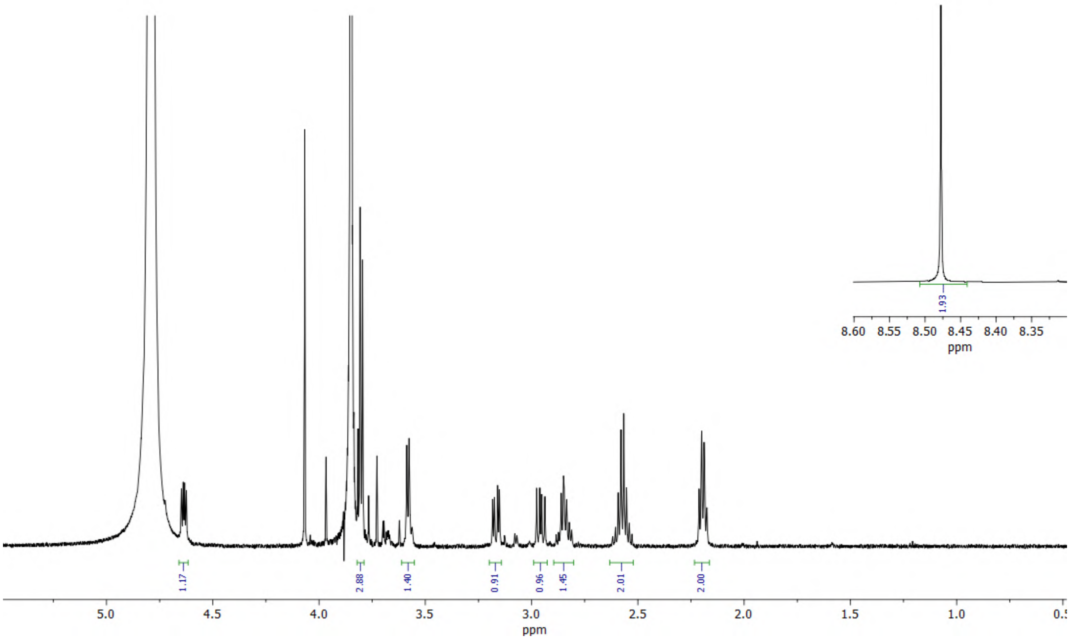

Figure S216. ^{1}H NMR spectrum of GSH in H-Gly-NH_2 buffer (pH 7.4, 100 mM) after addition of **VTT** in presence of 30 equiv. NaN_3. 2 equiv. sodium formate are used as an internal standard.

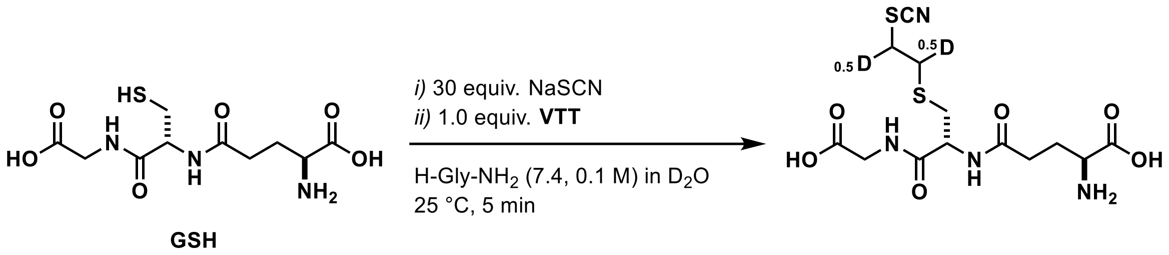

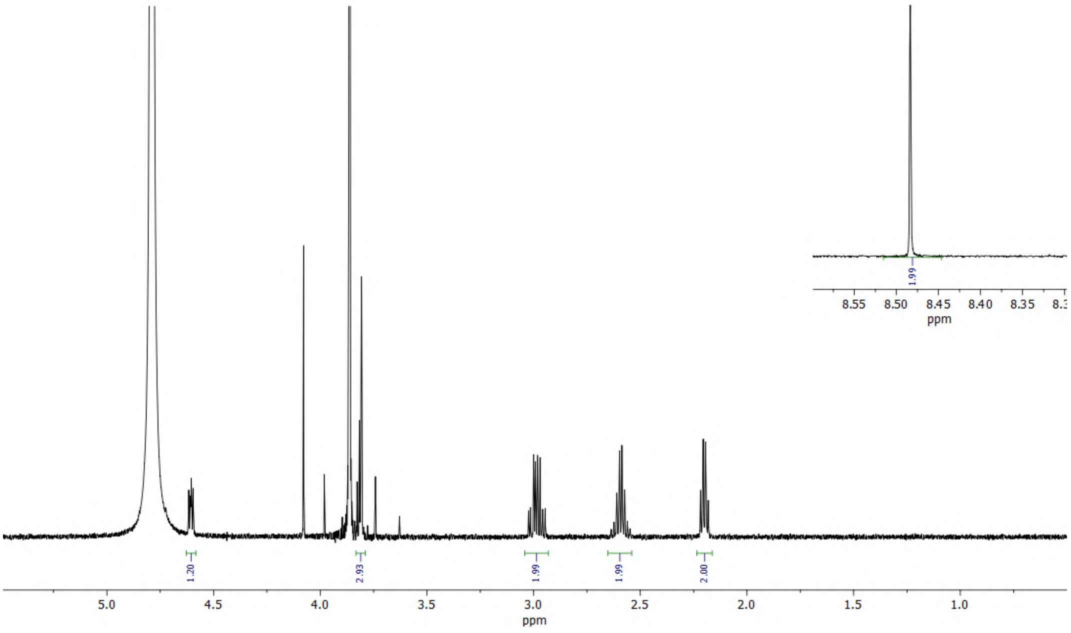

Figure S217. ^{1}H NMR spectrum of GSH in H-Gly-NH_2 buffer (pH 7.4, 100 mM) prior addition of **VTT** in presence of 30 equiv. NaSCN. 2 equiv. sodium formate are used as an internal standard.

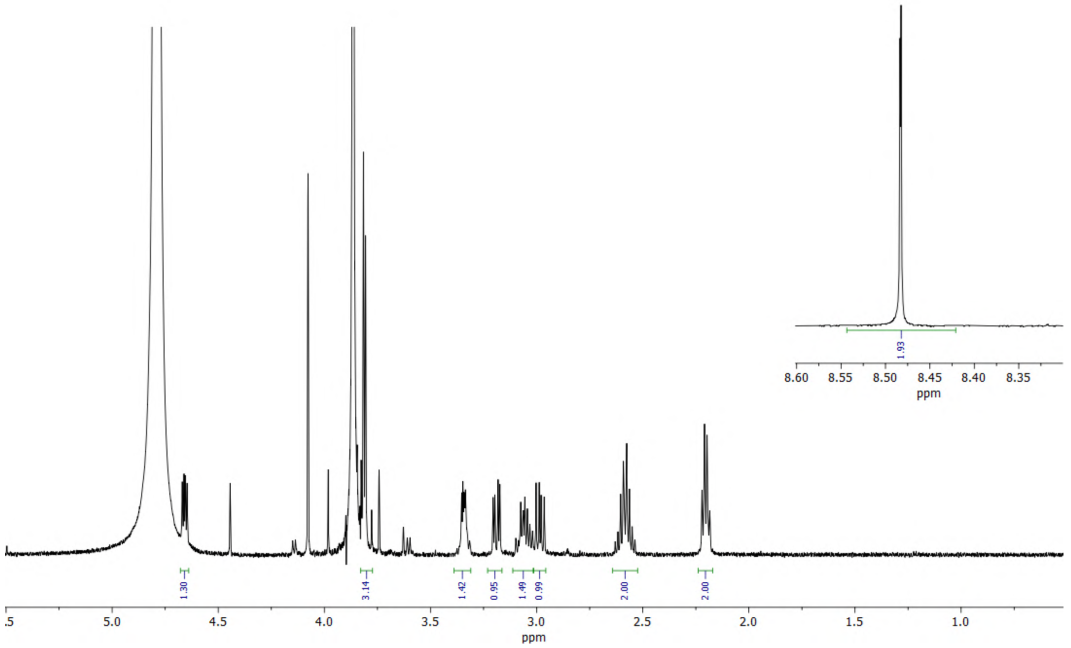

Figure S218. ^{1}H NMR spectrum of GSH in H-Gly-NH_2 buffer (pH 7.4, 100 mM) after addition of **VTT** in presence of 30 equiv. NaSCN. 2 equiv. sodium formate are used as an internal standard.

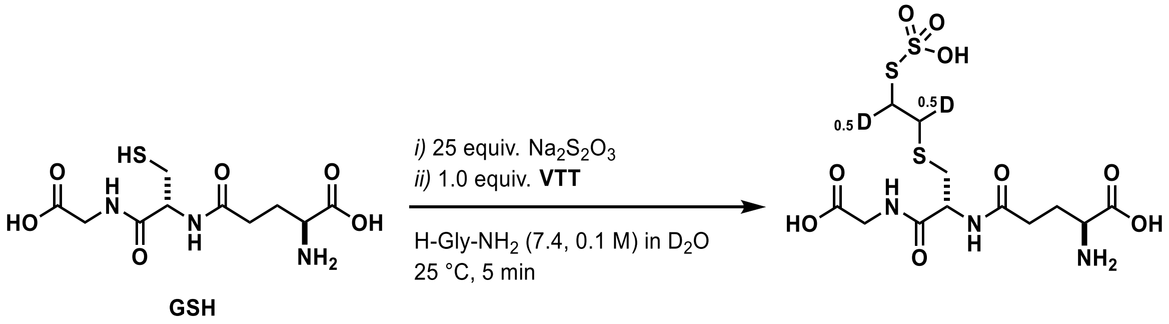

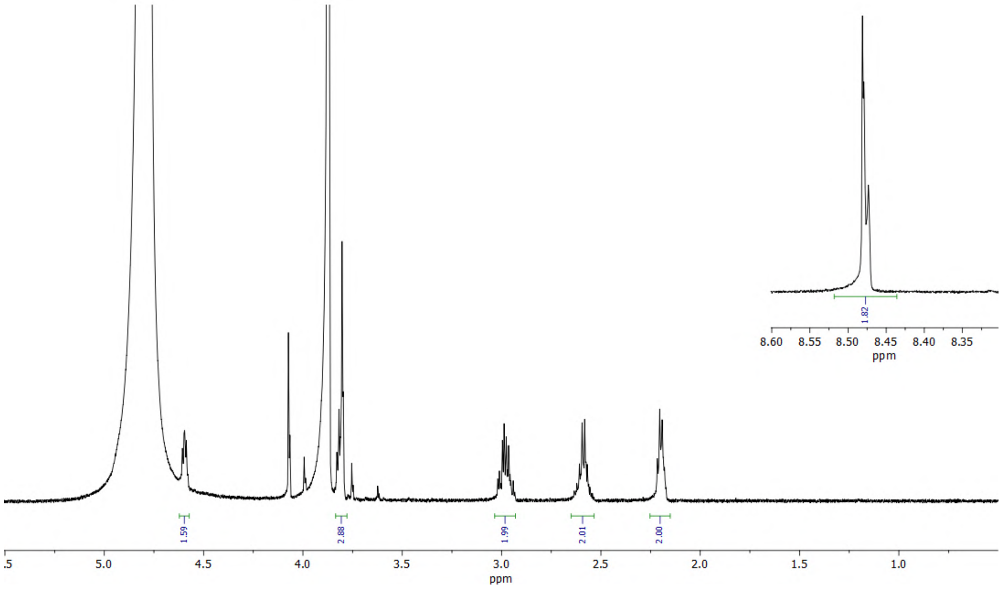

Figure S219. ^{1}H NMR spectrum of GSH in H-Gly-NH_2 buffer (pH 7.4, 100 mM) prior to addition of **VTT** in presence of 25 equiv. sodium thiosulfate. 2 equiv. sodium formate are used as an internal standard.

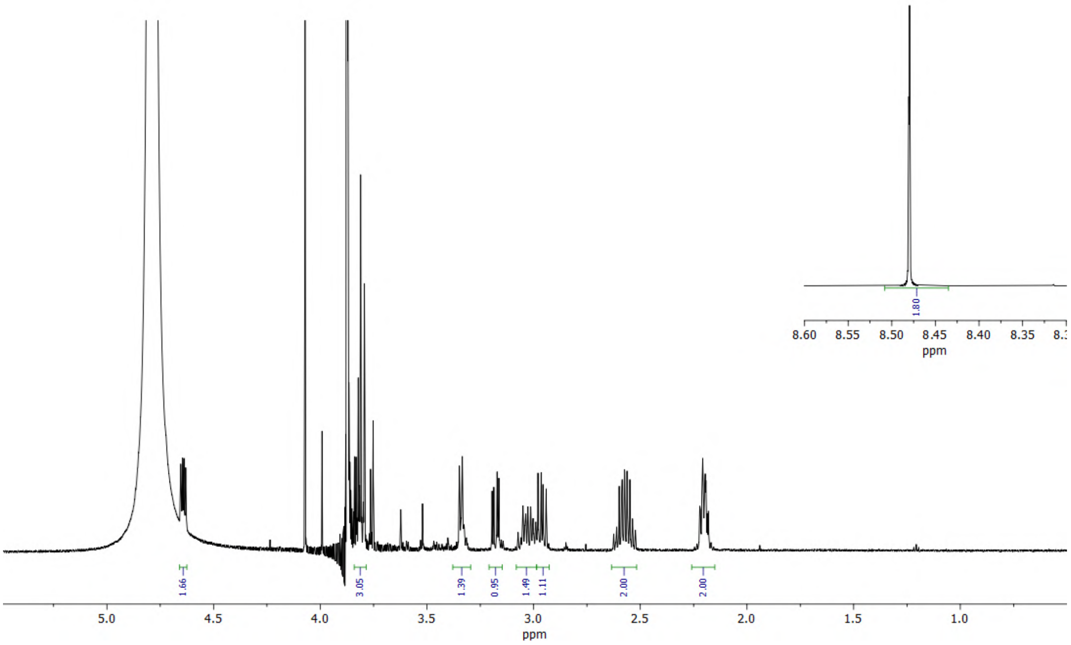

Figure S220. ^{1}H NMR spectrum of GSH in H-Gly-NH_2 buffer (pH 7.4, 100 mM) after addition of **VTT** in presence of 25 equiv. sodium thiosulfate. 2 equiv. sodium formate are used as an internal standard.

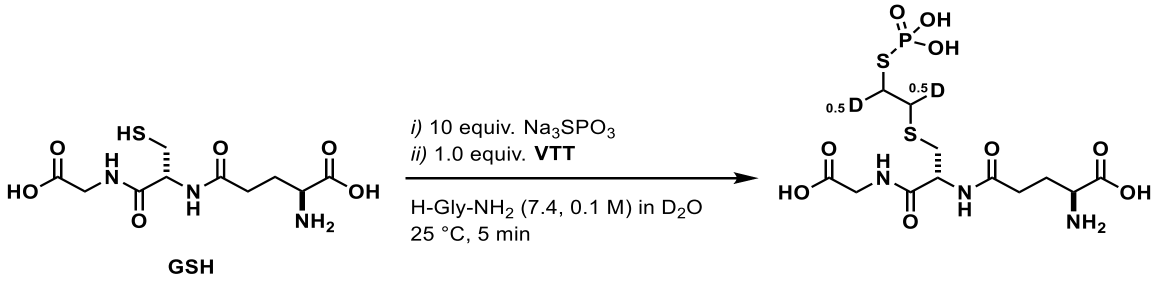

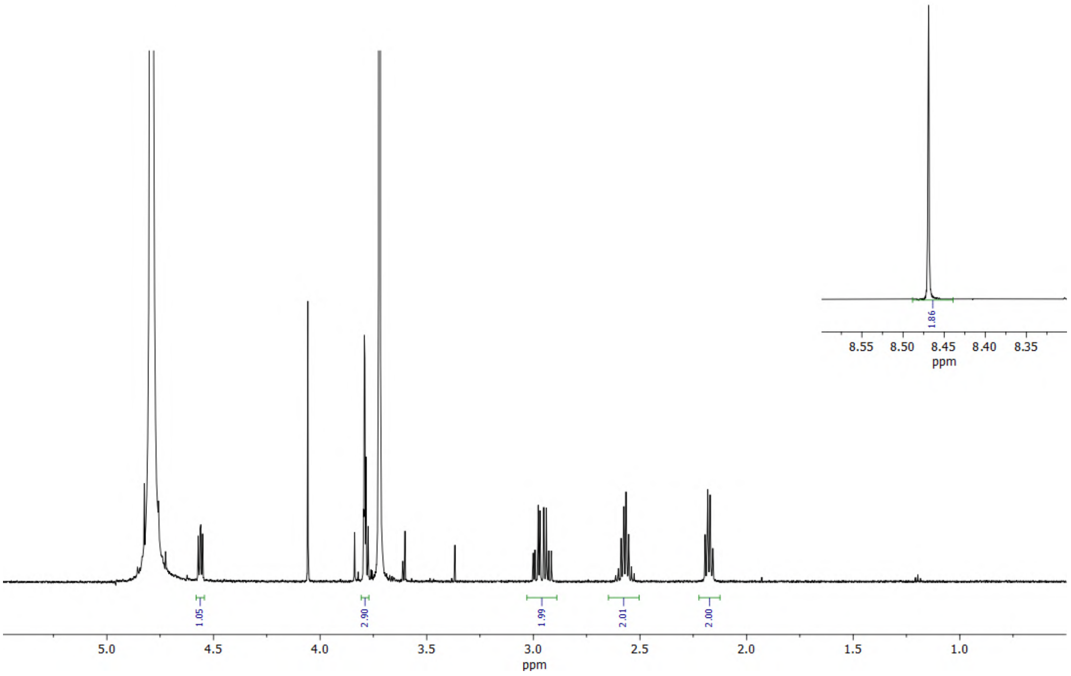

Figure S221. ^{1}H NMR spectrum of GSH in H-Gly-NH_2 buffer (pH 7.4, 100 mM) prior to addition of **VTT** in presence of 10 equiv. sodium thiophosphate. 2 equiv. sodium formate are used as an internal standard.

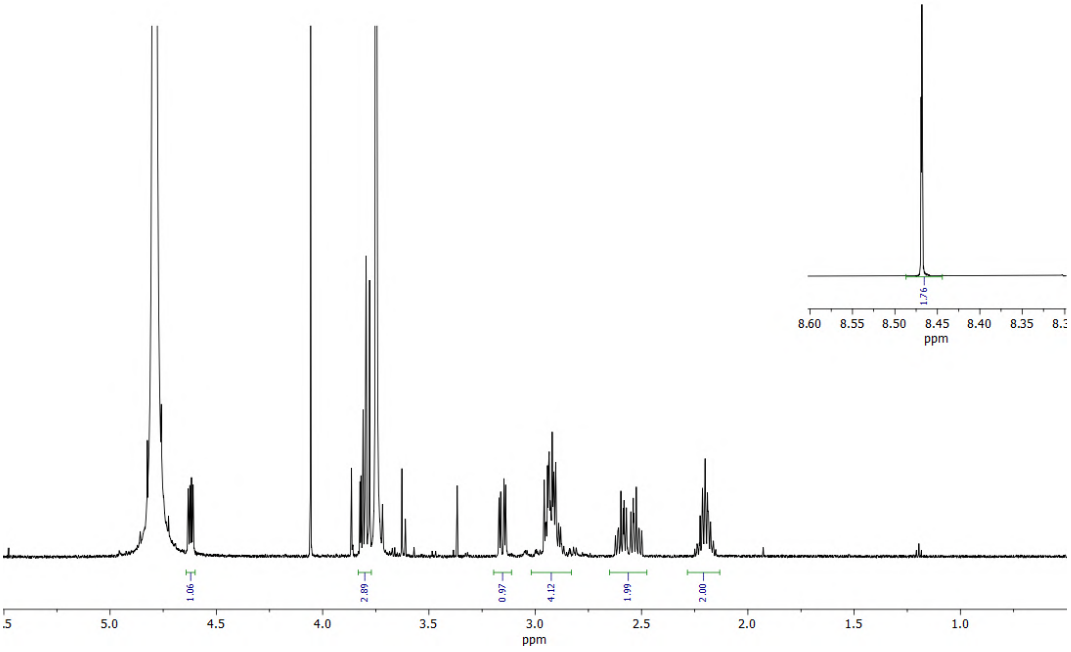

Figure S222. ^{1}H NMR spectrum of GSH in H-Gly-NH_2 buffer (pH 7.4, 100 mM) after addition of **VTT** in presence of 10 equiv. sodium thiophosphate. 2 equiv. sodium formate are used as an internal standard.

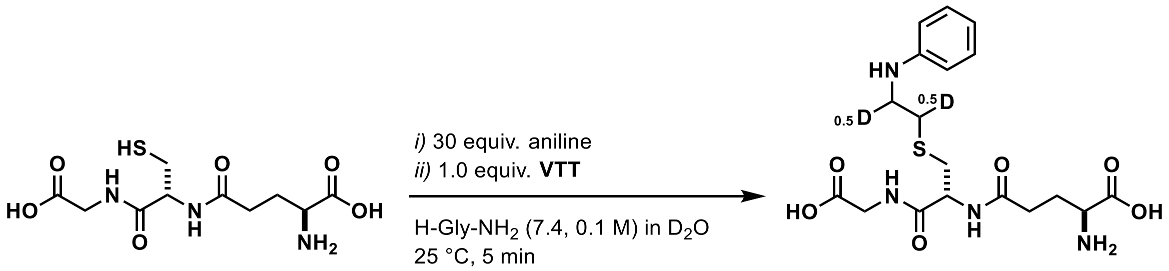

GSH

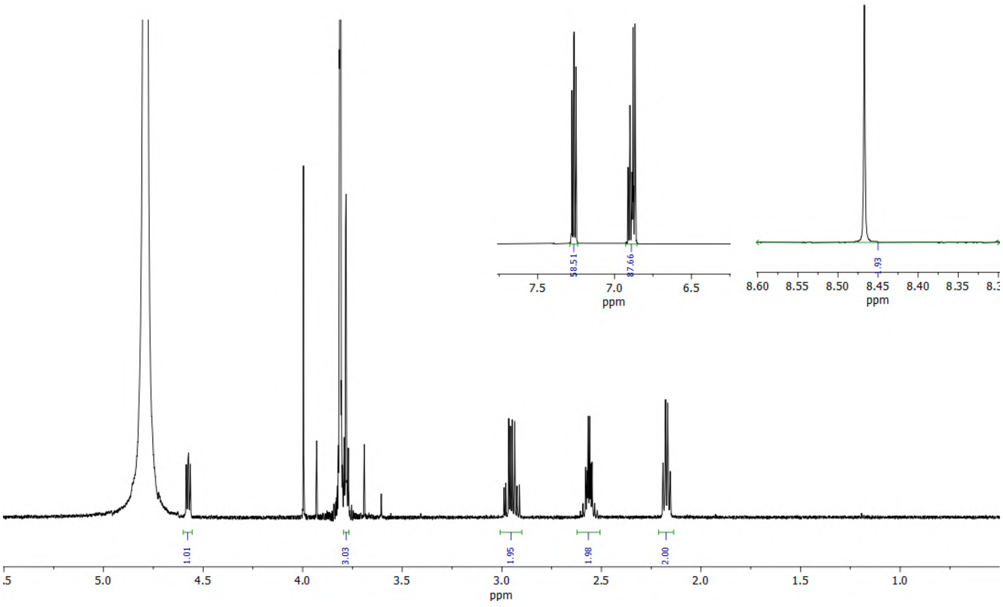

Figure S223. ^{1}H NMR spectrum of GSH in H-Gly-NH_2 buffer (pH 7.4, 100 mM) prior to addition of **VTT** in presence of 30 equiv. aniline. 2 equiv. sodium formate are used as an internal standard.

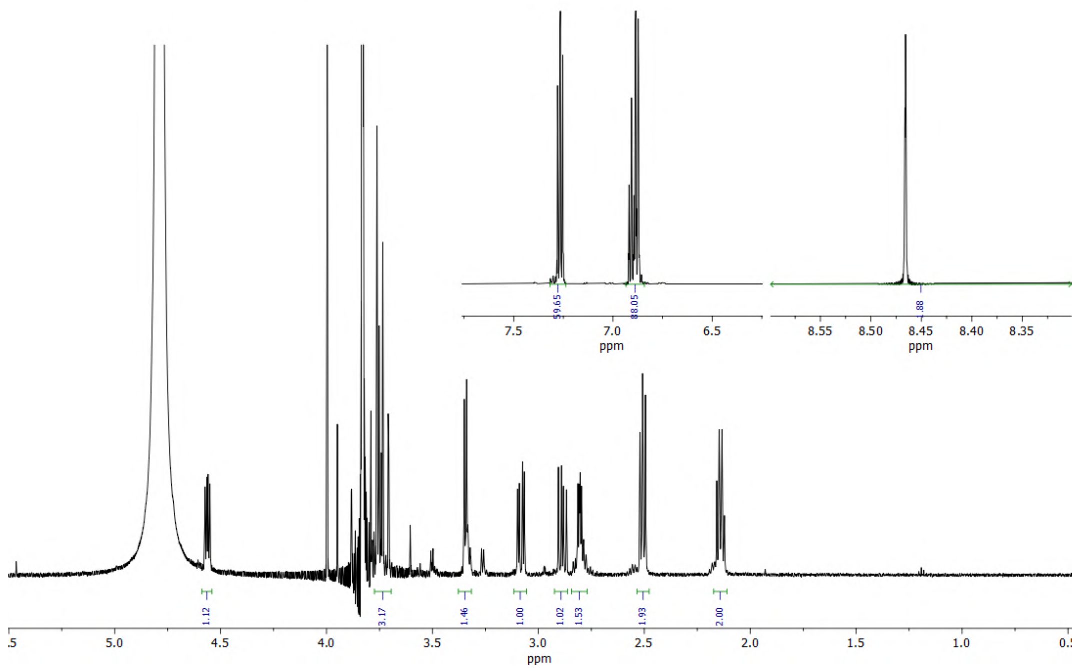

Figure S224. ^{1}H NMR spectrum of GSH in H-Gly-NH_2 buffer (pH 7.4, 100 mM) after addition of **VTT** in presence of 30 equiv. aniline. 2 equiv. sodium formate are used as an internal standard.

NMR DATA

NMR-Characterization of vinyl-thianthrenium reagents

^{1}H NMR of **VTFT**
600 MHz, CD_3CN, 298 K

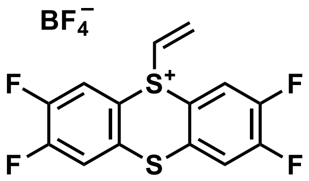

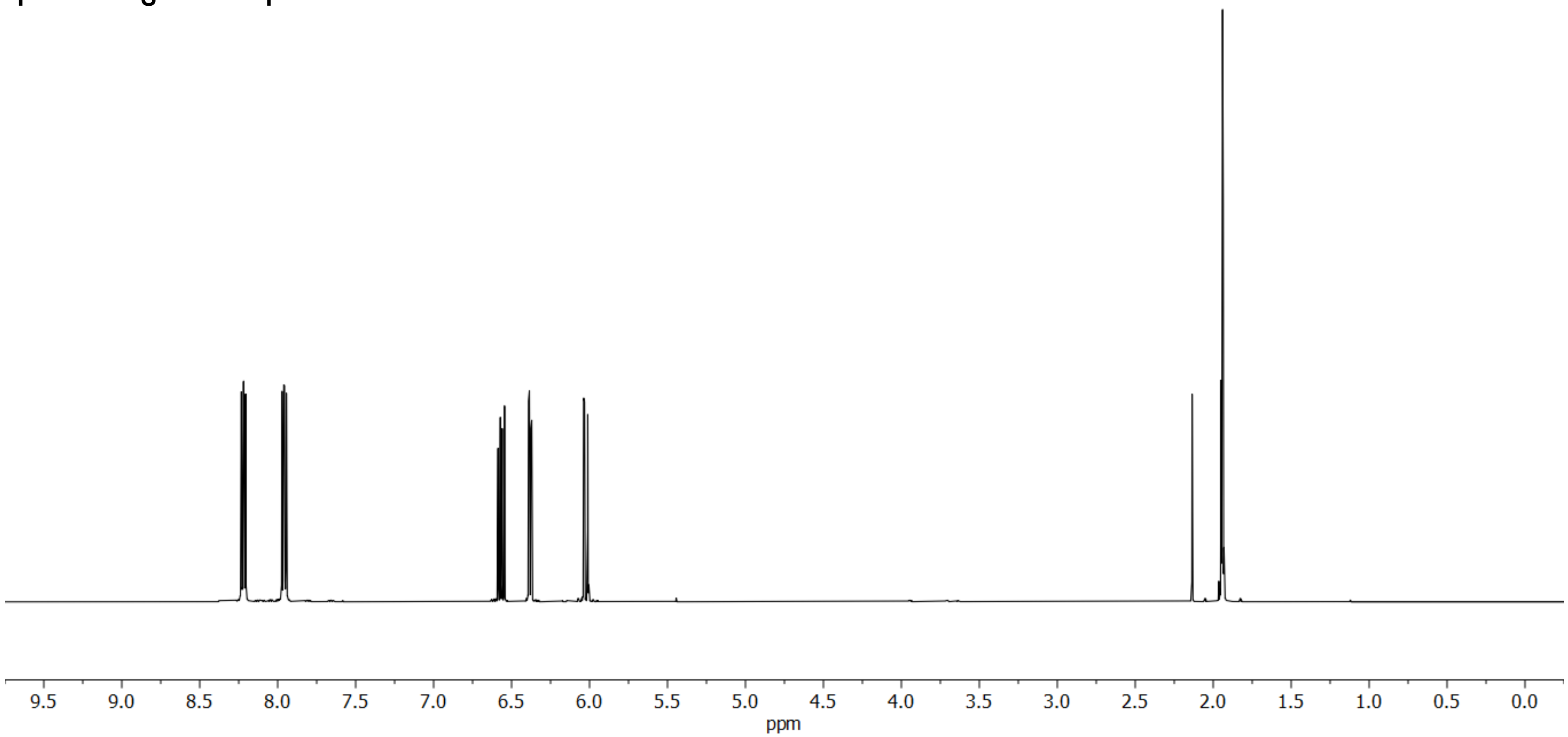

^{13}C NMR of **VTFT**
151 MHz, CD_3CN, 298 K

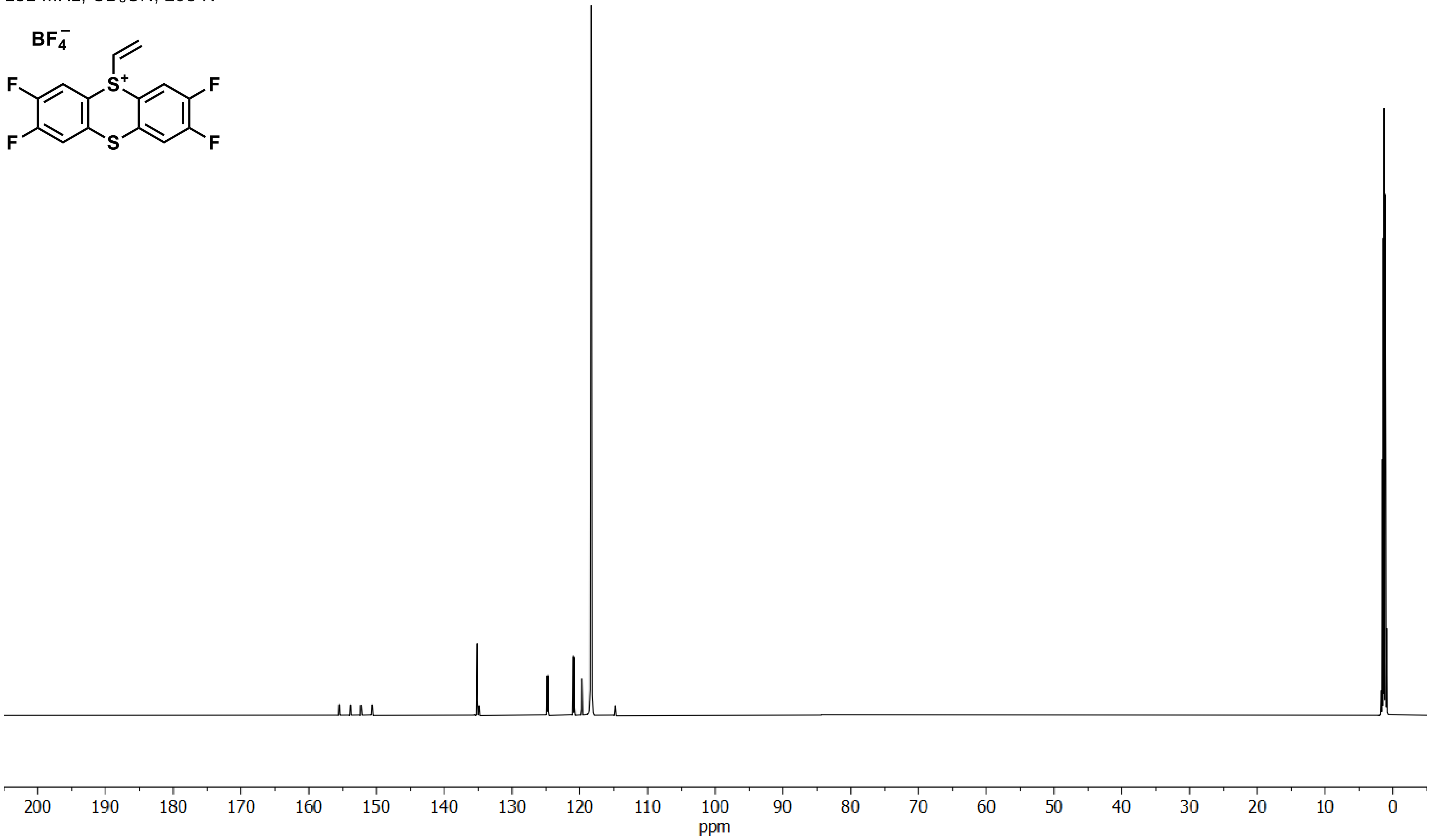

^{19}F NMR of **VTFT**
471 MHz, CD_3CN, 298 K

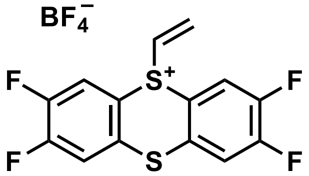

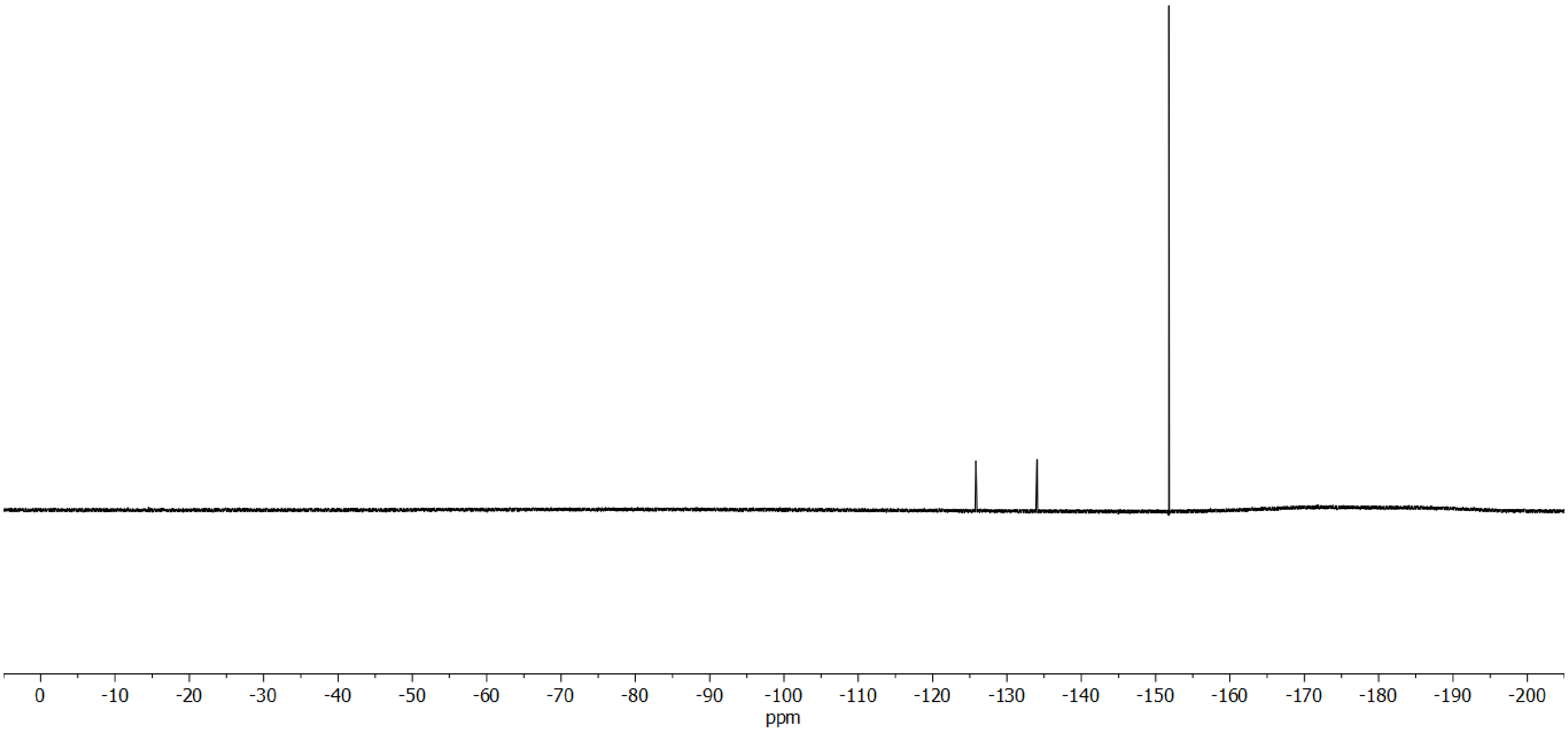

^{1}H NMR of **^{2}H$_{3}$-VTT**
500 MHz, CD_3CN, 298 K

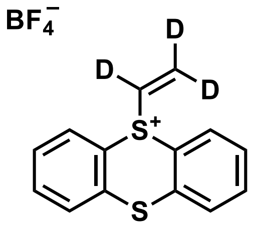

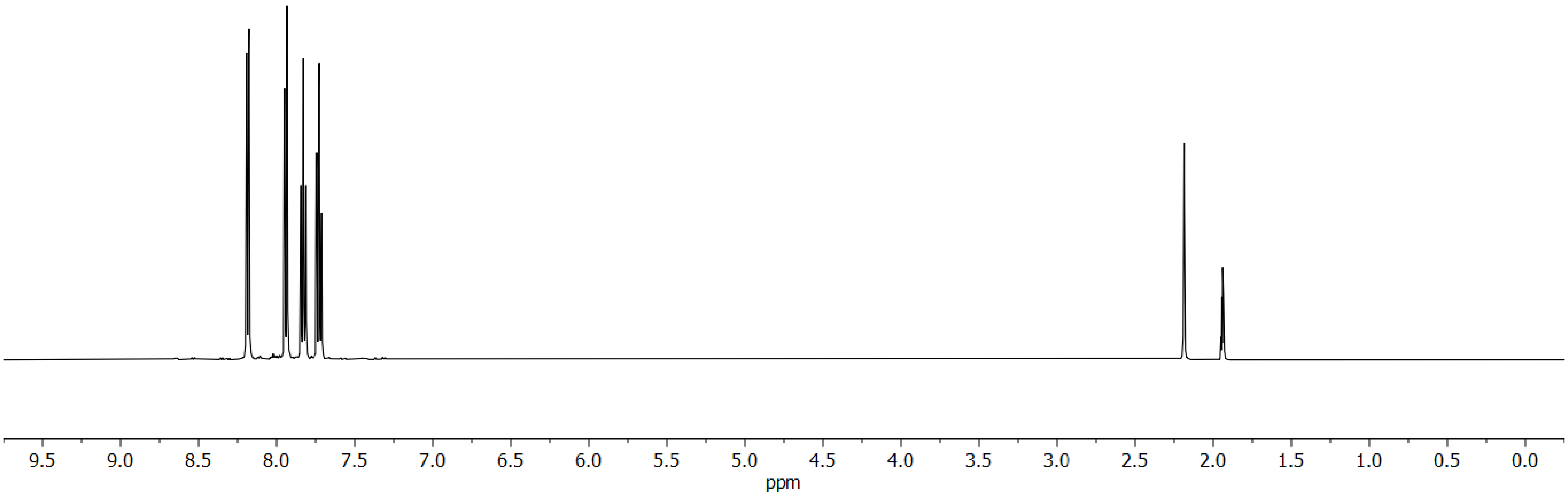

^{13}C NMR of $\mathbf{^{2}H_3}$**-VTT**
126 MHz, CD_3CN, 298 K

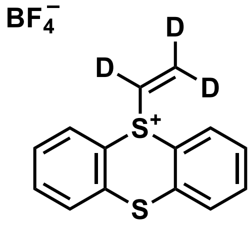

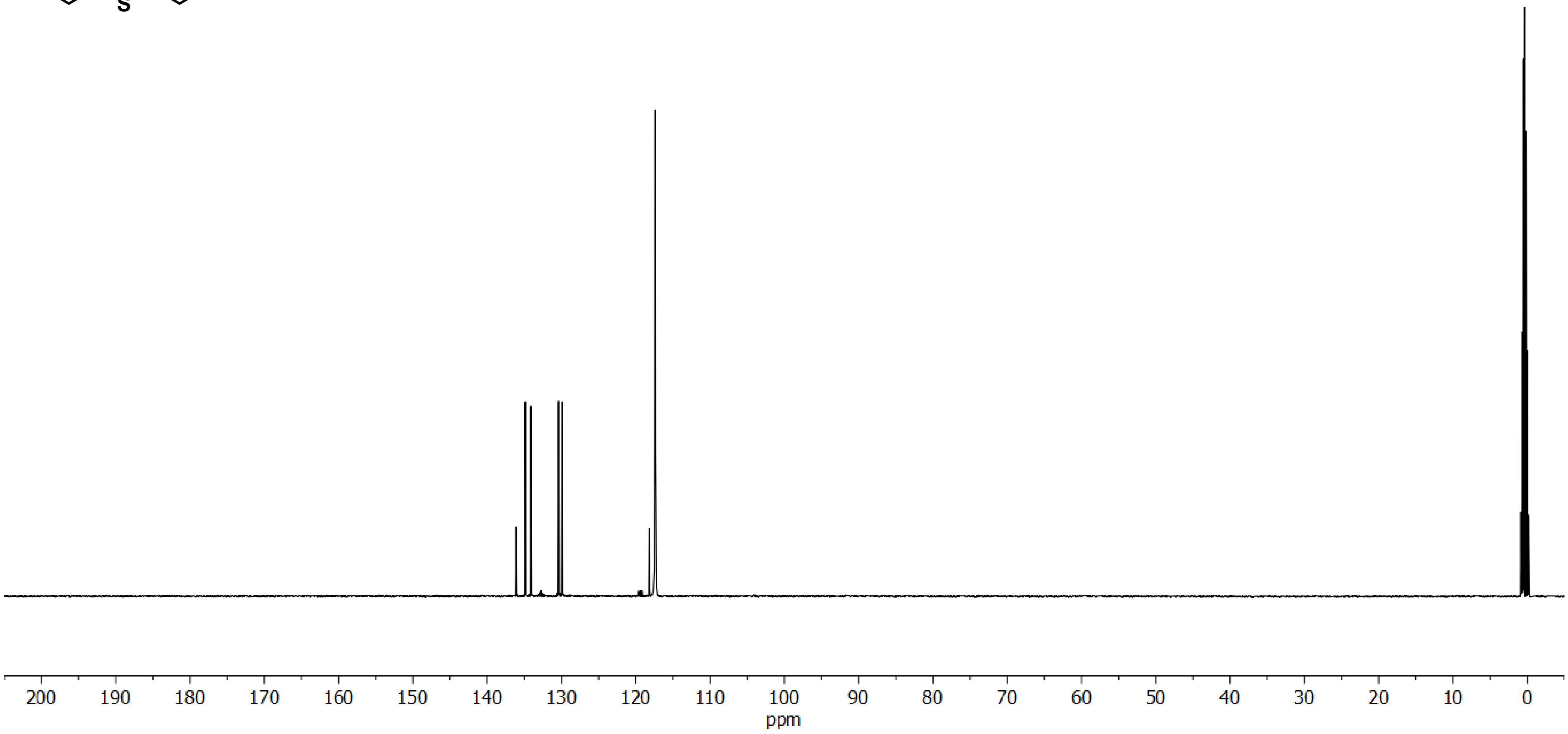

^{19}F NMR of **^{2}H$_3$-VTT**
471 MHz, CD_3CN, 298 K

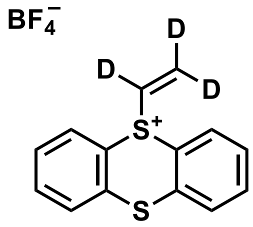

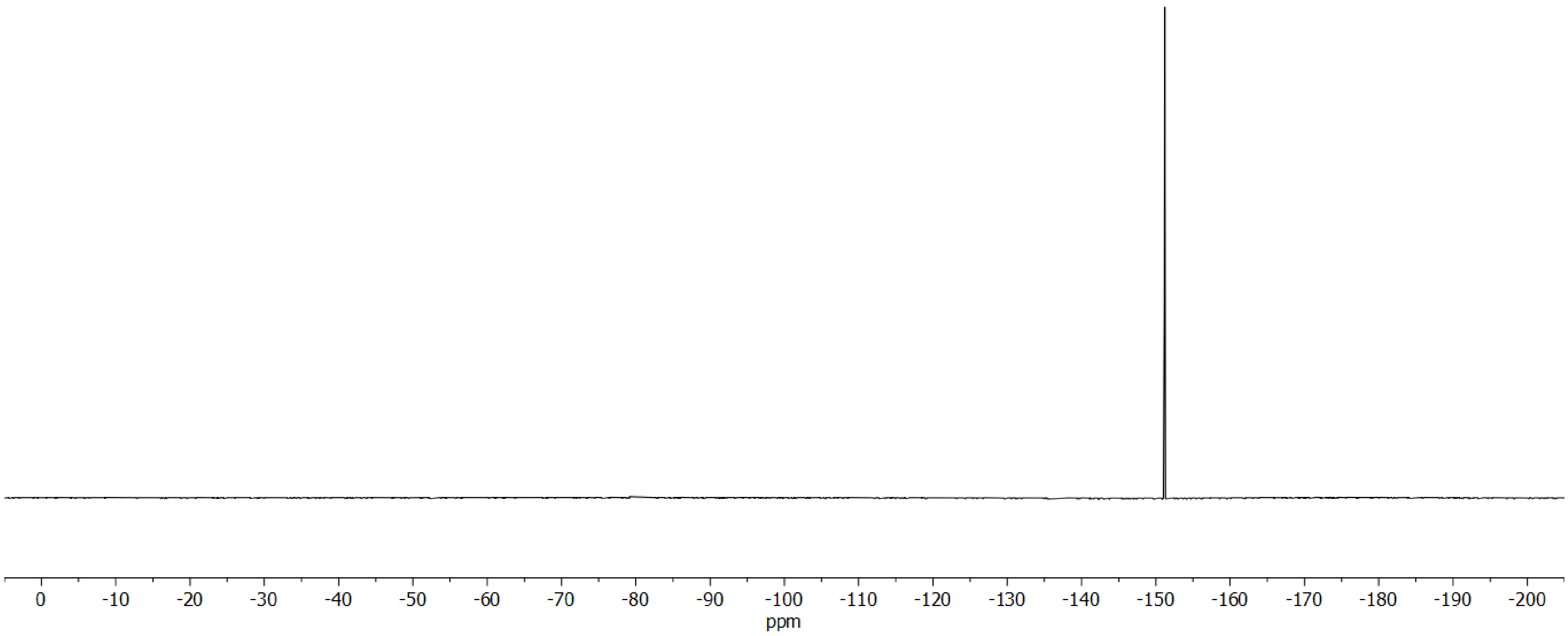

^{1}H NMR of **$^{13}C_2$-VTT**
600 MHz, CD_3CN, 298 K

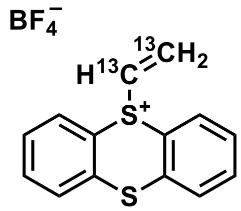

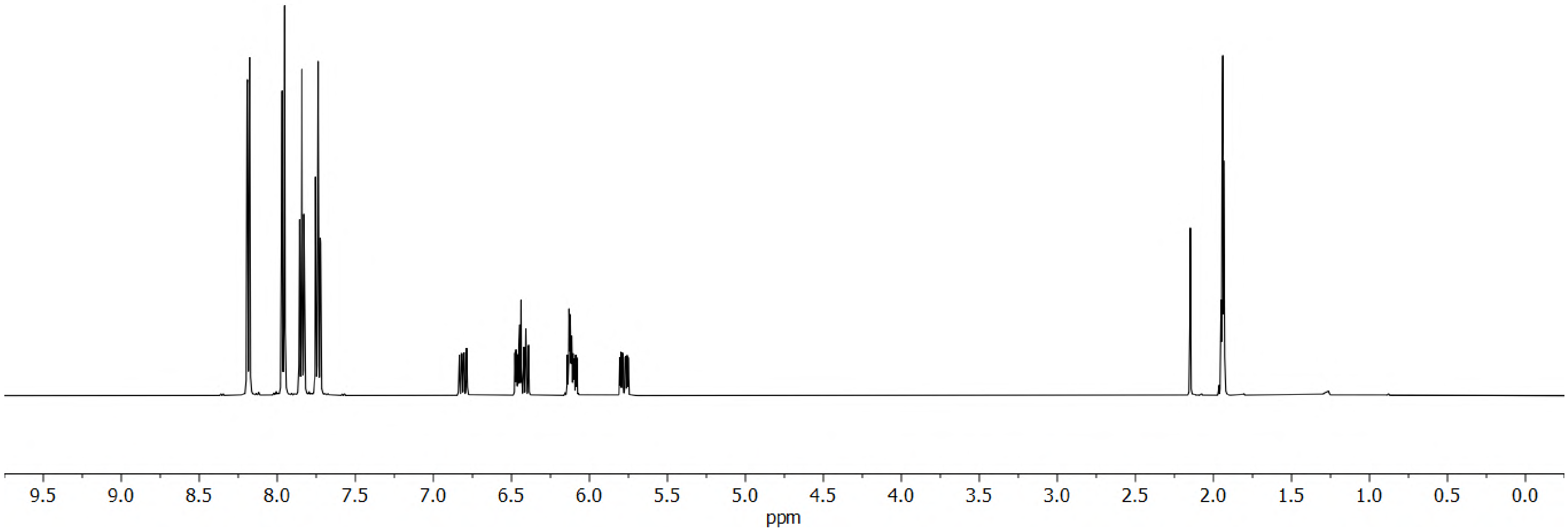

^{13}C NMR of **$^{13}C_2$-VTT**
151 MHz, CD_3CN, 298 K

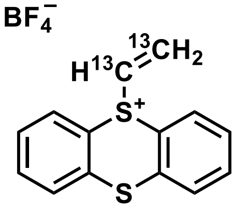

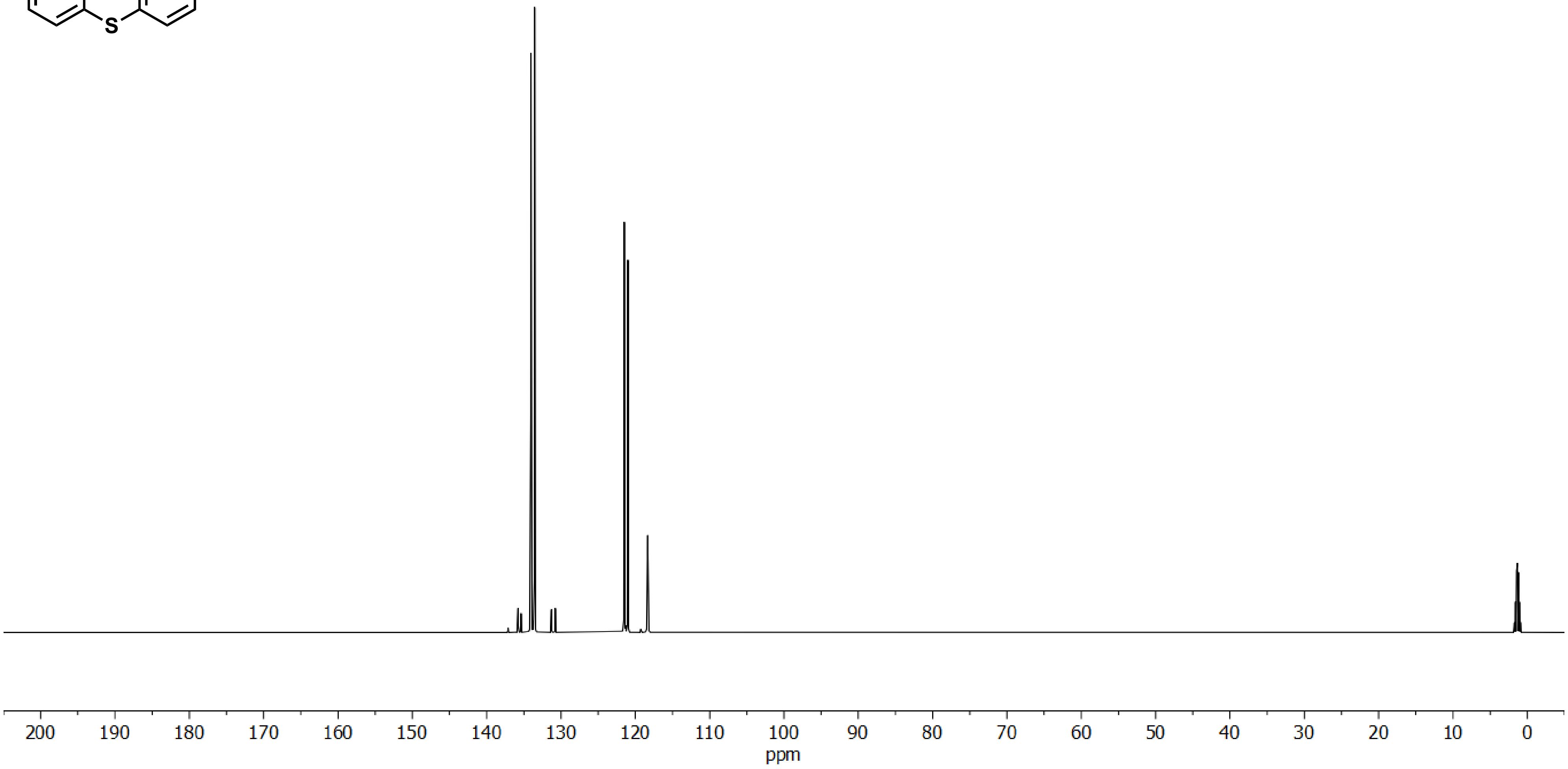

^{19}F NMR of **$^{13}C_2$-VTT**
471 MHz, CD_3CN, 298 K

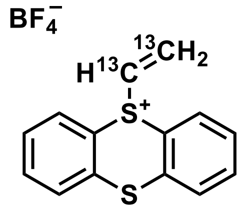

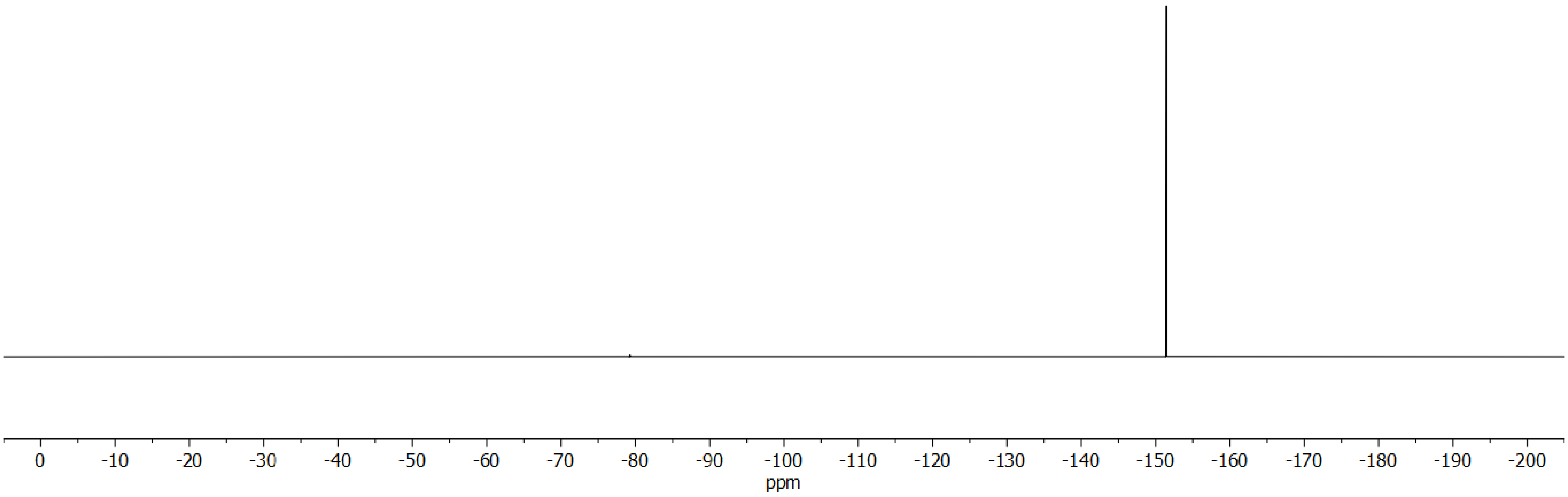

^{1}H NMR of **$^{2}H_3$-VTFT**
500 MHz, CD_3CN, 298 K

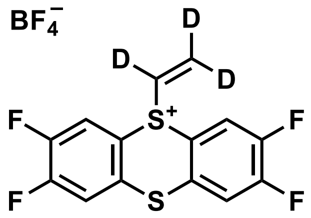

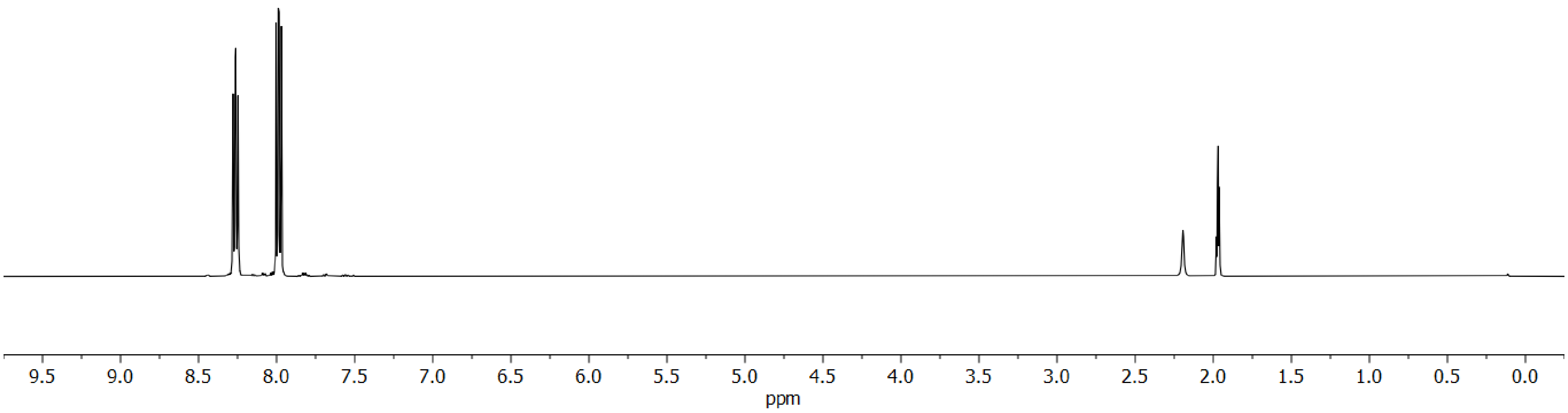

^{2}H NMR of **$^{2}H_3$-VTFT**
92 MHz, CD_3CN, 298 K

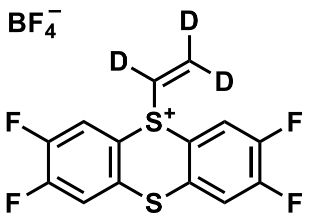

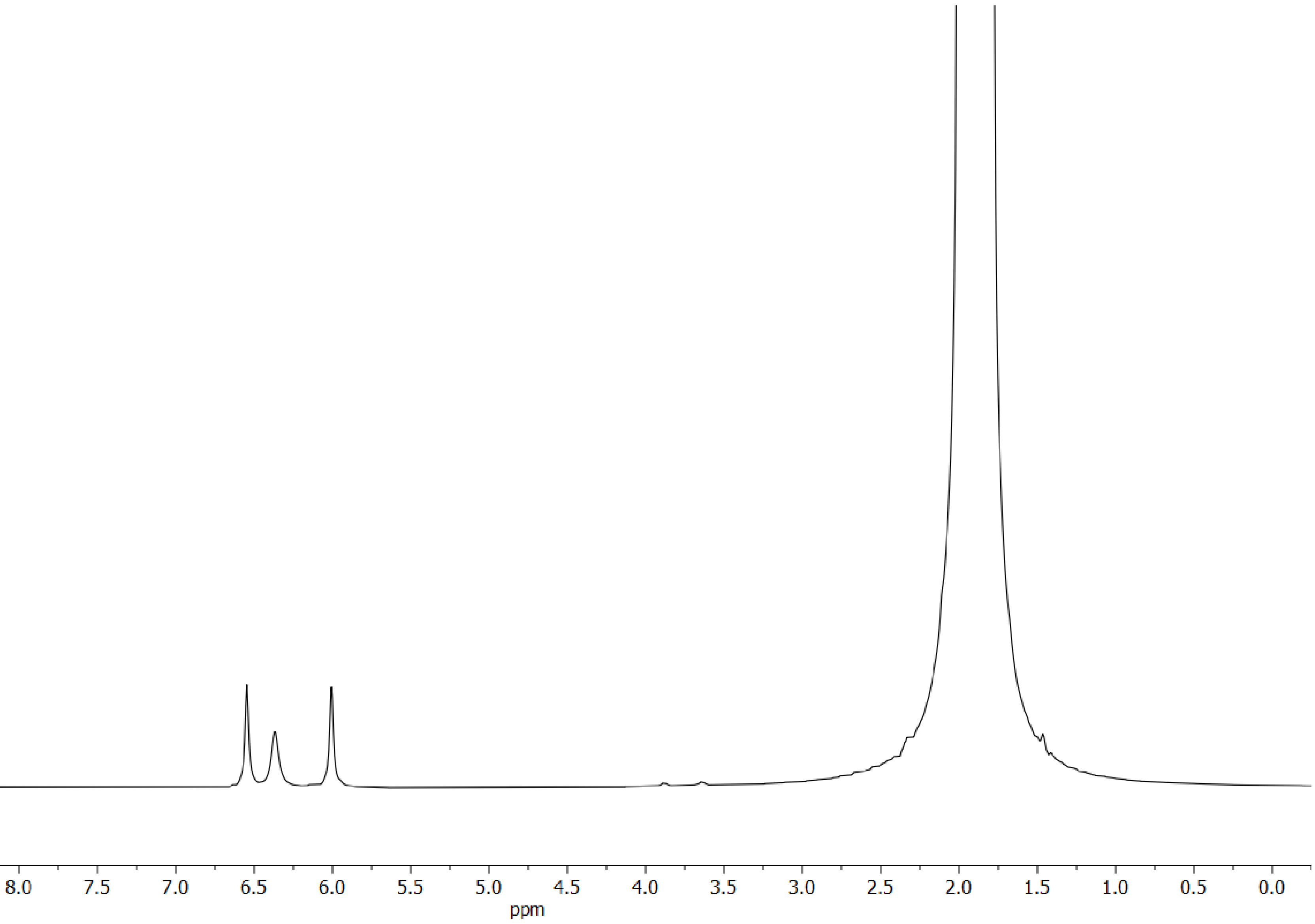

^{13}C NMR of **^{2}H$_3$-VTFT**
126 MHz, CD_3CN, 298 K

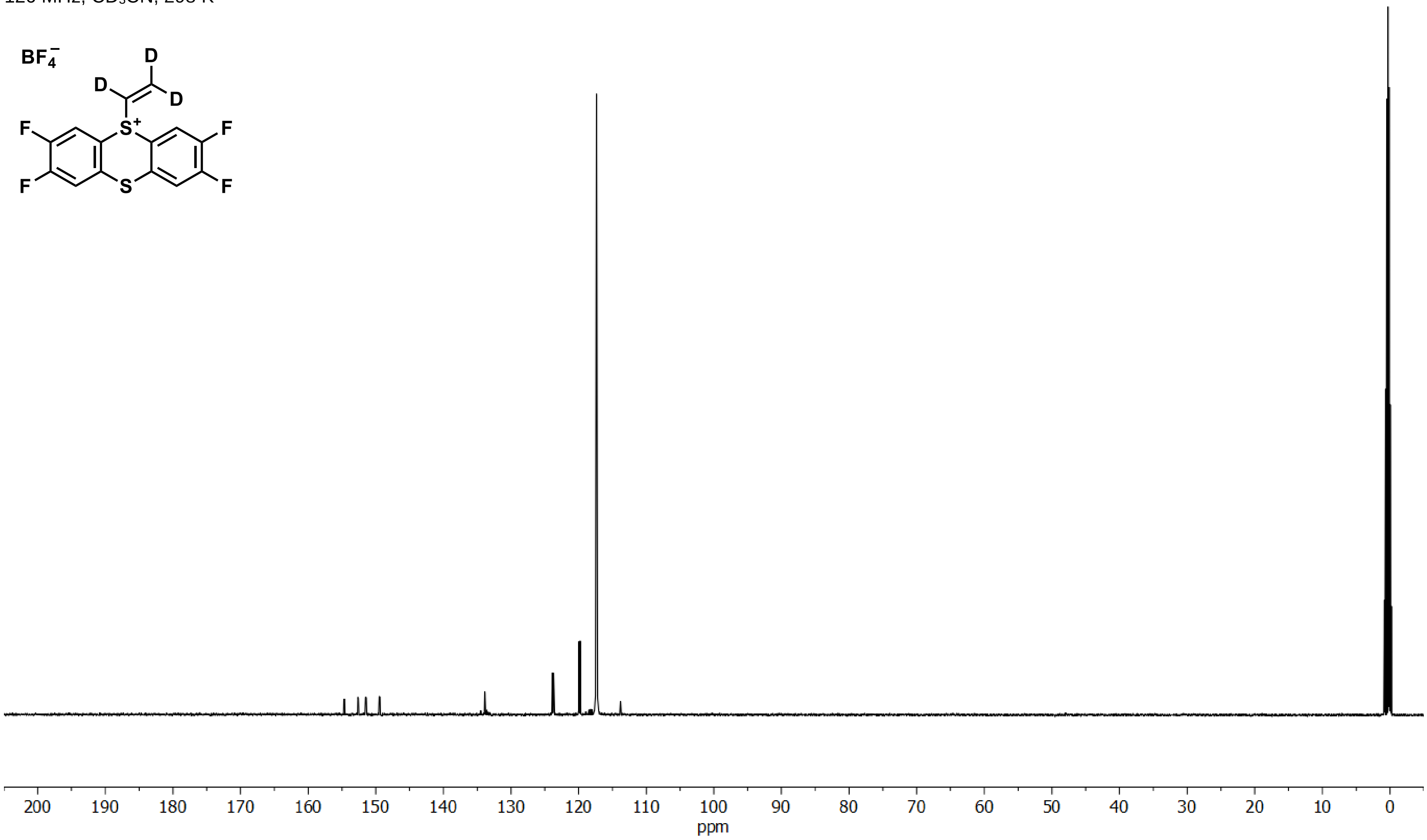

^{19}F NMR of **^{2}H$_3$-VTFT**
471 MHz, CD_3CN, 298 K

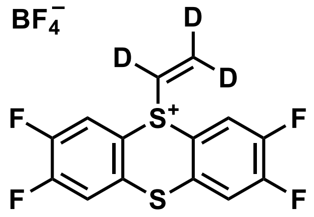

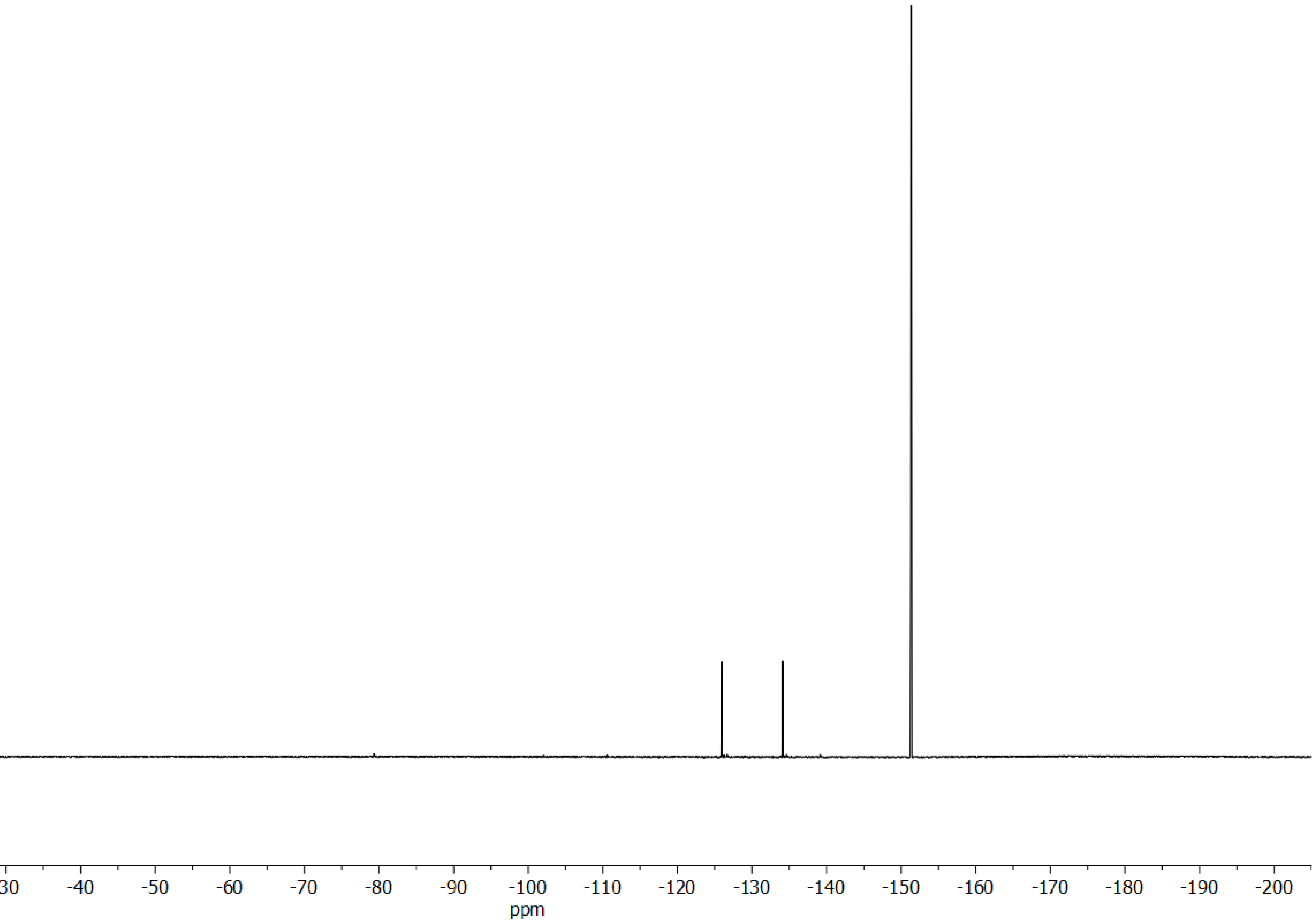

^{1}H NMR of **$^{13}C_2$-VTFT**
500 MHz, CD_3CN, 298 K

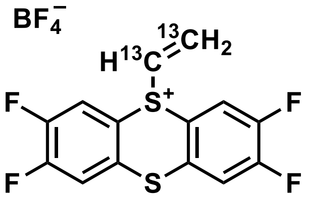

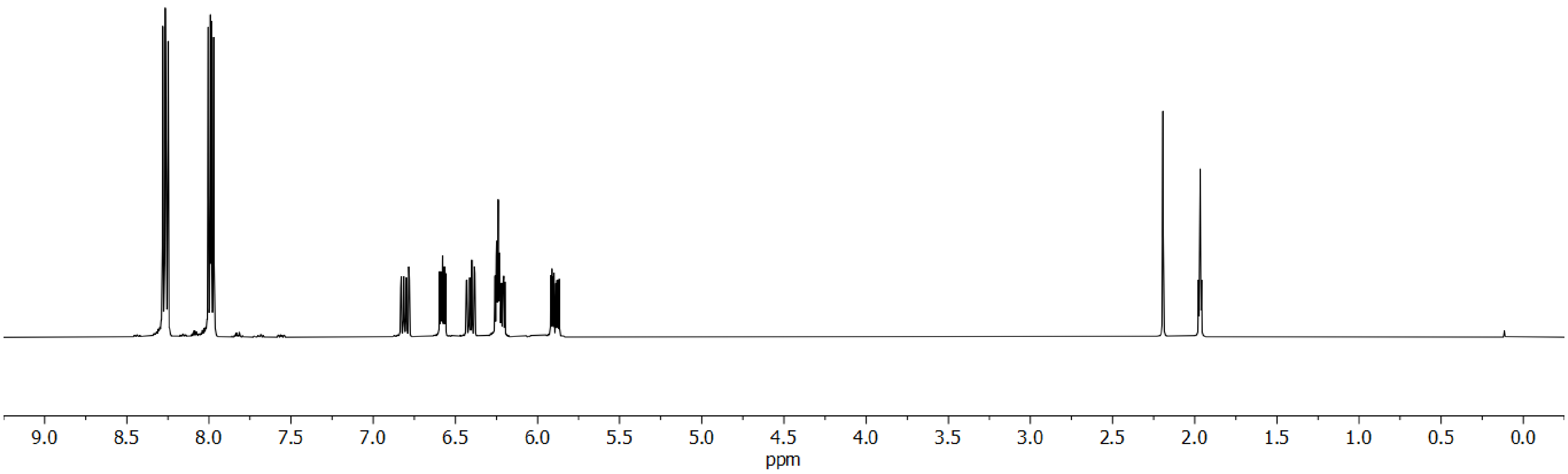

^{13}C NMR of **$^{13}C_2$-VTFT**
126 MHz, CD_3CN, 298 K

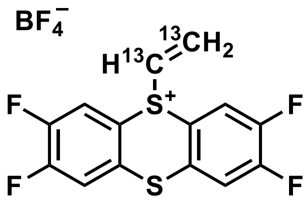

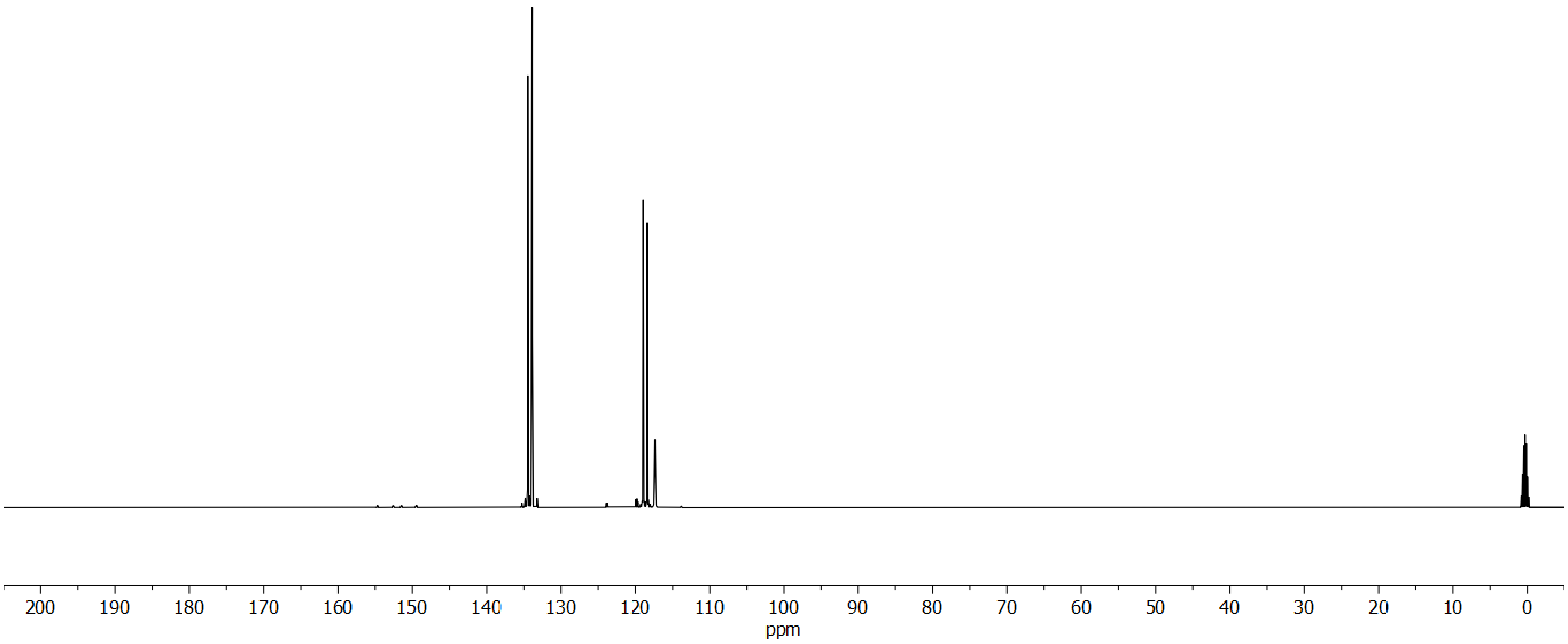

^{19}F NMR of **$^{13}C_2$-VTFT**
471 MHz, CD_3CN, 298 K

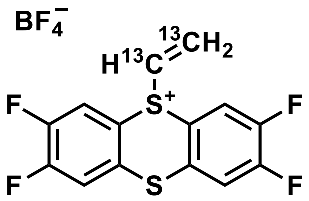

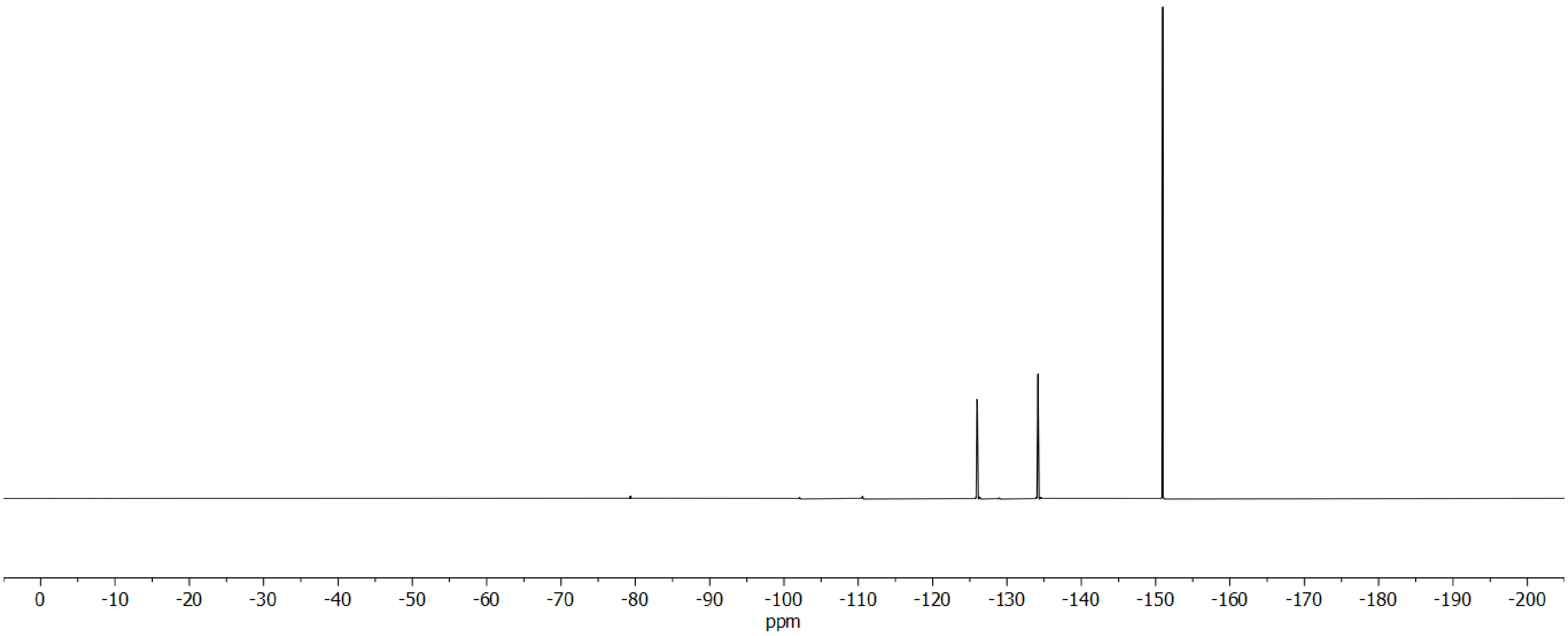

NMR-Characterization of glutathione derivatives

Glutathione–S–C_2H_4–N_3 trifluoroacetate (1)

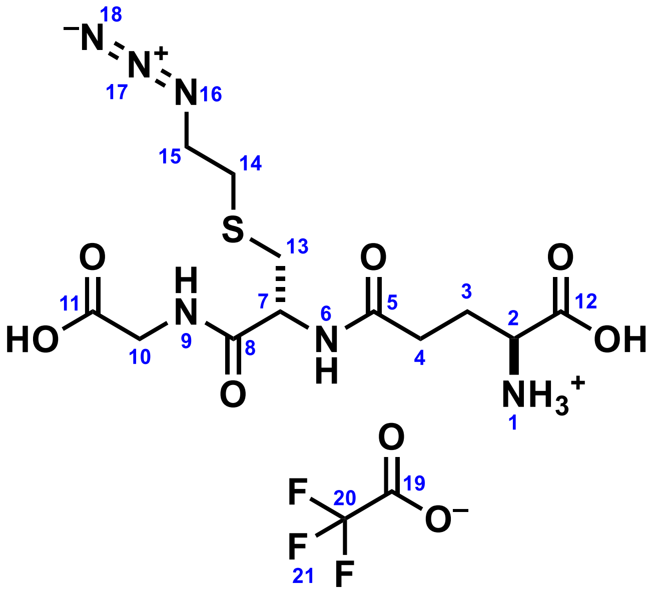

Table S19. Characterization table of Glutathione–S–C_2H_4–N_3 trifluoroacetate. The ^{15}N chemical shifts were referenced to NH_3(l).

Atom	δ (ppm)	J	HSQC	^{15}N-HSQC (in H_2O)	^{15}N-HMBC	HMQC	COSY	NOESY
**N1**	39,62				3			
**H1**	8.034? (in H_2O)	br s						H_2O
**C2**	53,59		2			3, 4		
**H2**	3,902	t 6.4(3)	2			3, 4, 12	3, 4	3, 4
**C3**	26,00		3			2, 4		
**H3**	2,212	m	3		1	2, 4, 5, 12	2, 4	2, 4
**C4**	31,23		4			2, 3		
**H4**	2,583	m	4			2, 3, 5	2, 3	2, 3, 6
**C5**	174,83					3, 4, 7		
**N6**	123,45			6	13a, 13b			
**H6**	8.550 (in H_2O)	d 7.6(7)		6				4

**C7**	53,27		7			13a, 13b		
**H7**	4,628	d 5.1(13a), d 8.7(13b)	7			5, 8, 13	13a, 13b	9, 10, 13a, 13b, 14, 15
**C8**	172,76					7, 10, 13a, 13b		
**N9**	111,52			9	10			
**H9**	8.573 (in H_2O)	d 6.1(10)		9			10	7, 10
**C10**	41,46		10					
**H10**	4,020	m	10		9	8, 11	9	7, 9
**C11**	173,37					10		
**C12**	173,29					2, 3		
**C13**	32,95		13a, 13b			7, 14		
**H13a**	3,142	d 5.1(7), d 14.1(13b)	13		6	7, 8, 14	7, 13b	7, 14, 15
**H13b**	2,961	d 8.7(7), d 14.1(13a)	13		6	7, 8, 14	7, 13a	7, 14, 15
**C14**	31,23		14			13a, 13b, 15		
**H14**	2,848	m	14		16	13, 15	15	7, 13a, 13b, 15
**C15**	50,50		15			14		
**H15**	3,578	t 6.4(14)	15		17	14	14	7, 13a, 13b, 14
**N16**	71,64				14			
**N17**	247,44				15			
**N18**	n.f.							
**C19**	163,09	q 35.5(21)						
**C20**	116,44	q 291.6(21)						
**F21**	–75,49	s						

^{1}H NMR of Glutathione–S–C_2H_4–N_3 trifluoroacetate (**1**):
600 MHz, D_2O, 298 K

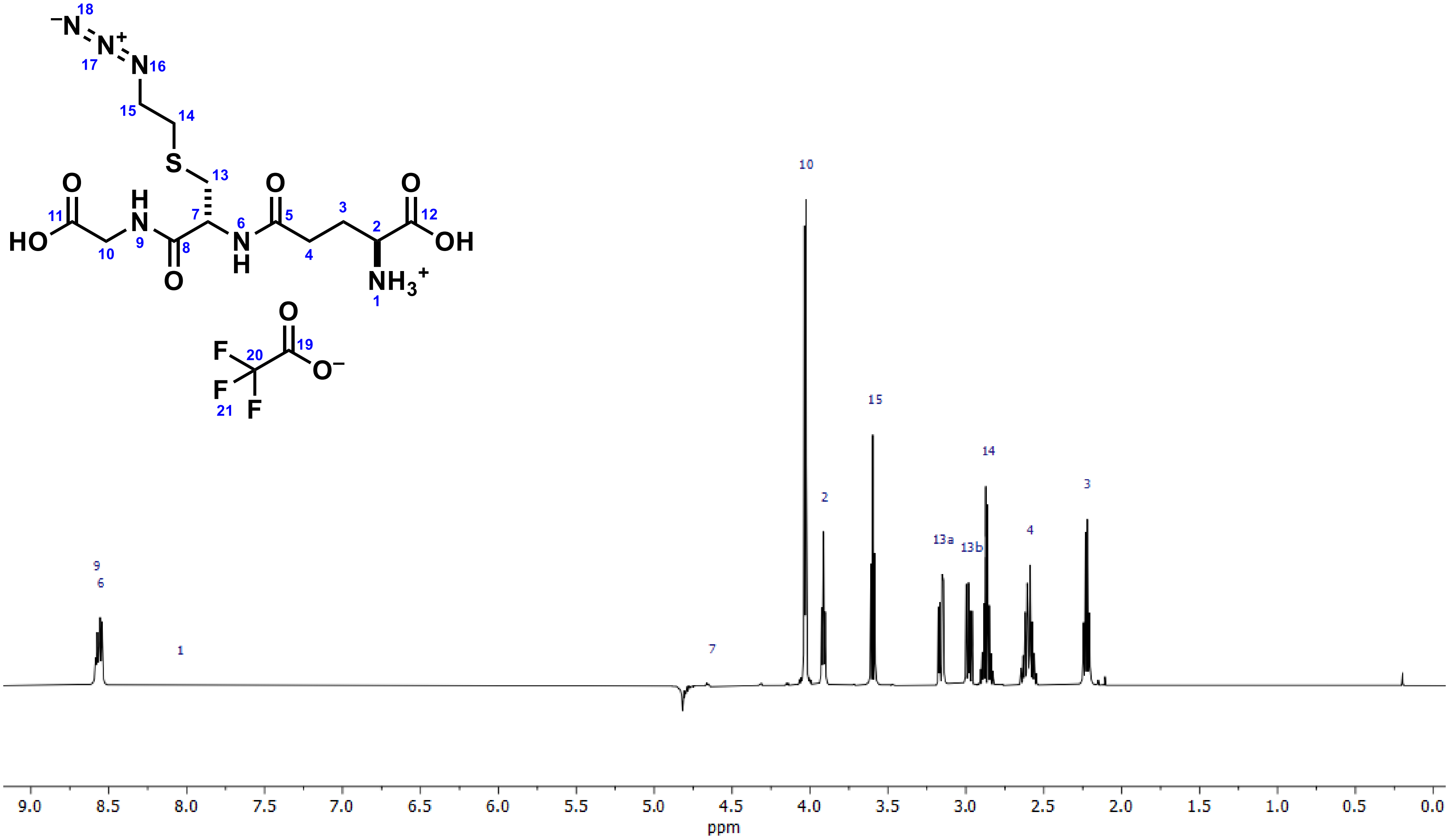

^{13}C NMR of Glutathione–S–C_2H_4–N_3 trifluoroacetate (**1**):
151 MHz, D_2O, 298 K

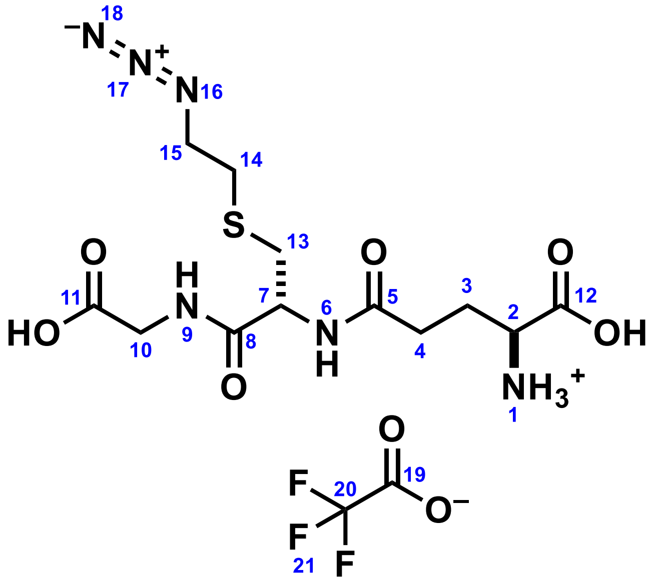

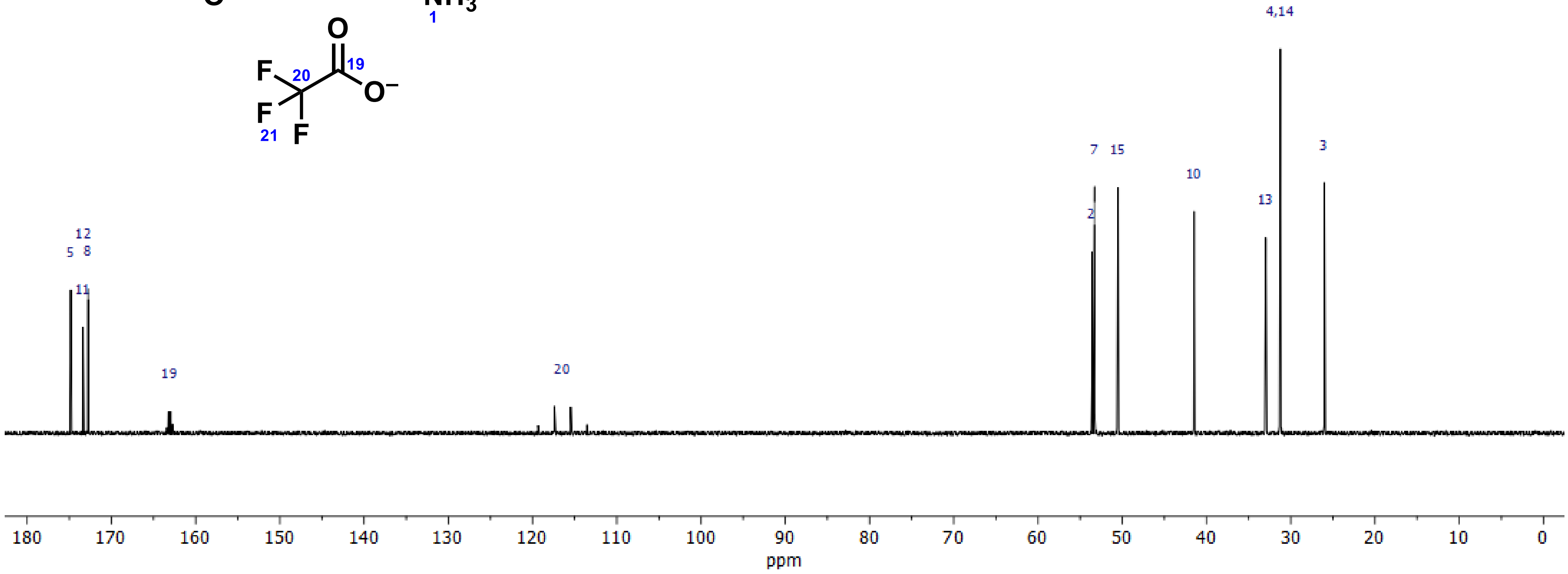

^{19}F NMR of Glutathione–S–C_2H_4–N_3 trifluoroacetate (**1**):
470 MHz, D_2O, 298 K

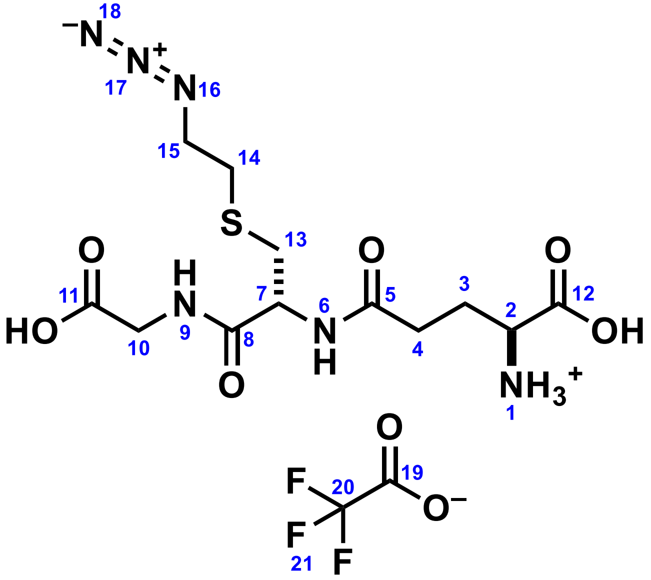

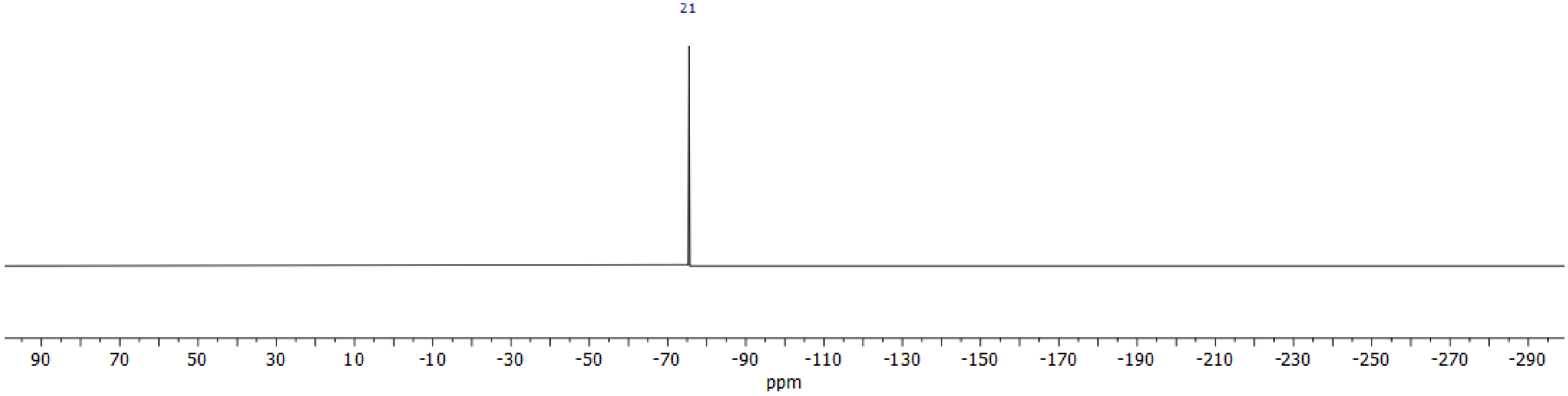

HSQC-NMR of Glutathione–S–C_2H_4–N_3 trifluoroacetate (**1**):
D_2O, 298 K

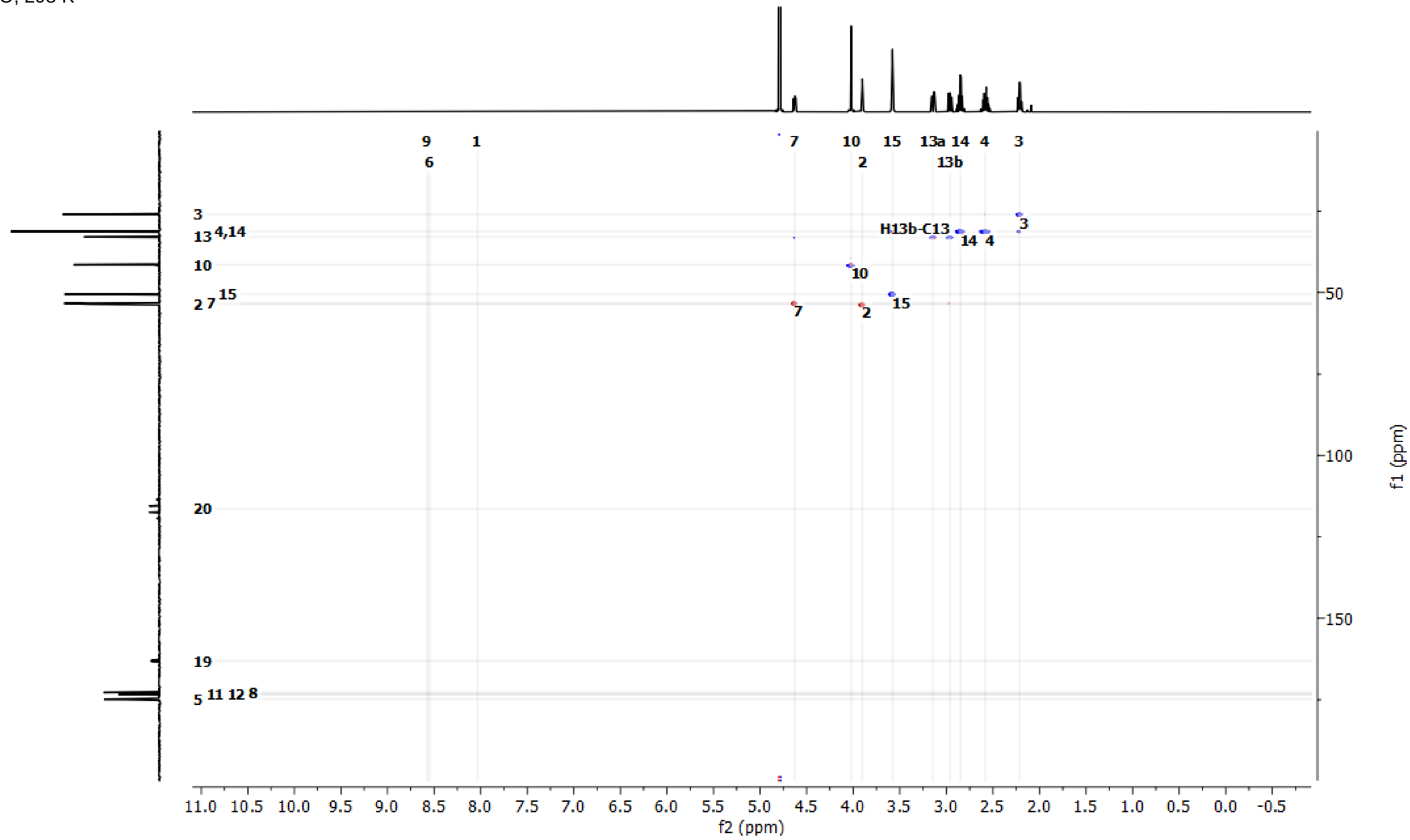

HMQC-NMR of Glutathione–S–C_2H_4–N_3 trifluoroacetate (**1**):
D_2O, 298 K

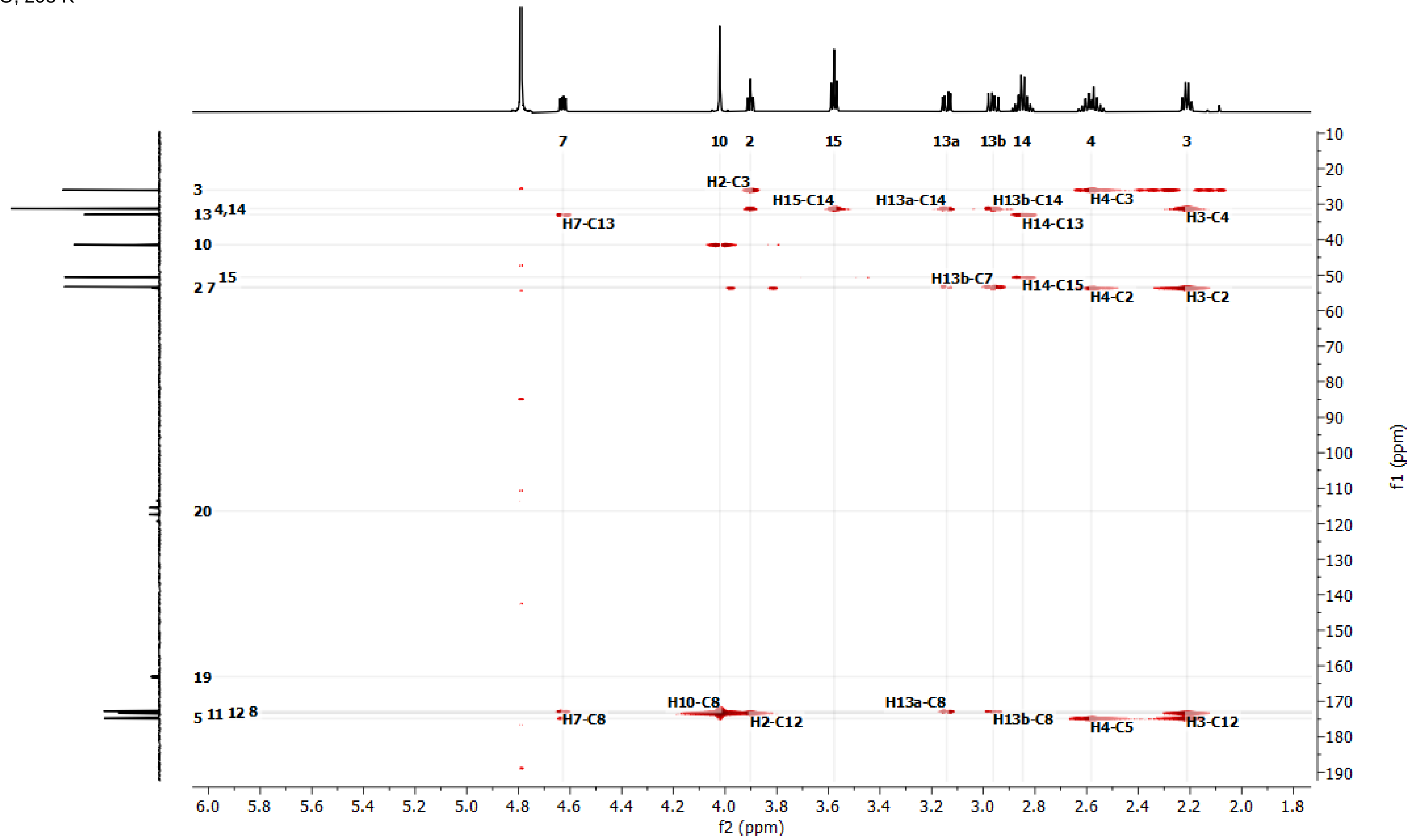

COSY-NMR of Glutathione–S–C_2H_4–N_3 trifluoroacetate (**1**):
D_2O, 298 K

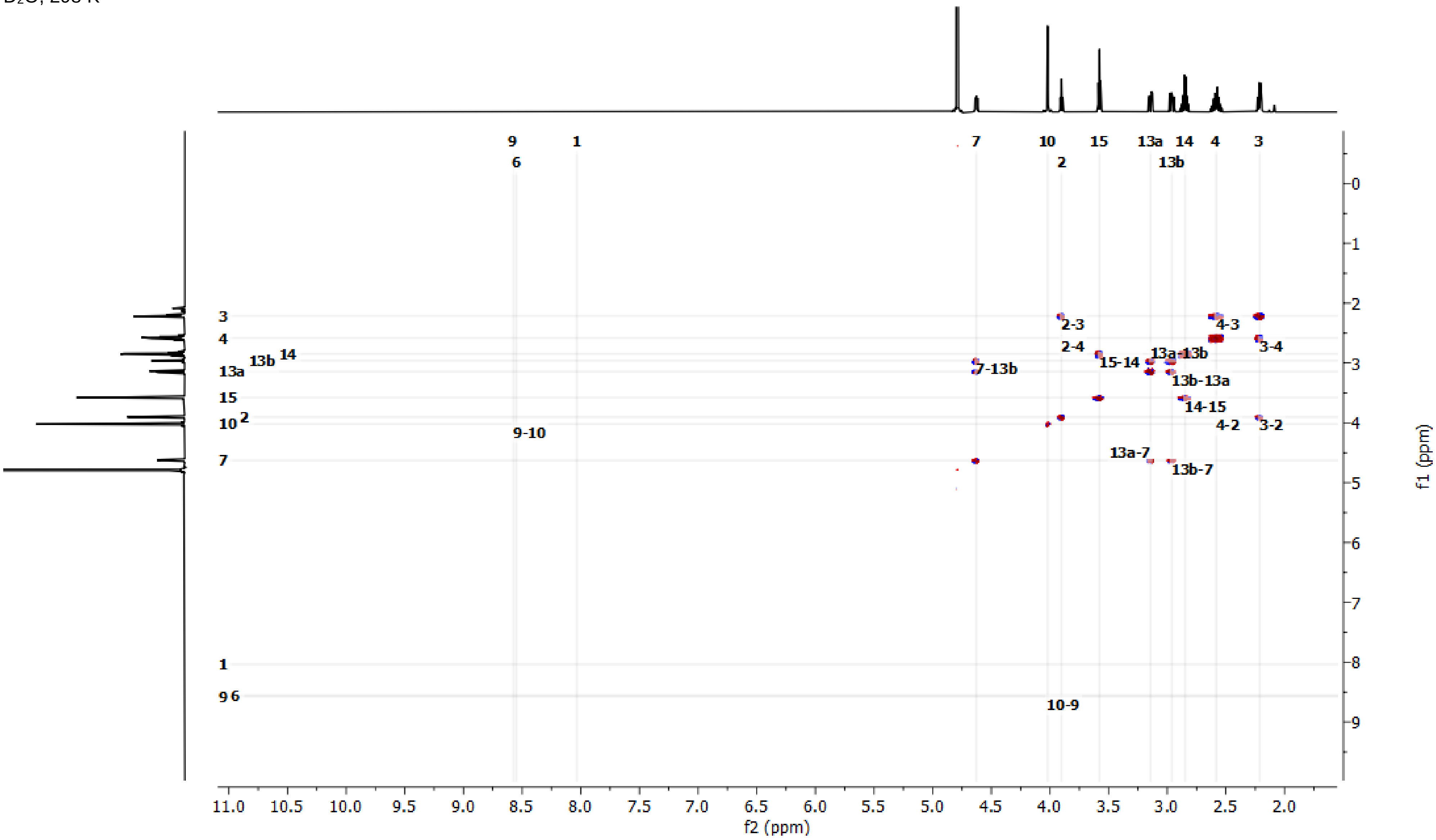

NOESY-NMR of Glutathione–S–C_2H_4–N_3 trifluoroacetate (**1**):
D_2O, 298 K

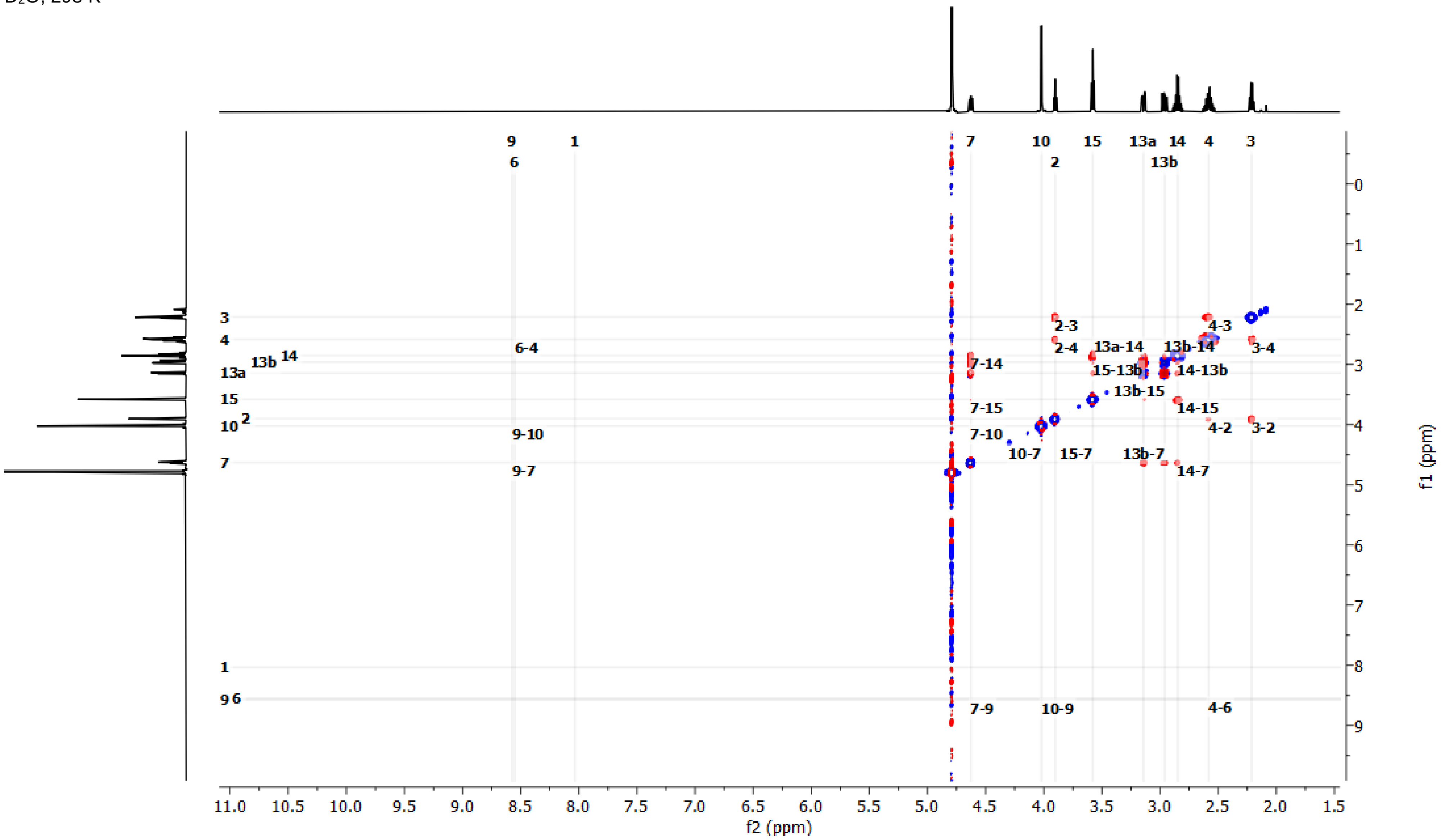

^{15}N HMBC-NMR of Glutathione–S–C_2H_4–N_3 trifluoroacetate (**1**):
D_2O, 298 K

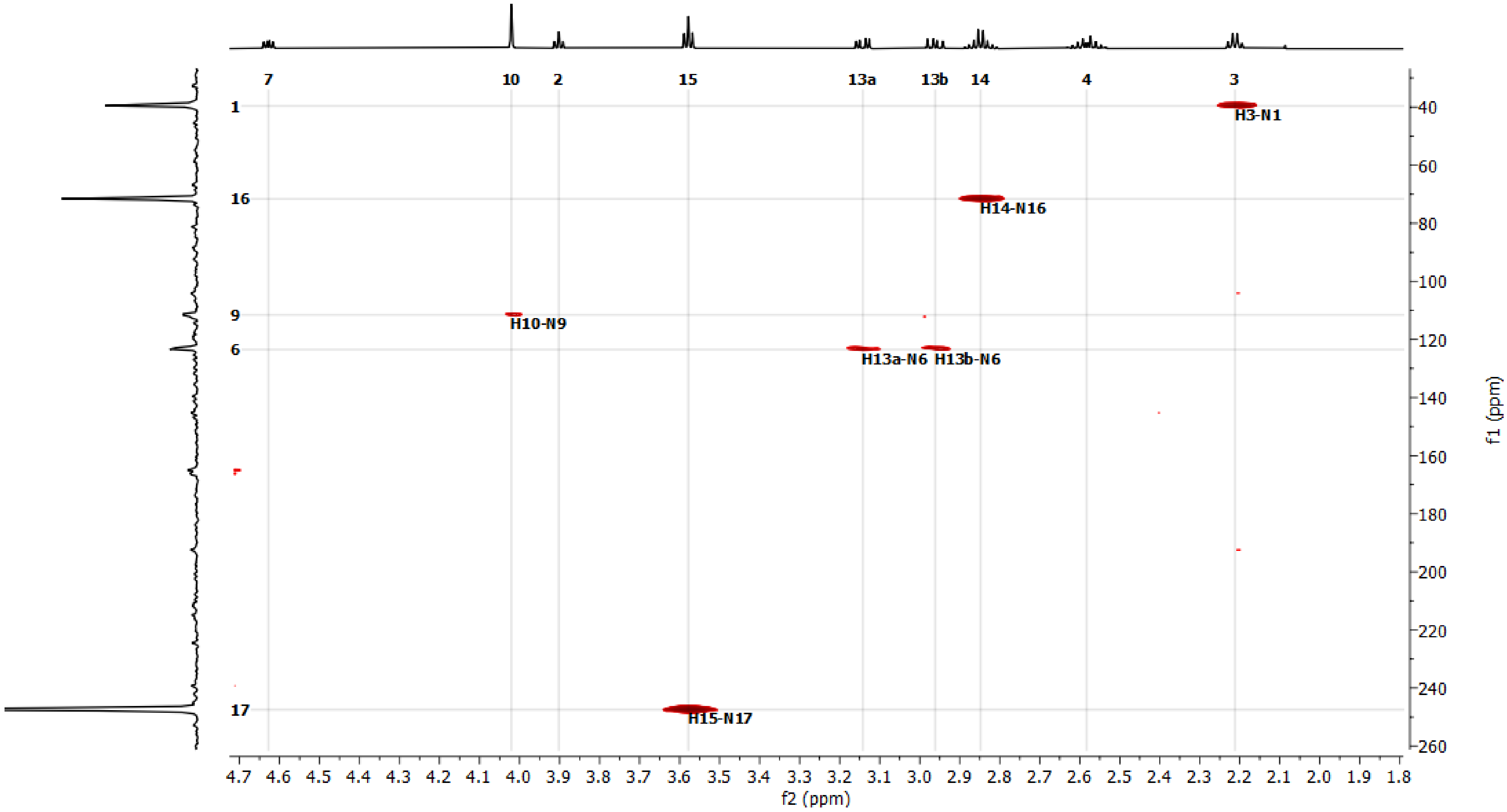

^{15}N HSQC-NMR of Glutathione–S–C_2H_4–N_3 trifluoroacetate (**1**):
D_2O, 298 K

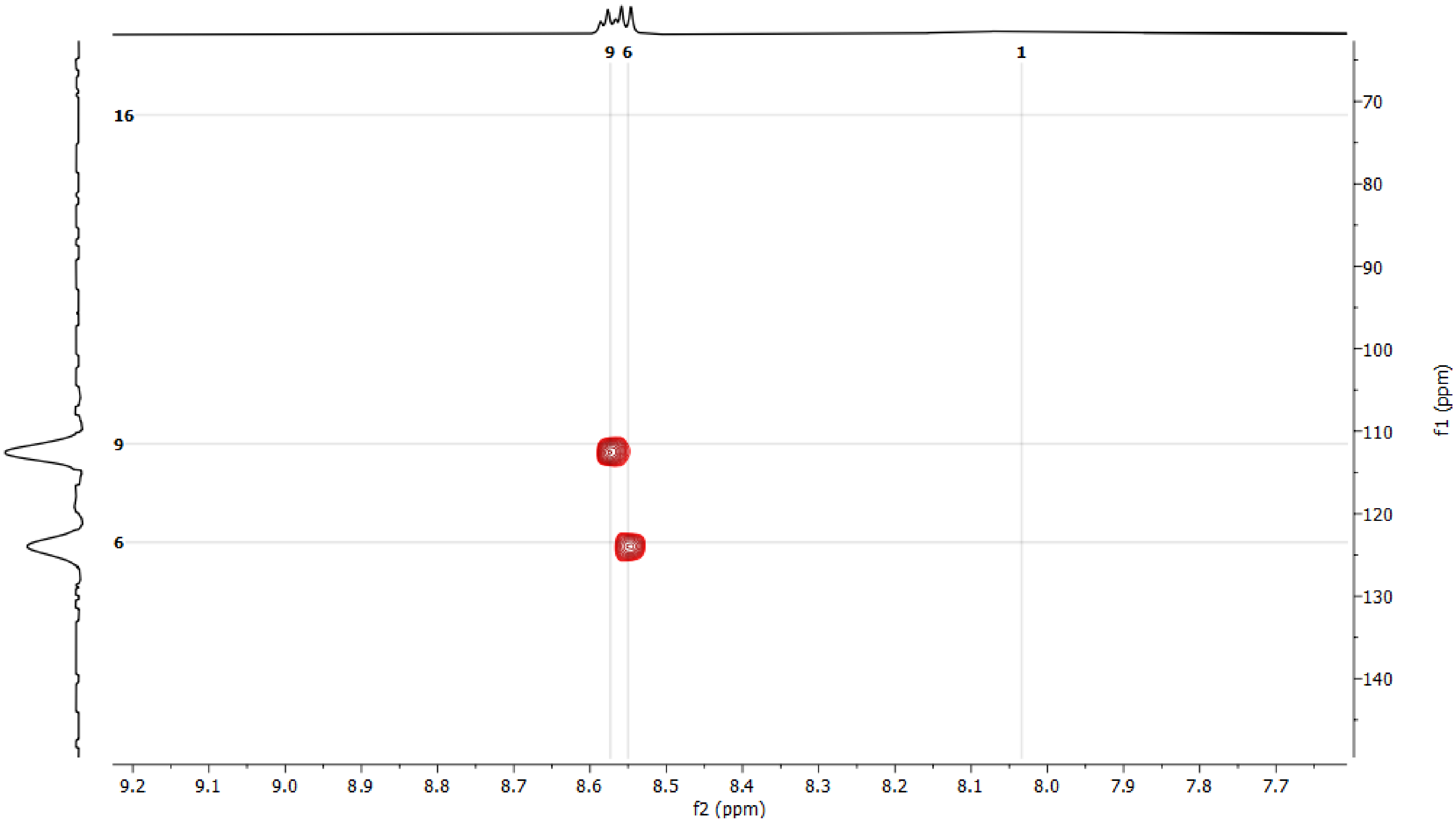

Glutathione–S–C_2H_3D–N_3 trifluoroacetate ($^2H_1$-1)

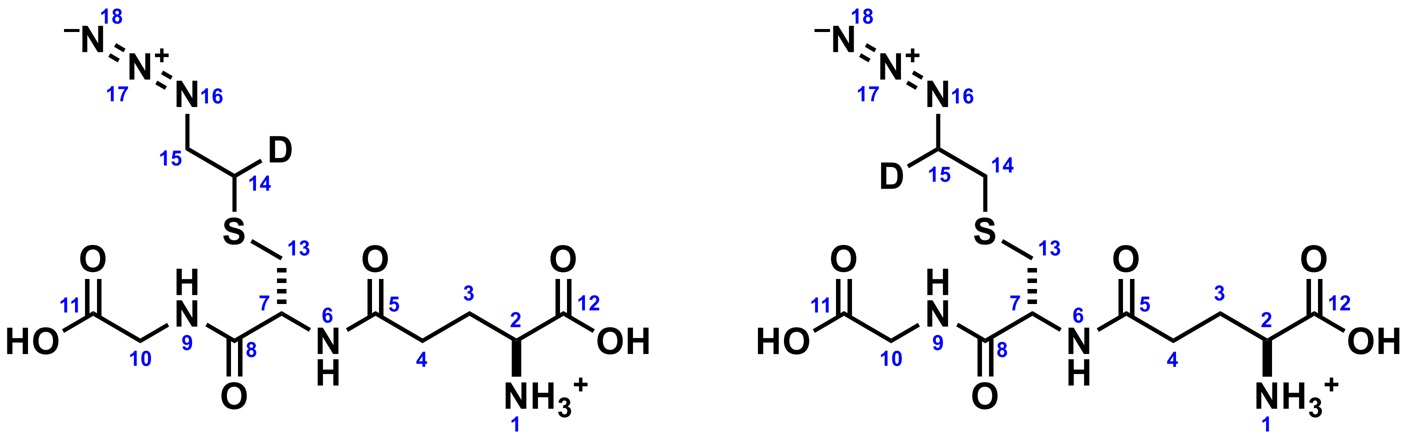

Table S20. Characterization table of Glutathione–S–C_2H_3D–N_3 trifluoroacetate. The ^{15}N chemical shifts were referenced to CH_3NO_2.

Atom	δ (ppm)	J	HSQC	HMQC	15N-HMBC	COSY	NOESY
**N1**	-340.9				3		
**H1**	8.079 (?)	br s					
**C2**	53.46		2	3, 4			
**H2**	3.906	t 6.5(3)	2	3, 4, 12		3, 4	3, 4
**C3**	25.94		3	2, 4			
**H3**	2.203	m	3	2, 4, 5, 12	1	2, 4	2, 4
**C4**	31.17		4	2, 3			
**H4**	2.572	m	4	2, 3, 5	6	2, 3	2, 3, 6
**C5**	174.76			3, 4, 7			
**N6**	-257.0				4, 7, 13a, 13b		
**H6**	8.516	d 7.5(7)				7	4, 7, 13b
**C7, C7'**	53.23, 53.24		7	13a, 13b			
**H7**	4.615	d 8.7(13b), d 5.2(13a)	7	5, 8, 13	6	6, 13a, 13b	6, 9, 10, 13a, 13b, 14, 15

**C8**	172.75			7, 10, 13a, 13b			
**N9**	-269.1				10		
**H9**	8.561	t 5.8(10)				10	7, 10
**C10**	41.36		10				
**H10**	4.015	m	10	8, 11	9	9	7, 9
**C11**	173.22			10			
**C12**	173.12			2, 3			
**C13, C13'**	32.88, 32.93		13a, 13b	7, 14			
**H13a**	3.126	d 5.2(7), d 14.1(13b)	13	7, 8, 14	6	7, 13b	7, 13b, 14, 15
**H13b**	2.945	d 8.7(7), d 14.1(13a)	13	7, 8, 14	6	7, 13a	6, 7, 13a, 14, 15
**C14**	30.91	t 21.3(D14)	14	13a, 13b, 15			
**H14**	2.816	m (o.l.)	14	13, 15	16	15	7, 13a, 13b, 15
**C14'**	31.10						
**H14'a**	2.845	d 14.1(14'b), d 6.4(15')					
**H14'b**	2.810	d 14.1(14'a), d 6.4(15')					
**C15**	50.39		15	14			
**H15**	3.559	d 6.4(14)	15	14	16, 17	14	7, 13a, 13b, 14
**C15'**	50.16	22.2(D15)					
**H15'**	3.550	t 6.4(14'a, 14'b)					
**N16**	-308.8				14, 15		
**N17**	-133.0				15		
**N18**	n.f.						

^{1}H NMR of Glutathione–S–C_2H_3D–N_3 (**^{2}H[1]-1**):
600 MHz, D_2O, 298 K

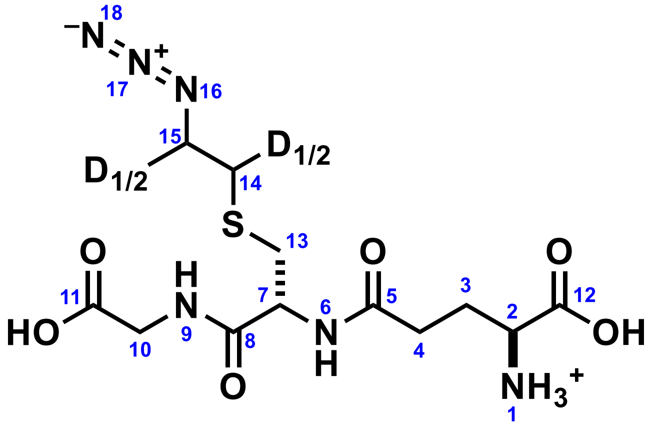

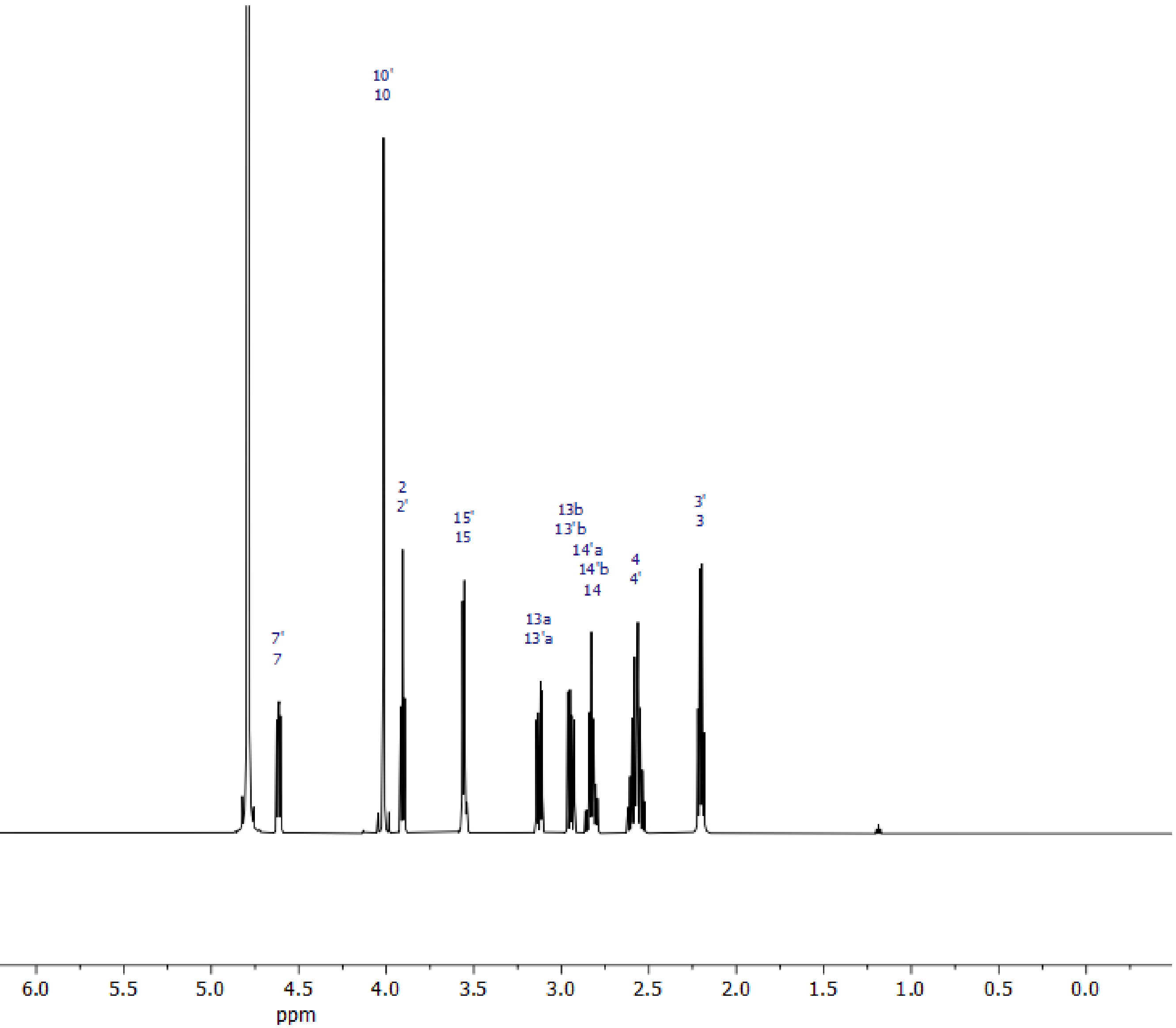

^{13}C NMR of Glutathione–S–C_2H_3D–N_3 (**^{2}H$_1$-1**):
151 MHz, D_2O, 298 K

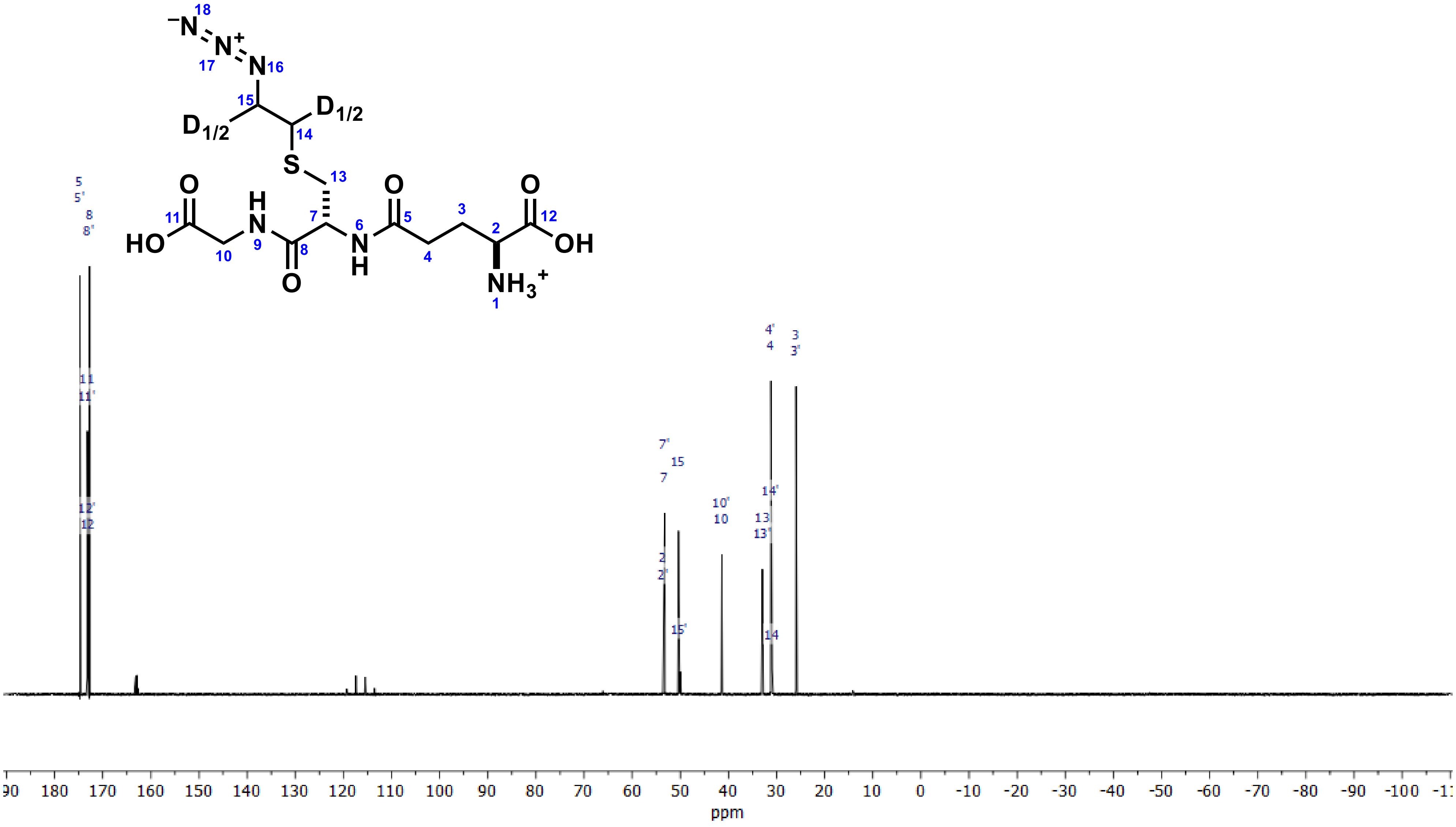

^{1}H & ^{13}C NMR of the ethylene linkage in Glutathione–S–C_2H_3D–N_3 (**$^{2}H_1$-1**):

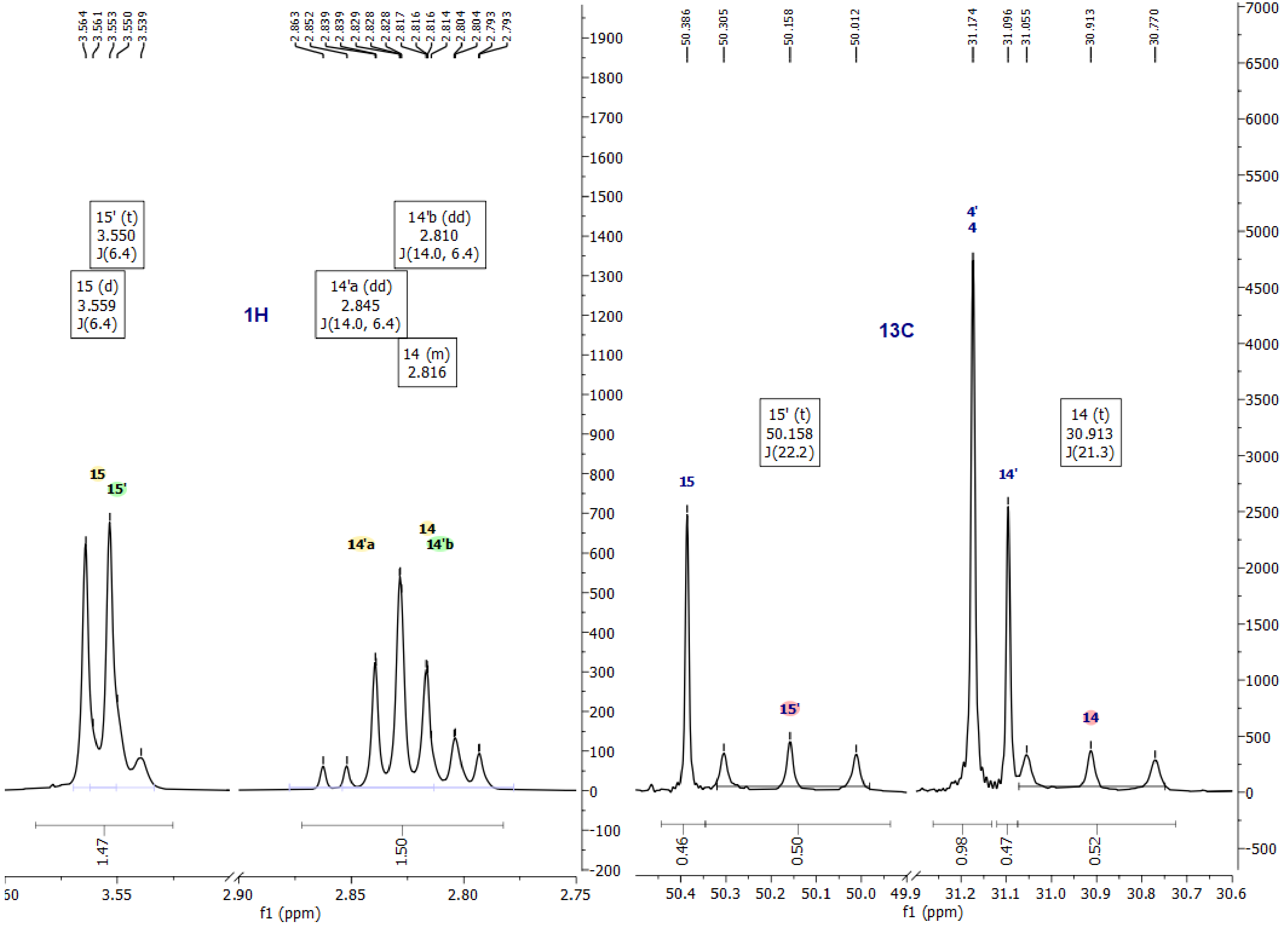

HSQC-NMR of Glutathione–S–C_2H_3D–N_3 (^{2}**H$_1$-1**):
D_2O, 298 K

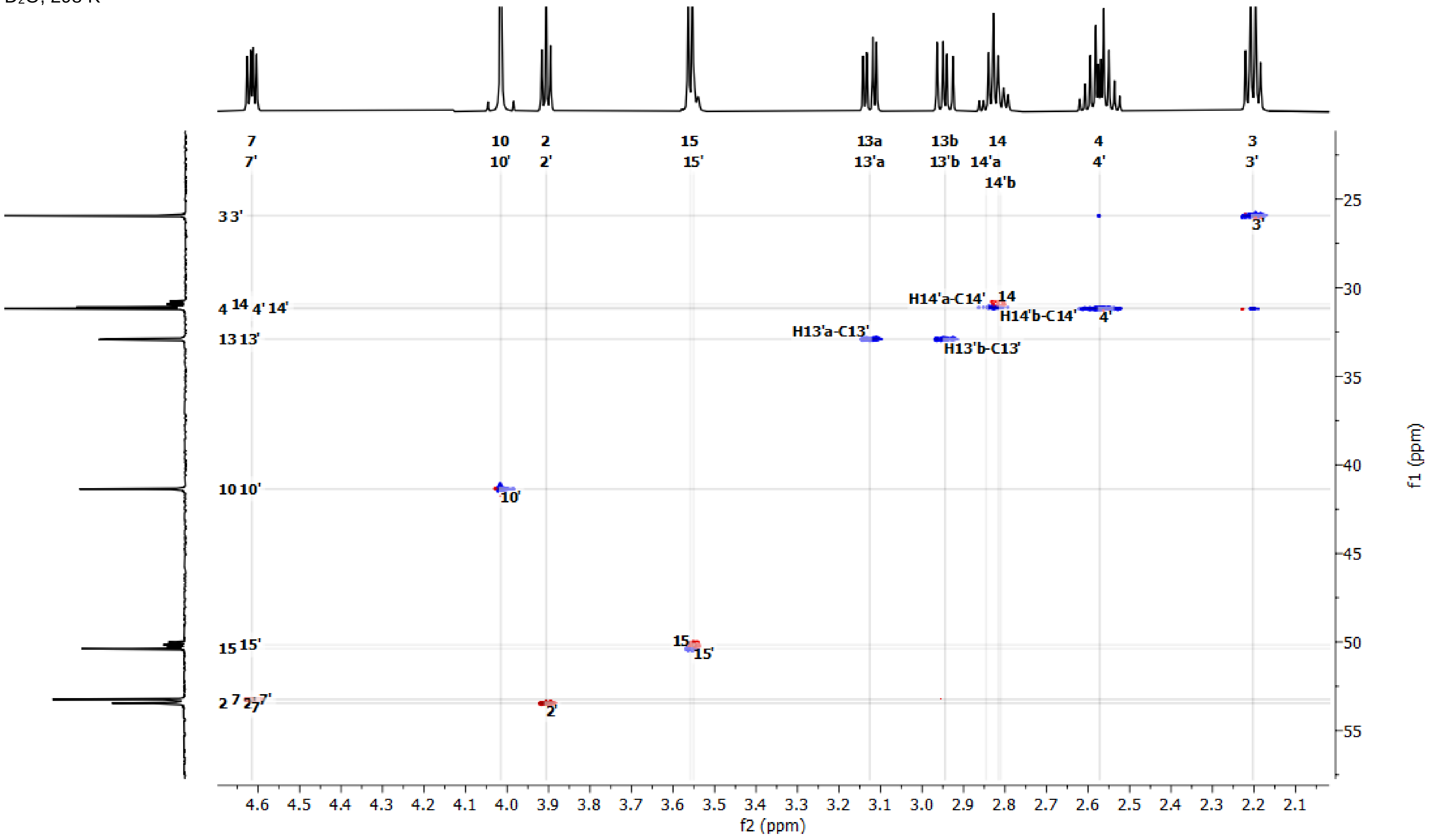

HMQC-NMR of Glutathione–S–C_2H_3D–N_3 ($^2H_1$-**1**):
D_2O, 298 K

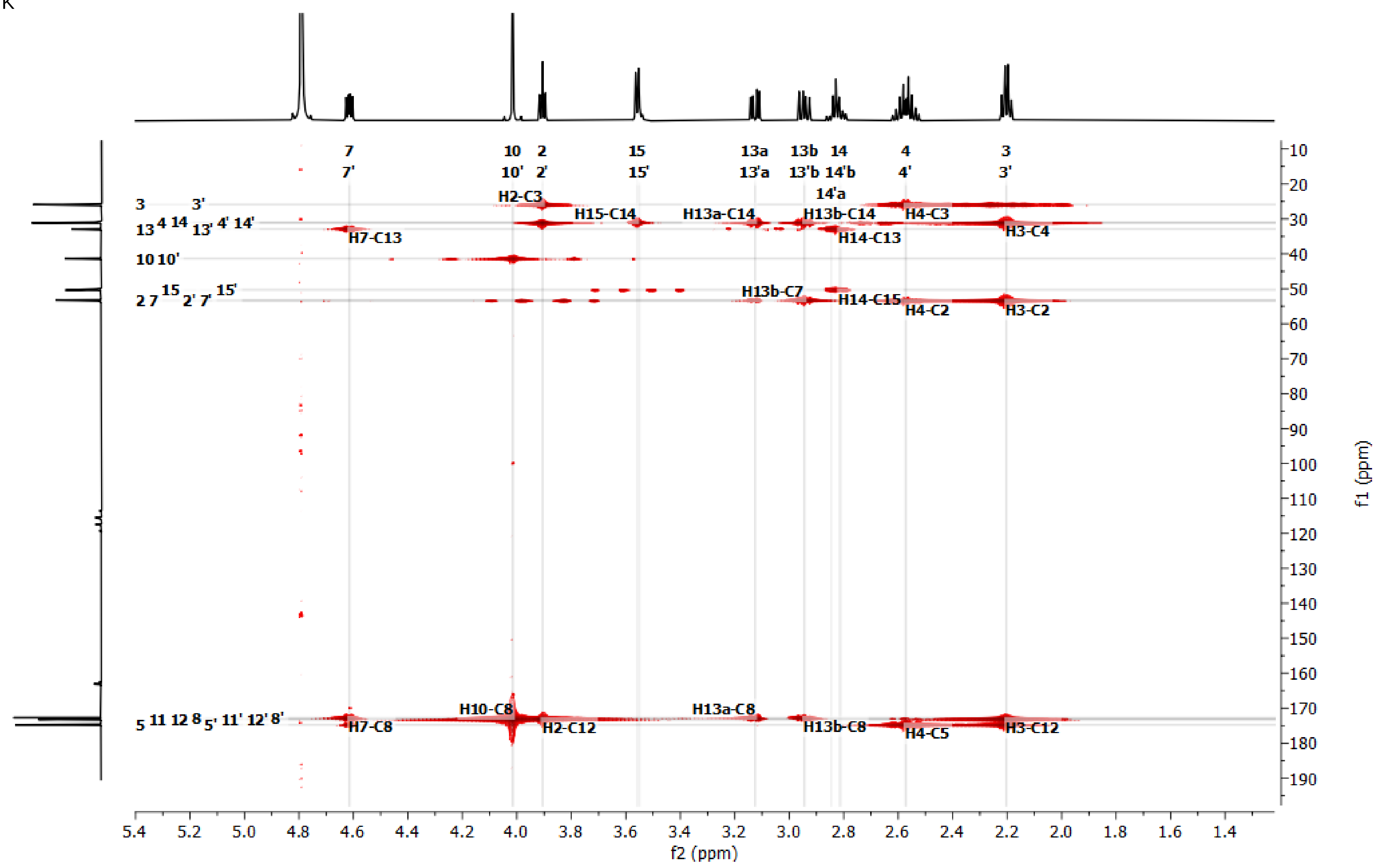

COSY-NMR of Glutathione–S–C_2H_3D–N_3 ($^2H_1$-**1**):
D_2O, 298 K

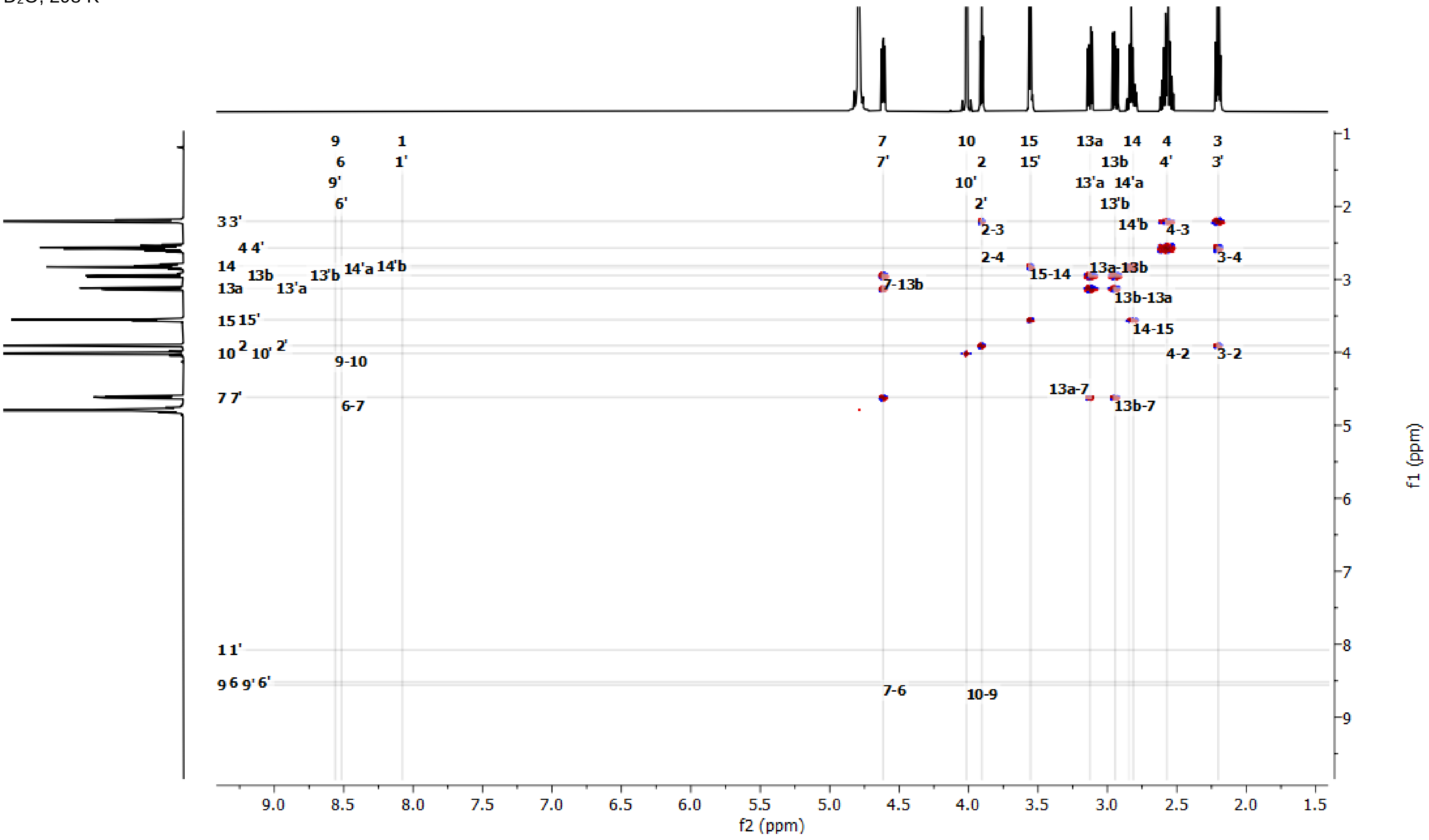

NOESY-NMR of Glutathione–S–C_2H_3D–N_3 (**$^2H_1$-1**):
D_2O, 298 K

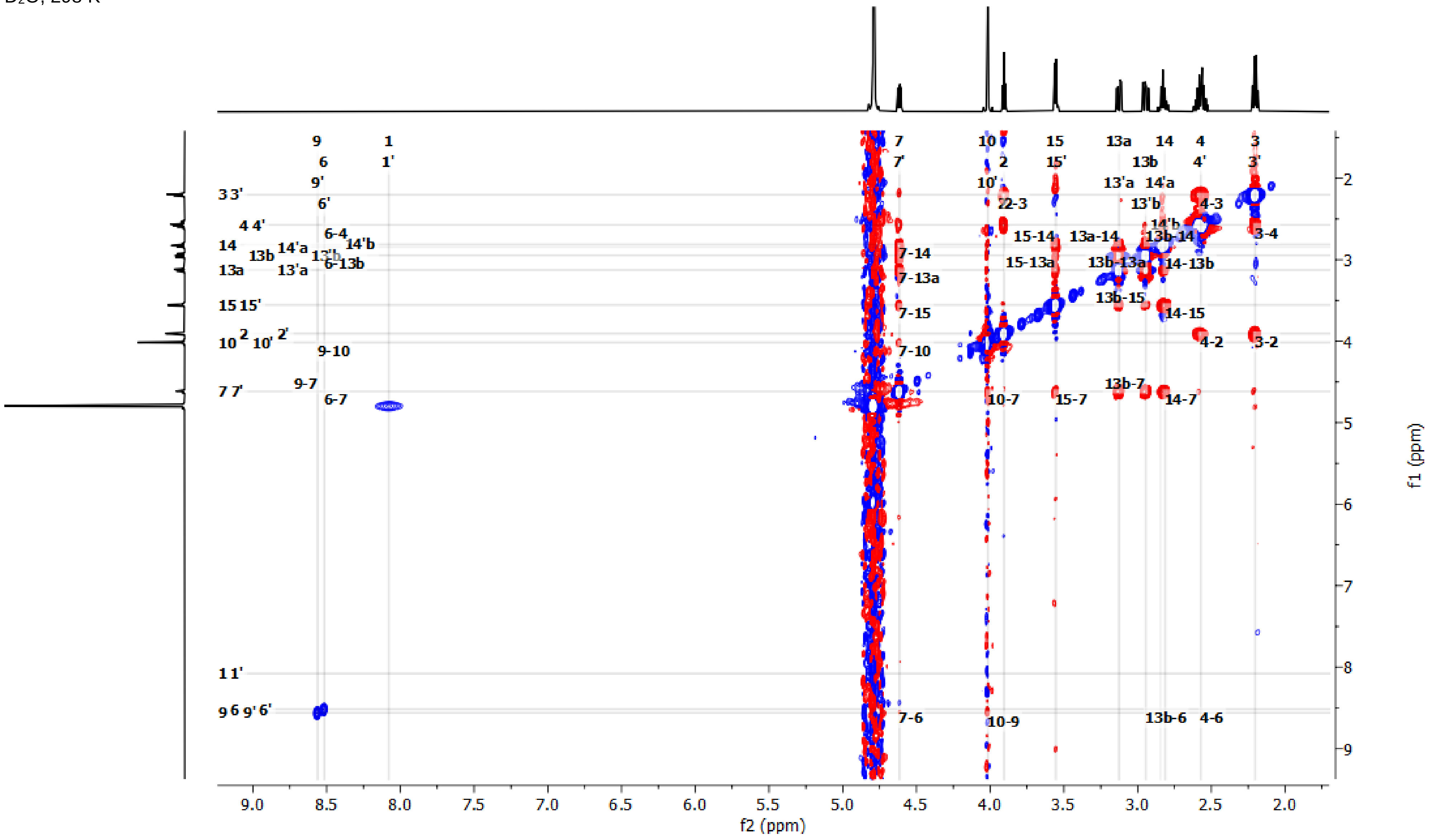

^{15}N HMBC-NMR of Glutathione–S–C_2H_3D–N_3 (**$^2H_1$-1**):
D_2O, 298 K

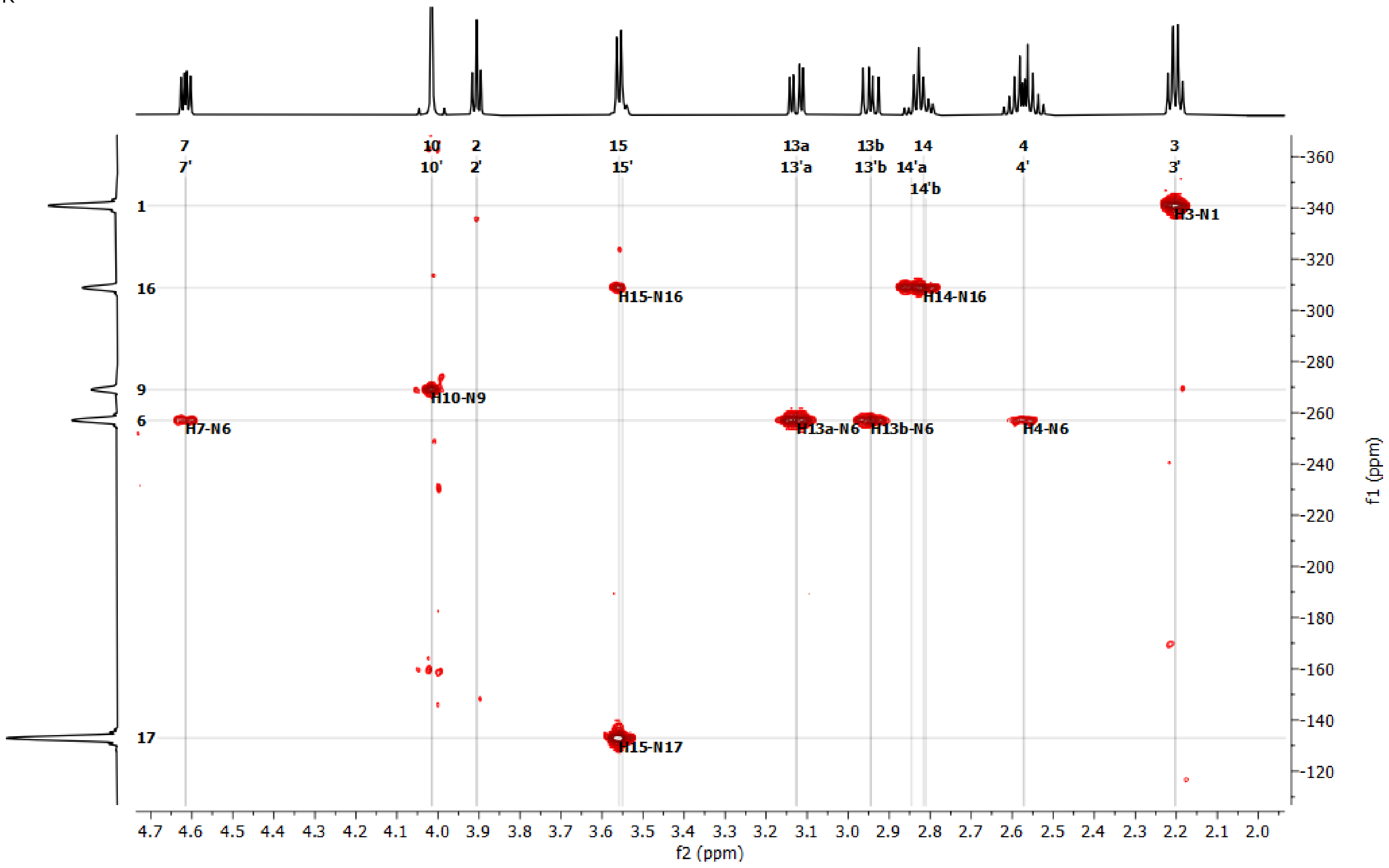

Glutathione–S–C_2H_4–SCN trifluoroacetate (S1)

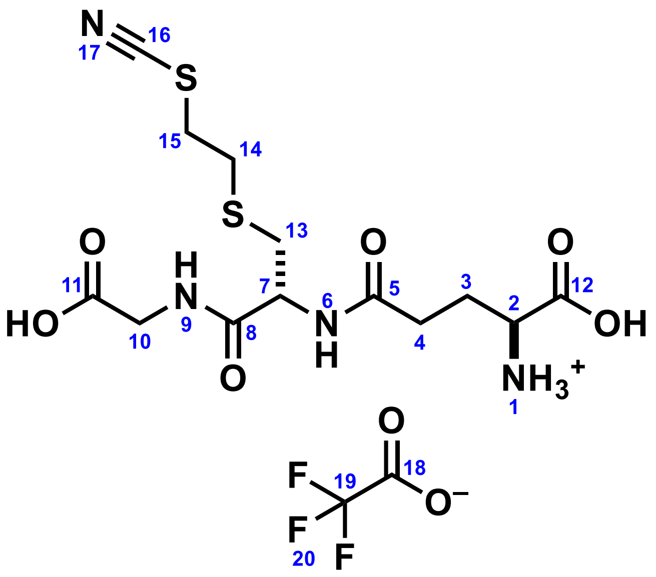

Table S21. Characterization table of Glutathione–S–C_2H_4–SCN trifluoroacetate. The ^{15}N chemical shifts were referenced to NH_3(l).

Atom	δ (ppm)	J	HSQC	^{15}N-HMBC	HMQC	COSY	NOESY
**N1**	39,11			3			
**H1**	8.143?	(weak/exchange, br s)					H_2O
**C2**	53,18		2		3, 4		
**H2**	3,961	t 6.3(3)	2		3, 4, 12	3, 4	3, 4
**C3**	25,84		3		2, 4		
**H3**	2,222	m	3	1	2, 4, 5, 12	2, 4	2, 4
**C4**	31,14		4		2, 3		
**H4**	2,589	m	4		2, 3, 5	2, 3	2, 3, 6
**C5**	174,67				3, 4, 7		
**N6**	123,14			13a, 13b			
**H6**	8,527	(weak/exchange, d 7.5(7))				7	4, 7, 13b
**C7**	53,15		7		13a, 13b		

**H7**	4,635	d 8.5(13b), d 5.2(13a)	7		5, 8, 13	6, 13a, 13b	6, 9, 13a, 13b, 14, 15
**C8**	172,65				7, 10, 13a, 13b		
**N9**	111,15			10			
**H9**	8,572	(weak/exchange, t 5.7(10))				10	7, 10
**C10**	41,29		10				
**H10**	4,031	m	10	9	8, 11	9	9
**C11**	173,12				10		
**C12**	172,77				2, 3		
**C13**	32,78		13a, 13b		7, 14		
**H13a**	3,142	d 5.2(7), d 14.1(13b)	13	6	7, 8, 14	7, 13b	7, 13b, 15
**H13b**	2,970	d 8.5(7), d 14.1(13a)	13	6	7, 8, 14	7, 13a	6, 7, 13a
**C14**	31,74		14		13a, 13b, 15		
**H14**	3,042	m	14		13, 15	15	7
**C15**	33,35		15		14		
**H15**	3,317	m	15		14, 16	14	7, 13a
**C16**	114,72				15		
**N17**	n.f.						
**C18**	163,07	q 35.7(20)					
**C19**	116,44	q 291.9(20)					
**F20**	–75,50	s					

^{1}H NMR of Glutathione–S–C_2H_4–SCN trifluoroacetate **(S1)**:
600 MHz, D_2O, 298 K

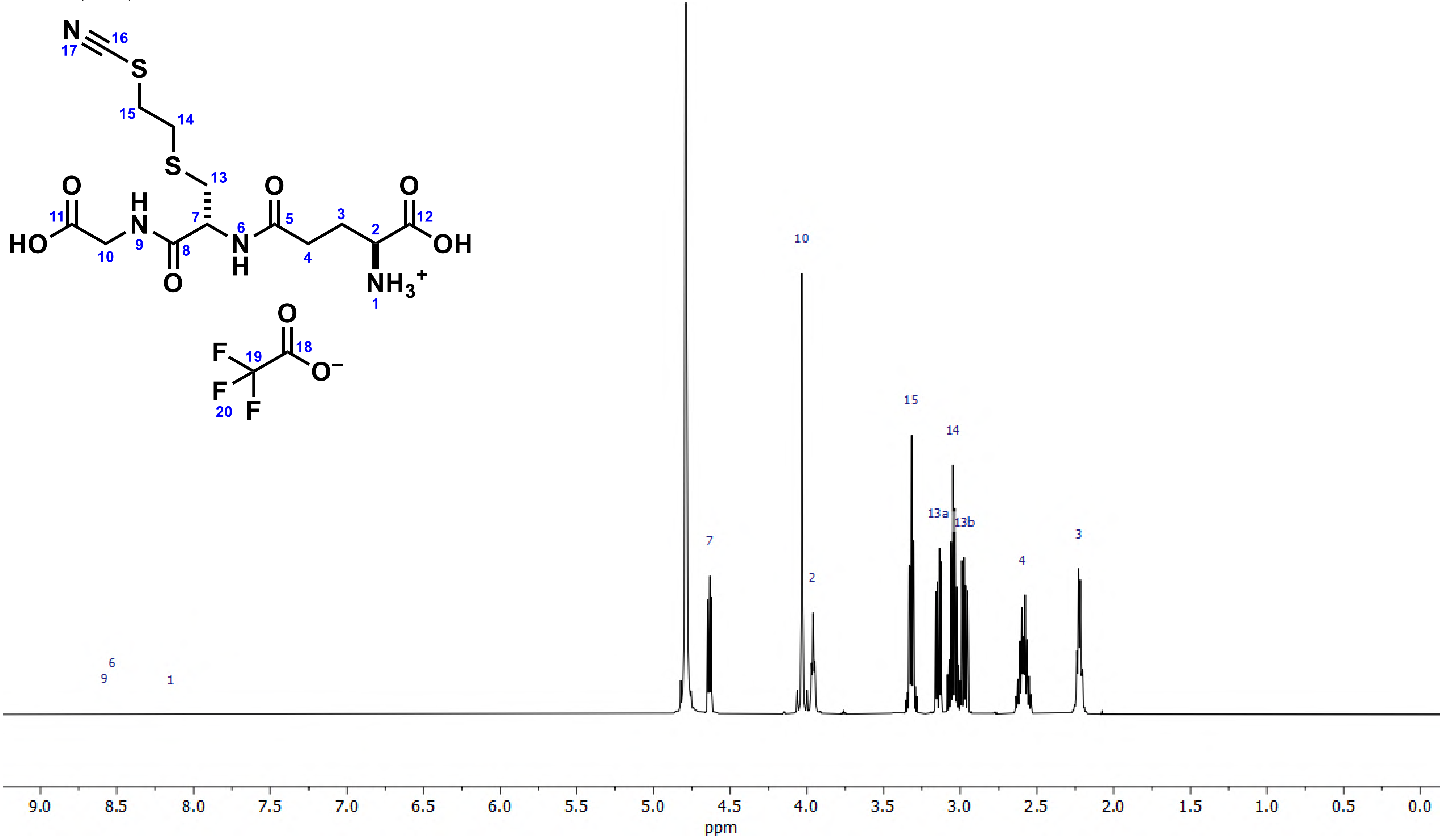

^{13}C NMR of Glutathione–S–C_2H_4–SCN trifluoroacetate **(S1)**:
151 MHz, D_2O, 298 K

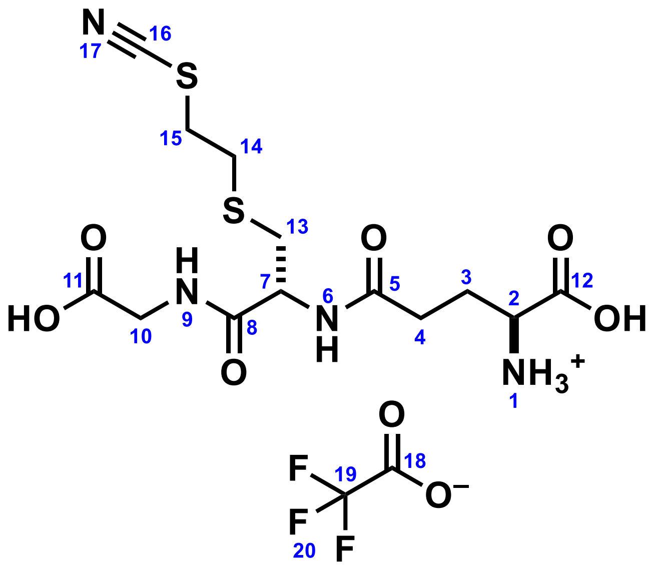

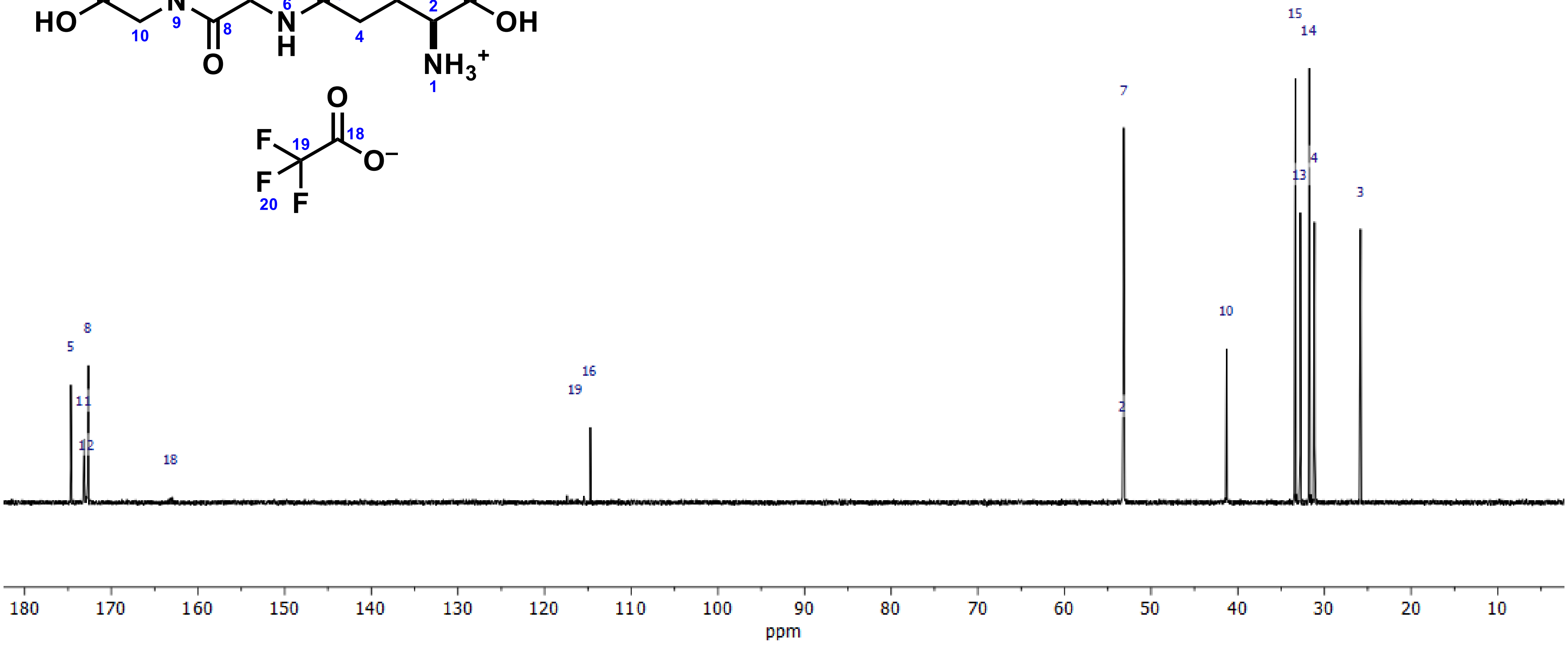

^{19}F NMR of Glutathione–S–C_2H_4–SCN trifluoroacetate **(S1)**:
470 MHz, D_2O, 298 K

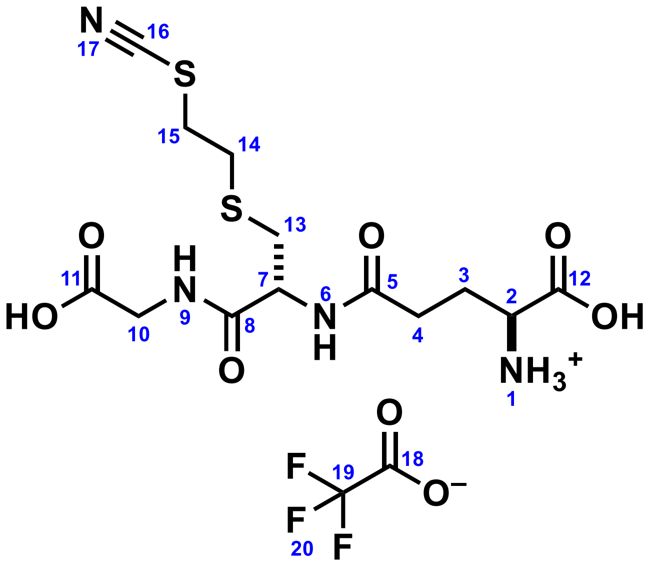

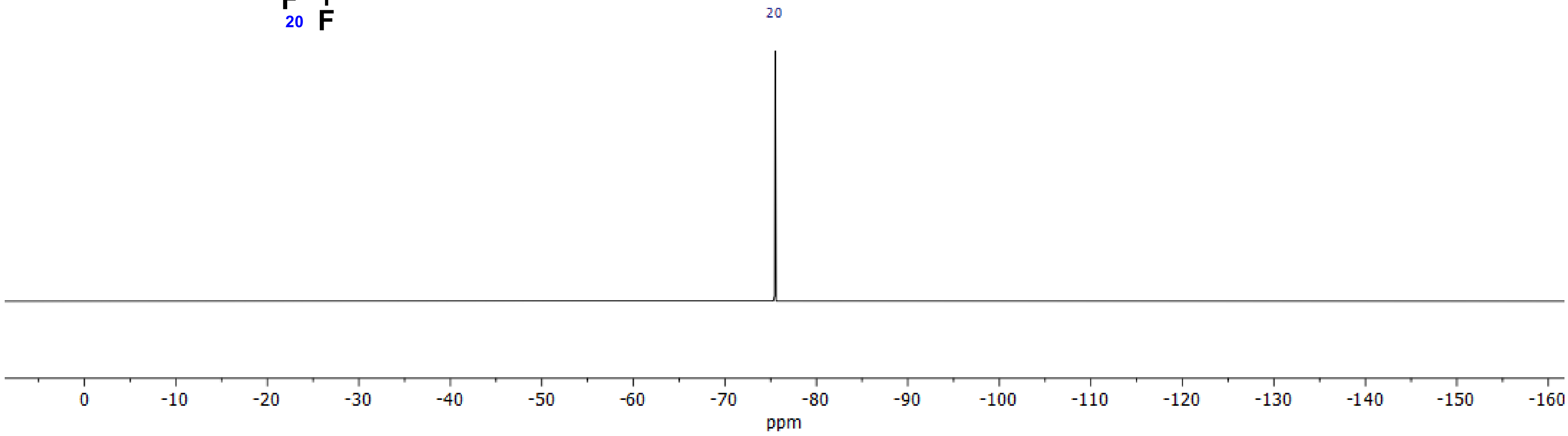

HSQC-NMR of Glutathione–S–C_2H_4–SCN trifluoroacetate **(S1)**:
D_2O, 298 K

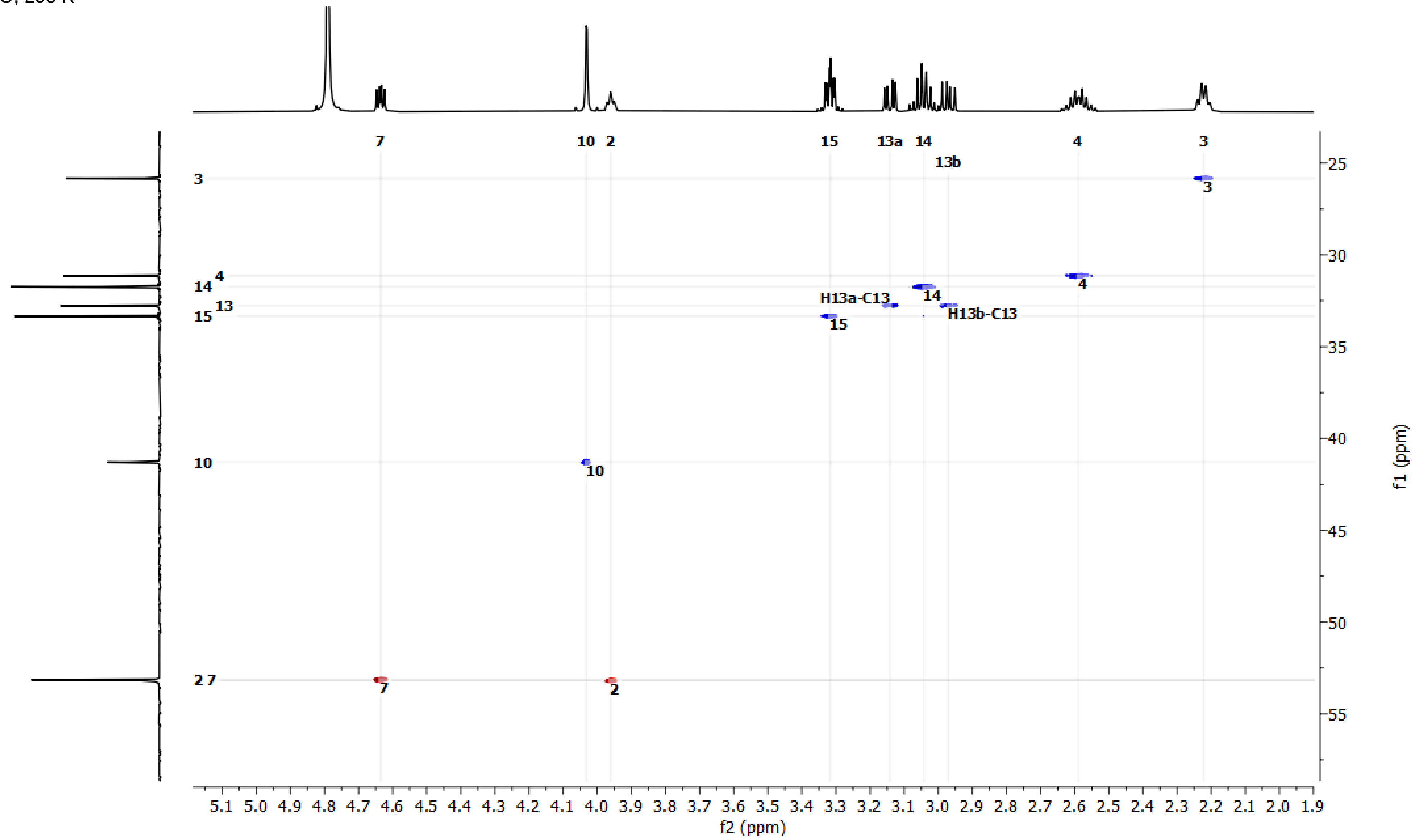

HMQC-NMR of Glutathione–S–C_2H_4–SCN trifluoroacetate **(S1)**:
D_2O, 298 K

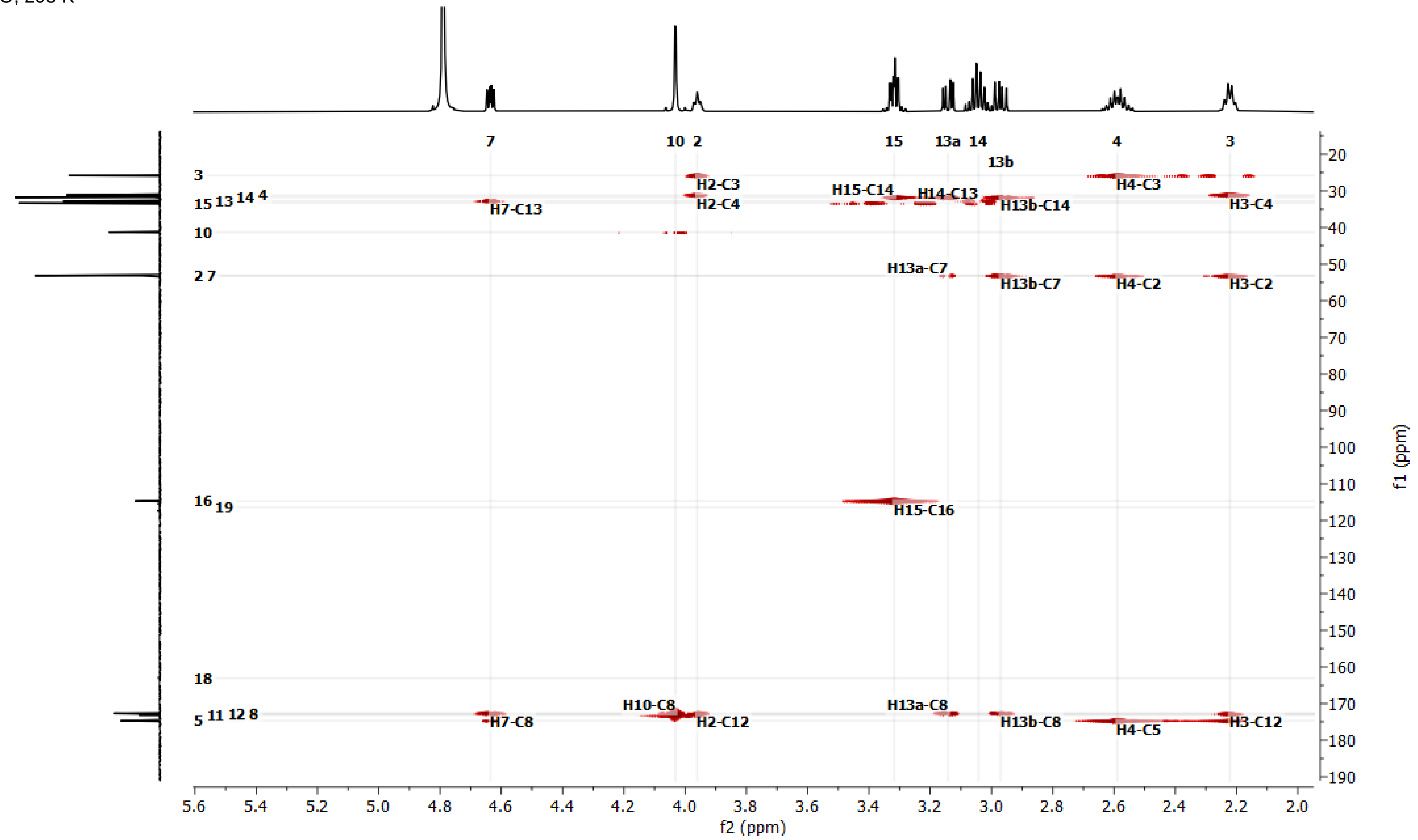

COSY-NMR of Glutathione–S–C_2H_4–SCN trifluoroacetate **(S1)**:
D_2O, 298 K

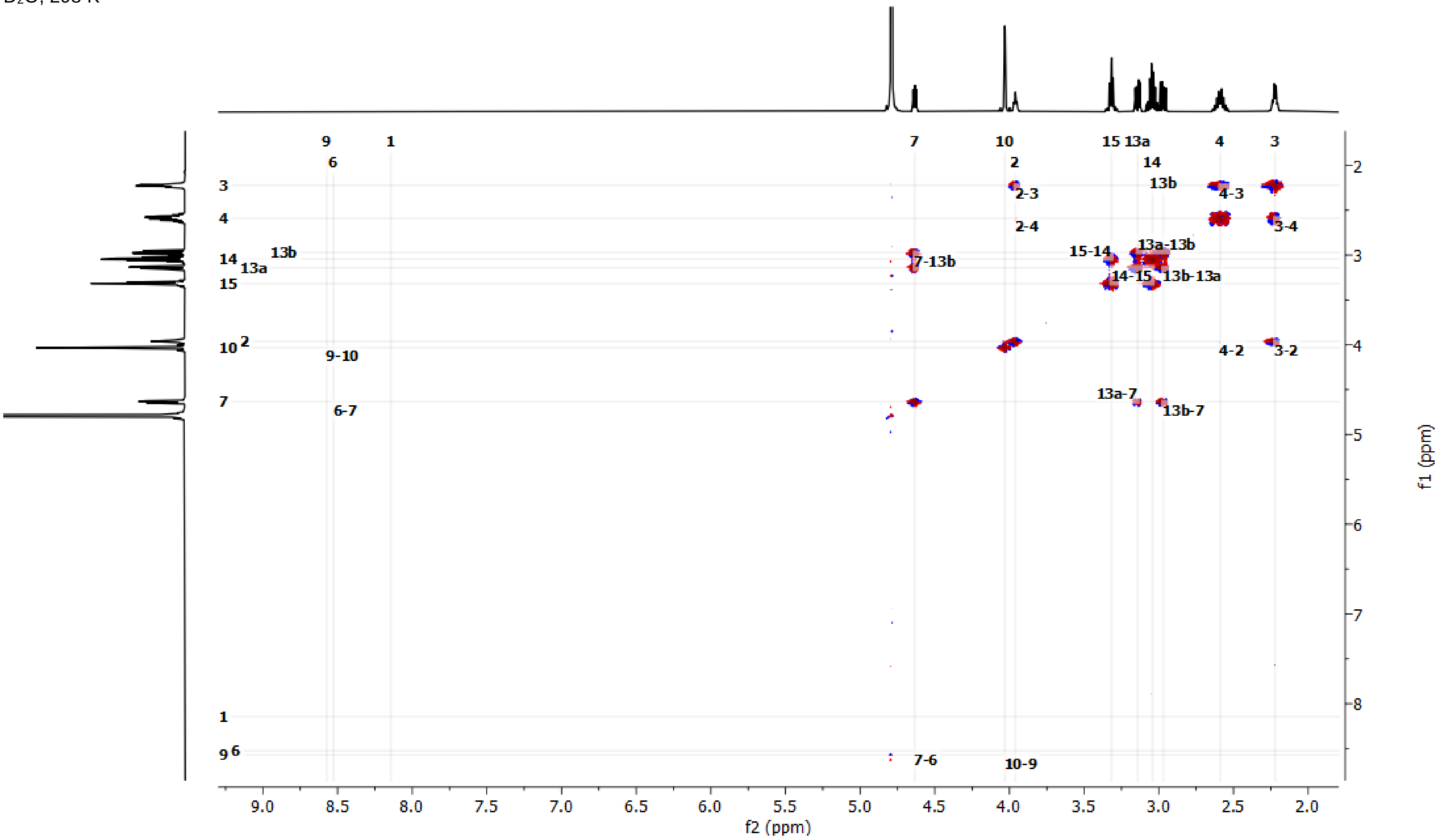

NOESY-NMR of Glutathione–S–C_2H_4–SCN trifluoroacetate **(S1)**:
D_2O, 298 K

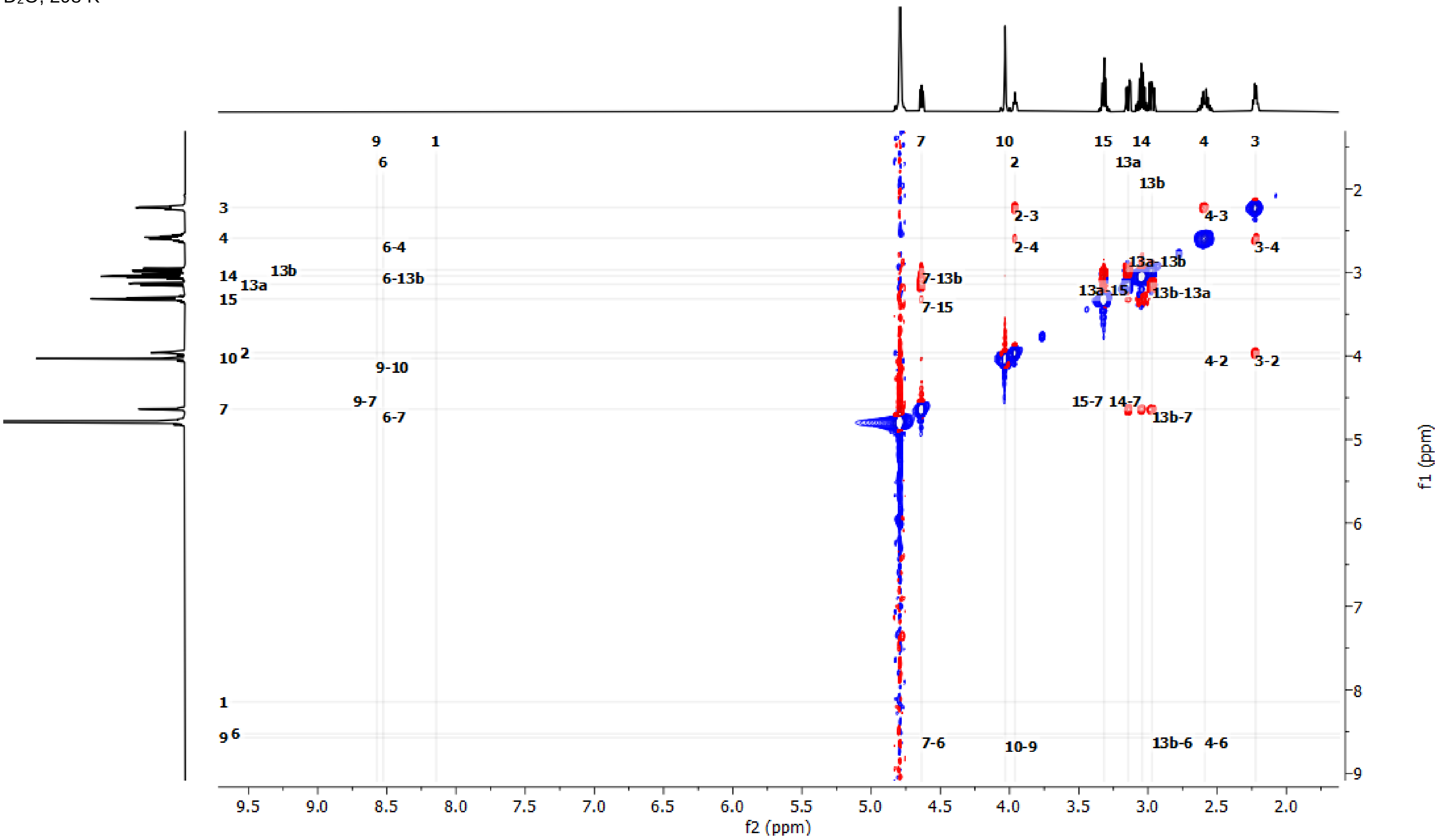

^{15}N-HMBC-NMR of Glutathione–S–C_2H_4–SCN trifluoroacetate **(S1)**:
D_2O, 298 K

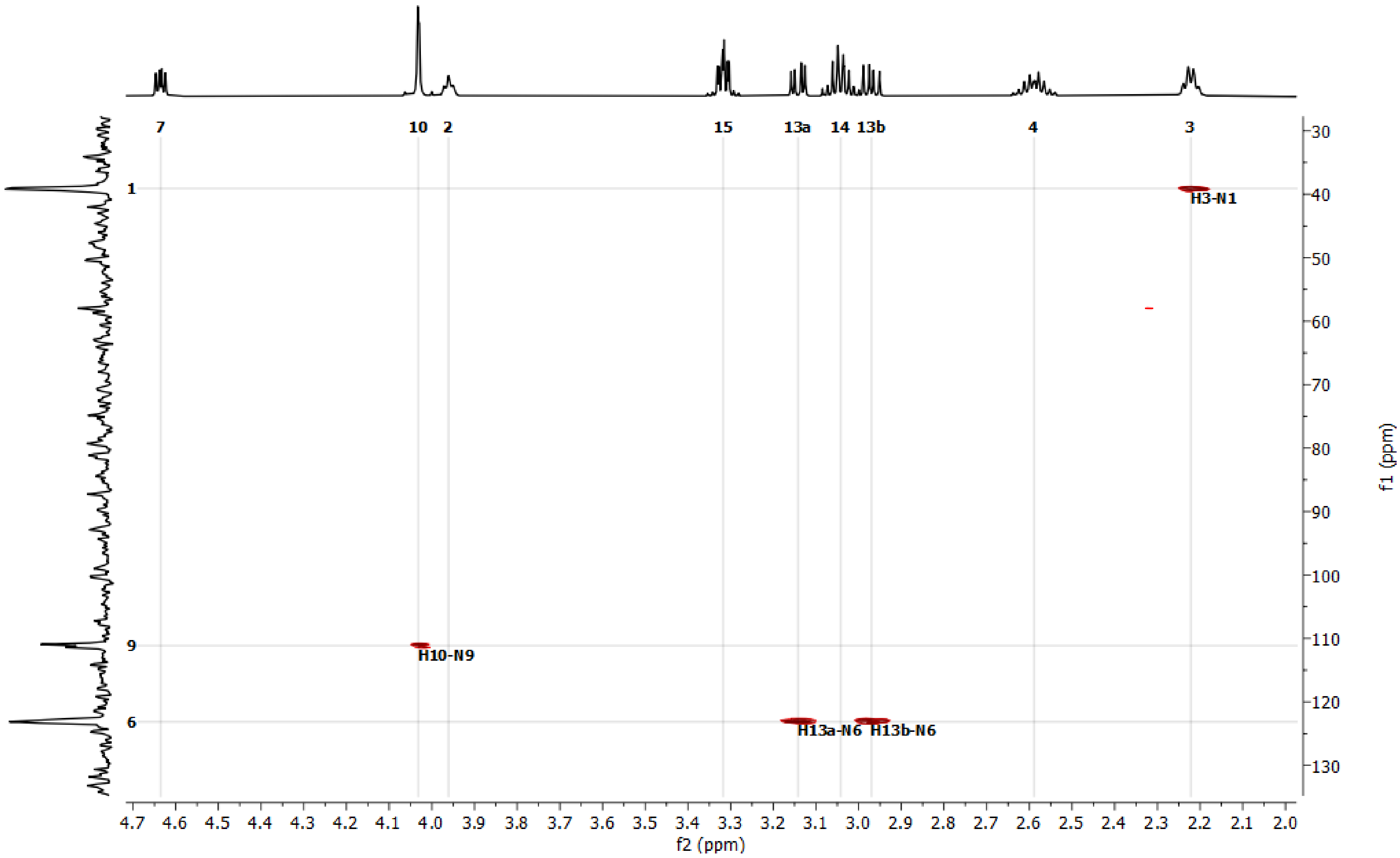

Glutathione–S–C_2H_4–N-aniline trifluoroacetate (S2):

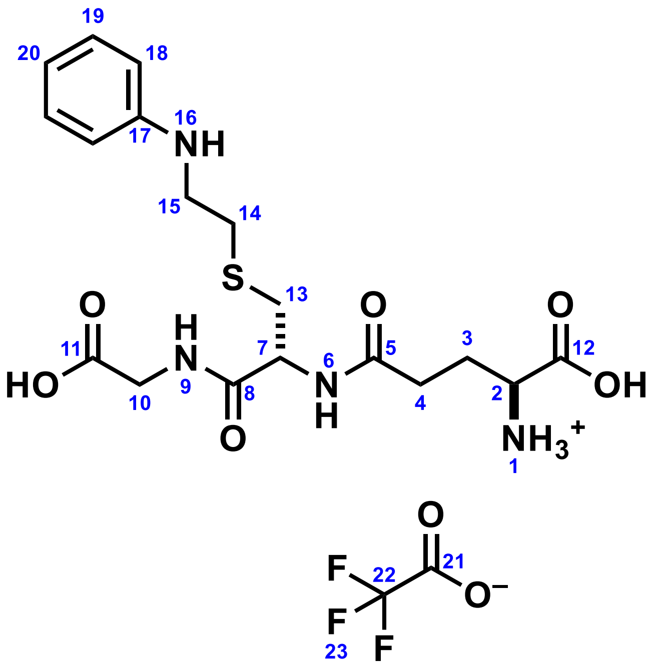

Table S22. Characterization table of Glutathione–S–C_2H_4–N-aniline trifluoroacetate. The ^{15}N chemical shifts were referenced to NH_3(l).

Atom	δ (ppm)	J	HSQC	^{15}N-HMBC	HMQC	COSY	NOESY
**N1**	39,92			3			
**H1**	7.96?	(weak/exchange, br)					
**C2**	53,85		2		3, 4		
**H2**	3,816	t 6.4(3)	2		3, 4, 12	3, 4	3, 4
**C3**	26,06		3		2, 4		
**H3**	2,16	m	3	1	2, 4, 5, 12	2, 4	2, 4
**C4**	31,299		4		2, 3		
**H4**	2,540	m	4		2, 3, 5	2, 3	2, 3, 7
**C5**	174,89				3, 4, 7		
**N6**	123,04			7, 13a, 13b			
**H6**	8,540	(weak/exchange, d 7.7(7))				7	

**C7**	53,00		7		13a, 13b		
**H7**	4,598	d 8.2(13b), d 5.3(13a)	7	6	5, 8, 13	6, 13a, 13b	4, 10, 13a, 13b, 14, 15
**C8**	172,43				7, 10, 13a, 13b		
**N9**	112,25			10			
**H9**	8,512	(weak/exchange, t 6.1(10))				10	
**C10**	41,74		10				
**H10**	3,945	s	10	9	8, 11	9	7
**C11**	173,78				10		
**C12**	173,64				2, 3		
**C13**	32,62		13a, 13b		7, 14		
**H13a**	3,102	d 5.3(7), d 14.2(13b)	13	6	7, 8, 14	7, 13b	7, 13b, 14, 15
**H13b**	2,942	d 8.2(7), d 14.2(13a)	13	6	7, 8, 14	7, 13a	7, 13a, 15
**C14**	27,18		14		13a, 13b, 15		
**H14**	2,886	m	14	16	13, 15	15	7, 13a, 15, 18
**C15**	49,52		15		14		
**H15**	3,670	t 6.9(14)	15		14, 17	14	7, 13a, 13b, 14, 18
**N16**	57,13			14, 18			
**H16**	n.f.						
**C17**	135,22				15, 19		
**C18**	121,97		18		20		
**H18**	7,448	m	18	16	20	19, 20	14, 15
**C19**	130,46		19				
**H19**	7,584	m	19		17	18, 20	
**C20**	129,13		20		18		
**H20**	7,520	m	20		18	18, 19	
**C21**	163,06	q 35.3(23)					
**C22**	116,42	q 291.5(23)					
**F23**	-75,50	s					

^{1}H NMR of Glutathione–S–C_2H_4–N-aniline trifluoroacetate **(S2)**:
600 MHz, D_2O, 298 K

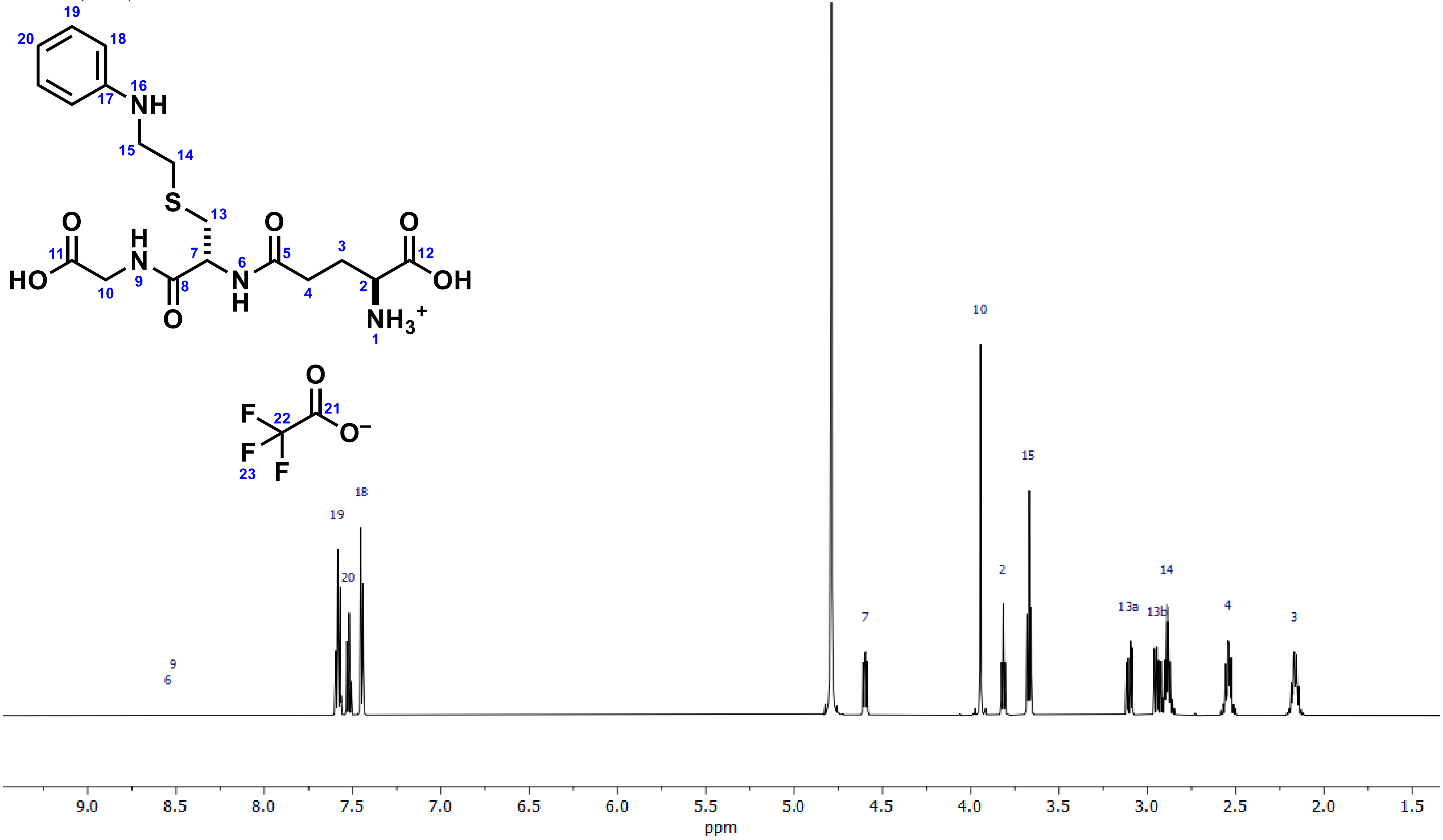

^{13}C NMR of Glutathione–S–C_2H_4–N-aniline trifluoroacetate **(S2)**:
151 MHz, D_2O, 298 K

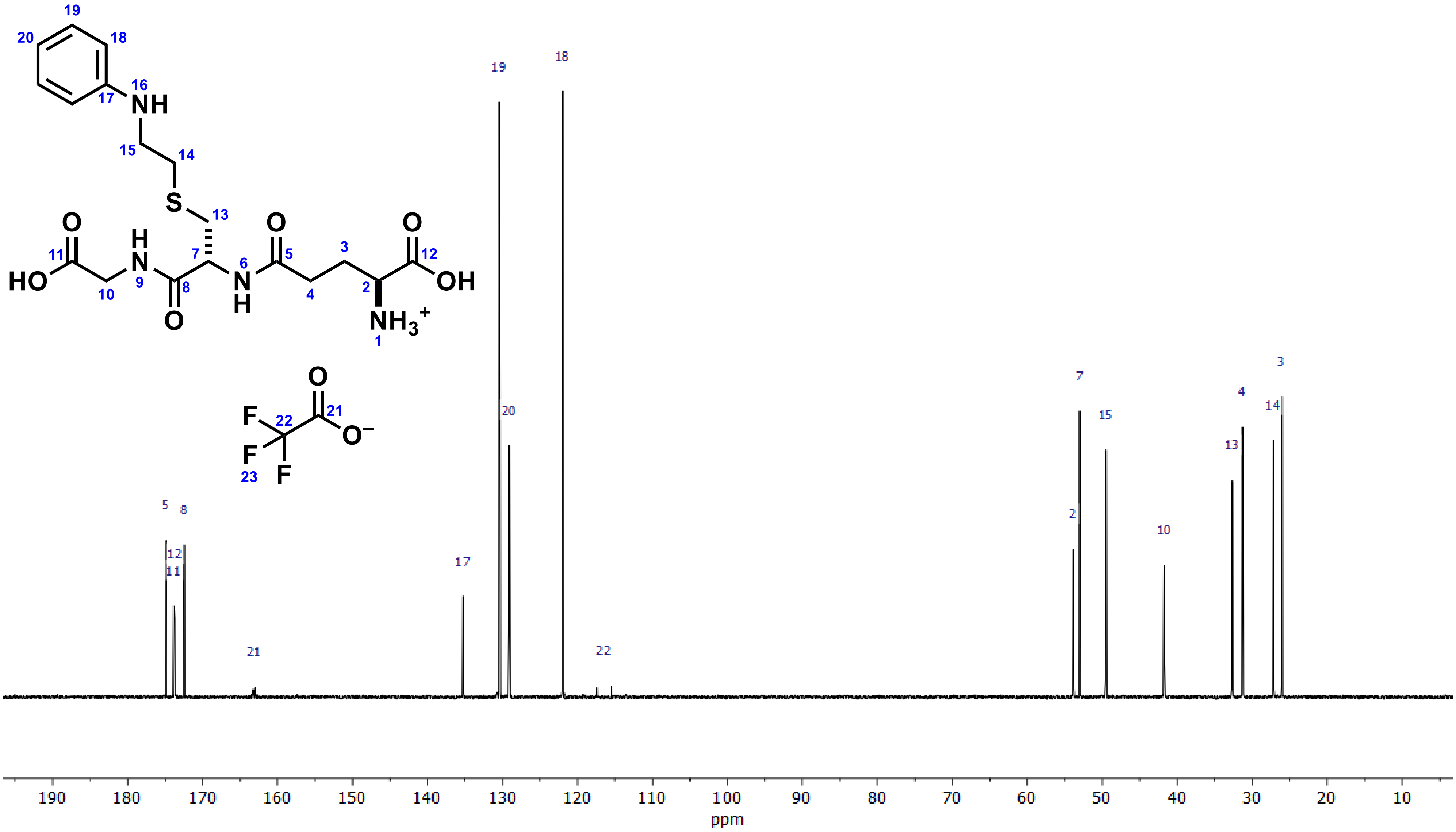

^{19}F NMR of Glutathione–S–C_2H_4–N-aniline trifluoroacetate **(S2)**:
470 MHz, D_2O, 298 K

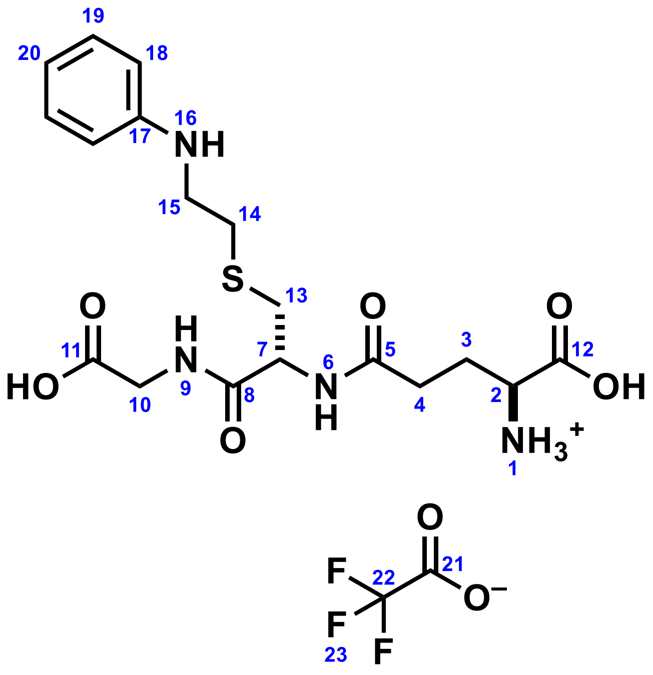

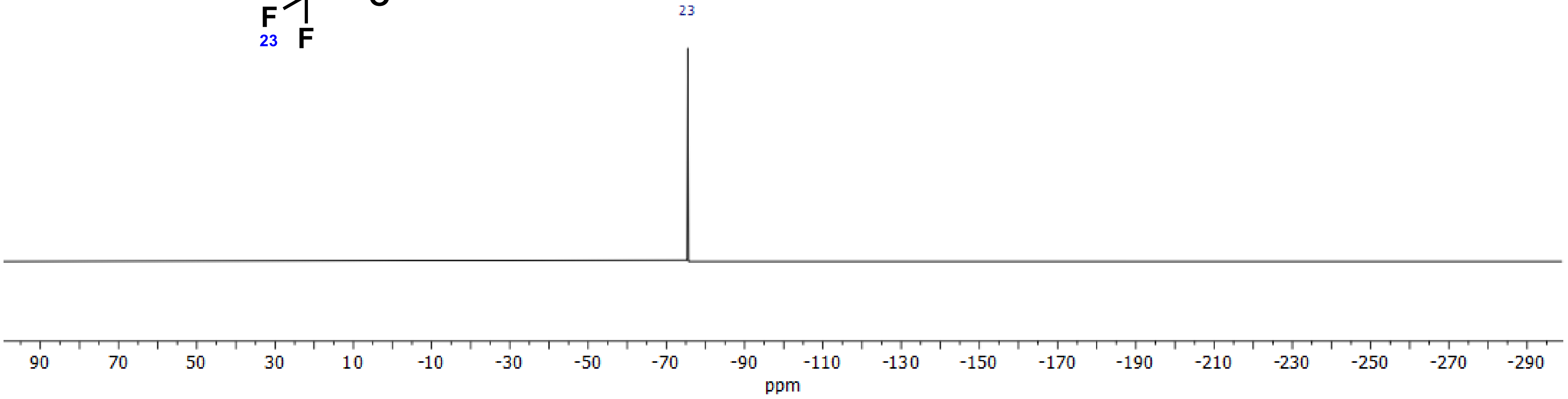

HSQC-NMR of Glutathione–S–C_2H_4–N-aniline trifluoroacetate **(S2)**:
D_2O, 298 K

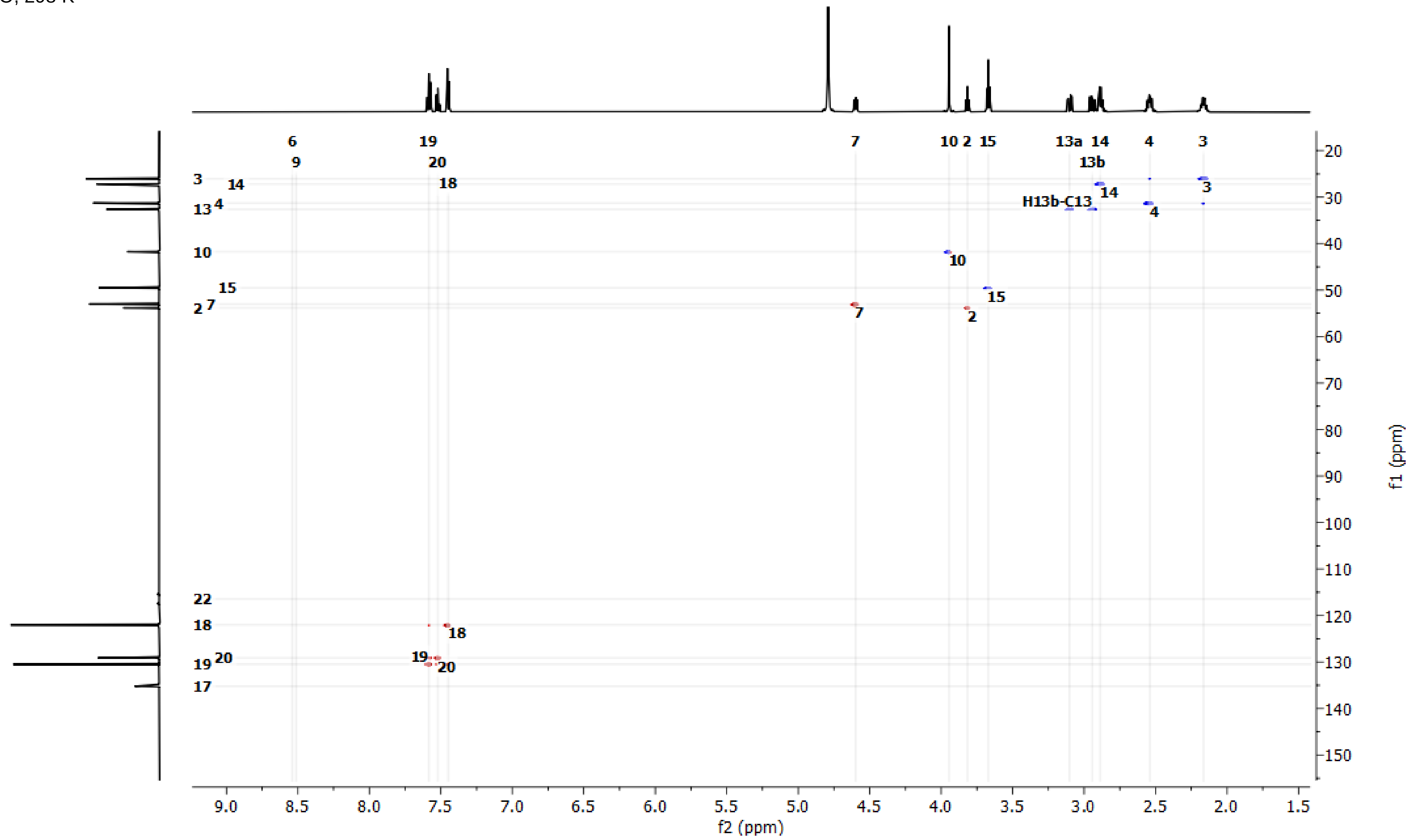

HMQC-NMR of Glutathione–S–C_2H_4–N-aniline trifluoroacetate **(S2)**:
D_2O, 298 K

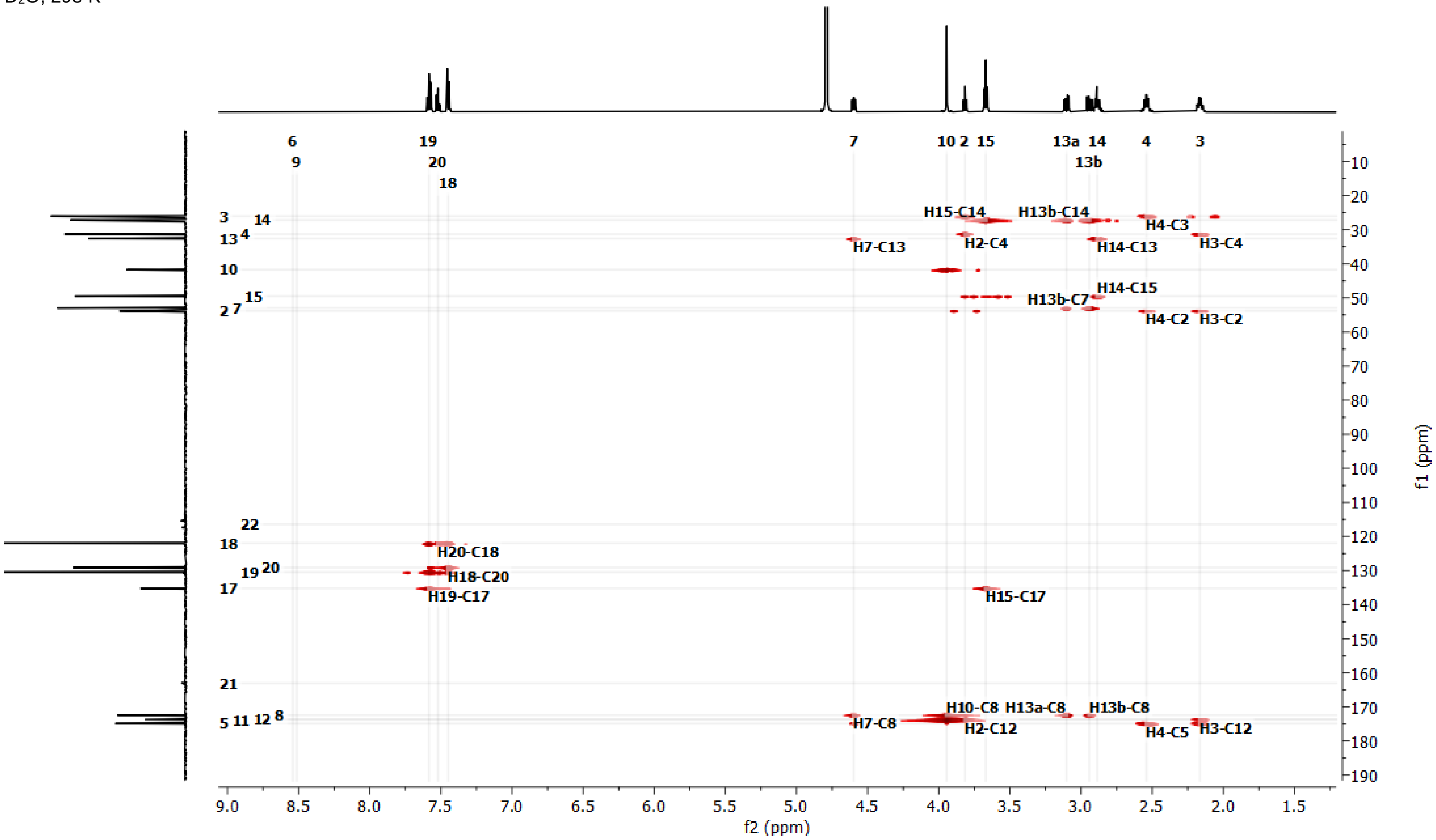

COSY-NMR of Glutathione–S–C_2H_4–N-aniline trifluoroacetate **(S2)**:
D_2O, 298 K

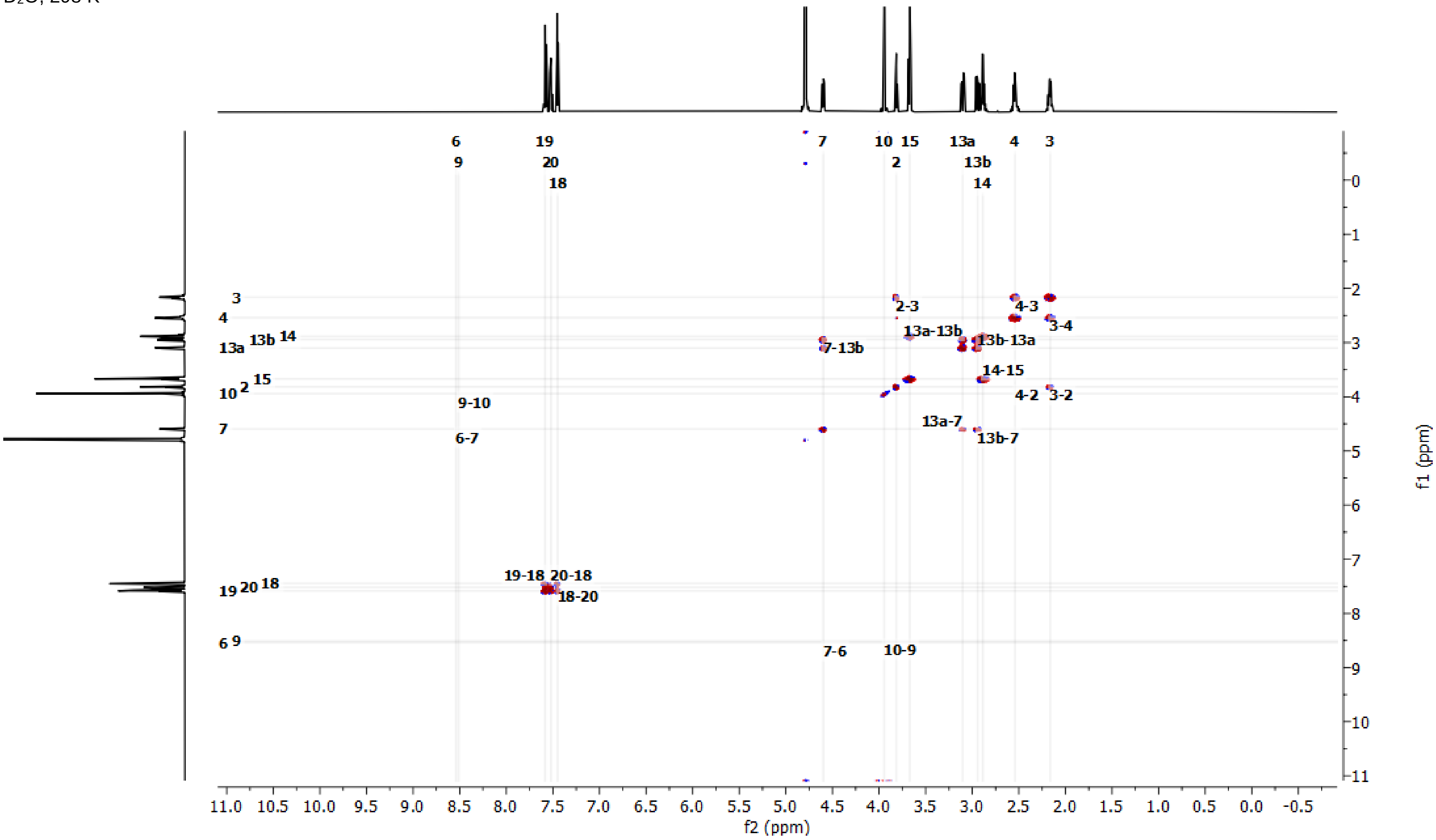

NOESY-NMR of Glutathione–S–C_2H_4–N-aniline trifluoroacetate **(S2)**:
D_2O, 298 K

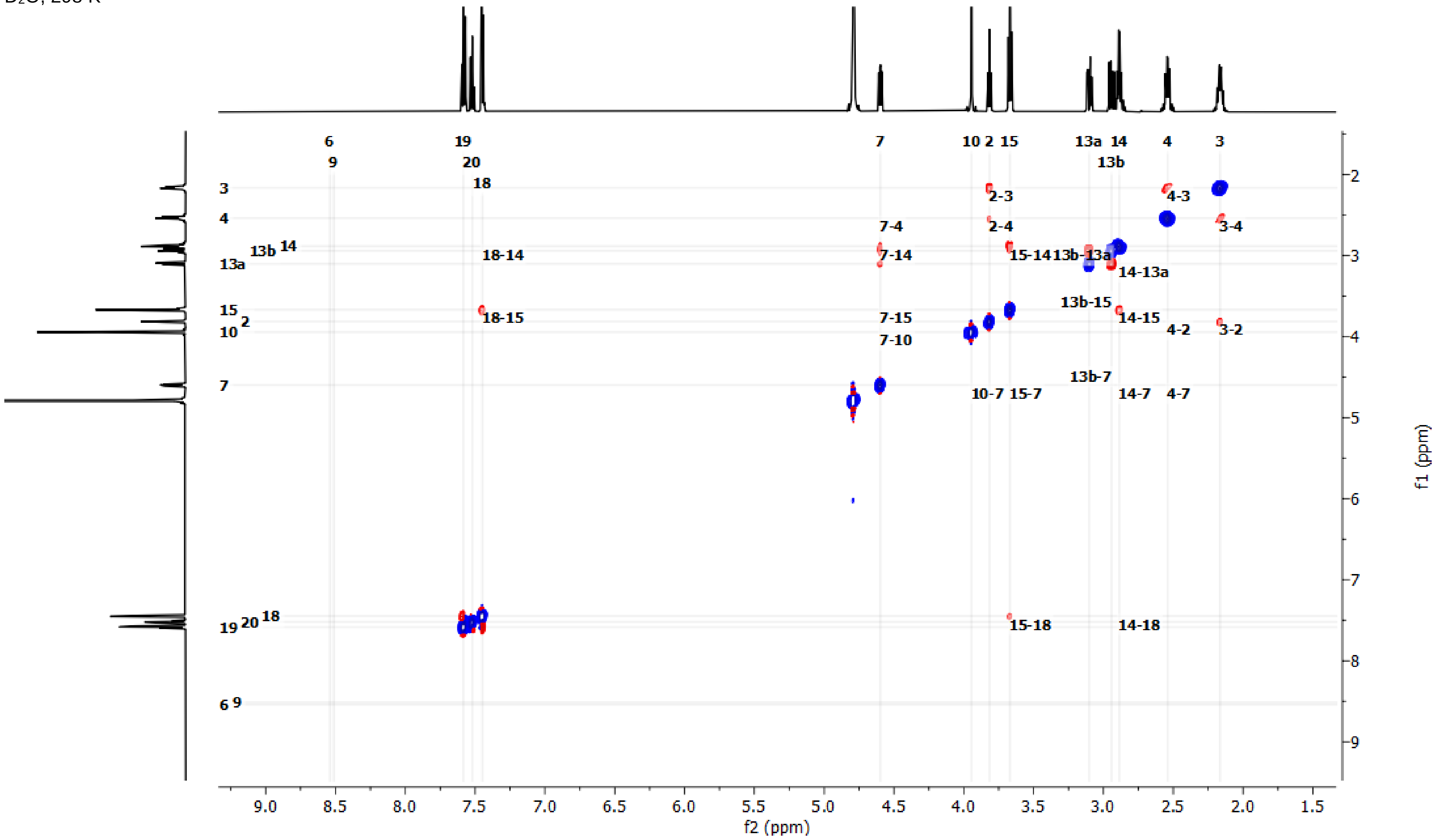

^{15}N-HMBC-NMR of Glutathione–S–C_2H_4–N-aniline trifluoroacetate **(S2)**:
D_2O, 298 K

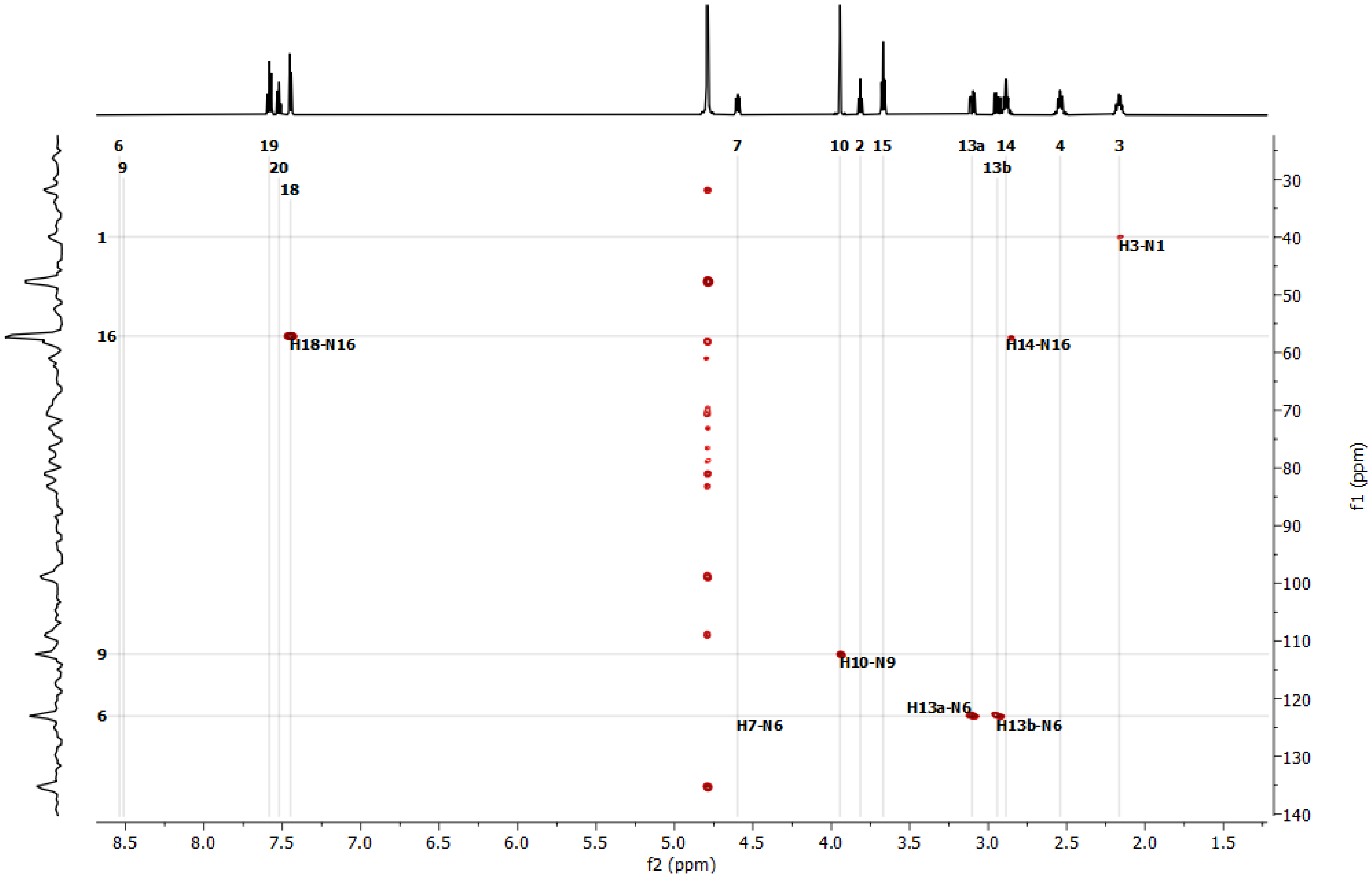

Glutathione–S–C_2H_4–N-(4-fluoroaniline) trifluoroacetate (S3):

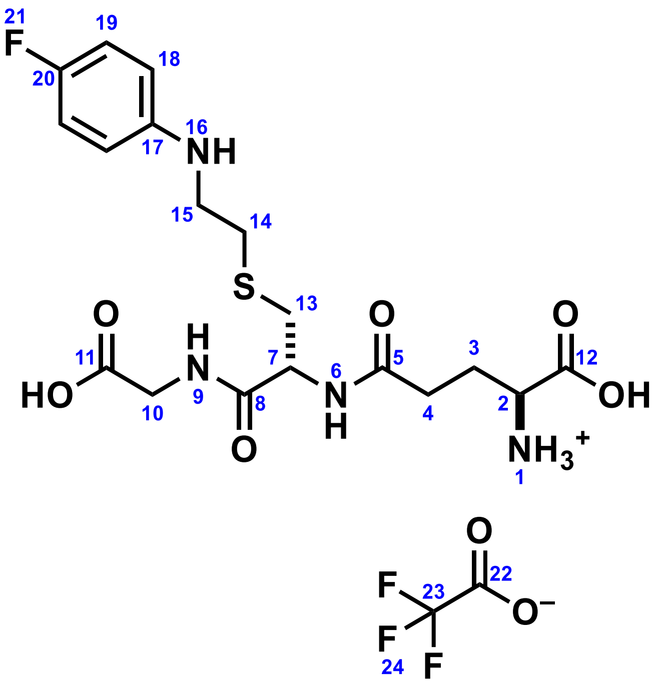

Table S23. Characterization table of Glutathione–S–C_2H_4–N-(4-fluoroaniline) trifluoroacetate. The ^{15}N chemical shifts were referenced to NH_3(l).

Atom	δ (ppm)	J	HSQC	^{15}N-HMBC	HMQC	COSY	NOESY
**N1**	39,71			3			
**H1**	n.f.						
**C2**	53,70		2		3, 4		
**H2**	3,841	t 6.4(3)	2		3, 4, 12	3	3, 4
**C3**	25,99		3		2, 4		
**H3**	2,174	m	3	1	2, 4, 5, 12	2, 4	2
**C4**	31,25		4		2, 3		
**H4**	2,551	m	4		2, 3, 5	3	2
**C5**	174,84				3, 4, 7		
**N6**	122,89			13a, 13b			

**H6**	8,548	(weak/exchange, m)					
**C7**	52,97		7		13a, 13b		
**H7**	4,602	d 8.2(13b), d 5.3(13a)	7		5, 8, 13	13a, 13b	13a, 13b, 14, 15
**C8**	172,49				7, 10, 13a, 13b		
**N9**	111,56			10			
**H9**	8,548	(weak/exchange, m)					
**C10**	41,53		10				
**H10**	3,972	m	10	9	8, 11		
**C11**	173,50				10		
**C12**	173,47				2, 3		
**C13**	32,56		13a, 13b		7, 14		
**H13a**	3,101	d 5.3(7), d 14.2(13b)	13	6	7, 8, 14	7, 13b	7, 13b, 14, 15
**H13b**	2,942	d 8.2(7), d 14.2(13a)	13	6	7, 8, 14	7, 13a	7, 13a, 15
**C14**	26,97		14		13a, 13b, 15		
**H14**	2,892	m	14	16	13, 15	15	7, 13a, 15, 18
**C15**	49,98		15		14		
**H15**	3,670	t 6.8(14)	15		14, 17	14	7, 13a, 13b, 14, 18
**N16**	55,38			14, 18			
**H16**	n.f.						
**C17**	130,73	d 2.9(21)			15, 18, 19		
**C18**	124,47	d 9.1(21)	18				
**H18**	7,503	dm 4.5(21)	18	16	17, 20	19	14, 15
**C19**	117,33	d 23.6(21)	19				
**H19**	7,326	dm 8.3(21)	19		17, 20	18	
**C20**	162,45	d 247.0(21)			18, 19		
**F21**	-111,97	t 8.3(19), t 4.5(18)					
**C22**	163,05	q 35.0(24)					
**C23**	116,40	q 292.5(24)					
**F24**	-75,51	s					

^{1}H NMR of Glutathione–S–C_2H_4–N-(4-fluoroaniline) trifluoroacetate **(S3)**:
600 MHz, D_2O, 298 K

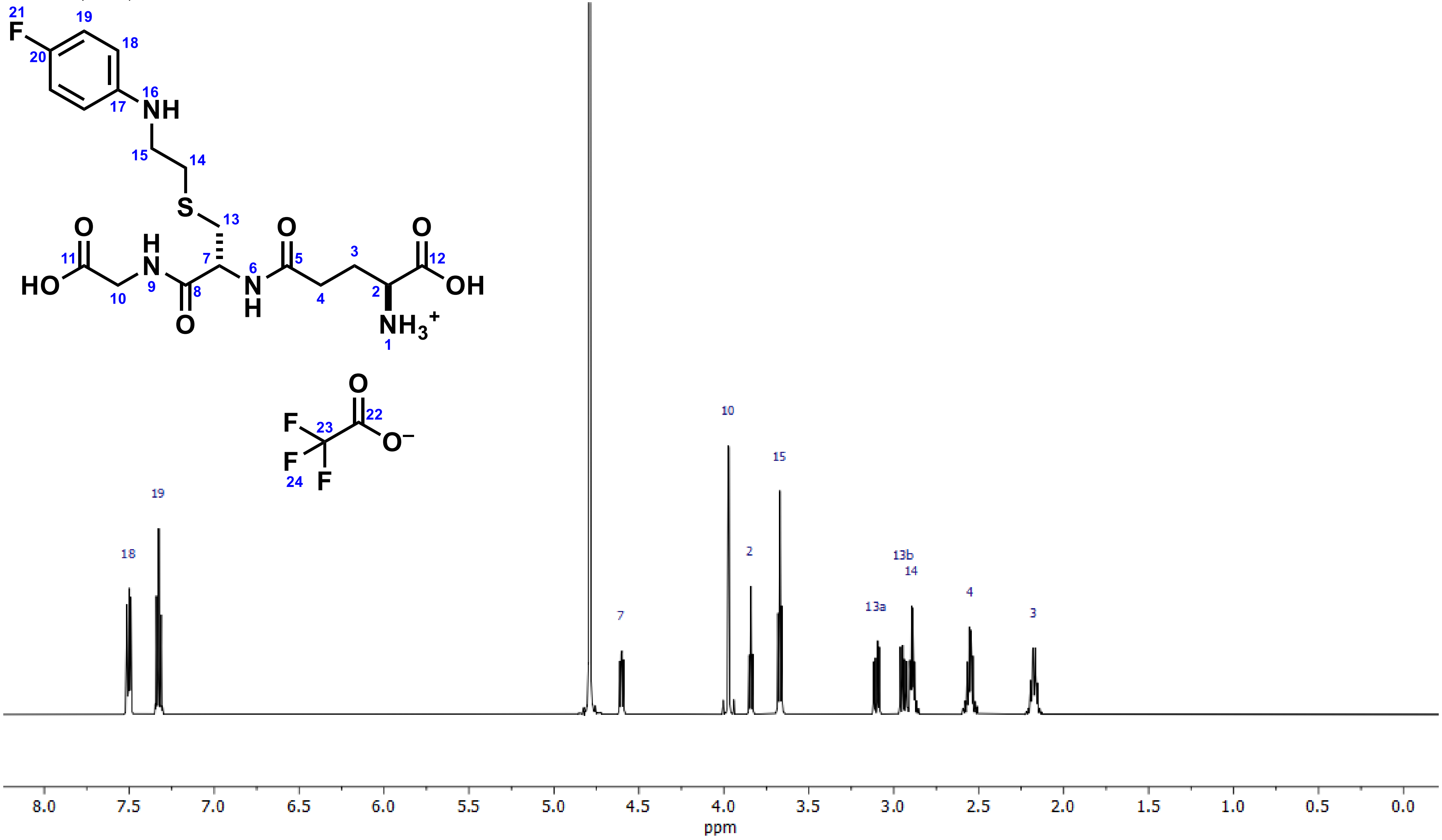

^{13}C NMR of Glutathione–S–C_2H_4–N-(4-fluoroaniline) trifluoroacetate **(S3)**:
151 MHz, D_2O, 298 K

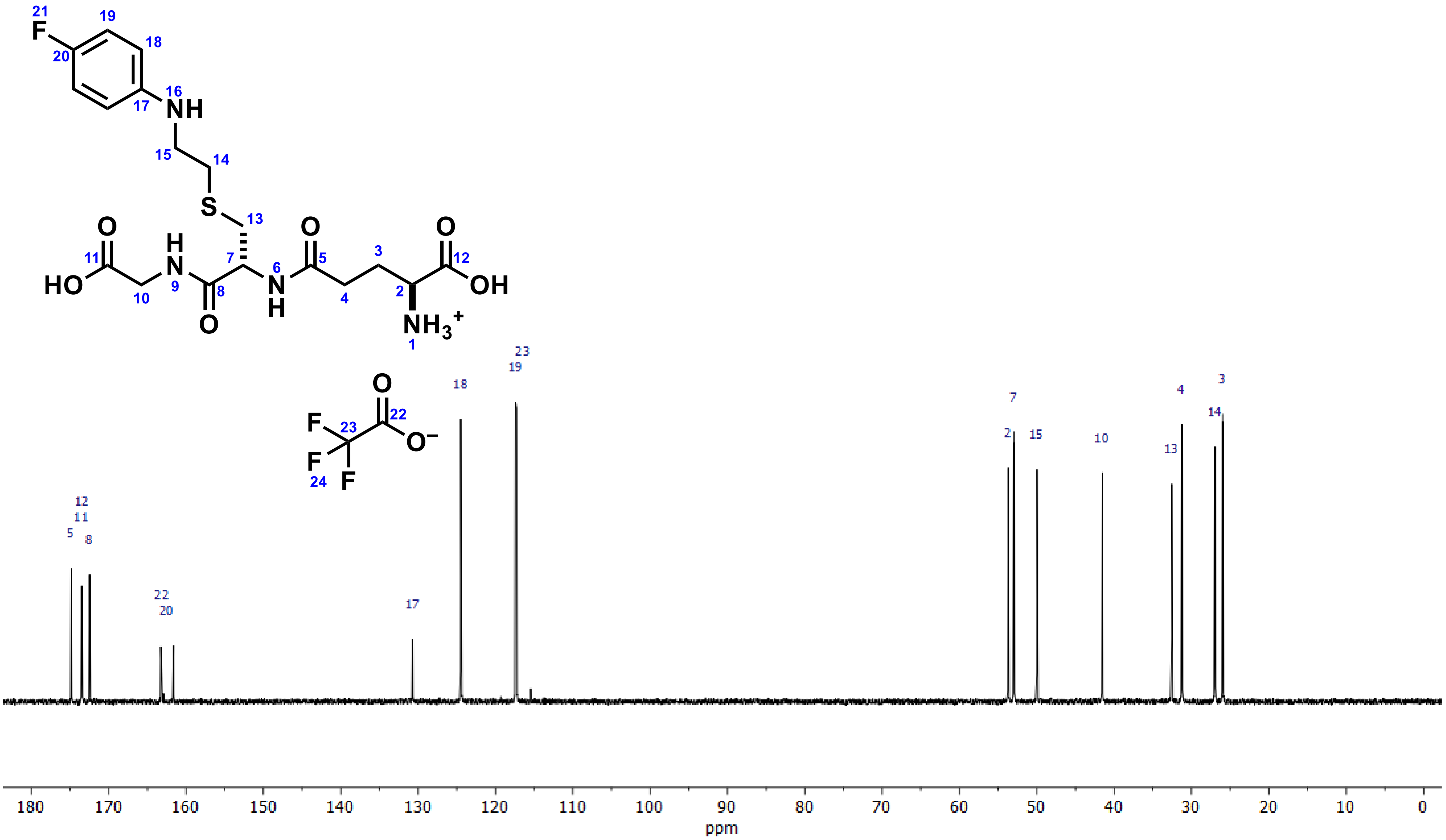

^{19}F NMR of Glutathione–S–C_2H_4–N-(4-fluoroaniline) trifluoroacetate **(S3)**:
470 MHz, D_2O, 298 K

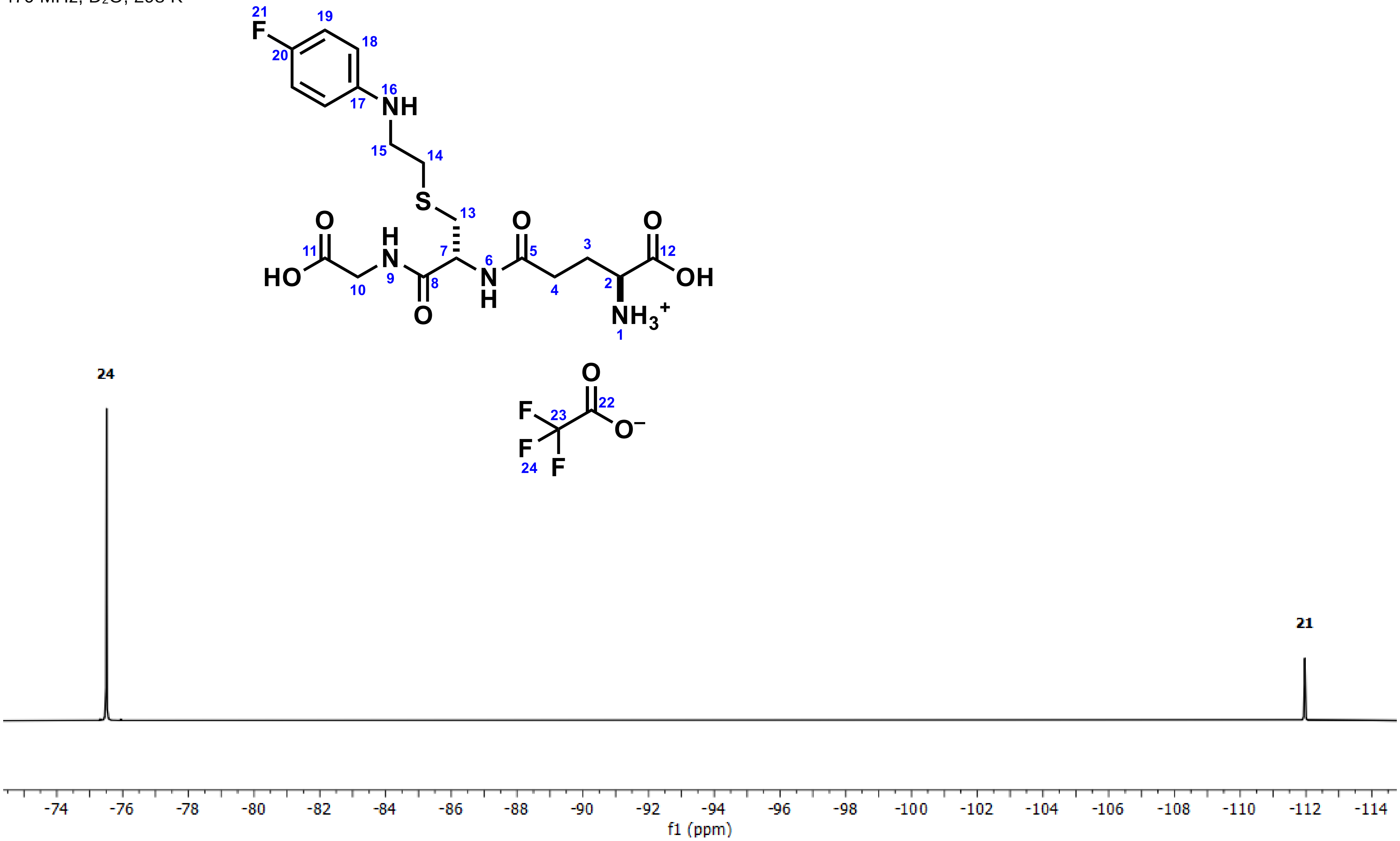

HSQC-NMR of Glutathione–S–C_2H_4–N-(4-fluoroaniline) trifluoroacetate **(S3)**:
D_2O, 298 K

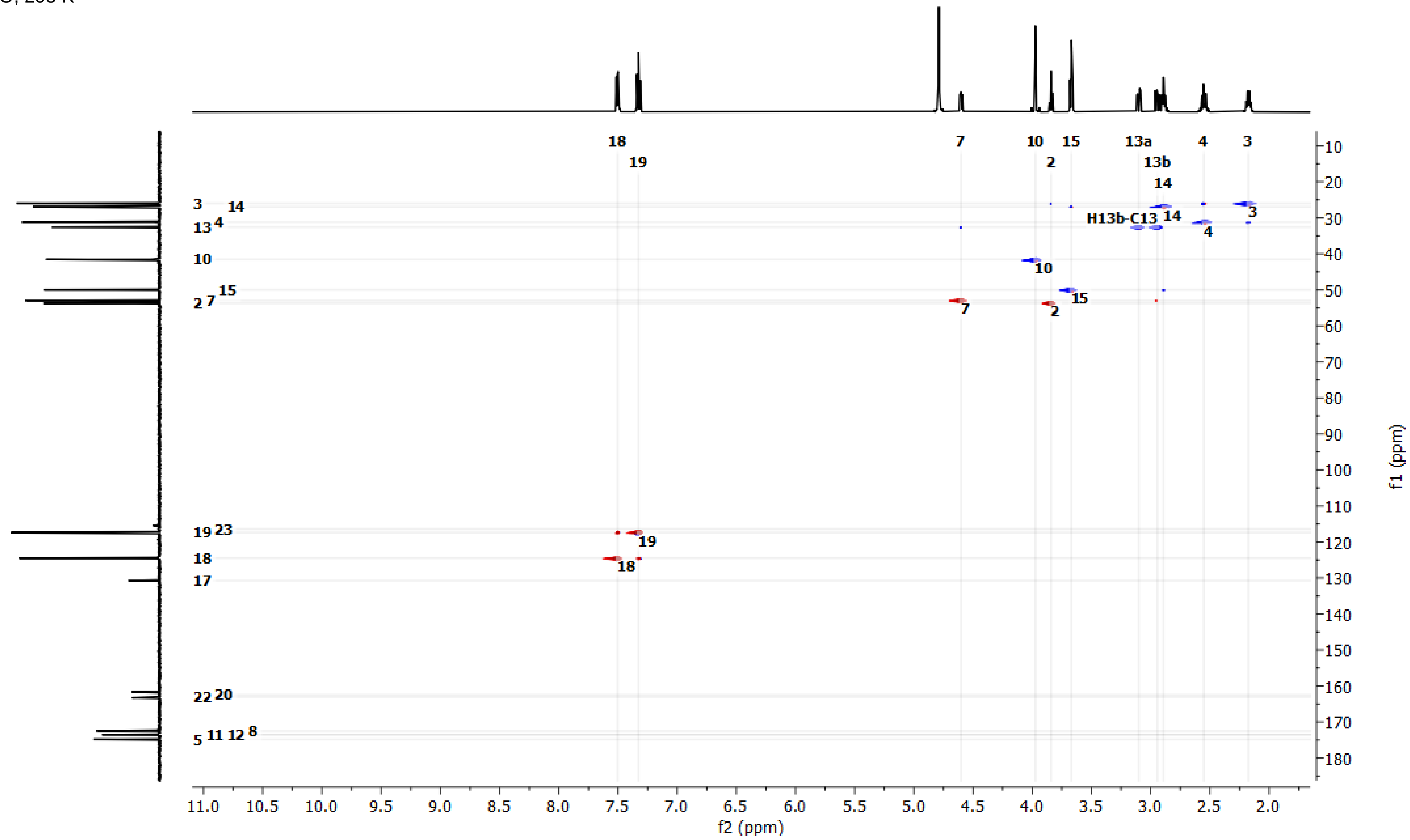

HMQC-NMR of Glutathione–S–C_2H_4–N-(4-fluoroaniline) trifluoroacetate **(S3)**:
D_2O, 298 K

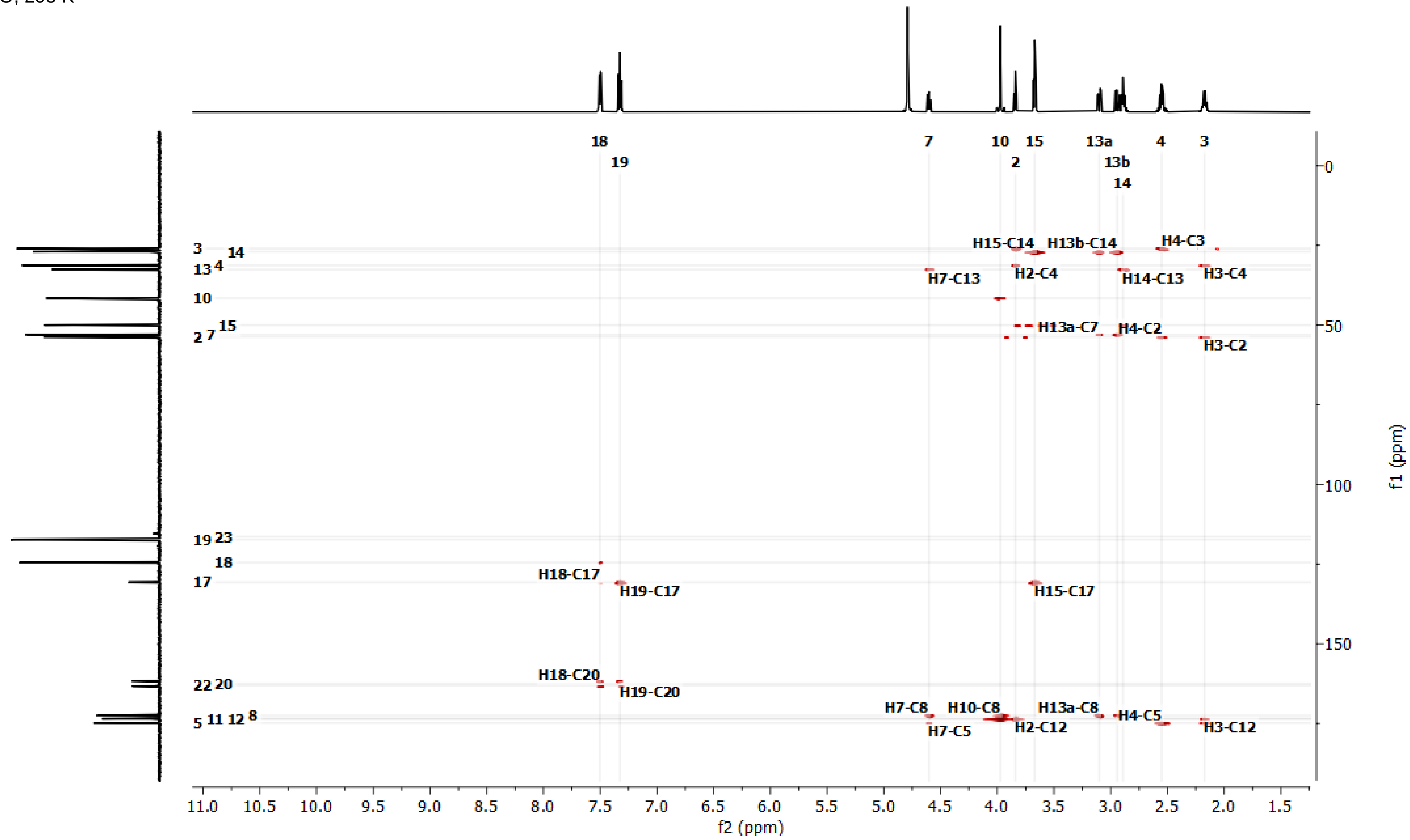

COSY-NMR of Glutathione–S–C_2H_4–N-(4-fluoroaniline) trifluoroacetate **(S3)**:
D_2O, 298 K

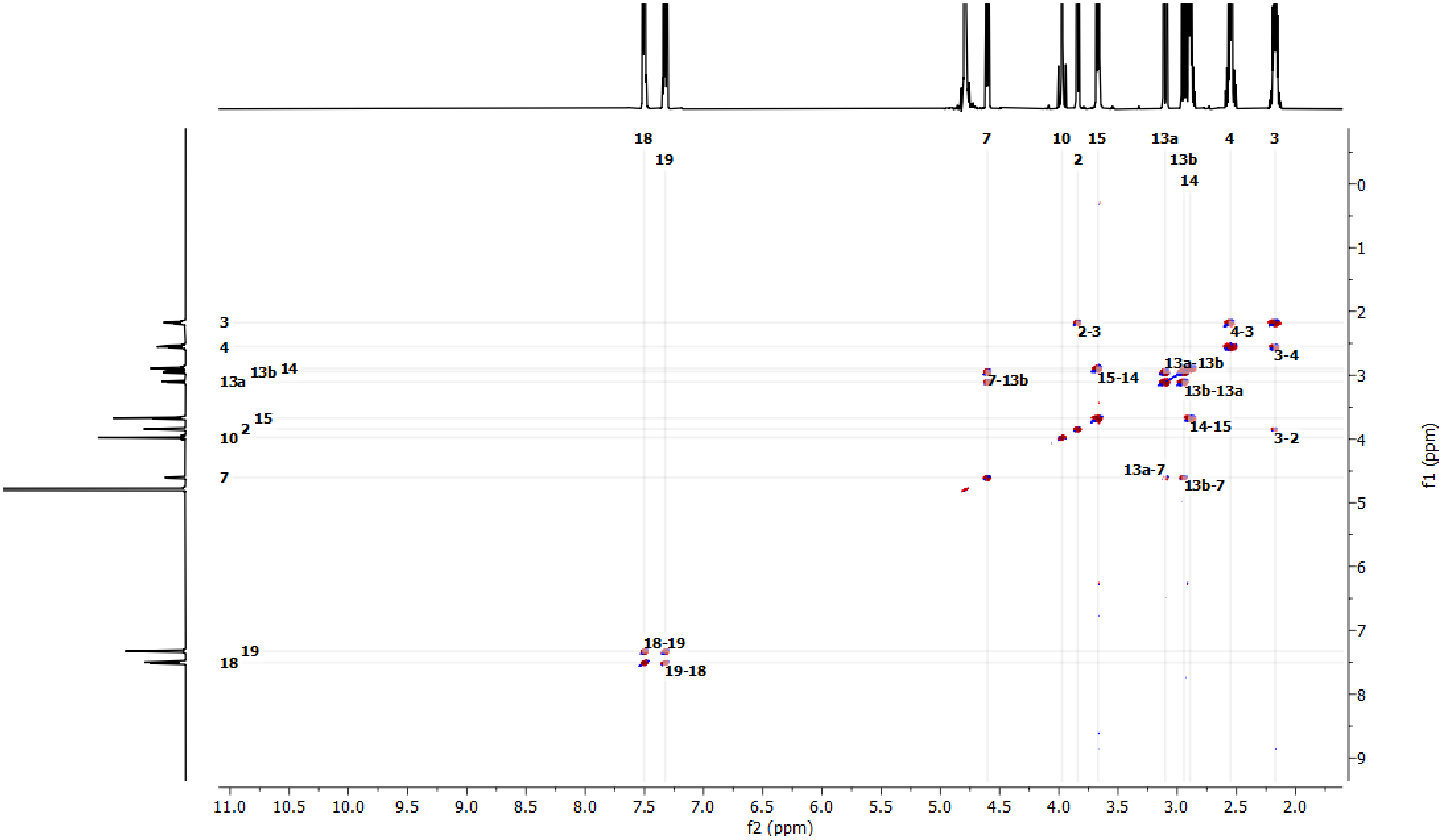

NOESY-NMR of Glutathione–S–C_2H_4–N-(4-fluoroaniline) trifluoroacetate **(S3)**:
D_2O, 298 K

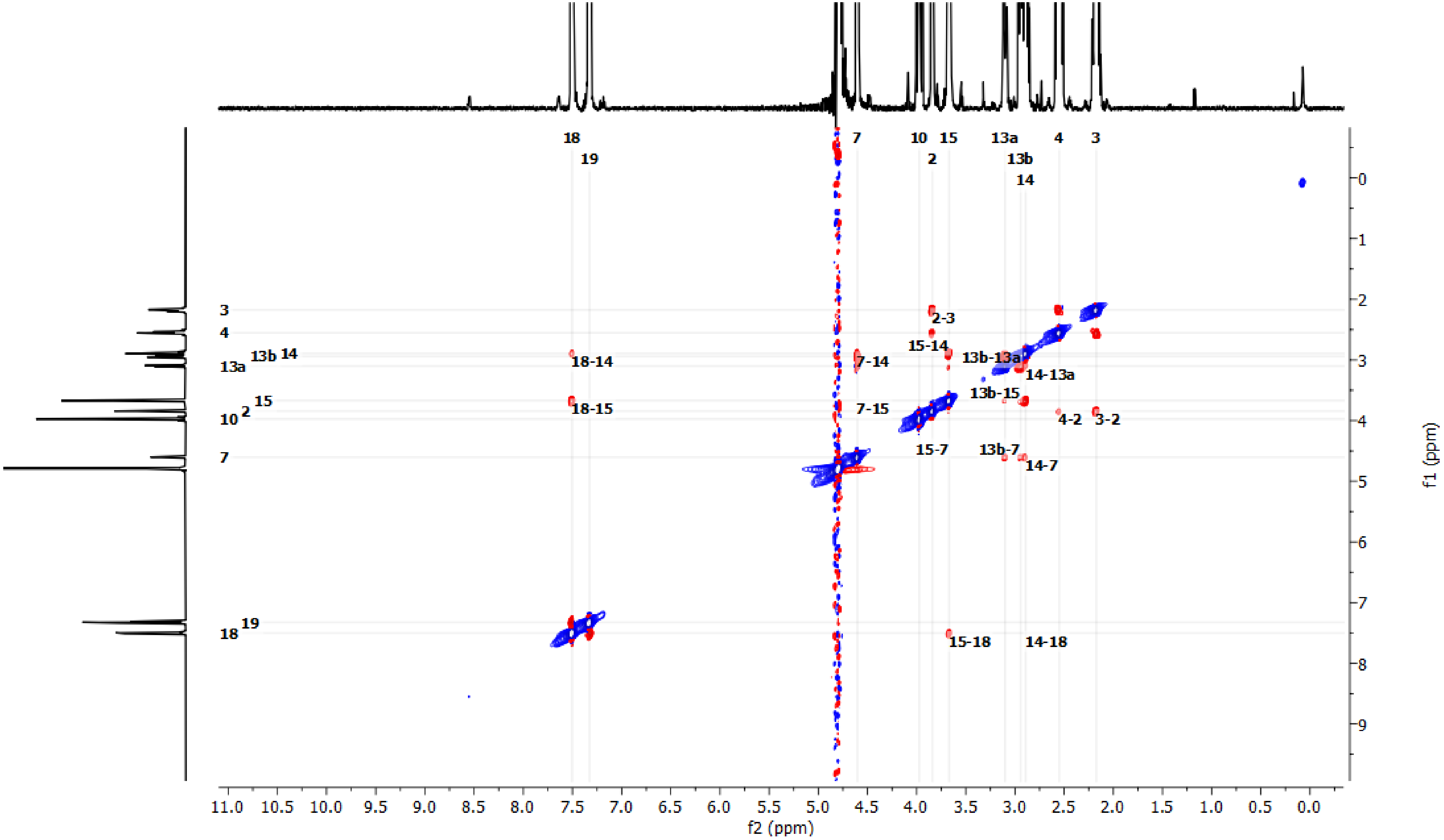

^{15}N-HMBC-NMR of Glutathione–S–C_2H_4–N-(4-fluoroaniline) trifluoroacetate **(S3)**:
D_2O, 298 K

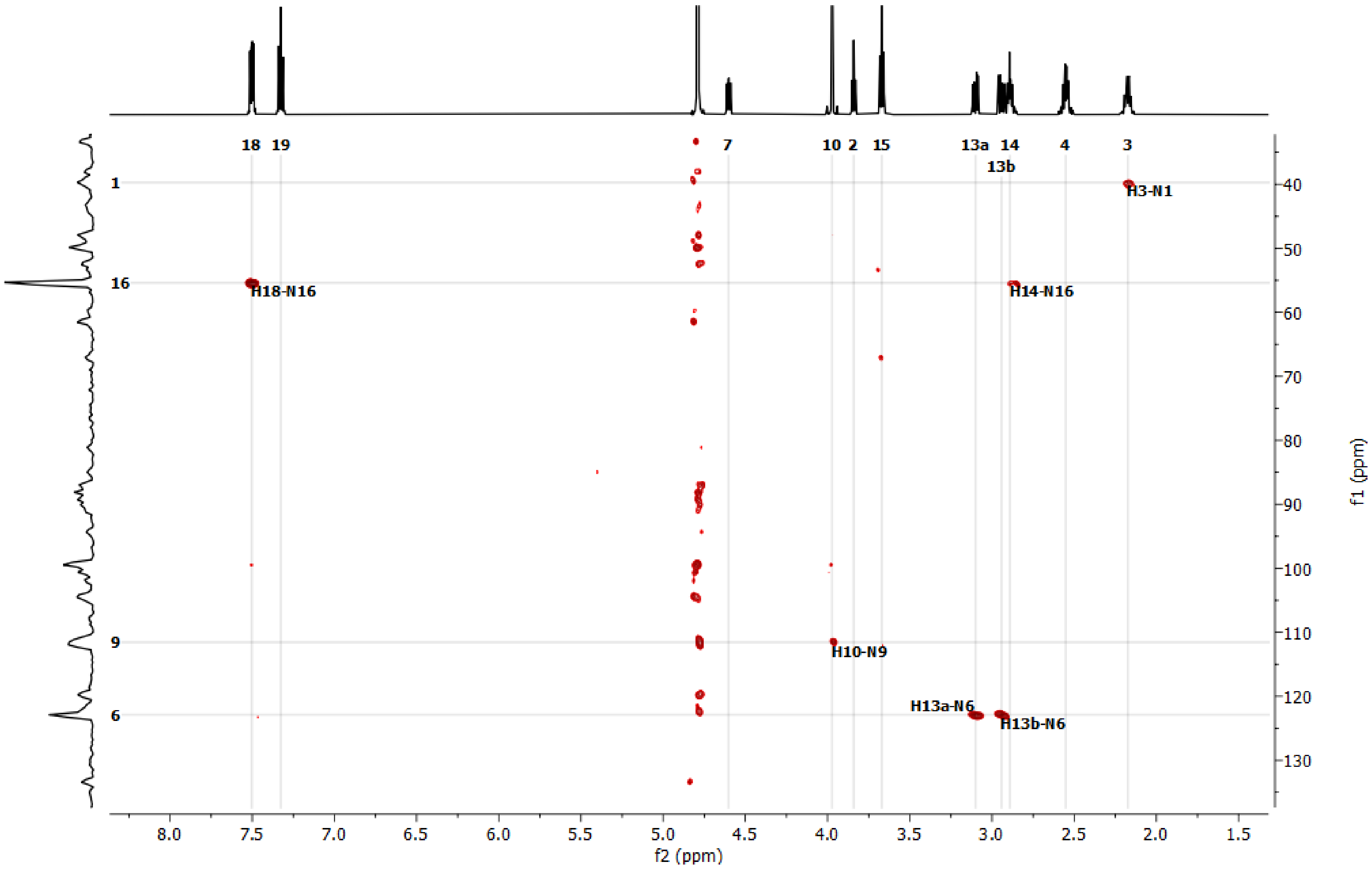

Glutathione–S–C_2H_4–N-(3-Aminophenylboronic acid) trifluoroacetate (S4):

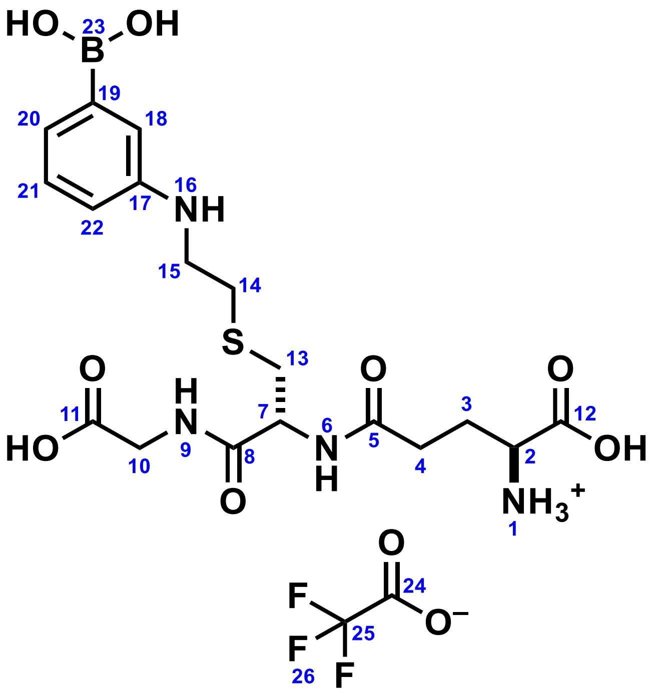

Table S24. Characterization table of Glutathione–S–C_2H_4–N-(3-Aminophenylboronic acid) trifluoroacetate. The ^{15}N chemical shifts were referenced to NH_3(l).

Atom	δ (ppm)	J	HSQC	^{15}N-HMBC	HMQC	COSY	NOESY
**N1**	39,85			3			
**H1**	n.f.						
**C2**	53,73		2		3, 4		
**H2**	3,831	t 6.4(3)	2		3, 4, 12	3	3, 4
**C3**	26,00		3		2, 4		
**H3**	2,164	m	3	1	2, 4, 5, 12	2, 4	2
**C4**	31,26		4		2, 3		
**H4**	2,538	m	4		2, 3, 5	3	2, 6
**C5**	174,83				3, 4, 7		
**N6**	123,00			13a, 13b			
**H6**	8,528	(weak/exchange, d 7.7(7))				7	4, 7

**C7**	52,98		7		13a, 13b		
**H7**	4,588	d 8.2(13b), d 5.3(13a)	7		5, 8, 13	6, 13a, 13b	6, 13a, 13b, 14, 15
**C8**	172,47				7, 10, 13a, 13b		
**N9**	111,90			10			
**H9**	8,519	(weak/exchange, t 5.9(10))				10	10
**C10**	41,55		10				
**H10**	3,953	m	10	9	8, 11	9	9
**C11**	173,53				10		
**C12**	173,51				2, 3		
**C13**	32,62		13a, 13b		7, 14		
**H13a**	3,092	d 5.3(7), d 14.1(13b)	13	6	7, 8, 14	7, 13b	7, 13b, 14, 15
**H13b**	2,934	d 8.2(7), d 14.1(13a)	13	6	7, 8, 14	7, 13a	7, 13a, 15
**C14**	27,11		14		13a, 13b, 15		
**H14**	2,884	m	14	16	13, 15	15	7, 13a, 15, 18, 22
**C15**	49,66		15		14		
**H15**	3,680	t 6.8(14)	15		14, 17	14	7, 13a, 13b, 14, 18, 22
**N16**	57,00			14, 18, 22			
**H16**	n.f.						
**C17**	134,72				15, 21		
**C18**	126,89		18		20, 22		
**H18**	7,766	d 2.3(22), m	18	16	20, 22	22	14, 15
**C19**	135,38				20, 21		
**C20**	134,30		20		18, 22		
**H20**	7,854	d 7.4(21), m	20		18, 19, 22	21	
**C21**	130,03		21				
**H21**	7,607	d 7.4(20), d 8.0(22), m	21		17, 19	20, 22	
**C22**	124,31		22		18, 20		
**H22**	7,542	d 2.3(18), d 8.0(21), m	22	16	18, 20	18, 21	14, 15
**B23**	28,80						
**C24**	163,06	q 35.5(26)					
**C25**	116,41	q 291.7(26)					
**F26**	-75,50	s					

^{1}H NMR of Glutathione–S–C_2H_4–N-(3-Aminophenylboronic acid) trifluoroacetate **(S4)**:
600 MHz, D_2O, 298 K

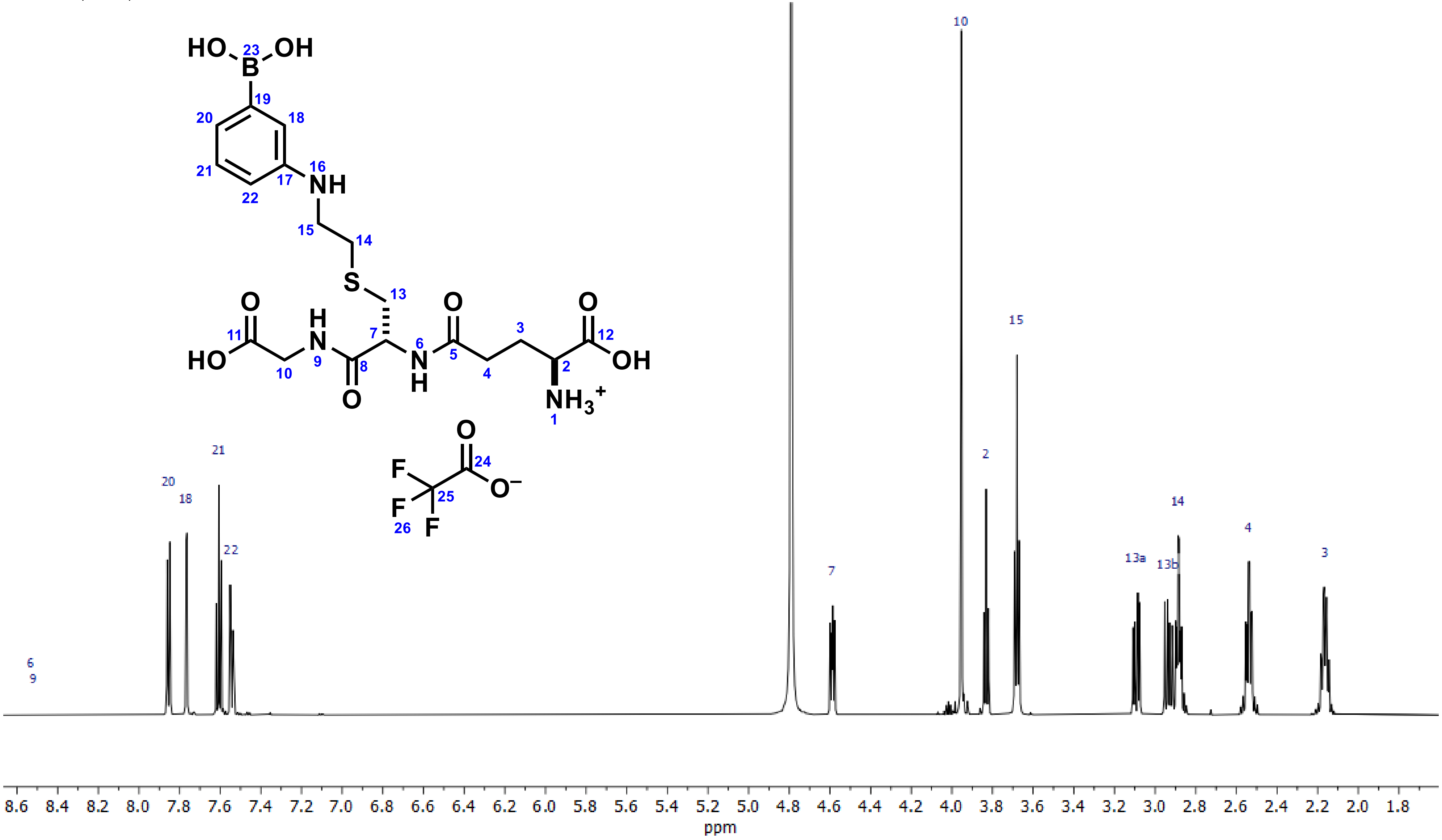

^{13}C NMR of Glutathione–S–C_2H_4–N-(3-Aminophenylboronic acid) trifluoroacetate **(S4)**:
151 MHz, D_2O, 298 K

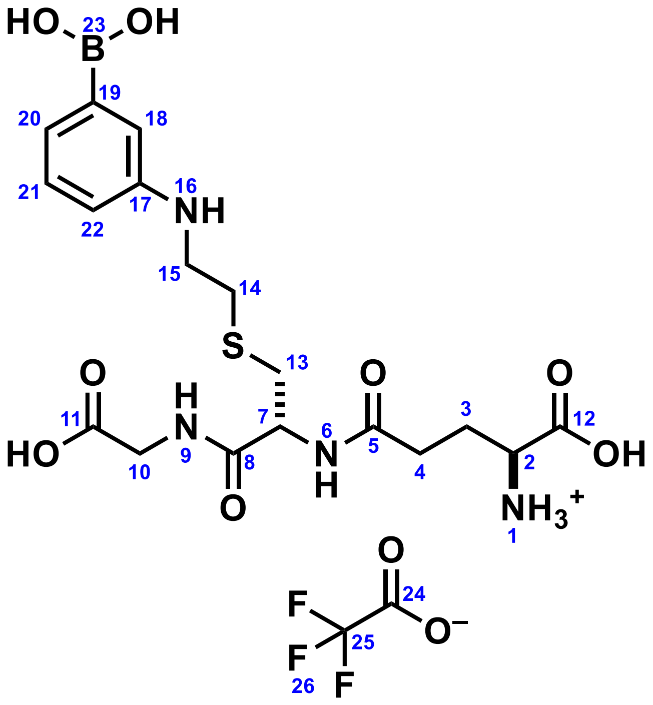

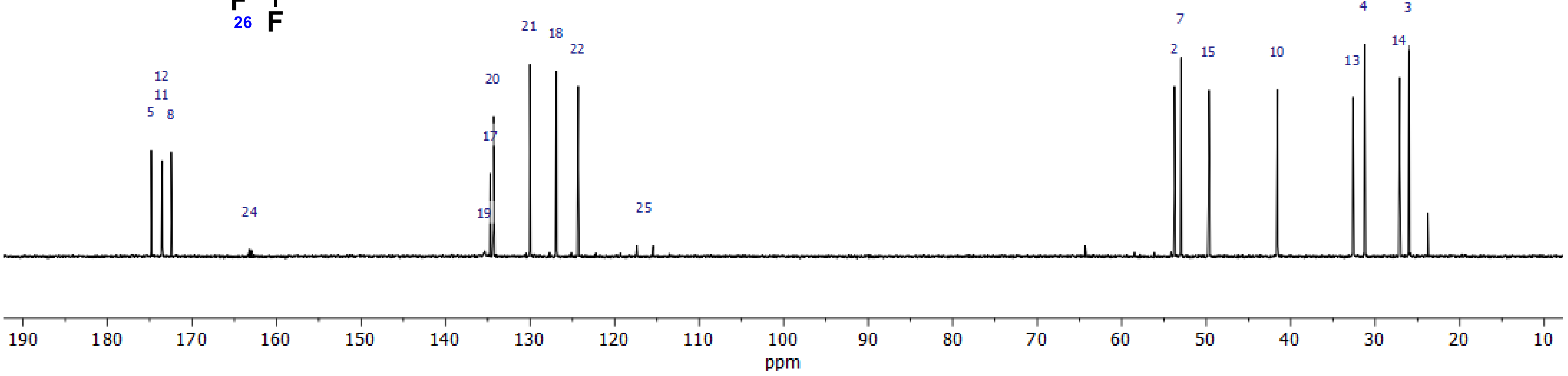

^{11}B NMR of Glutathione–S–C_2H_4–N-(3-Aminophenylboronic acid) trifluoroacetate **(S4)**:
160 MHz, D_2O, 298 K

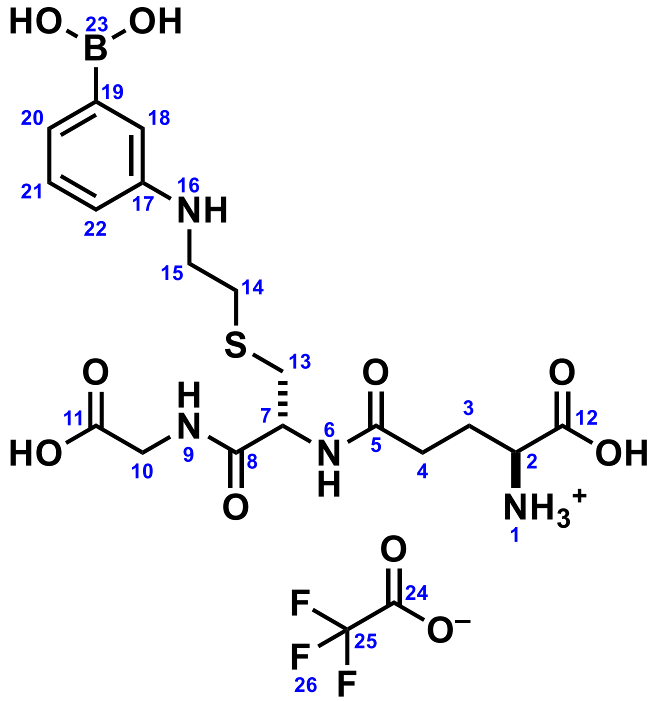

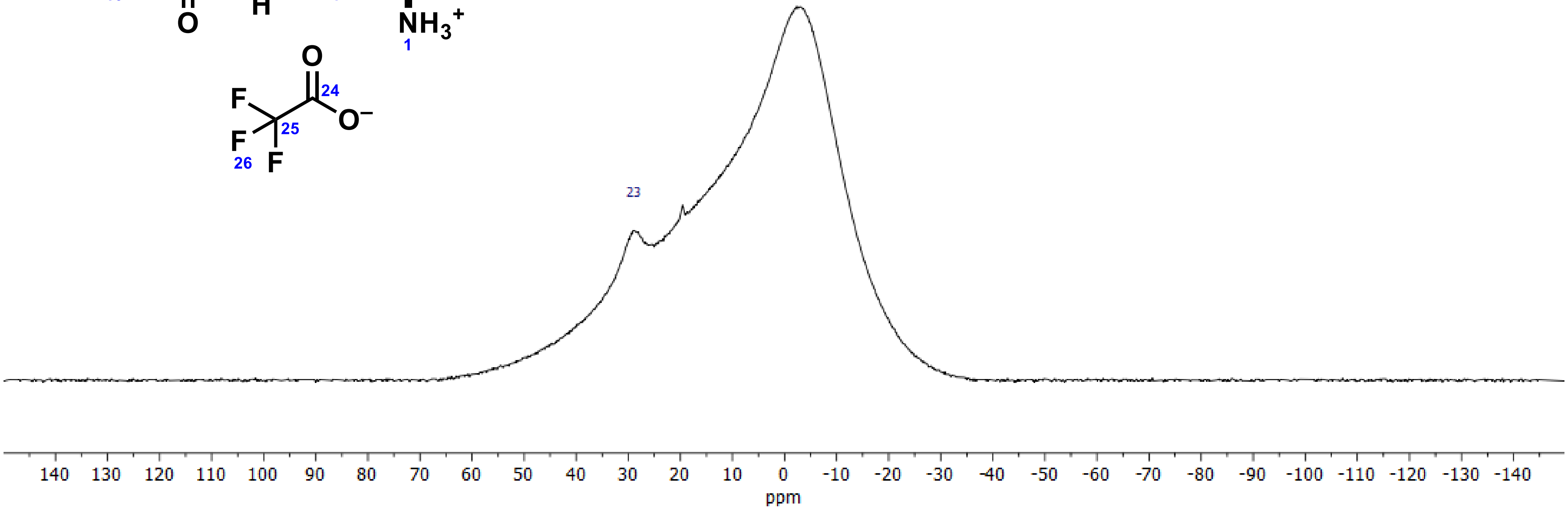

^{19}F NMR of Glutathione–S–C_2H_4–N-(3-Aminophenylboronic acid) trifluoroacetate **(S4)**:
470 MHz, D_2O, 298 K

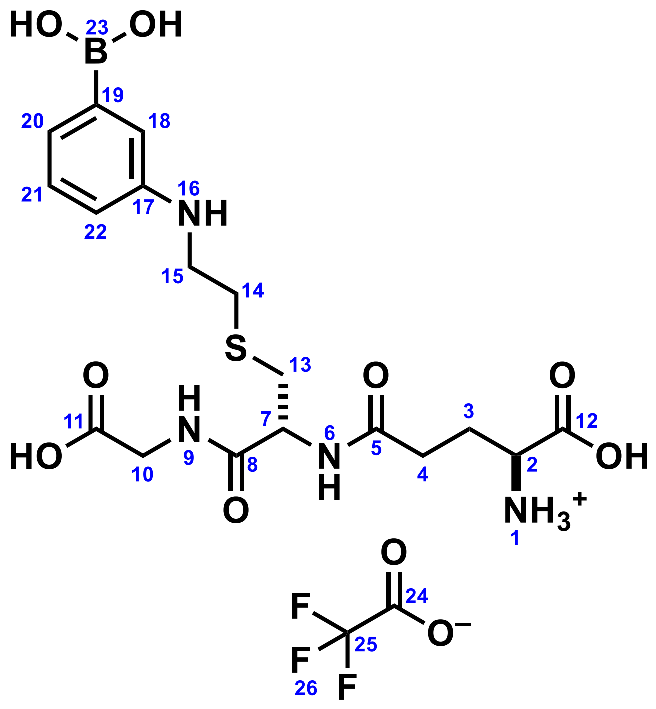

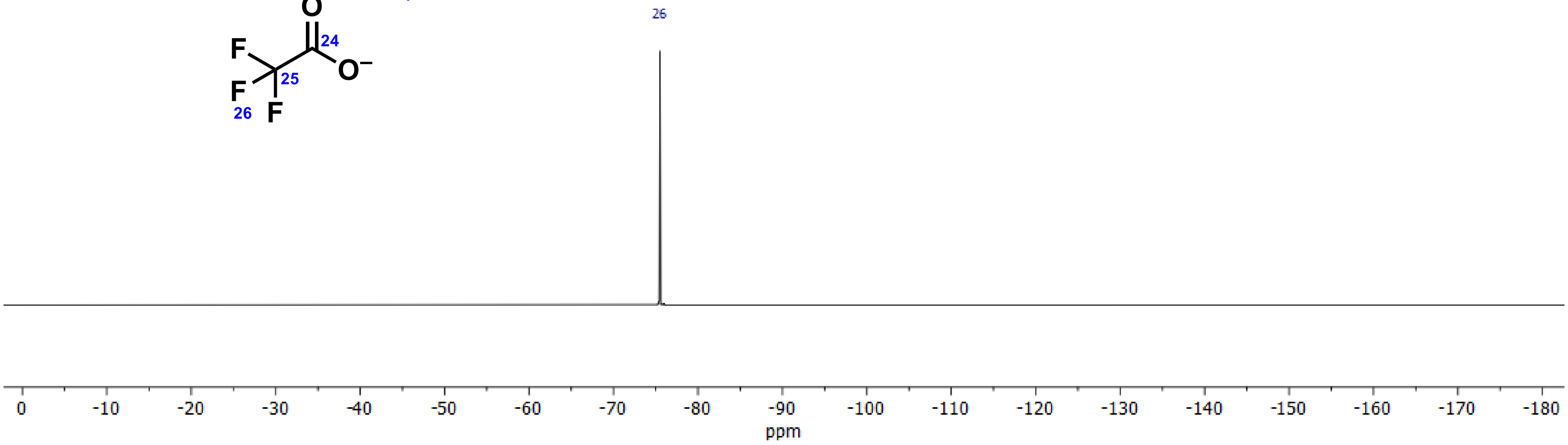

HSQC-NMR of Glutathione–S–C_2H_4–N-(3-Aminophenylboronic acid) trifluoroacetate **(S4)**:
D_2O, 298 K

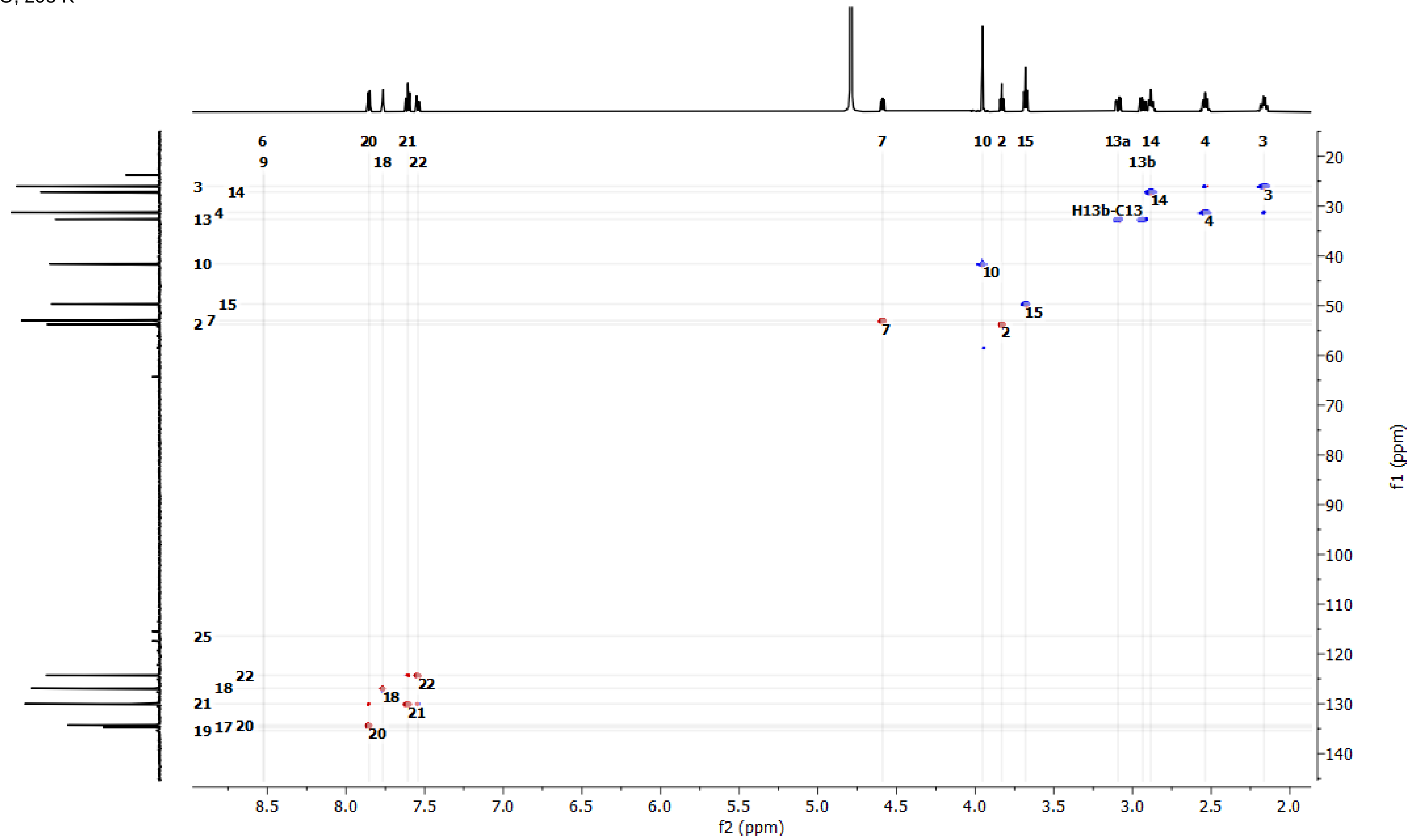

HMQC-NMR of Glutathione–S–C_2H_4–N-(3-Aminophenylboronic acid) trifluoroacetate **(S4)**:
D_2O, 298 K

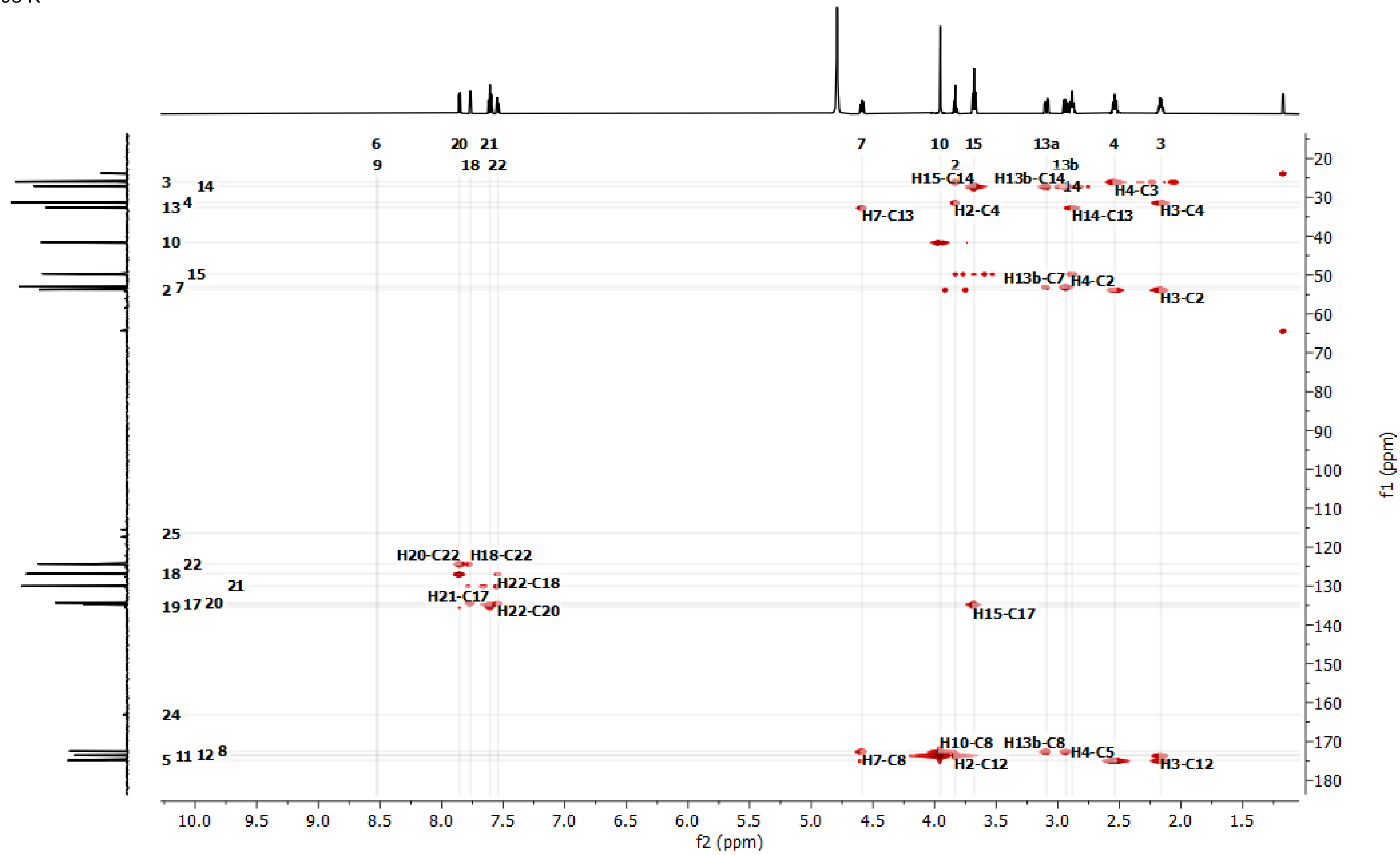

COSY-NMR of Glutathione–S–C_2H_4–N-(3-Aminophenylboronic acid) trifluoroacetate **(S4)**:
D_2O, 298 K

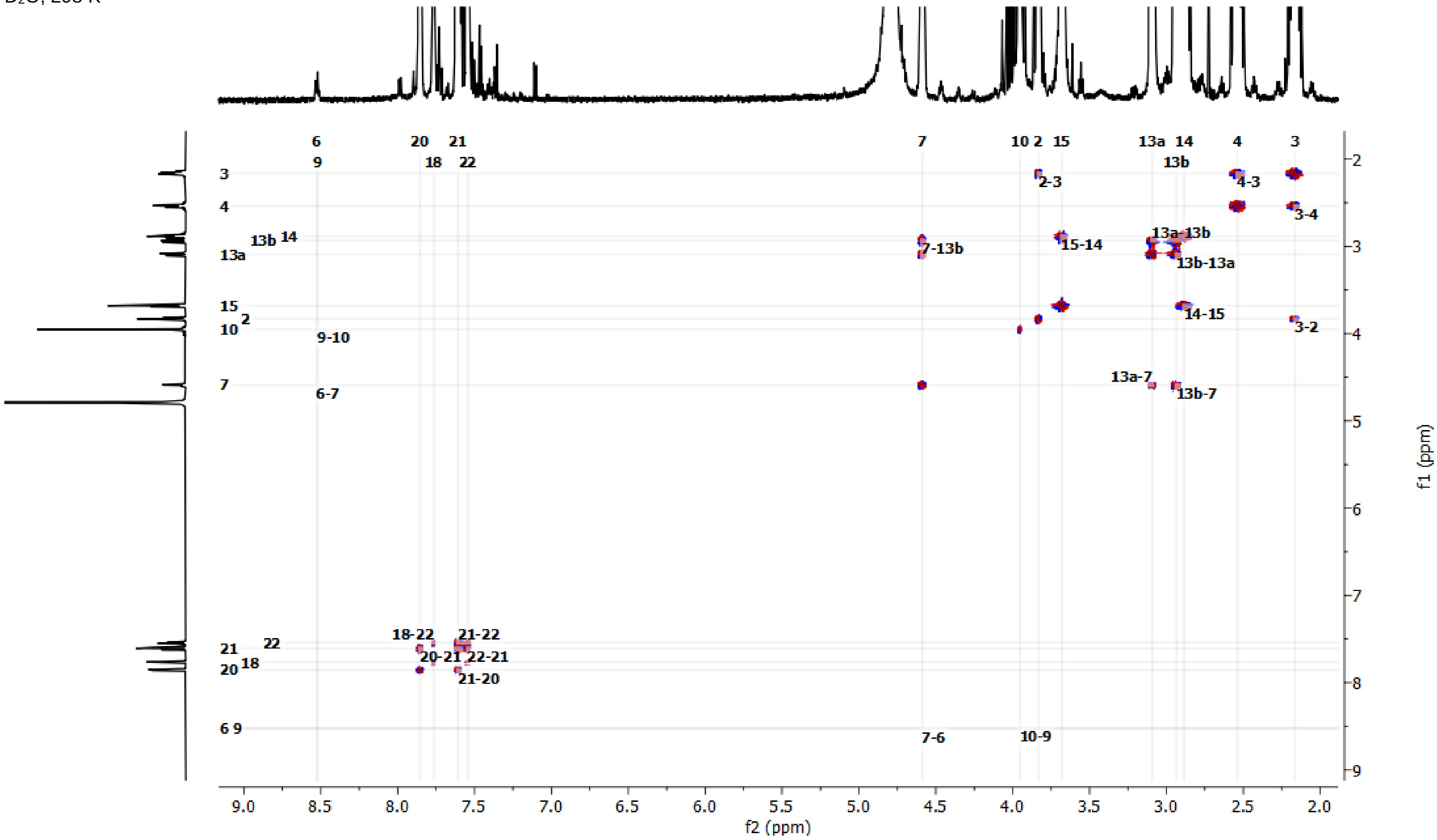

NOESY-NMR of Glutathione–S–C_2H_4–N-(3-Aminophenylboronic acid) trifluoroacetate **(S4)**:
D_2O, 298 K

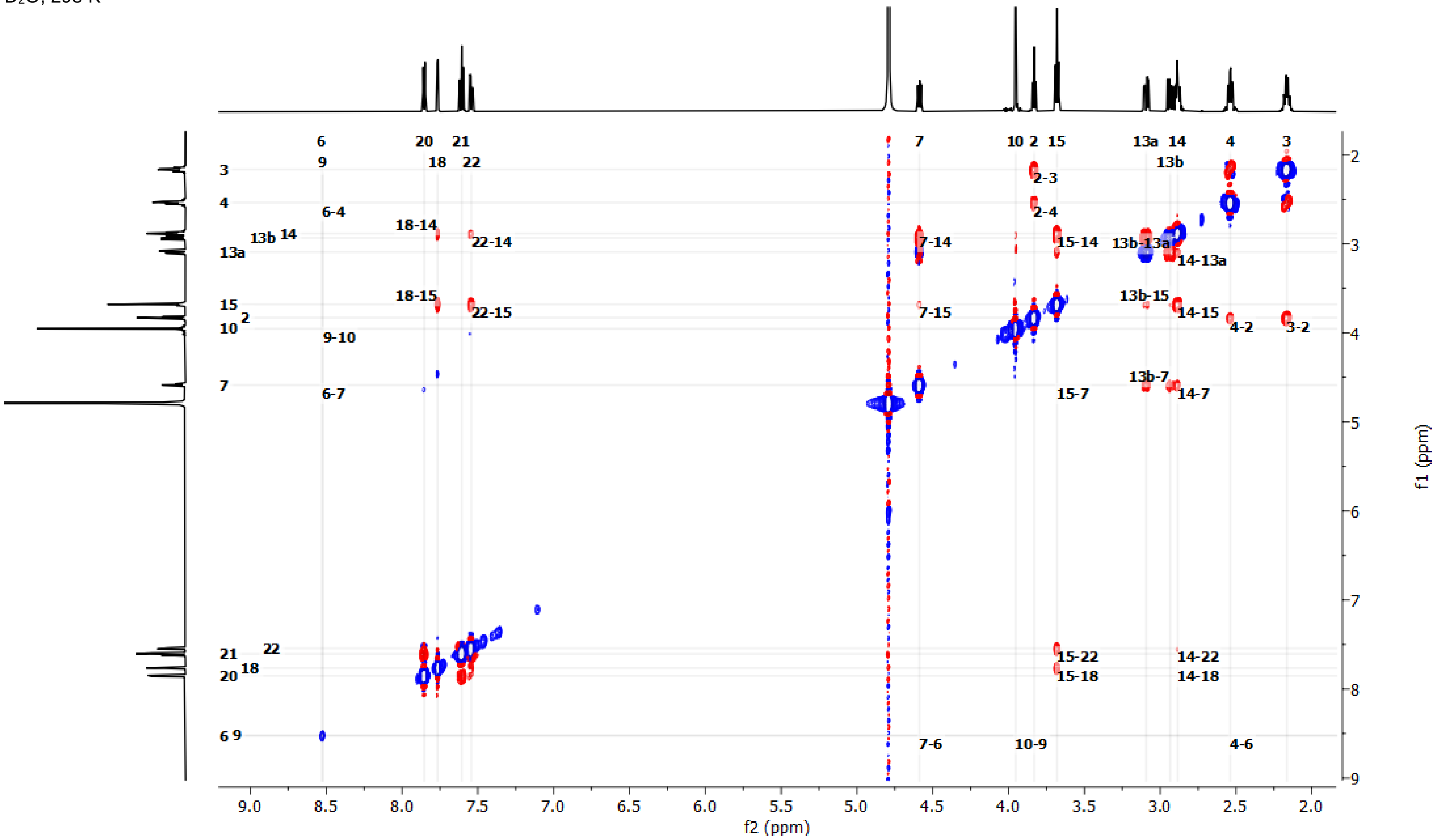

^{15}N-HMBC-NMR of Glutathione–S–C_2H_4–N-(3-Aminophenylboronic acid) trifluoroacetate **(S4)**:
D_2O, 298 K

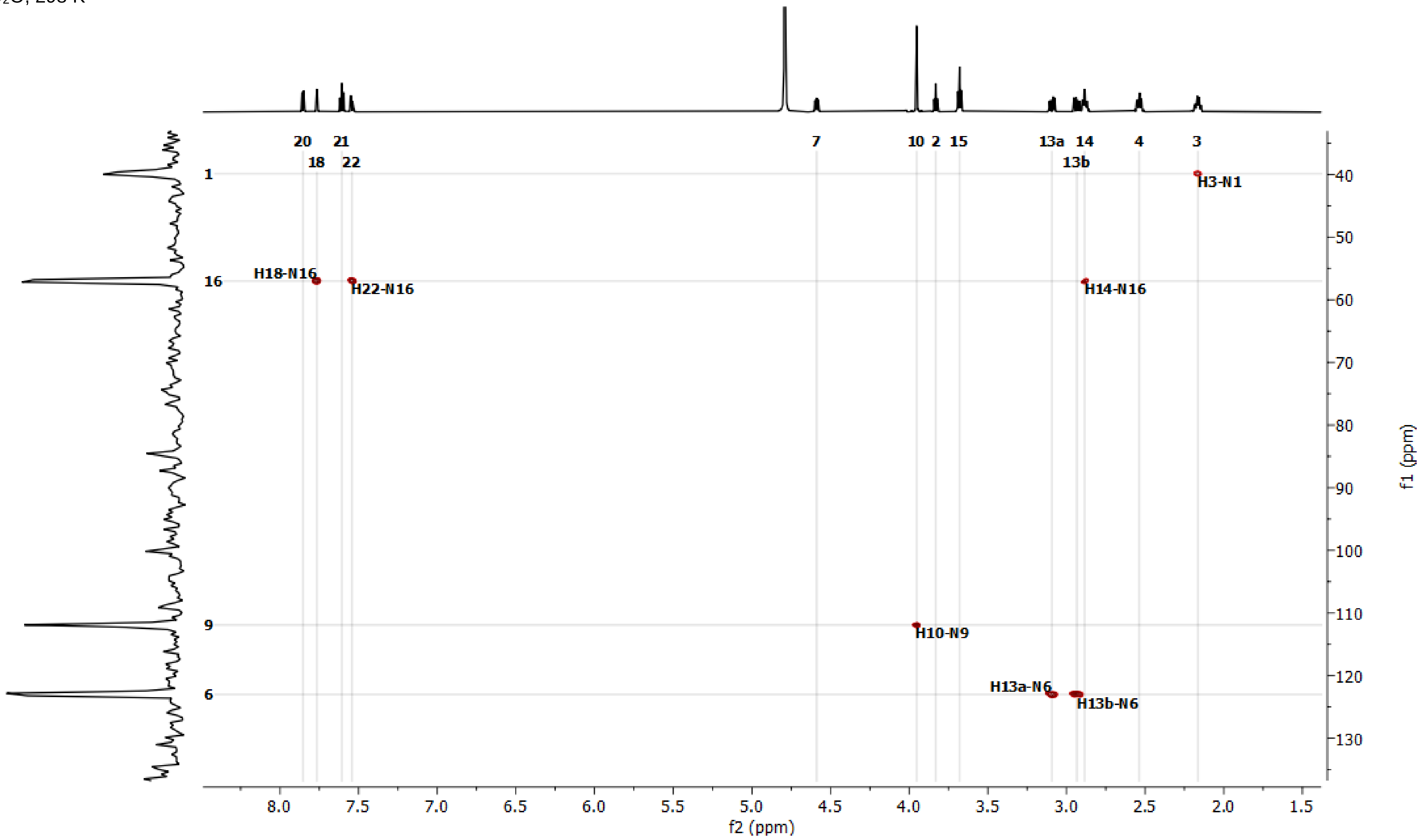

NMR-Characterization of cysteine derivatives

Cysteine–S–C_2H_4–thioglucose (S5)

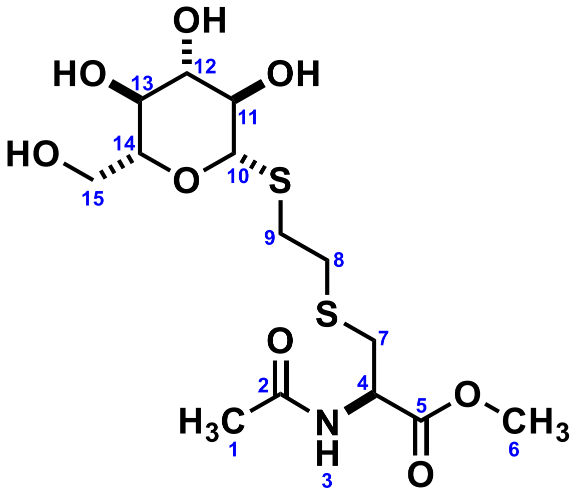

Table S25. Characterization table of Cysteine–S–C_2H_4–thioglucose.

Atom	δ (ppm)	J	HSQC	HMBC	COSY	NOESY
**C1**	21.71		1			
**H1**	2.081	s	1	2		3, 4, 7b, 10
**C2**	174.35			1, 4		
**N3**	n.d.					
**H3**	8.548	d 7.6(4), residual signal			4	1, 4, 7b
**C4**	52.63		4	7a, 7b		
**H4**	4.672	d 8.3(7b), d 4.9(7a)	4	2, 5, 7	3, 7a, 7b	1, 3, 6, 7a, 7b, 8
**C5**	172.75			4, 6, 7a, 7b		
**C6**	53.23		6			
**H6**	3.802	s	6	5		4
**C7**	32.43		7a, 7b	4, 8		
**H7a**	3.151	d 14.0(7b), d 4.9(4)	7	4, 5, 8	4, 7b	4
**H7b**	2.981	d 14.0(7a), d 8.3(4)	7	4, 5, 8	4, 7a	1, 3, 4

**C8**	32.28		8	7a, 7b, 9a, 9b		
**H8**	2.902	m	8	7, 9	9a, 9b	4, 10, 11
**C9**	29.95		9a, 9b	8, 10		
**H9a**	3.039	m	9	8, 10	8, 9b	10, 11
**H9b**	2.940	m	9	8, 10	8, 9a	10, 11
**C10**	85.45		10	9a, 9b, 11		
**H10**	4.587	d 9.9(11)	10	9, 11, 12, 14	11	1, 8, 9a, 9b, 12, 13, 14
**C11**	72.36		11	10, 12		
**H11**	3.335	d 9.9(10), d 9.0(12)	11	10, 12	10, 12	8, 9a, 9b, 13
**C12**	77.25		12	10, 11, 13		
**H12**	3.506	t 9.0(11, 13)	12	11	11, 13	10
**C13**	69.57		13	14, 15a, 15b		
**H13**	3.426	d 9.8(14), d 9.0(12)	13	12, 14, 15	12, 14	10, 11, 15a, 15b
**C14**	79.99		14	10, 13, 15b		
**H14**	3.483	d 9.8(13), d 5.8(15b), d 2.2(15a)	14	13	13, 15a, 15b	10, 15a, 15b
**C15**	60.93		15a, 15b	13		
**H15a**	3.916	d 12.5(15b), d 2.2(14)	15	13	14, 15b	13, 14
**H15b**	3.721	d 12.5(15a), d 5.8(14)	15	13, 14	14, 15a	13, 14

1H NMR of Cysteine–S–C_2H_4–thioglucose **(S5)**:
600 MHz, D_2O, 298 K

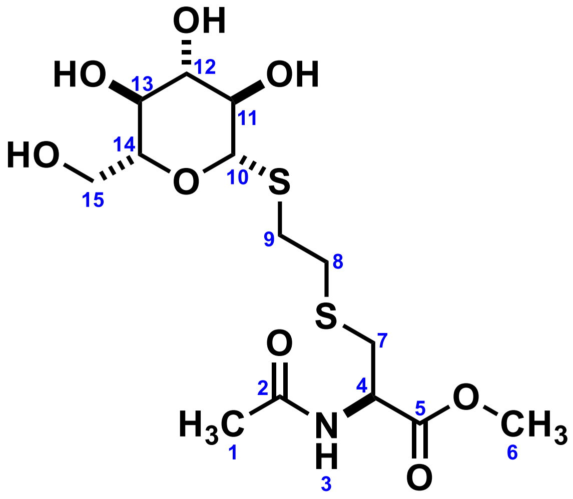

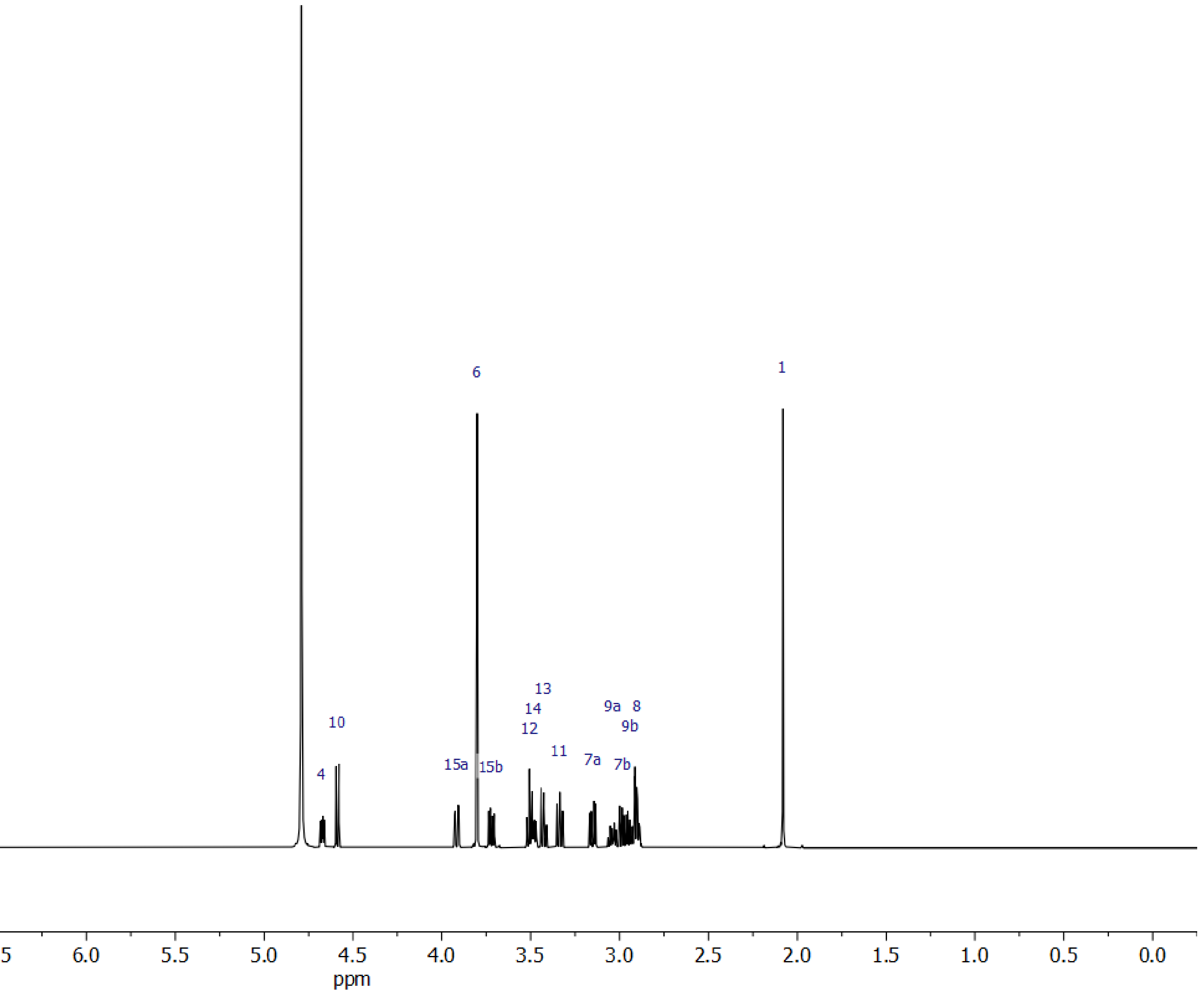

^{13}C NMR of Cysteine–S–C_2H_4–thioglucose **(S5)**:
151 MHz, D_2O, 298 K

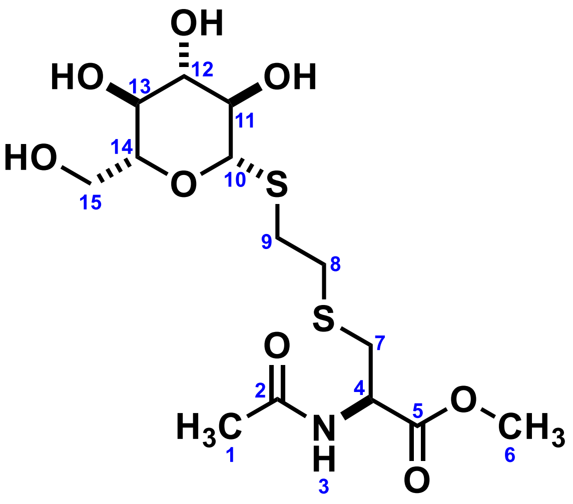

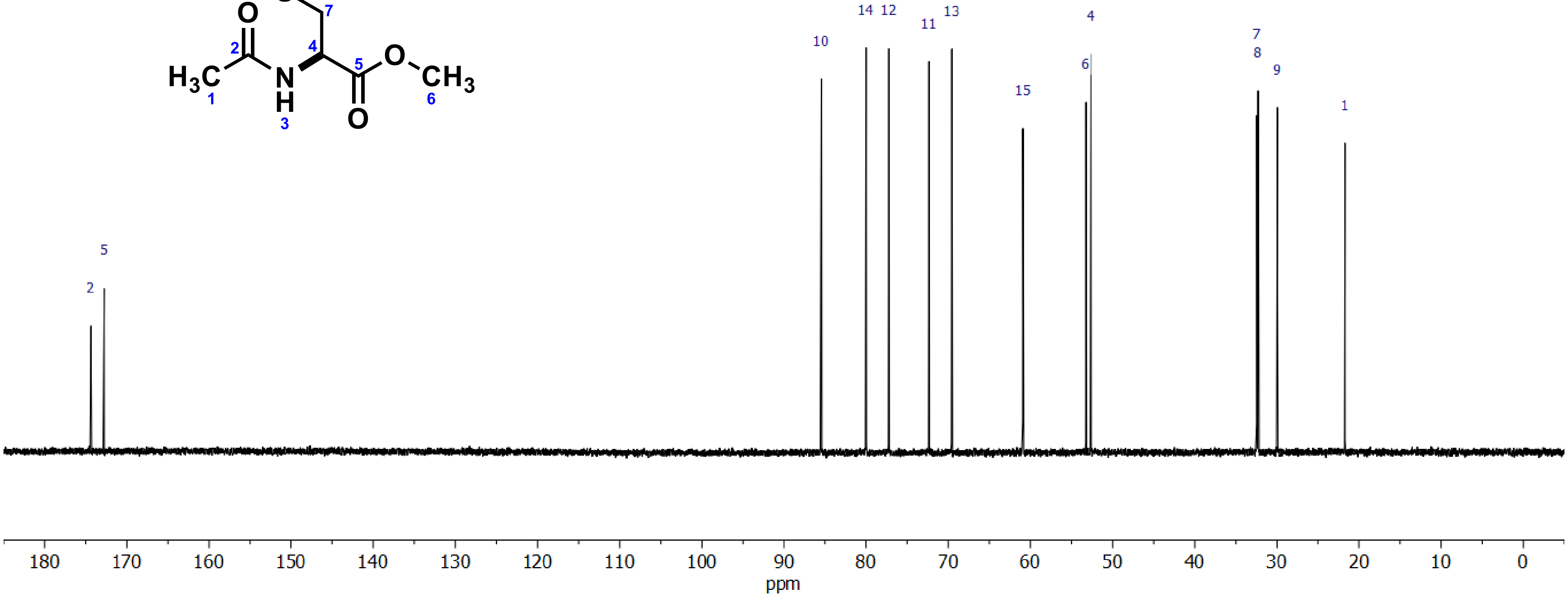

HSQC of Cysteine–S–C_2H_4–thioglucose **(S5)**:
D_2O, 298 K

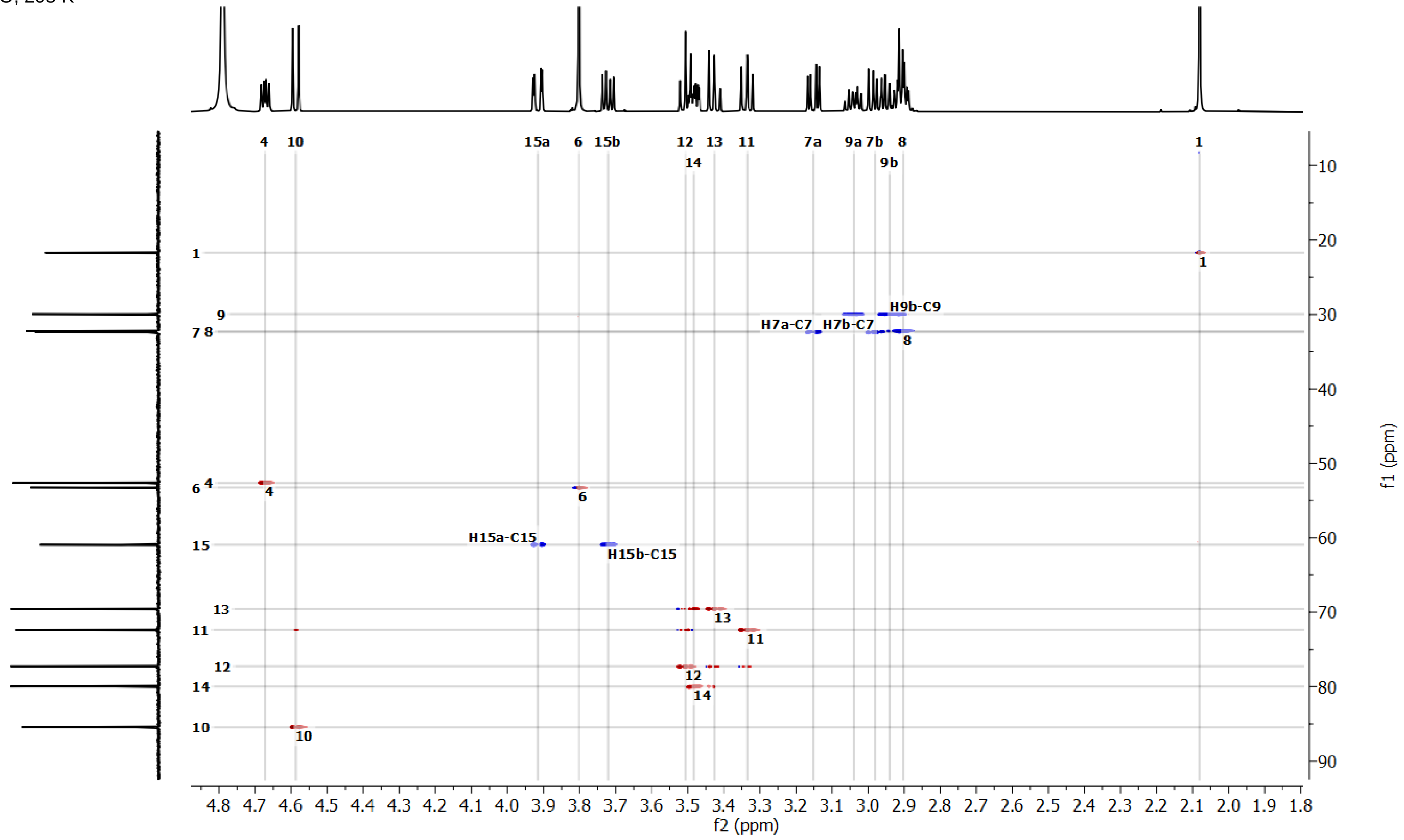

HMQC of Cysteine–S–C_2H_4–thioglucose **(S5)**:
D_2O, 298 K

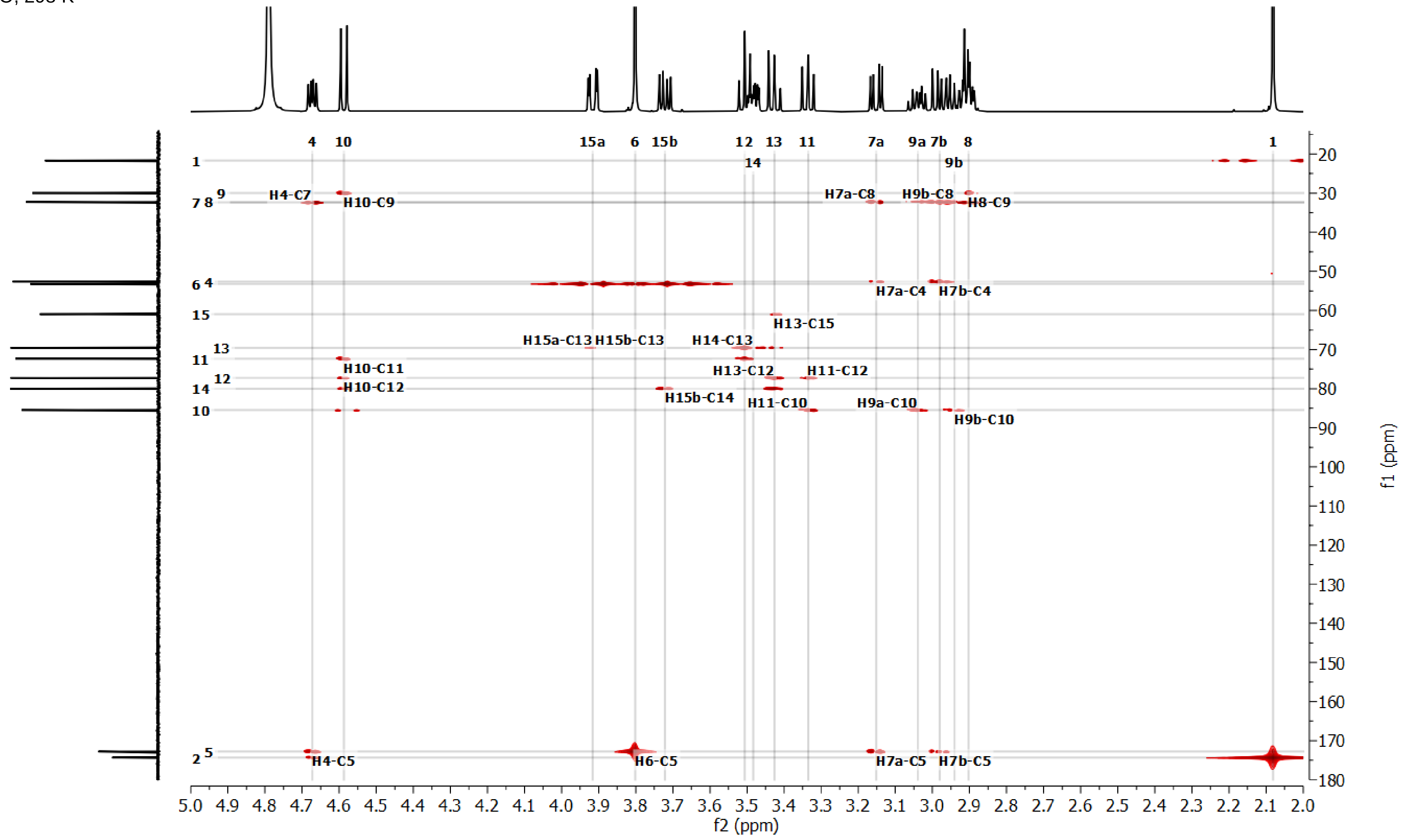

COSY of Cysteine–S–C_2H_4–thioglucose **(S5)**:
D_2O, 298 K

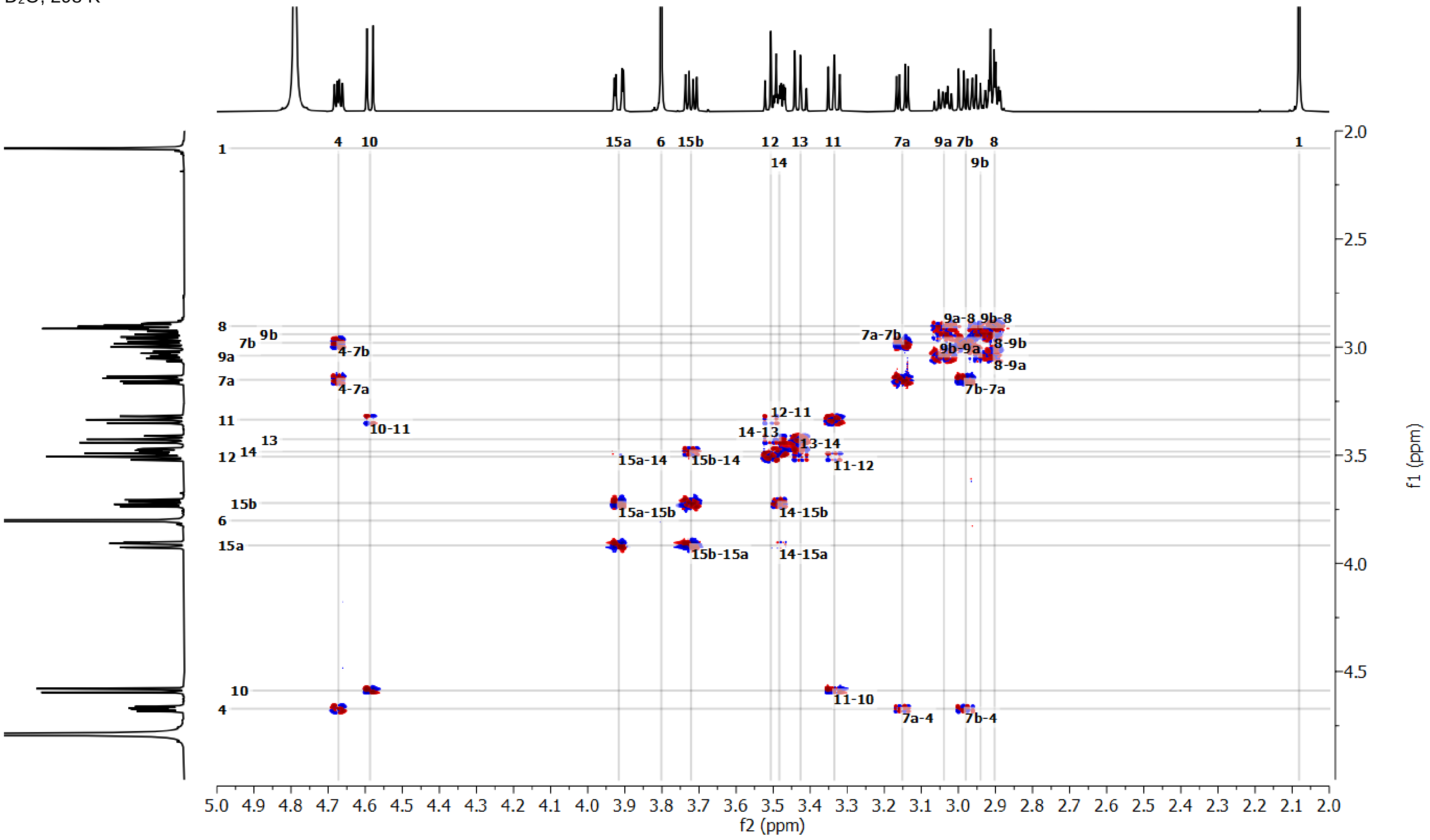

NOESY of Cysteine–S–C_2H_4–thioglucose **(S5)**:
D_2O, 298 K

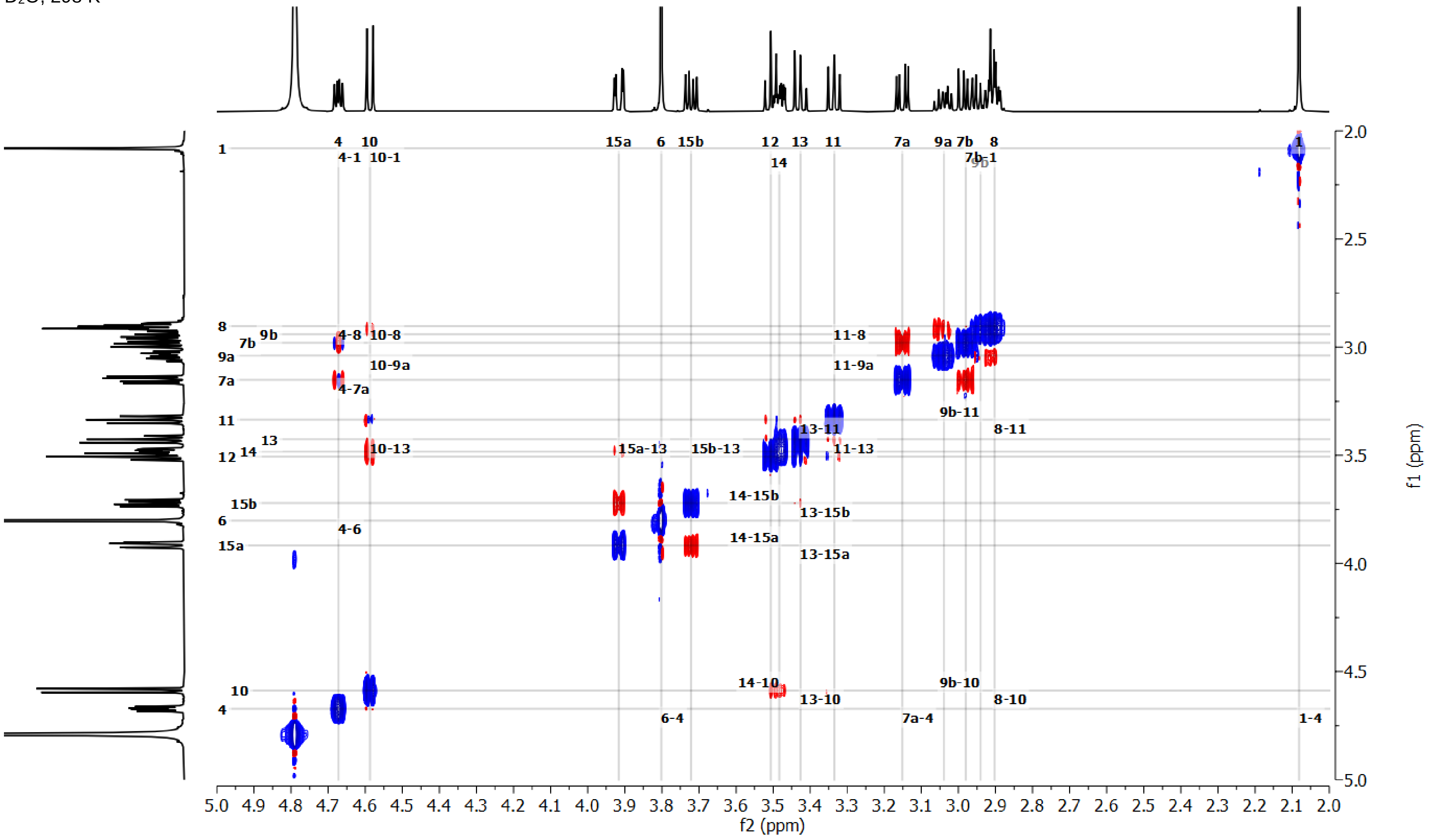

Cysteine–(S–C_2H_4)$_2$–dimethylammonium trifluoroacetate (S6)

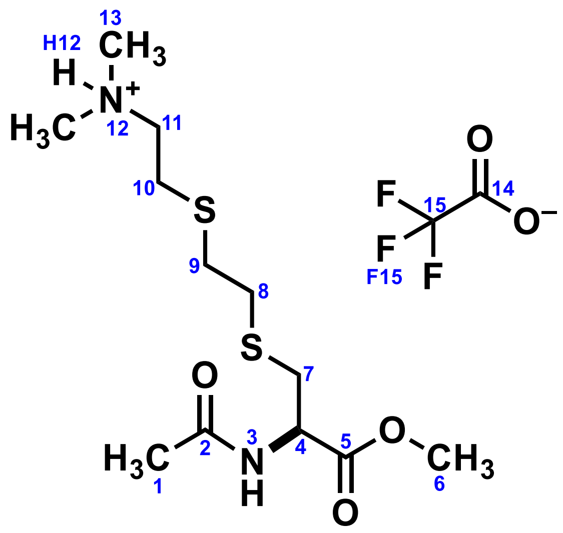

Table S26. Characterization table of Cysteine–(S–C_2H_4)$_2$–dimethylammonium trifluoroacetate. The ^{15}N chemical shifts were referenced to NH_3(l).

Atom	δ (ppm)	J	HSQC	^{15}N-HSQC	HMBC	COSY	NOESY
C1	22.10		1				
H1	1.965	s	1		2		3, 4, 7a, 11
C2	170.11				1, 3, 4		
N3	119.62			3			
H3	8.462	d 8.0(4)		3	2, 4, 7	4	1, 4, 7a, 7b
C4	52.90		4		3, 7a, 7b		
H4	4.608	t 8.0(3, 7b), d 5.7(7a)	4		2, 5, 7	3, 7a, 7b	1, 3, 7a, 7b, 8
C5	171.67				4, 6, 7a, 7b		
C6	52.04		6				
H6	3.705	s	6		5		
C7	33.22		7a, 7b		3, 4, 8		
H7a	3.042	d 13.7(7b), d 5.7(4)	7		4, 5, 8	4, 7b	1, 3, 4
H7b	2.904	d 13.7(7a), d 7.9(4)	7		4, 5, 8	4, 7a	3, 4
C8	32.19		8		7a, 7b, 9		

**H8**	2.840	m (o.l.)	8		7, 9		4
**C9**	31.44		9		8, 10		
**H9**	2.843	m (o.l.)	9		8, 10		
**C10**	25.35		10		9, 11		
**H10**	3.044	m (o.l.)	10		9	11	
**C11**	56.80		11		13		
**H11**	3.468	m	11		10, 13	10	1, 13
**N12**	n.f.						
**H12**	n.f.						
**C13**	42.39		13		11		
**H13**	3.000	s	13		11		11
**C14**	160.15	q 32(F15)					
**C15**	117.74	q 297(F15)					
**F15**	-74.9						

^{1}H NMR of Cysteine–(S–C_2H_4)$_2$–dimethylammonium trifluoroacetate **(S6)**:
600 MHz, DMF-d_7, 298 K

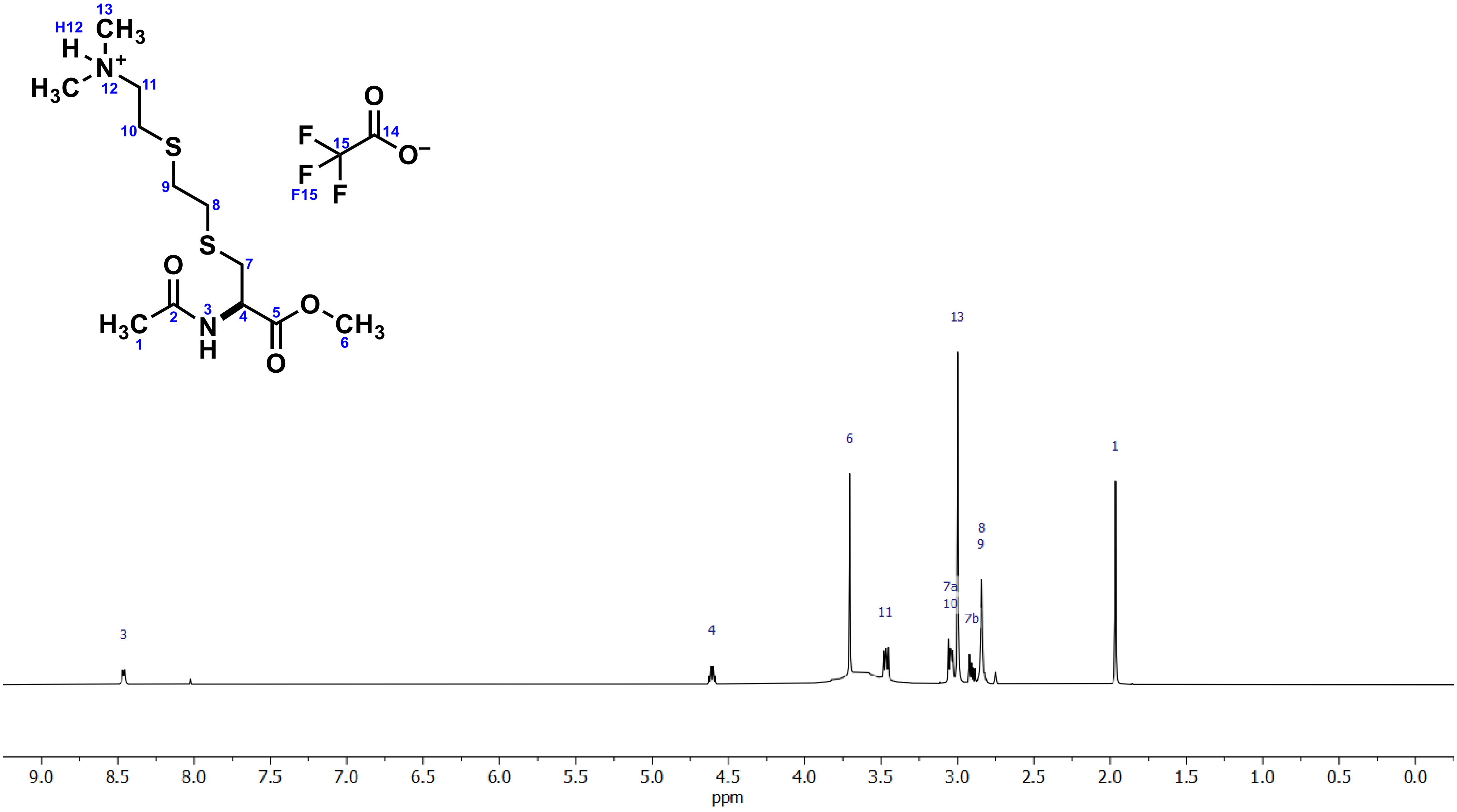

^{13}C NMR of Cysteine–(S–C_2H_4)$_2$–dimethylammonium trifluoroacetate **(S6)**:
151 MHz, DMF-d_7, 298 K

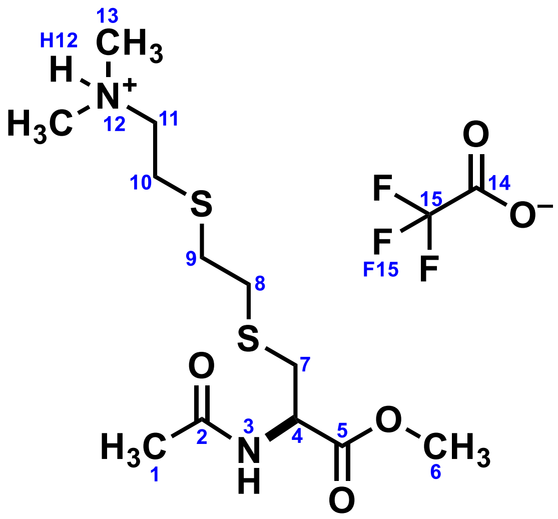

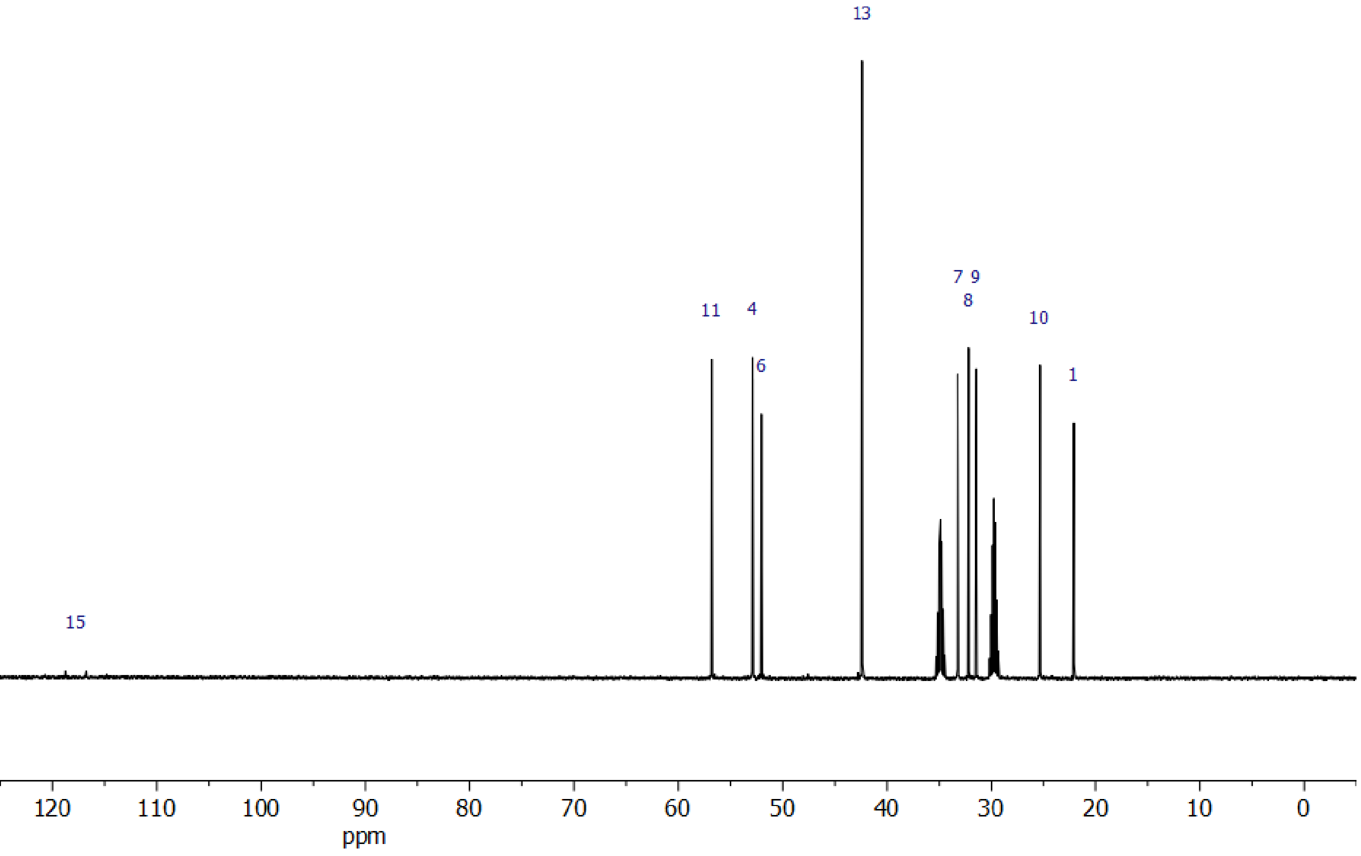

^{19}F NMR of Cysteine–(S–C_2H_4)$_2$–dimethylammonium trifluoroacetate **(S6)**:
471 MHz, DMF-d_7, 298 K

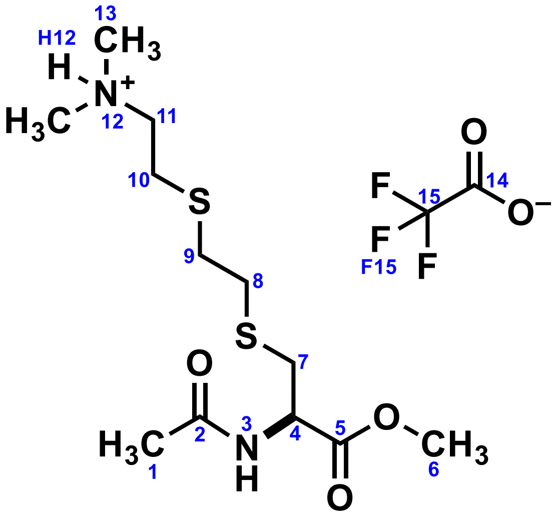

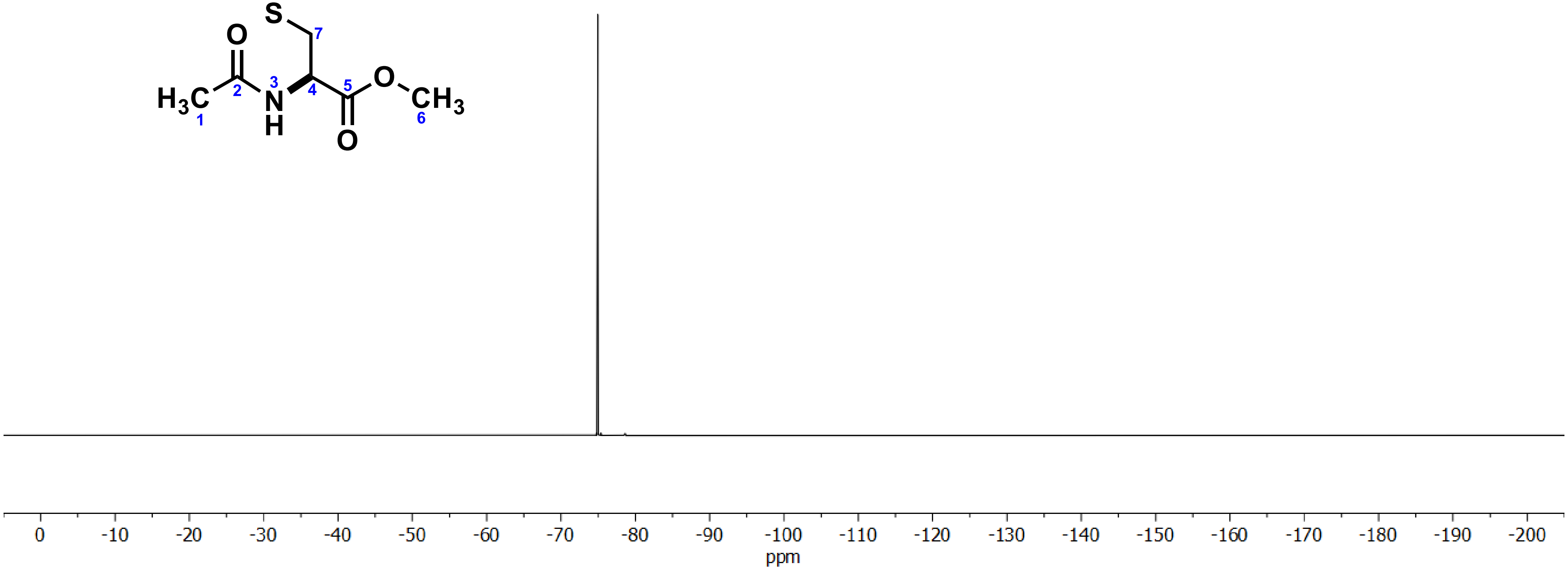

HSQC-NMR of Cysteine–(S–C_2H_4)$_2$–dimethylammonium trifluoroacetate **(S6)**:
DMF-d_7, 298 K

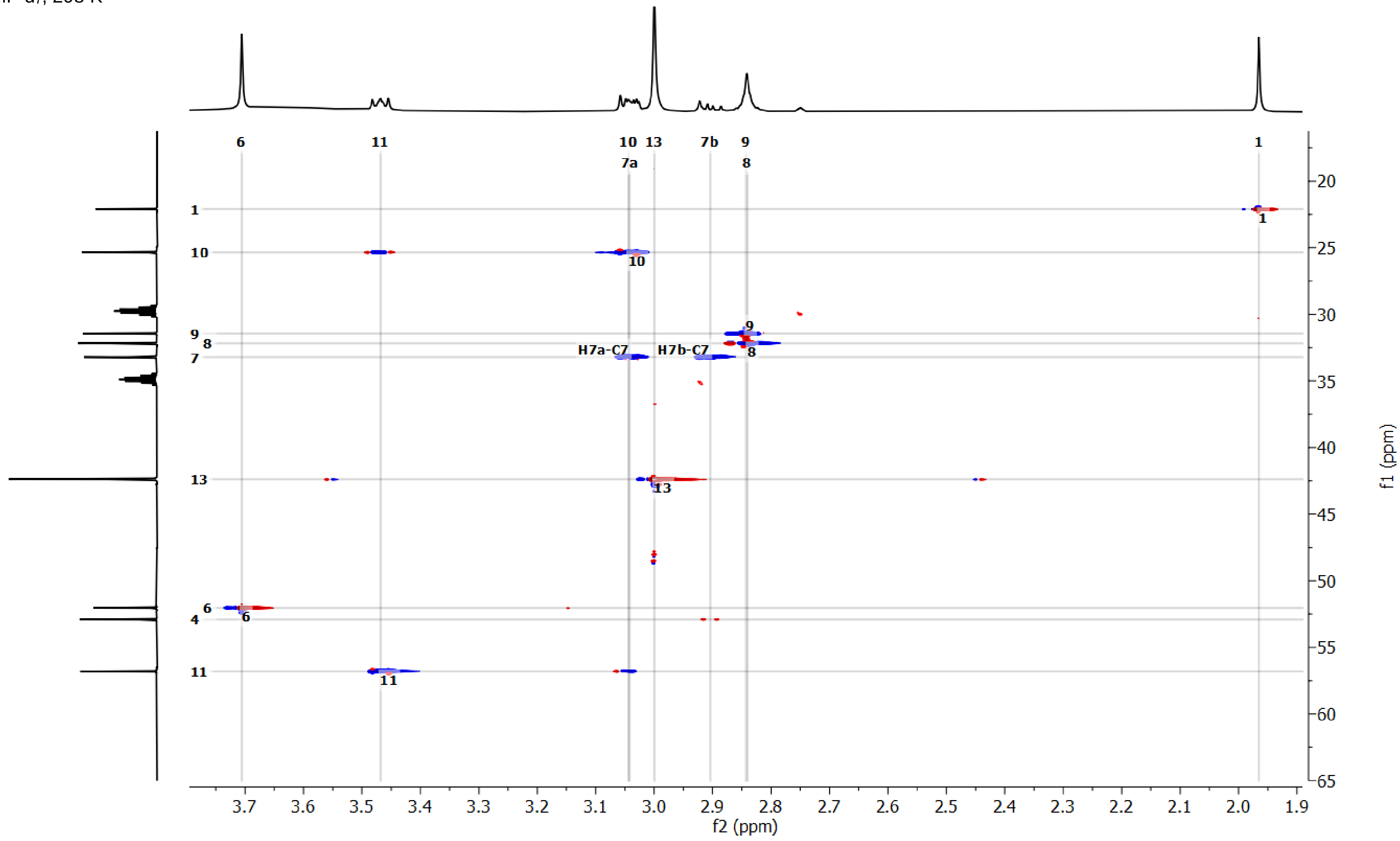

HMQC-NMR of Cysteine–(S–C_2H_4)$_2$–dimethylammonium trifluoroacetate **(S6)**:
DMF-d_7, 298 K

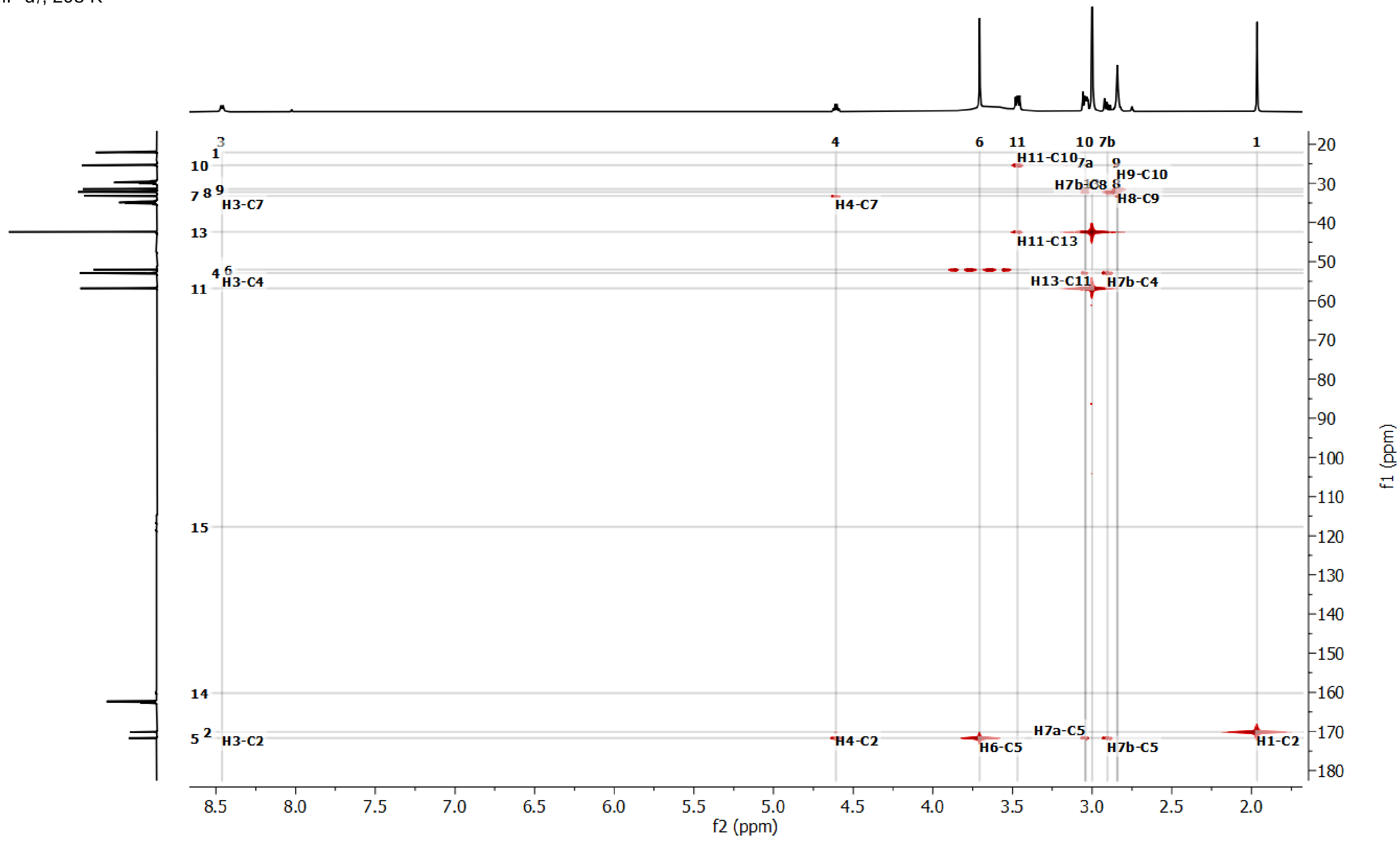

COSY-NMR of Cysteine–(S–C_2H_4)$_2$–dimethylammonium trifluoroacetate **(S6)**:
DMF-d_7, 298 K

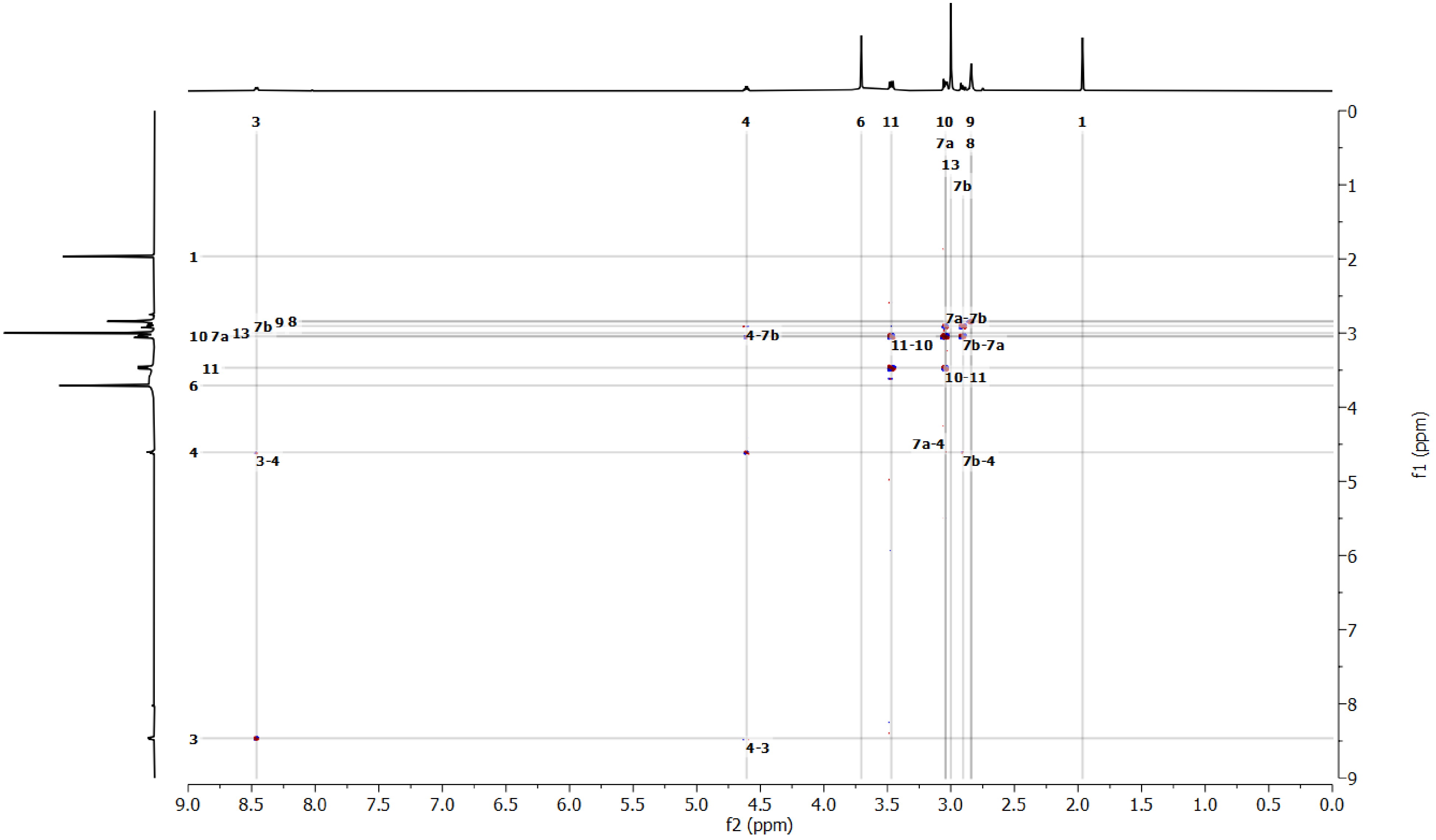

NOESY-NMR of Cysteine–(S–C_2H_4)$_2$–dimethylammonium trifluoroacetate **(S6)**:
DMF-d_7, 298 K

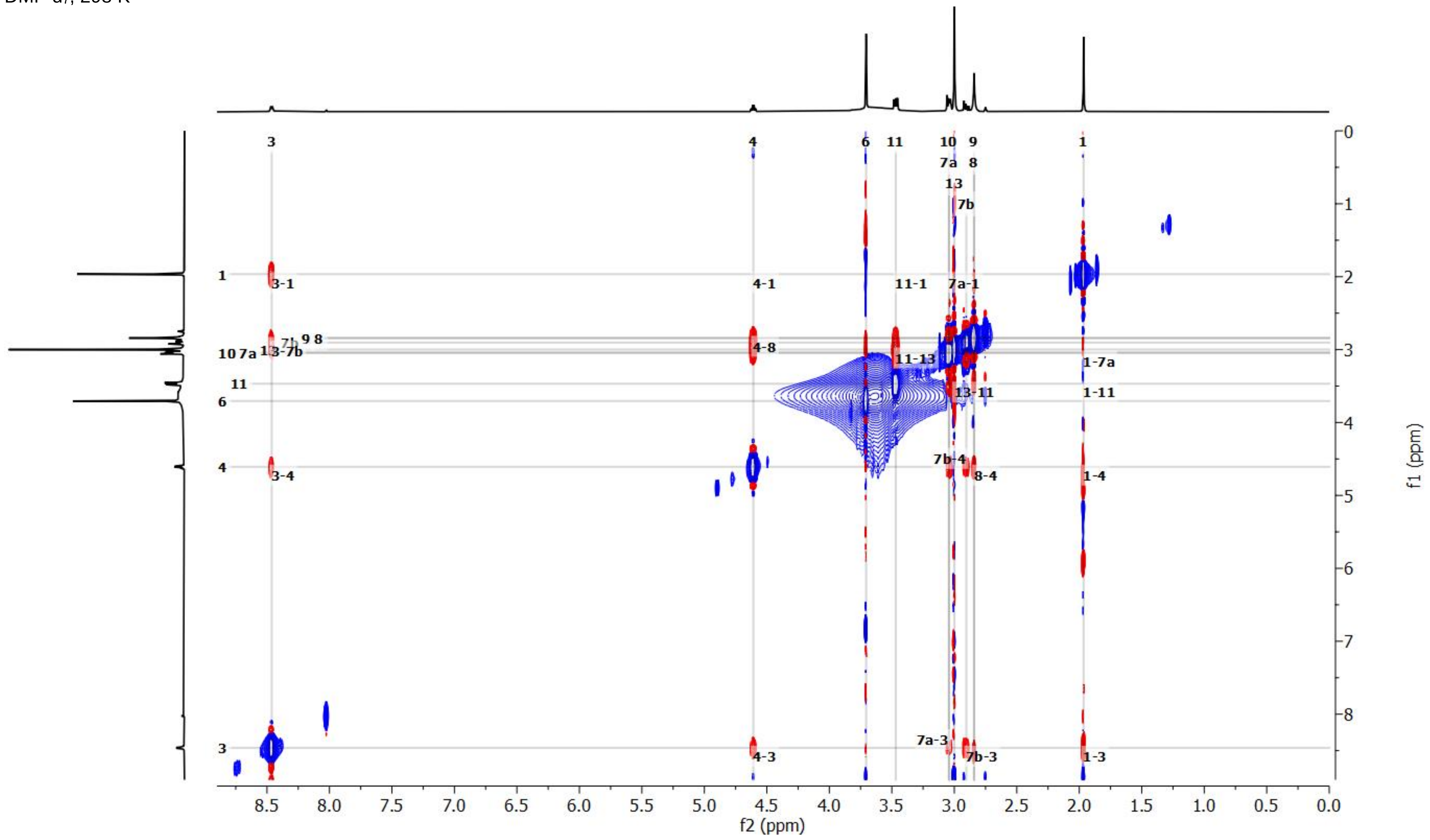

^{15}N-HSQC-NMR of Cysteine–(S–C_2H_4)$_2$–dimethylammonium trifluoroacetate **(S6)**:
DMF-d_7, 298 K

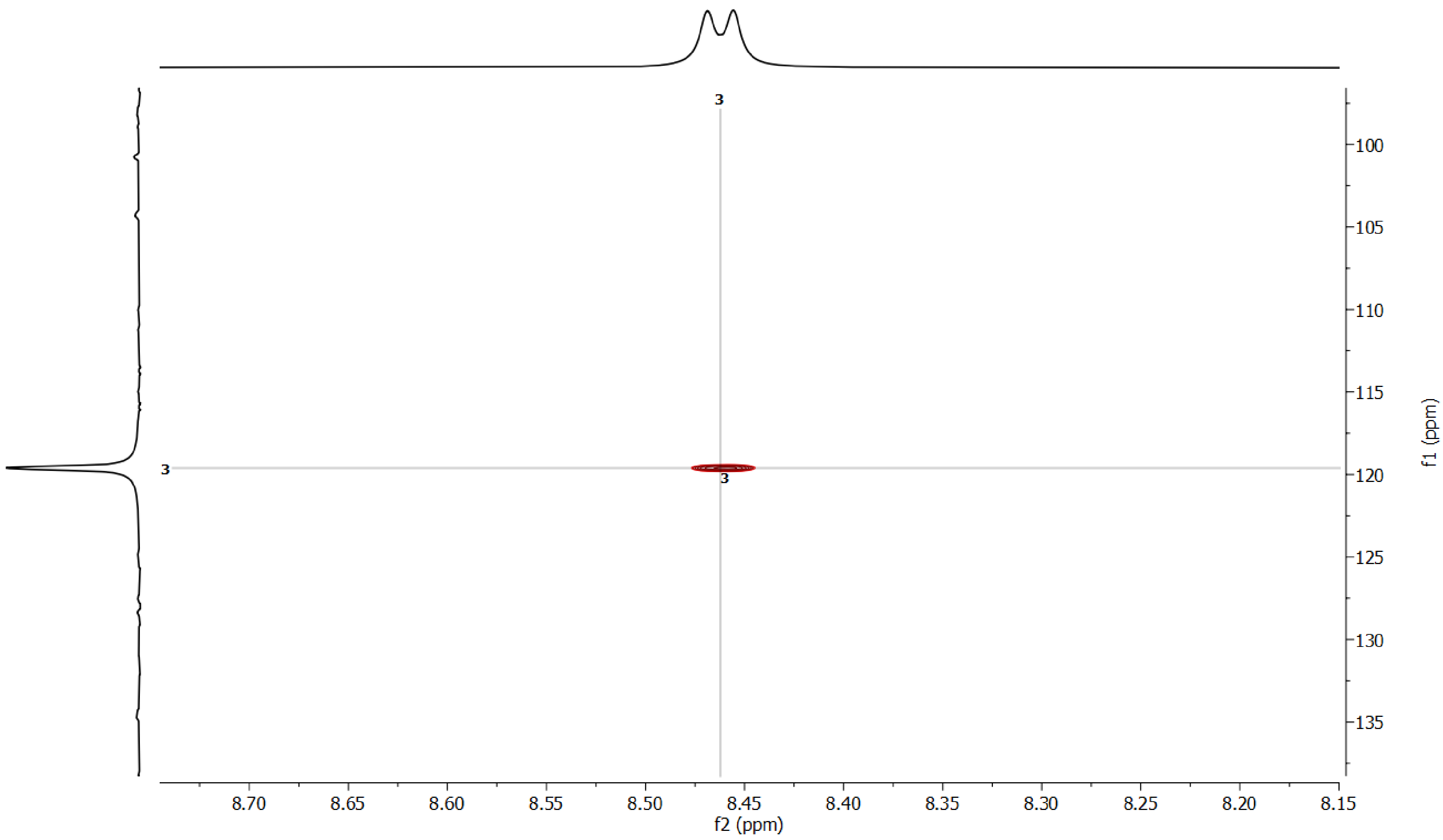

Cysteine–S–C_2H_4–thiosulfate (S7):

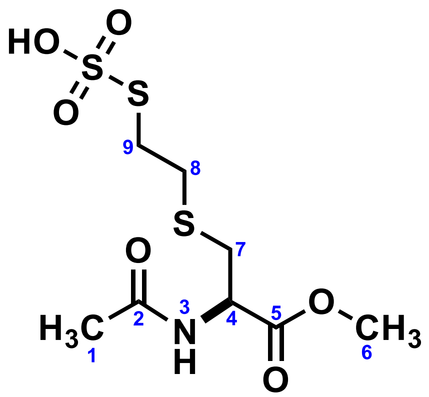

Table S27. Characterization table of Cysteine–S–C_2H_4–thiosulfate. The ^{15}N chemical shifts were referenced to NH_3(l).

Atom	δ (ppm)	J	HSQC	^{15}N-HMBC	HMBC	NOESY
C1	21.79		1			
H1	2.106	s	1	3	2	3, 4, 6, 7a, 7b, 8, 9
C2	174.34				1, 4	
N3	123.86			1, 7a, 7b		
H3	8.536	d 7.6(4), residual signal				1, 7b, 8
C4	52.85		4		7a, 7b	
H4	4.707	d 8.0(7b), d 4.9(7a)	4		2, 5, 7	1, 6, 7a, 7b, 8, 9
C5	172.74				4, 6, 7a, 7b	
C6	53.29		6			
H6	3.826	s	6		5	1, 4, 7a, 7b, 8, 9
C7	32.49		7a, 7b		4, 8	
H7a	3.191	d 14.0(7b), d 4.9(4)	7	3	4, 5, 8	1, 4, 6, 7b, 8, 9
H7b	3.050	d 14.0(7a), d 8.0(4)	7	3	4, 5, 8	1, 3, 4, 6, 7a
C8	31.68		8		7a, 7b, 9	
H8	3.043	m (o.l.)	8		7, 9	1, 3, 4, 6, 7a, 9
C9	34.76		9		8	
H9	3.343	m	9		8	1, 4, 6, 7a, 8

^{1}H NMR of Cysteine–S–C_2H_4–thiosulfate **(S7)**:
600 MHz, D_2O, 298 K

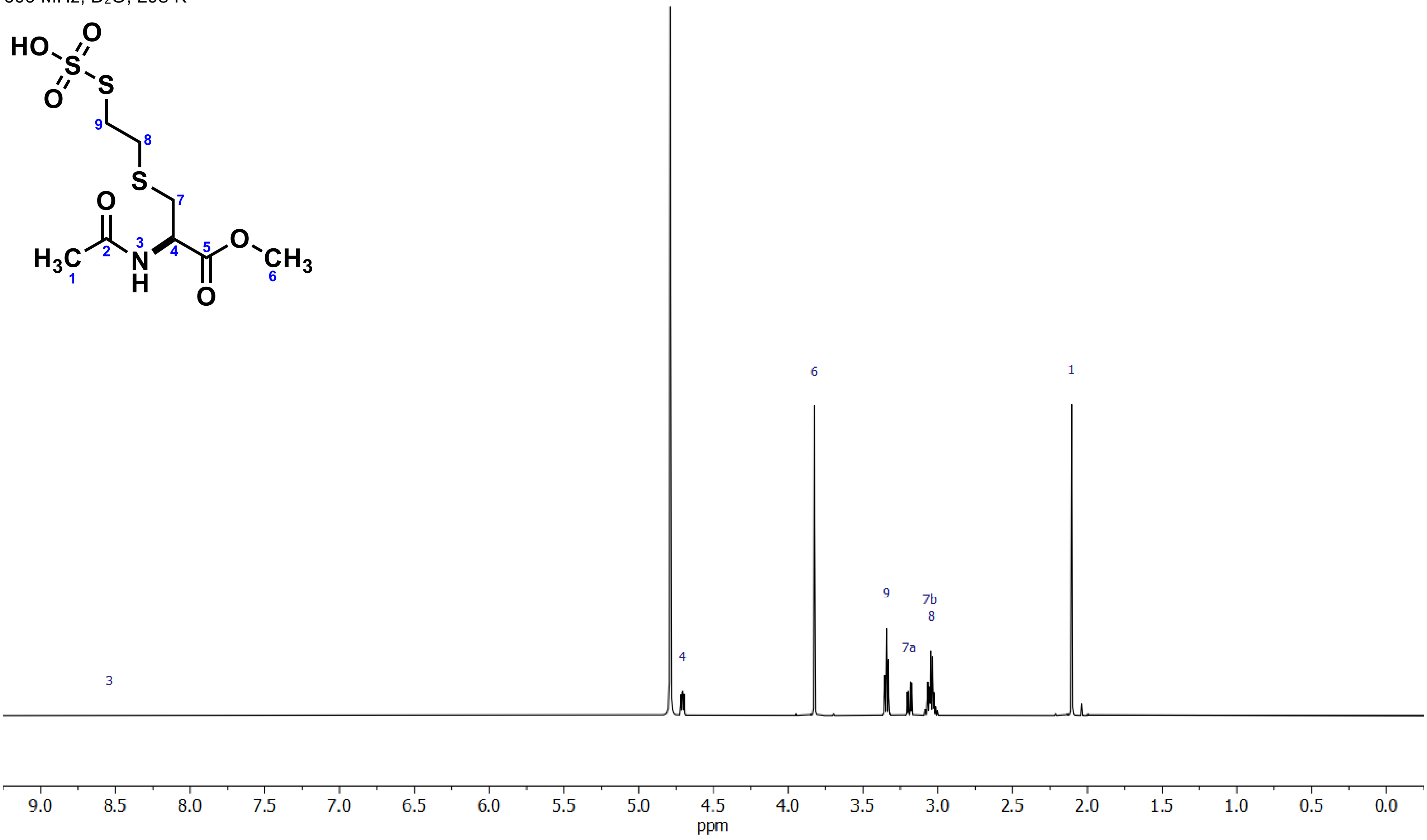

^{13}C NMR of Cysteine–S–C_2H_4–thiosulfate **(S7)**:
151 MHz, D_2O, 298 K

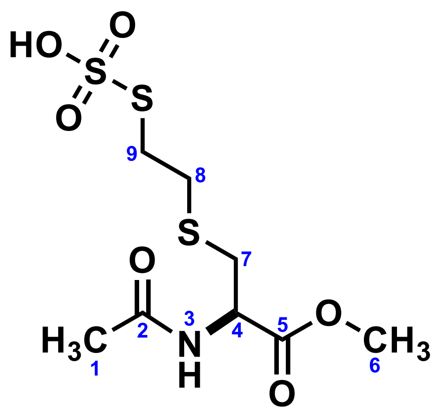

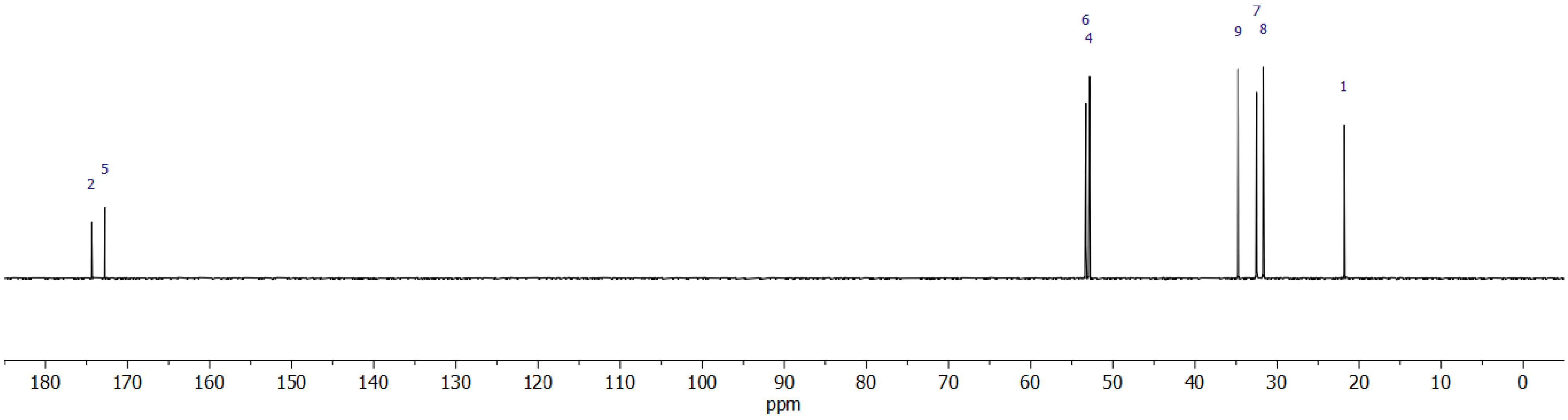

HSQC-NMR of Cysteine–S–C_2H_4–thiosulfate **(S7)**:
D_2O, 298 K

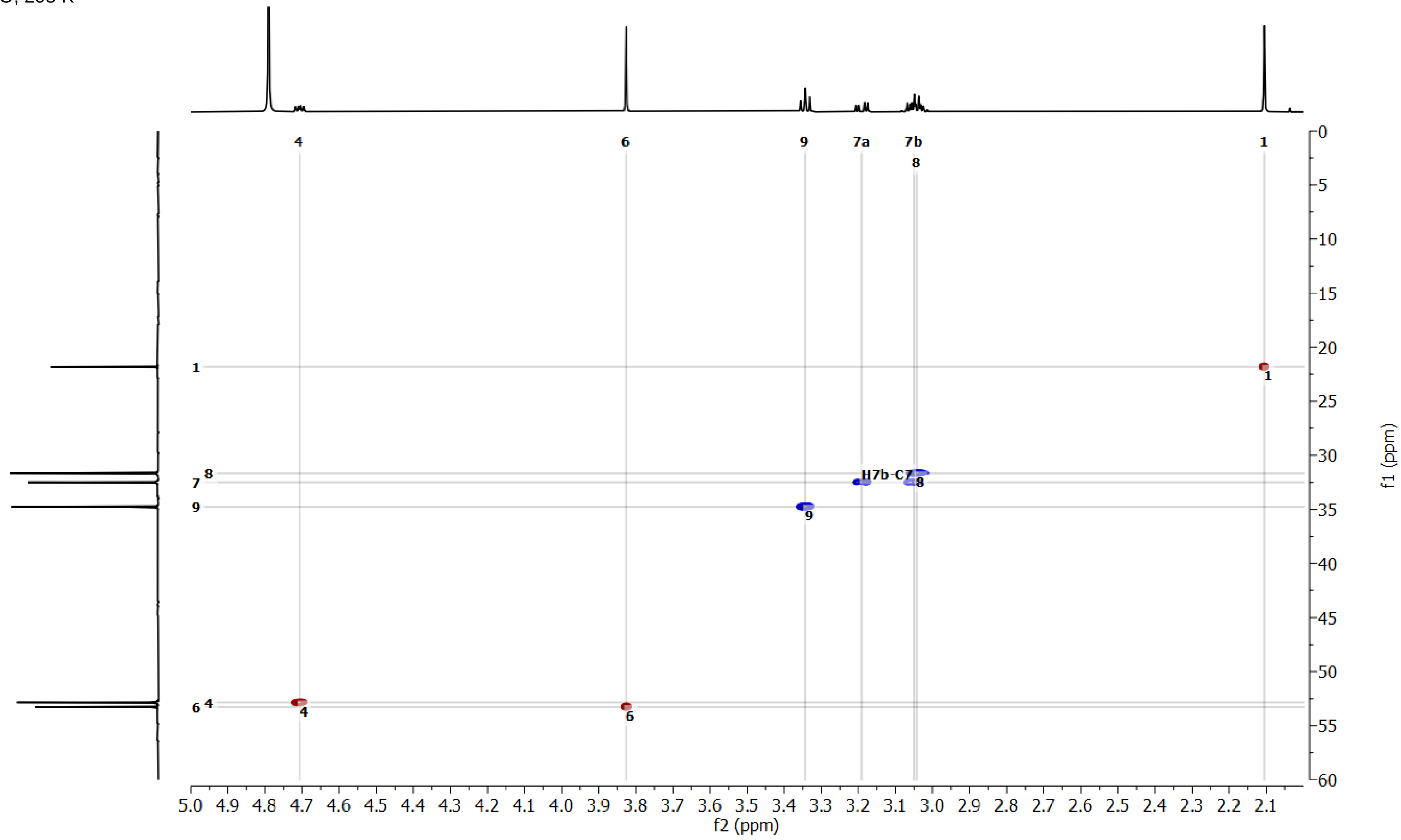

HMBC-NMR of Cysteine–S–C_2H_4–thiosulfate **(S7)**:
D_2O, 298 K

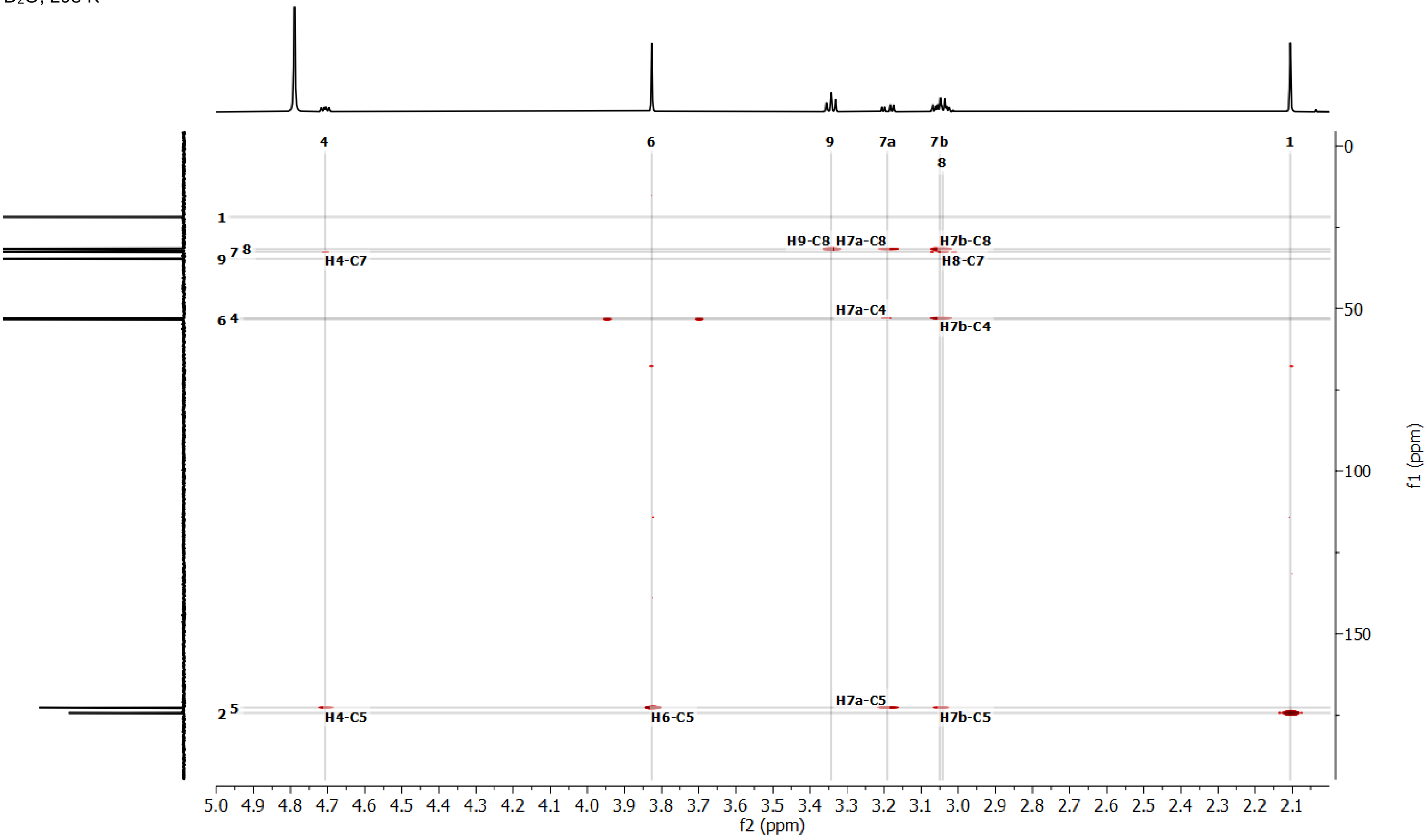

NOESY-NMR of Cysteine–S–C_2H_4–thiosulfate **(S7)**:
D_2O, 298 K

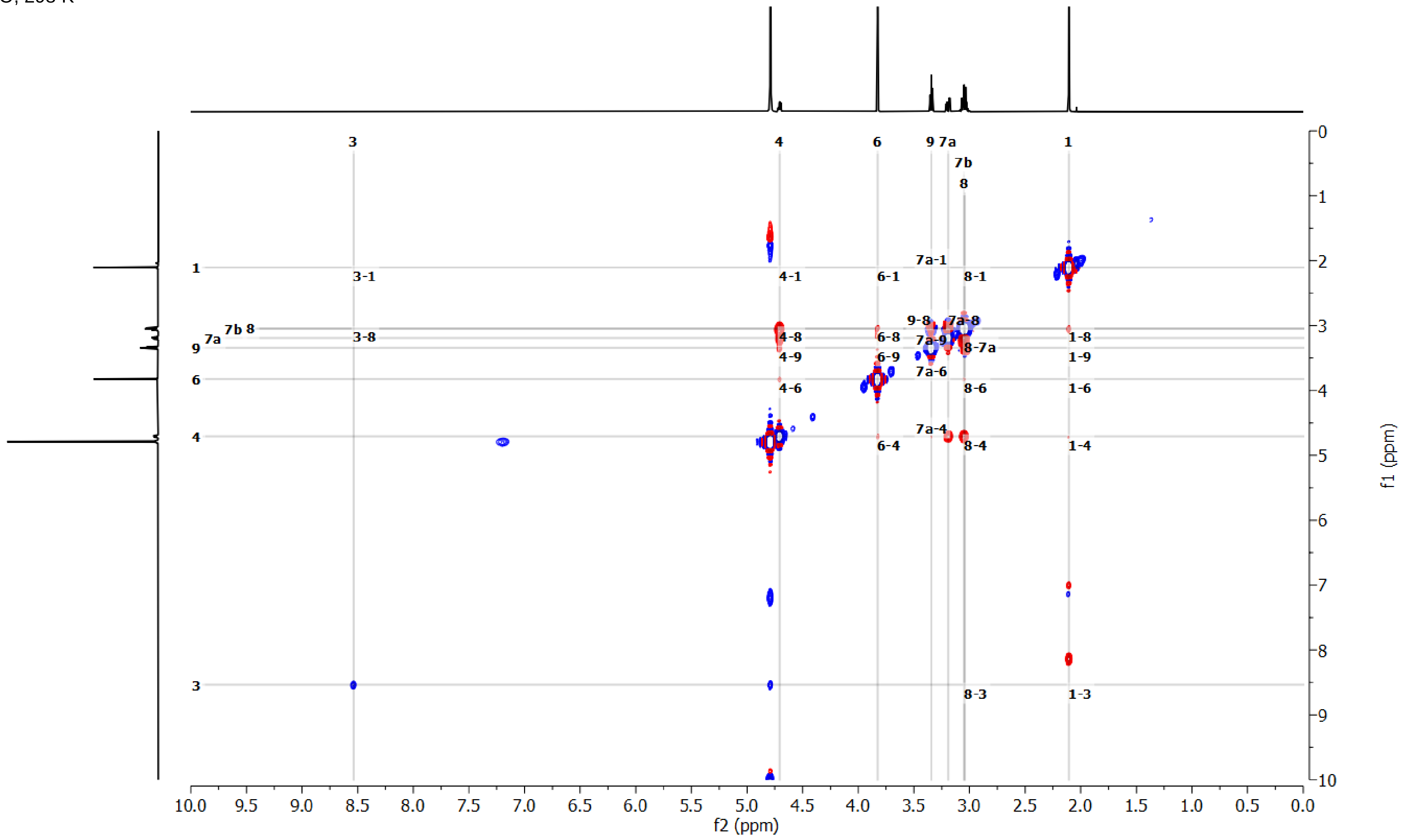

^{15}N-HMBC-NMR of Cysteine–S–C_2H_4–thiosulfate **(S7)**:
D_2O, 298 K

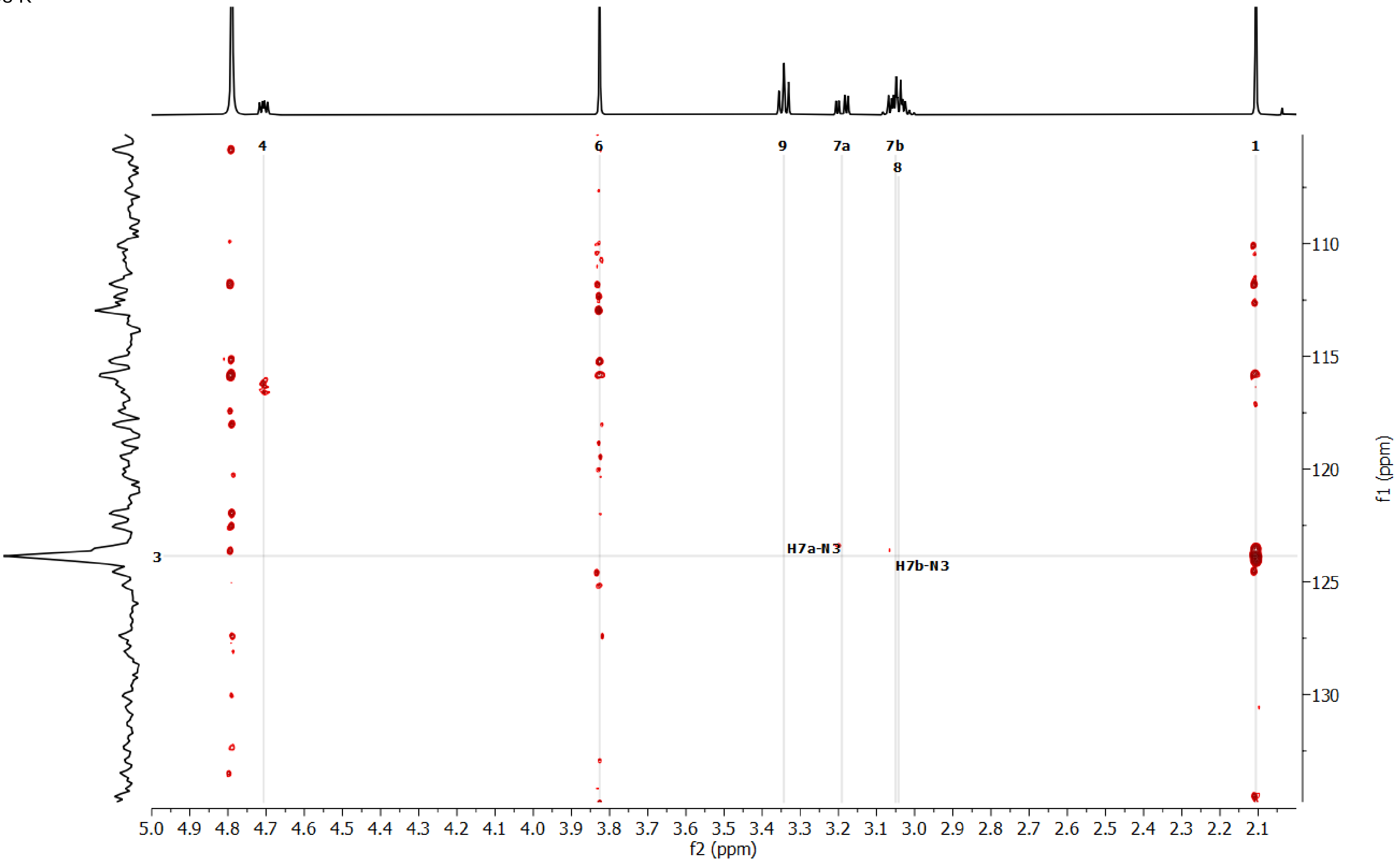

NMR-Characterization of peptides

Ac-Ala-Cys-Leu-Leu-Gln-Glu-Phe-Ala-Pro-Pro-Trp-Ile-NH_2 (S10):

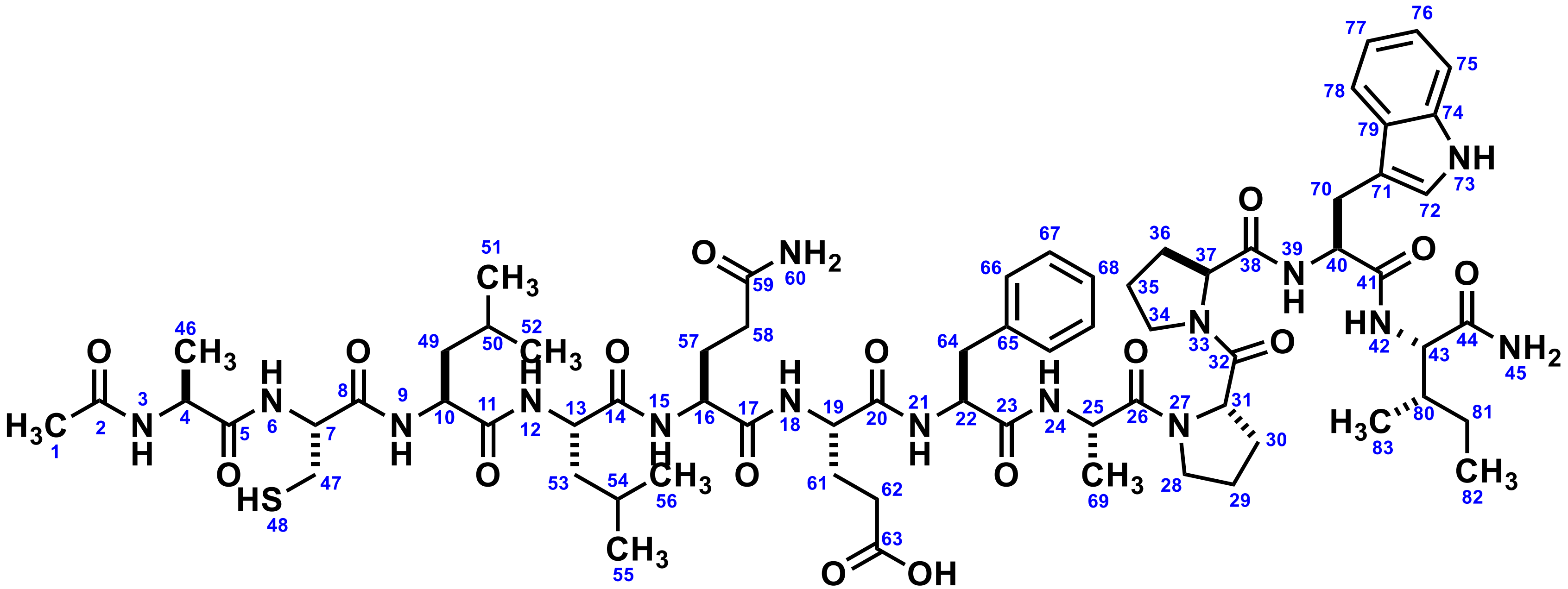

Table S28. Characterization table of Ac-Ala-Cys-Leu-Leu-Gln-Glu-Phe-Ala-Pro-Pro-Trp-Ile-NH_2. The ^{15}N chemical shifts were referenced to CH_3NO_2.

Atom	δ (ppm)	J	HSQC	^{15}N-HSQC	HMQC	COSY	ROESY	TOCSY
C1	22,44		1					
H1	1,835	s	1		2, 4			
C2	169,30				1, 3, 4			
N3	-254,29			3				
H3	8,112	d 7.0(4)		3	2, 4, 46	4	46	4, 46
C4	48,43		4		1, 3, 46			
H4	4,247	quint 7.1(4, 46)	4		2, 5, 46	3, 46	6	3, 46
C5	172,69				4, 6, 7			
N6	-266,01			6				

**H6**	8,054	d 7.9(7)		6	5, 7	7	4, 9, 46, 47a, 47b, 48	7, 47a, 47b, 48
**C7**	54,85		7		6, 47a, 47b, 48			
**H7**	4,358	d 7.9(6), d 7.4(47b), d 5.3(47a)	7		5, 8, 47	6, 47a, 47b	9	6, 47a, 47b, 48
**C8**	169,54				7, 9, 10, 47a, 47b			
**N9**	-260,38			9				
**H9**	7,953	d 7.9(10)		9	8, 10	10	6, 7, 10, 47a, 47b, 49, 50	10, 49, 51, 52
**C10**	51,23		10		9, 49			
**H10**	4,275	m (o.l.)	10		8, 11, 49, 50	9, 49	9, 12, 52	9, 49, 51, 52
**C11**	171,79				10, 12, 49			
**N12**	-261,98			12				
**H12**	7,885	d 7.8(13)		12	11, 13	13	10, 13, 15, 49, 50, 53, 54	13, 53, 55, 56
**C13**	50,98		13		12, 53			
**H13**	4,269	m (o.l.)	13		53, 54	12, 53	12, 15, 53, 54, 56	12, 53, 55, 56
**C14**	171,96				15, 53			
**N15**	-262,54			15				
**H15**	7,955	d 8.0(16)		15	14, 16	16	12, 13, 16, 18, 54, 55, 57a, 57b, 58	16, 57a, 57b, 58
**C16**	52,17		16		15, 57b			
**H16**	4,161	m (o.l.)	16		17, 57, 58	15, 57a, 57b	15, 18, 57a, 57b, 58	15, 57a, 57b, 58
**C17**	170,97				16, 18, 57b			
**N18**	-263,13			18				
**H18**	7,841	d 7.9(19)		18	17	19	15, 16, 19, 21, 57a, 58, 61a, 61b, 62	19, 61a, 61b, 62
**C19**	51,81		19		62			
**H19**	4,180	m (o.l.)	19		20, 61, 62	18, 61a, 61b	18, 21, 61a, 61b, 62	18, 61a, 61b, 62
**C20**	170,70				19, 21, 22			
**N21**	-263,61			21				
**H21**	7,911	d 8.2(22)		21	20	22	18, 19, 22, 24, 64a, 64b, 66	22, 64a, 64b
**C22**	53,46		22		64a, 64b			
**H22**	4,510	m (o.l.)	22		20, 23, 64, 65	21, 64a, 64b	21, 24, 66	21, 64a, 64b
**C23**	170,24				22, 24, 25, 64a, 64b			

**N24**	-258,69			24				
**H24**	8,137	d 7.2(25)		24	23	25	21, 22, 64a, 69	25, 69
**C25**	46,09		25		69			
**H25**	4,467	m (o.l.)	25		23, 26, 69	24, 69	28a, 28b, 29a, 29b	24, 69
**C26**	169,63				25, 31, 69			
**N27**	n.f.							
**C28**	46,53		28a, 28b		30b			
**H28a**	3,534	d 9.6(28b), t 7.0(29a, b)	28			28b, 29a, 29b	25, 28b, 30a	28b, 29a, 29b, 30a, 30b, 31
**H28b**	3,404	d 9.7(28a), t 7.5(29a, b)	28			28a, 29a, 29b	25, 28a, 69	28a, 29a, 29b, 30a, 30b, 31
**C29**	24,47		29a, 29b		30b, 31			
**H29a**	1,828	m (o.l.)	29			28a, 28b, 29b	25	28a, 28b, 29b, 30a, 30b, 31
**H29b**	1,748	m (o.l.)	29		31	28a, 28b, 29a	25	28a, 28b, 29a, 30a, 30b, 31
**C30**	27,73		30a, 30b		31			
**H30a**	1,992	m (o.l.)	30		32	30b, 31	28a, 30b, 31	28a, 28b, 29a, 29b, 30b, 31
**H30b**	1,571	m (o.l.)	30		28, 29, 32	30a, 31	30a, 31	28a, 28b, 29a, 29b, 30a, 31
**C31**	57,42		31		29b			
**H31**	4,469	m (o.l.)	31		26, 29, 30	30a, 30b	30a, 30b, 34a, 34b	28a, 28b, 29a, 29b, 30a, 30b
**C32**	170,31				30a, 30b			
**N33**	n.f.							
**C34**	46,57		34a, 34b					
**H34a**	3,596	d 9.6(34b), t 7.4(35)	34			34b, 35	31, 34b, 36a	34b, 35, 36a, 36b, 37

**H34b**	3,447	m	34			34a, 35	31, 34a	34a, 35, 36a, 36b, 37
**C35**	24,29		35		37			
**H35**	1,814	m (o.l.)	35			34a, 34b, 36a, 37		34a, 34b, 36a, 36b, 37
**C36**	28,61		36a, 36b		37			
**H36a**	1,950	m (o.l.)	36			35, 36b, 37	34a, 37	34a, 34b, 35, 37
**H36b**	1,819	m (o.l.)	36			36a, 37	37, 39	34a, 34b, 35, 37
**C37**	59,36		37					
**H37**	4,295	d 8.5(36a), d 2.7(36b)	37		35, 36	35, 36a, 36b	36a, 36b, 39	34a, 34b, 35, 36a, 36b
**C38**	171,34				39, 40			
**N39**	-264,23			39				
**H39**	7,759	d 7.6(40)		39	38, 40	40	36b, 37, 42, 70a, 70b, 72	40, 70a, 70b
**C40**	53,43		40		39, 70a, 70b			
**H40**	4,501	m (o.l.)	40		38, 41, 70, 71	39, 70a, 70b	42, 72, 78	39, 70a, 70b, 72
**C41**	170,88				40, 42, 43, 70a, 70b			
**N42**	-264,61			42				
**H42**	7,626	d 9.0(43)		42	41, 43	43	39, 40, 45a, 70a, 70b, 80, 81a, 81b, 83	43, 80, 81a, 81b, 82, 83
**C43**	56,71		43		42, 45b, 80, 81a, 81b, 83			
**H43**	4,125	d 9.0(42), d 6.9(80)	43		41, 44, 80, 81, 83	42, 80	45a, 80, 83	42, 80, 81a, 81b, 82, 83
**C44**	172,72				43, 45a, 46			
**N45**	-273,09			45a, 45b				
**H45a**	7,266	(br) m		45	44	45b	42, 43, 80, 83	45b
**H45b**	7,043	(br) m		45	43	45a		45a

**C46**	17,88		46		3, 4			
**H46**	1,181	d 7.1(4)	46		4, 44	4	3, 6	3, 4
**C47**	25,93		47a, 47b		7, 48			
**H47a**	2,781	d 13.5(47b), d 8.9(48), d 5.3(7)	47		7, 8	7, 47b, 48	6, 9	6, 7, 47b, 48
**H47b**	2,710	d 13.5(47a), d 8.0(48), d 7.4(7)	47		7, 8	7, 47a, 48	6, 9	6, 7, 47a, 48
**SH48**	2,299	d 8.9(47a), d 8.0(47b)			7, 47	47a, 47b	6	6, 7, 47a, 47b
**C49**	40,42		49		10, 51, 52			
**H49**	1,454	m (o.l.)	49		10, 11, 50, 51, 52	10, 50	9, 12, 51	9, 10, 51
**C50**	24,09		50		10, 49, 51, 52			
**H50**	1,585	m (o.l.)	50		51, 52	49, 51, 52	9, 12	
**C51**	23,06		51		49, 50, 52			
**H51**	0,869	d 6.6(50)	51		49, 50, 52	50	49	9, 10, 49, 52
**C52**	21,49		52		49, 50, 51			
**H52**	0,824	d 6.6(50)	52		49, 50, 51	50	10	9, 10, 51
**C53**	40,55		53		13, 55, 56			
**H53**	1,437	m (o.l.)	53		13, 14, 54, 55, 56	13, 54	12, 13, 55	12, 13
**C54**	24,04		54		13, 53, 55, 56			
**H54**	1,585	m (o.l.)	54		55, 56	53, 55, 56	12, 13, 15	
**C55**	23,05		55		53, 54, 56			
**H55**	0,857	d 6.4(54)	55		53, 54, 56	54	15, 53	12, 13, 56
**C56**	21,49		56		53, 54, 55			
**H56**	0,809	d 6.6(54)	56		53, 54, 55	54	13	12, 13, 55
**C57**	27,56		57a, 57b		16, 58			
**H57a**	1,818	m (o.l.)	57		58	16, 57b, 58	15, 16, 18	15, 16, 58
**H57b**	1,701	m (o.l.)	57		16, 17	16, 57a, 58	15, 16	15, 16, 58
**C58**	31,45		58		16, 57a			

**H58**	2,053	m	58		57, 59	57a, 57b	15, 16, 18, 60a, 60b	15, 16, 57a, 57b
**C59**	173,79				58			
**N60**	-271,89			60a, 60b				
**H60a**	7,196	(br) m		60		60b	58	60b
**H60b**	6,777	(br) m		60		60a	58	60a
**C61**	27,50		61a, 61b		19, 62			
**H61a**	1,793	m (o.l.)	61			19, 61b, 62	18, 19	18, 19, 62
**H61b**	1,655	m (o.l.)	61			19, 61a, 62	18, 19	18, 19, 62
**C62**	29,95		62		19			
**H62**	2,124	m	62		19, 61, 63	61a, 61b	18, 19	18, 19, 61a, 61b
**C63**	173,90				62			
**C64**	37,36		64a, 64b		22, 66			
**H64a**	2,986	d 13.7(64b), d 5.0(22)	64		22, 23, 65, 66	22, 64b	21, 24, 66	21, 22
**H64b**	2,742	d 13.7(64b), d 9.5(22)	64		22, 23, 65, 66	22, 64a	21, 66	21, 22
**C65**	137,54				22, 64a, 64b, 67			
**C66**	129,15		66		64a, 64b, 68			
**H66**	7,217	m (o.l.)	66		64, 68		21, 22, 64a, 64b	68
**C67**	127,98		67					
**H67**	7,217	m (o.l.)	67		65	68		68
**C68**	126,24		68		66			
**H68**	7,160	m	68		66	67		66, 67
**C69**	16,75		69		25			
**H69**	1,152	d 6.9(25)	69		25, 26	25	24, 28b	24, 25
**C70**	26,92		70a, 70b		40			
**H70a**	3,145	d 14.9(70b), d 5.3(40)	70		40, 41, 71, 72, 79	40, 70b, 72	39, 42, 72, 78	39, 40, 70b, 72

**H70b**	3,010	d 15.0(70a), d 7.8(40)	70		40, 41, 71, 72, 79	40, 70a	39, 42, 72, 78	39, 40, 70a, 72
**C71**	109,72				40, 70a, 70b, 72, 73, 78			
**C72**	123,47		72		70a, 70b, 73			
**H72**	7,113	d 2.4(73)	72		71, 74, 79	70a, 73	39, 40, 70a, 70b	40, 70a, 70b, 73
**N73**	-248,91			73				
**H73**	10,813	d 2.4(72)		73	71, 72, 74, 79	72, 78	75	72, 75, 77, 78
**C74**	135,98				72, 73, 76, 78			
**C75**	111,21		75		77			
**H75**	7,312	d 8.1(76), t 0.9(77, 78)	75		77, 79	76	73	73, 76, 77, 78
**C76**	120,83		76		78			
**H76**	7,049	d 8.2(75), d 7.0(77), d 1.2(78)	76		74, 78	75, 77		75, 77, 78
**C77**	118,22		77		75			
**H77**	6,959	d 8.0(78), d 6.9(76), d 1.0(75)	77		75, 79	76, 78		73, 75, 76, 78
**C78**	118,25		78		76			
**H78**	7,518	d 7.9(77), d 1.1(77), d 0.8(73?)	78		71, 74, 76, 79	73, 77	40, 70a, 70b	73, 75, 76, 77
**C79**	127,45				70a, 70b, 72, 73, 75, 77, 78			
**C80**	36,77		80		43, 81a, 81b, 82, 83			
**H80**	1,675	m (o.l.)	80		43, 83	43, 81b, 83	42, 43, 45a, 81a	42, 43, 81a, 81b, 82, 83
**C81**	24,13		81a, 81b		43, 82, 83			
**H81a**	1,379	d 13.7(81b), q 7.4(82), d 3.7(80)	81		43, 80, 82, 83	81b, 82	42, 80	42, 43, 80, 81b, 82, 83
**H81b**	1,047	d 13.7(81a), d 9.4(80), q 7.4(82)	81		43, 80, 82, 83	80, 81a, 82	42	42, 43, 80, 81a, 82, 83
**C82**	11,10		82		81a, 81b			

**H82**	0,801	t 7.4(81a, b)	82		80, 81	81a, 81b		42, 43, 80, 81a, 81b
**C83**	15,33		83		43, 80, 81a, 81b			
**H83**	0,800	d 6.8(80)	83		43, 80, 81	80	42, 43, 45a	42, 43, 80, 81a, 81b

^{1}H NMR of Ac-Ala-Cys-Leu-Leu-Gln-Glu-Phe-Ala-Pro-Pro-Trp-Ile-NH_2 **(S10):**
600 MHz, $(CD_3)_2SO$, 298 K

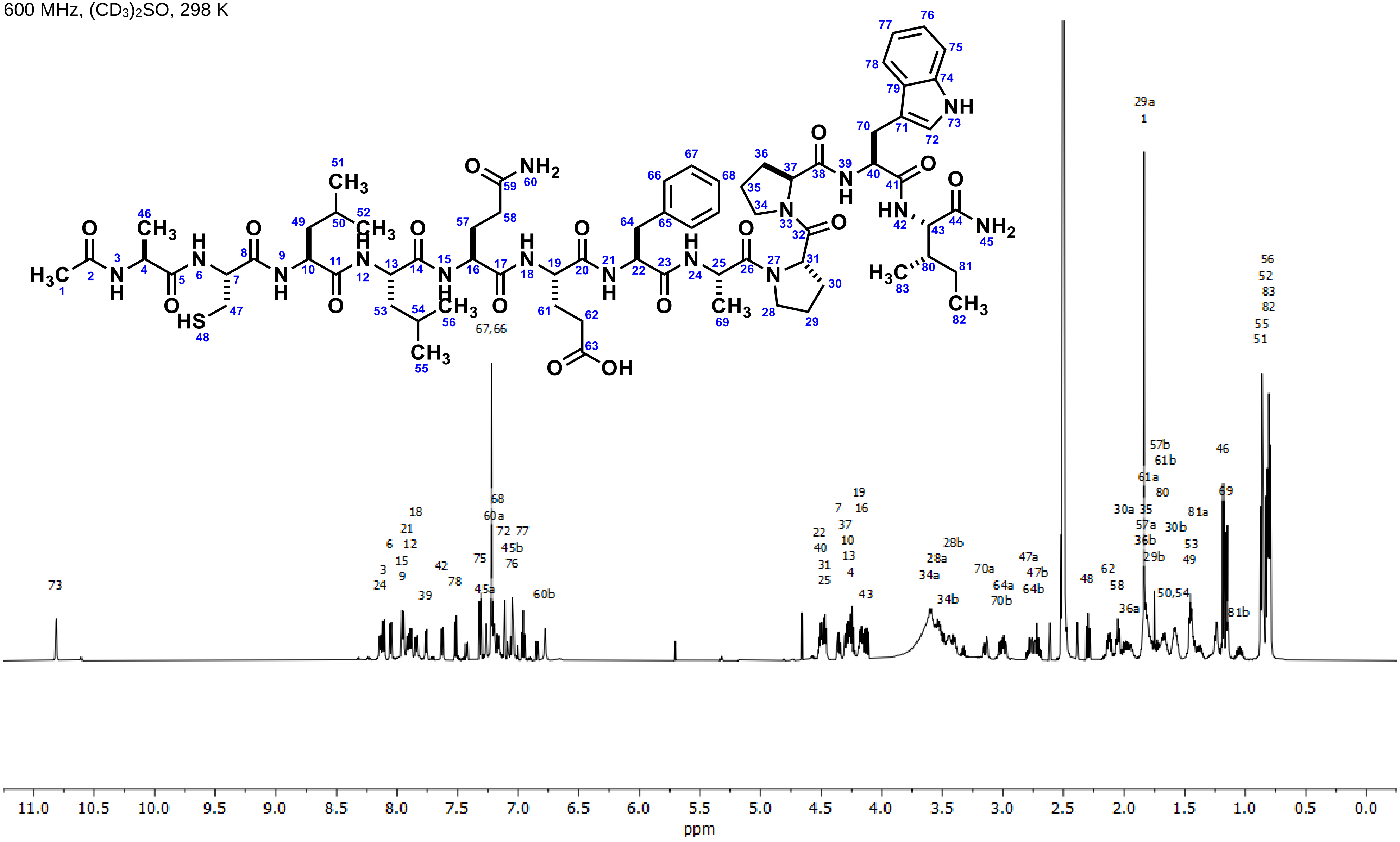

^{13}C NMR of Ac-Ala-Cys-Leu-Leu-Gln-Glu-Phe-Ala-Pro-Pro-Trp-Ile-NH$_2$ **(S10):**
151 MHz, $(CD_3)_2SO$, 298 K

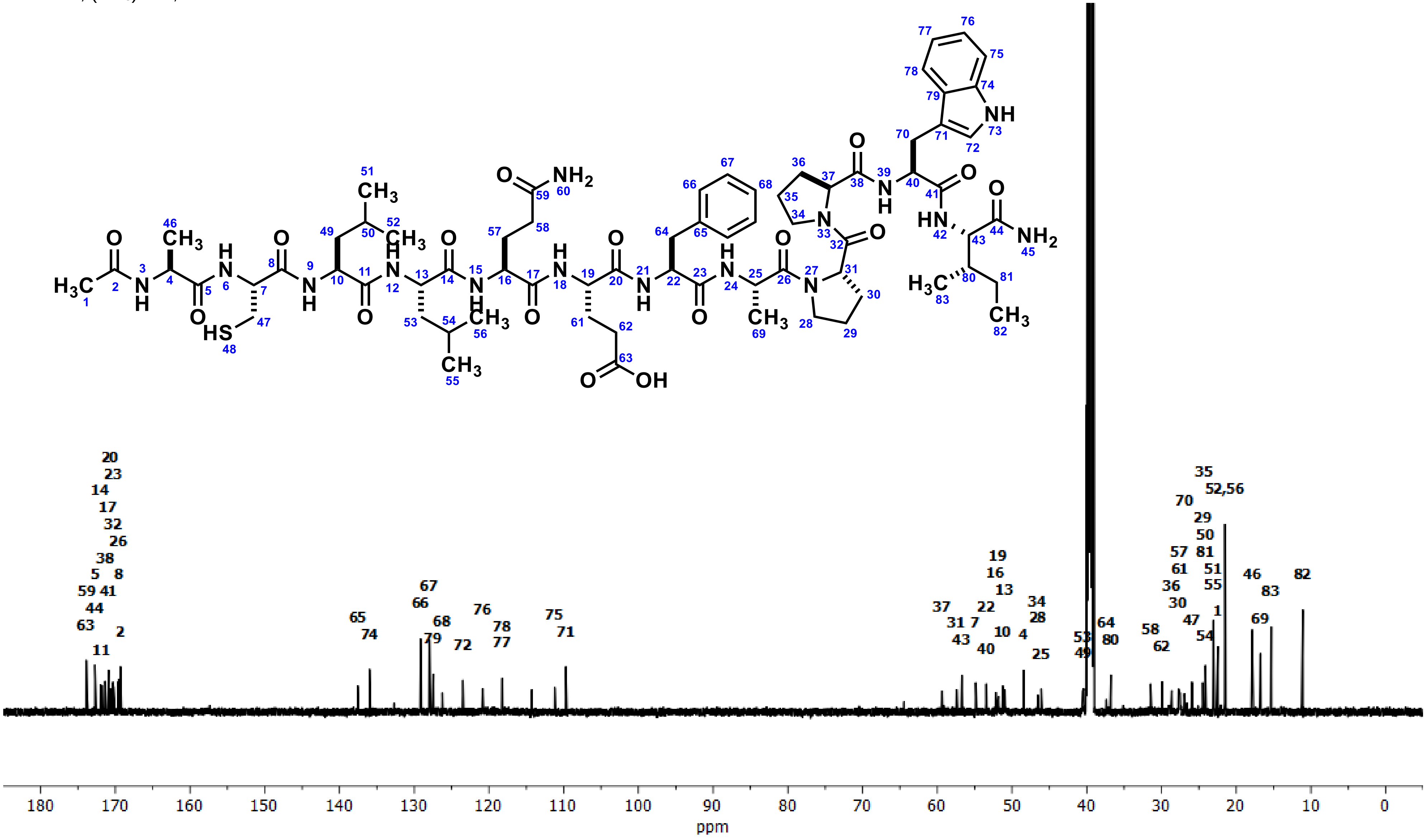

COSY-NMR of Ac-Ala-Cys-Leu-Leu-Gln-Glu-Phe-Ala-Pro-Pro-Trp-Ile-NH_2 **(S10):**
$(CD_3)_2SO$, 298 K

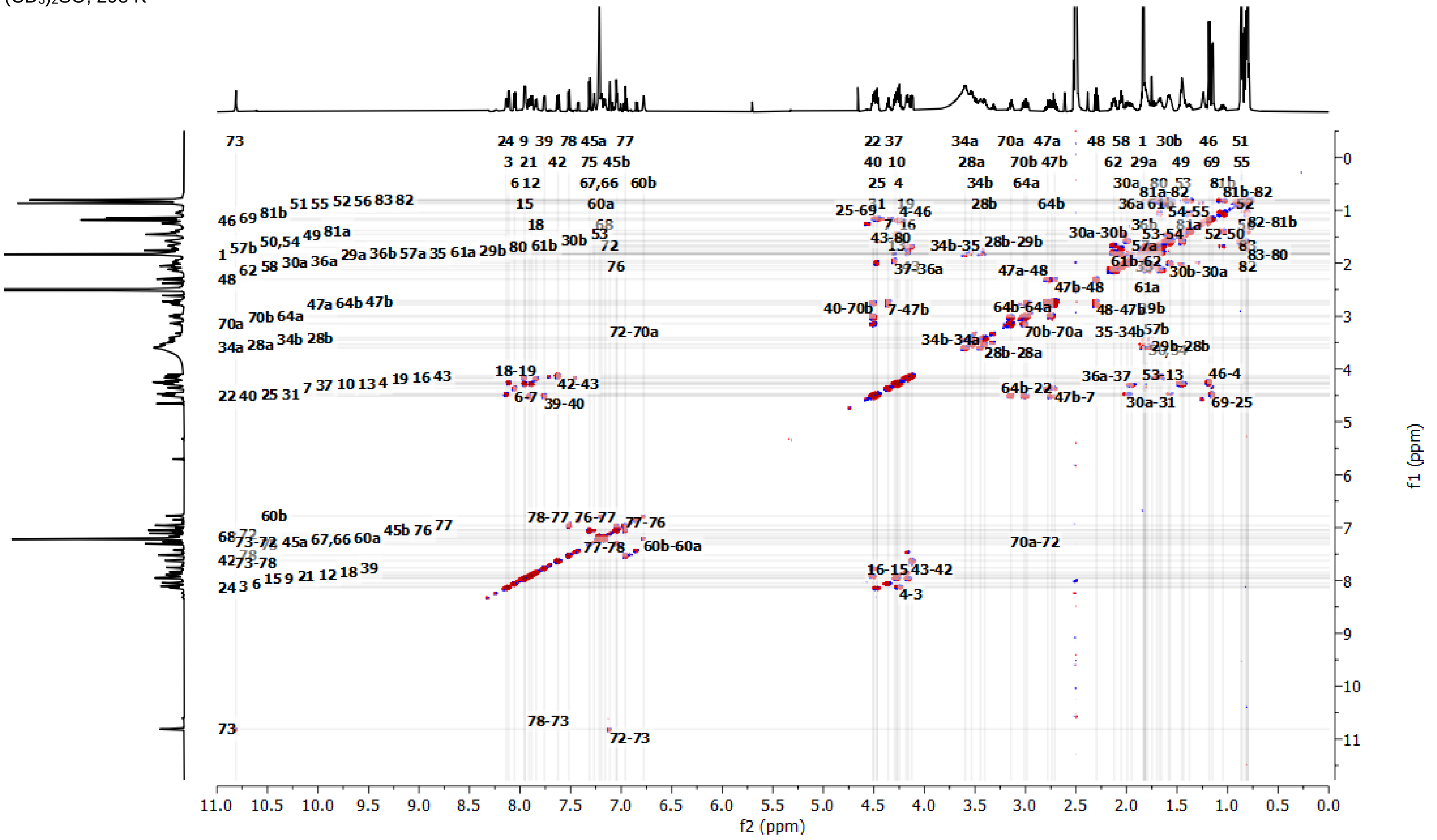

HSQC-NMR of Ac-Ala-Cys-Leu-Leu-Gln-Glu-Phe-Ala-Pro-Pro-Trp-Ile-NH_2 **(S10):**
$(CD_3)_2SO$, 298 K

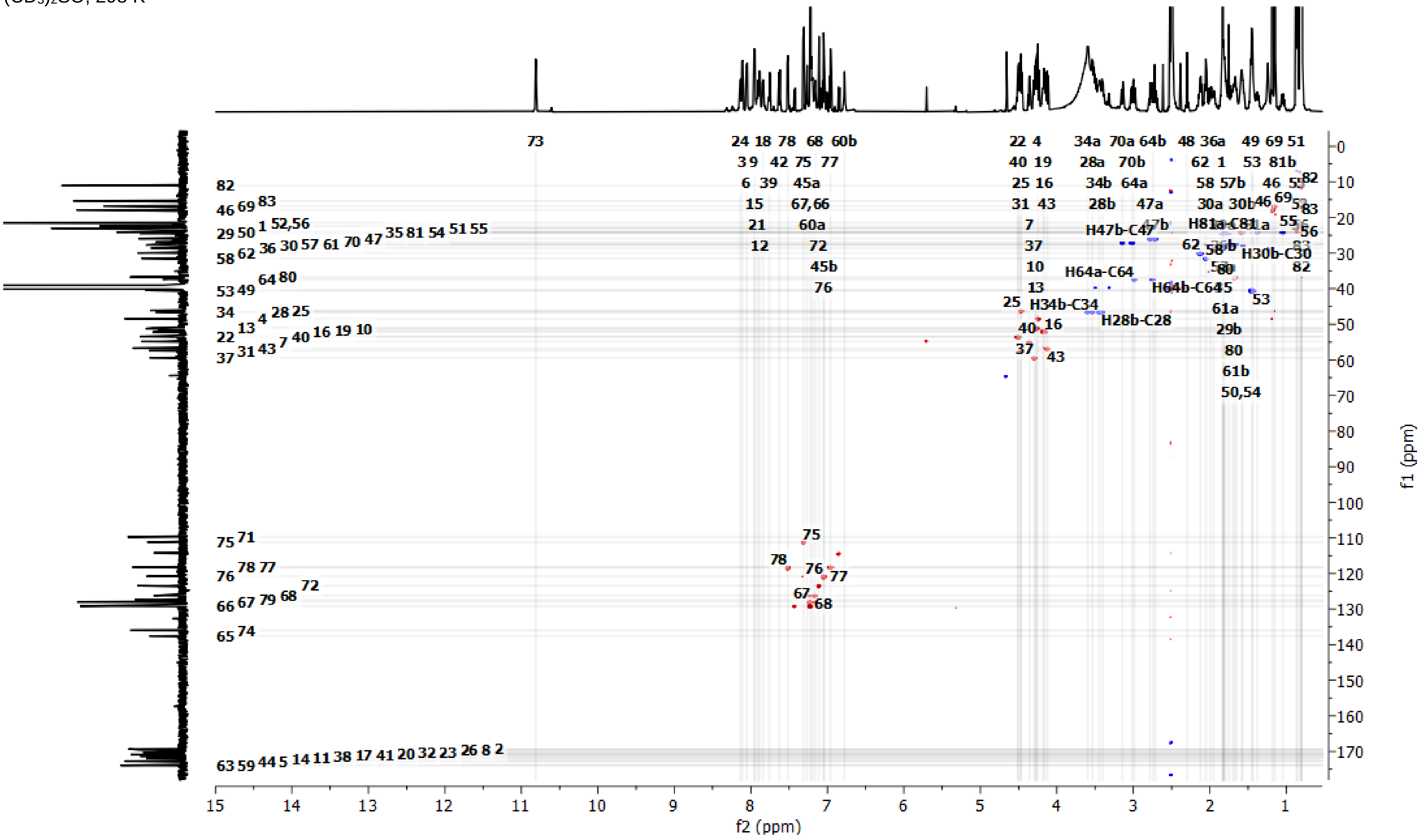

HMQC-NMR of Ac-Ala-Cys-Leu-Leu-Gln-Glu-Phe-Ala-Pro-Pro-Trp-Ile-NH_2 **(S10):**
$(CD_3)_2SO$, 298 K

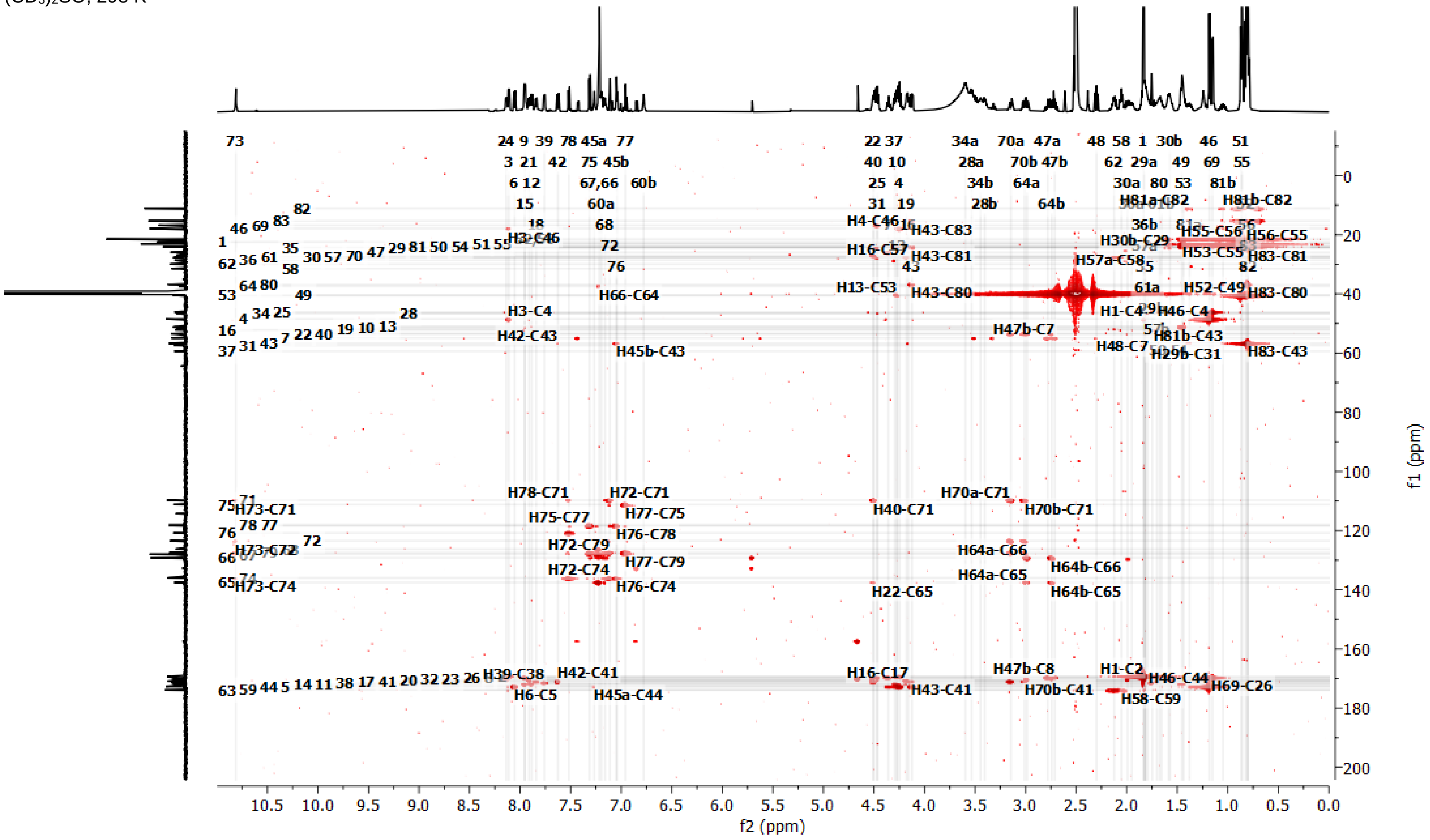

ROESY-NMR of Ac-Ala-Cys-Leu-Leu-Gln-Glu-Phe-Ala-Pro-Pro-Trp-Ile-NH_2 **(S10):**
$(CD_3)_2SO$, 298 K

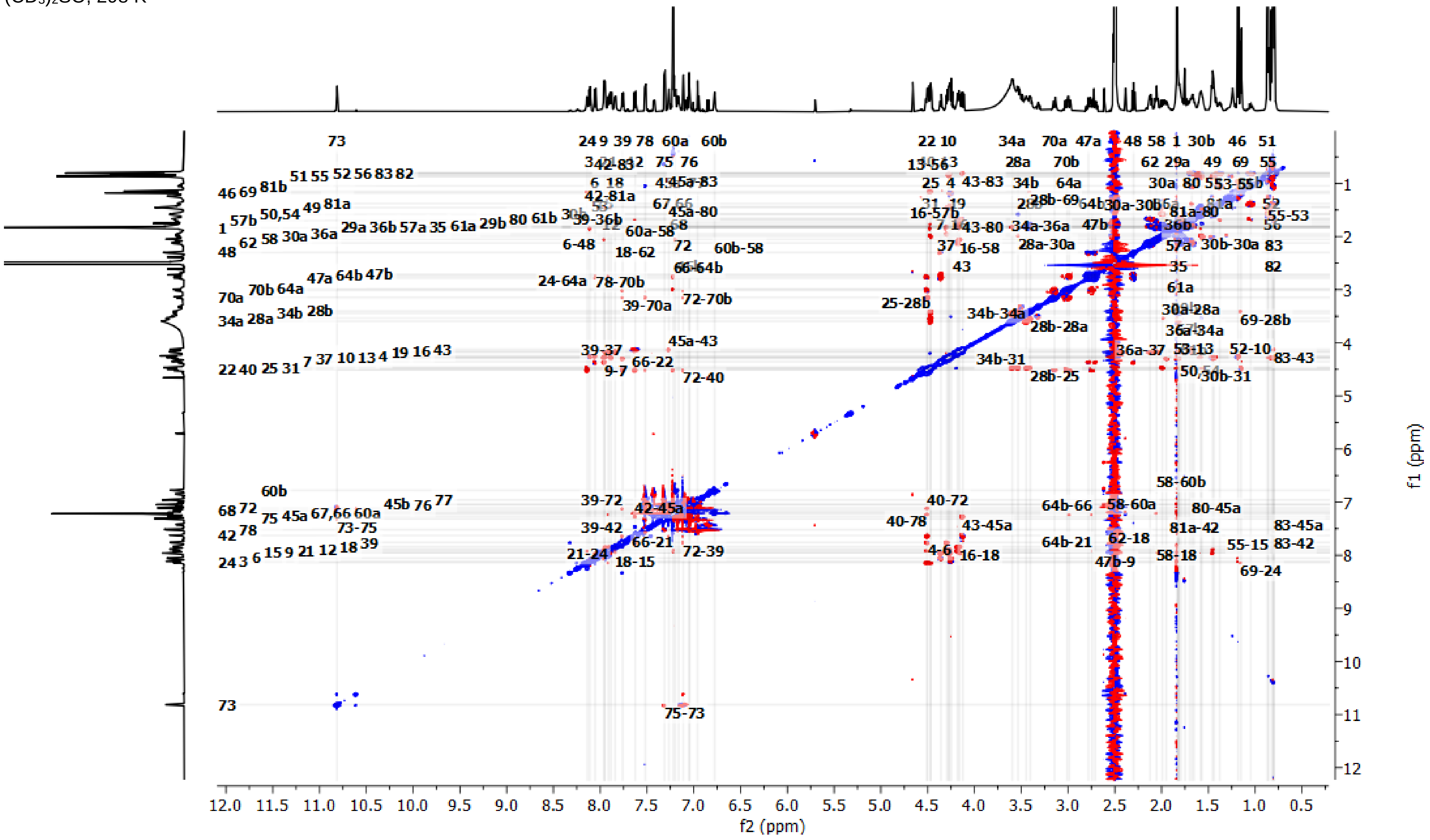

TOCSY-NMR of Ac-Ala-Cys-Leu-Leu-Gln-Glu-Phe-Ala-Pro-Pro-Trp-Ile-NH_2 **(S10):**
$(CD_3)_2SO$, 298 K

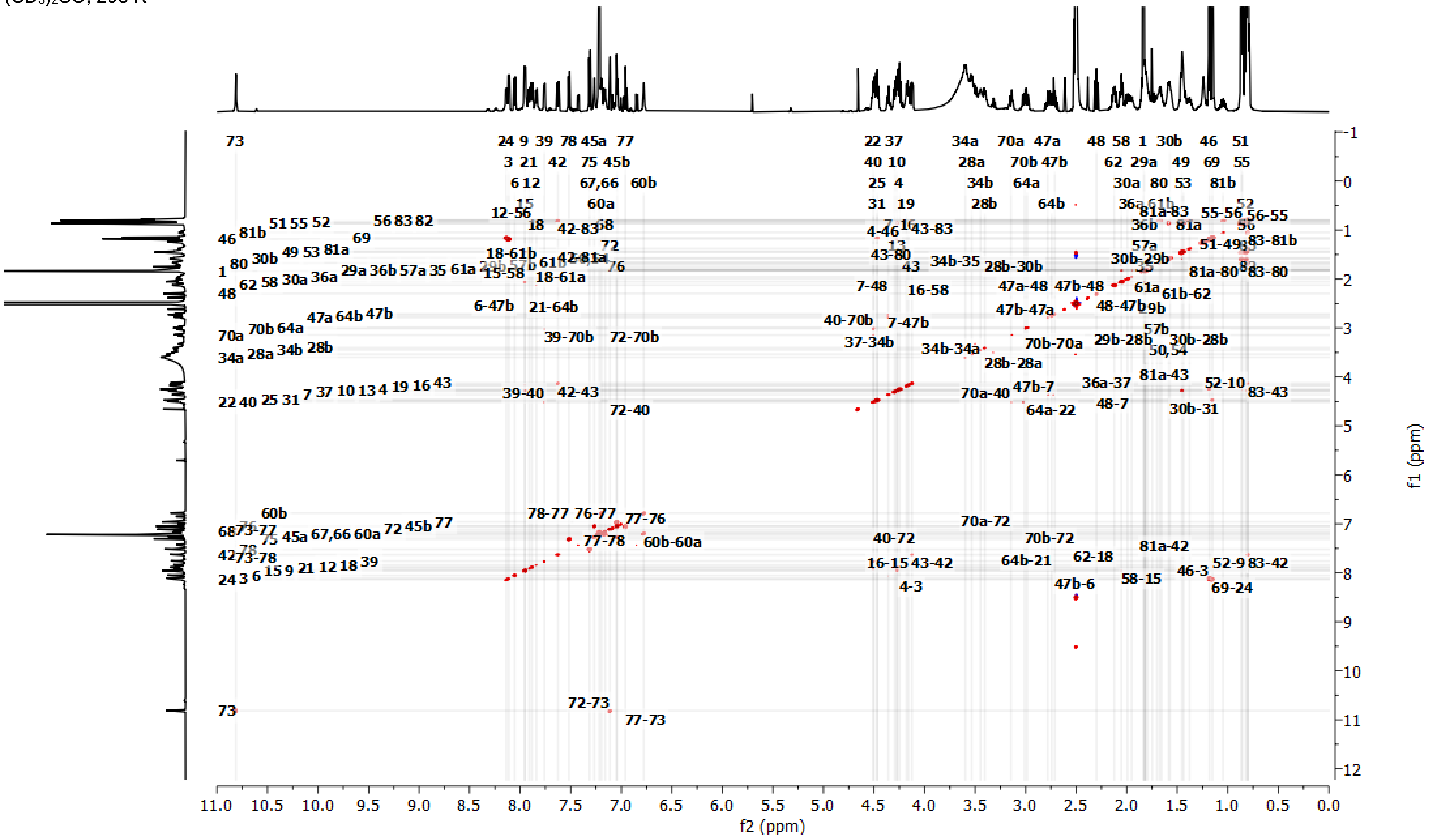

^{15}N-HSQC-NMR of Ac-Ala-Cys-Leu-Leu-Gln-Glu-Phe-Ala-Pro-Pro-Trp-Ile-NH_2 **(S10):**
$(CD_3)_2SO$, 298 K

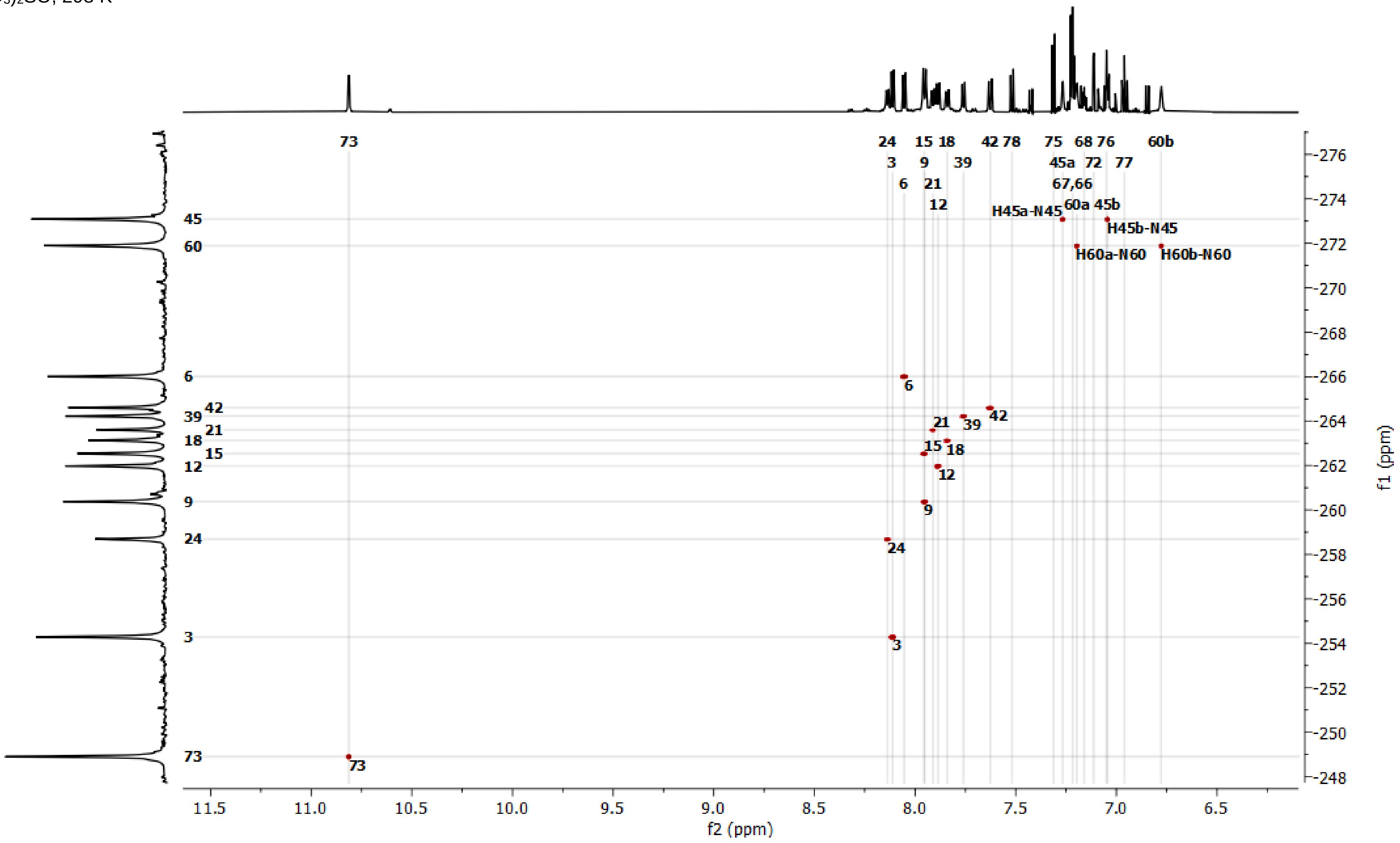

^{15}N-HMBC-NMR of Ac-Ala-Cys-Leu-Leu-Gln-Glu-Phe-Ala-Pro-Pro-Trp-Ile-NH$_2$ **(S10):**
$(CD_3)_2SO$, 298 K

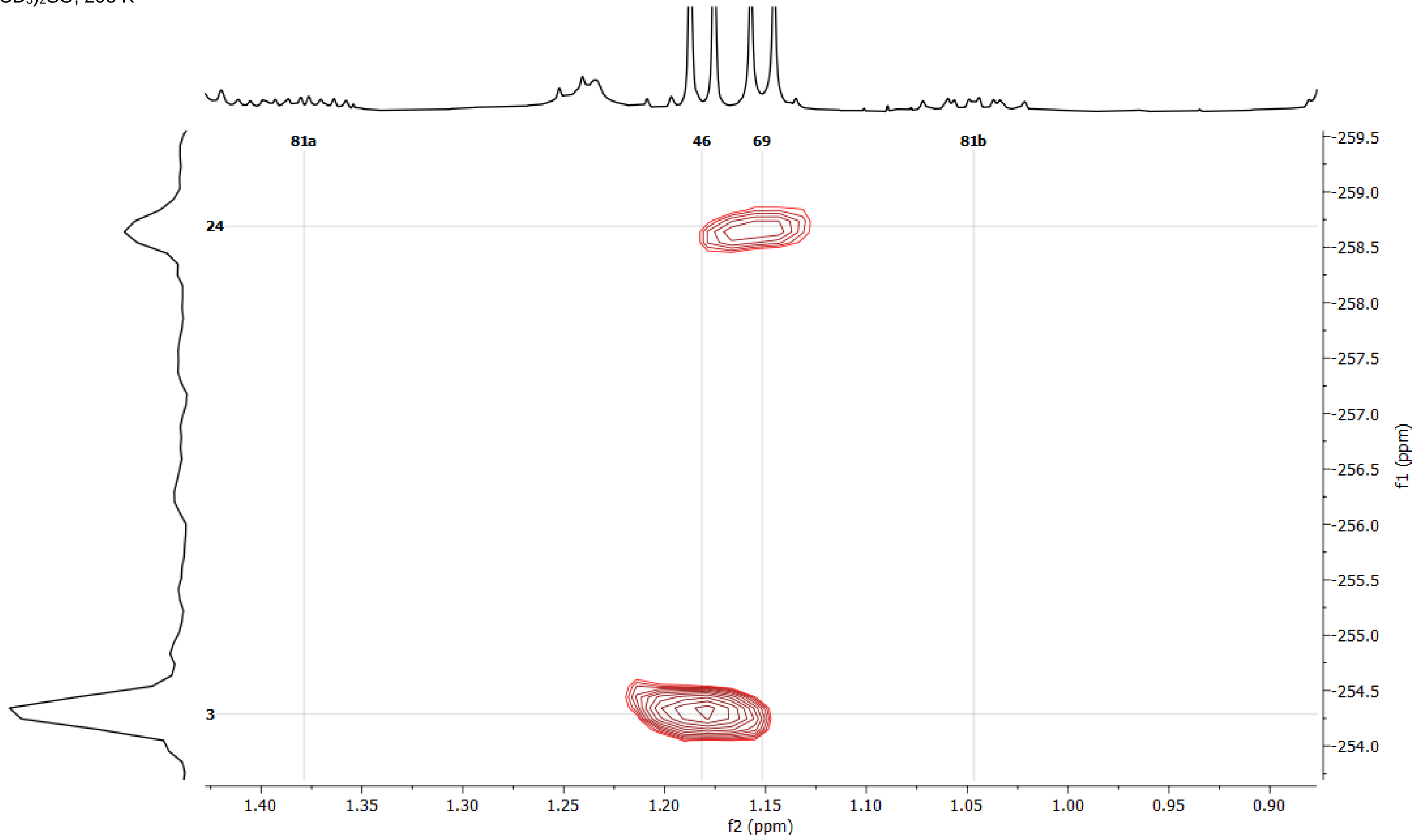

Ac-Ala-Cys-Leu-Leu-Gln-Cys-Phe-Ala-Pro-Pro-Trp-Ile-NH_2 (S11):

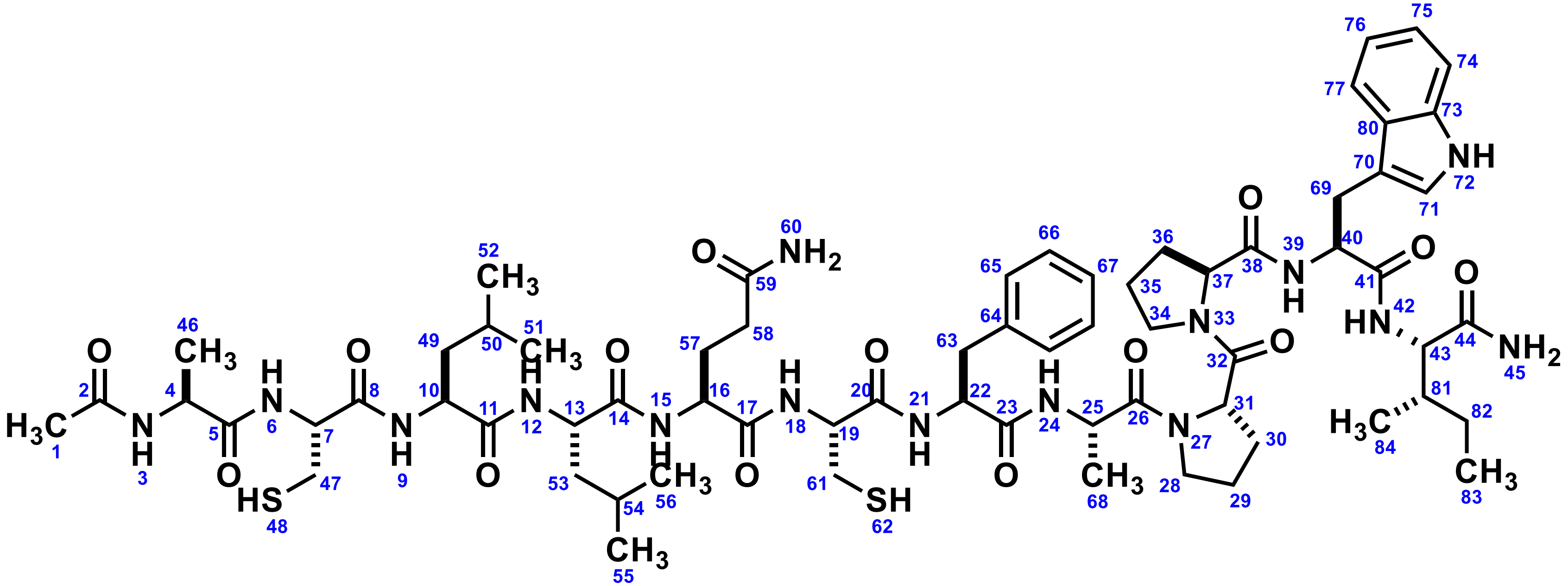

Table S29. Characterization table of Ac-Ala-Cys-Leu-Leu-Gln-Cys-Phe-Ala-Pro-Pro-Trp-Ile-NH_2. The ^{15}N chemical shifts were referenced to NH_3(l).

Atom	δ (ppm)	J	HSQC	^{15}N-HSQC	HMQC	COSY	ROESY	TOCSY
C1	22,45		1					
H1	1,835	s	1		2		3	
C2	169,30				1, 3, 4			
N3	125,41			3				
H3	8,118	d 7.0(4)		3	2, 4, 46	4, 46	1, 4	46
C4	48,46		4		3, 46			
H4	4,244	quint 7.1(3, 46)	4		2, 5, 46	3, 46	3, 6	
C5	172,71				4, 6, 7, 46			
N6	113,65			6				
H6	8,062	d 7.9(7)		6	5, 7, 47	7	4, 7, 9, 46, 47a, 47b	7, 47a, 47b, 48
C7	54,87		7		6, 47a, 47b			

**H7**	4,360	t 7.5(6, 47a/b), d 5.3(47a/b)	7		5, 8, 47	6, 47a, 47b	6, 9, 47b	6, 47a, 47b, 48
**C8**	169,60				7, 9, 47a, 47b			
**N9**	119,39			9				
**H9**	7,972	d (o.l.) 7.7(10)		9	8, 10	10	6, 7, 12, 47b, 49	10, 49, 50, 51, 52
**C10**	51,29		10		9, 49, 50			
**H10**	4,272	m (o.l.)	10		11, 49	9, 49	12, 50, 51, 52	9, 49, 50, 51, 52
**C11**	171,85				10, 12, 49			
**N12**	117,64			12				
**H12**	7,881	d 7.9(13)		12	11, 13	13	9, 10, 13, 15, 53, 54	13, 53, 54, 55, 56
**C13**	51,03		13		12, 53			
**H13**	4,273	m (o.l.)	13		14, 53	12, 53	12, 15, 54, 55, 56	12, 53, 54, 55, 56
**C14**	172,01				13, 15			
**N15**	116,82			15				
**H15**	7,972	d (o.l.) 7.7(16)		15	14, 16, 57	16	12, 13, 16, 18, 54, 57a, 57b, 58	16, 57a, 57b, 58
**C16**	52,30		16		15, 57a, 57b, 58			
**H16**	4,178	d 8.3(57a/b), d 7.7(15), d 5.5(57a/b)	16		17, 57, 58	15, 57a, 57b	15, 18, 57a, 58	15, 57a, 57b, 58
**C17**	171,13				16, 18, 57b			
**N18**	115,19			18				
**H18**	7,890	d 7.9(19)		18	17, 19	19	15, 16, 19, 21, 61	19, 61, 62
**C19**	54,92		19		18, 61			
**H19**	4,311	d 7.9(18), d 7.5(61a/b), d 5.3(61a/b)	19		20, 61	18, 61	18, 21, 61	18, 61, 62
**C20**	169,33				19, 21, 61			
**N21**	117,22			21				
**H21**	8,020	d 8.2(22)		21	20	22	18, 19, 22, 61, 63b, 65	22, 63a, 63b
**C22**	53,69		22		63a, 63b			
**H22**	4,511	m (o.l.)	22		23, 63, 64	21, 63a, 63b	21, 24, 63b, 65	21, 63a, 63b
**C23**	170,18				22, 24, 63a, 63b			
**N24**	120,89			24				
**H24**	8,126	d 7.4(25)		24	23, 25, 68	25, 68	22, 25, 63a, 68	25, 68

**C25**	46,12		25		24, 68			
**H25**	4,476	quint 7.1(24, 68)	25		26, 68	24, 68	24, 28a, 28b	24, 68
**C26**	169,65				25, 68			
**N27**	n.f.							
**C28**	46,55		28a, 28b		29a, 29b, 30a, 30b			
**H28a**	3,546	d 10.0(28b/29), t 6.8(28b/29)	28			28b, 29a, 29b	25, 29a, 29b	31
**H28b**	3,411	d 10.0(28a/29), t 6.9(28a/29)	28			28a, 29a, 29b	25, 29a, 29b, 68	31
**C29**	24,48		29a, 29b		31			
**H29a**	1,850	m (o.l.)	29		28, 30, 31	28a, 28b	28a, 28b	30b, 31
**H29b**	1,758	m (o.l.)	29		28, 30, 31	28a, 28b	28a, 28b	30b, 31
**C30**	27,74		30a, 30b		29a, 29b, 31			
**H30a**	1,991	m	30		28, 32	30b, 31	30b, 31	31
**H30b**	1,571	m (o.l.)	30		28, 32	30a, 31	30a, 31, 34a, 34b	29a, 29b, 31
**C31**	57,45		31		29a, 29b			
**H31**	4,465	m (o.l.)	31		29, 30	30a, 30b	30a, 30b, 34a, 34b	28a, 28b, 29a, 29b, 30a, 30b
**C32**	170,29				30a, 30b			
**N33**	n.f.							
**C34**	46,59		34a, 34b					
**H34a**	3,596	d 10.0(34b/35), t 7.2(34b/35)	34			34b, 35	30b, 31, 35	37
**H34b**	3,442	(br) m	34			34a, 35	30b, 31, 35	37
**C35**	24,29		35					
**H35**	1,816	m (o.l.)	35			34a, 34b	34a, 34b	
**C36**	28,62		36a, 36b		37			
**H36a**	1,945	m	36		38	36b, 37	37	36b, 37
**H36b**	1,821	m (o.l.)	36			36a, 37	37, 39	36a, 37

**C37**	59,38		37					
**H37**	4,294	m (o.l.)	37		36	36a, 36b	36a, 36b, 39	34a, 34b, 36a, 36b
**C38**	171,33				36a, 39			
**N39**	115,45			39				
**H39**	7,759	d 7.6(40)		39	38, 40, 69	40	36b, 37, 40, 42, 69b, 71	40, 69a, 69b, 71
**C40**	53,44		40		39, 69a, 69b			
**H40**	4,503	m (o.l.)	40		41, 69, 70	39, 69a, 69b	39, 42, 71	39, 69a, 69b, 71
**C41**	170,89				40, 42, 43, 69a, 69b			
**N42**	115,08			42				
**H42**	7,630	d 9.0(43)		42	41, 43	43	39, 40, 45a, 81, 82a, 82b, 84	43, 81, 83, 84
**C43**	56,72		43		42, 45b, 84			
**H43**	4,125	d 9.0(42), d 6.9(81)	43		41, 44, 81, 82, 84	42, 81	45a, 81, 82a, 82b, 84	42, 81, 82a, 82b, 83, 84
**C44**	172,73				43, 45a, 45b			
**N45**	106,58			45a, 45b				
**H45a**	7,268	(br) d 2.2(45b)		45	44	45b	42, 43, 45b, 81, 84	45b
**H45b**	7,043	(br) s		45	43, 44	45a	45a	45a
**C46**	17,87		46		3, 4			
**H46**	1,182	d 7.1(4)	46		4, 5	3, 4	6	3
**C47**	25,92		47a, 47b		6, 7			
**H47a**	2,778	m (o.l.)	47		7, 8	7, 47b, 48	6	6, 7, 47b, 48
**H47b**	2,717	m (o.l.)	47		7, 8	7, 47a, 48	6, 7, 9	6, 7, 47a, 48
**SH48**	2,309	d 8.8(47a/b), d 8.2(47a/b)				47a, 47b		6, 7, 47a, 47b
**C49**	40,38		49		10, 51, 52			
**H49**	1,463	m (o.l.)	49		10, 11	10	9	9, 10, 50, 51, 52
**C50**	24,09		50		51, 52			
**H50**	1,593	m (o.l.)	50		10, 51, 52	51, 52	10	9, 10, 49, 51, 52
**C51**	23,06		51		50, 52			

**H51**	0,871	d 6.6(50)	51		49, 50, 52	50	10	9, 10, 49, 50, 52
**C52**	21,49		52		50, 51			
**H52**	0,825	d 6.6(50)	52		49, 50, 51	50	10	9, 10, 49, 50, 51
**C53**	40,55		53		13, 55, 56			
**H53**	1,446	m (o.l.)	53		13	13	12, 55, 56	12, 13, 54, 55, 56
**C54**	24,04		54		55, 56			
**H54**	1,602	m (o.l.)	54		55, 56	55, 56	12, 13, 15, 55, 56	12, 13, 53, 55, 56
**C55**	23,09		55		54, 56			
**H55**	0,858	d 6.6(54)	55		53, 54, 56	54	13, 53, 54	12, 13, 53, 54, 56
**C56**	21,48		56		54, 55			
**H56**	0,809	d 6.6(54)	56		53, 54, 55	54	13, 53, 54	12, 13, 53, 54, 55
**C57**	27,58		57a, 57b		15, 16, 58			
**H57a**	1,840	m (o.l.)	57		16, 58, 59	16, 57b, 58	15, 16	15, 16, 57b, 58
**H57b**	1,736	m (o.l.)	57		16, 17, 58, 59	16, 57a, 58	15	15, 16, 57a, 58
**C58**	31,43		58		16, 57a, 57b, 60b			
**H58**	2,066	(br) t 7.8(57)	58		16, 57, 59	57a, 57b	15, 16, 60b	15, 16, 57a, 57b
**C59**	173,80				57a, 57b, 58, 60a, 60b			
**N60**	107,86			60a, 60b				
**H60a**	7,206	m (o.l.)		60	59	60b		60b
**H60b**	6,784	(br) d 1.9(60a)		60	58, 59	60a	58	60a
**C61**	26,23		61		19			
**H61**	2,657	m	61		19, 20	19, 62	18, 19, 21	18, 19, 62
**SH62**	2,226	d 9.3(61a/b), d 7.9(61a/b)				61		18, 19, 61
**C63**	37,31		63a, 63b		22, 65			
**H63a**	3,000	d 14.2(63b), d 4.1(22)	63		22, 23, 64, 65	22, 63b	24, 65	21, 22, 63b

**H63b**	2,755	d 14.2(63a), d 9.6(22)	63		22, 23, 64, 65	22, 63a	21, 22, 65	21, 22, 63a
**C64**	137,53				22, 63a, 63b, 66			
**C65**	129,17		65		63a, 63b, 67			
**H65**	7,220	m (o.l.)	65		63, 67		21, 22, 63a, 63b	
**C66**	128,02		66					
**H66**	7,225	m (o.l.)	66		64	67		67
**C67**	126,28		67		65			
**H67**	7,166	m	67		65	66		66
**C68**	16,79		68		24, 25			
**H68**	1,156	d 6.9(25)	68		25, 26	24, 25	24, 28b	24, 25
**C69**	26,93		69a, 69b		39, 40			
**H69a**	3,144	d 14.9(69b), d 5.2(40)	69		40, 41, 70, 71, 80	40, 69b, 71	71, 77	39, 40, 71
**H69b**	3,010	d 14.9(69a), d 7.4(40)	69		40, 41, 70, 71, 80	40, 69a, 71	39, 71	39, 40, 71
**C70**	109,72				40, 69a, 69b, 71, 72, 77			
**C71**	123,48		71		69a, 69b, 72			
**H71**	7,112	d 2.4(72)	71		70, 73, 80	69a, 69b, 72	39, 40, 69a, 69b, 72	39, 40, 69a, 69b, 72
**N72**	130,78			72				
**H72**	10,816	d 2.4(71)		72	70, 71, 73, 80	71, 77	71	71, 77
**C73**	135,98				71, 72, 75, 77			
**C74**	111,21		74		76			
**H74**	7,312	d 8.1(75), t 1.0(76, 77)	74		76, 80	75		75, 77
**C75**	120,83		75		77			
**H75**	7,049	d 8.1(74), d 7.0(76), d 1.2(77)	75		73, 77	74, 76, 77		74, 76
**C76**	118,22		76		74			
**H76**	6,959	d 7.9(77), d 7.0(75), d 1.0(74)	76		74, 80	75, 77		75, 77
**C77**	118,26		77		75			
**H77**	7,519	d 7.9(76), q 1.0(74, 75, 72)	77		70, 73, 75, 80	72, 75, 76	69a	72, 74, 76

**C80**	127,45				69a, 69b, 71, 72, 74, 76, 77			
**C81**	36,77		81		43, 82b, 83			
**H81**	1,675	d 9.0(82b), quint 6.8(43, 84), d 3.6(82a)	81			43, 82a, 82b, 84	42, 43, 45a, 82a, 84	42, 43, 82a, 82b, 83, 84
**C82**	24,14		82a, 82b		43			
**H82a**	1,378	d 14.0(82b), q 7.4(83), d 3.6(81)	82			81, 82b, 83	42, 43, 81, 82b, 83	43, 81, 83, 84
**H82b**	1,047	d 14.0(82a), q 7.4(83), d 9.0(81)	82		81	81, 82a, 83	42, 43, 82a	43, 81, 83, 84
**C83**	11,10		83					
**H83**	0,800	t 7.4(82a,b)	83		81	82a, 82b	82a	42, 43, 81, 82a, 82b
**C84**	15,33		84		43			
**H84**	0,800	d 6.8(81)	84		43	81	42, 43, 45a, 81	42, 43, 81, 82a, 82b

^{1}H NMR of Ac-Ala-Cys-Leu-Leu-Gln-Cys-Phe-Ala-Pro-Pro-Trp-Ile-NH_2 **(S11):**
600 MHz, $(CD_3)_2SO$, 298 K

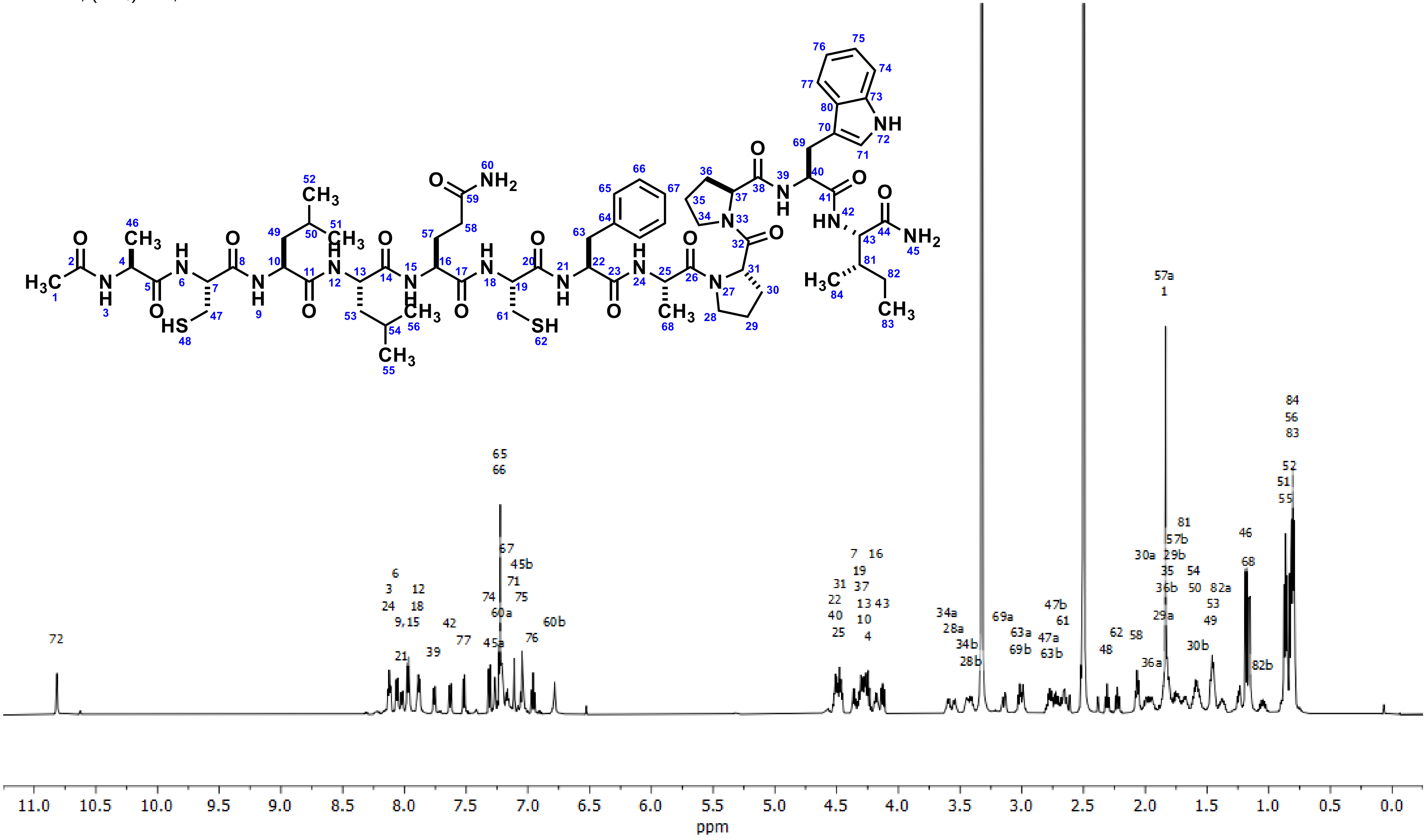

^{13}C NMR of Ac-Ala-Cys-Leu-Leu-Gln-Cys-Phe-Ala-Pro-Pro-Trp-Ile-NH_2 **(S11):**
151 MHz, $(CD_3)_2SO$, 298 K

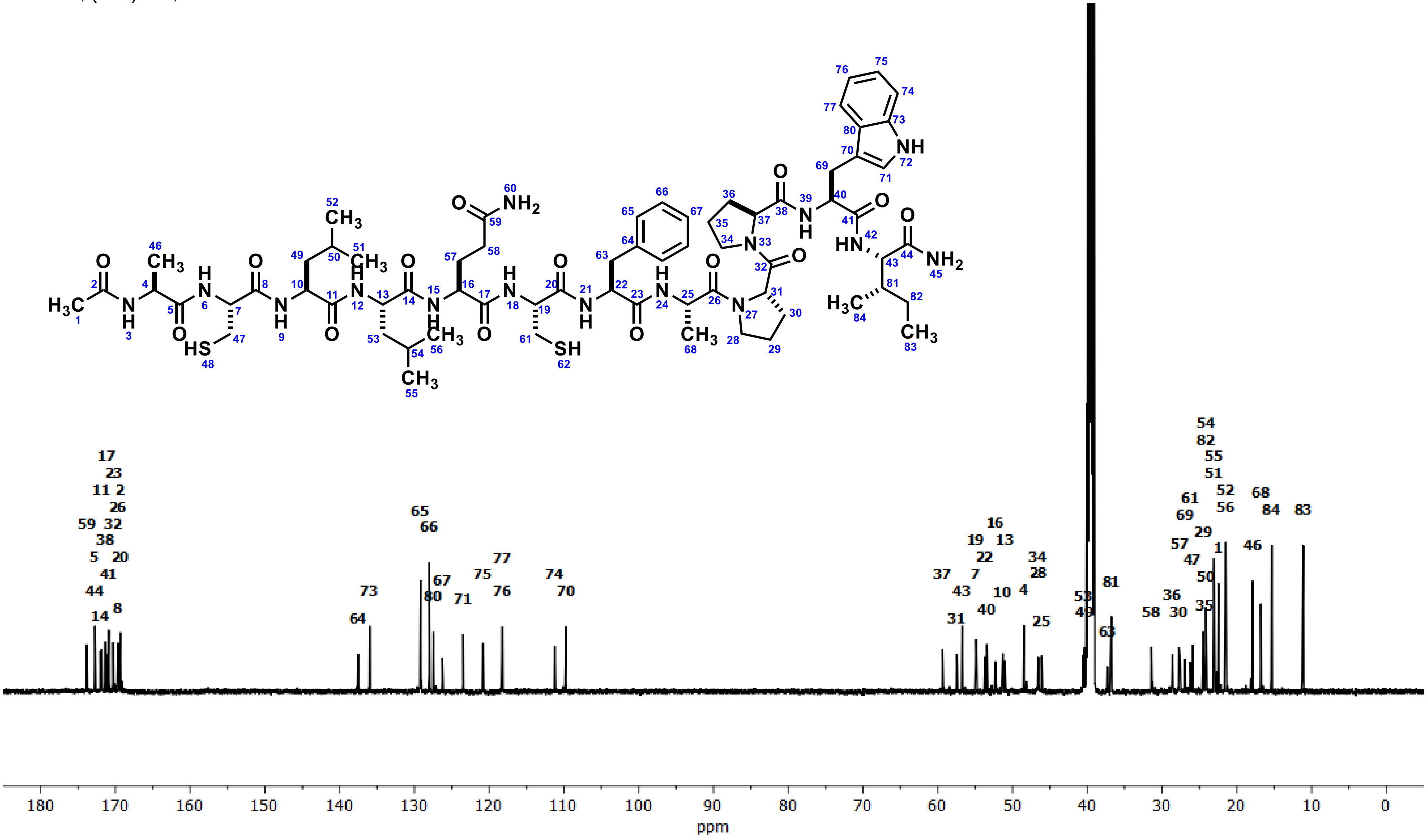

COSY-NMR of Ac-Ala-Cys-Leu-Leu-Gln-Cys-Phe-Ala-Pro-Pro-Trp-Ile-NH_2 **(S11):**
$(CD_3)_2SO$, 298 K

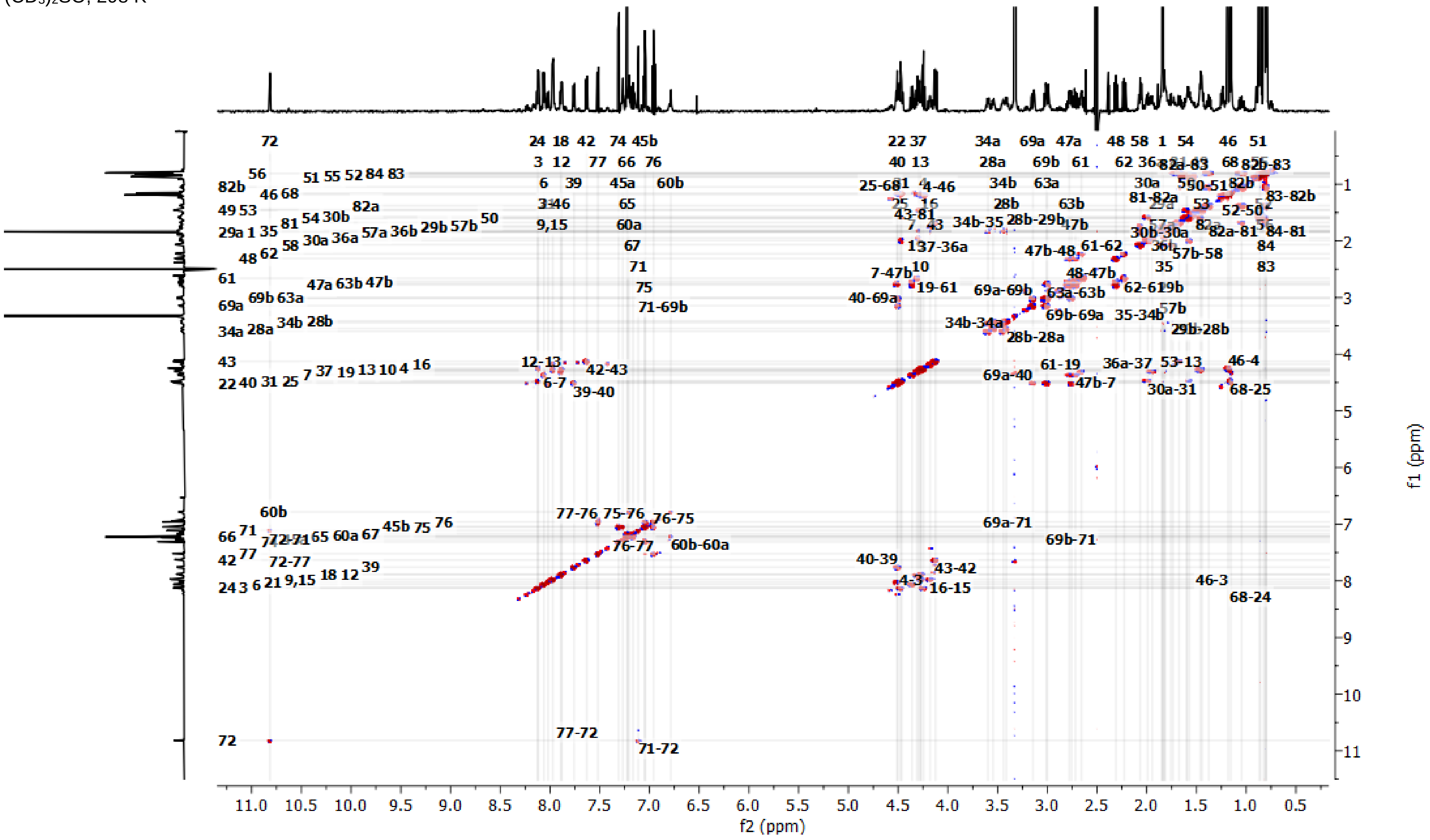

HSQC-NMR of Ac-Ala-Cys-Leu-Leu-Gln-Cys-Phe-Ala-Pro-Pro-Trp-Ile-NH_2 **(S11):**
$(CD_3)_2SO$, 298 K

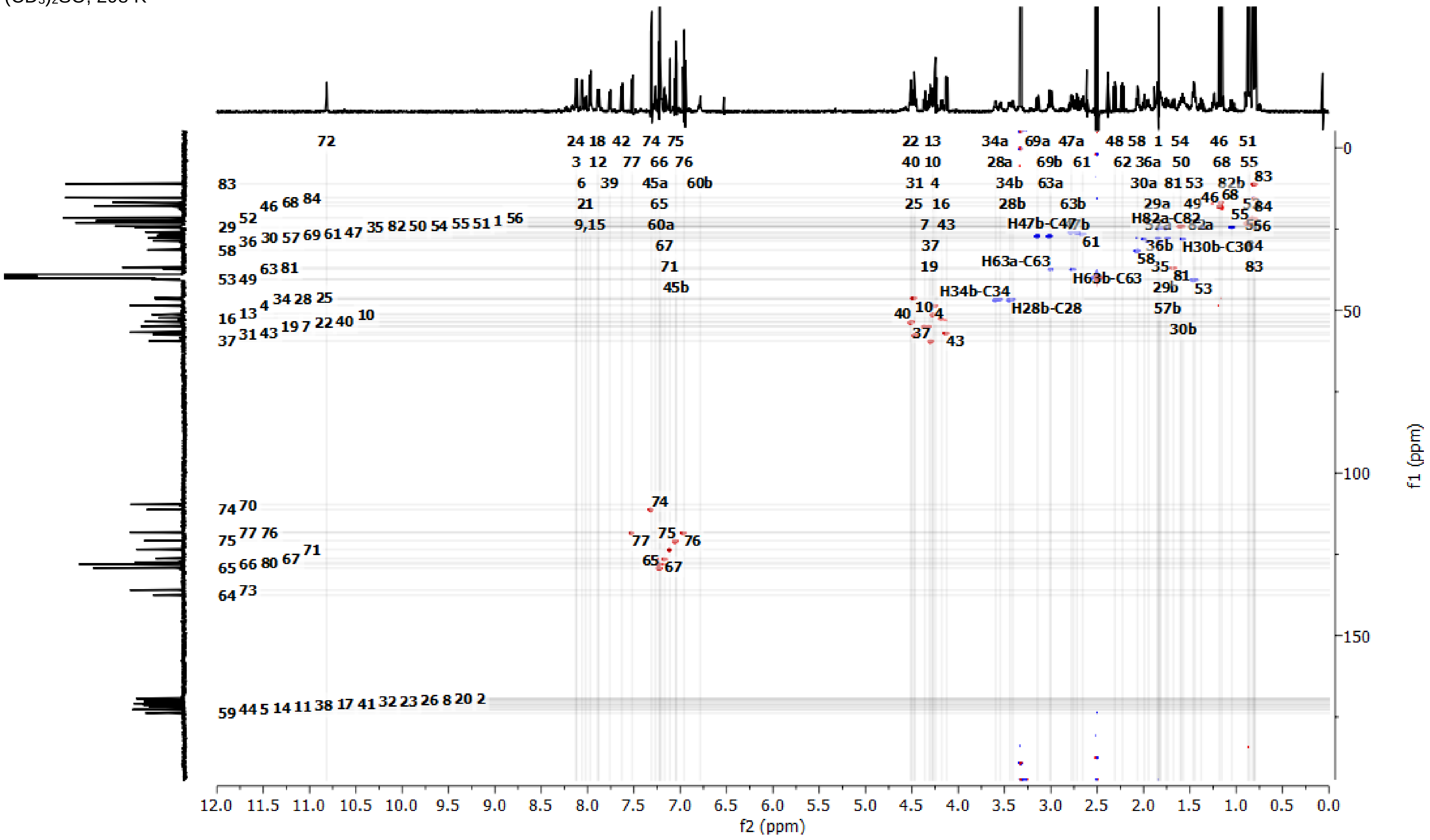

HMQC-NMR of Ac-Ala-Cys-Leu-Leu-Gln-Cys-Phe-Ala-Pro-Pro-Trp-Ile-NH_2 **(S11):**
$(CD_3)_2SO$, 298 K

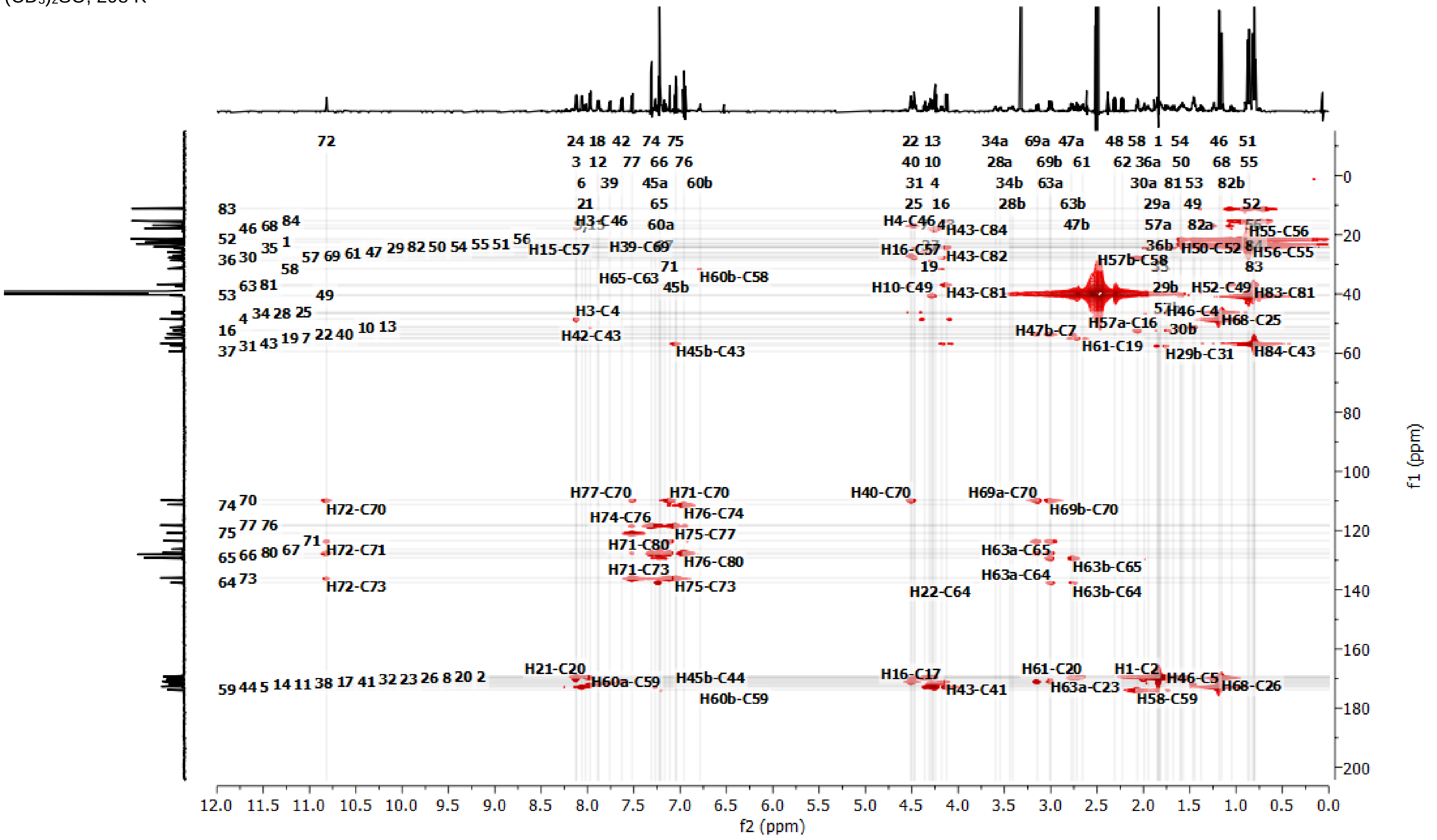

NOESY-NMR of Ac-Ala-Cys-Leu-Leu-Gln-Cys-Phe-Ala-Pro-Pro-Trp-Ile-NH2 **(S11):**
$(CD_3)_2SO$, 298 K

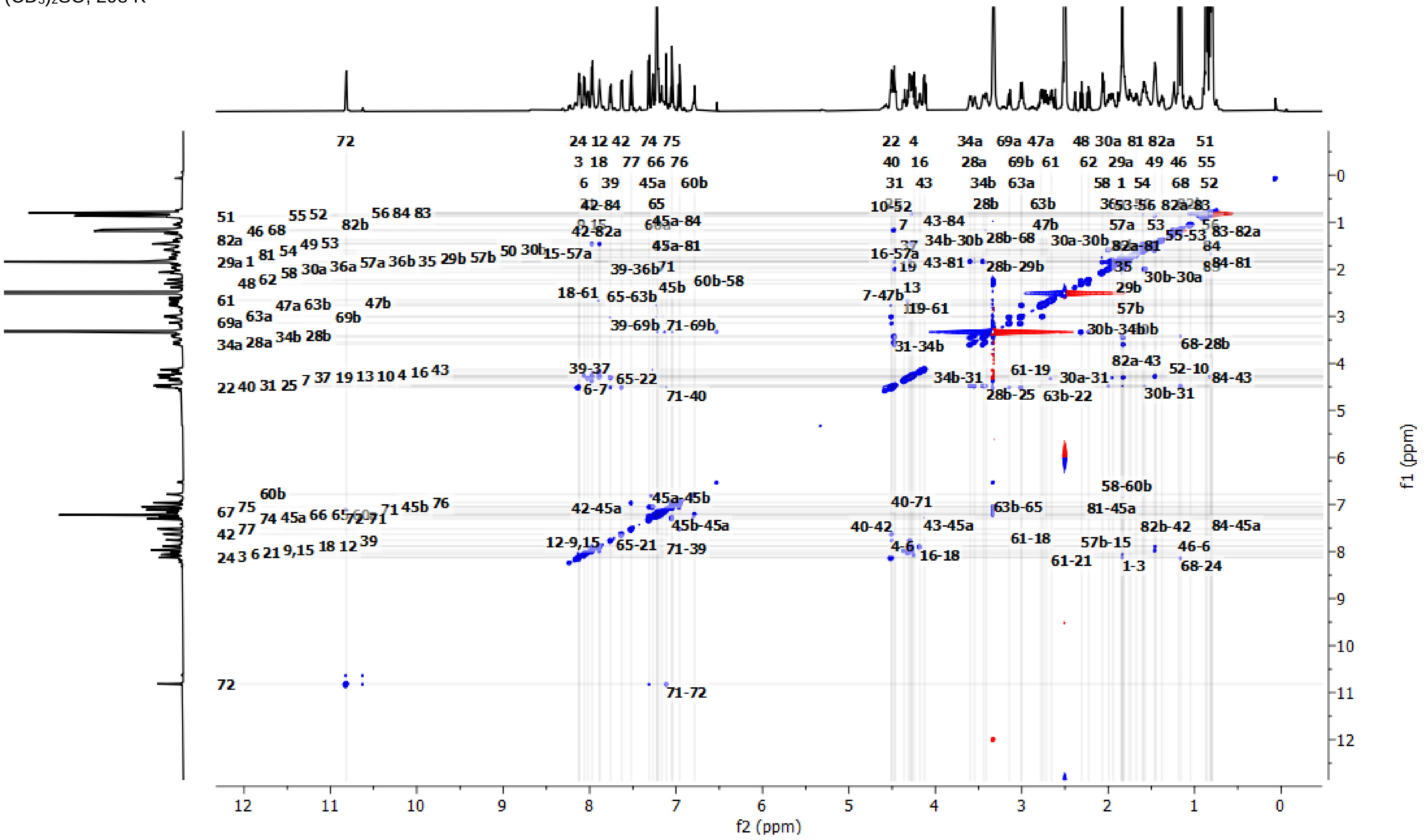

ROESY-NMR of Ac-Ala-Cys-Leu-Leu-Gln-Cys-Phe-Ala-Pro-Pro-Trp-Ile-NH2 **(S11):**
$(CD_3)_2SO$, 298 K

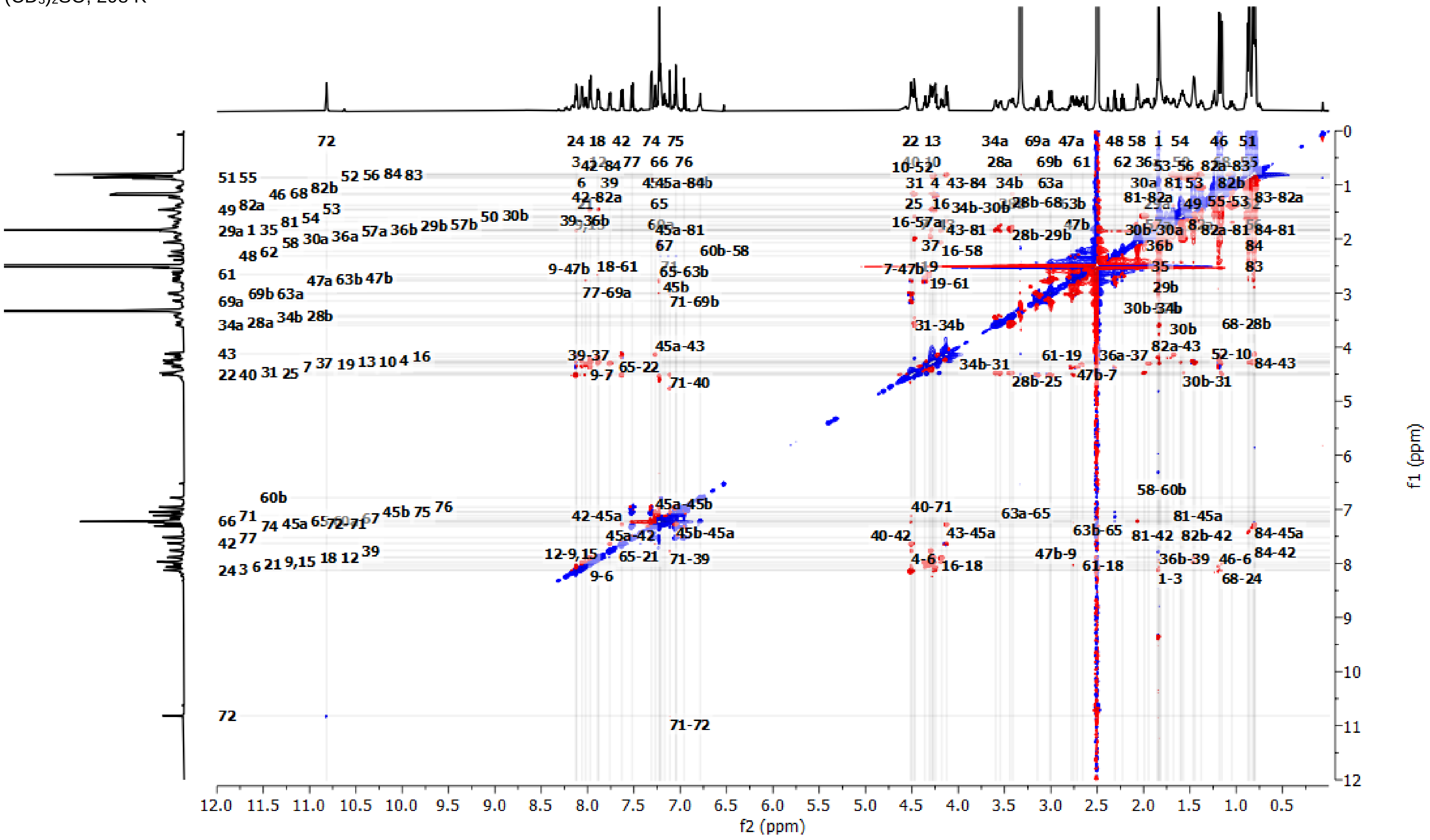

TOCSY-NMR of Ac-Ala-Cys-Leu-Leu-Gln-Cys-Phe-Ala-Pro-Pro-Trp-Ile-NH_2 **(S11):**
$(CD_3)_2SO$, 298 K

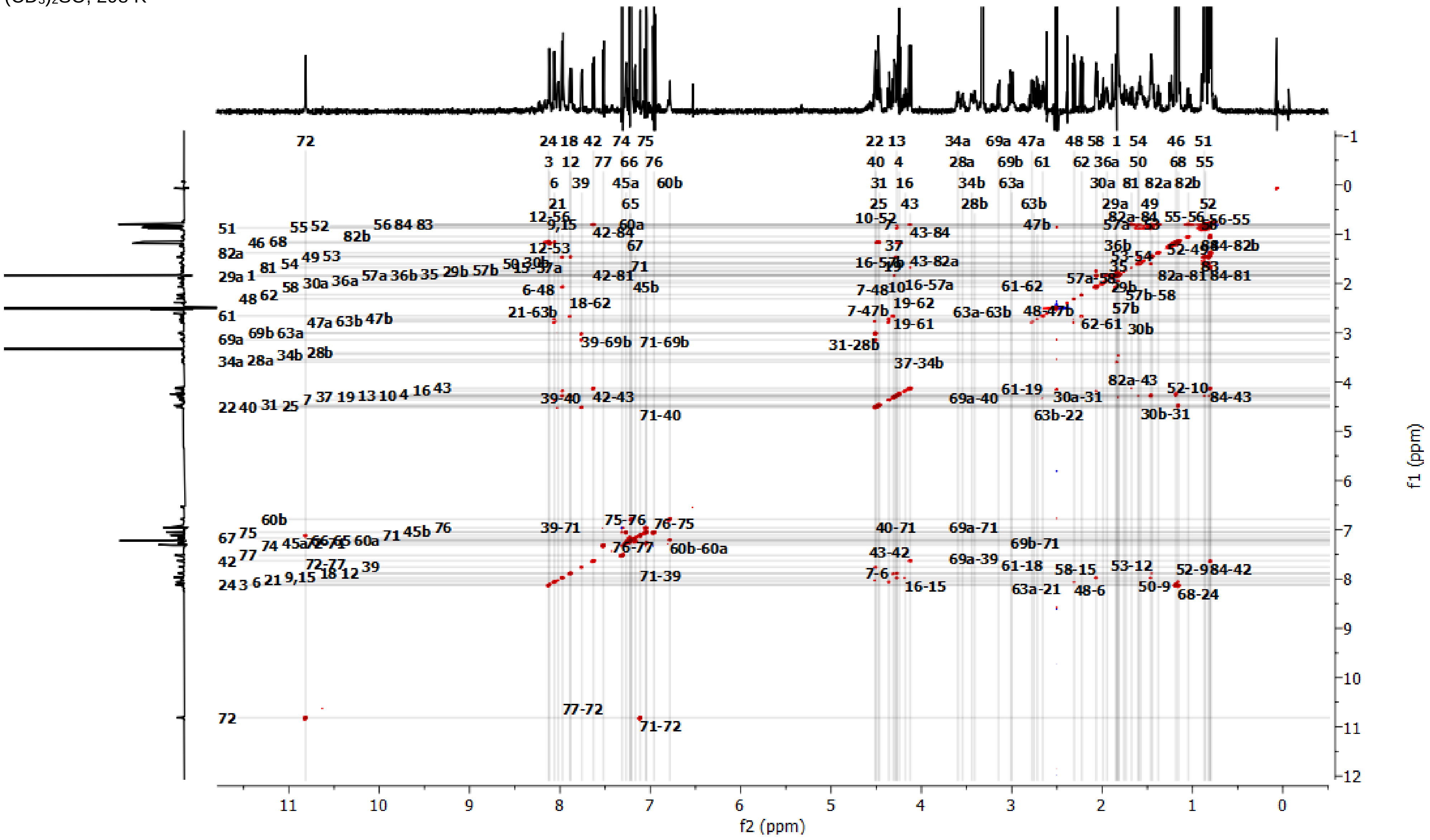

^{15}N-HSQC-NMR of Ac-Ala-Cys-Leu-Leu-Gln-Cys-Phe-Ala-Pro-Pro-Trp-Ile-NH$_2$ **(S11):**
$(CD_3)_2SO$, 298 K

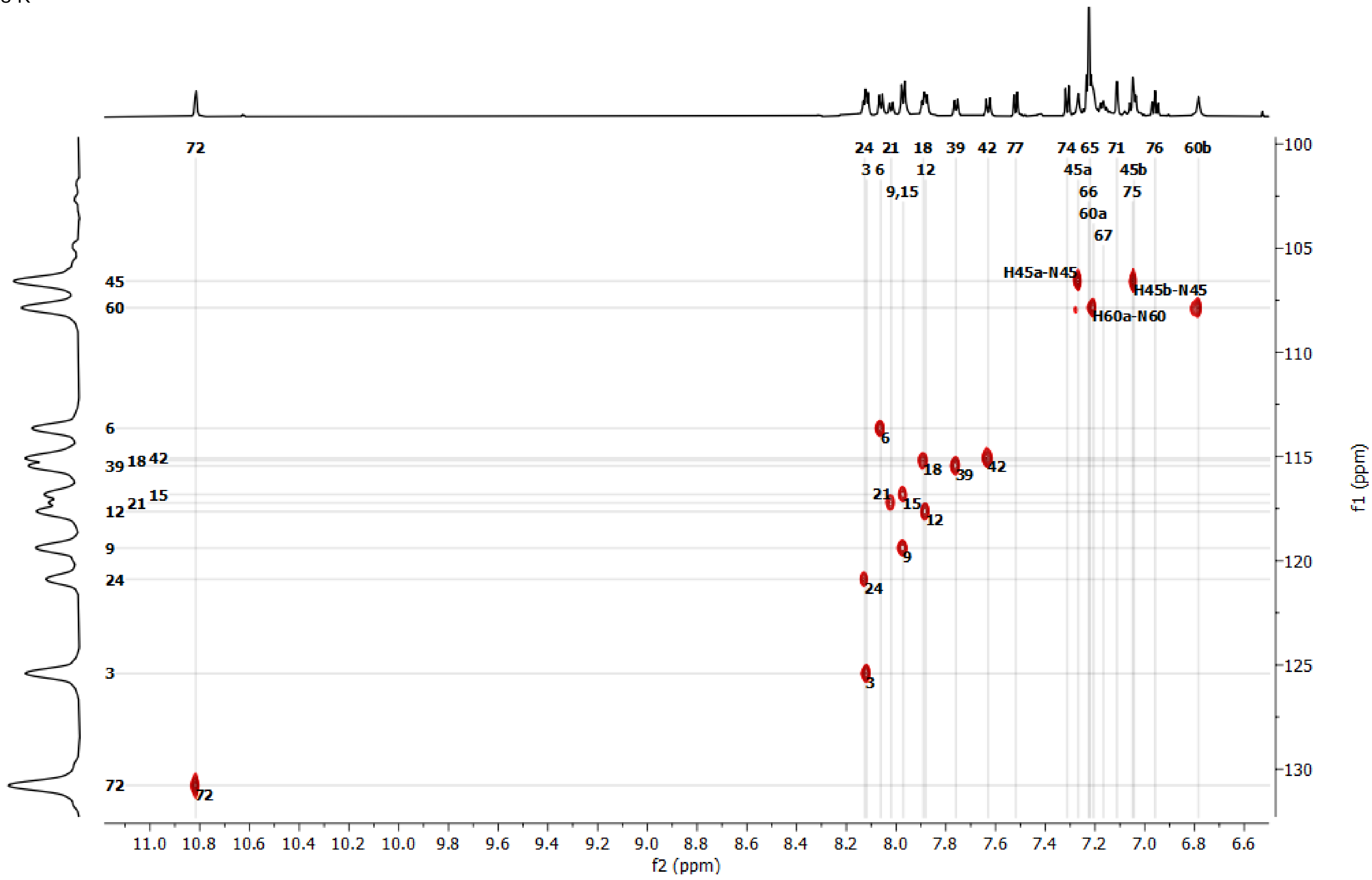

Ac-Ala-Cys-Leu-Leu-Gln-Lys-Phe-Ala-Pro-Pro-Trp-Ile-NH_2 (S12):

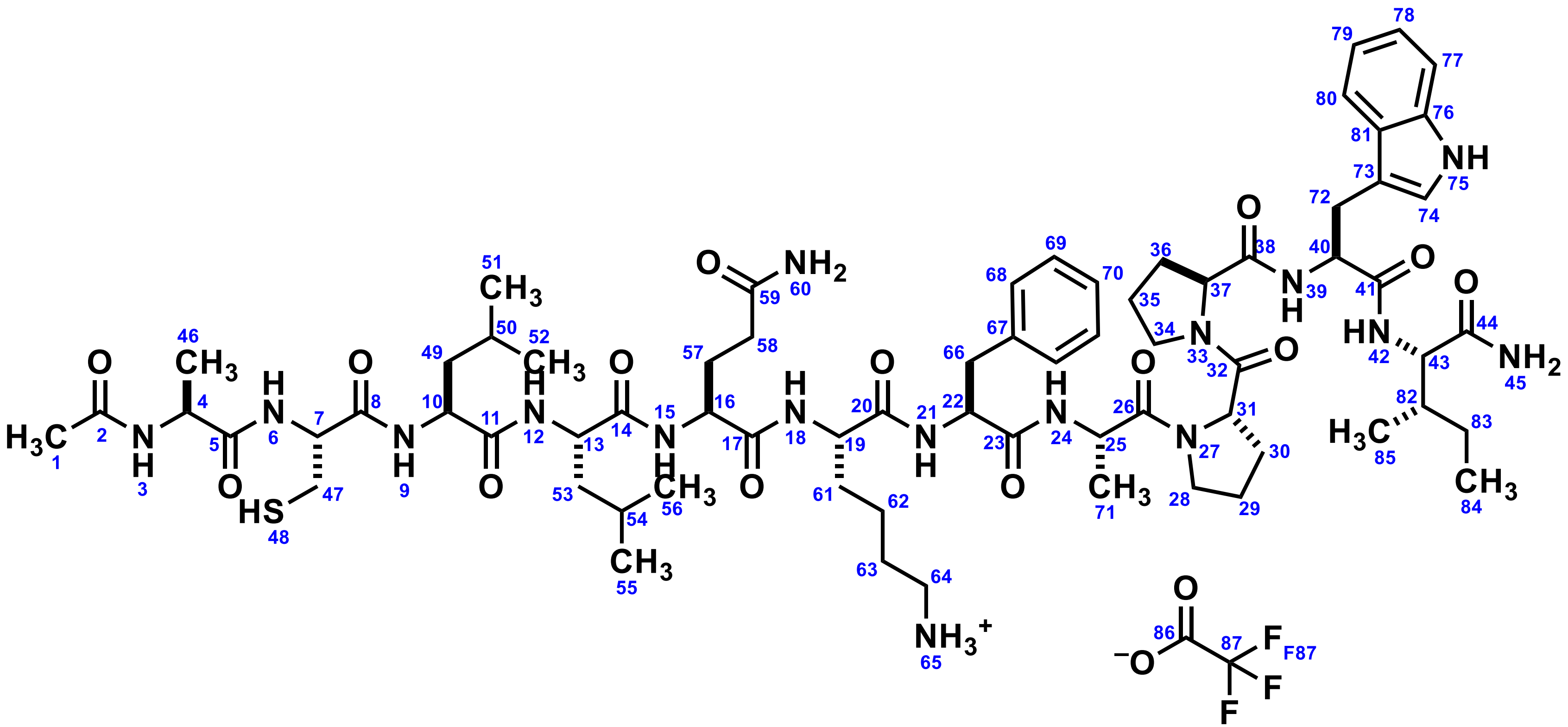

Table S30. Characterization table of Ac-Ala-Cys-Leu-Leu-Gln-Lys-Phe-Ala-Pro-Pro-Trp-Ile-NH_2. The ^{15}N chemical shifts were referenced to CH_3NO_2.

Atom	δ (ppm)	J	HSQC	^{15}N-HSQC	HMQC	COSY	ROESY	TOCSY
C1	22,44		1					
H1	1,841	s	1		2		3	
C2	169,47				1, 3, 4			
N3	-254,14			3				
H3	8,141	d 6.8(4)		3	2, 4, 46	4	1, 6, 46	4, 46
C4	48,62		4		3, 46			
H4	4,227	quint 7.1(4, 46)	4		2, 5, 46	3, 46	6	3, 46

**C5**	172,84				4, 6, 7, 46			
**N6**	-266,13			6				
**H6**	8,073	d 7.8(7)		6	5, 7, 47	7	3, 4, 9, 46, 47a, 47b, 48	7, 47a, 47b, 48
**C7**	54,95		7		6, 47a, 47b, 48			
**H7**	4,356	t 7.5(6, 47b), d 5.2(47a)	7		5, 8, 47	6, 47a, 47b	9	6, 47a, 47b, 48
**C8**	169,67				7, 9, 10, 47a, 47b			
**N9**	-260,33			9				
**H9**	7,963	d 7.9(10)		9	8, 10, 49	10	6, 7, 12, 47a, 47b, 49, 50	10, 49, 50, 51, 52
**C10**	51,35		10		9, 49, 50			
**H10**	4,256	m (o.l.)	10		8, 11, 49	9, 49	12, 50, 51, 52	9, 49, 50
**C11**	171,84				10, 12, 13, 49			
**N12**	-262,06			12				
**H12**	7,841	d 8.0(13)		12	11, 13, 53	13	9, 10, 13, 53, 54	13, 53, 54, 55, 56
**C13**	51,02		13		12, 53			
**H13**	4,263	m (o.l.)	13		11, 14, 53	12, 53	12, 15, 54, 55, 56	12, 53, 54
**C14**	171,97				13, 15, 53			
**N15**	-262,67			15				
**H15**	7,886	d 7.5(16)		15	14, 16	16	13, 16, 53, 57a, 57b, 58	16, 57a, 57b, 58
**C16**	52,27		16		15, 57a, 57b, 58			
**H16**	4,152	m (o.l.)	16		17, 57, 58	15, 57a, 57b	15, 18, 57a, 57b, 58	15, 57a, 57b, 58
**C17**	170,97				16, 18, 57b			
**N18**	-262,58			18				
**H18**	7,842	d 7.8(19)		18	17	19	16, 19, 58, 61a, 62	19, 61a, 61b, 62, 64, 65
**C19**	52,33		19		61b			
**H19**	4,142	m (o.l.)	19		20, 61, 62	18, 61a, 61b	18, 21, 61a, 61b, 62, 63	18, 61a, 61b, 62, 63, 64, 65
**C20**	171,07				19, 21, 22, 61b			
**N21**	-264,13			21				
**H21**	7,855	d 8.1(22)		21	20, 22	22	19, 24, 66a, 66b, 68	22, 66a, 66b

**C22**	53,25		22		21, 66a, 66b			
**H22**	4,535	d 9.2(66b), d 8.2(21), d 4.5(66a)	22		20, 23, 66, 67	21, 66a, 66b	24, 68	21, 66a, 66b
**C23**	170,27				22, 24, 66a, 66b			
**N24**	-258,35			24				
**H24**	8,155	d 7.3(25)		24	23, 25, 71	25	21, 22, 66a, 66b, 71	25, 71
**C25**	46,09		25		24, 71			
**H25**	4,462	quint 7.1(24, 71)	25		26, 71	24, 71	28a, 28b	24, 71
**C26**	169,62				25, 71			
**N27**	n. f.							
**C28**	46,53		28a, 28b		29a, 29b, 30a, 30b, 31			
**H28a**	3,523	d 9.6(28b), t 6.8(29a, b)	28			28b, 29a, 29b	25, 28b, 29a, 30a	28b, 29a, 29b, 30a, 30b, 31
**H28b**	3,407	d 9.6(28a), t 6.7(29a, b)	28			28a, 29a, 29b	25, 28a, 71	28a, 29a, 29b, 30a, 30b, 31
**C29**	24,46		29a, 29b		30a, 30b, 31			
**H29a**	1,841	m (o.l.)	29		28, 31	28a, 28b, 29b	28a, 31	28a, 28b, 30a, 30b, 31
**H29b**	1,747	m (o.l.)	29		28, 30, 31	28a, 28b, 29a	31	28a, 28b, 30a, 30b, 31
**C30**	27,73		30a, 30b		29b, 31			
**H30a**	1,996	m	30		28, 29, 31, 32	30b, 31	28a, 30b, 31	28a, 28b, 29a, 29b, 30b, 31
**H30b**	1,584	m (o.l.)	30		28, 29, 31, 32	30a, 31	30a, 31	28a, 28b, 29a, 29b, 30a, 31
**C31**	57,44		31		29a, 29b, 30a, 30b			
**H31**	4,468	d 8.5(30a), d 4.7(30b)	31		28, 29, 30, 32	30a, 30b	29a, 29b, 30a, 30b, 34a, 34b	28a, 28b, 29a, 29b, 30a, 30b
**C32**	170,24				30a, 30b, 31			
**N33**	n. f.							
**C34**	46,57		34a, 34b		35, 36a, 36b			

**H34a**	3,588	d 10.2(34b), t 7.3(35)	34			34b, 35	31, 34b, 35, 36a, 36b	34b, 35, 36a, 36b, 37
**H34b**	3,448	m	34			34a, 35	31, 34a, 39	34a, 35, 36a, 36b, 37
**C35**	24,28		35		37			
**H35**	1,818	m (o.l.)	35		34, 36	34a, 34b	34a	34a, 34b, 36a, 37
**C36**	28,63		36a, 36b		35, 37			
**H36a**	1,951	m	36		34, 37, 38	36b, 37	34a	34a, 34b, 35, 36b, 37
**H36b**	1,822	m (o.l.)	36		34, 37	36a, 37	34a	34a, 34b, 36a, 37
**C37**	59,36		37		36a, 36b			
**H37**	4,294	d 8.4(36a), d 3.0(36b)	37		35, 36, 38	36a, 36b	39	34a, 34b, 35, 36a, 36b
**C38**	171,30				36a, 37, 39, 40			
**N39**	-264,22			39				
**H39**	7,757	d 7.6(40)		39	38, 40, 72	40	34b, 37, 42, 72a, 72b, 74	40, 72a, 72b, 74
**C40**	53,41		40		39, 72a, 72b			
**H40**	4,510	t 7.5(39, 72b), d 5.4(72a)	40		38, 41, 72, 73	39, 72a, 72b	42, 74, 80	39, 72a, 72b, 74
**C41**	170,87				40, 42, 43, 72a, 72b			
**N42**	-264,52			42				
**H42**	7,638	d 9.0(43)		42	41, 43	43	39, 40, 45a, 72a, 72b, 82, 83a, 83b, 85	43, 82, 83a, 83b, 84, 85
**C43**	56,69		43		42, 45b, 82, 83a, 83b, 85			
**H43**	4,128	d 9.0(42), d 6.9(82)	43		41, 44, 82, 83, 85	42, 82	45a, 82, 83a, 83b, 85	42, 82, 83a, 83b
**C44**	172,72				43, 45a, 45b, 82			
**N45**	-273,00			45a, 45b				
**H45a**	7,273	(br) d ~2(45b)		45	44		42, 43, 82	45b
**H45b**	7,045	(br) d ~2(45a)		45	43, 44			45a
**C46**	17,78		46		3, 4			

**H46**	1,189	d 7.1(4)	46		4, 5	4	3, 6	3, 4
**C47**	25,84		47a, 47b		6, 7, 48			
**H47a**	2,792	d 13.7(47b), d 9.0(48), d 5.3(7)	47		7, 8	7, 47b, 48	6, 9	6, 7, 47b, 48
**H47b**	2,718	d 13.7(47b), d 8.1(48), d 7.5(7)	47		7, 8	7, 47a, 48	6, 9	6, 7, 47a, 48
**SH48**	2,315	d 8.9(47a), d 8.1(47b)			7, 47	47a, 47b	6	6, 7, 47a, 47b
**C49**	40,29		49		9, 10, 51, 52			
**H49**	1,471	m (o.l.)	49		10, 11, 51, 52	10, 50	9	9, 10, 50, 51, 52
**C50**	24,09		50		51, 52			
**H50**	1,597	m (o.l.)	50		10, 51, 52	49, 51, 52	9, 10	9, 10, 49, 51, 52
**C51**	23,02		51		49, 50, 52			
**H51**	0,876	d 6.6(50)	51		49, 50, 52	50	10	9, 49, 50
**C52**	21,50		52		49, 50, 51			
**H52**	0,825	d 6.5(50)	52		49, 50, 51	50	10	9, 49, 50
**C53**	40,48		53		12, 13, 54, 55, 56			
**H53**	1,447	m (o.l.)	53		13, 14, 54, 55, 56	13, 54	12, 15	12, 13, 54, 55, 56
**C54**	24,03		54		53, 55, 56			
**H54**	1,600	m (o.l.)	54		53, 55, 56	53, 55, 56	12, 13	12, 13, 53, 55, 56
**C55**	23,10		55		53, 54, 56			
**H55**	0,865	d 6.5(54)	55		53, 54, 56	54	13	12, 53, 54
**C56**	21,46		56		53, 54, 55			
**H56**	0,811	d 6.6(54)	56		53, 54, 55	54	13	12, 53, 54
**C57**	27,64		57a, 57b		16, 58			
**H57a**	1,832	m (o.l.)	57		16, 58, 59	16, 57b, 58	15, 16	15, 16, 58
**H57b**	1,729	m (o.l.)	57		16, 17, 59	16, 57a, 58	15, 16	15, 16, 58
**C58**	31,44		58		16, 57a, 60b			
**H58**	2,070	m	58		16, 57, 59	57a, 57b	15, 16, 18, 60a, 60b	15, 16, 57a, 57b
**C59**	173,81				57a, 57b, 58, 60a, 60b			

**N60**	-271,82			60a, 60b				
**H60a**	7,210	m (o.l.)		60	59	60b	58	60b
**H60b**	6,800	(br) d ~2(60a)		60	58, 59	60a	58	60a
**C61**	31,29		61a, 61b		19, 63			
**H61a**	1,525	m (o.l.)	61			19, 61b, 62	18, 19, 61b	18, 19, 62, 64, 65
**H61b**	1,415	m (o.l.)	61		19, 20, 63	19, 61a, 62	19, 61a	18, 19, 62, 64, 65
**C62**	21,96		62		19, 64			
**H62**	1,192	m (o.l.)	62			61a, 61b, 63	18, 19, 64, 65	18, 19, 61a, 61b, 63, 64, 65
**C63**	26,54		63		61b, 64, 65			
**H63**	1,450	m (o.l.)	63		61, 64	62, 64	19, 65	19, 62, 64, 65
**C64**	38,71		64		63, 65			
**H64**	2,701	m (o.l.)	64		62, 63	63, 65	62, 65	18, 19, 61a, 61b, 62, 63, 65
**N65**	-347,11			65				
**H65**	7,613	br t ~6(64)		65	63, 64	64	62, 63, 64	18, 19, 61a, 61b, 62, 63, 64
**C66**	37,39		66a, 66b		22, 68			
**H66a**	2,997	d 14.0(66b), d 4.6(22)	66		22, 23, 67, 68	22, 66b	21, 24, 68	21, 22, 66b
**H66b**	2,745	d 14.0(66a), d 9.2(22)	66		22, 23, 67, 68	22, 66a	21, 24, 68	21, 22, 66a
**C67**	137,54				22, 66a, 66b, 69			
**C68**	129,18		68		66a, 66b, 70			
**H68**	7,218	m (o.l.)	68		66, 70		21, 22, 66a, 66b	70
**C69**	127,97		69					
**H69**	7,222	m (o.l.)	69		67	70		70
**C70**	126,22		70		68			
**H70**	7,167	m	70		68	69		68, 69
**C71**	16,78		71		24, 25			
**H71**	1,155	d 6.9(25)	71		25, 26	25	24, 28b	24, 25
**C72**	26,97		72a, 72b		39, 40			

**H72a**	3,146	d 15.0(72b), d 5.5(40)	72		40, 41, 73, 74, 81	40, 72b	39, 42, 74, 80	39, 40, 72b, 74, 75
**H72b**	3,005	d 15.1(72a), d 7.5(40)	72		40, 41, 73, 74, 81	40, 72a	39, 42, 74, 80	39, 40, 72a, 74, 75
**C73**	109,72				40, 72a, 72b, 74, 75, 80			
**C74**	123,47		74		72a, 72b, 75			
**H74**	7,110	d 2.4(75)	74		73, 76, 81	75	39, 40, 72a, 72b	39, 40, 72a, 72b, 75
**N75**	-248,91			75				
**H75**	10,814	d 2.4(74)		75	73, 74, 76, 81	74, 80	77	72a, 72b, 74, 79, 80
**C76**	135,98				74, 75, 80			
**C77**	111,21		77		79			
**H77**	7,312	d 8.1(78), t 0.9(79, 80)	77		79, 81	78	75	78, 79, 80
**C78**	120,83		78		80			
**H78**	7,050	d 8.1(77), d 6.9(79), d 1.2(80)	78		80	77, 79		77, 79, 80
**C79**	118,22		79		77			
**H79**	6,960	d 7.9(80), d 6.9(78), d 1.0(77)	79		77, 80, 81	78, 80		75, 77, 78, 80
**C80**	118,27		80		78, 79			
**H80**	7,522	d 7.9(79), d 1.1(78), t 0.8(75, 77)	80		73, 76, 78, 81	75, 79	40, 72a, 72b	75, 77, 78, 79
**C81**	127,45				72a, 72b, 74, 75, 77, 79, 80			
**C82**	36,78		82		43, 83a, 83b, 85			
**H82**	1,677	d 9.4(83b), quint 6.8(43, 85), d 3.7(83a)	82		43, 44, 84, 85	43, 83b, 85	42, 43, 45a, 83a	42, 43, 83a, 83b, 84, 85
**C83**	24,13		83a, 83b		43, 84			
**H83a**	1,380	d 13.7(83b), q 7.4(84), d 3.7(82)	83		43, 82, 84, 85	83b, 84	42, 43, 82	42, 43, 82, 84, 85
**H83b**	1,047	d 13.7(83a), d 9.4(82), q 7.4(84)	83		43, 82, 84, 85	82, 83a, 84	42, 43	42, 43, 82, 84, 85
**C84**	11,10		84		82, 83a, 83b			
**H84**	0,802	t 7.4(83a, b)	84		83	83a, 83b		42, 82, 83a, 83b
**C85**	15,33		85		43, 82, 83a, 83b			

**H85**	0,802	d 6.8(82)	85		43, 82	82	42, 43	42, 82, 83a, 83b
**C86**	157,75	q 32(F87)						
**C87**	n. f.							
**F87**	-73,55	s						

^{1}H NMR of Ac-Ala-Cys-Leu-Leu-Gln-Lys-Phe-Ala-Pro-Pro-Trp-Ile-NH2 **(S12):**
600 MHz, $(CD_3)_2SO$, 298 K

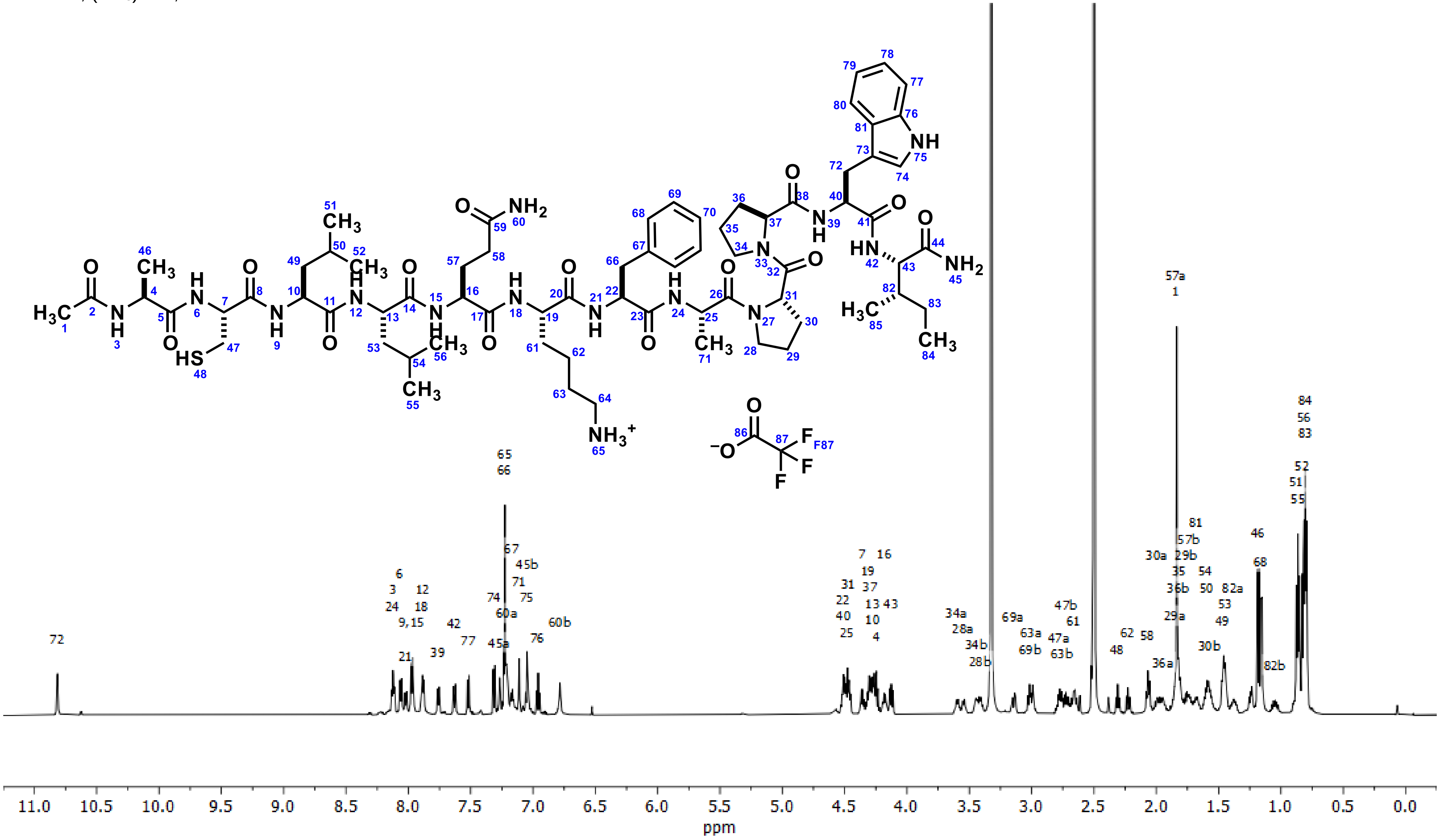

^{13}C NMR of Ac-Ala-Cys-Leu-Leu-Gln-Lys-Phe-Ala-Pro-Pro-Trp-Ile-NH_2 **(S12):**
151 MHz, $(CD_3)_2SO$, 298 K

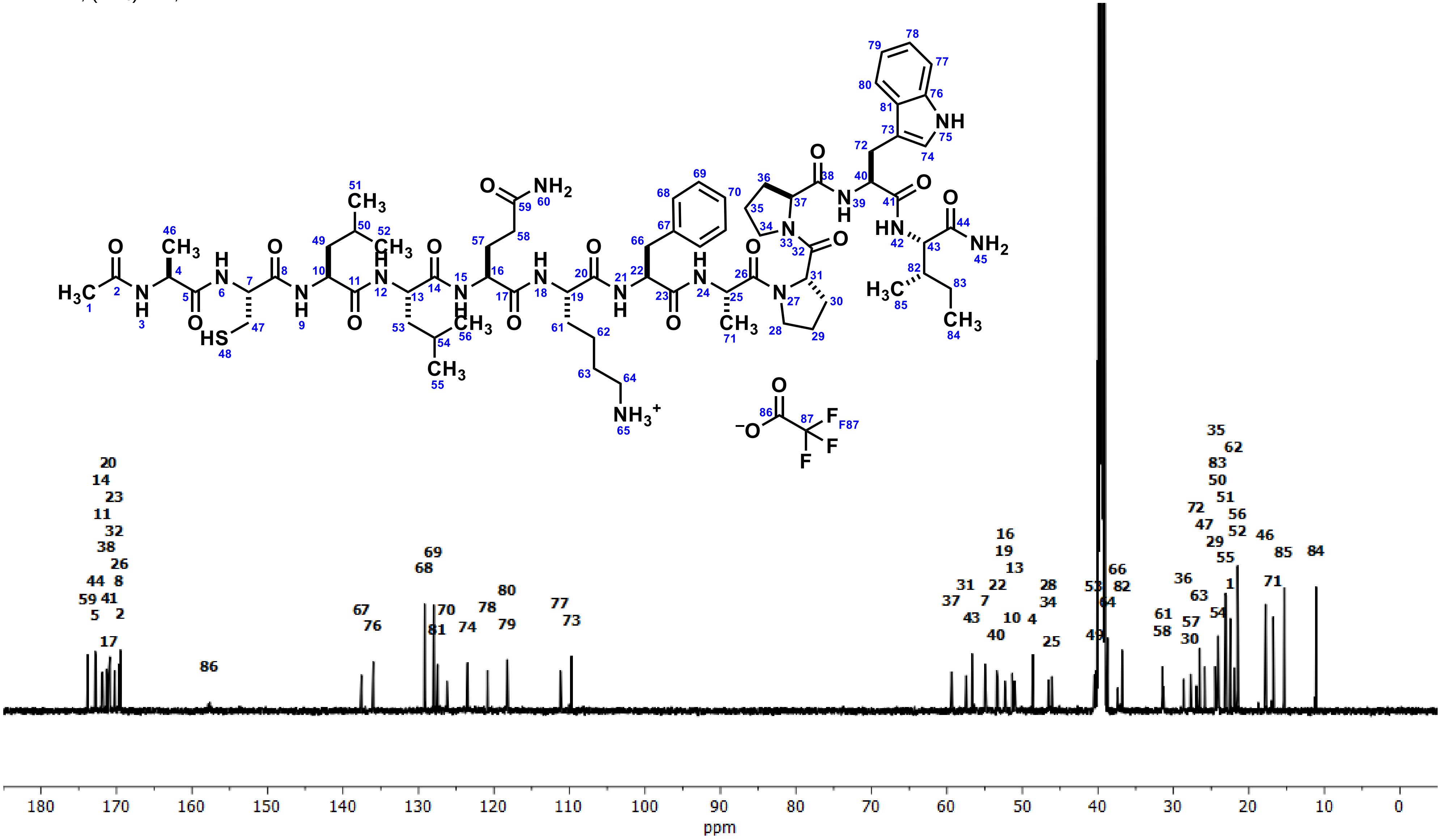

^{19}F NMR of Ac-Ala-Cys-Leu-Leu-Gln-Lys-Phe-Ala-Pro-Pro-Trp-Ile-NH$_2$ **(S12):**
470 MHz, $(CD_3)_2SO$, 298 K

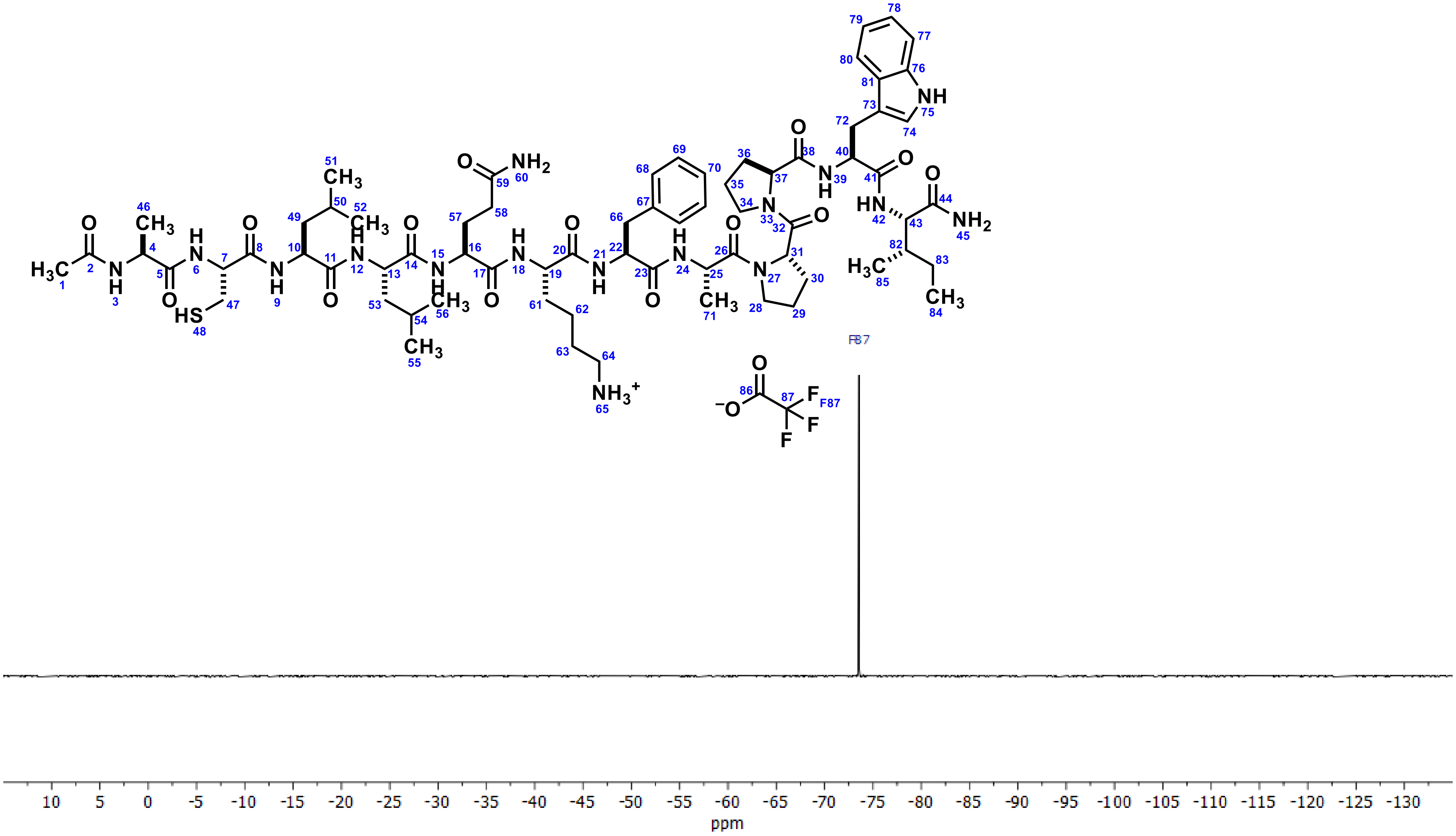

COSY-NMR of Ac-Ala-Cys-Leu-Leu-Gln-Lys-Phe-Ala-Pro-Pro-Trp-Ile-NH2 **(S12):**
$(CD_3)_2SO$, 298 K

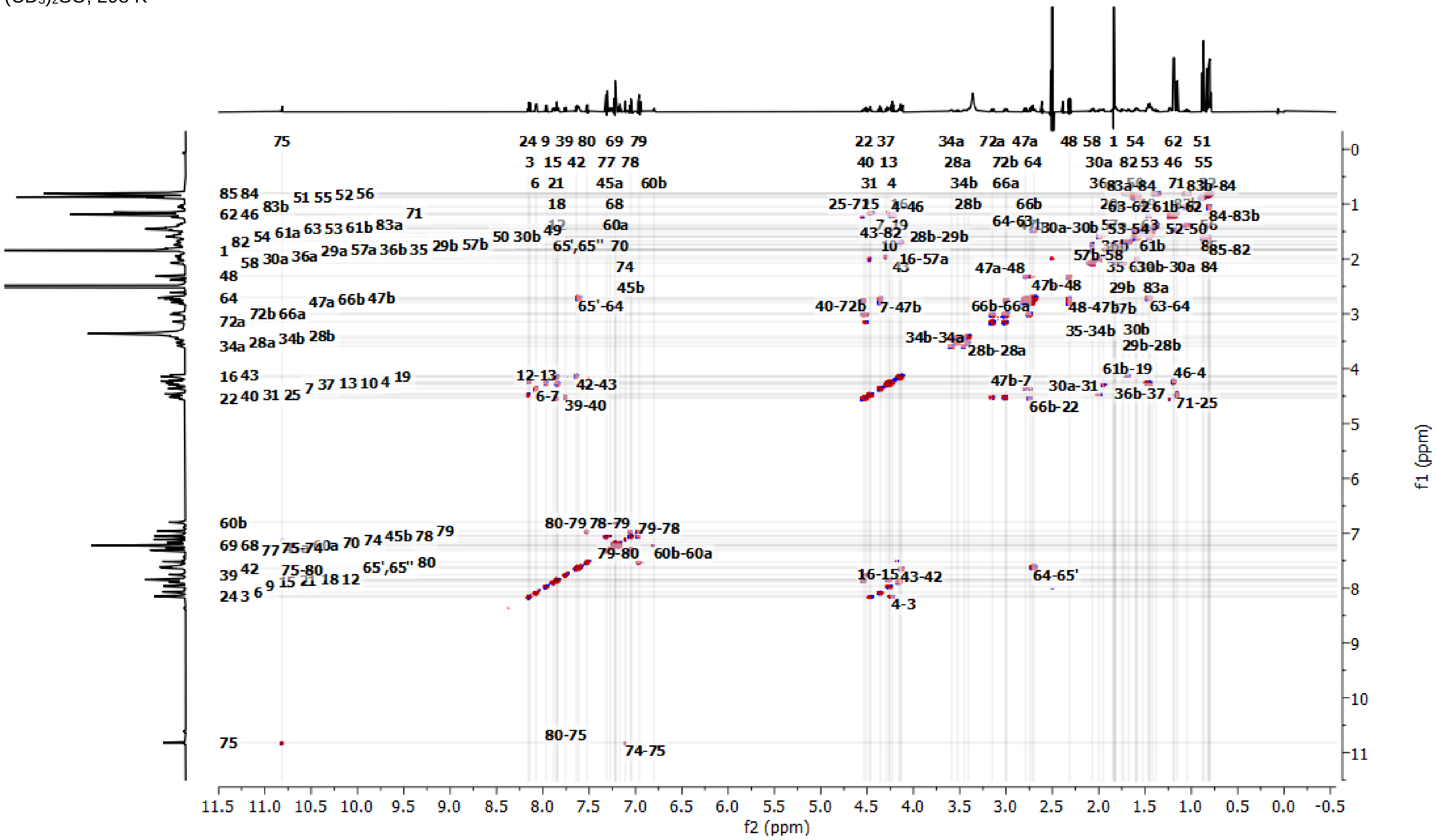

HMQC-NMR of Ac-Ala-Cys-Leu-Leu-Gln-Lys-Phe-Ala-Pro-Pro-Trp-Ile-NH₂ **(S12):**
$(CD_3)_2SO$, 298 K

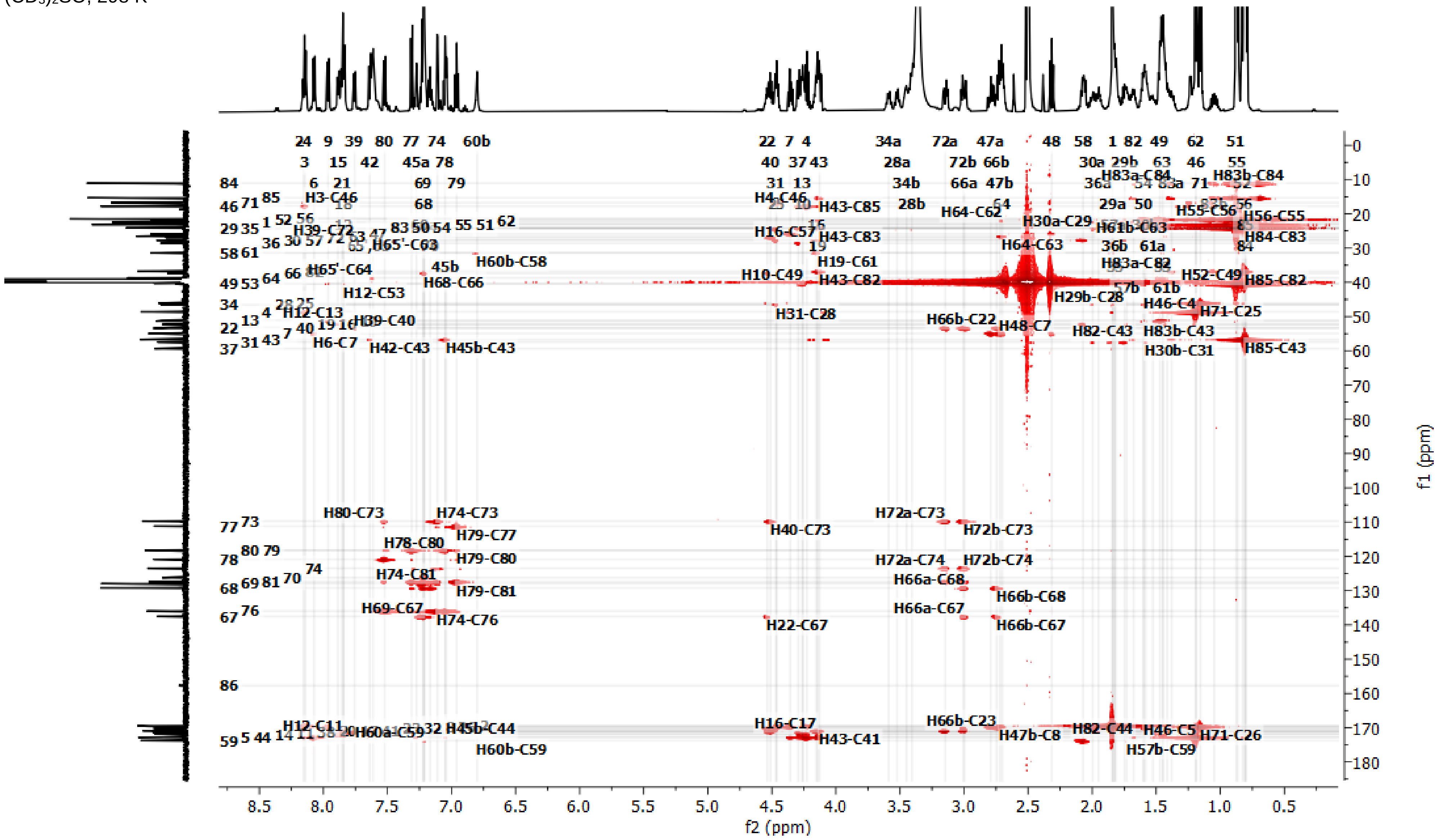

HSQC-NMR of Ac-Ala-Cys-Leu-Leu-Gln-Lys-Phe-Ala-Pro-Pro-Trp-Ile-NH_2 **(S12):**
$(CD_3)_2SO$, 298 K

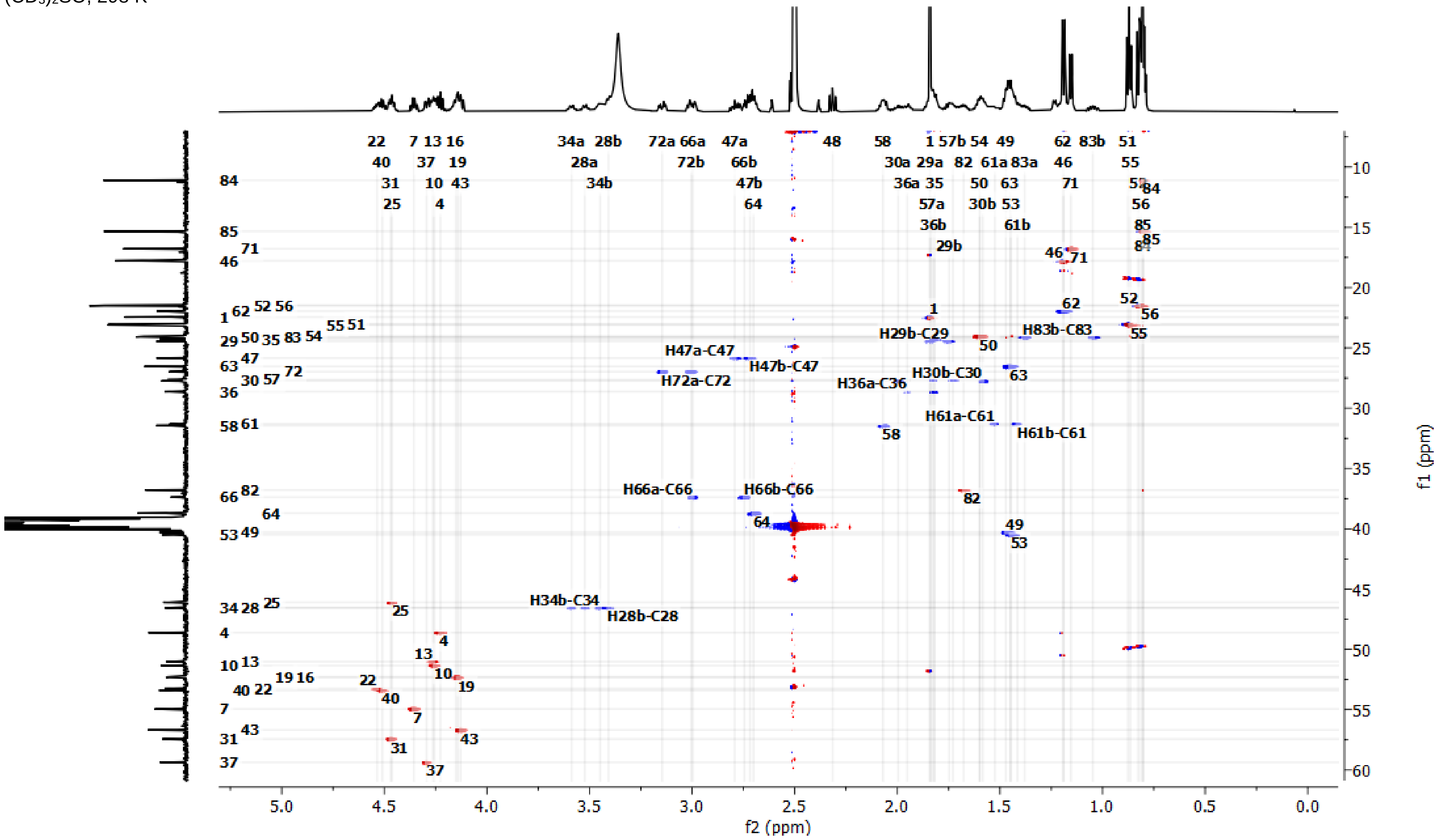

ROESY-NMR of Ac-Ala-Cys-Leu-Leu-Gln-Lys-Phe-Ala-Pro-Pro-Trp-Ile-NH_2 **(S12):**
$(CD_3)_2SO$, 298 K

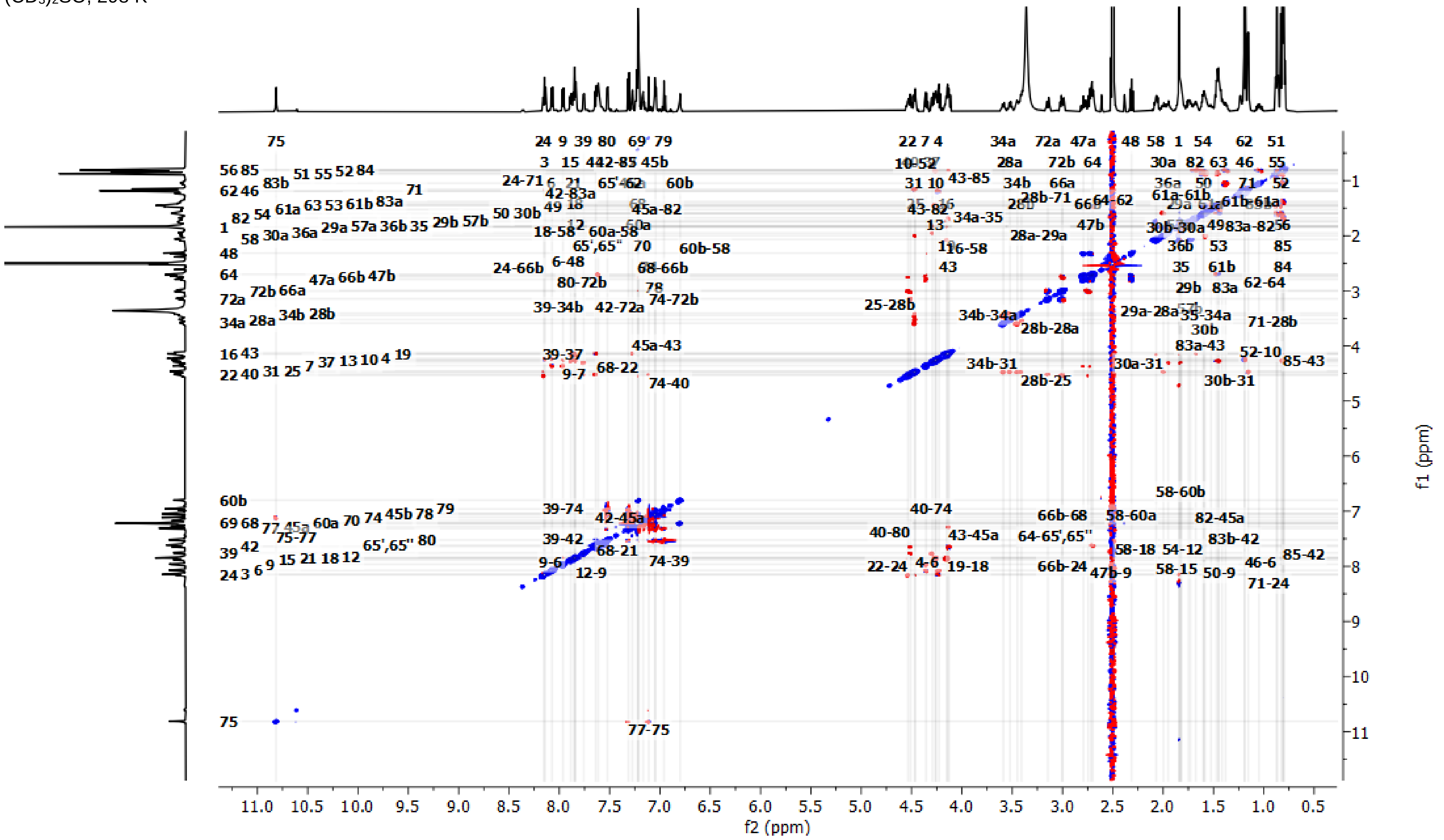

TOCSY-NMR of Ac-Ala-Cys-Leu-Leu-Gln-Lys-Phe-Ala-Pro-Pro-Trp-Ile-NH$_2$ **(S12):**
$(CD_3)_2SO$, 298 K

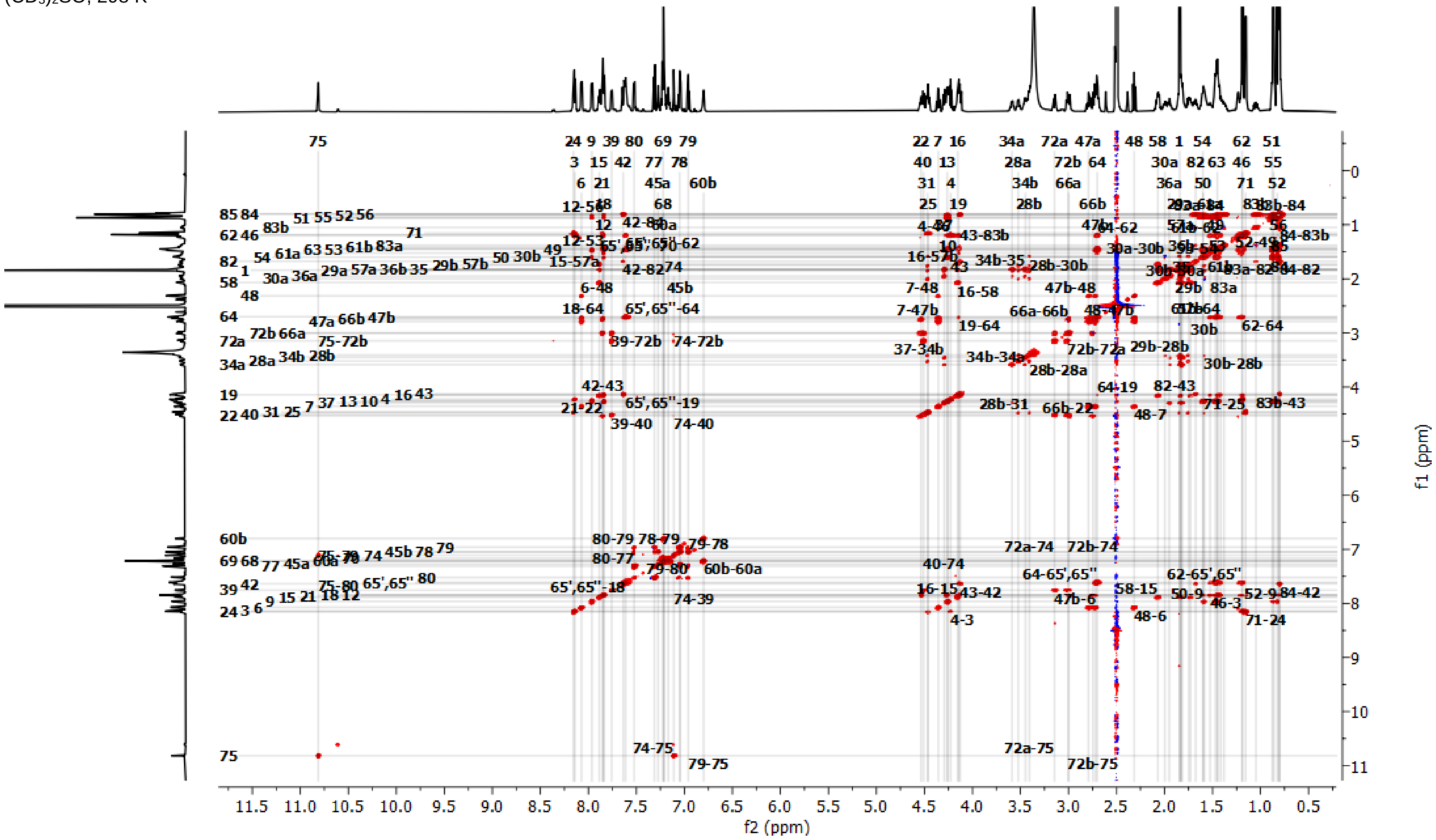

^{15}N-HSQC-NMR of Ac-Ala-Cys-Leu-Leu-Gln-Lys-Phe-Ala-Pro-Pro-Trp-Ile-NH$_2$ **(S12):**
$(CD_3)_2SO$, 298 K

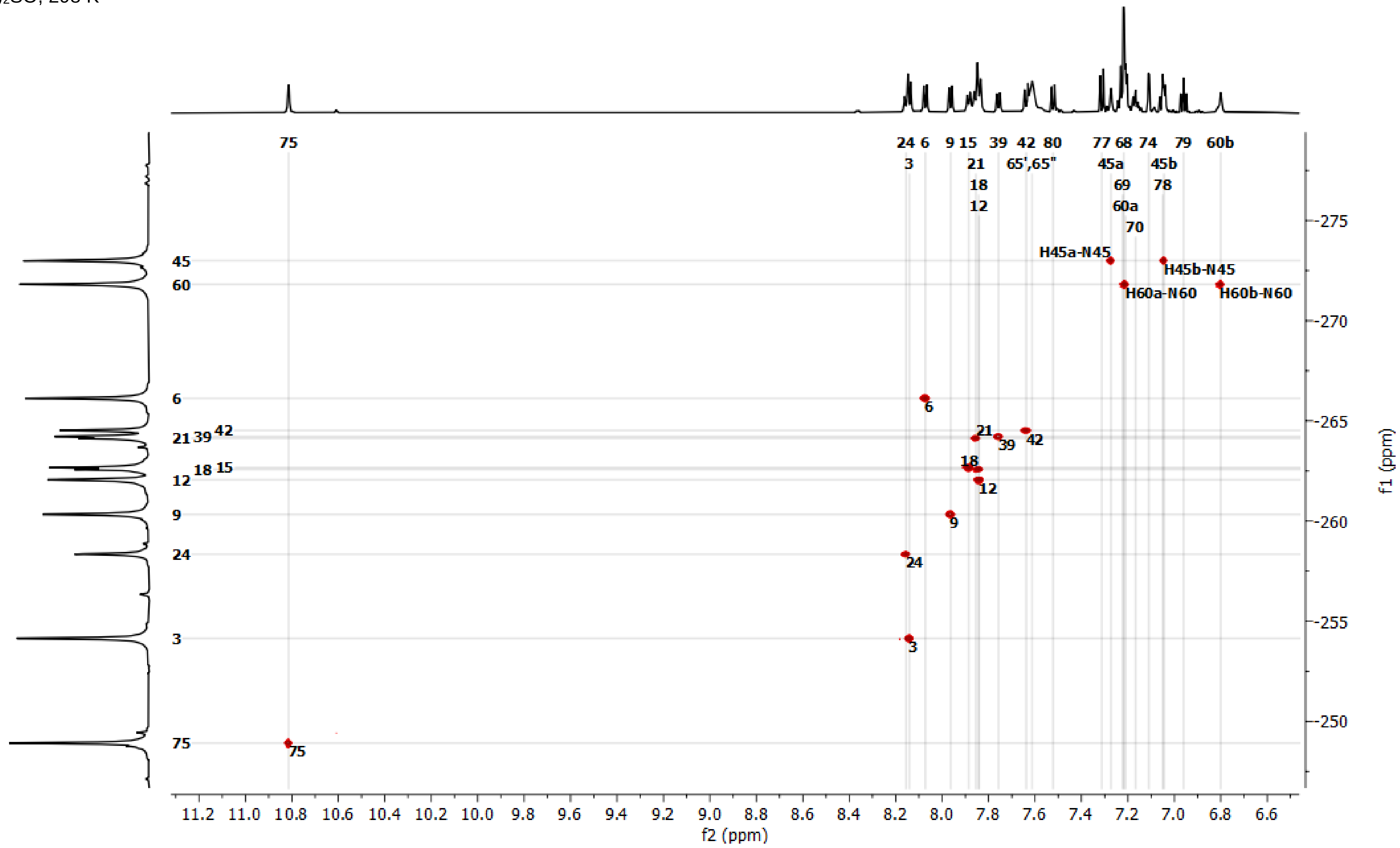

Stapled Ac-Ala-Cys-Leu-Leu-Gln-Glu-Phe-Ala-Pro-Pro-Trp-Ile-NH_2 (22):

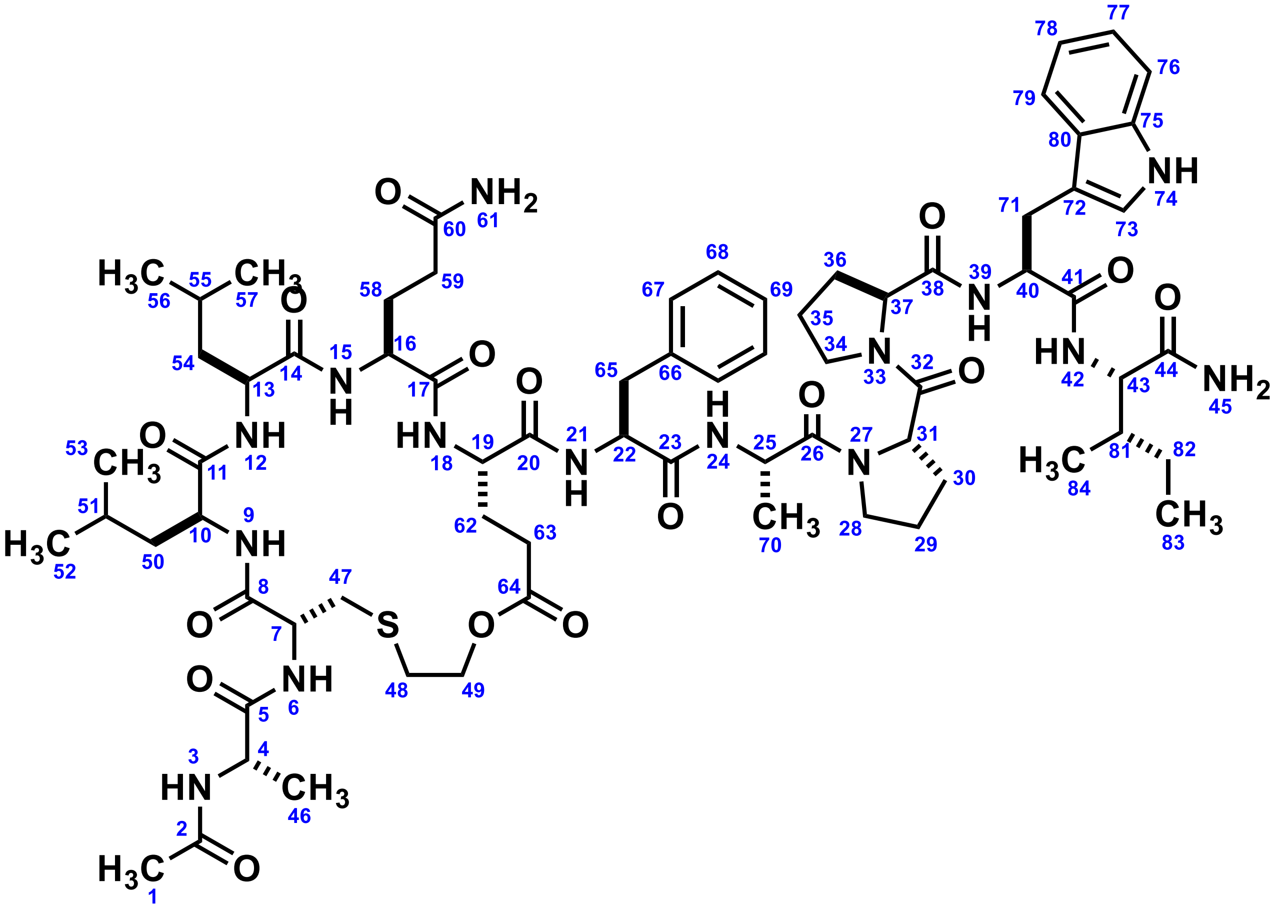

Table S31. Characterization table of stapled Ac-Ala-Cys-Leu-Leu-Gln-Glu-Phe-Ala-Pro-Pro-Trp-Ile-NH_2. The ^{15}N chemical shifts were referenced to CH_3NO_2.

Atom	δ (ppm)	J	HSQC	^{15}N-HSQC	HMQC	COSY	ROESY	TOCSY
**C1**	22,51		1					
**H1**	1,839	s	1		2		3	
**C2**	169,19				1, 3, 4			

**N3**	-254,33			3				
**H3**	8,123	d 7.1(4)		3	2, 4, 46	4	1, 6, 46	4, 46
**C4**	48,27		4		3, 46			
**H4**	4,268	quint 7.2(3, 46)	4		2, 5, 46	3, 46	6	3, 46
**C5**	172,75				4, 6, 46			
**N6**	-264,20			6				
**H6**	8,173	d 7.8(7)		6	5	7	3, 4, 7, 9, 46, 47b	7, 47a, 47b
**C7**	52,99		7		47b			
**H7**	4,417	t 7.9(6, 47a/b), d 6.2(47a/b)	7		8, 47	6, 47a, 47b	6, 9, 47a, 48a	6, 47a, 47b
**C8**	170,51				7, 9, 47a, 47b			
**N9**	-259,36			9				
**H9**	8,399	d 7.9(10)		9	8	10	6, 7, 10, 12, 47a, 50a, 50b	10, 50a, 50b, 51, 52, 53
**C10**	51,57		10					
**H10**	4,186	m (o.l.)	10			9, 50a, 50b	9, 12, 50a, 50b, 52, 53	9, 50a, 50b, 51, 52, 53
**C11**	171,93							
**N12**	-264,87			12				
**H12**	7,361	d 8.3(13)		12		13	9, 10, 13, 15, 54a, 55	13, 54a, 54b, 55, 56, 67
**C13**	51,10		13					
**H13**	4,238	m	13			12, 54a, 54b	12, 15, 54a, 54b, 56, 57	12, 54a, 54b, 55, 56, 57
**C14**	172,44				15			
**N15**	-263,08			15				
**H15**	8,183	d 5.6(16)		15	14	16	12, 13, 16, 18, 58a, 58b, 59a, 59b	16, 58a, 58b, 59a, 59b
**C16**	53,33		16		58a, 58b			
**H16**	4,011	d 8.0(58a/b), t 6.3(15, 58a/b)	16		17, 58, 59	15, 58a, 58b	15, 18, 58a, 58b, 59a, 59b	15, 58a, 58b, 59a, 59b
**C17**	171,40				16, 18			
**N18**	-262,71			18				
**H18**	7,758	d 8.4(19)		18	17	19	15, 16, 19, 21, 62a, 62b, 63a, 63b	19, 62a, 62b, 63a, 63b
**C19**	52,06		19					
**H19**	4,198	m (o.l.)	19			18, 62b	18, 21, 62a, 62b, 63a, 63b	18, 62a, 62b, 63a, 63b
**C20**	170,66				21			
**N21**	-264,64			21				

**H21**	7,830	d 8.5(22)		21	20	22	18, 19, 22, 24, 62a, 65b, 67	22, 65a, 65b
**C22**	53,57		22					
**H22**	4,489	m (o.l.)	22		23	21, 65a, 65b	21, 24, 67	21, 65a, 65b
**C23**	170,36				22, 24			
**N24**	-258,83			24				
**H24**	8,112	d 7.4(25)		24	23	25	21, 22, 25, 67, 70	25, 70
**C25**	46,14		25		70			
**H25**	4,464	quint 7.1(24, 70)	25		26, 70	24, 70	24, 28a	24, 70
**C26**	169,67				25, 70			
**N27**								
**C28**	46,55		28a, 28b					
**H28a**	3,556	d ~9.5(28b/29), t ~7.0(28b/29)	28			28b, 29a, 29b	25, 29a, 29b	29a, 29b, 30a, 30b, 31
**H28b**	3,395	m	28			28a, 29a, 29b	29a, 29b, 70	29a, 29b, 30a, 30b, 31
**C29**	24,49		35		31			
**H29a**	1,835	m (o.l.)			31	28a, 28b, 29b	28a, 28b, 29b, 31	28a, 28b, 30a, 30b, 31
**H29b**	1,763	m (o.l.)			31	28a, 28b, 29a	28a, 28b, 29a, 31	28a, 28b, 30a, 30b, 31
**C30**	27,74		30a, 30b		31			
**H30a**	2,005	m	30		32	30b, 31	30b, 31	28a, 28b, 29a, 29b, 31
**H30b**	1,564	m	30		32	30a, 31	30a	28a, 28b, 29a, 29b, 31
**C31**	57,45		31		29a, 29b			
**H31**	4,478	m (o.l.)	31		29, 30, 32	30a, 30b	29a, 29b, 30a, 34a, 34b	28a, 28b, 29a, 29b, 30a, 30b
**C32**	170,30				30a, 30b, 31			
**N33**								
**C34**	46,59		34a, 34b					
**H34a**	3,598	m	34			34b, 35	31, 35	35, 36a, 36b, 37
**H34b**	3,439	m	34			34a, 35	31, 35	35, 36a, 36b, 37
**C35**	24,29		35					
**H35**	1,813	m (o.l.)	29, 35			34a, 34b	34a, 34b	34a, 34b, 36a, 36b

**C36**	28,62		36a, 36b					
**H36a**	1,947	m	36			36b, 37	36b, 37	34a, 34b, 35, 37
**H36b**	1,808	m (o.l.)	36			36a, 37	36a, 37, 39	34a, 34b, 35, 37
**C37**	59,41		37					
**H37**	4,293	d 8.3(36a/b), d 3.2(36a/b)	37			36a, 36b	36a, 36b, 39	34a, 34b, 36a, 36b
**C38**	171,36				39, 40			
**N39**	-264,18			39				
**H39**	7,796	d 7.6(40)		39	38, 40	40	36b, 37, 40, 42, 71a, 71b, 73	40, 71a, 71b
**C40**	53,52		40		39, 71a, 71b			
**H40**	4,497	m (o.l.)	40		38, 41, 71, 72	39, 71a, 71b	39, 42, 73, 79	39, 71a, 71b, 73
**C41**	170,90				40, 42, 43, 71a			
**N42**	-264,50			42				
**H42**	7,680	d 9.0(43)		42	41	43	39, 40, 45a, 45b, 71a, 71b, 73, 81, 82a	43, 81, 82a, 82b, 83, 84
**C43**	56,81		43		45b, 84			
**H43**	4,115	d 9.0(42), d 6.9(81)	43		41, 44, 81, 82, 84	42, 81	45a, 45b, 81, 82a, 82b, 84	42, 81, 82a, 82b, 83, 84
**C44**	172,77				43, 45a			
**N45**	-273,00			45a, 45b				
**H45a**	7,301	m (o.l.)		45	44		42, 43	45b
**H45b**	7,049	m		45	43		42, 43	45a
**C46**	18,16		46		3, 4			
**H46**	1,158	d 7.2(4)	46		4, 5	4	3, 6	3, 4
**C47**	34,24		47a, 47b		7			
**H47a**	3,014	m (o.l.)	47		8, 48	7, 47b	7, 9, 49a	6, 7, 47b
**H47b**	2,756	m (o.l.)	47		7, 8, 48	7, 47a	6	6, 7, 47a
**C48**	31,12		48a, 48b		47a, 47b			
**H48a**	2,875	d 14.3(48b), d 7.6(49a/b), d 4.4(49a/b)	48			48b, 49a	7	48b, 49a, 49b
**H48b**	2,765	m (o.l.)	48			48a, 49b		48a, 49a, 49b
**C49**	64,13		49a, 49b					

**H49a**	4,192	m (o.l.)	49			48a	47a	48a, 48b
**H49b**	4,175	m (o.l.)	49			48b		48a, 48b
**C50**	39,73		50a, 50b		52, 53			
**H50a**	1,606	m (o.l.)	50			10, 50b	9, 10, 50b, 52, 53	9, 10, 50b, 52, 53
**H50b**	1,478	m (o.l.)	50			10, 50a	9, 10, 50a	9, 10, 50a, 52, 53
**C51**	24,15		51		52, 53			
**H51**	1,610	m (o.l.)	51			52, 53	52, 53	9, 10
**C52**	23,16		52		53			
**H52**	0,873	d 6.3(51)	52		50, 51, 53	51	10, 50a, 51, 53	9, 10, 50a, 50b, 53
**C53**	21,00		53		52			
**H53**	0,802	d 7.1(51)	53		50, 51, 52	51	10, 50a, 51, 52	9, 10, 50a, 50b, 52
**C54**	40,92		54a, 54b		56, 57			
**H54a**	1,486	m (o.l.)	54			13, 54b, 55	12, 13, 54b, 56, 57	12, 13, 56, 57
**H54b**	1,419	m	54			13, 54a, 55	13, 54a, 56, 57	12, 13, 55, 56, 57
**C55**	24,01		55		56, 57			
**H55**	1,655	m (o.l.)	55			54a, 54b, 56, 57	12, 56, 57	12, 13, 54b, 56, 57
**C56**	23,15		56		57			
**H56**	0,868	d 6.7(55)	56		54, 55, 57	55	13, 54a, 54b, 55	12, 13, 54a, 54b, 55
**C57**	21,44		57		56			
**H57**	0,850	d 6.6(55)	57		54, 55, 56	55	13, 54a, 54b, 55	13, 54a, 54b, 55
**C58**	26,26		58a, 58b		16, 59a, 59b			
**H58a**	1,819	m (o.l.)	58		16	16, 58b, 59a, 59b	15, 16, 59b	15, 16, 59a, 59b
**H58b**	1,761	m (o.l.)	58		16	16, 58a, 59a, 59b	15, 16, 59a	15, 16, 59a, 59b
**C59**	31,50		59a, 59b		16, 61b			
**H59a**	2,137	d 15.3(59b), d 9.2(58a/b), d 6.6(58a/b)	59		58, 60	58a, 58b, 59b	15, 16, 58b, 59b, 61a	15, 16, 58a, 58b

**H59b**	2,076	d 15.3(59a), d 9.1(58a/b), d 6.1(58a/b)	59		58, 60	58a, 58b, 59a	15, 16, 58a, 59a, 61a	15, 16, 58a, 58b
**C60**	173,81				59a, 59b			
**N61**	-271,68			61a, 61b				
**H61a**	7,301	m (o.l.)		61		61b	59a, 59b	61b
**H61b**	6,800	m		61	59	61a		61a
**C62**	26,67		62a, 62b					
**H62a**	1,797	m (o.l.)	62			62b, 63a, 63b	18, 19, 21, 62b, 63b	18, 19, 62b, 63a
**H62b**	1,701	m (o.l.)	62			19, 62a, 63a, 63b	18, 19, 62a, 63a	18, 19, 62a, 63b
**C63**	29,50		63a, 63b					
**H63a**	2,278	d 17.0(63b), d 10.0(62a/b), d 6.5(62a/b)	63		64	62a, 62b, 63b	18, 19, 62b	18, 19, 62a
**H63b**	2,230	d 17.0(63a), d 10.0(62a/b), d 5.8(62a/b)	63		64	62a, 62b, 63a	18, 19, 62a	18, 19, 62b
**C64**	172,69				63a, 63b			
**C65**	37,42		65a, 65b		67			
**H65a**	3,003	m (o.l.)	65		67	22, 65b	67	21, 22
**H65b**	2,742	m (o.l.)	65		67	22, 65a	21, 67	21, 22
**C66**	137,64				68			
**C67**	129,25		67		65a, 65b, 69			
**H67**	7,248	m (o.l.)	67		65, 69	68	21, 22, 24, 65a, 65b	12, 69
**C68**	128,01		68					
**H68**	7,213	m (o.l.)	68		66	67, 69		69
**C69**	126,24		69		67			
**H69**	7,155	m	69		67	68		67, 68
**C70**	16,70		70		25			
**H70**	1,160	d 7.0(25)	70		25, 26	25	24, 28b	24, 25

**C71**	26,93		71a, 71b		40			
**H71a**	3,145	d 5.2(40), d 14.9(71b)	71		40, 41, 72, 73, 80	40, 71b	39, 42, 73, 79	39, 40, 73
**H71b**	3,023	m (o.l.)	71		40, 72, 73, 80	40, 71a	39, 42, 73, 79	39, 40, 73
**C72**	109,69				40, 71a, 71b, 73, 74, 79			
**C73**	123,51		73		71a, 71b, 74			
**H73**	7,120	d 2.4(74)	73		72, 75, 80	74	39, 40, 42, 71a, 71b, 74	40, 71a, 71b, 74
**N74**	-248,61			74				
**H74**	10,871	d 2.4(73)		74	72, 73, 75, 80	73, 79	73	73
**C75**	135,99				73, 74, 77, 79			
**C76**	111,23		76		78			
**H76**	7,313	d 8.2(77), t 0.9(78, 79)	76		78, 80	77, 78		77, 79
**C77**	120,80		77		79			
**H77**	7,043	d 8.2(76), d 7.0(78), d 1.2(79)	77		75, 79	76, 78, 79		76, 78
**C78**	118,20		78		76, 79			
**H78**	6,953	d 1.0(76), d 7.0(77), d 7.9(79)	78		76, 80	76, 77, 79		77, 79
**C79**	118,25		79		77			
**H79**	7,515	q 0.9(74, 76, 77), d 7.9(78)	79		72, 75, 77, 78	74, 77, 78	40, 71a, 71b	76, 78
**C80**	127,45				71a, 71b, 73, 74, 76, 78			
**C81**	36,75		81		43, 82b, 83, 84			
**H81**	1,680	m (o.l.)	81			43, 82b, 84	42, 43, 82a, 84	42, 43, 82a, 83, 84
**C82**	24,16		82a, 82b		43, 83, 84			
**H82a**	1,375	m	82			82b, 83	42, 43, 81, 82b	42, 43, 81, 83, 84
**H82b**	1,047	m	82		81	81, 82a	43, 82a	42, 43, 83, 84
**C83**	11,10		83					
**H83**	0,796	t 7.4(82a/b)	83		81, 82	82a		42, 43, 81, 82a, 82b
**C84**	15,34		84		43			

**H84**	0,796	d 6.9(81)	84		43, 81, 82	81	43, 81	42, 43, 81, 82a, 82b

^{1}H NMR of **stapled** Ac-Ala-Cys-Leu-Leu-Gln-Glu-Phe-Ala-Pro-Pro-Trp-Ile-NH2 **(22):**
600 MHz, $(CD_3)_2SO$, 298 K

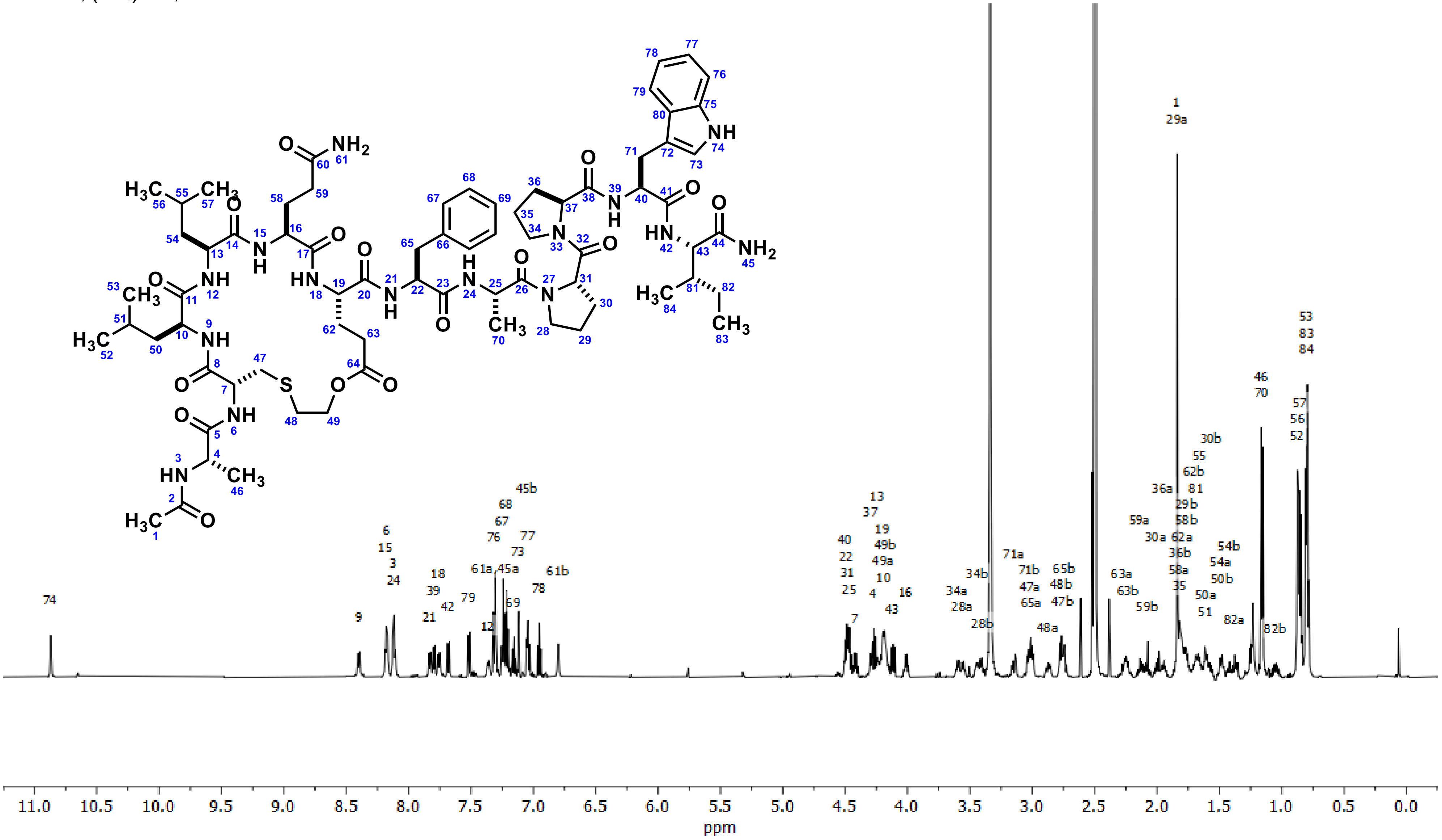

^{13}C NMR of **stapled** Ac-Ala-Cys-Leu-Leu-Gln-Glu-Phe-Ala-Pro-Pro-Trp-Ile-NH_2 **(22):**
151 MHz, $(CD_3)_2SO$, 298 K

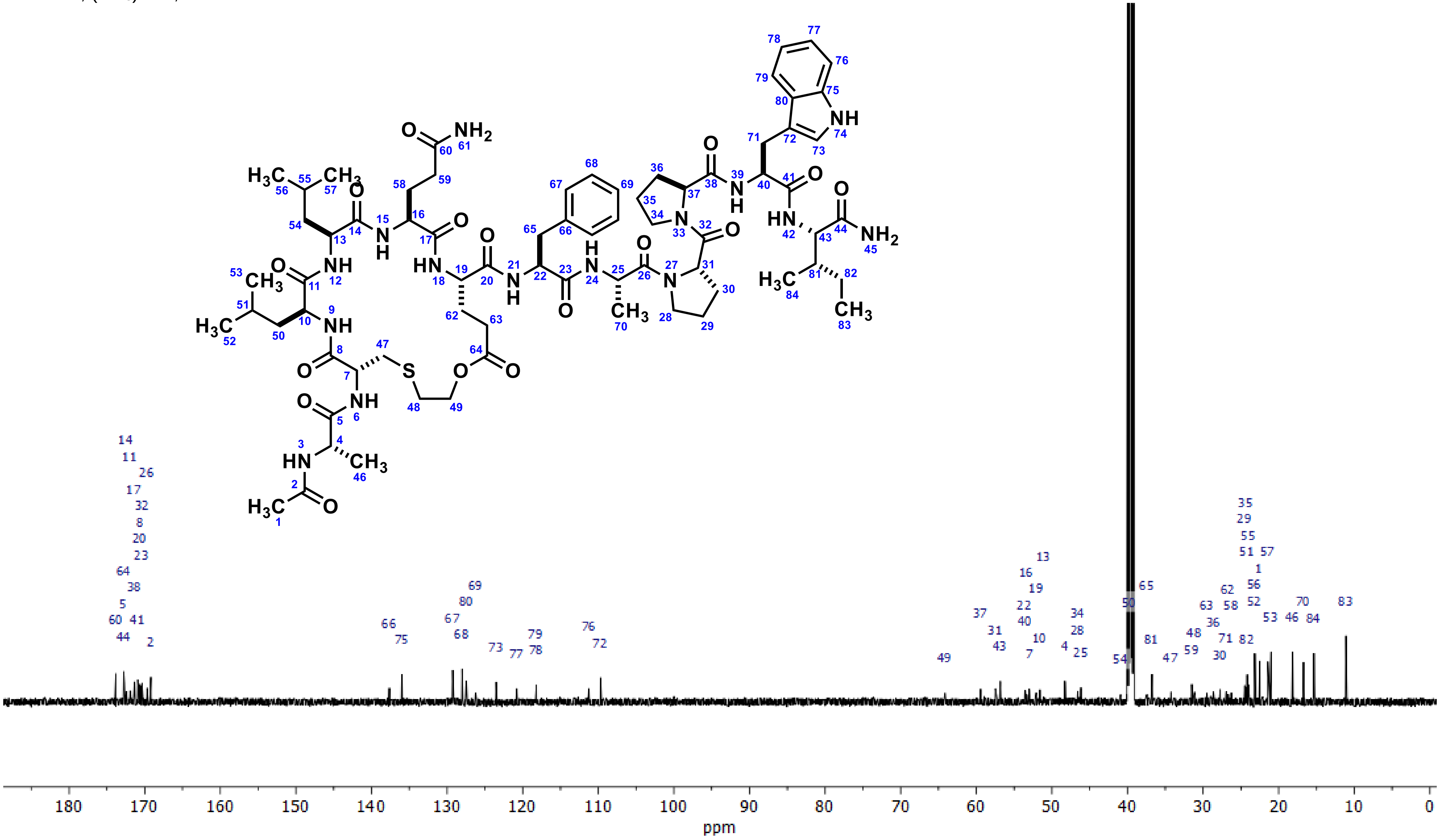

COSY-NMR of **stapled** Ac-Ala-Cys-Leu-Leu-Gln-Glu-Phe-Ala-Pro-Pro-Trp-Ile-NH_2 **(22):**
$(CD_3)_2SO$, 298 K

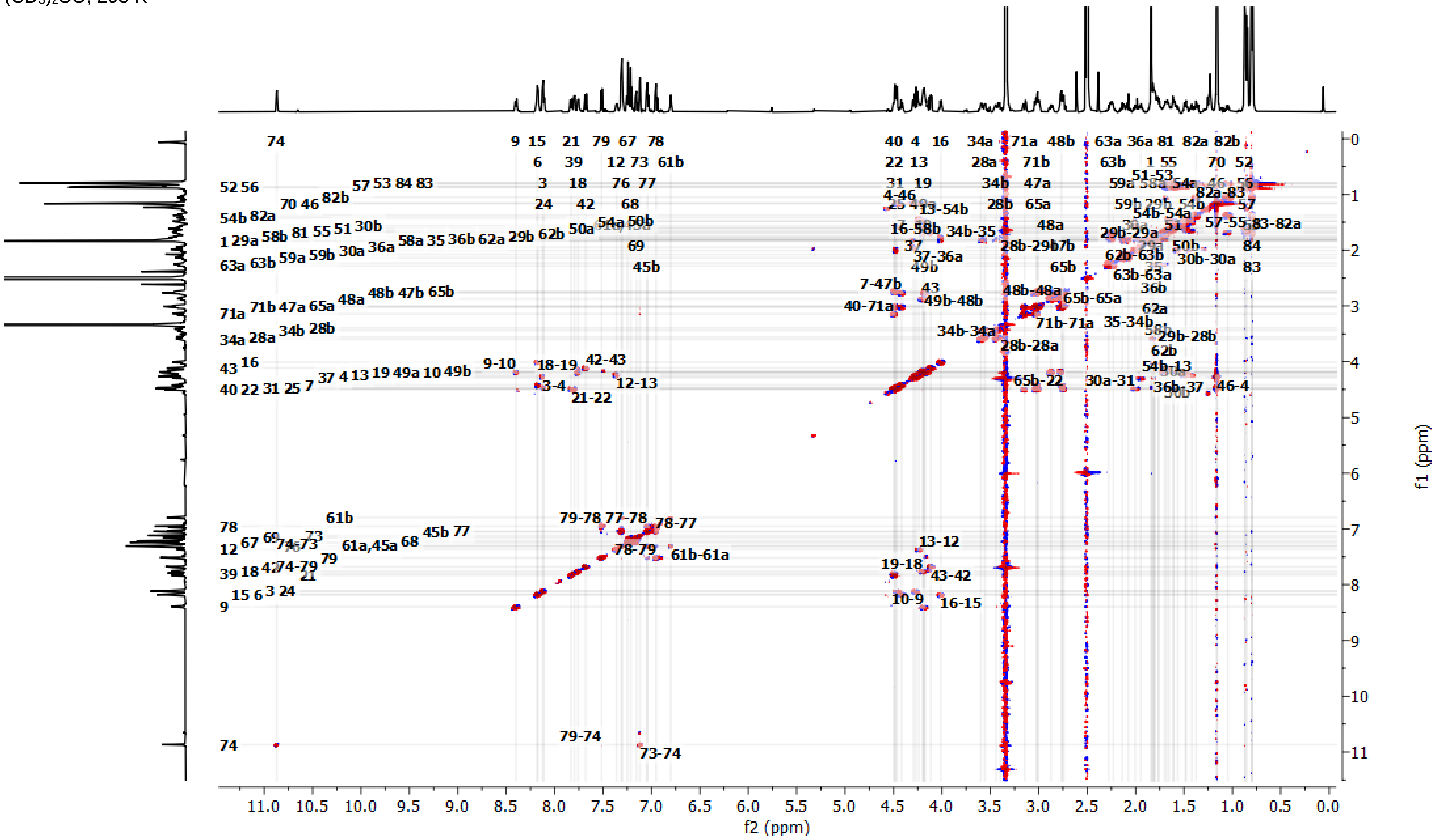

HSQC-NMR of **stapled** Ac-Ala-Cys-Leu-Leu-Gln-Glu-Phe-Ala-Pro-Pro-Trp-Ile-NH_2 **(22):**
$(CD_3)_2SO$, 298 K

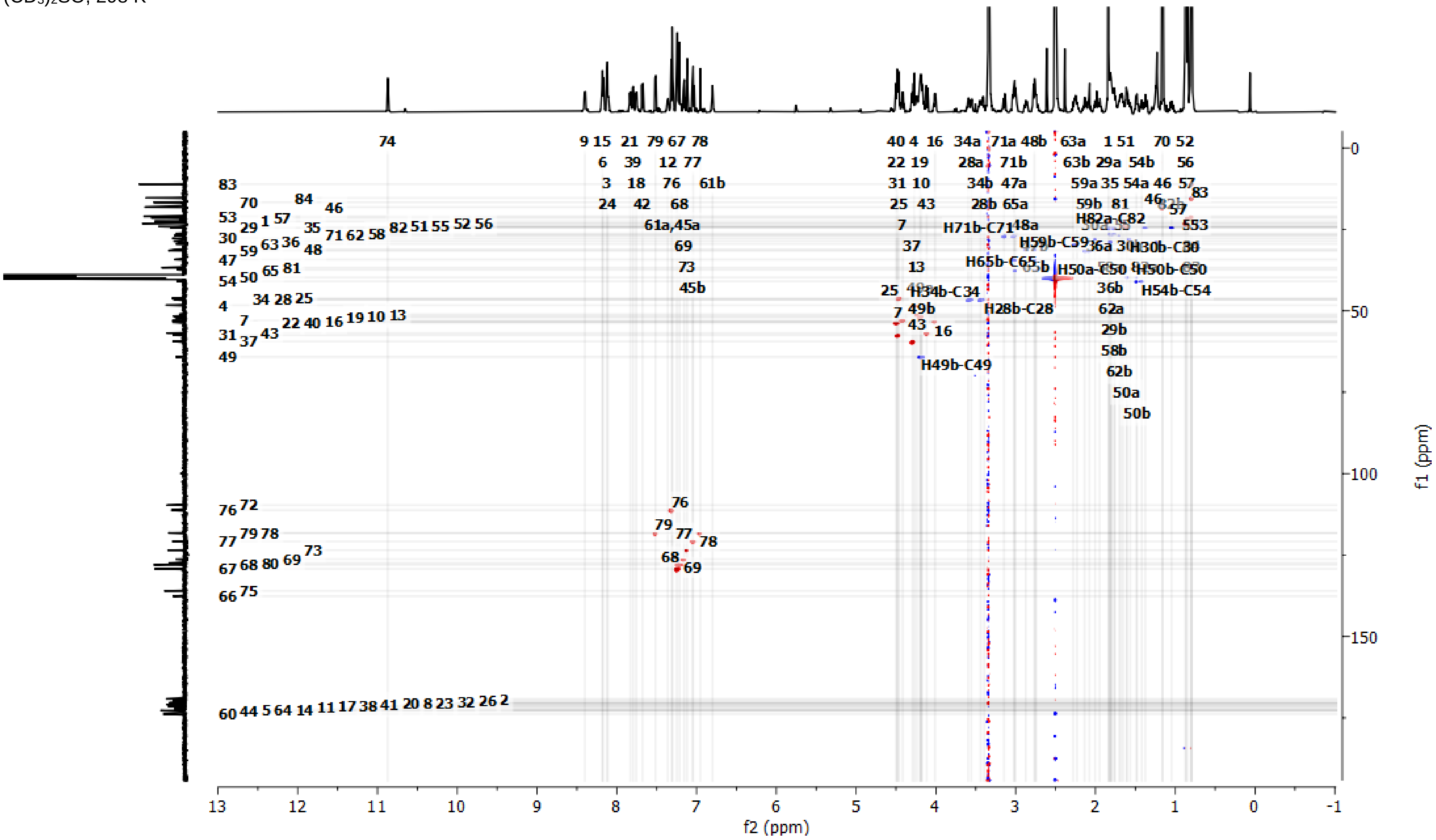

HMQC-NMR of **stapled** Ac-Ala-Cys-Leu-Leu-Gln-Glu-Phe-Ala-Pro-Pro-Trp-Ile-NH_2 **(22):**
$(CD_3)_2SO$, 298 K

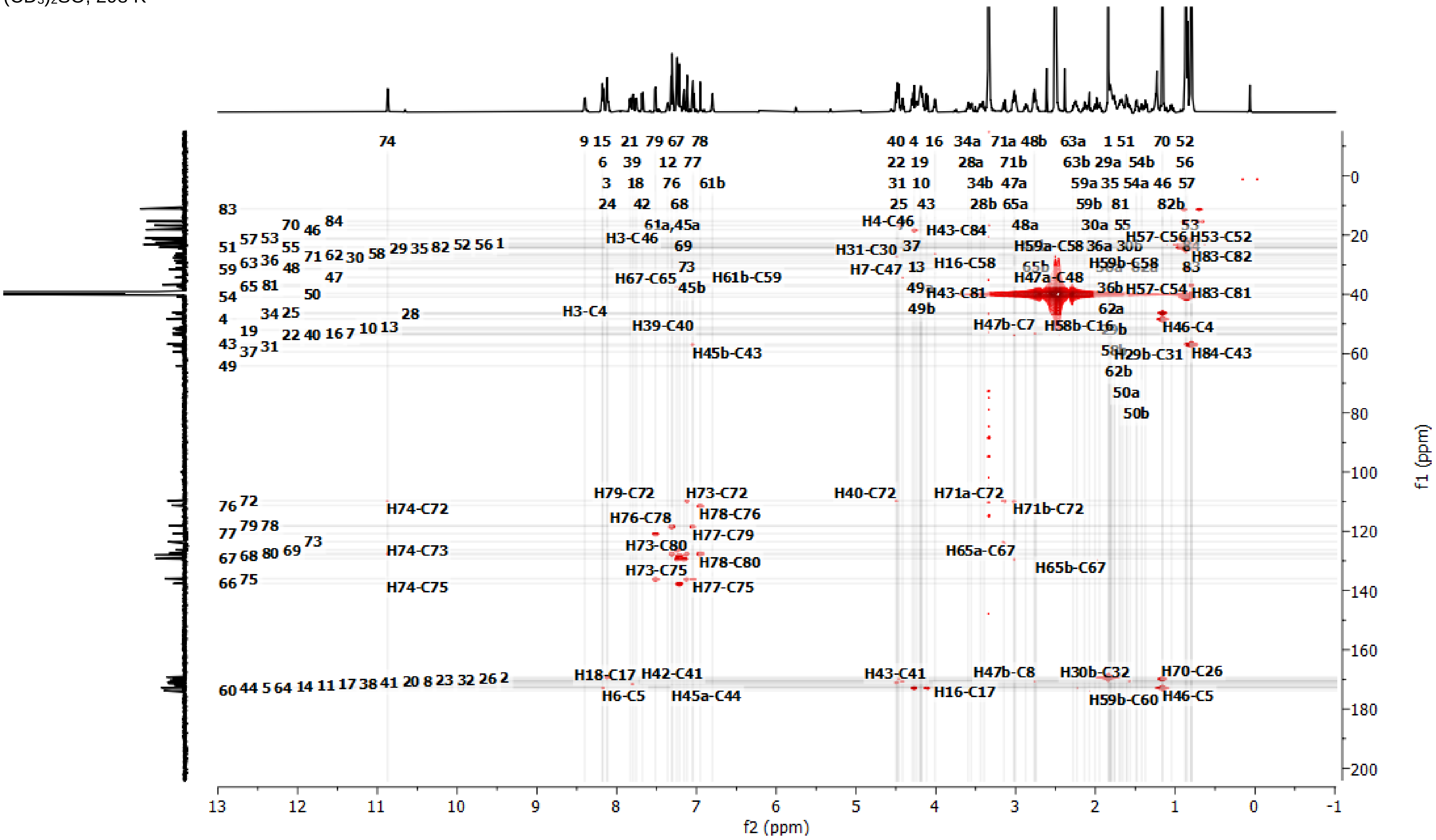

ROESY-NMR of **stapled** Ac-Ala-Cys-Leu-Leu-Gln-Glu-Phe-Ala-Pro-Pro-Trp-Ile-NH_2 **(22):**
$(CD_3)_2SO$, 298 K

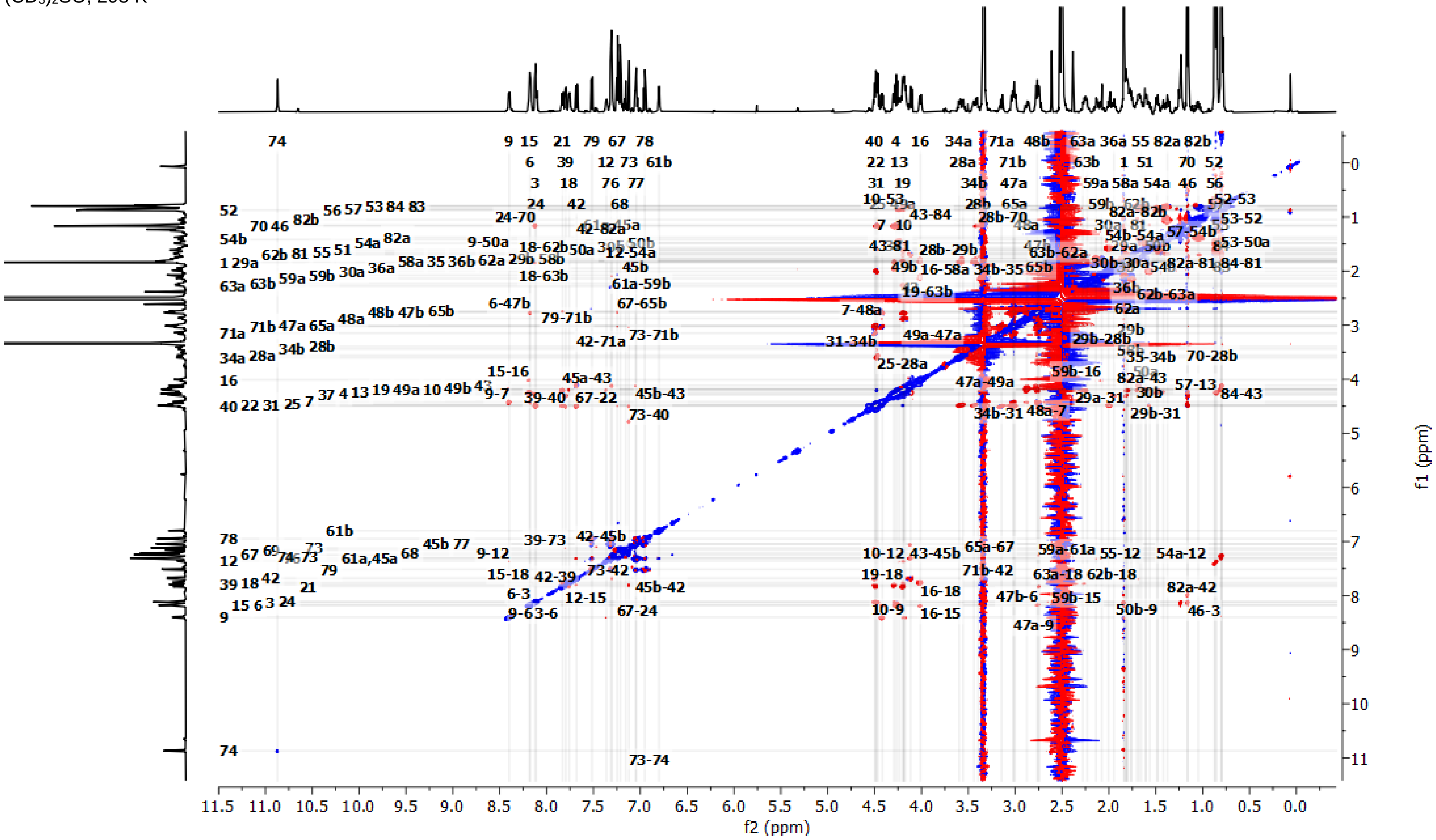

TOCSY-NMR of **stapled** Ac-Ala-Cys-Leu-Leu-Gln-Glu-Phe-Ala-Pro-Pro-Trp-Ile-NH_2 **(22)** with water suppression: $(CD_3)_2SO$, 298 K

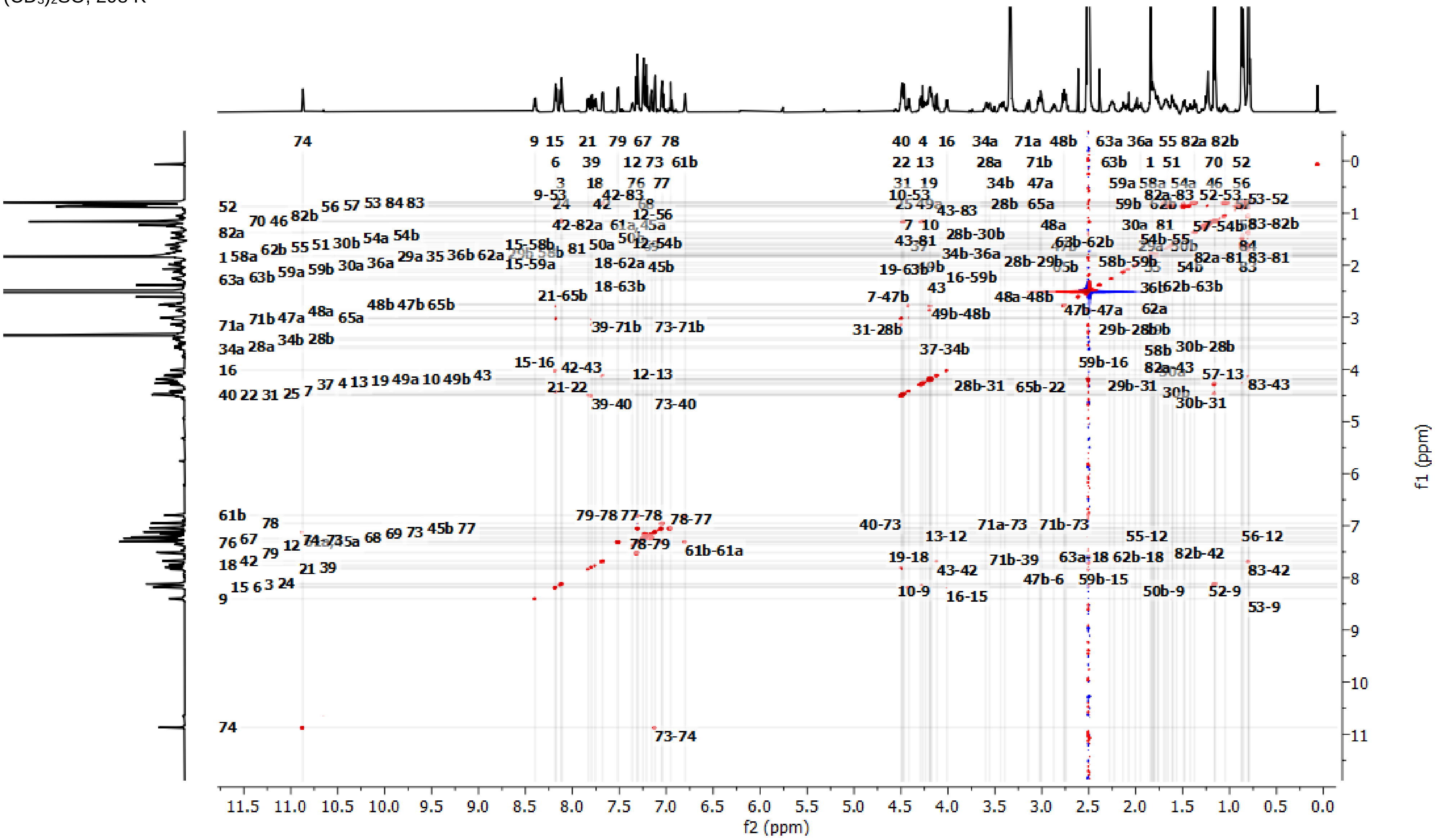

^{15}N-HSQC-NMR of **stapled** Ac-Ala-Cys-Leu-Leu-Gln-Glu-Phe-Ala-Pro-Pro-Trp-Ile-NH_2 **(22):**
$(CD_3)_2SO$, 298 K

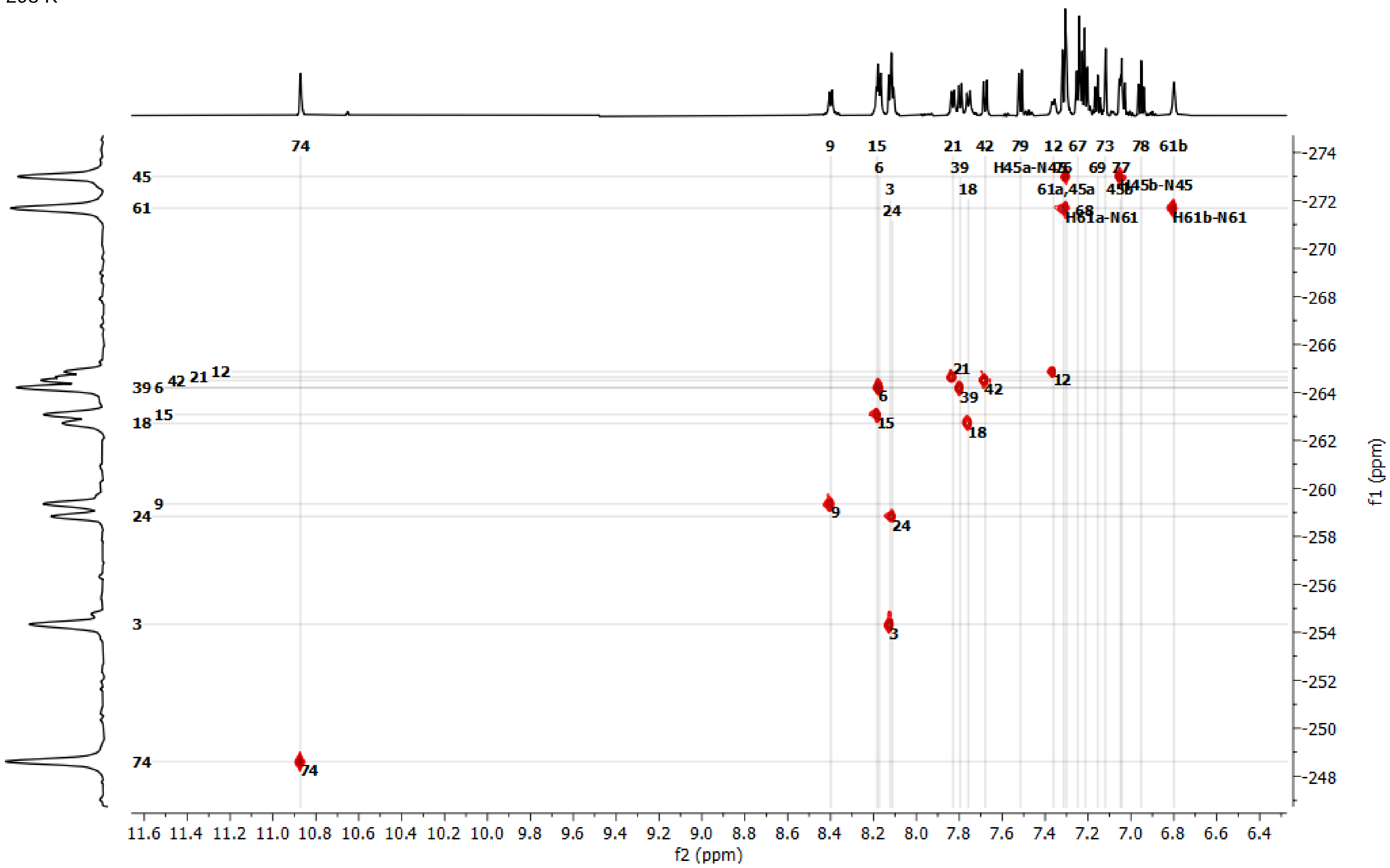

Stapled Ac-Ala-Cys-Leu-Leu-Gln-Cys-Phe-Ala-Pro-Pro-Trp-Ile-NH_2 (23):

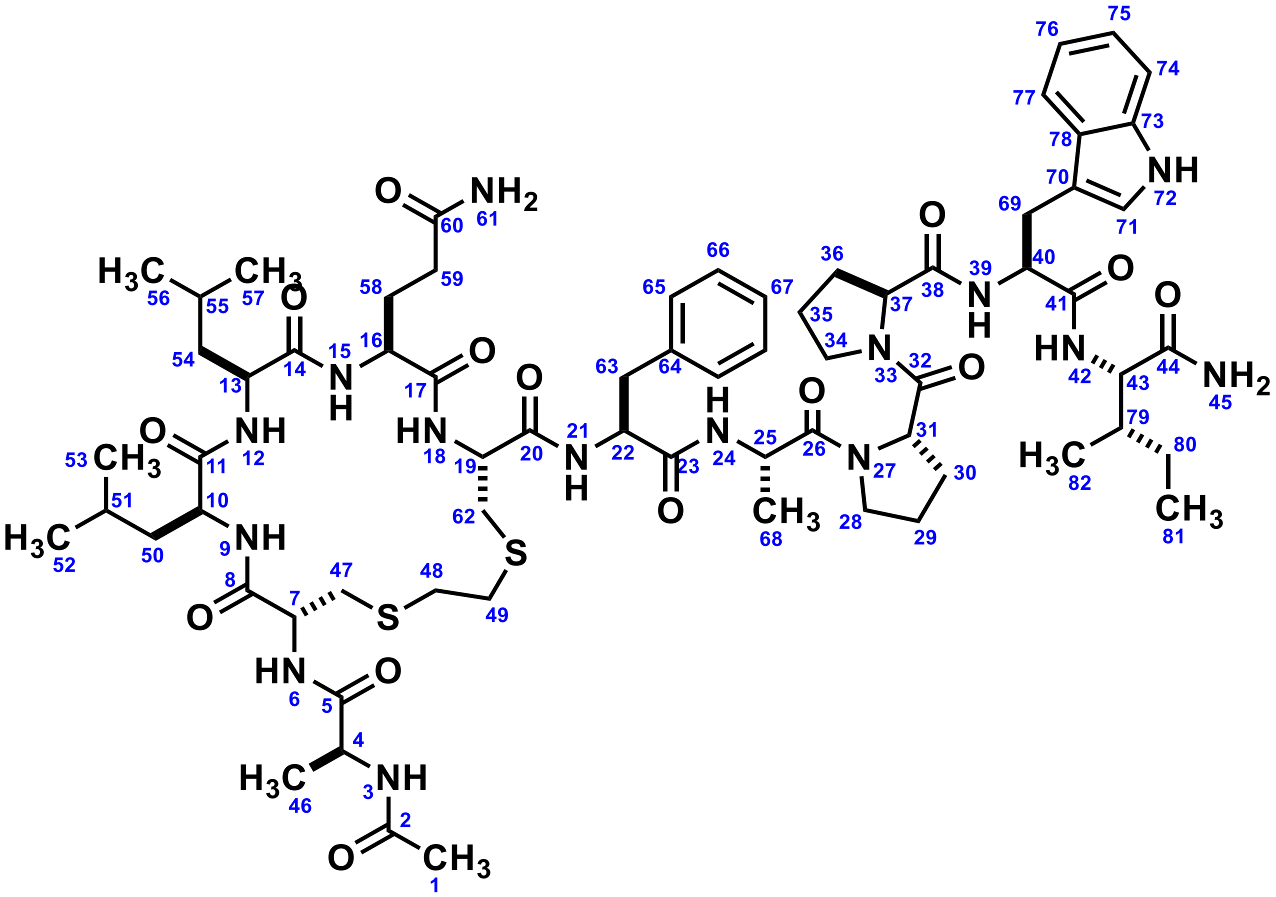

Table S32. Characterization table of stapled Ac-Ala-Cys-Leu-Leu-Gln-Cys-Phe-Ala-Pro-Pro-Trp-Ile-NH_2. The ^{15}N chemical shifts were referenced to NH_3(l).

Atom	δ (ppm)	#	J	HSQC	^{15}N-HSQC	HMBC	COSY	NOESY	TOCSY
**C1**	22,51			1					
**H1**	1,839	3H	s	1		2		3, 4, 6, 9, 46, 50, 52, 53	
**C2**	169,41					1, 3, 4			
**N3**	125,41				3				

**H3**	8,100	1H	d 7.0(4)		3	2, 4, 5, 46	4, 46	1, 4, 6, 46	4, 46
**C4**	48,49			4		3, 46			
**H4**	4,243	1H	quint 7.0(3, 46)	4		2, 5, 46	3, 46	1, 3, 6, 7, 46, 47a, 47b	3, 46
**C5**	173,18					3, 4, 6, 7, 46			
**N6**	115,72				6				
**H6**	8,229	1H	d 7.8(7)		6	5, 7, 47	7	1, 3, 4, 7, 12, 46, 47a, 47b, 48a, 48b	7, 47a, 47b
**C7**	52,87			7		6, 47b			
**H7**	4,474	1H	m (o.l.)	7		5, 8, 47	6, 47a, 47b	4, 6, 9, 10, 12, 47a, 47b, 48a, 48b, 50	6, 47a, 47b
**C8**	170,46					7, 9, 10, 47a, 47b			
**N9**	119,71				9				
**H9**	8,247	1H	d 8.3(10)		9	8, 10	10	1, 7, 10, 12, 13, 15, 47a, 47b, 48a, 48b, 50, 51, 52, 53	10, 50, 51, 52, 53
**C10**	52,29			10		9, 50, 51			
**H10**	4,097	1H	t 8.3(9, 50a/b), d 6.5(50a/b)	10		8, 11, 50, 51	9, 50	7, 9, 12, 13, 47a, 47b, 50, 51, 52, 53	9, 50, 51, 52, 53
**C11**	171,41					10, 12, 13, 50			
**N12**	112,53				12				
**H12**	7,333	1H	d 8.6(13)		12	11	13	6, 7, 9, 10, 13, 15, 16, 18, 46, 47a, 47b, 48a, 48b, 49a, 49b, 50, 51, 52, 53, 54, 55, 56, 57, 58a, 58b	13, 54, 55, 56, 57
**C13**	50,36			13		54, 55			
**H13**	4,267	1H	m (o.l.)	13		11, 14, 54, 55	12, 54	9, 10, 12, 15, 16, 54, 55, 56, 57, 58b	12, 54, 55, 56, 57
**C14**	171,84					13, 15, 16, 54			
**N15**	115,47				15				
**H15**	8,011	1H	d 6.3(16)		15	14, 16, 58	16	9, 12, 13, 16, 18, 54, 55, 56, 57, 58a, 58b, 59a, 59b	16, 58a, 58b, 59a, 59b
**C16**	52,99			16		15, 58a, 58b, 59a, 59b			
**H16**	4,052	1H	d 7.6(58a/b), t 6.3(15, 58a/b)	16		14, 17, 58, 59	15, 58a, 58b	12, 13, 15, 18, 19, 49a, 49b, 54, 58a, 58b, 59a, 59b, 62a, 62b	15, 58a, 58b, 59a, 59b
**C17**	171,06					16, 18, 19, 58a, 58b			

**N18**	118,16				18				
**H18**	8,155	1H	d 8.4(19)		18	17	19	12, 15, 16, 19, 21, 22, 24, 48a, 48b, 49a, 49b, 54, 58a, 58b, 59a, 59b, 62a, 62b, 65	19, 24, 62a, 62b
**C19**	54,71			19		62a, 62b			
**H19**	4,369	1H	d 8.4(18), d 7.8(62b), d 5.7(62a)	19		17, 20, 62	18, 62a, 62b	16, 18, 21, 22, 24, 48a, 48b, 49a, 49b, 62a, 62b, 63a, 63b, 65	18, 62a, 62b
**C20**	169,79					19, 21, 22, 62a, 62b			
**N21**	117,19				21				
**H21**	8,013	1H	d 8.1(22)		21	20, 22	22	18, 19, 22, 24, 25, 62a, 62b, 63a, 63b, 65, 68	22, 63a, 63b
**C22**	53,63			22		21, 63a, 63b			
**H22**	4,507	1H	m (o.l.)	22		20, 23, 63, 64	21, 63a, 63b	18, 19, 21, 24, 63a, 63b, 65	21, 63a, 63b
**C23**	170,10					22, 24, 25, 63a, 63b, 68			
**N24**	121,13				24				
**H24**	8,108	1H	d 7.5(25)		24	23, 25	25, 68	18, 19, 21, 22, 25, 28a, 28b, 63a, 63b, 65, 68	18, 25, 68
**C25**	46,09			25		24, 68			
**H25**	4,467	1H	m (o.l.)	25		23, 26, 68	24, 68	21, 24, 28a, 28b, 68	24, 68
**C26**	169,60					25, 68			
**N27**	n.f.								
**C28**	46,56			28a, 28b		29a, 29b, 30a, 30b, 31			
**H28a**	3,533	1H	d 9.7 (28b/29), t 7.1(28b/29)	28		29	28b, 29a, 29b	24, 25, 28b, 29a, 29b, 30a, 30b, 68	29a, 29b, 30a, 30b, 31
**H28b**	3,403	1H	m (o.l.)	28		29	28a, 29a, 29b	24, 25, 28a, 29a, 29b, 30a, 30b, 68	29a, 29b, 30a, 30b, 31
**C29**	24,49			29a, 29b		28a, 28b, 30a, 30b, 31			
**H29a**	1,835	1H	m (o.l.)	29		28, 30, 31	28a, 28b	28a, 28b, 29b, 30b, 31, 68	28a, 28b, 29b, 30a, 30b, 31
**H29b**	1,750	1H	m (o.l.)	29		28, 30, 31	28a, 28b	28a, 28b, 29a, 30a, 30b, 31, 68	28a, 28b, 29a, 30a, 30b, 31

**C30**	27,76			30a, 30b		29a, 29b, 31			
**H30a**	1,989	1H	m	30		28, 29, 31, 32	30b, 31	28a, 28b, 29b, 30b, 31, 34a, 34b, 68	28a, 28b, 29a, 29b, 30b, 31
**H30b**	1,569	1H	m (o.l.)	30		28, 29, 31, 32	30a, 31	28a, 28b, 29a, 29b, 30a, 31, 34a, 34b, 39, 68	28a, 28b, 29a, 29b, 30a, 31
**C31**	57,46			31		29a, 29b, 30a, 30b			
**H31**	4,458	1H	m (o.l.)	31		28, 29, 30, 32	30a, 30b	29a, 29b, 30a, 30b, 34a, 34b, 37, 39, 71	28a, 28b, 29a, 29b, 30a, 30b
**C32**	170,30					30a, 30b, 31			
**N33**	n.f.								
**C34**	46,61			34a, 34b		37			
**H34a**	3,594	1H	d 9.7(34b/35), t 7.1(34b/35)	34		35	34b, 35	30a, 30b, 31, 34b, 36a, 36b, 37, 39, 69a, 69b	34b, 35, 36a, 36b, 37
**H34b**	3,445	1H	m (o.l.)	34		35	34a, 35	30a, 30b, 31, 34a, 36a, 36b, 37, 39, 69a, 69b	34a, 35, 36a, 36b, 37
**C35**	24,31			35		34a, 34b, 36a, 36b, 37			
**H35**	1,820	2H	m (o.l.)	35		36	34a, 34b		34a, 34b, 36a, 37
**C36**	28,63			36a, 36b		35, 37			
**H36a**	1,943	1H	m	36		35, 38	36b, 37	34a, 34b, 36b, 37, 39, 42	34a, 34b, 35, 36b, 37
**H36b**	1,812	1H	m (o.l.)	36		35, 38	36a, 37	34a, 34b, 36a, 37, 39, 42, 69b, 71, 77	34a, 34b, 36a, 37
**C37**	59,39			37					
**H37**	4,293	1H	m (o.l.)	37		34, 35, 36, 38	36a, 36b	31, 34a, 34b, 36a, 36b, 39, 40, 42, 69a, 69b, 71	34a, 34b, 35, 36a, 36b
**C38**	171,35					36a, 36b, 37, 39, 40			
**N39**	115,56				39				
**H39**	7,755	1H	d 7.5(40)		39	38, 40, 69	40	30b, 31, 34a, 34b, 36a, 36b, 37, 40, 42, 69a, 69b, 71, 72, 77	40, 69a, 69b, 71
**C40**	53,44			40		39, 69a, 69b			

**H40**	4,504	1H	m (o.l.)	40		38, 41, 69, 70	39, 69a, 69b	37, 39, 42, 43, 69a, 69b, 71, 77	39, 69a, 69b, 71
**C41**	170,90					40, 42, 43, 69a, 69b			
**N42**	115,22				42				
**H42**	7,626	1H	d 9.0(43)		42	41, 43	43	36a, 36b, 37, 39, 40, 43, 45a, 45b, 69a, 69b, 71, 77, 79, 80a, 80b, 82	43, 79, 80a, 80b, 81, 82
**C43**	56,73			43		42, 45b, 80a, 80b, 82			
**H43**	4,124	1H	d 9.0(42), d 6.9(79)	43		41, 44, 79, 80, 82	42, 79	40, 42, 45a, 45b, 79, 82	42, 79, 80a, 80b, 81, 82
**C44**	172,75					43, 45a, 45b			
**N45**	106,73				45a, 45b				
**H45a**	7,265	1H	m		45	44	45b	42, 43, 45b, 79, 82	45b
**H45b**	7,043	1H	m		45	43, 44	45a	42, 43, 45a, 79, 82	45a
**C46**	18,21			46		3, 4			
**H46**	1,165	3H	d 7.0(4)	46		4, 5	3, 4	1, 3, 4, 6, 12, 47a, 47b, 50, 51, 52, 53	3, 4
**C47**	32,58			47a, 47b		6, 7, 48a, 48b			
**H47a**	3,042	1H	d 14.2(47b), d 5.3(7)	47		8, 48	7, 47b	4, 6, 7, 9, 10, 12, 46, 47b, 48a, 48b, 50	6, 7, 47b
**H47b**	2,706	1H	m (o.l.)	47		7, 8, 48	7, 47a	4, 6, 7, 9, 10, 12, 46, 47a, 48a, 48b, 50	6, 7, 47a
**C48**	31,29			48a, 48b		47a, 47b, 49a, 49b			
**H48a**	2,846	1H	d 13.5(48b), d 11.0(49a/b), d 5.3(49a/b)	48		47, 49	48b, 49a, 49b	6, 7, 9, 12, 18, 19, 47a, 47b, 48b, 49a, 49b, 50	48b, 49a, 49b
**H48b**	2,621	1H	m (o.l.)	48		47, 49	48a, 49a, 49b	6, 7, 9, 12, 18, 19, 47a, 47b, 48a, 49a, 49b, 50	48a, 49a, 49b
**C49**	33,05			49a, 49b		48a, 48b, 62a, 62b			
**H49a**	3,084	1H	t 11.5(49b, 48a/b), d 5.4(48a/b)	49		48, 62	48a, 48b, 49b	12, 16, 18, 19, 48a, 48b, 49b	48a, 48b, 49b

**H49b**	2,729	1H	m (o.l.)	49		48, 62	48a, 48b, 49a	12, 16, 18, 19, 48a, 48b, 49a	48a, 48b, 49a
**C50**	39,95			50		10, 51, 52, 53			
**H50**	1,505	2H	m (o.l.)	50		10, 11, 51, 52, 53	10, 51	1, 7, 9, 10, 12, 46, 47a, 47b, 48a, 48b, 51, 52, 53	9, 10, 51, 52, 53
**C51**	24,36			51		10, 50, 52, 53			
**H51**	1,580	1H	m (o.l.)	51		10, 50, 52, 53	50, 52, 53	9, 10, 12, 46, 50, 52, 53	9, 10, 50, 52, 53
**C52**	22,92			52		50, 51, 53			
**H52**	0,875	3H	d 6.5(51)	52		50, 51, 53	51	1, 9, 10, 12, 46, 50, 51	9, 10, 50, 51, 53
**C53**	21,13			53		50, 51, 52			
**H53**	0,789	3H	d 6.4(51)	53		50, 51, 52	51	1, 9, 10, 12, 46, 50, 51	9, 10, 50, 51, 52
**C54**	41,38			54		13, 55, 56, 57			
**H54**	1,497	2H	m (o.l.)	54		13, 14, 55, 56, 57	13, 55	12, 13, 15, 16, 18, 56, 57, 58a, 58b	12, 13, 55, 56, 57
**C55**	24,25			55		13, 54, 56, 57			
**H55**	1,549	1H	m (o.l.)	55		13, 54, 56, 57	54, 56, 57	12, 13, 15	12, 13, 54, 56, 57
**C56**	23,37			56		54, 55, 57			
**H56**	0,875	3H	d 6.5(55)	56		54, 55, 57	55	12, 13, 15, 54	12, 13, 54, 55
**C57**	21,39			57		54, 55, 56			
**H57**	0,870	3H	d 6.4(55)	57		54, 55, 56	55	12, 13, 15, 54	12, 13, 54, 55
**C58**	27,02			58a, 58b		15, 16, 59a, 59b			
**H58a**	1,808	1H	m (o.l.)	58		16, 17, 59	16, 58b, 59a, 59b	12, 15, 16, 18, 54, 59a, 59b, 65	15, 16, 58b, 59a, 59b
**H58b**	1,718	1H	m (o.l.)	58		16, 17, 59	16, 58a, 59a, 59b	12, 13, 15, 16, 18, 54, 59a, 59b, 65	15, 16, 58a, 59a, 59b

**C59**	31,30			59a, 59b		16, 58a, 58b, 61b			
**H59a**	2,137	1H	d 15.5(59b), d 10.0(58a/b), d 6.0(58a/b)	59		16, 58, 60	58a, 58b, 59b	15, 16, 18, 58a, 58b, 61a, 61b	15, 16, 58a, 58b, 59b
**H59b**	2,073	1H	d 15.5(59a), d 10.0(58a/b), d 6.0(58a/b)	59		16, 58, 60	58a, 58b, 59a	15, 16, 18, 58a, 58b, 61a, 61b	15, 16, 58a, 58b, 59a
**C60**	173,93					59a, 59b, 61a, 61b			
**N61**	108,01				61a, 61b				
**H61a**	7,252	1H	m		61	60	61b	59a, 59b, 61b	61b
**H61b**	6,813	1H	m		61	59, 60	61a	59a, 59b, 61a	61a
**C62**	34,12			62a, 62b		19, 49a, 49b			
**H62a**	2,770	1H	m (o.l.)	62		19, 20, 49	19	16, 18, 19, 21, 62b, 65, 68	18, 19, 62b
**H62b**	2,641	1H	d 13.8(62a), d 7.8(19)	62		19, 20, 49	19	16, 18, 19, 21, 62a, 65, 68	18, 19, 62a
**C63**	37,40			63a, 63b		22, 65			
**H63a**	2,982	1H	d 13.7(63b), d 4.5(22)	63		22, 23, 64, 65	22, 63b	19, 21, 22, 24, 63b, 65, 68	21, 22, 63b
**H63b**	2,753	1H	m (o.l.)	63		22, 23, 64, 65	22, 63a	19, 21, 22, 24, 63a, 65, 68	21, 22, 63a
**C64**	137,46					22, 63a, 63b, 66			
**C65**	129,19			65		63a, 63b, 67			
**H65**	7,200	2H	m	65		63, 67	66	18, 19, 21, 22, 24, 58a, 58b, 62a, 62b, 63a, 63b, 68	66, 67
**C66**	128,04			66					
**H66**	7,233	2H	m	66		64	65, 67	67	65, 67
**C67**	126,29			67		65			
**H67**	7,164	1H	m	67		65	66	66	65, 66
**C68**	16,84			68		25			
**H68**	1,148	3H	d 6.9(25)	68		23, 25, 26	24, 25	21, 24, 25, 28a, 28b, 29a, 29b, 30a, 30b, 62a, 62b, 63a, 63b, 65, 71	24, 25

**C69**	26,94			69a, 69b		39, 40			
**H69a**	3,144	1H	d 14.9(69b), d 5.2(40)	69		40, 41, 70, 71, 78	40, 69b, 71	34a, 34b, 37, 39, 40, 42, 69b, 71, 77	39, 40, 69b, 71
**H69b**	3,009	1H	d 14.9(69a), d 7.5(40)	69		40, 41, 70, 71, 78	40, 69a, 71	34a, 34b, 36b, 37, 39, 40, 42, 69a, 71, 77	39, 40, 69a, 71
**C70**	109,72					40, 69a, 69b, 71, 72, 77			
**C71**	123,49			71		69a, 69b, 72			
**H71**	7,110	1H	d 2.4(72)	71		70, 73, 78	69a, 69b, 72	31, 36b, 37, 39, 40, 42, 68, 69a, 69b, 72, 82	39, 40, 69a, 69b, 72
**N72**	130,89				72				
**H72**	10,811	1H	d 2.4(71)		72	70, 71, 73, 78	71, 77	39, 71, 74	71
**C73**	135,99					71, 72, 75, 77			
**C74**	111,22			74		76			
**H74**	7,312	1H	d 8.0(75), t 0.9(76, 77)	74		76, 78	75, 76	72, 75, 76	75, 76, 77
**C75**	120,85			75		77			
**H75**	7,049	1H	d 8.0(74), d 6.9(76), d 1.1(77)	75		73, 77	74, 76, 77	74, 76, 77	74, 76, 77
**C76**	118,24			76		74			
**H76**	6,959	1H	d 8.0(77), d 6.9(75), d 0.9(74)	76		74, 78	74, 75, 77	74, 75, 77	74, 75, 77
**C77**	118,27			77		75			
**H77**	7,518	1H	d 8.0(76)	77		70, 73, 75, 78	72, 75, 76	36b, 39, 40, 42, 69a, 69b, 75, 76	74, 75, 76
**C78**	127,46					69a, 69b, 71, 72, 74, 76, 77			
**C79**	36,78			79		43, 80a, 80b, 81, 82			

**H79**	1,675	1H	d 9.4(80b), d 6.9(43), q 6.6(82), d 3.7(80a)	79			43, 80b, 82	42, 43, 45a, 45b, 80a, 80b, 81, 82	42, 43, 80a, 80b, 81, 82
**C80**	24,14			80a, 80b		43, 81, 82			
**H80a**	1,377	1H	d 13.5(80b), q 7.5(81), d 3.7(79)	80		43, 79, 81, 82	80b, 81	42, 79, 80b, 81	42, 43, 79, 80b, 81, 82
**H80b**	1,046	1H	d 13.5(80a), d 8.4(79), q 7.5(81)	80		43, 79, 81, 82	79, 80a, 81	42, 79, 80a, 81	42, 43, 79, 80a, 81, 82
**C81**	11,11			81		80a, 80b			
**H81**	0,800	3H	t 7.5(80a,b)	81		79, 80	80a, 80b	79, 80a, 80b	42, 43, 79, 80a, 80b
**C82**	15,34			82		43, 80a, 80b			
**H82**	0,799	3H	d 6.6(79)	82		43, 79, 80	79	42, 43, 45a, 45b, 71, 79	42, 43, 79, 80a, 80b

^{1}H NMR of **stapled** Ac-Ala-Cys-Leu-Leu-Gln-Cys-Phe-Ala-Pro-Pro-Trp-Ile-NH_2 **(23):**
600 MHz, $(CD_3)_2SO$, 298 K

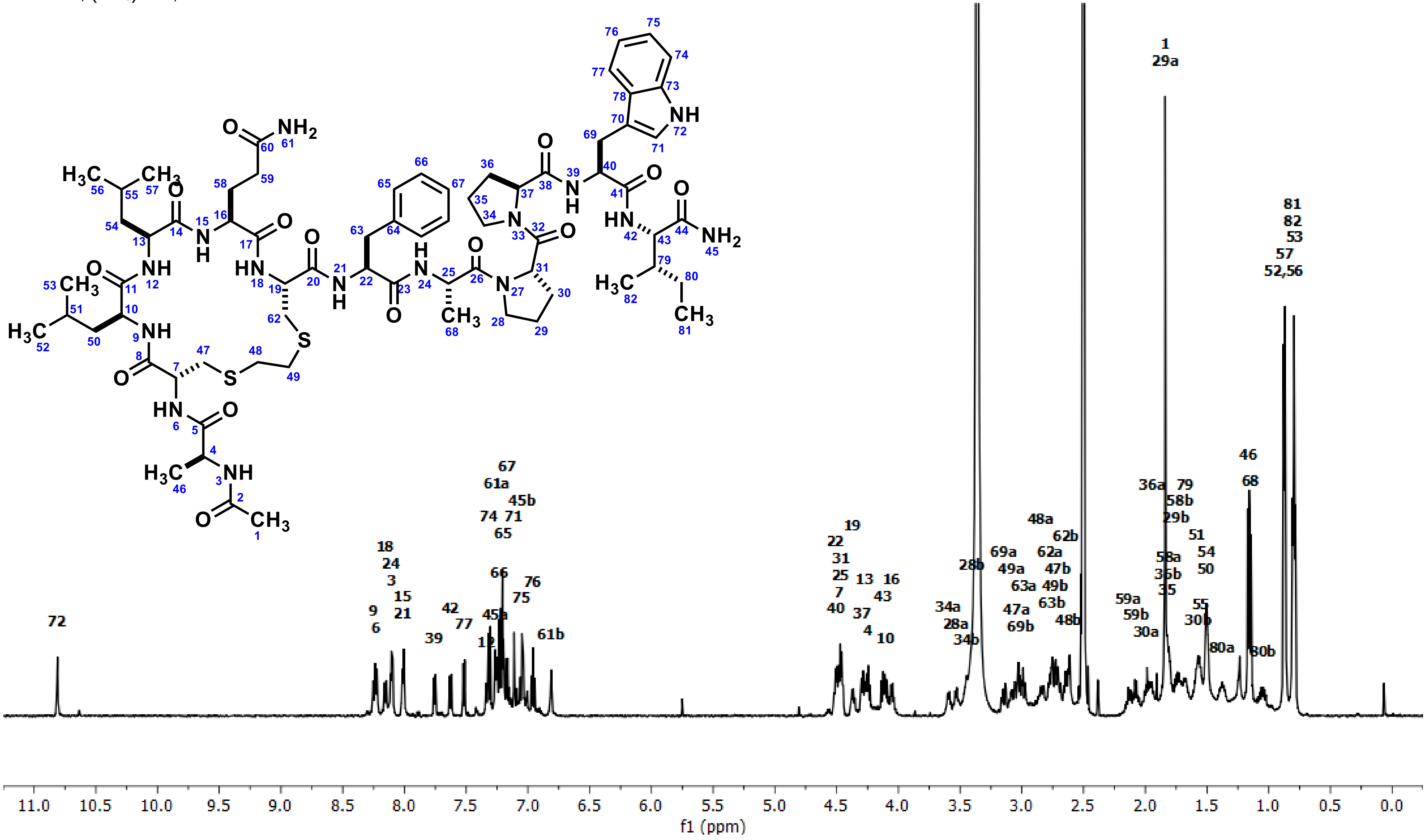

^{13}C NMR of **stapled** Ac-Ala-Cys-Leu-Leu-Gln-Cys-Phe-Ala-Pro-Pro-Trp-Ile-NH_2 **(23):**
151 MHz, $(CD_3)_2SO$, 298 K

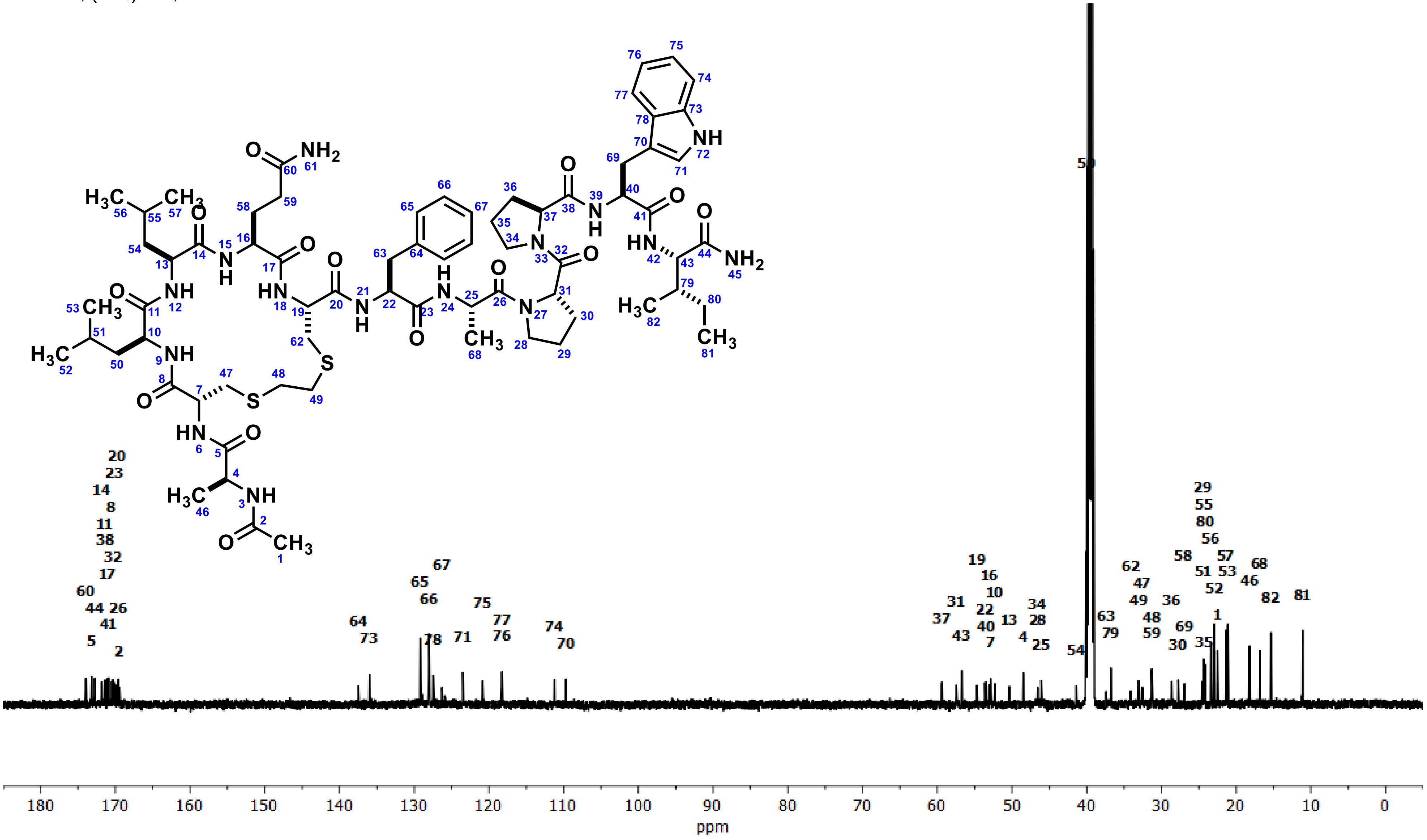

COSY-NMR of **stapled** Ac-Ala-Cys-Leu-Leu-Gln-Cys-Phe-Ala-Pro-Pro-Trp-Ile-NH$_2$ **(23):**
$(CD_3)_2SO$, 298 K

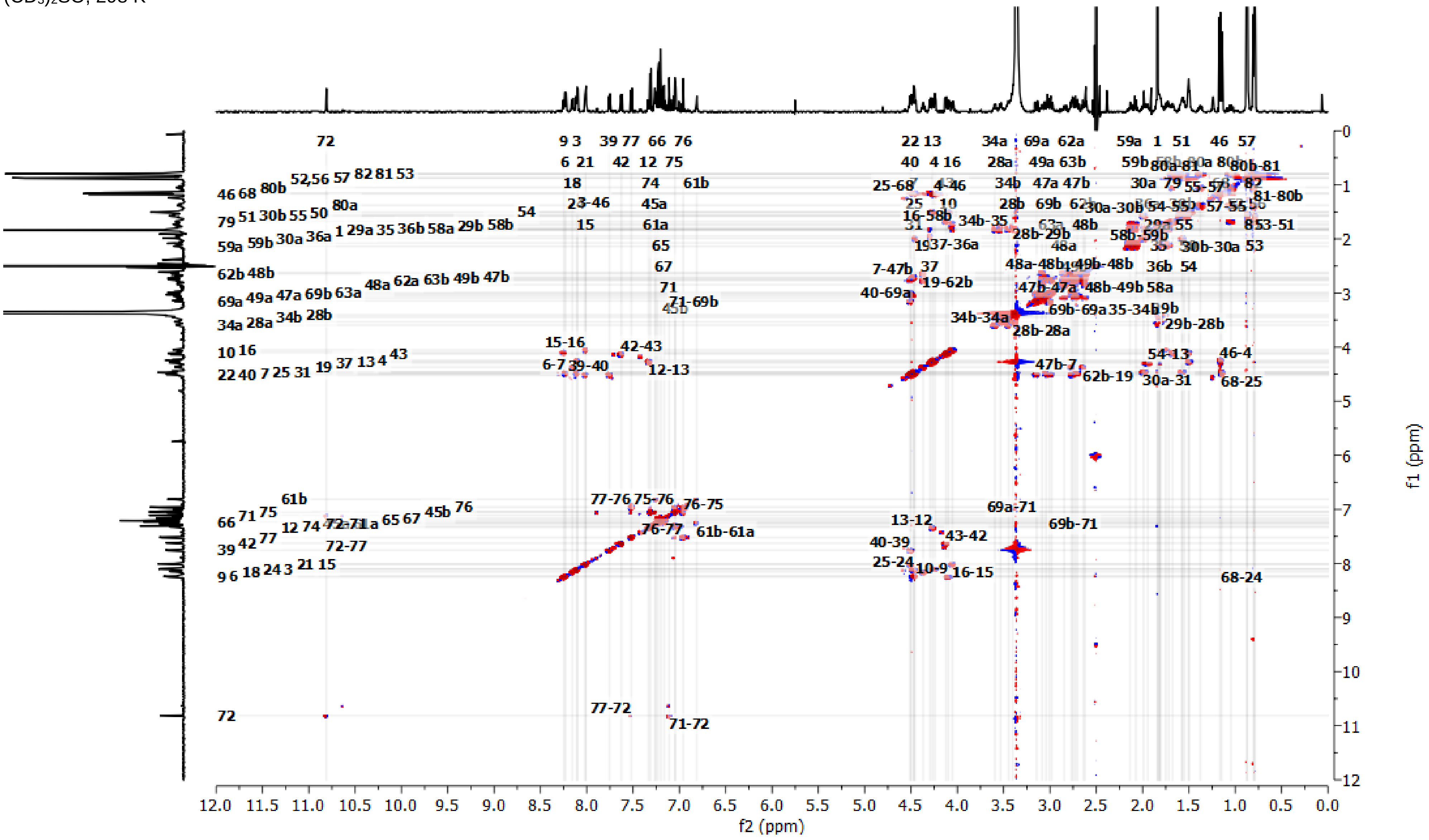

HSQC-NMR of **stapled** Ac-Ala-Cys-Leu-Leu-Gln-Cys-Phe-Ala-Pro-Pro-Trp-Ile-NH_2 **(23):**
$(CD_3)_2SO$, 298 K

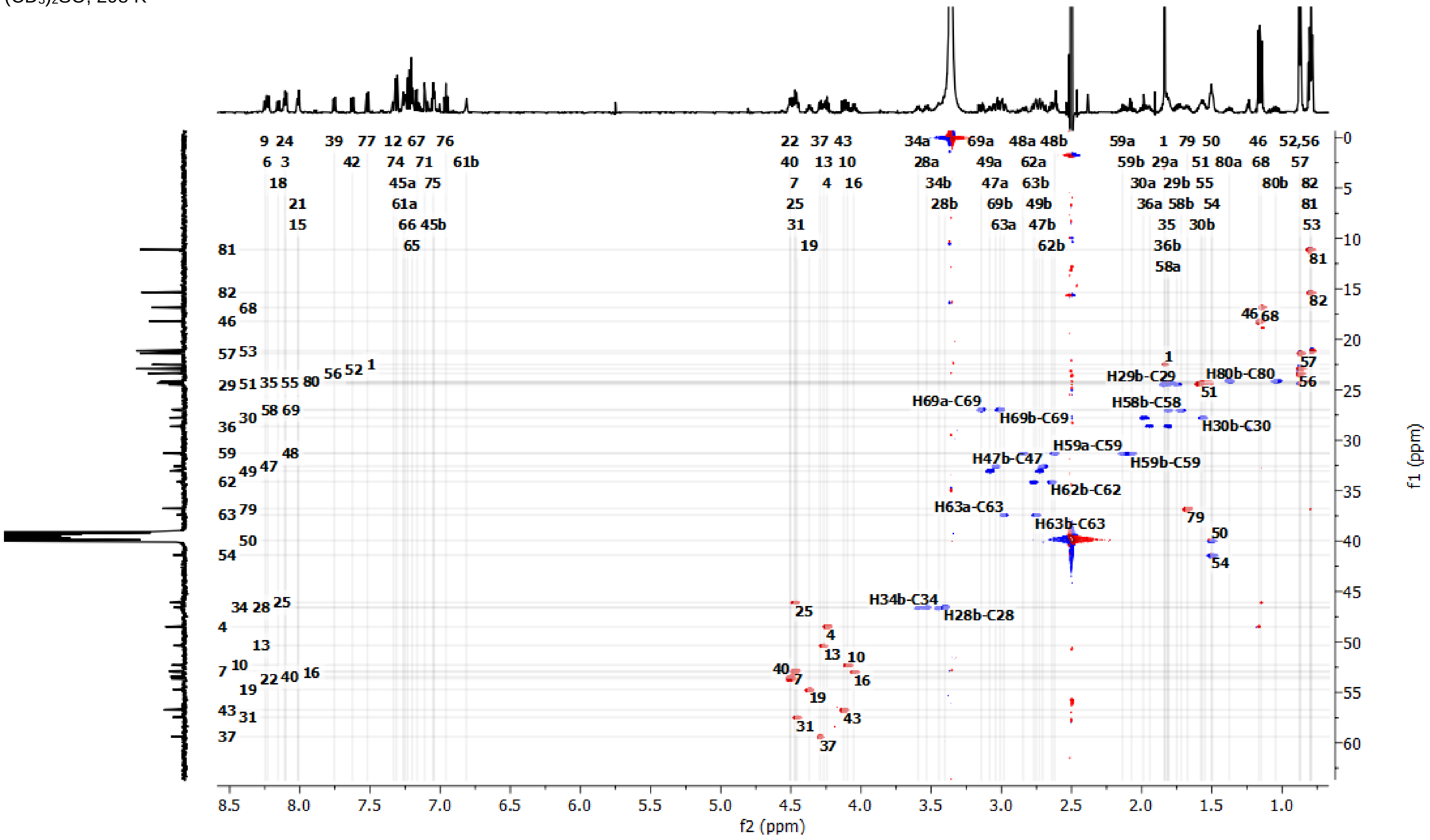

HMBC-NMR of **stapled** Ac-Ala-Cys-Leu-Leu-Gln-Cys-Phe-Ala-Pro-Pro-Trp-Ile-NH_2 **(23):**
$(CD_3)_2SO$, 298 K

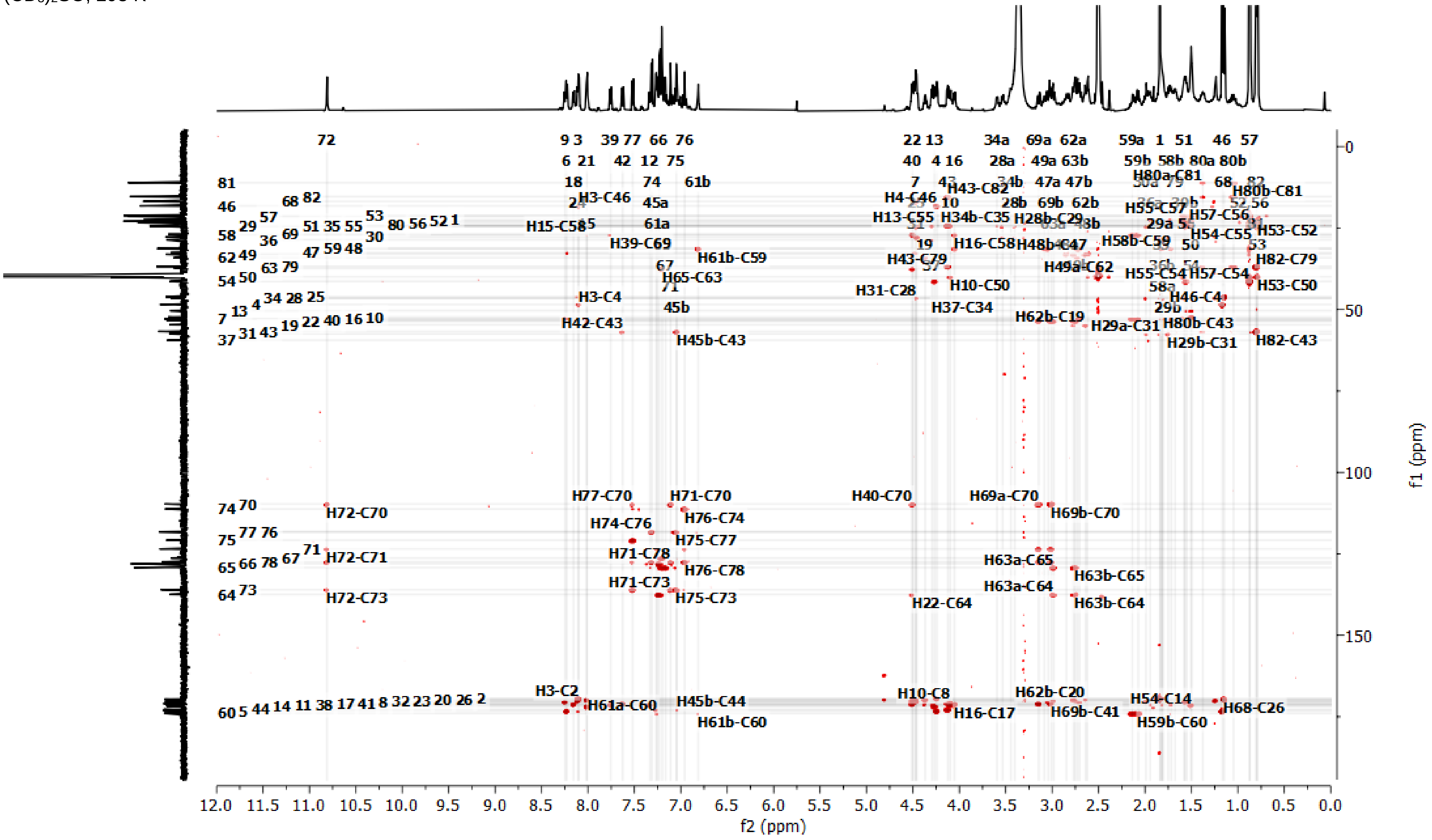

NOESY-NMR of **stapled** Ac-Ala-Cys-Leu-Leu-Gln-Cys-Phe-Ala-Pro-Pro-Trp-Ile-NH_2 **(23):**
$(CD_3)_2SO$, 298 K

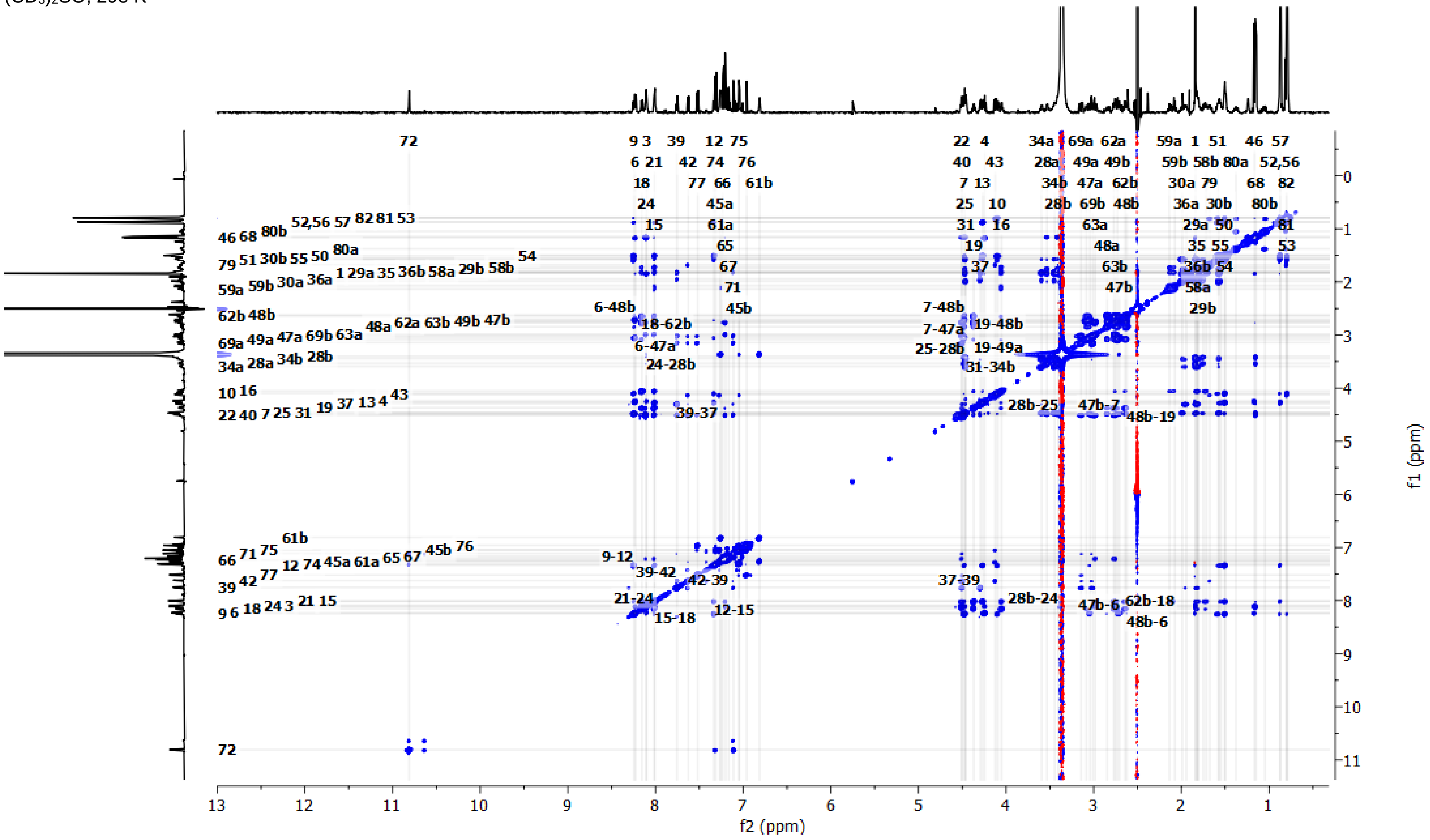

^{15}N-HSQC-NMR of **stapled** Ac-Ala-Cys-Leu-Leu-Gln-Cys-Phe-Ala-Pro-Pro-Trp-Ile-NH_2 **(23):**
$(CD_3)_2SO$, 298 K

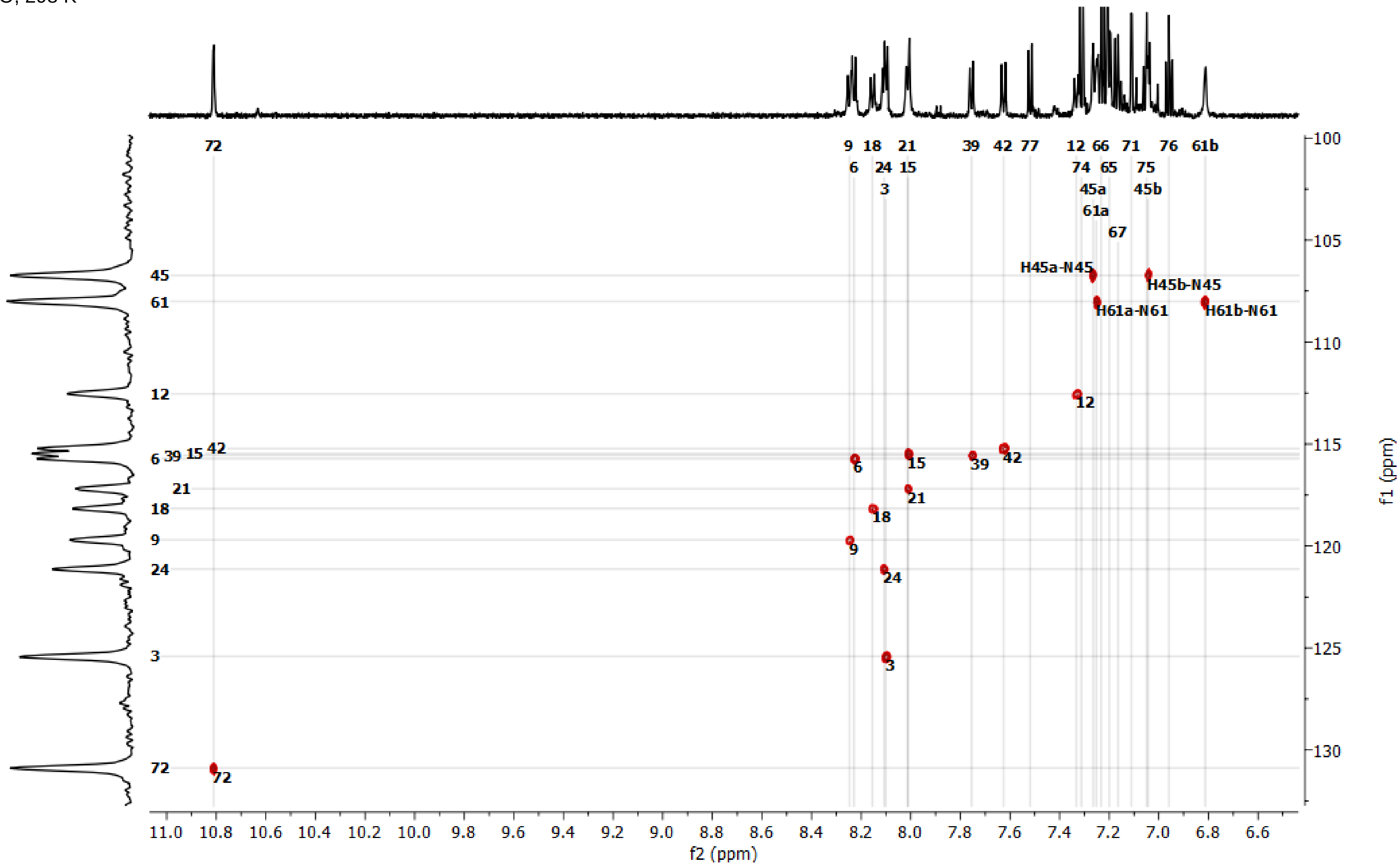

Stapled Ac-Ala-Cys-Leu-Leu-Gln-Lys-Phe-Ala-Pro-Pro-Trp-Ile-NH_2 (24):

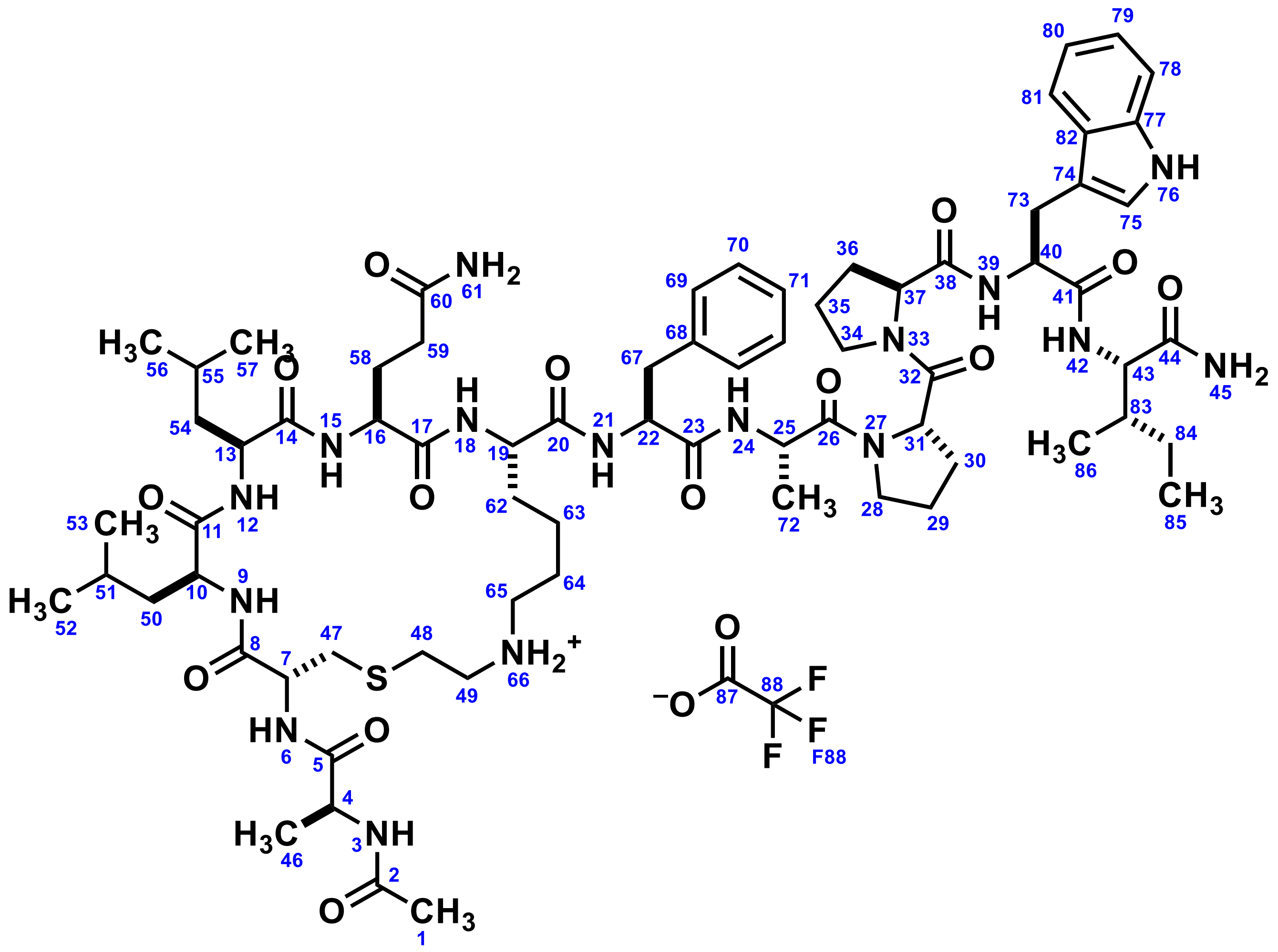

Table S33. Characterization table of stapled Ac-Ala-Cys-Leu-Leu-Gln-Glu-Phe-Ala-Pro-Pro-Trp-Ile-NH_2. The ^{15}N chemical shifts were referenced to NH_3(l).

Atom	δ (ppm)	#	J	HSQC	^{15}N-HSQC	HMBC	COSY	NOESY	TOCSY
**C1**	22,47			1					

**H1**	1,824	3H	s	1		2, 4		3, 6, 9, 46	
**C2**	169,05					1, 3, 4			
**N3**	125,12				3				
**H3**	8,062	1H	d 7.4(4)		3	2, 4, 5, 46	4, 46	1, 4, 6, 46	4, 46
**C4**	48,02			4		1, 3, 46			
**H4**	4,273	1H	quint 7.3(3, 46)	4		2, 5, 46	3, 46	3, 6, 7, 46, 47a, 47b	3, 46
**C5**	172,71					3, 4, 6, 7, 46			
**N6**	115,98				6				
**H6**	8,249	1H	d 7.8(7)		6	5, 7, 47	7	1, 3, 4, 7, 9, 12, 46, 47a, 47b, 48a, 48b, 49, 50a, 50b, 51	7, 47a, 47b
**C7**	52,41			7		6, 47a, 47b			
**H7**	4,433	1H	d 7.8(6), t 7.0(47a,b)	7		5, 8, 47	6, 47a, 47b	4, 6, 9, 10, 12, 47a, 47b, 48a, 48b, 49, 50a, 50b, 51, 54, 66	6, 47a, 47b
**C8**	170,34					7, 9, 10, 47a, 47b			
**N9**	121,37				9				
**H9**	8,403	1H	d 8.3(10)		9	8	10	1, 6, 7, 10, 12, 13, 15, 47a, 47b, 48a, 48b, 49, 50a, 50b, 51, 52, 53, 54, 65a	10, 50a, 50b, 51, 52, 53
**C10**	51,49			10		50a, 50b, 51			
**H10**	4,229	1H	m (o.l.)	10		8, 11, 50, 51	9, 50a, 50b	7, 9, 12, 13, 15, 50a, 50b, 51, 52, 53	9, 50a, 50b, 51, 52, 53
**C11**	171,66					10, 12, 13			
**N12**	115,15				12				
**H12**	7,387	1H	br d ~8(13)		12	11	13	6, 7, 9, 10, 13, 15, 16, 47a, 47b, 48a, 49, 50a, 50b, 51, 52, 53, 54, 55, 56, 57	13, 54, 55, 56, 57
**C13**	50,90			13		54, 55			
**H13**	4,309	1H	q 7.5(12, 54)	13		11, 14, 54, 55	12, 54	9, 10, 12, 15, 16, 18, 50a, 50b, 54, 55, 56, 57, 58a, 58b	12, 54, 55, 56, 57
**C14**	172,17					13, 15, 16, 54			
**N15**	118,62				15				
**H15**	8,101	1H	(br) d 6.9(16)		15	14	16	9, 10, 12, 13, 16, 18, 54, 55, 56, 57, 58a, 58b, 59a, 59b	16, 58a, 58b, 59a, 59b
**C16**	52,60			16		58a, 58b, 59a, 59b			

**H16**	4,091	1H	(br) q 7.1(15, 58a,b)	16		14, 17, 58, 59	15, 58a, 58b	12, 13, 15, 18, 19, 21, 58a, 58b, 59a, 59b, 62a, 62b, 63a, 63b, 64	15, 58a, 58b, 59a, 59b
**C17**	171,05					16, 18, 58a, 58b			
**N18**	117,72				18				
**H18**	7,943	1H	d 8.5(19)		18	17, 19	19	13, 15, 16, 19, 21, 22, 24, 58a, 58b, 59a, 59b, 62a, 62b, 63a, 63b, 64, 65a, 65b, 67a, 67b, 69	19, 62a, 62b, 63a, 63b, 64, 65a, 65b
**C19**	51,92			19		18, 62b, 63a, 63b			
**H19**	4,216	1H	m (o.l.)	19		20, 62	18, 62a, 62b	16, 18, 21, 22, 24, 62a, 62b, 63a, 63b, 64, 65a, 65b, 67a, 67b, 69	18, 62a, 62b, 63a, 63b
**C20**	171,24					19, 21, 22			
**N21**	115,01				21				
**H21**	7,848	1H	d 8.3(22)		21	20, 22	22	16, 18, 19, 22, 24, 62a, 62b, 63a, 63b, 64, 67a, 67b, 69, 72	22, 67a, 67b
**C22**	53,34			22		21, 67a, 67b			
**H22**	4,537	1H	d 9.5(67b), d 8.3(21), d 4.5(67a)	22		20, 23, 67, 68	21, 67a, 67b	18, 19, 21, 24, 25, 67a, 67b, 69, 72	21, 67a, 67b
**C23**	170,34					22, 24, 25, 67a, 67b			
**N24**	121,31				24				
**H24**	8,186	1H	d 7.3(25)		24	23, 25, 72	25, 72	18, 19, 21, 22, 25, 28a, 28b, 62a, 62b, 67a, 67b, 69, 72	25, 72
**C25**	46,10			25		24, 72			
**H25**	4,471	1H	quint 7.1(24, 72)	25		23, 26, 72	24, 72	22, 24, 28a, 28b, 69, 72	24, 72
**C26**	169,65					25, 72			
**N27**	n.f.								
**C28**	46,56			28a, 28b		29a, 29b, 30a, 30b, 31			
**H28a**	3,533	1H	d 9.7(28b/29), t 7.1(28b/29)	28		29, 30, 31	28b, 29a, 29b	24, 25, 28b, 29a, 29b, 30a, 30b, 72	28b, 29a, 29b, 30a, 30b, 31

**H28b**	3,411	1H	d 9.9(28a/29a/b), d 7.2(28a/29a/b), d 6.8(28a/29a/b)	28		29, 30	28a, 29a, 29b	24, 25, 28a, 29a, 29b, 30a, 30b, 72	28a, 29a, 29b, 30a, 30b, 31
**C29**	24,49			29a, 29b		28a, 28b, 30a, 30b, 31			
**H29a**	1,842	1H	m (o.l.)	29		28, 30, 31	28a, 28b, 29b, 30a, 30b	28a, 28b, 30a, 30b, 31	28a, 28b, 29b, 30a, 30b, 31
**H29b**	1,753	1H	m (o.l.)	29		28, 30, 31	28a, 28b, 29a, 30a, 30b	28a, 28b, 30a, 30b, 31	28a, 28b, 29a, 30a, 30b, 31
**C30**	27,75			30a, 30b		28a, 28b, 29a, 29b, 31			
**H30a**	1,997	1H	d 12.7(29/30b), q 8.0(29/30b/31)	30		28, 29, 31, 32	29a, 29b, 30b, 31	28a, 28b, 29a, 29b, 30b, 31, 34a, 34b	28a, 28b, 29a, 29b, 30b, 31
**H30b**	1,578	1H	m (o.l.)	30		28, 29, 31, 32	29a, 29b, 30a, 31	28a, 28b, 29a, 29b, 30a, 31, 34a, 34b, 39, 75	28a, 28b, 29a, 29b, 30a, 31
**C31**	57,47			31		28a, 29a, 29b, 30a, 30b			
**H31**	4,468	1H	d 8.3(30a), d 4.8(30b)	31		28, 29, 30, 32	30a, 30b	29a, 29b, 30a, 30b, 34a, 34b, 37, 39	28a, 28b, 29a, 29b, 30a, 30b
**C32**	170,28					30a, 30b, 31			
**N33**	n.f.								
**C34**	46,59			34a, 34b		37			
**H34a**	3,590	1H	d 9.5(34b/35), t 7.1(34b/35)	34		35, 36	34b, 35	30a, 30b, 31, 34b, 35, 36a, 37, 39	34b, 35, 36a, 36b, 37
**H34b**	3,453	1H	m	34		35, 36	34a, 35	30a, 30b, 31, 34a, 35, 36a, 37, 39	34a, 35, 36a, 36b, 37

**C35**	24,30			35		34a, 34b, 36a, 37			
**H35**	1,820	2H	m (o.l.)	35		36, 37	34a, 34b	34a, 34b, 36a	34a, 34b, 37
**C36**	28,64			36a, 36b		34a, 34b, 35, 37			
**H36a**	1,946	1H	m	36		35, 37, 38	36b, 37	34a, 34b, 35, 36b, 37, 39, 75	34a, 34b, 36b, 37
**H36b**	1,820	1H	m (o.l.)	36		37, 38	36a, 37	36a, 37, 39, 75	34a, 34b, 36a, 37
**C37**	59,39			37		35, 36a, 36b			
**H37**	4,294	1H	d 8.4(36a/b), d 3.1(36a/b)	37		34, 35, 36, 38	36a, 36b	31, 34a, 34b, 36a, 36b, 39, 40, 42, 73a, 73b, 75, 81	34a, 34b, 35, 36a, 36b
**C38**	171,33					36a, 36b, 37, 39, 40			
**N39**	115,58				39				
**H39**	7,762	1H	d 7.6(40)		39	38, 40, 73	40	30b, 31, 34a, 34b, 36a, 36b, 37, 40, 42, 73a, 73b, 75, 76, 81	40, 73a, 73b, 75
**C40**	53,44			40		39, 73a, 73b			
**H40**	4,508	1H	t 7.7(39, 73b), d 5.5(73a)	40		38, 41, 73, 74	39, 73a, 73b	37, 39, 42, 73a, 73b, 75, 81	39, 73a, 73b, 75
**C41**	170,90					40, 42, 43, 73a, 73b			
**N42**	115,26				42				
**H42**	7,636	1H	d 9.0(43)		42	41, 43, 44, 83	43	37, 39, 40, 43, 45a, 45b, 73a, 73b, 75, 81, 83, 84b	43, 83, 84a, 84b, 85, 86
**C43**	56,73			43		42, 45b, 83, 84a, 84b, 86			
**H43**	4,127	1H	d 9.0(42), d 6.9(83)	43		41, 44, 83, 84, 86	42, 83	42, 45a, 45b, 75, 81, 83, 86	42, 83, 84a, 84b, 85, 86
**C44**	172,75					42, 43, 45a, 45b			
**N45**	106,74				45a, 45b				
**H45a**	7,271	1H	(br) d 2.0(45b)		45	44	45b	42, 43, 83	45b

**H45b**	7,045	1H	m (o.l.)		45	43, 44	45a	42, 43, 83	45a
**C46**	18,20			46		3, 4			
**H46**	1,139	3H	d 7.1(4)	46		4, 5	3, 4	1, 3, 4, 6, 47a, 47b, 52, 53	3, 4
**C47**	32,63			47a, 47b		6, 7, 48a, 48b			
**H47a**	2,824	1H	d 14.0(47b), d 7.0(7)	47		7, 8, 48	7, 47b	4, 6, 7, 9, 12, 46, 47b, 49	6, 7, 47b
**H47b**	2,709	1H	d 14.0(47a), d 7.0(7)	47		7, 8, 48	7, 47a	4, 6, 7, 9, 12, 46, 47a, 48a, 49	6, 7, 47a
**C48**	27,02			48a, 48b		47a, 47b			
**H48a**	2,881	1H	m (o.l.)	48		47, 49	48b, 49	6, 7, 9, 12, 47b, 48b, 49	48b, 49
**H48b**	2,791	1H	m (o.l.)	48		47, 49	48a, 49	6, 7, 9, 48a, 49, 66	48a, 49
**C49**	45,88			49		48a, 48b			
**H49**	3,185	2H	br m	49			48a, 48b	6, 7, 9, 12, 47a, 47b, 48a, 48b, 64, 65a, 65b, 66	48a, 48b
**C50**	40,23			50a, 50b		10, 51, 52, 53			
**H50a**	1,567	1H	m (o.l.)	50		10, 51, 52, 53	10, 50b	6, 7, 9, 10, 12, 13, 52, 53	9, 10, 50b, 52, 53
**H50b**	1,477	1H	m (o.l.)	50		10, 53	10, 50a	6, 7, 9, 10, 12, 13, 52, 53	9, 10, 50a, 52, 53
**C51**	24,18			51		10, 50a, 52, 53			
**H51**	1,611	1H	m (o.l.)	51		10, 50, 52, 53	52, 53	6, 7, 9, 10, 12, 52, 53	9, 10, 52, 53
**C52**	23,09			52		50a, 51, 53			
**H52**	0,881	3H	d 6.4(51)	52		50, 51, 53	51	9, 10, 12, 46, 50a, 50b, 51, 53	9, 10, 50a, 50b, 51, 53
**C53**	20,95			53		50a, 50b, 51, 52			
**H53**	0,805	3H	d 6.3(51)	53		50, 51, 52	51	9, 10, 12, 46, 50a, 50b, 51, 52	9, 10, 50a, 50b, 51, 52
**C54**	41,27			54		13, 55, 56, 57			
**H54**	1,415	2H	m (o.l.)	54		13, 14, 55, 56, 57	13, 55	7, 9, 12, 13, 15, 55, 56, 57	12, 13, 55, 56, 57
**C55**	23,99			55		13, 54, 56, 57			

**H55**	1,627	1H	m (o.l.)	55		13, 54, 56, 57	54, 56, 57	12, 13, 15, 54, 56, 57	12, 13, 54, 56, 57
**C56**	23,14			56		54, 55, 57			
**H56**	0,883	3H	d 6.6(55)	56		54, 55, 57	55	12, 13, 15, 54, 55	12, 13, 54, 55, 57
**C57**	21,62			57		54, 55, 56			
**H57**	0,849	3H	d 6.6(55)	57		54, 55, 56	55	12, 13, 15, 54, 55	12, 13, 54, 55, 56
**C58**	26,77			58a, 58b		16, 59a, 59b			
**H58a**	1,788	1H	m (o.l.)	58		16, 17, 59, 60	16, 58b, 59a, 59b	13, 15, 16, 18, 59a, 59b, 61b, 69	15, 16, 58b, 59a, 59b
**H58b**	1,733	1H	m (o.l.)	58		16, 17, 59, 60	16, 58a, 59a, 59b	13, 15, 16, 18, 59a, 59b, 61b, 69	15, 16, 58a, 59a, 59b
**C59**	31,53			59a, 59b		16, 58a, 58b, 61b			
**H59a**	2,143	1H	d 15.6(59b), d 9.6(58a/b), d 6.3(58a/b)	59		16, 58, 60	58a, 58b, 59b	15, 16, 18, 58a, 58b, 59b, 61a, 61b	15, 16, 58a, 58b, 59b
**H59b**	2,073	1H	d 15.6(59a), d 9.6(58a/b), d 6.0(58a/b)	59		16, 58, 60	58a, 58b, 59a	15, 16, 18, 58a, 58b, 59a, 61a, 61b	15, 16, 58a, 58b, 59a
**C60**	173,68					58a, 58b, 59a, 59b, 61a, 61b			
**N61**	107,83				61a, 61b				
**H61a**	7,255	1H	(br) d 2.3(61b)		61	60	61b	59a, 59b, 61b	61b
**H61b**	6,806	1H	(br) d 2.3(61a)		61	59, 60	61a	58a, 58b, 59a, 59b, 61a	61a
**C62**	30,55			62a, 62b		19, 63a, 63b			
**H62a**	1,613	1H	m (o.l.)	62			19, 62b, 63a, 63b	16, 18, 19, 21, 24, 62b, 63a, 63b, 65a, 65b, 69	18, 19, 63a, 63b

**H62b**	1,431	1H	m (o.l.)	62		19	19, 62a, 63a, 63b	16, 18, 19, 21, 24, 62a, 63a, 63b, 65a, 65b, 69	18, 19, 63a, 63b
**C63**	22,26			63a, 63b					
**H63a**	1,304	1H	m	63		19, 62, 65	62a, 62b, 63b, 64	16, 18, 19, 21, 62a, 62b, 63b, 64, 65a, 65b	18, 19, 62a, 62b, 63b, 64, 65a, 65b
**H63b**	1,248	1H	m (o.l.)	63		19, 62, 65	62a, 62b, 63a, 64	16, 18, 19, 21, 62a, 62b, 63a, 64, 65a, 65b	18, 19, 62a, 62b, 63a, 64, 65b
**C64**	24,30			64					
**H64**	1,505	2H	m (o.l.)	64		65	63a, 63b, 65a, 65b	16, 18, 19, 21, 49, 63a, 63b, 65a, 65b, 66	18, 63a, 63b, 65a, 65b
**C65**	46,63			65a, 65b		63a, 63b, 64			
**H65a**	2,934	1H	br m	65			64, 65b	9, 18, 19, 49, 62a, 62b, 63a, 63b, 64, 65b	18, 63a, 64, 65b
**H65b**	2,839	1H	m (o.l.)	65			64, 65a	18, 19, 49, 62a, 62b, 63a, 63b, 64, 65a, 66	18, 63a, 63b, 64, 65a
**N66**	n.f.								
**H66**	8,400	2H	br! s (o.l.)					7, 48b, 49, 64, 65b	
**C67**	37,51			67a, 67b		22, 69			
**H67a**	2,988	1H	d 13.8(67b), d 4.5(22)	67		22, 23, 68, 69	22, 67b	18, 19, 21, 22, 24, 67b, 69, 72	21, 22, 67b
**H67b**	2,731	1H	d 13.8(67a), d 9.5(22)	67		22, 23, 68, 69	22, 67a	18, 19, 21, 22, 24, 67a, 69, 72	21, 22, 67a
**C68**	137,55					22, 67a, 67b, 70			
**C69**	129,21			69		67a, 67b, 71			
**H69**	7,216	2H	m (o.l.)	69		67, 71		18, 19, 21, 22, 24, 25, 58a, 58b, 62a, 62b, 67a, 67b, 72	71
**C70**	127,99			70					
**H70**	7,227	2H	m (o.l.)	70		68	71		71
**C71**	126,27			71		69			

**H71**	7,166	1H	m	71		69	70		69, 70
**C72**	16,82			72		24, 25			
**H72**	1,156	3H	d 6.9(25)	72		25, 26	24, 25	21, 22, 24, 25, 28a, 28b, 67a, 67b, 69	24, 25
**C73**	26,97			73a, 73b		39, 40, 75			
**H73a**	3,147	1H	d 15.0(73b), d 5.3(40)	73		40, 41, 74, 75, 82	40, 73b, 75	37, 39, 40, 42, 73b, 75, 81	39, 40, 73b, 75
**H73b**	3,009	1H	d 15.0(73a), d 7.7(40)	73		40, 41, 74, 75, 82	40, 73a, 75	37, 39, 40, 42, 73a, 75, 81	39, 40, 73a, 75
**C74**	109,73					40, 73a, 73b, 75, 76, 81			
**C75**	123,49			75		73a, 73b, 76			
**H75**	7,112	1H	d 2.4(76)	75		73, 74, 77, 82	73a, 73b, 76	30b, 36a, 36b, 37, 39, 40, 42, 43, 73a, 73b, 76	39, 40, 73a, 73b, 76
**N76**	130,91				76				
**H76**	10,820	1H	d 2.4(75)		76	74, 75, 77, 82	75, 81	39, 75, 78	75
**C77**	136,00					75, 76, 79, 81			
**C78**	111,23			78		80			
**H78**	7,313	1H	d 8.1(79), t 0.9(80, 81)	78		80, 82	79, 80	76	79, 80, 81
**C79**	120,85			79		81			
**H79**	7,050	1H	d 8.1(78), d 7.0(80), d 1.0(81)	79		77, 81	78, 80, 81		78, 80, 81
**C80**	118,24			80		78			
**H80**	6,960	1H	d 7.9(81), d 7.0(79), d 0.9(78)	80		78, 82	78, 79, 81	81	78, 79, 81
**C81**	118,28			81		79			
**H81**	7,521	1H	d 7.9(80), q 0.9(76, 78, 79)	81		74, 77, 79, 82	76, 79, 80	37, 39, 40, 42, 43, 73a, 73b, 80	78, 79, 80
**C82**	127,47					73a, 73b, 75, 76, 78, 80, 81			

**C83**	36,79			83		42, 43, 84a, 84b, 85, 86			
**H83**	1,678	1H	d 9.4(84b), quint 6.8(43, 86), d 3.7(84a)	83		43, 85, 86	43, 84a, 84b, 86	42, 43, 45a, 45b, 86	42, 43, 84a, 84b, 85, 86
**C84**	24,15			84a, 84b		43			
**H84a**	1,379	1H	d 13.5(84b), q 7.4(85), d 3.7(83)	84		43, 83, 85, 86	83, 84b, 85	84b, 85	42, 43, 83, 84b, 85, 86
**H84b**	1,047	1H	d 13.5(84a), d 9.4(83), q 7.4(85)	84		43, 83, 85, 86	83, 84a, 85	42, 84a, 85	42, 43, 83, 84a, 85, 86
**C85**	11,11			85		83, 84a, 84b			
**H85**	0,801	3H	t 7.4(84a,b)	85		83	84a, 84b	84a, 84b	42, 43, 83, 84a, 84b
**C86**	15,35			86		43, 83, 84a, 84b			
**H86**	0,801	3H	d 6.8(83)	86		43, 83	83	43, 83	42, 43, 83, 84a, 84b
**C87**	158,10		q 31(F88)						
**C88**	n.f.								
**F88**	-73,55		s						

^{1}H NMR of **stapled** Ac-Ala-Cys-Leu-Leu-Gln-Lys-Phe-Ala-Pro-Pro-Trp-Ile-NH_2 **(24):**
600 MHz, $(CD_3)_2SO$, 298 K

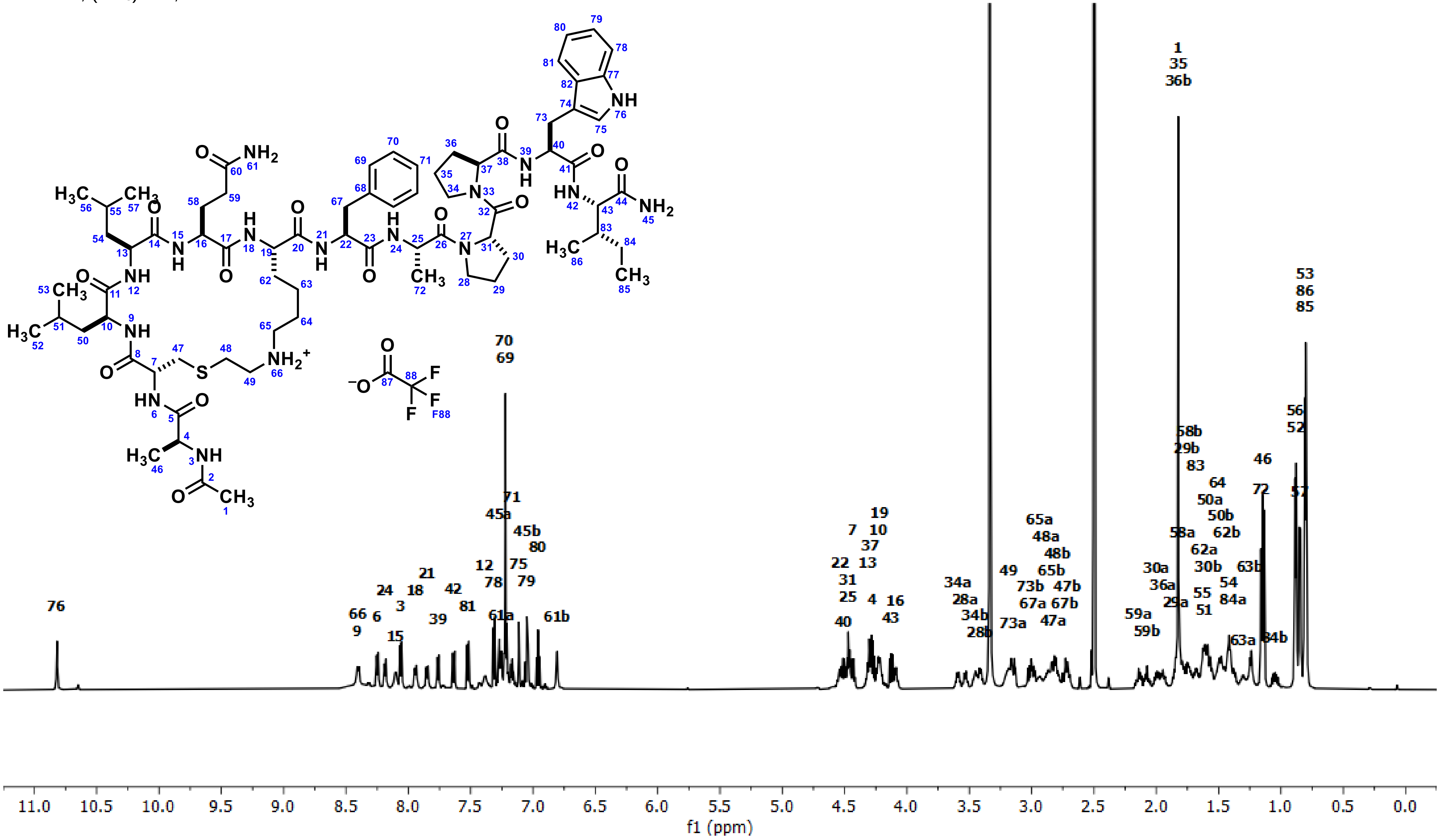

^{13}C NMR of **stapled** Ac-Ala-Cys-Leu-Leu-Gln-Lys-Phe-Ala-Pro-Pro-Trp-Ile-NH_2 **(24):**
151 MHz, $(CD_3)_2SO$, 298 K

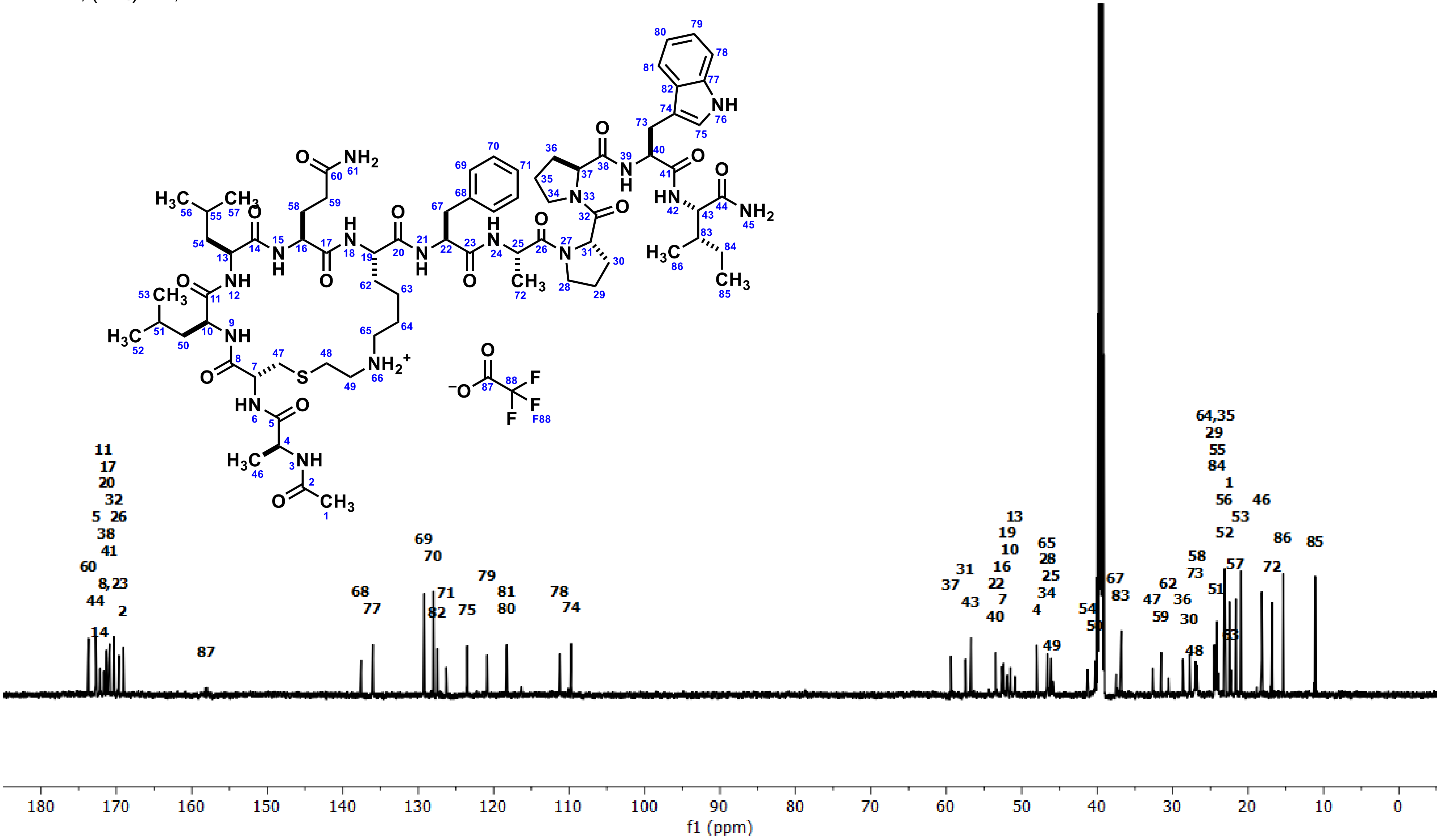

^{19}F NMR of **stapled** Ac-Ala-Cys-Leu-Leu-Gln-Lys-Phe-Ala-Pro-Pro-Trp-Ile-NH_2 **(24):**
151 MHz, $(CD_3)_2SO$, 298 K

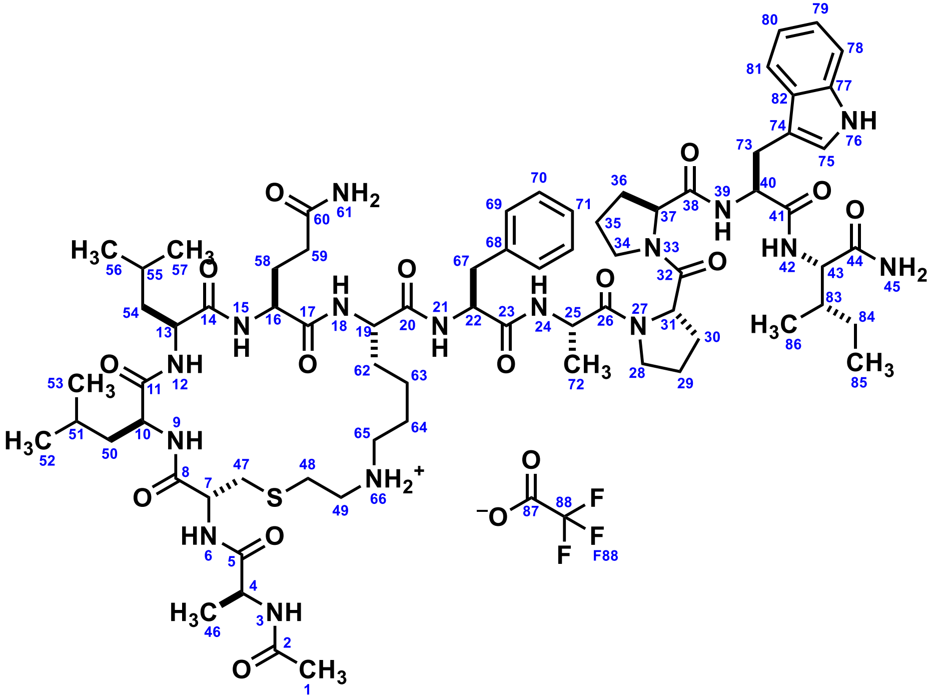

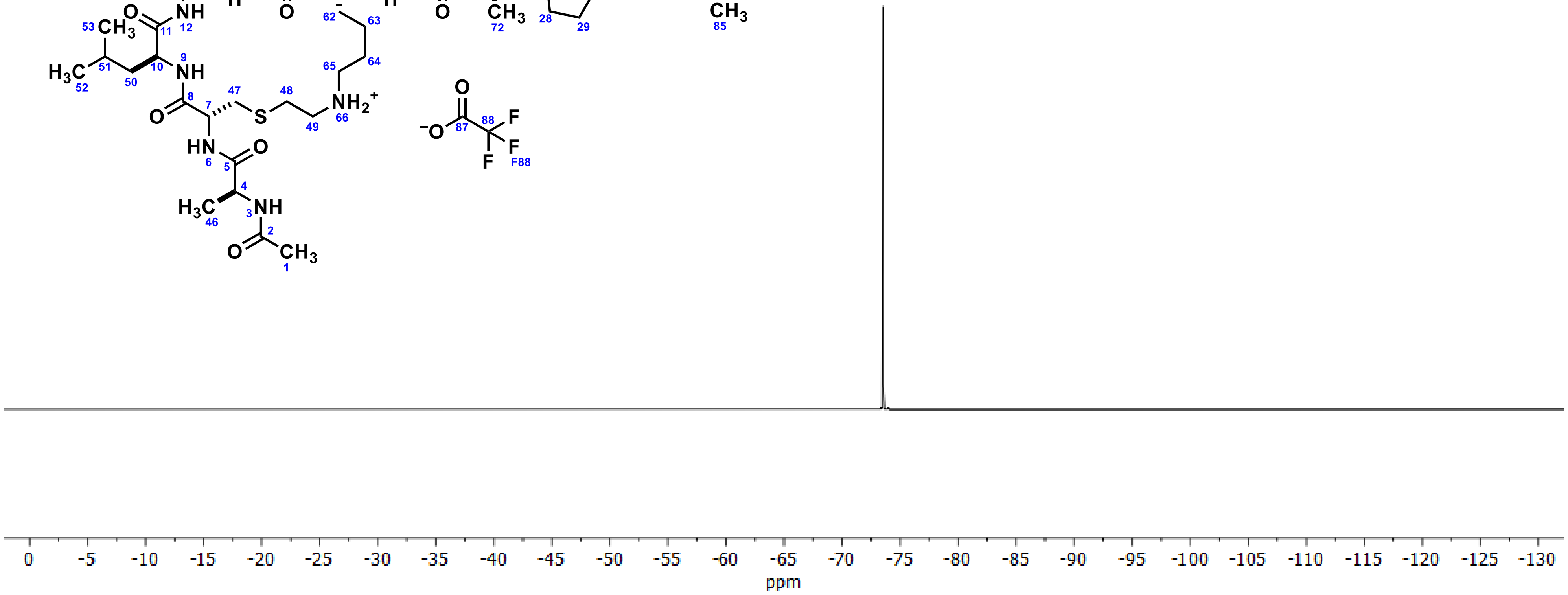

COSY-NMR of **stapled** Ac-Ala-Cys-Leu-Leu-Gln-Lys-Phe-Ala-Pro-Pro-Trp-Ile-NH_2 **(24):**
$(CD_3)_2SO$, 298 K

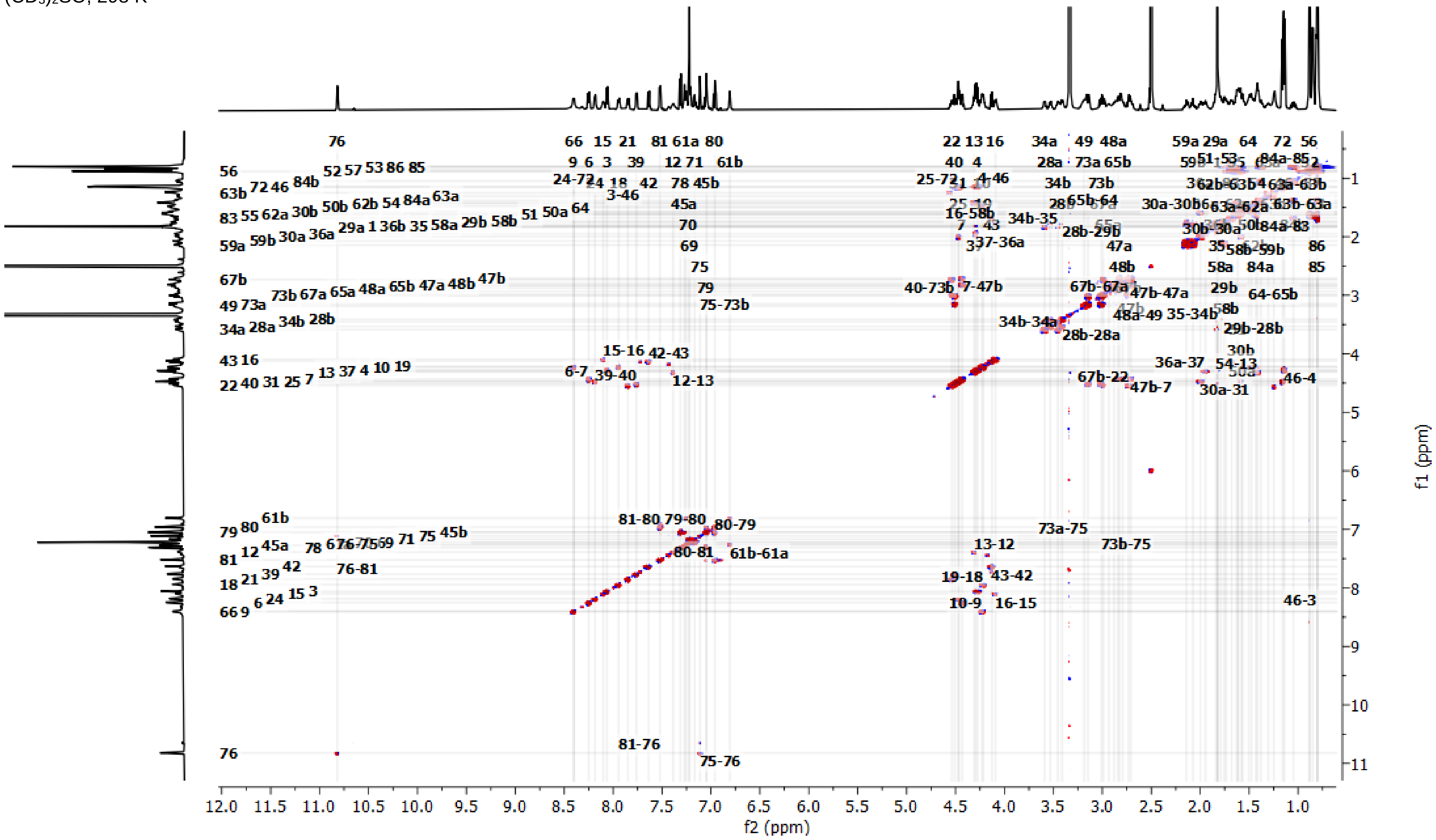

HSQC-NMR of **stapled** Ac-Ala-Cys-Leu-Leu-Gln-Lys-Phe-Ala-Pro-Pro-Trp-Ile-NH2 **(24):**
$(CD_3)_2SO$, 298 K

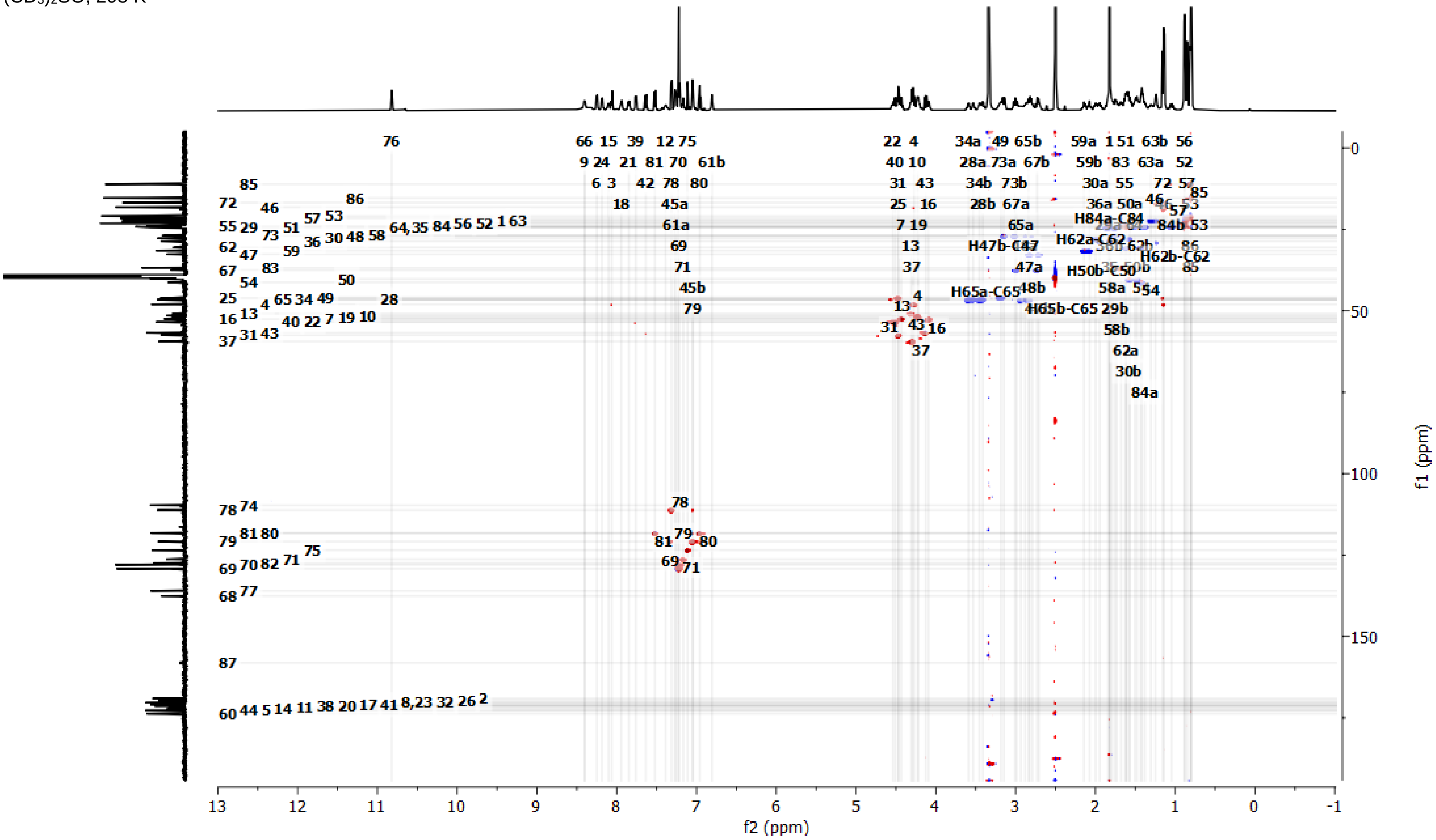

HMBC-NMR of **stapled** Ac-Ala-Cys-Leu-Leu-Gln-Lys-Phe-Ala-Pro-Pro-Trp-Ile-NH_2 **(24):**
$(CD_3)_2SO$, 298 K

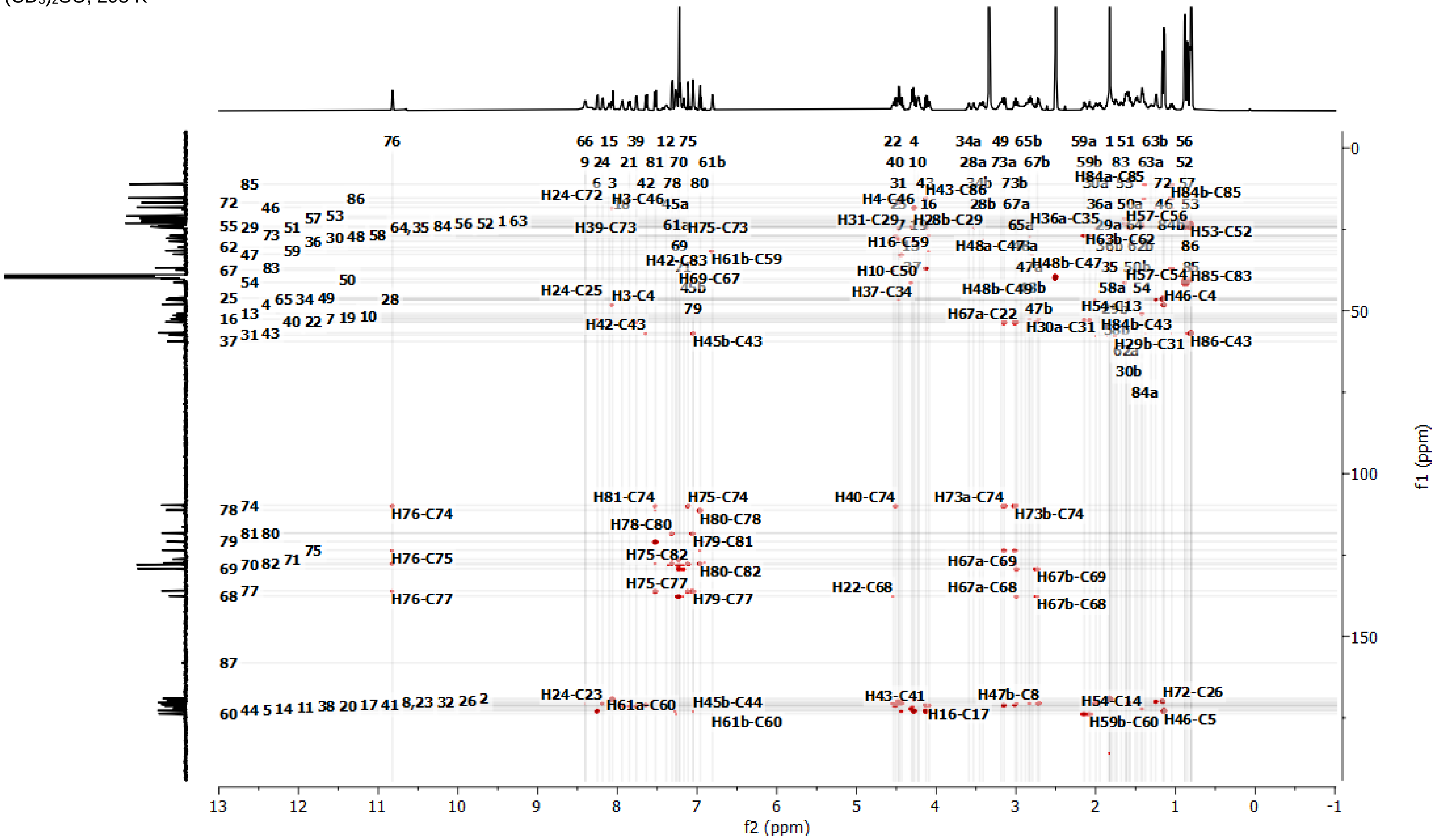

TOCSY-NMR of **stapled** Ac-Ala-Cys-Leu-Leu-Gln-Lys-Phe-Ala-Pro-Pro-Trp-Ile-NH_2 **(24):**
$(CD_3)_2SO$, 298 K

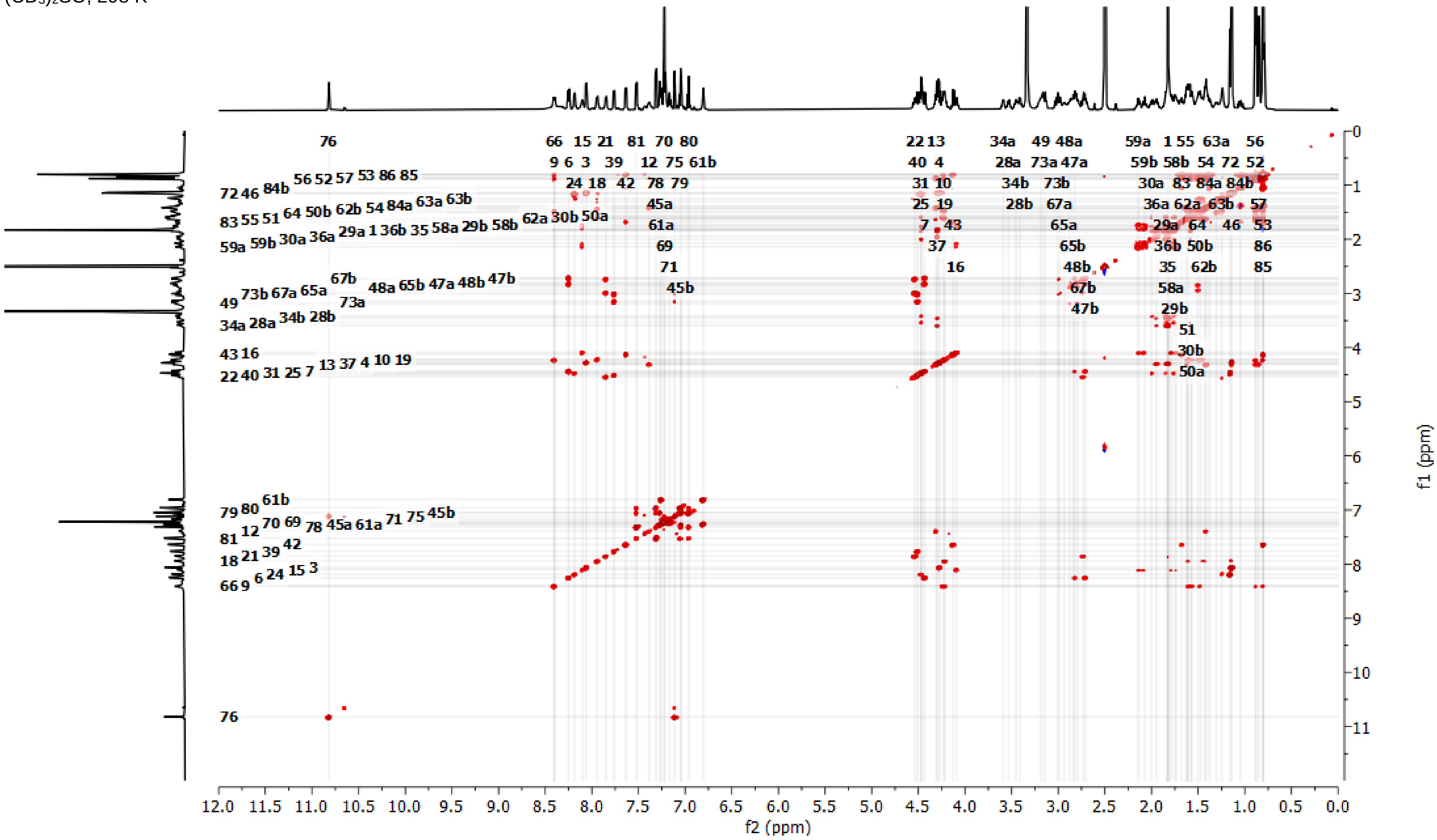

NOESY-NMR of **stapled** Ac-Ala-Cys-Leu-Leu-Gln-Lys-Phe-Ala-Pro-Pro-Trp-Ile-NH_2 **(24):**
$(CD_3)_2SO$, 298 K

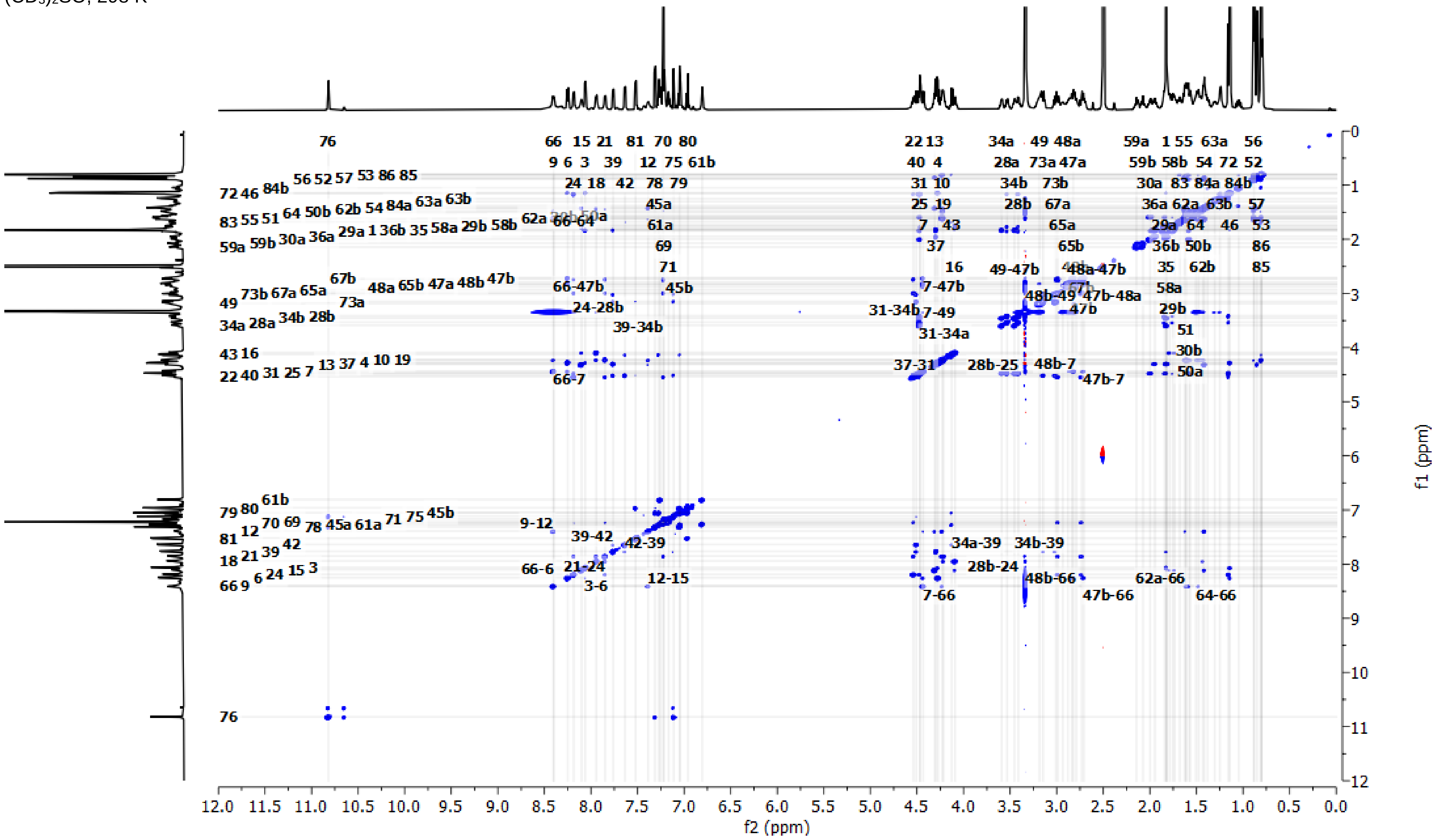

^{15}N-HSQC-NMR of **stapled** Ac-Ala-Cys-Leu-Leu-Gln-Lys-Phe-Ala-Pro-Pro-Trp-Ile-NH$_2$ **(24):**
$(CD_3)_2SO$, 298 K

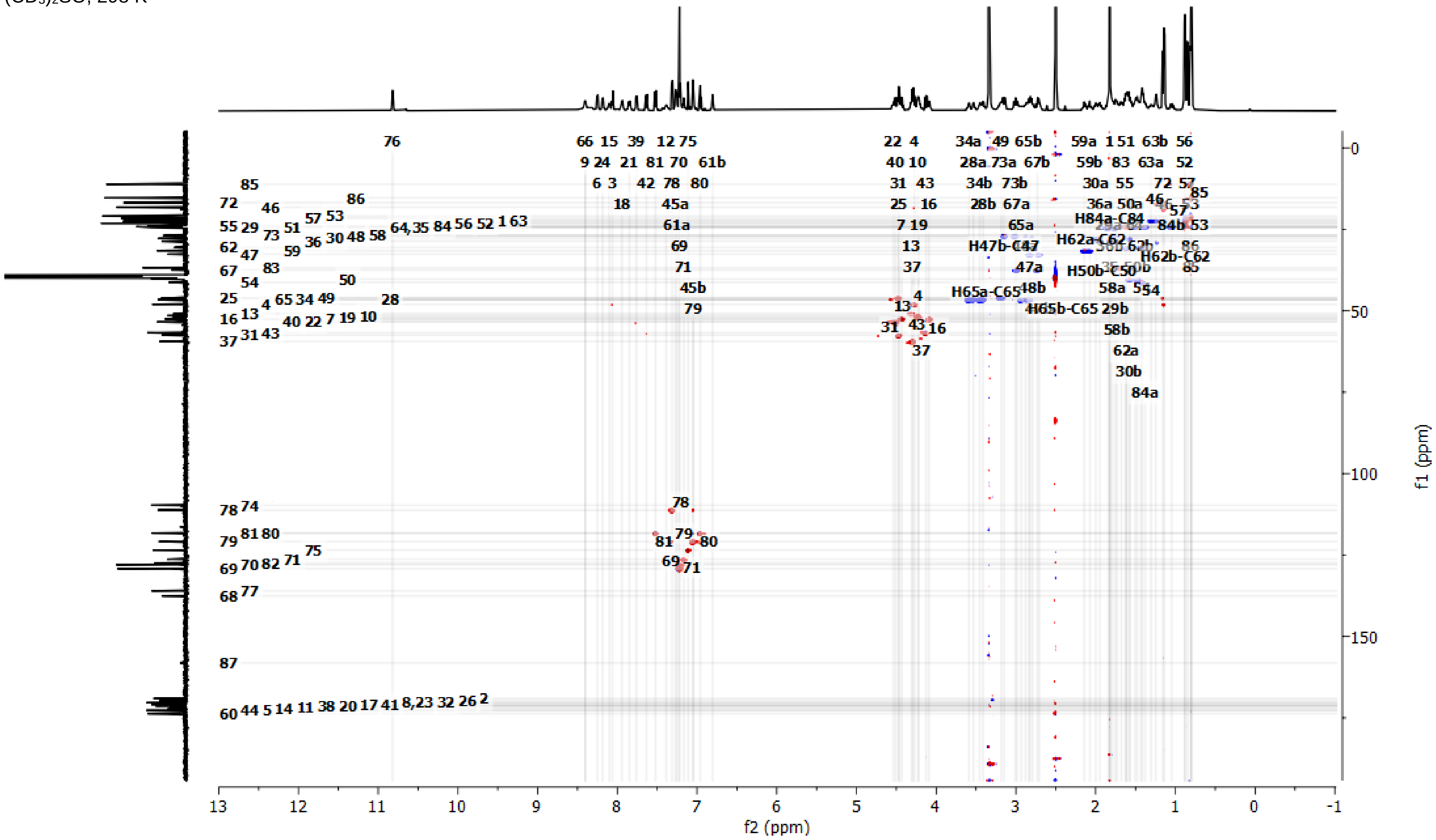

Ethylene-bridged Lypressin (25)

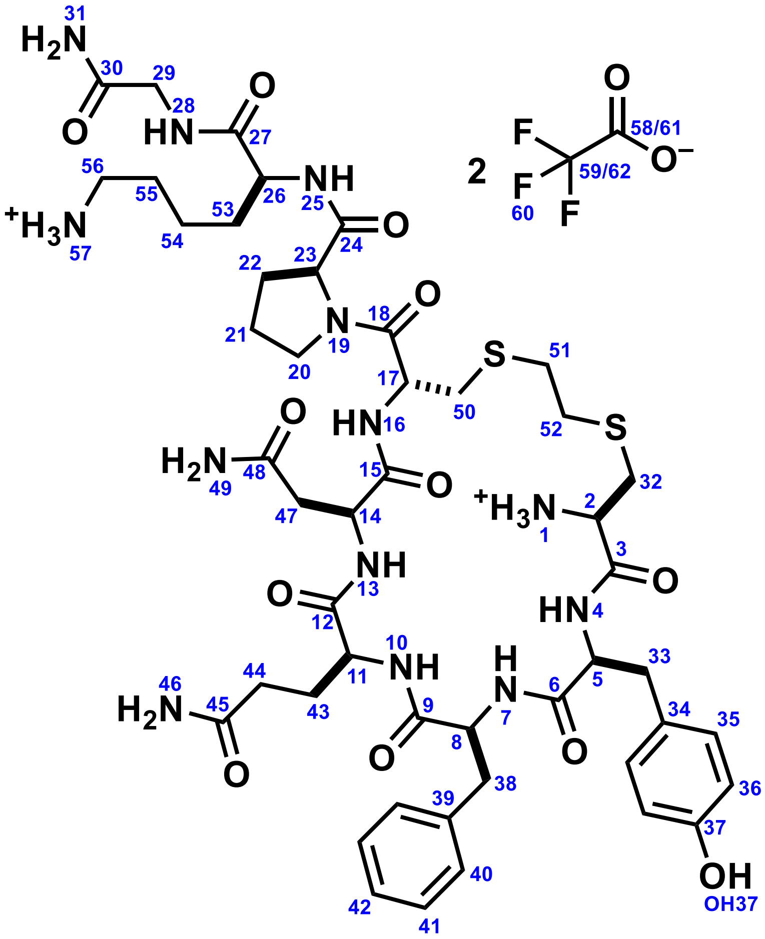

Table S34. Characterization table of ethylene-bridged Lypressin. The ^{15}N chemical shifts were referenced to NH_3(l).

Atom	δ (ppm)	J	HSQC	^{15}N-HSQC	HMQC	COSY	ROESY
**N1**	n.f.						
**H1**	7.88	br! s (o.l.)					
**C2**	52.59		2		32a, 32b		

**H2**	4.423	t 5.8 (32a,b)	2		3, 32	32a, 32b	4
**C3**	168.34				2, 32a, 32b		
**N4**	121.18			4			
**H4**	9.223	d 8.4(5)		4		5	2, 35
**C5**	56.00		5		33b		
**H5**	4.627	d 10.0(33b), d 8.4(4), d 5.3(33a)	5		6	4, 33a, 33b	7, 35
**C6**	172.91				5, 8, 33b		
**N7**	119.04			7			
**H7**	8.851	o.l., d ~5(8)		7		8	5, 8, 35, 38b
**C8**	57.06		8		38a, 38b		
**H8**	4.510	d 9.6(38b), t 5.3(7, 38a)	8		6, 9	7, 38a, 38b	7, 10, 40
**C9**	173.28				8, 10, 11, 38a, 38b		
**N10**	115.48			10			
**H10**	8.849	o.l., d ~5(10)		10	9, 12	11	8, 11, 13, 40, 44
**C11**	55.26		11		43a, 43b, 44		
**H11**	4.114	d 8.5(43b), t 5.0(10, 43a)	11		9, 12, 43, 44	10, 43a, 43b	10, 13
**C12**	171.89				10, 11, 13, 14, 43a, 43b		
**N13**	113.55			13			
**H13**	8.025	o.l., d 8.5(14)		13	12	14	10, 11, 16
**C14**	50.42		14		47a, 47b		
**H14**	4.832	t 8.5(13, 47a), d 5.3(47b)	14		12, 15, 47, 48	13, 47a, 47b	
**C15**	171.47				14, 16, 17, 47a, 47b		
**N16**	116.42			16			
**H16**	7.766	d 8.0(17)		16	15, 17	17	13, 20
**C17**	52.26		17		16, 50a, 50b		
**H17**	4.845	d 8.0(16), d 7.6(50b), d 5.6(50a)	17		15, 18, 50	16, 50a, 50b	20
**C18**	169.94				17, 23, 50a, 50b		
**N19**	n.f.						
**C20**	47.63		20		22a, 22b, 23		
**H20**	3.745	t 6.8(21)	20		21, 22	21a, 21b	16, 17, 22a, 25
**C21**	25.07		21a, 21b		20, 22a, 22b, 23		
**H21a**	2.007	m (o.l.)	21		22	20, 21b	
**H21b**	1.917	m (o.l.)	21		22, 23	20, 21a	
**C22**	29.38		22a, 22b		20, 21a, 21b, 23		
**H22a**	2.131	m (o.l.)	22		20, 21, 23, 24	22b, 23	20
**H22b**	1.960	m (o.l.)	22		20, 21, 23, 24	22a, 23	
**C23**	61.33		23		21b, 22a, 22b		

**H23**	4.392	d 8.5(22a/b), d 4.3(22a/b)	23		18, 20, 21, 22, 24	22a, 22b	25
**C24**	172.63				22a, 22b, 23, 25, 26		
**N25**	115.75			25			
**H25**	8.243	d 8.0(26)		25	24	26	20, 23, 53b, 54
**C26**	53.29		26		53b		
**H26**	4.333	d 10.0(53b), d 8.0(25), d 4.5(53a)	26		24, 27, 53, 54	25, 53a, 53b	28, 53a, 54
**C27**	172.29				26, 28, 29a, 29b, 53b		
**N28**	104.97			28			
**H28**	8.023	o.l., t 6.0(29a,b)		28	27, 29	29a, 29b	26
**C29**	42.54		29a, 29b		28, 31b		
**H29a**	3.843	d 16.9(29b), d 6.1(28)	29		27, 30	28, 29b	31a
**H29b**	3.764	d 16.9(29a), d 5.8(28)	29		27, 30	28, 29a	31a
**C30**	171.65				29a, 29b		
**N31**	101.26			31a, 31b			
**H31a**	7.359	(br) s		31		31b	29a, 29b
**H31b**	7.109	(br) s		31	29	31a	
**C32**	33.23		32a, 32b		2		
**H32a**	3.317	d 14.6(32b), d 5.1(2)	32		2, 3, 52	2, 32b	
**H32b**	3.171	d 14.6(32a), d 6.5(2)	32		2, 3, 52	2, 32a	
**C33**	36.59		33a, 33b		35		
**H33a**	3.110	d 13.9(33b), d 5.3(5)	33			5, 33b	35
**H33b**	2.815	d 13.9(33a), d 10.0(5)	33		5, 6	5, 33a	35
**C34**	128.25				36		
**C35**	130.37		35				
**H35**	7.057	m	35		33, 37	36	4, 5, 7, 33a, 33b
**C36**	115.34		36				
**H36**	6.714	m	36		34	35	OH37
**C37**	156.77				35		
**OH37**	9.555	br s					36
**C38**	37.31		38a, 38b		40		
**H38a**	3.279	d 14.1(38b), d 5.3(8)	38		8, 9, 39, 40	8, 38b	40

**H38b**	3.120	d 14.1(38a), d 9.6(8)	38		8, 9, 39, 40	8, 38a	7, 40
**C39**	137.98				38a, 38b, 41		
**C40**	129.64		40		38a, 38b, 42		
**H40**	7.338	m	40		38, 42	41	8, 10, 38a, 38b
**C41**	128.56		41				
**H41**	7.318	m	41		39	40, 42	
**C42**	126.75		42		40		
**H42**	7.244	m	42		40	41	
**C43**	26.92		43a, 43b		11, 44		
**H43a**	2.091	d 14.1(43b), t 7.4(44), d 5.0(11)	43		11, 12, 45	11, 44	
**H43b**	1.993	m (o.l.)	43		11, 12, 44, 45	11, 44	
**C44**	31.77		44		11, 43b, 46b		
**H44**	2.368	m	44		11, 43, 45	43a, 43b	10, 46a
**C45**	175.20				43a, 43b, 44		
**N46**	106.63			46a, 46b			
**H46a**	7.519	(br) d ~2(46b)		46		46b	44
**H46b**	6.916	(br) s		46	44	46a	
**C47**	37.03		47a, 47b		14, 49b		
**H47a**	2.875	d 15.8(47b), d 8.0(14)	47		14, 15, 48	14, 47b	49a
**H47b**	2.804	d 15.8(47a), d 5.1(14)	47		14, 15, 48	14, 47a	49a
**C48**	172.63				14, 47a, 47b, 49a, 49b		
**N49**	107.56			49a, 49b			
**H49a**	7.723	(br) d ~2(49b)		49	48	49b	47a, 47b
**H49b**	7.140	(br) d ~2(49a)		49	47, 48	49a	
**C50**	33.60		50a, 50b		17		
**H50a**	3.058	d 14.4(50b), d 5.6(17)	50		17, 18, 51	17, 50b	

**H50b**	2.942	d 14.4(50b), d 7.6(17)	50		17, 18, 51	17, 50a		
**C51**	32.81		51		50a, 50b, 52			
**H51**	2.853	m (o.l.)	51		52			
**C52**	32.67		52		32a, 32b, 51			
**H52**	2.798	m (o.l.)	52		51			
**C53**	30.76		53a, 53b		26, 55			
**H53a**	1.925	m (o.l.)	53		54	26, 53b, 54	26	
**H53b**	1.700	m (o.l.)	53		26, 27, 54, 55	26, 53a, 54	25	
**C54**	22.81		54		26, 53a, 53b, 56			
**H54**	1.512	m	54		55	53a, 53b, 55	25, 26, 56	
**C55**	26.89		55		53b, 54, 56			
**H55**	1.709	m (o.l.)	55		53, 56	54, 56		
**C56**	39.86		56		55			
**H56**	3.037	t 7.6(55)	56		54, 55	55	54	
**N57**	n.f.							
**H57**	7.88	br! s (o.l.)						
**C58/61**	159.60	br m						
**C59/62**	118.19	br q ~300(60)						
**F60**	-74.9							

^{1}H NMR of ethylene-bridged Lypressin **(25):**
600 MHz, DMF-d_7, 298 K

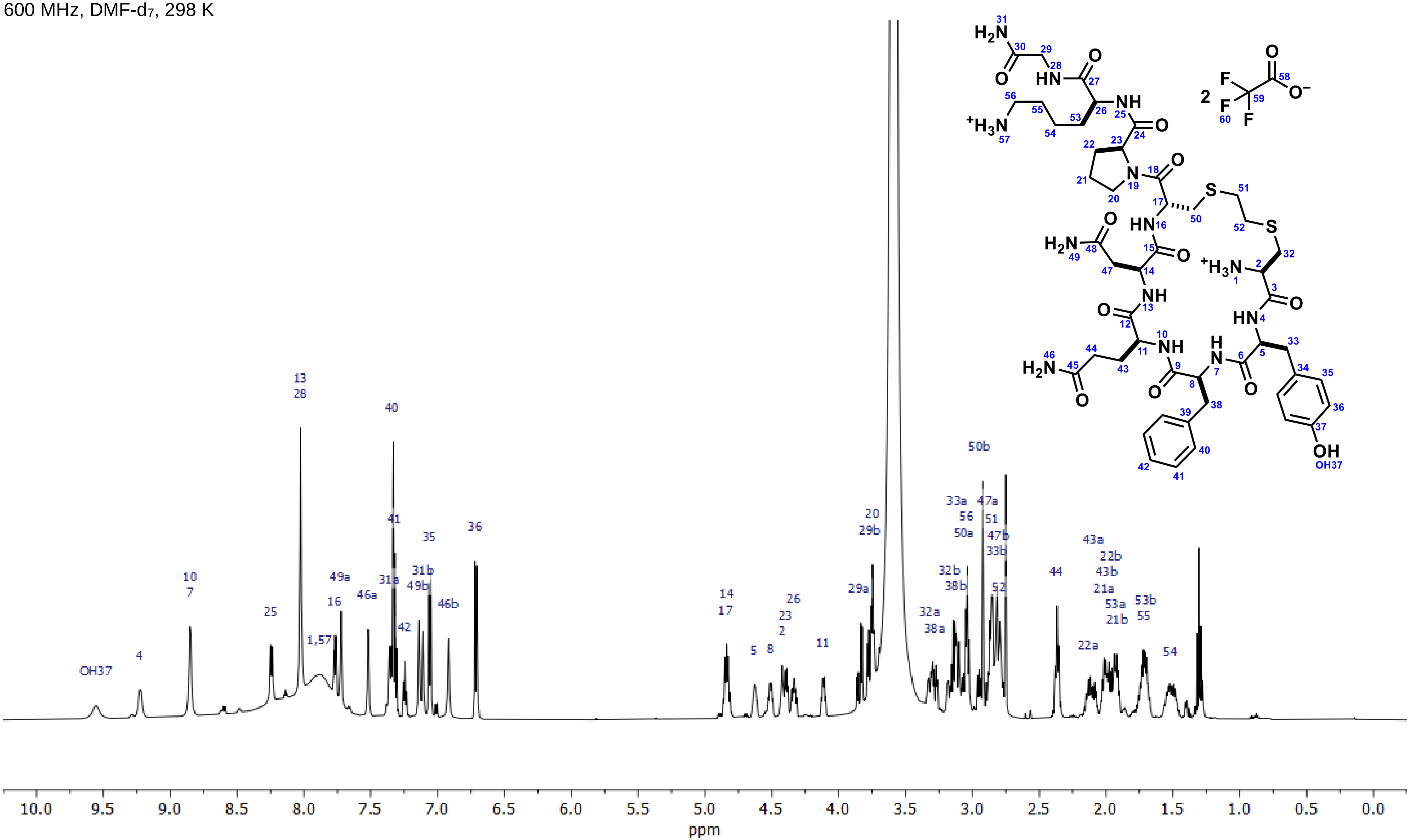

^{13}C NMR of ethylene-bridged Lypressin **(25):**
151 MHz, DMF-d$_7$, 298 K

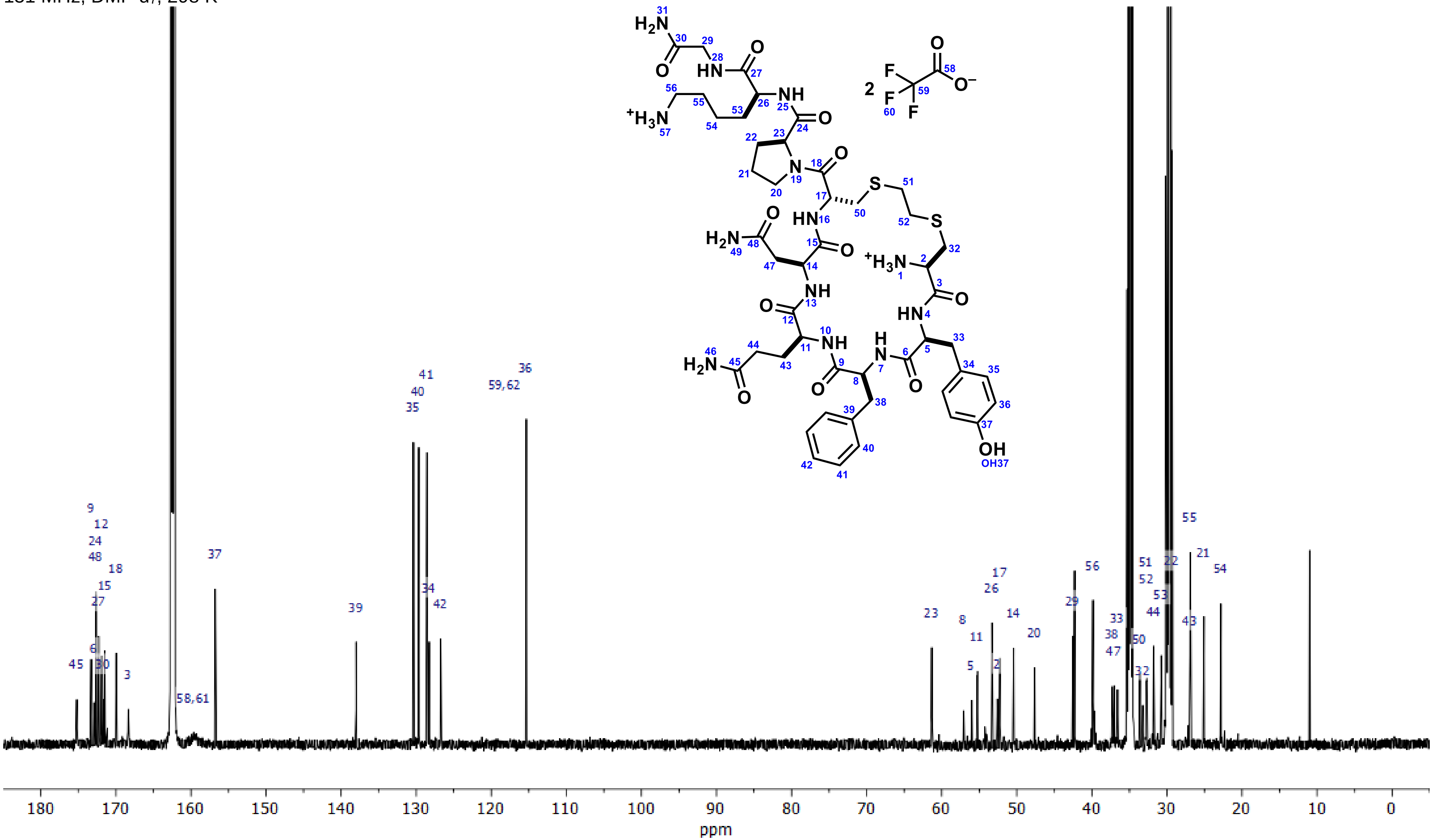

^{19}F NMR of ethylene-bridged Lypressin **(25):**
471 MHz, DMF-d$_7$, 298 K

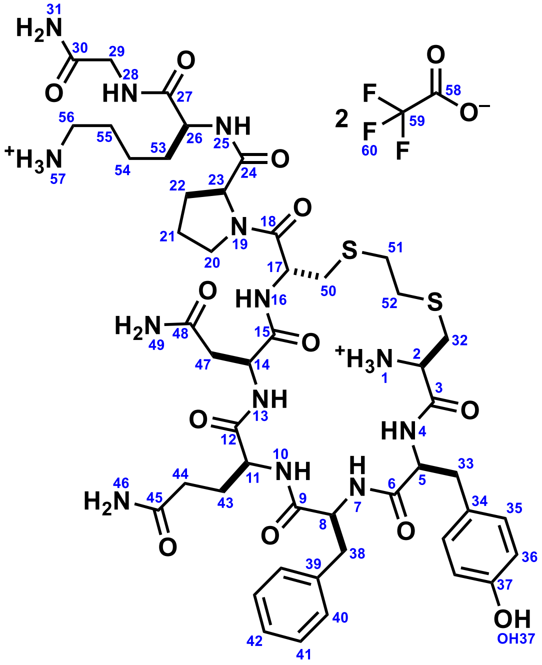

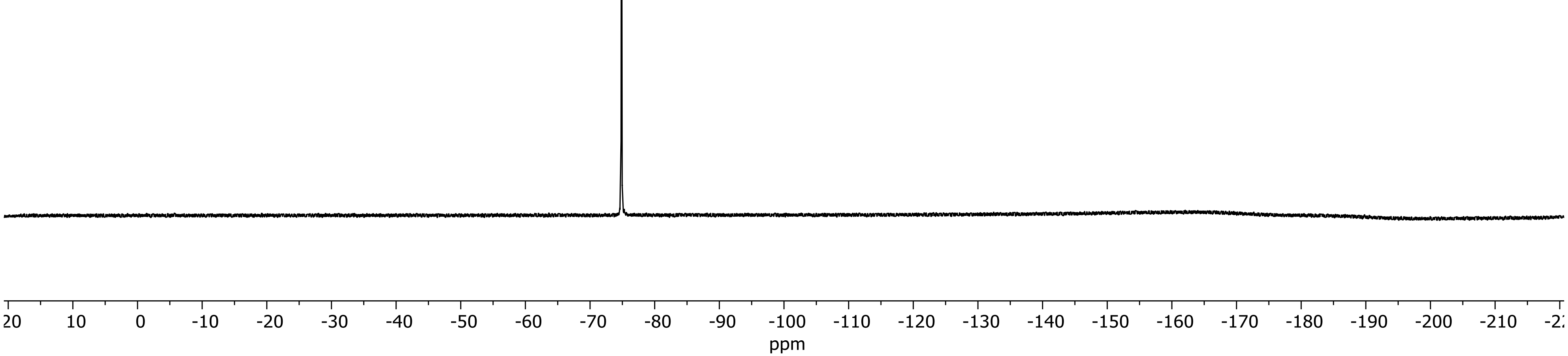

HSQC-NMR of ethylene-bridged Lypressin **(25):**
DMF-d$_7$, 298 K

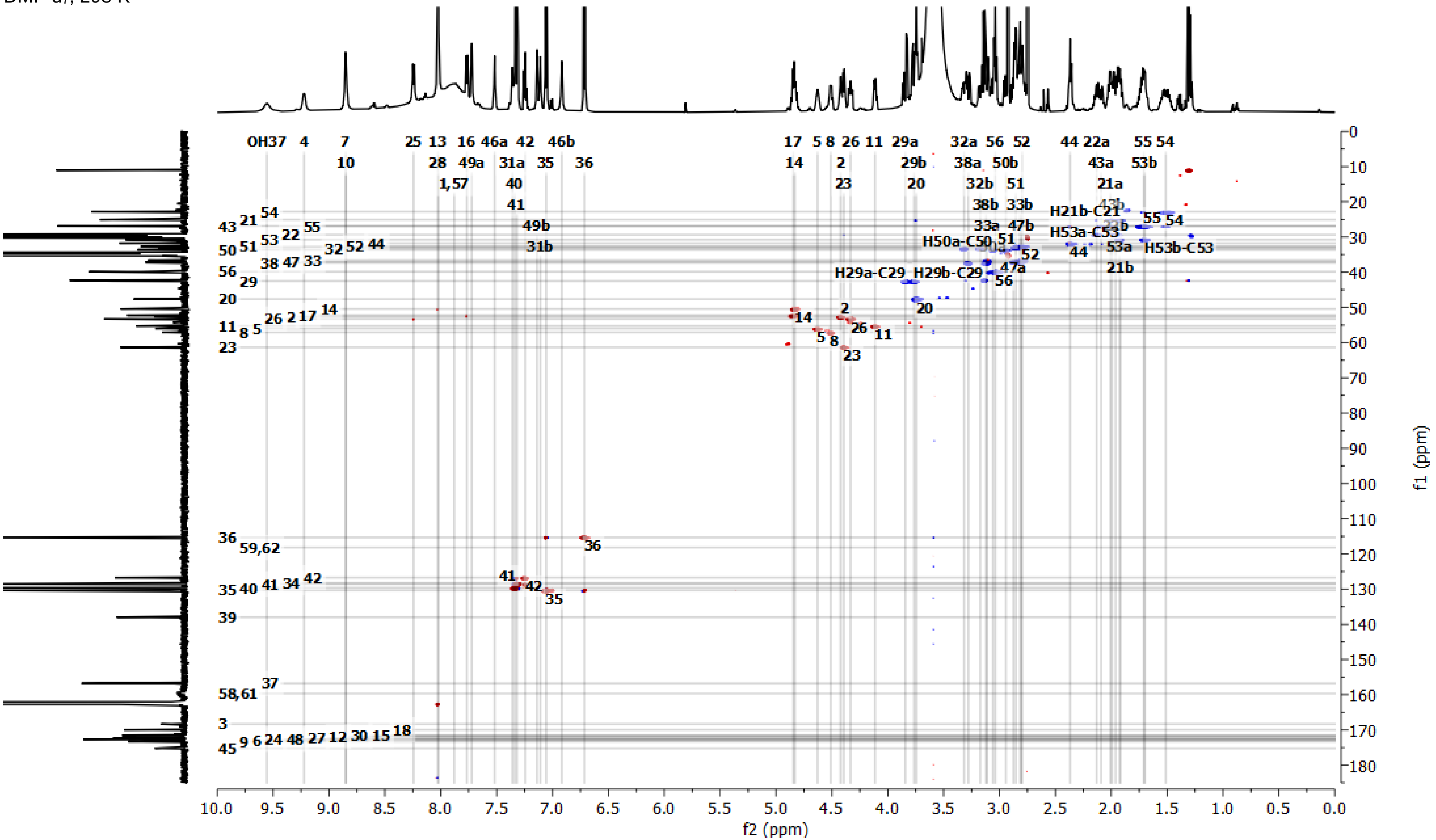

^{15}N-HSQC-NMR of ethylene-bridged Lypressin **(25):**
DMF-d$_7$, 298 K

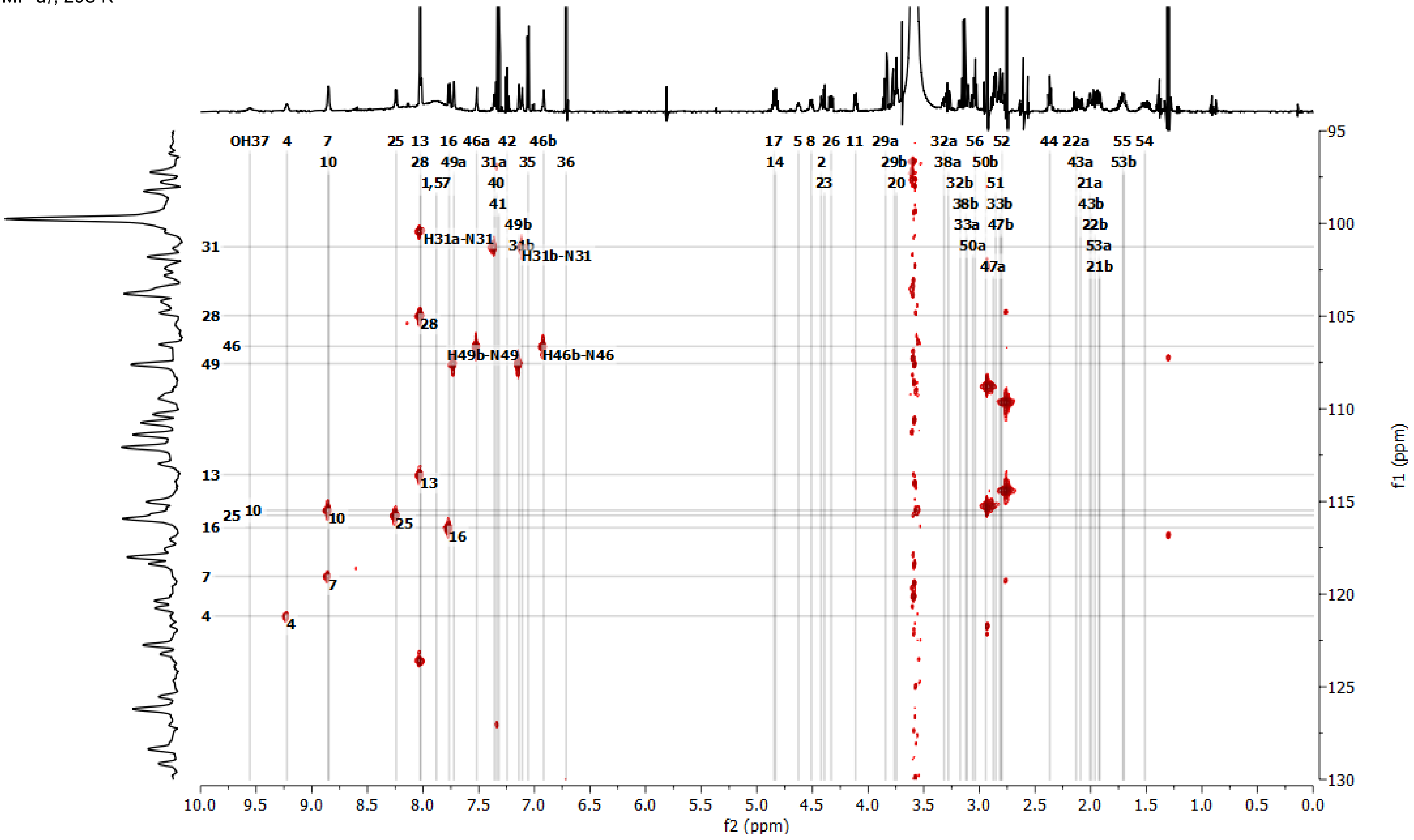

HMQC-NMR of ethylene-bridged Lypressin **(25):**
DMF-d$_7$, 298 K

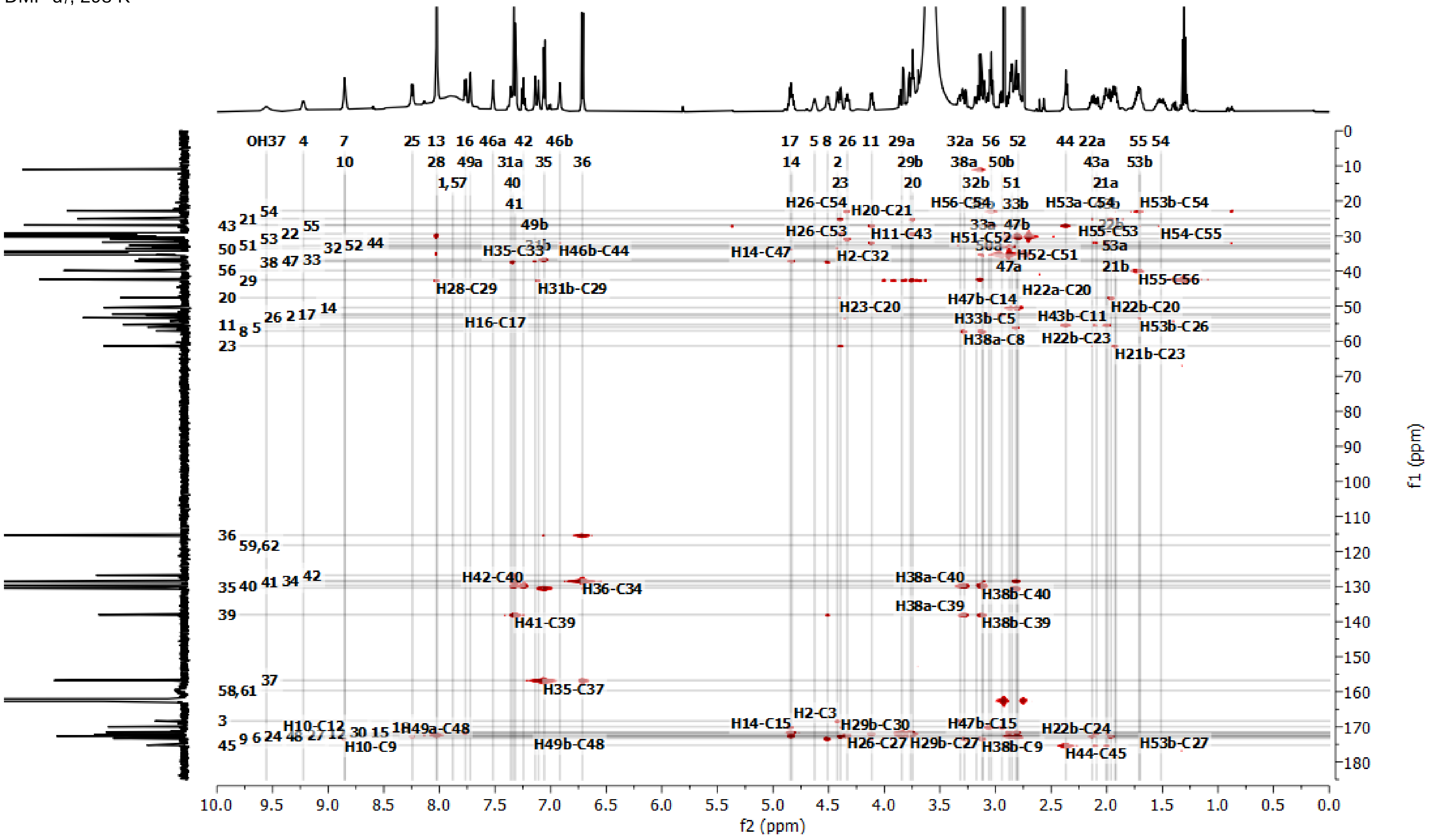

COSY-NMR of ethylene-bridged Lypressin **(25):**
DMF-d_7, 298 K

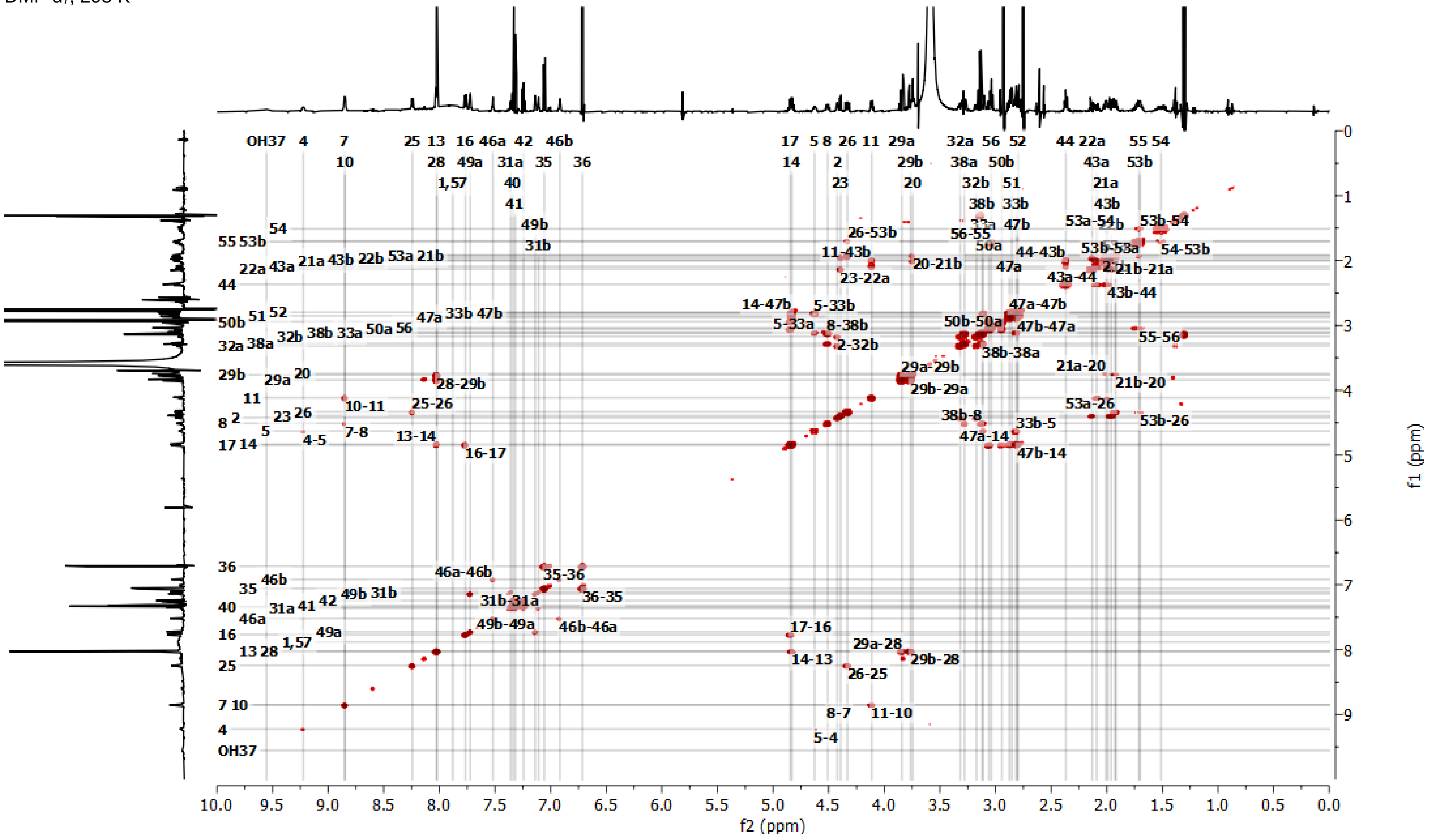

ROESY-NMR of ethylene-bridged Lypressin **(25):**
DMF-d7, 298 K

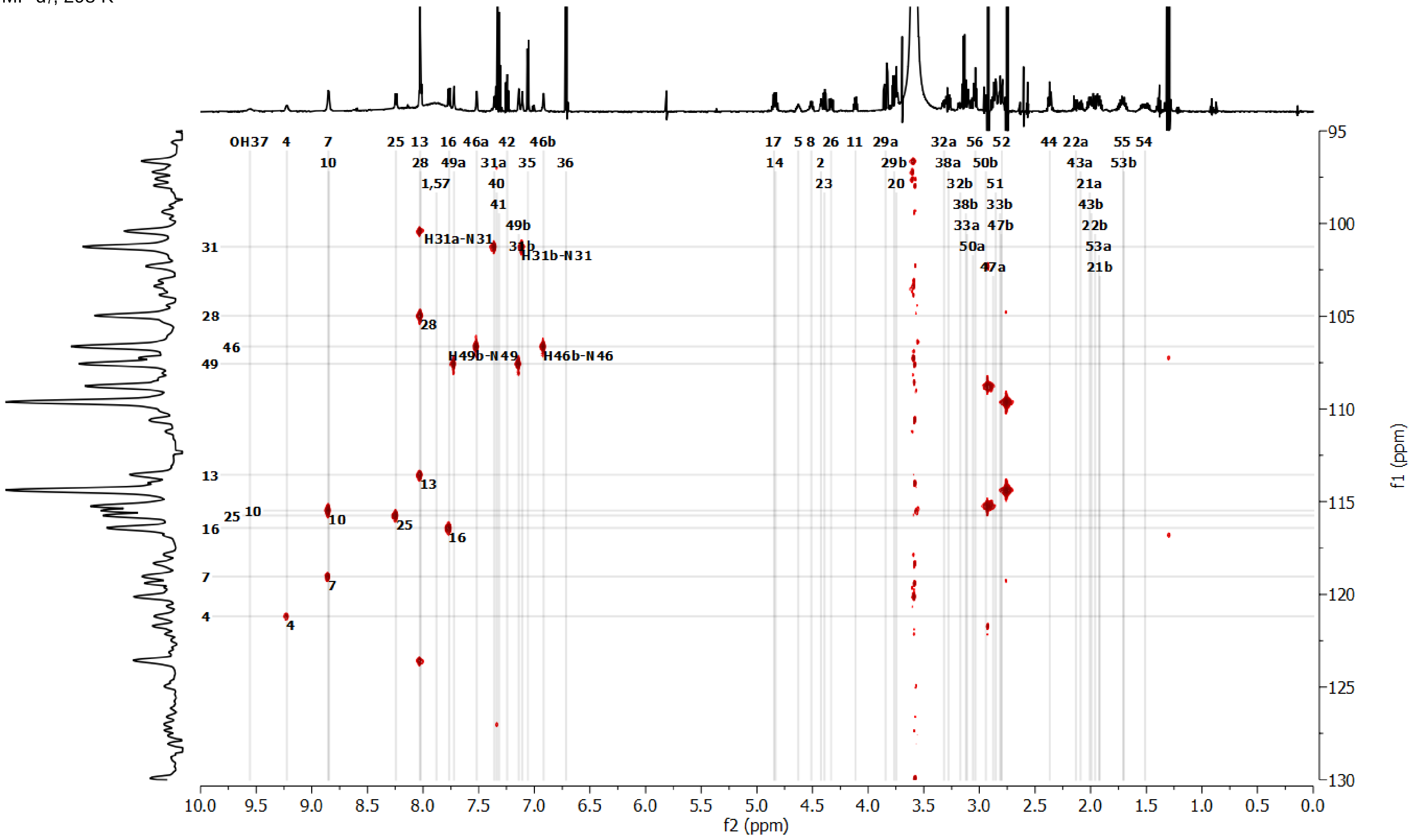

Ethylene-bridged Octreotide (26)

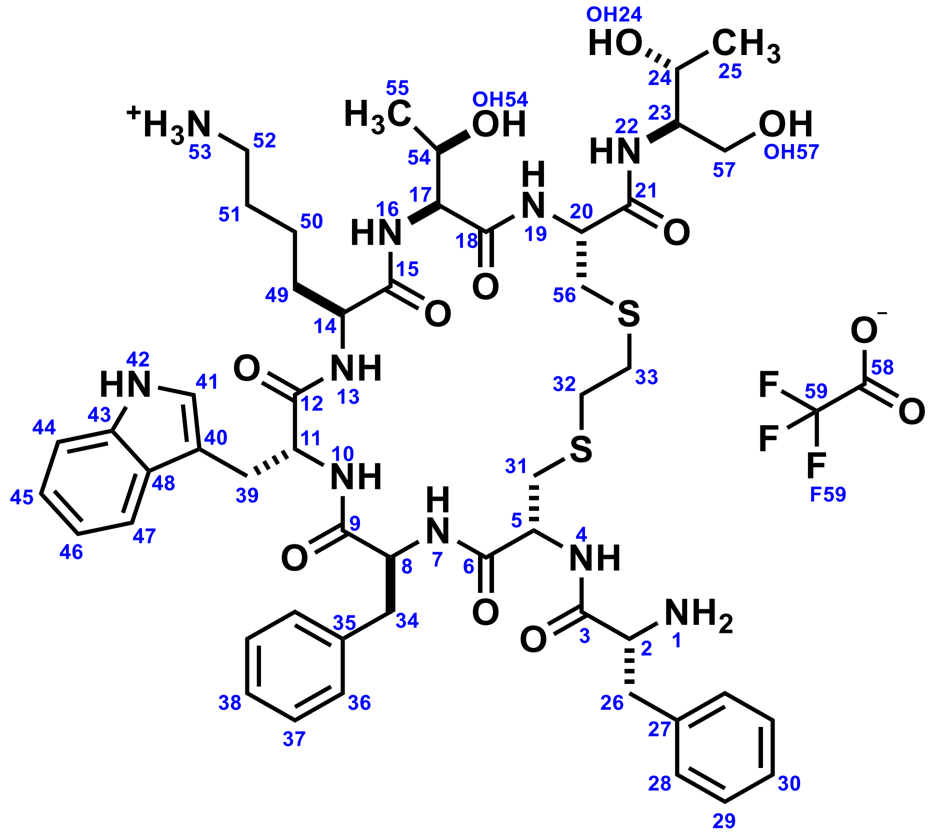

Table S35. Characterization table of ethylene-bridged Octreotide. The ^{15}N chemical shifts were referenced to NH_3(l).

Atom	δ (ppm)	J	HSQC	^{15}N-HSQC	HMQC	COSY	NOESY	TOCSY
N1	n.f.							
H1	8.300	br! s					4	
C2	55.26		2		26a			
H2	4.514	br s	2			26a, 26b	4, 28	26a, 26b

**C3**	170.16							
**N4**	122.81			4				
**H4**	9.360	(br) d 9.2(H5)		4		H5	1, 2, 7	H5, 31a, 31b
**C5**	51.43		H5		31b			
**H5**	5.192	d 9.2(4), t 6.5(31a,b)	5		6	4, 31a, 31b	7, 31a, 31b, 32a, 32b, 33a, 33b	4, 31a, 31b
**C6**	169.79				H5, 7, 8, 31a, 31b			
**N7**	121.24			7				
**H7**	8.564	d 7.6(8)		7	6, 8, 9	8	H5, 4, 8, 10, 16, 34, 55	8, 34
**C8**	55.12		8		7, 34			
**H8**	4.751	d 7.6(7), d 8.2(34a/b), d 6.4(34a/b)	8		6, 9, 34, 35	7, 34	7, 10, 34	7, 34
**C9**	171.86				7, 8, 10, 11, 34			
**N10**	129.69			10				
**H10**	9.062	d 5.0(11)		10	9, 11, 39	11	7, 8, 11, 13, 16, 36, 39a, 39b, 41, 47	11, 39a, 39b
**C11**	56.49		11		10, 39a, 39b			
**H11**	4.383	d 6.3(39b), d 5.0(10), d 9.2(39a)	11		9, 12, 39, 40	10, 39a, 39b	10, 13, 16, 39a, 39b, 41, 47	10, 39a, 39b
**C12**	172.30				H14, 11, 13, 39a, 39b			
**N13**	122.89			13				
**H13**	8.490	d 9.0(H14)		13	12, 14	H14	H14, 10, 11, 16, 41, 47, 49a, 49b, 50a, 50b, 55	H14, 49a, 49b, 50a, 50b, 51a, 51b, 52a, 52b
**C14**	52.98		H14		13			
**H14**	4.249	m (o.l.)	14		12, 15, 49, 50	13, 49b	13, 16, 49a, 49b, 50a, 50b	13, 49a, 49b, 50a, 50b, 51a, 51b, 52a, 52b
**C15**	171.68				H17, H14, 16			
**N16**	110.31			16				
**H16**	8.105	d 9.2(H17)		16	15, 17	H17	H17, H14, 7, 10, 11, 13, 19, 49a, 49b, 55	OH54, H17, 54, 55
**C17**	59.40		H17		16, 55			
**H17**	4.714	d 6.0(54), d 9.2(16)	17		15, 18, 54, 55	16, 54	16, 19, 54, 55	OH54, 16, 54, 55
**C18**	171.81				H17, 19, 20			
**N19**	123.03			19				

**H19**	8.856	d 8.9(20)		19	18, 20	20	H17, 16, 20, 22, 25, 54, 55, 56a, 56b	20, 56a, 56b
**C20**	51.82		20		19, 56b			
**H20**	5.180	d 8.9(19), t 7.5(56a,b)	20		18, 21, 56	19, 56a, 56b	19, 22, 32a, 32b, 33a, 33b, 56a, 56b	19, 56a, 56b
**C21**	170.55				20, 22, 56a, 56b			
**N22**	114.17			22				
**H22**	7.925	d 9.2(23)		22	21, 23	23	19, 20, 23, 25, 33a, 33b, 55, 56a, 56b, 57a, 57b	23, 24, 25, 57a, 57b
**C23**	56.23		23		22, 25, 57a, 57b			
**H23**	3.880	d 9.2(22), d 2.3(24), d 5.3(57b), d 7.8(57a)	23		57	22, 24, 57a, 57b	22, 24	22, 57a, 57b
**C24**	65.19		24		25, 57a, 57b			
**H24**	4.254	m (o.l.)	24		25	23, 25	23, 25	22, 25
**OH24**	5.165/6.167	br (o.l.)						
**C25**	19.93		25		24			
**H25**	1.146	d 6.4(24)	25		23, 24	24	19, 22, 24	22, 24
**C26**	38.59		26a, 26b		28			
**H26a**	3.508	m (o.l.)	26		2, 27, 28	2, 26b		2
**H26b**	3.155	m (o.l.)	26		27, 28	2, 26a		2
**C27**	135.87				26a, 26b, 29			
**C28**	130.12		28		26a, 26b, 30			
**H28**	7.504	m (o.l.)	28		26, 30	29	2, 29	
**C29**	128.91		29					
**H29**	7.373	m	29		27	28, 30	28, 30	
**C30**	127.51		30		28			
**H30**	7.326	m	30		28	29	29	
**C31**	34.10		31a, 31b		32a, 32b			
**H31a**	2.959	m (o.l.)	31		6, 32	H5, 31b	H5, 31b	H5, 4
**H31b**	2.608	m (o.l.)	31		5, 6, 32	H5, 31a	H5, 31a	H5, 4
**C32**	30.34		32a, 32b		31a, 31b			

**H32a**	2.775	m (o.l.)	32		31	32b, 33a, 33b	H5, 20, 32b, 33a, 33b		
**H32b**	2.677	m (o.l.)	32		31	32a, 33a, 33b	H5, 20, 32a, 33a, 33b		
**C33**	30.06		33a, 33b						
**H33a**	2.854	m (o.l.)	33		56	32a, 32b, 33b	H5, 20, 22, 32a, 32b, 33b		
**H33b**	2.638	m (o.l.)	33		56	32a, 32b, 33a	H5, 20, 22, 32a, 32b, 33a		
**C34**	38.84		34		8, 36				
**H34**	2.926	m (o.l.)	34		8, 9, 35, 36	8	7, 8, 36	7, 8	
**C35**	137.64				8, 34, 37				
**C36**	129.38		36		34, 38				
**H36**	7.186	m	36		34, 38	37	10, 34, 37		
**C37**	128.64		37						
**H37**	7.296	m	37		35	36, 38	36		
**C38**	126.91		38		36				
**H38**	7.254	m	38		36	37			
**C39**	26.41		39a, 39b		10, 11				
**H39a**	3.171	d 14.3(39b), d 9.2(11)	39		11, 12, 40, 41, 48	11, 39b	10, 11, 39b, 41	10, 11	
**H39b**	2.900	m (o.l.)	39		11, 12, 40, 41, 48	11, 39a	10, 11, 39a, 41	10, 11	
**C40**	109.74				11, 39a, 39b, 41, 47				
**C41**	124.06		41		39a, 39b				
**H41**	7.080	(br) s	41		40, 43, 48	42	10, 11, 13, 39a, 39b, 42	42	
**N42**	130.01			42					
**H42**	10.993	(br) s		42		41	41, 44	41	
**C43**	137.04				41, 45, 47				
**C44**	111.78		44		46				
**H44**	7.442	d 8.1(45)	44		46, 48	45	42, 45, 46		
**C45**	121.36		45		47				
**H45**	7.115	d 1.1(47), d 8.1(44), d 7.0(46)	45		43, 47	44, 46	44, 46		

**C46**	118.73		46		44			
**H46**	7.027	d 7.9(47), d 0.9(44), d 7.0(45)	46		44, 48	45, 47	44, 45	
**C47**	118.49		47		45			
**H47**	7.494	(o.l.) d 7.9(46)	47		40, 43, 45	46	10, 11, 13	
**C48**	127.70				39a, 39b, 41, 44, 46			
**C49**	30.55		49a, 49b		H14			
**H49a**	1.903	d 13.8(49b), d 7.1(50a), d 10.0(50b), d 3.5(14)	49			49b, 50a, 50b	H14, 13, 16, 49b, 50a, 50b	H14, 13
**H49b**	1.366	d 10.5(50a), d 4.5(50b), d 10.5(H14), d 13.8(49a)	49			H14, 49a, 50a, 50b	H14, 13, 16, 49a, 50a, 50b	H14, 13
**C50**	22.68		50a, 50b		H14			
**H50a**	0.960	m	50			49a, 49b, 50b, 51a, 51b	H14, 13, 49a, 49b, 50b	H14, 13
**H50b**	0.889	m	50			49a, 49b, 50a, 51a, 51b	H14, 13, 49a, 49b, 50a	H14, 13
**C51**	27.16		51a, 51b					
**H51a**	1.589	m	51			50a, 50b, 51b, 52a, 52b	51b	H14, 13
**H51b**	1.521	m	51			50a, 50b, 51a, 52a, 52b	51a	H14, 13
**C52**	39.72		52a, 52b					
**H52a**	2.917	m (o.l.)	52			51a, 51b		H14, 13
**H52b**	2.888	m (o.l.)	52			51a, 51b		H14, 13
**N53**								
**H53**	8.300(?)	br!						
**C54**	68.26		54		H17, 55			

**H54**	4.231	m (o.l.)	54			OH54, H17, 55	H17, 19, 55	OH54, H17, 16
**OH54**	5.005	br s			55	54		H17, 16, 54, 55
**C55**	19.74		55		OH54, H17			
**H55**	1.216	d 6.4(54)	55		17, 54	54	H17, 7, 13, 16, 19, 22, 54	OH54, H17, 16
**C56**	33.56		56a, 56b		20, 33a, 33b			
**H56a**	2.941	m (o.l.)	56		21	20, 56b	19, 20, 22, 56b	19, 20
**H56b**	2.735	m (o.l.)	56		20, 21	20, 56a	19, 20, 22, 56a	19, 20
**C57**	61.53		57a, 57b		23			
**H57a**	3.711	d 7.8(23), d 10.7(57b)	57		23, 24	23, 57b	22	22, 23
**H57b**	3.624	d 5.3(23), d 10.7(57a)	57		23, 24	23, 57a	22	22, 23
**OH57**	6.167/5.165	br!						
**C58**	159.49	br m						
**C59**	118.07	q 296(F59)						
**F59**	-74.8							

^{1}H NMR of ethylene-bridged Octreotide **(26):**
600 MHz, DMF-d7, 298 K

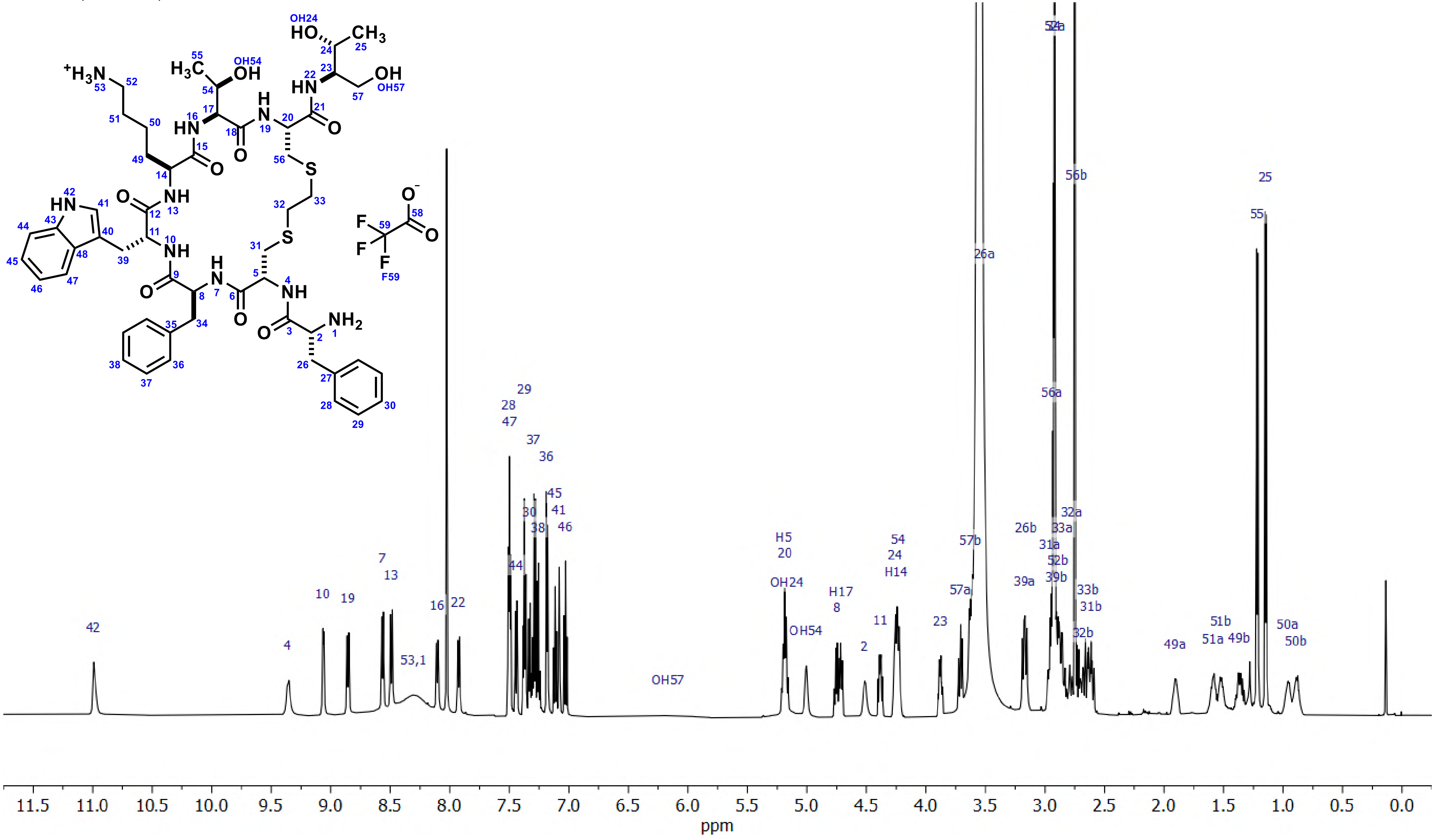

^{13}C NMR of ethylene-bridged Octreotide **(26):**
151 MHz, DMF-d_7, 298 K

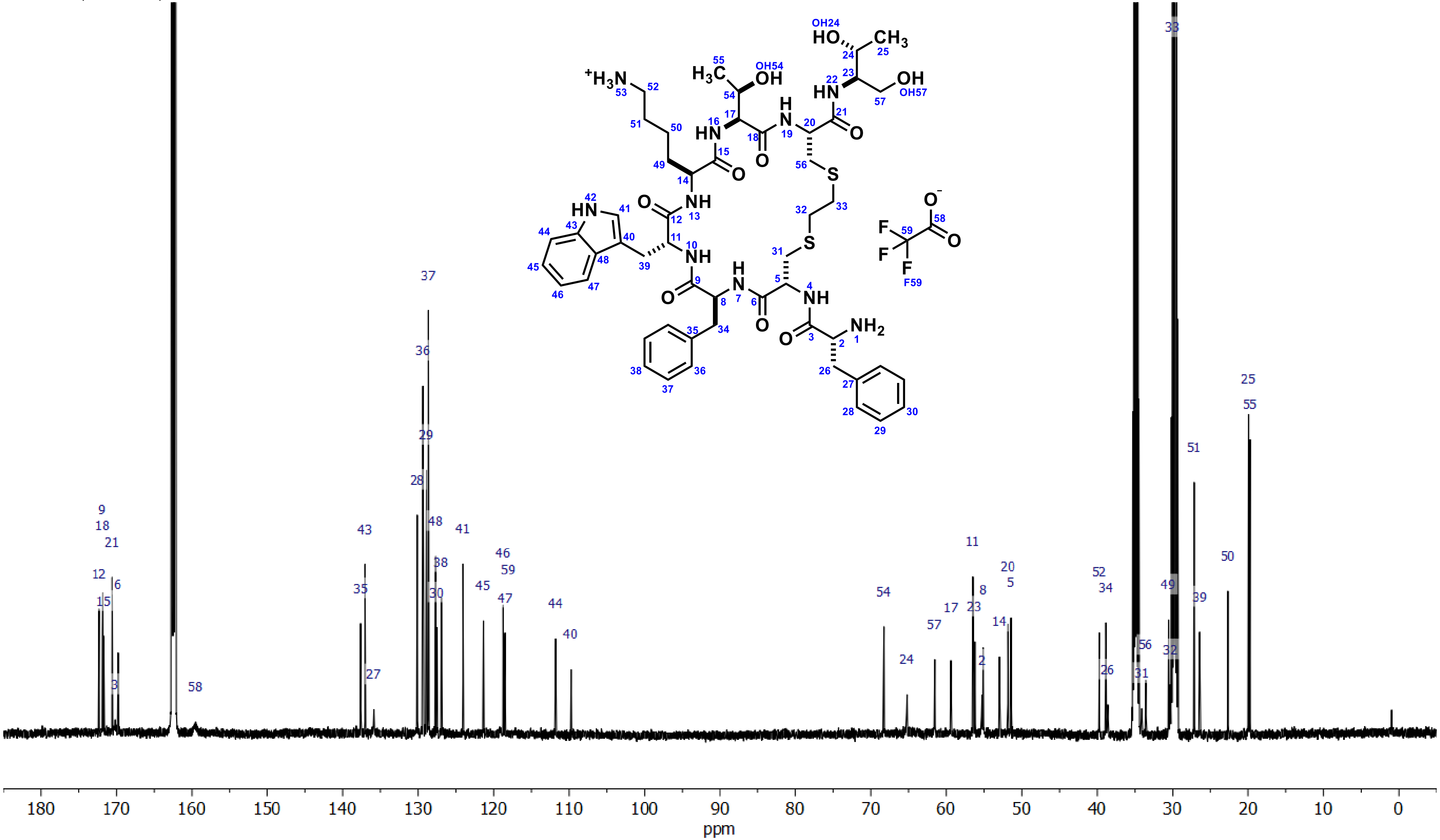

^{19}F NMR of ethylene-bridged Octreotide **(26):**
471 MHz, DMF-d_7, 298 K

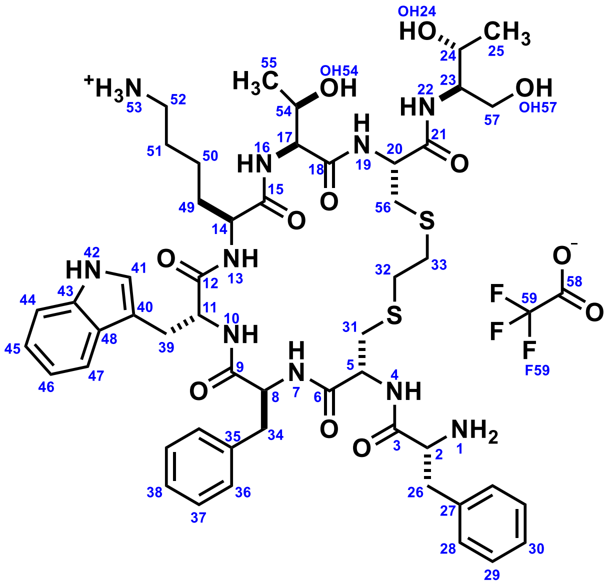

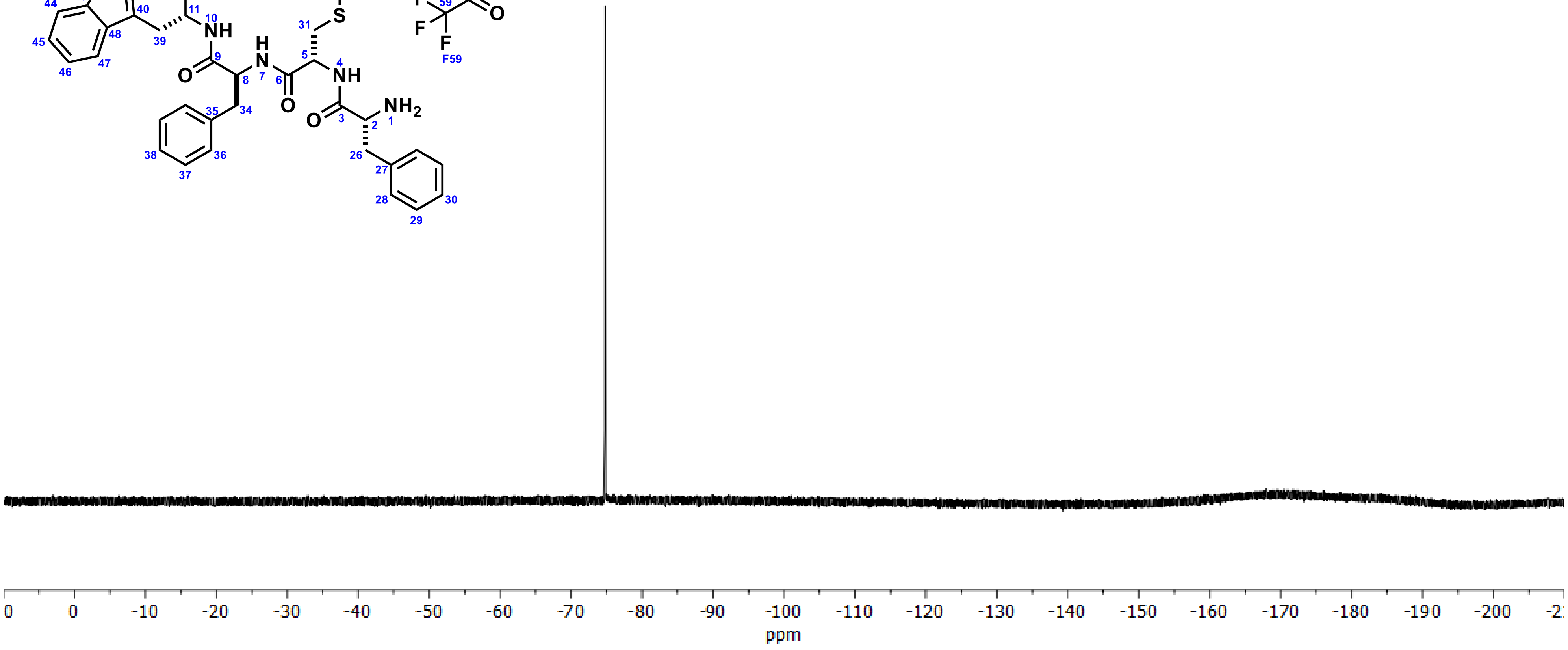

HSQC-NMR of ethylene-bridged Octreotide **(26):**
DMF-d_7, 298 K

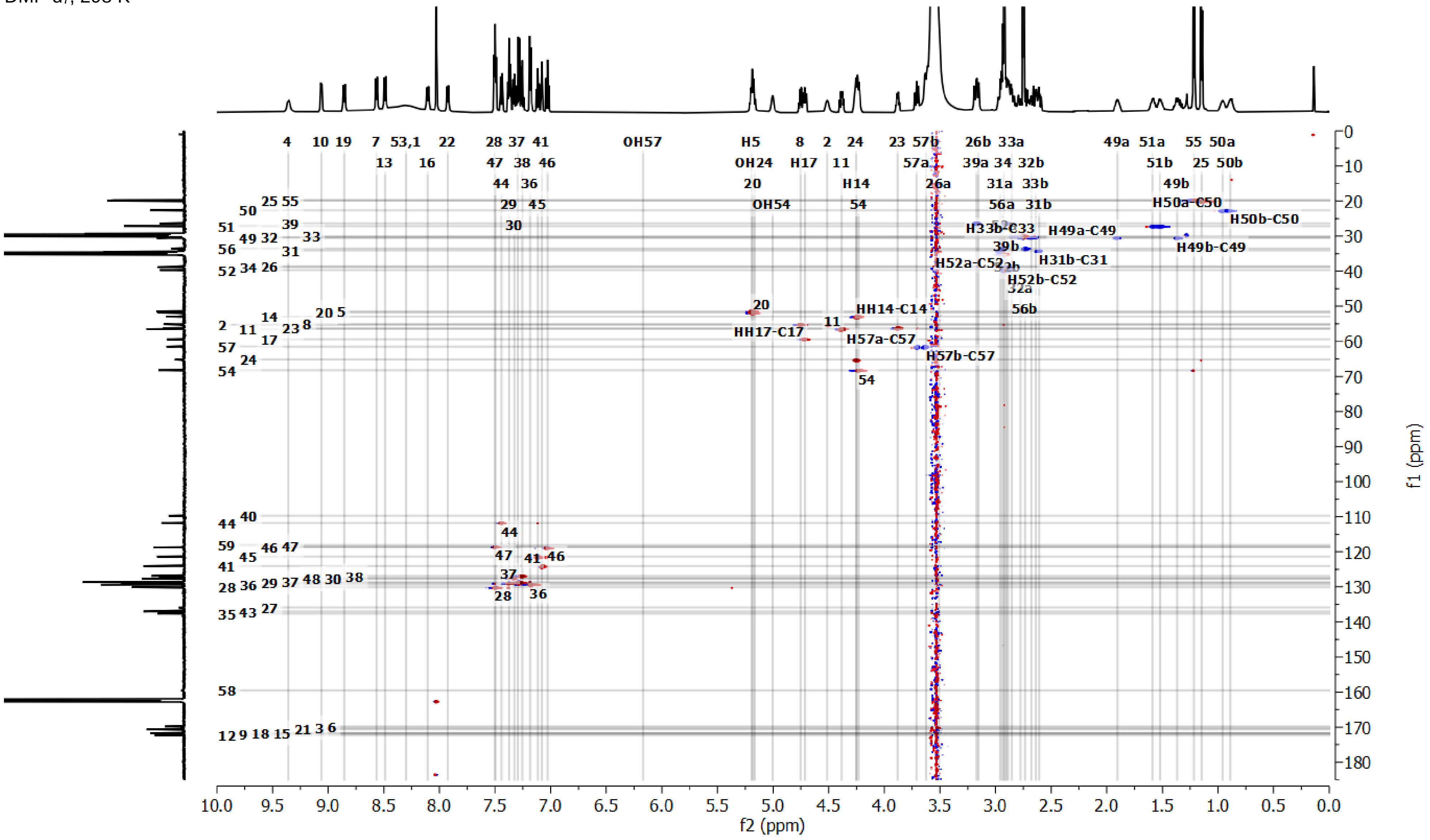

HMQC-NMR of ethylene-bridged Octreotide **(26):**
DMF-d$_7$, 298 K

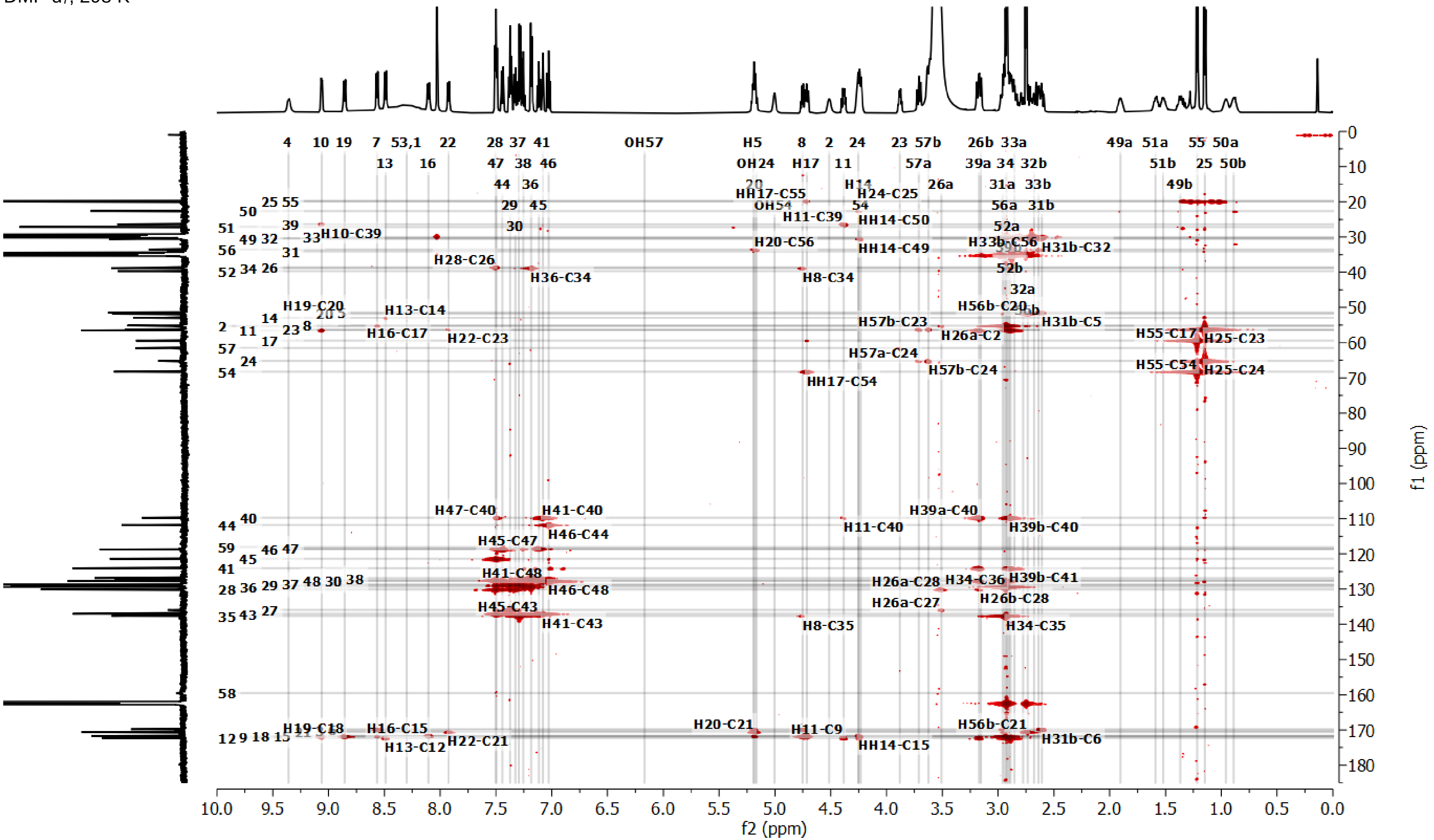

COSY-NMR of ethylene-bridged Octreotide **(26):**
DMF-d_7, 298 K

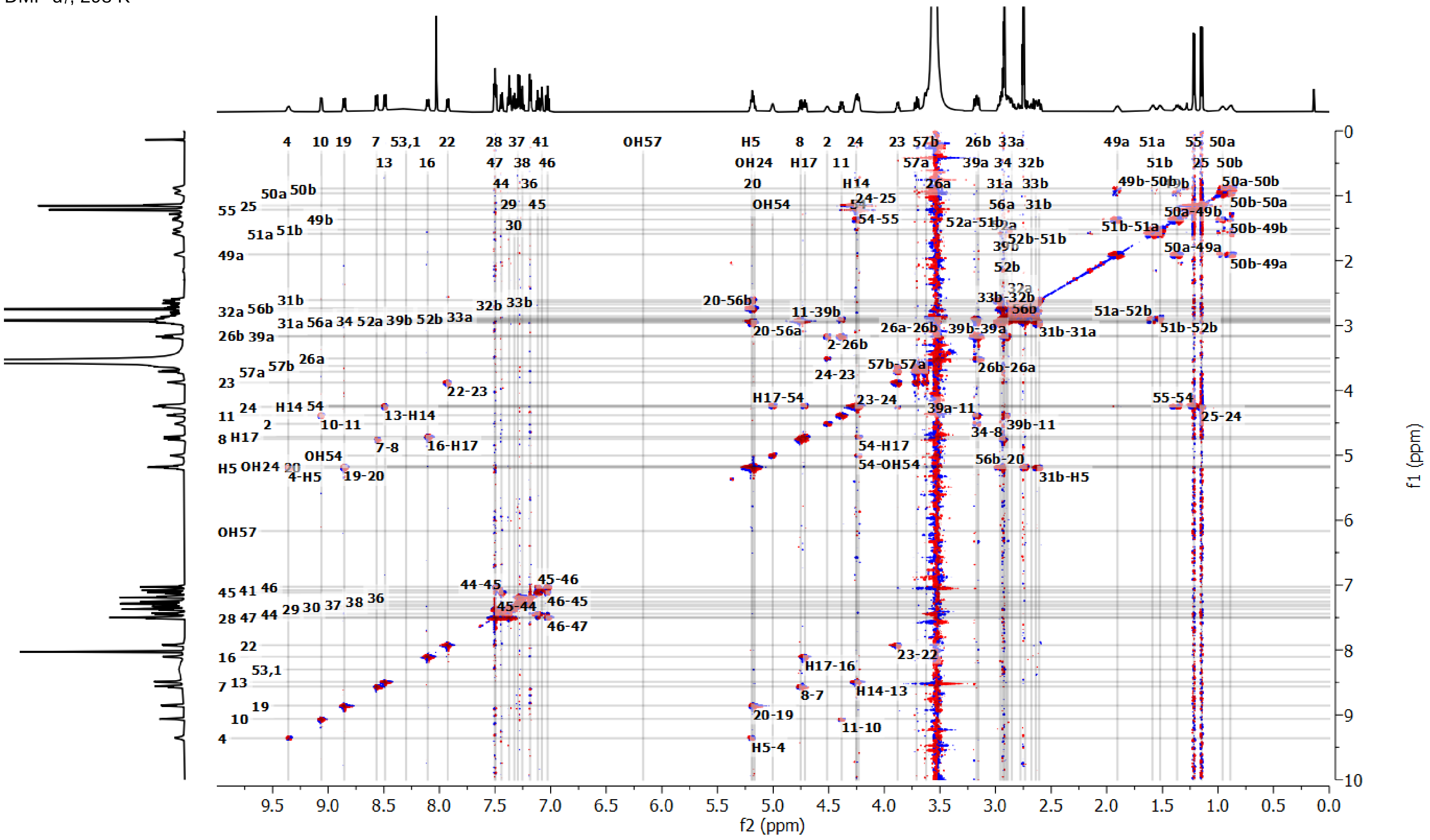

ROESY-NMR of ethylene-bridged Octreotide **(26):**
DMF-d$_7$, 298 K

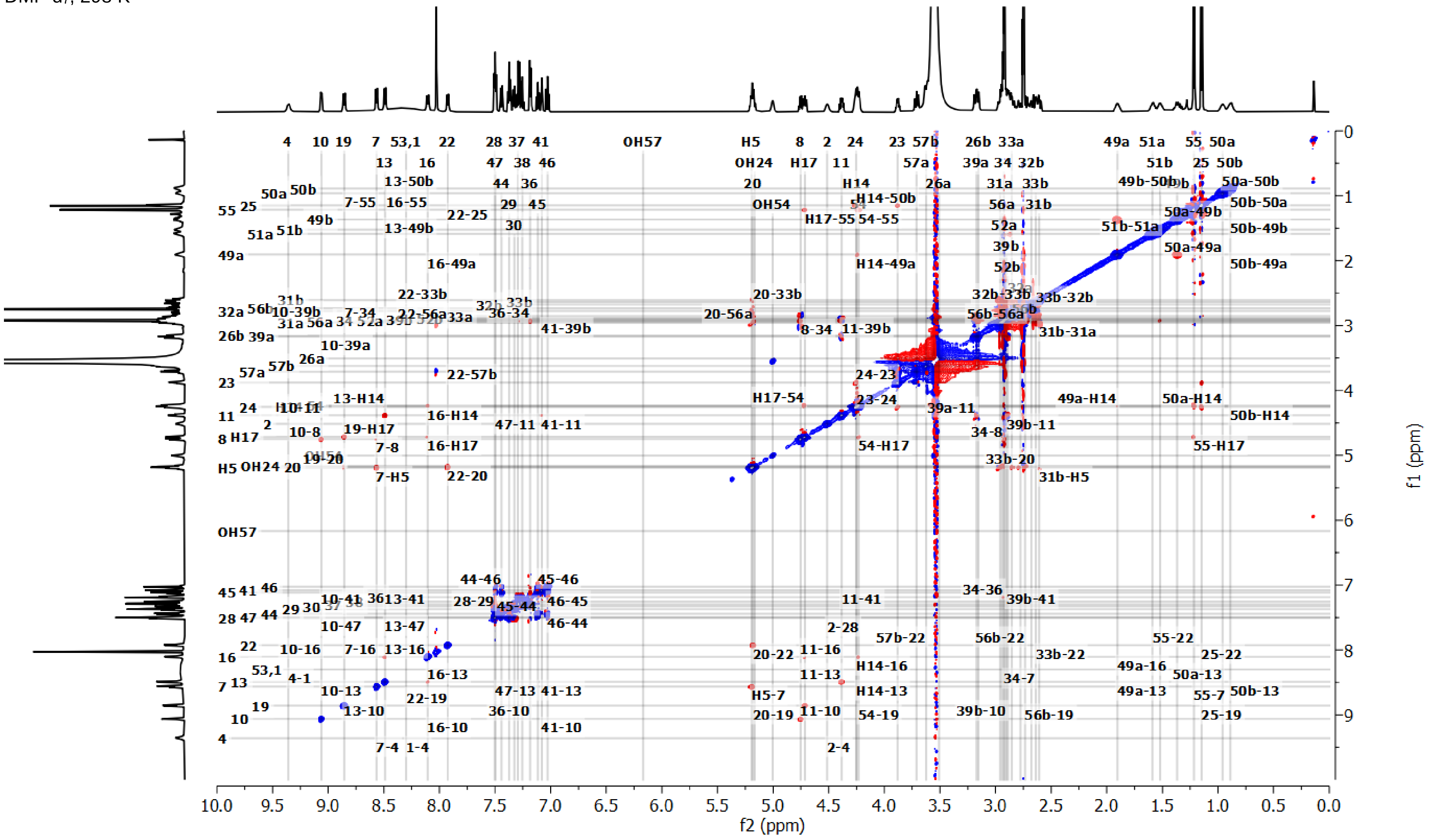

NOESY-NMR of ethylene-bridged Octreotide **(26):**
DMF-d$_7$, 298 K

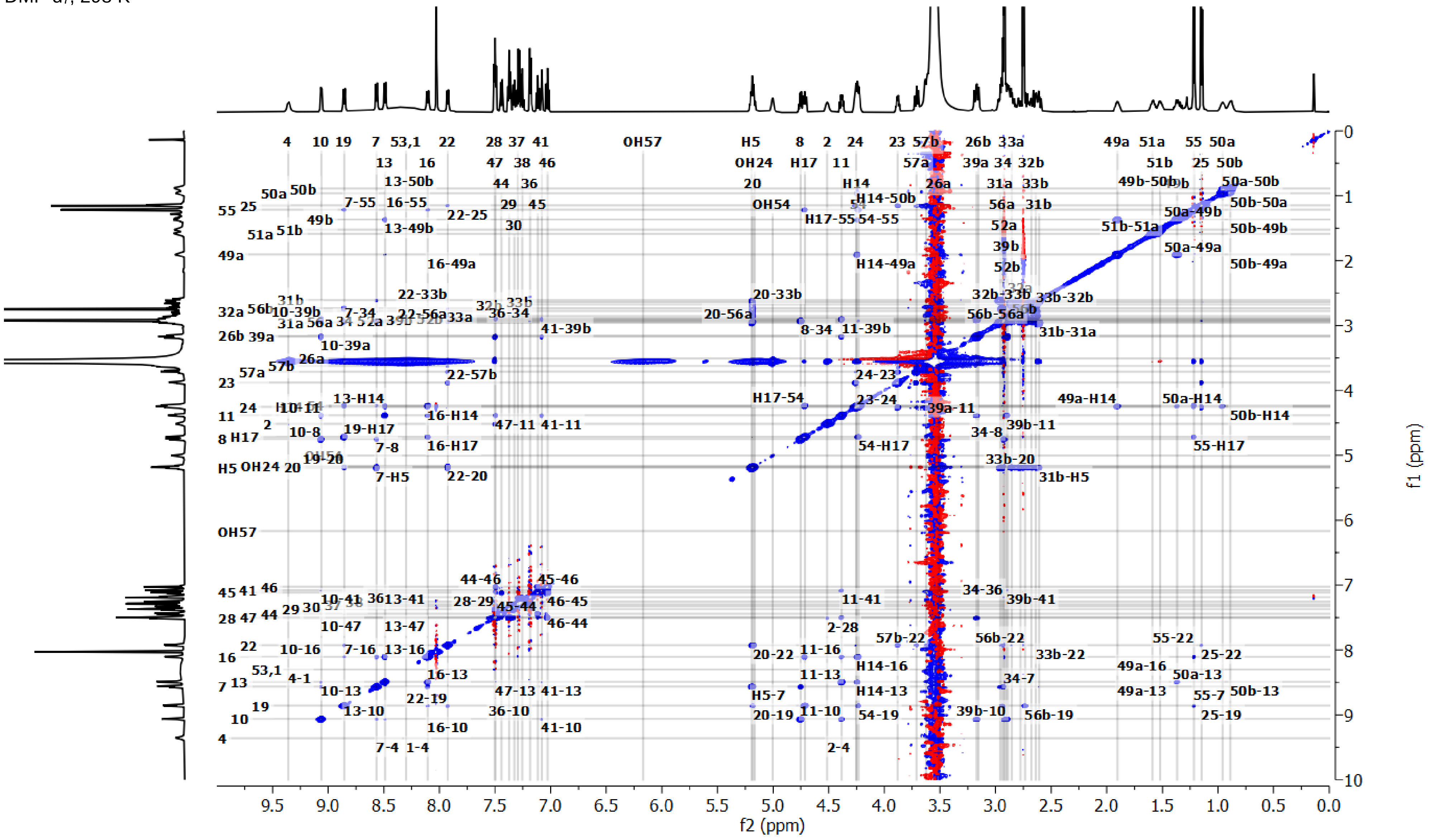

^{15}N-HSQC-NMR of ethylene-bridged Octreotide **(26):**
DMF-d$_7$, 298 K

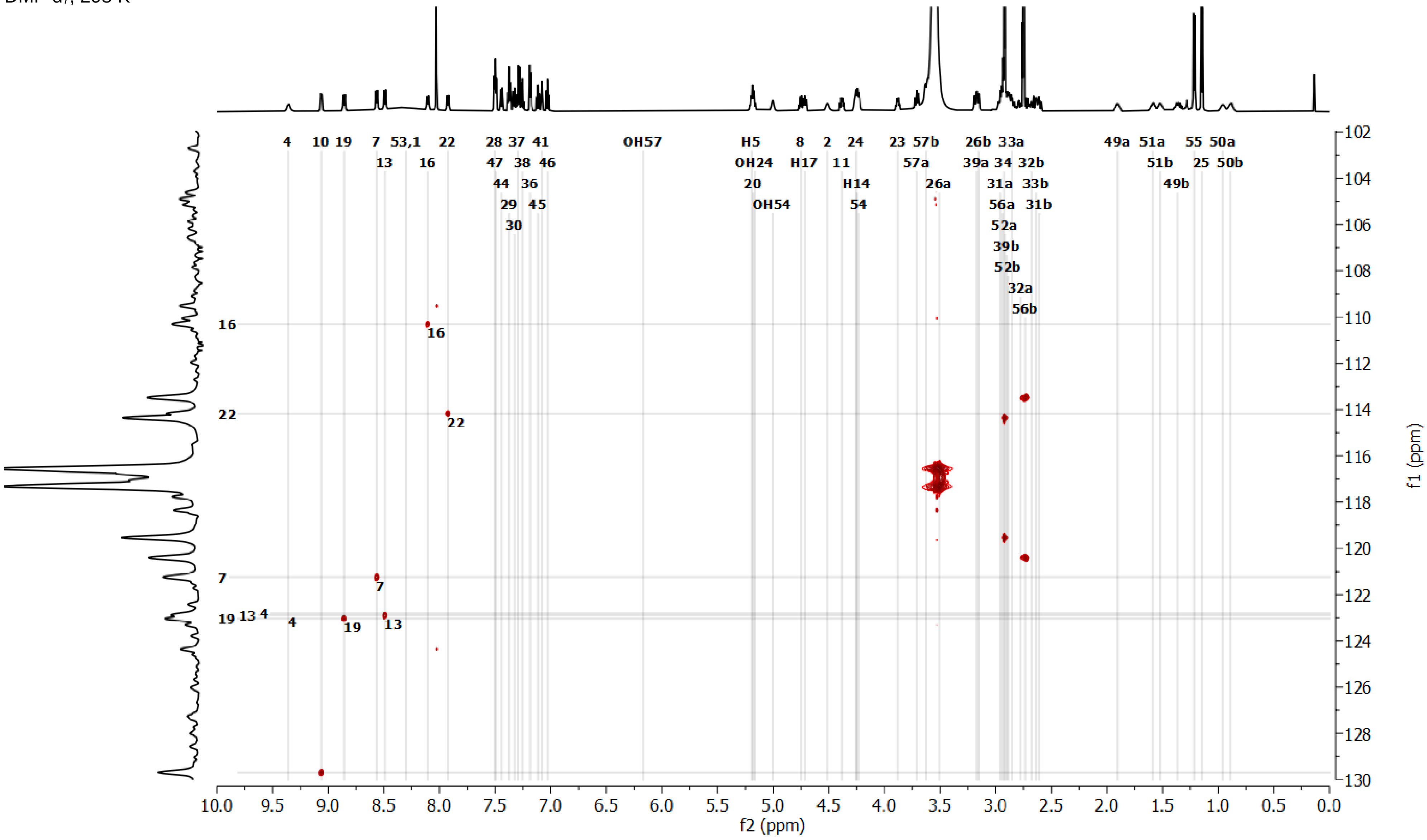

Ethylene-bridged Oxytocin (27)

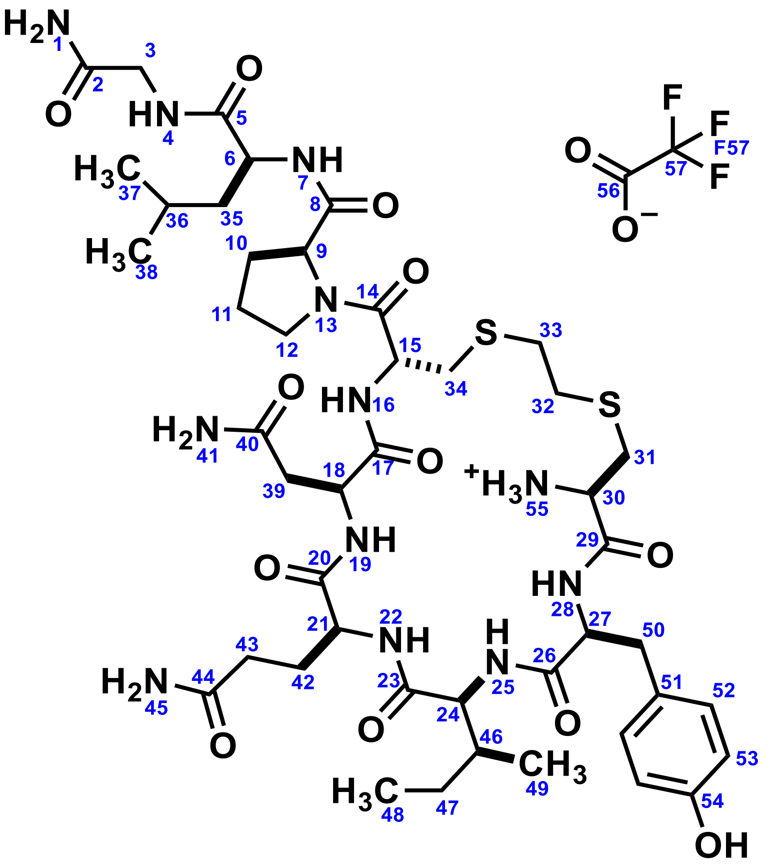

Table S36. Characterization table of ethylene-bridged Oxytocin. The ^{15}N chemical shifts were referenced to CH_3NO_2.

Atom	δ (ppm)	J	HSQC	HMQC	COSY	ROESY	^{15}N-HSQC	DIPSI
**N1**	-279.3						1a, 1b	
**H1a**	7.292	(br) d 1.5(1b)		2	1b	3a, 3b, 4, 6, 7, 9, 34	1	1b
**H1b**	7.091	(br) m		2, 3	1a	3a, 3b, 4, 34	1	1a
**C2**	171.78			1a, 1b, 3a, 3b				

**C3**	42.72		3a, 3b	1b, 4				
**H3a**	3.871	d 6.5(4), d 16.8(3b)	3	2, 5	3b, 4	1a, 1b		3b, 4
**H3b**	3.672	d 5.7(4), d 16.8(3a)	3	2, 5	3a, 4	1a, 1b		3a, 4
**N4**	-275.7						4	
**H4**	7.979	t 6.1(3a, b)		3, 5	3a, 3b	1a, 1b, 6, 7, 9, 34, 35, 36	4	3a, 3b
**C5**	172.83			3a, 3b, 4, 6, 35				
**C6**	52.13		6	7, 36, 37, 38				
**H6**	4.349	d 8.0(7), t 7.8(35)	6	5, 8, 35, 36	7, 35	1a, 4, 37, 38		7, 35, 36, 37, 38
**N7**	-265.5						7	
**H7**	8.198	d 8.0(6)		6, 8, 35	6	1a, 4, 9, 11, 12, 34, 35, 36	7	6, 35, 36, 37, 38
**C8**	172.81			6, 7, 9, 10a, 10b				
**C9**	61.83		9	10a, 10b, 12				
**H9**	4.397	m	9	8, 10, 11, 12	10a, 10b	1a, 4, 7, 10a, 12		10a, 10b, 11, 12
**C10**	29.43		10a, 10b	9, 12				
**H10a**	2.171	m	10	8, 9, 11, 12	9, 10b, 11, 12	9		9, 10b, 11, 12
**H10b**	1.950	m (o.l.)	10	8, 9, 11, 12	9, 10a, 12			9, 10a, 11, 12
**C11**	25.08		11	9, 10a, 10b, 12				
**H11**	1.963	m (o.l.)	11		10a, 12	7, 35, 36, 37, 38		9, 10a, 10b, 12
**C12**	47.57		12	9, 10a, 10b				
**H12**	3.723	m	12	9, 10, 11	10a, 10b, 11	7, 9, 15, 16, 33, 34		9, 10a, 10b, 11
**N13**	n.f.							
**C14**	170.20			15, 34				
**C15**	52.42		15	16, 34				
**H15**	4.925	d 8.5(16), t 6.4(34)	15	14, 34	16, 34	12		16, 34
**N16**	-265.1						16	
**H16**	7.721	d 8.5(15)		15, 17	15	12, 19, 24, 33	16	15, 34
**C17**	171.79			16, 18, 39a, 39b				
**C18**	50.66		18	19, 39a, 39b				
**H18**	4.908	d 4.5(39b), d 8.7(19), d 9.5(39a)	18	17, 20, 39, 40	19, 39a, 39b	41a		19, 39a, 39b
**N19**	-267.6						19	
**H19**	7.860	d 8.7(18)		18, 20	18	16, 21, 22, 28	19	18, 39a, 39b

**C20**	171.83			18, 19, 21, 42a, 42b				
**C21**	55.86		21	22, 42a, 42b, 43a, 43b				
**H21**	4.069	m (o.l.)	21	20, 23, 42, 43	22, 42a, 42b	19, 43a, 43b		22, 42a, 42b, 43a, 43b
**N22**	-263.2						22	
**H22**	8.882	d 4.5(21)		21, 42	21	19, 24, 42a, 42b, 45a, 45b, 46, 49, 50a, 52	22	21, 42a, 42b, 43a, 43b
**C23**	173.01			21				
**C24**	60.90	br	24	46, 47a, 47b, 49				
**H24**	4.082	m (o.l.)	24	26, 46, 47, 49	25, 46	16, 22, 25, 33, 34, 43a, 43b, 46, 47a, 47b, 48, 49		25, 46, 47a, 47b, 48, 49
**N25**	-259.9						25	
**H25**	8.922	br s			24	24, 27, 28	25	24, 46, 49
**C26**	174.36	br		24, 27, 50a, 50b				
**C27**	55.68		27	50a, 50b				
**H27**	4.789	d 8.1(28), d 11.3(50b), d 3.4(50a)	27	26, 29, 50, 51	28, 50a, 50b	25, 28, 50a, 52		28, 50a, 50b
**N28**	-258.2						28	
**H28**	9.434	d 8.1(27)		29	27	19, 25, 27, 30, 31a, 31b, 41a, 41b, 50b, 52	28	27, 50a, 50b
**C29**	168.27			27, 28, 31a, 31b				
**C30**	52.27		30	31a, 31b				
**H30**	4.524	br t 5.1(31a, b)	30		31a, 31b, 55	28, 31a, 31b, 32a, 52, 55		31a, 31b, 55
**C31**	33.02		31a, 31b	32a, 32b				
**H31a**	3.316	d 14.6(31b), d 5.1(30)	31	29, 30, 32	30, 31b	28, 30, 33		30, 31b, 55
**H31b**	3.255	d 5.8(30), d 14.6(31a)	31	29, 30, 32	30, 31a	28, 30, 33		30, 31a, 55
**C32**	32.52		32a, 32b	31a, 31b, 33				
**H32a**	2.788	m (o.l.)	32	31, 33	33	30		32b, 33
**H32b**	2.736	m (o.l.)	32	31, 33	33			32a, 33
**C33**	33.24		33	32a, 32b, 34				
**H33**	2.810	m (o.l.)	33	32, 34	32a, 32b	12, 16, 24, 31a, 31b, 34		32a, 32b

**C34**	33.86		34	15, 33				
**H34**	3.016	m	34	14, 15, 33	15	1a, 1b, 4, 7, 12, 24, 33		15, 16
**C35**	39.90		35	6, 7, 36, 37, 38				
**H35**	1.667	m (o.l.)	35	5, 37, 38	6	4, 7, 11		6, 7, 37, 38
**C36**	24.98		36	6, 37, 38				
**H36**	1.668	m (o.l.)	36	6, 35, 37, 38	37, 38	4, 7, 11		6, 7, 37, 38
**C37**	21.05		37	35, 36, 38				
**H37**	0.857	d 6.2(36)	37	6, 35, 36, 38	36	6, 11		6, 7, 35, 36, 38
**C38**	23.07		38	35, 36, 37				
**H38**	0.892	d 6.3(36)	38	6, 35, 36, 37	36	6, 11		6, 7, 35, 36, 37
**C39**	37.46	(br)	39a, 39b	18, 41b				
**H39a**	2.986	d 9.5(18), d 15.6(39b)	39	17, 18, 40	18, 39b	41a, 41b		18, 19, 39b
**H39b**	2.868	d 4.5(18), d 15.6(39a)	39	17, 18, 40	18, 39a	41a		18, 19, 39a
**C40**	172.31			18, 39a, 39b				
**N41**	-272.3						41a, 41b	
**H41a**	7.961	(br) s			41b	18, 28, 39a, 39b, 52	41	41b
**H41b**	7.068	(br) d1.7(41a)		39	41a	28, 39a	41	41a
**C42**	26.31		42a, 42b	21, 22, 43a, 43b				
**H42a**	2.015	m (o.l.)	42	20, 21, 43, 44	21, 43a, 43b	22		21, 22, 42b, 43a, 43b
**H42b**	1.979	m (o.l.)	42	20, 21, 43, 44	21, 43a, 43b	22		21, 22, 42a, 43a, 43b
**C43**	31.80		43a, 43b	21, 42a, 42b, 45b				
**H43a**	2.418	t 6.7(42a, b), d 16.0(43b)	43	21, 42, 44	42a, 42b	21, 24, 45a		21, 22, 42a, 42b
**H43b**	2.372	t 6.7(42a, b), d 16.0(43a)	43	21, 42, 44	42a, 42b	21, 24, 45a		21, 22, 42a, 42b
**C44**	175.70			42a, 42b, 43a, 43b, 45a, 45b				
**N45**	-273.5						45a, 45b	
**H45a**	7.620	(br) d 1.7(45b)		44	45b	22, 43a, 43b	45	45b

**H45b**	6.938	(br) d 1.7(45a)		43, 44	45a	22	45	45a
**C46**	36.33		46	24, 47a, 47b, 48, 49				
**H46**	1.928	m (o.l.)	46	24, 47, 48, 49	24, 47a, 47b, 49	22, 24, 48		24, 25, 47a, 47b, 48, 49
**C47**	26.01		47a, 47b	24, 46, 48, 49				
**H47a**	1.589	d 4.8(46), d 7.5(48), d 13.7(47b)	47	24, 46, 48, 49	46, 47b, 48	24, 49		24, 46, 47b, 48, 49
**H47b**	1.291	d 8.6(46), d 13.7(47a), d 7.4(48)	47	24, 46, 48, 49	46, 47a, 48	24, 49		24, 46, 47a, 48, 49
**C48**	11.35		48	46, 47a, 47b				
**H48**	0.914	t 7.4(47a, b)	48	46, 47	47a, 47b	24, 46, 49		24, 46, 47a, 47b, 49
**C49**	15.60		49	24, 46, 47a, 47b				
**H49**	0.994	d 6.9(46)	49	24, 46, 47	46	22, 24, 47a, 47b, 48		24, 25, 46, 47a, 47b, 48
**C50**	36.12		50a, 50b	27, 52				
**H50a**	3.375	d 3.4(27), d 14.2(50b)	50	26, 27, 51, 52	27, 50b	22, 27, 50b, 52		27, 28, 50b
**H50b**	2.996	d 11.3(27), d 14.2(50a)	50	26, 27, 51, 52	27, 50a	28, 50a, 52		27, 28, 50a
**C51**	128.71			27, 50a, 50b, 53				
**C52**	130.58		52	50a, 50b				
**H52**	7.257	m	52	50, 53, 54	53	22, 27, 28, 30, 41a, 50a, 50b		53
**C53**	115.23		53	52				
**H53**	6.742	m	53	51, 54	52			52
**C54**	156.72			52, 53				
**N55**	n.f.							
**H55**	8.697	br s			30	30		30, 31a, 31b
**C56**	159.29	q 35(F57)						
**C57**	116.98	q 293(F57)						
**F57**	-74.8							

^{1}H NMR of ethylene-bridged Oxytocin **(27):**
600 MHz, DMF-d$_7$, 298 K

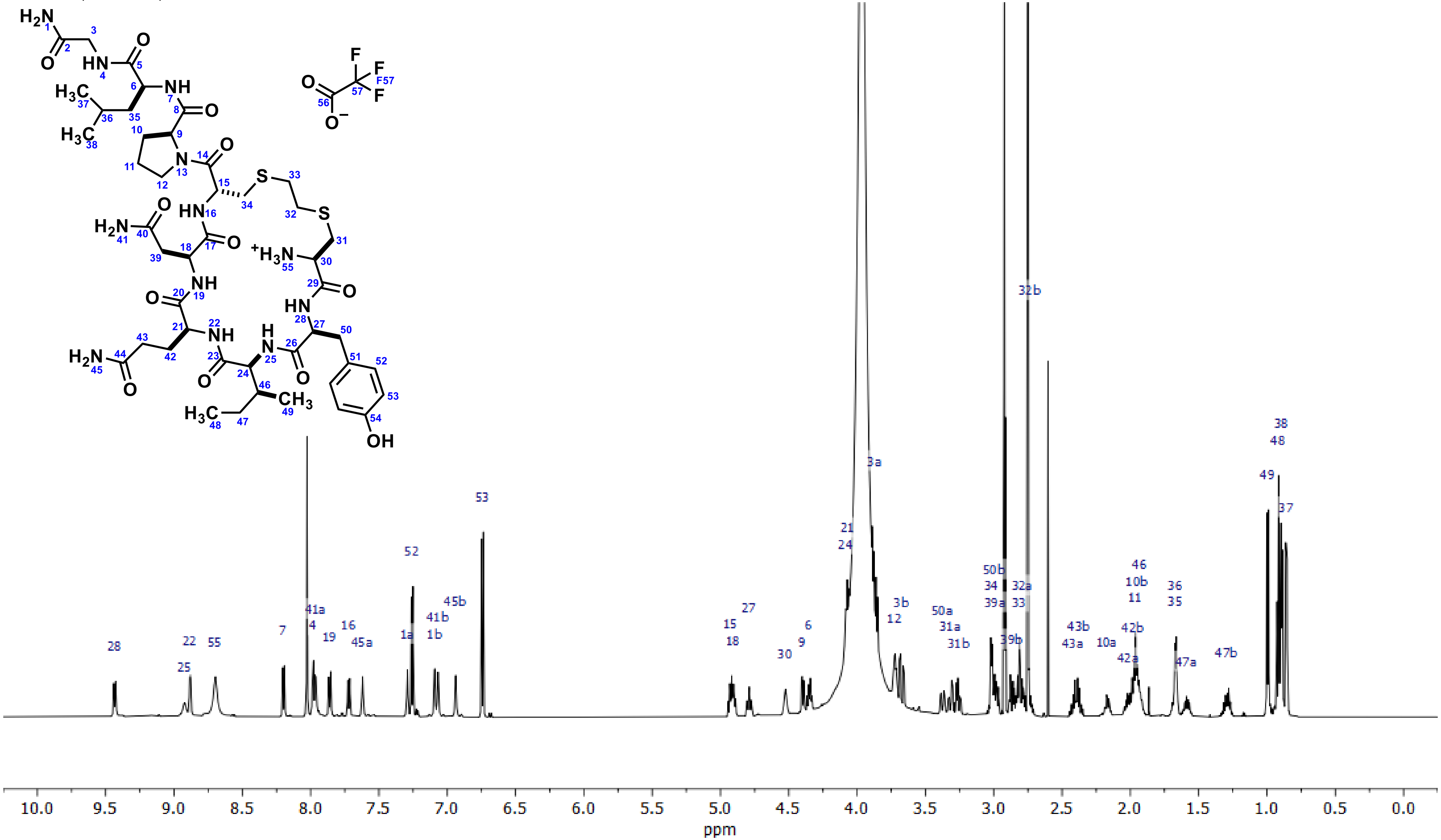

^{13}C NMR of ethylene-bridged Oxytocin **(27):**
151 MHz, DMF-d$_7$, 298 K

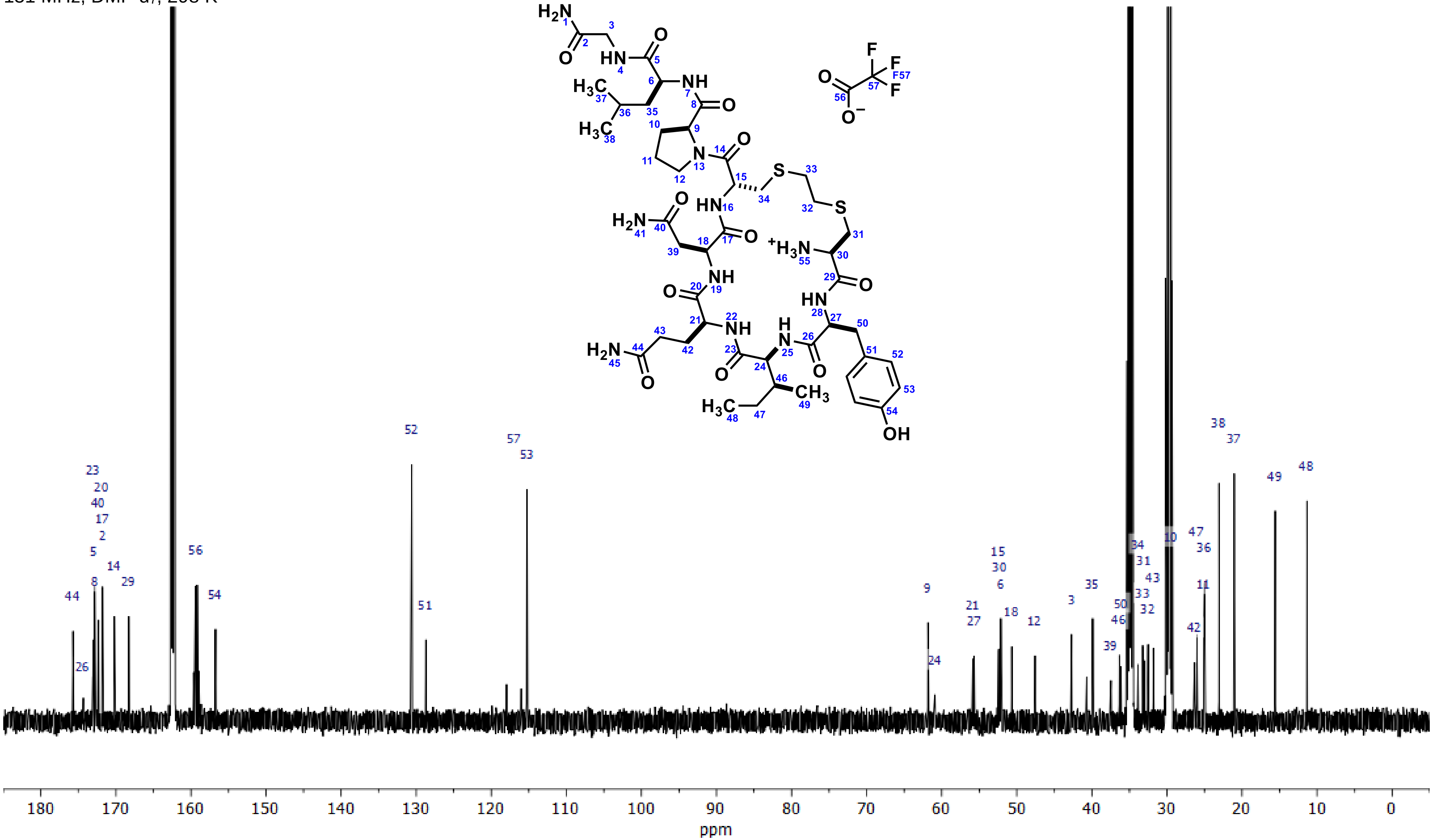

^{19}F NMR of ethylene-bridged Oxytocin **(27):**
471 MHz, DMF-d7, 298 K

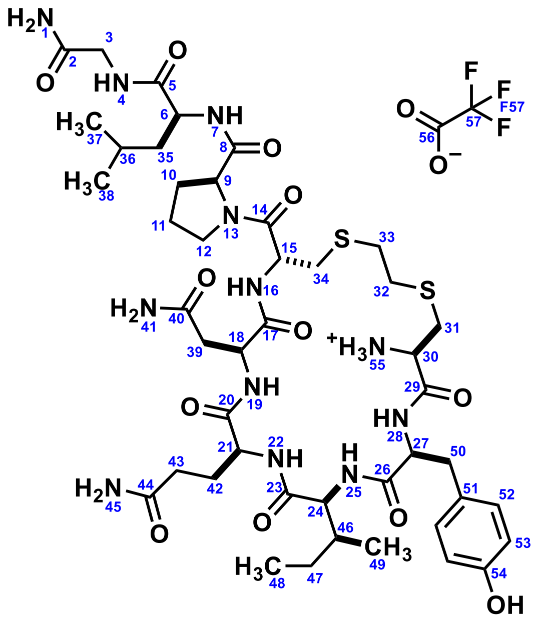

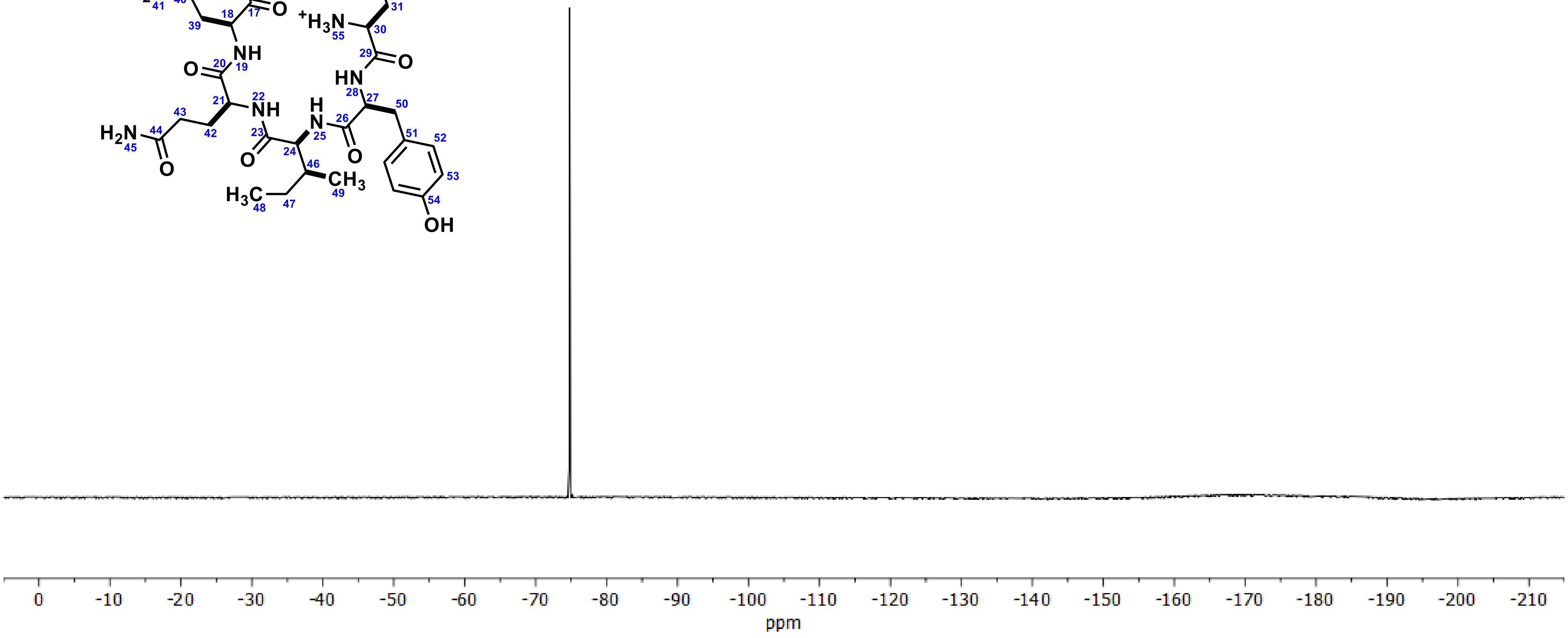

HSQC-NMR of ethylene-bridged Oxytocin **(27):**
DMF-d$_7$, 298 K

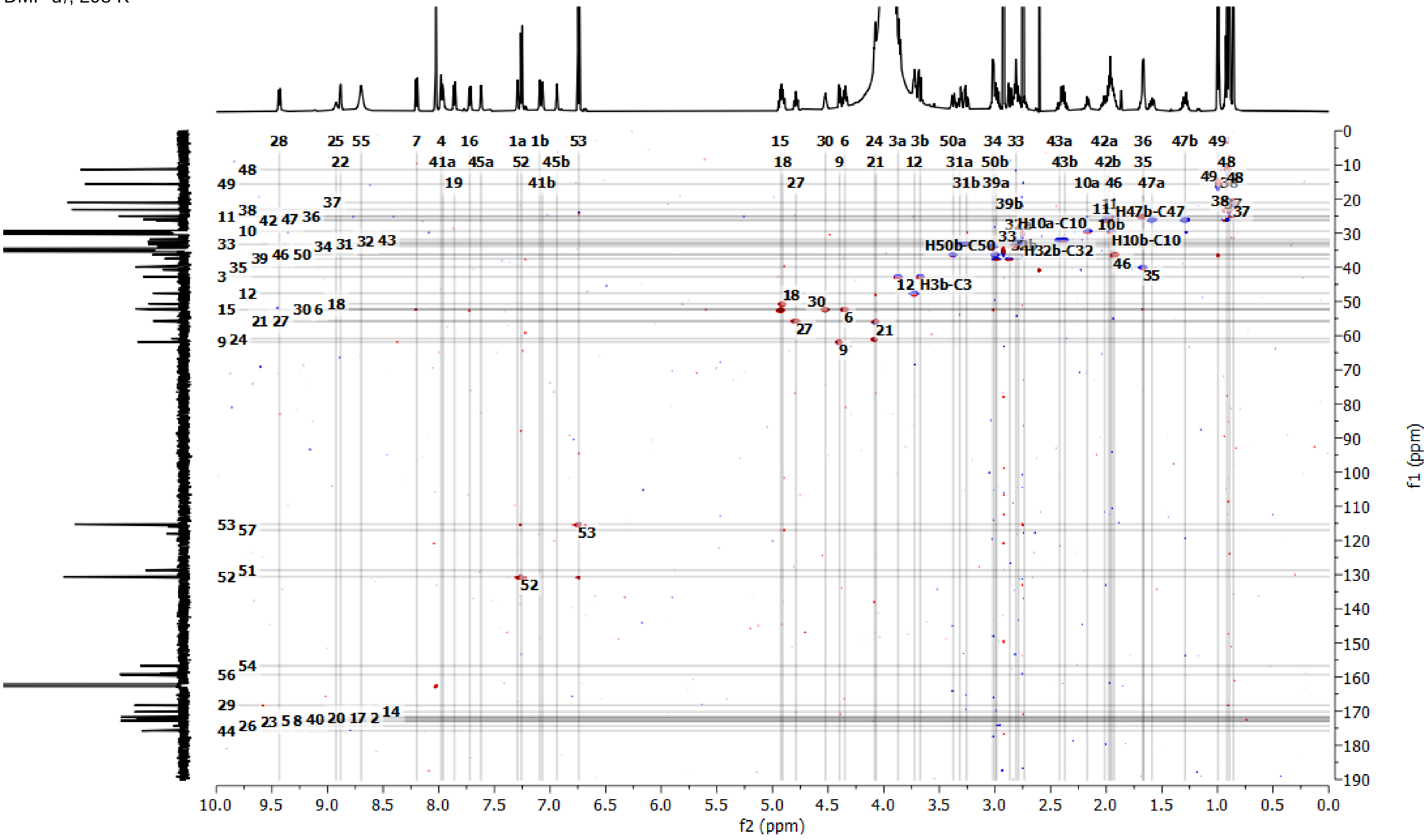

HMQC-NMR of ethylene-bridged Oxytocin **(27):**
DMF-d_7, 298 K

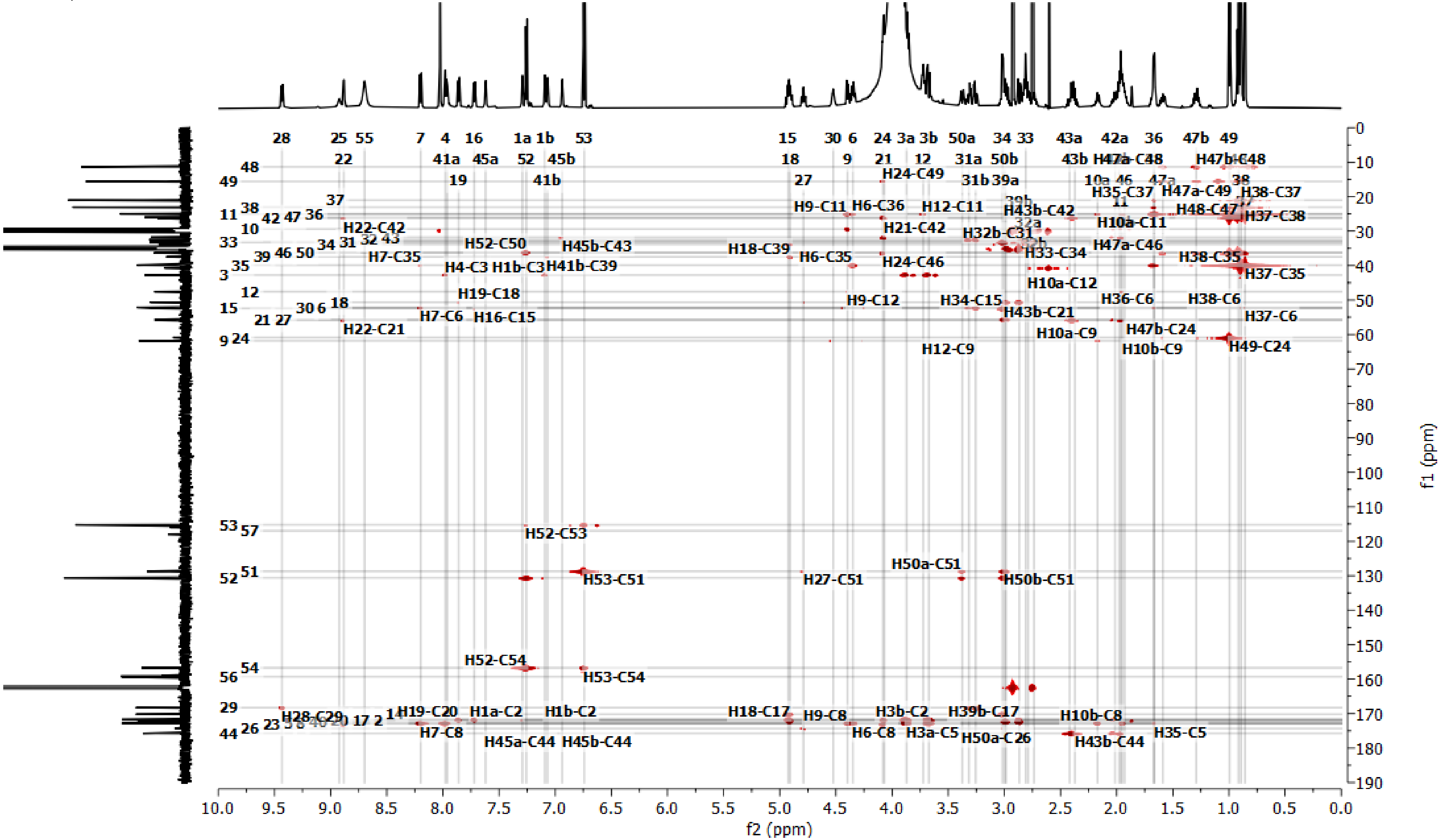

COSY-NMR of ethylene-bridged Oxytocin **(27):**
DMF-d_7, 298 K

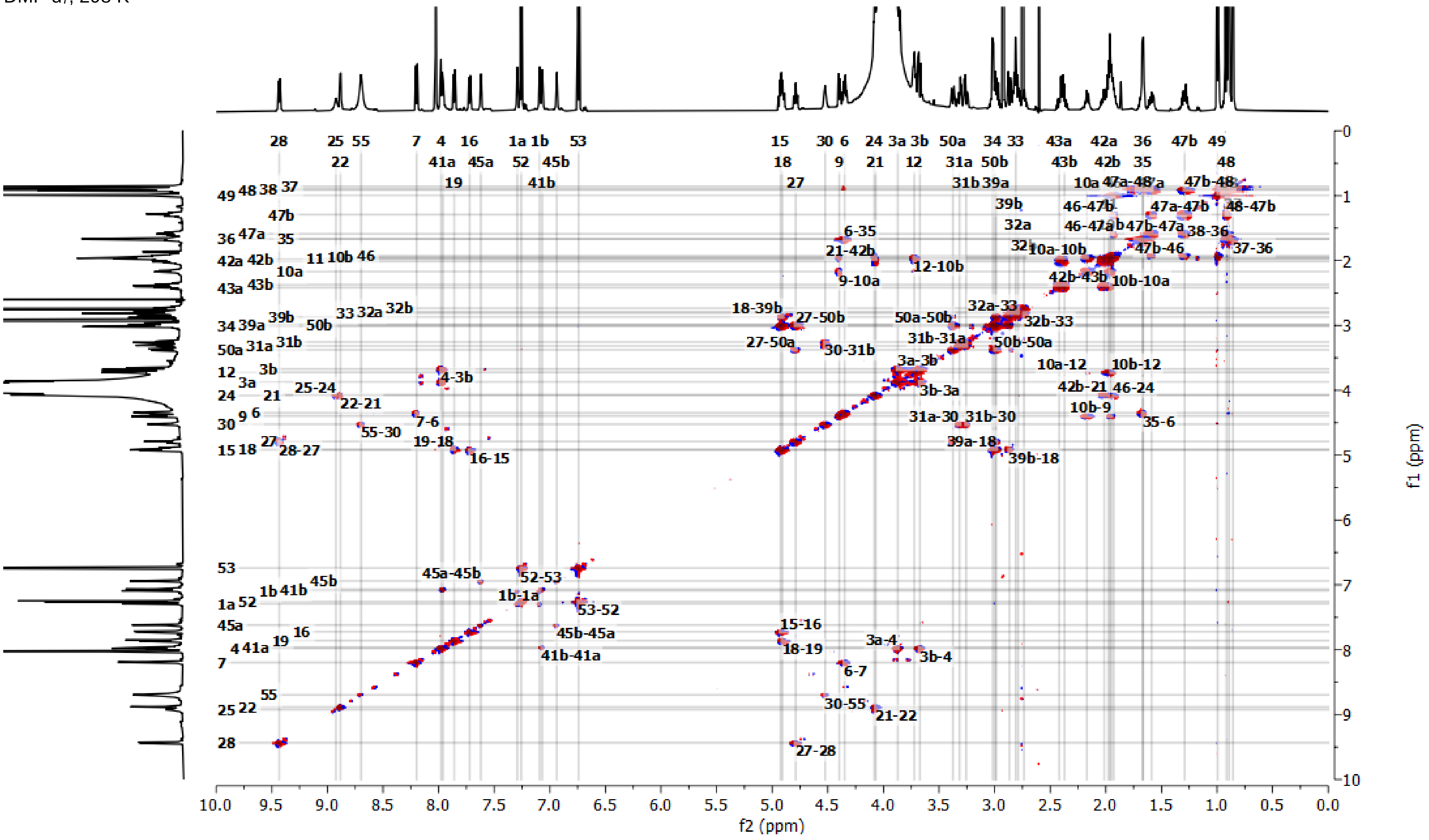

ROESY-NMR of ethylene-bridged Oxytocin **(27):**
DMF-d_7, 298 K

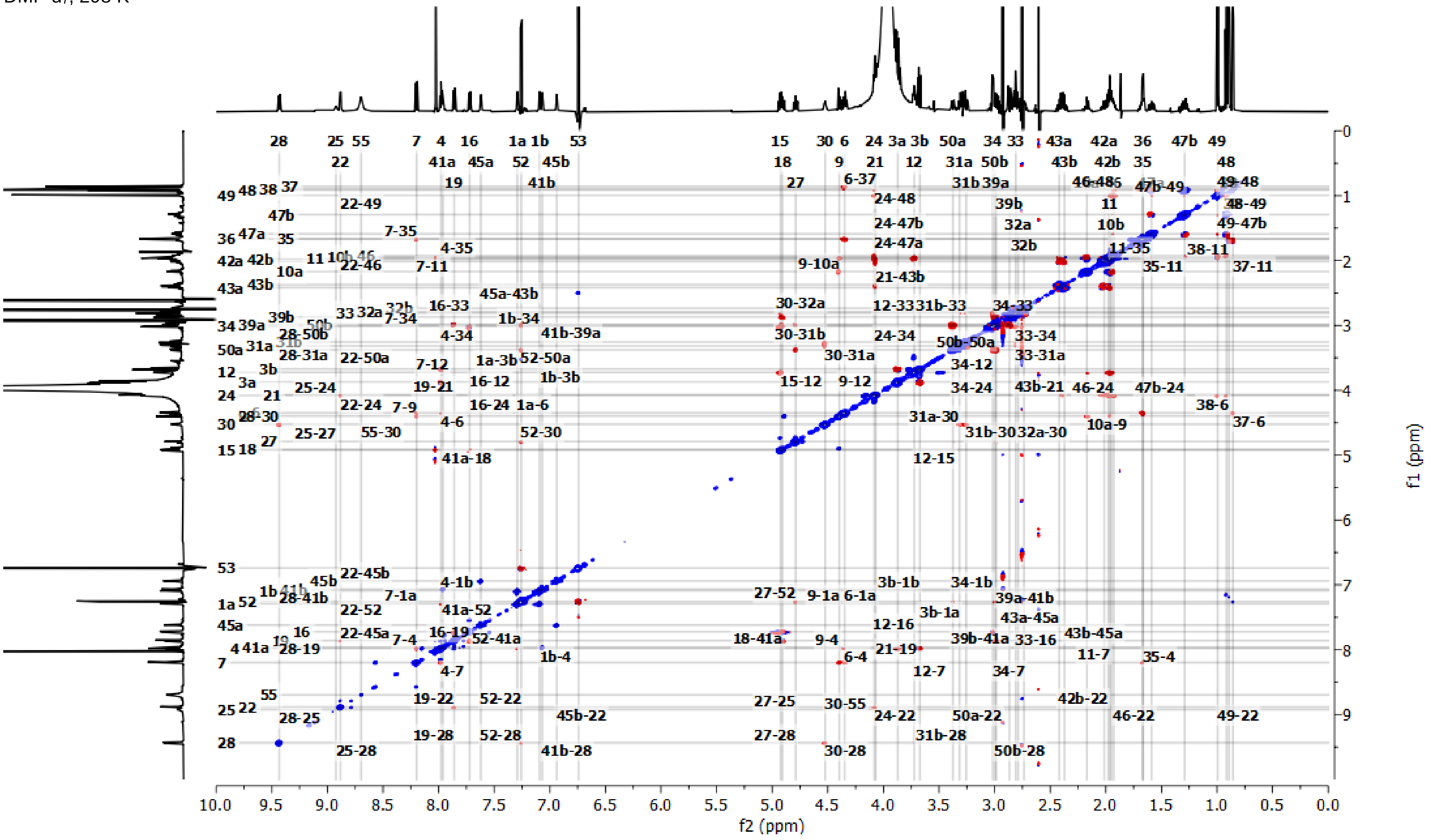

NOESY-NMR of ethylene-bridged Oxytocin **(27)**:
DMF-d_7, 298 K

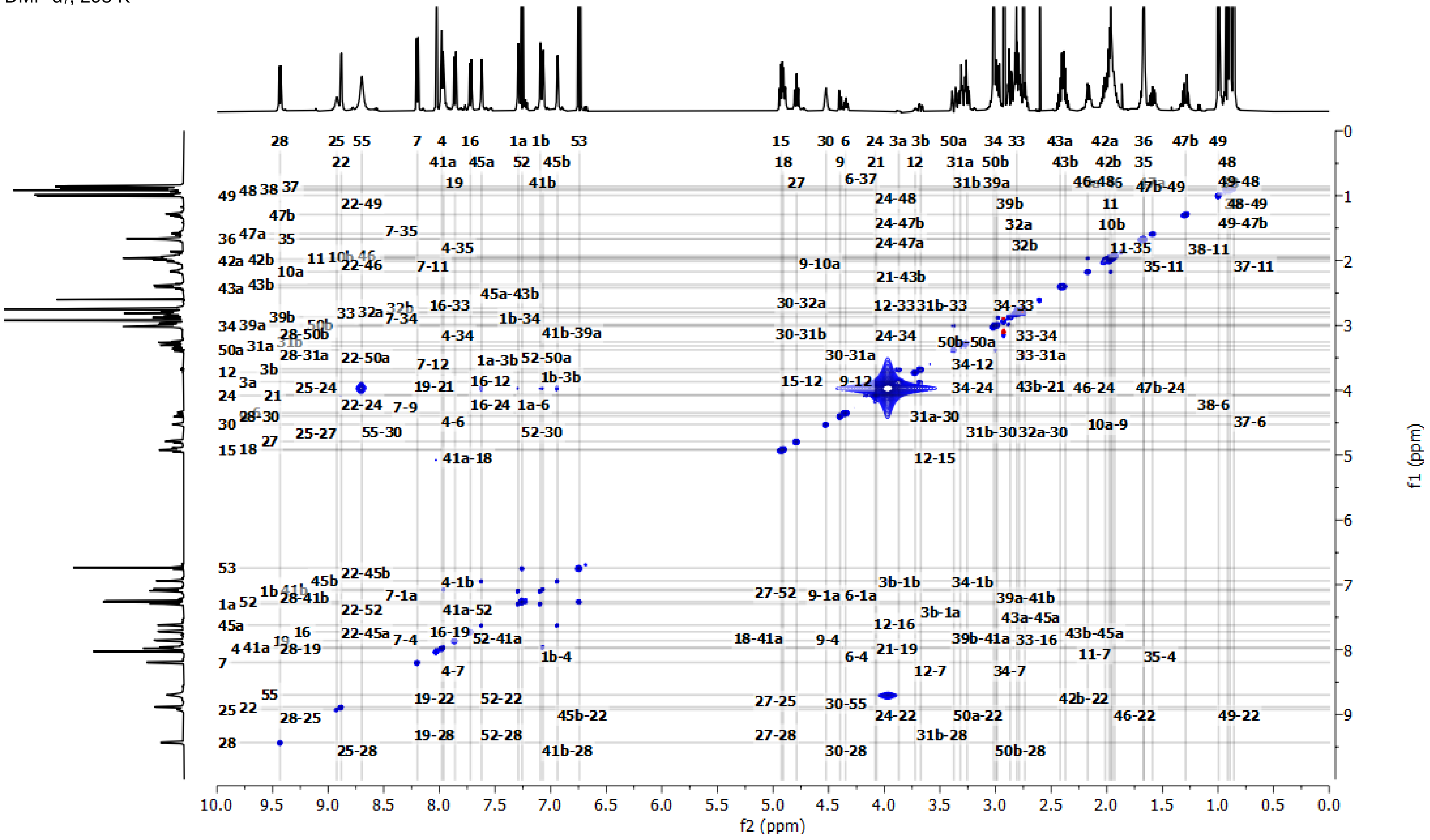

^{15}N-HSQC-NMR of ethylene-bridged Oxytocin **(27):**
DMF-d_7, 298 K

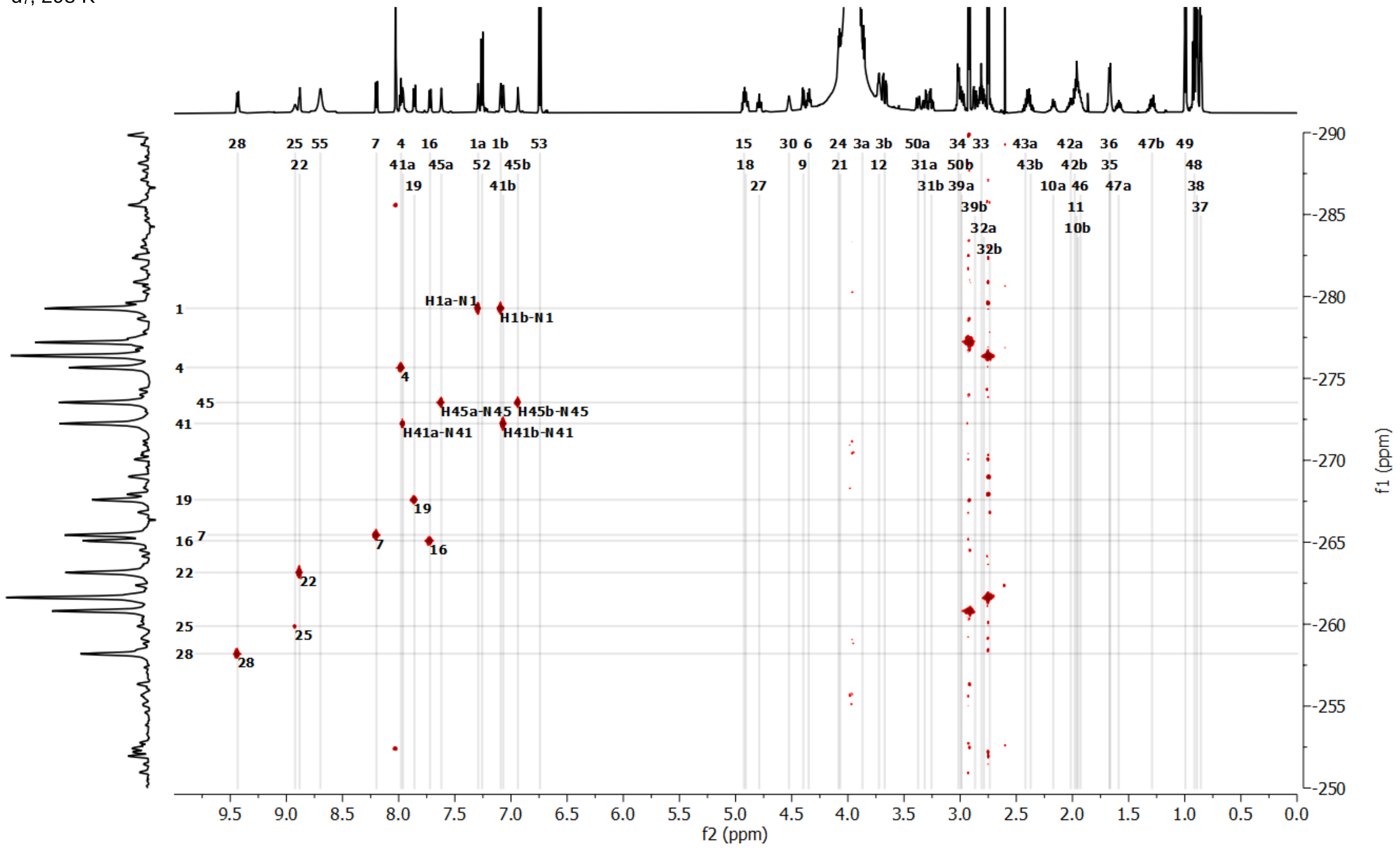

Protein NMR

General sample preparation and measurements

The 200-300 µL NMR samples with 0.4 – 1. mM of ^{15}N,^{13}C-Ubiquitin T12C, (^{15}N,^{13}C-Ubiquitin T12C)–S–$^{13}C_2H_4$–N_3 (**S15**), or (^{15}N,^{13}C-Ubiquitin T12C)–S–$^{13}C^2H_4$–(4-F-aniline) (**S17**) were prepared in sodium phosphate buffer (pH 7.0, 50 mM) containing 0.5 mM DTT and D_2O (10 %, v/v) within a water-matched 5 mm-Shigemi tube (Sigma-Aldrich). All NMR data were acquired at 298 K on an AVANCE III 600 spectrometer equipped with a triple-channel "TCI" cryogenic probehead (Bruker GmbH, Rheinstetten). All experiments used standard Bruker pulse sequence with standard parameter sets found in libraries of Topspin 3.6. The 3D spectra employed a non-uniform sampling (25% or more) scheme in the indirect dimension and were reconstructed and processed by NMRPipe/NMRDraw[32,33]. Backbone assignments were obtained using standard triple-resonance experiments (HNCO, HNCA, CBCAcoNH, HBHAcoNH)[34]. Aliphatic side-chain assignments were obtained from ^{13}C-edited 3D TOCSY (HcCH, hCCH, HcccoNH, and hCCcoNH) spectra. All spectra were analyzed with NMRFAM-SPARKY[35]. Spectra were referenced indirectly to DSS.

All models presented here are based on the first of the ten stopNMR-based structures of the human ubiquitin protein taken from the Protein Databank (pdb: 1d3z). These were modified at position 12 in PyMOL 2.5.4 (Schroedinger, Inc) and energy-minimized in Maestro 12.6.149 (Schroedinger, Inc)[36].

The corresponding ^{1}H, ^{13}C and ^{15}N chemical shifts for ^{15}N,^{13}C-Ubiquitin T12C and (^{15}N,^{13}C-Ubiquitin T12C)–S–$^{13}C_2H_4$–N_3 (**S15**) have been deposited in the BioMagRes-Bank[37], with accession no. 51721 and 51725, respectively.

The hydrogen exchange of exposed $NH/NH_2/NH_3^+$ groups with water is generally fast ($k_{ex} << 2\pi\Delta\delta$), so that these ^{1}H are often broadened beyond detection. Hence, HSQC cross-peaks for side-chain of Arginine and Lysine as well as N-terminal α-amine were not observed[38].

13C-HSQC of (^{15}N,^{13}C-Ubiquitin T12C)–S–$^{13}C_2H_4$–N_3 (S15)

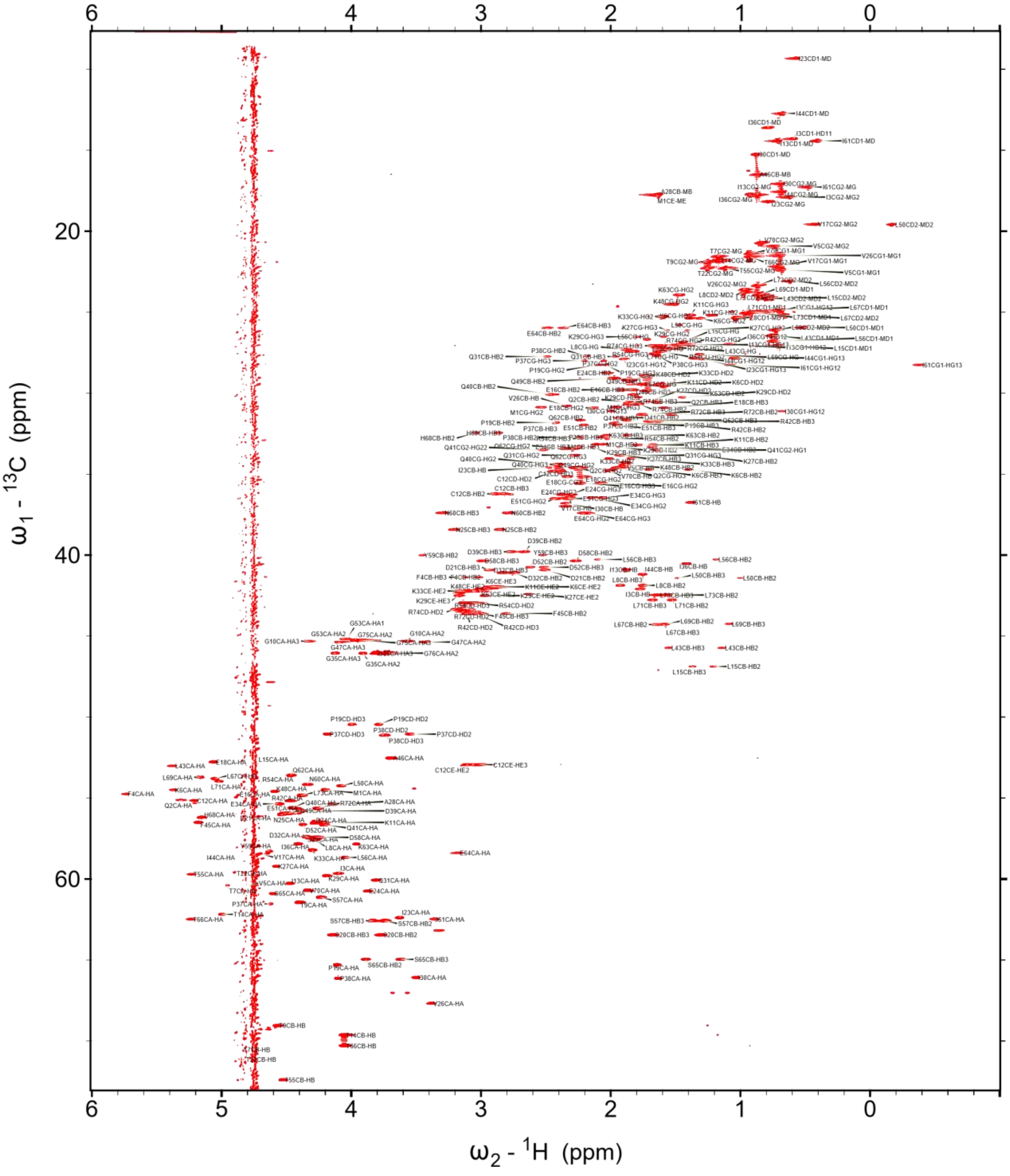

TURQUOISE: ^{13}C-HSQC of ^{15}N,^{13}C-Ubiquitin T12C

RED: ^{13}C-HSQC of (^{15}N,^{13}C-Ubiquitin T12C)–S–$^{13}C_2H_4$–N_3 (**S15**)

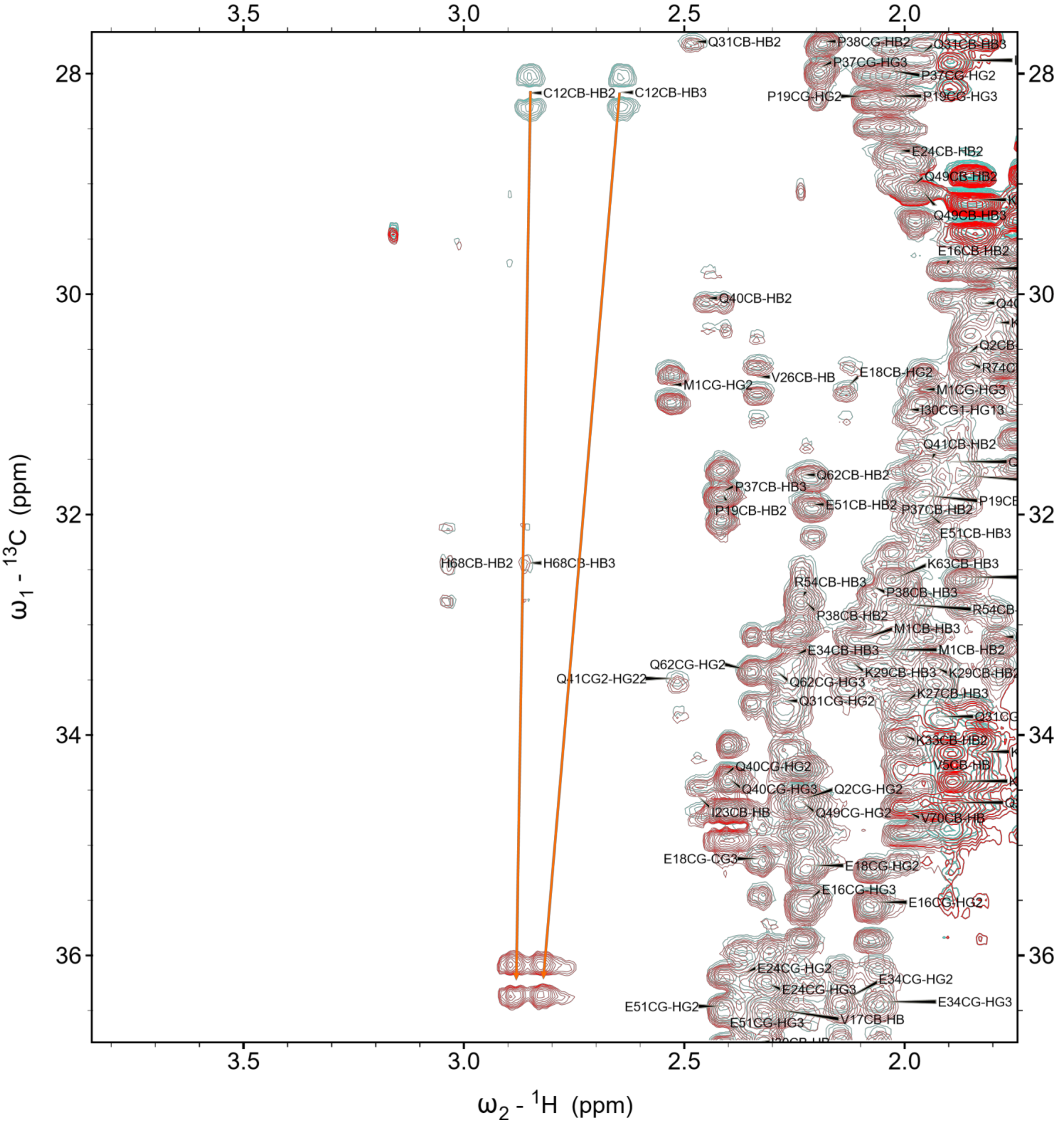

TURQUOISE: ^{13}C-HSQC of ^{15}N,^{13}C-Ubiquitin T12C

RED: ^{13}C-HSQC of (^{15}N,^{13}C-Ubiquitin T12C)–S–$^{13}C_2H_4$–N_3 (**S15**)

The spectra show the strong chemical shift variation of the ^{13}C-HSQC cross peaks of the β-carbon of cysteine 12.

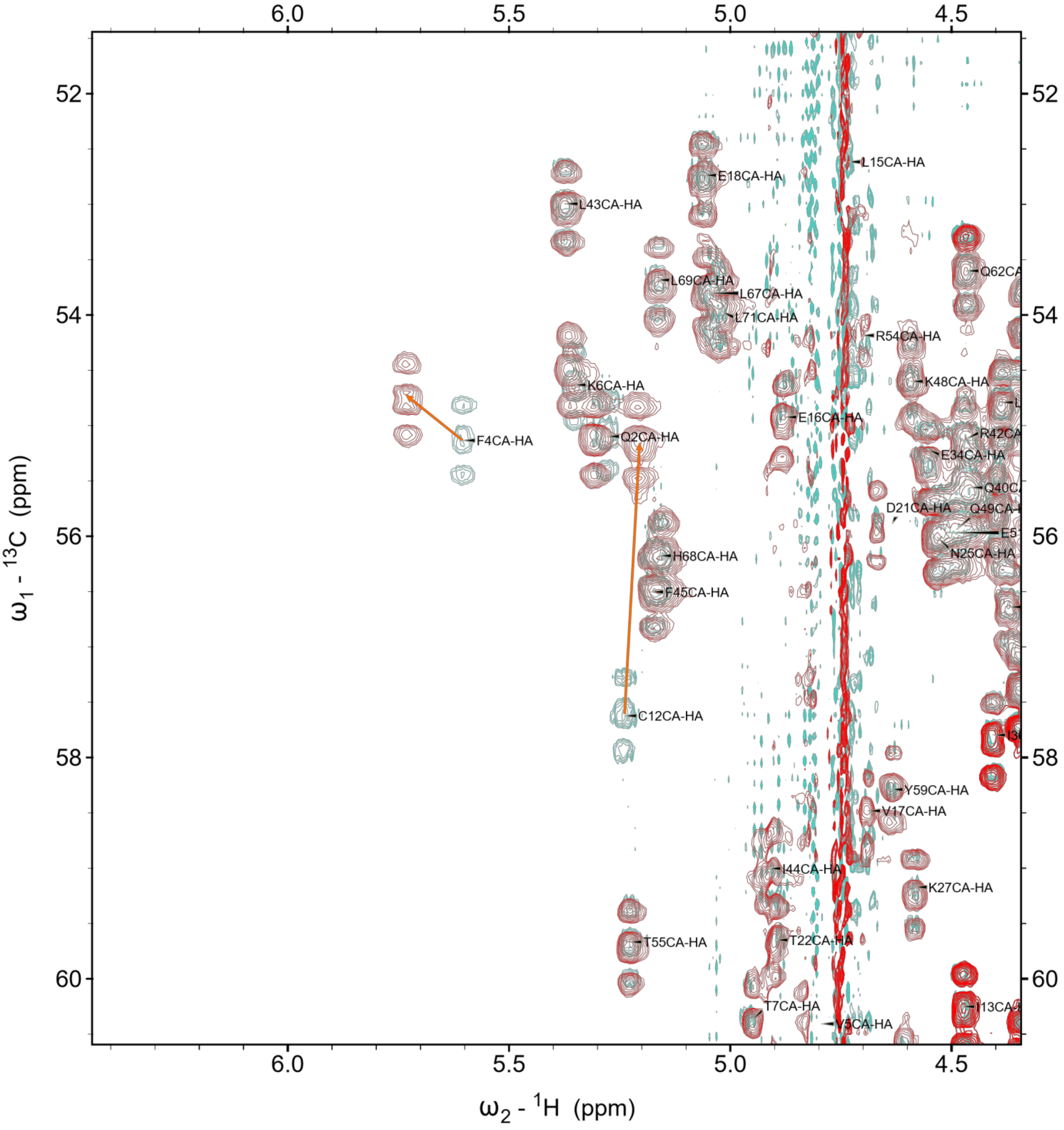

TURQUOISE: ^{13}C-HSQC of ^{15}N,^{13}C-Ubiquitin T12C

RED: ^{13}C-HSQC of (^{15}N,^{13}C-Ubiquitin T12C)–S–$^{13}C_2H_4$–N_3 (**S15**)

The spectra show the strong chemical shift variation of the ^{13}C-HSQC cross peak of the α-carbon of cysteine 12.

^{15}N-HSQC of (^{15}N,^{13}C-Ubiquitin T12C)–S–$^{13}C_2H_4$–N_3 (S15)

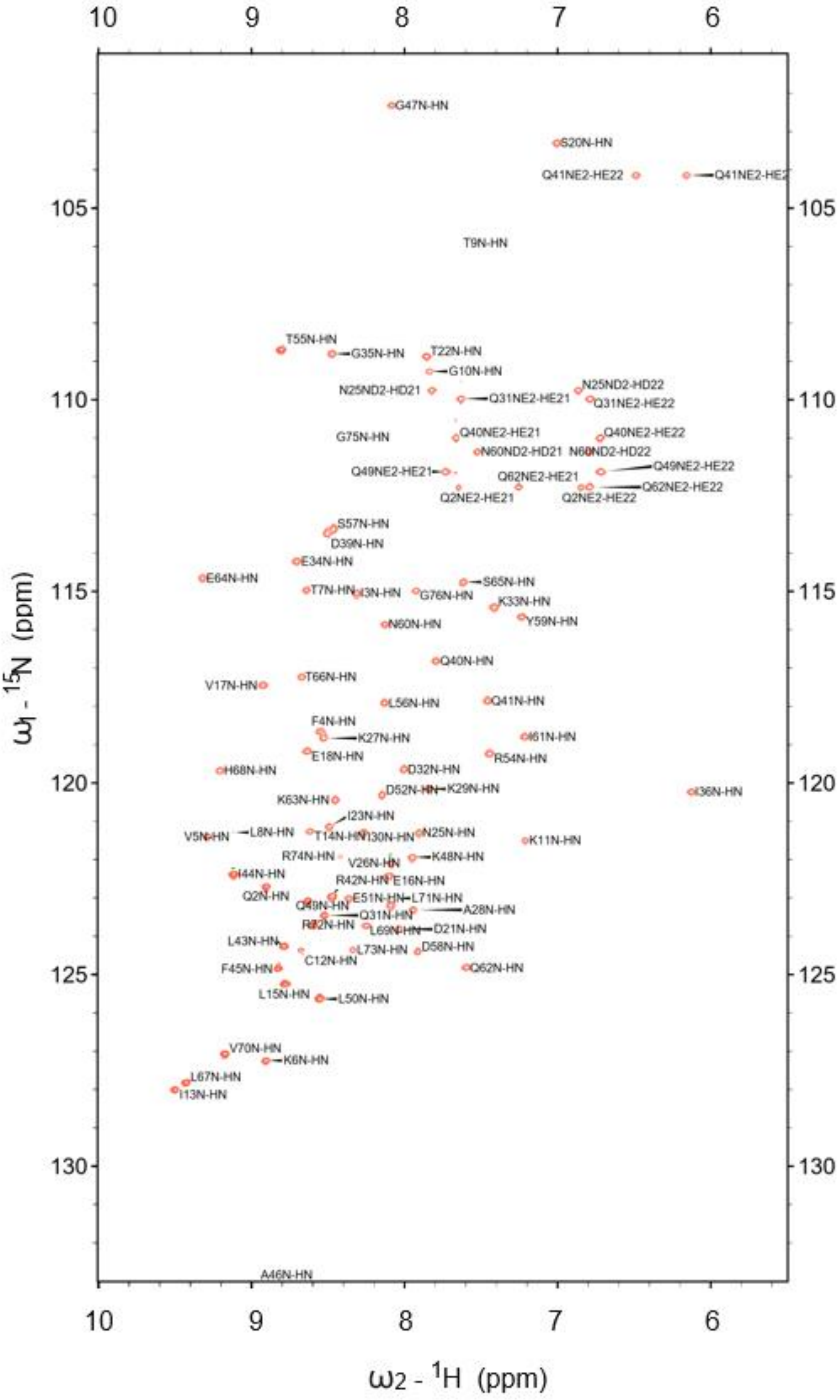

TURQUOISE: ^{15}N-HSQC of ^{15}N,^{13}C-Ubiquitin T12C

RED: ^{15}N-HSQC of (^{15}N,^{13}C-Ubiquitin T12C)–S–$^{13}C_2H_4$–N_3 (**S15**)

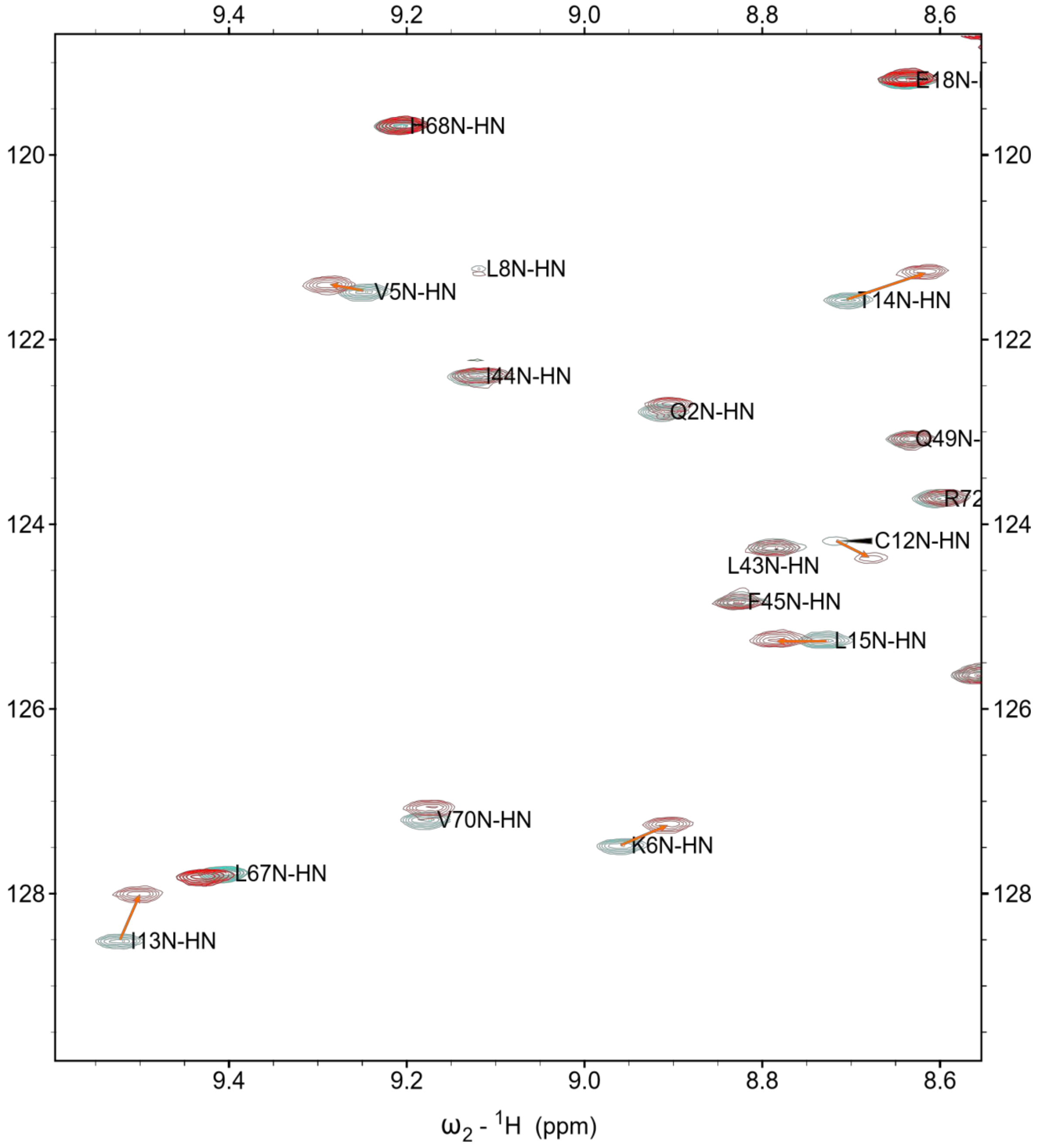

TURQUOISE: ^{15}N-HSQC of ^{15}N,^{13}C-Ubiquitin T12C

RED: ^{15}N-HSQC of (^{15}N,^{13}C-Ubiquitin T12C)–S–$^{13}C_2H_4$–N_3 (**S15**)

Zoom on relevant chemical shift variations of the ^{15}N-HSQC cross peaks including cysteine 12.

NMR shifts for (^{15}N,^{13}C-Ubiquitin T12C)–S–$^{13}C_2H_4$–N_3 (S15)

Primary sequence of ^{15}N,^{13}C-Ubiquitin T12C:

MQIFVKTLTGKCITLEVEPSDTIENVKAKIQDKEGIPPDQQRLIFAGKQLEDGRTLSDYNIQKESTLHLVLRL RGG

Table S37. ^{1}H and ^{15}N Chemical shifts of Human ^{15}N,^{13}C-Ubiquitin T12C with and without the ethyl-azide-linker and chemical shift differences (Δδ). Chemical shifts are referenced to DSS.

	Residue Atom Name (BMRB:IUPAC/IUB)			($^{15}N,^{13}C$-Ubiquitin T12C)–S–$^{13}C_2H_4$–N_3		$^{15}N,^{13}C$-Ubiquitin T12C		difference		scaled overall difference
Residue‡	Residue	H nucleus	N nucleus	δ(^{1}H)	δ(^{15}N)	δ(^{1}H)	δ(^{15}N)	Δδ(^{1}H)	Δδ(^{15}N)	Δδ *,**
				ppm	ppm	ppm	ppm	ppm	ppm	ppm
Q2N	Q2	HN	N	8.93	122.91	8.94	122.99	-0.01	-0.08	0.02
Q2NE2	Q2	HE21	NE2	7.67	112.50	7.67	112.51	0.00	-0.01	0.00
Q2NE2	Q2	HE22	NE2	6.87	112.50	6.89	112.51	-0.02	-0.01	0.01
I3N	I3	HN	N	8.34	115.26	8.32	115.25	0.02	0.01	0.01
F4N	F4	HN	N	8.58	118.87	8.59	118.67	-0.01	0.20	0.05
V5N	V5	HN	N	9.31	121.61	9.28	121.69	0.03	-0.08	0.03
K6N	K6	HN	N	8.93	127.46	8.99	127.69	-0.06	-0.23	0.07
T7N	T7	HN	N	8.67	115.18	8.64	115.06	0.03	0.12	0.04
L8N	L8	HN	N	9.15	121.50	9.15	121.44	0.00	0.06	0.02
T9N	T9	HN	N	7.62	105.87	7.63	105.94	-0.01	-0.07	0.02
G10N	G10	HN	N	7.86	109.47	7.87	109.48	-0.01	-0.01	0.01
K11N	K11	HN	N	7.24	121.72	7.25	121.75	-0.01	-0.03	0.01
C12N	C12	HN	N	8.70	124.57	8.75	124.39	-0.05	0.18	0.06
I13N	I13	HN	N	9.53	128.21	9.55	128.72	-0.02	-0.51	0.14
T14N	T14	HN	N	8.64	121.47	8.73	121.78	-0.09	-0.31	0.10
L15N	L15	HN	N	8.81	125.46	8.76	125.46	0.05	0.00	0.04
E16N	E16	HN	N	8.13	122.64	8.13	122.60	0.00	0.04	0.01
V17N	V17	HN	N	8.95	117.66	8.95	117.66	0.00	0.00	0.00
E18N	E18	HN	N	8.66	119.38	8.67	119.40	-0.01	-0.02	0.01
S20N	S20	HN	N	7.03	103.51	7.04	103.51	-0.01	0.00	0.01
D21N	D21	HN	N	8.06	124.02	8.06	124.02	0.00	0.00	0.00
T22N	T22	HN	N	7.88	109.08	7.88	109.08	0.00	0.00	0.00
I23N	I23	HN	N	8.52	121.35	8.52	121.37	0.00	-0.02	0.01
N25N	N25	HN	N	7.93	121.51	7.94	121.50	-0.01	0.01	0.01
N25ND2	N25	HD21	ND2	7.85	109.96	7.85	109.96	0.00	0.00	0.00
N25ND2	N25	HD22	ND2	6.89	109.96	6.89	109.96	0.00	0.00	0.00
V26N	V26	HN	N	8.12	122.30	8.12	122.29	0.00	0.01	0.00
K27N	K27	HN	N	8.55	119.03	8.55	119.02	0.00	0.01	0.00
A28N	A28	HN	N	7.97	123.52	7.98	123.50	-0.01	0.02	0.01
K29N	K29	HN	N	7.87	120.36	7.88	120.37	-0.01	-0.01	0.01
I30N	I30	HN	N	8.29	121.50	8.29	121.49	0.00	0.01	0.00
Q31N	Q31	HN	N	8.55	123.66	8.55	123.64	0.00	0.02	0.01
Q31NE2	Q31	HE21	NE2	7.66	110.18	7.66	110.18	0.00	0.00	0.00
Q31NE2	Q31	HE22	NE2	6.82	110.18	6.82	110.18	0.00	0.00	0.00
D32N	D32	HN	N	8.03	119.86	8.04	119.88	-0.01	-0.02	0.01
K33N	K33	HN	N	7.44	115.62	7.45	115.63	-0.01	-0.01	0.01
E34N	E34	HN	N	8.73	114.43	8.73	114.41	0.00	0.02	0.01
G35N	G35	HN	N	8.50	109.01	8.50	109.00	0.00	0.01	0.00
I36N	I36	HN	N	6.15	120.44	6.15	120.46	0.00	-0.02	0.01
D39N	D39	HN	N	8.53	113.69	8.53	113.69	0.00	0.00	0.00
Q40N	Q40	HN	N	7.82	117.03	7.82	117.04	0.00	-0.01	0.00

Q40NE2	Q40	HE21	NE2	7.69	111.20	7.69	111.14	0.00	0.06	0.02
Q40NE2	Q40	HE22	NE2	6.75	111.20	6.75	111.14	0.00	0.06	0.02
Q41N	Q41	HN	N	7.48	118.06	7.49	118.03	-0.01	0.03	0.01
Q41NE2	Q41	HE21	NE2	6.18	104.35	6.18	104.34	0.00	0.01	0.00
Q41NE2	Q41	HE22	NE2	6.51	104.35	6.51	104.34	0.00	0.01	0.00
R42N	R42	HN	N	8.50	123.17	8.50	123.12	0.00	0.05	0.01
L43N	L43	HN	N	8.81	124.47	8.81	124.45	0.00	0.02	0.01
I44N	I44	HN	N	9.14	122.59	9.15	122.60	-0.01	-0.01	0.01
F45N	F45	HN	N	8.85	125.05	8.85	125.05	0.00	0.00	0.00
A46N	A46	HN	N	9.00	133.06	9.00	133.05	0.00	0.01	0.00
G47N	G47	HN	N	8.11	102.53	8.12	102.54	-0.01	-0.01	0.01
K48N	K48	HN	N	7.98	122.15	7.97	122.13	0.01	0.02	0.01
Q49N	Q49	HN	N	8.66	123.29	8.66	123.28	0.00	0.01	0.00
Q49NE2	Q49	HE21	NE2	7.76	112.08	7.73	112.10	0.03	-0.02	0.02
Q49NE2	Q49	HE22	NE2	6.74	112.09	6.73	112.10	0.01	-0.01	0.01
L50N	L50	HN	N	8.58	125.84	8.59	125.84	-0.01	0.00	0.01
E51N	E51	HN	N	8.39	123.22	8.40	123.20	-0.01	0.02	0.01
D52N	D52	HN	N	8.17	120.53	8.18	120.52	-0.01	0.01	0.01
R54N	R54	HN	N	7.47	119.44	7.47	119.45	0.00	-0.01	0.00
T55N	T55	HN	N	8.83	108.91	8.83	108.90	0.00	0.01	0.00
L56N	L56	HN	N	8.16	118.12	8.16	118.12	0.00	0.00	0.00
S57N	S57	HN	N	8.49	113.58	8.49	113.58	0.00	0.00	0.00
D58N	D58	HN	N	7.94	124.61	7.95	124.64	-0.01	-0.03	0.01
Y59N	Y59	HN	N	7.26	115.87	7.26	115.87	0.00	0.00	0.00
N60N	N60	HN	N	8.15	116.07	8.16	116.06	-0.01	0.01	0.01
N60ND2	N60	HD21	ND2	7.55	111.57	7.56	111.58	-0.01	-0.01	0.01
N60ND2	N60	HD22	ND2	6.82	111.57	6.82	111.58	0.00	-0.01	0.00
I61N	I61	HN	N	7.24	119.01	7.25	119.01	-0.01	0.00	0.01
Q62N	Q62	HN	N	7.62	125.03	7.63	125.01	-0.01	0.02	0.01
Q62NE2	Q62	HE21	NE2	7.28	112.48	7.29	112.49	-0.01	-0.01	0.01
Q62NE2	Q62	HE22	NE2	6.82	112.49	6.82	112.49	0.00	0.00	0.00
K63N	K63	HN	N	8.48	120.65	8.49	120.67	-0.01	-0.02	0.01
E64N	E64	HN	N	9.35	114.87	9.34	114.82	0.01	0.05	0.02
S65N	S65	HN	N	7.64	114.97	7.68	115.05	-0.04	-0.08	0.04
T66N	T66	HN	N	8.70	117.44	8.71	117.54	-0.01	-0.10	0.03
L67N	L67	HN	N	9.45	128.03	9.44	127.99	0.01	0.04	0.01
H68N	H68	HN	N	9.23	119.89	9.24	119.89	-0.01	0.00	0.01
L69N	L69	HN	N	8.28	123.94	8.30	123.92	-0.02	0.02	0.02
V70N	V70	HN	N	9.20	127.28	9.21	127.41	-0.01	-0.13	0.04
L71N	L71	HN	N	8.12	123.41	8.14	123.49	-0.02	-0.08	0.03
R72N	R72	HN	N	8.62	123.92	8.63	123.93	-0.01	-0.01	0.01
L73N	L73	HN	N	8.36	124.57	8.37	124.53	-0.01	0.04	0.01
R74N	R74	HN	N	8.45	122.13	8.45	122.11	0.00	0.02	0.01
G75N	G75	HN	N	8.50	111.18	8.50	111.16	0.00	0.02	0.01
G76N	G76	HN	N	7.95	115.19	7.96	115.19	-0.01	0.00	0.01

‡: assignment for NMR analysis according to IUPAC: The first letter is the one letter code for the amino acid, the number describes the position of the amino acid in the primary sequence, the last letter N indicates that a nitrogen atom is considered. For Asn and Gln, the side-chain nitrogen atoms are designated as ND2, and NE2 respectively. In all other cases the backbone nitrogen is considered[39].

*: scaled perturbation corresponding to $\sqrt{\frac{(0.14 \times \Delta\delta(^{15}\mathrm{N})^2 + \Delta\delta(^{1}\mathrm{H})^2}{2}}$ as described before[40].

**: linear color scale from 0.00 ppm (RGB[255,255,255]) to 0.14 ppm (RGB[255,105,105])

Table S38. ^{1}H and ^{13}C Chemical shifts of Human ^{15}N,^{13}C-Ubiquitin T12C with and without the ethyl-azide-linker and chemical shift differences (Δδ). Chemical shifts are referenced to DSS.

	Residue Atom Name (BMRB:IUPAC/IUB)			(^{15}N,^{13}C-Ubiquitin T12C)–S–$^{13}C_2H_4$–N_3		^{15}N,^{13}C-Ubiquitin T12C		difference		scaled overall difference
Residue‡	Residue	H nucleus	C nucleus	δ(^{1}H)	δ(^{13}C)	δ(^{1}H)	δ(^{13}C)	Δδ(^{1}H)	Δδ(^{13}C)	Δδ*,**
				ppm	ppm	ppm	ppm	ppm	ppm	ppm
M1CA	M1	HA	CA	4.21	54.50	4.21	54.46	0.00	0.04	0.02
M1CB	M1	HB2	CB	2.05	33.16	2.05	33.23	0.00	-0.07	0.03
M1CB	M1	HB3	CB	2.11	33.15	2.10	33.13	0.01	0.02	0.01
M1CE	M1	ME	CE	1.65	17.78	1.65	17.78	0.00	0.00	0.00
M1CG	M1	HG2	CG	2.53	30.86	2.53	30.82	0.00	0.04	0.02
M1CG	M1	HG3	CG	1.96	30.86	1.96	30.86	0.00	0.00	0.00
Q2CA	Q2	HA	CA	5.31	55.12	5.28	55.10	0.03	0.02	0.02
Q2CB	Q2	HB2	CB	1.86	30.56	1.86	30.56	0.00	0.00	0.00
Q2CB	Q2	HB3	CB	1.64	30.57	1.61	30.56	0.03	0.01	0.02
Q2CG	Q2	HG2	CG	2.27	34.60	2.24	34.59	0.03	0.01	0.02
Q2CG	Q2	HG3	CG	1.95	34.60	1.90	34.61	0.05	-0.01	0.04
I3CA	I3	HA	CA	4.10	59.66	4.14	59.59	-0.04	0.07	0.04
I3CB	I3	HB	CB	1.77	42.09	1.75	42.03	0.02	0.06	0.03
I3CD1	I3	MD	CD1	0.59	14.29	0.58	14.24	0.01	0.05	0.02
I3CG1	I3	HG12	CG1	0.69	24.88	0.69	24.88	0.00	0.00	0.00
I3CG2	I3	MG2	CG2	0.62	17.89	0.61	17.85	0.01	0.04	0.02
F4CA	F4	HA	CA	5.74	54.74	5.61	55.13	0.13	-0.39	0.18
F4CB	F4	HB2	CB	3.02	41.37	2.97	41.29	0.05	0.08	0.05
F4CB	F4	HB3	CB	3.13	41.37	3.00	41.29	0.13	0.08	0.10
V5CA	V5	HA	CA	4.80	60.41	4.80	60.41	0.00	0.00	0.00
V5CB	V5	HB	CB	1.89	34.27	1.89	34.27	0.00	0.00	0.00
V5CG1	V5	MG1	CG1	0.68	22.38	0.68	22.38	0.00	0.00	0.00
V5CG2	V5	MG2	CG2	0.73	20.92	0.73	20.82	0.00	0.10	0.04
K6CA	K6	HA	CA	5.37	54.49	5.36	54.63	0.01	-0.14	0.05
K6CB	K6	HB2	CB	1.40	34.72	1.40	34.63	0.00	0.09	0.03
K6CB	K6	HB3	CB	1.70	34.72	1.71	34.61	-0.01	0.11	0.04
K6CD	K6	HD2	CD	1.58	29.50	1.60	29.42	-0.02	0.08	0.03
K6CE	K6	HE2	CE	2.87	41.96	2.90	41.94	-0.03	0.02	0.02
K6CE	K6	HE3	CE	2.90	41.96	2.90	41.94	0.00	0.02	0.01
K6CG	K6	HG2	CG	1.31	25.37	1.31	25.37	0.00	0.00	0.00
K6CG	K6	HG3	CG	1.39	25.37	1.39	25.36	0.00	0.01	0.00
T7CA	T7	HA	CA	4.96	60.40	4.96	60.40	0.00	0.00	0.00
T7CB	T7	HB	CB	4.85	70.70	4.85	70.70	0.00	0.00	0.00

T7CG2	T7	MG	CG2	1.17	21.55	1.18	21.45	-0.01	0.10	0.04
L8CA	L8	HA	CA	4.29	57.57	4.29	57.68	0.00	-0.11	0.04
L8CB	L8	HB2	CB	1.75	41.87	1.75	41.70	0.00	0.17	0.07
L8CB	L8	HB3	CB	1.93	41.87	1.92	41.81	0.01	0.06	0.02
L8CD1	L8	MD1	CD1	1.04	25.32	1.03	25.28	0.01	0.04	0.02
L8CD2	L8	MD2	CD2	0.97	23.76	0.97	23.75	0.00	0.01	0.00
L8CG	L8	HG	CG	1.88	27.31	1.87	27.29	0.01	0.02	0.01
T9CA	T9	HA	CA	4.41	61.44	4.40	61.46	0.01	-0.02	0.01
T9CB	T9	HB	CB	4.57	69.05	4.57	69.07	0.00	-0.02	0.01
T9CG2	T9	MG	CG2	1.26	21.88	1.25	21.83	0.01	0.05	0.02
G10CA	G10	HA2	CA	3.57	45.31	3.57	45.26	0.00	0.05	0.02
G10CA	G10	HA3	CA	4.33	45.31	4.35	45.29	-0.02	0.02	0.02
K11CA	K11	HA	CA	4.22	56.50	4.22	56.35	0.00	0.15	0.06
K11CB	K11	HB2	CB	1.67	33.19	1.67	33.05	0.00	0.14	0.05
K11CB	K11	HB3	CB	1.79	33.20	1.78	33.11	0.01	0.09	0.04
K11CD	K11	HD2	CD	1.62	29.47	1.61	29.42	0.01	0.05	0.02
K11CE	K11	HE2	CE	2.94	41.96	2.90	41.93	0.04	0.03	0.03
K11CG	K11	HG2	CG	1.22	25.19	1.22	25.10	0.00	0.09	0.03
K11CG	K11	HG3	CG	1.39	25.19	1.38	25.12	0.01	0.07	0.03
C12CA	C12	HA	CA	5.21	55.16	5.24	57.62	-0.03	-2.46	0.95
C12CB	C12	HB2	CB	2.90	36.22	2.85	28.17	0.05	8.05	3.12
C12CB	C12	HB3	CB	2.83	36.21	2.65	28.17	0.18	8.04	3.12
C12CD	C12	*HD2*	*CD*	*2.43*	*34.81*	-	-	-	-	-
C12CD	C12	*HD3*	*CD*	*2.39*	*34.80*	-	-	-	-	-
C12CE	C12	*HE2*	*CE*	*3.11*	*52.94*	-	-	-	-	-
C12CE	C12	*HE3*	*CE*	*3.02*	*52.94*	-	-	-	-	-
I13CA	I13	HA	CA	4.48	60.27	4.48	60.25	0.00	0.02	0.01
I13CB	I13	HB	CB	1.88	40.90	1.89	40.71	-0.01	0.19	0.07
I13CD1	I13	MD	CD1	0.71	14.43	0.72	14.47	-0.01	-0.04	0.02
I13CG1	I13	HG12	CG1	1.09	27.01	1.09	26.95	0.00	0.06	0.02
I13CG1	I13	HG13	CG1	1.48	27.01	1.48	27.01	0.00	0.00	0.00
I13CG2	I13	MG	CG2	0.87	17.76	0.87	17.75	0.00	0.01	0.00
T14CA	T14	HA	CA	4.95	62.13	5.00	62.18	-0.05	-0.05	0.04
T14CB	T14	HB	CB	4.06	69.63	4.04	69.58	0.02	0.05	0.02
T14CG2	T14	MG	CG2	1.17	21.79	1.12	21.84	0.05	-0.05	0.04
L15CA	L15	HA	CA	4.74	52.62	4.74	52.62	0.00	0.00	0.00
L15CB	L15	HB2	CB	1.21	46.88	1.20	46.91	0.01	-0.03	0.01
L15CB	L15	HB3	CB	1.37	46.87	1.35	46.91	0.02	-0.04	0.02
L15CD1	L15	MD1	CD1	0.71	27.07	0.70	27.04	0.01	0.03	0.01
L15CD2	L15	MD2	CD2	0.76	24.11	0.75	24.11	0.01	0.00	0.01
L15CG	L15	HG	CG	1.44	26.82	1.44	26.82	0.00	0.00	0.00
E16CA	E16	HA	CA	4.88	54.92	4.88	54.92	0.00	0.00	0.00
E16CB	E16	HB2	CB	1.91	29.79	1.91	29.76	0.00	0.03	0.01
E16CB	E16	HB3	CB	1.83	29.79	1.82	29.77	0.01	0.02	0.01
E16CG	E16	HG2	CG	2.09	35.56	2.08	35.52	0.01	0.04	0.02
E16CG	E16	HG3	CG	2.23	35.55	2.22	35.50	0.01	0.05	0.02
V17CA	V17	HA	CA	4.69	58.48	4.69	58.48	0.00	0.00	0.00

V17CB	V17	HB	CB	2.32	36.43	2.31	36.48	0.01	-0.05	0.02
V17CG1	V17	MG1	CG1	0.71	22.17	0.70	22.09	0.01	0.08	0.03
V17CG2	V17	MG2	CG2	0.42	19.57	0.42	19.54	0.00	0.03	0.01
E18CA	E18	HA	CA	5.06	52.77	5.06	52.74	0.00	0.03	0.01
E18CB	E18	HB3	CB	1.59	30.90	1.59	30.81	0.00	0.09	0.03
E18CB	E18	HG2	CB	2.14	30.90	2.14	30.86	0.00	0.04	0.02
E18CG	E18	CG3	CG	2.33	35.15	2.33	35.12	0.00	0.03	0.01
E18CG	E18	HG2	CG	2.21	35.15	2.22	35.18	-0.01	-0.03	0.01
P19CA	P19	HA	CA	4.11	65.30	4.11	65.18	0.00	0.12	0.05
P19CB	P19	HB2	CB	2.41	31.81	2.41	31.81	0.00	0.00	0.00
P19CB	P19	HB3	CB	1.96	31.82	1.96	31.82	0.00	0.00	0.00
P19CD	P19	HD2	CD	3.79	50.45	3.79	50.42	0.00	0.03	0.01
P19CD	P19	HD3	CD	4.00	50.45	4.00	50.41	0.00	0.04	0.02
P19CG	P19	HG2	CG	2.10	28.24	2.09	28.20	0.01	0.04	0.02
P19CG	P19	HG3	CG	2.04	28.25	2.04	28.20	0.00	0.05	0.02
S20CA	S20	HA	CA	4.35	57.40	4.35	57.40	0.00	0.00	0.00
S20CB	S20	HB2	CB	3.77	63.45	3.77	63.45	0.00	0.00	0.00
S20CB	S20	HB3	CB	4.14	63.45	4.15	63.44	-0.01	0.01	0.01
D21CA	D21	HA	CA	4.64	55.89	4.64	55.89	0.00	0.00	0.00
D21CB	D21	HB2	CB	2.51	40.91	2.51	40.91	0.00	0.00	0.00
D21CB	D21	HB3	CB	2.94	40.92	2.93	40.88	0.01	0.04	0.02
T22CA	T22	HA	CA	4.90	59.65	4.90	59.65	0.00	0.00	0.00
T22CB	T22	HB	CB	4.83	71.12	4.83	71.12	0.00	0.00	0.00
T22CG2	T22	MG	CG2	1.26	22.26	1.25	22.25	0.01	0.01	0.01
I23CA	I23	HA	CA	3.63	62.39	3.62	62.35	0.01	0.04	0.02
I23CB	I23	HB	CB	2.48	34.59	2.47	34.55	0.01	0.04	0.02
I23CD1	I23	MD	CD1	0.57	9.33	0.57	9.32	0.00	0.01	0.00
I23CG1	I23	HG12	CG1	1.90	27.89	1.90	27.88	0.00	0.01	0.00
I23CG1	I23	HG13	CG1	1.29	27.88	1.29	27.88	0.00	0.00	0.00
I23CG2	I23	MG	CG2	0.78	18.16	0.78	18.12	0.00	0.04	0.02
E24CA	E24	HA	CA	3.88	60.75	3.87	60.68	0.01	0.07	0.03
E24CB	E24	HB2	CB	2.02	28.75	2.02	28.70	0.00	0.05	0.02
E24CG	E24	HG2	CG	2.37	36.26	2.37	36.16	0.00	0.10	0.04
E24CG	E24	HG3	CG	2.31	36.26	2.31	36.26	0.00	0.00	0.00
N25CA	N25	HA	CA	4.54	56.00	4.54	56.00	0.00	0.00	0.00
N25CB	N25	HB2	CB	2.86	38.42	2.86	38.37	0.00	0.05	0.02
N25CB	N25	HB3	CB	3.22	38.42	3.21	38.40	0.01	0.02	0.01
V26CA	V26	HA	CA	3.39	67.68	3.38	67.64	0.01	0.04	0.02
V26CB	V26	HB	CB	2.34	30.78	2.34	30.75	0.00	0.03	0.01
V26CG1	V26	MG1	CG1	0.69	21.50	0.69	21.44	0.00	0.06	0.02
V26CG2	V26	MG2	CG2	0.97	23.61	0.97	23.53	0.00	0.08	0.03
K27CA	K27	HA	CA	4.58	59.22	4.58	59.17	0.00	0.05	0.02
K27CB	K27	HB2	CB	1.43	33.72	1.43	33.67	0.00	0.05	0.02
K27CB	K27	HB3	CB	2.00	33.71	2.00	33.72	0.00	-0.01	0.00
K27CD	K27	HD2	CD	1.70	30.51	1.70	30.51	0.00	0.00	0.00
K27CE	K27	HE2	CE	2.63	42.41	2.63	42.40	0.00	0.01	0.00
K27CG	K27	HG2	CG	1.41	26.12	1.41	26.12	0.00	0.00	0.00

K27CG	K27	HG3	CG	1.56	26.11	1.58	26.21	-0.02	-0.10	0.04
A28CA	A28	HA	CA	4.15	55.39	4.15	55.35	0.00	0.04	0.02
A28CB	A28	MB	CB	1.63	17.72	1.62	17.66	0.01	0.06	0.02
K29CA	K29	HA	CA	4.19	59.80	4.19	59.75	0.00	0.05	0.02
K29CB	K29	HB2	CB	1.94	33.33	1.93	33.38	0.01	-0.05	0.02
K29CB	K29	HB3	CB	2.14	33.33	2.12	33.33	0.02	0.00	0.01
K29CD	K29	HD2	CD	1.45	30.26	1.45	30.23	0.00	0.03	0.01
K29CD	K29	HD3	CD	1.79	30.26	1.79	30.26	0.00	0.00	0.00
K29CE	K29	HE2	CE	2.97	42.50	2.97	42.46	0.00	0.04	0.02
K29CE	K29	HE3	CE	3.17	42.50	3.17	42.50	0.00	0.00	0.00
K29CG	K29	HG2	CG	1.60	26.51	1.60	26.51	0.00	0.00	0.00
K29CG	K29	HG3	CG	1.79	26.52	1.78	26.47	0.01	0.05	0.02
I30CA	I30	HA	CA	3.50	66.09	3.49	66.04	0.01	0.05	0.02
I30CB	I30	HB	CB	2.35	36.80	2.34	36.79	0.01	0.01	0.01
I30CD1	I30	MD	CD1	0.89	15.26	0.88	15.24	0.01	0.02	0.01
I30CG1	I30	HG12	CG1	0.68	31.12	0.68	31.05	0.00	0.07	0.03
I30CG1	I30	HG13	CG1	2.00	31.11	2.00	31.05	0.00	0.06	0.02
I30CG2	I30	MG	CG2	0.69	17.07	0.68	17.02	0.01	0.05	0.02
Q31CA	Q31	HA	CA	3.81	60.07	3.81	60.03	0.00	0.04	0.02
Q31CB	Q31	HB2	CB	2.49	27.74	2.48	27.71	0.01	0.03	0.01
Q31CB	Q31	HB3	CB	1.97	27.74	1.97	27.83	0.00	-0.09	0.03
Q31CG	Q31	HG2	CG	2.28	33.82	2.27	33.69	0.01	0.13	0.05
Q31CG	Q31	HG3	CG	1.93	33.83	1.93	33.83	0.00	0.00	0.00
D32CA	D32	HA	CA	4.33	57.46	4.33	57.46	0.00	0.00	0.00
D32CB	D32	HB2	CB	2.75	41.08	2.74	41.02	0.01	0.06	0.02
D32CB	D32	HB3	CB	2.83	41.08	2.83	41.04	0.00	0.04	0.02
K33CA	K33	HA	CA	4.30	58.22	4.30	58.24	0.00	-0.02	0.01
K33CB	K33	HB2	CB	2.02	34.04	2.01	33.99	0.01	0.05	0.02
K33CB	K33	HB3	CB	1.84	34.03	1.84	34.15	0.00	-0.12	0.05
K33CD	K33	HD2	CD	1.71	28.91	1.70	28.87	0.01	0.04	0.02
K33CE	K33	HE2	CE	3.12	42.22	3.11	42.14	0.01	0.08	0.03
K33CG	K33	HG2	CG	1.60	25.26	1.59	25.21	0.01	0.05	0.02
E34CA	E34	HA	CA	4.56	55.36	4.56	55.21	0.00	0.15	0.06
E34CB	E34	HB2	CB	1.68	33.32	1.67	33.24	0.01	0.08	0.03
E34CB	E34	HB3	CB	2.25	33.32	2.25	33.28	0.00	0.04	0.02
E34CG	E34	HG2	CG	2.14	36.46	2.14	36.42	0.00	0.04	0.02
E34CG	E34	HG3	CG	2.06	36.45	2.06	36.42	0.00	0.03	0.01
G35CA	G35	HA2	CA	3.91	46.05	3.91	46.01	0.00	0.04	0.02
G35CA	G35	HA3	CA	4.13	46.04	4.13	46.04	0.00	0.00	0.00
I36CA	I36	HA	CA	4.41	57.83	4.41	57.80	0.00	0.03	0.01
I36CB	I36	HB	CB	1.41	40.52	1.41	40.48	0.00	0.04	0.02
I36CD1	I36	MD	CD1	0.79	13.60	0.78	13.57	0.01	0.03	0.01
I36CG1	I36	HG12	CG1	1.08	26.99	1.08	26.98	0.00	0.01	0.00
I36CG2	I36	MG	CG2	0.93	17.71	0.93	17.71	0.00	0.00	0.00
P37CA	P37	HA	CA	4.62	61.54	4.62	61.50	0.00	0.04	0.02
P37CB	P37	HB2	CB	1.99	31.85	1.99	31.85	0.00	0.00	0.00
P37CB	P37	HB3	CB	2.43	31.85	2.42	31.80	0.01	0.05	0.02

P37CD	P37	HD2	CD	3.55	51.05	3.55	51.02	0.00	0.03	0.01
P37CD	P37	HD3	CD	4.19	51.06	4.19	51.03	0.00	0.03	0.01
P37CG	P37	HG2	CG	2.05	28.00	2.05	27.96	0.00	0.04	0.02
P37CG	P37	HG3	CG	2.20	28.00	2.20	27.95	0.00	0.05	0.02
P38CA	P38	HA	CA	4.10	66.13	4.10	66.13	0.00	0.00	0.00
P38CB	P38	HB2	CB	2.25	32.73	2.23	32.76	0.02	-0.03	0.02
P38CB	P38	HB3	CB	2.08	32.72	2.07	32.65	0.01	0.07	0.03
P38CD	P38	HD2	CD	3.76	51.13	3.74	51.08	0.02	0.05	0.02
P38CD	P38	HD3	CD	3.73	51.13	3.73	51.08	0.00	0.05	0.02
P38CG	P38	HB2	CG	2.19	27.71	2.19	27.71	0.00	0.00	0.00
P38CG	P38	HG3	CG	1.64	27.72	1.64	27.67	0.00	0.05	0.02
D39CA	D39	HA	CA	4.40	55.79	4.40	55.76	0.00	0.03	0.01
D39CB	D39	HB2	CB	2.66	39.79	2.66	39.76	0.00	0.03	0.01
D39CB	D39	HB3	CB	2.77	39.79	2.76	39.76	0.01	0.03	0.01
Q40CA	Q40	HA	CA	4.46	55.61	4.46	55.56	0.00	0.05	0.02
Q40CB	Q40	HB2	CB	2.46	30.08	2.45	30.04	0.01	0.04	0.02
Q40CB	Q40	HB3	CB	1.83	30.08	1.83	30.08	0.00	0.00	0.00
Q40CG	Q40	HG2	CG	2.42	34.40	2.42	34.39	0.00	0.01	0.00
Q40CG	Q40	HG3	CG	2.41	34.40	2.40	34.39	0.01	0.01	0.01
Q41CA	Q41	HA	CA	4.21	56.66	4.21	56.66	0.00	0.00	0.00
Q41CB	Q41	HB2	CB	1.94	31.51	1.94	31.51	0.00	0.00	0.00
Q41CB	Q41	HB3	CB	1.90	31.52	1.90	31.52	0.00	0.00	0.00
Q41CG2	Q41	HG1	CG2	1.65	33.51	1.66	33.48	-0.01	0.03	0.01
Q41CG2	Q41	HG22	CG2	2.52	33.51	2.52	33.49	0.00	0.02	0.01
R42CA	R42	HA	CA	4.47	55.17	4.47	55.12	0.00	0.05	0.02
R42CB	R42	HB2	CB	1.63	31.74	1.62	31.70	0.01	0.04	0.02
R42CB	R42	HB3	CB	1.70	31.74	1.70	31.70	0.00	0.04	0.02
R42CD	R42	HD2	CD	3.11	43.62	3.11	43.62	0.00	0.00	0.00
R42CD	R42	HD3	CD	3.04	43.62	3.04	43.58	0.00	0.04	0.02
R42CG	R42	HG2	CG	1.39	27.02	1.39	27.02	0.00	0.00	0.00
L43CA	L43	HA	CA	5.38	53.02	5.38	53.00	0.00	0.02	0.01
L43CB	L43	HB2	CB	1.14	45.73	1.14	45.69	0.00	0.04	0.02
L43CB	L43	HB3	CB	1.55	45.72	1.55	45.68	0.00	0.04	0.02
L43CD1	L43	MD1	CD1	0.76	26.44	0.76	26.44	0.00	0.00	0.00
L43CD2	L43	MD2	CD2	0.80	24.15	0.80	24.15	0.00	0.00	0.00
L43CG	L43	HG	CG	1.48	27.22	1.48	27.22	0.00	0.00	0.00
I44CA	I44	HA	CA	4.92	59.00	4.92	59.00	0.00	0.00	0.00
I44CB	I44	HB	CB	1.75	41.19	1.74	41.21	0.01	-0.02	0.01
I44CD1	I44	MD	CD1	0.68	12.72	0.67	12.71	0.01	0.01	0.01
I44CG1	I44	HG12	CG1	1.35	27.83	1.35	27.83	0.00	0.00	0.00
I44CG1	I44	HG13	CG1	1.05	27.83	1.04	27.80	0.01	0.03	0.01
I44CG2	I44	MG	CG2	0.68	17.56	0.67	17.52	0.01	0.04	0.02
F45CA	F45	HA	CA	5.18	56.50	5.18	56.50	0.00	0.00	0.00
F45CB	F45	HB2	CB	2.81	43.61	2.81	43.58	0.00	0.03	0.01
F45CB	F45	HB3	CB	3.00	43.60	3.00	43.60	0.00	0.00	0.00
A46CA	A46	HA	CA	3.69	52.53	3.69	52.50	0.00	0.03	0.01
A46CB	A46	MB	CB	0.86	16.49	0.87	16.46	-0.01	0.03	0.01

G47CA	G47	HA2	CA	3.43	45.37	3.43	45.32	0.00	0.05	0.02
G47CA	G47	HA3	CA	4.09	45.37	4.09	45.31	0.00	0.06	0.02
K48CA	K48	HA	CA	4.59	54.60	4.59	54.60	0.00	0.00	0.00
K48CB	K48	HB2	CB	1.90	34.42	1.90	34.42	0.00	0.00	0.00
K48CD	K48	HD2	CD	1.86	29.18	1.85	29.14	0.01	0.04	0.02
K48CE	K48	HE2	CE	3.16	42.26	3.16	42.20	0.00	0.06	0.02
K48CG	K48	HG2	CG	1.51	24.50	1.50	24.46	0.01	0.04	0.02
Q49CA	Q49	HA	CA	4.49	55.95	4.49	55.95	0.00	0.00	0.00
Q49CB	Q49	HB2	CB	1.99	29.08	1.99	29.03	0.00	0.05	0.02
Q49CB	Q49	HB3	CB	1.96	29.09	1.97	29.03	-0.01	0.06	0.02
Q49CG	Q49	HG2	CG	2.24	34.60	2.24	34.60	0.00	0.00	0.00
L50CA	L50	HA	CA	4.08	54.24	4.08	54.22	0.00	0.02	0.01
L50CB	L50	HB2	CB	1.00	41.42	1.00	41.38	0.00	0.04	0.02
L50CB	L50	HB3	CB	1.48	41.42	1.48	41.38	0.00	0.04	0.02
L50CD1	L50	MD1	CD1	0.51	25.95	0.51	25.92	0.00	0.03	0.01
L50CD2	L50	MD2	CD2	0.17	19.57	0.18	19.53	-0.01	0.04	0.02
L50CG	L50	HG	CG	1.47	25.79	1.47	25.90	0.00	-0.11	0.04
E51CA	E51	HA	CA	4.51	55.97	4.51	55.97	0.00	0.00	0.00
E51CB	E51	HB2	CB	2.21	31.96	2.21	31.91	0.00	0.05	0.02
E51CB	E51	HB3	CB	1.95	31.96	1.95	31.96	0.00	0.00	0.00
E51CG	E51	HG2	CG	2.42	36.51	2.41	36.46	0.01	0.05	0.02
E51CG	E51	HG3	CG	2.33	36.51	2.33	36.51	0.00	0.00	0.00
D52CA	D52	HA	CA	4.37	56.64	4.37	56.64	0.00	0.00	0.00
D52CB	D52	HB2	CB	2.62	40.73	2.61	40.70	0.01	0.03	0.01
D52CB	D52	HB3	CB	2.52	40.75	2.51	40.62	0.01	0.13	0.05
G53CA	G53	HA1	CA	4.03	45.18	4.04	45.14	-0.01	0.04	0.02
G53CA	G53	HA2	CA	4.06	45.18	4.05	45.14	0.01	0.04	0.02
R54CA	R54	HA	CA	4.70	54.19	4.70	54.19	0.00	0.00	0.00
R54CB	R54	HB2	CB	2.03	32.81	2.03	32.81	0.00	0.00	0.00
R54CB	R54	HB3	CB	2.24	32.81	2.23	32.76	0.01	0.05	0.02
R54CD	R54	HD2	CD	3.07	42.95	3.07	42.91	0.00	0.04	0.02
R54CD	R54	HD3	CD	3.13	42.94	3.13	42.91	0.00	0.03	0.01
R54CG	R54	HG2	CG	1.61	27.42	1.61	27.40	0.00	0.02	0.01
R54CG	R54	HG3	CG	1.82	27.42	1.82	27.39	0.00	0.03	0.01
T55CA	T55	HA	CA	5.23	59.71	5.23	59.67	0.00	0.04	0.02
T55CB	T55	HB	CB	4.53	72.40	4.53	72.35	0.00	0.05	0.02
T55CG2	T55	MG	CG2	1.12	22.28	1.12	22.23	0.00	0.05	0.02
L56CA	L56	HA	CA	4.05	58.67	4.05	58.63	0.00	0.04	0.02
L56CB	L56	HB2	CB	1.19	40.27	1.19	40.23	0.00	0.04	0.02
L56CB	L56	HB3	CB	2.10	40.27	2.10	40.23	0.00	0.04	0.02
L56CD1	L56	MD1	CD1	0.75	26.78	0.75	26.78	0.00	0.00	0.00
L56CD2	L56	MD2	CD2	0.62	23.07	0.61	23.06	0.01	0.01	0.01
L56CG	L56	HG	CG	1.72	26.68	1.72	26.68	0.00	0.00	0.00
S57CA	S57	HA	CA	4.24	61.13	4.24	61.07	0.00	0.06	0.02
S57CB	S57	HB2	CB	3.75	62.58	3.74	62.55	0.01	0.03	0.01
S57CB	S57	HB3	CB	3.84	62.58	3.83	62.55	0.01	0.03	0.01
D58CA	D58	HA	CA	4.27	57.43	4.27	57.43	0.00	0.00	0.00

D58CB	D58	HB2	CB	2.27	40.35	2.27	40.34	0.00	0.01	0.00
D58CB	D58	HB3	CB	2.99	40.36	2.99	40.31	0.00	0.05	0.02
Y59CA	Y59	HA	CA	4.64	58.29	4.64	58.29	0.00	0.00	0.00
Y59CB	Y59	HB2	CB	3.46	40.01	3.45	39.97	0.01	0.04	0.02
Y59CB	Y59	HB3	CB	2.53	40.01	2.52	39.97	0.01	0.04	0.02
N60CA	N60	HA	CA	4.34	54.16	4.34	54.12	0.00	0.04	0.02
N60CB	N60	HB2	CB	2.79	37.38	2.79	37.35	0.00	0.03	0.01
N60CB	N60	HB3	CB	3.30	37.39	3.30	37.35	0.00	0.04	0.02
I61CA	I61	HA	CA	3.36	62.48	3.36	62.44	0.00	0.04	0.02
I61CB	I61	HB	CB	1.38	36.74	1.38	36.71	0.00	0.03	0.01
I61CD1	I61	MD	CD1	0.40	14.42	0.40	14.39	0.00	0.03	0.01
I61CG1	I61	HG12	CG1	1.08	28.25	1.08	28.19	0.00	0.06	0.02
I61CG1	I61	HG13	CG1	0.38	28.25	0.39	28.18	-0.01	0.07	0.03
I61CG2	I61	MG	CG2	0.47	17.27	0.46	17.22	0.01	0.05	0.02
Q62CA	Q62	HA	CA	4.47	53.60	4.47	53.60	0.00	0.00	0.00
Q62CB	Q62	HB2	CB	2.23	31.66	2.23	31.64	0.00	0.02	0.01
Q62CB	Q62	HB3	CB	1.87	31.66	1.88	31.64	-0.01	0.02	0.01
Q62CG	Q62	HG2	CG	2.35	33.41	2.35	33.41	0.00	0.00	0.00
Q62CG	Q62	HG3	CG	2.30	33.41	2.29	33.41	0.01	0.00	0.01
K63CA	K63	HA	CA	3.96	57.84	3.96	57.85	0.00	-0.01	0.00
K63CB	K63	HB2	CB	1.87	32.59	1.87	32.57	0.00	0.02	0.01
K63CB	K63	HB3	CB	2.03	32.59	2.03	32.59	0.00	0.00	0.00
K63CD	K63	HD2	CD	1.72	29.84	1.72	29.81	0.00	0.03	0.01
K63CE	K63	HE2	CE	3.02	42.08	3.02	42.04	0.00	0.04	0.02
K63CG	K63	HG2	CG	1.47	23.93	1.47	23.90	0.00	0.03	0.01
E64CA	E64	HA	CA	3.18	58.39	3.30	58.36	-0.12	0.03	0.09
E64CB	E64	HB2	CB	2.49	25.97	2.51	25.99	-0.02	-0.02	0.02
E64CB	E64	HB3	CB	2.36	25.97	2.39	25.99	-0.03	-0.02	0.02
E64CG	E64	HG2	CG	2.21	37.41	2.22	37.37	-0.01	0.04	0.02
E64CG	E64	HG3	CG	2.17	37.41	2.22	37.37	-0.05	0.04	0.04
S65CA	S65	HA	CA	4.61	60.89	4.61	60.89	0.00	0.00	0.00
S65CB	S65	HB2	CB	3.89	64.96	3.90	64.96	-0.01	0.00	0.01
S65CB	S65	HB3	CB	3.62	64.96	3.63	64.93	-0.01	0.03	0.01
T66CA	T66	HA	CA	5.24	62.49	5.27	62.48	-0.03	0.01	0.02
T66CB	T66	HB	CB	4.06	70.29	4.05	70.29	0.01	0.00	0.01
T66CG2	T66	MG	CG2	0.93	21.57	0.92	21.53	0.01	0.04	0.02
L67CA	L67	HA	CA	5.05	53.80	5.05	53.80	0.00	0.00	0.00
L67CB	L67	HB2	CB	1.65	44.28	1.63	44.30	0.02	-0.02	0.02
L67CB	L67	HB3	CB	1.59	44.28	1.59	44.28	0.00	0.00	0.00
L67CD1	L67	MD1	CD1	0.69	24.88	0.69	24.88	0.00	0.00	0.00
L67CD2	L67	MD2	CD2	0.66	25.18	0.66	25.18	0.00	0.00	0.00
L67CG	L67	HG	CG	1.76	29.44	1.76	29.44	0.00	0.00	0.00
H68CA	H68	HA	CA	5.15	56.19	5.16	56.18	-0.01	0.01	0.01
H68CB	H68	HB2	CB	3.04	32.45	3.04	32.44	0.00	0.01	0.00
H68CB	H68	HB3	CB	2.87	32.45	2.86	32.44	0.01	0.01	0.01
L69CA	L69	HA	CA	5.16	53.72	5.17	53.68	-0.01	0.04	0.02
L69CB	L69	HB2	CB	1.57	44.25	1.57	44.25	0.00	0.00	0.00

L69CB	L69	HB3	CB	1.09	44.25	1.08	44.26	0.01	-0.01	0.01
L69CD1	L69	MD1	CD1	0.86	23.92	0.85	23.89	0.01	0.03	0.01
L69CD2	L69	MD2	CD2	0.73	26.11	0.72	26.05	0.01	0.06	0.02
L69CG	L69	HG	CG	1.31	27.60	1.31	27.56	0.00	0.04	0.02
V70CA	V70	HA	CA	4.34	60.71	4.33	60.69	0.01	0.02	0.01
V70CB	V70	HB	CB	2.01	34.78	2.00	34.70	0.01	0.08	0.03
V70CG1	V70	MG1	CG1	0.93	21.37	0.92	21.32	0.01	0.05	0.02
V70CG2	V70	MG2	CG2	0.84	20.70	0.84	20.70	0.00	0.00	0.00
L71CA	L71	HA	CA	5.02	53.97	5.02	53.97	0.00	0.00	0.00
L71CB	L71	HB2	CB	1.53	42.77	1.52	42.71	0.01	0.06	0.02
L71CB	L71	HB3	CB	1.68	42.77	1.67	42.71	0.01	0.06	0.02
L71CD1	L71	MD1	CD1	0.96	25.04	0.96	25.01	0.00	0.03	0.01
L71CD2	L71	MD2	CD2	0.86	23.92	0.86	23.92	0.00	0.00	0.00
L71CG	L71	HG	CG	1.66	27.57	1.66	27.57	0.00	0.00	0.00
R72CA	R72	HA	CA	4.26	55.65	4.27	55.60	-0.01	0.05	0.02
R72CB	R72	HB2	CB	1.53	31.32	1.52	31.31	0.01	0.01	0.01
R72CB	R72	HB3	CB	1.76	31.31	1.75	31.26	0.01	0.05	0.02
R72CD	R72	HD2	CD	3.14	43.46	3.13	43.39	0.01	0.07	0.03
R72CG	R72	HG2	CG	1.53	27.22	1.53	27.22	0.00	0.00	0.00
L73CA	L73	HA	CA	4.39	54.84	4.39	54.79	0.00	0.05	0.02
L73CB	L73	HB2	CB	1.56	42.46	1.55	42.42	0.01	0.04	0.02
L73CB	L73	HB3	CB	1.64	42.46	1.63	42.45	0.01	0.01	0.01
L73CD1	L73	MD1	CD1	0.92	24.97	0.92	24.95	0.00	0.02	0.01
L73CD2	L73	MD2	CD2	0.87	23.33	0.87	23.32	0.00	0.01	0.00
L73CG	L73	HG	CG	1.62	27.09	1.62	27.09	0.00	0.00	0.00
R74CA	R74	HA	CA	4.29	56.54	4.29	56.54	0.00	0.00	0.00
R74CB	R74	HB2	CB	1.79	30.66	1.79	30.63	0.00	0.03	0.01
R74CB	R74	HB3	CB	1.86	30.66	1.86	30.61	0.00	0.05	0.02
R74CD	R74	HD2	CD	3.20	43.34	3.20	43.31	0.00	0.03	0.01
R74CG	R74	HG2	CG	1.60	27.08	1.60	27.08	0.00	0.00	0.00
R74CG	R74	HG3	CG	1.67	27.07	1.67	27.07	0.00	0.00	0.00
G75CA	G75	HA2	CA	3.97	45.24	3.97	45.24	0.00	0.00	0.00
G75CA	G75	HA3	CA	3.94	45.25	3.94	45.25	0.00	0.00	0.00
G76CA	G76	HA2	CA	3.73	46.05	3.72	46.02	0.01	0.03	0.01
G76CA	G76	HA3	CA	3.81	46.05	3.81	46.02	0.00	0.03	0.01

‡: assignment for NMR analysis: The first letter is the one letter code for the amino acid, the number describes the position of the amino acid in the primary sequence, the penultimate letter C indicates that a carbon atom is considered, and the last letter indicates the relative position of that carbon atom in respect to the amino and carboxyl group (A = α, B = β, G = γ, D = δ, E = ε)[39].

*: scaled perturbation corresponding to $\sqrt{\frac{(0.30 \times \Delta\delta(^{13}\mathrm{C})^2 + \Delta\delta(^{1}\mathrm{H})^2}{2}}$ as described before[40]

**: linear color scale from 0.00 ppm (RGB[255,255,255]) to 3.12 ppm (RGB[255,105,105])

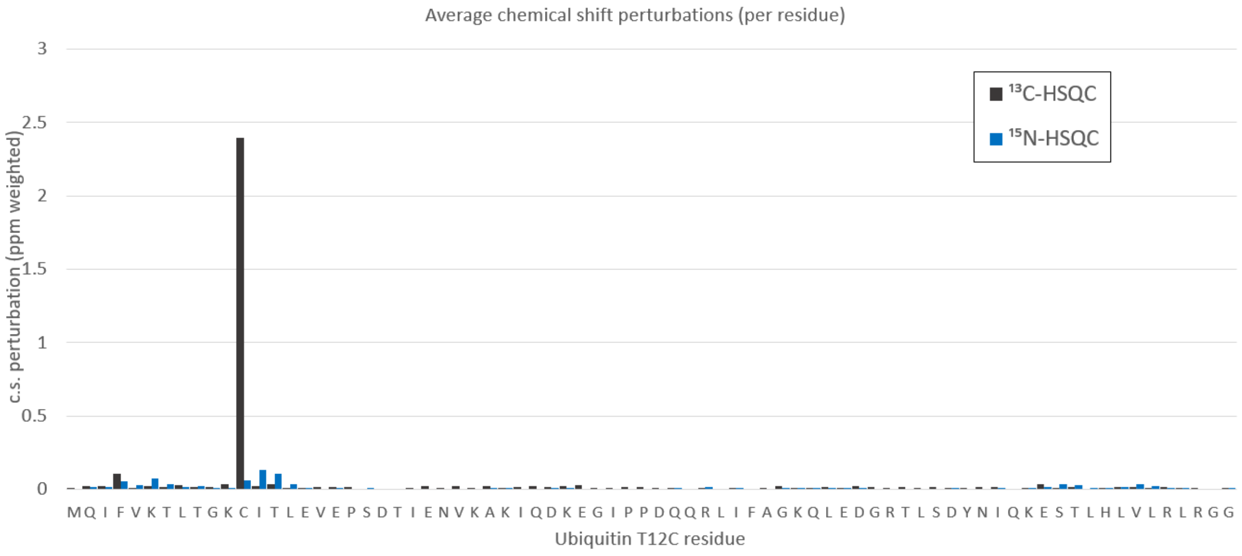

Figure S225 Average chemical shift perturbations per residue of ^{15}N,^{13}C-Ubiquitin T12C after the reaction with $^{13}C_2$-**VTT** and sodium azide. This graph is based on the values reported in Table S37 and **Table S**38.

NOESY-correlation between (Ubiquitin T12C)–S–C_2H_4–N_3 and Ubiquitin T12C

NOESY spectra were collected at 298 K on unlabeled 0.5 mM T12C-Ubiquitin in a 3 mm NMR tube with and without **tag,** using the standard Bruker pulse program noesyesgpph. To maximize the heights of short and long-range cross-peaks, mixing time was set to 200 ms.

The experimental datasets were processed with nmrPipe and converted to Sparky format for peak assignment and further analysis. root-mean square (RMS) noise was calculated in nmrPipe. The raw peak heights were obtained from nmrfam-Sparky.

Only the ratio between cross-peaks measured with the same mixing time of the NOESY is relevant for the distances (i.e. conservation of structure). 82 unambiguous cross peaks without overlaps, arising from nuclei belonging to residues within 10 Å of T12C-C_α, were selected for analysis. To correct for any systematic differences between the two separate samples and experiments, 6 well isolated diagonal peaks were identified. The ratio of the peak heights between the diagonal peaks of tagged-Ubiquitin and untagged-Ubiquitin was calculated and averaged to 0.93. The value was afterwards used as a scaling factor **(s)** for the untagged protein cross-peak heights to calculate scaled peak heights (I). For the tagged protein this factor *s* was set to 1. All raw peak heights were further scaled with a factor of 10^{10}:

$$I = \frac{Peak\ Height}{10^{10}} \times s$$

RMS noise (σ_{rms}) was also scaled accordingly and used as uncertainties for the scaled peak heights. The correlation plot of the scaled peak heights is shown in **Fig. S218.**

Noesy cross-peak heights (I) scale with internuclei distances (r) as $1/_{r^6}$, the quantity $I^{-\frac{1}{6}}$ was therefore calculated. Errors ($\sigma(I^{-\frac{1}{6}})$) were propagated accordingly and the correlation plot of $I^{-\frac{1}{6}}$ between the tagged and untagged ubiquitin is shown in **Fig. S219.**

$$\sigma(I^{-\frac{1}{6}}) = \left| \frac{I^{-\frac{1}{6}}}{I} \times \left(-\frac{1}{6}\right) \times \left(\frac{\sigma_{rms}}{10^{10}} \times s\right) \right|$$

First, the relative change in peak heights (ΔI) arising from tagging was calculated:

$$\Delta I = \left|\frac{I_{untagged} - I_{tagged}}{I_{untagged}}\right|$$

Contribution from noise ($\sigma_{\Delta I}$) in the relative change in peak heights was also calculated from SNR of individual peaks:

$$\sigma_{\Delta I} = \sqrt{\left(1/SNR_{tagged}\right)^2 + \left(s/SNR_{untagged}\right)^2}$$

Relative changes in internuclei distances (Δr) that arise from tagging were calculated from relative changes in peak heights after compensating for the noise contribution.

$$\Delta r = \left|1 - \frac{1}{\sqrt[6]{1 + (\Delta I - \sigma_{\Delta I})}}\right|$$

Table S39. NOESY measurements of Human untagged unlabeled Ubiquitin T12C.

Untagged Ubq-T12C

RMS Noise **9.90E+07**

Diagonal Peaks for Intensity Scaling

Resonance‡	δ_{1H} (ppm)	δ_{1H} (ppm)	Peak Height (a.u.)	$SNR_{untagged}$
V5H-5H	9.283	9.284	5.82E+10	588
I13H-I13H	9.56	9.561	4.96E+10	501
E64H-E64H	9.348	9.349	4.70E+10	474
L67H-L67H	9.443	9.443	4.27E+10	432
H68H-H68H	9.244	9.245	5.28E+10	533
V70H-V70H	9.214	9.215	5.37E+10	542

	Untagged Ubq-T12C Cross Peaks						
Resonance‡	δ_{1H} (ppm)	δ_{1H} (ppm)	Peak Height (a.u.)	$SNR_{untagged}$	$I_{untagged}$ (a.u.)	$(I_{untagged})^{-1/6}$ (a.u.)	$\sigma((I_{tagged})^{-1/6})$
E64HA-F4HD1	3.304	7.04	3.82E+09	39	0.354	1.189	0.005
G10HA2-G10H	3.577	7.878	3.36E+09	34	0.311	1.215	0.006
I13HD1-I13H	0.72	9.56	3.96E+09	40	0.366	1.182	0.005
T14HG2-L15H	1.121	8.767	1.26E+10	127	1.164	0.975	0.001
V5HG1-V5H	0.68	9.284	4.89E+09	49	0.453	1.141	0.004
T66HA-F4HD1	5.267	7.04	3.93E+09	40	0.364	1.183	0.005
H68HA-K6H	5.161	8.997	5.48E+09	55	0.507	1.120	0.003
G10HA3-G10H	4.404	7.878	2.01E+09	20	0.186	1.324	0.011
L69HB2-L67H	1.6	9.443	3.85E+09	39	0.357	1.187	0.005
K11HB2-K11H	1.677	7.259	4.64E+09	47	0.430	1.151	0.004
T7HA-V5H	4.944	9.284	2.99E+09	30	0.277	1.239	0.007
T66HG2-F4HD1	0.922	7.04	3.53E+09	36	0.327	1.205	0.006

I3HA-S65H	4.141	7.684	3.67E+09	37	0.340	1.197	0.005
E64HA-E64H	3.304	9.348	1.39E+10	140	1.285	0.959	0.001
F4HA-F4HE1	5.607	7.215	3.01E+09	30	0.279	1.237	0.007
S65HA-S65H	4.617	7.683	7.58E+09	77	0.702	1.061	0.002
K11HB3-K11H	1.782	7.259	4.17E+09	42	0.386	1.172	0.005
T66HG2-K6H	0.922	8.995	4.43E+09	45	0.410	1.160	0.004
K6HB2-K6H	1.398	8.995	5.83E+09	59	0.540	1.108	0.003
I3H-L15H	8.327	8.768	5.97E+09	60	0.553	1.104	0.003
V5HG2-V5H	0.725	9.284	9.95E+09	101	0.921	1.014	0.002
K6HB3-K6H	1.708	8.995	3.58E+09	36	0.332	1.202	0.006
L15HB2-L15H	1.204	8.767	3.26E+09	33	0.302	1.221	0.006
F4HA-F4H	5.607	8.601	2.64E+09	27	0.244	1.265	0.008
F4HA-L15H	5.607	8.767	2.36E+09	24	0.219	1.288	0.009
T14HG2-F4HD1	1.12	7.04	1.31E+10	132	1.210	0.969	0.001
L15HA-L15H	4.745	8.767	5.08E+09	51	0.470	1.134	0.004
V5HG2-K6H	0.726	8.995	3.71E+09	37	0.343	1.195	0.005
K6HA-K6H	5.355	8.996	3.34E+09	34	0.309	1.216	0.006
V5H-I13H	9.284	9.561	4.69E+09	47	0.434	1.149	0.004
K6HA-I13H	5.353	9.56	3.35E+09	34	0.310	1.216	0.006
L8HB3-L8H	1.923	9.158	1.53E+09	15	0.142	1.385	0.015
V5HG1-K6H	0.679	8.995	8.47E+09	86	0.784	1.041	0.002
E64HB3-F4HE1	2.387	7.218	6.14E+09	62	0.569	1.099	0.003
E64HA-S65H	3.304	7.686	4.48E+09	45	0.415	1.158	0.004
G10HA2-G10H	3.555	7.879	3.58E+09	36	0.331	1.202	0.006
F4HA-V5H	5.606	9.284	1.63E+10	165	1.513	0.933	0.001
V5HA-L67H	4.813	9.443	4.64E+09	47	0.430	1.151	0.004
K11HG3-K11H	1.377	7.256	1.20E+10	121	1.113	0.982	0.001
T7HA-T7H	4.96	8.65	5.35E+09	54	0.495	1.124	0.003
I13HA-T14H	4.479	8.742	2.47E+10	250	2.288	0.871	0.001
L67HD1-L67H	0.727	9.444	2.94E+09	30	0.272	1.242	0.007
K11HA-K11H	4.339	7.26	1.08E+10	109	0.998	1.000	0.002
K6HA-T7H	5.353	8.651	2.53E+10	255	2.341	0.868	0.001
S65HB2-S65H	3.897	7.685	5.55E+09	56	0.513	1.118	0.003
S65H-E64H	7.685	9.348	5.94E+09	60	0.550	1.105	0.003
L69H-K6H	8.303	8.994	1.88E+09	19	0.174	1.338	0.012
I13HG2-I13H	0.872	9.56	4.05E+09	41	0.375	1.178	0.005
F4HA-F4HD1	5.606	7.04	1.19E+10	120	1.101	0.984	0.001
K6H-L67H	8.995	9.444	1.96E+09	20	0.181	1.329	0.011
T7HG2-T7H	1.178	8.651	1.58E+10	160	1.464	0.938	0.001
K63HE-F4HD1	3.008	7.04	1.13E+10	114	1.043	0.993	0.001
I13HB-I13H	1.896	9.561	7.03E+09	71	0.651	1.074	0.003
L67HD2-L67H	0.682	9.443	6.31E+09	64	0.584	1.094	0.003
L67HB-L67H	1.637	9.444	4.44E+09	45	0.411	1.160	0.004
V5HA-V5H	4.812	9.284	3.52E+09	36	0.326	1.205	0.006
I13HG12-I13H	1.099	9.56	3.41E+09	34	0.316	1.212	0.006
H68HB3-H68H	2.855	9.245	5.63E+09	57	0.521	1.115	0.003
S65HB3-S65H	3.629	7.686	8.56E+09	86	0.792	1.040	0.002

T14HA-F4HD1	4.945	7.04	9.84E+09	99	0.911	1.016	0.002
T66HA-L67H	5.267	9.443	1.83E+10	185	1.692	0.916	0.001
V5HB-V5H	1.903	9.284	9.06E+09	91	0.839	1.030	0.002
K63HA-S65H	3.963	7.686	4.55E+09	46	0.421	1.155	0.004
L67HA-H68H	5.052	9.244	1.47E+10	149	1.362	0.950	0.001
L15HB3-L15H	1.346	8.768	5.30E+09	54	0.491	1.126	0.004
L67HA-L67H	5.052	9.443	3.40E+09	34	0.315	1.212	0.006
T66HG2-L67H	0.921	9.444	9.20E+09	93	0.852	1.027	0.002
T14HA-F4HE1	4.945	7.216	3.17E+09	32	0.293	1.227	0.006
L8HG-T9H	1.875	7.637	6.28E+09	63	0.582	1.095	0.003
K33HA-E34H	4.301	8.739	3.21E+09	32	0.297	1.224	0.006
L69HA-I13H	5.24	9.561	1.92E+10	194	1.777	0.909	0.001
T9HA-T9H	4.469	7.636	5.02E+09	51	0.465	1.136	0.004
L67HG-H68H	1.748	9.245	4.03E+09	41	0.373	1.179	0.005
I13HG13-I13H	1.458	9.561	4.31E+09	44	0.399	1.166	0.004
T7HA-L8H	4.96	9.158	2.50E+09	25	0.232	1.276	0.008
I3HA-E64H	4.141	9.348	4.92E+09	50	0.456	1.140	0.004
L8HD1-L8H	1.041	9.161	3.34E+09	34	0.310	1.216	0.006
L56HD2-S65H	0.612	7.686	9.65E+09	98	0.894	1.019	0.002
E34HB2-E34H	1.675	8.74	6.94E+09	70	0.642	1.077	0.003
L67HG-L67H	1.75	9.443	2.91E+09	29	0.269	1.245	0.007
V5HA-K6H	4.811	8.995	1.51E+10	152	1.395	0.946	0.001
I13HA-I13H	4.48	9.56	4.58E+09	46	0.424	1.154	0.004

‡: assignment for NMR analysis: The first letter is the one letter code for the amino acid, the number describes the position of the amino acid in the primary sequence, the letter H indicates that a hydrogen atom is considered, and the last letter (in combination with a number) indicates the relative position of that hydrogen atom in respect to the amino and carboxyl group (A = α, B = β, G = γ, D = δ, E = ε)[39].

Table S40. NOESY measurements of Human unlabeled Ubiquitin T12C tagged with the ethyl-azide-linker.

Tagged Ubq-T12C

RMS Noise **1.02E+08**

Resonance‡	δ_{1H} (ppm)	δ_{1H} (ppm)	Peak Height (a.u.)	SNR_{tagged}	$I_{tagged}/I_{untagged}$
V5H-5H	9.319	9.321	5.41E+10	530	0.930
I13H-I13H	9.534	9.536	4.68E+10	459	0.944
E64H-E64H	9.352	9.354	4.39E+10	431	0.935
L67H-L67H	9.461	9.463	3.88E+10	380	0.907
H68H-H68H	9.238	9.241	4.77E+10	467	0.903
V70H-V70H	9.207	9.209	5.03E+10	493	0.937
				**Average**	0.926

	Tagged Ubq-T12C Cross Peaks									
Resonance‡	δ_{1H} (ppm)	δ_{1H} (ppm)	Peak Height (a.u.)	SNR_{tagged}	I_{tagged} (a.u.)	$(I_{tagged})^{-1/6}$ (a.u.)	$\sigma((I_{tagged})^{-1/6})$	ΔI	$\sigma_{\Delta I}$	Δr
E64HA-F4HD1	3.17	7.087	2.75E+09	27	0.275	1.240	0.007	0.223	0.044	0.027
G10HA2-G10H	3.571	7.877	3.80E+09	37	0.380	1.175	0.005	0.222	0.038	0.028
I13HD1-I13H	0.704	9.535	2.93E+09	29	0.293	1.227	0.007	0.199	0.042	0.024
T14HG2-L15H	1.168	8.819	9.57E+09	94	0.957	1.007	0.002	0.178	0.013	0.025
V5HG1-V5H	0.67	9.321	3.75E+09	37	0.375	1.177	0.005	0.171	0.033	0.021
T66HA-F4HD1	5.227	7.084	3.02E+09	30	0.302	1.221	0.006	0.170	0.041	0.020
H68HA-K6H	5.147	8.943	4.28E+09	42	0.428	1.152	0.004	0.157	0.029	0.020
G10HA3-G10H	4.402	7.876	2.15E+09	21	0.215	1.292	0.009	0.154	0.066	0.014
L69HB2-L67H	1.581	9.463	3.02E+09	30	0.302	1.221	0.006	0.153	0.041	0.018
K11HB2-K11H	1.663	7.248	4.95E+09	49	0.495	1.124	0.004	0.152	0.029	0.019
T7HA-V5H	4.994	9.32	2.37E+09	23	0.237	1.271	0.008	0.144	0.053	0.014

T66HG2-F4HD1	0.919	7.084	2.83E+09	28	0.283	1.234	0.007	0.134	0.044	0.014
I3HA-S65H	4.095	7.652	2.96E+09	29	0.296	1.225	0.007	0.128	0.043	0.014
E64HA-E64H	3.172	9.353	1.12E+10	110	1.123	0.981	0.001	0.126	0.011	0.018
F4HA-F4HE1	5.728	7.215	3.12E+09	31	0.312	1.214	0.006	0.121	0.045	0.012
S65HA-S65H	4.604	7.652	6.18E+09	61	0.618	1.084	0.003	0.120	0.020	0.016
K11HB3-K11H	1.783	7.248	4.32E+09	42	0.432	1.150	0.004	0.119	0.032	0.014
T66HG2-K6H	0.919	8.943	3.61E+09	35	0.361	1.185	0.005	0.119	0.035	0.013
K6HB2-K6H	1.387	8.943	6.04E+09	59	0.604	1.088	0.003	0.119	0.023	0.015
I3H-L15H	8.347	8.818	4.87E+09	48	0.487	1.127	0.004	0.119	0.026	0.015
V5HG2-V5H	0.726	9.321	8.14E+09	80	0.814	1.035	0.002	0.116	0.016	0.016
K6HB3-K6H	1.693	8.944	3.69E+09	36	0.369	1.181	0.005	0.113	0.038	0.012
L15HB2-L15H	1.203	8.818	2.68E+09	26	0.268	1.246	0.007	0.112	0.047	0.010
F4HA-F4H	5.727	8.587	2.18E+09	21	0.218	1.289	0.009	0.107	0.058	0.008
F4HA-L15H	5.728	8.819	1.95E+09	19	0.195	1.313	0.011	0.107	0.065	0.007
T14HG2-F4HD1	1.167	7.086	1.08E+10	106	1.081	0.987	0.001	0.106	0.012	0.015
L15HA-L15H	4.754	8.818	4.23E+09	41	0.423	1.154	0.004	0.102	0.030	0.011
V5HG2-K6H	0.725	8.946	3.09E+09	30	0.309	1.216	0.006	0.101	0.041	0.010
K6HA-K6H	5.359	8.945	2.78E+09	27	0.278	1.238	0.007	0.099	0.046	0.009
V5H-I13H	9.32	9.536	4.77E+09	47	0.477	1.131	0.004	0.099	0.029	0.011
K6HA-I13H	5.359	9.536	2.81E+09	28	0.281	1.236	0.007	0.094	0.045	0.008
L8HB3-L8H	1.918	9.157	1.54E+09	15	0.154	1.366	0.014	0.086	0.089	0.001
V5HG1-K6H	0.671	8.942	7.17E+09	70	0.717	1.057	0.002	0.085	0.018	0.011
E64HB3-F4HE1	2.356	7.216	5.22E+09	51	0.522	1.114	0.003	0.082	0.025	0.009
E64HA-S65H	3.172	7.651	3.82E+09	37	0.382	1.174	0.005	0.079	0.034	0.007
G10HA2-G10H	3.55	7.877	3.57E+09	35	0.357	1.187	0.005	0.078	0.038	0.006
F4HA-V5H	5.728	9.321	1.40E+10	137	1.397	0.946	0.001	0.077	0.009	0.011
V5HA-L67H	4.824	9.462	4.61E+09	45	0.461	1.138	0.004	0.073	0.030	0.007
K11HG3-K11H	1.372	7.253	1.19E+10	116	1.188	0.972	0.001	0.068	0.011	0.009
T7HA-T7H	4.946	8.675	4.62E+09	45	0.462	1.137	0.004	0.067	0.028	0.006
I13HA-T14H	4.469	8.657	2.13E+10	209	2.134	0.881	0.001	0.067	0.006	0.010

L67HD1-L67H	0.725	9.463	2.90E+09	28	0.290	1.229	0.007	0.066	0.047	0.003
K11HA-K11H	4.335	7.254	1.06E+10	104	1.064	0.990	0.001	0.066	0.013	0.009
K6HA-T7H	5.359	8.676	2.19E+10	215	2.191	0.877	0.001	0.064	0.006	0.009
S65HB2-S65H	3.886	7.65	5.46E+09	54	0.546	1.106	0.003	0.064	0.025	0.006
S65H-E64H	7.649	9.354	5.85E+09	57	0.585	1.094	0.003	0.063	0.023	0.006
L69H-K6H	8.285	8.942	1.85E+09	18	0.185	1.325	0.011	0.061	0.074	0.002
I13HG2-I13H	0.863	9.536	3.53E+09	35	0.353	1.189	0.005	0.057	0.037	0.003
F4HA-F4HD1	5.728	7.085	1.04E+10	102	1.039	0.994	0.002	0.057	0.012	0.007
K6H-L67H	8.942	9.464	1.71E+09	17	0.171	1.342	0.012	0.056	0.076	0.003
T7HG2-T7H	1.161	8.674	1.55E+10	152	1.546	0.930	0.001	0.056	0.009	0.008
K63HE-F4HD1	3.119	7.086	9.86E+09	97	0.986	1.002	0.002	0.055	0.013	0.007
I13HB-I13H	1.875	9.536	6.87E+09	67	0.687	1.065	0.002	0.055	0.020	0.006
L67HD2-L67H	0.677	9.462	5.52E+09	54	0.552	1.104	0.003	0.054	0.024	0.005
L67HB-L67H	1.644	9.462	4.32E+09	42	0.432	1.150	0.004	0.051	0.031	0.003
V5HA-V5H	4.825	9.321	3.11E+09	30	0.311	1.215	0.006	0.047	0.042	0.001
I13HG12-I13H	1.082	9.535	3.29E+09	32	0.329	1.204	0.006	0.042	0.041	0.000
H68HB3-H68H	2.858	9.24	5.43E+09	53	0.543	1.107	0.003	0.041	0.025	0.003
S65HB3-S65H	3.612	7.65	8.22E+09	81	0.822	1.033	0.002	0.038	0.016	0.004
T14HA-F4HD1	4.994	7.085	8.79E+09	86	0.879	1.022	0.002	0.035	0.015	0.003
T66HA-L67H	5.228	9.463	1.63E+10	160	1.634	0.921	0.001	0.034	0.008	0.004
V5HB-V5H	1.877	9.321	8.67E+09	85	0.867	1.024	0.002	0.034	0.016	0.003
K63HA-S65H	3.958	7.651	4.07E+09	40	0.407	1.162	0.004	0.033	0.032	0.000
L67HA-H68H	5.048	9.24	1.41E+10	138	1.407	0.945	0.001	0.033	0.010	0.004
L15HB3-L15H	1.362	8.818	5.06E+09	50	0.506	1.120	0.003	0.031	0.027	0.001
L67HA-L67H	5.049	9.463	3.24E+09	32	0.324	1.207	0.006	0.029	0.041	0.002
T66HG2-L67H	0.919	9.463	8.29E+09	81	0.829	1.032	0.002	0.027	0.016	0.002
T14HA-F4HE1	4.994	7.216	2.86E+09	28	0.286	1.232	0.007	0.025	0.046	0.004
L8HG-T9H	1.868	7.636	5.67E+09	56	0.567	1.099	0.003	0.025	0.023	0.000
K33HA-E34H	4.3	8.741	3.03E+09	30	0.303	1.220	0.006	0.021	0.044	0.004
L69HA-I13H	5.204	9.536	1.74E+10	171	1.741	0.912	0.001	0.020	0.008	0.002

T9HA-T9H	4.463	7.634	4.74E+09	46	0.474	1.132	0.004	0.020	0.028	0.001
L67HG-H68H	1.744	9.24	3.68E+09	36	0.368	1.181	0.005	0.014	0.036	0.004
I13HG13-I13H	1.445	9.536	4.04E+09	40	0.404	1.163	0.005	0.014	0.033	0.003
T7HA-L8H	4.946	9.156	2.35E+09	23	0.235	1.273	0.009	0.013	0.057	0.007
I3HA-E64H	4.095	9.353	4.50E+09	44	0.450	1.142	0.004	0.013	0.029	0.003
L8HD1-L8H	1.04	9.152	3.13E+09	31	0.313	1.213	0.006	0.013	0.043	0.005
L56HD2-S65H	0.608	7.651	8.84E+09	87	0.884	1.021	0.002	0.011	0.015	0.001
E34HB2-E34H	1.669	8.739	6.38E+09	63	0.638	1.078	0.003	0.006	0.021	0.002
L67HG-L67H	1.746	9.463	2.68E+09	26	0.268	1.245	0.007	0.003	0.049	0.008
V5HA-K6H	4.825	8.944	1.40E+10	137	1.399	0.946	0.001	0.003	0.009	0.001
I13HA-I13H	4.469	9.535	4.23E+09	41	0.423	1.154	0.004	0.002	0.031	0.005

‡: assignment for NMR analysis: The first letter is the one letter code for the amino acid, the number describes the position of the amino acid in the primary sequence, the letter H indicates that a hydrogen atom is considered, and the last letter (in combination with a number) indicates the relative position of that hydrogen atom in respect to the amino and carboxyl group (A = α, B = β, G = γ, D = δ, E = ε)[39].

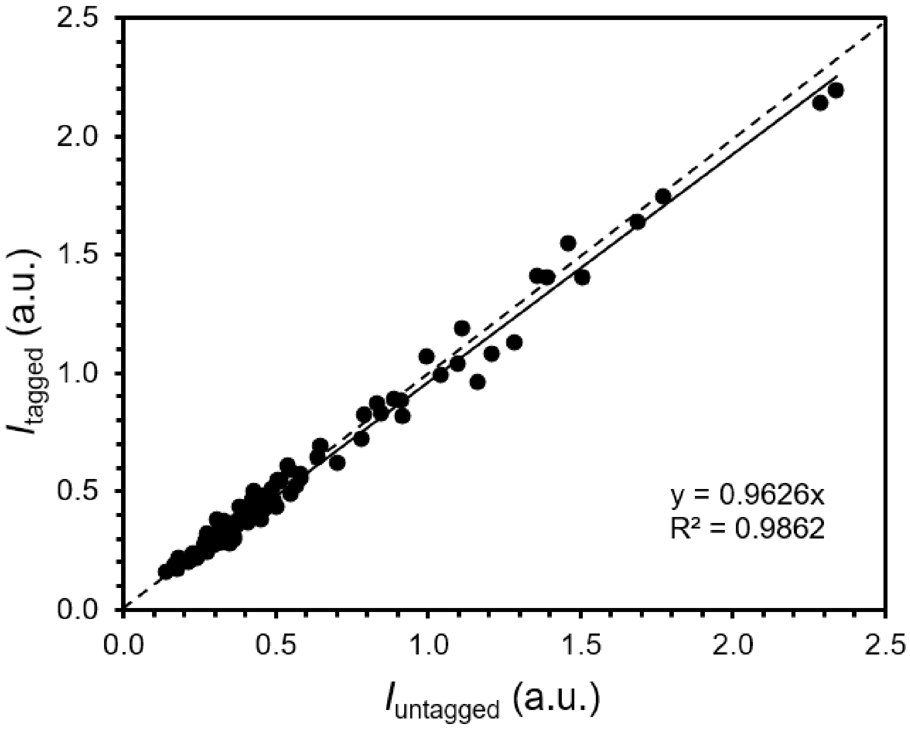

Figure S226. Correlation plot for NOESY shifts before and after functionalization of Ubiquitin T12C.

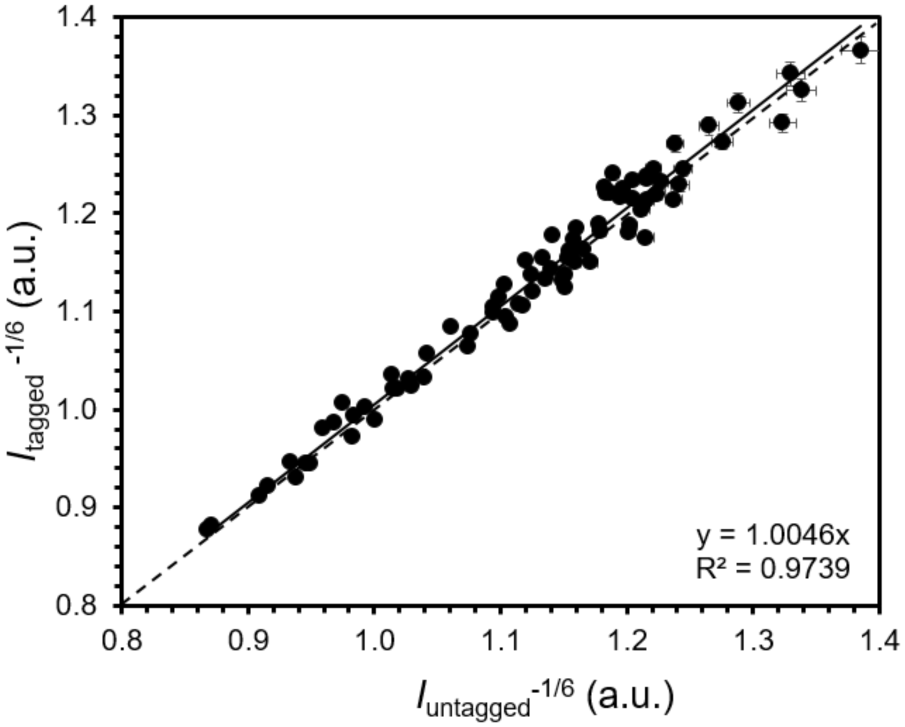

Figure S227. Scaled Correlation plot for NOESY shifts before and after functionalization of Ubiquitin T12C.

^{19}F NMR of (^{15}N,^{13}C-Ubiquitin T12C)–S–$^{13}C_2H_4$–(4-F-aniline)

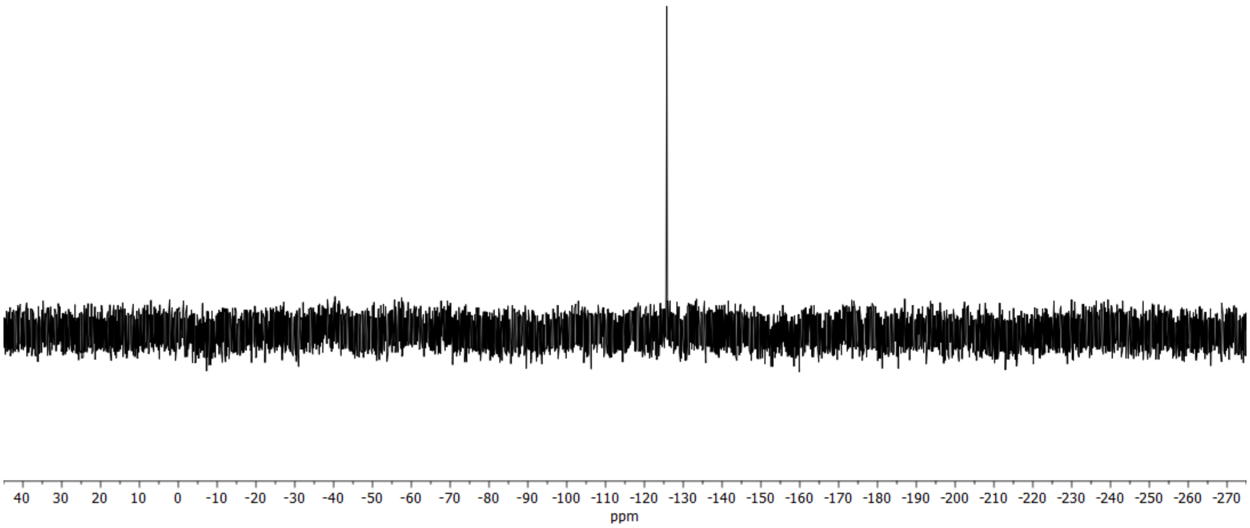

Figure S228. ^{19}F NMR of (^{15}N,^{13}C-Ubiquitin T12C)–S–$^{13}C_2H_4$–(4-F-aniline)

Miscellaneous NMR Data

^{1}H NMR of Thiophenol-substituted N-methylmaleimide **(S8):**
500 MHz, $CDCl_3$, 298 K

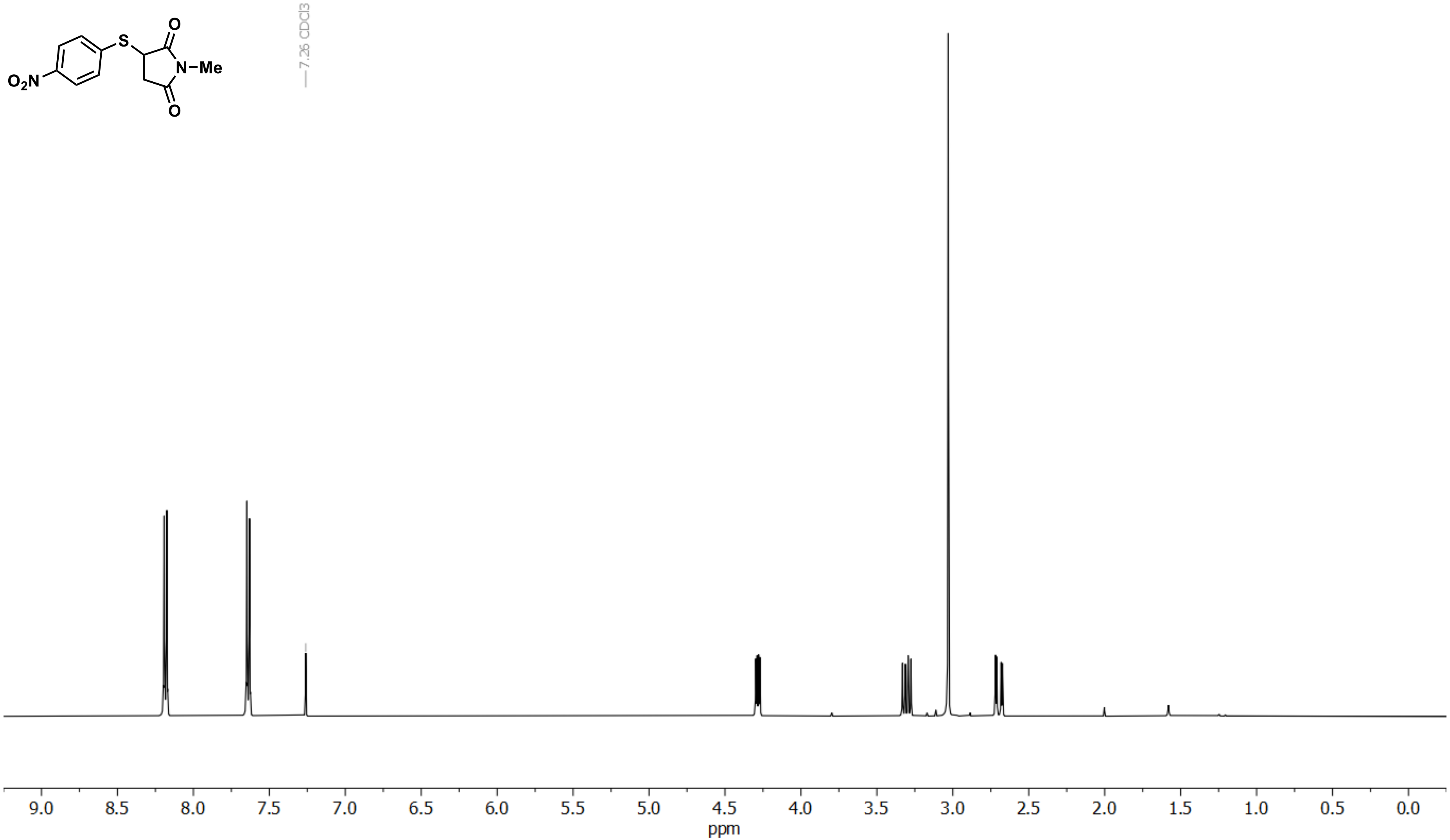

^{13}C NMR of Thiophenol-substituted N-methylmaleimide **(S8):**
126 MHz, $CDCl_3$, 298 K

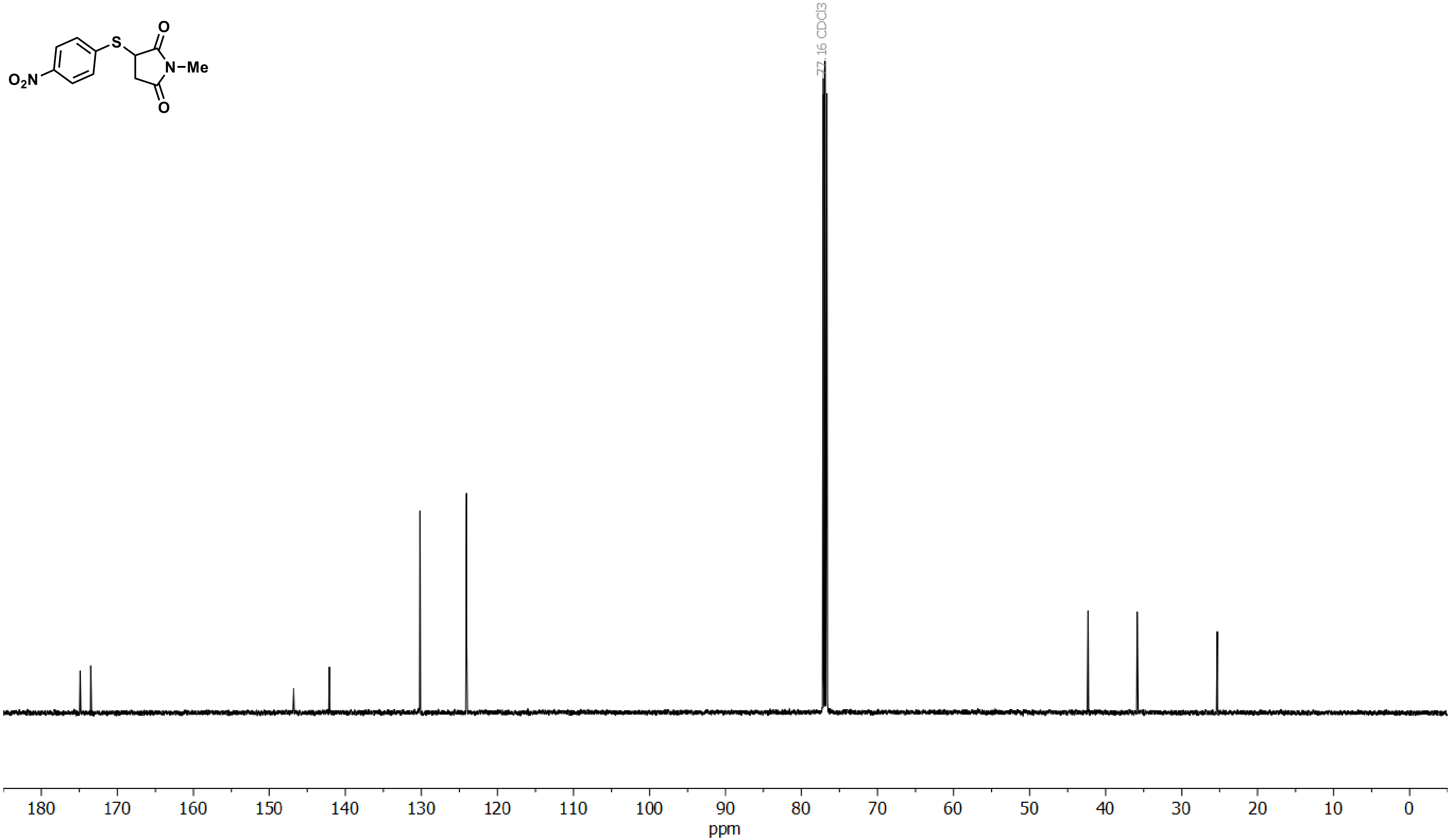

^{1}H NMR of thiophenol-derived azide **(S9):**
500 MHz, DMSO-d_6, 298 K

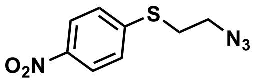

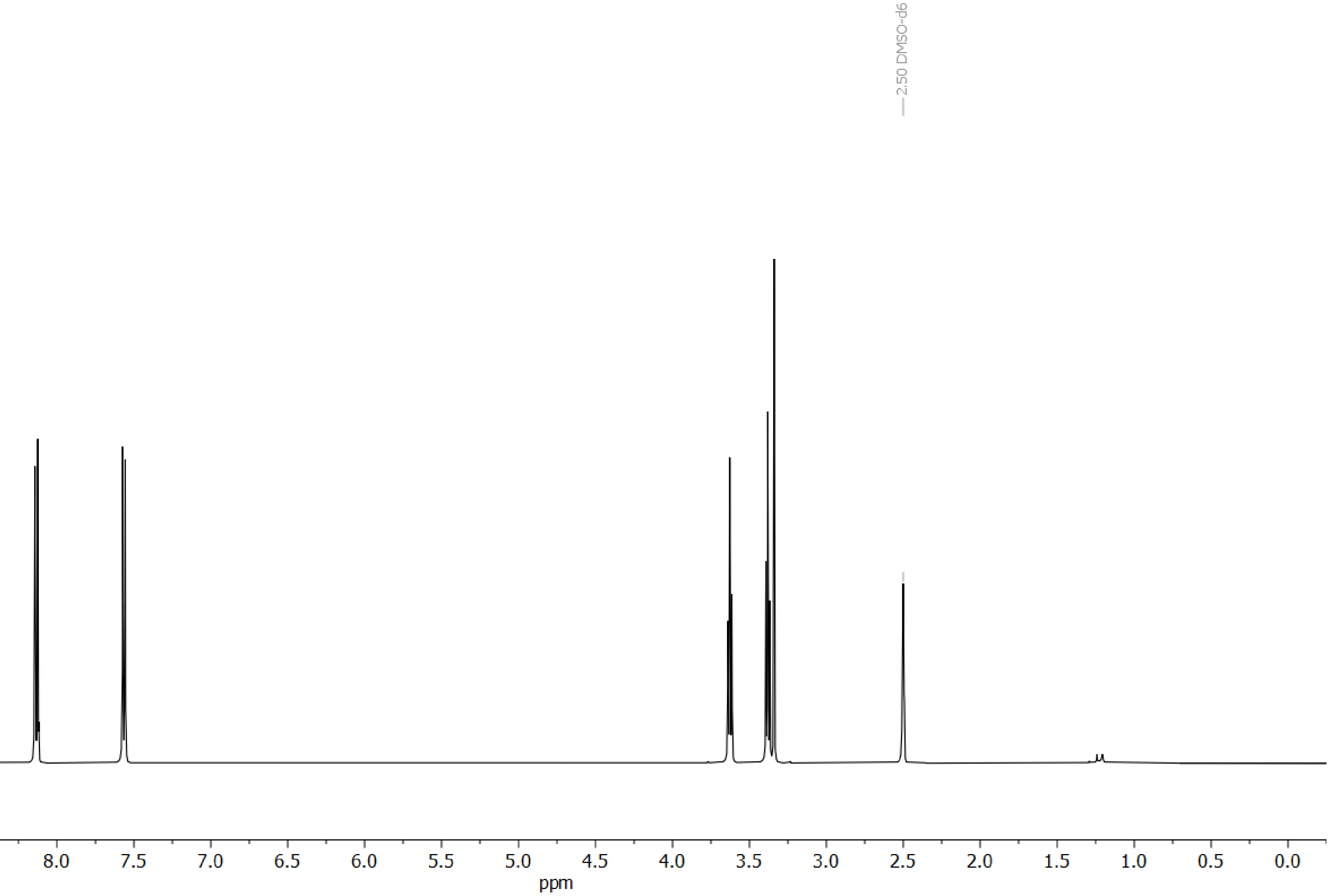

^{13}C NMR of thiophenol-derived azide **(S9):**
126 MHz, DMSO-d$_6$, 298 K

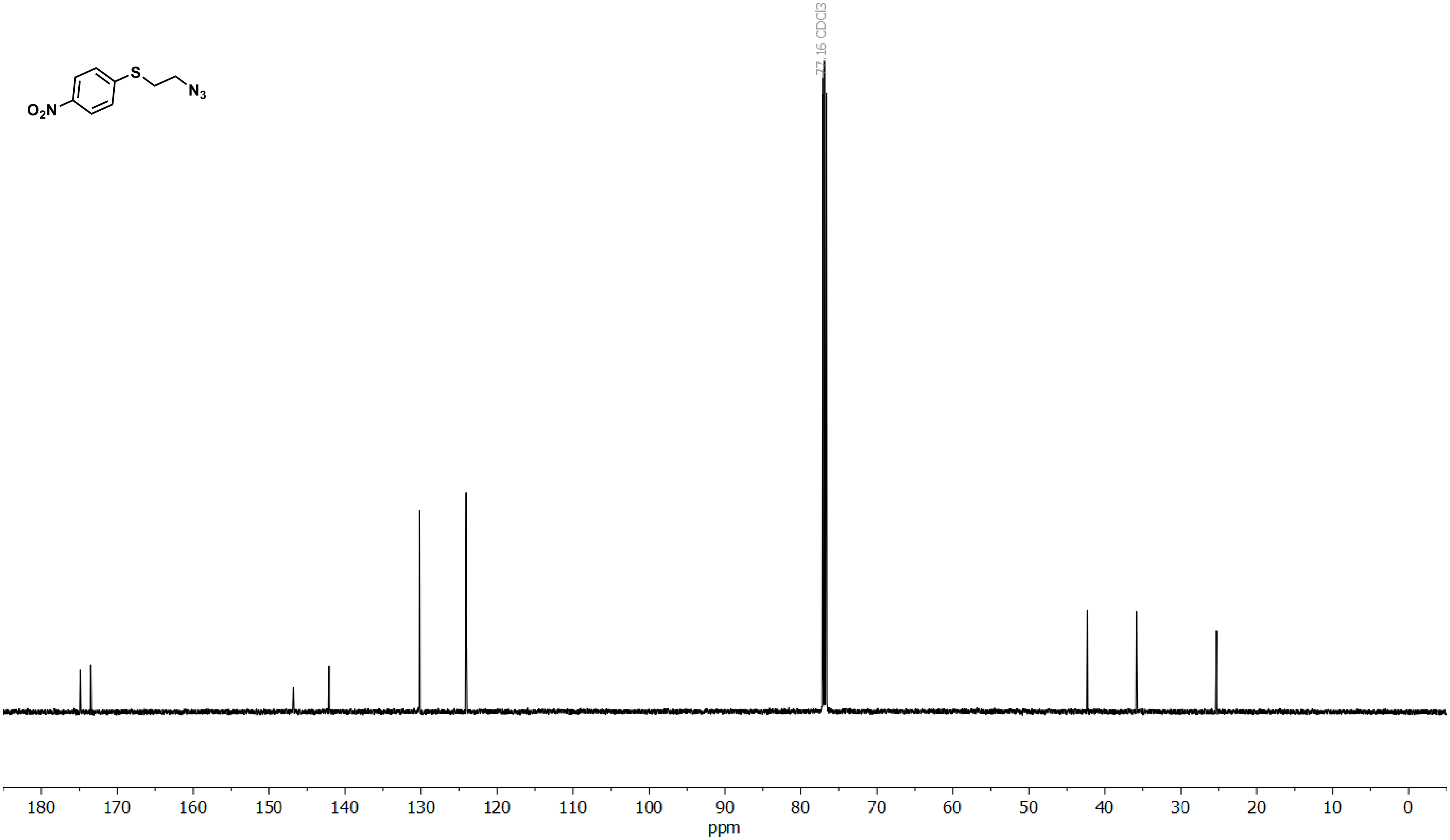

MS/MS DATA

MS/MS-Characterization of stapled peptides

Stapled Ac-Ala-Cys-Leu-Leu-Gln-Glu-Phe-Ala-Pro-Pro-Trp-Ile-NH_2 (22)

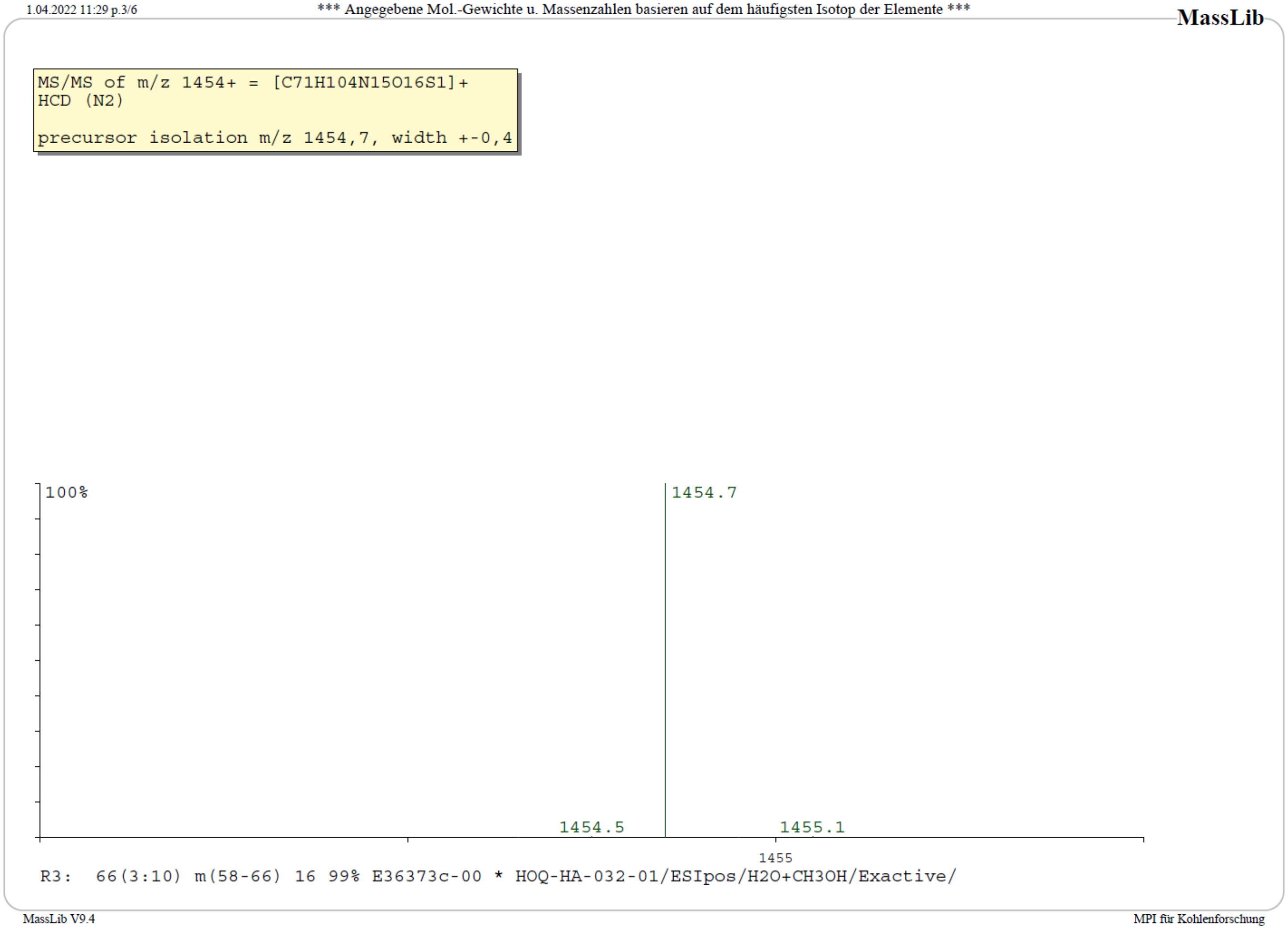

1.04.2022 11:29 p.4/6 *** Angegebene Mol.-Gewichte u. Massenzahlen basieren auf dem häufigsten Isotop der Elemente *** MassLib

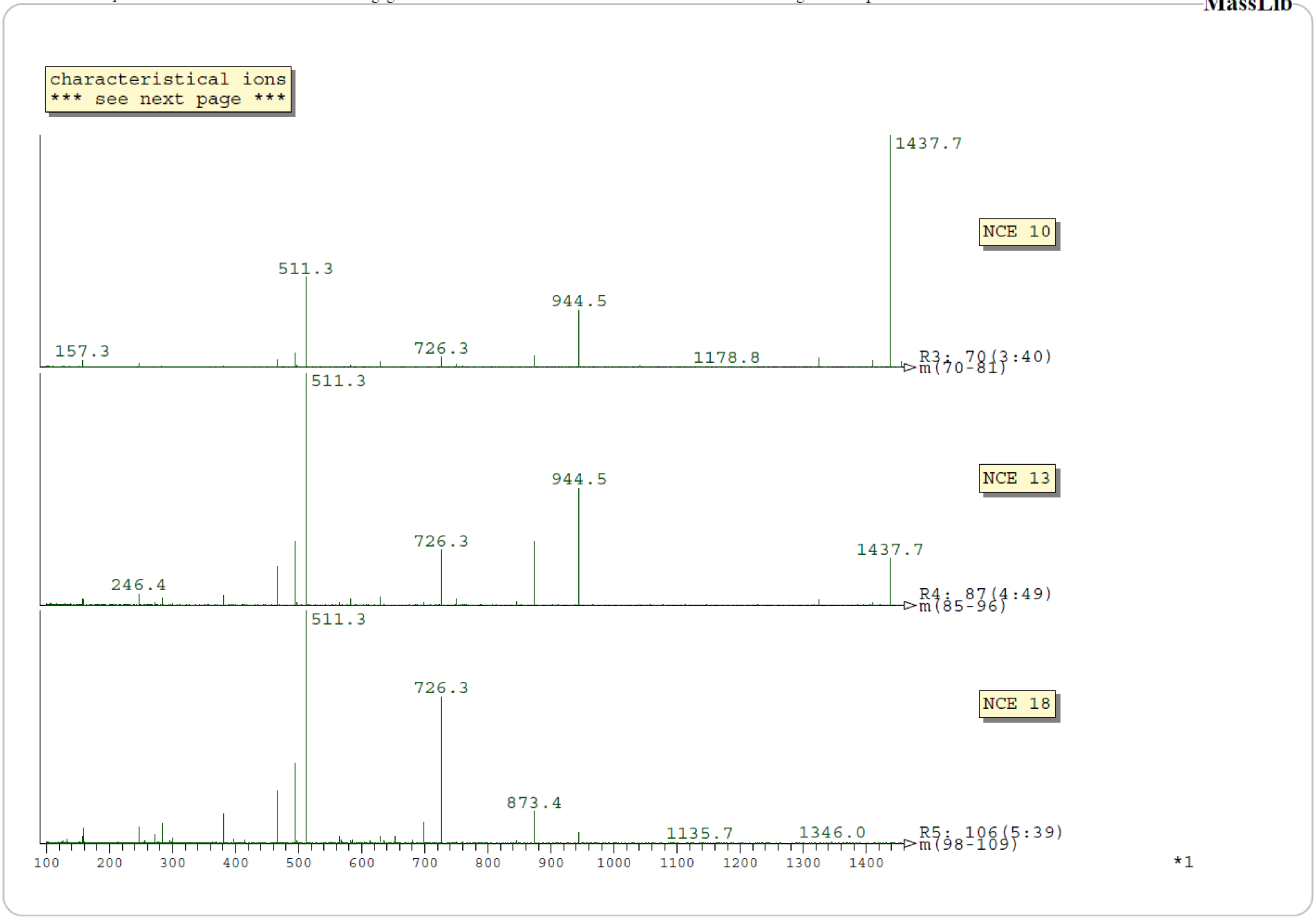

MassLib V9.4 MPI für Kohlenforschung

1.04.2022 11:29 p.5/6 *** Angegebene Mol.-Gewichte u. Massenzahlen basieren auf dem häufigsten Isotop der Elemente *** **MassLib**

characteristical ions
1437 = [C71H101N14O16S1]+
1409 = [C70H101N14O15S1]+
1324 = [C65H90N13O15S1]+
1041 = [C49H73N10O13S1]+
944 = [C44H66N9O12S1]+
873 = [C41H61N8O11S1]+
845 = [C40H61N8O10S1]+
726 = [C32H52N7O10S1]+
698 = [C31H52N7O9S1]+
582 = [C30H44N7O5]+
565 = [C30H41N6O5]+
511 = [C27H39N6O4]+
494 = [C27H36N5O4]+
466 = [C26H36N5O3]+
381 = [C21H25N4O3]+
300 = [C17H22N3O2]+
284 = [C16H18N3O2]+
272 = [C16H22N3O1]+
159 = [C10H11N2]+

additional characteristical ions
246, 749

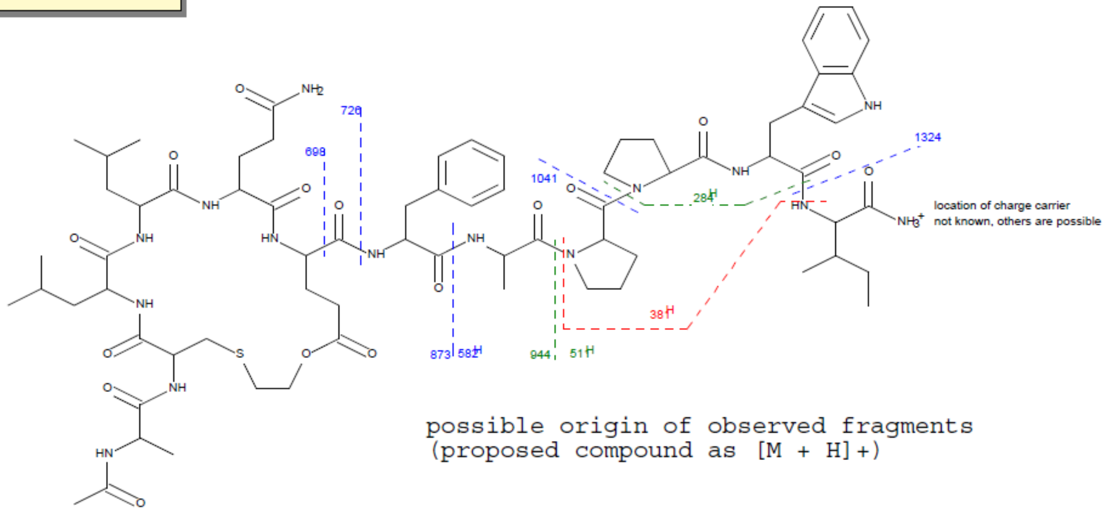

possible origin of observed fragments
(proposed compound as [M + H]+)

Stapled Ac-Ala-Cys-Leu-Leu-Gln-Cys-Phe-Ala-Pro-Pro-Trp-Ile-NH_2 (23)

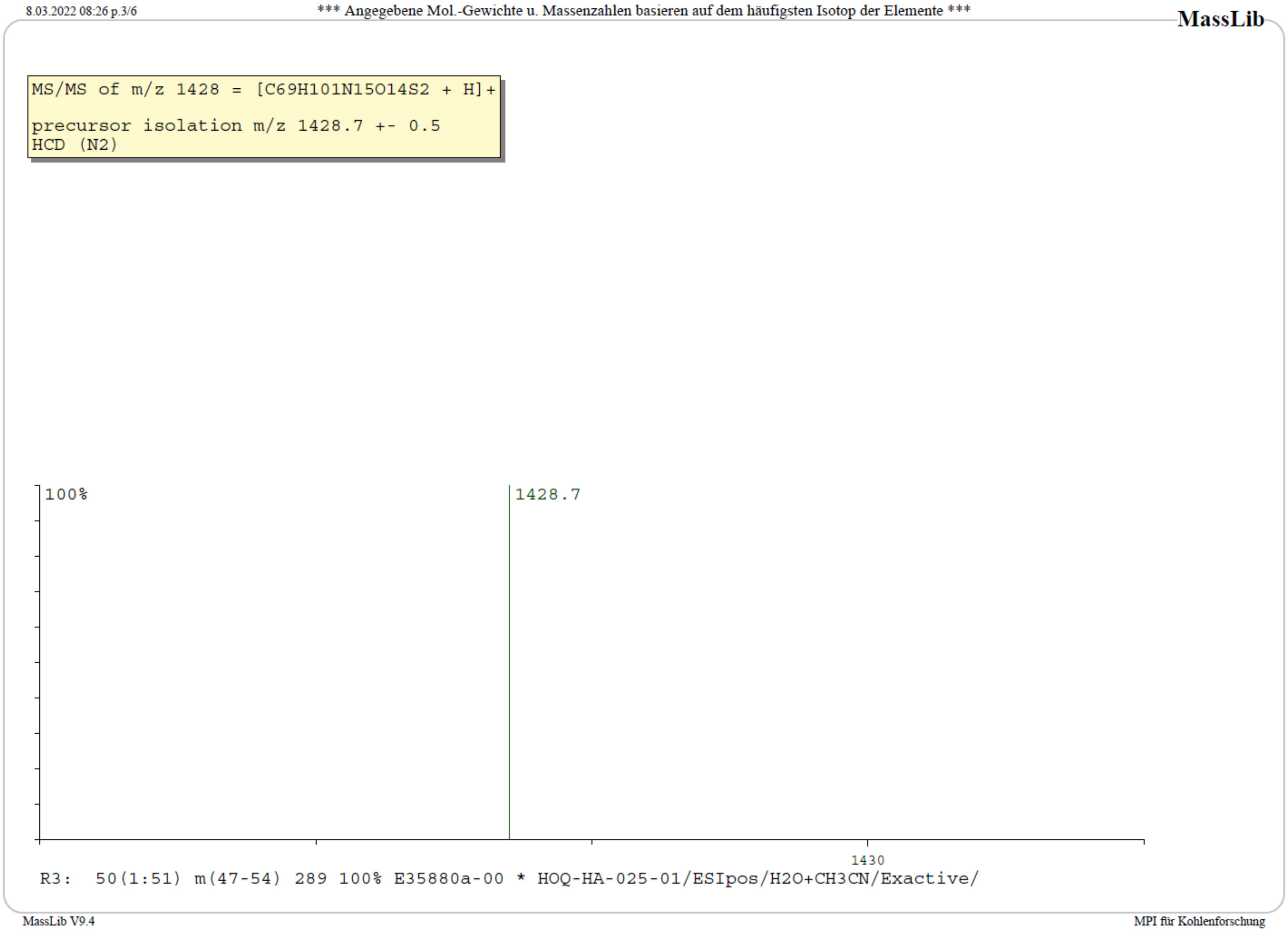

8.03.2022 08:26 p.4/6 *** Angegebene Mol.-Gewichte u. Massenzahlen basieren auf dem häufigsten Isotop der Elemente *** MassLib

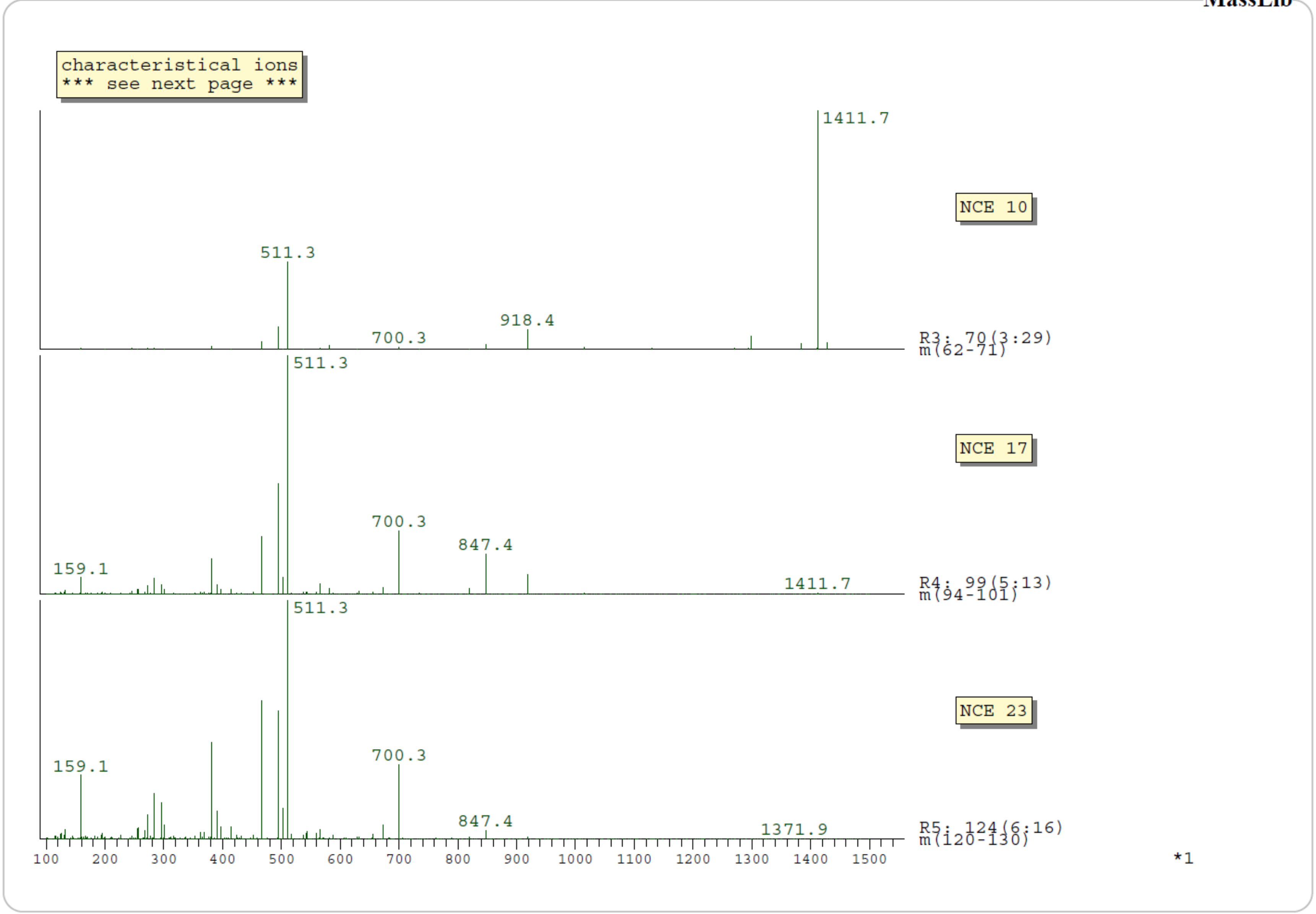

8.03.2022 08:26 p.6/6 *** Angegebene Mol.-Gewichte u. Massenzahlen basieren auf dem häufigsten Isotop der Elemente *** **MassLib**

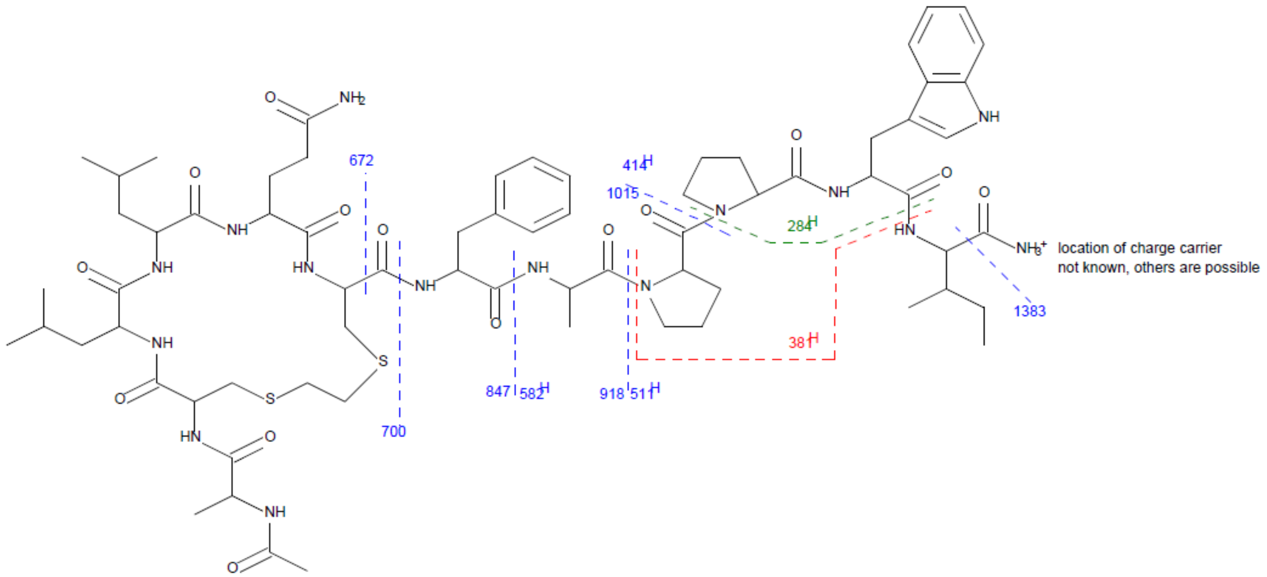


```
possible origin of observed ions
detected as 1428 = [M + H]+
```

Stapled Ac-Ala-Cys-Leu-Leu-Gln-Lys-Phe-Ala-Pro-Pro-Trp-Ile-NH_2 (24)

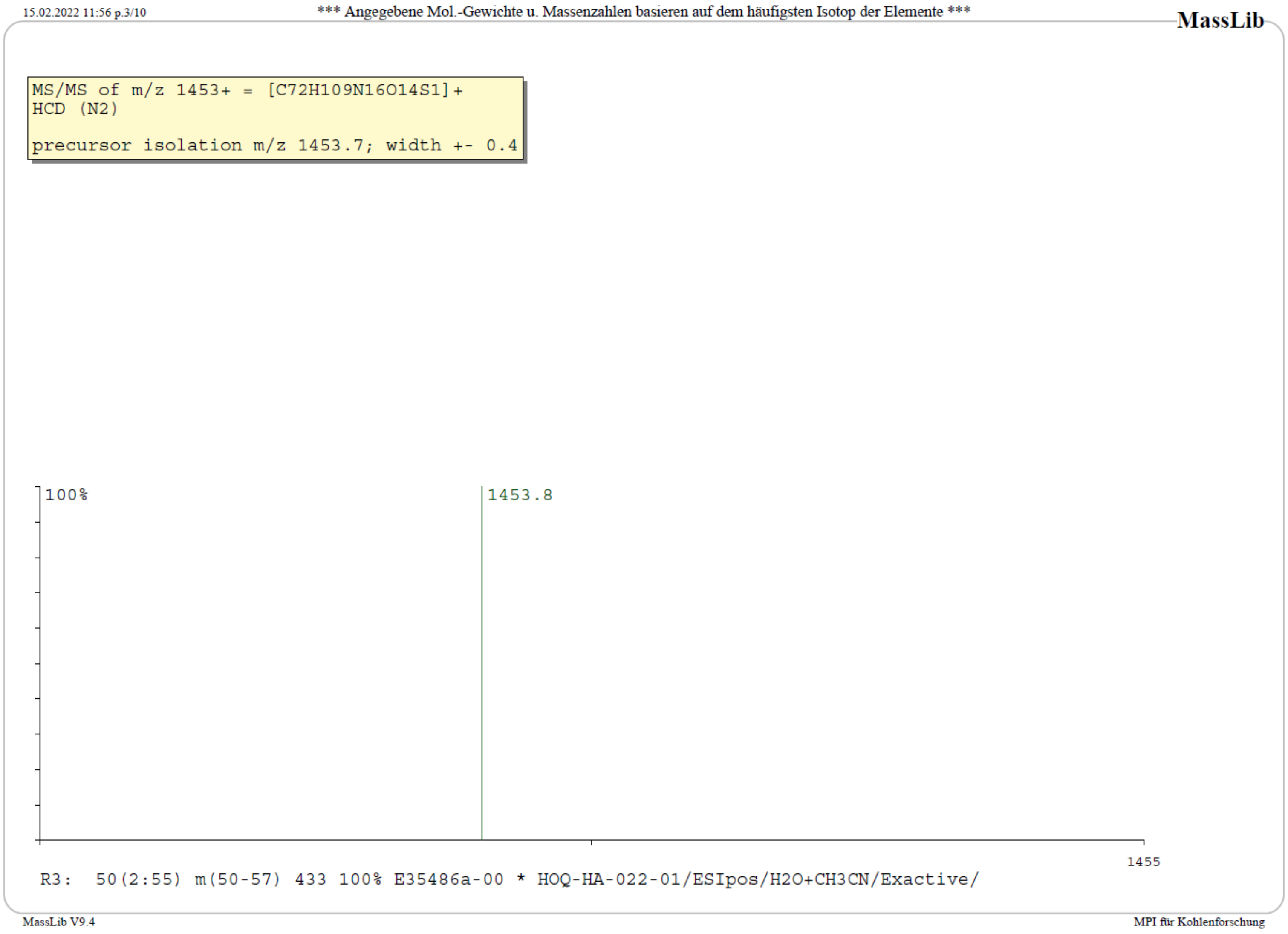

15.02.2022 11:56 p.4/10

*** Angegebene Mol.-Gewichte u. Massenzahlen basieren auf dem häufigsten Isotop der Elemente ***

MassLib

MS/MS of m/z 1453+ = [C72H109N16O14S1]+
HCD (N2)

precursor isolation m/z 1453.7; width +- 0.4

characteristical ions
*** see next page ***

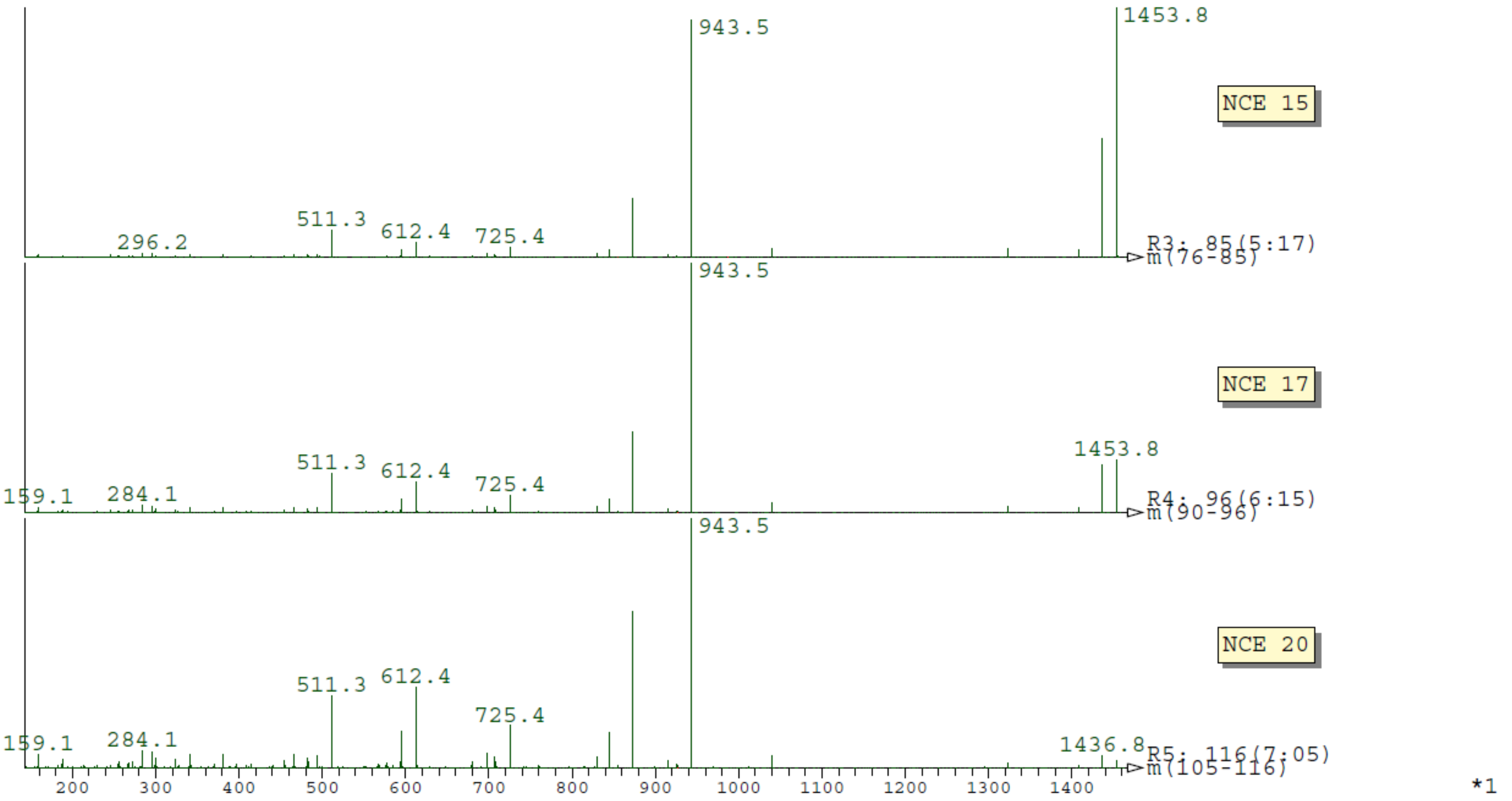

MassLib V9.4

MPI für Kohlenforschung

15.02.2022 11:56 p.5/10 *** Angegebene Mol.-Gewichte u. Massenzahlen basieren auf dem häufigsten Isotop der Elemente *** **MassLib**

characteristical ions
1436 = [1453 - NH3]+
1323 = [C66H95N14O13S1]+
1040 = [C50H78N11O11S1]+
943 = [C45H71N10O10S1]+
872 = [C42H66N9O9S1]+
844 = [C41H66N9O8S1]+
830 = [C40H64N9O8S1]+
725 = [C33H57N8O8S1]+
708 = [C33H54N7O8S1]+
707 = [C33H55N9O7S1]+
697 = [C32H57N8O7S1]+
612 = [C28H50N7O6S1]+
595 = [C28H47N6O6S1]+
511 = [C27H39N6O4]+
494 = [C27H36N5O4]+
466 = [C26H36N5O3]+
381 = [C21H25N4O3]+
353 = [C20H25N4O2]+
284 = [C16H18N3O2]+
256 = [C15H18N3O1]+
159 = [C10H11N2]+

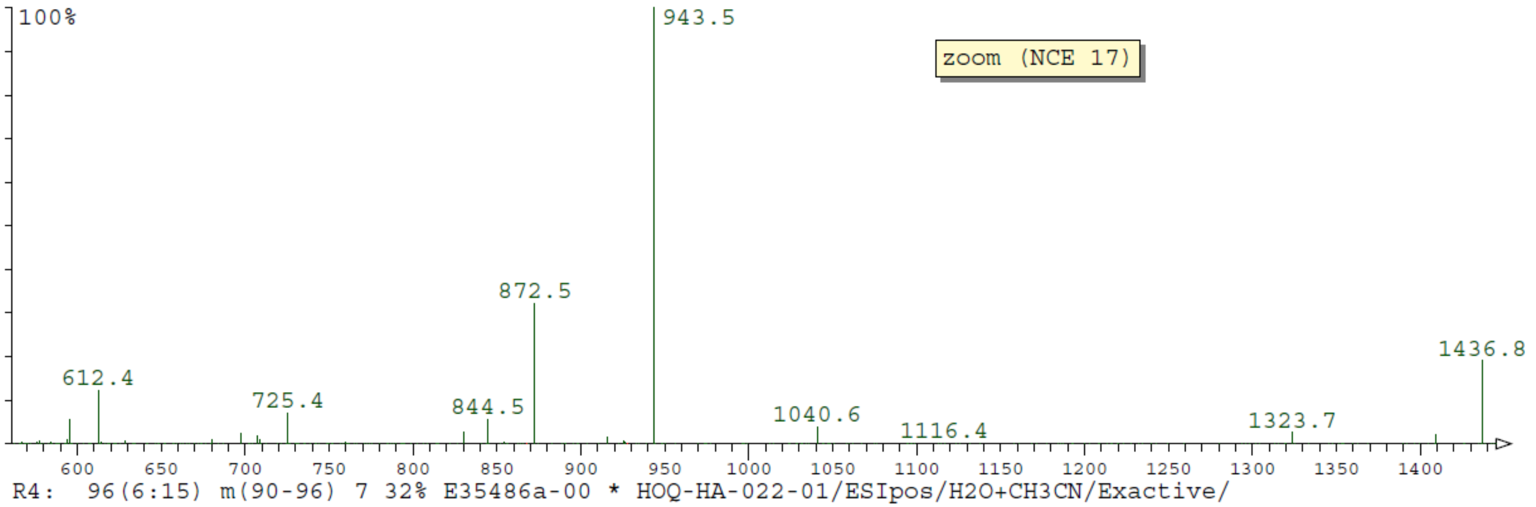

R4: 96(6:15) m(90-96) 7 32% E35486a-00 * HOQ-HA-022-01/ESIpos/H2O+CH3CN/Exactive/

MassLib V9.4 MPI für Kohlenforschung

15.02.2022 11:56 p.10/10 *** Angegebene Mol.-Gewichte u. Massenzahlen basieren auf dem häufigsten Isotop der Elemente *** **MassLib**

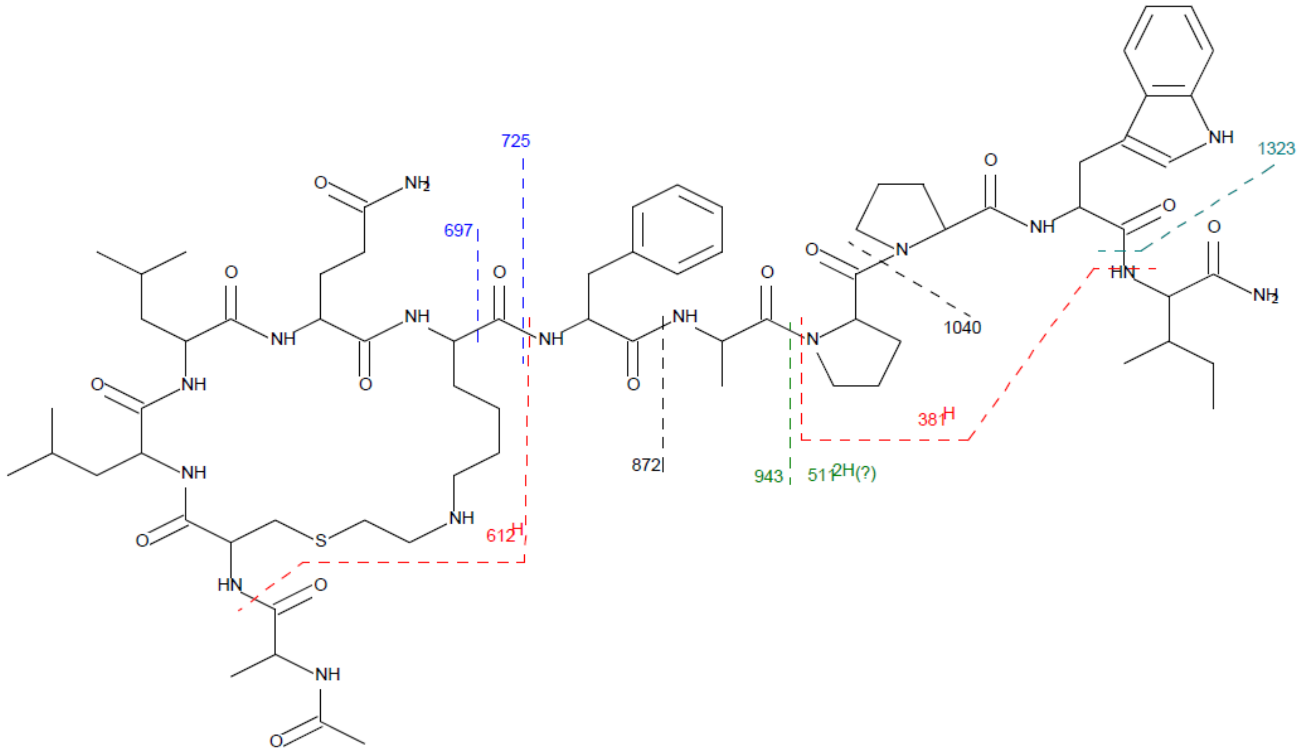

possible origin of fragments
(compound observed as [M + H]+ and [M + 2H]2+

Ethylene-bridged Lypressin (25)

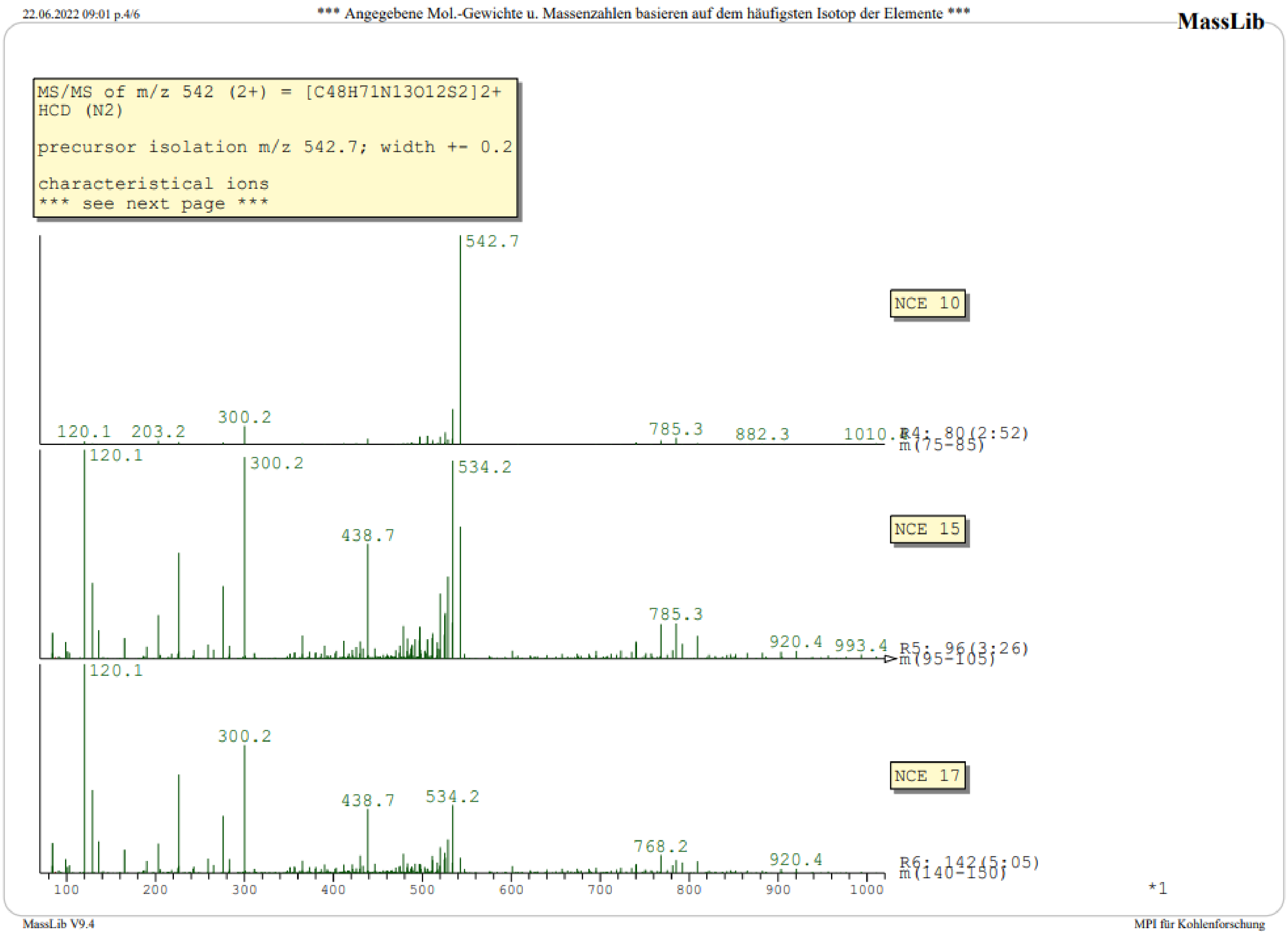

22.06.2022 09:01 p.5/6 *** Angegebene Mol.-Gewichte u. Massenzahlen basieren auf dem häufigsten Isotop der Elemente *** **MassLib**

characteristical ions
1010 = [C46H64N11O11S2]+
993 = [C46H61N10O11S2]+
976 = [C46H58N9O11S2]+
920 = [C39H58N11O11S2]+
903 = [C39H55N10O11S2]+
882 = [C40H52N9O10S2]+
865 = [C40H49N8O10S2]+
809 = [C34H53N10O9S2]+
792 = [C34H50N9O9S2]+
785 = [C35H45N8O9S2]+
781 = [C33H53N10O9S2]+
775 = [C34H47N8O9S2]+
768 = [C35H42N7O9S2]+
740 = [C34H42N7O8S2]+
723 = [C34H39N6O8S2]+
695 = [C30H47N8O7S2]+
390 = [C18H24N5O5]+
351 = [C16H19N2O3S2]+
300 = [C13H26N5O3]+
276 = [C14H18N3O3]+
259 = [C
129 = [C6H13N2O1]+
120 = [C8H10N1]+
additional characteristical ions
601, 190, 165

characteristical ions (doubly charged)
534 = [C48H68N12O12S2]2+
520 = [C47H68N12O11S2]2+
505 = [C46H65N11O11S2]2+
497 = [C46H62N10O11S2]2+
446 = [C38H59N11O10S2]2+
438 = [C38H59N11O9S2]2+
additional characteristical ions(possibly doubly charged)
528, 591, 478, 430, 411, 365

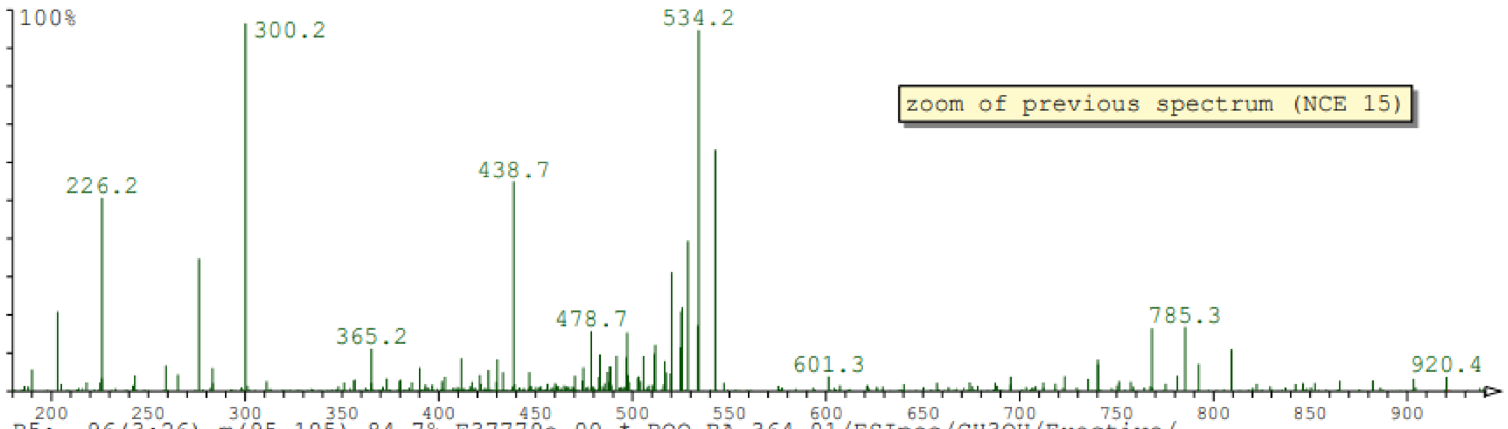

R5: 96(3:26) m(95-105) 84 7% E37770a-00 * BOO-BA-364-01/ESIpos/CH3OH/Exactive/

22.06.2022 09:01 p.6/6

*** Angegebene Mol.-Gewichte u. Massenzahlen basieren auf dem häufigsten Isotop der Elemente ***

MassLib

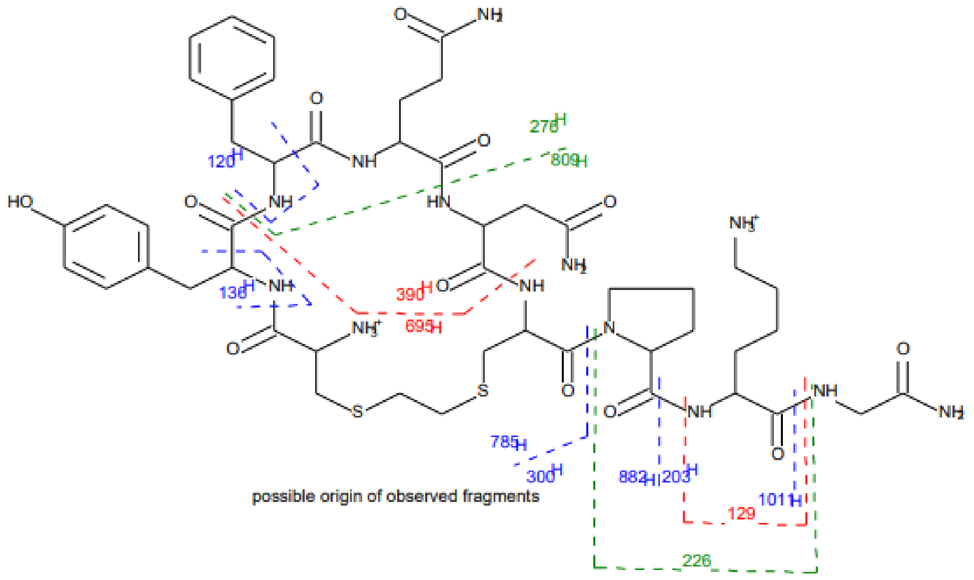

MassLib V9.4

MPI für Kohlenforschung

Ethylene-bridged Octreotide (26)

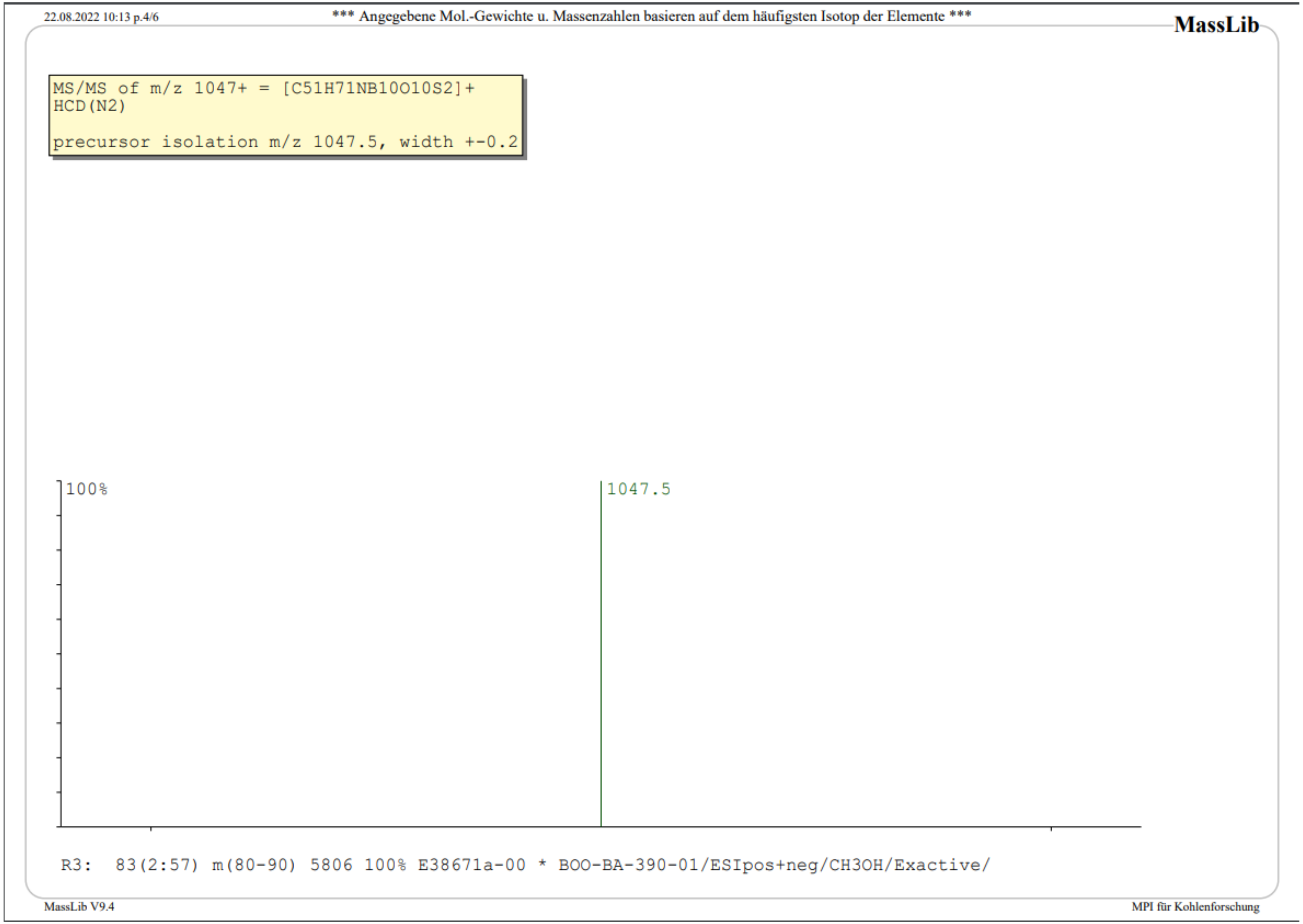

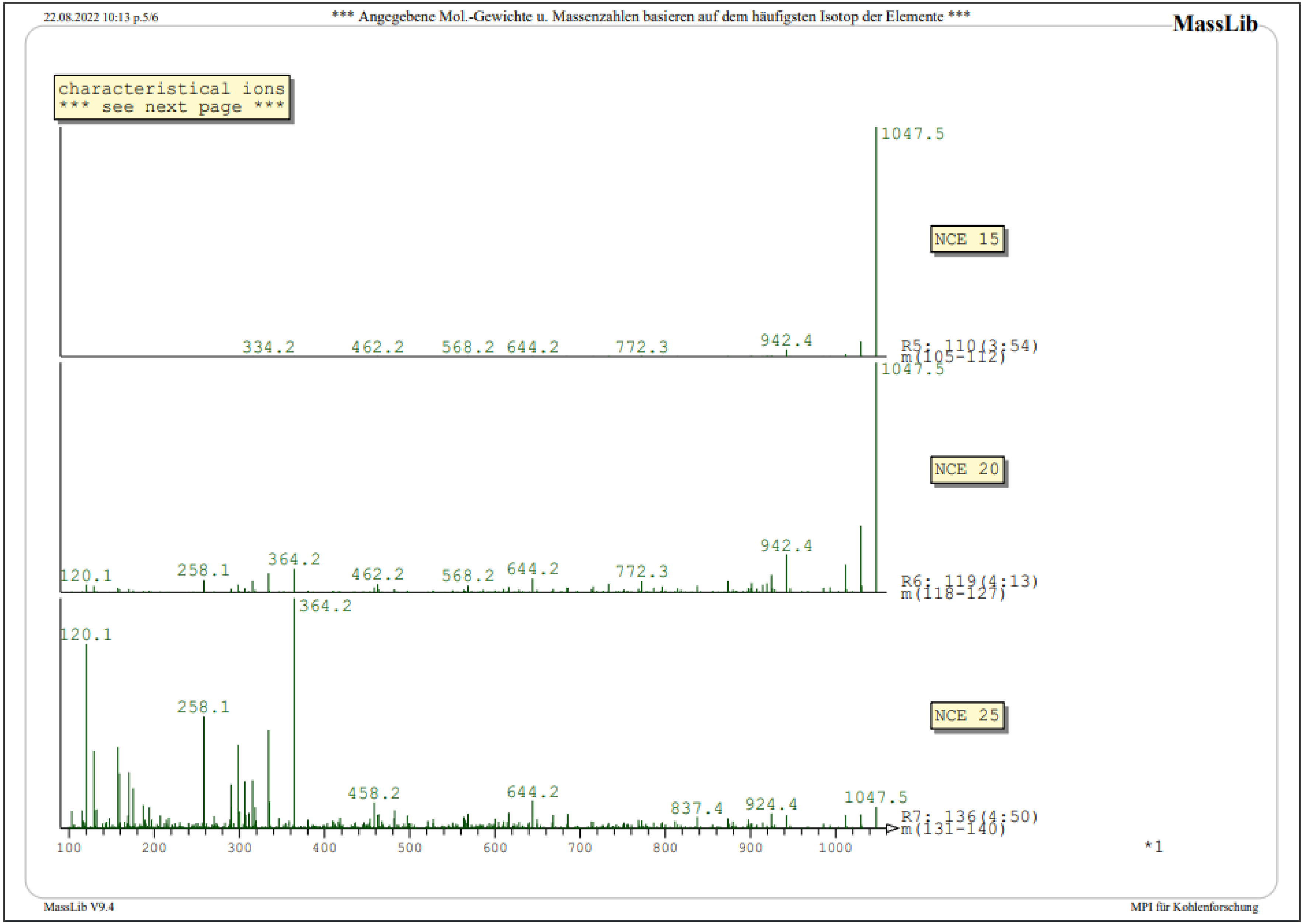
22.08.2022 10:13 p.5/6
*** Angegebene Mol.-Gewichte u. Massenzahlen basieren auf dem häufigsten Isotop der Elemente ***
MassLib
characteristical ions
*** see next page ***
1047.5
NCE 15
334.2
462.2
568.2
644.2
772.3
942.4
R5: 110(3:54)
m(105-112)
1047.5
NCE 20
120.1
258.1
364.2
462.2
568.2
644.2
772.3
942.4
R6: 119(4:13)
m(118-127)
364.2
120.1
258.1
NCE 25
458.2
644.2
837.4
924.4
1047.5
R7: 136(4:50)
m(131-140)
100
200
300
400
500
600
700
800
900
1000
*1
MassLib V9.4
MPI für Kohlenforschung

characteristical ions		
1030 =	[C51H68N9O10S2]+	(-NH3)
1029 =	[C51H69N10O9S2]+	(-H2O)
1012 =	[C51H66N9O9S2]+	
1011 =	[C51H67N10O8S2]+	
993 =	[C51H65N10O7S2]+	
942 =	[C47H60N9O8S2]+	
925 =	[C47H57N8O8S2]+	
924 =	[C47H58N9O7S2]+	
914 =	[C46H60N9O7S2]+	
897 =	[C46H57N8O7S2]+	
896 =	[C46H58N9O6S2]+	
873 =	[C44H57N8O7S2]+	
772 =	[C40H50N7O5S2]+	
644 =	[C34H38N5O4S2]+	
616 =	[C33H38N5O3S2]+	
586 =	[C25H40N5O7S2]+	
568 =	[C25H38N5O6S2]+	
462 =	[C26H32N5O3]+	
458 =	[C23H28N3O3S2]+	
364 =	[C21H22N3O3]+	
330 =	[C20H20N3O2]+	
315 =	[C17H23N4O2]+	
311 =	[C14H19N2O2S2]+	
306 =	[C19H20N3O1]+	
298 =	[C17H20N3O2]+	
283 =	[C13H19N2O1S2]+	
258 =	[C11H20N3O4]+	
187 =	[C11H11N2O1]+	
170 =	[C11H8N1O1]+	
159 =	[C10H11N2]+	
157 =	[C7H13N2O2]	
130 =	[C9H8N1]+	
129 =	[C6H13N2O1]+	
120 =	[C8H10N1]+	

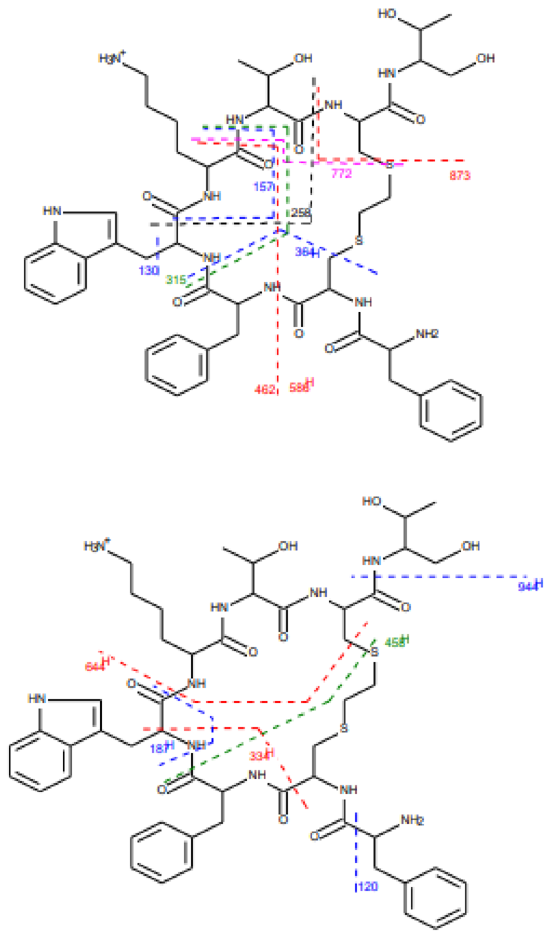

possible origin of observed fragments
(added twice for better visibility)

Ethylene-bridged Oxytocin (27)

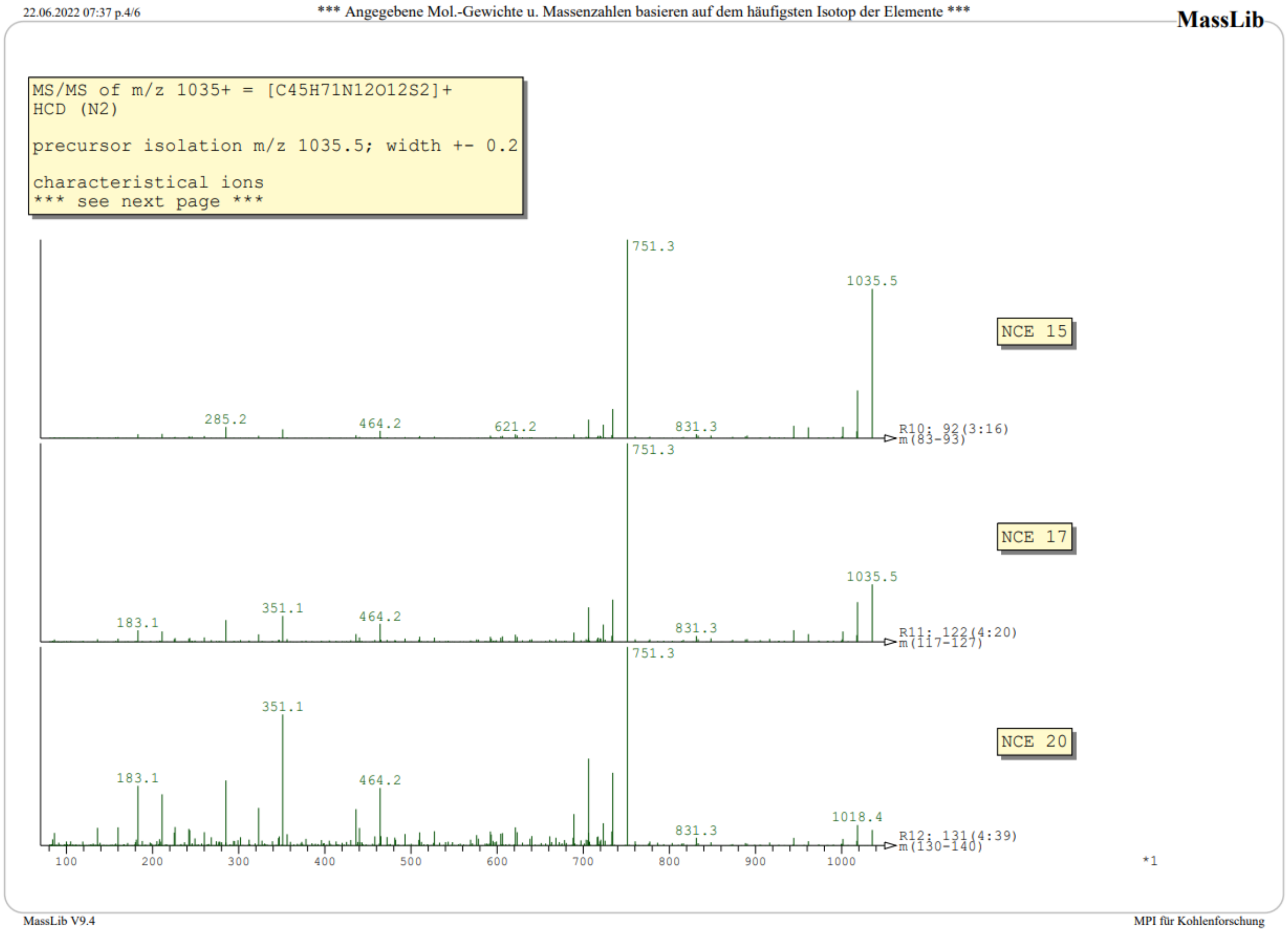

22.06.2022 07:37 p.5/6 *** Angegebene Mol.-Gewichte u. Massenzahlen basieren auf dem häufigsten Isotop der Elemente *** MassLib

characteristical ions

1018 = [C45H68N11O12S2]+	323 = [C15H19N2O2S2]+
1001 = [C45H65N10O12S2]+	285 = [C13H25N4O3]+
961 = [C43H65N10O11S2]+	211 = [C11H19N2O2]+
944 = [C43H62N9O11S2]+	183 = [C10H19O1N2]+
916 = [C42H62N9O10S2]+	160 = [C6H10N1S2]+
848 = [C37H54N9O10S2]+	136 = [C8H10N1O1]+
831 = [C37H51N8O10S2]+	
751 = [C32H47N8O9S2]+	additional characteristical ions
734 = [C32H44N7O9S2]+	640, 527, 464, 436, 260, 242, 226
723 = [C31H47N8O8S2]+	
706 = [C31H44N7O8S2]+	
689 = [C31H41N6O8S2]+	
661 = [C30H40N6O8S2]+	
638 = [C26H36N7O8S2]+	
623 = [C27H39N6O7S2]+	
606 = [C27H36N5O7S2]+	
621 = [C26H33N6O8S2]+	
604 = [C26H30N5O8S2]+	
593 = [C25H33N6O7S2]+	
576 = [C25H30N5O7S2]+	
510 = [C21H28N5O6S2]+	
493 = [C21H25N4O6S2]+	
351 = [C16H19N2O3S2]+	

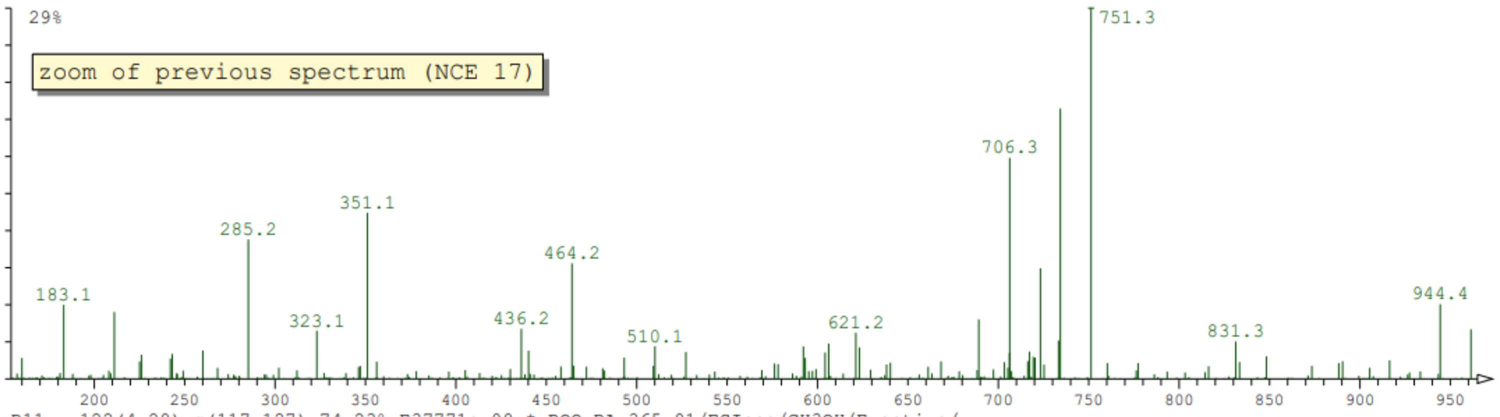

R11: 122(4:20) m(117-127) 74 23% E37771a-00 * BOO-BA-365-01/ESIpos/CH3OH/Exactive/

MassLib V9.4 MPI für Kohlenforschung

22.06.2022 07:37 p.6/6 *** Angegebene Mol.-Gewichte u. Massenzahlen basieren auf dem häufigsten Isotop der Elemente *** **MassLib**

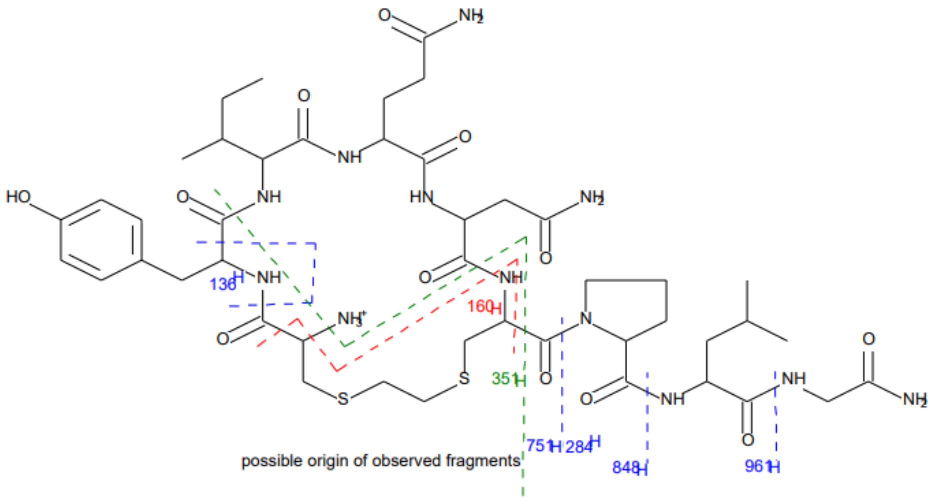

MassLib V9.4 MPI für Kohlenforschung

REFERENCES

1 Speicher, S., Plutschack, M. B. & Ritter, T. Late-stage C-H functionalization with 2,3,7,8-Tetrafluorothianthrene: Preparation of a tetrafluorothianthrenium-salt. *Org. Synth.* **98**, 531-552, doi:10.15227/orgsyn.098.0531 (2021).

2 Berger, F. *et al.* Site-selective and versatile aromatic C−H functionalization by thianthrenation. *Nature* **567**, 223-228, doi:10.1038/s41586-019-0982-0 (2019).

3 Fulmer, G. R. *et al.* NMR chemical shifts of trace impurities: common laboratory solvents, organics, and gases in deuterated solvents relevant to the organometallic chemist. *Organometallics* **29**, 2176-2179, doi:10.1021/om100106e (2010).

4 Marty, M. T. *et al.* Bayesian deconvolution of mass and ion mobility spectra: From binary interactions to polydisperse ensembles. *Anal. Chem.* **87**, 4370-4376, doi:10.1021/acs.analchem.5b00140 (2015).

5 *A Guide to polyacrylamide gel electrophoresis and detection, bio-rad.com*, 2017.

6 Julia, F., Yan, J. Y., Paulus, F. & Ritter, T. Vinyl thianthrenium tetrafluoroborate: A practical and versatile vinylating reagent made from ethylene. *J. Am. Chem. Soc.* **143**, 12992-12998, doi:10.1021/jacs.1c06632 (2021).

7 Amblard, M., Fehrentz, J.-A., Martinez, J. & Subra, G. Methods and protocols of modern solid phase peptide synthesis. *Mol. Biotechnology* **33**, 239-254, doi:10.1385/MB:33:3:239 (2006).

8 Subirós-Funosas, R., Prohens, R., Barbas, R., El-Faham, A. & Albericio, F. Oxyma: An efficient additive for peptide synthesis to replace the benzotriazole-based HOBt and HOAt with a lower risk of explosion. *Chem. Eur. J.* **15**, 9394-9403, doi:10.1002/chem.200900614 (2009).

9 Wingfield, P. T. N-Terminal methionine processing. *Curr. Protoc. Protein. Sci.* **88**, doi:10.1002/cpps.29 (2017).

10 Heim, R., Prasher, D. C. & Tsien, R. Y. Wavelength mutations and posttranslational autoxidation of green fluorescent protein. *Proc. Natl. Ac. Sci. USA* **91**, 12501-12504, doi:10.1073/pnas.91.26.12501 (1994).

11 Lazar, G. A., Desjarlais, J. R. & Handel, T. M. De novo design of the hydrophobic core of ubiquitin. *Protein Sci.* **6**, 1167-1178, doi:10.1002/pro.5560060605 (1997).

12 Spahr, P. F. Amino acid composition of ribosomes from Escherichia Coli. *J. Mol. Biol.* **4**, 395-406, doi:10.1016/S0022-2836(62)80020-5 (1962).

13 Hughes, C. S. *et al.* Single-pot, solid-phase-enhanced sample preparation for proteomics experiments. *Nat. Protoc.* **14**, 68-85, doi:10.1038/s41596-018-0082-x (2019).

14 *Escherichia coli. Proteome ID: UP000000625*, <https://www.uniprot.org/proteomes/UP000000625>

15 *Homo sapiens. Proteome ID: UP000005640*, <https://www.uniprot.org/proteomes/UP000005640>

16 *Saccharomyces cerevisiae. Proteome ID: UP000002311*, <https://www.uniprot.org/proteomes/UP000002311>

17 Suttapitugsakul, S., Xiao, H. P., Smeekens, J. & Wu, R. H. Evaluation and optimization of reduction and alkylation methods to maximize peptide identification with MS- based proteomics. *Mol. Biosyst.* **13**, 2574-2582, doi:10.1039/c7mb00393e (2017).

18 Abegg, D. *et al.* Chemoproteomic profiling by cysteine fluoroalkylation reveals Myrocin G as an inhibitor of the nonhomologous end joining DNA repair pathway. *J. Am. Chem. Soc.* **143**, 20332-20342, doi:10.1021/jacs.1c09724 (2021).

19 Ritchie, M. E. *et al.* limma powers differential expression analyses for RNA-sequencing and microarray studies. *Nuc. Ac. Res.* **43**, doi:10.1093/nar/gkv007 (2015).

20 Hacker, S. M. *et al.* Global profiling of lysine reactivity and ligandability in the human proteome. *Nature Chem.* **9**, 1181-1190, doi:10.1038/nchem.2826 (2017).

21 Jumper, J. *et al.* Highly accurate protein structure prediction with AlphaFold. *Nature* **596**, 583-589, doi:10.1038/s41586-021-03819-2 (2021).

22 Okuda, S. *et al.* jPOSTrepo: an international standard data repository for proteomes. *Nucleic Acids Res.* **45**, D1107-D1111, doi:10.1093/nar/gkw1080 (2016).

23 Kice, J. L., Anderson, J. M. & Pawlowsk.Ne. Mechanism of acid hydrolysis of Bunte salts (s-alkyl and s-aryl thiosulfates). *J. Am. Chem. Soc.* **88**, 5245-5250, doi:10.1021/ja00974a039 (1966).

24 Brustad, E. *et al.* A genetically encoded boronate-containing amino acid. *Angew. Chem. Int. Ed.* **47**, 8220-8223, doi:10.1002/anie.200803240 (2008).

25 Mollner, T. A. *et al.* Post-translational insertion of boron in proteins to probe and modulate function. *Nat. Chem. Bio.* **17**, 1245-1261, doi:10.1038/s41589-021-00883-7 (2021).

26 Josephson, B. *et al.* Light-driven post-translational installation of reactive protein side chains. *Nature* **585**, 530-537, doi:10.1038/s41586-020-2733-7 (2020).

27 Pédelacq, J.-D., Cabantous, S., Tran, T., Terwilliger, T. C. & Waldo, G. S. Engineering and characterization of a superfolder green fluorescent protein. *Nat. Biotechnol.* **24**, 79-88, doi:10.1038/nbt1172 (2006).

28 Burés, J. Variable time normalization analysis: general graphical elucidation of reaction orders from concentration profiles. *Angew. Chem. Int. Ed.* **55**, 16084-16087 (2016).

29 Burés, J. A simple graphical method to determine the order in catalyst. *Angew. Chem. Int. Ed.* **55**, 2028-2031 (2016).

30 Gómez-Gallego, M. & Sierra, M. A. Kinetic isotope effects in the study of organometallic reaction mechanisms. *Chem. Rev.* **111**, 4857-4963, doi:10.1021/cr100436k (2011).

31 Lee, I. Secondary kinetic isotope effects involving deuterated nucleophiles. *Chem. Soc. Rev.* **24**, 223-229, doi:10.1039/CS9952400223 (1995).

32 Delaglio, F. *et al.* NMRPipe: A multidimensional spectral processing system based on UNIX pipes. *J. Biomol. NMR* **6**, 277-293, doi:10.1007/BF00197809 (1995).

33 Ying, J., Delaglio, F., Torchia, D. A. & Bax, A. Sparse multidimensional iterative lineshape-enhanced (SMILE) reconstruction of both non-uniformly sampled and conventional NMR data. *J. Biomol. NMR* **68**, 101-118, doi:10.1007/s10858-016-0072-7 (2017).

34 Bax, A. & Grzesiek, S. in *NMR of proteins* (eds G. M. Clore & A. M. Gronenborn) 33-52 (Macmillan Education UK, 1993).

35 Lee, W., Tonelli, M. & Markley, J. L. NMRFAM-SPARKY: enhanced software for biomolecular NMR spectroscopy. *Bioinformatics* **31**, 1325-1327, doi:10.1093/bioinformatics/btu830 (2014).

36 Cornilescu, G., Marquardt, J. L., Ottiger, M. & Bax, A. Validation of protein structure from anisotropic carbonyl chemical shifts in a dilute liquid crystalline phase. *J. Am. Chem. Soc.* **120**, 6836-6837, doi:10.1021/ja9812610 (1998).

37 Ulrich, E. L. *et al.* BioMagResBank. *Nucleic Acids Res.* **36**, D402-D408, doi:10.1093/nar/gkm957 (2008).

38 Nguyen, D., Chen, C., Pettitt, B. M. & Iwahara, J. in *Meth. Enzymol.* Vol. 615 (ed A. Joshua Wand) 285-332 (Academic Press, 2019).

39 Markley, J. L. *et al.* Recommendations for the presentation of NMR structures of proteins and nucleic acids. *Eur. J. Biochem.* **256**, 1-15, doi:10.1046/j.1432-1327.1998.2560001.x (1998).

40 Williamson, M. P. Using chemical shift perturbation to characterise ligand binding. *Prog. Nucl. Magn. Reson. Spectrosc.* **73**, 1-16, doi:10.1016/j.pnmrs.2013.02.001 (2013).
