# Supplementary material for: Chemoselective umpolung of thiols to episulfoniums for cysteine bioconjugation
Source: Nat Chem. 2023 Dec 20;16(3):380–8. doi: 10.1038/s41557-023-01388-7 (PMC10914617; doi:10.1038/s41557-023-01388-7)
Supplement: Supplementary file 2 — Reporting Summary [file 41557_2023_1388_MOESM2_ESM.pdf]

nature portfolio

Corresponding author(s): Tobias Ritter
Last updated by author(s): Oct 26, 2023

Reporting Summary

Nature Portfolio wishes to improve the reproducibility of the work that we publish. This form provides structure for consistency and transparency in reporting. For further information on Nature Portfolio policies, see our Editorial Policies and the Editorial Policy Checklist.

Statistics

For all statistical analyses, confirm that the following items are present in the figure legend, table legend, main text, or Methods section.

n/a	Confirmed	
☐	☒	The exact sample size (*n*) for each experimental group/condition, given as a discrete number and unit of measurement
☐	☒	A statement on whether measurements were taken from distinct samples or whether the same sample was measured repeatedly
☐	☒	The statistical test(s) used AND whether they are one- or two-sided<br>*Only common tests should be described solely by name; describe more complex techniques in the Methods section.*
☐	☒	A description of all covariates tested
☒	☐	A description of any assumptions or corrections, such as tests of normality and adjustment for multiple comparisons
☐	☒	A full description of the statistical parameters including central tendency (e.g. means) or other basic estimates (e.g. regression coefficient) AND variation (e.g. standard deviation) or associated estimates of uncertainty (e.g. confidence intervals)
☐	☒	For null hypothesis testing, the test statistic (e.g. *F*, *t*, *r*) with confidence intervals, effect sizes, degrees of freedom and *P* value noted<br>*Give P values as exact values whenever suitable.*
☒	☐	For Bayesian analysis, information on the choice of priors and Markov chain Monte Carlo settings
☒	☐	For hierarchical and complex designs, identification of the appropriate level for tests and full reporting of outcomes
☐	☒	Estimates of effect sizes (e.g. Cohen's *d*, Pearson's *r*), indicating how they were calculated

Our web collection on statistics for biologists contains articles on many of the points above.

Software and code

Policy information about availability of computer code

Data collection	Data was collected utilizing the software that is provided by the instrument vendors and details can be found in the Methods section.
Data analysis	Data was analyzed with Unidec (open source, version 5.0.4), Mestrenova (Mestrelab research, version 14.2.3-29241), LabSolutions (Shimadzu Corporations, version V5.97 & V5.114), Microsoft Excel 16 (Microsoft, version 16.0.5369.1000), xiSEARCH (open source, version 1.7.6.7), MaxQuant (partially open source, version 2.1.3.0), Haddock 2.4 (open source, v2.4-2022.08), Origin 2019b (OriginLab Corporation, version 9.65), UVProbe (Shimadzu Corporations, version 2.50), PyMOL (Schroedinger, Inc., version 2.5.4 & 2.4), Maestro (Schroedinger, Inc., version 12.6.149), Xcalibur (Thermo Fisher Scientific Inc., version V4.1.31.9), MassLib (MSP Kofel, version V9.4-415), R (open source, 4.1.1), AlphaFold 2 (open source, version 2.0), Cary UV Workstation (Agilent Technologies, version: 1.0.1284.0), TopSpin (Bruker, version 3.6.2 & 3.6.3), NMRPipe (open source, version 11.0 Rev 2022.193.18.59), NMRFAM-SPARKY (open source, version 1.470), Image Lab (Bio-Rad Laboratories, Inc., version 6.1.0 build 7), LabSolutions Insight Explore (Shimadzu Corporations, version 3.8 SP4)

For manuscripts utilizing custom algorithms or software that are central to the research but not yet described in published literature, software must be made available to editors and reviewers. We strongly encourage code deposition in a community repository (e.g. GitHub). See the Nature Portfolio guidelines for submitting code & software for further information.

Data

Policy information about availability of data

All manuscripts must include a data availability statement. This statement should provide the following information, where applicable:

- Accession codes, unique identifiers, or web links for publicly available datasets
- A description of any restrictions on data availability
- For clinical datasets or third party data, please ensure that the statement adheres to our policy

Raw protein LC–MS, raw protein MS/MS, and raw protein NMR data are deposited in the repositories: ownCloud (https://owncloud.gwdg.de/index.php/s/uf0bv5a6HLflfcW), jPOSTrepo (https://repository.jpostdb.org/preview/169290337964832fb33c1bc, access key: 1252), BioMagRes-Bank (accession numbers: 51721, 51725), and Zenodo (https://doi.org/10.5281/zenodo.7472436). The following publicly available protein structures were used: sfGFP (PDB ID 2B3P), DHAR2 (PDB ID 5LOL), Ubiquitin (PDB ID 1D3Z & 1UBQ), Trxh1 (PDB ID 1XFL), and DHAR1 (PDB ID 5EL8), BSA (PDB ID 3V03).

Human research participants

Policy information about studies involving human research participants and Sex and Gender in Research.

Reporting on sex and gender	*Use the terms sex (biological attribute) and gender (shaped by social and cultural circumstances) carefully in order to avoid confusing both terms. Indicate if findings apply to only one sex or gender; describe whether sex and gender were considered in study design whether sex and/or gender was determined based on self-reporting or assigned and methods used. Provide in the source data disaggregated sex and gender data where this information has been collected, and consent has been obtained for sharing of individual-level data; provide overall numbers in this Reporting Summary. Please state if this information has not been collected. Report sex- and gender-based analyses where performed, justify reasons for lack of sex- and gender-based analysis.*
Population characteristics	*Describe the covariate-relevant population characteristics of the human research participants (e.g. age, genotypic information, past and current diagnosis and treatment categories). If you filled out the behavioural & social sciences study design questions and have nothing to add here, write "See above."*
Recruitment	*Describe how participants were recruited. Outline any potential self-selection bias or other biases that may be present and how these are likely to impact results.*
Ethics oversight	*Identify the organization(s) that approved the study protocol.*

Note that full information on the approval of the study protocol must also be provided in the manuscript.

Field-specific reporting

Please select the one below that is the best fit for your research. If you are not sure, read the appropriate sections before making your selection.

☒ Life sciences ☐ Behavioural & social sciences ☐ Ecological, evolutionary & environmental sciences

For a reference copy of the document with all sections, see nature.com/documents/nr-reporting-summary-flat.pdf

Life sciences study design

All studies must disclose on these points even when the disclosure is negative.

Sample size	Enzyme assays were conducted with n=2,3,6,9,18. The exact number of replicates is reported in the supplementary information for each experiment. LC-MS/MS analysis was carried out as technical replicates with n=2. Chemical synthesis and SDS-PAGE analysis were carried out as single experiments. Protein modifications were carried out as single experiments and the reported values for conversion and yield should be regarded as semi-quantitative. Synthetic reactions and protein modifications were carried out independently for multiple times and proved to be reliable and consistent.
Data exclusions	No data was excluded.
Replication	Enzyme assays were conducted with n=2,3,6,9,18. The exact number of replicates is reported in the supplementary information for each experiment. LC-MS/MS analysis was carried out as technical replicates with n=2. Chemical synthesis was carried out as single experiments. Protein modifications were carried out as single experiments and the reported values for conversion and yield should be regarded as semi-quantitative. Synthetic reactions and protein modifications were carried out independently for multiple times and proved to be reliable and consistent. All carried out reactions with proteins and small molecules could be repeated. The general replication of the described methods were successful.
Randomization	Not applicable

Blinding	Not applicable

Reporting for specific materials, systems and methods

We require information from authors about some types of materials, experimental systems and methods used in many studies. Here, indicate whether each material, system or method listed is relevant to your study. If you are not sure if a list item applies to your research, read the appropriate section before selecting a response.

Materials & experimental systems

n/a	Involved in the study	
☐	☒	Antibodies
☒	☐	Eukaryotic cell lines
☒	☐	Palaeontology and archaeology
☒	☐	Animals and other organisms
☒	☐	Clinical data
☒	☐	Dual use research of concern

Methods

n/a	Involved in the study	
☒	☐	ChIP-seq
☒	☐	Flow cytometry
☒	☐	MRI-based neuroimaging

Antibodies

Antibodies used	His-Tag (H-3; Lot # H2621; mouse monoclonal IgG1; 200 µg/ml; sc-8036; Santa Cruz Biotechnology), anti mouse IgG (peroxidase antibody produced in rabbit; buffered solution; Lot # 089M4797V; A9044-2ML; Sigma Aldrich)
Validation	https://www.scbt.com/de/p/his-probe-antibody-h-3<br>https://www.sigmaaldrich.com/DE/en/product/sigma/a9044
